# Recruitment of Reverse Transcriptase-Cas1 Fusion Proteins by Type VI-A CRISPR-Cas Systems

**DOI:** 10.3389/fmicb.2019.02160

**Published:** 2019-09-13

**Authors:** Nicolás Toro, Mario Rodríguez Mestre, Francisco Martínez-Abarca, Alejandro González-Delgado

**Affiliations:** Structure, Dynamics and Function of Rhizobacterial Genomes, Department of Soil Microbiology and Symbiotic Systems, Estación Experimental del Zaidín, Consejo Superior de Investigaciones Científicas, Granada, Spain

**Keywords:** reverse transcriptase, CRISPR-Cas, phylogeny, type VI CRISPR, Cas1

## Abstract

Type VI CRISPR–Cas systems contain a single effector nuclease (Cas13) that exclusively targets single-stranded RNA. It remains unknown how these systems acquire spacers. It has been suggested that type VI systems with adaptation modules can acquire spacers from RNA bacteriophages, but sequence similarities suggest that spacers may provide immunity to DNA phages. We searched databases for Cas13 proteins with linked RTs. We identified two different type VI-A systems with adaptation modules including an RT-Cas1 fusion and Cas2 proteins. Phylogenetic reconstruction analyses revealed that these adaptation modules were recruited by different effector Cas13a proteins, possibly from RT-associated type III-D systems within the bacterial classes Alphaproteobacteria and Clostridia. These type VI-A systems are predicted to acquire spacers from RNA molecules, paving the way for future studies investigating their role in bacterial adaptive immunity and biotechnological applications.

## Introduction

CRISPR-Cas systems, which consist of clusters of regularly interspaced short palindromic repeats (CRISPRs) and CRISPR-associated (Cas) proteins, provide prokaryotes with adaptive immunity to phages and other invasive mobile genetic elements, through the sequence-specific targeting of complementary nucleic acids (Sorek et al., 2013; Barrangou and Marraffini, 2014; Mohanraju et al., 2016; Barrangou and Horvath, 2017; Koonin et al., 2017). A typical CRISPR-Cas system consists of a CRISPR array containing the repeats (∼30 to 40 base-pair) separated by sequences (spacers) representing the memory of previous attacks, an adjacent adaptation module comprising the Cas1 and Cas2 proteins and the interference module, with different architectures, including unique signature proteins (Makarova et al., 2013, 2015). These systems are currently classified into two general classes on the basis of this interference module: class 1 (types I, III, and IV) systems, which have multi-subunit effector complexes, and class 2 (types II, V, and VI) systems, which have single-subunit effectors and can be further subdivided into different subtypes (Makarova et al., 2011, 2015, 2018; Makarova and Koonin, 2015). The CRISPR-Cas immune response has three stages: (1) adaptation, leading to the acquisition of spacers and their integration between two repeat sequences of the CRISPR array, (2) expression and processing, leading to the generation of mature small CRISPR RNAs (crRNAs), and (3) interference, in which the crRNA bound to the processing complex is used as a guide for the recognition of complementary foreign sequences, which are then cleaved and inactivated (van der Oost et al., 2014; Amitai and Sorek, 2016). Most CRISPR-Cas systems target DNA, with the exception of type III systems, which have a characteristic targeting mechanism resulting in the cotranscriptional cleavage of the target DNA and its transcript (Staals et al., 2014; Tamulaitis et al., 2014; Pyenson and Marraffini, 2017), and the rare type VI effectors (Cas13), which specifically target RNA (O’Connell, 2019).

Type VI CRISPR–Cas systems have been classified into four subtypes (A, B1/2, C, and D) on the basis of Cas13 phylogeny (Shmakov et al., 2017; Konermann et al., 2018; Yan et al., 2018). Cas13 (C2c2) exhibits RNA-guided RNase function and can be programmed to knock down specific mRNAs (Abudayyeh et al., 2016). Cas13 proteins have two different ribonuclease activities, one responsible for pre-crRNA processing, and the other, mediated by the two higher eukaryotes and prokaryotes nucleotide-binding (HEPN) domains, responsible for target RNA degradation during the interference stage (East-Seletsky et al., 2016; East-Seletsky et al., 2017; Shmakov et al., 2017; Smargon et al., 2017; Konermann et al., 2018). It has also been suggested that the function of type VI systems might serve as a defense against DNA phages (Smargon et al., 2017; Konermann et al., 2018) mediated by the recognition of a cognate phage transcript (Shmakov et al., 2017). It has also recently been suggested that the RNase activity of Cas13a might promote a state of cell dormancy enabling the infected cells to stop the invading double-stranded DNA phages, thereby protecting uninfected cells (Meeske et al., 2019).

Reverse transcriptases (RTs), the enzymes responsible for converting RNA into cDNA, have been found in association with CRISPR-Cas systems, either adjacent to or fused at the C-terminus to Cas1. These enzymes have been classified into 14 distinct clades, 12 of which contain RTs phylogenetically related to those encoded by mobile group II introns (Toro et al., 2017, 2018, 2019). These RTs, which appear to be typically associated with type III systems of all known subtypes (III-A, III-B, III-C, and III-D), have been reported to mediate spacer acquisition from RNA molecules (Silas et al., 2016; Mohr et al., 2018; Schmidt et al., 2018).

It remains unclear how spacers are acquired in type VI systems, and the possibility of spacer acquisition directly from RNA molecules remains hypothetical (Zhu et al., 2018), because no consistent evidence for associated RTs has been obtained (Shmakov et al., 2015). We searched databases for Cas13 effectors with associated RTs. We identified two different type VI-A systems including RT-Cas1 fusions. Phylogenetic reconstruction analyses suggested that the corresponding adaptation modules, comprising RT-Cas1 and Cas2, were recruited independently on separate occasions, possibly from type III-D systems, by Cas13a effector proteins, these events occurring in the bacterial classes Alphaproteobacteria and Clostridia. These findings are consistent with the acquisition of spacers from RNA molecules by these type VI-A systems.

## Materials and Methods

### Computational Sequence Analysis

For characterization of the association of RTs with type VI effector Cas13 proteins, we first searched the non-redundant protein database (nr) with hmmscan from the HMMER 3.2.1 suite, using protein profiles for the different Cas13 subtypes (A–D) (Toro et al., 2019). We then used a previously described computational pipeline to obtain information about the neighborhood (±30 kb) of the genes encoding putative Cas13 proteins (Toro et al., 2019). This approach uses CRT (Bland et al., 2007) to predict the CRISPR arrays and hmmsearch to identify the annotated entries. It also retrieves taxonomic information and classifies the CRISPR-Cas systems.

Cas1 and Cas2 proteins closely related to those associated with Cas13a clustered in clade 2 were also identified in databases with phmmer on the HMMER webserver^1^, with an *E*-value ≤ 1.1e-70 and *E*-value ≤ 2e-15, respectively.

### Metagenomic Sequence Search

We increased the number and diversity of Cas13 homologs identified, with the sequence search service from the MGnify resource (Mitchell et al., 2017), which uses phmmer from HmmerWeb version 1.3.1 for iterative searches in a database of proteins derived from metagenomic studies.

### Spacers and Direct Repeat Sequence Analyses

We investigated the origins of the spacers of type VI-A/RT systems, by performing BLASTn searches against the NCBI nucleotide database, the IMG/VR database (Paez-Espino et al., 2017) and the Human Gut virome database (Gregory et al., 2019) with parameters described elsewhere (blastn -task “blastn-short” -db dbname -query queryname -out outputfile -outfmt ‘6 stdqcovs’ -*e*value 0.003 -word_size 7 -gapopen 10 -gapextend 2 -penalty -1 -max_target_seqs 1000) (Díez-Villaseñor and Rodriguez-Valera, 2019). The secondary structure of the direct repeats was predicted with RNA structure software (Bellaousov et al., 2013), with manual refinement.

### Phylogenetic Analyses of Cas Sequences

We used MAFFT software (Katoh and Standley, 2013) and progressive methods for MSAs. The phylogenetic trees were constructed with the FastTree program (Price et al., 2010), as previously described (Toro et al., 2018, 2019).

## Results and Discussion

### RT-Cas1 Fusions Associated With CRISPR-Cas Type VI Systems

Type VI systems usually lack the *cas1* and *cas2* genes of the adaptation module. The acquisition of these adaptation genes by some type VI systems probably increased the specificity and efficiency of the effector protein (Koonin and Makarova, 2019), and may have facilitated the acquisition of new spacers from RNA molecules, because these systems exclusively target single-stranded RNA (Shmakov et al., 2015). However, only one of the type VI systems described to date includes a separate RT. This exception is Lachnospiraceae bacterium MA2020, but this RT actually corresponds to a group II intron-encoded RT, as the characteristic group II intron ribozyme DV and DVI domains (Michel et al., 1989) are readily identifiable immediately downstream (data not shown). We nevertheless performed a thorough database analysis, to search for type VI systems with linked RTs.

We first searched a non-redundant (nr) protein sequence database (NCBI) with hmmscan, to identify Cas13 homologs based on protein profiles for the different Cas13 subtypes (A–D) (Toro et al., 2019). This strategy identified several Cas13 homologs (62 Cas13a, 236 Cas13b, 21 Cas13c, and 27 Cas13d), which were then further analyzed with a computational pipeline described elsewhere (Toro et al., 2019), to search for a neighboring RT gene. Interestingly, we found some type VI systems with linked adaptation modules including RT-Cas1 fusions, but all were Cas13a-containing type VI CRISPR-Cas systems (approximately 15%), raising the possibility that these systems acquire spacers directly from RNA. The implications of this apparently specific association with type VI-A systems are unclear, but this association may reflect differences in the mechanistic functioning of the adaptation and effector complexes of these CRISPR-Cas systems. However, it cannot be rule out that RTs can be recruited by other Cas13 subtypes as a differentiated response to particular pressures on the bacterial ecosystems leading to environmental changes. Furthermore, most of the bacterial hosts harboring type VI-A systems have RT genes in their genomes (Supplementary Table S1). It is, therefore, not possible to exclude the possibility that type VI-A systems lacking an associated RT also acquire spacers from RNA through *trans*-RT activity.

**FIGURE 1:**
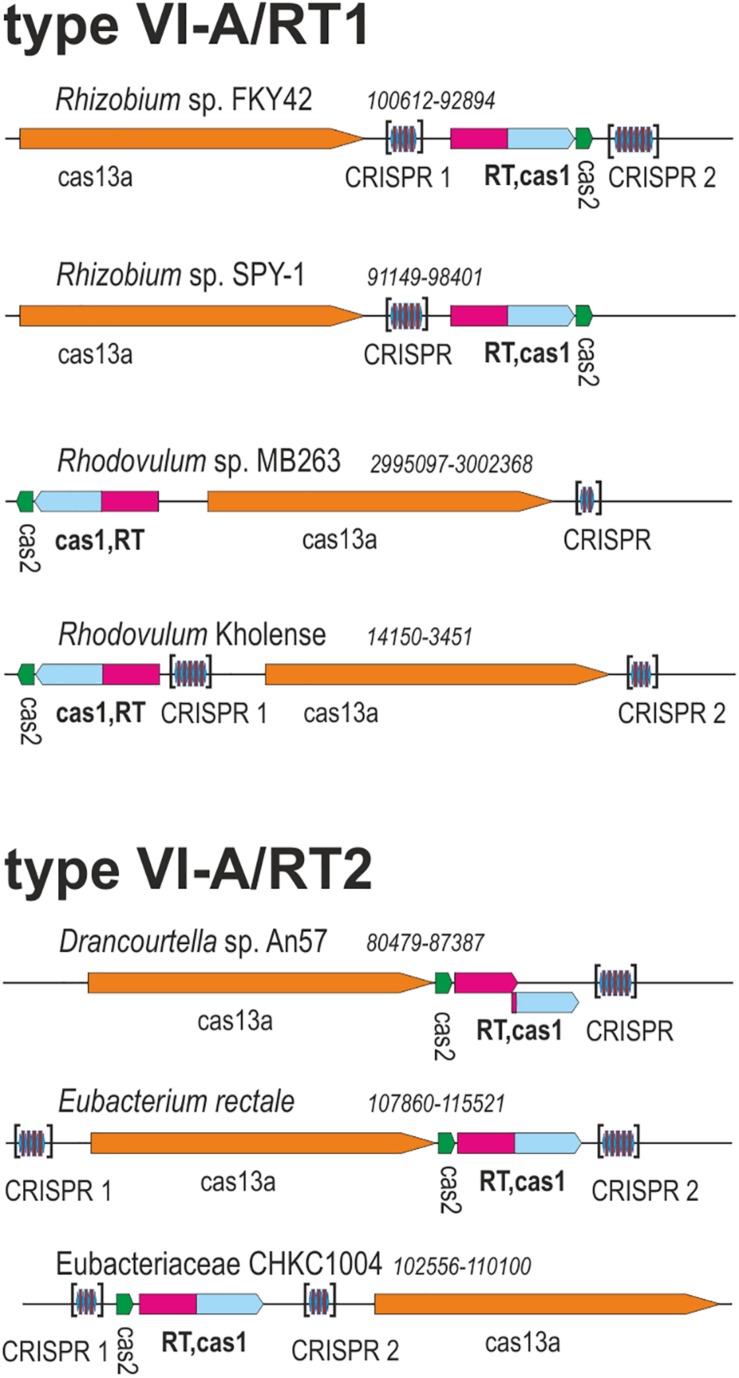
Architectures of genomic loci for the representative variants of type VI-A/RT1 and type VI-A/RT2 systems. For each locus, the species, nucleotide coordinates, and loci are indicated. Genes are shown roughly to scale; CRISPR arrays are indicated in brackets and are not shown to scale.

Two of these RT-Cas1 fusion proteins, from *Rhodovulum* sp. MB263 (WP_080615428.1) and Eubacteriaceae bacterium CHKCI004 (WP_090127495.1) (Figure 1), were already reported in a dataset of 9,141 unique RTs; in the CRISPR-Cas/RT phylogeny, they cluster in clades 7 and clade 12, respectively (Toro et al., 2019). These RT-Cas1 fusions were not previously classified within a particular CRISPR-Cas subtype because they lack the type III effector module characteristic of the corresponding RT clades. In addition to their different positions in the RT domain phylogeny, the two fusion proteins identified also appear to have Cas1 proteins of different origins, because all the RT-Cas1 sequences of clade 12 contain a distinctive type I-B CRISPR-associated endonuclease Cas1 (Toro et al., 2019). These findings suggest that the two variant type VI-A systems containing RTs may have evolved independently.

### Phylogenetic Relationships of the Cas13a Proteins of the Type VI-A Systems Encoding RTs

**FIGURE 2:**
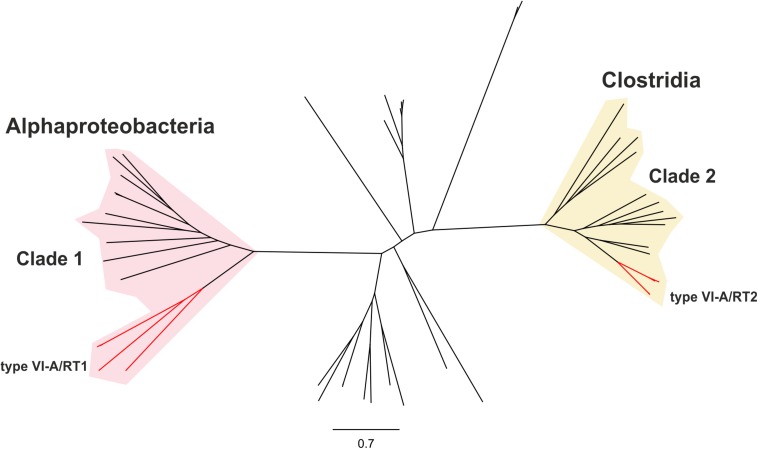
Phylogeny of Cas13a proteins. The unrooted tree was constructed with the FastTree program from an alignment of 81 unique predicted Cas13a proteins identified in genomics and metagenomics databases. The corresponding sequence, accession number, species name, status of the sequencing project, and FTP directory of GenBank assembly, when available are provided in Supplementary Table S1. The tree newick file is provided as Supplementary File S1. The branches corresponding to the Cas13a with an associated RT-Cas1 fusion denoted type VI-A/RT1 and type VI-A/RT2 are indicated in red. These Cas13a sequences split into two distinct clades clustering with other closely related Cas13a proteins lacking the RT-Cas1 fusion. Clade 1 is mostly restricted to Alphaproteobacteria and clade 2 to Clostridia.

We investigated the phylogenetic relationships of the Cas13a proteins of the type VI-A systems encoding RTs, by constructing a dataset of the Cas13a sequences described above (Supplementary Tables S1, S2). A phylogenetic tree was then constructed from a dataset containing 49 unique Cas13a sequences (Figure 2). The Cas13a from *Rhodovulum* sp. MB263 and those from *Rhodovulum kholense, Rhizobium* sp. SPY-1 and *Rhizobium* sp. FKY42, all with an associated RT-Cas1 protein, were grouped together (referred to hereafter as the type VI-A/RT1 system), whereas the Cas13a sequence from Eubacteriaceae bacterium CHKCI004 clustered with the Cas13a sequences from *Eubacterium rectale* strains and *Drancourtella* sp. An57, which also have an associated RT-Cas1 fusion (referred to hereafter as the type VI-A/RT2 system), with a frameshift mutation in the RT domain in the case of *Drancourtella* sp. An57 (Figure 1).

In *E. rectale*, the type VI-A CRISPR-Cas locus encoding the RT-Cas1 fusion protein was identified in strains AF25-15, AF18-16LB, and AF18-18LB, although, in the last of these strains, a genomic island of ∼22 kb separated the Cas2 gene from the RT-Cas1 genes. Other *E. rectale* strains presented frameshift mutations in the RT sequence (strains AF19-4, AF19-3AC, and AM29-10), or had lost the RT-Cas1 fusion (strain TM10-3) or the complete adaptation module (strain T1-815). The Cas13a protein of the T1-815 strain (EreCas13a) has been characterized in terms of its pre-cRNA processing and ribonuclease activities, and has been shown to cleave preferentially at adenosine residues during *trans*-ssRNA target cleavage (East-Seletsky et al., 2017). These findings suggest that the type VI-A CRISPR-Cas-associated genes encoding RT-Cas1 and Cas2 are frequently gained and lost, possibly due to the dynamics of microbial host-phage interactions.

The Cas13a protein sequences of the type VI-A/RT1 and VI-A/RT2 systems clustered separately into two distinct clades with other Cas13a sequences lacking RT-Cas1 (Figure 2), hereafter referred as to clades 1 and 2, respectively. Clade 1 comprises Cas13a sequences from bacteria from three families (Rhodobacteraceae, Rhodospirillaceae, and Rhizobiaceae) of the Alphaproteobacteria class plus one sequence from a bacterium from class Spirochaetia, possibly corresponding to a lateral transfer. By contrast, all members of clade 2 originate from bacteria from three families of class Clostridia (Lachnopiraceae, Ruminococcaceae, and Eubacteriaceae). These different phylogenetic and taxonomic distributions provide additional support for the hypothesis that the two RT-containing type VI-A variants arose independently during evolution.

### Recruitment of Adaptation Modules Containing a RT-Cas1 Fusion by Type VI-A-Systems

The Cas13a proteins of clade 1 are either associated with RT-Cas1 and Cas2, or they lack the adaptation module. Given that the closest RT-Cas1 relatives within clade 7 of the RT phylogeny are mostly associated with type III-D systems (Toro et al., 2019), it is plausible that CRISPR-Cas adaptation genes from one of these type III CRISPR-Cas loci were captured by a CRISPR/Cas13a system.

**FIGURE 3:**
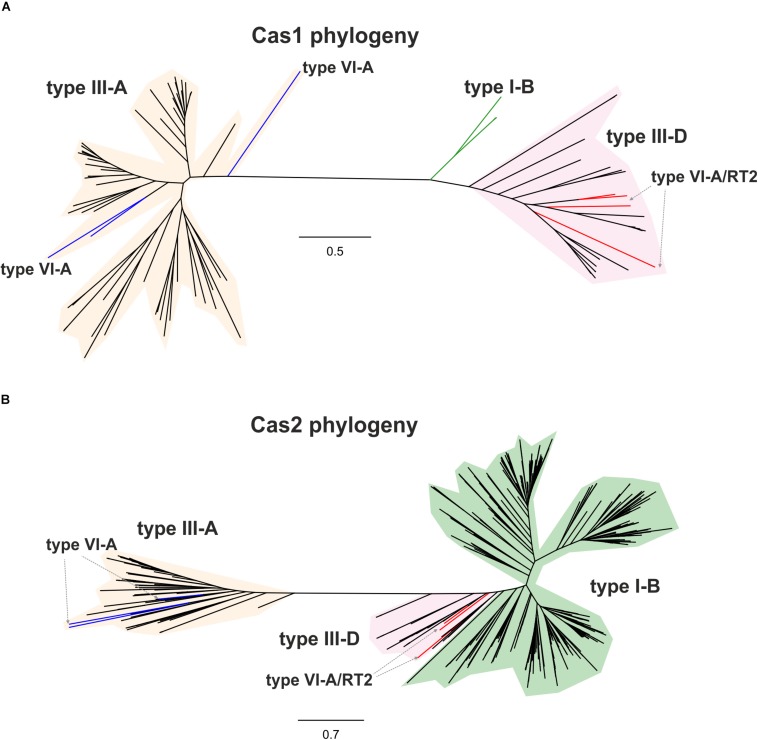
Phylogeny of RTCas1/Cas1 and Cas2 proteins associated with clade 2 Cas13a sequences. The unrooted trees were constructed with FastTree from alignments of 99 closely related Cas1 sequences **(A)**, and 537 closely related Cas2 sequences **(B)** mostly harbored by bacteria from phylum Firmicutes. The Cas1 and Cas2 sequences and their associated CRISPR-Cas loci are shown in Supplementary Tables S3, S4, respectively. The Cas1 and Cas2 tree newick files are provided as Supplementary Files S2, S3, respectively. Branches in red indicate the RT-Cas1 and adjacent Cas2 sequences associated with Cas13a proteins in type VI-A/RT2 systems, and branches in blue show Cas1 sequences lacking an RT domain and Cas2-associated sequences within clade 2. Other identified CRISPR-Cas systems, classified according to the interference module, are also specified.

By contrast, the Cas13a proteins of clade 2 that lack an RT-Cas1 fusion appear to contain a different adaptation module including the products of the *cas1* and *cas2* genes (Supplementary Table S2), probably reflecting separate capture events, potentially making it possible to trace the origin of these sequences and their phylogenetic relationships. We tested the evolutionary scenario proposed above, by first searching (phmmer) databases for sequences displaying similarity to those of the Cas1 and RT-Cas1 proteins linked to Cas13a within clade 2. We selected unique sequences and constructed a dataset of 99 closely related Cas1 sequences harbored mostly by bacteria from phylum Firmicutes (Supplementary Table S3). The phylogenetic tree (Figure 3A) inferred from a multiple sequence alignment (MSA) of these sequences clustered the Cas1 and RT-Cas1 sequences into two clearly differentiated groups. The MSA also revealed that some of the Cas1 proteins apparently lacking the RT domain had undergone RT loss events. This was the case for the *Butyrivibrio* sp. YAB3001 CRISPR-associated Cas1 protein, which still had some of the conserved amino-acid residues downstream from RT domain 7 at its N-terminus (data not shown). The Cas1 proteins not fused to an RT domain (i.e., CRISPR-associated endonuclease Cas1 from *Blautia* sp. Marseille-P2398, Lachnospiraceae bacterium NE2001 and *Clostridium aminophilum* strain F) clustered with other Cas1 sequences mostly linked to type III-A systems, whereas the RT-Cas1 sequences associated with Cas13a (i.e., the Eubacteriaceae bacterium CHKCI004, *E. rectale*, *Drancourtella* sp. An57 and *Butyrivibrio* sp. YAB3001) clustered with other RT-Cas1 sequences linked to type III-D systems. These findings highlight the diversity of adaptation modules captured by type VI-A systems and suggest that type VI systems within clade 2 captured the Cas1 and RT-Cas 1 endonucleases independently, possibly from adaptation modules of types III-A and III-D, respectively. Furthermore, several CRISPR-associated Cas1 proteins from different species of genus *Caldicellulosiruptor* linked to type I-B systems were found at the base of the cluster containing RT-Cas1 sequences, suggesting that the ancestor of the Cas1 domain of these RT-Cas1 fusions may have come from a type I-B adaptation module.

**FIGURE 4:**
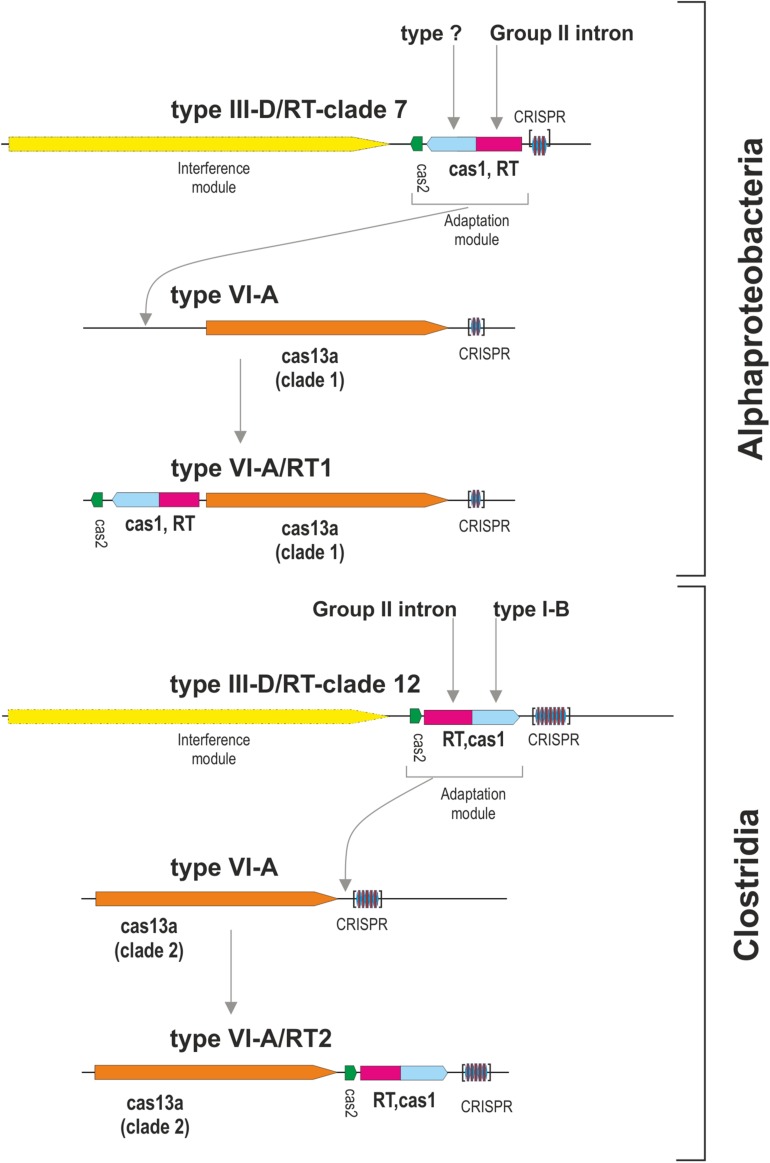
Origin of the type VI-A/RT1 and type VI-A/RT2 subtypes. The figure depicts a hypothetical scenario for the origin of adaptation modules for type VI-A systems containing an associated RT. Different adaptation modules containing RT-Cas1 and Cas2 proteins were captured independently, probably from type III-D systems, by phylogenetically different Cas13a-containing type VI CRISPR-Cas systems. Note that the interference module of type III systems encodes a multi-subunit effector complex and that the analysis of metagenomic datasets revealed that a variant of the type VI-A/RT1 system containing an Cas6-RT-Cas1 fusion may exist in uncultivated microorganisms.

We then tried to infer the origin and evolution of the Cas2 proteins associated with the Cas13a-containing type VI CRISPR-Cas systems within clade 2, through an approach similar to that described above for the RT-Cas1/Cas1 sequences, based on this sequence diversity. The Cas2 proteins linked to Cas13a within clade 2 were used as a query to search for Cas2 homologs. We constructed a dataset of 537 sequences from the results of this query (Supplementary Table S4). The taxonomic distribution was wider than expected in this dataset, but most of the sequences nevertheless came from phylum Firmicutes. The phylogenetic tree constructed (Figure 3B) grouped the Cas2 sequences of type VI-A/RT2 systems with other Cas2 sequences mostly linked to type III-D systems, whereas the Cas2 sequences of type VI-A systems, which lack the RT domain, were grouped together in a separate clade with other sequences mostly associated with type III-A systems. Thus, the Cas2 phylogeny is consistent with the RT-Cas1/Cas1 phylogeny, providing further evidence to suggest that the genes encoding the RT-Cas1 and Cas2 proteins of the type VI-A/RT2 system were captured together, possibly from a type III-D system, in a series of evolutionary events occurring in Clostridia (Figure 4). These results are consistent with the proposed evolutionary scenario of multiple independent acquisitions of adaptation modules by the Cas13-CRISPR containing type VI systems (Koonin and Makarova, 2019).

### Origin of the CRISPR Arrays Associated With Type VI-A/RT1 and RT2 Systems

**FIGURE 5:**
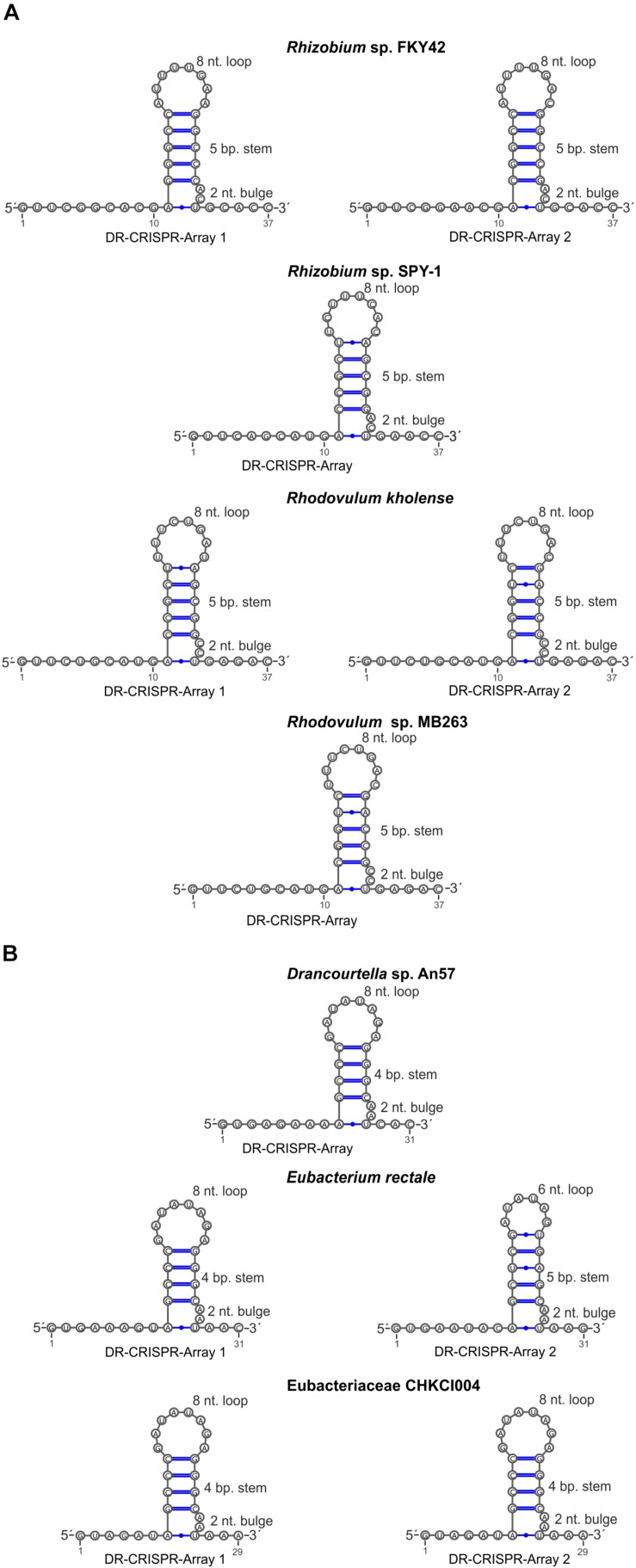
Sequence and predicted secondary structure of CRISPR array repeats in the putative pre-crRNA transcript. Typical short harpin-loop structures characteristic of type VI-A DRs for type VI-A/RT1 **(A)** and type VI-A/RT2 systems **(B)** are shown and the numbers of the arrays correspond to those indicated in Figure 1.

Type VI-A/RT1 and RT2 systems encompass either one or two CRISPR arrays, each containing two to six spacers of 30–49 nt in length (Figure 1). In type VI-A/RT1 systems, the direct repeats (DRs) are 6/8-nt longer (37-nt) than those of type VI-A/RT2 systems (29/31-nt). The spacers of these arrays did not yield significant matches to sequences in databases, with the exception of a few spacers from *E. rectale* that matched intergenic regions or other CRISPR arrays from the same species. Interestingly, the DRs of the type VI-A/RT1 and RT2 systems have a typical short harpin-loop structure characteristic of type VI-A DRs (O’Connell, 2019), with a loop region of 8 nt and a stem of five Watson–Crick base pairs in RT1 systems, and usually 1 nt less in the RT2 system. This stem is disrupted by an invariant 2-nt bulge: AC/CC in RT1 systems and AA in RT2 systems. The single-stranded regions upstream and downstream from the stem-loop required for initial Cas13a recognition and function are larger in the RT1 system (10/5-nt) than in the RT2 system (6/3-nt) (Figure 5). Together, these data indicate that the CRISPR arrays of type VI-A/RT1 and RT2 systems are characteristic of type VI-A systems. Their association with Cas13a therefore preceded the acquisition of the RT-Cas1/Cas2 adaptation modules.

### Type VI-A Systems With Linked RT-Cas1 Fusion Proteins From Uncultivated Microorganisms

With the aim of increasing the number and diversity of known type VI-A systems associated with RT-Cas1 fusion proteins, we used phmmer to search the MGnify protein sequence database^2^, which includes sequences predicted from assemblies generated from publicly available metagenomic datasets. This strategy identified 78 unique type VI-A/RT1 and RT2 Cas13a homologs from different environmental sources (Supplementary Table S5) and added to our initial dataset of 49 Cas13a sequences (Supplementary Table S1). The phylogenetic tree constructed from the MSA comprising these 127 sequences (Supplementary Figure S1 and Supplementary File S4) showed that most of the metagenomic Cas13a sequences clustered outside of clades 1 and 2, and an analysis of neighboring coding sequences in the corresponding Cas13a-containing contigs indicated that they lack an associated RT-Cas1 fusion. Nevertheless, we found three Cas13a metagenomic homologs within clade 2 (MGYP000178941783, MGYP000661043385, and MGYP000252681734) from the human gut metagenome with an associated RT-Cas1 fusion similar to that of type VI-A/RT2 systems. Surprisingly, we also identified a Cas13a sequence at the base of clade 1 (MGYP000128950304) that was obtained from sediment metagenome samples, the genome-assembly contigs of which harbored an associated Cas6-RT-Cas1 fusion (Supplementary Table S6). In the RT phylogeny, the fusions proteins with these three domains clustered in clades 8 and 11 (Toro et al., 2019). A phylogenetic tree constructed from a MSA comprising protein sequences from these clades and the Cas6-RT-Cas1 fusion associated with Cas13a indicated that this fusion protein belonged to clade 8 (data not shown). These findings reflect a highly dynamic association of different RT-Cas1 fusions with type VI-A systems and predict that the probable discovery of novel variants by the mining of metagenome datasets.

## Conclusion

We identified two different type VI-A systems with adaptation modules including RT-Cas1 fusion and Cas2 proteins, paving the way for further work investigating the RT-mediated acquisition of spacers for these CRISPR-Cas type VI loci. Phylogenetic reconstruction analyses revealed that these adaptation modules were recruited by different effector Cas13a proteins on independent occasions, possibly from type III-D systems from the bacterial classes Alphaproteobacteria and Clostridia. Our data expand the known types and subtypes of CRISPR-Cas systems with linked RTs, providing additional support for the highly dynamic association of RTs with CRISPR-Cas adaptive immune systems. Nevertheless, many issues remain concerning the association of an adaptation module carrying genes encoding RT-Cas1 and Cas2 proteins with type VI-systems, including the apparent restriction to type VI-A systems or to RT-Cas1 proteins clustered within RT clades 7 and 12 in isolated representatives, expanded to a Cas6-RT-Cas1 fusion from clade 8 in metagenomic datasets. We predict that the list of variants of type VI-A systems with linked RT-Cas1 fusions will be expanded in the future by the mining of metagenomics databases. Moreover, the processes by which these particular adaptation modules, like those observed in *E. rectale* strains, are gained and lost, and their possible relationship to self-targeting through the incorporation of spacers from the host or the ecological response of microbial populations to viral infection require further investigation.

Cas13 nucleases have been engineered as RNA-binding modules for diagnostic purposes and for RNA editing in diverse organisms (O’Connell, 2019). From a biotechnological perspective, our findings for type VI-A systems with an associated RT-Cas1 fusion should lead to further characterization of the mechanisms of spacer acquisition and *cis*- and *trans*-RNA cleavage activities, potentially providing novel and exciting opportunities in synthetic biology and engineering.

## Data Availability

All datasets generated for this study are included in the manuscript and/or the Supplementary Files.

## Author Contributions

NT came up with the idea for the study, performed the phylogenetic analyses, and wrote the manuscript with contributions from all the authors. MM and AG-D obtained protein sequences from databases. MM identified Cas13 homologs and identified the Cas13a neighboring genes with the previously designed computational pipeline. FM-A carried out manual inspection of the CRISPR-Cas loci.

## Conflict of Interest Statement

The authors declare that the research was conducted in the absence of any commercial or financial relationships that could be construed as a potential conflict of interest.

## Footnotes

1 http://www.ebi.ac.uk/Tools/hmmer/

2 https://www.ebi.ac.uk/metagenomics/
