# Supplementary material for: Recruitment of Reverse Transcriptase-Cas1 Fusion Proteins by Type VI-A CRISPR-Cas Systems
Source: Front Microbiol. 2019 Sep 13;10:2160. doi: 10.3389/fmicb.2019.02160 (PMC6753606; doi:10.3389/fmicb.2019.02160)
Supplement: Supplementary file 1 [file Data_Sheet_1.PDF]

Supplementary information

Recruitment of Reverse Transcriptases-Cas1 fusion proteins by type VI-A CRISPR-Cas systems

Nicolás Toro*, **Mario Rodríguez Mestre, Francisco Martínez-Abarca & Alejandro González-Delgado**

Structure, Dynamics and Function of Rhizobacterial Genomes (Grupo de Ecología Genética de la Rizosfera), Department of Soil Microbiology and Symbiotic Systems, Estación Experimental del Zaidín, Consejo Superior de Investigaciones Científicas, C/Profesor Albareda 1, 18008, Granada, Spain

***For correspondence**: nicolas.toro@eez.csic.es

Supplementary Figure 1: Phylogeny of Cas13a proteins including the metagenomics Cas13a homologs. The ID (Supplementary Table 5) of relevant sequences described in the main text are indicated.

Supplementary Table 1: List of Cas13a used in this study

Supplementary Table 2: Computational analysis of the neighborhood of Cas13a sequences belonging to genomic datasets used in Figure 2

Supplementary Table 3: Computational analysis of the neighborhood of Cas1 sequences used in Figure 3

Supplementary Table 4: Computational analysis of the neighborhood of Cas2 sequences used in Figure 3

Supplementary Table 5: List of Cas13a homologs from metagenomic datasets

Supplementary Table 6: Analysis of metagenomic Type VI-A/RT systems

Supplementary File 1: Newick file for representing tree shown in Figure 2

Supplementary File 2: Newick file for representing tree shown in Figure 3A

Supplementary File 3: Newick file for representing tree shown in Figure 3B

Supplementary File 4: Newick file for representing tree shown in Supplementary Figure 1

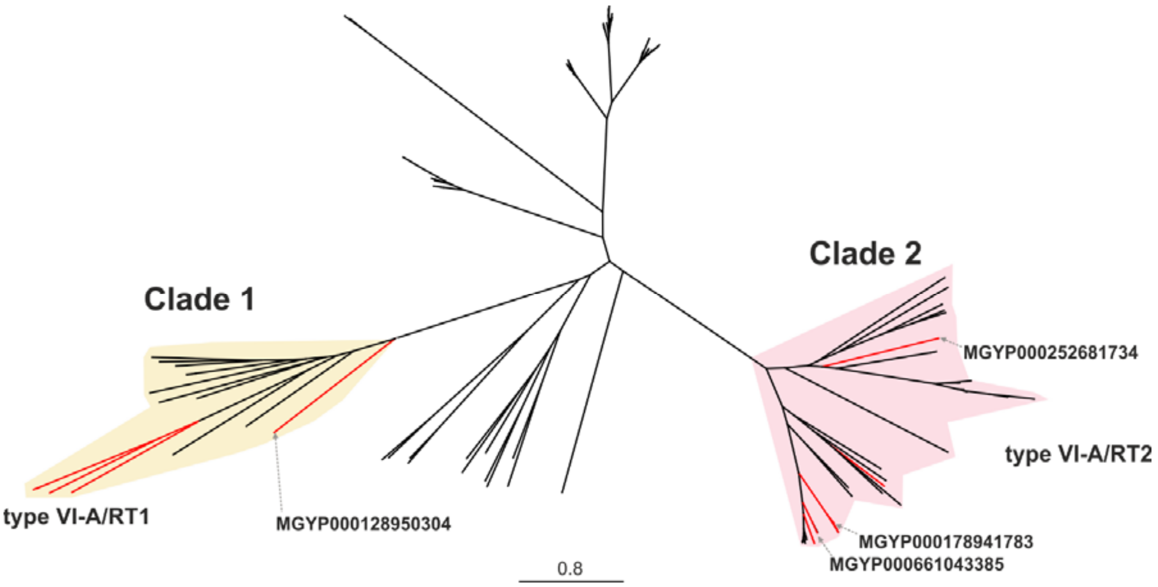

Supplementary Figure 1. Phylogeny of Cas13a proteins including the metagenomics Cas13a homologs. The ID (Supplementary Table 5) of relevant sequences described in the main text are indicated.

Table S1: List of Cas13a used in Figure 1

Clade	Node	Source	Nucleotide ID	Cas13a Protein ID	RTCas1[a]	Cas1	Cas2	Surrounding Arrays nº(repeats)	Trans CRISPR-Cas systems	Trans RTs (nº)	Status
	1	Herbinix hemicellulosilytica	NZ_QNRW01000010.1	**WP_103203632.1**				1(6)	2x acquisition modules	2	Contig
	2	Leptotrichia wadei F0279	NZ_KI271395.1	**WP_036059678.1**		WP_021746002.1	WP_021746001.1	1(3)		0	Scaffold
	3	Bacteroides ihuae	NZ_FNVX01000005.1	**WP_071146234.1**				1(13)		2	Contig
	4	Paludibacter propionicigenes	NC_014734.1	**WP_013443710.1**				1(27)		0	Complete Genome
	5	Carnobacterium gallinarum	NZ_JQLU01000005.1	**WP_034560163.1**				2(7/10)	I-B / Acquisition module	3	Contig
	6	Carnobacterium gallinarum	NZ_JQLU01000005.1	**WP_034563842.1**				2(7/8)		3	Contig
	7	Listeria newyorkensis	NZ_UAWS01000035.1	**WP_036091002.1**				1(5)		1	Contig
	8	Listeria weihenstephanensis	NZ_CP011102.1	**WP_118907415.1**				2(4/9)	I-B	3	Complete Genome
	9	Listeria costaricensis	NZ_FXUT01000037.1	**WP_099225408.1**				1(4)		1	Scaffold
	10	Listeria seeligeri	NC_013891.1	**WP_012985477.1**				1(6)	I-B/A	0	Complete Genome
1	11	Rhizobium sp. SPY-1	NZ_SMTL01000009.1	**WP_133318297.1**	WP_133318296.1		WP_133318295.1	1(6)		0	Contig
1	12	Rhizobium sp. FKY42	NZ_STGA01000016.1	**WP_137134457.1**	WP_137134456		WP_137134455.1	2(5/3)	I-E	1	Contig
1	13	Rhodovulum sp. MB263	NZ_CP020384.1	**WP_080615427.1**	WP_080615428.1		WP_080615428.1	1(3)	Acquisition module / I-E	0	Complete Genome
1	14	Rhodovulum kholense	NZ_QAYC01000027.1	**WP_108028905.1**	WP_108028906.1		WP_108028907.1	2(4/6)		1	Contig
1	15	Rhodobacter capsulatus	NZ_AYQC01000019.1	**WP_023911507.1**				1(5)	I-C	3	Scaffold
1	16	Ruegeria sp. 318-1	NZ_SMUV01000032.1	**WP_133357912.1**						0	Contig
1	17	Spirochaeta sp. LUC14_002_19_P3	MUIB01000034.1	**OQX30025.1**					V-B	0	Contig
1	18	Insolitispirillum peregrinum	NZ_FTOA01000001.1	**WP_076398593.1**				1(4)	I-E / II-C / I-C	0	Scaffold
1	19	Rhodovulum viride	NZ_MUAV01000038.1	**WP_112317339.1**				1(9)		1	Contig
1	20	Thalassospira sp. TSL5-1	NZ_KV880638.1	**WP_073955355.1**				1(6)		1	Scaffold
1	21	Thalassospira profundimaris	NZ_JPWH01000001.1	**WP_114086813.1**				1(20)		1	Contig
1	22	Rhodovulum steppense	NZ_SLVM01000007.1	**WP_132694182.1**				1(9)	I-E / I-E		Contig
1	23	Ferrovibrio sp.	PEKV01000005.1	**PJI41863.1**				1(3)		1	Scaffold
1	24	Bradyrhizobium sp. TSA1	NZ_LFJC01000003.1	**WP_100176879.1**						1	Scaffold
	25	Maledivibacter halophilus	NZ_FUZT01000022.1	**WP_079495749.1**		WP_079495748.1	WP_079495747.1			9	Scaffold
	26	Leptotrichia massiliensis	NZ_FNVZ01000004.1	**WP_071124126.1**		WP_071124127.1	WP_071124128.1	1(7)		0	Contig
	27	Leptotrichia wadei F0279	KI271424.1	**ERK47820.1**		ERK47819.1	ERK47818.1		Acquisition module	0	Scaffold
	28	Leptotrichia wadei	NZ_KI271421.1	**WP_021746774.1**		WP_021746773.1	WP_021746772.1	2(5/4)		0	Scaffold
	29	Leptotrichia buccalis	NC_013192.1	**WP_015770004.1**					I-B / III-D		Complete Genome
	30	Leptotrichia massiliensis	NZ_FNVZ01000005.1	**WP_071125398.1**				1(6)		0	Contig
	31	Leptotrichia sp. oral taxon 225	NZ_KI272904.1	**WP_021768357.1**						1	Scaffold
	32	Leptotrichia shahii	NZ_KB890278.1	**WP_018451595.1**		WP_083917144.1	WP_018451593.1	1(4)	I-B / III-A/D	0	Scaffold
	33	Leptotrichia sp. oral taxon 879	NZ_KI271320.1	**WP_021744063.1**		WP_021744062.1	WP_021744061.1		III-A	0	Scaffold
2	34	Ruminococcus sp. AM40-10AC	NZ_QUIR01000043.1	**WP_118572797.1**				1(7)	II-A / I-B / I-C / I-E	6	Scaffold
2	35	[Eubacterium] rectale AF19-3AC	NZ_QRWK01000025.1	**WP_117998314.1**	WP_117998320.1[b]	WP_117998323.1[b]	WP_117998317.1	1(8)		1	Scaffold
2	36	**Ruminococcus sp. TF11-2AC**	NZ_QUKI01000013.1	**WP_118614261.1**				1(5)	III-D / I-B	3	Scaffold
2	37	**Blautia sp. Marseille-P2398**	NZ_LT546010.1	**WP_062808098.1**		WP_062808097.1	WP_062808329.1	1(16)	III-A/D / I-B	6	Scaffold
2	38	*Lachnospiraceae bacterium NK4A179*	NZ_ATWC01000054.1	**WP_022785443.1**				2(4/7)	II-A	2	Contig
2	39	**Lachnospiraceae bacterium NE2001**	NZ_FOEK01000016.1	**WP_089928016.1**		WP_089928019.1	WP_089928054.1	1(10)		0	Scaffold
2	40	Butyrivibrio sp. YAB3001	NZ_FOKR01000002.1	**WP_092321585.1**		WP_092321588.1	WP_092321591.1	1(13)		2	Scaffold
2	41	*Lachnospiraceae bacterium MA2020*	NZ_JQKK01000015.1	**WP_044921188.1**		WP_081903028.1	WP_081903027.1	3(8/11/3)	II-A	1	Scaffold
2	42	**Pseudobutyrivibrio sp. OR37**	NZ_FOQF01000039.1	**WP_090551759.1**						1	Scaffold
2	43	*Lachnospiraceae bacterium NK4A144*	NZ_AUJT01000030.1	**WP_027114339.1**		WP_027114338.1	WP_044982681.1	2(7/4)	I-C / I-E	2	Scaffold
2	44	**[Clostridium] aminophilum**	NZ_FOZC01000010.1	**WP_031473346.1**		WP_081844215.1	WP_038288458.1	1(12)	III A/D	4	Scaffold
2	45	*Drancourtella sp. An57*	NZ_NFHY01000010.1	**WP_087253216.1**	WP_087253220.1[b]/WP_087253222.1[b]		WP_087253218.1	1(6)		1	Contig
2	46	**Eubacteriaceae bacterium CHKCI004**	NZ_FCNR01000048.1	**WP_090127496.1**	WP_090127495.1		WP_090127494.1	2(4/4)	III	7	Scaffold
2	47	[Eubacterium] rectale T1-815	NZ_CVRQ01000008.1	**WP_055061018.1**				2(5/8)		3	Contig
2	48	[Eubacterium] rectale TM10-3	NZ_QSOB01000012.1	**WP_117482613.1**			WP_117482614.1	1(6)		1	Scaffold
2	49	[Eubacterium] rectale AF25-15	NZ_QRUJ01000006.1	**WP_118003838.1**	WP_118003845.1		WP_117482614.1	1(5/6)		3	Scaffold

a) RT-Cas1 fusions are highlighted in red

b) A frameshift split RT and cas1 domains

Table S2: Computational analysis of the neighborhood of cas13a sequences belonging to genomic datasets used in Figure 2

Node	(Sub)type/Coordinates	Strand	Protein_id	Genome_partition	Genome_name	Domain	Phylum	Class	Order	Family	Genus	Description	cas_gene	profiles	subtype	repeats	avg_spacer_length	avg_repeat_length
1	CAS-VI-A | WP_103203632.1																	
	5466..5831	array		NZ_QNRW01000010.1	Herbinix hemicellulosilytica	Bacteria	Firmicutes	Clostridia	Clostridiales	Lachnospirac	Herbinix		CRISPR array			6	34	32
	5942..9800	-	WP_103203632.1	NZ_QNRW01000010.1	Herbinix hemicellulosilytica	Bacteria	Firmicutes	Clostridia	Clostridiales	Lachnospirac	Herbinix	CRISPR-associated endoribonuclease Cas13a	**cas13a**	cas13a	CAS-VI-A			
2	CAS-VI-A | ERK53440.1																	
	24882..25200	-	ERK53438.1	KI271395.1	Leptotrichia wadei F0279	Bacteria	Fusobacteria	Fusobacteriia	Fusobacterial	Leptotrichiac	Leptotrichia	CRISPR-associated endoribonuclease Cas2	cas2	mkCas0206	CAS-I,CAS-II,CAS-III			
	25189..26113	-	ERK53439.1	KI271395.1	Leptotrichia wadei F0279	Bacteria	Fusobacteria	Fusobacteriia	Fusobacterial	Leptotrichiac	Leptotrichia	CRISPR-associated endonuclease Cas1, NMENI subty	cas1	cd09720	CAS-II-A,CAS-II-B,CAS-II-C			
	26118..29667	-	ERK53440.1	KI271395.1	Leptotrichia wadei F0279	Bacteria	Fusobacteria	Fusobacteriia	Fusobacterial	Leptotrichiac	Leptotrichia	hypothetical protein	**cas13a**	cas13a	CAS-VI-A			
3	CAS-VI-A | WP_071146234.1																	
	492746..496163	+	WP_071146234.1	NZ_FNVX01000005.1	Bacteroides ihuae	Bacteria	Bacteroidetes	Bacteroidia	Bacteroidales	Bacteroidacea	Bacteroides	hypothetical protein	**cas13a**	cas13a	CAS-VI-A			
	496255..497082	array		NZ_FNVX01000005.1	Bacteroides ihuae	Bacteria	Bacteroidetes	Bacteroidia	Bacteroidales	Bacteroidacea	Bacteroides		CRISPR array			13	30	36
4	CAS-VI-A | WP_013443710.1																	
	223908..225657	array		NC_014734.1	Paludibacter propionicigenes	Bacteria	Bacteroidetes	Bacteroidia	Bacteroidales	Paludibactera	Paludibacter		CRISPR array			27	29	36
	225748..229213	-	WP_013443710.1	NC_014734.1	Paludibacter propionicigenes	Bacteria	Bacteroidetes	Bacteroidia	Bacteroidales	Paludibactera	Paludibacter	CRISPR-associated endoribonuclease Cas13a	**cas13a**	cas13a	CAS-VI-A			
5	CAS-VI-A | WP_034560163.1																	
	162240..165768	-	WP_034560163.1	NZ_JQLU01000005.1	Carnobacterium gallinarum	Bacteria	Firmicutes	Bacilli	Lactobacillale	Carnobacteria	Carnobacteriu	hypothetical protein	**cas13a**	cas13a	CAS-VI-A			
	165904..166335	array		NZ_JQLU01000005.1	Carnobacterium gallinarum	Bacteria	Firmicutes	Bacilli	Lactobacillale	Carnobacteria	Carnobacteriu		CRISPR array			7	30	36
	167669..168298	array		NZ_JQLU01000005.1	Carnobacterium gallinarum	Bacteria	Firmicutes	Bacilli	Lactobacillale	Carnobacteria	Carnobacteriu		CRISPR array			10	30	36
6	CAS-VI-A | WP_034563842.1																	
	2459220..2459651	array		NZ_JQLU01000005.1	Carnobacterium gallinarum	Bacteria	Firmicutes	Bacilli	Lactobacillale	Carnobacteria	Carnobacteriu		CRISPR array			7	30	36
	2459831..2463326	+	WP_034563842.1	NZ_JQLU01000005.1	Carnobacterium gallinarum	Bacteria	Firmicutes	Bacilli	Lactobacillale	Carnobacteria	Carnobacteriu	hypothetical protein	**cas13a**	cas13a	CAS-VI-A			
	2463599..2464096	array		NZ_JQLU01000005.1	Carnobacterium gallinarum	Bacteria	Firmicutes	Bacilli	Lactobacillale	Carnobacteria	Carnobacteriu		CRISPR array			8	30	36
7	CAS-VI-A | WP_036091002.1																	
	24712..25001	array		NZ_UAWS01000035.1	Listeria newyorkensis	Bacteria	Firmicutes	Bacilli	Bacillales	Listeriaceae	Listeria		CRISPR array			5	38	27
	25264..28420	+	WP_036091002.1	NZ_UAWS01000035.1	Listeria newyorkensis	Bacteria	Firmicutes	Bacilli	Bacillales	Listeriaceae	Listeria	hypothetical protein	**cas13a**	cas13a	CAS-VI-A			
8	CAS-VI-A | WP_118907415.1																	
	840040..840258	array		NZ_CP011102.1	Listeria weihenstephanensis	Bacteria	Firmicutes	Bacilli	Bacillales	Listeriaceae	Listeria		CRISPR array			4	45	21
	840536..843692	+	WP_118907415.1	NZ_CP011102.1	Listeria weihenstephanensis	Bacteria	Firmicutes	Bacilli	Bacillales	Listeriaceae	Listeria	hypothetical protein	**cas13a**	cas13a	CAS-VI-A			
	843755..844300	array		NZ_CP011102.1	Listeria weihenstephanensis	Bacteria	Firmicutes	Bacilli	Bacillales	Listeriaceae	Listeria		CRISPR array			9	46	19
9	CAS-VI-A | WP_099225408.1																	
	274..3514	+	WP_099225408.1	NZ_FXUT01000037.1	Listeria costaricensis	Bacteria	Firmicutes	Bacilli	Bacillales	Listeriaceae	Listeria	hypothetical protein	**cas13a**	cas13a	CAS-VI-A			
	3517..3747	array		NZ_FXUT01000037.1	Listeria costaricensis	Bacteria	Firmicutes	Bacilli	Bacillales	Listeriaceae	Listeria		CRISPR array			4	30	35
10	CAS-VI-A | WP_012985477.1																	
	1174057..1174422	array		NC_013891.1	Listeria seeligeri	Bacteria	Firmicutes	Bacilli	Bacillales	Listeriaceae	Listeria		CRISPR array			6	30	36
	1174427..1177790	+	WP_012985477.1	NC_013891.1	Listeria seeligeri	Bacteria	Firmicutes	Bacilli	Bacillales	Listeriaceae	Listeria	CRISPR-associated endoribonuclease Cas13a	**cas13a**	cas13a	CAS-VI-A			
11	CAS-VI-A | WP_133318297.1																	
	32739..33180	-	WP_133318295.1	NZ_SMTL01000009.1	Rhizobium sp. SPY-1	Bacteria	Proteobacteri	Alphaproteob	Rhizobiales	Rhizobiaceae	Rhizobium	CRISPR-associated endonuclease Cas2	cas2	pfam09827	CAS-I,CAS-II,CAS-III			
	33183..35160	-	WP_133318296.1	NZ_SMTL01000009.1	Rhizobium sp. SPY-1	Bacteria	Proteobacteri	Alphaproteob	Rhizobiales	Rhizobiaceae	Rhizobium	CRISPR-associated endonuclease Cas1	**RT,cas1**	pfam00078,cd09634	CAS-I,CAS-III			
	35207..35582	array		NZ_SMTL01000009.1	Rhizobium sp. SPY-1	Bacteria	Proteobacteri	Alphaproteob	Rhizobiales	Rhizobiaceae	Rhizobium		CRISPR array			6	30	37
	35720..39992	-	WP_133318297.1	NZ_SMTL01000009.1	Rhizobium sp. SPY-1	Bacteria	Proteobacteri	Alphaproteob	Rhizobiales	Rhizobiaceae	Rhizobium	hypothetical protein	**cas13a**	cas13a	CAS-VI-A			
12	CAS-VI-A | WP_137134456.1																	
	92894..93354	array		NZ_STGA01000016.1	Rhizobium sp. FKY42	Bacteria	Proteobacteri	Alphaproteob	Rhizobiales	Rhizobiaceae	Rhizobium		CRISPR array			7	33	37
	93549..93840	-	WP_137134455.1	NZ_STGA01000016.1	Rhizobium sp. FKY42	Bacteria	Proteobacteri	Alphaproteob	Rhizobiales	Rhizobiaceae	Rhizobium	CRISPR-associated endonuclease Cas2	cas2	cd09725	CAS-I,CAS-II,CAS-III,CAS-V			
	93845..95822	-	WP_137134456.1	NZ_STGA01000016.1	Rhizobium sp. FKY42	Bacteria	Proteobacteri	Alphaproteob	Rhizobiales	Rhizobiaceae	Rhizobium	CRISPR-associated endonuclease Cas1	**RT,cas1**	pfam00078,cd09634	CAS-I,CAS-III			
	95891..96203	array		NZ_STGA01000016.1	Rhizobium sp. FKY42	Bacteria	Proteobacteri	Alphaproteob	Rhizobiales	Rhizobiaceae	Rhizobium		CRISPR array			5	32	37
	96361..100612	-	WP_137134457.1	NZ_STGA01000016.1	Rhizobium sp. FKY42	Bacteria	Proteobacteri	Alphaproteob	Rhizobiales	Rhizobiaceae	Rhizobium	hypothetical protein	**cas13a**	cas13a	CAS-VI-A			
13	CAS-VI-A | WP_080615427.1																	
	858203..858375	array		NZ_CP020384.1	Rhodovulum sp. MB263	Bacteria	Proteobacteri	Alphaproteob	Rhodobactera	Rhodobactera	Rhodovulum		CRISPR array			3	31	37
	858500..862703	-	WP_080615427.1	NZ_CP020384.1	Rhodovulum sp. MB263	Bacteria	Proteobacteri	Alphaproteob	Rhodobactera	Rhodobactera	Rhodovulum	hypothetical protein	**cas13a**	cas13a	CAS-VI-A			
	863316..865179	+	WP_080615428.1	NZ_CP020384.1	Rhodovulum sp. MB263	Bacteria	Proteobacteri	Alphaproteob	Rhodobactera	Rhodobactera	Rhodovulum	CRISPR-associated endonuclease Cas1	**RT,cas1**	pfam00078,cd09634	CAS-I,CAS-III			
	865198..865474	+	WP_080615429.1	NZ_CP020384.1	Rhodovulum sp. MB263	Bacteria	Proteobacteri	Alphaproteob	Rhodobactera	Rhodobactera	Rhodovulum	CRISPR-associated endonuclease Cas2	cas2	cd09725	CAS-I,CAS-II,CAS-III,CAS-V			
14	CAS-VI-A | WP_108028905.1																	
	3451..3688	array		NZ_QAYC01000027.1	Rhodovulum kholense	Bacteria	Proteobacteri	Alphaproteob	Rhodobactera	Rhodobactera	Rhodovulum		CRISPR array			4	30	37
	3814..8005	-	WP_108028905.1	NZ_QAYC01000027.1	Rhodovulum kholense	Bacteria	Proteobacteri	Alphaproteob	Rhodobactera	Rhodobactera	Rhodovulum	hypothetical protein	**cas13a**	cas13a	CAS-VI-A			
	8332..8716	array		NZ_QAYC01000027.1	Rhodovulum kholense	Bacteria	Proteobacteri	Alphaproteob	Rhodobactera	Rhodobactera	Rhodovulum		CRISPR array			6	49	23
	8806..10777	+	WP_108028906.1	NZ_QAYC01000027.1	Rhodovulum kholense	Bacteria	Proteobacteri	Alphaproteob	Rhodobactera	Rhodobactera	Rhodovulum	CRISPR-associated endonuclease Cas1	**RT,cas1**	pfam00078,cd09634	CAS-I,CAS-III			
	10796..11072	+	WP_108028907.1	NZ_QAYC01000027.1	Rhodovulum kholense	Bacteria	Proteobacteri	Alphaproteob	Rhodobactera	Rhodobactera	Rhodovulum	CRISPR-associated endonuclease Cas2	cas2	cd09725	CAS-I,CAS-II,CAS-III,CAS-V			
15	CAS-VI-A | WP_023911507.1																	
	321133..321443	array		NZ_AYQC01000019.1	Rhodobacter capsulatus	Bacteria	Proteobacteri	Alphaproteob	Rhodobactera	Rhodobactera	Rhodobacter		CRISPR array			5	31	37
	321813..325671	+	WP_023911507.1	NZ_AYQC01000019.1	Rhodobacter capsulatus	Bacteria	Proteobacteri	Alphaproteob	Rhodobactera	Rhodobactera	Rhodobacter	hypothetical protein	**cas13a**	cas13a,cas13a	CAS-VI-A			
	326332..327637	+	WP_131725996.1	NZ_AYQC01000019.1	Rhodobacter capsulatus	Bacteria	Proteobacteri	Alphaproteob	Rhodobactera	Rhodobactera	Rhodobacter	hypothetical protein	CorA	cluster29				
16	CAS-VI-A | WP_133357912.1																	
	13717..17032	-	WP_133357912.1	NZ_SMUV01000032.1	Ruegeria sp. 318-1	Bacteria	Proteobacteri	Alphaproteob	Rhodobactera	Rhodobactera	Ruegeria	hypothetical protein	**cas13a**	cas13a,cas13a	CAS-VI-A			
17	CAS-VI-A | OQX30025.1																	
	3451..6857	+	OQX30025.1	MUIB01000034.1	Spirochaeta sp. LUC14_002_19_P3	Bacteria	Spirochaetes	Spirochaetia	Spirochaetale	Spirochaetac	Spirochaeta	hypothetical protein	**cas13a**	cas13a	CAS-VI-A			
18	CAS-VI-A | WP_076398593.1																	
	626412..626645	array		NZ_FTOA01000001.1	Insolitispirillum peregrinum	Bacteria	Proteobacteri	Alphaproteob	Rhodospirillal	Rhodospirillac	Insolitispirillu		CRISPR array			4	30	36

626724..629973	-	WP_076398593.1	NZ_FTOA01000001.1	Insolitispirillum peregrinum	Bacteria	Proteobacteri	Alphaproteob	Rhodospirillal	Rhodospirillac	Insolitispirillu	hypothetical protein	cas13a	cas13a	CAS-VI-A			
19 CAS-VI-A	WP_112317339.1																
1743..5295	-	WP_112317339.1	NZ_MUAV01000038.1	Rhodovulum viride	Bacteria	Proteobacteri	Alphaproteob	Rhodobactera	Rhodobactera	Rhodovulum	hypothetical protein	cas13a	cas13a,cas13a	CAS-VI-A			
5546..6130	array		NZ_MUAV01000038.1	Rhodovulum viride	Bacteria	Proteobacteri	Alphaproteob	Rhodobactera	Rhodobactera	Rhodovulum		CRISPR array			9	31	37
20 CAS-VI-A	WP_073955355.1																
551582..555290	+	WP_073955355.1	NZ_KV880638.1	Thalassospira sp. TSL5-1	Bacteria	Proteobacteri	Alphaproteob	Rhodospirillal	Rhodospirillac	Thalassospira	hypothetical protein	cas13a	cas13a	CAS-VI-A			
555342..555707	array		NZ_KV880638.1	Thalassospira sp. TSL5-1	Bacteria	Proteobacteri	Alphaproteob	Rhodospirillal	Rhodospirillac	Thalassospira		CRISPR array			6	30	36
21 CAS-VI-A	WP_114086813.1																
556739..560393	+	WP_114086813.1	NZ_JPWH01000001.1	Thalassospira profundimaris	Bacteria	Proteobacteri	Alphaproteob	Rhodospirillal	Rhodospirillac	Thalassospira	hypothetical protein	cas13a	cas13a	CAS-VI-A			
560545..561834	array		NZ_JPWH01000001.1	Thalassospira profundimaris	Bacteria	Proteobacteri	Alphaproteob	Rhodospirillal	Rhodospirillac	Thalassospira		CRISPR array			20	30	36
22 CAS-VI-A	WP_132694182.1																
61780..62348	array		NZ_SLVM01000007.1	Rhodovulum steppense	Bacteria	Proteobacteri	Alphaproteob	Rhodobactera	Rhodobactera	Rhodovulum		CRISPR array			9	30	36
62644..66475	+	WP_132694182.1	NZ_SLVM01000007.1	Rhodovulum steppense	Bacteria	Proteobacteri	Alphaproteob	Rhodobactera	Rhodobactera	Rhodovulum	hypothetical protein	cas13a	cas13a	CAS-VI-A			
23 CAS-VI-A	PJI41863.1																
64998..67962	+	PJI41863.1	PEKV01000005.1	Ferrovibrio sp.	Bacteria	Proteobacteri	Alphaproteob	Rhodospirillal	Rhodospirillac	Ferrovibrio	hypothetical protein	cas13a	cas13a	CAS-VI-A			
68250..68415	array		PEKV01000005.1	Ferrovibrio sp.	Bacteria	Proteobacteri	Alphaproteob	Rhodospirillal	Rhodospirillac	Ferrovibrio		CRISPR array			3	30	35
24 CAS-VI-A	WP_100176879.1																
2829952..2833651	+	WP_100176879.1	NZ_LFJC01000003.1	Bradyrhizobium sp. TSA1	Bacteria	Proteobacteri	Alphaproteob	Rhizobiales	Bradyrhizobia	Bradyrhizobiu	hypothetical protein	cas13a	cas13a	CAS-VI-A			
25 CAS-VI-A	WP_079495749.1																
9298..9625	-	WP_079495747.1	NZ_FUZT01000022.1	Maledivibacter halophilus	Bacteria	Firmicutes	Clostridia	Clostridiales	Clostridiaceae	Maledivibacte	CRISPR-associated endonuclease Cas2	cas2	COG3512	CAS-II-A,CAS-II-B,CAS-II-C			
9614..10517	-	WP_079495748.1	NZ_FUZT01000022.1	Maledivibacter halophilus	Bacteria	Firmicutes	Clostridia	Clostridiales	Clostridiaceae	Maledivibacte	type II CRISPR-associated endonuclease Cas1	cas1	cd09720	CAS-II-A,CAS-II-B,CAS-II-C			
10544..14420	-	WP_079495749.1	NZ_FUZT01000022.1	Maledivibacter halophilus	Bacteria	Firmicutes	Clostridia	Clostridiales	Clostridiaceae	Maledivibacte	hypothetical protein	cas13a	cas13a,cas13a	CAS-VI-A			
26 CAS-VI-A	WP_071124126.1																
398572..401980	+	WP_071124126.1	NZ_FNVZ01000004.1	Leptotrichia massiliensis	Bacteria	Fusobacteria	Fusobacteriia	Fusobacterial	Leptotrichiace	Leptotrichia	hypothetical protein	cas13a	cas13a	CAS-VI-A			
401995..402910	+	WP_071124127.1	NZ_FNVZ01000004.1	Leptotrichia massiliensis	Bacteria	Fusobacteria	Fusobacteriia	Fusobacterial	Leptotrichiace	Leptotrichia	type II CRISPR-associated endonuclease Cas1	cas1	cd09720	CAS-II-A,CAS-II-B,CAS-II-C			
402899..403217	+	WP_071124128.1	NZ_FNVZ01000004.1	Leptotrichia massiliensis	Bacteria	Fusobacteria	Fusobacteriia	Fusobacterial	Leptotrichiace	Leptotrichia	CRISPR-associated endonuclease Cas2	cas2	mkCas0206	CAS-I,CAS-II,CAS-III			
403357..403797	array		NZ_FNVZ01000004.1	Leptotrichia massiliensis	Bacteria	Fusobacteria	Fusobacteriia	Fusobacterial	Leptotrichiace	Leptotrichia		CRISPR array			7	30	37
27 CAS-VI-A	ERK47820.1																
222710..223040	-	ERK47818.1	KI271424.1	Leptotrichia wadei F0279	Bacteria	Fusobacteria	Fusobacteriia	Fusobacterial	Leptotrichiace	Leptotrichia	CRISPR-associated endoribonuclease Cas2	cas2	mkCas0206	CAS-I,CAS-II,CAS-III			
223023..223941	-	ERK47819.1	KI271424.1	Leptotrichia wadei F0279	Bacteria	Fusobacteria	Fusobacteriia	Fusobacterial	Leptotrichiace	Leptotrichia	CRISPR-associated endonuclease Cas1, NMENI subty	cas1	cd09720	CAS-II-A,CAS-II-B,CAS-II-C			
223960..227554	-	ERK47820.1	KI271424.1	Leptotrichia wadei F0279	Bacteria	Fusobacteria	Fusobacteriia	Fusobacterial	Leptotrichiace	Leptotrichia	hypothetical protein	cas13a	cas13a	CAS-VI-A			
28 CAS-VI-A	WP_021746774.1																
63701..64006	array		NZ_KI271421.1	Leptotrichia wadei	Bacteria	Fusobacteria	Fusobacteriia	Fusobacterial	Leptotrichiace	Leptotrichia		CRISPR array			5	29	38
64094..64331	array		NZ_KI271421.1	Leptotrichia wadei	Bacteria	Fusobacteria	Fusobacteriia	Fusobacterial	Leptotrichiace	Leptotrichia		CRISPR array			4	31	36
64575..64899	-	WP_021746772.1	NZ_KI271421.1	Leptotrichia wadei	Bacteria	Fusobacteria	Fusobacteriia	Fusobacterial	Leptotrichiace	Leptotrichia	CRISPR-associated endonuclease Cas2	cas2	COG3512	CAS-II-A,CAS-II-B,CAS-II-C			
64888..65800	-	WP_021746773.1	NZ_KI271421.1	Leptotrichia wadei	Bacteria	Fusobacteria	Fusobacteriia	Fusobacterial	Leptotrichiace	Leptotrichia	type II CRISPR-associated endonuclease Cas1	cas1	cd09720	CAS-II-A,CAS-II-B,CAS-II-C			
65813..69272	-	WP_021746774.1	NZ_KI271421.1	Leptotrichia wadei	Bacteria	Fusobacteria	Fusobacteriia	Fusobacterial	Leptotrichiace	Leptotrichia	hypothetical protein	cas13a	cas13a	CAS-VI-A			
29 CAS-VI-A	WP_015770004.1																
1880458..1883938	-	WP_015770004.1	NC_013192.1	Leptotrichia buccalis	Bacteria	Fusobacteria	Fusobacteriia	Fusobacterial	Leptotrichiace	Leptotrichia	CRISPR-associated endoribonuclease Cas13a	cas13a	cas13a	CAS-VI-A			
30 CAS-VI-A	WP_071125398.1																
1528875..1532382	+	WP_071125398.1	NZ_FNVZ01000005.1	Leptotrichia massiliensis	Bacteria	Fusobacteria	Fusobacteriia	Fusobacterial	Leptotrichiace	Leptotrichia	hypothetical protein	cas13a	cas13a	CAS-VI-A			
1532639..1533013	array		NZ_FNVZ01000005.1	Leptotrichia massiliensis	Bacteria	Fusobacteria	Fusobacteriia	Fusobacterial	Leptotrichiace	Leptotrichia		CRISPR array			6	31	36
31 CAS-VI-A	WP_021768357.1																
13871..16613	+	WP_021768357.1	NZ_KI272904.1	Leptotrichia sp. oral taxon 225	Bacteria	Fusobacteria	Fusobacteriia	Fusobacterial	Leptotrichiace	Leptotrichia	hypothetical protein	cas13a	cas13a	CAS-VI-A			
32 CAS-VI-A	WP_018451595.1																
34127..34365	array		NZ_KB890278.1	Leptotrichia shahii	Bacteria	Fusobacteria	Fusobacteriia	Fusobacterial	Leptotrichiace	Leptotrichia		CRISPR array			4	31	36
34504..34822	-	WP_018451593.1	NZ_KB890278.1	Leptotrichia shahii	Bacteria	Fusobacteria	Fusobacteriia	Fusobacterial	Leptotrichiace	Leptotrichia	CRISPR-associated endonuclease Cas2	cas2	mkCas0206	CAS-I,CAS-II,CAS-III			
34811..35753	-	WP_083917144.1	NZ_KB890278.1	Leptotrichia shahii	Bacteria	Fusobacteria	Fusobacteriia	Fusobacterial	Leptotrichiace	Leptotrichia	type II CRISPR-associated endonuclease Cas1	cas1	cd09720	CAS-II-A,CAS-II-B,CAS-II-C			
35755..39925	-	WP_018451595.1	NZ_KB890278.1	Leptotrichia shahii	Bacteria	Fusobacteria	Fusobacteriia	Fusobacterial	Leptotrichiace	Leptotrichia	type VI-A CRISPR-associated effector C2c2	cas13a	cas13a	CAS-VI-A			
33 CAS-VI-A	WP_021744063.1																
15428..15746	-	WP_021744061.1	NZ_KI271320.1	Leptotrichia sp. oral taxon 879	Bacteria	Fusobacteria	Fusobacteriia	Fusobacterial	Leptotrichiace	Leptotrichia	CRISPR-associated endonuclease Cas2	cas2	mkCas0206	CAS-I,CAS-II,CAS-III			
15735..16677	-	WP_021744062.1	NZ_KI271320.1	Leptotrichia sp. oral taxon 879	Bacteria	Fusobacteria	Fusobacteriia	Fusobacterial	Leptotrichiace	Leptotrichia	type II CRISPR-associated endonuclease Cas1	cas1	cd09720	CAS-II-A,CAS-II-B,CAS-II-C			
16679..20837	-	WP_021744063.1	NZ_KI271320.1	Leptotrichia sp. oral taxon 879	Bacteria	Fusobacteria	Fusobacteriia	Fusobacterial	Leptotrichiace	Leptotrichia	type VI-A CRISPR-associated effector C2c2	cas13a	cas13a	CAS-VI-A			
34 CAS-VI-A	WP_118572797.1																
22759..26923	-	WP_118572797.1	NZ_QUIR01000043.1	Ruminococcus sp. AM40-10AC	Bacteria	Firmicutes	Clostridia	Clostridiales	Ruminococca	Ruminococcu	hypothetical protein	cas13a	cas13a	CAS-VI-A			
27014..29114	-	WP_118572798.1	NZ_QUIR01000043.1	Ruminococcus sp. AM40-10AC	Bacteria	Firmicutes	Clostridia	Clostridiales	Ruminococca	Ruminococcu	hypothetical protein						
29184..31389	-	WP_118572799.1	NZ_QUIR01000043.1	Ruminococcus sp. AM40-10AC	Bacteria	Firmicutes	Clostridia	Clostridiales	Ruminococca	Ruminococcu	hypothetical protein						
31324..33094	-	WP_118572800.1	NZ_QUIR01000043.1	Ruminococcus sp. AM40-10AC	Bacteria	Firmicutes	Clostridia	Clostridiales	Ruminococca	Ruminococcu	hypothetical protein						
33544..34019	array		NZ_QUIR01000043.1	Ruminococcus sp. AM40-10AC	Bacteria	Firmicutes	Clostridia	Clostridiales	Ruminococca	Ruminococcu		CRISPR array			7	36	37
35 CAS-VI-A	WP_117998314.1																
11122..14929	+	WP_117998314.1	NZ_QRWK01000025.1	[Eubacterium] rectale	Bacteria	Firmicutes	Clostridia	Clostridiales	Lachnospiraceae		hypothetical protein	cas13a	cas13a	CAS-VI-A			
14932..15193	+	WP_117998317.1	NZ_QRWK01000025.1	[Eubacterium] rectale	Bacteria	Firmicutes	Clostridia	Clostridiales	Lachnospiraceae		CRISPR-associated endonuclease Cas2	cas2	cd09725	CAS-I,CAS-II,CAS-III,CAS-V			
15179..15911	+	WP_117998320.1	NZ_QRWK01000025.1	[Eubacterium] rectale	Bacteria	Firmicutes	Clostridia	Clostridiales	Lachnospiraceae		hypothetical protein	**RT**	pfam00078	CAS-I,CAS-III			
15769..16903	+	WP_117998323.1	NZ_QRWK01000025.1	[Eubacterium] rectale	Bacteria	Firmicutes	Clostridia	Clostridiales	Lachnospiraceae		CRISPR-associated endonuclease Cas1	**cas1**	cd09722	CAS-I-B			
17068..17588	array		NZ_QRWK01000025.1	[Eubacterium] rectale	Bacteria	Firmicutes	Clostridia	Clostridiales	Lachnospiraceae			CRISPR array			8	39	31
36 CAS-VI-A	WP_118614261.1																
79799..83876	-	WP_118614261.1	NZ_QUKI01000013.1	Ruminococcus sp. TF11-2AC	Bacteria	Firmicutes	Clostridia	Clostridiales	Ruminococca	Ruminococcu	hypothetical protein	cas13a	cas13a	CAS-VI-A			

83965..84382	-	WP_118614263.1	NZ_QUKI01000013.1	Ruminococcus sp. TF11-2AC	Bacteria	Firmicutes	Clostridia	Clostridiales	Ruminococca	Ruminococcu	hypothetical protein						
84518..84841	array		NZ_QUKI01000013.1	Ruminococcus sp. TF11-2AC	Bacteria	Firmicutes	Clostridia	Clostridiales	Ruminococca	Ruminococcu		CRISPR array			5	34	37
37 CAS-VI-A | WP_062808098.1																	
1869431..1869746	-	WP_062808329.1	NZ_LT546010.1	Blautia sp. Marseille-P2398	Bacteria	Firmicutes	Clostridia	Clostridiales	Lachnospirace	Blautia	CRISPR-associated endonuclease Cas2	cas2	cd09725	CAS-I,CAS-II,CAS-III,CAS-V			
1869756..1870758	-	WP_062808097.1	NZ_LT546010.1	Blautia sp. Marseille-P2398	Bacteria	Firmicutes	Clostridia	Clostridiales	Lachnospirace	Blautia	CRISPR-associated endonuclease Cas1	cas1	cd09634	CAS-I,CAS-II,CAS-III,CAS-V			
1870784..1871888	array		NZ_LT546010.1	Blautia sp. Marseille-P2398	Bacteria	Firmicutes	Clostridia	Clostridiales	Lachnospirace	Blautia		CRISPR array			16	35	36
1872030..1875861	-	WP_062808098.1	NZ_LT546010.1	Blautia sp. Marseille-P2398	Bacteria	Firmicutes	Clostridia	Clostridiales	Lachnospirace	Blautia	hypothetical protein	cas13a	cas13a	CAS-VI-A			
38 CAS-VI-A | WP_022785443.1																	
48..289	array		NZ_ATWC01000054.1	Lachnospiraceae bacterium NK4A179	Bacteria	Firmicutes	Clostridia	Clostridiales	Lachnospiraceae			CRISPR array			4	32	36
492..1173	-	WP_022785439.1	NZ_ATWC01000054.1	Lachnospiraceae bacterium NK4A179	Bacteria	Firmicutes	Clostridia	Clostridiales	Lachnospiraceae		DUF4230 domain-containing protein						
1272..2298	-	WP_022785440.1	NZ_ATWC01000054.1	Lachnospiraceae bacterium NK4A179	Bacteria	Firmicutes	Clostridia	Clostridiales	Lachnospiraceae		WYL domain-containing protein	WYL	pfam13280	CAS-I,CAS-III			
2294..3581	-	WP_022785441.1	NZ_ATWC01000054.1	Lachnospiraceae bacterium NK4A179	Bacteria	Firmicutes	Clostridia	Clostridiales	Lachnospiraceae		WYL domain-containing protein	WYL	pfam13280	CAS-I,CAS-III			
3604..5830	-	WP_022785442.1	NZ_ATWC01000054.1	Lachnospiraceae bacterium NK4A179	Bacteria	Firmicutes	Clostridia	Clostridiales	Lachnospiraceae		hypothetical protein						
5841..10155	-	WP_022785443.1	NZ_ATWC01000054.1	Lachnospiraceae bacterium NK4A179	Bacteria	Firmicutes	Clostridia	Clostridiales	Lachnospiraceae		CRISPR-associated endoribonuclease Cas13a	cas13a	cas13a	CAS-VI-A			
10151..10337	-	WP_022785444.1	NZ_ATWC01000054.1	Lachnospiraceae bacterium NK4A179	Bacteria	Firmicutes	Clostridia	Clostridiales	Lachnospiraceae		hypothetical protein						
10469..10913	array		NZ_ATWC01000054.1	Lachnospiraceae bacterium NK4A179	Bacteria	Firmicutes	Clostridia	Clostridiales	Lachnospiraceae			CRISPR array			7	33	35
39 CAS-VI-A | WP_089928016.1																	
72908..77072	+	WP_089928016.1	NZ_FOEK01000016.1	Lachnospiraceae bacterium NE2001	Bacteria	Firmicutes	Clostridia	Clostridiales	Lachnospiraceae		hypothetical protein	cas13a	cas13a	CAS-VI-A			
77161..78199	+	WP_089928019.1	NZ_FOEK01000016.1	Lachnospiraceae bacterium NE2001	Bacteria	Firmicutes	Clostridia	Clostridiales	Lachnospiraceae		CRISPR-associated endonuclease Cas1	cas1	cd09634	CAS-I,CAS-II,CAS-III,CAS-V			
78207..78495	+	WP_089928054.1	NZ_FOEK01000016.1	Lachnospiraceae bacterium NE2001	Bacteria	Firmicutes	Clostridia	Clostridiales	Lachnospiraceae		CRISPR-associated endonuclease Cas2	cas2	cd09725	CAS-I,CAS-II,CAS-III,CAS-V			
78625..79288	array		NZ_FOEK01000016.1	Lachnospiraceae bacterium NE2001	Bacteria	Firmicutes	Clostridia	Clostridiales	Lachnospiraceae			CRISPR array			10	33	36
40 CAS-VI-A | WP_092321585.1																	
6257..10289	+	WP_092321585.1	NZ_FOKR01000002.1	Butyrivibrio sp. YAB3001	Bacteria	Firmicutes	Clostridia	Clostridiales	Lachnospirace	Butyrivibrio	hypothetical protein	cas13a	cas13a	CAS-VI-A			
10440..11302	array		NZ_FOKR01000002.1	Butyrivibrio sp. YAB3001	Bacteria	Firmicutes	Clostridia	Clostridiales	Lachnospirace	Butyrivibrio		CRISPR array			13	32	36
11477..12524	+	WP_092321588.1	NZ_FOKR01000002.1	Butyrivibrio sp. YAB3001	Bacteria	Firmicutes	Clostridia	Clostridiales	Lachnospirace	Butyrivibrio	CRISPR-associated endonuclease Cas1	cas1	cd09722	CAS-I-B			
12537..12798	+	WP_092321591.1	NZ_FOKR01000002.1	Butyrivibrio sp. YAB3001	Bacteria	Firmicutes	Clostridia	Clostridiales	Lachnospirace	Butyrivibrio	CRISPR-associated endonuclease Cas2	cas2	cd09725	CAS-I,CAS-II,CAS-III,CAS-V			
41 CAS-VI-A | WP_044921188.1																	
89513..90259	array		NZ_JQKK01000015.1	Lachnospiraceae bacterium MA2020	Bacteria	Firmicutes	Clostridia	Clostridiales	Lachnospiraceae			CRISPR array			11	36	35
90406..90658	-	WP_081903027.1	NZ_JQKK01000015.1	Lachnospiraceae bacterium MA2020	Bacteria	Firmicutes	Clostridia	Clostridiales	Lachnospiraceae		CRISPR-associated endonuclease Cas2	cas2	cd09725	CAS-I,CAS-II,CAS-III,CAS-V			
90638..90788	-	WP_081903028.1	NZ_JQKK01000015.1	Lachnospiraceae bacterium MA2020	Bacteria	Firmicutes	Clostridia	Clostridiales	Lachnospiraceae		hypothetical protein	cas1	pfam01867	CAS-I,CAS-II,CAS-III			
90804..94827	-	WP_044921188.1	NZ_JQKK01000015.1	Lachnospiraceae bacterium MA2020	Bacteria	Firmicutes	Clostridia	Clostridiales	Lachnospiraceae		hypothetical protein	cas13a	cas13a	CAS-VI-A			
42 CAS-VI-A | WP_090551759.1																	
18445..22384	-	WP_090551759.1	NZ_FOQF01000039.1	Pseudobutyrivibrio sp. OR37	Bacteria	Firmicutes	Clostridia	Clostridiales	Lachnospirace	Pseudobutyri	hypothetical protein	cas13a	cas13a	CAS-VI-A			
43 CAS-VI-A | WP_027114339.1																	
11361..12360	+	WP_027114338.1	NZ_AUJT01000030.1	Lachnospiraceae bacterium NK4A144	Bacteria	Firmicutes	Clostridia	Clostridiales	Lachnospiraceae		CRISPR-associated endonuclease Cas1	cas1	cd09634	CAS-I,CAS-II,CAS-III,CAS-V			
12364..12649	+	WP_044982681.1	NZ_AUJT01000030.1	Lachnospiraceae bacterium NK4A144	Bacteria	Firmicutes	Clostridia	Clostridiales	Lachnospiraceae		CRISPR-associated endonuclease Cas2	cas2	cd09725	CAS-I,CAS-II,CAS-III,CAS-V			
12861..13319	array		NZ_AUJT01000030.1	Lachnospiraceae bacterium NK4A144	Bacteria	Firmicutes	Clostridia	Clostridiales	Lachnospiraceae			CRISPR array			7	35	35
13595..17600	+	WP_027114339.1	NZ_AUJT01000030.1	Lachnospiraceae bacterium NK4A144	Bacteria	Firmicutes	Clostridia	Clostridiales	Lachnospiraceae		hypothetical protein	cas13a	cas13a	CAS-VI-A			
17681..19403	+	WP_027114340.1	NZ_AUJT01000030.1	Lachnospiraceae bacterium NK4A144	Bacteria	Firmicutes	Clostridia	Clostridiales	Lachnospiraceae		hypothetical protein						
19399..21382	+	WP_027114341.1	NZ_AUJT01000030.1	Lachnospiraceae bacterium NK4A144	Bacteria	Firmicutes	Clostridia	Clostridiales	Lachnospiraceae		hypothetical protein						
21382..23560	+	WP_027114342.1	NZ_AUJT01000030.1	Lachnospiraceae bacterium NK4A144	Bacteria	Firmicutes	Clostridia	Clostridiales	Lachnospiraceae		hypothetical protein						
23724..24537	+	WP_027114343.1	NZ_AUJT01000030.1	Lachnospiraceae bacterium NK4A144	Bacteria	Firmicutes	Clostridia	Clostridiales	Lachnospiraceae		hypothetical protein						
24566..24761	+	WP_027114344.1	NZ_AUJT01000030.1	Lachnospiraceae bacterium NK4A144	Bacteria	Firmicutes	Clostridia	Clostridiales	Lachnospiraceae		hypothetical protein						
24932..25169	array		NZ_AUJT01000030.1	Lachnospiraceae bacterium NK4A144	Bacteria	Firmicutes	Clostridia	Clostridiales	Lachnospiraceae			CRISPR array			4	43	27
44 CAS-VI-A | WP_031473346.1																	
1530..2330	array		NZ_FOZC01000010.1	[Clostridium] aminophilum	Bacteria	Firmicutes	Clostridia	Clostridiales	Lachnospirace	Lachnoclostri		CRISPR array			12	34	35
2499..2781	+	WP_031473334.1	NZ_FOZC01000010.1	[Clostridium] aminophilum	Bacteria	Firmicutes	Clostridia	Clostridiales	Lachnospirace	Lachnoclostri	hypothetical protein						
2976..3984	+	WP_081844215.1	NZ_FOZC01000010.1	[Clostridium] aminophilum	Bacteria	Firmicutes	Clostridia	Clostridiales	Lachnospirace	Lachnoclostri	CRISPR-associated endonuclease Cas1	cas1	cd09634	CAS-I,CAS-II,CAS-III,CAS-V			
4003..4276	+	WP_038288458.1	NZ_FOZC01000010.1	[Clostridium] aminophilum	Bacteria	Firmicutes	Clostridia	Clostridiales	Lachnospirace	Lachnoclostri	CRISPR-associated endonuclease Cas2	cas2	pfam09827	CAS-I,CAS-II,CAS-III			
4444..6202	+	WP_031473340.1	NZ_FOZC01000010.1	[Clostridium] aminophilum	Bacteria	Firmicutes	Clostridia	Clostridiales	Lachnospirace	Lachnoclostri	hypothetical protein						
6191..7937	+	WP_031473342.1	NZ_FOZC01000010.1	[Clostridium] aminophilum	Bacteria	Firmicutes	Clostridia	Clostridiales	Lachnospirace	Lachnoclostri	hypothetical protein						
7937..10130	+	WP_031473344.1	NZ_FOZC01000010.1	[Clostridium] aminophilum	Bacteria	Firmicutes	Clostridia	Clostridiales	Lachnospirace	Lachnoclostri	hypothetical protein						
10185..14343	+	WP_031473346.1	NZ_FOZC01000010.1	[Clostridium] aminophilum	Bacteria	Firmicutes	Clostridia	Clostridiales	Lachnospirace	Lachnoclostri	hypothetical protein	cas13a	cas13a	CAS-VI-A			
45 CAS-VI-A | WP_087253216.1																	
80478..84588	+	WP_087253216.1	NZ_NFHY01000010.1	Drancourtella sp. An57	Bacteria	Firmicutes	Clostridia	Clostridiales	Ruminococca	Drancourtella	hypothetical protein	cas13a	cas13a	CAS-VI-A			
84591..84852	+	WP_087253218.1	NZ_NFHY01000010.1	Drancourtella sp. An57	Bacteria	Firmicutes	Clostridia	Clostridiales	Ruminococca	Drancourtella	CRISPR-associated endonuclease Cas2	cas2	cd09725	CAS-I,CAS-II,CAS-III,CAS-V			
84864..85434	+	WP_087253220.1	NZ_NFHY01000010.1	Drancourtella sp. An57	Bacteria	Firmicutes	Clostridia	Clostridiales	Ruminococca	Drancourtella	hypothetical protein	**RT**	pfam00078	CAS-I,CAS-III			
85406..86759	+	WP_087253222.1	NZ_NFHY01000010.1	Drancourtella sp. An57	Bacteria	Firmicutes	Clostridia	Clostridiales	Ruminococca	Drancourtella	CRISPR-associated endonuclease Cas1	**RT,cas1**	pfam00078,cd09722	CAS-I,CAS-III			
86966..87387	array		NZ_NFHY01000010.1	Drancourtella sp. An57	Bacteria	Firmicutes	Clostridia	Clostridiales	Ruminococca	Drancourtella		CRISPR array			6	47	31
46 CAS-VI-A | WP_090127496.1																	
102556..102791	array		NZ_FCNR01000048.1	Eubacteriaceae bacterium CHKCI004	Bacteria	Firmicutes	Clostridia	Clostridiales	Eubacteriaceae			CRISPR array			4	38	30
102881..103142	+	WP_090127494.1	NZ_FCNR01000048.1	Eubacteriaceae bacterium CHKCI004	Bacteria	Firmicutes	Clostridia	Clostridiales	Eubacteriaceae		CRISPR-associated endonuclease Cas2	cas2	cd09725	CAS-I,CAS-II,CAS-III,CAS-V			
103162..105070	+	WP_090127495.1	NZ_FCNR01000048.1	Eubacteriaceae bacterium CHKCI004	Bacteria	Firmicutes	Clostridia	Clostridiales	Eubacteriaceae		type I-B CRISPR-associated endonuclease Cas1	**RT,cas1**	pfam00078,cd09722	CAS-I,CAS-III			
105345..105568	array		NZ_FCNR01000048.1	Eubacteriaceae bacterium CHKCI004	Bacteria	Firmicutes	Clostridia	Clostridiales	Eubacteriaceae			CRISPR array			4	37	28
105978..110100	+	WP_090127496.1	NZ_FCNR01000048.1	Eubacteriaceae bacterium CHKCI004	Bacteria	Firmicutes	Clostridia	Clostridiales	Eubacteriaceae		hypothetical protein	cas13a	cas13a	CAS-VI-A			
47 CAS-VI-A | WP_055061018.1																	
58872..59187	array		NZ_CVRQ01000008.1	[Eubacterium] rectale	Bacteria	Firmicutes	Clostridia	Clostridiales	Lachnospiraceae			CRISPR array			5	40	31
59671..63706	+	WP_055061018.1	NZ_CVRQ01000008.1	[Eubacterium] rectale	Bacteria	Firmicutes	Clostridia	Clostridiales	Lachnospiraceae		hypothetical protein	cas13a	cas13a	CAS-VI-A			
63967..64487	array		NZ_CVRQ01000008.1	[Eubacterium] rectale	Bacteria	Firmicutes	Clostridia	Clostridiales	Lachnospiraceae			CRISPR array			8	39	31
48 CAS-VI-A | WP_117482613.1																	
64229..68378	+	WP_117482613.1	NZ_QSOB01000012.1	[Eubacterium] rectale	Bacteria	Firmicutes	Clostridia	Clostridiales	Lachnospiraceae		hypothetical protein	cas13a	cas13a	CAS-VI-A			
68395..68656	+	WP_117482614.1	NZ_QSOB01000012.1	[Eubacterium] rectale	Bacteria	Firmicutes	Clostridia	Clostridiales	Lachnospiraceae		CRISPR-associated endonuclease Cas2	cas2	cd09725	CAS-I,CAS-II,CAS-III,CAS-V			
68809..69205	array		NZ_QSOB01000012.1	[Eubacterium] rectale	Bacteria	Firmicutes	Clostridia	Clostridiales	Lachnospiraceae			CRISPR array			6	41	32

49	CAS-VI-A | WP_118003838.1															
	107860..108174	array		NZ_QRUJ01000006.1	[Eubacterium] rectale	Bacteria	Firmicutes	Clostridia	Clostridiales	Lachnospiraceae		CRISPR array			5	38	32
	108543..112782	+	WP_118003838.1	NZ_QRUJ01000006.1	[Eubacterium] rectale	Bacteria	Firmicutes	Clostridia	Clostridiales	Lachnospiraceae	hypothetical protein	**cas13a**	cas13a	CAS-VI-A			
	112799..113060	+	WP_117482614.1	NZ_QRUJ01000006.1	[Eubacterium] rectale	Bacteria	Firmicutes	Clostridia	Clostridiales	Lachnospiraceae	CRISPR-associated endonuclease Cas2	cas2	cd09725	CAS-I,CAS-II,CAS-III,CAS-V			
	113078..114974	+	WP_118003845.1	NZ_QRUJ01000006.1	[Eubacterium] rectale	Bacteria	Firmicutes	Clostridia	Clostridiales	Lachnospiraceae	CRISPR-associated endonuclease Cas1	**RT,cas1**	pfam00078,cd09722	CAS-I,CAS-III			
	115140..115521	array		NZ_QRUJ01000006.1	[Eubacterium] rectale	Bacteria	Firmicutes	Clostridia	Clostridiales	Lachnospiraceae		CRISPR array			6	39	31

Table S3: Computational analysis of the neighborhood of cas1 sequences used in Figure 2. Type VI-A loci are indicated in grey background.

Node	(Sub)type/Coordinates	Strand	Protein_id	Genome_partition	Genome_name	Domain	Phylum	Class	Order	Family	Genus	Description	cas_gene	profiles	subtype	repeats	avg_spacer_length	avg_repeat_length
1	CAS-III-D | SES98376.1																	
	44548..44779	-	SES97982.1	FOHN01000006.1	[Clostridium] polysaccharolyticum	Bacteria	Firmicutes	Clostridia	Clostridiales	Lachnospiraceae	Lachnoclostridium	hypothetical protein	csx1	cd09732	CAS-III			
	45069..47058	-	SES98000.1	FOHN01000006.1	[Clostridium] polysaccharolyticum	Bacteria	Firmicutes	Clostridia	Clostridiales	Lachnospiraceae	Lachnoclostridium	CRISPR-associated protein	csm3gr7	cls000253	CAS-III-A,CAS-III-D			
	47062..47434	-	SES98017.1	FOHN01000006.1	[Clostridium] polysaccharolyticum	Bacteria	Firmicutes	Clostridia	Clostridiales	Lachnospiraceae	Lachnoclostridium	hypothetical protein	csx19	mkCas0132	CAS-III-D			
	47423..48752	-	SES98041.1	FOHN01000006.1	[Clostridium] polysaccharolyticum	Bacteria	Firmicutes	Clostridia	Clostridiales	Lachnospiraceae	Lachnoclostridium	CRISPR/Cas system CSM-associated protein Csm3, group 7 of RAMP superfamily	csm3gr7,cmr6gr7,csm3gr7	cd09726,COG	CAS-III-D			
	48755..50963	-	SES98068.1	FOHN01000006.1	[Clostridium] polysaccharolyticum	Bacteria	Firmicutes	Clostridia	Clostridiales	Lachnospiraceae	Lachnoclostridium	CRISPR/Cas system CSM-associated protein Csm3, group 7 of RAMP superfamily	csm3gr7,csx10gr5	mkCas0198,cl	CAS-III-A,CAS-III-D			
	50964..52614	-	SES98079.1	FOHN01000006.1	[Clostridium] polysaccharolyticum	Bacteria	Firmicutes	Clostridia	Clostridiales	Lachnospiraceae	Lachnoclostridium	hypothetical protein	cas10	mkCas0133	CAS-III			
	52606..54295	-	SES98230.1	FOHN01000006.1	[Clostridium] polysaccharolyticum	Bacteria	Firmicutes	Clostridia	Clostridiales	Lachnospiraceae	Lachnoclostridium	hypothetical protein	Csx1	cluster2				
	54291..55050	-	SES98245.1	FOHN01000006.1	[Clostridium] polysaccharolyticum	Bacteria	Firmicutes	Clostridia	Clostridiales	Lachnospiraceae	Lachnoclostridium	CRISPR-associated endoribonuclease Cas6	cas6	pfam10040	CAS-III,CAS-IV			
	55186..55441	-	SES98267.1	FOHN01000006.1	[Clostridium] polysaccharolyticum	Bacteria	Firmicutes	Clostridia	Clostridiales	Lachnospiraceae	Lachnoclostridium	hypothetical protein						
	55433..55889	-	SES98290.1	FOHN01000006.1	[Clostridium] polysaccharolyticum	Bacteria	Firmicutes	Clostridia	Clostridiales	Lachnospiraceae	Lachnoclostridium	4Fe-4S single cluster domain-containing protein						
	55878..56586	-	SES98303.1	FOHN01000006.1	[Clostridium] polysaccharolyticum	Bacteria	Firmicutes	Clostridia	Clostridiales	Lachnospiraceae	Lachnoclostridium	putative peptide transport system permease protein						
	56592..56919	-	SES98326.1	FOHN01000006.1	[Clostridium] polysaccharolyticum	Bacteria	Firmicutes	Clostridia	Clostridiales	Lachnospiraceae	Lachnoclostridium	ABC transporter	Cas_RecF	cluster17				
	57029..57221	-	SES98334.1	FOHN01000006.1	[Clostridium] polysaccharolyticum	Bacteria	Firmicutes	Clostridia	Clostridiales	Lachnospiraceae	Lachnoclostridium	ABC transporter						
	59730..60057	-	SES98352.1	FOHN01000006.1	[Clostridium] polysaccharolyticum	Bacteria	Firmicutes	Clostridia	Clostridiales	Lachnospiraceae	Lachnoclostridium	CRISPR-associated protein, Cas2 family	cas2	pfam09827	CAS-I,CAS-II,CAS-III			
	60068..61070	-	SES98376.1	FOHN01000006.1	[Clostridium] polysaccharolyticum	Bacteria	Firmicutes	Clostridia	Clostridiales	Lachnospiraceae	Lachnoclostridium	CRISP-associated protein Cas1	**cas1**	cd09634	CAS-I,CAS-II,CAS-III,CAS-V			
2	CAS-III-A | OLR64025.1																	
	520551..521017	array		MJIG01000003.1	Anaerostipes sp. 992a	Bacteria	Firmicutes	Clostridia	Clostridiales	Lachnospiraceae	Anaerostipes		CRISPR array			7	37	35
	521184..522513	+	OLR64018.1	MJIG01000003.1	Anaerostipes sp. 992a	Bacteria	Firmicutes	Clostridia	Clostridiales	Lachnospiraceae	Anaerostipes	hypothetical protein	csm6	cd09746	CAS-III-A			
	522514..523270	+	OLR64019.1	MJIG01000003.1	Anaerostipes sp. 992a	Bacteria	Firmicutes	Clostridia	Clostridiales	Lachnospiraceae	Anaerostipes	CRISPR-associated endoribonuclease Cas6	cas6	pfam10040	CAS-III,CAS-IV			
	523259..525617	+	OLR64020.1	MJIG01000003.1	Anaerostipes sp. 992a	Bacteria	Firmicutes	Clostridia	Clostridiales	Lachnospiraceae	Anaerostipes	type III-A CRISPR-associated protein Cas10/Csm1	cas10,cas10	cd09680,cls0	CAS-III			
	525609..526041	+	OLR64021.1	MJIG01000003.1	Anaerostipes sp. 992a	Bacteria	Firmicutes	Clostridia	Clostridiales	Lachnospiraceae	Anaerostipes	type III-A CRISPR-associated protein Csm2	csm2gr11	cd09647	CAS-III-A			
	526050..526713	+	OLR64022.1	MJIG01000003.1	Anaerostipes sp. 992a	Bacteria	Firmicutes	Clostridia	Clostridiales	Lachnospiraceae	Anaerostipes	type III-A CRISPR-associated RAMP protein Csm3	csm3gr7	cd09684	CAS-III-A,CAS-III-D			
	526714..527647	+	OLR64023.1	MJIG01000003.1	Anaerostipes sp. 992a	Bacteria	Firmicutes	Clostridia	Clostridiales	Lachnospiraceae	Anaerostipes	type III-A CRISPR-associated RAMP protein Csm4	csm4gr5	COG1567	CAS-III-A			
	527639..528752	+	OLR64024.1	MJIG01000003.1	Anaerostipes sp. 992a	Bacteria	Firmicutes	Clostridia	Clostridiales	Lachnospiraceae	Anaerostipes	type III-A CRISPR-associated RAMP protein Csm5	csm5gr7,csm5gr7	cd09662,cd09	CAS-III-A			
	528882..529641	array		MJIG01000003.1	Anaerostipes sp. 992a	Bacteria	Firmicutes	Clostridia	Clostridiales	Lachnospiraceae	Anaerostipes		CRISPR array			11	36	36
	529673..530678	+	OLR64025.1	MJIG01000003.1	Anaerostipes sp. 992a	Bacteria	Firmicutes	Clostridia	Clostridiales	Lachnospiraceae	Anaerostipes	CRISPR-associated endonuclease Cas1	**cas1**	cd09634	CAS-I,CAS-II,CAS-III,CAS-V			
	530694..531009	+	OLR64066.1	MJIG01000003.1	Anaerostipes sp. 992a	Bacteria	Firmicutes	Clostridia	Clostridiales	Lachnospiraceae	Anaerostipes	CRISPR-associated endonuclease Cas2	cas2	cd09725	CAS-I,CAS-II,CAS-III,CAS-V			
3	CAS-III-A | ASS37303.1																	
	338281..339031	+	ASS37296.1	CP016199.1	Mogibacterium pumilum	Bacteria	Firmicutes	Clostridia	Clostridiales	Clostridiales Family XIII. Incertae Sedis	Mogibacterium	CRISPR-associated endoribonuclease Cas6	cas6	COG5551	CAS-III			
	339034..341404	+	ASS37297.1	CP016199.1	Mogibacterium pumilum	Bacteria	Firmicutes	Clostridia	Clostridiales	Clostridiales Family XIII. Incertae Sedis	Mogibacterium	type III-A CRISPR-associated protein Cas10/Csm1	cas10	cd09680	CAS-III			
	341406..341829	+	ASS37298.1	CP016199.1	Mogibacterium pumilum	Bacteria	Firmicutes	Clostridia	Clostridiales	Clostridiales Family XIII. Incertae Sedis	Mogibacterium	type III-A CRISPR-associated protein Csm2	csm2gr11	cd09647	CAS-III-A			
	341843..342518	+	ASS37299.1	CP016199.1	Mogibacterium pumilum	Bacteria	Firmicutes	Clostridia	Clostridiales	Clostridiales Family XIII. Incertae Sedis	Mogibacterium	type III-A CRISPR-associated RAMP protein Csm3	csm3gr7	cd09684	CAS-III-A,CAS-III-D			
	342520..343447	+	ASS37300.1	CP016199.1	Mogibacterium pumilum	Bacteria	Firmicutes	Clostridia	Clostridiales	Clostridiales Family XIII. Incertae Sedis	Mogibacterium	type III-A CRISPR-associated RAMP protein Csm4	csm4gr5	COG1567	CAS-III-A			
	343436..344561	+	ASS37301.1	CP016199.1	Mogibacterium pumilum	Bacteria	Firmicutes	Clostridia	Clostridiales	Clostridiales Family XIII. Incertae Sedis	Mogibacterium	type III-A CRISPR-associated RAMP protein Csm5	csm5gr7,csm5gr7	cd09662,cd09	CAS-III-A			
	344690..345368	array		CP016199.1	Mogibacterium pumilum	Bacteria	Firmicutes	Clostridia	Clostridiales	Clostridiales Family XIII. Incertae Sedis	Mogibacterium		CRISPR array			10	36	35
	345434..345785	+	ASS37302.1	CP016199.1	Mogibacterium pumilum	Bacteria	Firmicutes	Clostridia	Clostridiales	Clostridiales Family XIII. Incertae Sedis	Mogibacterium	hypothetical protein						
	345835..346287	array		CP016199.1	Mogibacterium pumilum	Bacteria	Firmicutes	Clostridia	Clostridiales	Clostridiales Family XIII. Incertae Sedis	Mogibacterium		CRISPR array			7	51	21
	346324..347329	+	ASS37303.1	CP016199.1	Mogibacterium pumilum	Bacteria	Firmicutes	Clostridia	Clostridiales	Clostridiales Family XIII. Incertae Sedis	Mogibacterium	CRISPR-associated endonuclease Cas1	**cas1**	cd09634	CAS-I,CAS-II,CAS-III,CAS-V			
	347351..347663	+	ASS38486.1	CP016199.1	Mogibacterium pumilum	Bacteria	Firmicutes	Clostridia	Clostridiales	Clostridiales Family XIII. Incertae Sedis	Mogibacterium	CRISPR-associated endonuclease Cas2	cas2	cd09725	CAS-I,CAS-II,CAS-III,CAS-V			
	347702..348713	+	ASS37304.1	CP016199.1	Mogibacterium pumilum	Bacteria	Firmicutes	Clostridia	Clostridiales	Clostridiales Family XIII. Incertae Sedis	Mogibacterium	hypothetical protein	csm6	pfam09659	CAS-III-A			
4	CAS-III-A | EMZ15703.1																	
	61984..62796	array		KB822565.1	Clostridium sp. ASF356	Bacteria	Firmicutes	Clostridia	Clostridiales	Clostridiaceae	Clostridium		CRISPR array			12	34	36
	63011..64352	-	EMZ15701.1	KB822565.1	Clostridium sp. ASF356	Bacteria	Firmicutes	Clostridia	Clostridiales	Clostridiaceae	Clostridium	CRISPR type III-a/mtube-associated protein csm6	csm6	pfam09659	CAS-III-A			
	64386..64719	-	EMZ15702.1	KB822565.1	Clostridium sp. ASF356	Bacteria	Firmicutes	Clostridia	Clostridiales	Clostridiaceae	Clostridium	CRISPR-associated endoribonuclease cas2	cas2	cd09725	CAS-I,CAS-II,CAS-III,CAS-V			
	64715..65723	-	EMZ15703.1	KB822565.1	Clostridium sp. ASF356	Bacteria	Firmicutes	Clostridia	Clostridiales	Clostridiaceae	Clostridium	CRISPR-associated endonuclease cas1	**cas1**	cd09634	CAS-I,CAS-II,CAS-III,CAS-V			
	65727..66822	-	EMZ15704.1	KB822565.1	Clostridium sp. ASF356	Bacteria	Firmicutes	Clostridia	Clostridiales	Clostridiaceae	Clostridium	CRISPR type III-a/mtube-associated ramp protein csm5	csm5gr7,csm5gr7	cd09662,COG	CAS-III-A			
	66821..67754	-	EMZ15705.1	KB822565.1	Clostridium sp. ASF356	Bacteria	Firmicutes	Clostridia	Clostridiales	Clostridiaceae	Clostridium	CRISPR type III-a/mtube-associated ramp protein csm4	csm4gr5	COG1567	CAS-III-A			
	67756..68416	-	EMZ15706.1	KB822565.1	Clostridium sp. ASF356	Bacteria	Firmicutes	Clostridia	Clostridiales	Clostridiaceae	Clostridium	CRISPR type III-a/mtube-associated ramp protein csm3	csm3gr7	cd09684	CAS-III-A,CAS-III-D			
	68428..68803	-	EMZ15707.1	KB822565.1	Clostridium sp. ASF356	Bacteria	Firmicutes	Clostridia	Clostridiales	Clostridiaceae	Clostridium	CRISPR type III-a/mtube-associated protein csm2	csm2gr11	pfam03750	CAS-III-A			
	68804..71129	-	EMZ15708.1	KB822565.1	Clostridium sp. ASF356	Bacteria	Firmicutes	Clostridia	Clostridiales	Clostridiaceae	Clostridium	CRISPR-associated protein cas10/csm1, subtype III-a/mtube	cas10	cd09680	CAS-III			
	71121..71880	-	EMZ15709.1	KB822565.1	Clostridium sp. ASF356	Bacteria	Firmicutes	Clostridia	Clostridiales	Clostridiaceae	Clostridium	CRISPR-associated endoribonuclease cas6	cas6	pfam10040	CAS-III,CAS-IV			
5	CAS-III-A | RHR83755.1																	
	3212..4538	-	RHR83753.1	QUHE01000024.1	Butyricicoccus sp. AF15-40	Bacteria	Firmicutes	Clostridia	Clostridiales	Clostridiaceae	Butyricicoccus	CRISPR-associated protein Csm6	csm6	pfam09659	CAS-III-A			
	4556..4880	-	RHR83754.1	QUHE01000024.1	Butyricicoccus sp. AF15-40	Bacteria	Firmicutes	Clostridia	Clostridiales	Clostridiaceae	Butyricicoccus	CRISPR-associated endonuclease Cas2	cas2	cd09725	CAS-I,CAS-II,CAS-III,CAS-V			
	4893..5895	-	RHR83755.1	QUHE01000024.1	Butyricicoccus sp. AF15-40	Bacteria	Firmicutes	Clostridia	Clostridiales	Clostridiaceae	Butyricicoccus	CRISPR-associated endonuclease Cas1	**cas1**	cd09634	CAS-I,CAS-II,CAS-III,CAS-V			
	5913..7088	array		QUHE01000024.1	Butyricicoccus sp. AF15-40	Bacteria	Firmicutes	Clostridia	Clostridiales	Clostridiaceae	Butyricicoccus		CRISPR array			17	35	36
	7275..8391	-	RHR83756.1	QUHE01000024.1	Butyricicoccus sp. AF15-40	Bacteria	Firmicutes	Clostridia	Clostridiales	Clostridiaceae	Butyricicoccus	type III-A CRISPR-associated RAMP protein Csm5	csm5gr7	COG1332	CAS-III-A			
	8393..9329	-	RHR83757.1	QUHE01000024.1	Butyricicoccus sp. AF15-40	Bacteria	Firmicutes	Clostridia	Clostridiales	Clostridiaceae	Butyricicoccus	type III-A CRISPR-associated RAMP protein Csm4	csm4gr5	COG1567	CAS-III-A			
	9332..9992	-	RHR83758.1	QUHE01000024.1	Butyricicoccus sp. AF15-40	Bacteria	Firmicutes	Clostridia	Clostridiales	Clostridiaceae	Butyricicoccus	type III-A CRISPR-associated RAMP protein Csm3	csm3gr7	cd09684	CAS-III-A,CAS-III-D			
	10009..10396	-	RHR83759.1	QUHE01000024.1	Butyricicoccus sp. AF15-40	Bacteria	Firmicutes	Clostridia	Clostridiales	Clostridiaceae	Butyricicoccus	type III-A CRISPR-associated protein Csm2	csm2gr11	pfam03750	CAS-III-A			
	10398..12717	-	RHR83760.1	QUHE01000024.1	Butyricicoccus sp. AF15-40	Bacteria	Firmicutes	Clostridia	Clostridiales	Clostridiaceae	Butyricicoccus	type III-A CRISPR-associated protein Cas10/Csm1	cas10	cd09680	CAS-III			
	12697..13471	-	RHR83761.1	QUHE01000024.1	Butyricicoccus sp. AF15-40	Bacteria	Firmicutes	Clostridia	Clostridiales	Clostridiaceae	Butyricicoccus	CRISPR system precrRNA processing endoribonuclease RAMP protein Cas6	cas6	COG5551	CAS-III			
6	CAS-III-A | SCG93998.1																	
	140058..140817	+	SCG93766.1	FMDZ01000001.1	uncultured Eubacterium sp.	Bacteria	Firmicutes	Clostridia	Clostridiales	Eubacteriaceae	Eubacterium	CRISPR-associated endoribonuclease Cas6	cas6	COG5551	CAS-III			
	140820..143163	+	SCG93805.1	FMDZ01000001.1	uncultured Eubacterium sp.	Bacteria	Firmicutes	Clostridia	Clostridiales	Eubacteriaceae	Eubacterium	CRISPR-associated protein Cas10/Csm1%2C subtype III-A/MTUBE	cas10	cd09680	CAS-III			
	143140..143527	+	SCG93843.1	FMDZ01000001.1	uncultured Eubacterium sp.	Bacteria	Firmicutes	Clostridia	Clostridiales	Eubacteriaceae	Eubacterium	CRISPR type III-A/MTUBE-associated protein Csm2	csm2gr11	cd09647	CAS-III-A			
	143540..144209	+	SCG93882.1	FMDZ01000001.1	uncultured Eubacterium sp.	Bacteria	Firmicutes	Clostridia	Clostridiales	Eubacteriaceae	Eubacterium	CRISPR type III-A/MTUBE-associated RAMP protein Csm3	csm3gr7	cd09684	CAS-III-A,CAS-III-D			
	144211..145144	+	SCG93924.1	FMDZ01000001.1	uncultured Eubacterium sp.	Bacteria	Firmicutes	Clostridia	Clostridiales	Eubacteriaceae	Eubacterium	CRISPR type III-A/MTUBE-associated RAMP protein Csm4	csm4gr5	COG1567	CAS-III-A			
	145145..146273	+	SCG93960.1	FMDZ01000001.1	uncultured Eubacterium sp.	Bacteria	Firmicutes	Clostridia	Clostridiales	Eubacteriaceae	Eubacterium	CRISPR type III-A/MTUBE-associated RAMP protein Csm5	csm5gr7,csm5gr7	cd09662,cd09	CAS-III-A			
	146407..147021	array		FMDZ01000001.1	uncultured Eubacterium sp.	Bacteria	Firmicutes	Clostridia	Clostridiales	Eubacteriaceae	Eubacterium		CRISPR array			9	36	36
	147123..148125	+	SCG93998.1	FMDZ01000001.1	uncultured Eubacterium sp.	Bacteria	Firmicutes	Clostridia	Clostridiales	Eubacteriaceae	Eubacterium	CRISPR-associated endonuclease Cas1	**cas1**	cd09634	CAS-I,CAS-II,CAS-III,CAS-V			
	148132..148450	+	SCG94033.1	FMDZ01000001.1	uncultured Eubacterium sp.	Bacteria	Firmicutes	Clostridia	Clostridiales	Eubacteriaceae	Eubacterium	CRISPR-associated endonuclease Cas2	cas2	cd09725	CAS-I,CAS-II,CAS-III,CAS-V			
	148469..149798	+	SCG94074.1	FMDZ01000001.1	uncultured Eubacterium sp.	Bacteria	Firmicutes	Clostridia	Clostridiales	Eubacteriaceae	Eubacterium	CRISPR type III-A/MTUBE-associated protein Csm6	csm6	pfam09659	CAS-III-A			
7	CAS-III-A | RHP28154.1																	
	347871..348627	+	RHP28148.1	QUEB01000001.1	Clostridium sp. AF34-13	Bacteria	Firmicutes	Clostridia	Clostridiales	Clostridiaceae	Clostridium	CRISPR system precrRNA processing endoribonuclease RAMP protein Cas6	cas6	COG5551	CAS-III			
	348628..351085	+	RHP28149.1	QUEB01000001.1	Clostridium sp. AF34-13	Bacteria	Firmicutes	Clostridia	Clostridiales	Clostridiaceae	Clostridium	type III-A CRISPR-associated protein Cas10/Csm1	cas10	cd09680	CAS-III			
	351163..351583	+	RHP28150.1	QUEB01000001.1	Clostridium sp. AF34-13	Bacteria	Firmicutes	Clostridia	Clostridiales	Clostridiaceae	Clostridium	type III-A CRISPR-associated protein Csm2	csm2gr11	pfam03750	CAS-III-A			
	351619..352300	+	RHP28151.1	QUEB01000001.1	Clostridium sp. AF34-13	Bacteria	Firmicutes	Clostridia	Clostridiales	Clostridiaceae	Clostridium	type III-A CRISPR-associated RAMP protein Csm3	csm3gr7	cd09684	CAS-III-A,CAS-III-D			
	352314..353238	+	RHP28152.1	QUEB01000001.1	Clostridium sp. AF34-13	Bacteria	Firmicutes	Clostridia	Clostridiales	Clostridiaceae	Clostridium	type III-A CRISPR-associated RAMP protein Csm4	csm4gr5	COG1567	CAS-III-A			
	353241..354315	+	RHP28153.1	QUEB01000001.1	Clostridium sp. AF34-13	Bacteria	Firmicutes	Clostridia	Clostridiales	Clostridiaceae	Clostridium	type III-A CRISPR-associated RAMP protein Csm5	csm5gr7,csm5gr7	cd09662,COG	CAS-III-A			
	354451..355478	array		QUEB01000001.1	Clostridium sp. AF34-13	Bacteria	Firmicutes	Clostridia	Clostridiales	Clostridiaceae	Clostridium		CRISPR array			15	34	36
	355575..356586	+	RHP28154.1	QUEB01000001.1	Clostridium sp. AF34-13	Bacteria	Firmicutes	Clostridia	Clostridiales	Clostridiaceae	Clostridium	CRISPR-associated endonuclease Cas1	**cas1**	cd09634	CAS-I,CAS-II,CAS-III,CAS-V			
	356653..356983	+	RHP28155.1	QUEB01000001.1	Clostridium sp. AF34-13	Bacteria	Firmicutes	Clostridia	Clostridiales	Clostridiaceae	Clostridium	CRISPR-associated endonuclease Cas2	cas2	cd09725	CAS-I,CAS-II,CAS-III,CAS-V			
	357000..358341	+	RHP28156.1	QUEB01000001.1	Clostridium sp. AF34-13	Bacteria	Firmicutes	Clostridia	Clostridiales	Clostridiaceae	Clostridium	CRISPR-associated protein Csm6	csm6	pfam09659	CAS-III-A			
8	partial CAS-III-A | CCZ41463.1																	
	635..1640	+	CCZ41463.1	HF995923.1	Clostridium sp. CAG:122	Bacteria	Firmicutes	Clostridia	Clostridiales	Clostridiaceae	Clostridium	cRISPR-associated endonuclease Cas1	**cas1**	cd09634	CAS-I,CAS-II,CAS-III,CAS-V			
	1706..2036	+	CCZ41464.1	HF995923.1	Clostridium sp. CAG:122	Bacteria	Firmicutes	Clostridia	Clostridiales	Clostridiaceae	Clostridium	cRISPR-associated endonuclease Cas2 2	cas2	cd09725	CAS-I,CAS-II,CAS-III,CAS-V			
	2053..3394	+	CCZ41465.1	HF995923.1	Clostridium sp. CAG:122	Bacteria	Firmicutes	Clostridia	Clostridiales	Clostridiaceae	Clostridium	putative uncharacterized protein	csm6	pfam09659	CAS-III-A			
9	CAS-III-A | KQC84610.1																	
	193480..194821	-	KQC84608.1	LLKB01000005.1	Lachnospiraceae bacterium TF01-11	Bacteria	Firmicutes	Clostridia	Clostridiales	Lachnospiraceae		hypothetical protein	csm6	pfam09659	CAS-III-A			
	194838..195168	-	KQC84609.1	LLKB01000005.1	Lachnospiraceae bacterium TF01-11	Bacteria	Firmicutes	Clostridia	Clostridiales	Lachnospiraceae		hypothetical protein	cas2	cd09725	CAS-I,CAS-II,CAS-III,CAS-V			
	195235..196246	-	KQC84610.1	LLKB01000005.1	Lachnospiraceae bacterium TF01-11	Bacteria	Firmicutes	Clostridia	Clostridiales	Lachnospiraceae		CRISPR-associated protein Cas1	**cas1**	cd09634	CAS-I,CAS-II,CAS-III,CAS-V			
	197362..198972	array		LLKB01000005.1	Lachnospiraceae bacterium TF01-11	Bacteria	Firmicutes	Clostridia	Clostridiales	Lachnospiraceae			CRISPR array			23	35	36
	199107..200217	-	KQC84611.1	LLKB01000005.1	Lachnospiraceae bacterium TF01-11	Bacteria	Firmicutes	Clostridia	Clostridiales	Lachnospiraceae		hypothetical protein	csm5gr7,csm5gr7	cd09662,cd09	CAS-III-A			
	200220..201144	-	KQC84612.1	LLKB01000005.1	Lachnospiraceae bacterium TF01-11	Bacteria	Firmicutes	Clostridia	Clostridiales	Lachnospiraceae		hypothetical protein	csm4gr5	COG1567	CAS-III-A			
	201158..201815	-	KQC84613.1	LLKB01000005.1	Lachnospiraceae bacterium TF01-11	Bacteria	Firmicutes	Clostridia	Clostridiales	Lachnospiraceae		type III-A CRISPR-associated RAMP protein Csm3	csm3gr7	cd09684	CAS-III-A,CAS-III-D			
	201851..202199	-	KQC85580.1	LLKB01000005.1	Lachnospiraceae bacterium TF01-11	Bacteria	Firmicutes	Clostridia	Clostridiales	Lachnospiraceae		hypothetical protein	csm2gr11	pfam03750	CAS-III-A			
	202307..204698	-	KQC84614.1	LLKB01000005.1	Lachnospiraceae bacterium TF01-11	Bacteria	Firmicutes	Clostridia	Clostridiales	Lachnospiraceae		type III-A CRISPR-associated protein Cas10/Csm1	cas10	cd09680	CAS-III			
	204699..205455	-	KQC84615.1	LLKB01000005.1	Lachnospiraceae bacterium TF01-11	Bacteria	Firmicutes	Clostridia	Clostridiales	Lachnospiraceae		hypothetical protein	cas6	COG5551	CAS-III			
10	CAS-III-A | CUO16841.1																	
	154793..156119	+	CUO16576.1	CYZM01000004.1	Anaerostipes hadrus	Bacteria	Firmicutes	Clostridia	Clostridiales	Lachnospiraceae	Anaerostipes	CRISPR type III-A/MTUBE-associated protein Csm6	csm6	cd09746	CAS-III-A			
	156115..156856	+	CUO16613.1	CYZM01000004.1	Anaerostipes hadrus	Bacteria	Firmicutes	Clostridia	Clostridiales	Lachnospiraceae	Anaerostipes	CRISPR-associated endoribonuclease Cas6	cas6	COG5551	CAS-III			
	156860..159194	+	CUO16648.1	CYZM01000004.1	Anaerostipes hadrus	Bacteria	Firmicutes	Clostridia	Clostridiales	Lachnospiraceae	Anaerostipes	Predicted HD superfamily hydrolase	cas10	cd09680	CAS-III			
	159194..159572	+	CUO16682.1	CYZM01000004.1	Anaerostipes hadrus	Bacteria	Firmicutes	Clostridia	Clostridiales	Lachnospiraceae	Anaerostipes	CRISPR type III-A/MTUBE-associated protein Csm2	csm2gr11	cd09647	CAS-III-A			
	159589..160267	+	CUO16726.1	CYZM01000004.1	Anaerostipes hadrus	Bacteria	Firmicutes	Clostridia	Clostridiales	Lachnospiraceae	Anaerostipes	CRISPR type III-A/MTUBE-associated RAMP protein Csm3	csm3gr7	cd09684	CAS-III-A,CAS-III-D			
	160266..161196	+	CUO16763.1	CYZM01000004.1	Anaerostipes hadrus	Bacteria	Firmicutes	Clostridia	Clostridiales	Lachnospiraceae	Anaerostipes	CRISPR type III-A/MTUBE-associated RAMP protein Csm4	csm4gr5	cd09663	CAS-III-A			
	161195..162308	+	CUO16802.1	CYZM01000004.1	Anaerostipes hadrus	Bacteria	Firmicutes	Clostridia	Clostridiales	Lachnospiraceae	Anaerostipes	CRISPR type III-A/MTUBE-associated RAMP protein Csm5	csm5gr7,csm5gr7	cd09662,cd09	CAS-III-A			

162444..163132	array		CYZM01000004.1	Anaerostipes hadrus	Bacteria	Firmicutes	Clostridia	Clostridiales	Lachnospiraceae	Anaerostipes		CRISPR array			10	37	35
163375..164230	+	CUO16841.1	CYZM01000004.1	Anaerostipes hadrus	Bacteria	Firmicutes	Clostridia	Clostridiales	Lachnospiraceae	Anaerostipes	CRISPR-associated endonuclease Cas1	cas1	cd09634	CAS-I,CAS-II,CAS-III,CAS-V			
164381..164699	+	CUO16880.1	CYZM01000004.1	Anaerostipes hadrus	Bacteria	Firmicutes	Clostridia	Clostridiales	Lachnospiraceae	Anaerostipes	CRISPR-associated endoribonuclease Cas2	cas2	cd09725	CAS-I,CAS-II,CAS-III,CAS-V			
11 CAS-III-A | ESL02973.1																	
352442..352820	+	ESL02958.1	KI535368.1	Catonella morbi ATCC 51271	Bacteria	Firmicutes	Clostridia	Clostridiales	Lachnospiraceae	Catonella	hypothetical protein	WYL	pfam13280	CAS-I,CAS-III			
352843..353626	+	ESL02959.1	KI535368.1	Catonella morbi ATCC 51271	Bacteria	Firmicutes	Clostridia	Clostridiales	Lachnospiraceae	Catonella	CRISPR-associated endoribonuclease Cas6	cas6	COG5551	CAS-III			
353606..355874	+	ESL02960.1	KI535368.1	Catonella morbi ATCC 51271	Bacteria	Firmicutes	Clostridia	Clostridiales	Lachnospiraceae	Catonella	CRISPR-associated protein, Csm1 family	cas10	cd09680	CAS-III			
355870..356263	+	ESL02961.1	KI535368.1	Catonella morbi ATCC 51271	Bacteria	Firmicutes	Clostridia	Clostridiales	Lachnospiraceae	Catonella	CRISPR-associated protein, Csm2 family	csm2gr11	cd09647	CAS-III-A			
356252..356942	+	ESL02962.1	KI535368.1	Catonella morbi ATCC 51271	Bacteria	Firmicutes	Clostridia	Clostridiales	Lachnospiraceae	Catonella	CRISPR-associated RAMP protein, Csm3 family	csm3gr7	cd09684	CAS-III-A,CAS-III-D			
356941..357871	+	ESL02963.1	KI535368.1	Catonella morbi ATCC 51271	Bacteria	Firmicutes	Clostridia	Clostridiales	Lachnospiraceae	Catonella	CRISPR-associated RAMP protein, Csm4 family	csm4gr5	COG1567	CAS-III-A			
357870..359016	+	ESL02964.1	KI535368.1	Catonella morbi ATCC 51271	Bacteria	Firmicutes	Clostridia	Clostridiales	Lachnospiraceae	Catonella	CRISPR-associated RAMP protein, Csm5 family	csm5gr7	cd09662	CAS-III-A			
359096..359402	-	ESL02965.1	KI535368.1	Catonella morbi ATCC 51271	Bacteria	Firmicutes	Clostridia	Clostridiales	Lachnospiraceae	Catonella	hypothetical protein						
359151..362243	array		KI535368.1	Catonella morbi ATCC 51271	Bacteria	Firmicutes	Clostridia	Clostridiales	Lachnospiraceae	Catonella		CRISPR array			44	35	36
359423..359600	+	ESL02966.1	KI535368.1	Catonella morbi ATCC 51271	Bacteria	Firmicutes	Clostridia	Clostridiales	Lachnospiraceae	Catonella	hypothetical protein						
359767..359911	-	ESL02967.1	KI535368.1	Catonella morbi ATCC 51271	Bacteria	Firmicutes	Clostridia	Clostridiales	Lachnospiraceae	Catonella	hypothetical protein						
359959..360145	+	ESL02968.1	KI535368.1	Catonella morbi ATCC 51271	Bacteria	Firmicutes	Clostridia	Clostridiales	Lachnospiraceae	Catonella	hypothetical protein						
360396..360537	-	ESL02969.1	KI535368.1	Catonella morbi ATCC 51271	Bacteria	Firmicutes	Clostridia	Clostridiales	Lachnospiraceae	Catonella	hypothetical protein						
360883..361096	+	ESL02970.1	KI535368.1	Catonella morbi ATCC 51271	Bacteria	Firmicutes	Clostridia	Clostridiales	Lachnospiraceae	Catonella	hypothetical protein						
361681..361819	-	ESL02971.1	KI535368.1	Catonella morbi ATCC 51271	Bacteria	Firmicutes	Clostridia	Clostridiales	Lachnospiraceae	Catonella	hypothetical protein						
362040..362244	-	ESL02972.1	KI535368.1	Catonella morbi ATCC 51271	Bacteria	Firmicutes	Clostridia	Clostridiales	Lachnospiraceae	Catonella	hypothetical protein						
362258..363281	+	ESL02973.1	KI535368.1	Catonella morbi ATCC 51271	Bacteria	Firmicutes	Clostridia	Clostridiales	Lachnospiraceae	Catonella	CRISPR-associated endonuclease Cas1	cas1	cd09634	CAS-I,CAS-II,CAS-III,CAS-V			
363286..363574	+	ESL02974.1	KI535368.1	Catonella morbi ATCC 51271	Bacteria	Firmicutes	Clostridia	Clostridiales	Lachnospiraceae	Catonella	CRISPR-associated protein Cas2	cas2	cd09725	CAS-I,CAS-II,CAS-III,CAS-V			
363797..363995	-	ESL02975.1	KI535368.1	Catonella morbi ATCC 51271	Bacteria	Firmicutes	Clostridia	Clostridiales	Lachnospiraceae	Catonella	hypothetical protein						
364586..364724	+	ESL02976.1	KI535368.1	Catonella morbi ATCC 51271	Bacteria	Firmicutes	Clostridia	Clostridiales	Lachnospiraceae	Catonella	hypothetical protein						
364985..366338	+	ESL02977.1	KI535368.1	Catonella morbi ATCC 51271	Bacteria	Firmicutes	Clostridia	Clostridiales	Lachnospiraceae	Catonella	CRISPR-associated protein, Csm6 family	csm6	cd09746	CAS-III-A			
12 CAS-IIIA | PTL29107.1																	
909119..912023	array		PYXF01000001.1	Lachnospiraceae bacterium oral taxon 096	Bacteria	Firmicutes	Clostridia	Clostridiales	Lachnospiraceae			CRISPR array			40	37	36
912685..913774	+	PTL29105.1	PYXF01000001.1	Lachnospiraceae bacterium oral taxon 096	Bacteria	Firmicutes	Clostridia	Clostridiales	Lachnospiraceae		transposase		c2c9				
914114..915239	-	PTL29106.1	PYXF01000001.1	Lachnospiraceae bacterium oral taxon 096	Bacteria	Firmicutes	Clostridia	Clostridiales	Lachnospiraceae		hypothetical protein	WYL	pfam13280	CAS-I,CAS-III			
915485..916490	+	PTL29107.1	PYXF01000001.1	Lachnospiraceae bacterium oral taxon 096	Bacteria	Firmicutes	Clostridia	Clostridiales	Lachnospiraceae		CRISPR-associated endonuclease Cas1	cas1	cd09634	CAS-I,CAS-II,CAS-III,CAS-V			
916500..916776	+	PTL29108.1	PYXF01000001.1	Lachnospiraceae bacterium oral taxon 096	Bacteria	Firmicutes	Clostridia	Clostridiales	Lachnospiraceae		CRISPR-associated endonuclease Cas2	cas2	cd09725	CAS-I,CAS-II,CAS-III,CAS-V			
917529..917808	+	PTL29109.1	PYXF01000001.1	Lachnospiraceae bacterium oral taxon 096	Bacteria	Firmicutes	Clostridia	Clostridiales	Lachnospiraceae		hypothetical protein						
917846..918758	+	PTL29110.1	PYXF01000001.1	Lachnospiraceae bacterium oral taxon 096	Bacteria	Firmicutes	Clostridia	Clostridiales	Lachnospiraceae		hypothetical protein						
918723..919482	+	PTL29111.1	PYXF01000001.1	Lachnospiraceae bacterium oral taxon 096	Bacteria	Firmicutes	Clostridia	Clostridiales	Lachnospiraceae		CRISPR-associated endoribonuclease Cas6	cas6	pfam10040	CAS-III,CAS-IV			
919478..921830	+	PTL29112.1	PYXF01000001.1	Lachnospiraceae bacterium oral taxon 096	Bacteria	Firmicutes	Clostridia	Clostridiales	Lachnospiraceae		type III-A CRISPR-associated protein Cas10/Csm1	cas10	cd09680	CAS-III			
921813..922206	+	PTL29113.1	PYXF01000001.1	Lachnospiraceae bacterium oral taxon 096	Bacteria	Firmicutes	Clostridia	Clostridiales	Lachnospiraceae		type III-A CRISPR-associated protein Csm2	csm2gr11	cd09647	CAS-III-A			
922207..922903	+	PTL29114.1	PYXF01000001.1	Lachnospiraceae bacterium oral taxon 096	Bacteria	Firmicutes	Clostridia	Clostridiales	Lachnospiraceae		type III-A CRISPR-associated RAMP protein Csm3	csm3gr7	cd09684	CAS-III-A,CAS-III-D			
922904..923840	+	PTL29115.1	PYXF01000001.1	Lachnospiraceae bacterium oral taxon 096	Bacteria	Firmicutes	Clostridia	Clostridiales	Lachnospiraceae		type III-A CRISPR-associated RAMP protein Csm4	csm4gr5	cd09663	CAS-III-A			
923841..924990	+	PTL29116.1	PYXF01000001.1	Lachnospiraceae bacterium oral taxon 096	Bacteria	Firmicutes	Clostridia	Clostridiales	Lachnospiraceae		type III-A CRISPR-associated RAMP protein Csm5	csm5gr7	COG1332	CAS-III-A			
925009..926341	+	PTL29117.1	PYXF01000001.1	Lachnospiraceae bacterium oral taxon 096	Bacteria	Firmicutes	Clostridia	Clostridiales	Lachnospiraceae		CRISPR-associated protein Csm6	csm6	pfam09659	CAS-III-A			
13 CAS-III-A | EHI56780.1																	
250396..251758	-	EHI56778.1	JH378829.1	Johnsonella ignava ATCC 51276	Bacteria	Firmicutes	Clostridia	Clostridiales	Lachnospiraceae	Johnsonella	hypothetical protein	csm6	cd09746	CAS-III-A			
251785..252139	-	EHI56779.1	JH378829.1	Johnsonella ignava ATCC 51276	Bacteria	Firmicutes	Clostridia	Clostridiales	Lachnospiraceae	Johnsonella	hypothetical protein	cas2	cd09725	CAS-I,CAS-II,CAS-III,CAS-V			
252141..253152	-	EHI56780.1	JH378829.1	Johnsonella ignava ATCC 51276	Bacteria	Firmicutes	Clostridia	Clostridiales	Lachnospiraceae	Johnsonella	CRISPR-associated protein cas1	cas1	cd09634	CAS-I,CAS-II,CAS-III,CAS-V			
253797..254418	-	EHI56781.1	JH378829.1	Johnsonella ignava ATCC 51276	Bacteria	Firmicutes	Clostridia	Clostridiales	Lachnospiraceae	Johnsonella	hypothetical protein						
254799..255276	array		JH378829.1	Johnsonella ignava ATCC 51276	Bacteria	Firmicutes	Clostridia	Clostridiales	Lachnospiraceae	Johnsonella		CRISPR array			7	38	35
255358..255643	+	EHI56782.1	JH378829.1	Johnsonella ignava ATCC 51276	Bacteria	Firmicutes	Clostridia	Clostridiales	Lachnospiraceae	Johnsonella	hypothetical protein						
255987..256418	+	EHI56553.1	JH378829.1	Johnsonella ignava ATCC 51276	Bacteria	Firmicutes	Clostridia	Clostridiales	Lachnospiraceae	Johnsonella	hypothetical protein						
256499..257329	array		JH378829.1	Johnsonella ignava ATCC 51276	Bacteria	Firmicutes	Clostridia	Clostridiales	Lachnospiraceae	Johnsonella		CRISPR array			12	38	34
257498..258656	-	EHI56554.1	JH378829.1	Johnsonella ignava ATCC 51276	Bacteria	Firmicutes	Clostridia	Clostridiales	Lachnospiraceae	Johnsonella	csm5 family CRISPR-associated ramp protein	csm5gr7	cd09662	CAS-III-A			
258685..259753	-	EHI56555.1	JH378829.1	Johnsonella ignava ATCC 51276	Bacteria	Firmicutes	Clostridia	Clostridiales	Lachnospiraceae	Johnsonella	csm4 family CRISPR-associated ramp protein	csm4gr5,csm4gr5	cd09663,cd09	CAS-III-A			
259742..260459	-	EHI56556.1	JH378829.1	Johnsonella ignava ATCC 51276	Bacteria	Firmicutes	Clostridia	Clostridiales	Lachnospiraceae	Johnsonella	csm3 family CRISPR-associated ramp protein	csm3gr7	cd09684	CAS-III-A,CAS-III-D			
260471..260900	-	EHI56557.1	JH378829.1	Johnsonella ignava ATCC 51276	Bacteria	Firmicutes	Clostridia	Clostridiales	Lachnospiraceae	Johnsonella	hypothetical protein	csm2gr11	cd09647	CAS-III-A			
260902..261955	-	EHI56558.1	JH378829.1	Johnsonella ignava ATCC 51276	Bacteria	Firmicutes	Clostridia	Clostridiales	Lachnospiraceae	Johnsonella	hypothetical protein	cas10	cd09680	CAS-III			
261969..263391	-	EHI56559.1	JH378829.1	Johnsonella ignava ATCC 51276	Bacteria	Firmicutes	Clostridia	Clostridiales	Lachnospiraceae	Johnsonella	hypothetical protein	cas10	cd09680	CAS-III			
263410..264163	-	EHI56560.1	JH378829.1	Johnsonella ignava ATCC 51276	Bacteria	Firmicutes	Clostridia	Clostridiales	Lachnospiraceae	Johnsonella	CRISPR-associated protein cas6	cas6	cd09652	CAS-I,CAS-III			
264317..265631	-	EHI56561.1	JH378829.1	Johnsonella ignava ATCC 51276	Bacteria	Firmicutes	Clostridia	Clostridiales	Lachnospiraceae	Johnsonella	hypothetical protein	C3a214	cluster214				
14 CAS-III-A | EGG91032.1																	
2021..2333	-	EGG91031.1	GL890597.1	Lachnospiraceae oral taxon 107 str. F0167	Bacteria	Firmicutes	Clostridia	Clostridiales	Lachnospiraceae		CRISPR-associated protein cas2	cas2	cd09725	CAS-I,CAS-II,CAS-III,CAS-V			
2340..3345	-	EGG91032.1	GL890597.1	Lachnospiraceae oral taxon 107 str. F0167	Bacteria	Firmicutes	Clostridia	Clostridiales	Lachnospiraceae		CRISPR-associated protein cas1	cas1	cd09634	CAS-I,CAS-II,CAS-III,CAS-V			
3354..4500	-	EGG91033.1	GL890597.1	Lachnospiraceae oral taxon 107 str. F0167	Bacteria	Firmicutes	Clostridia	Clostridiales	Lachnospiraceae		csm5 family CRISPR-associated ramp protein	csm5gr7	cd09662	CAS-III-A			
4499..5441	-	EGG91034.1	GL890597.1	Lachnospiraceae oral taxon 107 str. F0167	Bacteria	Firmicutes	Clostridia	Clostridiales	Lachnospiraceae		csm4 family CRISPR-associated ramp protein	csm4gr5	COG1567	CAS-III-A			
5442..6117	-	EGG91035.1	GL890597.1	Lachnospiraceae oral taxon 107 str. F0167	Bacteria	Firmicutes	Clostridia	Clostridiales	Lachnospiraceae		csm3 family CRISPR-associated ramp protein	csm3gr7	cd09684	CAS-III-A,CAS-III-D			
6129..6570	-	EGG91036.1	GL890597.1	Lachnospiraceae oral taxon 107 str. F0167	Bacteria	Firmicutes	Clostridia	Clostridiales	Lachnospiraceae		hypothetical protein	csm2gr11	cd09647	CAS-III-A			
6570..8778	-	EGG91037.1	GL890597.1	Lachnospiraceae oral taxon 107 str. F0167	Bacteria	Firmicutes	Clostridia	Clostridiales	Lachnospiraceae		csm1 family CRISPR-associated protein	cas10	cd09680	CAS-III			
8770..9502	-	EGG91038.1	GL890597.1	Lachnospiraceae oral taxon 107 str. F0167	Bacteria	Firmicutes	Clostridia	Clostridiales	Lachnospiraceae		CRISPR-associated protein cas6	cas6	pfam10040	CAS-III,CAS-IV			
9495..10884	-	EGG91039.1	GL890597.1	Lachnospiraceae oral taxon 107 str. F0167	Bacteria	Firmicutes	Clostridia	Clostridiales	Lachnospiraceae		hypothetical protein	csm6	cd09746	CAS-III-A			
11042..11675	-	EGG91040.1	GL890597.1	Lachnospiraceae oral taxon 107 str. F0167	Bacteria	Firmicutes	Clostridia	Clostridiales	Lachnospiraceae		hypothetical protein						
12336..12504	-	EGG91041.1	GL890597.1	Lachnospiraceae oral taxon 107 str. F0167	Bacteria	Firmicutes	Clostridia	Clostridiales	Lachnospiraceae		hypothetical protein						
13082..13342	array		GL890597.1	Lachnospiraceae oral taxon 107 str. F0167	Bacteria	Firmicutes	Clostridia	Clostridiales	Lachnospiraceae			CRISPR array			4	37	37
15 CAS-III-D | EJZ68972.1																	
2798538..2799459	+	EJZ68961.1	JH815185.1	Lachnoanaerobaculum sp. OBRC5-5	Bacteria	Firmicutes	Clostridia	Clostridiales	Lachnospiraceae	Lachnoanaerobaculum	hypothetical protein	C3a178	cluster178				
2801325..2802060	+	EJZ68962.1	JH815185.1	Lachnoanaerobaculum sp. OBRC5-5	Bacteria	Firmicutes	Clostridia	Clostridiales	Lachnospiraceae	Lachnoanaerobaculum	CRISPR-associated endoribonuclease cas6	cas6	COG5551	CAS-III			
2802162..2802261	+	EJZ68963.1	JH815185.1	Lachnoanaerobaculum sp. OBRC5-5	Bacteria	Firmicutes	Clostridia	Clostridiales	Lachnospiraceae	Lachnoanaerobaculum	hypothetical protein						
2802483..2803128	+	EJZ68964.1	JH815185.1	Lachnoanaerobaculum sp. OBRC5-5	Bacteria	Firmicutes	Clostridia	Clostridiales	Lachnospiraceae	Lachnoanaerobaculum	hypothetical protein	csx1	cd09732	CAS-III			
2803145..2804444	+	EJZ68965.1	JH815185.1	Lachnoanaerobaculum sp. OBRC5-5	Bacteria	Firmicutes	Clostridia	Clostridiales	Lachnospiraceae	Lachnoanaerobaculum	hypothetical protein						
2804455..2806036	+	EJZ68966.1	JH815185.1	Lachnoanaerobaculum sp. OBRC5-5	Bacteria	Firmicutes	Clostridia	Clostridiales	Lachnospiraceae	Lachnoanaerobaculum	hypothetical protein	cas10	mkCas0133	CAS-III			
2806028..2808038	+	EJZ68967.1	JH815185.1	Lachnoanaerobaculum sp. OBRC5-5	Bacteria	Firmicutes	Clostridia	Clostridiales	Lachnospiraceae	Lachnoanaerobaculum	hypothetical protein	csm3gr7,csx10gr5	mkCas0198,cl	CAS-III-A,CAS-III-D			
2808034..2809258	+	EJZ68968.1	JH815185.1	Lachnoanaerobaculum sp. OBRC5-5	Bacteria	Firmicutes	Clostridia	Clostridiales	Lachnospiraceae	Lachnoanaerobaculum	hypothetical protein	csm3gr7,csm3gr7	cd09726,pfan	CAS-III-D			
2809260..2809776	+	EJZ68969.1	JH815185.1	Lachnoanaerobaculum sp. OBRC5-5	Bacteria	Firmicutes	Clostridia	Clostridiales	Lachnospiraceae	Lachnoanaerobaculum	tigr03984 family CRISPR-associated protein	csx19,csx19	mkCas0147,cl	CAS-III-D			
2809777..2811808	+	EJZ68970.1	JH815185.1	Lachnoanaerobaculum sp. OBRC5-5	Bacteria	Firmicutes	Clostridia	Clostridiales	Lachnospiraceae	Lachnoanaerobaculum	CRISPR-associated protein	csm3gr7	cls000253	CAS-III-A,CAS-III-D			
2811809..2813153	+	EJZ68971.1	JH815185.1	Lachnoanaerobaculum sp. OBRC5-5	Bacteria	Firmicutes	Clostridia	Clostridiales	Lachnospiraceae	Lachnoanaerobaculum	tm1812 family CRISPR-associated protein	csx1	cd09732	CAS-III			
2813170..2814175	+	EJZ68972.1	JH815185.1	Lachnoanaerobaculum sp. OBRC5-5	Bacteria	Firmicutes	Clostridia	Clostridiales	Lachnospiraceae	Lachnoanaerobaculum	CRISPR-associated endonuclease cas1	cas1	cd09634	CAS-I,CAS-II,CAS-III,CAS-V			
2814174..2814471	+	EJZ68973.1	JH815185.1	Lachnoanaerobaculum sp. OBRC5-5	Bacteria	Firmicutes	Clostridia	Clostridiales	Lachnospiraceae	Lachnoanaerobaculum	CRISPR-associated endoribonuclease cas2	cas2	cd09725	CAS-I,CAS-II,CAS-III,CAS-V			
2814506..2816663	+	EJZ68974.1	JH815185.1	Lachnoanaerobaculum sp. OBRC5-5	Bacteria	Firmicutes	Clostridia	Clostridiales	Lachnospiraceae	Lachnoanaerobaculum	anaerobic ribonucleoside-triphosphate reductase						
2816952..2818337	array		JH815185.1	Lachnoanaerobaculum sp. OBRC5-5	Bacteria	Firmicutes	Clostridia	Clostridiales	Lachnospiraceae	Lachnoanaerobaculum		CRISPR array			19	39	36
2818482..2819382	+	EJZ68975.1	JH815185.1	Lachnoanaerobaculum sp. OBRC5-5	Bacteria	Firmicutes	Clostridia	Clostridiales	Lachnospiraceae	Lachnoanaerobaculum	hypothetical protein	C3a178	cluster178				
16 CAS-III-A | RGF90396.1																	
359..1115	+	RGF90390.1	QTVU01000076.1	Firmicutes bacterium AM55-24TS	Bacteria	Firmicutes					CRISPR system precrRNA processing endoribonuclease RAMP protein Cas6	cas6	pfam10040	CAS-III,CAS-IV			
1101..3372	+	RGF90391.1	QTVU01000076.1	Firmicutes bacterium AM55-24TS	Bacteria	Firmicutes					type III-A CRISPR-associated protein Cas10/Csm1	cas10,cas10	cd09680,cls0(	CAS-III			
3378..3750	+	RGF90392.1	QTVU01000076.1	Firmicutes bacterium AM55-24TS	Bacteria	Firmicutes					type III-A CRISPR-associated protein Csm2	csm2gr11	cd09647	CAS-III-A			
3752..4427	+	RGF90393.1	QTVU01000076.1	Firmicutes bacterium AM55-24TS	Bacteria	Firmicutes					type III-A CRISPR-associated RAMP protein Csm3	csm3gr7	cd09684	CAS-III-A,CAS-III-D			
4430..5357	+	RGF90394.1	QTVU01000076.1	Firmicutes bacterium AM55-24TS	Bacteria	Firmicutes					type III-A CRISPR-associated RAMP protein Csm4	csm4gr5	cd09663	CAS-III-A			
5367..6459	+	RGF90395.1	QTVU01000076.1	Firmicutes bacterium AM55-24TS	Bacteria	Firmicutes					type III-A CRISPR-associated RAMP protein Csm5	csm5gr7	cd09662	CAS-III-A			
6489..7491	+	RGF90396.1	QTVU01000076.1	Firmicutes bacterium AM55-24TS	Bacteria	Firmicutes					CRISPR-associated endonuclease Cas1	cas1	cd09634	CAS-I,CAS-II,CAS-III,CAS-V			
7516..7828	+	RGF90397.1	QTVU01000076.1	Firmicutes bacterium AM55-24TS	Bacteria	Firmicutes					CRISPR-associated endonuclease Cas2	cas2	cd09725	CAS-I,CAS-II,CAS-III,CAS-V			
8065..8243	array		QTVU01000076.1	Firmicutes bacterium AM55-24TS	Bacteria	Firmicutes						CRISPR array			3	37	35
17 CAS-III-A | EAX48915.1																	
198420..200423	array		AAWL01000001.1	Thermosinus carboxydivorans Nor1	Bacteria	Firmicutes	Negativicutes	Selenomonadales	Sporomusaceae	Thermosinus		CRISPR array			27	40	35
200646..201645	-	EAX48905.1	AAWL01000001.1	Thermosinus carboxydivorans Nor1	Bacteria	Firmicutes	Negativicutes	Selenomonadales	Sporomusaceae	Thermosinus	CRISPR-associated protein Cas1	cas1	cd09634	CAS-I,CAS-II,CAS-III,CAS-V			
201664..202000	-	EAX49041.1	AAWL01000001.1	Thermosinus carboxydivorans Nor1	Bacteria	Firmicutes	Negativicutes	Selenomonadales	Sporomusaceae	Thermosinus	CRISPR-associated protein Cas2	cas2	cd09725	CAS-I,CAS-II,CAS-III,CAS-V			
202009..203311	-	EAX48906.1	AAWL01000001.1	Thermosinus carboxydivorans Nor1	Bacteria	Firmicutes	Negativicutes	Selenomonadales	Sporomusaceae	Thermosinus	hypothetical protein	csx1	cd09747	CAS-III			
203325..204516	-	EAX48907.1	AAWL01000001.1	Thermosinus carboxydivorans Nor1	Bacteria	Firmicutes	Negativicutes	Selenomonadales	Sporomusaceae	Thermosinus	hypothetical protein	csx1	pfam09002	CAS-III			
204522..205386	-	EAX48908.1	AAWL01000001.1	Thermosinus carboxydivorans Nor1	Bacteria	Firmicutes	Negativicutes	Selenomonadales	Sporomusaceae	Thermosinus	conserved hypothetical protein	cas6	pfam10040	CAS-III,CAS-IV			
205382..206567	-	EAX48909.1	AAWL01000001.1	Thermosinus carboxydivorans Nor1	Bacteria	Firmicutes	Negativicutes	Selenomonadales	Sporomusaceae	Thermosinus	CRISPR-associated RAMP protein, Csm5 family	csm5gr7	COG1332	CAS-III-A			
206563..207589	-	EAX48910.1	AAWL01000001.1	Thermosinus carboxydivorans Nor1	Bacteria	Firmicutes	Negativicutes	Selenomonadales	Sporomusaceae	Thermosinus	CRISPR-associated RAMP protein, Csm4 family	csm4gr5	COG1567	CAS-III-A			
207569..208286	-	EAX48911.1	AAWL01000001.1	Thermosinus carboxydivorans Nor1	Bacteria	Firmicutes	Negativicutes	Selenomonadales	Sporomusaceae	Thermosinus	CRISPR-associated RAMP protein, Csm3 family	csm3gr7	cd09684	CAS-III-A,CAS-III-D			
208288..208693	-	EAX48912.1	AAWL01000001.1	Thermosinus carboxydivorans Nor1	Bacteria	Firmicutes	Negativicutes	Selenomonadales	Sporomusaceae	Thermosinus	CRISPR-associated protein, Csm2 family	csm2gr11	pfam03750	CAS-III-A			
208692..211209	-	EAX48913.1	AAWL01000001.1	Thermosinus carboxydivorans Nor1	Bacteria	Firmicutes	Negativicutes	Selenomonadales	Sporomusaceae	Thermosinus	CRISPR-associated protein, Csm1 family	cas10	cd09680	CAS-III			
211443..214298	array		AAWL01000001.1	Thermosinus carboxydivorans Nor1	Bacteria	Firmicutes	Negativicutes	Selenomonadales	Sporomusaceae	Thermosinus		CRISPR array			37	43	35
214471..214807	-	EAX48914.1	AAWL01000001.1	Thermosinus carboxydivorans Nor1	Bacteria	Firmicutes	Negativicutes	Selenomonadales	Sporomusaceae	Thermosinus	CRISPR-associated protein Cas2	cas2	pfam09827	CAS-I,CAS-II,CAS-III			
214833..215832	-	EAX48915.1	AAWL01000001.1	Thermosinus carboxydivorans Nor1	Bacteria	Firmicutes	Negativicutes	Selenomonadales	Sporomusaceae	Thermosinus	CRISPR-associated protein Cas1	cas1	cd09634	CAS-I,CAS-II,CAS-III,CAS-V			

18 CAS-III-A | EFQ03341.1																	
25051..25823	array		GL538212.1	Megasphaera micronuciformis F0359	Bacteria	Firmicutes	Negativicutes	Veillonellales	Veillonellaceae	Megasphaera		CRISPR array			11	37	36
25464..25632	-	EFQ03444.1	GL538212.1	Megasphaera micronuciformis F0359	Bacteria	Firmicutes	Negativicutes	Veillonellales	Veillonellaceae	Megasphaera	hypothetical protein						
25696..25816	-	EFQ03445.1	GL538212.1	Megasphaera micronuciformis F0359	Bacteria	Firmicutes	Negativicutes	Veillonellales	Veillonellaceae	Megasphaera	hypothetical protein						
25820..26150	array		GL538212.1	Megasphaera micronuciformis F0359	Bacteria	Firmicutes	Negativicutes	Veillonellales	Veillonellaceae	Megasphaera		CRISPR array			5	37	36
26147..26617	array		GL538212.1	Megasphaera micronuciformis F0359	Bacteria	Firmicutes	Negativicutes	Veillonellales	Veillonellaceae	Megasphaera		CRISPR array			7	36	36
26161..27472	array		GL538212.1	Megasphaera micronuciformis F0359	Bacteria	Firmicutes	Negativicutes	Veillonellales	Veillonellaceae	Megasphaera		CRISPR array			17	59	21
26615..26869	array		GL538212.1	Megasphaera micronuciformis F0359	Bacteria	Firmicutes	Negativicutes	Veillonellales	Veillonellaceae	Megasphaera		CRISPR array			4	37	36
26727..26863	-	EFQ03401.1	GL538212.1	Megasphaera micronuciformis F0359	Bacteria	Firmicutes	Negativicutes	Veillonellales	Veillonellaceae	Megasphaera	hypothetical protein						
27721..28762	+	EFQ03341.1	GL538212.1	Megasphaera micronuciformis F0359	Bacteria	Firmicutes	Negativicutes	Veillonellales	Veillonellaceae	Megasphaera	CRISPR-associated endonuclease Cas1	cas1	cd09634	CAS-I,CAS-II,CAS-III,CAS-V			
28766..29069	+	EFQ03342.1	GL538212.1	Megasphaera micronuciformis F0359	Bacteria	Firmicutes	Negativicutes	Veillonellales	Veillonellaceae	Megasphaera	CRISPR-associated protein Cas2	cas2	cd09725	CAS-I,CAS-II,CAS-III,CAS-V			
29087..31544	+	EFQ03343.1	GL538212.1	Megasphaera micronuciformis F0359	Bacteria	Firmicutes	Negativicutes	Veillonellales	Veillonellaceae	Megasphaera	CRISPR-associated protein, Csm1 family	cas10	cd09680	CAS-III			
31559..32012	+	EFQ03344.1	GL538212.1	Megasphaera micronuciformis F0359	Bacteria	Firmicutes	Negativicutes	Veillonellales	Veillonellaceae	Megasphaera	CRISPR-associated protein, Csm2 family	csm2gr11	pfam03750	CAS-III-A			
31989..32700	+	EFQ03345.1	GL538212.1	Megasphaera micronuciformis F0359	Bacteria	Firmicutes	Negativicutes	Veillonellales	Veillonellaceae	Megasphaera	CRISPR-associated RAMP protein, Csm3 family	csm3gr7	cd09684	CAS-III-A,CAS-III-D			
32699..33704	+	EFQ03346.1	GL538212.1	Megasphaera micronuciformis F0359	Bacteria	Firmicutes	Negativicutes	Veillonellales	Veillonellaceae	Megasphaera	CRISPR-associated RAMP protein, Csm4 family	csm4gr5	COG1567	CAS-III-A			
33700..34927	+	EFQ03347.1	GL538212.1	Megasphaera micronuciformis F0359	Bacteria	Firmicutes	Negativicutes	Veillonellales	Veillonellaceae	Megasphaera	CRISPR-associated RAMP protein, Csm5 family	csm5gr7	cd09662	CAS-III-A			
34989..35685	+	EFQ03348.1	GL538212.1	Megasphaera micronuciformis F0359	Bacteria	Firmicutes	Negativicutes	Veillonellales	Veillonellaceae	Megasphaera	putative CRISPR-associated endoribonuclease Cas6	cas6	pfam10040	CAS-III,CAS-IV			
35691..37032	+	EFQ03349.1	GL538212.1	Megasphaera micronuciformis F0359	Bacteria	Firmicutes	Negativicutes	Veillonellales	Veillonellaceae	Megasphaera	putative CRISPR-associated protein, Csm6 family	csm6	pfam09659	CAS-III-A			
19 CAS-III-A | EFR42041.1																	
146124..148641	+	EFR42010.1	AENV01000002.1	Selenomonas sp. oral taxon 137 str. F0430	Bacteria	Firmicutes	Negativicutes	Selenomonadales	Selenomonadaceae	Selenomonas	CRISPR-associated protein, Csm1 family	cas10	cd09680	CAS-III			
148637..149021	+	EFR41917.1	AENV01000002.1	Selenomonas sp. oral taxon 137 str. F0430	Bacteria	Firmicutes	Negativicutes	Selenomonadales	Selenomonadaceae	Selenomonas	CRISPR-associated protein, Csm2 family	csm2gr11	pfam03750	CAS-III-A			
149020..149737	+	EFR42048.1	AENV01000002.1	Selenomonas sp. oral taxon 137 str. F0430	Bacteria	Firmicutes	Negativicutes	Selenomonadales	Selenomonadaceae	Selenomonas	CRISPR-associated RAMP protein, Csm3 family	csm3gr7	cd09684	CAS-III-A,CAS-III-D			
149738..150734	+	EFR41951.1	AENV01000002.1	Selenomonas sp. oral taxon 137 str. F0430	Bacteria	Firmicutes	Negativicutes	Selenomonadales	Selenomonadaceae	Selenomonas	CRISPR-associated RAMP protein, Csm4 family	csm4gr5	COG1567	CAS-III-A			
150734..151919	+	EFR41986.1	AENV01000002.1	Selenomonas sp. oral taxon 137 str. F0430	Bacteria	Firmicutes	Negativicutes	Selenomonadales	Selenomonadaceae	Selenomonas	CRISPR-associated RAMP protein, Csm5 family	csm5gr7	cd09662	CAS-III-A			
152283..152661	+	EFR41891.1	AENV01000002.1	Selenomonas sp. oral taxon 137 str. F0430	Bacteria	Firmicutes	Negativicutes	Selenomonadales	Selenomonadaceae	Selenomonas	hypothetical protein	cas6	pfam10040	CAS-III,CAS-IV			
152861..155944	array		AENV01000002.1	Selenomonas sp. oral taxon 137 str. F0430	Bacteria	Firmicutes	Negativicutes	Selenomonadales	Selenomonadaceae	Selenomonas		CRISPR array			43	37	35
156162..157164	+	EFR42041.1	AENV01000002.1	Selenomonas sp. oral taxon 137 str. F0430	Bacteria	Firmicutes	Negativicutes	Selenomonadales	Selenomonadaceae	Selenomonas	CRISPR-associated endonuclease Cas1	cas1	cd09634	CAS-I,CAS-II,CAS-III,CAS-V			
157183..157513	+	EFR41895.1	AENV01000002.1	Selenomonas sp. oral taxon 137 str. F0430	Bacteria	Firmicutes	Negativicutes	Selenomonadales	Selenomonadaceae	Selenomonas	CRISPR-associated protein Cas2	cas2	cd09725	CAS-I,CAS-II,CAS-III,CAS-V			
157683..159375	+	EFR41934.1	AENV01000002.1	Selenomonas sp. oral taxon 137 str. F0430	Bacteria	Firmicutes	Negativicutes	Selenomonadales	Selenomonadaceae	Selenomonas	hypothetical protein	cas10	mkCas0133	CAS-III			
159371..160049	+	EFR41913.1	AENV01000002.1	Selenomonas sp. oral taxon 137 str. F0430	Bacteria	Firmicutes	Negativicutes	Selenomonadales	Selenomonadaceae	Selenomonas	CRISPR-associated RAMP protein	csm3gr7	mkCas0198	CAS-III-A,CAS-III-D			
160030..161632	+	EFR42056.1	AENV01000002.1	Selenomonas sp. oral taxon 137 str. F0430	Bacteria	Firmicutes	Negativicutes	Selenomonadales	Selenomonadaceae	Selenomonas	hypothetical protein	csx10gr5,csx10gr5	cls000715,cls	CAS-III-D			
161628..163086	+	EFR41977.1	AENV01000002.1	Selenomonas sp. oral taxon 137 str. F0430	Bacteria	Firmicutes	Negativicutes	Selenomonadales	Selenomonadaceae	Selenomonas	CRISPR-associated RAMP protein	csm3gr7,csm3gr7	cd09726,pfan	CAS-III-D			
163082..163637	+	EFR42038.1	AENV01000002.1	Selenomonas sp. oral taxon 137 str. F0430	Bacteria	Firmicutes	Negativicutes	Selenomonadales	Selenomonadaceae	Selenomonas	CRISPR-associated protein, TIGR03984 family	csx19	cls001376	CAS-III-D			
163652..165740	+	EFR41963.1	AENV01000002.1	Selenomonas sp. oral taxon 137 str. F0430	Bacteria	Firmicutes	Negativicutes	Selenomonadales	Selenomonadaceae	Selenomonas	CRISPR-associated protein	csm3gr7,csm3gr7	cls000253,cls	CAS-III-A,CAS-III-D			
165736..166552	+	EFR42018.1	AENV01000002.1	Selenomonas sp. oral taxon 137 str. F0430	Bacteria	Firmicutes	Negativicutes	Selenomonadales	Selenomonadaceae	Selenomonas	putative CRISPR-associated endoribonuclease Cas6	cas6	pfam10040	CAS-III,CAS-IV			
166567..168877	+	EFR41955.1	AENV01000002.1	Selenomonas sp. oral taxon 137 str. F0430	Bacteria	Firmicutes	Negativicutes	Selenomonadales	Selenomonadaceae	Selenomonas	putative flagellar protein FliS	csx1,csx1	pfam09455,p	CAS-III			
168968..169244	+	EFR42027.1	AENV01000002.1	Selenomonas sp. oral taxon 137 str. F0430	Bacteria	Firmicutes	Negativicutes	Selenomonadales	Selenomonadaceae	Selenomonas	CRISPR-associated endoribonuclease Cas2	cas2	pfam09827	CAS-I,CAS-II,CAS-III			
169270..170122	+	EFR42035.1	AENV01000002.1	Selenomonas sp. oral taxon 137 str. F0430	Bacteria	Firmicutes	Negativicutes	Selenomonadales	Selenomonadaceae	Selenomonas	CRISPR-associated endonuclease Cas1	cas1	pfam01867	CAS-I,CAS-II,CAS-III			
170302..170935	+	EFR42019.1	AENV01000002.1	Selenomonas sp. oral taxon 137 str. F0430	Bacteria	Firmicutes	Negativicutes	Selenomonadales	Selenomonadaceae	Selenomonas	hypothetical protein	csx1	pfam09455	CAS-III			
171031..172168	+	EFR41970.1	AENV01000002.1	Selenomonas sp. oral taxon 137 str. F0430	Bacteria	Firmicutes	Negativicutes	Selenomonadales	Selenomonadaceae	Selenomonas	hypothetical protein	Csx1	cluster2				
172171..172978	-	EFR41910.1	AENV01000002.1	Selenomonas sp. oral taxon 137 str. F0430	Bacteria	Firmicutes	Negativicutes	Selenomonadales	Selenomonadaceae	Selenomonas	hypothetical protein	WYL	pfam13280	CAS-I,CAS-III			
173135..174350	-	EFR41999.1	AENV01000002.1	Selenomonas sp. oral taxon 137 str. F0430	Bacteria	Firmicutes	Negativicutes	Selenomonadales	Selenomonadaceae	Selenomonas	hypothetical protein	WYL	COG2378	CAS-I,CAS-III			
174768..175494	array		AENV01000002.1	Selenomonas sp. oral taxon 137 str. F0430	Bacteria	Firmicutes	Negativicutes	Selenomonadales	Selenomonadaceae	Selenomonas		CRISPR array			11	34	35
20 CAS-III-A | ADG82860.1																	
2078271..2080300	array		CP002028.1	Thermincola potens JR	Bacteria	Firmicutes	Clostridia	Clostridiales	Peptococcaceae	Thermincola		CRISPR array			28	37	36
2080473..2080809	-	ADG82859.1	CP002028.1	Thermincola potens JR	Bacteria	Firmicutes	Clostridia	Clostridiales	Peptococcaceae	Thermincola	CRISPR-associated protein Cas2	cas2	cd09725	CAS-I,CAS-II,CAS-III,CAS-V			
2080819..2081827	-	ADG82860.1	CP002028.1	Thermincola potens JR	Bacteria	Firmicutes	Clostridia	Clostridiales	Peptococcaceae	Thermincola	CRISPR-associated protein Cas1	cas1	cd09634	CAS-I,CAS-II,CAS-III,CAS-V			
2081823..2083206	-	ADG82861.1	CP002028.1	Thermincola potens JR	Bacteria	Firmicutes	Clostridia	Clostridiales	Peptococcaceae	Thermincola	CRISPR-associated protein Csm6	csm6	cd09746	CAS-III-A			
2083198..2083948	-	ADG82862.1	CP002028.1	Thermincola potens JR	Bacteria	Firmicutes	Clostridia	Clostridiales	Peptococcaceae	Thermincola	Protein of unknown function DUF2276	cas6	COG5551	CAS-III			
2083941..2085231	-	ADG82863.1	CP002028.1	Thermincola potens JR	Bacteria	Firmicutes	Clostridia	Clostridiales	Peptococcaceae	Thermincola	CRISPR-associated RAMP protein, Csm5 family	csm5gr7	COG1332	CAS-III-A			
2085227..2086208	-	ADG82864.1	CP002028.1	Thermincola potens JR	Bacteria	Firmicutes	Clostridia	Clostridiales	Peptococcaceae	Thermincola	CRISPR-associated RAMP protein, Csm4 family	csm4gr5	COG1567	CAS-III-A			
2086204..2086948	-	ADG82865.1	CP002028.1	Thermincola potens JR	Bacteria	Firmicutes	Clostridia	Clostridiales	Peptococcaceae	Thermincola	CRISPR-associated RAMP protein, Csm3 family	csm3gr7	cd09684	CAS-III-A,CAS-III-D			
2086952..2087330	-	ADG82866.1	CP002028.1	Thermincola potens JR	Bacteria	Firmicutes	Clostridia	Clostridiales	Peptococcaceae	Thermincola	CRISPR-associated protein, Csm2 family	csm2gr11	cd09647	CAS-III-A			
2087322..2090037	-	ADG82867.1	CP002028.1	Thermincola potens JR	Bacteria	Firmicutes	Clostridia	Clostridiales	Peptococcaceae	Thermincola	CRISPR-associated protein, Csm1 family	cas10	cd09680	CAS-III			
21 CAS-III-A | EFG29102.1																	
646447..647933	array		GG770381.1	Fusobacterium periodonticum 1_1_41FAA	Bacteria	Fusobacteria	Fusobacteriia	Fusobacteriales	Fusobacteriaceae	Fusobacterium		CRISPR array			20	41	35
646614..646809	+	EFG29099.1	GG770381.1	Fusobacterium periodonticum 1_1_41FAA	Bacteria	Fusobacteria	Fusobacteriia	Fusobacteriales	Fusobacteriaceae	Fusobacterium	hypothetical protein						
647709..647889	+	EFG29100.1	GG770381.1	Fusobacterium periodonticum 1_1_41FAA	Bacteria	Fusobacteria	Fusobacteriia	Fusobacteriales	Fusobacteriaceae	Fusobacterium	hypothetical protein						
648181..648511	-	EFG29101.1	GG770381.1	Fusobacterium periodonticum 1_1_41FAA	Bacteria	Fusobacteria	Fusobacteriia	Fusobacteriales	Fusobacteriaceae	Fusobacterium	CRISPR-associated protein Cas2	cas2	cd09725	CAS-I,CAS-II,CAS-III,CAS-V			
648515..649523	-	EFG29102.1	GG770381.1	Fusobacterium periodonticum 1_1_41FAA	Bacteria	Fusobacteria	Fusobacteriia	Fusobacteriales	Fusobacteriaceae	Fusobacterium	CRISPR-associated endonuclease Cas1	cas1	cd09634	CAS-I,CAS-II,CAS-III,CAS-V			
649536..650928	-	EFG29103.1	GG770381.1	Fusobacterium periodonticum 1_1_41FAA	Bacteria	Fusobacteria	Fusobacteriia	Fusobacteriales	Fusobacteriaceae	Fusobacterium	putative CRISPR-associated protein, Csm6 family	csx1	pfam09670	CAS-III			
650920..651643	-	EFG29104.1	GG770381.1	Fusobacterium periodonticum 1_1_41FAA	Bacteria	Fusobacteria	Fusobacteriia	Fusobacteriales	Fusobacteriaceae	Fusobacterium	putative CRISPR-associated endoribonuclease Cas6	cas6	pfam10040	CAS-III,CAS-IV			
651626..652793	-	EFG29105.1	GG770381.1	Fusobacterium periodonticum 1_1_41FAA	Bacteria	Fusobacteria	Fusobacteriia	Fusobacteriales	Fusobacteriaceae	Fusobacterium	CRISPR-associated RAMP protein, Csm5 family	csm5gr7	cd09662	CAS-III-A			
652789..653794	-	EFG29106.1	GG770381.1	Fusobacterium periodonticum 1_1_41FAA	Bacteria	Fusobacteria	Fusobacteriia	Fusobacteriales	Fusobacteriaceae	Fusobacterium	CRISPR-associated RAMP protein, Csm4 family	csm4gr5	cd09663	CAS-III-A			
653793..654501	-	EFG29107.1	GG770381.1	Fusobacterium periodonticum 1_1_41FAA	Bacteria	Fusobacteria	Fusobacteriia	Fusobacteriales	Fusobacteriaceae	Fusobacterium	CRISPR-associated RAMP protein, Csm3 family	csm3gr7	cd09684	CAS-III-A,CAS-III-D			
654537..654897	-	EFG29108.1	GG770381.1	Fusobacterium periodonticum 1_1_41FAA	Bacteria	Fusobacteria	Fusobacteriia	Fusobacteriales	Fusobacteriaceae	Fusobacterium	CRISPR-associated protein, Csm2 family	csm2gr11	cd09647	CAS-III-A			
654900..657441	-	EFG29109.1	GG770381.1	Fusobacterium periodonticum 1_1_41FAA	Bacteria	Fusobacteria	Fusobacteriia	Fusobacteriales	Fusobacteriaceae	Fusobacterium	CRISPR-associated protein, Csm1 family	cas10	cd09680	CAS-III			
22 CAS-III-A | EKA93813.1																	
31875..33645	+	EKA93805.1	JH815370.1	Fusobacterium periodonticum D10	Bacteria	Fusobacteria	Fusobacteriia	Fusobacteriales	Fusobacteriaceae	Fusobacterium	CRISPR-associated protein cas10/csm1, subtype III-a/mtube	cas10	cd09680	CAS-III			
33648..34017	+	EKA93806.1	JH815370.1	Fusobacterium periodonticum D10	Bacteria	Fusobacteria	Fusobacteriia	Fusobacteriales	Fusobacteriaceae	Fusobacterium	CRISPR type III-a/mtube-associated protein csm2	csm2gr11	cd09647	CAS-III-A			
34053..34761	+	EKA93807.1	JH815370.1	Fusobacterium periodonticum D10	Bacteria	Fusobacteria	Fusobacteriia	Fusobacteriales	Fusobacteriaceae	Fusobacterium	CRISPR type III-a/mtube-associated ramp protein csm3	csm3gr7	cd09684	CAS-III-A,CAS-III-D			
34760..35768	+	EKA93808.1	JH815370.1	Fusobacterium periodonticum D10	Bacteria	Fusobacteria	Fusobacteriia	Fusobacteriales	Fusobacteriaceae	Fusobacterium	CRISPR type III-a/mtube-associated ramp protein csm4	csm4gr5	cd09663	CAS-III-A			
35764..36934	+	EKA93809.1	JH815370.1	Fusobacterium periodonticum D10	Bacteria	Fusobacteria	Fusobacteriia	Fusobacteriales	Fusobacteriaceae	Fusobacterium	CRISPR type III-a/mtube-associated ramp protein csm5	csm5gr7	COG1332	CAS-III-A			
36917..37640	+	EKA93810.1	JH815370.1	Fusobacterium periodonticum D10	Bacteria	Fusobacteria	Fusobacteriia	Fusobacteriales	Fusobacteriaceae	Fusobacterium	CRISPR-associated endoribonuclease cas6	cas6	pfam10040	CAS-III,CAS-IV			
37632..38763	+	EKA93811.1	JH815370.1	Fusobacterium periodonticum D10	Bacteria	Fusobacteria	Fusobacteriia	Fusobacteriales	Fusobacteriaceae	Fusobacterium	hypothetical protein	csm6	pfam09659	CAS-III-A			
38771..39023	+	EKA93812.1	JH815370.1	Fusobacterium periodonticum D10	Bacteria	Fusobacteria	Fusobacteriia	Fusobacteriales	Fusobacteriaceae	Fusobacterium	hypothetical protein	csx1	mkCas0109	CAS-III			
39036..40044	+	EKA93813.1	JH815370.1	Fusobacterium periodonticum D10	Bacteria	Fusobacteria	Fusobacteriia	Fusobacteriales	Fusobacteriaceae	Fusobacterium	CRISPR-associated endonuclease cas1	cas1	cd09634	CAS-I,CAS-II,CAS-III,CAS-V			
40048..40378	+	EKA93814.1	JH815370.1	Fusobacterium periodonticum D10	Bacteria	Fusobacteria	Fusobacteriia	Fusobacteriales	Fusobacteriaceae	Fusobacterium	CRISPR-associated endoribonuclease cas2	cas2	cd09725	CAS-I,CAS-II,CAS-III,CAS-V			
40627..40821	array		JH815370.1	Fusobacterium periodonticum D10	Bacteria	Fusobacteria	Fusobacteriia	Fusobacteriales	Fusobacteriaceae	Fusobacterium		CRISPR array			3	43	36
23 CAS-III-A | ABI69047.1																	
1994951..1996193	-	ABI69044.1	CP000448.1	Syntrophomonas wolfei subsp. wolfei str. Goettingen G311	Bacteria	Firmicutes	Clostridia	Clostridiales	Syntrophomonadaceae	Syntrophomonas	hypothetical protein	csm6	pfam09659	CAS-III-A			
1996473..1997694	-	ABI69045.1	CP000448.1	Syntrophomonas wolfei subsp. wolfei str. Goettingen G311	Bacteria	Firmicutes	Clostridia	Clostridiales	Syntrophomonadaceae	Syntrophomonas	hypothetical protein						
1997890..1998220	-	ABI69046.1	CP000448.1	Syntrophomonas wolfei subsp. wolfei str. Goettingen G311	Bacteria	Firmicutes	Clostridia	Clostridiales	Syntrophomonadaceae	Syntrophomonas	CRISPR-associated protein, Cas2 family	cas2	cd09725	CAS-I,CAS-II,CAS-III,CAS-V			
1998216..1999227	-	ABI69047.1	CP000448.1	Syntrophomonas wolfei subsp. wolfei str. Goettingen G311	Bacteria	Firmicutes	Clostridia	Clostridiales	Syntrophomonadaceae	Syntrophomonas	CRISPR-associated protein, Cas1 family	cas1	cd09634	CAS-I,CAS-II,CAS-III,CAS-V			
1999255..2000383	array		CP000448.1	Syntrophomonas wolfei subsp. wolfei str. Goettingen G311	Bacteria	Firmicutes	Clostridia	Clostridiales	Syntrophomonadaceae	Syntrophomonas		CRISPR array			16	36	36
2000509..2001787	+	ABI69048.1	CP000448.1	Syntrophomonas wolfei subsp. wolfei str. Goettingen G311	Bacteria	Firmicutes	Clostridia	Clostridiales	Syntrophomonadaceae	Syntrophomonas	transposase	c2c4*	c2c4				
2002263..2002514	array		CP000448.1	Syntrophomonas wolfei subsp. wolfei str. Goettingen G311	Bacteria	Firmicutes	Clostridia	Clostridiales	Syntrophomonadaceae	Syntrophomonas		CRISPR array			4	36	36
2002711..2003461	-	ABI69049.1	CP000448.1	Syntrophomonas wolfei subsp. wolfei str. Goettingen G311	Bacteria	Firmicutes	Clostridia	Clostridiales	Syntrophomonadaceae	Syntrophomonas	CRISPR-associated protein, Cas6 family	cas6	COG5551	CAS-III			
2003611..2004754	-	ABI69050.1	CP000448.1	Syntrophomonas wolfei subsp. wolfei str. Goettingen G311	Bacteria	Firmicutes	Clostridia	Clostridiales	Syntrophomonadaceae	Syntrophomonas	CRISPR-associated protein, Csm5 family	csm5gr7	COG1332	CAS-III-A			
2004750..2005698	-	ABI69051.1	CP000448.1	Syntrophomonas wolfei subsp. wolfei str. Goettingen G311	Bacteria	Firmicutes	Clostridia	Clostridiales	Syntrophomonadaceae	Syntrophomonas	CRISPR-associated protein, Csm4 family	csm4gr5	COG1567	CAS-III-A			
2005672..2006374	-	ABI69052.1	CP000448.1	Syntrophomonas wolfei subsp. wolfei str. Goettingen G311	Bacteria	Firmicutes	Clostridia	Clostridiales	Syntrophomonadaceae	Syntrophomonas	CRISPR-associated protein, Csm3 family	csm3gr7	cd09684	CAS-III-A,CAS-III-D			
2006387..2006864	-	ABI69053.1	CP000448.1	Syntrophomonas wolfei subsp. wolfei str. Goettingen G311	Bacteria	Firmicutes	Clostridia	Clostridiales	Syntrophomonadaceae	Syntrophomonas	hypothetical protein	csm2gr11	pfam03750	CAS-III-A			
2006856..2009283	-	ABI69054.1	CP000448.1	Syntrophomonas wolfei subsp. wolfei str. Goettingen G311	Bacteria	Firmicutes	Clostridia	Clostridiales	Syntrophomonadaceae	Syntrophomonas	CRISPR-associated protein, Csm1 family	cas10	cd09680	CAS-III			
25 CAS-VI-A | WP_062808097.1																	
1869431..1869746	-	WP_062808329.	NZ_LT546010.1	Blautia sp. Marseille-P2398	Bacteria	Firmicutes	Clostridia	Clostridiales	Lachnospiraceae	Blautia	CRISPR-associated endonuclease Cas2	cas2	cd09725	CAS-I,CAS-II,CAS-III,CAS-V			
1869756..1870758	-	WP_062808097.	NZ_LT546010.1	Blautia sp. Marseille-P2398	Bacteria	Firmicutes	Clostridia	Clostridiales	Lachnospiraceae	Blautia	CRISPR-associated endonuclease Cas1	cas1	cd09634	CAS-I,CAS-II,CAS-III,CAS-V			
1870784..1871888	array		NZ_LT546010.1	Blautia sp. Marseille-P2398	Bacteria	Firmicutes	Clostridia	Clostridiales	Lachnospiraceae	Blautia		CRISPR array			16	35	36
1872030..1875861	-	WP_062808098.	NZ_LT546010.1	Blautia sp. Marseille-P2398	Bacteria	Firmicutes	Clostridia	Clostridiales	Lachnospiraceae	Blautia	hypothetical protein	cas13a	cas13a	CAS-VI-A			
26 CAS-VI-A | SEQ67608.1																	
72908..77072	+	SEQ67578.1	FOEK01000016.1	Lachnospiraceae bacterium NE2001	Bacteria	Firmicutes	Clostridia	Clostridiales	Lachnospiraceae		hypothetical protein	cas13a	cas13a	CAS-VI-A			
77161..78199	+	SEQ67608.1	FOEK01000016.1	Lachnospiraceae bacterium NE2001	Bacteria	Firmicutes	Clostridia	Clostridiales	Lachnospiraceae		CRISP-associated protein Cas1	cas1	cd09634	CAS-I,CAS-II,CAS-III,CAS-V			
78207..78495	+	SEQ67639.1	FOEK01000016.1	Lachnospiraceae bacterium NE2001	Bacteria	Firmicutes	Clostridia	Clostridiales	Lachnospiraceae		CRISPR associated protein Cas2	cas2	cd09725	CAS-I,CAS-II,CAS-III,CAS-V			
78625..79288	array		FOEK01000016.1	Lachnospiraceae bacterium NE2001	Bacteria	Firmicutes	Clostridia	Clostridiales	Lachnospiraceae			CRISPR array			10	33	36
27 CAS-III-A | EEF92713.1																	
43627..45062	array		ACCK01000438.1	Catenibacterium mitsuokai DSM 15897	Bacteria	Firmicutes	Erysipelotrichia	Erysipelotrichales	Erysipelotrichaceae	Catenibacterium		CRISPR array			20	38	35
43655..43808	+	EEF92709.1	ACCK01000438.1	Catenibacterium mitsuokai DSM 15897	Bacteria	Firmicutes	Erysipelotrichia	Erysipelotrichales	Erysipelotrichaceae	Catenibacterium	hypothetical protein						
44774..44993	+	EEF92710.1	ACCK01000438.1	Catenibacterium mitsuokai DSM 15897	Bacteria	Firmicutes	Erysipelotrichia	Erysipelotrichales	Erysipelotrichaceae	Catenibacterium	hypothetical protein						
45249..46587	-	EEF92711.1	ACCK01000438.1	Catenibacterium mitsuokai DSM 15897	Bacteria	Firmicutes	Erysipelotrichia	Erysipelotrichales	Erysipelotrichaceae	Catenibacterium	putative CRISPR-associated protein, Csm6 family	csm6	cd09746	CAS-III-A			
46588..46909	-	EEF92712.1	ACCK01000438.1	Catenibacterium mitsuokai DSM 15897	Bacteria	Firmicutes	Erysipelotrichia	Erysipelotrichales	Erysipelotrichaceae	Catenibacterium	CRISPR-associated protein Cas2	cas2	cd09725	CAS-I,CAS-II,CAS-III,CAS-V			
46914..47913	-	EEF92713.1	ACCK01000438.1	Catenibacterium mitsuokai DSM 15897	Bacteria	Firmicutes	Erysipelotrichia	Erysipelotrichales	Erysipelotrichaceae	Catenibacterium	CRISPR-associated endonuclease Cas1	cas1	cd09634	CAS-I,CAS-II,CAS-III,CAS-V			

47978..49121	-	EEF92714.1	ACCK01000438.1	Catenibacterium mitsuokai DSM 15897	Bacteria	Firmicutes	Erysipelotrichia	Erysipelotrichales	Erysipelotrichaceae	Catenibacterium	CRISPR-associated RAMP protein, Csm5 family	csm5gr7	cd09662	CAS-III-A			
49117..50035	-	EEF92715.1	ACCK01000438.1	Catenibacterium mitsuokai DSM 15897	Bacteria	Firmicutes	Erysipelotrichia	Erysipelotrichales	Erysipelotrichaceae	Catenibacterium	CRISPR-associated RAMP protein, Csm4 family	csm4gr5	COG1567	CAS-III-A			
50053..50719	-	EEF92716.1	ACCK01000438.1	Catenibacterium mitsuokai DSM 15897	Bacteria	Firmicutes	Erysipelotrichia	Erysipelotrichales	Erysipelotrichaceae	Catenibacterium	CRISPR-associated RAMP protein, Csm3 family	csm3gr7	cd09684	CAS-III-A,CAS-III-D			
50723..51101	-	EEF92717.1	ACCK01000438.1	Catenibacterium mitsuokai DSM 15897	Bacteria	Firmicutes	Erysipelotrichia	Erysipelotrichales	Erysipelotrichaceae	Catenibacterium	CRISPR-associated protein, Csm2 family	csm2gr11	cd09647	CAS-III-A			
51104..53453	-	EEF92718.1	ACCK01000438.1	Catenibacterium mitsuokai DSM 15897	Bacteria	Firmicutes	Erysipelotrichia	Erysipelotrichales	Erysipelotrichaceae	Catenibacterium	CRISPR-associated protein, Csm1 family	cas10	cd09680	CAS-III			
53449..54193	-	EEF92719.1	ACCK01000438.1	Catenibacterium mitsuokai DSM 15897	Bacteria	Firmicutes	Erysipelotrichia	Erysipelotrichales	Erysipelotrichaceae	Catenibacterium	CRISPR-associated endoribonuclease Cas6	cas6	pfam10040	CAS-III,CAS-IV			
54581..55286	array		ACCK01000438.1	Catenibacterium mitsuokai DSM 15897	Bacteria	Firmicutes	Erysipelotrichia	Erysipelotrichales	Erysipelotrichaceae	Catenibacterium		CRISPR array			10	39	35
28 CAS-III-A | SER78299.1																	
25105..25840	+	SER78179.1	FOGW01000010.1	Lachnobacterium bovis	Bacteria	Firmicutes	Clostridia	Clostridiales	Lachnospiraceae	Lachnobacterium	CRISPR-associated endoribonuclease Cas6	cas6	COG5551	CAS-III			
25843..28270	+	SER78199.1	FOGW01000010.1	Lachnobacterium bovis	Bacteria	Firmicutes	Clostridia	Clostridiales	Lachnospiraceae	Lachnobacterium	CRISPR-associated protein Csm1	cas10	cd09680	CAS-III			
28277..28742	+	SER78218.1	FOGW01000010.1	Lachnobacterium bovis	Bacteria	Firmicutes	Clostridia	Clostridiales	Lachnospiraceae	Lachnobacterium	CRISPR-associated protein Csm2	csm2gr11	pfam03750	CAS-III-A			
28761..29442	+	SER78242.1	FOGW01000010.1	Lachnobacterium bovis	Bacteria	Firmicutes	Clostridia	Clostridiales	Lachnospiraceae	Lachnobacterium	CRISPR-associated protein Csm3	csm3gr7	cd09684	CAS-III-A,CAS-III-D			
29444..30386	+	SER78262.1	FOGW01000010.1	Lachnobacterium bovis	Bacteria	Firmicutes	Clostridia	Clostridiales	Lachnospiraceae	Lachnobacterium	CRISPR-associated protein Csm4	csm4gr5	COG1567	CAS-III-A			
30385..31525	+	SER78280.1	FOGW01000010.1	Lachnobacterium bovis	Bacteria	Firmicutes	Clostridia	Clostridiales	Lachnospiraceae	Lachnobacterium	CRISPR-associated protein Csm5	csm5gr7	cd09662	CAS-III-A			
31550..32552	+	SER78299.1	FOGW01000010.1	Lachnobacterium bovis	Bacteria	Firmicutes	Clostridia	Clostridiales	Lachnospiraceae	Lachnobacterium	CRISP-associated protein Cas1	cas1	cd09634	CAS-I,CAS-II,CAS-III,CAS-V			
32563..32881	+	SER78324.1	FOGW01000010.1	Lachnobacterium bovis	Bacteria	Firmicutes	Clostridia	Clostridiales	Lachnospiraceae	Lachnobacterium	CRISPR-associated protein Cas2	cas2	cd09725	CAS-I,CAS-II,CAS-III,CAS-V			
32903..34253	+	SER78348.1	FOGW01000010.1	Lachnobacterium bovis	Bacteria	Firmicutes	Clostridia	Clostridiales	Lachnospiraceae	Lachnobacterium	CRISPR type III-A/MTUBE-associated protein Csm6	csm6	pfam09659	CAS-III-A			
29 CAS-III-A | EFV01914.1																	
1259337..1260747	-	EFV01912.1	GL622359.1	Pseudoramibacter alactolyticus ATCC 23263	Bacteria	Firmicutes	Clostridia	Clostridiales	Eubacteriaceae	Pseudoramibacter	putative CRISPR-associated protein, Csm6 family	csm6	cd09746	CAS-III-A			
1260767..1261091	-	EFV01913.1	GL622359.1	Pseudoramibacter alactolyticus ATCC 23263	Bacteria	Firmicutes	Clostridia	Clostridiales	Eubacteriaceae	Pseudoramibacter	CRISPR-associated protein Cas2	cas2	cd09725	CAS-I,CAS-II,CAS-III,CAS-V			
1261095..1262094	-	EFV01914.1	GL622359.1	Pseudoramibacter alactolyticus ATCC 23263	Bacteria	Firmicutes	Clostridia	Clostridiales	Eubacteriaceae	Pseudoramibacter	CRISPR-associated endonuclease Cas1	cas1	cd09634	CAS-I,CAS-II,CAS-III,CAS-V			
1262135..1262454	array		GL622359.1	Pseudoramibacter alactolyticus ATCC 23263	Bacteria	Firmicutes	Clostridia	Clostridiales	Eubacteriaceae	Pseudoramibacter		CRISPR array			5	35	36
1262300..1262468	+	EFV01915.1	GL622359.1	Pseudoramibacter alactolyticus ATCC 23263	Bacteria	Firmicutes	Clostridia	Clostridiales	Eubacteriaceae	Pseudoramibacter	hypothetical protein						
1262448..1262628	-	EFV01916.1	GL622359.1	Pseudoramibacter alactolyticus ATCC 23263	Bacteria	Firmicutes	Clostridia	Clostridiales	Eubacteriaceae	Pseudoramibacter	hypothetical protein						
1262650..1262830	-	EFV01917.1	GL622359.1	Pseudoramibacter alactolyticus ATCC 23263	Bacteria	Firmicutes	Clostridia	Clostridiales	Eubacteriaceae	Pseudoramibacter	hypothetical protein						
1262826..1262946	-	EFV01918.1	GL622359.1	Pseudoramibacter alactolyticus ATCC 23263	Bacteria	Firmicutes	Clostridia	Clostridiales	Eubacteriaceae	Pseudoramibacter	hypothetical protein						
1263015..1263775	array		GL622359.1	Pseudoramibacter alactolyticus ATCC 23263	Bacteria	Firmicutes	Clostridia	Clostridiales	Eubacteriaceae	Pseudoramibacter		CRISPR array			11	36	36
1263769..1263943	-	EFV01919.1	GL622359.1	Pseudoramibacter alactolyticus ATCC 23263	Bacteria	Firmicutes	Clostridia	Clostridiales	Eubacteriaceae	Pseudoramibacter	hypothetical protein						
1263908..1265000	-	EFV01920.1	GL622359.1	Pseudoramibacter alactolyticus ATCC 23263	Bacteria	Firmicutes	Clostridia	Clostridiales	Eubacteriaceae	Pseudoramibacter	CRISPR-associated RAMP protein, Csm5 family	csm5gr7	cd09662	CAS-III-A			
1264996..1265941	-	EFV01921.1	GL622359.1	Pseudoramibacter alactolyticus ATCC 23263	Bacteria	Firmicutes	Clostridia	Clostridiales	Eubacteriaceae	Pseudoramibacter	CRISPR-associated RAMP protein, Csm4 family	csm4gr5	COG1567	CAS-III-A			
1265937..1266639	-	EFV01922.1	GL622359.1	Pseudoramibacter alactolyticus ATCC 23263	Bacteria	Firmicutes	Clostridia	Clostridiales	Eubacteriaceae	Pseudoramibacter	CRISPR-associated RAMP protein, Csm3 family	csm3gr7	cd09684	CAS-III-A,CAS-III-D			
1266644..1267043	-	EFV01923.1	GL622359.1	Pseudoramibacter alactolyticus ATCC 23263	Bacteria	Firmicutes	Clostridia	Clostridiales	Eubacteriaceae	Pseudoramibacter	CRISPR-associated protein, Csm2 family	csm2gr11	cd09647	CAS-III-A			
1267029..1269441	-	EFV01924.1	GL622359.1	Pseudoramibacter alactolyticus ATCC 23263	Bacteria	Firmicutes	Clostridia	Clostridiales	Eubacteriaceae	Pseudoramibacter	CRISPR-associated protein, Csm1 family	cas10	cd09680	CAS-III			
1269430..1270195	-	EFV01925.1	GL622359.1	Pseudoramibacter alactolyticus ATCC 23263	Bacteria	Firmicutes	Clostridia	Clostridiales	Eubacteriaceae	Pseudoramibacter	putative CRISPR-associated endoribonuclease Cas6	cas6	COG5551	CAS-III			
30 CAS-III-A | SET01848.1																	
91436..92186	+	SET01630.1	FOIL01000003.1	[Clostridium] aminophilum	Bacteria	Firmicutes	Clostridia	Clostridiales	Lachnospiraceae	Lachnoclostridium	CRISPR-associated endoribonuclease Cas6	cas6	COG5551	CAS-III			
92187..94542	+	SET01664.1	FOIL01000003.1	[Clostridium] aminophilum	Bacteria	Firmicutes	Clostridia	Clostridiales	Lachnospiraceae	Lachnoclostridium	CRISPR-associated protein Csm1	cas10	cd09680	CAS-III			
94538..94934	+	SET01695.1	FOIL01000003.1	[Clostridium] aminophilum	Bacteria	Firmicutes	Clostridia	Clostridiales	Lachnospiraceae	Lachnoclostridium	CRISPR-associated protein Csm2	csm2gr11	cd09647	CAS-III-A			
94945..95611	+	SET01723.1	FOIL01000003.1	[Clostridium] aminophilum	Bacteria	Firmicutes	Clostridia	Clostridiales	Lachnospiraceae	Lachnoclostridium	CRISPR-associated protein Csm3	csm3gr7	cd09684	CAS-III-A,CAS-III-D			
95626..96454	+	SET01759.1	FOIL01000003.1	[Clostridium] aminophilum	Bacteria	Firmicutes	Clostridia	Clostridiales	Lachnospiraceae	Lachnoclostridium	CRISPR-associated protein Csm4	csm4gr5	COG1567	CAS-III-A			
96546..97647	+	SET01802.1	FOIL01000003.1	[Clostridium] aminophilum	Bacteria	Firmicutes	Clostridia	Clostridiales	Lachnospiraceae	Lachnoclostridium	CRISPR-associated protein Csm5	csm5gr7,csm5gr7	cd09662,COG	CAS-III-A			
97788..98670	array		FOIL01000003.1	[Clostridium] aminophilum	Bacteria	Firmicutes	Clostridia	Clostridiales	Lachnospiraceae	Lachnoclostridium		CRISPR array			13	35	35
98718..99720	+	SET01848.1	FOIL01000003.1	[Clostridium] aminophilum	Bacteria	Firmicutes	Clostridia	Clostridiales	Lachnospiraceae	Lachnoclostridium	CRISP-associated protein Cas1	cas1	cd09634	CAS-I,CAS-II,CAS-III,CAS-V			
100055..101363	+	SET01885.1	FOIL01000003.1	[Clostridium] aminophilum	Bacteria	Firmicutes	Clostridia	Clostridiales	Lachnospiraceae	Lachnoclostridium	CRISPR type III-A/MTUBE-associated protein Csm6	csm6	cd09746	CAS-III-A			
31 CAS-III-A | SFR79451.1																	
97062..97812	+	SFR79406.1	FOZC01000008.1	[Clostridium] aminophilum	Bacteria	Firmicutes	Clostridia	Clostridiales	Lachnospiraceae	Lachnoclostridium	CRISPR-associated endoribonuclease Cas6	cas6	COG5551	CAS-III			
97813..100168	+	SFR79414.1	FOZC01000008.1	[Clostridium] aminophilum	Bacteria	Firmicutes	Clostridia	Clostridiales	Lachnospiraceae	Lachnoclostridium	CRISPR-associated protein Csm1	cas10	cd09680	CAS-III			
100164..100560	+	SFR79421.1	FOZC01000008.1	[Clostridium] aminophilum	Bacteria	Firmicutes	Clostridia	Clostridiales	Lachnospiraceae	Lachnoclostridium	CRISPR-associated protein Csm2	csm2gr11	cd09647	CAS-III-A			
100571..101237	+	SFR79430.1	FOZC01000008.1	[Clostridium] aminophilum	Bacteria	Firmicutes	Clostridia	Clostridiales	Lachnospiraceae	Lachnoclostridium	CRISPR-associated protein Csm3	csm3gr7	cd09684	CAS-III-A,CAS-III-D			
101252..102170	+	SFR79436.1	FOZC01000008.1	[Clostridium] aminophilum	Bacteria	Firmicutes	Clostridia	Clostridiales	Lachnospiraceae	Lachnoclostridium	CRISPR-associated protein Csm4	csm4gr5	COG1567	CAS-III-A			
102173..103274	+	SFR79443.1	FOZC01000008.1	[Clostridium] aminophilum	Bacteria	Firmicutes	Clostridia	Clostridiales	Lachnospiraceae	Lachnoclostridium	CRISPR-associated protein Csm5	csm5gr7,csm5gr7	cd09662,COG	CAS-III-A			
103415..103930	array		FOZC01000008.1	[Clostridium] aminophilum	Bacteria	Firmicutes	Clostridia	Clostridiales	Lachnospiraceae	Lachnoclostridium		CRISPR array			8	32	36
103978..104980	+	SFR79451.1	FOZC01000008.1	[Clostridium] aminophilum	Bacteria	Firmicutes	Clostridia	Clostridiales	Lachnospiraceae	Lachnoclostridium	CRISP-associated protein Cas1	cas1	cd09634	CAS-I,CAS-II,CAS-III,CAS-V			
105315..106623	+	SFR79457.1	FOZC01000008.1	[Clostridium] aminophilum	Bacteria	Firmicutes	Clostridia	Clostridiales	Lachnospiraceae	Lachnoclostridium	CRISPR type III-A/MTUBE-associated protein Csm6	csm6	cd09746	CAS-III-A			
32 CAS-III-D | EMZ39669.1																	
178..428	array		KB822471.1	Eubacterium plexicaudatum ASF492	Bacteria	Firmicutes	Clostridia	Clostridiales	Eubacteriaceae	Eubacterium		CRISPR array			4	33	38
793..1795	+	EMZ39669.1	KB822471.1	Eubacterium plexicaudatum ASF492	Bacteria	Firmicutes	Clostridia	Clostridiales	Eubacteriaceae	Eubacterium	CRISPR-associated endonuclease cas1	cas1	cd09634	CAS-I,CAS-II,CAS-III,CAS-V			
1912..2119	+	EMZ39670.1	KB822471.1	Eubacterium plexicaudatum ASF492	Bacteria	Firmicutes	Clostridia	Clostridiales	Eubacteriaceae	Eubacterium	CRISPR-associated endoribonuclease cas2	cas2	cd09725	CAS-I,CAS-II,CAS-III,CAS-V			
2135..2981	+	EMZ39671.1	KB822471.1	Eubacterium plexicaudatum ASF492	Bacteria	Firmicutes	Clostridia	Clostridiales	Eubacteriaceae	Eubacterium	hypothetical protein						
3000..4518	+	EMZ39672.1	KB822471.1	Eubacterium plexicaudatum ASF492	Bacteria	Firmicutes	Clostridia	Clostridiales	Eubacteriaceae	Eubacterium	tm1812 family CRISPR-associated protein	csx1	cd09732	CAS-III			
4563..6048	+	EMZ39673.1	KB822471.1	Eubacterium plexicaudatum ASF492	Bacteria	Firmicutes	Clostridia	Clostridiales	Eubacteriaceae	Eubacterium	hypothetical protein	cas10	mkCas0133	CAS-III			
6056..6638	+	EMZ39674.1	KB822471.1	Eubacterium plexicaudatum ASF492	Bacteria	Firmicutes	Clostridia	Clostridiales	Eubacteriaceae	Eubacterium	hypothetical protein	csm3gr7	mkCas0198	CAS-III-A,CAS-III-D			
6651..8178	+	EMZ39675.1	KB822471.1	Eubacterium plexicaudatum ASF492	Bacteria	Firmicutes	Clostridia	Clostridiales	Eubacteriaceae	Eubacterium	hypothetical protein	csx10gr5	cls000715	CAS-III-D			
8178..9531	+	EMZ39676.1	KB822471.1	Eubacterium plexicaudatum ASF492	Bacteria	Firmicutes	Clostridia	Clostridiales	Eubacteriaceae	Eubacterium	hypothetical protein	csm3gr7,csm3gr7	pfam03787,p	CAS-III-A,CAS-III-D			
9527..9965	+	EMZ39677.1	KB822471.1	Eubacterium plexicaudatum ASF492	Bacteria	Firmicutes	Clostridia	Clostridiales	Eubacteriaceae	Eubacterium	hypothetical protein	csx19	mkCas0147	CAS-III-D			
9968..11930	+	EMZ39678.1	KB822471.1	Eubacterium plexicaudatum ASF492	Bacteria	Firmicutes	Clostridia	Clostridiales	Eubacteriaceae	Eubacterium	CRISPR-associated protein	csm3gr7	cls000253	CAS-III-A,CAS-III-D			
11984..13538	+	EMZ39679.1	KB822471.1	Eubacterium plexicaudatum ASF492	Bacteria	Firmicutes	Clostridia	Clostridiales	Eubacteriaceae	Eubacterium	tigr02710 family CRISPR-associated protein	csx1	cd09747	CAS-III			
13564..14992	+	EMZ39680.1	KB822471.1	Eubacterium plexicaudatum ASF492	Bacteria	Firmicutes	Clostridia	Clostridiales	Eubacteriaceae	Eubacterium	hypothetical protein						
14936..15326	+	EMZ39681.1	KB822471.1	Eubacterium plexicaudatum ASF492	Bacteria	Firmicutes	Clostridia	Clostridiales	Eubacteriaceae	Eubacterium	hypothetical protein						
15687..16056	+	EMZ39682.1	KB822471.1	Eubacterium plexicaudatum ASF492	Bacteria	Firmicutes	Clostridia	Clostridiales	Eubacteriaceae	Eubacterium	hypothetical protein						
16055..18944	+	EMZ39683.1	KB822471.1	Eubacterium plexicaudatum ASF492	Bacteria	Firmicutes	Clostridia	Clostridiales	Eubacteriaceae	Eubacterium	hypothetical protein						
19092..20433	+	EMZ39684.1	KB822471.1	Eubacterium plexicaudatum ASF492	Bacteria	Firmicutes	Clostridia	Clostridiales	Eubacteriaceae	Eubacterium	MATE efflux family protein						
20470..21232	+	EMZ39685.1	KB822471.1	Eubacterium plexicaudatum ASF492	Bacteria	Firmicutes	Clostridia	Clostridiales	Eubacteriaceae	Eubacterium	CRISPR-associated endoribonuclease cas6	cas6	COG5551	CAS-III			
34 partial | CCX82921.1																	
334..1336	+	CCX82921.1	HF987621.1	Eubacterium sp. CAG:86	Bacteria	Firmicutes	Clostridia	Clostridiales	Eubacteriaceae	Eubacterium	cRISPR-associated endonuclease Cas1	cas1	cd09634	CAS-I,CAS-II,CAS-III,CAS-V			
1344..1689	+	CCX82922.1	HF987621.1	Eubacterium sp. CAG:86	Bacteria	Firmicutes	Clostridia	Clostridiales	Eubacteriaceae	Eubacterium	cRISPR-associated endoribonuclease Cas2 2	cas2	pfam09827	CAS-I,CAS-II,CAS-III			
35 CAS-III-A | OKZ95402.1																	
1471..2209	+	OKZ95396.1	MNSH01000002.1	Eubacterium sp. 36_13	Bacteria	Firmicutes	Clostridia	Clostridiales	Eubacteriaceae	Eubacterium	CRISPR-associated endoribonuclease Cas6	cas6	COG5551	CAS-III			
2215..4684	+	OKZ95397.1	MNSH01000002.1	Eubacterium sp. 36_13	Bacteria	Firmicutes	Clostridia	Clostridiales	Eubacteriaceae	Eubacterium	type III-A CRISPR-associated protein Cas10/Csm1	cas10	cd09680	CAS-III			
4667..5105	+	OKZ95398.1	MNSH01000002.1	Eubacterium sp. 36_13	Bacteria	Firmicutes	Clostridia	Clostridiales	Eubacteriaceae	Eubacterium	type III-A CRISPR-associated protein Csm2	csm2gr11	cd09647	CAS-III-A			
5117..5792	+	OKZ95399.1	MNSH01000002.1	Eubacterium sp. 36_13	Bacteria	Firmicutes	Clostridia	Clostridiales	Eubacteriaceae	Eubacterium	type III-A CRISPR-associated RAMP protein Csm3	csm3gr7	cd09684	CAS-III-A,CAS-III-D			
5802..6726	+	OKZ95400.1	MNSH01000002.1	Eubacterium sp. 36_13	Bacteria	Firmicutes	Clostridia	Clostridiales	Eubacteriaceae	Eubacterium	type III-A CRISPR-associated RAMP protein Csm4	csm4gr5	COG1567	CAS-III-A			
6722..7850	+	OKZ95401.1	MNSH01000002.1	Eubacterium sp. 36_13	Bacteria	Firmicutes	Clostridia	Clostridiales	Eubacteriaceae	Eubacterium	type III-A CRISPR-associated RAMP protein Csm5	csm5gr7,csm5gr7	cd09662,cd0	CAS-III-A			
8065..9310	array		MNSH01000002.1	Eubacterium sp. 36_13	Bacteria	Firmicutes	Clostridia	Clostridiales	Eubacteriaceae	Eubacterium		CRISPR array			18	36	35
9403..10405	+	OKZ95402.1	MNSH01000002.1	Eubacterium sp. 36_13	Bacteria	Firmicutes	Clostridia	Clostridiales	Eubacteriaceae	Eubacterium	CRISPR-associated endonuclease Cas1	cas1	cd09634	CAS-I,CAS-II,CAS-III,CAS-V			
10413..10707	+	OKZ95403.1	MNSH01000002.1	Eubacterium sp. 36_13	Bacteria	Firmicutes	Clostridia	Clostridiales	Eubacteriaceae	Eubacterium	CRISPR-associated endonuclease Cas2	cas2	cd09725	CAS-I,CAS-II,CAS-III,CAS-V			
36 CAS-III-A | CCX73455.1																	
54366..55686	-	CCX73451.1	HF987137.1	Dorea sp. CAG:105	Bacteria	Firmicutes	Clostridia	Clostridiales	Lachnospiraceae	Dorea	putative uncharacterized protein	csm6	cd09746	CAS-III-A			
55700..56021	-	CCX73453.1	HF987137.1	Dorea sp. CAG:105	Bacteria	Firmicutes	Clostridia	Clostridiales	Lachnospiraceae	Dorea	cRISPR-associated endoribonuclease Cas2 2	cas2	cd09725	CAS-I,CAS-II,CAS-III,CAS-V			
56032..57034	-	CCX73455.1	HF987137.1	Dorea sp. CAG:105	Bacteria	Firmicutes	Clostridia	Clostridiales	Lachnospiraceae	Dorea	cRISPR-associated endonuclease Cas1	cas1	cd09634	CAS-I,CAS-II,CAS-III,CAS-V			
57361..57571	-	CCX73456.1	HF987137.1	Dorea sp. CAG:105	Bacteria	Firmicutes	Clostridia	Clostridiales	Lachnospiraceae	Dorea	putative uncharacterized protein						
58069..59339	array		HF987137.1	Dorea sp. CAG:105	Bacteria	Firmicutes	Clostridia	Clostridiales	Lachnospiraceae	Dorea		CRISPR array			18	37	35
58074..58599	array		HF987137.1	Dorea sp. CAG:105	Bacteria	Firmicutes	Clostridia	Clostridiales	Lachnospiraceae	Dorea		CRISPR array			8	42	29
59474..60596	-	CCX73457.1	HF987137.1	Dorea sp. CAG:105	Bacteria	Firmicutes	Clostridia	Clostridiales	Lachnospiraceae	Dorea	cRISPR-associated protein	csm5gr7,csm5gr7	cd09662,cd0	CAS-III-A			
60592..61513	-	CCX73458.1	HF987137.1	Dorea sp. CAG:105	Bacteria	Firmicutes	Clostridia	Clostridiales	Lachnospiraceae	Dorea	cRISPR-associated protein	csm4gr5	COG1567	CAS-III-A			
61534..62200	-	CCX73459.1	HF987137.1	Dorea sp. CAG:105	Bacteria	Firmicutes	Clostridia	Clostridiales	Lachnospiraceae	Dorea	putative uncharacterized protein	csm3gr7	cd09684	CAS-III-A,CAS-III-D			
62221..62623	-	CCX73460.1	HF987137.1	Dorea sp. CAG:105	Bacteria	Firmicutes	Clostridia	Clostridiales	Lachnospiraceae	Dorea	cRISPR-associated protein Csm2 family	csm2gr11	cd09647	CAS-III-A			
62626..64975	-	CCX73461.1	HF987137.1	Dorea sp. CAG:105	Bacteria	Firmicutes	Clostridia	Clostridiales	Lachnospiraceae	Dorea	putative uncharacterized protein	cas10	cd09680	CAS-III			
64985..65711	-	CCX73462.1	HF987137.1	Dorea sp. CAG:105	Bacteria	Firmicutes	Clostridia	Clostridiales	Lachnospiraceae	Dorea	putative uncharacterized protein	cas6	COG5551	CAS-III			
65991..66735	-	CCX73463.1	HF987137.1	Dorea sp. CAG:105	Bacteria	Firmicutes	Clostridia	Clostridiales	Lachnospiraceae	Dorea	putative uncharacterized protein	Cas_RecF	cluster17				
37 CAS-III-A | CUO70210.1																	
15999..17328	+	CUO69714.1	CYZL01000021.1	Anaerobutyricum hallii	Bacteria	Firmicutes	Clostridia	Clostridiales	Lachnospiraceae	Anaerobutyricum	CRISPR type III-A/MTUBE-associated protein Csm6	csm6	cd09746	CAS-III-A			
17329..18085	+	CUO69760.1	CYZL01000021.1	Anaerobutyricum hallii	Bacteria	Firmicutes	Clostridia	Clostridiales	Lachnospiraceae	Anaerobutyricum	Uncharacterized protein predicted to be involved in DNA repair (RAMP superfamily)	cas6	pfam10040	CAS-III,CAS-IV			
18074..20447	+	CUO69866.1	CYZL01000021.1	Anaerobutyricum hallii	Bacteria	Firmicutes	Clostridia	Clostridiales	Lachnospiraceae	Anaerobutyricum	CRISPR-associated protein Cas10/Csm1%2C subtype III-A/MTUBE	cas10	cd09680	CAS-III			
20430..20820	+	CUO69962.1	CYZL01000021.1	Anaerobutyricum hallii	Bacteria	Firmicutes	Clostridia	Clostridiales	Lachnospiraceae	Anaerobutyricum	CRISPR type III-A/MTUBE-associated protein Csm2	csm2gr11	cd09647	CAS-III-A			
20829..21492	+	CUO70065.1	CYZL01000021.1	Anaerobutyricum hallii	Bacteria	Firmicutes	Clostridia	Clostridiales	Lachnospiraceae	Anaerobutyricum	CRISPR type III-A/MTUBE-associated RAMP protein Csm3	csm3gr7	cd09684	CAS-III-A,CAS-III-D			
21495..23529	+	CUO70173.1	CYZL01000021.1	Anaerobutyricum hallii	Bacteria	Firmicutes	Clostridia	Clostridiales	Lachnospiraceae	Anaerobutyricum	CRISPR type III-A/MTUBE-associated RAMP protein Csm4	csm4gr5,csm5gr7	cd09663,cd0	CAS-III-A			
23691..24312	array		CYZL01000021.1	Anaerobutyricum hallii	Bacteria	Firmicutes	Clostridia	Clostridiales	Lachnospiraceae	Anaerobutyricum		CRISPR array			9	37	36
24346..25348	+	CUO70210.1	CYZL01000021.1	Anaerobutyricum hallii	Bacteria	Firmicutes	Clostridia	Clostridiales	Lachnospiraceae	Anaerobutyricum	CRISPR-associated endonuclease Cas1	cas1	cd09634	CAS-I,CAS-II,CAS-III,CAS-V			
25325..25682	+	CUO70252.1	CYZL01000021.1	Anaerobutyricum hallii	Bacteria	Firmicutes	Clostridia	Clostridiales	Lachnospiraceae	Anaerobutyricum	CRISPR-associated endoribonuclease Cas2	cas2	cd09725	CAS-I,CAS-II,CAS-III,CAS-V			

38 CAS-III-A | CUQ01118.1																	
23691..25014	-	CUQ01060.1	CZBJ01000007.1	[Ruminococcus] torques	Bacteria	Firmicutes	Clostridia	Clostridiales	Lachnospiraceae	Blautia	CRISPR type III-A/MTUBE-associated protein Csm6	csm6	pfam09659	CAS-III-A			
25026..25365	-	CUQ01087.1	CZBJ01000007.1	[Ruminococcus] torques	Bacteria	Firmicutes	Clostridia	Clostridiales	Lachnospiraceae	Blautia	CRISPR-associated endoribonuclease Cas2	cas2	cd09725	CAS-I,CAS-II,CAS-III,CAS-V			
25342..26344	-	CUQ01118.1	CZBJ01000007.1	[Ruminococcus] torques	Bacteria	Firmicutes	Clostridia	Clostridiales	Lachnospiraceae	Blautia	CRISPR-associated endonuclease Cas1	cas1	cd09634	CAS-I,CAS-II,CAS-III,CAS-V			
26362..27758	array		CZBJ01000007.1	[Ruminococcus] torques	Bacteria	Firmicutes	Clostridia	Clostridiales	Lachnospiraceae	Blautia		CRISPR array			20	35	36
27330..27717	-	CUQ01149.1	CZBJ01000007.1	[Ruminococcus] torques	Bacteria	Firmicutes	Clostridia	Clostridiales	Lachnospiraceae	Blautia	Uncharacterised protein						
27892..28990	-	CUQ01183.1	CZBJ01000007.1	[Ruminococcus] torques	Bacteria	Firmicutes	Clostridia	Clostridiales	Lachnospiraceae	Blautia	CRISPR type III-A/MTUBE-associated RAMP protein Csm5	csm5gr7	cd09662	CAS-III-A			
28986..29913	-	CUQ01212.1	CZBJ01000007.1	[Ruminococcus] torques	Bacteria	Firmicutes	Clostridia	Clostridiales	Lachnospiraceae	Blautia	CRISPR type III-A/MTUBE-associated RAMP protein Csm4	csm4gr5	COG1567	CAS-III-A			
29932..30598	-	CUQ01242.1	CZBJ01000007.1	[Ruminococcus] torques	Bacteria	Firmicutes	Clostridia	Clostridiales	Lachnospiraceae	Blautia	CRISPR type III-A/MTUBE-associated RAMP protein Csm3	csm3gr7	cd09684	CAS-III-A,CAS-III-D			
30610..31012	-	CUQ01271.1	CZBJ01000007.1	[Ruminococcus] torques	Bacteria	Firmicutes	Clostridia	Clostridiales	Lachnospiraceae	Blautia	CRISPR type III-A/MTUBE-associated protein Csm2	csm2gr11	cd09647	CAS-III-A			
31013..33350	-	CUQ01300.1	CZBJ01000007.1	[Ruminococcus] torques	Bacteria	Firmicutes	Clostridia	Clostridiales	Lachnospiraceae	Blautia	CRISPR-associated protein Cas10/Csm1%2C subtype III-A/MTUBE	cas10	cd09680	CAS-III			
33342..34080	-	CUQ01334.1	CZBJ01000007.1	[Ruminococcus] torques	Bacteria	Firmicutes	Clostridia	Clostridiales	Lachnospiraceae	Blautia	CRISPR-associated endoribonuclease Cas6	cas6	COG5551	CAS-III			
39 CAS-III-A | SCG89713.1																	
45644..46964	-	SCG89711.1	FMDP01000019.1	uncultured Clostridium sp.	Bacteria	Firmicutes	Clostridia	Clostridiales	Clostridiaceae	Clostridium	CRISPR type III-A/MTUBE-associated protein Csm6	csm6	cd09746	CAS-III-A			
46975..47317	-	SCG89712.1	FMDP01000019.1	uncultured Clostridium sp.	Bacteria	Firmicutes	Clostridia	Clostridiales	Clostridiaceae	Clostridium	CRISPR-associated endoribonuclease Cas2	cas2	pfam09827	CAS-I,CAS-II,CAS-III			
47294..48296	-	SCG89713.1	FMDP01000019.1	uncultured Clostridium sp.	Bacteria	Firmicutes	Clostridia	Clostridiales	Clostridiaceae	Clostridium	CRISPR-associated endonuclease Cas1	cas1	cd09634	CAS-I,CAS-II,CAS-III,CAS-V			
48463..49814	array		FMDP01000019.1	uncultured Clostridium sp.	Bacteria	Firmicutes	Clostridia	Clostridiales	Clostridiaceae	Clostridium		CRISPR array			19	37	36
49948..51037	-	SCG89714.1	FMDP01000019.1	uncultured Clostridium sp.	Bacteria	Firmicutes	Clostridia	Clostridiales	Clostridiaceae	Clostridium	CRISPR type III-A/MTUBE-associated RAMP protein Csm5	csm5gr7	cd09662	CAS-III-A			
51033..51960	-	SCG89715.1	FMDP01000019.1	uncultured Clostridium sp.	Bacteria	Firmicutes	Clostridia	Clostridiales	Clostridiaceae	Clostridium	CRISPR type III-A/MTUBE-associated RAMP protein Csm4	csm4gr5	COG1567	CAS-III-A			
51971..52643	-	SCG89716.1	FMDP01000019.1	uncultured Clostridium sp.	Bacteria	Firmicutes	Clostridia	Clostridiales	Clostridiaceae	Clostridium	CRISPR type III-A/MTUBE-associated RAMP protein Csm3	csm3gr7	cd09684	CAS-III-A,CAS-III-D			
52657..53053	-	SCG89717.1	FMDP01000019.1	uncultured Clostridium sp.	Bacteria	Firmicutes	Clostridia	Clostridiales	Clostridiaceae	Clostridium	CRISPR type III-A/MTUBE-associated protein Csm2	csm2gr11	cd09647	CAS-III-A			
53054..55397	-	SCG89718.1	FMDP01000019.1	uncultured Clostridium sp.	Bacteria	Firmicutes	Clostridia	Clostridiales	Clostridiaceae	Clostridium	Predicted HD superfamily hydrolase	cas10	cd09680	CAS-III			
55371..56130	-	SCG89719.1	FMDP01000019.1	uncultured Clostridium sp.	Bacteria	Firmicutes	Clostridia	Clostridiales	Clostridiaceae	Clostridium	CRISPR-associated endoribonuclease Cas6	cas6	COG5551	CAS-III			
40 partial CAS-III-A | ERI97392.1																	
1..598	array		KE993343.1	Blautia sp. KLE 1732	Bacteria	Firmicutes	Clostridia	Clostridiales	Lachnospiraceae	Blautia		CRISPR array			9	42	29
463..640	+	ERI97391.1	KE993343.1	Blautia sp. KLE 1732	Bacteria	Firmicutes	Clostridia	Clostridiales	Lachnospiraceae	Blautia	hypothetical protein						
651..1668	+	ERI97392.1	KE993343.1	Blautia sp. KLE 1732	Bacteria	Firmicutes	Clostridia	Clostridiales	Lachnospiraceae	Blautia	CRISPR-associated endonuclease Cas1	cas1	cd09634	CAS-I,CAS-II,CAS-III,CAS-V			
1645..1981	+	ERI97393.1	KE993343.1	Blautia sp. KLE 1732	Bacteria	Firmicutes	Clostridia	Clostridiales	Lachnospiraceae	Blautia	CRISPR-associated protein Cas2	cas2	pfam09827	CAS-I,CAS-II,CAS-III			
2017..3331	+	ERI97394.1	KE993343.1	Blautia sp. KLE 1732	Bacteria	Firmicutes	Clostridia	Clostridiales	Lachnospiraceae	Blautia	CRISPR-associated protein, Csm6 family	csm6	cd09746	CAS-III-A			
41 CAS-III-A | EES78311.1																	
2298275..2299592	-	EES78308.1	ACII02000003.1	Ruminococcus sp. 5_1_39BFAA	Bacteria	Firmicutes	Clostridia	Clostridiales	Ruminococcaceae	Ruminococcus	CRISPR type III-a/mtube-associated protein csm6	csm6	cd09746	CAS-III-A			
2299625..2299961	-	EQM98871.1	ACII02000003.1	Ruminococcus sp. 5_1_39BFAA	Bacteria	Firmicutes	Clostridia	Clostridiales	Ruminococcaceae	Ruminococcus	CRISPR-associated endoribonuclease cas2	cas2	cd09725	CAS-I,CAS-II,CAS-III,CAS-V			
2299938..2300940	-	EES78311.1	ACII02000003.1	Ruminococcus sp. 5_1_39BFAA	Bacteria	Firmicutes	Clostridia	Clostridiales	Ruminococcaceae	Ruminococcus	CRISPR-associated endonuclease cas1	cas1	cd09634	CAS-I,CAS-II,CAS-III,CAS-V			
2301013..2302414	array		ACII02000003.1	Ruminococcus sp. 5_1_39BFAA	Bacteria	Firmicutes	Clostridia	Clostridiales	Ruminococcaceae	Ruminococcus		CRISPR array			20	35	36
2302581..2303676	-	EES78312.2	ACII02000003.1	Ruminococcus sp. 5_1_39BFAA	Bacteria	Firmicutes	Clostridia	Clostridiales	Ruminococcaceae	Ruminococcus	CRISPR type III-a/mtube-associated ramp protein csm5	csm5gr7	COG1332	CAS-III-A			
2303677..2304598	-	EES78313.1	ACII02000003.1	Ruminococcus sp. 5_1_39BFAA	Bacteria	Firmicutes	Clostridia	Clostridiales	Ruminococcaceae	Ruminococcus	CRISPR type III-a/mtube-associated ramp protein csm4	csm4gr5	COG1567	CAS-III-A			
2304618..2305284	-	EES78314.1	ACII02000003.1	Ruminococcus sp. 5_1_39BFAA	Bacteria	Firmicutes	Clostridia	Clostridiales	Ruminococcaceae	Ruminococcus	CRISPR type III-a/mtube-associated ramp protein csm3	csm3gr7	cd09684	CAS-III-A,CAS-III-D			
2305296..2305692	-	EES78315.1	ACII02000003.1	Ruminococcus sp. 5_1_39BFAA	Bacteria	Firmicutes	Clostridia	Clostridiales	Ruminococcaceae	Ruminococcus	CRISPR type III-a/mtube-associated protein csm2	csm2gr11	cd09647	CAS-III-A			
2305681..2308066	-	EES78316.2	ACII02000003.1	Ruminococcus sp. 5_1_39BFAA	Bacteria	Firmicutes	Clostridia	Clostridiales	Ruminococcaceae	Ruminococcus	CRISPR-associated protein cas10/csm1, subtype III-a/mtube	cas10	cd09680	CAS-III			
2308058..2308799	-	EES78317.1	ACII02000003.1	Ruminococcus sp. 5_1_39BFAA	Bacteria	Firmicutes	Clostridia	Clostridiales	Ruminococcaceae	Ruminococcus	CRISPR-associated endoribonuclease cas6	cas6	pfam10040	CAS-III,CAS-IV			
42 CAS-III-A | RJW30536.1																	
74705..75446	+	RJW30530.1	QVHO01000002.1	Ruminococcus sp. OM02-16LB	Bacteria	Firmicutes	Clostridia	Clostridiales	Ruminococcaceae	Ruminococcus	CRISPR-associated endoribonuclease Cas6	cas6	pfam10040	CAS-III,CAS-IV			
75432..77823	+	RJW30531.1	QVHO01000002.1	Ruminococcus sp. OM02-16LB	Bacteria	Firmicutes	Clostridia	Clostridiales	Ruminococcaceae	Ruminococcus	type III-A CRISPR-associated protein Cas10/Csm1	cas10	cd09680	CAS-III			
77812..78208	+	RJW30532.1	QVHO01000002.1	Ruminococcus sp. OM02-16LB	Bacteria	Firmicutes	Clostridia	Clostridiales	Ruminococcaceae	Ruminococcus	type III-A CRISPR-associated protein Csm2	csm2gr11	cd09647	CAS-III-A			
78220..78886	+	RJW30533.1	QVHO01000002.1	Ruminococcus sp. OM02-16LB	Bacteria	Firmicutes	Clostridia	Clostridiales	Ruminococcaceae	Ruminococcus	type III-A CRISPR-associated RAMP protein Csm3	csm3gr7	cd09684	CAS-III-A,CAS-III-D			
78906..79827	+	RJW30534.1	QVHO01000002.1	Ruminococcus sp. OM02-16LB	Bacteria	Firmicutes	Clostridia	Clostridiales	Ruminococcaceae	Ruminococcus	type III-A CRISPR-associated RAMP protein Csm4	csm4gr5	COG1567	CAS-III-A			
79708..80923	+	RJW30535.1	QVHO01000002.1	Ruminococcus sp. OM02-16LB	Bacteria	Firmicutes	Clostridia	Clostridiales	Ruminococcaceae	Ruminococcus	type III-A CRISPR-associated RAMP protein Csm5	csm5gr7	cd09662	CAS-III-A			
81091..82419	array		QVHO01000002.1	Ruminococcus sp. OM02-16LB	Bacteria	Firmicutes	Clostridia	Clostridiales	Ruminococcaceae	Ruminococcus		CRISPR array			19	35	36
82491..83493	+	RJW30536.1	QVHO01000002.1	Ruminococcus sp. OM02-16LB	Bacteria	Firmicutes	Clostridia	Clostridiales	Ruminococcaceae	Ruminococcus	CRISPR-associated endonuclease Cas1	cas1	cd09634	CAS-I,CAS-II,CAS-III,CAS-V			
83470..83806	+	RJW30537.1	QVHO01000002.1	Ruminococcus sp. OM02-16LB	Bacteria	Firmicutes	Clostridia	Clostridiales	Ruminococcaceae	Ruminococcus	CRISPR-associated endonuclease Cas2	cas2	cd09725	CAS-I,CAS-II,CAS-III,CAS-V			
83839..85156	+	RJW30538.1	QVHO01000002.1	Ruminococcus sp. OM02-16LB	Bacteria	Firmicutes	Clostridia	Clostridiales	Ruminococcaceae	Ruminococcus	CRISPR-associated protein Csm6	csm6	cd09746	CAS-III-A			
45 partial CAS-III-A | RGB74594.1																	
14899..16282	-	RGB74593.1	QVEP01000053.1	Coprococcus catus	Bacteria	Firmicutes	Clostridia	Clostridiales	Lachnospiraceae	Coprococcus	CRISPR-associated protein Csm6	csm6	pfam09659	CAS-III-A			
16343..16685	-	RGB74595.1	QVEP01000053.1	Coprococcus catus	Bacteria	Firmicutes	Clostridia	Clostridiales	Lachnospiraceae	Coprococcus	CRISPR-associated endonuclease Cas2	cas2	cd09725	CAS-I,CAS-II,CAS-III,CAS-V			
16731..17733	-	RGB74594.1	QVEP01000053.1	Coprococcus catus	Bacteria	Firmicutes	Clostridia	Clostridiales	Lachnospiraceae	Coprococcus	CRISPR-associated endonuclease Cas1	cas1	cd09634	CAS-I,CAS-II,CAS-III,CAS-V			
17771..18023	array		QVEP01000053.1	Coprococcus catus	Bacteria	Firmicutes	Clostridia	Clostridiales	Lachnospiraceae	Coprococcus		CRISPR array			4	39	34
46 CAS-III-A | RGC44628.1																	
12824..13562	+	RGC44622.1	QVFD01000014.1	Coprococcus catus	Bacteria	Firmicutes	Clostridia	Clostridiales	Lachnospiraceae	Coprococcus	CRISPR-associated endoribonuclease Cas6	cas6	COG5551	CAS-III			
13558..15919	+	RGC44623.1	QVFD01000014.1	Coprococcus catus	Bacteria	Firmicutes	Clostridia	Clostridiales	Lachnospiraceae	Coprococcus	type III-A CRISPR-associated protein Cas10/Csm1	cas10	cd09680	CAS-III			
15935..16346	+	RGC44624.1	QVFD01000014.1	Coprococcus catus	Bacteria	Firmicutes	Clostridia	Clostridiales	Lachnospiraceae	Coprococcus	type III-A CRISPR-associated protein Csm2	csm2gr11	cd09647	CAS-III-A			
16363..17029	+	RGC44625.1	QVFD01000014.1	Coprococcus catus	Bacteria	Firmicutes	Clostridia	Clostridiales	Lachnospiraceae	Coprococcus	type III-A CRISPR-associated RAMP protein Csm3	csm3gr7	cd09684	CAS-III-A,CAS-III-D			
17046..18012	+	RGC44626.1	QVFD01000014.1	Coprococcus catus	Bacteria	Firmicutes	Clostridia	Clostridiales	Lachnospiraceae	Coprococcus	type III-A CRISPR-associated RAMP protein Csm4	csm4gr5	COG1567	CAS-III-A			
18008..19130	+	RGC44627.1	QVFD01000014.1	Coprococcus catus	Bacteria	Firmicutes	Clostridia	Clostridiales	Lachnospiraceae	Coprococcus	type III-A CRISPR-associated RAMP protein Csm5	csm5gr7	COG1332	CAS-III-A			
19425..20978	array		QVFD01000014.1	Coprococcus catus	Bacteria	Firmicutes	Clostridia	Clostridiales	Lachnospiraceae	Coprococcus		CRISPR array			22	36	36
21013..22015	+	RGC44628.1	QVFD01000014.1	Coprococcus catus	Bacteria	Firmicutes	Clostridia	Clostridiales	Lachnospiraceae	Coprococcus	CRISPR-associated endonuclease Cas1	cas1	cd09634	CAS-I,CAS-II,CAS-III,CAS-V			
22061..22403	+	RGC44690.1	QVFD01000014.1	Coprococcus catus	Bacteria	Firmicutes	Clostridia	Clostridiales	Lachnospiraceae	Coprococcus	CRISPR-associated endonuclease Cas2	cas2	cd09725	CAS-I,CAS-II,CAS-III,CAS-V			
22464..23838	+	RGC44629.1	QVFD01000014.1	Coprococcus catus	Bacteria	Firmicutes	Clostridia	Clostridiales	Lachnospiraceae	Coprococcus	CRISPR-associated protein Csm6	csm6	pfam09659	CAS-III-A			
47 CAS-III-A | RRD93722.1																	
24032..25412	-	RRD93719.1	RQYD01000028.1	Clostridiales bacterium COT073_COT-073	Bacteria	Firmicutes	Clostridia	Clostridiales			CRISPR-associated protein Csm6	csm6	cd09746	CAS-III-A			
25426..25615	-	RRD93720.1	RQYD01000028.1	Clostridiales bacterium COT073_COT-073	Bacteria	Firmicutes	Clostridia	Clostridiales			hypothetical protein						
25625..27390	array		RQYD01000028.1	Clostridiales bacterium COT073_COT-073	Bacteria	Firmicutes	Clostridia	Clostridiales				CRISPR array			25	36	36
27633..27948	-	RRD93721.1	RQYD01000028.1	Clostridiales bacterium COT073_COT-073	Bacteria	Firmicutes	Clostridia	Clostridiales			CRISPR-associated endonuclease Cas2	cas2	cd09725	CAS-I,CAS-II,CAS-III,CAS-V			
27959..28958	-	RRD93722.1	RQYD01000028.1	Clostridiales bacterium COT073_COT-073	Bacteria	Firmicutes	Clostridia	Clostridiales			CRISPR-associated endonuclease Cas1	cas1	cd09634	CAS-I,CAS-II,CAS-III,CAS-V			
29211..30423	-	RRD93723.1	RQYD01000028.1	Clostridiales bacterium COT073_COT-073	Bacteria	Firmicutes	Clostridia	Clostridiales			type III-A CRISPR-associated RAMP protein Csm5	csm5gr7	COG1332	CAS-III-A			
30349..31279	-	RRD93724.1	RQYD01000028.1	Clostridiales bacterium COT073_COT-073	Bacteria	Firmicutes	Clostridia	Clostridiales			type III-A CRISPR-associated RAMP protein Csm4	csm4gr5	COG1567	CAS-III-A			
31288..31948	-	RRD93737.1	RQYD01000028.1	Clostridiales bacterium COT073_COT-073	Bacteria	Firmicutes	Clostridia	Clostridiales			type III-A CRISPR-associated RAMP protein Csm3	csm3gr7	cd09684	CAS-III-A,CAS-III-D			
31998..32403	-	RRD93725.1	RQYD01000028.1	Clostridiales bacterium COT073_COT-073	Bacteria	Firmicutes	Clostridia	Clostridiales			type III-A CRISPR-associated protein Csm2	csm2gr11	cd09647	CAS-III-A			
32421..34716	-	RRD93726.1	RQYD01000028.1	Clostridiales bacterium COT073_COT-073	Bacteria	Firmicutes	Clostridia	Clostridiales			type III-A CRISPR-associated protein Cas10/Csm1	cas10	cd09680	CAS-III			
34681..35461	-	RRD93727.1	RQYD01000028.1	Clostridiales bacterium COT073_COT-073	Bacteria	Firmicutes	Clostridia	Clostridiales			CRISPR-associated endoribonuclease Cas6	cas6	pfam10040	CAS-III,CAS-IV			
48 CAS-III-A | RGK40245.1																	
58604..59354	+	RGK40239.1	QSQN01000015.1	Ruminococcus lactaris	Bacteria	Firmicutes	Clostridia	Clostridiales	Ruminococcaceae	Ruminococcus	CRISPR-associated endoribonuclease Cas6	cas6	pfam10040	CAS-III,CAS-IV			
59350..61663	+	RGK40240.1	QSQN01000015.1	Ruminococcus lactaris	Bacteria	Firmicutes	Clostridia	Clostridiales	Ruminococcaceae	Ruminococcus	type III-A CRISPR-associated protein Cas10/Csm1	cas10	cd09680	CAS-III			
61662..62061	+	RGK40241.1	QSQN01000015.1	Ruminococcus lactaris	Bacteria	Firmicutes	Clostridia	Clostridiales	Ruminococcaceae	Ruminococcus	type III-A CRISPR-associated protein Csm2	csm2gr11	cd09647	CAS-III-A			
62097..62754	+	RGK40242.1	QSQN01000015.1	Ruminococcus lactaris	Bacteria	Firmicutes	Clostridia	Clostridiales	Ruminococcaceae	Ruminococcus	type III-A CRISPR-associated RAMP protein Csm3	csm3gr7	cd09684	CAS-III-A,CAS-III-D			
62766..63684	+	RGK40243.1	QSQN01000015.1	Ruminococcus lactaris	Bacteria	Firmicutes	Clostridia	Clostridiales	Ruminococcaceae	Ruminococcus	type III-A CRISPR-associated RAMP protein Csm4	csm4gr5	COG1567	CAS-III-A			
63680..64814	+	RGK40244.1	QSQN01000015.1	Ruminococcus lactaris	Bacteria	Firmicutes	Clostridia	Clostridiales	Ruminococcaceae	Ruminococcus	type III-A CRISPR-associated RAMP protein Csm5	csm5gr7,csm5gr7	cd09662,COG	CAS-III-A			
64997..66900	array		QSQN01000015.1	Ruminococcus lactaris	Bacteria	Firmicutes	Clostridia	Clostridiales	Ruminococcaceae	Ruminococcus		CRISPR array			27	35	36
66956..67964	+	RGK40245.1	QSQN01000015.1	Ruminococcus lactaris	Bacteria	Firmicutes	Clostridia	Clostridiales	Ruminococcaceae	Ruminococcus	CRISPR-associated endonuclease Cas1	cas1	cd09634	CAS-I,CAS-II,CAS-III,CAS-V			
67968..68283	+	RGK40246.1	QSQN01000015.1	Ruminococcus lactaris	Bacteria	Firmicutes	Clostridia	Clostridiales	Ruminococcaceae	Ruminococcus	CRISPR-associated endonuclease Cas2	cas2	cd09725	CAS-I,CAS-II,CAS-III,CAS-V			
68311..69694	+	RGK40247.1	QSQN01000015.1	Ruminococcus lactaris	Bacteria	Firmicutes	Clostridia	Clostridiales	Ruminococcaceae	Ruminococcus	hypothetical protein	csm6	pfam09659	CAS-III-A			
49 CAS-III-A | EFE91105.1																	
364979..366131	-	EFE91097.1	GG729935.1	Oribacterium sp. oral taxon 078 str. F0262	Bacteria	Firmicutes	Clostridia	Clostridiales	Lachnospiraceae	Oribacterium	CRISPR-associated RAMP protein, Csm5 family	csm5gr7	cd09662	CAS-III-A			
366127..367045	-	EFE91098.1	GG729935.1	Oribacterium sp. oral taxon 078 str. F0262	Bacteria	Firmicutes	Clostridia	Clostridiales	Lachnospiraceae	Oribacterium	CRISPR-associated RAMP protein, Csm4 family	csm4gr5	cd09663	CAS-III-A			
367041..367779	-	EFE91099.1	GG729935.1	Oribacterium sp. oral taxon 078 str. F0262	Bacteria	Firmicutes	Clostridia	Clostridiales	Lachnospiraceae	Oribacterium	CRISPR-associated RAMP protein, Csm3 family	csm3gr7	cd09684	CAS-III-A,CAS-III-D			
367794..368268	-	EFE91100.1	GG729935.1	Oribacterium sp. oral taxon 078 str. F0262	Bacteria	Firmicutes	Clostridia	Clostridiales	Lachnospiraceae	Oribacterium	CRISPR-associated protein, Csm2 family	csm2gr11	cd09647	CAS-III-A			
368245..370636	-	EFE91101.1	GG729935.1	Oribacterium sp. oral taxon 078 str. F0262	Bacteria	Firmicutes	Clostridia	Clostridiales	Lachnospiraceae	Oribacterium	CRISPR-associated protein, Csm1 family	cas10	cd09680	CAS-III			
370589..371378	-	EFE91102.1	GG729935.1	Oribacterium sp. oral taxon 078 str. F0262	Bacteria	Firmicutes	Clostridia	Clostridiales	Lachnospiraceae	Oribacterium	CRISPR-associated endoribonuclease Cas6	cas6	pfam10040	CAS-III,CAS-IV			
371536..372841	-	EFE91103.1	GG729935.1	Oribacterium sp. oral taxon 078 str. F0262	Bacteria	Firmicutes	Clostridia	Clostridiales	Lachnospiraceae	Oribacterium	putative CRISPR-associated protein, Csm6 family	csm6	pfam09659	CAS-III-A			
372912..373230	-	EFE91104.1	GG729935.1	Oribacterium sp. oral taxon 078 str. F0262	Bacteria	Firmicutes	Clostridia	Clostridiales	Lachnospiraceae	Oribacterium	CRISPR-associated protein Cas2	cas2	pfam09827	CAS-I,CAS-II,CAS-III			
373235..374234	-	EFE91105.1	GG729935.1	Oribacterium sp. oral taxon 078 str. F0262	Bacteria	Firmicutes	Clostridia	Clostridiales	Lachnospiraceae	Oribacterium	CRISPR-associated endonuclease Cas1	cas1	cd09634	CAS-I,CAS-II,CAS-III,CAS-V			
374505..377305	array		GG729935.1	Oribacterium sp. oral taxon 078 str. F0262	Bacteria	Firmicutes	Clostridia	Clostridiales	Lachnospiraceae	Oribacterium		CRISPR array			40	34	36
50 CAS-III-A | ERL20509.1																	
58653..59793	-	ERL20500.1	KI273116.1	Oribacterium sp. oral taxon 078 str. F0263	Bacteria	Firmicutes	Clostridia	Clostridiales	Lachnospiraceae	Oribacterium	CRISPR-associated RAMP protein, Csm5 family	csm5gr7	cd09662	CAS-III-A			
59789..60707	-	ERL20501.1	KI273116.1	Oribacterium sp. oral taxon 078 str. F0263	Bacteria	Firmicutes	Clostridia	Clostridiales	Lachnospiraceae	Oribacterium	CRISPR-associated RAMP protein, Csm4 family	csm4gr5	cd09663	CAS-III-A			
60675..60966	-	ERL20502.1	KI273116.1	Oribacterium sp. oral taxon 078 str. F0263	Bacteria	Firmicutes	Clostridia	Clostridiales	Lachnospiraceae	Oribacterium	hypothetical protein	csm3gr7	cd09684	CAS-III-A,CAS-III-D			
60910..61423	-	ERL20503.1	KI273116.1	Oribacterium sp. oral taxon 078 str. F0263	Bacteria	Firmicutes	Clostridia	Clostridiales	Lachnospiraceae	Oribacterium	CRISPR-associated RAMP protein, Csm3 family	csm3gr7	cd09684	CAS-III-A,CAS-III-D			
61432..61957	-	ERL20504.1	KI273116.1	Oribacterium sp. oral taxon 078 str. F0263	Bacteria	Firmicutes	Clostridia	Clostridiales	Lachnospiraceae	Oribacterium	CRISPR-associated protein, Csm2 family	csm2gr11	cd09647	CAS-III-A			
61934..64328	-	ERL20505.1	KI273116.1	Oribacterium sp. oral taxon 078 str. F0263	Bacteria	Firmicutes	Clostridia	Clostridiales	Lachnospiraceae	Oribacterium	CRISPR-associated protein, Csm1 family	cas10	cd09680	CAS-III			

64281..65070	-	ERL20506.1	KI273116.1	Oribacterium sp. oral taxon 078 str. F0263	Bacteria	Firmicutes	Clostridia	Clostridiales	Lachnospiraceae	Oribacterium	CRISPR-associated endoribonuclease Cas6	cas6	pfam10040	CAS-III,CAS-IV			
65228..66521	-	ERL20507.1	KI273116.1	Oribacterium sp. oral taxon 078 str. F0263	Bacteria	Firmicutes	Clostridia	Clostridiales	Lachnospiraceae	Oribacterium	CRISPR-associated protein, Csm6 family	csm6	cd09746	CAS-III-A			
66592..66919	-	ERL20508.1	KI273116.1	Oribacterium sp. oral taxon 078 str. F0263	Bacteria	Firmicutes	Clostridia	Clostridiales	Lachnospiraceae	Oribacterium	CRISPR-associated protein Cas2	cas2	pfam09827	CAS-I,CAS-II,CAS-III			
66915..67758	-	ERL20509.1	KI273116.1	Oribacterium sp. oral taxon 078 str. F0263	Bacteria	Firmicutes	Clostridia	Clostridiales	Lachnospiraceae	Oribacterium	CRISPR-associated endonuclease Cas1	**cas1**	cd09634	CAS-I,CAS-II,CAS-III,CAS-V			
67773..67914	-	ERL20510.1	KI273116.1	Oribacterium sp. oral taxon 078 str. F0263	Bacteria	Firmicutes	Clostridia	Clostridiales	Lachnospiraceae	Oribacterium	hypothetical protein	cas1*	pfam01867				
68184..68501	array		KI273116.1	Oribacterium sp. oral taxon 078 str. F0263	Bacteria	Firmicutes	Clostridia	Clostridiales	Lachnospiraceae	Oribacterium		CRISPR array			5	34	36
68593..68837	array		KI273116.1	Oribacterium sp. oral taxon 078 str. F0263	Bacteria	Firmicutes	Clostridia	Clostridiales	Lachnospiraceae	Oribacterium		CRISPR array			4	32	37
68927..69241	array		KI273116.1	Oribacterium sp. oral taxon 078 str. F0263	Bacteria	Firmicutes	Clostridia	Clostridiales	Lachnospiraceae	Oribacterium		CRISPR array			5	33	36
68989..69193	+	ERL20424.1	KI273116.1	Oribacterium sp. oral taxon 078 str. F0263	Bacteria	Firmicutes	Clostridia	Clostridiales	Lachnospiraceae	Oribacterium	hypothetical protein						
69332..69657	array		KI273116.1	Oribacterium sp. oral taxon 078 str. F0263	Bacteria	Firmicutes	Clostridia	Clostridiales	Lachnospiraceae	Oribacterium		CRISPR array			5	36	36
69753..69996	array		KI273116.1	Oribacterium sp. oral taxon 078 str. F0263	Bacteria	Firmicutes	Clostridia	Clostridiales	Lachnospiraceae	Oribacterium		CRISPR array			4	38	32
69855..69990	+	ERL20285.1	KI273116.1	Oribacterium sp. oral taxon 078 str. F0263	Bacteria	Firmicutes	Clostridia	Clostridiales	Lachnospiraceae	Oribacterium	hypothetical protein						
70085..70403	array		KI273116.1	Oribacterium sp. oral taxon 078 str. F0263	Bacteria	Firmicutes	Clostridia	Clostridiales	Lachnospiraceae	Oribacterium		CRISPR array			5	34	36
70090..70398	+	ERL20222.1	KI273116.1	Oribacterium sp. oral taxon 078 str. F0263	Bacteria	Firmicutes	Clostridia	Clostridiales	Lachnospiraceae	Oribacterium	hypothetical protein						
70493..70742	array		KI273116.1	Oribacterium sp. oral taxon 078 str. F0263	Bacteria	Firmicutes	Clostridia	Clostridiales	Lachnospiraceae	Oribacterium		CRISPR array			4	35	36
70603..70738	-	ERL20156.1	KI273116.1	Oribacterium sp. oral taxon 078 str. F0263	Bacteria	Firmicutes	Clostridia	Clostridiales	Lachnospiraceae	Oribacterium	hypothetical protein						
70831..71218	array		KI273116.1	Oribacterium sp. oral taxon 078 str. F0263	Bacteria	Firmicutes	Clostridia	Clostridiales	Lachnospiraceae	Oribacterium		CRISPR array			6	34	36
71074..71214	-	ERL20017.1	KI273116.1	Oribacterium sp. oral taxon 078 str. F0263	Bacteria	Firmicutes	Clostridia	Clostridiales	Lachnospiraceae	Oribacterium	hypothetical protein						
71310..71559	array		KI273116.1	Oribacterium sp. oral taxon 078 str. F0263	Bacteria	Firmicutes	Clostridia	Clostridiales	Lachnospiraceae	Oribacterium		CRISPR array			4	35	36
71651..71899	array		KI273116.1	Oribacterium sp. oral taxon 078 str. F0263	Bacteria	Firmicutes	Clostridia	Clostridiales	Lachnospiraceae	Oribacterium		CRISPR array			4	35	36
51 CAS-III-A | RGG19527.1																	
114279..115023	+	RGG19521.1	QTWD01000001.1	Blautia sp. AF26-2	Bacteria	Firmicutes	Clostridia	Clostridiales	Lachnospiraceae	Blautia	CRISPR-associated endoribonuclease Cas6	cas6	cd09652	CAS-I,CAS-III			
115019..117329	+	RGG19522.1	QTWD01000001.1	Blautia sp. AF26-2	Bacteria	Firmicutes	Clostridia	Clostridiales	Lachnospiraceae	Blautia	type III-A CRISPR-associated protein Cas10/Csm1	cas10	cd09680	CAS-III			
117325..117718	+	RGG19523.1	QTWD01000001.1	Blautia sp. AF26-2	Bacteria	Firmicutes	Clostridia	Clostridiales	Lachnospiraceae	Blautia	type III-A CRISPR-associated protein Csm2	csm2gr11	cd09647	CAS-III-A			
117730..118384	+	RGG19524.1	QTWD01000001.1	Blautia sp. AF26-2	Bacteria	Firmicutes	Clostridia	Clostridiales	Lachnospiraceae	Blautia	type III-A CRISPR-associated RAMP protein Csm3	csm3gr7	cd09684	CAS-III-A,CAS-III-D			
118394..119306	+	RGG19525.1	QTWD01000001.1	Blautia sp. AF26-2	Bacteria	Firmicutes	Clostridia	Clostridiales	Lachnospiraceae	Blautia	type III-A CRISPR-associated RAMP protein Csm4	csm4gr5	COG1567	CAS-III-A			
119308..120445	+	RGG19526.1	QTWD01000001.1	Blautia sp. AF26-2	Bacteria	Firmicutes	Clostridia	Clostridiales	Lachnospiraceae	Blautia	type III-A CRISPR-associated RAMP protein Csm5	csm5gr7	cd09662	CAS-III-A			
120586..121699	array		QTWD01000001.1	Blautia sp. AF26-2	Bacteria	Firmicutes	Clostridia	Clostridiales	Lachnospiraceae	Blautia		CRISPR array			16	35	36
121828..122833	+	RGG19527.1	QTWD01000001.1	Blautia sp. AF26-2	Bacteria	Firmicutes	Clostridia	Clostridiales	Lachnospiraceae	Blautia	CRISPR-associated endonuclease Cas1	**cas1**	cd09634	CAS-I,CAS-II,CAS-III,CAS-V			
122852..123170	+	RGG19528.1	QTWD01000001.1	Blautia sp. AF26-2	Bacteria	Firmicutes	Clostridia	Clostridiales	Lachnospiraceae	Blautia	CRISPR-associated endonuclease Cas2	cas2	pfam09827	CAS-I,CAS-II,CAS-III			
123182..124529	+	RGG19529.1	QTWD01000001.1	Blautia sp. AF26-2	Bacteria	Firmicutes	Clostridia	Clostridiales	Lachnospiraceae	Blautia	CRISPR-associated protein Csm6	csm6	pfam09659	CAS-III-A			
52 CAS-III-A | RHP10704.1																	
329478..330225	+	RHP11205.1	QUDV01000001.1	Dorea sp. AF36-15AT	Bacteria	Firmicutes	Clostridia	Clostridiales	Lachnospiraceae	Dorea	CRISPR-associated endoribonuclease Cas6	cas6	cd09652	CAS-I,CAS-III			
330221..332534	+	RHP10699.1	QUDV01000001.1	Dorea sp. AF36-15AT	Bacteria	Firmicutes	Clostridia	Clostridiales	Lachnospiraceae	Dorea	type III-A CRISPR-associated protein Cas10/Csm1	cas10	cd09680	CAS-III			
332533..332938	+	RHP10700.1	QUDV01000001.1	Dorea sp. AF36-15AT	Bacteria	Firmicutes	Clostridia	Clostridiales	Lachnospiraceae	Dorea	type III-A CRISPR-associated protein Csm2	csm2gr11	cd09647	CAS-III-A			
332953..333619	+	RHP10701.1	QUDV01000001.1	Dorea sp. AF36-15AT	Bacteria	Firmicutes	Clostridia	Clostridiales	Lachnospiraceae	Dorea	type III-A CRISPR-associated RAMP protein Csm3	csm3gr7	cd09684	CAS-III-A,CAS-III-D			
333630..334539	+	RHP10702.1	QUDV01000001.1	Dorea sp. AF36-15AT	Bacteria	Firmicutes	Clostridia	Clostridiales	Lachnospiraceae	Dorea	type III-A CRISPR-associated RAMP protein Csm4	csm4gr5	COG1567	CAS-III-A			
334541..335663	+	RHP10703.1	QUDV01000001.1	Dorea sp. AF36-15AT	Bacteria	Firmicutes	Clostridia	Clostridiales	Lachnospiraceae	Dorea	type III-A CRISPR-associated RAMP protein Csm5	csm5gr7	cd09662	CAS-III-A			
335800..337241	array		QUDV01000001.1	Dorea sp. AF36-15AT	Bacteria	Firmicutes	Clostridia	Clostridiales	Lachnospiraceae	Dorea		CRISPR array			21	35	35
337370..338375	+	RHP10704.1	QUDV01000001.1	Dorea sp. AF36-15AT	Bacteria	Firmicutes	Clostridia	Clostridiales	Lachnospiraceae	Dorea	CRISPR-associated endonuclease Cas1	**cas1**	cd09634	CAS-I,CAS-II,CAS-III,CAS-V			
338398..338716	+	RHP10705.1	QUDV01000001.1	Dorea sp. AF36-15AT	Bacteria	Firmicutes	Clostridia	Clostridiales	Lachnospiraceae	Dorea	CRISPR-associated endonuclease Cas2	cas2	cd09725	CAS-I,CAS-II,CAS-III,CAS-V			
338740..340078	+	RHP10706.1	QUDV01000001.1	Dorea sp. AF36-15AT	Bacteria	Firmicutes	Clostridia	Clostridiales	Lachnospiraceae	Dorea	CRISPR-associated protein Csm6	csm6	cd09746	CAS-III-A			
53 CAS-III-A | CDD66308.1																	
39983..41327	-	CDD66306.1	FR895390.1	Firmicutes bacterium CAG:882	Bacteria	Firmicutes					cRISPR-associated protein Csm6 family	csm6	cd09746	CAS-III-A			
41390..41705	-	CDD66307.1	FR895390.1	Firmicutes bacterium CAG:882	Bacteria	Firmicutes					cRISPR-associated endoribonuclease Cas2 2	cas2	pfam09827	CAS-I,CAS-II,CAS-III			
41764..42769	-	CDD66308.1	FR895390.1	Firmicutes bacterium CAG:882	Bacteria	Firmicutes					cRISPR-associated endonuclease Cas1 2	**cas1**	cd09634	CAS-I,CAS-II,CAS-III,CAS-V			
43002..44145	-	CDD66309.1	FR895390.1	Firmicutes bacterium CAG:882	Bacteria	Firmicutes					cRISPR-associated RAMP protein Csm5 family	csm5gr7	COG1332	CAS-III-A			
44182..45091	-	CDD66310.1	FR895390.1	Firmicutes bacterium CAG:882	Bacteria	Firmicutes					cRISPR-associated RAMP protein Csm4 family	csm4gr5	COG1567	CAS-III-A			
45163..45838	-	CDD66311.1	FR895390.1	Firmicutes bacterium CAG:882	Bacteria	Firmicutes					cRISPR-associated protein Csm3 family	csm3gr7	cd09684	CAS-III-A,CAS-III-D			
45849..46248	-	CDD66312.1	FR895390.1	Firmicutes bacterium CAG:882	Bacteria	Firmicutes					cRISPR-associated protein Csm2 family	csm2gr11	cd09647	CAS-III-A			
46244..48581	-	CDD66313.1	FR895390.1	Firmicutes bacterium CAG:882	Bacteria	Firmicutes					cRISPR-associated protein Csm1 family	cas10	cd09680	CAS-III			
48577..49327	-	CDD66314.1	FR895390.1	Firmicutes bacterium CAG:882	Bacteria	Firmicutes					cRISPR-associated protein Cas6	cas6	pfam10040	CAS-III,CAS-IV			
54 CAS-III-A | CUN22620.1																	
28298..29042	+	CUN22556.1	CYYA01000022.1	Eubacterium ramulus	Bacteria	Firmicutes	Clostridia	Clostridiales	Eubacteriaceae	Eubacterium	CRISPR-associated endoribonuclease Cas6	cas6	COG5551	CAS-III			
29038..31393	+	CUN22566.1	CYYA01000022.1	Eubacterium ramulus	Bacteria	Firmicutes	Clostridia	Clostridiales	Eubacteriaceae	Eubacterium	Predicted HD superfamily hydrolase	cas10	cd09680	CAS-III			
31389..31782	+	CUN22575.1	CYYA01000022.1	Eubacterium ramulus	Bacteria	Firmicutes	Clostridia	Clostridiales	Eubacteriaceae	Eubacterium	CRISPR type III-A/MTUBE-associated protein Csm2	csm2gr11	cd09647	CAS-III-A			
31797..32466	+	CUN22584.1	CYYA01000022.1	Eubacterium ramulus	Bacteria	Firmicutes	Clostridia	Clostridiales	Eubacteriaceae	Eubacterium	CRISPR type III-A/MTUBE-associated RAMP protein Csm3	csm3gr7	cd09684	CAS-III-A,CAS-III-D			
32484..33393	+	CUN22593.1	CYYA01000022.1	Eubacterium ramulus	Bacteria	Firmicutes	Clostridia	Clostridiales	Eubacteriaceae	Eubacterium	CRISPR type III-A/MTUBE-associated RAMP protein Csm4	csm4gr5	COG1567	CAS-III-A			
33396..34521	+	CUN22602.1	CYYA01000022.1	Eubacterium ramulus	Bacteria	Firmicutes	Clostridia	Clostridiales	Eubacteriaceae	Eubacterium	CRISPR type III-A/MTUBE-associated RAMP protein Csm5	csm5gr7,csm5gr7	cd09662,cd0(	CAS-III-A			
34657..36490	array		CYYA01000022.1	Eubacterium ramulus	Bacteria	Firmicutes	Clostridia	Clostridiales	Eubacteriaceae	Eubacterium		CRISPR array			26	35	36
35075..35555	-	CUN22611.1	CYYA01000022.1	Eubacterium ramulus	Bacteria	Firmicutes	Clostridia	Clostridiales	Eubacteriaceae	Eubacterium	Uncharacterised protein						
36522..37527	+	CUN22620.1	CYYA01000022.1	Eubacterium ramulus	Bacteria	Firmicutes	Clostridia	Clostridiales	Eubacteriaceae	Eubacterium	CRISPR-associated endonuclease Cas1	**cas1**	cd09634	CAS-I,CAS-II,CAS-III,CAS-V			
37553..37871	+	CUN22631.1	CYYA01000022.1	Eubacterium ramulus	Bacteria	Firmicutes	Clostridia	Clostridiales	Eubacteriaceae	Eubacterium	CRISPR-associated endoribonuclease Cas2	cas2	cd09725	CAS-I,CAS-II,CAS-III,CAS-V			
37889..39215	+	CUN22640.1	CYYA01000022.1	Eubacterium ramulus	Bacteria	Firmicutes	Clostridia	Clostridiales	Eubacteriaceae	Eubacterium	CRISPR type III-A/MTUBE-associated protein Csm6	csm6	pfam09659	CAS-III-A			
55 CAS-III-A | CDE69450.1																	
49976..51305	-	CDE69448.1	FR900940.1	Clostridium sp. CAG:277	Bacteria	Firmicutes	Clostridia	Clostridiales	Clostridiaceae	Clostridium	cRISPR-associated protein Csm6 family	csm6	cd09746	CAS-III-A			
51320..51638	-	CDE69449.1	FR900940.1	Clostridium sp. CAG:277	Bacteria	Firmicutes	Clostridia	Clostridiales	Clostridiaceae	Clostridium	cRISPR-associated endoribonuclease Cas2 2	cas2	cd09725	CAS-I,CAS-II,CAS-III,CAS-V			
51654..52659	-	CDE69450.1	FR900940.1	Clostridium sp. CAG:277	Bacteria	Firmicutes	Clostridia	Clostridiales	Clostridiaceae	Clostridium	cRISPR-associated endonuclease Cas1 2	**cas1**	cd09634	CAS-I,CAS-II,CAS-III,CAS-V			
52709..53027	array		FR900940.1	Clostridium sp. CAG:277	Bacteria	Firmicutes	Clostridia	Clostridiales	Clostridiaceae	Clostridium		CRISPR array			5	36	35
53230..54349	-	CDE69451.1	FR900940.1	Clostridium sp. CAG:277	Bacteria	Firmicutes	Clostridia	Clostridiales	Clostridiaceae	Clostridium	cRISPR-associated RAMP protein Csm5 family	csm5gr7,csm5gr7	cd09662,COG	CAS-III-A			
54351..55260	-	CDE69452.1	FR900940.1	Clostridium sp. CAG:277	Bacteria	Firmicutes	Clostridia	Clostridiales	Clostridiaceae	Clostridium	cRISPR-associated RAMP protein Csm4 family	csm4gr5	COG1567	CAS-III-A			
55271..55928	-	CDE69453.1	FR900940.1	Clostridium sp. CAG:277	Bacteria	Firmicutes	Clostridia	Clostridiales	Clostridiaceae	Clostridium	cRISPR-associated RAMP protein Csm3 family	csm3gr7	cd09684	CAS-III-A,CAS-III-D			
55942..56335	-	CDE69454.1	FR900940.1	Clostridium sp. CAG:277	Bacteria	Firmicutes	Clostridia	Clostridiales	Clostridiaceae	Clostridium	cRISPR-associated protein Csm2 family	csm2gr11	pfam03750	CAS-III-A			
56335..58642	-	CDE69455.1	FR900940.1	Clostridium sp. CAG:277	Bacteria	Firmicutes	Clostridia	Clostridiales	Clostridiaceae	Clostridium	cRISPR-associated protein Csm1 family	cas10	cd09680	CAS-III			
58638..59385	-	CDE69456.1	FR900940.1	Clostridium sp. CAG:277	Bacteria	Firmicutes	Clostridia	Clostridiales	Clostridiaceae	Clostridium	cRISPR-associated protein Cas6	cas6	pfam10040	CAS-III,CAS-IV			
56 partial CAS-III-A | RHP82145.1																	
29709..31038	-	RHP82149.1	QUET01000032.1	Roseburia sp. AM59-24XD	Bacteria	Firmicutes	Clostridia	Clostridiales	Lachnospiraceae	Roseburia	CRISPR-associated protein Csm6	csm6	pfam09659	CAS-III-A			
31054..31372	-	RHP82144.1	QUET01000032.1	Roseburia sp. AM59-24XD	Bacteria	Firmicutes	Clostridia	Clostridiales	Lachnospiraceae	Roseburia	CRISPR-associated endonuclease Cas2	cas2	pfam09827	CAS-I,CAS-II,CAS-III			
31384..32389	-	RHP82145.1	QUET01000032.1	Roseburia sp. AM59-24XD	Bacteria	Firmicutes	Clostridia	Clostridiales	Lachnospiraceae	Roseburia	CRISPR-associated endonuclease Cas1	**cas1**	cd09634	CAS-I,CAS-II,CAS-III,CAS-V			
32439..33536	array		QUET01000032.1	Roseburia sp. AM59-24XD	Bacteria	Firmicutes	Clostridia	Clostridiales	Lachnospiraceae	Roseburia		CRISPR array			16	39	32
57 CAS-III-A | CUO30722.1																	
2502..3828	-	CUO30670.1	CYYY01000022.1	Dorea longicatena	Bacteria	Firmicutes	Clostridia	Clostridiales	Lachnospiraceae	Dorea	CRISPR type III-A/MTUBE-associated protein Csm6	csm6	cd09746	CAS-III-A			
3846..4164	-	CUO30698.1	CYYY01000022.1	Dorea longicatena	Bacteria	Firmicutes	Clostridia	Clostridiales	Lachnospiraceae	Dorea	CRISPR-associated endoribonuclease Cas2	cas2	pfam09827	CAS-I,CAS-II,CAS-III			
4200..5205	-	CUO30722.1	CYYY01000022.1	Dorea longicatena	Bacteria	Firmicutes	Clostridia	Clostridiales	Lachnospiraceae	Dorea	CRISPR-associated endonuclease Cas1	**cas1**	cd09634	CAS-I,CAS-II,CAS-III,CAS-V			
5307..7261	array		CYYY01000022.1	Dorea longicatena	Bacteria	Firmicutes	Clostridia	Clostridiales	Lachnospiraceae	Dorea		CRISPR array			28	35	36
7398..8520	-	CUO30749.1	CYYY01000022.1	Dorea longicatena	Bacteria	Firmicutes	Clostridia	Clostridiales	Lachnospiraceae	Dorea	CRISPR type III-A/MTUBE-associated RAMP protein Csm5	csm5gr7	cd09662	CAS-III-A			
8522..9431	-	CUO30768.1	CYYY01000022.1	Dorea longicatena	Bacteria	Firmicutes	Clostridia	Clostridiales	Lachnospiraceae	Dorea	CRISPR type III-A/MTUBE-associated RAMP protein Csm4	csm4gr5	COG1567	CAS-III-A			
9442..10099	-	CUO30796.1	CYYY01000022.1	Dorea longicatena	Bacteria	Firmicutes	Clostridia	Clostridiales	Lachnospiraceae	Dorea	CRISPR type III-A/MTUBE-associated RAMP protein Csm3	csm3gr7	cd09684	CAS-III-A,CAS-III-D			
10113..10506	-	CUO30826.1	CYYY01000022.1	Dorea longicatena	Bacteria	Firmicutes	Clostridia	Clostridiales	Lachnospiraceae	Dorea	CRISPR type III-A/MTUBE-associated protein Csm2	csm2gr11	cd09647	CAS-III-A			
10486..12790	-	CUO30856.1	CYYY01000022.1	Dorea longicatena	Bacteria	Firmicutes	Clostridia	Clostridiales	Lachnospiraceae	Dorea	CRISPR-associated protein Cas10/Csm1%2C subtype III-A/MTUBE	cas10	cd09680	CAS-III			
12783..13533	-	CUO30879.1	CYYY01000022.1	Dorea longicatena	Bacteria	Firmicutes	Clostridia	Clostridiales	Lachnospiraceae	Dorea	CRISPR-associated endoribonuclease Cas6	cas6	pfam10040	CAS-III,CAS-IV			
13723..14002	+	CUO30905.1	CYYY01000022.1	Dorea longicatena	Bacteria	Firmicutes	Clostridia	Clostridiales	Lachnospiraceae	Dorea	Uncharacterised protein family (UPF0150)						
14141..14348	+	CUO30940.1	CYYY01000022.1	Dorea longicatena	Bacteria	Firmicutes	Clostridia	Clostridiales	Lachnospiraceae	Dorea	SSP-3						
14529..16563	-	CUO30963.1	CYYY01000022.1	Dorea longicatena	Bacteria	Firmicutes	Clostridia	Clostridiales	Lachnospiraceae	Dorea	ATP-dependent DNA helicase recG	cas3	cd09639	CAS-I			
58 CAS-III-A| EOS81372.1																	
234272..235004	+	EOS81499.1	KE159726.1	Dorea sp. 5-2	Bacteria	Firmicutes	Clostridia	Clostridiales	Lachnospiraceae	Dorea	CRISPR-associated endoribonuclease cas6	cas6	COG5551	CAS-III			
235051..237364	+	EOS81500.1	KE159726.1	Dorea sp. 5-2	Bacteria	Firmicutes	Clostridia	Clostridiales	Lachnospiraceae	Dorea	CRISPR-associated protein cas10/csm1, subtype III-a/mtube	cas10	cd09680	CAS-III			
237360..237753	+	EOS81501.1	KE159726.1	Dorea sp. 5-2	Bacteria	Firmicutes	Clostridia	Clostridiales	Lachnospiraceae	Dorea	CRISPR type III-a/mtube-associated protein csm2	csm2gr11	cd09647	CAS-III-A			
237765..238422	+	EOS81502.1	KE159726.1	Dorea sp. 5-2	Bacteria	Firmicutes	Clostridia	Clostridiales	Lachnospiraceae	Dorea	CRISPR type III-a/mtube-associated ramp protein csm3	csm3gr7	cd09684	CAS-III-A,CAS-III-D			
238425..239343	+	EOS81503.1	KE159726.1	Dorea sp. 5-2	Bacteria	Firmicutes	Clostridia	Clostridiales	Lachnospiraceae	Dorea	CRISPR type III-a/mtube-associated ramp protein csm4	csm4gr5	COG1567	CAS-III-A			
239346..240471	+	EOS81504.1	KE159726.1	Dorea sp. 5-2	Bacteria	Firmicutes	Clostridia	Clostridiales	Lachnospiraceae	Dorea	CRISPR type III-a/mtube-associated ramp protein csm5	csm5gr7,csm5gr7	cd09662,cd0(	CAS-III-A			
240638..241035	array		KE159726.1	Dorea sp. 5-2	Bacteria	Firmicutes	Clostridia	Clostridiales	Lachnospiraceae	Dorea		CRISPR array			6	36	36
241411..242419	+	EOS81372.1	KE159726.1	Dorea sp. 5-2	Bacteria	Firmicutes	Clostridia	Clostridiales	Lachnospiraceae	Dorea	CRISPR-associated endonuclease cas1	**cas1**	cd09634	CAS-I,CAS-II,CAS-III,CAS-V			
242423..242738	+	EOS81373.1	KE159726.1	Dorea sp. 5-2	Bacteria	Firmicutes	Clostridia	Clostridiales	Lachnospiraceae	Dorea	CRISPR-associated endoribonuclease cas2	cas2	pfam09827	CAS-I,CAS-II,CAS-III			
242749..244120	+	EOS81374.1	KE159726.1	Dorea sp. 5-2	Bacteria	Firmicutes	Clostridia	Clostridiales	Lachnospiraceae	Dorea	CRISPR type III-a/mtube-associated protein csm6	csm6	cd09746	CAS-III-A			
60 CAS-III-A | RHA66006.1																	
26194..27433	-	RHA66003.1	QSFP01000014.1	Roseburia intestinalis	Bacteria	Firmicutes	Clostridia	Clostridiales	Lachnospiraceae	Roseburia	ATP-binding protein	Cas_RecF	cluster17				
27538..28894	-	RHA66004.1	QSFP01000014.1	Roseburia intestinalis	Bacteria	Firmicutes	Clostridia	Clostridiales	Lachnospiraceae	Roseburia	CRISPR-associated protein Csm6	csm6	cd09746	CAS-III-A			
28950..29268	array		QSFP01000014.1	Roseburia intestinalis	Bacteria	Firmicutes	Clostridia	Clostridiales	Lachnospiraceae	Roseburia		CRISPR array			5	34	36
29571..29910	-	RHA66005.1	QSFP01000014.1	Roseburia intestinalis	Bacteria	Firmicutes	Clostridia	Clostridiales	Lachnospiraceae	Roseburia	CRISPR-associated endonuclease Cas2	cas2	cd09725	CAS-I,CAS-II,CAS-III,CAS-V			

29922..30927	-	RHA66006.1	QSFP01000014.1	Roseburia intestinalis	Bacteria	Firmicutes	Clostridia	Clostridiales	Lachnospiraceae	Roseburia	CRISPR-associated endonuclease Cas1	cas1	cd09634	CAS-I,CAS-II,CAS-III,CAS-V			
31015..31766	array		QSFP01000014.1	Roseburia intestinalis	Bacteria	Firmicutes	Clostridia	Clostridiales	Lachnospiraceae	Roseburia		CRISPR array			11	35	36
31904..33026	-	RHA66007.1	QSFP01000014.1	Roseburia intestinalis	Bacteria	Firmicutes	Clostridia	Clostridiales	Lachnospiraceae	Roseburia	type III-A CRISPR-associated RAMP protein Csm5	csm5gr7,csm5gr7	cd09662,COG	CAS-III-A			
33022..33949	-	RHA66078.1	QSFP01000014.1	Roseburia intestinalis	Bacteria	Firmicutes	Clostridia	Clostridiales	Lachnospiraceae	Roseburia	type III-A CRISPR-associated RAMP protein Csm4	csm4gr5	COG1567	CAS-III-A			
33992..34655	-	RHA66008.1	QSFP01000014.1	Roseburia intestinalis	Bacteria	Firmicutes	Clostridia	Clostridiales	Lachnospiraceae	Roseburia	type III-A CRISPR-associated RAMP protein Csm3	csm3gr7	cd09684	CAS-III-A,CAS-III-D			
34670..35063	-	RHA66009.1	QSFP01000014.1	Roseburia intestinalis	Bacteria	Firmicutes	Clostridia	Clostridiales	Lachnospiraceae	Roseburia	type III-A CRISPR-associated protein Csm2	csm2gr11	cd09647	CAS-III-A			
35063..37373	-	RHA66010.1	QSFP01000014.1	Roseburia intestinalis	Bacteria	Firmicutes	Clostridia	Clostridiales	Lachnospiraceae	Roseburia	type III-A CRISPR-associated protein Cas10/Csm1	cas10	cd09680	CAS-III			
37369..38116	-	RHA66011.1	QSFP01000014.1	Roseburia intestinalis	Bacteria	Firmicutes	Clostridia	Clostridiales	Lachnospiraceae	Roseburia	CRISPR-associated endoribonuclease Cas6	cas6	pfam10040	CAS-III,CAS-IV			
61 CAS-III-A | RHM06245.1																	
112438..113794	-	RHM06243.1	QRPI01000004.1	Roseburia intestinalis	Bacteria	Firmicutes	Clostridia	Clostridiales	Lachnospiraceae	Roseburia	CRISPR-associated protein Csm6	csm6	cd09746	CAS-III-A			
114400..114739	-	RHM06244.1	QRPI01000004.1	Roseburia intestinalis	Bacteria	Firmicutes	Clostridia	Clostridiales	Lachnospiraceae	Roseburia	CRISPR-associated endonuclease Cas2	cas2	cd09725	CAS-I,CAS-II,CAS-III,CAS-V			
114751..115756	-	RHM06245.1	QRPI01000004.1	Roseburia intestinalis	Bacteria	Firmicutes	Clostridia	Clostridiales	Lachnospiraceae	Roseburia	CRISPR-associated endonuclease Cas1	cas1	cd09634	CAS-I,CAS-II,CAS-III,CAS-V			
115843..116960	array		QRPI01000004.1	Roseburia intestinalis	Bacteria	Firmicutes	Clostridia	Clostridiales	Lachnospiraceae	Roseburia		CRISPR array			16	36	36
117098..118220	-	RHM06246.1	QRPI01000004.1	Roseburia intestinalis	Bacteria	Firmicutes	Clostridia	Clostridiales	Lachnospiraceae	Roseburia	type III-A CRISPR-associated RAMP protein Csm5	csm5gr7,csm5gr7	cd09662,COG	CAS-III-A			
118216..119143	-	RHM06430.1	QRPI01000004.1	Roseburia intestinalis	Bacteria	Firmicutes	Clostridia	Clostridiales	Lachnospiraceae	Roseburia	type III-A CRISPR-associated RAMP protein Csm4	csm4gr5	COG1567	CAS-III-A			
119186..119849	-	RHM06247.1	QRPI01000004.1	Roseburia intestinalis	Bacteria	Firmicutes	Clostridia	Clostridiales	Lachnospiraceae	Roseburia	type III-A CRISPR-associated RAMP protein Csm3	csm3gr7	cd09684	CAS-III-A,CAS-III-D			
119864..120257	-	RHM06248.1	QRPI01000004.1	Roseburia intestinalis	Bacteria	Firmicutes	Clostridia	Clostridiales	Lachnospiraceae	Roseburia	type III-A CRISPR-associated protein Csm2	csm2gr11	cd09647	CAS-III-A			
120257..122567	-	RHM06249.1	QRPI01000004.1	Roseburia intestinalis	Bacteria	Firmicutes	Clostridia	Clostridiales	Lachnospiraceae	Roseburia	type III-A CRISPR-associated protein Cas10/Csm1	cas10	cd09680	CAS-III			
122563..123310	-	RHM06250.1	QRPI01000004.1	Roseburia intestinalis	Bacteria	Firmicutes	Clostridia	Clostridiales	Lachnospiraceae	Roseburia	CRISPR-associated endoribonuclease Cas6	cas6	pfam10040	CAS-III,CAS-IV			
62 CAS-III-A | OLA55795.1																	
92975..94214	-	OLA55793.1	MNTJ01000057.1	Roseburia intestinalis	Bacteria	Firmicutes	Clostridia	Clostridiales	Lachnospiraceae	Roseburia	hypothetical protein	Cas_RecF	cluster17				
96339..96660	-	OLA55794.1	MNTJ01000057.1	Roseburia intestinalis	Bacteria	Firmicutes	Clostridia	Clostridiales	Lachnospiraceae	Roseburia	CRISPR-associated endonuclease Cas2	cas2	cd09725	CAS-I,CAS-II,CAS-III,CAS-V			
96690..97695	-	OLA55795.1	MNTJ01000057.1	Roseburia intestinalis	Bacteria	Firmicutes	Clostridia	Clostridiales	Lachnospiraceae	Roseburia	CRISPR-associated endonuclease Cas1	cas1	cd09634	CAS-I,CAS-II,CAS-III,CAS-V			
98098..99220	-	OLA55796.1	MNTJ01000057.1	Roseburia intestinalis	Bacteria	Firmicutes	Clostridia	Clostridiales	Lachnospiraceae	Roseburia	type III-A CRISPR-associated RAMP protein Csm5	csm5gr7,csm5gr7	cd09662,COG	CAS-III-A			
99216..100131	-	OLA55811.1	MNTJ01000057.1	Roseburia intestinalis	Bacteria	Firmicutes	Clostridia	Clostridiales	Lachnospiraceae	Roseburia	type III-A CRISPR-associated RAMP protein Csm4	csm4gr5	COG1567	CAS-III-A			
100186..100849	-	OLA55797.1	MNTJ01000057.1	Roseburia intestinalis	Bacteria	Firmicutes	Clostridia	Clostridiales	Lachnospiraceae	Roseburia	type III-A CRISPR-associated RAMP protein Csm3	csm3gr7	cd09684	CAS-III-A,CAS-III-D			
100864..101257	-	OLA55798.1	MNTJ01000057.1	Roseburia intestinalis	Bacteria	Firmicutes	Clostridia	Clostridiales	Lachnospiraceae	Roseburia	type III-A CRISPR-associated protein Csm2	csm2gr11	cd09647	CAS-III-A			
101257..103567	-	OLA55799.1	MNTJ01000057.1	Roseburia intestinalis	Bacteria	Firmicutes	Clostridia	Clostridiales	Lachnospiraceae	Roseburia	type III-A CRISPR-associated protein Cas10/Csm1	cas10	cd09680	CAS-III			
103563..104310	-	OLA55800.1	MNTJ01000057.1	Roseburia intestinalis	Bacteria	Firmicutes	Clostridia	Clostridiales	Lachnospiraceae	Roseburia	CRISPR-associated endoribonuclease Cas6	cas6	pfam10040	CAS-III,CAS-IV			
63 CAS-III-A | RHF95484.1																	
105304..106660	-	RHF95481.1	QRHS01000006.1	Roseburia sp. AM23-20	Bacteria	Firmicutes	Clostridia	Clostridiales	Lachnospiraceae	Roseburia	CRISPR-associated protein Csm6	csm6	pfam09659	CAS-III-A			
106984..107185	-	RHF95482.1	QRHS01000006.1	Roseburia sp. AM23-20	Bacteria	Firmicutes	Clostridia	Clostridiales	Lachnospiraceae	Roseburia	hypothetical protein						
107336..107675	-	RHF95483.1	QRHS01000006.1	Roseburia sp. AM23-20	Bacteria	Firmicutes	Clostridia	Clostridiales	Lachnospiraceae	Roseburia	CRISPR-associated endonuclease Cas2	cas2	cd09725	CAS-I,CAS-II,CAS-III,CAS-V			
107687..108692	-	RHF95484.1	QRHS01000006.1	Roseburia sp. AM23-20	Bacteria	Firmicutes	Clostridia	Clostridiales	Lachnospiraceae	Roseburia	CRISPR-associated endonuclease Cas1	cas1	cd09634	CAS-I,CAS-II,CAS-III,CAS-V			
108779..109175	array		QRHS01000006.1	Roseburia sp. AM23-20	Bacteria	Firmicutes	Clostridia	Clostridiales	Lachnospiraceae	Roseburia		CRISPR array			6	36	36
109313..110435	-	RHF95485.1	QRHS01000006.1	Roseburia sp. AM23-20	Bacteria	Firmicutes	Clostridia	Clostridiales	Lachnospiraceae	Roseburia	type III-A CRISPR-associated RAMP protein Csm5	csm5gr7,csm5gr7	cd09662,COG	CAS-III-A			
110431..111358	-	RHF95529.1	QRHS01000006.1	Roseburia sp. AM23-20	Bacteria	Firmicutes	Clostridia	Clostridiales	Lachnospiraceae	Roseburia	type III-A CRISPR-associated RAMP protein Csm4	csm4gr5	COG1567	CAS-III-A			
111401..112064	-	RHF95486.1	QRHS01000006.1	Roseburia sp. AM23-20	Bacteria	Firmicutes	Clostridia	Clostridiales	Lachnospiraceae	Roseburia	type III-A CRISPR-associated RAMP protein Csm3	csm3gr7	cd09684	CAS-III-A,CAS-III-D			
112079..112472	-	RHF95487.1	QRHS01000006.1	Roseburia sp. AM23-20	Bacteria	Firmicutes	Clostridia	Clostridiales	Lachnospiraceae	Roseburia	type III-A CRISPR-associated protein Csm2	csm2gr11	cd09647	CAS-III-A			
112472..114782	-	RHF95488.1	QRHS01000006.1	Roseburia sp. AM23-20	Bacteria	Firmicutes	Clostridia	Clostridiales	Lachnospiraceae	Roseburia	type III-A CRISPR-associated protein Cas10/Csm1	cas10	cd09680	CAS-III			
114778..115525	-	RHF95489.1	QRHS01000006.1	Roseburia sp. AM23-20	Bacteria	Firmicutes	Clostridia	Clostridiales	Lachnospiraceae	Roseburia	CRISPR-associated endoribonuclease Cas6	cas6	pfam10040	CAS-III,CAS-IV			
64 CAS-III-A | EEV02630.1																	
109995..111351	-	EEV02627.1	GG692714.1	Roseburia intestinalis L1-82	Bacteria	Firmicutes	Clostridia	Clostridiales	Lachnospiraceae	Roseburia	putative CRISPR-associated protein, Csm6 family	csm6	cd09746	CAS-III-A			
111408..112295	array		GG692714.1	Roseburia intestinalis L1-82	Bacteria	Firmicutes	Clostridia	Clostridiales	Lachnospiraceae	Roseburia		CRISPR array			13	36	35
112303..112501	-	EEV02628.1	GG692714.1	Roseburia intestinalis L1-82	Bacteria	Firmicutes	Clostridia	Clostridiales	Lachnospiraceae	Roseburia	hypothetical protein						
112599..112938	-	EEV02629.1	GG692714.1	Roseburia intestinalis L1-82	Bacteria	Firmicutes	Clostridia	Clostridiales	Lachnospiraceae	Roseburia	CRISPR-associated protein Cas2	cas2	cd09725	CAS-I,CAS-II,CAS-III,CAS-V			
112950..113955	-	EEV02630.1	GG692714.1	Roseburia intestinalis L1-82	Bacteria	Firmicutes	Clostridia	Clostridiales	Lachnospiraceae	Roseburia	CRISPR-associated endonuclease Cas1	cas1	cd09634	CAS-I,CAS-II,CAS-III,CAS-V			
114042..114708	array		GG692714.1	Roseburia intestinalis L1-82	Bacteria	Firmicutes	Clostridia	Clostridiales	Lachnospiraceae	Roseburia		CRISPR array			10	46	25
114727..114871	-	EEV02631.1	GG692714.1	Roseburia intestinalis L1-82	Bacteria	Firmicutes	Clostridia	Clostridiales	Lachnospiraceae	Roseburia	hypothetical protein						
114857..115979	-	EEV02632.1	GG692714.1	Roseburia intestinalis L1-82	Bacteria	Firmicutes	Clostridia	Clostridiales	Lachnospiraceae	Roseburia	CRISPR-associated RAMP protein, Csm5 family	csm5gr7,csm5gr7	cd09662,COG	CAS-III-A			
115975..116902	-	EEV02633.1	GG692714.1	Roseburia intestinalis L1-82	Bacteria	Firmicutes	Clostridia	Clostridiales	Lachnospiraceae	Roseburia	CRISPR-associated RAMP protein, Csm4 family	csm4gr5	COG1567	CAS-III-A			
116945..117608	-	EEV02634.1	GG692714.1	Roseburia intestinalis L1-82	Bacteria	Firmicutes	Clostridia	Clostridiales	Lachnospiraceae	Roseburia	CRISPR-associated RAMP protein, Csm3 family	csm3gr7	cd09684	CAS-III-A,CAS-III-D			
117623..118016	-	EEV02635.1	GG692714.1	Roseburia intestinalis L1-82	Bacteria	Firmicutes	Clostridia	Clostridiales	Lachnospiraceae	Roseburia	CRISPR-associated protein, Csm2 family	csm2gr11	cd09647	CAS-III-A			
118016..120326	-	EEV02636.1	GG692714.1	Roseburia intestinalis L1-82	Bacteria	Firmicutes	Clostridia	Clostridiales	Lachnospiraceae	Roseburia	CRISPR-associated protein, Csm1 family	cas10	cd09680	CAS-III			
120322..121069	-	EEV02637.1	GG692714.1	Roseburia intestinalis L1-82	Bacteria	Firmicutes	Clostridia	Clostridiales	Lachnospiraceae	Roseburia	CRISPR-associated endoribonuclease Cas6	cas6	pfam10040	CAS-III,CAS-IV			
65 CAS-III-A | VCV21263.1																	
1216580..1217327	+	VCV21256.1	LR027880.1	Roseburia intestinalis L1-82	Bacteria	Firmicutes	Clostridia	Clostridiales	Lachnospiraceae	Roseburia	CRISPR-associated endoribonuclease Cas6	cas6	pfam10040	CAS-III,CAS-IV			
1217323..1219633	+	VCV21257.1	LR027880.1	Roseburia intestinalis L1-82	Bacteria	Firmicutes	Clostridia	Clostridiales	Lachnospiraceae	Roseburia	CRISPR-associated protein Cas10/Csm1	cas10	cd09680	CAS-III			
1219633..1220026	+	VCV21258.1	LR027880.1	Roseburia intestinalis L1-82	Bacteria	Firmicutes	Clostridia	Clostridiales	Lachnospiraceae	Roseburia	CRISPR type III-associated protein Csm2	csm2gr11	cd09647	CAS-III-A			
1220041..1220704	+	VCV21259.1	LR027880.1	Roseburia intestinalis L1-82	Bacteria	Firmicutes	Clostridia	Clostridiales	Lachnospiraceae	Roseburia	CRISPR type III-associated RAMP protein Csm3	csm3gr7	cd09684	CAS-III-A,CAS-III-D			
1220759..1221674	+	VCV21260.1	LR027880.1	Roseburia intestinalis L1-82	Bacteria	Firmicutes	Clostridia	Clostridiales	Lachnospiraceae	Roseburia	CRISPR type III-associated RAMP protein Csm4	csm4gr5	COG1567	CAS-III-A			
1221670..1222792	+	VCV21261.1	LR027880.1	Roseburia intestinalis L1-82	Bacteria	Firmicutes	Clostridia	Clostridiales	Lachnospiraceae	Roseburia	hypothetical protein	csm5gr7,csm5gr7	cd09662,COG	CAS-III-A			
1222778..1222922	+	VCV21262.1	LR027880.1	Roseburia intestinalis L1-82	Bacteria	Firmicutes	Clostridia	Clostridiales	Lachnospiraceae	Roseburia	hypothetical protein						
1222942..1223608	array		LR027880.1	Roseburia intestinalis L1-82	Bacteria	Firmicutes	Clostridia	Clostridiales	Lachnospiraceae	Roseburia		CRISPR array			10	46	25
1223694..1224699	+	VCV21263.1	LR027880.1	Roseburia intestinalis L1-82	Bacteria	Firmicutes	Clostridia	Clostridiales	Lachnospiraceae	Roseburia	CRISPR-associated endonuclease Cas1	cas1	cd09634	CAS-I,CAS-II,CAS-III,CAS-V			
1224729..1225050	+	VCV21264.1	LR027880.1	Roseburia intestinalis L1-82	Bacteria	Firmicutes	Clostridia	Clostridiales	Lachnospiraceae	Roseburia	CRISPR-associated endonuclease Cas2	cas2	cd09725	CAS-I,CAS-II,CAS-III,CAS-V			
1225369..1226242	array		LR027880.1	Roseburia intestinalis L1-82	Bacteria	Firmicutes	Clostridia	Clostridiales	Lachnospiraceae	Roseburia		CRISPR array			13	50	21
1226310..1227654	+	VCV21265.1	LR027880.1	Roseburia intestinalis L1-82	Bacteria	Firmicutes	Clostridia	Clostridiales	Lachnospiraceae	Roseburia	CRISPR-associated protein Csm6	csm6	cd09746	CAS-III-A			
66 CAS-III-A | RHG29044.1																	
139700..141056	-	RHG29042.1	QRID01000006.1	Roseburia intestinalis	Bacteria	Firmicutes	Clostridia	Clostridiales	Lachnospiraceae	Roseburia	CRISPR-associated protein Csm6	csm6	pfam09659	CAS-III-A			
141114..142006	array		QRID01000006.1	Roseburia intestinalis	Bacteria	Firmicutes	Clostridia	Clostridiales	Lachnospiraceae	Roseburia		CRISPR array			13	37	34
142309..142630	-	RHG29043.1	QRID01000006.1	Roseburia intestinalis	Bacteria	Firmicutes	Clostridia	Clostridiales	Lachnospiraceae	Roseburia	CRISPR-associated endonuclease Cas2	cas2	cd09725	CAS-I,CAS-II,CAS-III,CAS-V			
142660..143665	-	RHG29044.1	QRID01000006.1	Roseburia intestinalis	Bacteria	Firmicutes	Clostridia	Clostridiales	Lachnospiraceae	Roseburia	CRISPR-associated endonuclease Cas1	cas1	cd09634	CAS-I,CAS-II,CAS-III,CAS-V			
143753..144294	array		QRID01000006.1	Roseburia intestinalis	Bacteria	Firmicutes	Clostridia	Clostridiales	Lachnospiraceae	Roseburia		CRISPR array			8	36	36
144432..145554	-	RHG29045.1	QRID01000006.1	Roseburia intestinalis	Bacteria	Firmicutes	Clostridia	Clostridiales	Lachnospiraceae	Roseburia	type III-A CRISPR-associated RAMP protein Csm5	csm5gr7	cd09662	CAS-III-A			
145550..146477	-	RHG29126.1	QRID01000006.1	Roseburia intestinalis	Bacteria	Firmicutes	Clostridia	Clostridiales	Lachnospiraceae	Roseburia	type III-A CRISPR-associated RAMP protein Csm4	csm4gr5	COG1567	CAS-III-A			
146520..147183	-	RHG29046.1	QRID01000006.1	Roseburia intestinalis	Bacteria	Firmicutes	Clostridia	Clostridiales	Lachnospiraceae	Roseburia	type III-A CRISPR-associated RAMP protein Csm3	csm3gr7	cd09684	CAS-III-A,CAS-III-D			
147198..147591	-	RHG29047.1	QRID01000006.1	Roseburia intestinalis	Bacteria	Firmicutes	Clostridia	Clostridiales	Lachnospiraceae	Roseburia	type III-A CRISPR-associated protein Csm2	csm2gr11	cd09647	CAS-III-A			
147591..149901	-	RHG29048.1	QRID01000006.1	Roseburia intestinalis	Bacteria	Firmicutes	Clostridia	Clostridiales	Lachnospiraceae	Roseburia	type III-A CRISPR-associated protein Cas10/Csm1	cas10	cd09680	CAS-III			
149897..150644	-	RHG29049.1	QRID01000006.1	Roseburia intestinalis	Bacteria	Firmicutes	Clostridia	Clostridiales	Lachnospiraceae	Roseburia	CRISPR-associated endoribonuclease Cas6	cas6	pfam10040	CAS-III,CAS-IV			
67 CAS-III-A | CBL13036.1																	
2596301..2597048	+	CBL13030.1	FP929050.1	Roseburia intestinalis XB6B4	Bacteria	Firmicutes	Clostridia	Clostridiales	Lachnospiraceae	Roseburia	CRISPR-associated protein Cas6	cas6	pfam10040	CAS-III,CAS-IV			
2597044..2599354	+	CBL13031.1	FP929050.1	Roseburia intestinalis XB6B4	Bacteria	Firmicutes	Clostridia	Clostridiales	Lachnospiraceae	Roseburia	CRISPR-associated protein, Csm1 family	cas10	cd09680	CAS-III			
2599354..2599747	+	CBL13032.1	FP929050.1	Roseburia intestinalis XB6B4	Bacteria	Firmicutes	Clostridia	Clostridiales	Lachnospiraceae	Roseburia	CRISPR-associated protein, Csm2 family	csm2gr11	cd09647	CAS-III-A			
2599762..2600425	+	CBL13033.1	FP929050.1	Roseburia intestinalis XB6B4	Bacteria	Firmicutes	Clostridia	Clostridiales	Lachnospiraceae	Roseburia	CRISPR-associated protein, Csm3 family	csm3gr7	cd09684	CAS-III-A,CAS-III-D			
2600480..2601395	+	CBL13034.1	FP929050.1	Roseburia intestinalis XB6B4	Bacteria	Firmicutes	Clostridia	Clostridiales	Lachnospiraceae	Roseburia	CRISPR-associated RAMP protein, Csm4 family	csm4gr5	COG1567	CAS-III-A			
2602847..2603671	array		FP929050.1	Roseburia intestinalis XB6B4	Bacteria	Firmicutes	Clostridia	Clostridiales	Lachnospiraceae	Roseburia		CRISPR array			12	35	36
2603757..2604027	+	CBL13035.1	FP929050.1	Roseburia intestinalis XB6B4	Bacteria	Firmicutes	Clostridia	Clostridiales	Lachnospiraceae	Roseburia	Uncharacterized protein predicted to be involved in DNA repair	cas1	pfam01867	CAS-I,CAS-II,CAS-III			
2604063..2604762	+	CBL13036.1	FP929050.1	Roseburia intestinalis XB6B4	Bacteria	Firmicutes	Clostridia	Clostridiales	Lachnospiraceae	Roseburia	CRISPR-associated protein Cas1	cas1	cd09634	CAS-I,CAS-II,CAS-III,CAS-V			
2604792..2604882	+	CBL13037.1	FP929050.1	Roseburia intestinalis XB6B4	Bacteria	Firmicutes	Clostridia	Clostridiales	Lachnospiraceae	Roseburia	hypothetical protein						
2604903..2605110	+	CBL13038.1	FP929050.1	Roseburia intestinalis XB6B4	Bacteria	Firmicutes	Clostridia	Clostridiales	Lachnospiraceae	Roseburia	CRISPR-associated protein Cas2	cas2	cd09725	CAS-I,CAS-II,CAS-III,CAS-V			
68 partial CAS-III-A | SFE73823.1																	
491..1349	+	SFE73823.1	FONP01000010.1	Peptostreptococcus sp. D1	Bacteria	Firmicutes	Clostridia	Clostridiales	Peptostreptococcaceae	Peptostreptococcus	CRISP-associated protein Cas1	cas1	cd09634	CAS-I,CAS-II,CAS-III,CAS-V			
1345..1486	+	SFE73888.1	FONP01000010.1	Peptostreptococcus sp. D1	Bacteria	Firmicutes	Clostridia	Clostridiales	Peptostreptococcaceae	Peptostreptococcus	hypothetical protein						
1492..1825	+	SFE73898.1	FONP01000010.1	Peptostreptococcus sp. D1	Bacteria	Firmicutes	Clostridia	Clostridiales	Peptostreptococcaceae	Peptostreptococcus	CRISPR-associated protein Cas2	cas2	cd09725	CAS-I,CAS-II,CAS-III,CAS-V			
1839..3183	+	SFE73908.1	FONP01000010.1	Peptostreptococcus sp. D1	Bacteria	Firmicutes	Clostridia	Clostridiales	Peptostreptococcaceae	Peptostreptococcus	CRISPR type III-A/MTUBE-associated protein Csm6	csm6	cd09746	CAS-III-A			
69 CAS-VI-A | SFR81217.1																	
1530..2330	array		FOZC01000010.1	[Clostridium] aminophilum	Bacteria	Firmicutes	Clostridia	Clostridiales	Lachnospiraceae	Lachnoclostridium		CRISPR array			12	34	35
2499..2781	+	SFR81207.1	FOZC01000010.1	[Clostridium] aminophilum	Bacteria	Firmicutes	Clostridia	Clostridiales	Lachnospiraceae	Lachnoclostridium	hypothetical protein						
2979..3984	+	SFR81217.1	FOZC01000010.1	[Clostridium] aminophilum	Bacteria	Firmicutes	Clostridia	Clostridiales	Lachnospiraceae	Lachnoclostridium	CRISP-associated protein Cas1	cas1	cd09634	CAS-I,CAS-II,CAS-III,CAS-V			
3991..4276	+	SFR81225.1	FOZC01000010.1	[Clostridium] aminophilum	Bacteria	Firmicutes	Clostridia	Clostridiales	Lachnospiraceae	Lachnoclostridium	CRISPR-associated protein Cas2	cas2	pfam09827	CAS-I,CAS-II,CAS-III			
4444..6202	+	SFR81231.1	FOZC01000010.1	[Clostridium] aminophilum	Bacteria	Firmicutes	Clostridia	Clostridiales	Lachnospiraceae	Lachnoclostridium	hypothetical protein						
6191..7937	+	SFR81241.1	FOZC01000010.1	[Clostridium] aminophilum	Bacteria	Firmicutes	Clostridia	Clostridiales	Lachnospiraceae	Lachnoclostridium	hypothetical protein						
7937..10130	+	SFR81248.1	FOZC01000010.1	[Clostridium] aminophilum	Bacteria	Firmicutes	Clostridia	Clostridiales	Lachnospiraceae	Lachnoclostridium	hypothetical protein						
10185..14343	+	SFR81258.1	FOZC01000010.1	[Clostridium] aminophilum	Bacteria	Firmicutes	Clostridia	Clostridiales	Lachnospiraceae	Lachnoclostridium	hypothetical protein	cas13a	cas13a	CAS-VI-A			
70 CAS-I-B | AEM74967.1																	
2611321..2611729	-	AEM74964.1	CP003001.1	Caldicellulosiruptor lactoaceticus 6A	Bacteria	Firmicutes	Clostridia	Thermoanaerobacterales	Thermoanaerobacterales Family III. Incertae Sedis	Caldicellulosiruptor	hypothetical protein	csx20	cls000023	CAS-I,CAS-III			
2612217..2616136	array		CP003001.1	Caldicellulosiruptor lactoaceticus 6A	Bacteria	Firmicutes	Clostridia	Thermoanaerobacterales	Thermoanaerobacterales Family III. Incertae Sedis	Caldicellulosiruptor		CRISPR array			59	37	30
2616286..2616667	+	AEM74965.1	CP003001.1	Caldicellulosiruptor lactoaceticus 6A	Bacteria	Firmicutes	Clostridia	Thermoanaerobacterales	Thermoanaerobacterales Family III. Incertae Sedis	Caldicellulosiruptor	hypothetical protein						

2616814..2617318	+	AEM74966.1	CP003001.1	Caldicellulosiruptor lactoaceticus 6A	Bacteria	Firmicutes	Clostridia	Thermoanaerobacterales	Thermoanaerobacterales Family III. Incertae Sedis	Caldicellulosiruptor	CRISPR-associated protein Cas4	cas4	pfam01930	CAS-I			
2617314..2618310	+	AEM74967.1	CP003001.1	Caldicellulosiruptor lactoaceticus 6A	Bacteria	Firmicutes	Clostridia	Thermoanaerobacterales	Thermoanaerobacterales Family III. Incertae Sedis	Caldicellulosiruptor	CRISPR-associated protein Cas1	cas1	cd09722	CAS-I-B			
2618325..2618589	+	AEM74968.1	CP003001.1	Caldicellulosiruptor lactoaceticus 6A	Bacteria	Firmicutes	Clostridia	Thermoanaerobacterales	Thermoanaerobacterales Family III. Incertae Sedis	Caldicellulosiruptor	CRISPR-associated protein Cas2	cas2	cd09725	CAS-I,CAS-II,CAS-III,CAS-V			
2618665..2620588	+	AEM74969.1	CP003001.1	Caldicellulosiruptor lactoaceticus 6A	Bacteria	Firmicutes	Clostridia	Thermoanaerobacterales	Thermoanaerobacterales Family III. Incertae Sedis	Caldicellulosiruptor	CRISPR-associated protein Csh1 domain protein	cas8b1	cd09730	CAS-I-B			
2620652..2621633	+	AEM74970.1	CP003001.1	Caldicellulosiruptor lactoaceticus 6A	Bacteria	Firmicutes	Clostridia	Thermoanaerobacterales	Thermoanaerobacterales Family III. Incertae Sedis	Caldicellulosiruptor	CRISPR-associated protein, Csh2 family	cas7	cd09690	CAS-I-B			
2621655..2622387	+	AEM74971.1	CP003001.1	Caldicellulosiruptor lactoaceticus 6A	Bacteria	Firmicutes	Clostridia	Thermoanaerobacterales	Thermoanaerobacterales Family III. Incertae Sedis	Caldicellulosiruptor	CRISPR-associated protein Cas5, Hmari subtype	cas5	cls001430	CAS-I-B			
2622361..2624830	+	AEM74972.1	CP003001.1	Caldicellulosiruptor lactoaceticus 6A	Bacteria	Firmicutes	Clostridia	Thermoanaerobacterales	Thermoanaerobacterales Family III. Incertae Sedis	Caldicellulosiruptor	CRISPR-associated HD domain protein	cas3HD,cas3	cd09641,COG	CAS-I			
2625113..2625344	-	AEM74973.1	CP003001.1	Caldicellulosiruptor lactoaceticus 6A	Bacteria	Firmicutes	Clostridia	Thermoanaerobacterales	Thermoanaerobacterales Family III. Incertae Sedis	Caldicellulosiruptor	hypothetical protein						
2625215..2625640	array		CP003001.1	Caldicellulosiruptor lactoaceticus 6A	Bacteria	Firmicutes	Clostridia	Thermoanaerobacterales	Thermoanaerobacterales Family III. Incertae Sedis	Caldicellulosiruptor		CRISPR array			7	37	29
71 CAS-I-B | ADQ47296.1																	
2725100..2725725	array		CP002330.1	Caldicellulosiruptor kronotskyensis 2002	Bacteria	Firmicutes	Clostridia	Thermoanaerobacterales	Thermoanaerobacterales Family III. Incertae Sedis	Caldicellulosiruptor		CRISPR array			10	36	30
2725202..2725802	-	ADQ47293.1	CP002330.1	Caldicellulosiruptor kronotskyensis 2002	Bacteria	Firmicutes	Clostridia	Thermoanaerobacterales	Thermoanaerobacterales Family III. Incertae Sedis	Caldicellulosiruptor	hypothetical protein						
2725925..2726222	+	ADQ47294.1	CP002330.1	Caldicellulosiruptor kronotskyensis 2002	Bacteria	Firmicutes	Clostridia	Thermoanaerobacterales	Thermoanaerobacterales Family III. Incertae Sedis	Caldicellulosiruptor	hypothetical protein						
2726569..2726833	-	ADQ47295.1	CP002330.1	Caldicellulosiruptor kronotskyensis 2002	Bacteria	Firmicutes	Clostridia	Thermoanaerobacterales	Thermoanaerobacterales Family III. Incertae Sedis	Caldicellulosiruptor	CRISPR-associated protein Cas2	cas2	cd09725	CAS-I,CAS-II,CAS-III,CAS-V			
2726838..2727831	-	ADQ47296.1	CP002330.1	Caldicellulosiruptor kronotskyensis 2002	Bacteria	Firmicutes	Clostridia	Thermoanaerobacterales	Thermoanaerobacterales Family III. Incertae Sedis	Caldicellulosiruptor	CRISPR-associated protein Cas1	cas1	cd09722	CAS-I-B			
2727877..2728375	-	ADQ47297.1	CP002330.1	Caldicellulosiruptor kronotskyensis 2002	Bacteria	Firmicutes	Clostridia	Thermoanaerobacterales	Thermoanaerobacterales Family III. Incertae Sedis	Caldicellulosiruptor	CRISPR-associated protein Cas4	cas4	pfam01930	CAS-I			
2728403..2730827	-	ADQ47298.1	CP002330.1	Caldicellulosiruptor kronotskyensis 2002	Bacteria	Firmicutes	Clostridia	Thermoanaerobacterales	Thermoanaerobacterales Family III. Incertae Sedis	Caldicellulosiruptor	metal dependent phosphohydrolase	cas3HD,cas3	cd09641,COG	CAS-I			
2730918..2731671	-	ADQ47299.1	CP002330.1	Caldicellulosiruptor kronotskyensis 2002	Bacteria	Firmicutes	Clostridia	Thermoanaerobacterales	Thermoanaerobacterales Family III. Incertae Sedis	Caldicellulosiruptor	CRISPR-associated protein Cas5, Hmari subtype	cas5	cd09692	CAS-I-B			
2731691..2732597	-	ADQ47300.1	CP002330.1	Caldicellulosiruptor kronotskyensis 2002	Bacteria	Firmicutes	Clostridia	Thermoanaerobacterales	Thermoanaerobacterales Family III. Incertae Sedis	Caldicellulosiruptor	CRISPR-associated protein, Csh2 family	cas7b	pfam05107	CAS-I-B,CAS-I-C			
2732617..2734681	-	ADQ47301.1	CP002330.1	Caldicellulosiruptor kronotskyensis 2002	Bacteria	Firmicutes	Clostridia	Thermoanaerobacterales	Thermoanaerobacterales Family III. Incertae Sedis	Caldicellulosiruptor	CRISPR-associated protein, Csh1 family	cas8b1	pfam09484	CAS-I-B			
2735113..2736658	array		CP002330.1	Caldicellulosiruptor kronotskyensis 2002	Bacteria	Firmicutes	Clostridia	Thermoanaerobacterales	Thermoanaerobacterales Family III. Incertae Sedis	Caldicellulosiruptor		CRISPR array			24	35	30
72 CAS-I-B | ACM61721.1																	
2788963..2789125	array		CP001393.1	Caldicellulosiruptor bescii DSM 6725	Bacteria	Firmicutes	Clostridia	Thermoanaerobacterales	Thermoanaerobacterales Family III. Incertae Sedis	Caldicellulosiruptor		CRISPR array			3	36	30
2789180..2791535	+	ACM61717.1	CP001393.1	Caldicellulosiruptor bescii DSM 6725	Bacteria	Firmicutes	Clostridia	Thermoanaerobacterales	Thermoanaerobacterales Family III. Incertae Sedis	Caldicellulosiruptor	hypothetical protein						
2791632..2792742	+	ACM61718.1	CP001393.1	Caldicellulosiruptor bescii DSM 6725	Bacteria	Firmicutes	Clostridia	Thermoanaerobacterales	Thermoanaerobacterales Family III. Incertae Sedis	Caldicellulosiruptor	hypothetical protein						
2794735..2795182	+	ACM61719.1	CP001393.1	Caldicellulosiruptor bescii DSM 6725	Bacteria	Firmicutes	Clostridia	Thermoanaerobacterales	Thermoanaerobacterales Family III. Incertae Sedis	Caldicellulosiruptor	hypothetical protein						
2795379..2795643	-	ACM61720.1	CP001393.1	Caldicellulosiruptor bescii DSM 6725	Bacteria	Firmicutes	Clostridia	Thermoanaerobacterales	Thermoanaerobacterales Family III. Incertae Sedis	Caldicellulosiruptor	CRISPR-associated protein Cas2	cas2	cd09725	CAS-I,CAS-II,CAS-III,CAS-V			
2795648..2796641	-	ACM61721.1	CP001393.1	Caldicellulosiruptor bescii DSM 6725	Bacteria	Firmicutes	Clostridia	Thermoanaerobacterales	Thermoanaerobacterales Family III. Incertae Sedis	Caldicellulosiruptor	CRISPR-associated protein Cas1	cas1	cd09722	CAS-I-B			
2796687..2797245	-	ACM61722.1	CP001393.1	Caldicellulosiruptor bescii DSM 6725	Bacteria	Firmicutes	Clostridia	Thermoanaerobacterales	Thermoanaerobacterales Family III. Incertae Sedis	Caldicellulosiruptor	CRISPR-associated protein Cas4	cas4	pfam01930	CAS-I			
2797213..2799637	-	ACM61723.1	CP001393.1	Caldicellulosiruptor bescii DSM 6725	Bacteria	Firmicutes	Clostridia	Thermoanaerobacterales	Thermoanaerobacterales Family III. Incertae Sedis	Caldicellulosiruptor	CRISPR-associated helicase Cas3	cas3HD,cas3	cd09641,COG	CAS-I			
2799728..2800481	-	ACM61724.1	CP001393.1	Caldicellulosiruptor bescii DSM 6725	Bacteria	Firmicutes	Clostridia	Thermoanaerobacterales	Thermoanaerobacterales Family III. Incertae Sedis	Caldicellulosiruptor	CRISPR-associated protein Cas5, Hmari subtype	cas5	cd09692	CAS-I-B			
2800501..2801407	-	ACM61725.1	CP001393.1	Caldicellulosiruptor bescii DSM 6725	Bacteria	Firmicutes	Clostridia	Thermoanaerobacterales	Thermoanaerobacterales Family III. Incertae Sedis	Caldicellulosiruptor	CRISPR-associated protein, Csh2 family	cas7b	pfam05107	CAS-I-B,CAS-I-C			
2801427..2803488	-	ACM61726.1	CP001393.1	Caldicellulosiruptor bescii DSM 6725	Bacteria	Firmicutes	Clostridia	Thermoanaerobacterales	Thermoanaerobacterales Family III. Incertae Sedis	Caldicellulosiruptor	CRISPR-associated protein, Csh1 family	cas8b1	pfam09484	CAS-I-B			
2803920..2807402	array		CP001393.1	Caldicellulosiruptor bescii DSM 6725	Bacteria	Firmicutes	Clostridia	Thermoanaerobacterales	Thermoanaerobacterales Family III. Incertae Sedis	Caldicellulosiruptor		CRISPR array			53	36	30
73 CAS-I-B | ADQ41960.1																	
2652729..2654679	array		CP002326.1	Caldicellulosiruptor kristjanssonii I77R1B	Bacteria	Firmicutes	Clostridia	Thermoanaerobacterales	Thermoanaerobacterales Family III. Incertae Sedis	Caldicellulosiruptor		CRISPR array			30	36	30
2655064..2656768	+	ADQ41956.1	CP002326.1	Caldicellulosiruptor kristjanssonii I77R1B	Bacteria	Firmicutes	Clostridia	Thermoanaerobacterales	Thermoanaerobacterales Family III. Incertae Sedis	Caldicellulosiruptor	transposase IS4 family protein						
2656917..2657166	-	ADQ41957.1	CP002326.1	Caldicellulosiruptor kristjanssonii I77R1B	Bacteria	Firmicutes	Clostridia	Thermoanaerobacterales	Thermoanaerobacterales Family III. Incertae Sedis	Caldicellulosiruptor	hypothetical protein						
2657380..2657653	-	ADQ41958.1	CP002326.1	Caldicellulosiruptor kristjanssonii I77R1B	Bacteria	Firmicutes	Clostridia	Thermoanaerobacterales	Thermoanaerobacterales Family III. Incertae Sedis	Caldicellulosiruptor	hypothetical protein	Csm6	cluster3				
2657832..2658096	-	ADQ41959.1	CP002326.1	Caldicellulosiruptor kristjanssonii I77R1B	Bacteria	Firmicutes	Clostridia	Thermoanaerobacterales	Thermoanaerobacterales Family III. Incertae Sedis	Caldicellulosiruptor	CRISPR-associated protein Cas2	cas2	cd09725	CAS-I,CAS-II,CAS-III,CAS-V			
2658101..2659094	-	ADQ41960.1	CP002326.1	Caldicellulosiruptor kristjanssonii I77R1B	Bacteria	Firmicutes	Clostridia	Thermoanaerobacterales	Thermoanaerobacterales Family III. Incertae Sedis	Caldicellulosiruptor	CRISPR-associated protein Cas1	cas1	cd09722	CAS-I-B			
2659135..2659633	-	ADQ41961.1	CP002326.1	Caldicellulosiruptor kristjanssonii I77R1B	Bacteria	Firmicutes	Clostridia	Thermoanaerobacterales	Thermoanaerobacterales Family III. Incertae Sedis	Caldicellulosiruptor	CRISPR-associated protein Cas4	cas4	pfam01930	CAS-I			
2659662..2662086	-	ADQ41962.1	CP002326.1	Caldicellulosiruptor kristjanssonii I77R1B	Bacteria	Firmicutes	Clostridia	Thermoanaerobacterales	Thermoanaerobacterales Family III. Incertae Sedis	Caldicellulosiruptor	CRISPR-associated HD domain protein	cas3HD,cas3	cd09641,COG	CAS-I			
2662175..2662928	-	ADQ41963.1	CP002326.1	Caldicellulosiruptor kristjanssonii I77R1B	Bacteria	Firmicutes	Clostridia	Thermoanaerobacterales	Thermoanaerobacterales Family III. Incertae Sedis	Caldicellulosiruptor	CRISPR-associated protein Cas5, Hmari subtype	cas5	cd09692	CAS-I-B			
2662949..2663834	-	ADQ41964.1	CP002326.1	Caldicellulosiruptor kristjanssonii I77R1B	Bacteria	Firmicutes	Clostridia	Thermoanaerobacterales	Thermoanaerobacterales Family III. Incertae Sedis	Caldicellulosiruptor	CRISPR-associated protein, Csh2 family	cas7b	pfam05107	CAS-I-B,CAS-I-C			
2663855..2665919	-	ADQ41965.1	CP002326.1	Caldicellulosiruptor kristjanssonii I77R1B	Bacteria	Firmicutes	Clostridia	Thermoanaerobacterales	Thermoanaerobacterales Family III. Incertae Sedis	Caldicellulosiruptor	CRISPR-associated protein, Csh1 family	cas8b1	pfam09484	CAS-I-B			
2666352..2668033	array		CP002326.1	Caldicellulosiruptor kristjanssonii I77R1B	Bacteria	Firmicutes	Clostridia	Thermoanaerobacterales	Thermoanaerobacterales Family III. Incertae Sedis	Caldicellulosiruptor		CRISPR array			26	36	30
74 CAS-I-B | AEM74919.1																	
2552404..2554751	array		CP003001.1	Caldicellulosiruptor lactoaceticus 6A	Bacteria	Firmicutes	Clostridia	Thermoanaerobacterales	Thermoanaerobacterales Family III. Incertae Sedis	Caldicellulosiruptor		CRISPR array			36	36	30
2554908..2555907	+	AEM74917.1	CP003001.1	Caldicellulosiruptor lactoaceticus 6A	Bacteria	Firmicutes	Clostridia	Thermoanaerobacterales	Thermoanaerobacterales Family III. Incertae Sedis	Caldicellulosiruptor	Conserved hypothetical protein CHP01784						
2555896..2556160	-	AEM74918.1	CP003001.1	Caldicellulosiruptor lactoaceticus 6A	Bacteria	Firmicutes	Clostridia	Thermoanaerobacterales	Thermoanaerobacterales Family III. Incertae Sedis	Caldicellulosiruptor	CRISPR-associated protein Cas2	cas2	cd09725	CAS-I,CAS-II,CAS-III,CAS-V			
2556165..2557158	-	AEM74919.1	CP003001.1	Caldicellulosiruptor lactoaceticus 6A	Bacteria	Firmicutes	Clostridia	Thermoanaerobacterales	Thermoanaerobacterales Family III. Incertae Sedis	Caldicellulosiruptor	CRISPR-associated protein Cas1	cas1	cd09722	CAS-I-B			
2557199..2557697	-	AEM74920.1	CP003001.1	Caldicellulosiruptor lactoaceticus 6A	Bacteria	Firmicutes	Clostridia	Thermoanaerobacterales	Thermoanaerobacterales Family III. Incertae Sedis	Caldicellulosiruptor	CRISPR-associated protein Cas4	cas4	pfam01930	CAS-I			
2557726..2560150	-	AEM74921.1	CP003001.1	Caldicellulosiruptor lactoaceticus 6A	Bacteria	Firmicutes	Clostridia	Thermoanaerobacterales	Thermoanaerobacterales Family III. Incertae Sedis	Caldicellulosiruptor	metal dependent phosphohydrolase	cas3HD,cas3	cd09641,COG	CAS-I			
2560239..2560992	-	AEM74922.1	CP003001.1	Caldicellulosiruptor lactoaceticus 6A	Bacteria	Firmicutes	Clostridia	Thermoanaerobacterales	Thermoanaerobacterales Family III. Incertae Sedis	Caldicellulosiruptor	CRISPR-associated protein Cas5, Hmari subtype	cas5	cd09692	CAS-I-B			
2561013..2561898	-	AEM74923.1	CP003001.1	Caldicellulosiruptor lactoaceticus 6A	Bacteria	Firmicutes	Clostridia	Thermoanaerobacterales	Thermoanaerobacterales Family III. Incertae Sedis	Caldicellulosiruptor	CRISPR-associated protein, Csh2 family	cas7b	pfam05107	CAS-I-B,CAS-I-C			
2561919..2563983	-	AEM74924.1	CP003001.1	Caldicellulosiruptor lactoaceticus 6A	Bacteria	Firmicutes	Clostridia	Thermoanaerobacterales	Thermoanaerobacterales Family III. Incertae Sedis	Caldicellulosiruptor	CRISPR-associated protein Csh1 domain protein	cas8b1	pfam09484	CAS-I-B			
2564413..2566152	array		CP003001.1	Caldicellulosiruptor lactoaceticus 6A	Bacteria	Firmicutes	Clostridia	Thermoanaerobacterales	Thermoanaerobacterales Family III. Incertae Sedis	Caldicellulosiruptor		CRISPR array			27	35	30
2566391..2567590	-	AEM74925.1	CP003001.1	Caldicellulosiruptor lactoaceticus 6A	Bacteria	Firmicutes	Clostridia	Thermoanaerobacterales	Thermoanaerobacterales Family III. Incertae Sedis	Caldicellulosiruptor	CRISPR-associated protein, APE2256 family	csm6	cd09742	CAS-III-A,CAS-III-D			
75 CAS-III-D | SCX12018.1																	
29061..29783	array		FMUA01000006.1	Ruminococcaceae bacterium P7	Bacteria	Firmicutes	Clostridia	Clostridiales	Ruminococcaceae			CRISPR array			12	38	25
29961..30225	-	SCX12007.1	FMUA01000006.1	Ruminococcaceae bacterium P7	Bacteria	Firmicutes	Clostridia	Clostridiales	Ruminococcaceae		CRISPR-associated protein Cas2	cas2	cd09725	CAS-I,CAS-II,CAS-III,CAS-V			
30224..32138	-	SCX12018.1	FMUA01000006.1	Ruminococcaceae bacterium P7	Bacteria	Firmicutes	Clostridia	Clostridiales	Ruminococcaceae		CRISPR-associated endonuclease Cas1	RT,cas1	pfam00078,cc	CAS-I,CAS-III			
32368..32851	array		FMUA01000006.1	Ruminococcaceae bacterium P7	Bacteria	Firmicutes	Clostridia	Clostridiales	Ruminococcaceae			CRISPR array			8	37	28
32999..34973	-	SCX12029.1	FMUA01000006.1	Ruminococcaceae bacterium P7	Bacteria	Firmicutes	Clostridia	Clostridiales	Ruminococcaceae		CRISPR-associated protein	csm3gr7,csm3gr7	cls000253,cls(	CAS-III-A,CAS-III-D			
34972..35419	-	SCX12041.1	FMUA01000006.1	Ruminococcaceae bacterium P7	Bacteria	Firmicutes	Clostridia	Clostridiales	Ruminococcaceae		hypothetical protein	Cas11/Csx19	cluster5				
35411..36632	-	SCX12054.1	FMUA01000006.1	Ruminococcaceae bacterium P7	Bacteria	Firmicutes	Clostridia	Clostridiales	Ruminococcaceae		CRISPR/Cas system CSM-associated protein Csm3, group 7 of RAMP superfamily	csm3gr7,csm3gr7	cd09726,cd09	CAS-III-D			
36633..38643	-	SCX12065.1	FMUA01000006.1	Ruminococcaceae bacterium P7	Bacteria	Firmicutes	Clostridia	Clostridiales	Ruminococcaceae		CRISPR/Cas system CSM-associated protein Csm3, group 7 of RAMP superfamily	csm3gr7,csm3gr7,csx10gr5	mkCas0198,cc	CAS-III-A,CAS-III-D			
38614..40042	-	SCX12077.1	FMUA01000006.1	Ruminococcaceae bacterium P7	Bacteria	Firmicutes	Clostridia	Clostridiales	Ruminococcaceae		hypothetical protein	cas10	mkCas0133	CAS-III			
40113..40821	+	SCX12088.1	FMUA01000006.1	Ruminococcaceae bacterium P7	Bacteria	Firmicutes	Clostridia	Clostridiales	Ruminococcaceae		CRISPR-associated endoribonuclease Cas6	cas6	COG1583	CAS-I,CAS-III,CAS-IV			
40908..41682	+	SCX12099.1	FMUA01000006.1	Ruminococcaceae bacterium P7	Bacteria	Firmicutes	Clostridia	Clostridiales	Ruminococcaceae		hypothetical protein	PD-DExK	mkCas0090	CAS-I,CAS-III			
76 Partial | SCX12129.1																	
42905..43320	array		FMUA01000006.1	Ruminococcaceae bacterium P7	Bacteria	Firmicutes	Clostridia	Clostridiales	Ruminococcaceae			CRISPR array			7	36	28
43499..43763	-	SCX12118.1	FMUA01000006.1	Ruminococcaceae bacterium P7	Bacteria	Firmicutes	Clostridia	Clostridiales	Ruminococcaceae		CRISPR-associated protein Cas2	cas2	cd09725	CAS-I,CAS-II,CAS-III,CAS-V			
43762..45676	-	SCX12129.1	FMUA01000006.1	Ruminococcaceae bacterium P7	Bacteria	Firmicutes	Clostridia	Clostridiales	Ruminococcaceae		CRISPR-associated endonuclease Cas1/group II intron reverse transcriptase/maturase,TIGR04416	RT,cas1	pfam00078,cc	CAS-I,CAS-III			
45905..46259	array		FMUA01000006.1	Ruminococcaceae bacterium P7	Bacteria	Firmicutes	Clostridia	Clostridiales	Ruminococcaceae			CRISPR array			6	37	28
77 CAS-III-B | OUQ05160.1																	
142196..143597	+	OUQ05153.1	NFLB01000007.1	[Clostridium] spiroforme	Bacteria	Firmicutes	Erysipelotrichia	Erysipelotrichales	Erysipelotrichaceae	Erysipelatoclostridium	hypothetical protein	cmr1gr7	mkCas0086	CAS-III-B			
143600..145184	+	OUQ05154.1	NFLB01000007.1	[Clostridium] spiroforme	Bacteria	Firmicutes	Erysipelotrichia	Erysipelotrichales	Erysipelotrichaceae	Erysipelatoclostridium	hypothetical protein	cas10,cas10	pfam12469,cc	CAS-III-B			
145189..147136	+	OUQ05155.1	NFLB01000007.1	[Clostridium] spiroforme	Bacteria	Firmicutes	Erysipelotrichia	Erysipelotrichales	Erysipelotrichaceae	Erysipelatoclostridium	type III-B CRISPR module RAMP protein Cmr4	cmr3gr5,cmr4gr7	pfam09700,cc	CAS-III-B			
147149..147524	+	OUQ05156.1	NFLB01000007.1	[Clostridium] spiroforme	Bacteria	Firmicutes	Erysipelotrichia	Erysipelotrichales	Erysipelotrichaceae	Erysipelatoclostridium	hypothetical protein	cmr5gr11	mkCas0098	CAS-III-B			
147526..148396	+	OUQ05157.1	NFLB01000007.1	[Clostridium] spiroforme	Bacteria	Firmicutes	Erysipelotrichia	Erysipelotrichales	Erysipelotrichaceae	Erysipelatoclostridium	type III-B CRISPR module RAMP protein Cmr6	cmr6gr7	cd09661	CAS-III-B			
148388..150548	+	OUQ05158.1	NFLB01000007.1	[Clostridium] spiroforme	Bacteria	Firmicutes	Erysipelotrichia	Erysipelotrichales	Erysipelotrichaceae	Erysipelatoclostridium	hypothetical protein	CorA	icity0111				
150872..151931	+	OUQ05159.1	NFLB01000007.1	[Clostridium] spiroforme	Bacteria	Firmicutes	Erysipelotrichia	Erysipelotrichales	Erysipelotrichaceae	Erysipelatoclostridium	hypothetical protein						
152269..154159	+	OUQ05160.1	NFLB01000007.1	[Clostridium] spiroforme	Bacteria	Firmicutes	Erysipelotrichia	Erysipelotrichales	Erysipelotrichaceae	Erysipelatoclostridium	CRISPR-associated endonuclease Cas1	RT,cas1	pfam00078,cc	CAS-I,CAS-III			
154161..154422	+	OUQ05161.1	NFLB01000007.1	[Clostridium] spiroforme	Bacteria	Firmicutes	Erysipelotrichia	Erysipelotrichales	Erysipelotrichaceae	Erysipelatoclostridium	CRISPR-associated endonuclease Cas2	cas2	cd09725	CAS-I,CAS-II,CAS-III,CAS-V			
154693..155150	array		NFLB01000007.1	[Clostridium] spiroforme	Bacteria	Firmicutes	Erysipelotrichia	Erysipelotrichales	Erysipelotrichaceae	Erysipelatoclostridium		CRISPR array			7	42	29
155200..155935	+	OUQ05162.1	NFLB01000007.1	[Clostridium] spiroforme	Bacteria	Firmicutes	Erysipelotrichia	Erysipelotrichales	Erysipelotrichaceae	Erysipelatoclostridium	CRISPR-associated endoribonuclease Cas6	cas6	COG1583	CAS-I,CAS-III,CAS-IV			
156309..156552	array		NFLB01000007.1	[Clostridium] spiroforme	Bacteria	Firmicutes	Erysipelotrichia	Erysipelotrichales	Erysipelotrichaceae	Erysipelatoclostridium		CRISPR array			4	42	29
78 CAS-III-D | RGG82469.1																	
8633..9282	array		QTXB01000028.1	Coprobacillus sp. AF17-11AC	Bacteria	Firmicutes	Erysipelotrichia	Erysipelotrichales	Erysipelotrichaceae	Coprobacillus		CRISPR array			10	36	32
9404..10961	+	RGG82468.1	QTXB01000028.1	Coprobacillus sp. AF17-11AC	Bacteria	Firmicutes	Erysipelotrichia	Erysipelotrichales	Erysipelotrichaceae	Coprobacillus	hypothetical protein	csx1	pfam09455	CAS-III			
10963..12856	+	RGG82469.1	QTXB01000028.1	Coprobacillus sp. AF17-11AC	Bacteria	Firmicutes	Erysipelotrichia	Erysipelotrichales	Erysipelotrichaceae	Coprobacillus	CRISPR-associated endonuclease Cas1	RT,cas1	pfam00078,cc	CAS-I,CAS-III			
12855..13119	+	RGG82470.1	QTXB01000028.1	Coprobacillus sp. AF17-11AC	Bacteria	Firmicutes	Erysipelotrichia	Erysipelotrichales	Erysipelotrichaceae	Coprobacillus	CRISPR-associated endonuclease Cas2	cas2	cd09725	CAS-I,CAS-II,CAS-III,CAS-V			
13559..13775	array		QTXB01000028.1	Coprobacillus sp. AF17-11AC	Bacteria	Firmicutes	Erysipelotrichia	Erysipelotrichales	Erysipelotrichaceae	Coprobacillus		CRISPR array			4	41	23
14344..14779	+	RGG82471.1	QTXB01000028.1	Coprobacillus sp. AF17-11AC	Bacteria	Firmicutes	Erysipelotrichia	Erysipelotrichales	Erysipelotrichaceae	Coprobacillus	hypothetical protein						
14876..15560	+	RGG82472.1	QTXB01000028.1	Coprobacillus sp. AF17-11AC	Bacteria	Firmicutes	Erysipelotrichia	Erysipelotrichales	Erysipelotrichaceae	Coprobacillus	hypothetical protein						
15731..17222	+	RGG82473.1	QTXB01000028.1	Coprobacillus sp. AF17-11AC	Bacteria	Firmicutes	Erysipelotrichia	Erysipelotrichales	Erysipelotrichaceae	Coprobacillus	hypothetical protein	cas10	mkCas0133	CAS-III			
17214..19215	+	RGG82474.1	QTXB01000028.1	Coprobacillus sp. AF17-11AC	Bacteria	Firmicutes	Erysipelotrichia	Erysipelotrichales	Erysipelotrichaceae	Coprobacillus	hypothetical protein	csm3gr7	mkCas0198	CAS-III-A,CAS-III-D			
19211..20423	+	RGG82475.1	QTXB01000028.1	Coprobacillus sp. AF17-11AC	Bacteria	Firmicutes	Erysipelotrichia	Erysipelotrichales	Erysipelotrichaceae	Coprobacillus	hypothetical protein	csm3gr7,csm3gr7	cd09726,pfam	CAS-III-D			
20416..20875	+	RGG82476.1	QTXB01000028.1	Coprobacillus sp. AF17-11AC	Bacteria	Firmicutes	Erysipelotrichia	Erysipelotrichales	Erysipelotrichaceae	Coprobacillus	hypothetical protein	csx19	cls001376	CAS-III-D			
20867..22610	+	RGG82477.1	QTXB01000028.1	Coprobacillus sp. AF17-11AC	Bacteria	Firmicutes	Erysipelotrichia	Erysipelotrichales	Erysipelotrichaceae	Coprobacillus	hypothetical protein	csm3gr7,csm3gr7	cls000253,cls(	CAS-III-A,CAS-III-D			
22575..23286	+	RGG82478.1	QTXB01000028.1	Coprobacillus sp. AF17-11AC	Bacteria	Firmicutes	Erysipelotrichia	Erysipelotrichales	Erysipelotrichaceae	Coprobacillus	CRISPR-associated endoribonuclease Cas6	cas6	COG1583	CAS-I,CAS-III,CAS-IV			
23300..23660	+	RGG82479.1	QTXB01000028.1	Coprobacillus sp. AF17-11AC	Bacteria	Firmicutes	Erysipelotrichia	Erysipelotrichales	Erysipelotrichaceae	Coprobacillus	CRISPR-associated protein	csx20	cls000118	CAS-I,CAS-III			
24023..26795	+	RGG82480.1	QTXB01000028.1	Coprobacillus sp. AF17-11AC	Bacteria	Firmicutes	Erysipelotrichia	Erysipelotrichales	Erysipelotrichaceae	Coprobacillus	WYL domain-containing protein	WYL,WYL	pfam13280,pt	CAS-I,CAS-III			
79 CAS-III-D | RGI19592.1																	
8764..9283	array		QTYY01000006.1	Ruminococcus sp. TF08-4	Bacteria	Firmicutes	Clostridia	Clostridiales	Ruminococcaceae	Ruminococcus		CRISPR array			8	41	29
9580..11482	-	RGI19592.1	QTYY01000006.1	Ruminococcus sp. TF08-4	Bacteria	Firmicutes	Clostridia	Clostridiales	Ruminococcaceae	Ruminococcus	CRISPR-associated endonuclease Cas1	RT,cas1	pfam00078,cc	CAS-I,CAS-III			
11509..11770	-	RGI19744.1	QTYY01000006.1	Ruminococcus sp. TF08-4	Bacteria	Firmicutes	Clostridia	Clostridiales	Ruminococcaceae	Ruminococcus	CRISPR-associated endonuclease Cas2	cas2	cd09725	CAS-I,CAS-II,CAS-III,CAS-V			
11785..12349	array		QTYY01000006.1	Ruminococcus sp. TF08-4	Bacteria	Firmicutes	Clostridia	Clostridiales	Ruminococcaceae	Ruminococcus		CRISPR array			9	38	29

12606..13329	-	RGI19593.1	QTYY01000006.1	Ruminococcus sp. TF08-4	Bacteria	Firmicutes	Clostridia	Clostridiales	Ruminococcaceae	Ruminococcus	CRISPR-associated endoribonuclease Cas6	cas6	COG1583	CAS-I,CAS-III,CAS-IV			
13389..15555	-	RGI19594.1	QTYY01000006.1	Ruminococcus sp. TF08-4	Bacteria	Firmicutes	Clostridia	Clostridiales	Ruminococcaceae	Ruminococcus	TIGR03986 family CRISPR-associated RAMP protein	csm3gr7	cls000253	CAS-III-A,CAS-III-D			
15556..16093	-	RGI19595.1	QTYY01000006.1	Ruminococcus sp. TF08-4	Bacteria	Firmicutes	Clostridia	Clostridiales	Ruminococcaceae	Ruminococcus	hypothetical protein	Cas11/Csx19	cluster5				
16089..17364	-	RGI19596.1	QTYY01000006.1	Ruminococcus sp. TF08-4	Bacteria	Firmicutes	Clostridia	Clostridiales	Ruminococcaceae	Ruminococcus	hypothetical protein	csm3gr7,csm3gr7	cd09726,cd0	CAS-III-D			
17360..19532	-	RGI19597.1	QTYY01000006.1	Ruminococcus sp. TF08-4	Bacteria	Firmicutes	Clostridia	Clostridiales	Ruminococcaceae	Ruminococcus	hypothetical protein	csm3gr7,csx10gr5	mkCas0198,cl	CAS-III-A,CAS-III-D			
19513..21022	-	RGI19598.1	QTYY01000006.1	Ruminococcus sp. TF08-4	Bacteria	Firmicutes	Clostridia	Clostridiales	Ruminococcaceae	Ruminococcus	hypothetical protein	cas10	mkCas0133	CAS-III			
21034..21397	-	RGI19599.1	QTYY01000006.1	Ruminococcus sp. TF08-4	Bacteria	Firmicutes	Clostridia	Clostridiales	Ruminococcaceae	Ruminococcus	CRISPR-associated protein	csx20	cls000118	CAS-I,CAS-III			
21426..22968	-	RGI19600.1	QTYY01000006.1	Ruminococcus sp. TF08-4	Bacteria	Firmicutes	Clostridia	Clostridiales	Ruminococcaceae	Ruminococcus	hypothetical protein	csx1	pfam09455	CAS-III			

80 CAS-III-D | CDC93231.1

128..566	array		FR891835.1	Firmicutes bacterium CAG:227	Bacteria	Firmicutes						CRISPR array			7	39	29
793..1183	+	CDC93212.1	FR891835.1	Firmicutes bacterium CAG:227	Bacteria	Firmicutes					unknown						
1362..2142	+	CDC93213.1	FR891835.1	Firmicutes bacterium CAG:227	Bacteria	Firmicutes					uncharacterized protein						
2125..3739	+	CDC93214.1	FR891835.1	Firmicutes bacterium CAG:227	Bacteria	Firmicutes					traG/TraD family protein						
4119..4350	+	CDC93217.1	FR891835.1	Firmicutes bacterium CAG:227	Bacteria	Firmicutes					aTP-dependent Clp protease proteolytic subunit						
4762..5050	array		FR891835.1	Firmicutes bacterium CAG:227	Bacteria	Firmicutes						CRISPR array			5	36	29
5180..7622	-	CDC93219.1	FR891835.1	Firmicutes bacterium CAG:227	Bacteria	Firmicutes					putative uncharacterized protein	CARF	icity0119				
7870..8230	+	CDC93221.1	FR891835.1	Firmicutes bacterium CAG:227	Bacteria	Firmicutes					putative uncharacterized protein	csx20	cls000118	CAS-I,CAS-III			
8280..9786	+	CDC93223.1	FR891835.1	Firmicutes bacterium CAG:227	Bacteria	Firmicutes					putative uncharacterized protein	cas10	mkCas0133	CAS-III			
9757..11911	+	CDC93224.1	FR891835.1	Firmicutes bacterium CAG:227	Bacteria	Firmicutes					putative uncharacterized protein	csm3gr7,csm4gr5,csx10gr5	mkCas0198,m	CAS-III-A,CAS-III-D			
11907..13179	+	CDC93225.1	FR891835.1	Firmicutes bacterium CAG:227	Bacteria	Firmicutes					putative uncharacterized protein	csm3gr7,csm3gr7	cd09726,cd0	CAS-III-D			
13175..13556	+	CDC93226.1	FR891835.1	Firmicutes bacterium CAG:227	Bacteria	Firmicutes					putative uncharacterized protein	Csx19	icity0015				
13558..15685	+	CDC93227.1	FR891835.1	Firmicutes bacterium CAG:227	Bacteria	Firmicutes					putative uncharacterized protein	csm3gr7	cls000253	CAS-III-A,CAS-III-D			
15687..16413	+	CDC93228.1	FR891835.1	Firmicutes bacterium CAG:227	Bacteria	Firmicutes					putative uncharacterized protein	cas6	COG1583	CAS-I,CAS-III,CAS-IV			
16581..17085	array		FR891835.1	Firmicutes bacterium CAG:227	Bacteria	Firmicutes						CRISPR array			8	37	30
17139..17400	+	CDC93229.1	FR891835.1	Firmicutes bacterium CAG:227	Bacteria	Firmicutes					cRISPR-associated endoribonuclease Cas2	cas2	cd09725	CAS-I,CAS-II,CAS-III,CAS-V			
17409..18036	+	CDC93230.1	FR891835.1	Firmicutes bacterium CAG:227	Bacteria	Firmicutes					cRISPR-associated endonuclease Cas1 2	RT	pfam00078	CAS-I,CAS-III			
18036..19314	+	CDC93231.1	FR891835.1	Firmicutes bacterium CAG:227	Bacteria	Firmicutes					cRISPR-associated endonuclease Cas1 2	RT,cas1	pfam00078,cc	CAS-I,CAS-III			
19506..19874	array		FR891835.1	Firmicutes bacterium CAG:227	Bacteria	Firmicutes						CRISPR array			6	39	29

81 CAS-III-D | CUN56577.1

56025..56391	+	CUN56023.1	CYYV01000002.1	Fusicatenibacter saccharivorans	Bacteria	Firmicutes	Clostridia	Clostridiales	Lachnospiraceae	Fusicatenibacter	Uncharacterised protein	csx20	cls000118	CAS-I,CAS-III			
56410..57946	+	CUN56097.1	CYYV01000002.1	Fusicatenibacter saccharivorans	Bacteria	Firmicutes	Clostridia	Clostridiales	Lachnospiraceae	Fusicatenibacter	CRISPR-associated protein Cas10/Csm1%2C subtype III-A/MTUBE	cas10	mkCas0133	CAS-III			
57936..60066	+	CUN56168.1	CYYV01000002.1	Fusicatenibacter saccharivorans	Bacteria	Firmicutes	Clostridia	Clostridiales	Lachnospiraceae	Fusicatenibacter	CRISPR-associated RAMP protein%2C Csx10 family	csm3gr7,csx10gr5	mkCas0198,cl	CAS-III-A,CAS-III-D			
60062..61430	+	CUN56243.1	CYYV01000002.1	Fusicatenibacter saccharivorans	Bacteria	Firmicutes	Clostridia	Clostridiales	Lachnospiraceae	Fusicatenibacter	CRISPR-associated RAMP protein%2C SSO1426 family	csm3gr7,csm3gr7	cd09726,cd0	CAS-III-D			
61435..61807	+	CUN56311.1	CYYV01000002.1	Fusicatenibacter saccharivorans	Bacteria	Firmicutes	Clostridia	Clostridiales	Lachnospiraceae	Fusicatenibacter	Uncharacterised protein	Csx19	icity0015				
61808..63626	+	CUN56377.1	CYYV01000002.1	Fusicatenibacter saccharivorans	Bacteria	Firmicutes	Clostridia	Clostridiales	Lachnospiraceae	Fusicatenibacter	CRISPR-associated protein	csm3gr7,csm3gr7	cls000253,cls	CAS-III-A,CAS-III-D			
63628..64351	+	CUN56445.1	CYYV01000002.1	Fusicatenibacter saccharivorans	Bacteria	Firmicutes	Clostridia	Clostridiales	Lachnospiraceae	Fusicatenibacter	Uncharacterized protein predicted to be involved in DNA repair (RAMP superfamily)	cas6	COG1583	CAS-I,CAS-III,CAS-IV			
64533..65386	array		CYYV01000002.1	Fusicatenibacter saccharivorans	Bacteria	Firmicutes	Clostridia	Clostridiales	Lachnospiraceae	Fusicatenibacter		CRISPR array			13	39	29
65408..65669	+	CUN56512.1	CYYV01000002.1	Fusicatenibacter saccharivorans	Bacteria	Firmicutes	Clostridia	Clostridiales	Lachnospiraceae	Fusicatenibacter	CRISPR-associated endoribonuclease Cas2	cas2	cd09725	CAS-I,CAS-II,CAS-III,CAS-V			
65676..67575	+	CUN56577.1	CYYV01000002.1	Fusicatenibacter saccharivorans	Bacteria	Firmicutes	Clostridia	Clostridiales	Lachnospiraceae	Fusicatenibacter	Group II intron-encoded protein ltrA	RT,cas1	pfam00078,cc	CAS-I,CAS-III			
67767..68125	array		CYYV01000002.1	Fusicatenibacter saccharivorans	Bacteria	Firmicutes	Clostridia	Clostridiales	Lachnospiraceae	Fusicatenibacter		CRISPR array			6	37	29

82 CAS-VI-A | WP_087253222.1

80478..84588	+	WP_087253216.	NZ_NFHY01000010.1	Drancourtella sp. An57	Bacteria	Firmicutes	Clostridia	Clostridiales	Ruminococcaceae	Drancourtella	hypothetical protein	cas13a	cas13a	CAS-VI-A			
84591..84852	+	WP_087253218.	NZ_NFHY01000010.1	Drancourtella sp. An57	Bacteria	Firmicutes	Clostridia	Clostridiales	Ruminococcaceae	Drancourtella	CRISPR-associated endonuclease Cas2	cas2	cd09725	CAS-I,CAS-II,CAS-III,CAS-V			
84864..85434	+	WP_087253220.	NZ_NFHY01000010.1	Drancourtella sp. An57	Bacteria	Firmicutes	Clostridia	Clostridiales	Ruminococcaceae	Drancourtella	hypothetical protein	RT	pfam00078	CAS-I,CAS-III			
85406..86759	+	WP_087253222.	NZ_NFHY01000010.1	Drancourtella sp. An57	Bacteria	Firmicutes	Clostridia	Clostridiales	Ruminococcaceae	Drancourtella	CRISPR-associated endonuclease Cas1	RT,cas1	pfam00078,cc	CAS-I,CAS-III			
86966..87387	array		NZ_NFHY01000010.1	Drancourtella sp. An57	Bacteria	Firmicutes	Clostridia	Clostridiales	Ruminococcaceae	Drancourtella		CRISPR array			6	47	31

83 CAS-VI-A | RGR54906.1

107860..108174	array		QRUJ01000006.1	[Eubacterium] rectale	Bacteria	Firmicutes	Clostridia	Clostridiales	Lachnospiraceae			CRISPR array			5	38	32
108543..112782	+	RGR54904.1	QRUJ01000006.1	[Eubacterium] rectale	Bacteria	Firmicutes	Clostridia	Clostridiales	Lachnospiraceae		hypothetical protein	cas13a	cas13a	CAS-VI-A			
112799..113060	+	RGR54905.1	QRUJ01000006.1	[Eubacterium] rectale	Bacteria	Firmicutes	Clostridia	Clostridiales	Lachnospiraceae		CRISPR-associated endonuclease Cas2	cas2	cd09725	CAS-I,CAS-II,CAS-III,CAS-V			
113078..114974	+	RGR54906.1	QRUJ01000006.1	[Eubacterium] rectale	Bacteria	Firmicutes	Clostridia	Clostridiales	Lachnospiraceae		CRISPR-associated endonuclease Cas1	RT,cas1	pfam00078,cc	CAS-I,CAS-III			
115140..115521	array		QRUJ01000006.1	[Eubacterium] rectale	Bacteria	Firmicutes	Clostridia	Clostridiales	Lachnospiraceae			CRISPR array			6	39	31

84 CAS-VI-A | WP_090127495.1

102556..102791	array		NZ_FCNR01000048.1	Eubacteriaceae bacterium CHKCI004	Bacteria	Firmicutes	Clostridia	Clostridiales	Eubacteriaceae			CRISPR array			4	38	30
102881..103142	+	WP_090127494.	NZ_FCNR01000048.1	Eubacteriaceae bacterium CHKCI004	Bacteria	Firmicutes	Clostridia	Clostridiales	Eubacteriaceae		CRISPR-associated endonuclease Cas2	cas2	cd09725	CAS-I,CAS-II,CAS-III,CAS-V			
103162..105070	+	WP_090127495.	NZ_FCNR01000048.1	Eubacteriaceae bacterium CHKCI004	Bacteria	Firmicutes	Clostridia	Clostridiales	Eubacteriaceae		type I-B CRISPR-associated endonuclease Cas1	RT,cas1	pfam00078,cc	CAS-I,CAS-III			
105345..105568	array		NZ_FCNR01000048.1	Eubacteriaceae bacterium CHKCI004	Bacteria	Firmicutes	Clostridia	Clostridiales	Eubacteriaceae			CRISPR array			4	37	28
105978..110100	+	WP_090127496.	NZ_FCNR01000048.1	Eubacteriaceae bacterium CHKCI004	Bacteria	Firmicutes	Clostridia	Clostridiales	Eubacteriaceae		hypothetical protein	cas13a	cas13a	CAS-VI-A			

85 CAS-III-D | SCW73731.1

39808..40543	-	SCW73595.1	FMTN01000026.1	Lachnospiraceae bacterium C10	Bacteria	Firmicutes	Clostridia	Clostridiales	Lachnospiraceae		CRISPR-associated endoribonuclease Cas6	cas6	COG1583	CAS-I,CAS-III,CAS-IV			
40724..41087	+	SCW73608.1	FMTN01000026.1	Lachnospiraceae bacterium C10	Bacteria	Firmicutes	Clostridia	Clostridiales	Lachnospiraceae		hypothetical protein	csx20	cls000118	CAS-I,CAS-III			
41279..41975	+	SCW73621.1	FMTN01000026.1	Lachnospiraceae bacterium C10	Bacteria	Firmicutes	Clostridia	Clostridiales	Lachnospiraceae		hypothetical protein	C3a43	cluster43				
41993..43490	+	SCW73633.1	FMTN01000026.1	Lachnospiraceae bacterium C10	Bacteria	Firmicutes	Clostridia	Clostridiales	Lachnospiraceae		CRISPR-associated (Cas) DxTHG family protein	CARF	icity0119				
43511..45170	+	SCW73646.1	FMTN01000026.1	Lachnospiraceae bacterium C10	Bacteria	Firmicutes	Clostridia	Clostridiales	Lachnospiraceae		hypothetical protein	cas10	mkCas0133	CAS-III			
45157..47359	+	SCW73658.1	FMTN01000026.1	Lachnospiraceae bacterium C10	Bacteria	Firmicutes	Clostridia	Clostridiales	Lachnospiraceae		CRISPR/Cas system CSM-associated protein Csm3, group 7 of RAMP superfamily	csm3gr7,csx10gr5	mkCas0198,cl	CAS-III-A,CAS-III-D			
47361..48687	+	SCW73674.1	FMTN01000026.1	Lachnospiraceae bacterium C10	Bacteria	Firmicutes	Clostridia	Clostridiales	Lachnospiraceae		CRISPR/Cas system CSM-associated protein Csm3, group 7 of RAMP superfamily	csm3gr7,csm3gr7	cd09726,cd0	CAS-III-D			
48683..49160	+	SCW73687.1	FMTN01000026.1	Lachnospiraceae bacterium C10	Bacteria	Firmicutes	Clostridia	Clostridiales	Lachnospiraceae		hypothetical protein	Cas11/Csx19	cluster5				
49273..51037	+	SCW73700.1	FMTN01000026.1	Lachnospiraceae bacterium C10	Bacteria	Firmicutes	Clostridia	Clostridiales	Lachnospiraceae		CRISPR-associated protein	csm3gr7,csm3gr7	cls000253,cls	CAS-III-A,CAS-III-D			
51085..51346	+	SCW73718.1	FMTN01000026.1	Lachnospiraceae bacterium C10	Bacteria	Firmicutes	Clostridia	Clostridiales	Lachnospiraceae		CRISPR-associated protein Cas2	cas2	cd09725	CAS-I,CAS-II,CAS-III,CAS-V			
51326..53252	+	SCW73731.1	FMTN01000026.1	Lachnospiraceae bacterium C10	Bacteria	Firmicutes	Clostridia	Clostridiales	Lachnospiraceae		CRISPR-associated endonuclease Cas1	RT,cas1	pfam00078,cc	CAS-I,CAS-III			
53564..54087	array		FMTN01000026.1	Lachnospiraceae bacterium C10	Bacteria	Firmicutes	Clostridia	Clostridiales	Lachnospiraceae			CRISPR array			8	41	29

86 CAS-III-D | EFU76454.1

1666272..1666511	array		GL622296.1	Lachnoanaerobaculum saburreum DSM 3986	Bacteria	Firmicutes	Clostridia	Clostridiales	Lachnospiraceae	Lachnoanaerobaculum		CRISPR array			4	38	31
1667102..1667453	+	EFU76493.1	GL622296.1	Lachnoanaerobaculum saburreum DSM 3986	Bacteria	Firmicutes	Clostridia	Clostridiales	Lachnospiraceae	Lachnoanaerobaculum	CRISPR-associated DxTHG motif protein	csx1	cd09732	CAS-III			
1667430..1668009	+	EFU76494.1	GL622296.1	Lachnoanaerobaculum saburreum DSM 3986	Bacteria	Firmicutes	Clostridia	Clostridiales	Lachnospiraceae	Lachnoanaerobaculum	hypothetical protein	Csx1	cluster2				
1669598..1671155	+	EFU76445.1	GL622296.1	Lachnoanaerobaculum saburreum DSM 3986	Bacteria	Firmicutes	Clostridia	Clostridiales	Lachnospiraceae	Lachnoanaerobaculum	hypothetical protein	csx1	cd09732	CAS-III			
1671183..1672914	+	EFU76446.1	GL622296.1	Lachnoanaerobaculum saburreum DSM 3986	Bacteria	Firmicutes	Clostridia	Clostridiales	Lachnospiraceae	Lachnoanaerobaculum	hypothetical protein	cas10	mkCas0133	CAS-III			
1672861..1675195	+	EFU76447.1	GL622296.1	Lachnoanaerobaculum saburreum DSM 3986	Bacteria	Firmicutes	Clostridia	Clostridiales	Lachnospiraceae	Lachnoanaerobaculum	CRISPR-associated RAMP protein	csm3gr7,csx10gr5	mkCas0198,cl	CAS-III-A,CAS-III-D			
1675208..1676684	+	EFU76448.1	GL622296.1	Lachnoanaerobaculum saburreum DSM 3986	Bacteria	Firmicutes	Clostridia	Clostridiales	Lachnospiraceae	Lachnoanaerobaculum	CRISPR-associated RAMP protein	csm3gr7,csm3gr7	cd09726,cd0	CAS-III-D			
1676658..1677195	+	EFU76449.1	GL622296.1	Lachnoanaerobaculum saburreum DSM 3986	Bacteria	Firmicutes	Clostridia	Clostridiales	Lachnospiraceae	Lachnoanaerobaculum	putative CRISPR-associated protein, TIGR03984 family	csx19	cls001376	CAS-III-D			
1677197..1679108	+	EFU76450.1	GL622296.1	Lachnoanaerobaculum saburreum DSM 3986	Bacteria	Firmicutes	Clostridia	Clostridiales	Lachnospiraceae	Lachnoanaerobaculum	CRISPR-associated protein	csm3gr7	cls000253	CAS-III-A,CAS-III-D			
1679200..1680706	+	EFU76451.1	GL622296.1	Lachnoanaerobaculum saburreum DSM 3986	Bacteria	Firmicutes	Clostridia	Clostridiales	Lachnospiraceae	Lachnoanaerobaculum	hypothetical protein	csx1	pfam09455	CAS-III			
1680719..1681025	+	EFU76452.1	GL622296.1	Lachnoanaerobaculum saburreum DSM 3986	Bacteria	Firmicutes	Clostridia	Clostridiales	Lachnospiraceae	Lachnoanaerobaculum	addiction module antitoxin, RelB/DinJ family						
1681122..1681997	array		GL622296.1	Lachnoanaerobaculum saburreum DSM 3986	Bacteria	Firmicutes	Clostridia	Clostridiales	Lachnospiraceae	Lachnoanaerobaculum		CRISPR array			13	40	30
1681899..1682019	+	EFU76453.1	GL622296.1	Lachnoanaerobaculum saburreum DSM 3986	Bacteria	Firmicutes	Clostridia	Clostridiales	Lachnospiraceae	Lachnoanaerobaculum	hypothetical protein						
1682448..1684368	-	EFU76454.1	GL622296.1	Lachnoanaerobaculum saburreum DSM 3986	Bacteria	Firmicutes	Clostridia	Clostridiales	Lachnospiraceae	Lachnoanaerobaculum	CRISPR-associated endonuclease Cas1	RT,cas1	pfam00078,cc	CAS-I,CAS-III			
1684376..1684637	-	EFU76455.1	GL622296.1	Lachnoanaerobaculum saburreum DSM 3986	Bacteria	Firmicutes	Clostridia	Clostridiales	Lachnospiraceae	Lachnoanaerobaculum	CRISPR-associated protein Cas2	cas2	cd09725	CAS-I,CAS-II,CAS-III,CAS-V			
1684689..1685706	array		GL622296.1	Lachnoanaerobaculum saburreum DSM 3986	Bacteria	Firmicutes	Clostridia	Clostridiales	Lachnospiraceae	Lachnoanaerobaculum		CRISPR array			15	40	30

87 CAS-III-D | AYA99695.1

1537755..1538541	array		CP032364.1	Lachnoanaerobaculum umeaense	Bacteria	Firmicutes	Clostridia	Clostridiales	Lachnospiraceae	Lachnoanaerobaculum		CRISPR array			12	38	30
1538605..1538866	+	AYA99694.1	CP032364.1	Lachnoanaerobaculum umeaense	Bacteria	Firmicutes	Clostridia	Clostridiales	Lachnospiraceae	Lachnoanaerobaculum	CRISPR-associated endonuclease Cas2	cas2	cd09725	CAS-I,CAS-II,CAS-III,CAS-V			
1538874..1540794	+	AYA99695.1	CP032364.1	Lachnoanaerobaculum umeaense	Bacteria	Firmicutes	Clostridia	Clostridiales	Lachnospiraceae	Lachnoanaerobaculum	type I-B CRISPR-associated endonuclease Cas1	RT,cas1	pfam00078,cc	CAS-I,CAS-III			
1540907..1542413	-	AYB00826.1	CP032364.1	Lachnoanaerobaculum umeaense	Bacteria	Firmicutes	Clostridia	Clostridiales	Lachnospiraceae	Lachnoanaerobaculum	hypothetical protein	csx1	pfam09455	CAS-III			
1544091..1545666	-	AYA99696.1	CP032364.1	Lachnoanaerobaculum umeaense	Bacteria	Firmicutes	Clostridia	Clostridiales	Lachnospiraceae	Lachnoanaerobaculum	IS1182 family transposase						
1545736..1547779	array		CP032364.1	Lachnoanaerobaculum umeaense	Bacteria	Firmicutes	Clostridia	Clostridiales	Lachnospiraceae	Lachnoanaerobaculum		CRISPR array			29	41	30
1547877..1548186	-	AYA99697.1	CP032364.1	Lachnoanaerobaculum umeaense	Bacteria	Firmicutes	Clostridia	Clostridiales	Lachnospiraceae	Lachnoanaerobaculum	type II toxin-antitoxin system RelB/DinJ family antitoxin						
1548739..1550749	-	AYA99698.1	CP032364.1	Lachnoanaerobaculum umeaense	Bacteria	Firmicutes	Clostridia	Clostridiales	Lachnospiraceae	Lachnoanaerobaculum	TIGR03986 family CRISPR-associated RAMP protein	csm3gr7	cls000253	CAS-III-A,CAS-III-D			
1550751..1551288	-	AYA99699.1	CP032364.1	Lachnoanaerobaculum umeaense	Bacteria	Firmicutes	Clostridia	Clostridiales	Lachnospiraceae	Lachnoanaerobaculum	hypothetical protein	csx19,csx19	mkCas0132,cl	CAS-III-D			
1551268..1552786	-	AYA99700.1	CP032364.1	Lachnoanaerobaculum umeaense	Bacteria	Firmicutes	Clostridia	Clostridiales	Lachnospiraceae	Lachnoanaerobaculum	CRISPR-associated protein	csm3gr7,csm3gr7	cd09726,cd0	CAS-III-D			
1552799..1555268	-	AYA99701.1	CP032364.1	Lachnoanaerobaculum umeaense	Bacteria	Firmicutes	Clostridia	Clostridiales	Lachnospiraceae	Lachnoanaerobaculum	CRISPR-associated protein	csm3gr7,csx10gr5	mkCas0198,cl	CAS-III-A,CAS-III-D			
1555215..1556952	-	AYA99702.1	CP032364.1	Lachnoanaerobaculum umeaense	Bacteria	Firmicutes	Clostridia	Clostridiales	Lachnospiraceae	Lachnoanaerobaculum	hypothetical protein	cas10	mkCas0133	CAS-III			
1557006..1557363	-	AYA99703.1	CP032364.1	Lachnoanaerobaculum umeaense	Bacteria	Firmicutes	Clostridia	Clostridiales	Lachnospiraceae	Lachnoanaerobaculum	hypothetical protein	csx20	cls000118	CAS-I,CAS-III			
1558094..1559135	array		CP032364.1	Lachnoanaerobaculum umeaense	Bacteria	Firmicutes	Clostridia	Clostridiales	Lachnospiraceae	Lachnoanaerobaculum		CRISPR array			15	42	30
1559308..1561063	-	AYA99704.1	CP032364.1	Lachnoanaerobaculum umeaense	Bacteria	Firmicutes	Clostridia	Clostridiales	Lachnospiraceae	Lachnoanaerobaculum	IS1182 family transposase						
1561250..1562756	-	AYB00827.1	CP032364.1	Lachnoanaerobaculum umeaense	Bacteria	Firmicutes	Clostridia	Clostridiales	Lachnospiraceae	Lachnoanaerobaculum	hypothetical protein	csx1	pfam09455	CAS-III			

88 partial | ETO97675.1

356..2012	-	ETO97675.1	AZXX01000055.1	Lachnoanaerobaculum sp. MSX33	Bacteria	Firmicutes	Clostridia	Clostridiales	Lachnospiraceae	Lachnoanaerobaculum	CRISPR-associated endonuclease Cas1	RT,cas1	pfam00078,cc	CAS-I,CAS-III			

89 CAS-III-D | EHO48118.1

27239..27747	array		JH591179.1	Lachnospiraceae bacterium oral taxon 082 str. F0431	Bacteria	Firmicutes	Clostridia	Clostridiales	Lachnospiraceae			CRISPR array			8	38	30

27476..27713	+	EHO48121.1	JH591179.1	Lachnospiraceae bacterium oral taxon 082 str. F0431	Bacteria	Firmicutes	Clostridia	Clostridiales	Lachnospiraceae		hypothetical protein						
27793..27919	+	EHO48116.1	JH591179.1	Lachnospiraceae bacterium oral taxon 082 str. F0431	Bacteria	Firmicutes	Clostridia	Clostridiales	Lachnospiraceae		hypothetical protein						
27896..28157	+	EHO48117.1	JH591179.1	Lachnospiraceae bacterium oral taxon 082 str. F0431	Bacteria	Firmicutes	Clostridia	Clostridiales	Lachnospiraceae		CRISPR-associated protein Cas2	cas2	cd09725	CAS-I,CAS-II,CAS-III,CAS-V			
28165..30085	+	EHO48118.1	JH591179.1	Lachnospiraceae bacterium oral taxon 082 str. F0431	Bacteria	Firmicutes	Clostridia	Clostridiales	Lachnospiraceae		CRISPR-associated endonuclease Cas1	**RT,cas1**	pfam00078,cc	CAS-I,CAS-III			
30809..31115	-	EHO48074.1	JH591179.1	Lachnospiraceae bacterium oral taxon 082 str. F0431	Bacteria	Firmicutes	Clostridia	Clostridiales	Lachnospiraceae		addiction module antitoxin, RelB/DinJ family						
31128..32673	-	EHO48075.1	JH591179.1	Lachnospiraceae bacterium oral taxon 082 str. F0431	Bacteria	Firmicutes	Clostridia	Clostridiales	Lachnospiraceae		hypothetical protein	csx1	pfam09455	CAS-III			
32726..34625	-	EHO48076.1	JH591179.1	Lachnospiraceae bacterium oral taxon 082 str. F0431	Bacteria	Firmicutes	Clostridia	Clostridiales	Lachnospiraceae		CRISPR-associated protein	csm3gr7	cls000253	CAS-III-A,CAS-III-D			
34614..35163	-	EHO48077.1	JH591179.1	Lachnospiraceae bacterium oral taxon 082 str. F0431	Bacteria	Firmicutes	Clostridia	Clostridiales	Lachnospiraceae		hypothetical protein	csx19	cls001376	CAS-III-D			
35137..36613	-	EHO48078.1	JH591179.1	Lachnospiraceae bacterium oral taxon 082 str. F0431	Bacteria	Firmicutes	Clostridia	Clostridiales	Lachnospiraceae		CRISPR-associated RAMP protein	csm3gr7,csm3gr7	cd09726,cd0§	CAS-III-D			
36626..38960	-	EHO48079.1	JH591179.1	Lachnospiraceae bacterium oral taxon 082 str. F0431	Bacteria	Firmicutes	Clostridia	Clostridiales	Lachnospiraceae		CRISPR-associated RAMP protein	csm3gr7,csx10gr5	mkCas0198,cl	CAS-III-A,CAS-III-D			
38907..40644	-	EHO48080.1	JH591179.1	Lachnospiraceae bacterium oral taxon 082 str. F0431	Bacteria	Firmicutes	Clostridia	Clostridiales	Lachnospiraceae		hypothetical protein	cas10	mkCas0133	CAS-III			
40672..42286	-	EHO48081.1	JH591179.1	Lachnospiraceae bacterium oral taxon 082 str. F0431	Bacteria	Firmicutes	Clostridia	Clostridiales	Lachnospiraceae		macrophage scavenger receptor	CARF	icity0119				
42336..43077	-	EHO48082.1	JH591179.1	Lachnospiraceae bacterium oral taxon 082 str. F0431	Bacteria	Firmicutes	Clostridia	Clostridiales	Lachnospiraceae		CRISPR-associated endoribonuclease Cas6	cas6	COG1583	CAS-I,CAS-III,CAS-IV			
43103..43460	-	EHO48083.1	JH591179.1	Lachnospiraceae bacterium oral taxon 082 str. F0431	Bacteria	Firmicutes	Clostridia	Clostridiales	Lachnospiraceae		hypothetical protein	csx20	cls000118	CAS-I,CAS-III			

90 CAS-III-D | KXB53086.1

219899..220210	array		KQ959848.1	Lachnoanaerobaculum saburreum	Bacteria	Firmicutes	Clostridia	Clostridiales	Lachnospiraceae	Lachnoanaerobaculum		CRISPR array			5	40	30
220317..220701	array		KQ959848.1	Lachnoanaerobaculum saburreum	Bacteria	Firmicutes	Clostridia	Clostridiales	Lachnospiraceae	Lachnoanaerobaculum		CRISPR array			6	39	31
221536..221893	+	KXB53076.1	KQ959848.1	Lachnoanaerobaculum saburreum	Bacteria	Firmicutes	Clostridia	Clostridiales	Lachnospiraceae	Lachnoanaerobaculum	hypothetical protein	csx20	cls000118	CAS-I,CAS-III			
221919..222672	+	KXB53077.1	KQ959848.1	Lachnoanaerobaculum saburreum	Bacteria	Firmicutes	Clostridia	Clostridiales	Lachnospiraceae	Lachnoanaerobaculum	CRISPR-associated endoribonuclease Cas6	cas6	COG1583	CAS-I,CAS-III,CAS-IV			
222713..224258	+	KXB53078.1	KQ959848.1	Lachnoanaerobaculum saburreum	Bacteria	Firmicutes	Clostridia	Clostridiales	Lachnospiraceae	Lachnoanaerobaculum	hypothetical protein	csx1	cd09732	CAS-III			
224297..226022	+	KXB53079.1	KQ959848.1	Lachnoanaerobaculum saburreum	Bacteria	Firmicutes	Clostridia	Clostridiales	Lachnospiraceae	Lachnoanaerobaculum	hypothetical protein	cas10	mkCas0133	CAS-III			
225963..228459	+	KXB53080.1	KQ959848.1	Lachnoanaerobaculum saburreum	Bacteria	Firmicutes	Clostridia	Clostridiales	Lachnospiraceae	Lachnoanaerobaculum	CRISPR-associated RAMP protein	csm3gr7,csx10gr5	mkCas0198,cc	CAS-III-A,CAS-III-D			
228472..229990	+	KXB53081.1	KQ959848.1	Lachnoanaerobaculum saburreum	Bacteria	Firmicutes	Clostridia	Clostridiales	Lachnospiraceae	Lachnoanaerobaculum	CRISPR-associated RAMP protein	csm3gr7,csm3gr7	cd09726,cd0§	CAS-III-D			
229961..230495	+	KXB53082.1	KQ959848.1	Lachnoanaerobaculum saburreum	Bacteria	Firmicutes	Clostridia	Clostridiales	Lachnospiraceae	Lachnoanaerobaculum	CRISPR-associated protein family	csx19	cls001376	CAS-III-D			
230497..232495	+	KXB53083.1	KQ959848.1	Lachnoanaerobaculum saburreum	Bacteria	Firmicutes	Clostridia	Clostridiales	Lachnospiraceae	Lachnoanaerobaculum	CRISPR-associated protein	csm3gr7	cls000253	CAS-III-A,CAS-III-D			
232581..234087	+	KXB53084.1	KQ959848.1	Lachnoanaerobaculum saburreum	Bacteria	Firmicutes	Clostridia	Clostridiales	Lachnospiraceae	Lachnoanaerobaculum	hypothetical protein	csx1	pfam09455	CAS-III			
234100..234406	+	KXB53085.1	KQ959848.1	Lachnoanaerobaculum saburreum	Bacteria	Firmicutes	Clostridia	Clostridiales	Lachnospiraceae	Lachnoanaerobaculum	addiction module antitoxin, RelB/DinJ family						
234502..234998	array		KQ959848.1	Lachnoanaerobaculum saburreum	Bacteria	Firmicutes	Clostridia	Clostridiales	Lachnospiraceae	Lachnoanaerobaculum		CRISPR array			8	36	30
235460..237380	-	KXB53086.1	KQ959848.1	Lachnoanaerobaculum saburreum	Bacteria	Firmicutes	Clostridia	Clostridiales	Lachnospiraceae	Lachnoanaerobaculum	CRISPR-associated endonuclease Cas1	**RT,cas1**	pfam00078,cc	CAS-I,CAS-III			
237388..237649	-	KXB53087.1	KQ959848.1	Lachnoanaerobaculum saburreum	Bacteria	Firmicutes	Clostridia	Clostridiales	Lachnospiraceae	Lachnoanaerobaculum	CRISPR-associated protein Cas2	cas2	cd09725	CAS-I,CAS-II,CAS-III,CAS-V			
237698..238029	array		KQ959848.1	Lachnoanaerobaculum saburreum	Bacteria	Firmicutes	Clostridia	Clostridiales	Lachnospiraceae	Lachnoanaerobaculum		CRISPR array			5	45	30

91 CAS-VI-A | SFB66471.1

6257..10289	+	SFB66467.1	FOKR01000002.1	Butyrivibrio sp. YAB3001	Bacteria	Firmicutes	Clostridia	Clostridiales	Lachnospiraceae	Butyrivibrio	hypothetical protein	cas13a	cas13a	CAS-VI-A			
10440..11302	array		FOKR01000002.1	Butyrivibrio sp. YAB3001	Bacteria	Firmicutes	Clostridia	Clostridiales	Lachnospiraceae	Butyrivibrio		CRISPR array			13	32	36
11477..12524	+	SFB66471.1	FOKR01000002.1	Butyrivibrio sp. YAB3001	Bacteria	Firmicutes	Clostridia	Clostridiales	Lachnospiraceae	Butyrivibrio	CRISP-associated protein Cas1	**cas1***	cd09722	CAS-I-B			
12537..12798	+	SFB66474.1	FOKR01000002.1	Butyrivibrio sp. YAB3001	Bacteria	Firmicutes	Clostridia	Clostridiales	Lachnospiraceae	Butyrivibrio	CRISPR-associated protein Cas2	cas2	cd09725	CAS-I,CAS-II,CAS-III,CAS-V			

93 CAS-III-D | OLA06495.1

40490..41231	-	OLA06483.1	MNSI01000033.1	Eubacterium sp. 38_16	Bacteria	Firmicutes	Clostridia	Clostridiales	Eubacteriaceae	Eubacterium	CRISPR-associated endoribonuclease Cas6	cas6	COG1583	CAS-I,CAS-III,CAS-IV			
41351..41621	+	OLA06484.1	MNSI01000033.1	Eubacterium sp. 38_16	Bacteria	Firmicutes	Clostridia	Clostridiales	Eubacteriaceae	Eubacterium	hypothetical protein	cas10	mkCas0133	CAS-III			
41655..43056	+	OLA06485.1	MNSI01000033.1	Eubacterium sp. 38_16	Bacteria	Firmicutes	Clostridia	Clostridiales	Eubacteriaceae	Eubacterium	hypothetical protein	cas10	mkCas0133	CAS-III			
43036..45400	+	OLA06486.1	MNSI01000033.1	Eubacterium sp. 38_16	Bacteria	Firmicutes	Clostridia	Clostridiales	Eubacteriaceae	Eubacterium	hypothetical protein	csm3gr7,csx10gr5	mkCas0198,cl	CAS-III-A,CAS-III-D			
45402..46752	+	OLA06487.1	MNSI01000033.1	Eubacterium sp. 38_16	Bacteria	Firmicutes	Clostridia	Clostridiales	Eubacteriaceae	Eubacterium	hypothetical protein	csm3gr7,csm3gr7,csm3gr7	cd09726,COG	CAS-III-D			
46751..47255	+	OLA06488.1	MNSI01000033.1	Eubacterium sp. 38_16	Bacteria	Firmicutes	Clostridia	Clostridiales	Eubacteriaceae	Eubacterium	hypothetical protein	csx19,csx19	mkCas0132,cl	CAS-III-D			
47267..49250	+	OLA06489.1	MNSI01000033.1	Eubacterium sp. 38_16	Bacteria	Firmicutes	Clostridia	Clostridiales	Eubacteriaceae	Eubacterium	CRISPR-associated RAMP family protein	csm3gr7	cls000253	CAS-III-A,CAS-III-D			
49640..50273	array		MNSI01000033.1	Eubacterium sp. 38_16	Bacteria	Firmicutes	Clostridia	Clostridiales	Eubacteriaceae	Eubacterium		CRISPR array			10	38	29
50353..51294	array		MNSI01000033.1	Eubacterium sp. 38_16	Bacteria	Firmicutes	Clostridia	Clostridiales	Eubacteriaceae	Eubacterium		CRISPR array			14	41	29
51362..53273	+	OLA06495.1	MNSI01000033.1	Eubacterium sp. 38_16	Bacteria	Firmicutes	Clostridia	Clostridiales	Eubacteriaceae	Eubacterium	CRISPR-associated endonuclease Cas1	**RT,cas1**	pfam00078,cc	CAS-I,CAS-III			
53299..53560	+	OLA06490.1	MNSI01000033.1	Eubacterium sp. 38_16	Bacteria	Firmicutes	Clostridia	Clostridiales	Eubacteriaceae	Eubacterium	CRISPR-associated endonuclease Cas2	cas2	cd09725	CAS-I,CAS-II,CAS-III,CAS-V			
53556..53919	+	OLA06491.1	MNSI01000033.1	Eubacterium sp. 38_16	Bacteria	Firmicutes	Clostridia	Clostridiales	Eubacteriaceae	Eubacterium	hypothetical protein	csx20	cls000118	CAS-I,CAS-III			

94 CAS-III-D | SOB72962.1

2457296..2458985	-	SOB72959.1	LT907978.1	Anaerobutyricum hallii	Bacteria	Firmicutes	Clostridia	Clostridiales	Lachnospiraceae	Anaerobutyricum	CRISPR-associated (Cas) DxTHG family	csx1	pfam09455	CAS-III			
2459007..2459370	-	SOB72960.1	LT907978.1	Anaerobutyricum hallii	Bacteria	Firmicutes	Clostridia	Clostridiales	Lachnospiraceae	Anaerobutyricum	Hypothetical protein	csx20	cls000118	CAS-I,CAS-III			
2459366..2459627	-	SOB72961.1	LT907978.1	Anaerobutyricum hallii	Bacteria	Firmicutes	Clostridia	Clostridiales	Lachnospiraceae	Anaerobutyricum	CRISPR-associated endoribonuclease Cas2 [cas2].	cas2	cd09725	CAS-I,CAS-II,CAS-III,CAS-V			
2459653..2461582	-	SOB72962.1	LT907978.1	Anaerobutyricum hallii	Bacteria	Firmicutes	Clostridia	Clostridiales	Lachnospiraceae	Anaerobutyricum	cas1: CRISPR-associated endonuclease Cas1	**RT,cas1**	pfam00078,cc	CAS-I,CAS-III			
2461821..2462679	-	SOB72963.1	LT907978.1	Anaerobutyricum hallii	Bacteria	Firmicutes	Clostridia	Clostridiales	Lachnospiraceae	Anaerobutyricum	Integrase, catalytic core						
2462629..2462977	-	SOB72964.1	LT907978.1	Anaerobutyricum hallii	Bacteria	Firmicutes	Clostridia	Clostridiales	Lachnospiraceae	Anaerobutyricum	Transposase						
2463065..2465456	array		LT907978.1	Anaerobutyricum hallii	Bacteria	Firmicutes	Clostridia	Clostridiales	Lachnospiraceae	Anaerobutyricum		CRISPR array			36	38	29
2465846..2467769	-	SOB72965.1	LT907978.1	Anaerobutyricum hallii	Bacteria	Firmicutes	Clostridia	Clostridiales	Lachnospiraceae	Anaerobutyricum	CRISPR type III-associated RAMP protein	csm3gr7	cls000253	CAS-III-A,CAS-III-D			
2467781..2468285	-	SOB72966.1	LT907978.1	Anaerobutyricum hallii	Bacteria	Firmicutes	Clostridia	Clostridiales	Lachnospiraceae	Anaerobutyricum	Hypothetical protein	csx19	cls001376	CAS-III-D			
2468284..2469634	-	SOB72967.1	LT907978.1	Anaerobutyricum hallii	Bacteria	Firmicutes	Clostridia	Clostridiales	Lachnospiraceae	Anaerobutyricum	CRISPR type III-associated RAMP protein	csm3gr7,csm3gr7,csm3gr7	cd09726,COG	CAS-III-D			
2469636..2472000	-	SOB72968.1	LT907978.1	Anaerobutyricum hallii	Bacteria	Firmicutes	Clostridia	Clostridiales	Lachnospiraceae	Anaerobutyricum	RAMP_I_III	csm3gr7,csx10gr5	mkCas0198,cl	CAS-III-A,CAS-III-D			
2471980..2473681	-	SOB72969.1	LT907978.1	Anaerobutyricum hallii	Bacteria	Firmicutes	Clostridia	Clostridiales	Lachnospiraceae	Anaerobutyricum	Hypothetical protein	cas10	mkCas0133	CAS-III			
2473801..2474542	+	SOB72970.1	LT907978.1	Anaerobutyricum hallii	Bacteria	Firmicutes	Clostridia	Clostridiales	Lachnospiraceae	Anaerobutyricum	CRISPR associated protein Cas6	cas6	COG1583	CAS-I,CAS-III,CAS-IV			

96 partial | EEG94369.1

134..395	-	EEG94368.1	ACFY01000067.1	Roseburia inulinivorans DSM 16841	Bacteria	Firmicutes	Clostridia	Clostridiales	Lachnospiraceae	Roseburia	CRISPR-associated protein Cas2	cas2	cd09725	CAS-I,CAS-II,CAS-III,CAS-V			
403..1483	-	EEG94369.1	ACFY01000067.1	Roseburia inulinivorans DSM 16841	Bacteria	Firmicutes	Clostridia	Clostridiales	Lachnospiraceae	Roseburia	CRISPR-associated endonuclease Cas1	**cas1**	cd09722	CAS-I-B			
1483..2305	-	EEG94370.1	ACFY01000067.1	Roseburia inulinivorans DSM 16841	Bacteria	Firmicutes	Clostridia	Clostridiales	Lachnospiraceae	Roseburia	reverse transcriptase (RNA-dependent DNA polymerase)	RT	pfam00078	CAS-I,CAS-III			
2333..2633	array		ACFY01000067.1	Roseburia inulinivorans DSM 16841	Bacteria	Firmicutes	Clostridia	Clostridiales	Lachnospiraceae	Roseburia		CRISPR array			5	42	26

97 partial | CRL43259.1

296..557	-	CRL43258.1	CVRS01000124.1	Roseburia inulinivorans	Bacteria	Firmicutes	Clostridia	Clostridiales	Lachnospiraceae	Roseburia	hypothetical protein	cas2	cd09725	CAS-I,CAS-II,CAS-III,CAS-V			
565..2467	-	CRL43259.1	CVRS01000124.1	Roseburia inulinivorans	Bacteria	Firmicutes	Clostridia	Clostridiales	Lachnospiraceae	Roseburia	hypothetical protein	**RT,cas1**	pfam00078,cc	CAS-I,CAS-III			
2495..3005	array		CVRS01000124.1	Roseburia inulinivorans	Bacteria	Firmicutes	Clostridia	Clostridiales	Lachnospiraceae	Roseburia		CRISPR array			8	39	29

98 CAS-III-D | RGX90666.1

728..1509	array		QSCK01000038.1	Roseburia sp. OF03-24	Bacteria	Firmicutes	Clostridia	Clostridiales	Lachnospiraceae	Roseburia		CRISPR array			12	39	29
1576..2374	+	RGX90665.1	QSCK01000038.1	Roseburia sp. OF03-24	Bacteria	Firmicutes	Clostridia	Clostridiales	Lachnospiraceae	Roseburia	Retron-type reverse transcriptase	RT	pfam00078	CAS-I,CAS-III			
2289..3483	+	RGX90666.1	QSCK01000038.1	Roseburia sp. OF03-24	Bacteria	Firmicutes	Clostridia	Clostridiales	Lachnospiraceae	Roseburia	CRISPR-associated endonuclease Cas1	**cas1**	cd09634	CAS-I,CAS-II,CAS-III,CAS-V			
3841..4102	+	RGX90667.1	QSCK01000038.1	Roseburia sp. OF03-24	Bacteria	Firmicutes	Clostridia	Clostridiales	Lachnospiraceae	Roseburia	CRISPR-associated endonuclease Cas2	cas2	cd09725	CAS-I,CAS-II,CAS-III,CAS-V			
4121..4484	+	RGX90668.1	QSCK01000038.1	Roseburia sp. OF03-24	Bacteria	Firmicutes	Clostridia	Clostridiales	Lachnospiraceae	Roseburia	hypothetical protein	csx20	cls000118	CAS-I,CAS-III			
4495..5902	+	RGX90669.1	QSCK01000038.1	Roseburia sp. OF03-24	Bacteria	Firmicutes	Clostridia	Clostridiales	Lachnospiraceae	Roseburia	hypothetical protein	csx1,csx1	pfam09623,cc	CAS-III			
5925..7458	+	RGX90670.1	QSCK01000038.1	Roseburia sp. OF03-24	Bacteria	Firmicutes	Clostridia	Clostridiales	Lachnospiraceae	Roseburia	hypothetical protein	cas10	mkCas0133	CAS-III			
7423..9541	+	RGX90671.1	QSCK01000038.1	Roseburia sp. OF03-24	Bacteria	Firmicutes	Clostridia	Clostridiales	Lachnospiraceae	Roseburia	hypothetical protein	csm3gr7,csx10gr5	mkCas0198,cl	CAS-III-A,CAS-III-D			
9537..10845	+	RGX90672.1	QSCK01000038.1	Roseburia sp. OF03-24	Bacteria	Firmicutes	Clostridia	Clostridiales	Lachnospiraceae	Roseburia	hypothetical protein	csm3gr7,csm3gr7	cd09726,cd0§	CAS-III-D			
10841..11243	+	RGX90673.1	QSCK01000038.1	Roseburia sp. OF03-24	Bacteria	Firmicutes	Clostridia	Clostridiales	Lachnospiraceae	Roseburia	hypothetical protein	Csx19	icity0015				
11245..13129	+	RGX90674.1	QSCK01000038.1	Roseburia sp. OF03-24	Bacteria	Firmicutes	Clostridia	Clostridiales	Lachnospiraceae	Roseburia	TIGR03986 family CRISPR-associated RAMP protein	csm3gr7	cls000253	CAS-III-A,CAS-III-D			
13131..13854	+	RGX90675.1	QSCK01000038.1	Roseburia sp. OF03-24	Bacteria	Firmicutes	Clostridia	Clostridiales	Lachnospiraceae	Roseburia	CRISPR-associated endoribonuclease Cas6	cas6	COG1583	CAS-I,CAS-III,CAS-IV			
13866..14928	-	RGX90676.1	QSCK01000038.1	Roseburia sp. OF03-24	Bacteria	Firmicutes	Clostridia	Clostridiales	Lachnospiraceae	Roseburia	WYL domain-containing protein	WYL	pfam13280	CAS-I,CAS-III			

Table S4: Computational analysis of the neighborhood of cas2 sequences used in Figure 2. Type VI-A loci are indicated in grey background

Node	(Sub)type/Coordinates	Strand	Protein_id	Genome_partition	Genome_name	Domain	Phylum	Class	Order	Family	Genus	Description	cas_gene	profiles	subtype	repeats	avg_spacer_length	avg_repeat_length
1	CAS-I-B | SHJ82104.1																	
	409..2494	+	SHJ81929.1	FQZM01000068.1	Desulfofundulus thermosubterraneus DSM 16057	Bacteria	Firmicutes	Clostridia	Clostridiales	Peptococcaceae	Desulfofundulus	CRISPR-associated protein TM1802 (cas_TM1802)	cas8b1	mkCas0074	CAS-I-B			
	2516..3458	+	SHJ81962.1	FQZM01000068.1	Desulfofundulus thermosubterraneus DSM 16057	Bacteria	Firmicutes	Clostridia	Clostridiales	Peptococcaceae	Desulfofundulus	CRISPR-associated protein, Csh2 family	cas7b	pfam05107	CAS-I-B,CAS-I-C			
	3486..4221	+	SHJ81987.1	FQZM01000068.1	Desulfofundulus thermosubterraneus DSM 16057	Bacteria	Firmicutes	Clostridia	Clostridiales	Peptococcaceae	Desulfofundulus	CRISPR-associated protein Cas5h	cas5	cls001430	CAS-I-B			
	4208..6617	+	SHJ82020.1	FQZM01000068.1	Desulfofundulus thermosubterraneus DSM 16057	Bacteria	Firmicutes	Clostridia	Clostridiales	Peptococcaceae	Desulfofundulus	CRISPR-associated helicase Cas3/CRISPR-associated endonuclease Cas3-HD	cas3HD,cas3	cd09641,COG1203	CAS-I			
	6591..7122	+	SHJ82048.1	FQZM01000068.1	Desulfofundulus thermosubterraneus DSM 16057	Bacteria	Firmicutes	Clostridia	Clostridiales	Peptococcaceae	Desulfofundulus	CRISPR-associated exonuclease, Cas4 family	cas4	pfam01930	CAS-I			
	7159..8155	+	SHJ82076.1	FQZM01000068.1	Desulfofundulus thermosubterraneus DSM 16057	Bacteria	Firmicutes	Clostridia	Clostridiales	Peptococcaceae	Desulfofundulus	CRISPR-associated protein, Cas1 family	cas1	cd09722	CAS-I-B			
	8272..8536	+	SHJ82104.1	FQZM01000068.1	Desulfofundulus thermosubterraneus DSM 16057	Bacteria	Firmicutes	Clostridia	Clostridiales	Peptococcaceae	Desulfofundulus	CRISPR-associated protein Cas2	cas2	cd09725	CAS-I,CAS-II,CAS-III,CAS-V			
	8682..8975	array		FQZM01000068.1	Desulfofundulus thermosubterraneus DSM 16057	Bacteria	Firmicutes	Clostridia	Clostridiales	Peptococcaceae	Desulfofundulus		CRISPR array			5	36	30
2	CAS-I-B | SFM78002.1																	
	212..476	-	SFM78002.1	FOUU01000004.1	Thermodesulforhabdus norvegica	Bacteria	Proteobacteria	Deltaproteobacteria	Syntrophobacterales	Syntrophobacteraceae	Thermodesulforhabdus	CRISPR-associated protein, Cas2 family	cas2	cd09725	CAS-I,CAS-II,CAS-III,CAS-V			
	481..1474	-	SFM78028.1	FOUU01000004.1	Thermodesulforhabdus norvegica	Bacteria	Proteobacteria	Deltaproteobacteria	Syntrophobacterales	Syntrophobacteraceae	Thermodesulforhabdus	CRISP-associated protein Cas1	cas1	cd09722	CAS-I-B			
	1485..1998	-	SFM78050.1	FOUU01000004.1	Thermodesulforhabdus norvegica	Bacteria	Proteobacteria	Deltaproteobacteria	Syntrophobacterales	Syntrophobacteraceae	Thermodesulforhabdus	CRISPR-associated exonuclease, Cas4 family	cas4	pfam01930	CAS-I			
	2023..4303	-	SFM78071.1	FOUU01000004.1	Thermodesulforhabdus norvegica	Bacteria	Proteobacteria	Deltaproteobacteria	Syntrophobacterales	Syntrophobacteraceae	Thermodesulforhabdus	CRISPR-associated helicase, Cas3 family	cas3HD,cas3	cd09641,COG1203	CAS-I			
	4277..4979	-	SFM78093.1	FOUU01000004.1	Thermodesulforhabdus norvegica	Bacteria	Proteobacteria	Deltaproteobacteria	Syntrophobacterales	Syntrophobacteraceae	Thermodesulforhabdus	CRISPR-associated protein Cas5t	cas5	COG1688	CAS-I			
	5005..6067	-	SFM78110.1	FOUU01000004.1	Thermodesulforhabdus norvegica	Bacteria	Proteobacteria	Deltaproteobacteria	Syntrophobacterales	Syntrophobacteraceae	Thermodesulforhabdus	CRISPR-associated autoregulator, Cst2 family	cas7	cd09687	CAS-I-B			
	6082..7777	-	SFM78132.1	FOUU01000004.1	Thermodesulforhabdus norvegica	Bacteria	Proteobacteria	Deltaproteobacteria	Syntrophobacterales	Syntrophobacteraceae	Thermodesulforhabdus	CRISPR-associated protein Cst1	cas8b2,cas8b2	cls000029,cd09665	CAS-I-B			
3	CAS-I-B | ACV64952.1																	
	4450625..4450940	-	ACV64951.1	CP001720.1	Desulfofarcimen acetoxidans DSM 771	Bacteria	Firmicutes	Clostridia	Clostridiales	Peptococcaceae	Desulfofarcimen	transcriptional regulator, ArsR family	csa3	COG0640	CAS-I-A			
	4451140..4452435	array		CP001720.1	Desulfofarcimen acetoxidans DSM 771	Bacteria	Firmicutes	Clostridia	Clostridiales	Peptococcaceae	Desulfofarcimen		CRISPR array			20	36	30
	4452617..4452881	-	ACV64952.1	CP001720.1	Desulfofarcimen acetoxidans DSM 771	Bacteria	Firmicutes	Clostridia	Clostridiales	Peptococcaceae	Desulfofarcimen	CRISPR-associated protein Cas2	cas2	cd09725	CAS-I,CAS-II,CAS-III,CAS-V			
	4452914..4453907	-	ACV64953.1	CP001720.1	Desulfofarcimen acetoxidans DSM 771	Bacteria	Firmicutes	Clostridia	Clostridiales	Peptococcaceae	Desulfofarcimen	CRISPR-associated protein Cas1	cas1	cd09722	CAS-I-B			
	4453922..4454432	-	ACV64954.1	CP001720.1	Desulfofarcimen acetoxidans DSM 771	Bacteria	Firmicutes	Clostridia	Clostridiales	Peptococcaceae	Desulfofarcimen	CRISPR-associated protein Cas4	cas4	pfam01930	CAS-I			
	4454486..4456997	-	ACV64955.1	CP001720.1	Desulfofarcimen acetoxidans DSM 771	Bacteria	Firmicutes	Clostridia	Clostridiales	Peptococcaceae	Desulfofarcimen	CRISPR-associated helicase Cas3	cas3HD,cas3	cd09641,COG1203	CAS-I			
	4457065..4457827	-	ACV64956.1	CP001720.1	Desulfofarcimen acetoxidans DSM 771	Bacteria	Firmicutes	Clostridia	Clostridiales	Peptococcaceae	Desulfofarcimen	CRISPR-associated protein Cas5	cas5	cd09692	CAS-I-B			
	4457830..4458901	-	ACV64957.1	CP001720.1	Desulfofarcimen acetoxidans DSM 771	Bacteria	Firmicutes	Clostridia	Clostridiales	Peptococcaceae	Desulfofarcimen	CRISPR-associated protein, CT1132 family	cas7b	pfam05107	CAS-I-B,CAS-I-C			
	4458916..4460983	-	ACV64958.1	CP001720.1	Desulfofarcimen acetoxidans DSM 771	Bacteria	Firmicutes	Clostridia	Clostridiales	Peptococcaceae	Desulfofarcimen	hypothetical protein	cas8b1	pfam09484	CAS-I-B			
	4461310..4462045	-	ACV64959.1	CP001720.1	Desulfofarcimen acetoxidans DSM 771	Bacteria	Firmicutes	Clostridia	Clostridiales	Peptococcaceae	Desulfofarcimen	CRISPR-associated protein Cas6	cas6	COG1583	CAS-I,CAS-III,CAS-IV			
4	CAS-III-B | SDZ02948.1																	
	7574..8727	array		FNPV01000007.1	Tindallia californiensis	Bacteria	Firmicutes	Clostridia	Clostridiales	Clostridiaceae	Tindallia		CRISPR array			18	40	26
	8908..9157	-	SDZ02948.1	FNPV01000007.1	Tindallia californiensis	Bacteria	Firmicutes	Clostridia	Clostridiales	Clostridiaceae	Tindallia	CRISPR-associated protein, Cas2 family	cas2	cd09725	CAS-I,CAS-II,CAS-III,CAS-V			
	9289..10285	-	SDZ02971.1	FNPV01000007.1	Tindallia californiensis	Bacteria	Firmicutes	Clostridia	Clostridiales	Clostridiaceae	Tindallia	CRISP-associated protein Cas1	cas1	cd09722	CAS-I-B			
	10315..10816	-	SDZ02991.1	FNPV01000007.1	Tindallia californiensis	Bacteria	Firmicutes	Clostridia	Clostridiales	Clostridiaceae	Tindallia	CRISPR-associated exonuclease, Cas4 family	cas4	pfam01930	CAS-I			
	11212..11950	-	SDZ03007.1	FNPV01000007.1	Tindallia californiensis	Bacteria	Firmicutes	Clostridia	Clostridiales	Clostridiaceae	Tindallia	hypothetical protein	PD-DExK	mkCas0090	CAS-I,CAS-III			
	12137..13310	-	SDZ03030.1	FNPV01000007.1	Tindallia californiensis	Bacteria	Firmicutes	Clostridia	Clostridiales	Clostridiaceae	Tindallia	CRISPR-associated protein Cmr6	cmr6gr7	cd09661	CAS-III-B			
	13327..13702	-	SDZ03052.1	FNPV01000007.1	Tindallia californiensis	Bacteria	Firmicutes	Clostridia	Clostridiales	Clostridiaceae	Tindallia	CRISPR-associated protein Cmr5	cmr5gr11	pfam09701	CAS-III-B			
	13717..14626	-	SDZ03071.1	FNPV01000007.1	Tindallia californiensis	Bacteria	Firmicutes	Clostridia	Clostridiales	Clostridiaceae	Tindallia	CRISPR-associated protein Cmr4	cmr4gr7	COG1336	CAS-III-C			
	14670..15831	-	SDZ03093.1	FNPV01000007.1	Tindallia californiensis	Bacteria	Firmicutes	Clostridia	Clostridiales	Clostridiaceae	Tindallia	CRISPR-associated protein Cmr3	cmr3gr5	pfam09700	CAS-III-B			
	15830..17537	-	SDZ03115.1	FNPV01000007.1	Tindallia californiensis	Bacteria	Firmicutes	Clostridia	Clostridiales	Clostridiaceae	Tindallia	CRISPR-associated protein, Cmr2 family	cas10	cd09679	CAS-III			
	17533..18523	-	SDZ03136.1	FNPV01000007.1	Tindallia californiensis	Bacteria	Firmicutes	Clostridia	Clostridiales	Clostridiaceae	Tindallia	CRISPR type III-B/RAMP module RAMP protein Cmr1	cmr1gr7	COG1367	CAS-III-B			
	18542..19916	-	SDZ03152.1	FNPV01000007.1	Tindallia californiensis	Bacteria	Firmicutes	Clostridia	Clostridiales	Clostridiaceae	Tindallia	CRISPR-associated protein, TIGR02710 family	csx1	pfam09670	CAS-III			
	20439..20727	-	SDZ03171.1	FNPV01000007.1	Tindallia californiensis	Bacteria	Firmicutes	Clostridia	Clostridiales	Clostridiaceae	Tindallia	hypothetical protein						
5	CAS-I-B | SHI17228.1																	
	49309..50056	+	SHI17162.1	FQXR01000017.1	Sporanaerobacter acetigenes DSM 13106	Bacteria	Firmicutes	Tissierellia	Tissierellales		Sporanaerobacter	CRISPR-associated endoribonuclease Cas6	cas6	COG1583	CAS-I,CAS-III,CAS-IV			
	50070..51999	+	SHI17171.1	FQXR01000017.1	Sporanaerobacter acetigenes DSM 13106	Bacteria	Firmicutes	Tissierellia	Tissierellales		Sporanaerobacter	CRISPR-associated protein, Csh1 family	cas8b1	pfam09484	CAS-I-B			
	51988..52864	+	SHI17179.1	FQXR01000017.1	Sporanaerobacter acetigenes DSM 13106	Bacteria	Firmicutes	Tissierellia	Tissierellales		Sporanaerobacter	CRISPR-associated protein, Csh2 family	cas7b	pfam05107	CAS-I-B,CAS-I-C			
	52874..53591	+	SHI17190.1	FQXR01000017.1	Sporanaerobacter acetigenes DSM 13106	Bacteria	Firmicutes	Tissierellia	Tissierellales		Sporanaerobacter	CRISPR-associated protein, Cas5h family	cas5	cls001430	CAS-I-B			
	53606..55919	+	SHI17199.1	FQXR01000017.1	Sporanaerobacter acetigenes DSM 13106	Bacteria	Firmicutes	Tissierellia	Tissierellales		Sporanaerobacter	CRISPR-associated helicase, Cas3 family	cas3HD,cas3	cd09641,COG1203	CAS-I			
	55929..56439	+	SHI17209.1	FQXR01000017.1	Sporanaerobacter acetigenes DSM 13106	Bacteria	Firmicutes	Tissierellia	Tissierellales		Sporanaerobacter	CRISPR-associated exonuclease, Cas4 family	cas4	pfam01930	CAS-I			
	56450..57443	+	SHI17219.1	FQXR01000017.1	Sporanaerobacter acetigenes DSM 13106	Bacteria	Firmicutes	Tissierellia	Tissierellales		Sporanaerobacter	CRISP-associated protein Cas1	cas1	cd09722	CAS-I-B			
	57455..57719	+	SHI17228.1	FQXR01000017.1	Sporanaerobacter acetigenes DSM 13106	Bacteria	Firmicutes	Tissierellia	Tissierellales		Sporanaerobacter	CRISPR-associated protein, Cas2 family	cas2	cd09725	CAS-I,CAS-II,CAS-III,CAS-V			
	58021..58252	array		FQXR01000017.1	Sporanaerobacter acetigenes DSM 13106	Bacteria	Firmicutes	Tissierellia	Tissierellales		Sporanaerobacter		CRISPR array			4	37	30
6	CAS-I-B | SHD77697.1																	
	2150705..2153016	array		LT669839.1	[Clostridium] ultunense Esp	Bacteria	Firmicutes	Tissierellia	Tissierellales	Tissierellaceae			CRISPR array			35	37	30
	2153224..2153488	-	SHD77697.1	LT669839.1	[Clostridium] ultunense Esp	Bacteria	Firmicutes	Tissierellia	Tissierellales	Tissierellaceae		CRISPR-associated endoribonuclease Cas2	cas2	cd09725	CAS-I,CAS-II,CAS-III,CAS-V			
	2153500..2154493	-	SHD77698.1	LT669839.1	[Clostridium] ultunense Esp	Bacteria	Firmicutes	Tissierellia	Tissierellales	Tissierellaceae		CRISPR-associated endonuclease Cas1 2	cas1	cd09722	CAS-I-B			
	2154508..2155036	-	SHD77699.1	LT669839.1	[Clostridium] ultunense Esp	Bacteria	Firmicutes	Tissierellia	Tissierellales	Tissierellaceae		CRISPR-associated protein Cas4	cas4	pfam01930	CAS-I			
	2155020..2157333	-	SHD77700.1	LT669839.1	[Clostridium] ultunense Esp	Bacteria	Firmicutes	Tissierellia	Tissierellales	Tissierellaceae		CRISPR-associated helicase Cas3	cas3HD,cas3	cd09641,COG1203	CAS-I			
	2157349..2158066	-	SHD77701.1	LT669839.1	[Clostridium] ultunense Esp	Bacteria	Firmicutes	Tissierellia	Tissierellales	Tissierellaceae		CRISPR-associated protein Cas5, Hmari subtype	cas5	cd09692	CAS-I-B			
	2158076..2158958	-	SHD77702.1	LT669839.1	[Clostridium] ultunense Esp	Bacteria	Firmicutes	Tissierellia	Tissierellales	Tissierellaceae		CRISPR-associated protein, Csh2 family	cas7b	pfam05107	CAS-I-B,CAS-I-C			
	2158941..2160858	-	SHD77703.1	LT669839.1	[Clostridium] ultunense Esp	Bacteria	Firmicutes	Tissierellia	Tissierellales	Tissierellaceae		CRISPR-associated protein, Csh1 family	cas8b1	pfam09484	CAS-I-B			
	2160903..2161641	-	SHD77704.1	LT669839.1	[Clostridium] ultunense Esp	Bacteria	Firmicutes	Tissierellia	Tissierellales	Tissierellaceae		CRISPR-associated endoribonuclease	cas6	COG1583	CAS-I,CAS-III,CAS-IV			
7	CAS-I-B | SDZ39330.1																	
	330..2271	+	SDZ39244.1	FNQE01000052.1	Proteiniborus ethanoligenes	Bacteria	Firmicutes	Clostridia	Clostridiales		Proteiniborus	CRISPR-associated protein Csh1	cas8b1	pfam09484	CAS-I-B			
	2260..3199	+	SDZ39257.1	FNQE01000052.1	Proteiniborus ethanoligenes	Bacteria	Firmicutes	Clostridia	Clostridiales		Proteiniborus	CRISPR-associated protein, Csh2 family	cas7b	pfam05107	CAS-I-B,CAS-I-C			
	3211..4021	+	SDZ39267.1	FNQE01000052.1	Proteiniborus ethanoligenes	Bacteria	Firmicutes	Clostridia	Clostridiales		Proteiniborus	CRISPR-associated protein Cas5h	cas5	cd09692	CAS-I-B			
	3998..6500	+	SDZ39278.1	FNQE01000052.1	Proteiniborus ethanoligenes	Bacteria	Firmicutes	Clostridia	Clostridiales		Proteiniborus	CRISPR-associated helicase, Cas3 family	cas3HD,cas3	cd09641,COG1203	CAS-I			
	6518..7256	+	SDZ39290.1	FNQE01000052.1	Proteiniborus ethanoligenes	Bacteria	Firmicutes	Clostridia	Clostridiales		Proteiniborus	CRISPR-associated endoribonuclease Cas6	cas6	COG1583	CAS-I,CAS-III,CAS-IV			
	7292..7802	+	SDZ39302.1	FNQE01000052.1	Proteiniborus ethanoligenes	Bacteria	Firmicutes	Clostridia	Clostridiales		Proteiniborus	CRISPR-associated exonuclease, Cas4 family	cas4	pfam01930	CAS-I			
	7812..8805	+	SDZ39316.1	FNQE01000052.1	Proteiniborus ethanoligenes	Bacteria	Firmicutes	Clostridia	Clostridiales		Proteiniborus	CRISP-associated protein Cas1	cas1	cd09722	CAS-I-B			
	8817..9081	+	SDZ39330.1	FNQE01000052.1	Proteiniborus ethanoligenes	Bacteria	Firmicutes	Clostridia	Clostridiales		Proteiniborus	CRISPR-associated protein, Cas2 family	cas2	cd09725	CAS-I,CAS-II,CAS-III,CAS-V			
	9259..9423	array		FNQE01000052.1	Proteiniborus ethanoligenes	Bacteria	Firmicutes	Clostridia	Clostridiales		Proteiniborus		CRISPR array			3	36	31
8	CAS-I-B | SDW90812.1																	
	55774..57706	+	SDW90444.1	FNNG01000005.1	Tepidimicrobium xylanilyticum	Bacteria	Firmicutes	Tissierellia			Tepidimicrobium	CRISPR-associated protein, Csh1 family	cas8b1	pfam09484	CAS-I-B			
	57695..58637	+	SDW90489.1	FNNG01000005.1	Tepidimicrobium xylanilyticum	Bacteria	Firmicutes	Tissierellia			Tepidimicrobium	CRISPR-associated protein, Csh2 family	cas7b	pfam05107	CAS-I-B,CAS-I-C			
	58649..59459	+	SDW90533.1	FNNG01000005.1	Tepidimicrobium xylanilyticum	Bacteria	Firmicutes	Tissierellia			Tepidimicrobium	CRISPR-associated protein Cas5h	cas5	cd09692	CAS-I-B			
	59436..61938	+	SDW90579.1	FNNG01000005.1	Tepidimicrobium xylanilyticum	Bacteria	Firmicutes	Tissierellia			Tepidimicrobium	CRISPR-associated helicase, Cas3 family	cas3HD,cas3	cd09641,COG1203	CAS-I			
	61954..62695	+	SDW90639.1	FNNG01000005.1	Tepidimicrobium xylanilyticum	Bacteria	Firmicutes	Tissierellia			Tepidimicrobium	CRISPR-associated endoribonuclease Cas6	cas6	COG1583	CAS-I,CAS-III,CAS-IV			
	62729..63230	+	SDW90706.1	FNNG01000005.1	Tepidimicrobium xylanilyticum	Bacteria	Firmicutes	Tissierellia			Tepidimicrobium	CRISPR-associated exonuclease, Cas4 family	cas4	pfam01930	CAS-I			
	63241..64234	+	SDW90767.1	FNNG01000005.1	Tepidimicrobium xylanilyticum	Bacteria	Firmicutes	Tissierellia			Tepidimicrobium	CRISP-associated protein Cas1	cas1	cd09722	CAS-I-B			
	64244..64508	+	SDW90812.1	FNNG01000005.1	Tepidimicrobium xylanilyticum	Bacteria	Firmicutes	Tissierellia			Tepidimicrobium	CRISPR-associated protein, Cas2 family	cas2	cd09725	CAS-I,CAS-II,CAS-III,CAS-V			
	64802..65633	array		FNNG01000005.1	Tepidimicrobium xylanilyticum	Bacteria	Firmicutes	Tissierellia			Tepidimicrobium		CRISPR array			13	36	30
9	CAS-I-B | SES90532.1																	
	16861..17821	array		FOIF01000018.1	Anaerobranca gottschalkii DSM 13577	Bacteria	Firmicutes	Clostridia	Clostridiales	Proteinivoraceae	Anaerobranca		CRISPR array			15	36	30
	18028..18313	-	SES90532.1	FOIF01000018.1	Anaerobranca gottschalkii DSM 13577	Bacteria	Firmicutes	Clostridia	Clostridiales	Proteinivoraceae	Anaerobranca	CRISPR-associated protein, Cas2 family	cas2	cd09725	CAS-I,CAS-II,CAS-III,CAS-V			
	18296..19289	-	SES90548.1	FOIF01000018.1	Anaerobranca gottschalkii DSM 13577	Bacteria	Firmicutes	Clostridia	Clostridiales	Proteinivoraceae	Anaerobranca	CRISP-associated protein Cas1	cas1	cd09722	CAS-I-B			
	19300..19735	-	SES90565.1	FOIF01000018.1	Anaerobranca gottschalkii DSM 13577	Bacteria	Firmicutes	Clostridia	Clostridiales	Proteinivoraceae	Anaerobranca	CRISPR-associated exonuclease, Cas4 family	cas4	pfam01930	CAS-I			
	19826..22148	-	SES90582.1	FOIF01000018.1	Anaerobranca gottschalkii DSM 13577	Bacteria	Firmicutes	Clostridia	Clostridiales	Proteinivoraceae	Anaerobranca	CRISPR-associated helicase, Cas3 family	cas3HD,cas3	cd09641,COG1203	CAS-I			
	22157..22892	-	SES90603.1	FOIF01000018.1	Anaerobranca gottschalkii DSM 13577	Bacteria	Firmicutes	Clostridia	Clostridiales	Proteinivoraceae	Anaerobranca	CRISPR-associated protein Cas5h	cas5	cd09692	CAS-I-B			
	22897..23791	-	SES90620.1	FOIF01000018.1	Anaerobranca gottschalkii DSM 13577	Bacteria	Firmicutes	Clostridia	Clostridiales	Proteinivoraceae	Anaerobranca	CRISPR-associated protein, Csh2 family	cas7	cd09690	CAS-I-B			
	23780..25706	-	SES90649.1	FOIF01000018.1	Anaerobranca gottschalkii DSM 13577	Bacteria	Firmicutes	Clostridia	Clostridiales	Proteinivoraceae	Anaerobranca	CRISPR-associated protein Csh1	cas8b1	pfam09484	CAS-I-B			
10	CAS-I-B | SHK38018.1																	
	509..1409	+	SHK37840.1	FRAI01000041.1	Anaerobranca californiensis DSM 14826	Bacteria	Firmicutes	Clostridia	Clostridiales	Proteinivoraceae	Anaerobranca	CRISPR-associated protein, Csh2 family	cas7	cd09690	CAS-I-B			
	1413..2148	+	SHK37872.1	FRAI01000041.1	Anaerobranca californiensis DSM 14826	Bacteria	Firmicutes	Clostridia	Clostridiales	Proteinivoraceae	Anaerobranca	CRISPR-associated protein Cas5h	cas5	cd09692	CAS-I-B			
	2157..4473	+	SHK37907.1	FRAI01000041.1	Anaerobranca californiensis DSM 14826	Bacteria	Firmicutes	Clostridia	Clostridiales	Proteinivoraceae	Anaerobranca	CRISPR-associated helicase, Cas3 family	cas3HD,cas3	cd09641,COG1203	CAS-I			
	4496..5000	+	SHK37951.1	FRAI01000041.1	Anaerobranca californiensis DSM 14826	Bacteria	Firmicutes	Clostridia	Clostridiales	Proteinivoraceae	Anaerobranca	CRISPR-associated exonuclease, Cas4 family	cas4	pfam01930	CAS-I			
	5011..6004	+	SHK37983.1	FRAI01000041.1	Anaerobranca californiensis DSM 14826	Bacteria	Firmicutes	Clostridia	Clostridiales	Proteinivoraceae	Anaerobranca	CRISP-associated protein Cas1	cas1	cd09722	CAS-I-B			
	6008..6272	+	SHK38018.1	FRAI01000041.1	Anaerobranca californiensis DSM 14826	Bacteria	Firmicutes	Clostridia	Clostridiales	Proteinivoraceae	Anaerobranca	CRISPR-associated protein, Cas2 family	cas2	cd09725	CAS-I,CAS-II,CAS-III,CAS-V			
	6476..6703	array		FRAI01000041.1	Anaerobranca californiensis DSM 14826	Bacteria	Firmicutes	Clostridia	Clostridiales	Proteinivoraceae	Anaerobranca		CRISPR array			4	37	29
11	CAS-I-B | KXG74356.1																	
	162..5127	array		LOEE01000056.1	Thermotalea metallivorans	Bacteria	Firmicutes	Clostridia	Clostridiales	Clostridiaceae	Thermotalea		CRISPR array			75	36	30
	5325..5589	-	KXG74356.1	LOEE01000056.1	Thermotalea metallivorans	Bacteria	Firmicutes	Clostridia	Clostridiales	Clostridiaceae	Thermotalea	CRISPR-associated endoribonuclease Cas2	cas2	cd09725	CAS-I,CAS-II,CAS-III,CAS-V			
	5601..6594	-	KXG74357.1	LOEE01000056.1	Thermotalea metallivorans	Bacteria	Firmicutes	Clostridia	Clostridiales	Clostridiaceae	Thermotalea	CRISPR-associated endonuclease Cas1	cas1	cd09722	CAS-I-B			
	6606..7107	-	KXG74358.1	LOEE01000056.1	Thermotalea metallivorans	Bacteria	Firmicutes	Clostridia	Clostridiales	Clostridiaceae	Thermotalea	hypothetical protein	cas4	pfam01930	CAS-I			
	7142..7913	-	KXG74359.1	LOEE01000056.1	Thermotalea metallivorans	Bacteria	Firmicutes	Clostridia	Clostridiales	Clostridiaceae	Thermotalea	hypothetical protein	cas6	COG1583	CAS-I,CAS-III,CAS-IV			
	7935..10443	-	KXG74360.1	LOEE01000056.1	Thermotalea metallivorans	Bacteria	Firmicutes	Clostridia	Clostridiales	Clostridiaceae	Thermotalea	CRISPR-associated nuclease/helicase Cas3	cas3	COG1203	CAS-I			
	10420..11230	-	KXG74361.1	LOEE01000056.1	Thermotalea metallivorans	Bacteria	Firmicutes	Clostridia	Clostridiales	Clostridiaceae	Thermotalea	hypothetical protein	cas5	cd09692	CAS-I-B			
	11242..12184	-	KXG74362.1	LOEE01000056.1	Thermotalea metallivorans	Bacteria	Firmicutes	Clostridia	Clostridiales	Clostridiaceae	Thermotalea	hypothetical protein	cas7b	pfam05107	CAS-I-B,CAS-I-C			
	12167..14108	-	KXG74363.1	LOEE01000056.1	Thermotalea metallivorans	Bacteria	Firmicutes	Clostridia	Clostridiales	Clostridiaceae	Thermotalea	hypothetical protein	cas8b1	pfam09484	CAS-I-B			
12	partial | KGG79513.1																	
	226..920	array		AZTB01000091.1	Caloranaerobacter azorensis H53214	Bacteria	Firmicutes	Clostridia	Clostridiales	Clostridiaceae	Caloranaerobacter		CRISPR array			11	36	30
	1172..1436	-	KGG79513.1	AZTB01000091.1	Caloranaerobacter azorensis H53214	Bacteria	Firmicutes	Clostridia	Clostridiales	Clostridiaceae	Caloranaerobacter	CRISPR-associated protein Cas2	cas2	COG1343	CAS-I,CAS-II,CAS-III			
	1588..3424	array		AZTB01000091.1	Caloranaerobacter azorensis H53214	Bacteria	Firmicutes	Clostridia	Clostridiales	Clostridiaceae	Caloranaerobacter		CRISPR array			28	36	30
13	CAS-I-B | SHK18635.1																	
	391..1141	+	SHK18445.1	FRAJ01000011.1	Caminicella sporogenes DSM 14501	Bacteria	Firmicutes	Clostridia	Clostridiales	Clostridiaceae	Caminicella	CRISPR-associated endoribonuclease Cas6	cas6	COG1583	CAS-I,CAS-III,CAS-IV			
	1145..2921	+	SHK18473.1	FRAJ01000011.1	Caminicella sporogenes DSM 14501	Bacteria	Firmicutes	Clostridia	Clostridiales	Clostridiaceae	Caminicella	CRISPR-associated protein Csh1	cas8b1	cd09730	CAS-I-B			
	2913..3813	+	SHK18500.1	FRAJ01000011.1	Caminicella sporogenes DSM 14501	Bacteria	Firmicutes	Clostridia	Clostridiales	Clostridiaceae	Caminicella	CRISPR-associated protein, Csh2 family	cas7	cd09690	CAS-I-B			
	3823..4525	+	SHK18525.1	FRAJ01000011.1	Caminicella sporogenes DSM 14501	Bacteria	Firmicutes	Clostridia	Clostridiales	Clostridiaceae	Caminicella	CRISPR-associated protein, Cas5h family	cas5	cls001430	CAS-I-B			
	4511..6770	+	SHK18551.1	FRAJ01000011.1	Caminicella sporogenes DSM 14501	Bacteria	Firmicutes	Clostridia	Clostridiales	Clostridiaceae	Caminicella	CRISPR-associated endonuclease/helicase Cas3	cas3HD,cas3	cd09641,COG1203	CAS-I			
	6806..6977	+	SHK18580.1	FRAJ01000011.1	Caminicella sporogenes DSM 14501	Bacteria	Firmicutes	Clostridia	Clostridiales	Clostridiaceae	Caminicella	protein of unknown function DUF83	cas4	pfam01930	CAS-I			
	7063..7894	+	SHK18615.1	FRAJ01000011.1	Caminicella sporogenes DSM 14501	Bacteria	Firmicutes	Clostridia	Clostridiales	Clostridiaceae	Caminicella	CRISP-associated protein Cas1	cas1	cd09722	CAS-I-B			
	7906..8170	+	SHK18635.1	FRAJ01000011.1	Caminicella sporogenes DSM 14501	Bacteria	Firmicutes	Clostridia	Clostridiales	Clostridiaceae	Caminicella	CRISPR-associated protein, Cas2 family	cas2	COG1343	CAS-I,CAS-II,CAS-III			
	8411..8983	array		FRAJ01000011.1	Caminicella sporogenes DSM 14501	Bacteria	Firmicutes	Clostridia	Clostridiales	Clostridiaceae	Caminicella		CRISPR array			9	37	30
14	CAS-I-B | AEE97372.1																	
	2332557..2332821	-	AEE97372.1	CP002360.1	Mahella australiensis 50-1 BON	Bacteria	Firmicutes	Clostridia	Thermoanaerobacterales	Thermoanaerobacterales Family IV. Incertae Sedis	Mahella	CRISPR-associated protein, Cas2 family	cas2	COG1343	CAS-I,CAS-II,CAS-III			
	2332824..2333820	-	AEE97373.1	CP002360.1	Mahella australiensis 50-1 BON	Bacteria	Firmicutes	Clostridia	Thermoanaerobacterales	Thermoanaerobacterales Family IV. Incertae Sedis	Mahella	CRISPR-associated protein, Cas1 family	cas1	cd09722	CAS-I-B			
	2333834..2334335	-	AEE97374.1	CP002360.1	Mahella australiensis 50-1 BON	Bacteria	Firmicutes	Clostridia	Thermoanaerobacterales	Thermoanaerobacterales Family IV. Incertae Sedis	Mahella	CRISPR-associated exonuclease, Cas4 family	cas4	pfam01930	CAS-I			
	2336692..2342216	array		CP002360.1	Mahella australiensis 50-1 BON	Bacteria	Firmicutes	Clostridia	Thermoanaerobacterales	Thermoanaerobacterales Family IV. Incertae Sedis	Mahella		CRISPR array			83	37	30
	2342439..2344887	-	AEE97375.1	CP002360.1	Mahella australiensis 50-1 BON	Bacteria	Firmicutes	Clostridia	Thermoanaerobacterales	Thermoanaerobacterales Family IV. Incertae Sedis	Mahella	CRISPR-associated helicase Cas3	cas3HD,cas3	cd09641,COG1203	CAS-I			
	2344907..2345675	-	AEE97376.1	CP002360.1	Mahella australiensis 50-1 BON	Bacteria	Firmicutes	Clostridia	Thermoanaerobacterales	Thermoanaerobacterales Family IV. Incertae Sedis	Mahella	CRISPR-associated protein Cas5	cas5	cd09692	CAS-I-B			
	2345719..2346637	-	AEE97377.1	CP002360.1	Mahella australiensis 50-1 BON	Bacteria	Firmicutes	Clostridia	Thermoanaerobacterales	Thermoanaerobacterales Family IV. Incertae Sedis	Mahella	CRISPR-associated protein, Csh2 family	cas7b	pfam05107	CAS-I-B,CAS-I-C			
	2346629..2348597	-	AEE97378.1	CP002360.1	Mahella australiensis 50-1 BON	Bacteria	Firmicutes	Clostridia	Thermoanaerobacterales	Thermoanaerobacterales Family IV. Incertae Sedis	Mahella	CRISPR-associated protein, Csh1 family	cas8b1	pfam09484	CAS-I-B			
	2348589..2349360	-	AEE97379.1	CP002360.1	Mahella australiensis 50-1 BON	Bacteria	Firmicutes	Clostridia	Thermoanaerobacterales	Thermoanaerobacterales Family IV. Incertae Sedis	Mahella	CRISPR-associated protein Cas6	cas6	COG1583	CAS-I,CAS-III,CAS-IV			
	2349544..2351401	-	AEE97380.1	CP002360.1	Mahella australiensis 50-1 BON	Bacteria	Firmicutes	Clostridia	Thermoanaerobacterales	Thermoanaerobacterales Family IV. Incertae Sedis	Mahella	hypothetical protein	HerA	cluster57				

15 CAS-I-B | SHJ60937.1																	
514..2416	+	SHJ60818.1	FRAG01000004.1	Paramaledivibacter caminithermalis DSM 15212	Bacteria	Firmicutes	Clostridia	Clostridiales	Clostridiaceae	Paramaledivibacter	CRISPR-associated protein Csh1	cas8b1	pfam09484	CAS-I-B			
2408..3299	+	SHJ60841.1	FRAG01000004.1	Paramaledivibacter caminithermalis DSM 15212	Bacteria	Firmicutes	Clostridia	Clostridiales	Clostridiaceae	Paramaledivibacter	CRISPR-associated protein, Csh2 family	cas7	cd09690	CAS-I-B			
3304..4054	+	SHJ60861.1	FRAG01000004.1	Paramaledivibacter caminithermalis DSM 15212	Bacteria	Firmicutes	Clostridia	Clostridiales	Clostridiaceae	Paramaledivibacter	CRISPR-associated protein Cas5h	cas5	cd09692	CAS-I-B			
4065..6360	+	SHJ60873.1	FRAG01000004.1	Paramaledivibacter caminithermalis DSM 15212	Bacteria	Firmicutes	Clostridia	Clostridiales	Clostridiaceae	Paramaledivibacter	CRISPR-associated helicase, Cas3 family	cas3HD,cas3	cd09641,COG1203	CAS-I			
6395..6896	+	SHJ60895.1	FRAG01000004.1	Paramaledivibacter caminithermalis DSM 15212	Bacteria	Firmicutes	Clostridia	Clostridiales	Clostridiaceae	Paramaledivibacter	CRISPR-associated exonuclease, Cas4 family	cas4	pfam01930	CAS-I			
6908..7904	+	SHJ60917.1	FRAG01000004.1	Paramaledivibacter caminithermalis DSM 15212	Bacteria	Firmicutes	Clostridia	Clostridiales	Clostridiaceae	Paramaledivibacter	CRISP-associated protein Cas1	cas1	cd09722	CAS-I-B			
7910..8174	+	SHJ60937.1	FRAG01000004.1	Paramaledivibacter caminithermalis DSM 15212	Bacteria	Firmicutes	Clostridia	Clostridiales	Clostridiaceae	Paramaledivibacter	CRISPR-associated protein, Cas2 family	cas2	cd09725	CAS-I,CAS-II,CAS-III,CAS-V			
8235..8976	+	SHJ60951.1	FRAG01000004.1	Paramaledivibacter caminithermalis DSM 15212	Bacteria	Firmicutes	Clostridia	Clostridiales	Clostridiaceae	Paramaledivibacter	CRISPR-associated endoribonuclease Cas6	cas6	COG1583	CAS-I,CAS-III,CAS-IV			
12517..12907	+	SHJ60970.1	FRAG01000004.1	Paramaledivibacter caminithermalis DSM 15212	Bacteria	Firmicutes	Clostridia	Clostridiales	Clostridiaceae	Paramaledivibacter	hypothetical protein	csx20	cls000023	CAS-I,CAS-III			
13198..14359	+	SHJ60992.1	FRAG01000004.1	Paramaledivibacter caminithermalis DSM 15212	Bacteria	Firmicutes	Clostridia	Clostridiales	Clostridiaceae	Paramaledivibacter	protein of unknown function	csx1	pfam09002	CAS-III			
16270..16537	+	SHJ61009.1	FRAG01000004.1	Paramaledivibacter caminithermalis DSM 15212	Bacteria	Firmicutes	Clostridia	Clostridiales	Clostridiaceae	Paramaledivibacter	hypothetical protein						
16678..17767	+	SHJ61028.1	FRAG01000004.1	Paramaledivibacter caminithermalis DSM 15212	Bacteria	Firmicutes	Clostridia	Clostridiales	Clostridiaceae	Paramaledivibacter	pseudouridine kinase	casR	cls001593	CAS-I			
16 CAS-I-B | ABB14072.1																	
1926257..1930135	array		CP000141.1	Carboxydothermus hydrogenoformans Z-2901	Bacteria	Firmicutes	Clostridia	Thermoanaerobacterales	Thermoanaerobacteraceae	Carboxydothermus		CRISPR array			59	36	30
1930462..1930726	-	ABB14072.1	CP000141.1	Carboxydothermus hydrogenoformans Z-2901	Bacteria	Firmicutes	Clostridia	Thermoanaerobacterales	Thermoanaerobacteraceae	Carboxydothermus	CRISPR-associated protein Cas2	cas2	cd09725	CAS-I,CAS-II,CAS-III,CAS-V			
1930727..1931720	-	ABB14658.1	CP000141.1	Carboxydothermus hydrogenoformans Z-2901	Bacteria	Firmicutes	Clostridia	Thermoanaerobacterales	Thermoanaerobacteraceae	Carboxydothermus	CRISPR-associated protein Cas1	cas1	cd09722	CAS-I-B			
1931730..1932231	-	ABB14082.1	CP000141.1	Carboxydothermus hydrogenoformans Z-2901	Bacteria	Firmicutes	Clostridia	Thermoanaerobacterales	Thermoanaerobacteraceae	Carboxydothermus	CRISPR-associated protein Cas4	cas4	pfam01930	CAS-I			
1932315..1934760	-	ABB14293.1	CP000141.1	Carboxydothermus hydrogenoformans Z-2901	Bacteria	Firmicutes	Clostridia	Thermoanaerobacterales	Thermoanaerobacteraceae	Carboxydothermus	CRISPR-associated helicase Cas3	cas3	COG1203	CAS-I			
1934759..1935506	-	ABB15084.1	CP000141.1	Carboxydothermus hydrogenoformans Z-2901	Bacteria	Firmicutes	Clostridia	Thermoanaerobacterales	Thermoanaerobacteraceae	Carboxydothermus	CRISPR-associated protein Cas5, Hmari subtype	cas5	cd09692	CAS-I-B			
1935522..1936464	-	ABB14134.1	CP000141.1	Carboxydothermus hydrogenoformans Z-2901	Bacteria	Firmicutes	Clostridia	Thermoanaerobacterales	Thermoanaerobacteraceae	Carboxydothermus	CRISPR-associated protein, Csh2 family	cas7b	pfam05107	CAS-I-B,CAS-I-C			
1936464..1938405	-	ABB14905.1	CP000141.1	Carboxydothermus hydrogenoformans Z-2901	Bacteria	Firmicutes	Clostridia	Thermoanaerobacterales	Thermoanaerobacteraceae	Carboxydothermus	CRISPR-associated protein, Csh1 family	cas8b1	pfam09484	CAS-I-B			
1938476..1939223	-	ABB15795.1	CP000141.1	Carboxydothermus hydrogenoformans Z-2901	Bacteria	Firmicutes	Clostridia	Thermoanaerobacterales	Thermoanaerobacteraceae	Carboxydothermus	CRISPR-associated protein Cas6	cas6	COG1583	CAS-I,CAS-III,CAS-IV			
17 CAS-I-B | GAV23064.1																	
13662..14025	array		BDJK01000027.1	Carboxydothermus pertinax	Bacteria	Firmicutes	Clostridia	Thermoanaerobacterales	Thermoanaerobacteraceae	Carboxydothermus		CRISPR array			6	36	30
14133..14823	+	GAV23050.1	BDJK01000027.1	Carboxydothermus pertinax	Bacteria	Firmicutes	Clostridia	Thermoanaerobacterales	Thermoanaerobacteraceae	Carboxydothermus	hypothetical protein						
15667..16021	+	GAV23051.1	BDJK01000027.1	Carboxydothermus pertinax	Bacteria	Firmicutes	Clostridia	Thermoanaerobacterales	Thermoanaerobacteraceae	Carboxydothermus	hypothetical protein						
16153..17224	+	GAV23052.1	BDJK01000027.1	Carboxydothermus pertinax	Bacteria	Firmicutes	Clostridia	Thermoanaerobacterales	Thermoanaerobacteraceae	Carboxydothermus	hypothetical protein						
17570..18727	array		BDJK01000027.1	Carboxydothermus pertinax	Bacteria	Firmicutes	Clostridia	Thermoanaerobacterales	Thermoanaerobacteraceae	Carboxydothermus		CRISPR array			18	36	30
18777..18981	+	GAV23053.1	BDJK01000027.1	Carboxydothermus pertinax	Bacteria	Firmicutes	Clostridia	Thermoanaerobacterales	Thermoanaerobacteraceae	Carboxydothermus	conserved domain protein						
19402..19693	-	GAV23054.1	BDJK01000027.1	Carboxydothermus pertinax	Bacteria	Firmicutes	Clostridia	Thermoanaerobacterales	Thermoanaerobacteraceae	Carboxydothermus	hypothetical protein						
19786..20146	+	GAV23055.1	BDJK01000027.1	Carboxydothermus pertinax	Bacteria	Firmicutes	Clostridia	Thermoanaerobacterales	Thermoanaerobacteraceae	Carboxydothermus	hypothetical protein						
20321..20930	+	GAV23056.1	BDJK01000027.1	Carboxydothermus pertinax	Bacteria	Firmicutes	Clostridia	Thermoanaerobacterales	Thermoanaerobacteraceae	Carboxydothermus	CAAX protease family protein						
21150..21897	+	GAV23057.1	BDJK01000027.1	Carboxydothermus pertinax	Bacteria	Firmicutes	Clostridia	Thermoanaerobacterales	Thermoanaerobacteraceae	Carboxydothermus	CRISPR-associated protein Cas6	cas6	COG1583	CAS-I,CAS-III,CAS-IV			
21968..23909	+	GAV23058.1	BDJK01000027.1	Carboxydothermus pertinax	Bacteria	Firmicutes	Clostridia	Thermoanaerobacterales	Thermoanaerobacteraceae	Carboxydothermus	type I-B CRISPR-associated protein Cas8b/Csh1	cas8b1	pfam09484	CAS-I-B			
23909..24851	+	GAV23059.1	BDJK01000027.1	Carboxydothermus pertinax	Bacteria	Firmicutes	Clostridia	Thermoanaerobacterales	Thermoanaerobacteraceae	Carboxydothermus	type I-B CRISPR-associated protein Cas7/Csh2	cas7	cd09690	CAS-I-B			
24867..25614	+	GAV23060.1	BDJK01000027.1	Carboxydothermus pertinax	Bacteria	Firmicutes	Clostridia	Thermoanaerobacterales	Thermoanaerobacteraceae	Carboxydothermus	CRISPR-associated protein Cas5	cas5	cd09692	CAS-I-B			
25613..28058	+	GAV23061.1	BDJK01000027.1	Carboxydothermus pertinax	Bacteria	Firmicutes	Clostridia	Thermoanaerobacterales	Thermoanaerobacteraceae	Carboxydothermus	CRISPR-associated helicase/endonuclease Cas3	cas3HD,cas3	cd09641,COG1203	CAS-I			
28150..28582	+	GAV23062.1	BDJK01000027.1	Carboxydothermus pertinax	Bacteria	Firmicutes	Clostridia	Thermoanaerobacterales	Thermoanaerobacteraceae	Carboxydothermus	CRISPR-associated protein Cas4	cas4	pfam01930	CAS-I			
28592..29585	+	GAV23063.1	BDJK01000027.1	Carboxydothermus pertinax	Bacteria	Firmicutes	Clostridia	Thermoanaerobacterales	Thermoanaerobacteraceae	Carboxydothermus	subtype I-B CRISPR-associated endonuclease Cas1	cas1	cd09722	CAS-I-B			
29586..29850	+	GAV23064.1	BDJK01000027.1	Carboxydothermus pertinax	Bacteria	Firmicutes	Clostridia	Thermoanaerobacterales	Thermoanaerobacteraceae	Carboxydothermus	CRISPR-associated endonuclease Cas2	cas2	cd09725	CAS-I,CAS-II,CAS-III,CAS-V			
30175..35460	array		BDJK01000027.1	Carboxydothermus pertinax	Bacteria	Firmicutes	Clostridia	Thermoanaerobacterales	Thermoanaerobacteraceae	Carboxydothermus		CRISPR array			80	36	30
18 CAS-I-B | OQX85653.1																	
0..645	+	OQX85645.1	NATM01000098.1	Candidatus Omnitrophica bacterium 4484_70.2	Bacteria	Candidatus Omnitrophica					hypothetical protein	cas8b1	mkCas0195	CAS-I-B			
665..1745	+	OQX85646.1	NATM01000098.1	Candidatus Omnitrophica bacterium 4484_70.2	Bacteria	Candidatus Omnitrophica					type I-B CRISPR-associated protein Cas7/Cst2/DevR	cas7	cd09687	CAS-I-B			
1750..2476	+	OQX85647.1	NATM01000098.1	Candidatus Omnitrophica bacterium 4484_70.2	Bacteria	Candidatus Omnitrophica					hypothetical protein	cas5	cd09658	CAS-I-B			
2453..3863	+	OQX85648.1	NATM01000098.1	Candidatus Omnitrophica bacterium 4484_70.2	Bacteria	Candidatus Omnitrophica					hypothetical protein	cas3HD,cas3	cd09641,cd09639	CAS-I			
3829..4678	+	OQX85649.1	NATM01000098.1	Candidatus Omnitrophica bacterium 4484_70.2	Bacteria	Candidatus Omnitrophica					hypothetical protein	cas3	COG1203	CAS-I			
5139..6390	+	OQX85650.1	NATM01000098.1	Candidatus Omnitrophica bacterium 4484_70.2	Bacteria	Candidatus Omnitrophica					ATPase						
6391..6907	+	OQX85651.1	NATM01000098.1	Candidatus Omnitrophica bacterium 4484_70.2	Bacteria	Candidatus Omnitrophica					CRISPR-associated protein Cas4	cas4	pfam01930	CAS-I			
7319..8315	+	OQX85652.1	NATM01000098.1	Candidatus Omnitrophica bacterium 4484_70.2	Bacteria	Candidatus Omnitrophica					subtype I-B CRISPR-associated endonuclease Cas1	cas1	cd09722	CAS-I-B			
8316..8580	+	OQX85653.1	NATM01000098.1	Candidatus Omnitrophica bacterium 4484_70.2	Bacteria	Candidatus Omnitrophica					CRISPR-associated endonuclease Cas2	cas2	COG1343	CAS-I,CAS-II,CAS-III			
8846..9409	array		NATM01000098.1	Candidatus Omnitrophica bacterium 4484_70.2	Bacteria	Candidatus Omnitrophica						CRISPR array			9	36	30
19 CAS-I-B | AMM41387.1																	
1573130..1574909	+	AMM41378.1	CP013015.1	Candidatus Desulfofervidus auxilii	Bacteria	Proteobacteria	Deltaproteobacteria		Candidatus Desulfofervidaceae	Candidatus Desulfofervidus	CRISPR-associated protein Csh1	cas8b1	cd09730	CAS-I-B			
1574892..1575870	+	AMM41379.1	CP013015.1	Candidatus Desulfofervidus auxilii	Bacteria	Proteobacteria	Deltaproteobacteria		Candidatus Desulfofervidaceae	Candidatus Desulfofervidus	CRISPR-associated protein, Csh2 family	cas7	cd09690	CAS-I-B			
1575857..1576577	+	AMM41380.1	CP013015.1	Candidatus Desulfofervidus auxilii	Bacteria	Proteobacteria	Deltaproteobacteria		Candidatus Desulfofervidaceae	Candidatus Desulfofervidus	CRISPR-associated protein Cas5	cas5	cls001430	CAS-I-B			
1576578..1578888	+	AMM41381.1	CP013015.1	Candidatus Desulfofervidus auxilii	Bacteria	Proteobacteria	Deltaproteobacteria		Candidatus Desulfofervidaceae	Candidatus Desulfofervidus	CRISPR-associated protein Cas3	cas3HD,cas3	cd09641,COG1203	CAS-I			
1579135..1579378	+	AMM41382.1	CP013015.1	Candidatus Desulfofervidus auxilii	Bacteria	Proteobacteria	Deltaproteobacteria		Candidatus Desulfofervidaceae	Candidatus Desulfofervidus	addiction module antitoxin						
1579599..1580094	+	AMM41383.1	CP013015.1	Candidatus Desulfofervidus auxilii	Bacteria	Proteobacteria	Deltaproteobacteria		Candidatus Desulfofervidaceae	Candidatus Desulfofervidus	CRISPR-associated protein Cas4	cas4	pfam01930	CAS-I			
1580155..1580383	+	AMM41384.1	CP013015.1	Candidatus Desulfofervidus auxilii	Bacteria	Proteobacteria	Deltaproteobacteria		Candidatus Desulfofervidaceae	Candidatus Desulfofervidus	transcriptional regulator, AbrB family						
1580366..1580810	+	AMM41385.1	CP013015.1	Candidatus Desulfofervidus auxilii	Bacteria	Proteobacteria	Deltaproteobacteria		Candidatus Desulfofervidaceae	Candidatus Desulfofervidus	protein containing PilT protein domain						
1580908..1581901	+	AMM41386.1	CP013015.1	Candidatus Desulfofervidus auxilii	Bacteria	Proteobacteria	Deltaproteobacteria		Candidatus Desulfofervidaceae	Candidatus Desulfofervidus	CRISPR-associated protein Cas1	cas1	cd09722	CAS-I-B			
1581976..1582240	+	AMM41387.1	CP013015.1	Candidatus Desulfofervidus auxilii	Bacteria	Proteobacteria	Deltaproteobacteria		Candidatus Desulfofervidaceae	Candidatus Desulfofervidus	CRISPR-associated protein Cas2	cas2	cd09725	CAS-I,CAS-II,CAS-III,CAS-V			
1582463..1585620	array		CP013015.1	Candidatus Desulfofervidus auxilii	Bacteria	Proteobacteria	Deltaproteobacteria		Candidatus Desulfofervidaceae	Candidatus Desulfofervidus		CRISPR array			48	36	30
20 CAS-I-B | PWI57015.1																	
4669..5428	+	PWI57009.1	MPDK01000020.1	Acidibacillus sulfuroxidans	Bacteria	Firmicutes	Bacilli	Bacillales		Acidibacillus	CRISPR-associated endoribonuclease Cas6	cas6	COG1583	CAS-I,CAS-III,CAS-IV			
5433..7497	+	PWI57010.1	MPDK01000020.1	Acidibacillus sulfuroxidans	Bacteria	Firmicutes	Bacilli	Bacillales		Acidibacillus	type I-B CRISPR-associated protein Cas8b/Csh1	cas8b1	pfam09484	CAS-I-B			
7533..8478	+	PWI57011.1	MPDK01000020.1	Acidibacillus sulfuroxidans	Bacteria	Firmicutes	Bacilli	Bacillales		Acidibacillus	type I-B CRISPR-associated protein Cas7/Csh2	cas7	cd09690	CAS-I-B			
8474..9272	+	PWI57012.1	MPDK01000020.1	Acidibacillus sulfuroxidans	Bacteria	Firmicutes	Bacilli	Bacillales		Acidibacillus	hypothetical protein	cas5	cd09692	CAS-I-B			
9268..11803	+	PWI57013.1	MPDK01000020.1	Acidibacillus sulfuroxidans	Bacteria	Firmicutes	Bacilli	Bacillales		Acidibacillus	hypothetical protein	cas3HD,cas3	cd09641,COG1203	CAS-I			
11816..12329	+	PWI57014.1	MPDK01000020.1	Acidibacillus sulfuroxidans	Bacteria	Firmicutes	Bacilli	Bacillales		Acidibacillus	CRISPR-associated protein Cas4	cas4	pfam01930	CAS-I			
12344..13337	+	PWI57043.1	MPDK01000020.1	Acidibacillus sulfuroxidans	Bacteria	Firmicutes	Bacilli	Bacillales		Acidibacillus	subtype I-B CRISPR-associated endonuclease Cas1	cas1	cd09722	CAS-I-B			
13354..13618	+	PWI57015.1	MPDK01000020.1	Acidibacillus sulfuroxidans	Bacteria	Firmicutes	Bacilli	Bacillales		Acidibacillus	CRISPR-associated endonuclease Cas2	cas2	cd09725	CAS-I,CAS-II,CAS-III,CAS-V			
13886..15779	array		MPDK01000020.1	Acidibacillus sulfuroxidans	Bacteria	Firmicutes	Bacilli	Bacillales		Acidibacillus		CRISPR array			29	36	30
21 CAS-I-B | SHG49963.1																	
89987..90734	+	SHG49718.1	FQWY01000004.1	Thermosyntropha lipolytica DSM 11003	Bacteria	Firmicutes	Clostridia	Clostridiales	Syntrophomonadaceae	Thermosyntropha	CRISPR-associated endoribonuclease Cas6	cas6	COG1583	CAS-I,CAS-III,CAS-IV			
90736..92686	+	SHG49766.1	FQWY01000004.1	Thermosyntropha lipolytica DSM 11003	Bacteria	Firmicutes	Clostridia	Clostridiales	Syntrophomonadaceae	Thermosyntropha	CRISPR-associated protein Csh1	cas8b1	pfam09484	CAS-I-B			
92672..93548	+	SHG49794.1	FQWY01000004.1	Thermosyntropha lipolytica DSM 11003	Bacteria	Firmicutes	Clostridia	Clostridiales	Syntrophomonadaceae	Thermosyntropha	CRISPR-associated protein, Csh2 family	cas7b	pfam05107	CAS-I-B,CAS-I-C			
93567..94320	+	SHG49828.1	FQWY01000004.1	Thermosyntropha lipolytica DSM 11003	Bacteria	Firmicutes	Clostridia	Clostridiales	Syntrophomonadaceae	Thermosyntropha	CRISPR-associated protein Cas5h	cas5	cd09692	CAS-I-B			
94338..96690	+	SHG49864.1	FQWY01000004.1	Thermosyntropha lipolytica DSM 11003	Bacteria	Firmicutes	Clostridia	Clostridiales	Syntrophomonadaceae	Thermosyntropha	CRISPR-associated helicase, Cas3 family	cas3HD,cas3	cd09641,cd09639	CAS-I			
96689..97208	+	SHG49891.1	FQWY01000004.1	Thermosyntropha lipolytica DSM 11003	Bacteria	Firmicutes	Clostridia	Clostridiales	Syntrophomonadaceae	Thermosyntropha	CRISPR-associated exonuclease, Cas4 family	cas4	pfam01930	CAS-I			
97226..98219	+	SHG49925.1	FQWY01000004.1	Thermosyntropha lipolytica DSM 11003	Bacteria	Firmicutes	Clostridia	Clostridiales	Syntrophomonadaceae	Thermosyntropha	CRISPR-associated protein, Cas1 family	cas1	cd09722	CAS-I-B			
98233..98497	+	SHG49963.1	FQWY01000004.1	Thermosyntropha lipolytica DSM 11003	Bacteria	Firmicutes	Clostridia	Clostridiales	Syntrophomonadaceae	Thermosyntropha	CRISPR-associated protein Cas2	cas2	COG1343	CAS-I,CAS-II,CAS-III			
22 partial CAS-I-B | KUK35957.1																	
550..1066	+	KUK35955.1	LGFO01000205.1	Thermacetogenium phaeum	Bacteria	Firmicutes	Clostridia	Thermoanaerobacterales	Thermoanaerobacteraceae	Thermacetogenium	CRISPR-associated protein Cas4	cas4	pfam01930	CAS-I			
1077..2070	+	KUK35956.1	LGFO01000205.1	Thermacetogenium phaeum	Bacteria	Firmicutes	Clostridia	Thermoanaerobacterales	Thermoanaerobacteraceae	Thermacetogenium	CRISPR-associated endonuclease Cas1	cas1	cd09722	CAS-I-B			
2072..2336	+	KUK35957.1	LGFO01000205.1	Thermacetogenium phaeum	Bacteria	Firmicutes	Clostridia	Thermoanaerobacterales	Thermoanaerobacteraceae	Thermacetogenium	CRISPR-associated endoribonuclease Cas2	cas2	cd09725	CAS-I,CAS-II,CAS-III,CAS-V			
2349..2966	+	KUK35958.1	LGFO01000205.1	Thermacetogenium phaeum	Bacteria	Firmicutes	Clostridia	Thermoanaerobacterales	Thermoanaerobacteraceae	Thermacetogenium	CRISPR-associated endoribonuclease Cas6	cas6	COG1583	CAS-I,CAS-III,CAS-IV			
23 CAS-I-B | GAF26200.1																	
1514061..1515495	+	GAF26192.1	DF238840.1	Moorella thermoacetica Y72	Bacteria	Firmicutes	Clostridia	Thermoanaerobacterales	Thermoanaerobacteraceae	Moorella	single-stranded DNA-specific exonuclease	cas8b1	pfam09484	CAS-I-B			
1515499..1515910	+	GAF26193.1	DF238840.1	Moorella thermoacetica Y72	Bacteria	Firmicutes	Clostridia	Thermoanaerobacterales	Thermoanaerobacteraceae	Moorella	2-methylthioadenine synthetase	cas8b1	mkCas0119	CAS-I-B			
1515890..1516814	+	GAF26194.1	DF238840.1	Moorella thermoacetica Y72	Bacteria	Firmicutes	Clostridia	Thermoanaerobacterales	Thermoanaerobacteraceae	Moorella	uncharacterized protein	cas7	cd09690	CAS-I-B			
1516810..1517578	+	GAF26195.1	DF238840.1	Moorella thermoacetica Y72	Bacteria	Firmicutes	Clostridia	Thermoanaerobacterales	Thermoanaerobacteraceae	Moorella	uncharacterized protein	cas5	cls001430	CAS-I-B			
1517519..1519811	+	GAF26196.1	DF238840.1	Moorella thermoacetica Y72	Bacteria	Firmicutes	Clostridia	Thermoanaerobacterales	Thermoanaerobacteraceae	Moorella	predicted helicases	cas3HD,cas3	cd09641,cd09639	CAS-I			
1519793..1519898	-	GAF26197.1	DF238840.1	Moorella thermoacetica Y72	Bacteria	Firmicutes	Clostridia	Thermoanaerobacterales	Thermoanaerobacteraceae	Moorella	hypothetical protein						
1519811..1520318	+	GAF26198.1	DF238840.1	Moorella thermoacetica Y72	Bacteria	Firmicutes	Clostridia	Thermoanaerobacterales	Thermoanaerobacteraceae	Moorella	RecB family exonuclease	cas4	pfam01930	CAS-I			
1520305..1521328	+	GAF26199.1	DF238840.1	Moorella thermoacetica Y72	Bacteria	Firmicutes	Clostridia	Thermoanaerobacterales	Thermoanaerobacteraceae	Moorella	uncharacterized protein	cas1	cd09722	CAS-I-B			
1521329..1521593	+	GAF26200.1	DF238840.1	Moorella thermoacetica Y72	Bacteria	Firmicutes	Clostridia	Thermoanaerobacterales	Thermoanaerobacteraceae	Moorella	uncharacterized protein	cas2	cd09725	CAS-I,CAS-II,CAS-III,CAS-V			
1521607..1522351	+	GAF26201.1	DF238840.1	Moorella thermoacetica Y72	Bacteria	Firmicutes	Clostridia	Thermoanaerobacterales	Thermoanaerobacteraceae	Moorella	uncharacterized protein	cas6	COG1583	CAS-I,CAS-III,CAS-IV			
1521727..1521907	-	GAF26202.1	DF238840.1	Moorella thermoacetica Y72	Bacteria	Firmicutes	Clostridia	Thermoanaerobacterales	Thermoanaerobacteraceae	Moorella	non-ribosomal peptide synthetase modules and related proteins						
1522807..1528879	array		DF238840.1	Moorella thermoacetica Y72	Bacteria	Firmicutes	Clostridia	Thermoanaerobacterales	Thermoanaerobacteraceae	Moorella		CRISPR array			92	36	30
24 CAS-I-B | PRR75058.1																	
202934..214646	array		PVXM01000006.1	Moorella humiferrea	Bacteria	Firmicutes	Clostridia	Thermoanaerobacterales	Thermoanaerobacteraceae	Moorella		CRISPR array			177	36	30
214809..215553	-	PRR75057.1	PVXM01000006.1	Moorella humiferrea	Bacteria	Firmicutes	Clostridia	Thermoanaerobacterales	Thermoanaerobacteraceae	Moorella	CRISPR associated protein Cas6	cas6	COG1583	CAS-I,CAS-III,CAS-IV			
215578..215842	-	PRR75058.1	PVXM01000006.1	Moorella humiferrea	Bacteria	Firmicutes	Clostridia	Thermoanaerobacterales	Thermoanaerobacteraceae	Moorella	CRISPR-associated endoribonuclease Cas2	cas2	cd09725	CAS-I,CAS-II,CAS-III,CAS-V			
215844..216837	-	PRR75059.1	PVXM01000006.1	Moorella humiferrea	Bacteria	Firmicutes	Clostridia	Thermoanaerobacterales	Thermoanaerobacteraceae	Moorella	CRISPR-associated endonuclease Cas1	cas1	cd09722	CAS-I-B			
216850..217363	-	PRR75060.1	PVXM01000006.1	Moorella humiferrea	Bacteria	Firmicutes	Clostridia	Thermoanaerobacterales	Thermoanaerobacteraceae	Moorella	PD-(D/E)XK nuclease superfamily protein	cas4	pfam01930	CAS-I			
217355..219632	-	PRR75061.1	PVXM01000006.1	Moorella humiferrea	Bacteria	Firmicutes	Clostridia	Thermoanaerobacterales	Thermoanaerobacteraceae	Moorella	helicase Cas3	cas3HD,cas3	cd09641,cd09639	CAS-I			
219618..220341	-	PRR75062.1	PVXM01000006.1	Moorella humiferrea	Bacteria	Firmicutes	Clostridia	Thermoanaerobacterales	Thermoanaerobacteraceae	Moorella	CRISPR-associated protein Cas5	cas5	cls001430	CAS-I-B			
220346..221261	-	PRR75063.1	PVXM01000006.1	Moorella humiferrea	Bacteria	Firmicutes	Clostridia	Thermoanaerobacterales	Thermoanaerobacteraceae	Moorella	hypothetical protein	cas7	cd09690	CAS-I-B			
221291..221705	-	PRR75064.1	PVXM01000006.1	Moorella humiferrea	Bacteria	Firmicutes	Clostridia	Thermoanaerobacterales	Thermoanaerobacteraceae	Moorella	CRISPR-associated protein	cas8b1	mkCas0119	CAS-I-B			
221694..223149	-	PRR75065.1	PVXM01000006.1	Moorella humiferrea	Bacteria	Firmicutes	Clostridia	Thermoanaerobacterales	Thermoanaerobacteraceae	Moorella	CRISPR-associated protein	cas8b1	cd09730	CAS-I-B			
25 CAS-I-B | EGL83992.1																	
153..417	-	EGL83992.1	AFCE01000059.1	Caldalkalibacillus thermarum TA2.A1	Bacteria	Firmicutes	Bacilli	Bacillales	Bacillaceae	Caldalkalibacillus	CRISPR-associated protein Cas2	cas2	cd09725	CAS-I,CAS-II,CAS-III,CAS-V			
447..1452	-	EGL83993.1	AFCE01000059.1	Caldalkalibacillus thermarum TA2.A1	Bacteria	Firmicutes	Bacilli	Bacillales	Bacillaceae	Caldalkalibacillus	CRISPR-associated protein Cas1	cas1	cd09722	CAS-I-B			
1453..1984	-	EGL83994.1	AFCE01000059.1	Caldalkalibacillus thermarum TA2.A1	Bacteria	Firmicutes	Bacilli	Bacillales	Bacillaceae	Caldalkalibacillus	CRISPR-associated protein Cas4	cas4	pfam01930	CAS-I			
2125..4501	-	EGL83995.1	AFCE01000059.1	Caldalkalibacillus thermarum TA2.A1	Bacteria	Firmicutes	Bacilli	Bacillales	Bacillaceae	Caldalkalibacillus	CRISPR-associated HD domain protein	cas3HD,cas3	cd09641,COG1203	CAS-I			
4497..5280	-	EGL83996.1	AFCE01000059.1	Caldalkalibacillus thermarum TA2.A1	Bacteria	Firmicutes	Bacilli	Bacillales	Bacillaceae	Caldalkalibacillus	CRISPR-associated protein Cas5, Hmari subtype	cas5	cls001430	CAS-I-B			
5294..6251	-	EGL83997.1	AFCE01000059.1	Caldalkalibacillus thermarum TA2.A1	Bacteria	Firmicutes	Bacilli	Bacillales	Bacillaceae	Caldalkalibacillus	CRISPR-associated protein, Csh2 family	cas7	cd09690	CAS-I-B			
6253..8056	-	EGL83998.1	AFCE01000059.1	Caldalkalibacillus thermarum TA2.A1	Bacteria	Firmicutes	Bacilli	Bacillales	Bacillaceae	Caldalkalibacillus	CRISPR-associated protein, TM1802 family	cas8b1	cd09730	CAS-I-B			
26 CAS-I-B | KYC61680.1																	
6410..9656	+	KYC61672.1	LQYG01000065.1	Bacillus coagulans	Bacteria	Firmicutes	Bacilli	Bacillales	Bacillaceae	Bacillus	hypothetical protein	ZorD	ZorD				
10141..10897	+	KYC61673.1	LQYG01000065.1	Bacillus coagulans	Bacteria	Firmicutes	Bacilli	Bacillales	Bacillaceae	Bacillus	hypothetical protein	cas6	COG1583	CAS-I,CAS-III,CAS-IV			
10948..12973	+	KYC61674.1	LQYG01000065.1	Bacillus coagulans	Bacteria	Firmicutes	Bacilli	Bacillales	Bacillaceae	Bacillus	hypothetical protein	cas8b1	mkCas0074	CAS-I-B			
12959..13916	+	KYC61675.1	LQYG01000065.1	Bacillus coagulans	Bacteria	Firmicutes	Bacilli	Bacillales	Bacillaceae	Bacillus	hypothetical protein	cas7b	pfam05107	CAS-I-B,CAS-I-C			
13928..14618	+	KYC61676.1	LQYG01000065.1	Bacillus coagulans	Bacteria	Firmicutes	Bacilli	Bacillales	Bacillaceae	Bacillus	hypothetical protein	cas5	cls001430	CAS-I-B			
14605..16969	+	KYC61677.1	LQYG01000065.1	Bacillus coagulans	Bacteria	Firmicutes	Bacilli	Bacillales	Bacillaceae	Bacillus	hypothetical protein	cas3HD,cas3	cd09641,COG1203	CAS-I			
16981..17491	+	KYC61678.1	LQYG01000065.1	Bacillus coagulans	Bacteria	Firmicutes	Bacilli	Bacillales	Bacillaceae	Bacillus	hypothetical protein	cas4	pfam01930	CAS-I			
17492..18497	+	KYC61679.1	LQYG01000065.1	Bacillus coagulans	Bacteria	Firmicutes	Bacilli	Bacillales	Bacillaceae	Bacillus	hypothetical protein	cas1	cd09722	CAS-I-B			
18506..18770	+	KYC61680.1	LQYG01000065.1	Bacillus coagulans	Bacteria	Firmicutes	Bacilli	Bacillales	Bacillaceae	Bacillus	hypothetical protein	cas2	cd09725	CAS-I,CAS-II,CAS-III,CAS-V			
19479..20104	array		LQYG01000065.1	Bacillus coagulans	Bacteria	Firmicutes	Bacilli	Bacillales	Bacillaceae	Bacillus		CRISPR array			10	36	30
27 CAS-I-B | AXF57070.1																	
2840976..2841768	+	AXF57064.1	CP031092.1	Ruegeria sp. NKC1-1	Bacteria	Proteobacteria	Alphaproteobacteria	Rhodobacterales	Rhodobacteraceae	Ruegeria	CRISPR-associated endoribonuclease Cas6	cas6	COG1583	CAS-I,CAS-III,CAS-IV			
2841825..2843751	+	AXF57065.1	CP031092.1	Ruegeria sp. NKC1-1	Bacteria	Proteobacteria	Alphaproteobacteria	Rhodobacterales	Rhodobacteraceae	Ruegeria	type I-B CRISPR-associated protein Cas8b/Csh1	cas8b1	pfam09484	CAS-I-B			
2843740..2844691	+	AXF57066.1	CP031092.1	Ruegeria sp. NKC1-1	Bacteria	Proteobacteria	Alphaproteobacteria	Rhodobacterales	Rhodobacteraceae	Ruegeria	type I-B CRISPR-associated protein Cas7/Csh2	cas7b	pfam05107	CAS-I-B,CAS-I-C			
2844705..2845452	+	AXF57067.1	CP031092.1	Ruegeria sp. NKC1-1	Bacteria	Proteobacteria	Alphaproteobacteria	Rhodobacterales	Rhodobacteraceae	Ruegeria	type I-B CRISPR-associated protein Cas5	cas5	cd09692	CAS-I-B			
2845465..2847925	+	AXF57068.1	CP031092.1	Ruegeria sp. NKC1-1	Bacteria	Proteobacteria	Alphaproteobacteria	Rhodobacterales	Rhodobacteraceae	Ruegeria	CRISPR-associated helicase Cas3'	cas3HD,cas3	cd09641,COG1203	CAS-I			

2847956..2848454	+	AXF58011.1	CP031092.1	Ruegeria sp. NKC1-1	Bacteria	Proteobacteria	Alphaproteobacteria	Rhodobacterales	Rhodobacteraceae	Ruegeria	CRISPR-associated protein Cas4	cas4	pfam01930	CAS-I			
2848456..2849461	+	AXF57069.1	CP031092.1	Ruegeria sp. NKC1-1	Bacteria	Proteobacteria	Alphaproteobacteria	Rhodobacterales	Rhodobacteraceae	Ruegeria	type I-B CRISPR-associated endonuclease Cas1	cas1	cd09722	CAS-I-B			
2849476..2849740	+	AXF57070.1	CP031092.1	Ruegeria sp. NKC1-1	Bacteria	Proteobacteria	Alphaproteobacteria	Rhodobacterales	Rhodobacteraceae	Ruegeria	CRISPR-associated endonuclease Cas2	cas2	cd09725	CAS-I,CAS-II,CAS-III,CAS-V			
2849948..2854326	array		CP031092.1	Ruegeria sp. NKC1-1	Bacteria	Proteobacteria	Alphaproteobacteria	Rhodobacterales	Rhodobacteraceae	Ruegeria		CRISPR array			66	36	30

28 CAS-I-B | ONK21387.1

118..2075	array		MSRF01000276.1	Bacillus sp. VT-16-64	Bacteria	Firmicutes	Bacilli	Bacillales	Bacillaceae	Bacillus		CRISPR array			30	36	30
2247..2511	-	ONK21387.1	MSRF01000276.1	Bacillus sp. VT-16-64	Bacteria	Firmicutes	Bacilli	Bacillales	Bacillaceae	Bacillus	CRISPR-associated endonuclease Cas2	cas2	cd09725	CAS-I,CAS-II,CAS-III,CAS-V			
2527..3538	-	ONK21388.1	MSRF01000276.1	Bacillus sp. VT-16-64	Bacteria	Firmicutes	Bacilli	Bacillales	Bacillaceae	Bacillus	subtype I-B CRISPR-associated endonuclease Cas1	cas1	cd09722	CAS-I-B			
3538..4042	-	ONK21518.1	MSRF01000276.1	Bacillus sp. VT-16-64	Bacteria	Firmicutes	Bacilli	Bacillales	Bacillaceae	Bacillus	CRISPR-associated protein Cas4	cas4	pfam01930	CAS-I			
4071..6480	-	ONK21389.1	MSRF01000276.1	Bacillus sp. VT-16-64	Bacteria	Firmicutes	Bacilli	Bacillales	Bacillaceae	Bacillus	hypothetical protein	cas3HD,cas3	cd09641,COG1203	CAS-I			
6467..7145	-	ONK21390.1	MSRF01000276.1	Bacillus sp. VT-16-64	Bacteria	Firmicutes	Bacilli	Bacillales	Bacillaceae	Bacillus	hypothetical protein	cas5	cls001430	CAS-I-B			
7159..8092	-	ONK21519.1	MSRF01000276.1	Bacillus sp. VT-16-64	Bacteria	Firmicutes	Bacilli	Bacillales	Bacillaceae	Bacillus	type I-B CRISPR-associated protein Cas7/Csh2	cas7b	pfam05107	CAS-I-B,CAS-I-C			
8081..10085	-	ONK21391.1	MSRF01000276.1	Bacillus sp. VT-16-64	Bacteria	Firmicutes	Bacilli	Bacillales	Bacillaceae	Bacillus	hypothetical protein	cas8b1	mkCas0074	CAS-I-B			
10109..10853	-	ONK21392.1	MSRF01000276.1	Bacillus sp. VT-16-64	Bacteria	Firmicutes	Bacilli	Bacillales	Bacillaceae	Bacillus	CRISPR-associated endoribonuclease Cas6	cas6	COG1583	CAS-I,CAS-III,CAS-IV			
11537..13227	array		MSRF01000276.1	Bacillus sp. VT-16-64	Bacteria	Firmicutes	Bacilli	Bacillales	Bacillaceae	Bacillus		CRISPR array			26	36	30

29 CAS-I-B | PWA13090.1

113269..121949	array		QCZG01000003.1	Pueribacillus theae	Bacteria	Firmicutes	Bacilli	Bacillales	Bacillaceae	Pueribacillus		CRISPR array			131	36	30
122142..122406	-	PWA13090.1	QCZG01000003.1	Pueribacillus theae	Bacteria	Firmicutes	Bacilli	Bacillales	Bacillaceae	Pueribacillus	CRISPR-associated endonuclease Cas2	cas2	cd09725	CAS-I,CAS-II,CAS-III,CAS-V			
122424..123435	-	PWA13091.1	QCZG01000003.1	Pueribacillus theae	Bacteria	Firmicutes	Bacilli	Bacillales	Bacillaceae	Pueribacillus	subtype I-B CRISPR-associated endonuclease Cas1	cas1	cd09722	CAS-I-B			
123434..123950	-	PWA13092.1	QCZG01000003.1	Pueribacillus theae	Bacteria	Firmicutes	Bacilli	Bacillales	Bacillaceae	Pueribacillus	CRISPR-associated protein Cas4	cas4	pfam01930	CAS-I			
123976..126367	-	PWA13093.1	QCZG01000003.1	Pueribacillus theae	Bacteria	Firmicutes	Bacilli	Bacillales	Bacillaceae	Pueribacillus	CRISPR-associated helicase/endonuclease Cas3	cas3HD,cas3	cd09641,COG1203	CAS-I			
126354..127038	-	PWA13094.1	QCZG01000003.1	Pueribacillus theae	Bacteria	Firmicutes	Bacilli	Bacillales	Bacillaceae	Pueribacillus	CRISPR-associated protein Cas5	cas5	cls001430	CAS-I-B			
127052..128006	-	PWA13095.1	QCZG01000003.1	Pueribacillus theae	Bacteria	Firmicutes	Bacilli	Bacillales	Bacillaceae	Pueribacillus	type I-B CRISPR-associated protein Cas7/Csh2	cas7b	pfam05107	CAS-I-B,CAS-I-C			
127992..129999	-	PWA13096.1	QCZG01000003.1	Pueribacillus theae	Bacteria	Firmicutes	Bacilli	Bacillales	Bacillaceae	Pueribacillus	hypothetical protein	cas8b1	mkCas0074	CAS-I-B			
130021..130798	-	PWA13097.1	QCZG01000003.1	Pueribacillus theae	Bacteria	Firmicutes	Bacilli	Bacillales	Bacillaceae	Pueribacillus	CRISPR-associated endoribonuclease Cas6	cas6	COG1583	CAS-I,CAS-III,CAS-IV			
131387..134261	array		QCZG01000003.1	Pueribacillus theae	Bacteria	Firmicutes	Bacilli	Bacillales	Bacillaceae	Pueribacillus		CRISPR array			44	36	30

30 CAS-I-B | PDO09916.1

26745..27483	+	PDO09911.1	MOXJ01000024.1	Paenibacillaceae bacterium JTherm	Bacteria	Firmicutes	Bacilli	Bacillales	Paenibacillaceae		CRISPR-associated endoribonuclease Cas6	cas6	pfam01881	CAS-I,CAS-III			
28183..30241	+	PDO09912.1	MOXJ01000024.1	Paenibacillaceae bacterium JTherm	Bacteria	Firmicutes	Bacilli	Bacillales	Paenibacillaceae		hypothetical protein	cas8b1	mkCas0074	CAS-I-B			
30243..31203	+	PDO09924.1	MOXJ01000024.1	Paenibacillaceae bacterium JTherm	Bacteria	Firmicutes	Bacilli	Bacillales	Paenibacillaceae		CRISPR-associated protein Csh2	cas7b	pfam05107	CAS-I-B,CAS-I-C			
31216..31906	+	PDO09925.1	MOXJ01000024.1	Paenibacillaceae bacterium JTherm	Bacteria	Firmicutes	Bacilli	Bacillales	Paenibacillaceae		CRISPR-associated protein Cas5	cas5	cd09692	CAS-I-B			
31893..34413	+	PDO09913.1	MOXJ01000024.1	Paenibacillaceae bacterium JTherm	Bacteria	Firmicutes	Bacilli	Bacillales	Paenibacillaceae		CRISPR-associated helicase Cas3'	cas3HD,cas3	cd09641,cd09639	CAS-I			
34477..34996	+	PDO09914.1	MOXJ01000024.1	Paenibacillaceae bacterium JTherm	Bacteria	Firmicutes	Bacilli	Bacillales	Paenibacillaceae		CRISPR-associated protein Cas4	cas4	pfam01930	CAS-I			
34992..36093	+	PDO09915.1	MOXJ01000024.1	Paenibacillaceae bacterium JTherm	Bacteria	Firmicutes	Bacilli	Bacillales	Paenibacillaceae		subtype I-B CRISPR-associated endonuclease Cas1	cas1	cd09634	CAS-I,CAS-II,CAS-III,CAS-V			
36199..36463	+	PDO09916.1	MOXJ01000024.1	Paenibacillaceae bacterium JTherm	Bacteria	Firmicutes	Bacilli	Bacillales	Paenibacillaceae		CRISPR-associated endonuclease Cas2	cas2	cd09725	CAS-I,CAS-II,CAS-III,CAS-V			
36696..41668	array		MOXJ01000024.1	Paenibacillaceae bacterium JTherm	Bacteria	Firmicutes	Bacilli	Bacillales	Paenibacillaceae			CRISPR array			74	37	30

31 CAS-I-B | KIO69860.1

11445..12195	+	KIO69852.1	JXLR01000002.1	Bacillus thermoamylovorans	Bacteria	Firmicutes	Bacilli	Bacillales	Bacillaceae	Bacillus	hypothetical protein	cas6	COG1583	CAS-I,CAS-III,CAS-IV			
13011..14811	+	KIO69853.1	JXLR01000002.1	Bacillus thermoamylovorans	Bacteria	Firmicutes	Bacilli	Bacillales	Bacillaceae	Bacillus	hypothetical protein	cas8b1	cd09730	CAS-I-B			
14812..15769	+	KIO69854.1	JXLR01000002.1	Bacillus thermoamylovorans	Bacteria	Firmicutes	Bacilli	Bacillales	Bacillaceae	Bacillus	hypothetical protein	cas7	cd09690	CAS-I-B			
15782..16565	+	KIO69855.1	JXLR01000002.1	Bacillus thermoamylovorans	Bacteria	Firmicutes	Bacilli	Bacillales	Bacillaceae	Bacillus	hypothetical protein	cas5	cls001430	CAS-I-B			
16561..18937	+	KIO69856.1	JXLR01000002.1	Bacillus thermoamylovorans	Bacteria	Firmicutes	Bacilli	Bacillales	Bacillaceae	Bacillus	hypothetical protein	cas3HD,cas3	cd09641,COG1203	CAS-I			
19511..19970	+	KIO69857.1	JXLR01000002.1	Bacillus thermoamylovorans	Bacteria	Firmicutes	Bacilli	Bacillales	Bacillaceae	Bacillus	hypothetical protein	cas1,cas1	cd09722,cd09722	CAS-I-B			
20528..20693	+	KIO69858.1	JXLR01000002.1	Bacillus thermoamylovorans	Bacteria	Firmicutes	Bacilli	Bacillales	Bacillaceae	Bacillus	hypothetical protein						
20883..21150	+	KIO69859.1	JXLR01000002.1	Bacillus thermoamylovorans	Bacteria	Firmicutes	Bacilli	Bacillales	Bacillaceae	Bacillus	hypothetical protein	cas1	cd09722	CAS-I-B			
21563..21827	+	KIO69860.1	JXLR01000002.1	Bacillus thermoamylovorans	Bacteria	Firmicutes	Bacilli	Bacillales	Bacillaceae	Bacillus	hypothetical protein	cas2	cd09725	CAS-I,CAS-II,CAS-III,CAS-V			
22251..22411	array		JXLR01000002.1	Bacillus thermoamylovorans	Bacteria	Firmicutes	Bacilli	Bacillales	Bacillaceae	Bacillus		CRISPR array			3	34	31
23020..23353	+	KIO69861.1	JXLR01000002.1	Bacillus thermoamylovorans	Bacteria	Firmicutes	Bacilli	Bacillales	Bacillaceae	Bacillus	hypothetical protein	csx1	pfam09455	CAS-III			
23373..23766	+	KIO69862.1	JXLR01000002.1	Bacillus thermoamylovorans	Bacteria	Firmicutes	Bacilli	Bacillales	Bacillaceae	Bacillus	hypothetical protein	csx20	xls002255	CAS-I,CAS-III			
24047..24437	+	KIO69863.1	JXLR01000002.1	Bacillus thermoamylovorans	Bacteria	Firmicutes	Bacilli	Bacillales	Bacillaceae	Bacillus	hypothetical protein	csx20	cls000023	CAS-I,CAS-III			
24503..24896	+	KIO69864.1	JXLR01000002.1	Bacillus thermoamylovorans	Bacteria	Firmicutes	Bacilli	Bacillales	Bacillaceae	Bacillus	hypothetical protein	csx1	cd09732	CAS-III			

32 CAS-I-B | PAC34137.1

14985..15741	+	PAC34133.1	NJGA01000053.1	Bacillus hisashii	Bacteria	Firmicutes	Bacilli	Bacillales	Bacillaceae	Bacillus	CRISPR-associated endoribonuclease Cas6	cas6	COG1583	CAS-I,CAS-III,CAS-IV			
17828..18782	+	PAC34139.1	NJGA01000053.1	Bacillus hisashii	Bacteria	Firmicutes	Bacilli	Bacillales	Bacillaceae	Bacillus	CRISPR-associated protein	cas7b	pfam05107	CAS-I-B,CAS-I-C			
18791..19472	+	PAC34134.1	NJGA01000053.1	Bacillus hisashii	Bacteria	Firmicutes	Bacilli	Bacillales	Bacillaceae	Bacillus	CRISPR-associated protein Cas5	cas5	cls001430	CAS-I-B			
19459..21793	+	PAC34135.1	NJGA01000053.1	Bacillus hisashii	Bacteria	Firmicutes	Bacilli	Bacillales	Bacillaceae	Bacillus	CRISPR-associated helicase/endonuclease Cas3	cas3HD,cas3	cd09641,COG1203	CAS-I			
21786..22302	+	PAC34136.1	NJGA01000053.1	Bacillus hisashii	Bacteria	Firmicutes	Bacilli	Bacillales	Bacillaceae	Bacillus	CRISPR-associated protein Cas4	cas4	pfam01930	CAS-I			
23884..24148	+	PAC34137.1	NJGA01000053.1	Bacillus hisashii	Bacteria	Firmicutes	Bacilli	Bacillales	Bacillaceae	Bacillus	CRISPR-associated endonuclease Cas2	cas2	COG1343	CAS-I,CAS-II,CAS-III			
24555..25647	array		NJGA01000053.1	Bacillus hisashii	Bacteria	Firmicutes	Bacilli	Bacillales	Bacillaceae	Bacillus		CRISPR array			17	36	30

33 CAS-I-B | OBY76623.1

223..487	-	OBY76623.1	MAIS01000133.1	Paenibacillus sp. KS1	Bacteria	Firmicutes	Bacilli	Bacillales	Paenibacillaceae	Paenibacillus	CRISPR-associated endonuclease Cas2	cas2	cd09725	CAS-I,CAS-II,CAS-III,CAS-V			
495..1497	-	OBY76617.1	MAIS01000133.1	Paenibacillus sp. KS1	Bacteria	Firmicutes	Bacilli	Bacillales	Paenibacillaceae	Paenibacillus	subtype I-B CRISPR-associated endonuclease Cas1	cas1	cd09722	CAS-I-B			
1500..1998	-	OBY76624.1	MAIS01000133.1	Paenibacillus sp. KS1	Bacteria	Firmicutes	Bacilli	Bacillales	Paenibacillaceae	Paenibacillus	CRISPR-associated protein Cas4	cas4	pfam01930	CAS-I			
2021..4358	-	OBY76618.1	MAIS01000133.1	Paenibacillus sp. KS1	Bacteria	Firmicutes	Bacilli	Bacillales	Paenibacillaceae	Paenibacillus	CRISPR-associated helicase/endonuclease Cas3	cas3HD,cas3	cd09641,COG1203	CAS-I			
4345..5029	-	OBY76625.1	MAIS01000133.1	Paenibacillus sp. KS1	Bacteria	Firmicutes	Bacilli	Bacillales	Paenibacillaceae	Paenibacillus	CRISPR-associated protein Cas5	cas5	cls001430	CAS-I-B			
5038..5998	-	OBY76619.1	MAIS01000133.1	Paenibacillus sp. KS1	Bacteria	Firmicutes	Bacilli	Bacillales	Paenibacillaceae	Paenibacillus	CRISPR-associated protein Csh2	cas7b	pfam05107	CAS-I-B,CAS-I-C			
6002..7994	-	OBY76620.1	MAIS01000133.1	Paenibacillus sp. KS1	Bacteria	Firmicutes	Bacilli	Bacillales	Paenibacillaceae	Paenibacillus	hypothetical protein	cas8b1	mkCas0074	CAS-I-B			
8006..8759	-	OBY76621.1	MAIS01000133.1	Paenibacillus sp. KS1	Bacteria	Firmicutes	Bacilli	Bacillales	Paenibacillaceae	Paenibacillus	CRISPR-associated endoribonuclease Cas6	cas6	COG1583	CAS-I,CAS-III,CAS-IV			

34 CAS-I-B | GAC90887.1

156849..158036	array		BARH01000009.1	Anoxybacillus flavithermus NBRC 109594	Bacteria	Firmicutes	Bacilli	Bacillales	Bacillaceae	Anoxybacillus		CRISPR array			18	38	30
158074..158698	-	GAC90880.1	BARH01000009.1	Anoxybacillus flavithermus NBRC 109594	Bacteria	Firmicutes	Bacilli	Bacillales	Bacillaceae	Anoxybacillus	thermonuclease	C3a212	cluster212				
159046..160972	+	GAC90881.1	BARH01000009.1	Anoxybacillus flavithermus NBRC 109594	Bacteria	Firmicutes	Bacilli	Bacillales	Bacillaceae	Anoxybacillus	CRISPR-associated protein Csh1 domain-containing protein	cas8b1	pfam09484	CAS-I-B			
160996..161932	+	GAC90882.1	BARH01000009.1	Anoxybacillus flavithermus NBRC 109594	Bacteria	Firmicutes	Bacilli	Bacillales	Bacillaceae	Anoxybacillus	CRISPR-associated protein Csh2	cas7b	pfam05107	CAS-I-B,CAS-I-C			
161947..162682	+	GAC90883.1	BARH01000009.1	Anoxybacillus flavithermus NBRC 109594	Bacteria	Firmicutes	Bacilli	Bacillales	Bacillaceae	Anoxybacillus	CRISPR-associated protein Cas6	cas5	cd09692	CAS-I-B			
162729..165213	+	GAC90884.1	BARH01000009.1	Anoxybacillus flavithermus NBRC 109594	Bacteria	Firmicutes	Bacilli	Bacillales	Bacillaceae	Anoxybacillus	CRISPR-associated HD domain-containing protein	cas3HD,cas3	cd09641,COG1203	CAS-I			
165212..165710	+	GAC90885.1	BARH01000009.1	Anoxybacillus flavithermus NBRC 109594	Bacteria	Firmicutes	Bacilli	Bacillales	Bacillaceae	Anoxybacillus	CRISPR-associated protein Cas4	cas4	pfam01930	CAS-I			
165712..166720	+	GAC90886.1	BARH01000009.1	Anoxybacillus flavithermus NBRC 109594	Bacteria	Firmicutes	Bacilli	Bacillales	Bacillaceae	Anoxybacillus	CRISPR-associated protein Cas1	cas1	cd09722	CAS-I-B			
166730..166994	+	GAC90887.1	BARH01000009.1	Anoxybacillus flavithermus NBRC 109594	Bacteria	Firmicutes	Bacilli	Bacillales	Bacillaceae	Anoxybacillus	CRISPR-associated protein Cas2	cas2	cd09725	CAS-I,CAS-II,CAS-III,CAS-V			
167008..167758	+	GAC90888.1	BARH01000009.1	Anoxybacillus flavithermus NBRC 109594	Bacteria	Firmicutes	Bacilli	Bacillales	Bacillaceae	Anoxybacillus	CRISPR-associated protein Cas6	cas6	COG1583	CAS-I,CAS-III,CAS-IV			
167940..169450	array		BARH01000009.1	Anoxybacillus flavithermus NBRC 109594	Bacteria	Firmicutes	Bacilli	Bacillales	Bacillaceae	Anoxybacillus		CRISPR array			23	37	30

35 CAS-I-B | ADP76105.1

3431460..3432954	array		CP002293.1	Geobacillus sp. Y4.1MC1	Bacteria	Firmicutes	Bacilli	Bacillales	Bacillaceae	Geobacillus		CRISPR array			23	36	30
3433136..3433883	-	ADP76104.1	CP002293.1	Geobacillus sp. Y4.1MC1	Bacteria	Firmicutes	Bacilli	Bacillales	Bacillaceae	Geobacillus	CRISPR-associated protein Cas6	cas6	COG1583	CAS-I,CAS-III,CAS-IV			
3434157..3434421	-	ADP76105.1	CP002293.1	Geobacillus sp. Y4.1MC1	Bacteria	Firmicutes	Bacilli	Bacillales	Bacillaceae	Geobacillus	CRISPR-associated protein Cas2	cas2	cd09725	CAS-I,CAS-II,CAS-III,CAS-V			
3434430..3435438	-	ADP76106.1	CP002293.1	Geobacillus sp. Y4.1MC1	Bacteria	Firmicutes	Bacilli	Bacillales	Bacillaceae	Geobacillus	CRISPR-associated protein Cas1	cas1	cd09722	CAS-I-B			
3435440..3435938	-	ADP76107.1	CP002293.1	Geobacillus sp. Y4.1MC1	Bacteria	Firmicutes	Bacilli	Bacillales	Bacillaceae	Geobacillus	CRISPR-associated protein Cas4	cas4	pfam01930	CAS-I			
3435937..3438421	-	ADP76108.1	CP002293.1	Geobacillus sp. Y4.1MC1	Bacteria	Firmicutes	Bacilli	Bacillales	Bacillaceae	Geobacillus	CRISPR-associated HD domain protein	cas3HD,cas3	cd09641,COG1203	CAS-I			
3438465..3439200	-	ADP76109.1	CP002293.1	Geobacillus sp. Y4.1MC1	Bacteria	Firmicutes	Bacilli	Bacillales	Bacillaceae	Geobacillus	CRISPR-associated protein Cas5, Hmari subtype	cas5	cls001430	CAS-I-B			
3439214..3440150	-	ADP76110.1	CP002293.1	Geobacillus sp. Y4.1MC1	Bacteria	Firmicutes	Bacilli	Bacillales	Bacillaceae	Geobacillus	CRISPR-associated protein, Csh2 family	cas7b	pfam05107	CAS-I-B,CAS-I-C			
3440312..3442283	-	ADP76111.1	CP002293.1	Geobacillus sp. Y4.1MC1	Bacteria	Firmicutes	Bacilli	Bacillales	Bacillaceae	Geobacillus	CRISPR-associated protein, Csh1 family	cas8b1	pfam09484	CAS-I-B			
3442782..3443538	-	ADP76112.1	CP002293.1	Geobacillus sp. Y4.1MC1	Bacteria	Firmicutes	Bacilli	Bacillales	Bacillaceae	Geobacillus	transcriptional regulator, DeoR family	WYL	COG2378	CAS-I,CAS-III			

36 CAS-I-B | ANB64529.1

3218596..3220561	+	ANB62924.1	CP015436.1	Anoxybacillus sp. B7M1	Bacteria	Firmicutes	Bacilli	Bacillales	Bacillaceae	Anoxybacillus	CRISPR-associated family protein	cas8b1	pfam09484	CAS-I-B			
3220560..3221496	+	ANB63065.1	CP015436.1	Anoxybacillus sp. B7M1	Bacteria	Firmicutes	Bacilli	Bacillales	Bacillaceae	Anoxybacillus	CRISPR-associated, family protein	cas7b	pfam05107	CAS-I-B,CAS-I-C			
3221510..3222245	+	ANB65481.1	CP015436.1	Anoxybacillus sp. B7M1	Bacteria	Firmicutes	Bacilli	Bacillales	Bacillaceae	Anoxybacillus	CRISPR-associated Cas5 domain protein	cas5	cd09692	CAS-I-B			
3222289..3224773	+	ANB63882.1	CP015436.1	Anoxybacillus sp. B7M1	Bacteria	Firmicutes	Bacilli	Bacillales	Bacillaceae	Anoxybacillus	CRISPR-associated endonuclease Cas3-HD	cas3HD,cas3	cd09641,COG1203	CAS-I			
3224772..3225273	+	ANB63700.1	CP015436.1	Anoxybacillus sp. B7M1	Bacteria	Firmicutes	Bacilli	Bacillales	Bacillaceae	Anoxybacillus	CRISPR-associated protein Cas4	cas4	pfam01930	CAS-I			
3225272..3226280	+	ANB62834.1	CP015436.1	Anoxybacillus sp. B7M1	Bacteria	Firmicutes	Bacilli	Bacillales	Bacillaceae	Anoxybacillus	CRISPR-associated endonuclease Cas1	cas1	cd09722	CAS-I-B			
3226288..3226552	+	ANB64529.1	CP015436.1	Anoxybacillus sp. B7M1	Bacteria	Firmicutes	Bacilli	Bacillales	Bacillaceae	Anoxybacillus	CRISPR-associated endonuclease Cas2	cas2	COG1343	CAS-I,CAS-II,CAS-III			
3226561..3227308	+	ANB64734.1	CP015436.1	Anoxybacillus sp. B7M1	Bacteria	Firmicutes	Bacilli	Bacillales	Bacillaceae	Anoxybacillus	CRISPR-associated endoribonuclease Cas6	cas6	COG1583	CAS-I,CAS-III,CAS-IV			

37 CAS-I-B | KYD32438.1

1305..3132	array		LQYW01000013.1	Parageobacillus toebii	Bacteria	Firmicutes	Bacilli	Bacillales	Bacillaceae	Parageobacillus		CRISPR array			28	36	30
2176..2302	-	KYD32435.1	LQYW01000013.1	Parageobacillus toebii	Bacteria	Firmicutes	Bacilli	Bacillales	Bacillaceae	Parageobacillus	hypothetical protein						
2518..2647	-	KYD32436.1	LQYW01000013.1	Parageobacillus toebii	Bacteria	Firmicutes	Bacilli	Bacillales	Bacillaceae	Parageobacillus	hypothetical protein						
3314..4061	-	KYD32437.1	LQYW01000013.1	Parageobacillus toebii	Bacteria	Firmicutes	Bacilli	Bacillales	Bacillaceae	Parageobacillus	hypothetical protein	cas6	COG1583	CAS-I,CAS-III,CAS-IV			
4305..4569	-	KYD32438.1	LQYW01000013.1	Parageobacillus toebii	Bacteria	Firmicutes	Bacilli	Bacillales	Bacillaceae	Parageobacillus	hypothetical protein	cas2	cd09725	CAS-I,CAS-II,CAS-III,CAS-V			
4577..5585	-	KYD32439.1	LQYW01000013.1	Parageobacillus toebii	Bacteria	Firmicutes	Bacilli	Bacillales	Bacillaceae	Parageobacillus	hypothetical protein	cas1	cd09722	CAS-I-B			
5584..6097	-	KYD32440.1	LQYW01000013.1	Parageobacillus toebii	Bacteria	Firmicutes	Bacilli	Bacillales	Bacillaceae	Parageobacillus	hypothetical protein	cas4	pfam01930	CAS-I			
6084..8568	-	KYD32441.1	LQYW01000013.1	Parageobacillus toebii	Bacteria	Firmicutes	Bacilli	Bacillales	Bacillaceae	Parageobacillus	hypothetical protein	cas3HD,cas3	cd09641,COG1203	CAS-I			
8612..9347	-	KYD32442.1	LQYW01000013.1	Parageobacillus toebii	Bacteria	Firmicutes	Bacilli	Bacillales	Bacillaceae	Parageobacillus	hypothetical protein	cas5	cls001430	CAS-I-B			
9361..10297	-	KYD32443.1	LQYW01000013.1	Parageobacillus toebii	Bacteria	Firmicutes	Bacilli	Bacillales	Bacillaceae	Parageobacillus	hypothetical protein	cas7b	pfam05107	CAS-I-B,CAS-I-C			
10459..12430	-	KYD32444.1	LQYW01000013.1	Parageobacillus toebii	Bacteria	Firmicutes	Bacilli	Bacillales	Bacillaceae	Parageobacillus	hypothetical protein	cas8b1	pfam09484	CAS-I-B			
13462..15871	array		LQYW01000013.1	Parageobacillus toebii	Bacteria	Firmicutes	Bacilli	Bacillales	Bacillaceae	Parageobacillus		CRISPR array			37	37	29

38 CAS-I-B | SFA45838.1

39843..41150	array		FOJS01000010.1	Parageobacillus thermantarcticus	Bacteria	Firmicutes	Bacilli	Bacillales	Bacillaceae	Parageobacillus		CRISPR array			20	37	30
41489..41621	+	SFA45808.1	FOJS01000010.1	Parageobacillus thermantarcticus	Bacteria	Firmicutes	Bacilli	Bacillales	Bacillaceae	Parageobacillus	hypothetical protein						
41787..43764	+	SFA45811.1	FOJS01000010.1	Parageobacillus thermantarcticus	Bacteria	Firmicutes	Bacilli	Bacillales	Bacillaceae	Parageobacillus	CRISPR-associated protein, Csh1 family	cas8b1	pfam09484	CAS-I-B			
43903..44842	+	SFA45817.1	FOJS01000010.1	Parageobacillus thermantarcticus	Bacteria	Firmicutes	Bacilli	Bacillales	Bacillaceae	Parageobacillus	CRISPR-associated protein, Csh2 family	cas7b	pfam05107	CAS-I-B,CAS-I-C			
44856..45591	+	SFA45820.1	FOJS01000010.1	Parageobacillus thermantarcticus	Bacteria	Firmicutes	Bacilli	Bacillales	Bacillaceae	Parageobacillus	CRISPR-associated protein Cas5h	cas5	cls001430	CAS-I-B			
45635..48122	+	SFA45824.1	FOJS01000010.1	Parageobacillus thermantarcticus	Bacteria	Firmicutes	Bacilli	Bacillales	Bacillaceae	Parageobacillus	CRISPR-associated helicase, Cas3 family	cas3HD,cas3	cd09641,COG1203	CAS-I			
48121..48619	+	SFA45830.1	FOJS01000010.1	Parageobacillus thermantarcticus	Bacteria	Firmicutes	Bacilli	Bacillales	Bacillaceae	Parageobacillus	CRISPR-associated exonuclease Cas4	cas4	pfam01930	CAS-I			
48621..49629	+	SFA45833.1	FOJS01000010.1	Parageobacillus thermantarcticus	Bacteria	Firmicutes	Bacilli	Bacillales	Bacillaceae	Parageobacillus	CRISP-associated protein Cas1	cas1	cd09722	CAS-I-B			
49638..49902	+	SFA45838.1	FOJS01000010.1	Parageobacillus thermantarcticus	Bacteria	Firmicutes	Bacilli	Bacillales	Bacillaceae	Parageobacillus	CRISPR-associated protein Cas2	cas2	COG1343	CAS-I,CAS-II,CAS-III			
50254..51001	+	SFA45843.1	FOJS01000010.1	Parageobacillus thermantarcticus	Bacteria	Firmicutes	Bacilli	Bacillales	Bacillaceae	Parageobacillus	CRISPR-associated endoribonuclease Cas6	cas6	COG1583	CAS-I,CAS-III,CAS-IV			
53153..54227	+	SFA45846.1	FOJS01000010.1	Parageobacillus thermantarcticus	Bacteria	Firmicutes	Bacilli	Bacillales	Bacillaceae	Parageobacillus	spermidine/putrescine transport system ATP-binding protein	Cas_RecF	cluster17				

39 CAS-I-B | ACS23238.1

348817..350124	array		CP001638.1	Geobacillus sp. WCH70	Bacteria	Firmicutes	Bacilli	Bacillales	Bacillaceae	Geobacillus		CRISPR array			20	37	30
350423..352145	+	ACS23232.1	CP001638.1	Geobacillus sp. WCH70	Bacteria	Firmicutes	Bacilli	Bacillales	Bacillaceae	Geobacillus	CRISPR-associated protein, TM1802 family	cas8b1	cd09730	CAS-I-B			
352146..353103	+	ACS23233.1	CP001638.1	Geobacillus sp. WCH70	Bacteria	Firmicutes	Bacilli	Bacillales	Bacillaceae	Geobacillus	CRISPR-associated protein, Csh2 family	cas7	cd09690	CAS-I-B			
353118..353856	+	ACS23234.1	CP001638.1	Geobacillus sp. WCH70	Bacteria	Firmicutes	Bacilli	Bacillales	Bacillaceae	Geobacillus	CRISPR-associated protein Cas5, Hmari subtype	cas5	cls001430	CAS-I-B			
353860..356194	+	ACS23235.1	CP001638.1	Geobacillus sp. WCH70	Bacteria	Firmicutes	Bacilli	Bacillales	Bacillaceae	Geobacillus	CRISPR-associated helicase Cas3	cas3HD,cas3	cd09641,COG1203	CAS-I			
356215..356713	+	ACS23236.1	CP001638.1	Geobacillus sp. WCH70	Bacteria	Firmicutes	Bacilli	Bacillales	Bacillaceae	Geobacillus	CRISPR-associated protein Cas4	cas4	pfam01930	CAS-I			
356715..357717	+	ACS23237.1	CP001638.1	Geobacillus sp. WCH70	Bacteria	Firmicutes	Bacilli	Bacillales	Bacillaceae	Geobacillus	CRISPR-associated protein Cas1	cas1	cd09722	CAS-I-B			
357726..357990	+	ACS23238.1	CP001638.1	Geobacillus sp. WCH70	Bacteria	Firmicutes	Bacilli	Bacillales	Bacillaceae	Geobacillus	CRISPR-associated protein Cas2	cas2	cd09725	CAS-I,CAS-II,CAS-III,CAS-V			
358004..358754	+	ACS23239.1	CP001638.1	Geobacillus sp. WCH70	Bacteria	Firmicutes	Bacilli	Bacillales	Bacillaceae	Geobacillus	CRISPR-associated protein Cas6	cas6	COG1583	CAS-I,CAS-III,CAS-IV			
358937..361432	array		CP001638.1	Geobacillus sp. WCH70	Bacteria	Firmicutes	Bacilli	Bacillales	Bacillaceae	Geobacillus		CRISPR array			38	36	30

40 CAS-I-B | OAT71453.1

116443..117202	array		LXMA01000043.1	Parageobacillus thermoglucosidasius	Bacteria	Firmicutes	Bacilli	Bacillales	Bacillaceae	Parageobacillus		CRISPR array			12	36	30
117386..118136	-	OAT71403.1	LXMA01000043.1	Parageobacillus thermoglucosidasius	Bacteria	Firmicutes	Bacilli	Bacillales	Bacillaceae	Parageobacillus	CRISPR-associated endoribonuclease Cas6	cas6	COG1583	CAS-I,CAS-III,CAS-IV			
118150..118414	-	OAT71453.1	LXMA01000043.1	Parageobacillus thermoglucosidasius	Bacteria	Firmicutes	Bacilli	Bacillales	Bacillaceae	Parageobacillus	CRISPR-associated endonuclease Cas2	cas2	cd09725	CAS-I,CAS-II,CAS-III,CAS-V			
118423..119422	-	OAT71404.1	LXMA01000043.1	Parageobacillus thermoglucosidasius	Bacteria	Firmicutes	Bacilli	Bacillales	Bacillaceae	Parageobacillus	subtype I-B CRISPR-associated endonuclease Cas1	cas1	cd09722	CAS-I-B			
119424..119934	-	OAT71454.1	LXMA01000043.1	Parageobacillus thermoglucosidasius	Bacteria	Firmicutes	Bacilli	Bacillales	Bacillaceae	Parageobacillus	CRISPR-associated protein Cas4	cas4	pfam01930	CAS-I			
119943..122277	-	OAT71405.1	LXMA01000043.1	Parageobacillus thermoglucosidasius	Bacteria	Firmicutes	Bacilli	Bacillales	Bacillaceae	Parageobacillus	CRISPR-associated helicase/endonuclease Cas3	cas3HD,cas3	cd09641,COG1203	CAS-I			
122281..123019	-	OAT71406.1	LXMA01000043.1	Parageobacillus thermoglucosidasius	Bacteria	Firmicutes	Bacilli	Bacillales	Bacillaceae	Parageobacillus	type I-B CRISPR-associated protein Cas5	cas5	cls001430	CAS-I-B			
123034..123991	-	OAT71407.1	LXMA01000043.1	Parageobacillus thermoglucosidasius	Bacteria	Firmicutes	Bacilli	Bacillales	Bacillaceae	Parageobacillus	type I-B CRISPR-associated protein Cas7/Csh2	cas7	cd09690	CAS-I-B			
123992..125711	-	OAT71455.1	LXMA01000043.1	Parageobacillus thermoglucosidasius	Bacteria	Firmicutes	Bacilli	Bacillales	Bacillaceae	Parageobacillus	type I-B CRISPR-associated protein Cas8b/Csh1	cas8b1	cd09730	CAS-I-B			
126013..126448	array		LXMA01000043.1	Parageobacillus thermoglucosidasius	Bacteria	Firmicutes	Bacilli	Bacillales	Bacillaceae	Parageobacillus		CRISPR array			7	37	30
41 CAS-I-B | GAD15322.1																	
524..2240	+	GAD15315.1	BASG01000069.1	Geobacillus kaustophilus GBlys	Bacteria	Firmicutes	Bacilli	Bacillales	Bacillaceae	Geobacillus	CRISPR-associated protein	cas8b1	pfam09484	CAS-I-B			
2244..3204	+	GAD15316.1	BASG01000069.1	Geobacillus kaustophilus GBlys	Bacteria	Firmicutes	Bacilli	Bacillales	Bacillaceae	Geobacillus	csh2 family CRISPR-associated protein	cas7	cd09690	CAS-I-B			
3207..3960	+	GAD15317.1	BASG01000069.1	Geobacillus kaustophilus GBlys	Bacteria	Firmicutes	Bacilli	Bacillales	Bacillaceae	Geobacillus	CRISPR-associated protein Cas5	cas5	cls001430	CAS-I-B			
4066..4189	-	GAD15318.1	BASG01000069.1	Geobacillus kaustophilus GBlys	Bacteria	Firmicutes	Bacilli	Bacillales	Bacillaceae	Geobacillus	hypothetical protein						
4216..6292	+	GAD15319.1	BASG01000069.1	Geobacillus kaustophilus GBlys	Bacteria	Firmicutes	Bacilli	Bacillales	Bacillaceae	Geobacillus	CRISPR-associated helicase Cas3	cas3HD,cas3	cd09641,COG1203	CAS-I			
6301..6811	+	GAD15320.1	BASG01000069.1	Geobacillus kaustophilus GBlys	Bacteria	Firmicutes	Bacilli	Bacillales	Bacillaceae	Geobacillus	CRISPR-associated protein Cas4	cas4	pfam01930	CAS-I			
6813..7815	+	GAD15321.1	BASG01000069.1	Geobacillus kaustophilus GBlys	Bacteria	Firmicutes	Bacilli	Bacillales	Bacillaceae	Geobacillus	CRISPR-associated protein Cas1	cas1	cd09722	CAS-I-B			
7831..8089	+	GAD15322.1	BASG01000069.1	Geobacillus kaustophilus GBlys	Bacteria	Firmicutes	Bacilli	Bacillales	Bacillaceae	Geobacillus	CRISPR-associated protein Cas2	cas2	cd09725	CAS-I,CAS-II,CAS-III,CAS-V			
8147..8876	+	GAD15323.1	BASG01000069.1	Geobacillus kaustophilus GBlys	Bacteria	Firmicutes	Bacilli	Bacillales	Bacillaceae	Geobacillus	CRISPR-associated protein Cas6	cas6	COG1583	CAS-I,CAS-III,CAS-IV			
8972..9095	+	GAD15324.1	BASG01000069.1	Geobacillus kaustophilus GBlys	Bacteria	Firmicutes	Bacilli	Bacillales	Bacillaceae	Geobacillus	hypothetical protein						
9061..10211	array		BASG01000069.1	Geobacillus kaustophilus GBlys	Bacteria	Firmicutes	Bacilli	Bacillales	Bacillaceae	Geobacillus		CRISPR array			18	35	30
42 CAS-I-B | ALA70693.1																	
2111171..2111660	array		CP008934.1	Geobacillus stearothermophilus 10	Bacteria	Firmicutes	Bacilli	Bacillales	Bacillaceae	Geobacillus		CRISPR array			8	35	30
2111858..2113517	+	ALA70686.1	CP008934.1	Geobacillus stearothermophilus 10	Bacteria	Firmicutes	Bacilli	Bacillales	Bacillaceae	Geobacillus	transposase						
2113732..2115448	+	ALA70687.1	CP008934.1	Geobacillus stearothermophilus 10	Bacteria	Firmicutes	Bacilli	Bacillales	Bacillaceae	Geobacillus	CRISPR-associated protein	cas8b1	cd09730	CAS-I-B			
2115449..2116406	+	ALA70688.1	CP008934.1	Geobacillus stearothermophilus 10	Bacteria	Firmicutes	Bacilli	Bacillales	Bacillaceae	Geobacillus	CRISPR-associated protein Csh2	cas7	cd09690	CAS-I-B			
2116421..2117165	+	ALA70689.1	CP008934.1	Geobacillus stearothermophilus 10	Bacteria	Firmicutes	Bacilli	Bacillales	Bacillaceae	Geobacillus	CRISPR-associated protein Cas5	cas5	cls001430	CAS-I-B			
2117166..2119497	+	ALA70690.1	CP008934.1	Geobacillus stearothermophilus 10	Bacteria	Firmicutes	Bacilli	Bacillales	Bacillaceae	Geobacillus	CRISPR-associated protein Cas3	cas3HD,cas3	cd09641,COG1203	CAS-I			
2119506..2120016	+	ALA70691.1	CP008934.1	Geobacillus stearothermophilus 10	Bacteria	Firmicutes	Bacilli	Bacillales	Bacillaceae	Geobacillus	CRISPR-associated protein Cas4	cas4	pfam01930	CAS-I			
2120019..2121021	+	ALA70692.1	CP008934.1	Geobacillus stearothermophilus 10	Bacteria	Firmicutes	Bacilli	Bacillales	Bacillaceae	Geobacillus	CRISPR-associated protein Cas1	cas1	cd09722	CAS-I-B			
2121031..2121295	+	ALA70693.1	CP008934.1	Geobacillus stearothermophilus 10	Bacteria	Firmicutes	Bacilli	Bacillales	Bacillaceae	Geobacillus	CRISPR-associated protein Cas2	cas2	COG1343	CAS-I,CAS-II,CAS-III			
2121331..2122078	+	ALA70694.1	CP008934.1	Geobacillus stearothermophilus 10	Bacteria	Firmicutes	Bacilli	Bacillales	Bacillaceae	Geobacillus	CRISPR-associated protein Cas6	cas6	COG1583	CAS-I,CAS-III,CAS-IV			
2122259..2123356	array		CP008934.1	Geobacillus stearothermophilus 10	Bacteria	Firmicutes	Bacilli	Bacillales	Bacillaceae	Geobacillus		CRISPR array			17	36	30
43 CAS-I-B | KYD25558.1																	
26120..27895	array		LQYX01000092.1	Geobacillus sp. B4113_201601	Bacteria	Firmicutes	Bacilli	Bacillales	Bacillaceae	Geobacillus		CRISPR array			27	37	30
26702..26843	-	KYD25553.1	LQYX01000092.1	Geobacillus sp. B4113_201601	Bacteria	Firmicutes	Bacilli	Bacillales	Bacillaceae	Geobacillus	hypothetical protein						
26797..27037	-	KYD25554.1	LQYX01000092.1	Geobacillus sp. B4113_201601	Bacteria	Firmicutes	Bacilli	Bacillales	Bacillaceae	Geobacillus	hypothetical protein						
27198..27333	-	KYD25555.1	LQYX01000092.1	Geobacillus sp. B4113_201601	Bacteria	Firmicutes	Bacilli	Bacillales	Bacillaceae	Geobacillus	hypothetical protein						
27600..27729	-	KYD25556.1	LQYX01000092.1	Geobacillus sp. B4113_201601	Bacteria	Firmicutes	Bacilli	Bacillales	Bacillaceae	Geobacillus	hypothetical protein						
28073..28820	-	KYD25557.1	LQYX01000092.1	Geobacillus sp. B4113_201601	Bacteria	Firmicutes	Bacilli	Bacillales	Bacillaceae	Geobacillus	hypothetical protein	cas6	COG1583	CAS-I,CAS-III,CAS-IV			
28992..29256	-	KYD25558.1	LQYX01000092.1	Geobacillus sp. B4113_201601	Bacteria	Firmicutes	Bacilli	Bacillales	Bacillaceae	Geobacillus	hypothetical protein	cas2	cd09725	CAS-I,CAS-II,CAS-III,CAS-V			
29267..30269	-	KYD25559.1	LQYX01000092.1	Geobacillus sp. B4113_201601	Bacteria	Firmicutes	Bacilli	Bacillales	Bacillaceae	Geobacillus	hypothetical protein	cas1	cd09722	CAS-I-B			
30271..30781	-	KYD25560.1	LQYX01000092.1	Geobacillus sp. B4113_201601	Bacteria	Firmicutes	Bacilli	Bacillales	Bacillaceae	Geobacillus	hypothetical protein	cas4	pfam01930	CAS-I			
30790..33124	-	KYD25561.1	LQYX01000092.1	Geobacillus sp. B4113_201601	Bacteria	Firmicutes	Bacilli	Bacillales	Bacillaceae	Geobacillus	hypothetical protein	cas3HD,cas3	cd09641,COG1203	CAS-I			
33125..33869	-	KYD25562.1	LQYX01000092.1	Geobacillus sp. B4113_201601	Bacteria	Firmicutes	Bacilli	Bacillales	Bacillaceae	Geobacillus	hypothetical protein	cas5	cls001430	CAS-I-B			
33884..34841	-	KYD25563.1	LQYX01000092.1	Geobacillus sp. B4113_201601	Bacteria	Firmicutes	Bacilli	Bacillales	Bacillaceae	Geobacillus	hypothetical protein	cas7	cd09690	CAS-I-B			
34842..35856	-	KYD25564.1	LQYX01000092.1	Geobacillus sp. B4113_201601	Bacteria	Firmicutes	Bacilli	Bacillales	Bacillaceae	Geobacillus	hypothetical protein	cas8b1	pfam09484	CAS-I-B			
44 CAS-I-B | AMX84678.1																	
2888741..2889563	array		CP014342.1	Geobacillus subterraneus	Bacteria	Firmicutes	Bacilli	Bacillales	Bacillaceae	Geobacillus		CRISPR array			13	36	30
2889742..2890489	-	AMX84676.1	CP014342.1	Geobacillus subterraneus	Bacteria	Firmicutes	Bacilli	Bacillales	Bacillaceae	Geobacillus	CRISPR-associated protein Cas6	cas6	COG1583	CAS-I,CAS-III,CAS-IV			
2890579..2891827	-	AMX84677.1	CP014342.1	Geobacillus subterraneus	Bacteria	Firmicutes	Bacilli	Bacillales	Bacillaceae	Geobacillus	transposase	cas14u	cas14u	CAS-V			
2891987..2892251	-	AMX84678.1	CP014342.1	Geobacillus subterraneus	Bacteria	Firmicutes	Bacilli	Bacillales	Bacillaceae	Geobacillus	CRISPR-associated protein Cas2	cas2	cd09725	CAS-I,CAS-II,CAS-III,CAS-V			
2892262..2893222	-	AMX84679.1	CP014342.1	Geobacillus subterraneus	Bacteria	Firmicutes	Bacilli	Bacillales	Bacillaceae	Geobacillus	type I-B CRISPR-associated endonuclease Cas1	cas1	cd09722	CAS-I-B			
2893260..2894394	+	AMX84680.1	CP014342.1	Geobacillus subterraneus	Bacteria	Firmicutes	Bacilli	Bacillales	Bacillaceae	Geobacillus	transposase						
2894524..2895034	-	AMX84681.1	CP014342.1	Geobacillus subterraneus	Bacteria	Firmicutes	Bacilli	Bacillales	Bacillaceae	Geobacillus	CRISPR-associated protein Cas4	cas4	pfam01930	CAS-I			
2895043..2897377	-	AMX84682.1	CP014342.1	Geobacillus subterraneus	Bacteria	Firmicutes	Bacilli	Bacillales	Bacillaceae	Geobacillus	CRISPR-associated protein Cas3	cas3HD,cas3	cd09641,COG1203	CAS-I			
2897378..2898122	-	AMX84683.1	CP014342.1	Geobacillus subterraneus	Bacteria	Firmicutes	Bacilli	Bacillales	Bacillaceae	Geobacillus	type I-B CRISPR-associated protein Cas5	cas5	cls001430	CAS-I-B			
2898137..2899094	-	AMX84684.1	CP014342.1	Geobacillus subterraneus	Bacteria	Firmicutes	Bacilli	Bacillales	Bacillaceae	Geobacillus	type I-B CRISPR-associated protein Cas7/Csh2	cas7	cd09690	CAS-I-B			
2899095..2900811	-	AMX84685.1	CP014342.1	Geobacillus subterraneus	Bacteria	Firmicutes	Bacilli	Bacillales	Bacillaceae	Geobacillus	type I-B CRISPR-associated protein Cas8b/Csh1	cas8b1	pfam09484	CAS-I-B			
2901224..2902109	-	AMX84686.1	CP014342.1	Geobacillus subterraneus	Bacteria	Firmicutes	Bacilli	Bacillales	Bacillaceae	Geobacillus	transposase	C3a178	cluster178				
45 CAS-I-B | RAK19108.1																	
19872..21603	+	RAK19102.1	QLMH01000007.1	Anoxybacillus vitaminiphilus	Bacteria	Firmicutes	Bacilli	Bacillales	Bacillaceae	Anoxybacillus	CRISPR-associated Csh1 family protein	cas8b1	cd09730	CAS-I-B			
21592..22555	+	RAK19103.1	QLMH01000007.1	Anoxybacillus vitaminiphilus	Bacteria	Firmicutes	Bacilli	Bacillales	Bacillaceae	Anoxybacillus	CRISPR-associated Csh2 family protein	cas7	cd09690	CAS-I-B			
22566..23286	+	RAK19104.1	QLMH01000007.1	Anoxybacillus vitaminiphilus	Bacteria	Firmicutes	Bacilli	Bacillales	Bacillaceae	Anoxybacillus	CRISPR-associated protein Cas5h	cas5	cls001430	CAS-I-B			
23340..25680	+	RAK19105.1	QLMH01000007.1	Anoxybacillus vitaminiphilus	Bacteria	Firmicutes	Bacilli	Bacillales	Bacillaceae	Anoxybacillus	CRISPR-associated Cas3 family helicase	cas3HD,cas3	cd09641,COG1203	CAS-I			
25689..26199	+	RAK19106.1	QLMH01000007.1	Anoxybacillus vitaminiphilus	Bacteria	Firmicutes	Bacilli	Bacillales	Bacillaceae	Anoxybacillus	CRISPR-associated exonuclease Cas4	cas4	pfam01930	CAS-I			
26201..27203	+	RAK19107.1	QLMH01000007.1	Anoxybacillus vitaminiphilus	Bacteria	Firmicutes	Bacilli	Bacillales	Bacillaceae	Anoxybacillus	CRISPR-associated protein Cas1	cas1	cd09722	CAS-I-B			
27212..27476	+	RAK19108.1	QLMH01000007.1	Anoxybacillus vitaminiphilus	Bacteria	Firmicutes	Bacilli	Bacillales	Bacillaceae	Anoxybacillus	CRISPR-associated protein Cas2	cas2	cd09725	CAS-I,CAS-II,CAS-III,CAS-V			
27491..28241	+	RAK19109.1	QLMH01000007.1	Anoxybacillus vitaminiphilus	Bacteria	Firmicutes	Bacilli	Bacillales	Bacillaceae	Anoxybacillus	CRISPR-associated Cas6 family protein	cas6	COG1583	CAS-I,CAS-III,CAS-IV			
28446..32754	array		QLMH01000007.1	Anoxybacillus vitaminiphilus	Bacteria	Firmicutes	Bacilli	Bacillales	Bacillaceae	Anoxybacillus		CRISPR array			65	36	30
46 partial CAS-I-B | KZM55205.1																	
44943..45945	+	KZM55204.1	LVHY01000098.1	Aeribacillus pallidus	Bacteria	Firmicutes	Bacilli	Bacillales	Bacillaceae	Aeribacillus	subtype I-B CRISPR-associated endonuclease Cas1	cas1	cd09722	CAS-I-B			
45954..46218	+	KZM55205.1	LVHY01000098.1	Aeribacillus pallidus	Bacteria	Firmicutes	Bacilli	Bacillales	Bacillaceae	Aeribacillus	CRISPR-associated endonuclease Cas2	cas2	cd09725	CAS-I,CAS-II,CAS-III,CAS-V			
46233..46983	+	KZM55206.1	LVHY01000098.1	Aeribacillus pallidus	Bacteria	Firmicutes	Bacilli	Bacillales	Bacillaceae	Aeribacillus	CRISPR-associated endoribonuclease Cas6	cas6	COG1583	CAS-I,CAS-III,CAS-IV			
47168..47598	array		LVHY01000098.1	Aeribacillus pallidus	Bacteria	Firmicutes	Bacilli	Bacillales	Bacillaceae	Aeribacillus		CRISPR array			7	36	30
47 Partial | KZN97410.1																	
69955..70250	array		LWBR01000010.1	Aeribacillus pallidus	Bacteria	Firmicutes	Bacilli	Bacillales	Bacillaceae	Aeribacillus		CRISPR array			5	35	31
70433..71183	-	KZN97409.1	LWBR01000010.1	Aeribacillus pallidus	Bacteria	Firmicutes	Bacilli	Bacillales	Bacillaceae	Aeribacillus	CRISPR-associated endoribonuclease Cas6	cas6	COG1583	CAS-I,CAS-III,CAS-IV			
71198..71462	-	KZN97410.1	LWBR01000010.1	Aeribacillus pallidus	Bacteria	Firmicutes	Bacilli	Bacillales	Bacillaceae	Aeribacillus	CRISPR-associated endonuclease Cas2	cas2	cd09725	CAS-I,CAS-II,CAS-III,CAS-V			
48 CAS-I-B | OOE05107.1																	
789487..791491	array		MQAD01000005.1	Anoxybacillus sp. 103	Bacteria	Firmicutes	Bacilli	Bacillales	Bacillaceae	Anoxybacillus		CRISPR array			30	38	30
791671..792421	-	OOE05041.1	MQAD01000005.1	Anoxybacillus sp. 103	Bacteria	Firmicutes	Bacilli	Bacillales	Bacillaceae	Anoxybacillus	CRISPR-associated endoribonuclease Cas6	cas6	COG1583	CAS-I,CAS-III,CAS-IV			
792435..792699	-	OOE05107.1	MQAD01000005.1	Anoxybacillus sp. 103	Bacteria	Firmicutes	Bacilli	Bacillales	Bacillaceae	Anoxybacillus	CRISPR-associated endonuclease Cas2	cas2	COG1343	CAS-I,CAS-II,CAS-III			
792708..793710	-	OOE05042.1	MQAD01000005.1	Anoxybacillus sp. 103	Bacteria	Firmicutes	Bacilli	Bacillales	Bacillaceae	Anoxybacillus	subtype I-B CRISPR-associated endonuclease Cas1	cas1	cd09722	CAS-I-B			
793711..794221	-	OOE05108.1	MQAD01000005.1	Anoxybacillus sp. 103	Bacteria	Firmicutes	Bacilli	Bacillales	Bacillaceae	Anoxybacillus	CRISPR-associated protein Cas4	cas4	pfam01930	CAS-I			
794229..796572	-	OOE05043.1	MQAD01000005.1	Anoxybacillus sp. 103	Bacteria	Firmicutes	Bacilli	Bacillales	Bacillaceae	Anoxybacillus	CRISPR-associated helicase/endonuclease Cas3	cas3HD,cas3	cd09641,COG1203	CAS-I			
796619..797339	-	OOE05044.1	MQAD01000005.1	Anoxybacillus sp. 103	Bacteria	Firmicutes	Bacilli	Bacillales	Bacillaceae	Anoxybacillus	type I-B CRISPR-associated protein Cas5	cas5	cls001430	CAS-I-B			
797351..798314	-	OOE05045.1	MQAD01000005.1	Anoxybacillus sp. 103	Bacteria	Firmicutes	Bacilli	Bacillales	Bacillaceae	Anoxybacillus	type I-B CRISPR-associated protein Cas7/Csh2	cas7	cd09690	CAS-I-B			
798294..800022	-	OOE05046.1	MQAD01000005.1	Anoxybacillus sp. 103	Bacteria	Firmicutes	Bacilli	Bacillales	Bacillaceae	Anoxybacillus	type I-B CRISPR-associated protein Cas8b/Csh1	cas8b1	cd09730	CAS-I-B			
800413..803088	array		MQAD01000005.1	Anoxybacillus sp. 103	Bacteria	Firmicutes	Bacilli	Bacillales	Bacillaceae	Anoxybacillus		CRISPR array			40	37	30
49 CAS-I-B | CUA79980.1																	
163725..165861	array		CYGZ01000006.1	Anoxybacillus suryakundensis	Bacteria	Firmicutes	Bacilli	Bacillales	Bacillaceae	Anoxybacillus		CRISPR array			32	37	30
166042..166792	-	CUA79979.1	CYGZ01000006.1	Anoxybacillus suryakundensis	Bacteria	Firmicutes	Bacilli	Bacillales	Bacillaceae	Anoxybacillus	CRISPR-associated endoribonuclease Cas6	cas6	COG1583	CAS-I,CAS-III,CAS-IV			
166806..167070	-	CUA79980.1	CYGZ01000006.1	Anoxybacillus suryakundensis	Bacteria	Firmicutes	Bacilli	Bacillales	Bacillaceae	Anoxybacillus	CRISPR-associated endonuclease Cas2	cas2	cd09725	CAS-I,CAS-II,CAS-III,CAS-V			
167079..168081	-	CUA79981.1	CYGZ01000006.1	Anoxybacillus suryakundensis	Bacteria	Firmicutes	Bacilli	Bacillales	Bacillaceae	Anoxybacillus	CRISPR-associated endonuclease Cas1	cas1	cd09722	CAS-I-B			
168082..168607	-	CUA79982.1	CYGZ01000006.1	Anoxybacillus suryakundensis	Bacteria	Firmicutes	Bacilli	Bacillales	Bacillaceae	Anoxybacillus	CRISPR-associated protein Cas4	cas4	pfam01930	CAS-I			
168600..170940	-	CUA79983.1	CYGZ01000006.1	Anoxybacillus suryakundensis	Bacteria	Firmicutes	Bacilli	Bacillales	Bacillaceae	Anoxybacillus	CRISPR-associated helicase, Cas3 family	cas3HD,cas3	cd09641,COG1203	CAS-I			
170990..171710	-	CUA79984.1	CYGZ01000006.1	Anoxybacillus suryakundensis	Bacteria	Firmicutes	Bacilli	Bacillales	Bacillaceae	Anoxybacillus	CRISPR-associated protein Cas5, N-terminal domain	cas5	cls001430	CAS-I-B			
171722..172685	-	CUA79985.1	CYGZ01000006.1	Anoxybacillus suryakundensis	Bacteria	Firmicutes	Bacilli	Bacillales	Bacillaceae	Anoxybacillus	CRISPR-associated protein, Csh2 family	cas7	cd09690	CAS-I-B			
172665..174393	-	CUA79986.1	CYGZ01000006.1	Anoxybacillus suryakundensis	Bacteria	Firmicutes	Bacilli	Bacillales	Bacillaceae	Anoxybacillus	CRISPR-associated protein, Csh1 family	cas8b1	cd09730	CAS-I-B			
174856..175288	+	CUA79987.1	CYGZ01000006.1	Anoxybacillus suryakundensis	Bacteria	Firmicutes	Bacilli	Bacillales	Bacillaceae	Anoxybacillus	Predicted nuclease of the RNAse H fold, HicB family						
175583..176777	array		CYGZ01000006.1	Anoxybacillus suryakundensis	Bacteria	Firmicutes	Bacilli	Bacillales	Bacillaceae	Anoxybacillus		CRISPR array			18	38	30
50 CAS-I-B | OAO81655.1																	
207..1658	array		LUCQ01000045.1	Anoxybacillus flavithermus	Bacteria	Firmicutes	Bacilli	Bacillales	Bacillaceae	Anoxybacillus		CRISPR array			22	37	30
690..804	-	OAO81653.1	LUCQ01000045.1	Anoxybacillus flavithermus	Bacteria	Firmicutes	Bacilli	Bacillales	Bacillaceae	Anoxybacillus	hypothetical protein						
1840..2590	-	OAO81654.1	LUCQ01000045.1	Anoxybacillus flavithermus	Bacteria	Firmicutes	Bacilli	Bacillales	Bacillaceae	Anoxybacillus	hypothetical protein	cas6	COG1583	CAS-I,CAS-III,CAS-IV			
2604..2874	-	OAO81655.1	LUCQ01000045.1	Anoxybacillus flavithermus	Bacteria	Firmicutes	Bacilli	Bacillales	Bacillaceae	Anoxybacillus	CRISPR-associated protein Cas2	cas2	cd09725	CAS-I,CAS-II,CAS-III,CAS-V			
2877..3879	-	OAO81656.1	LUCQ01000045.1	Anoxybacillus flavithermus	Bacteria	Firmicutes	Bacilli	Bacillales	Bacillaceae	Anoxybacillus	CRISPR-associated protein Cas1	cas1	cd09722	CAS-I-B			
3880..4393	-	OAO81657.1	LUCQ01000045.1	Anoxybacillus flavithermus	Bacteria	Firmicutes	Bacilli	Bacillales	Bacillaceae	Anoxybacillus	CRISPR-associated RecB family exonuclease Cas4a	cas4	pfam01930	CAS-I			
4408..6748	-	OAO81658.1	LUCQ01000045.1	Anoxybacillus flavithermus	Bacteria	Firmicutes	Bacilli	Bacillales	Bacillaceae	Anoxybacillus	CRISPR-associated helicase Cas3	cas3HD,cas3	cd09641,COG1203	CAS-I			
6735..7422	-	OAO81659.1	LUCQ01000045.1	Anoxybacillus flavithermus	Bacteria	Firmicutes	Bacilli	Bacillales	Bacillaceae	Anoxybacillus	CRISPR-associated protein Cas5h family	cas5	cls001430	CAS-I-B			
7435..8407	-	OAO81660.1	LUCQ01000045.1	Anoxybacillus flavithermus	Bacteria	Firmicutes	Bacilli	Bacillales	Bacillaceae	Anoxybacillus	CRISPR-associated protein	cas7b	pfam05107	CAS-I-B,CAS-I-C			
8419..10429	-	OAO81661.1	LUCQ01000045.1	Anoxybacillus flavithermus	Bacteria	Firmicutes	Bacilli	Bacillales	Bacillaceae	Anoxybacillus	hypothetical protein	cas8b1	mkCas0074	CAS-I-B			
10648..11008	array		LUCQ01000045.1	Anoxybacillus flavithermus	Bacteria	Firmicutes	Bacilli	Bacillales	Bacillaceae	Anoxybacillus		CRISPR array			6	36	30
51 CAS-I-B | ASA97889.1																	
15969..17351	array		CP021838.1	Anoxybacillus flavithermus	Bacteria	Firmicutes	Bacilli	Bacillales	Bacillaceae	Anoxybacillus		CRISPR array			21	37	30
17563..19579	+	ASA95381.1	CP021838.1	Anoxybacillus flavithermus	Bacteria	Firmicutes	Bacilli	Bacillales	Bacillaceae	Anoxybacillus	hypothetical protein	cas8b1	mkCas0074	CAS-I-B			
19597..20563	+	ASA97887.1	CP021838.1	Anoxybacillus flavithermus	Bacteria	Firmicutes	Bacilli	Bacillales	Bacillaceae	Anoxybacillus	CRISPR-associated protein	cas7b	pfam05107	CAS-I-B,CAS-I-C			
20567..21263	+	ASA95382.1	CP021838.1	Anoxybacillus flavithermus	Bacteria	Firmicutes	Bacilli	Bacillales	Bacillaceae	Anoxybacillus	CRISPR-associated protein Cas5	cas5	cls001430	CAS-I-B			
21250..23590	+	ASA95383.1	CP021838.1	Anoxybacillus flavithermus	Bacteria	Firmicutes	Bacilli	Bacillales	Bacillaceae	Anoxybacillus	CRISPR-associated helicase Cas3'	cas3HD,cas3	cd09641,COG1203	CAS-I			
23620..24118	+	ASA97888.1	CP021838.1	Anoxybacillus flavithermus	Bacteria	Firmicutes	Bacilli	Bacillales	Bacillaceae	Anoxybacillus	CRISPR-associated protein Cas4	cas4	pfam01930	CAS-I			
24119..25121	+	ASA95384.1	CP021838.1	Anoxybacillus flavithermus	Bacteria	Firmicutes	Bacilli	Bacillales	Bacillaceae	Anoxybacillus	subtype I-B CRISPR-associated endonuclease Cas1	cas1	cd09722	CAS-I-B			
25130..25394	+	ASA97889.1	CP021838.1	Anoxybacillus flavithermus	Bacteria	Firmicutes	Bacilli	Bacillales	Bacillaceae	Anoxybacillus	CRISPR-associated endonuclease Cas2	cas2	cd09725	CAS-I,CAS-II,CAS-III,CAS-V			
25408..26158	+	ASA95385.1	CP021838.1	Anoxybacillus flavithermus	Bacteria	Firmicutes	Bacilli	Bacillales	Bacillaceae	Anoxybacillus	CRISPR-associated endoribonuclease Cas6	cas6	COG1583	CAS-I,CAS-III,CAS-IV			
26341..27315	array		CP021838.1	Anoxybacillus flavithermus	Bacteria	Firmicutes	Bacilli	Bacillales	Bacillaceae	Anoxybacillus		CRISPR array			15	37	30
52 CAS-I-B | OYD06232.1																	
10299..10796	array		NOWF01000015.1	Paludifilum halophilum	Bacteria	Firmicutes	Bacilli	Bacillales	Thermoactinomycetaceae	Paludifilum		CRISPR array			8	47	21
11365..11662	array		NOWF01000015.1	Paludifilum halophilum	Bacteria	Firmicutes	Bacilli	Bacillales	Thermoactinomycetaceae	Paludifilum		CRISPR array			5	37	30
11899..12661	+	OYD06225.1	NOWF01000015.1	Paludifilum halophilum	Bacteria	Firmicutes	Bacilli	Bacillales	Thermoactinomycetaceae	Paludifilum	CRISPR-associated endoribonuclease Cas6	cas6	COG1583	CAS-I,CAS-III,CAS-IV			
12657..14424	+	OYD06226.1	NOWF01000015.1	Paludifilum halophilum	Bacteria	Firmicutes	Bacilli	Bacillales	Thermoactinomycetaceae	Paludifilum	hypothetical protein	cas8b1	cd09730	CAS-I-B			
14420..15380	+	OYD06227.1	NOWF01000015.1	Paludifilum halophilum	Bacteria	Firmicutes	Bacilli	Bacillales	Thermoactinomycetaceae	Paludifilum	type I-B CRISPR-associated protein Cas7/Csh2	cas7	cd09690	CAS-I-B			
15391..16138	+	OYD06228.1	NOWF01000015.1	Paludifilum halophilum	Bacteria	Firmicutes	Bacilli	Bacillales	Thermoactinomycetaceae	Paludifilum	type I-B CRISPR-associated protein Cas5	cas5	cls001430	CAS-I-B			
16134..18495	+	OYD06229.1	NOWF01000015.1	Paludifilum halophilum	Bacteria	Firmicutes	Bacilli	Bacillales	Thermoactinomycetaceae	Paludifilum	hypothetical protein	cas3HD,cas3	cd09641,COG1203	CAS-I			
18449..18977	+	OYD06230.1	NOWF01000015.1	Paludifilum halophilum	Bacteria	Firmicutes	Bacilli	Bacillales	Thermoactinomycetaceae	Paludifilum	CRISPR-associated protein Cas4	cas4	pfam01930	CAS-I			
18991..19984	+	OYD06231.1	NOWF01000015.1	Paludifilum halophilum	Bacteria	Firmicutes	Bacilli	Bacillales	Thermoactinomycetaceae	Paludifilum	subtype I-B CRISPR-associated endonuclease Cas1	cas1	cd09722	CAS-I-B			
19996..20260	+	OYD06232.1	NOWF01000015.1	Paludifilum halophilum	Bacteria	Firmicutes	Bacilli	Bacillales	Thermoactinomycetaceae	Paludifilum	CRISPR-associated endonuclease Cas2	cas2	cd09725	CAS-I,CAS-II,CAS-III,CAS-V			
21017..21868	array		NOWF01000015.1	Paludifilum halophilum	Bacteria	Firmicutes	Bacilli	Bacillales	Thermoactinomycetaceae	Paludifilum		CRISPR array			13	38	30

22182..22954	array		NOWF01000015.1	Paludifilum halophilum	Bacteria	Firmicutes	Bacilli	Bacillales	Thermoactinomycetaceae	Paludifilum		CRISPR array			12	37	30
23266..23691	array		NOWF01000015.1	Paludifilum halophilum	Bacteria	Firmicutes	Bacilli	Bacillales	Thermoactinomycetaceae	Paludifilum		CRISPR array			7	43	24
23996..24498	array		NOWF01000015.1	Paludifilum halophilum	Bacteria	Firmicutes	Bacilli	Bacillales	Thermoactinomycetaceae	Paludifilum		CRISPR array			8	37	30
24817..25311	array		NOWF01000015.1	Paludifilum halophilum	Bacteria	Firmicutes	Bacilli	Bacillales	Thermoactinomycetaceae	Paludifilum		CRISPR array			8	36	30
53 CAS-I-B | AQS56075.1																	
2025465..2026506	-	AQS56069.1	CP019699.1	Novibacillus thermophilus	Bacteria	Firmicutes	Bacilli	Bacillales	Thermoactinomycetaceae	Novibacillus	luciferase	C3a156	cluster156				
2026822..2028995	array		CP019699.1	Novibacillus thermophilus	Bacteria	Firmicutes	Bacilli	Bacillales	Thermoactinomycetaceae	Novibacillus		CRISPR array			33	37	30
2029210..2029984	+	AQS56070.1	CP019699.1	Novibacillus thermophilus	Bacteria	Firmicutes	Bacilli	Bacillales	Thermoactinomycetaceae	Novibacillus	CRISPR-associated endoribonuclease Cas6	cas6	COG1583	CAS-I,CAS-III,CAS-IV			
2029973..2032073	+	AQS56071.1	CP019699.1	Novibacillus thermophilus	Bacteria	Firmicutes	Bacilli	Bacillales	Thermoactinomycetaceae	Novibacillus	hypothetical protein	cas8b1	mkCas0074	CAS-I-B			
2032104..2033055	+	AQS57493.1	CP019699.1	Novibacillus thermophilus	Bacteria	Firmicutes	Bacilli	Bacillales	Thermoactinomycetaceae	Novibacillus	CRISPR-associated protein Csh2	cas7b	pfam05107	CAS-I-B,CAS-I-C			
2033079..2033784	+	AQS57494.1	CP019699.1	Novibacillus thermophilus	Bacteria	Firmicutes	Bacilli	Bacillales	Thermoactinomycetaceae	Novibacillus	CRISPR-associated protein Cas5	cas5	cd09692	CAS-I-B			
2033777..2036198	+	AQS56072.1	CP019699.1	Novibacillus thermophilus	Bacteria	Firmicutes	Bacilli	Bacillales	Thermoactinomycetaceae	Novibacillus	hypothetical protein	cas3HD,cas3	cd09641,COG1203	CAS-I			
2036238..2036742	+	AQS56073.1	CP019699.1	Novibacillus thermophilus	Bacteria	Firmicutes	Bacilli	Bacillales	Thermoactinomycetaceae	Novibacillus	CRISPR-associated protein Cas4	cas4	pfam01930	CAS-I			
2036773..2037766	+	AQS56074.1	CP019699.1	Novibacillus thermophilus	Bacteria	Firmicutes	Bacilli	Bacillales	Thermoactinomycetaceae	Novibacillus	subtype I-B CRISPR-associated endonuclease Cas1	cas1	cd09722	CAS-I-B			
2037821..2038085	+	AQS56075.1	CP019699.1	Novibacillus thermophilus	Bacteria	Firmicutes	Bacilli	Bacillales	Thermoactinomycetaceae	Novibacillus	CRISPR-associated endonuclease Cas2	**cas2**	cd09725	CAS-I,CAS-II,CAS-III,CAS-V			
54 partial CAS-I-B | OUM88221.1																	
16716..17478	+	OUM88225.1	LZRT01000064.1	Bacillus thermozeamaize	Bacteria	Firmicutes	Bacilli	Bacillales	Bacillaceae	Bacillus	CRISPR-associated endoribonuclease Cas6	cas6	COG1583	CAS-I,CAS-III,CAS-IV			
17516..18023	+	OUM88219.1	LZRT01000064.1	Bacillus thermozeamaize	Bacteria	Firmicutes	Bacilli	Bacillales	Bacillaceae	Bacillus	CRISPR-associated protein Cas4	cas4	pfam01930	CAS-I			
18039..19032	+	OUM88220.1	LZRT01000064.1	Bacillus thermozeamaize	Bacteria	Firmicutes	Bacilli	Bacillales	Bacillaceae	Bacillus	subtype I-B CRISPR-associated endonuclease Cas1	cas1	cd09722	CAS-I-B			
19120..19384	+	OUM88221.1	LZRT01000064.1	Bacillus thermozeamaize	Bacteria	Firmicutes	Bacilli	Bacillales	Bacillaceae	Bacillus	CRISPR-associated endonuclease Cas2	**cas2**	cd09725	CAS-I,CAS-II,CAS-III,CAS-V			
19634..21198	array		LZRT01000064.1	Bacillus thermozeamaize	Bacteria	Firmicutes	Bacilli	Bacillales	Bacillaceae	Bacillus		CRISPR array			24	36	30
55 CAS-I-B | SFU92278.1																	
93187..97986	array		FPBV01000013.1	Alicyclobacillus macrosporangiidus	Bacteria	Firmicutes	Bacilli	Bacillales	Alicyclobacillaceae	Alicyclobacillus		CRISPR array			72	37	30
98272..98491	+	SFU92147.1	FPBV01000013.1	Alicyclobacillus macrosporangiidus	Bacteria	Firmicutes	Bacilli	Bacillales	Alicyclobacillaceae	Alicyclobacillus	hypothetical protein						
98606..99431	+	SFU92167.1	FPBV01000013.1	Alicyclobacillus macrosporangiidus	Bacteria	Firmicutes	Bacilli	Bacillales	Alicyclobacillaceae	Alicyclobacillus	CRISPR-associated endoribonuclease Cas6	cas6	COG1583	CAS-I,CAS-III,CAS-IV			
99399..101520	+	SFU92179.1	FPBV01000013.1	Alicyclobacillus macrosporangiidus	Bacteria	Firmicutes	Bacilli	Bacillales	Alicyclobacillaceae	Alicyclobacillus	CRISPR-associated protein TM1802 (cas_TM1802)	cas8b1	mkCas0074	CAS-I-B			
101626..102586	+	SFU92196.1	FPBV01000013.1	Alicyclobacillus macrosporangiidus	Bacteria	Firmicutes	Bacilli	Bacillales	Alicyclobacillaceae	Alicyclobacillus	CRISPR-associated protein Csh2	cas7b	pfam05107	CAS-I-B,CAS-I-C			
102597..103326	+	SFU92216.1	FPBV01000013.1	Alicyclobacillus macrosporangiidus	Bacteria	Firmicutes	Bacilli	Bacillales	Alicyclobacillaceae	Alicyclobacillus	CRISPR-associated protein Cas5h	cas5	cls001430	CAS-I-B			
103319..105836	+	SFU92227.1	FPBV01000013.1	Alicyclobacillus macrosporangiidus	Bacteria	Firmicutes	Bacilli	Bacillales	Alicyclobacillaceae	Alicyclobacillus	CRISPR-associated helicase Cas3/CRISPR-associated endonuclease Cas3-HD	cas3HD,cas3	cd09641,COG1203	CAS-I			
105889..106393	+	SFU92245.1	FPBV01000013.1	Alicyclobacillus macrosporangiidus	Bacteria	Firmicutes	Bacilli	Bacillales	Alicyclobacillaceae	Alicyclobacillus	CRISPR-associated exonuclease Cas4	cas4	pfam01930	CAS-I			
106450..107446	+	SFU92263.1	FPBV01000013.1	Alicyclobacillus macrosporangiidus	Bacteria	Firmicutes	Bacilli	Bacillales	Alicyclobacillaceae	Alicyclobacillus	CRISP-associated protein Cas1	cas1	cd09722	CAS-I-B			
107514..107778	+	SFU92278.1	FPBV01000013.1	Alicyclobacillus macrosporangiidus	Bacteria	Firmicutes	Bacilli	Bacillales	Alicyclobacillaceae	Alicyclobacillus	CRISPR-associated protein Cas2	**cas2**	cd09725	CAS-I,CAS-II,CAS-III,CAS-V			
108036..110153	array		FPBV01000013.1	Alicyclobacillus macrosporangiidus	Bacteria	Firmicutes	Bacilli	Bacillales	Alicyclobacillaceae	Alicyclobacillus		CRISPR array			32	37	30
56 CAS-I-B | ADG07371.1																	
2748616..2749912	+	ADG07369.1	CP002017.1	Kyrpidia tusciae DSM 2912	Bacteria	Firmicutes	Bacilli	Bacillales	Alicyclobacillaceae	Kyrpidia	CRISPR-associated protein, TM1812 family	csx1	cd09732	CAS-III			
2750070..2768077	array		CP002017.1	Kyrpidia tusciae DSM 2912	Bacteria	Firmicutes	Bacilli	Bacillales	Alicyclobacillaceae	Kyrpidia		CRISPR array			266	37	30
2768764..2770000	+	ADG07370.1	CP002017.1	Kyrpidia tusciae DSM 2912	Bacteria	Firmicutes	Bacilli	Bacillales	Alicyclobacillaceae	Kyrpidia	transposase IS116/IS110/IS902 family protein						
2770123..2778650	array		CP002017.1	Kyrpidia tusciae DSM 2912	Bacteria	Firmicutes	Bacilli	Bacillales	Alicyclobacillaceae	Kyrpidia		CRISPR array			127	37	30
2779064..2779328	-	ADG07371.1	CP002017.1	Kyrpidia tusciae DSM 2912	Bacteria	Firmicutes	Bacilli	Bacillales	Alicyclobacillaceae	Kyrpidia	CRISPR-associated protein Cas2	**cas2**	cd09725	CAS-I,CAS-II,CAS-III,CAS-V			
2779520..2780516	-	ADG07372.1	CP002017.1	Kyrpidia tusciae DSM 2912	Bacteria	Firmicutes	Bacilli	Bacillales	Alicyclobacillaceae	Kyrpidia	CRISPR-associated protein Cas1	cas1	cd09722	CAS-I-B			
2780533..2781037	-	ADG07373.1	CP002017.1	Kyrpidia tusciae DSM 2912	Bacteria	Firmicutes	Bacilli	Bacillales	Alicyclobacillaceae	Kyrpidia	CRISPR-associated protein Cas4	cas4	pfam01930	CAS-I			
2781076..2783497	-	ADG07374.1	CP002017.1	Kyrpidia tusciae DSM 2912	Bacteria	Firmicutes	Bacilli	Bacillales	Alicyclobacillaceae	Kyrpidia	CRISPR-associated helicase Cas3	cas3HD,cas3	cd09641,COG1203	CAS-I			
2783490..2784195	-	ADG07375.1	CP002017.1	Kyrpidia tusciae DSM 2912	Bacteria	Firmicutes	Bacilli	Bacillales	Alicyclobacillaceae	Kyrpidia	CRISPR-associated protein Cas5	cas5	cd09692	CAS-I-B			
2784210..2785188	-	ADG07376.1	CP002017.1	Kyrpidia tusciae DSM 2912	Bacteria	Firmicutes	Bacilli	Bacillales	Alicyclobacillaceae	Kyrpidia	CRISPR-associated protein, CT1132 family	cas7b	pfam05107	CAS-I-B,CAS-I-C			
2785266..2787369	-	ADG07377.1	CP002017.1	Kyrpidia tusciae DSM 2912	Bacteria	Firmicutes	Bacilli	Bacillales	Alicyclobacillaceae	Kyrpidia	conserved hypothetical protein	cas8b1	mkCas0074	CAS-I-B			
2787337..2788159	-	ADG07378.1	CP002017.1	Kyrpidia tusciae DSM 2912	Bacteria	Firmicutes	Bacilli	Bacillales	Alicyclobacillaceae	Kyrpidia	CRISPR-associated protein Cas6	cas6	COG1583	CAS-I,CAS-III,CAS-IV			
2788541..2789315	-	ADG07379.1	CP002017.1	Kyrpidia tusciae DSM 2912	Bacteria	Firmicutes	Bacilli	Bacillales	Alicyclobacillaceae	Kyrpidia	Silent information regulator protein Sir2						
2789599..2790064	+	ADG07380.1	CP002017.1	Kyrpidia tusciae DSM 2912	Bacteria	Firmicutes	Bacilli	Bacillales	Alicyclobacillaceae	Kyrpidia	protein of unknown function DUF16	CorA	icity0114				
57 CAS-I-B | ATY85843.1																	
2729499..2739035	array		CP024955.1	Kyrpidia spormannii	Bacteria	Firmicutes	Bacilli	Bacillales	Alicyclobacillaceae	Kyrpidia		CRISPR array			141	37	30
2739113..2740589	+	ATY85837.1	CP024955.1	Kyrpidia spormannii	Bacteria	Firmicutes	Bacilli	Bacillales	Alicyclobacillaceae	Kyrpidia	hypothetical protein						
2740604..2741363	+	ATY85838.1	CP024955.1	Kyrpidia spormannii	Bacteria	Firmicutes	Bacilli	Bacillales	Alicyclobacillaceae	Kyrpidia	AAA family ATPase						
2741642..2746838	array		CP024955.1	Kyrpidia spormannii	Bacteria	Firmicutes	Bacilli	Bacillales	Alicyclobacillaceae	Kyrpidia		CRISPR array			77	37	30
2747111..2748287	+	ATY86612.1	CP024955.1	Kyrpidia spormannii	Bacteria	Firmicutes	Bacilli	Bacillales	Alicyclobacillaceae	Kyrpidia	IS256 family transposase						
2748332..2752961	array		CP024955.1	Kyrpidia spormannii	Bacteria	Firmicutes	Bacilli	Bacillales	Alicyclobacillaceae	Kyrpidia		CRISPR array			69	37	30
2753074..2754550	+	ATY85839.1	CP024955.1	Kyrpidia spormannii	Bacteria	Firmicutes	Bacilli	Bacillales	Alicyclobacillaceae	Kyrpidia	hypothetical protein						
2754565..2755324	+	ATY85840.1	CP024955.1	Kyrpidia spormannii	Bacteria	Firmicutes	Bacilli	Bacillales	Alicyclobacillaceae	Kyrpidia	AAA family ATPase						
2755635..2763225	array		CP024955.1	Kyrpidia spormannii	Bacteria	Firmicutes	Bacilli	Bacillales	Alicyclobacillaceae	Kyrpidia		CRISPR array			112	38	30
2763767..2764031	-	ATY85841.1	CP024955.1	Kyrpidia spormannii	Bacteria	Firmicutes	Bacilli	Bacillales	Alicyclobacillaceae	Kyrpidia	hypothetical protein						
2764123..2764399	-	ATY85842.1	CP024955.1	Kyrpidia spormannii	Bacteria	Firmicutes	Bacilli	Bacillales	Alicyclobacillaceae	Kyrpidia	hypothetical protein						
2764771..2765035	-	ATY85843.1	CP024955.1	Kyrpidia spormannii	Bacteria	Firmicutes	Bacilli	Bacillales	Alicyclobacillaceae	Kyrpidia	CRISPR-associated endonuclease Cas2	**cas2**	cd09725	CAS-I,CAS-II,CAS-III,CAS-V			
2765095..2766091	-	ATY85844.1	CP024955.1	Kyrpidia spormannii	Bacteria	Firmicutes	Bacilli	Bacillales	Alicyclobacillaceae	Kyrpidia	subtype I-B CRISPR-associated endonuclease Cas1	cas1	cd09722	CAS-I-B			
2766108..2766612	-	ATY85845.1	CP024955.1	Kyrpidia spormannii	Bacteria	Firmicutes	Bacilli	Bacillales	Alicyclobacillaceae	Kyrpidia	CRISPR-associated protein Cas4	cas4	pfam01930	CAS-I			
2766651..2769078	-	ATY85846.1	CP024955.1	Kyrpidia spormannii	Bacteria	Firmicutes	Bacilli	Bacillales	Alicyclobacillaceae	Kyrpidia	hypothetical protein	cas3HD,cas3	cd09641,COG1203	CAS-I			
2769065..2769770	-	ATY85847.1	CP024955.1	Kyrpidia spormannii	Bacteria	Firmicutes	Bacilli	Bacillales	Alicyclobacillaceae	Kyrpidia	CRISPR-associated protein Cas5	cas5	cd09692	CAS-I-B			
2769785..2770763	-	ATY85848.1	CP024955.1	Kyrpidia spormannii	Bacteria	Firmicutes	Bacilli	Bacillales	Alicyclobacillaceae	Kyrpidia	CRISPR-associated protein	cas7b	pfam05107	CAS-I-B,CAS-I-C			
2770843..2772958	-	ATY85849.1	CP024955.1	Kyrpidia spormannii	Bacteria	Firmicutes	Bacilli	Bacillales	Alicyclobacillaceae	Kyrpidia	hypothetical protein	cas8b1	mkCas0074	CAS-I-B			
2772920..2773742	-	ATY85850.1	CP024955.1	Kyrpidia spormannii	Bacteria	Firmicutes	Bacilli	Bacillales	Alicyclobacillaceae	Kyrpidia	CRISPR-associated endoribonuclease Cas6	cas6	COG1583	CAS-I,CAS-III,CAS-IV			
58 CAS-I-B | ABO49086.1																	
579303..580062	+	ABO49079.1	CP000612.1	Desulfotomaculum reducens MI-1	Bacteria	Firmicutes	Clostridia	Clostridiales	Peptococcaceae	Desulfotomaculum	CRISPR-associated protein, Cas6 family	cas6	COG1583	CAS-I,CAS-III,CAS-IV			
580077..582114	+	ABO49080.1	CP000612.1	Desulfotomaculum reducens MI-1	Bacteria	Firmicutes	Clostridia	Clostridiales	Peptococcaceae	Desulfotomaculum	hypothetical protein	cas8b1	pfam09484	CAS-I-B			
582113..583103	+	ABO49081.1	CP000612.1	Desulfotomaculum reducens MI-1	Bacteria	Firmicutes	Clostridia	Clostridiales	Peptococcaceae	Desulfotomaculum	CRISPR-associated protein, Csh2 family	cas7b	pfam05107	CAS-I-B,CAS-I-C			
583104..583833	+	ABO49082.1	CP000612.1	Desulfotomaculum reducens MI-1	Bacteria	Firmicutes	Clostridia	Clostridiales	Peptococcaceae	Desulfotomaculum	CRISPR-associated protein, Cas5h family	cas5	cd09692	CAS-I-B			
583829..586181	+	ABO49083.1	CP000612.1	Desulfotomaculum reducens MI-1	Bacteria	Firmicutes	Clostridia	Clostridiales	Peptococcaceae	Desulfotomaculum	CRISPR-associated helicase, Cas3 family	cas3HD,cas3	cd09641,COG1203	CAS-I			
586379..587743	array		CP000612.1	Desulfotomaculum reducens MI-1	Bacteria	Firmicutes	Clostridia	Clostridiales	Peptococcaceae	Desulfotomaculum		CRISPR array			21	36	30
587824..588331	+	ABO49084.1	CP000612.1	Desulfotomaculum reducens MI-1	Bacteria	Firmicutes	Clostridia	Clostridiales	Peptococcaceae	Desulfotomaculum	CRISPR-associated exonuclease, Cas4 family	cas4	pfam01930	CAS-I			
588349..589342	+	ABO49085.1	CP000612.1	Desulfotomaculum reducens MI-1	Bacteria	Firmicutes	Clostridia	Clostridiales	Peptococcaceae	Desulfotomaculum	CRISPR-associated protein, Cas1 family	cas1	cd09722	CAS-I-B			
589366..589630	+	ABO49086.1	CP000612.1	Desulfotomaculum reducens MI-1	Bacteria	Firmicutes	Clostridia	Clostridiales	Peptococcaceae	Desulfotomaculum	CRISPR-associated protein, Cas2 family	**cas2**	cd09725	CAS-I,CAS-II,CAS-III,CAS-V			
590920..591463	+	ABO49087.1	CP000612.1	Desulfotomaculum reducens MI-1	Bacteria	Firmicutes	Clostridia	Clostridiales	Peptococcaceae	Desulfotomaculum	protein of unknown function DUF88	NYN	icity0084				
59 CAS-I-B | AEG61207.1																	
3057773..3061057	array		CP002780.1	Desulfotomaculum ruminis DSM 2154	Bacteria	Firmicutes	Clostridia	Clostridiales	Peptococcaceae	Desulfotomaculum		CRISPR array			50	36	30
3061240..3061504	-	AEG61207.1	CP002780.1	Desulfotomaculum ruminis DSM 2154	Bacteria	Firmicutes	Clostridia	Clostridiales	Peptococcaceae	Desulfotomaculum	CRISPR-associated protein Cas2	**cas2**	cd09725	CAS-I,CAS-II,CAS-III,CAS-V			
3061546..3062539	-	AEG61208.1	CP002780.1	Desulfotomaculum ruminis DSM 2154	Bacteria	Firmicutes	Clostridia	Clostridiales	Peptococcaceae	Desulfotomaculum	CRISPR-associated protein Cas1	cas1	cd09722	CAS-I-B			
3062559..3063066	-	AEG61209.1	CP002780.1	Desulfotomaculum ruminis DSM 2154	Bacteria	Firmicutes	Clostridia	Clostridiales	Peptococcaceae	Desulfotomaculum	CRISPR-associated protein Cas4	cas4	pfam01930	CAS-I			
3063198..3065574	-	AEG61210.1	CP002780.1	Desulfotomaculum ruminis DSM 2154	Bacteria	Firmicutes	Clostridia	Clostridiales	Peptococcaceae	Desulfotomaculum	CRISPR-associated HD domain protein	cas3HD,cas3	cd09641,COG1203	CAS-I			
3065570..3066299	-	AEG61211.1	CP002780.1	Desulfotomaculum ruminis DSM 2154	Bacteria	Firmicutes	Clostridia	Clostridiales	Peptococcaceae	Desulfotomaculum	CRISPR-associated protein Cas5	cas5	cd09692	CAS-I-B			
3066295..3067273	-	AEG61212.1	CP002780.1	Desulfotomaculum ruminis DSM 2154	Bacteria	Firmicutes	Clostridia	Clostridiales	Peptococcaceae	Desulfotomaculum	CRISPR-associated protein, Csh2 family	cas7b	pfam05107	CAS-I-B,CAS-I-C			
3067287..3069309	-	AEG61213.1	CP002780.1	Desulfotomaculum ruminis DSM 2154	Bacteria	Firmicutes	Clostridia	Clostridiales	Peptococcaceae	Desulfotomaculum	CRISPR-associated protein TM1802	cas8b1	pfam09484	CAS-I-B			
3069324..3070077	-	AEG61214.1	CP002780.1	Desulfotomaculum ruminis DSM 2154	Bacteria	Firmicutes	Clostridia	Clostridiales	Peptococcaceae	Desulfotomaculum	CRISPR-associated protein Cas6	cas6	COG1583	CAS-I,CAS-III,CAS-IV			
60 CAS-I-B | CUH97861.1																	
50..684	array		CYSP01000033.1	Propionispora sp. 2/2-37	Bacteria	Firmicutes	Negativicutes	Selenomonadales	Sporomusaceae	Propionispora		CRISPR array			10	37	30
881..1145	-	CUH97861.1	CYSP01000033.1	Propionispora sp. 2/2-37	Bacteria	Firmicutes	Negativicutes	Selenomonadales	Sporomusaceae	Propionispora	hypothetical protein	**cas2**	cd09725	CAS-I,CAS-II,CAS-III,CAS-V			
1192..2185	-	CUH97862.1	CYSP01000033.1	Propionispora sp. 2/2-37	Bacteria	Firmicutes	Negativicutes	Selenomonadales	Sporomusaceae	Propionispora	CRISPR-associated endonuclease Cas1 2	cas1	cd09722	CAS-I-B			
2213..2714	-	CUH97863.1	CYSP01000033.1	Propionispora sp. 2/2-37	Bacteria	Firmicutes	Negativicutes	Selenomonadales	Sporomusaceae	Propionispora	hypothetical protein	cas4	pfam01930	CAS-I			
2715..3144	-	CUH97864.1	CYSP01000033.1	Propionispora sp. 2/2-37	Bacteria	Firmicutes	Negativicutes	Selenomonadales	Sporomusaceae	Propionispora	hypothetical protein						
3147..3573	-	CUH97865.1	CYSP01000033.1	Propionispora sp. 2/2-37	Bacteria	Firmicutes	Negativicutes	Selenomonadales	Sporomusaceae	Propionispora	hypothetical protein						
3635..5975	-	CUH97866.1	CYSP01000033.1	Propionispora sp. 2/2-37	Bacteria	Firmicutes	Negativicutes	Selenomonadales	Sporomusaceae	Propionispora	hypothetical protein	cas3HD,cas3	cd09641,COG1203	CAS-I			
6001..6727	-	CUH97867.1	CYSP01000033.1	Propionispora sp. 2/2-37	Bacteria	Firmicutes	Negativicutes	Selenomonadales	Sporomusaceae	Propionispora	hypothetical protein	cas5	cd09692	CAS-I-B			
6740..7631	-	CUH97868.1	CYSP01000033.1	Propionispora sp. 2/2-37	Bacteria	Firmicutes	Negativicutes	Selenomonadales	Sporomusaceae	Propionispora	hypothetical protein	cas7	cd09690	CAS-I-B			
7593..9573	-	CUH97869.1	CYSP01000033.1	Propionispora sp. 2/2-37	Bacteria	Firmicutes	Negativicutes	Selenomonadales	Sporomusaceae	Propionispora	hypothetical protein	cas8b1	pfam09484	CAS-I-B			
61 CAS-I-B | BBB93527.1																	
4751728..4754560	array		AP018449.1	Methylomusa anaerophila	Bacteria	Firmicutes	Negativicutes	Selenomonadales	Sporomusaceae	Methylomusa		CRISPR array			43	36	30
4754782..4755094	-	BBB93527.1	AP018449.1	Methylomusa anaerophila	Bacteria	Firmicutes	Negativicutes	Selenomonadales	Sporomusaceae	Methylomusa	CRISPR-associated endoribonuclease Cas2	**cas2**	cd09725	CAS-I,CAS-II,CAS-III,CAS-V			
4755090..4756083	-	BBB93528.1	AP018449.1	Methylomusa anaerophila	Bacteria	Firmicutes	Negativicutes	Selenomonadales	Sporomusaceae	Methylomusa	CRISPR-associated endonuclease Cas1	cas1	cd09722	CAS-I-B			
4756110..4756611	-	BBB93529.1	AP018449.1	Methylomusa anaerophila	Bacteria	Firmicutes	Negativicutes	Selenomonadales	Sporomusaceae	Methylomusa	hypothetical protein	cas4	pfam01930	CAS-I			
4756612..4757044	-	BBB93530.1	AP018449.1	Methylomusa anaerophila	Bacteria	Firmicutes	Negativicutes	Selenomonadales	Sporomusaceae	Methylomusa	hypothetical protein						
4757033..4757462	-	BBB93531.1	AP018449.1	Methylomusa anaerophila	Bacteria	Firmicutes	Negativicutes	Selenomonadales	Sporomusaceae	Methylomusa	nucleotidyltransferase domain protein						
4757577..4758869	array		AP018449.1	Methylomusa anaerophila	Bacteria	Firmicutes	Negativicutes	Selenomonadales	Sporomusaceae	Methylomusa		CRISPR array			20	36	30
4759354..4761622	-	BBB93532.1	AP018449.1	Methylomusa anaerophila	Bacteria	Firmicutes	Negativicutes	Selenomonadales	Sporomusaceae	Methylomusa	CRISPR-associated endonuclease/helicase Cas3	cas3HD,cas3	cd09641,cd09639	CAS-I			
4761572..4762289	-	BBB93533.1	AP018449.1	Methylomusa anaerophila	Bacteria	Firmicutes	Negativicutes	Selenomonadales	Sporomusaceae	Methylomusa	CRISPR-associated protein	cas5	cls001430	CAS-I-B			
4762294..4763203	-	BBB93534.1	AP018449.1	Methylomusa anaerophila	Bacteria	Firmicutes	Negativicutes	Selenomonadales	Sporomusaceae	Methylomusa	hypothetical protein	cas7	cd09690	CAS-I-B			
4763237..4763654	-	BBB93535.1	AP018449.1	Methylomusa anaerophila	Bacteria	Firmicutes	Negativicutes	Selenomonadales	Sporomusaceae	Methylomusa	hypothetical protein	cas8b1	mkCas0119	CAS-I-B			
4763650..4765177	-	BBB93536.1	AP018449.1	Methylomusa anaerophila	Bacteria	Firmicutes	Negativicutes	Selenomonadales	Sporomusaceae	Methylomusa	CRISPR-associated protein	cas8b1	cd09730	CAS-I-B			
4765190..4765925	-	BBB93537.1	AP018449.1	Methylomusa anaerophila	Bacteria	Firmicutes	Negativicutes	Selenomonadales	Sporomusaceae	Methylomusa	CRISPR associated protein Cas6	cas6	COG1583	CAS-I,CAS-III,CAS-IV			
62 partial CAS-I-B | KFD40924.1																	
1162..1669	+	KFD40922.1	JJNX02000076.1	Peptococcaceae bacterium SCADC1_2_3	Bacteria	Firmicutes	Clostridia	Clostridiales	Peptococcaceae		CRISPR-associated protein Cas4	cas4	pfam01930	CAS-I			
1760..2753	+	KFD40923.1	JJNX02000076.1	Peptococcaceae bacterium SCADC1_2_3	Bacteria	Firmicutes	Clostridia	Clostridiales	Peptococcaceae		CRISPR-associated protein Cas1	cas1	cd09722	CAS-I-B			
2832..3096	+	KFD40924.1	JJNX02000076.1	Peptococcaceae bacterium SCADC1_2_3	Bacteria	Firmicutes	Clostridia	Clostridiales	Peptococcaceae		CRISPR-associated protein Cas2	cas2	cd09725	CAS-I,CAS-II,CAS-III,CAS-V			
3311..10181	array		JJNX02000076.1	Peptococcaceae bacterium SCADC1_2_3	Bacteria	Firmicutes	Clostridia	Clostridiales	Peptococcaceae			CRISPR array			104	36	30
64 CAS-I-B | OPX90215.1																	
56967..64944	array		MVQL01000133.1	Pelotomaculum sp. PtaB.Bin104	Bacteria	Firmicutes	Clostridia	Clostridiales	Peptococcaceae	Pelotomaculum		CRISPR array			120	36	30
65120..65384	-	OPX90215.1	MVQL01000133.1	Pelotomaculum sp. PtaB.Bin104	Bacteria	Firmicutes	Clostridia	Clostridiales	Peptococcaceae	Pelotomaculum	CRISPR-associated endoribonuclease Cas2	cas2	cd09725	CAS-I,CAS-II,CAS-III,CAS-V			
65409..66402	-	OPX90216.1	MVQL01000133.1	Pelotomaculum sp. PtaB.Bin104	Bacteria	Firmicutes	Clostridia	Clostridiales	Peptococcaceae	Pelotomaculum	CRISPR-associated endonuclease Cas1	cas1	cd09722	CAS-I-B			
66477..66993	-	OPX90217.1	MVQL01000133.1	Pelotomaculum sp. PtaB.Bin104	Bacteria	Firmicutes	Clostridia	Clostridiales	Peptococcaceae	Pelotomaculum	hypothetical protein	cas4	pfam01930	CAS-I			
67217..69569	-	OPX90218.1	MVQL01000133.1	Pelotomaculum sp. PtaB.Bin104	Bacteria	Firmicutes	Clostridia	Clostridiales	Peptococcaceae	Pelotomaculum	helicase Cas3	cas3HD,cas3	cd09641,COG1203	CAS-I			
69565..70294	-	OPX90219.1	MVQL01000133.1	Pelotomaculum sp. PtaB.Bin104	Bacteria	Firmicutes	Clostridia	Clostridiales	Peptococcaceae	Pelotomaculum	CRISPR-associated protein (Cas_Cas5)	cas5	cd09692	CAS-I-B			
70303..71284	-	OPX90220.1	MVQL01000133.1	Pelotomaculum sp. PtaB.Bin104	Bacteria	Firmicutes	Clostridia	Clostridiales	Peptococcaceae	Pelotomaculum	hypothetical protein	cas7b	pfam05107	CAS-I-B,CAS-I-C			
71300..73340	-	OPX90221.1	MVQL01000133.1	Pelotomaculum sp. PtaB.Bin104	Bacteria	Firmicutes	Clostridia	Clostridiales	Peptococcaceae	Pelotomaculum	CRISPR-associated protein (cas_TM1802)	cas8b1	pfam09484	CAS-I-B			
73445..74183	-	OPX90222.1	MVQL01000133.1	Pelotomaculum sp. PtaB.Bin104	Bacteria	Firmicutes	Clostridia	Clostridiales	Peptococcaceae	Pelotomaculum	CRISPR associated protein Cas6	cas6	COG1583	CAS-I,CAS-III,CAS-IV			
66 CAS-I-B | BAF60094.1																	
2024396..2026557	array		AP009389.1	Pelotomaculum thermopropionicum SI	Bacteria	Firmicutes	Clostridia	Clostridiales	Peptococcaceae	Pelotomaculum		CRISPR array			33	36	30
2026732..2027056	-	BAF60094.1	AP009389.1	Pelotomaculum thermopropionicum SI	Bacteria	Firmicutes	Clostridia	Clostridiales	Peptococcaceae	Pelotomaculum	Uncharacterized protein	**cas2**	cd09725	CAS-I,CAS-II,CAS-III,CAS-V			
2027073..2028108	-	BAF60095.1	AP009389.1	Pelotomaculum thermopropionicum SI	Bacteria	Firmicutes	Clostridia	Clostridiales	Peptococcaceae	Pelotomaculum	Uncharacterized protein	cas1	cd09722	CAS-I-B			
2028095..2028599	-	BAF60096.1	AP009389.1	Pelotomaculum thermopropionicum SI	Bacteria	Firmicutes	Clostridia	Clostridiales	Peptococcaceae	Pelotomaculum	RecB family exonuclease	cas4	pfam01930	CAS-I			
2028822..2031240	-	BAF60097.1	AP009389.1	Pelotomaculum thermopropionicum SI	Bacteria	Firmicutes	Clostridia	Clostridiales	Peptococcaceae	Pelotomaculum	predicted helicases	cas3HD,cas3	cd09641,COG1203	CAS-I			
2031227..2031956	-	BAF60098.1	AP009389.1	Pelotomaculum thermopropionicum SI	Bacteria	Firmicutes	Clostridia	Clostridiales	Peptococcaceae	Pelotomaculum	hypothetical protein	cas5	cd09692	CAS-I-B			
2031986..2032955	-	BAF60099.1	AP009389.1	Pelotomaculum thermopropionicum SI	Bacteria	Firmicutes	Clostridia	Clostridiales	Peptococcaceae	Pelotomaculum	Uncharacterized protein	cas7b	pfam05107	CAS-I-B,CAS-I-C			
2033065..2035144	-	BAF60100.1	AP009389.1	Pelotomaculum thermopropionicum SI	Bacteria	Firmicutes	Clostridia	Clostridiales	Peptococcaceae	Pelotomaculum	hypothetical protein	cas8b1	mkCas0074	CAS-I-B			
2035596..2036496	+	BAF60101.1	AP009389.1	Pelotomaculum thermopropionicum SI	Bacteria	Firmicutes	Clostridia	Clostridiales	Peptococcaceae	Pelotomaculum	uncharacterized conserved protein	C3a174	cluster174				

2036497..2037253	-	BAF60102.1	AP009389.1	Pelotomaculum thermopropionicum SI	Bacteria	Firmicutes	Clostridia	Clostridiales	Peptococcaceae	Pelotomaculum	Uncharacterized protein	cas6	COG1583	CAS-I,CAS-III,CAS-IV			
67 CAS-I-B | AGL00364.1																	
823371..824136	+	AGL00355.1	CP003273.1	Desulfallas gibsoniae DSM 7213	Bacteria	Firmicutes	Clostridia	Clostridiales	Peptococcaceae	Desulfallas	CRISPR-associated endoribonuclease Cas6	cas6	COG1583	CAS-I,CAS-III,CAS-IV			
824196..824307	-	AGL00356.1	CP003273.1	Desulfallas gibsoniae DSM 7213	Bacteria	Firmicutes	Clostridia	Clostridiales	Peptococcaceae	Desulfallas	hypothetical protein						
824293..824548	-	AGL00357.1	CP003273.1	Desulfallas gibsoniae DSM 7213	Bacteria	Firmicutes	Clostridia	Clostridiales	Peptococcaceae	Desulfallas	hypothetical protein						
824742..826512	+	AGL00358.1	CP003273.1	Desulfallas gibsoniae DSM 7213	Bacteria	Firmicutes	Clostridia	Clostridiales	Peptococcaceae	Desulfallas	CRISPR-associated protein, TM1802 family	cas8b1	cd09730	CAS-I-B			
826501..827458	+	AGL00359.1	CP003273.1	Desulfallas gibsoniae DSM 7213	Bacteria	Firmicutes	Clostridia	Clostridiales	Peptococcaceae	Desulfallas	CRISPR-associated protein Cas7/Csh2, subtype I-B/HMARI	cas7	cd09690	CAS-I-B			
827468..828197	+	AGL00360.1	CP003273.1	Desulfallas gibsoniae DSM 7213	Bacteria	Firmicutes	Clostridia	Clostridiales	Peptococcaceae	Desulfallas	CRISPR-associated protein Cas5, subtype I-B/HMARI	cas5	cls001430	CAS-I-B			
828349..830698	+	AGL00361.1	CP003273.1	Desulfallas gibsoniae DSM 7213	Bacteria	Firmicutes	Clostridia	Clostridiales	Peptococcaceae	Desulfallas	CRISPR-associated helicase Cas3/CRISPR-associated endonuclease Cas3-HD	cas3HD,cas3	cd09641,COG1203	CAS-I			
830923..831430	+	AGL00362.1	CP003273.1	Desulfallas gibsoniae DSM 7213	Bacteria	Firmicutes	Clostridia	Clostridiales	Peptococcaceae	Desulfallas	CRISPR-associated protein Cas4	cas4	pfam01930	CAS-I			
831501..832494	+	AGL00363.1	CP003273.1	Desulfallas gibsoniae DSM 7213	Bacteria	Firmicutes	Clostridia	Clostridiales	Peptococcaceae	Desulfallas	CRISPR-associated endonuclease Cas1	cas1	cd09722	CAS-I-B			
832522..832786	+	AGL00364.1	CP003273.1	Desulfallas gibsoniae DSM 7213	Bacteria	Firmicutes	Clostridia	Clostridiales	Peptococcaceae	Desulfallas	CRISPR-associated endoribonuclease Cas2	cas2	cd09725	CAS-I,CAS-II,CAS-III,CAS-V			
832966..834392	array		CP003273.1	Desulfallas gibsoniae DSM 7213	Bacteria	Firmicutes	Clostridia	Clostridiales	Peptococcaceae	Desulfallas		CRISPR array			22	36	30
68 CAS-I-B | OAT86764.1																	
114925..116851	+	OAT86758.1	LYVF01000009.1	Desulfotomaculum copahuensis	Bacteria	Firmicutes	Clostridia	Clostridiales	Peptococcaceae	Desulfotomaculum	hypothetical protein	cas8b1	mkCas0074	CAS-I-B			
116944..117913	+	OAT86759.1	LYVF01000009.1	Desulfotomaculum copahuensis	Bacteria	Firmicutes	Clostridia	Clostridiales	Peptococcaceae	Desulfotomaculum	CRISPR-associated protein Csh2	cas7b	pfam05107	CAS-I-B,CAS-I-C			
117941..118670	+	OAT86760.1	LYVF01000009.1	Desulfotomaculum copahuensis	Bacteria	Firmicutes	Clostridia	Clostridiales	Peptococcaceae	Desulfotomaculum	hypothetical protein	cas5	cls001430	CAS-I-B			
118657..121075	+	OAT86761.1	LYVF01000009.1	Desulfotomaculum copahuensis	Bacteria	Firmicutes	Clostridia	Clostridiales	Peptococcaceae	Desulfotomaculum	hypothetical protein	cas3HD,cas3	cd09641,COG1203	CAS-I			
121109..121616	+	OAT86762.1	LYVF01000009.1	Desulfotomaculum copahuensis	Bacteria	Firmicutes	Clostridia	Clostridiales	Peptococcaceae	Desulfotomaculum	CRISPR-associated protein Cas4	cas4	pfam01930	CAS-I			
121639..122632	+	OAT86763.1	LYVF01000009.1	Desulfotomaculum copahuensis	Bacteria	Firmicutes	Clostridia	Clostridiales	Peptococcaceae	Desulfotomaculum	subtype I-B CRISPR-associated endonuclease Cas1	cas1	cd09722	CAS-I-B			
122969..123233	+	OAT86764.1	LYVF01000009.1	Desulfotomaculum copahuensis	Bacteria	Firmicutes	Clostridia	Clostridiales	Peptococcaceae	Desulfotomaculum	CRISPR-associated endonuclease Cas2	cas2	cd09725	CAS-I,CAS-II,CAS-III,CAS-V			
69 CAS-I-B | CCO07749.1																	
1283..5087	array		HF586928.1	Desulfotomaculum hydrothermale Lam5 = DSM 18033	Bacteria	Firmicutes	Clostridia	Clostridiales	Peptococcaceae	Desulfotomaculum		CRISPR array			58	36	30
5292..6576	-	CCO07748.1	HF586928.1	Desulfotomaculum hydrothermale Lam5 = DSM 18033	Bacteria	Firmicutes	Clostridia	Clostridiales	Peptococcaceae	Desulfotomaculum	transposase						
6735..7552	array		HF586928.1	Desulfotomaculum hydrothermale Lam5 = DSM 18033	Bacteria	Firmicutes	Clostridia	Clostridiales	Peptococcaceae	Desulfotomaculum		CRISPR array			13	35	30
7715..7964	-	CCO07749.1	HF586928.1	Desulfotomaculum hydrothermale Lam5 = DSM 18033	Bacteria	Firmicutes	Clostridia	Clostridiales	Peptococcaceae	Desulfotomaculum	conserved hypothetical protein	cas2	cd09725	CAS-I,CAS-II,CAS-III,CAS-V			
8141..9182	-	CCO07750.1	HF586928.1	Desulfotomaculum hydrothermale Lam5 = DSM 18033	Bacteria	Firmicutes	Clostridia	Clostridiales	Peptococcaceae	Desulfotomaculum	conserved hypothetical protein	cas1	cd09722	CAS-I-B			
9184..9658	-	CCO07751.1	HF586928.1	Desulfotomaculum hydrothermale Lam5 = DSM 18033	Bacteria	Firmicutes	Clostridia	Clostridiales	Peptococcaceae	Desulfotomaculum	CRISPR-associated protein Cas4	cas4	pfam01930	CAS-I			
9874..12196	-	CCO07752.1	HF586928.1	Desulfotomaculum hydrothermale Lam5 = DSM 18033	Bacteria	Firmicutes	Clostridia	Clostridiales	Peptococcaceae	Desulfotomaculum	CRISPR-associated helicase Cas3	cas3HD,cas3	cd09641,COG1203	CAS-I			
12206..12956	-	CCO07753.1	HF586928.1	Desulfotomaculum hydrothermale Lam5 = DSM 18033	Bacteria	Firmicutes	Clostridia	Clostridiales	Peptococcaceae	Desulfotomaculum	CRISPR-associated protein Cas5	cas5	cd09693	CAS-I			
12973..13846	-	CCO07754.1	HF586928.1	Desulfotomaculum hydrothermale Lam5 = DSM 18033	Bacteria	Firmicutes	Clostridia	Clostridiales	Peptococcaceae	Desulfotomaculum	CRISPR-associated regulatory protein, DevR family	cas7	cd09687	CAS-I-B			
13902..15612	-	CCO07755.1	HF586928.1	Desulfotomaculum hydrothermale Lam5 = DSM 18033	Bacteria	Firmicutes	Clostridia	Clostridiales	Peptococcaceae	Desulfotomaculum	conserved hypothetical protein	cas8b1	mkCas0195	CAS-I-B			
15686..16445	-	CCO07756.1	HF586928.1	Desulfotomaculum hydrothermale Lam5 = DSM 18033	Bacteria	Firmicutes	Clostridia	Clostridiales	Peptococcaceae	Desulfotomaculum	CRISPR-associated protein Cas6	cas6	COG1583	CAS-I,CAS-III,CAS-IV			
70 CAS-I-B | AQS58408.1																	
873290..874034	+	AQS58400.1	CP019698.1	Desulfotomaculum ferrireducens	Bacteria	Firmicutes	Clostridia	Clostridiales	Peptococcaceae	Desulfotomaculum	CRISPR-associated endoribonuclease Cas6	cas6	COG1583	CAS-I,CAS-III,CAS-IV			
874071..875583	+	AQS58401.1	CP019698.1	Desulfotomaculum ferrireducens	Bacteria	Firmicutes	Clostridia	Clostridiales	Peptococcaceae	Desulfotomaculum	hypothetical protein	cas8b1	cd09730	CAS-I-B			
875587..875995	+	AQS58402.1	CP019698.1	Desulfotomaculum ferrireducens	Bacteria	Firmicutes	Clostridia	Clostridiales	Peptococcaceae	Desulfotomaculum	CRISPR-associated protein	cas8b1	mkCas0119	CAS-I-B			
876013..876916	+	AQS58403.1	CP019698.1	Desulfotomaculum ferrireducens	Bacteria	Firmicutes	Clostridia	Clostridiales	Peptococcaceae	Desulfotomaculum	type I-B CRISPR-associated protein Cas7/Csh2	cas7	cd09690	CAS-I-B			
876920..877631	+	AQS58404.1	CP019698.1	Desulfotomaculum ferrireducens	Bacteria	Firmicutes	Clostridia	Clostridiales	Peptococcaceae	Desulfotomaculum	type I-B CRISPR-associated protein Cas5	cas5	cls001430	CAS-I-B			
877605..879801	+	AQS58405.1	CP019698.1	Desulfotomaculum ferrireducens	Bacteria	Firmicutes	Clostridia	Clostridiales	Peptococcaceae	Desulfotomaculum	hypothetical protein	cas3HD,cas3	cd09641,cd09639	CAS-I			
879837..880344	+	AQS58406.1	CP019698.1	Desulfotomaculum ferrireducens	Bacteria	Firmicutes	Clostridia	Clostridiales	Peptococcaceae	Desulfotomaculum	CRISPR-associated protein Cas4	cas4	pfam01930	CAS-I			
880361..881354	+	AQS58407.1	CP019698.1	Desulfotomaculum ferrireducens	Bacteria	Firmicutes	Clostridia	Clostridiales	Peptococcaceae	Desulfotomaculum	subtype I-B CRISPR-associated endonuclease Cas1	cas1	cd09722	CAS-I-B			
881410..881674	+	AQS58408.1	CP019698.1	Desulfotomaculum ferrireducens	Bacteria	Firmicutes	Clostridia	Clostridiales	Peptococcaceae	Desulfotomaculum	CRISPR-associated endonuclease Cas2	cas2	cd09725	CAS-I,CAS-II,CAS-III,CAS-V			
881837..886794	array		CP019698.1	Desulfotomaculum ferrireducens	Bacteria	Firmicutes	Clostridia	Clostridiales	Peptococcaceae	Desulfotomaculum		CRISPR array			75	36	30
887070..887778	-	AQS58409.1	CP019698.1	Desulfotomaculum ferrireducens	Bacteria	Firmicutes	Clostridia	Clostridiales	Peptococcaceae	Desulfotomaculum	RNA polymerase subunit sigma-70	casR*	cls001593				
71 CAS-I-B | SHF29859.1																	
55179..55968	+	SHF29519.1	FQUY01000017.1	Desulfotomaculum putei DSM 12395	Bacteria	Firmicutes	Clostridia	Clostridiales	Peptococcaceae	Desulfotomaculum	CRISPR-associated protein, Cas6 family	cas6	COG1583	CAS-I,CAS-III,CAS-IV			
55951..57700	+	SHF29548.1	FQUY01000017.1	Desulfotomaculum putei DSM 12395	Bacteria	Firmicutes	Clostridia	Clostridiales	Peptococcaceae	Desulfotomaculum	CRISPR-associated protein, Csh1 family	cas8b1	cd09730	CAS-I-B			
57689..58652	+	SHF29587.1	FQUY01000017.1	Desulfotomaculum putei DSM 12395	Bacteria	Firmicutes	Clostridia	Clostridiales	Peptococcaceae	Desulfotomaculum	CRISPR-associated protein, Csh2 family	cas7	cd09690	CAS-I-B			
58664..59393	+	SHF29613.1	FQUY01000017.1	Desulfotomaculum putei DSM 12395	Bacteria	Firmicutes	Clostridia	Clostridiales	Peptococcaceae	Desulfotomaculum	CRISPR-associated protein Cas5h	cas5	cls001430	CAS-I-B			
59576..60080	-	SHF29641.1	FQUY01000017.1	Desulfotomaculum putei DSM 12395	Bacteria	Firmicutes	Clostridia	Clostridiales	Peptococcaceae	Desulfotomaculum	Transposase IS116/IS110/IS902 family protein						
60088..60805	-	SHF29665.1	FQUY01000017.1	Desulfotomaculum putei DSM 12395	Bacteria	Firmicutes	Clostridia	Clostridiales	Peptococcaceae	Desulfotomaculum	Transposase						
61159..63589	+	SHF29692.1	FQUY01000017.1	Desulfotomaculum putei DSM 12395	Bacteria	Firmicutes	Clostridia	Clostridiales	Peptococcaceae	Desulfotomaculum	CRISPR-associated helicase, Cas3 family	cas3HD,cas3	cd09641,COG1203	CAS-I			
63617..64139	+	SHF29720.1	FQUY01000017.1	Desulfotomaculum putei DSM 12395	Bacteria	Firmicutes	Clostridia	Clostridiales	Peptococcaceae	Desulfotomaculum	CRISPR-associated exonuclease, Cas4 family	cas4	pfam01930	CAS-I			
64156..65149	+	SHF29774.1	FQUY01000017.1	Desulfotomaculum putei DSM 12395	Bacteria	Firmicutes	Clostridia	Clostridiales	Peptococcaceae	Desulfotomaculum	CRISPR-associated protein, Cas1 family	cas1	cd09722	CAS-I-B			
65232..65436	+	SHF29800.1	FQUY01000017.1	Desulfotomaculum putei DSM 12395	Bacteria	Firmicutes	Clostridia	Clostridiales	Peptococcaceae	Desulfotomaculum	hypothetical protein						
65566..65704	+	SHF29831.1	FQUY01000017.1	Desulfotomaculum putei DSM 12395	Bacteria	Firmicutes	Clostridia	Clostridiales	Peptococcaceae	Desulfotomaculum	hypothetical protein						
65954..66275	+	SHF29859.1	FQUY01000017.1	Desulfotomaculum putei DSM 12395	Bacteria	Firmicutes	Clostridia	Clostridiales	Peptococcaceae	Desulfotomaculum	CRISPR-associated protein, Cas2 family	cas2	cd09725	CAS-I,CAS-II,CAS-III,CAS-V			
72 CAS-I-B | PHJ39528.1																	
13..4562	array		AWQQ01000017.1	Desulfotomaculum profundi	Bacteria	Firmicutes	Clostridia	Clostridiales	Peptococcaceae	Desulfotomaculum		CRISPR array			69	36	30
4731..4995	-	PHJ39528.1	AWQQ01000017.1	Desulfotomaculum profundi	Bacteria	Firmicutes	Clostridia	Clostridiales	Peptococcaceae	Desulfotomaculum	CRISPR-associated protein Cas2	cas2	cd09725	CAS-I,CAS-II,CAS-III,CAS-V			
5080..6073	-	PHJ39529.1	AWQQ01000017.1	Desulfotomaculum profundi	Bacteria	Firmicutes	Clostridia	Clostridiales	Peptococcaceae	Desulfotomaculum	CRISPR-associated protein Cas1	cas1	cd09722	CAS-I-B			
6090..6597	-	PHJ39530.1	AWQQ01000017.1	Desulfotomaculum profundi	Bacteria	Firmicutes	Clostridia	Clostridiales	Peptococcaceae	Desulfotomaculum	CRISPR-associated protein Cas4	cas4	pfam01930	CAS-I			
6603..6726	-	PHJ39625.1	AWQQ01000017.1	Desulfotomaculum profundi	Bacteria	Firmicutes	Clostridia	Clostridiales	Peptococcaceae	Desulfotomaculum	hypothetical protein						
6715..7954	-	PHJ39626.1	AWQQ01000017.1	Desulfotomaculum profundi	Bacteria	Firmicutes	Clostridia	Clostridiales	Peptococcaceae	Desulfotomaculum	hypothetical protein	cas3	COG1203	CAS-I			
7965..8613	-	PHJ39627.1	AWQQ01000017.1	Desulfotomaculum profundi	Bacteria	Firmicutes	Clostridia	Clostridiales	Peptococcaceae	Desulfotomaculum	hypothetical protein	cas3	COG1203	CAS-I			
8702..8945	+	PHJ39628.1	AWQQ01000017.1	Desulfotomaculum profundi	Bacteria	Firmicutes	Clostridia	Clostridiales	Peptococcaceae	Desulfotomaculum	hypothetical protein						
8898..9609	-	PHJ39531.1	AWQQ01000017.1	Desulfotomaculum profundi	Bacteria	Firmicutes	Clostridia	Clostridiales	Peptococcaceae	Desulfotomaculum	CRISPR-associated protein Cas5	cas5	cls001430	CAS-I-B			
9613..10528	-	PHJ39532.1	AWQQ01000017.1	Desulfotomaculum profundi	Bacteria	Firmicutes	Clostridia	Clostridiales	Peptococcaceae	Desulfotomaculum	CRISPR-associated protein Csh2	cas7	cd09690	CAS-I-B			
10551..10962	-	PHJ39533.1	AWQQ01000017.1	Desulfotomaculum profundi	Bacteria	Firmicutes	Clostridia	Clostridiales	Peptococcaceae	Desulfotomaculum	CRISPR-associated protein	cas8b1	mkCas0119	CAS-I-B			
10966..12493	-	PHJ39629.1	AWQQ01000017.1	Desulfotomaculum profundi	Bacteria	Firmicutes	Clostridia	Clostridiales	Peptococcaceae	Desulfotomaculum	hypothetical protein	cas8b1	cd09730	CAS-I-B			
73 CAS-I-B | KEQ26917.1																	
242051..242614	array		JNVM01000005.1	Paenibacillus tyrfis	Bacteria	Firmicutes	Bacilli	Bacillales	Paenibacillaceae	Paenibacillus		CRISPR array			9	36	30
243580..244294	+	KEQ26911.1	JNVM01000005.1	Paenibacillus tyrfis	Bacteria	Firmicutes	Bacilli	Bacillales	Paenibacillaceae	Paenibacillus	hypothetical protein	cas6	COG1583	CAS-I,CAS-III,CAS-IV			
244401..246471	+	KEQ26912.1	JNVM01000005.1	Paenibacillus tyrfis	Bacteria	Firmicutes	Bacilli	Bacillales	Paenibacillaceae	Paenibacillus	hypothetical protein	cas8b1	pfam09484	CAS-I-B			
246460..247381	+	KEQ26913.1	JNVM01000005.1	Paenibacillus tyrfis	Bacteria	Firmicutes	Bacilli	Bacillales	Paenibacillaceae	Paenibacillus	CRISPR-associated protein Csd2	cas7b	pfam05107	CAS-I-B,CAS-I-C			
248198..250601	+	KEQ26914.1	JNVM01000005.1	Paenibacillus tyrfis	Bacteria	Firmicutes	Bacilli	Bacillales	Paenibacillaceae	Paenibacillus	hypothetical protein	cas3	COG1203	CAS-I			
250666..251161	+	KEQ26915.1	JNVM01000005.1	Paenibacillus tyrfis	Bacteria	Firmicutes	Bacilli	Bacillales	Paenibacillaceae	Paenibacillus	CRISPR-associated protein Cas4	cas4	pfam01930	CAS-I			
251197..252190	+	KEQ26916.1	JNVM01000005.1	Paenibacillus tyrfis	Bacteria	Firmicutes	Bacilli	Bacillales	Paenibacillaceae	Paenibacillus	CRISPR-associated protein Cas1	cas1	cd09722	CAS-I-B			
252232..252496	+	KEQ26917.1	JNVM01000005.1	Paenibacillus tyrfis	Bacteria	Firmicutes	Bacilli	Bacillales	Paenibacillaceae	Paenibacillus	CRISPR-associated protein Cas2	cas2	cd09725	CAS-I,CAS-II,CAS-III,CAS-V			
252648..253617	array		JNVM01000005.1	Paenibacillus tyrfis	Bacteria	Firmicutes	Bacilli	Bacillales	Paenibacillaceae	Paenibacillus		CRISPR array			15	37	30
74 CAS-I-B| AGA59185.1																	
3284570..3285001	array		CP003255.1	Thermobacillus composti KWC4	Bacteria	Firmicutes	Bacilli	Bacillales	Paenibacillaceae	Thermobacillus		CRISPR array			7	37	30
3285168..3285432	-	AGA59185.1	CP003255.1	Thermobacillus composti KWC4	Bacteria	Firmicutes	Bacilli	Bacillales	Paenibacillaceae	Thermobacillus	CRISPR-associated endoribonuclease Cas2	cas2	cd09725	CAS-I,CAS-II,CAS-III,CAS-V			
3285464..3286457	-	AGA59186.1	CP003255.1	Thermobacillus composti KWC4	Bacteria	Firmicutes	Bacilli	Bacillales	Paenibacillaceae	Thermobacillus	CRISPR-associated endonuclease Cas1	cas1	cd09722	CAS-I-B			
3286501..3287020	-	AGA59187.1	CP003255.1	Thermobacillus composti KWC4	Bacteria	Firmicutes	Bacilli	Bacillales	Paenibacillaceae	Thermobacillus	CRISPR-associated protein Cas4	cas4	pfam01930	CAS-I			
3287048..3289439	-	AGA59188.1	CP003255.1	Thermobacillus composti KWC4	Bacteria	Firmicutes	Bacilli	Bacillales	Paenibacillaceae	Thermobacillus	CRISPR-associated helicase Cas3	cas3HD,cas3	cd09641,cd09639	CAS-I			
3289426..3290116	-	AGA59189.1	CP003255.1	Thermobacillus composti KWC4	Bacteria	Firmicutes	Bacilli	Bacillales	Paenibacillaceae	Thermobacillus	CRISPR-associated protein Cas5	cas5	cls001430	CAS-I-B			
3290129..3291092	-	AGA59190.1	CP003255.1	Thermobacillus composti KWC4	Bacteria	Firmicutes	Bacilli	Bacillales	Paenibacillaceae	Thermobacillus	CRISPR-associated protein, CT1132 family	cas7b	pfam05107	CAS-I-B,CAS-I-C			
3291072..3293124	-	AGA59191.1	CP003255.1	Thermobacillus composti KWC4	Bacteria	Firmicutes	Bacilli	Bacillales	Paenibacillaceae	Thermobacillus	hypothetical protein	cas8b1	mkCas0074	CAS-I-B			
3293140..3293890	-	AGA59192.1	CP003255.1	Thermobacillus composti KWC4	Bacteria	Firmicutes	Bacilli	Bacillales	Paenibacillaceae	Thermobacillus	CRISPR-associated endoribonuclease Cas6	cas6	COG1583	CAS-I,CAS-III,CAS-IV			
3294083..3295376	+	AGA59193.1	CP003255.1	Thermobacillus composti KWC4	Bacteria	Firmicutes	Bacilli	Bacillales	Paenibacillaceae	Thermobacillus	transposase						
75 CAS-I-B | OUM86956.1																	
510..2562	+	OUM86951.1	LZRT01000082.1	Bacillus thermozeamaize	Bacteria	Firmicutes	Bacilli	Bacillales	Bacillaceae	Bacillus	hypothetical protein	cas8b1	mkCas0074	CAS-I-B			
2542..3502	+	OUM86952.1	LZRT01000082.1	Bacillus thermozeamaize	Bacteria	Firmicutes	Bacilli	Bacillales	Bacillaceae	Bacillus	CRISPR-associated protein Csh2	cas7b	pfam05107	CAS-I-B,CAS-I-C			
3515..4202	+	OUM86953.1	LZRT01000082.1	Bacillus thermozeamaize	Bacteria	Firmicutes	Bacilli	Bacillales	Bacillaceae	Bacillus	CRISPR-associated protein Cas5	cas5	cls001430	CAS-I-B			
4189..6580	+	OUM86954.1	LZRT01000082.1	Bacillus thermozeamaize	Bacteria	Firmicutes	Bacilli	Bacillales	Bacillaceae	Bacillus	CRISPR-associated helicase Cas3'	cas3HD,cas3	cd09641,COG1203	CAS-I			
6621..7131	+	OUM86957.1	LZRT01000082.1	Bacillus thermozeamaize	Bacteria	Firmicutes	Bacilli	Bacillales	Bacillaceae	Bacillus	CRISPR-associated protein Cas4	cas4	pfam01930	CAS-I			
7175..8168	+	OUM86955.1	LZRT01000082.1	Bacillus thermozeamaize	Bacteria	Firmicutes	Bacilli	Bacillales	Bacillaceae	Bacillus	subtype I-B CRISPR-associated endonuclease Cas1	cas1	cd09722	CAS-I-B			
8191..8455	+	OUM86956.1	LZRT01000082.1	Bacillus thermozeamaize	Bacteria	Firmicutes	Bacilli	Bacillales	Bacillaceae	Bacillus	CRISPR-associated endonuclease Cas2	cas2	cd09725	CAS-I,CAS-II,CAS-III,CAS-V			
8633..13254	array		LZRT01000082.1	Bacillus thermozeamaize	Bacteria	Firmicutes	Bacilli	Bacillales	Bacillaceae	Bacillus		CRISPR array			69	37	30
76 CAS-I-B | KGP75685.1																	
190..454	-	KGP75685.1	JQID01000121.1	Desulfosporosinus sp. Tol-M	Bacteria	Firmicutes	Clostridia	Clostridiales	Peptococcaceae	Desulfosporosinus	CRISPR-associated protein Cas2	cas2	cd09725	CAS-I,CAS-II,CAS-III,CAS-V			
490..1486	-	KGP75686.1	JQID01000121.1	Desulfosporosinus sp. Tol-M	Bacteria	Firmicutes	Clostridia	Clostridiales	Peptococcaceae	Desulfosporosinus	CRISPR-associated protein Cas1	cas1	cd09722	CAS-I-B			
1606..2119	-	KGP75687.1	JQID01000121.1	Desulfosporosinus sp. Tol-M	Bacteria	Firmicutes	Clostridia	Clostridiales	Peptococcaceae	Desulfosporosinus	CRISPR-associated protein Cas4	cas4	pfam01930	CAS-I			
2303..4703	-	KGP75688.1	JQID01000121.1	Desulfosporosinus sp. Tol-M	Bacteria	Firmicutes	Clostridia	Clostridiales	Peptococcaceae	Desulfosporosinus	CRISPR-associated protein Cas3	cas3HD,cas3	cd09641,COG1203	CAS-I			
4690..5380	-	KGP75689.1	JQID01000121.1	Desulfosporosinus sp. Tol-M	Bacteria	Firmicutes	Clostridia	Clostridiales	Peptococcaceae	Desulfosporosinus	CRISPR-associated protein Cas5	cas5	cls001430	CAS-I-B			
5392..6256	-	KGP75690.1	JQID01000121.1	Desulfosporosinus sp. Tol-M	Bacteria	Firmicutes	Clostridia	Clostridiales	Peptococcaceae	Desulfosporosinus	CRISPR-associated protein Csh2	cas7b	pfam05107	CAS-I-B,CAS-I-C			
78 CAS-I-B | OZC22041.1																	
5704..7400	array		LSLL01000046.1	Sporomusa acidovorans DSM 3132	Bacteria	Firmicutes	Negativicutes	Selenomonadales	Sporomusaceae	Sporomusa		CRISPR array			26	36	30
7482..8226	+	OZC22034.1	LSLL01000046.1	Sporomusa acidovorans DSM 3132	Bacteria	Firmicutes	Negativicutes	Selenomonadales	Sporomusaceae	Sporomusa	CRISPR associated protein Cas6	cas6	COG1583	CAS-I,CAS-III,CAS-IV			
8637..10668	+	OZC22035.1	LSLL01000046.1	Sporomusa acidovorans DSM 3132	Bacteria	Firmicutes	Negativicutes	Selenomonadales	Sporomusaceae	Sporomusa	hypothetical protein	cas8b1	mkCas0074	CAS-I-B			
10660..11611	+	OZC22036.1	LSLL01000046.1	Sporomusa acidovorans DSM 3132	Bacteria	Firmicutes	Negativicutes	Selenomonadales	Sporomusaceae	Sporomusa	hypothetical protein	cas7b	pfam05107	CAS-I-B,CAS-I-C			
11623..12304	+	OZC22037.1	LSLL01000046.1	Sporomusa acidovorans DSM 3132	Bacteria	Firmicutes	Negativicutes	Selenomonadales	Sporomusaceae	Sporomusa	hypothetical protein	cas5	cls001430	CAS-I-B			
12291..14715	+	OZC22038.1	LSLL01000046.1	Sporomusa acidovorans DSM 3132	Bacteria	Firmicutes	Negativicutes	Selenomonadales	Sporomusaceae	Sporomusa	helicase Cas3	cas3HD,cas3	cd09641,COG1203	CAS-I			
14742..15252	+	OZC22039.1	LSLL01000046.1	Sporomusa acidovorans DSM 3132	Bacteria	Firmicutes	Negativicutes	Selenomonadales	Sporomusaceae	Sporomusa	PD-(D/E)XK nuclease superfamily protein	cas4	pfam01930	CAS-I			
15265..16261	+	OZC22040.1	LSLL01000046.1	Sporomusa acidovorans DSM 3132	Bacteria	Firmicutes	Negativicutes	Selenomonadales	Sporomusaceae	Sporomusa	CRISPR-associated endonuclease Cas1	cas1	cd09722	CAS-I-B			
16431..16695	+	OZC22041.1	LSLL01000046.1	Sporomusa acidovorans DSM 3132	Bacteria	Firmicutes	Negativicutes	Selenomonadales	Sporomusaceae	Sporomusa	CRISPR-associated endoribonuclease Cas2	cas2	cd09725	CAS-I,CAS-II,CAS-III,CAS-V			
17060..18746	array		LSLL01000046.1	Sporomusa acidovorans DSM 3132	Bacteria	Firmicutes	Negativicutes	Selenomonadales	Sporomusaceae	Sporomusa		CRISPR array			26	36	30
79 CAS-I-B | EHL03891.1																	
1..226	array		JH414494.1	Desulfitobacterium hafniense DP7	Bacteria	Firmicutes	Clostridia	Clostridiales	Peptococcaceae	Desulfitobacterium		CRISPR array			4	36	29
75..224	-	EHL03898.1	JH414494.1	Desulfitobacterium hafniense DP7	Bacteria	Firmicutes	Clostridia	Clostridiales	Peptococcaceae	Desulfitobacterium	hypothetical protein						
232..1125	array		JH414494.1	Desulfitobacterium hafniense DP7	Bacteria	Firmicutes	Clostridia	Clostridiales	Peptococcaceae	Desulfitobacterium		CRISPR array			14	36	30
1353..1646	array		JH414494.1	Desulfitobacterium hafniense DP7	Bacteria	Firmicutes	Clostridia	Clostridiales	Peptococcaceae	Desulfitobacterium		CRISPR array			5	36	30
1811..2039	array		JH414494.1	Desulfitobacterium hafniense DP7	Bacteria	Firmicutes	Clostridia	Clostridiales	Peptococcaceae	Desulfitobacterium		CRISPR array			4	36	30
2183..2447	-	EHL03891.1	JH414494.1	Desulfitobacterium hafniense DP7	Bacteria	Firmicutes	Clostridia	Clostridiales	Peptococcaceae	Desulfitobacterium	CRISPR-associated protein Cas2	cas2	cd09725	CAS-I,CAS-II,CAS-III,CAS-V			
2547..3543	-	EHL03892.1	JH414494.1	Desulfitobacterium hafniense DP7	Bacteria	Firmicutes	Clostridia	Clostridiales	Peptococcaceae	Desulfitobacterium	CRISPR-associated endonuclease Cas1, HMARI/TNEAP subtype	cas1	cd09722	CAS-I-B			
3611..4121	-	EHL03893.1	JH414494.1	Desulfitobacterium hafniense DP7	Bacteria	Firmicutes	Clostridia	Clostridiales	Peptococcaceae	Desulfitobacterium	CRISPR-associated protein Cas4	cas4	pfam01930	CAS-I			
4143..6372	-	EHL03894.1	JH414494.1	Desulfitobacterium hafniense DP7	Bacteria	Firmicutes	Clostridia	Clostridiales	Peptococcaceae	Desulfitobacterium	CRISPR-associated helicase Cas3	cas3HD,cas3	cd09641,COG1203	CAS-I			
6375..6582	+	EHL03895.1	JH414494.1	Desulfitobacterium hafniense DP7	Bacteria	Firmicutes	Clostridia	Clostridiales	Peptococcaceae	Desulfitobacterium	hypothetical protein						
6959..7142	+	EHL03896.1	JH414494.1	Desulfitobacterium hafniense DP7	Bacteria	Firmicutes	Clostridia	Clostridiales	Peptococcaceae	Desulfitobacterium	hypothetical protein						
7118..7322	-	EHL03897.1	JH414494.1	Desulfitobacterium hafniense DP7	Bacteria	Firmicutes	Clostridia	Clostridiales	Peptococcaceae	Desulfitobacterium	hypothetical protein						
7609..8033	array		JH414494.1	Desulfitobacterium hafniense DP7	Bacteria	Firmicutes	Clostridia	Clostridiales	Peptococcaceae	Desulfitobacterium		CRISPR array			7	35	30
8320..8680	array		JH414494.1	Desulfitobacterium hafniense DP7	Bacteria	Firmicutes	Clostridia	Clostridiales	Peptococcaceae	Desulfitobacterium		CRISPR array			6	36	30
8689..9044	array		JH414494.1	Desulfitobacterium hafniense DP7	Bacteria	Firmicutes	Clostridia	Clostridiales	Peptococcaceae	Desulfitobacterium		CRISPR array			6	35	30
9050..9279	array		JH414494.1	Desulfitobacterium hafniense DP7	Bacteria	Firmicutes	Clostridia	Clostridiales	Peptococcaceae	Desulfitobacterium		CRISPR array			4	36	30
9486..10182	-	EHL03878.1	JH414494.1	Desulfitobacterium hafniense DP7	Bacteria	Firmicutes	Clostridia	Clostridiales	Peptococcaceae	Desulfitobacterium	putative CRISPR-associated protein Cas5, Hmari subtype	cas5	cls001430	CAS-I-B			
10225..11191	-	EHL03879.1	JH414494.1	Desulfitobacterium hafniense DP7	Bacteria	Firmicutes	Clostridia	Clostridiales	Peptococcaceae	Desulfitobacterium	hypothetical protein	cas7b	pfam05107	CAS-I-B,CAS-I-C			

11168..13241	-	EHL03880.1	JH414494.1	Desulfitobacterium hafniense DP7	Bacteria	Firmicutes	Clostridia	Clostridiales	Peptococcaceae	Desulfitobacterium	hypothetical protein	cas8b1	mkCas0074	CAS-I-B			
13396..13741	-	EHL03881.1	JH414494.1	Desulfitobacterium hafniense DP7	Bacteria	Firmicutes	Clostridia	Clostridiales	Peptococcaceae	Desulfitobacterium	putative toxin-antitoxin system, antitoxin component						
13730..14039	-	EHL03882.1	JH414494.1	Desulfitobacterium hafniense DP7	Bacteria	Firmicutes	Clostridia	Clostridiales	Peptococcaceae	Desulfitobacterium	nucleotidyltransferase domain protein						
14630..16046	+	EHL03883.1	JH414494.1	Desulfitobacterium hafniense DP7	Bacteria	Firmicutes	Clostridia	Clostridiales	Peptococcaceae	Desulfitobacterium	hypothetical protein	C3a187,C3a187	cluster187,cluster187				
16042..16726	+	EHL03884.1	JH414494.1	Desulfitobacterium hafniense DP7	Bacteria	Firmicutes	Clostridia	Clostridiales	Peptococcaceae	Desulfitobacterium	hypothetical protein						
16691..18359	+	EHL03885.1	JH414494.1	Desulfitobacterium hafniense DP7	Bacteria	Firmicutes	Clostridia	Clostridiales	Peptococcaceae	Desulfitobacterium	ADP-ribosylglycohydrolase						
18393..18639	-	EHL03886.1	JH414494.1	Desulfitobacterium hafniense DP7	Bacteria	Firmicutes	Clostridia	Clostridiales	Peptococcaceae	Desulfitobacterium	hypothetical protein						
18771..19557	-	EHL03887.1	JH414494.1	Desulfitobacterium hafniense DP7	Bacteria	Firmicutes	Clostridia	Clostridiales	Peptococcaceae	Desulfitobacterium	CRISPR-associated endoribonuclease Cas6	cas6	COG1583	CAS-I,CAS-III,CAS-IV			

80 CAS-I-B | SEP04969.1

46911..47267	array		FODY01000009.1	Propionispora vibrioides	Bacteria	Firmicutes	Negativicutes	Selenomonadales	Sporomusaceae	Propionispora		CRISPR array			6	35	30
47791..47917	+	SEP04733.1	FODY01000009.1	Propionispora vibrioides	Bacteria	Firmicutes	Negativicutes	Selenomonadales	Sporomusaceae	Propionispora	hypothetical protein						
48123..50106	+	SEP04768.1	FODY01000009.1	Propionispora vibrioides	Bacteria	Firmicutes	Negativicutes	Selenomonadales	Sporomusaceae	Propionispora	CRISPR-associated protein, Csh1 family	cas8b1	pfam09484	CAS-I-B			
50068..50962	+	SEP04808.1	FODY01000009.1	Propionispora vibrioides	Bacteria	Firmicutes	Negativicutes	Selenomonadales	Sporomusaceae	Propionispora	CRISPR-associated protein, Csh2 family	cas7	cd09690	CAS-I-B			
50972..51698	+	SEP04843.1	FODY01000009.1	Propionispora vibrioides	Bacteria	Firmicutes	Negativicutes	Selenomonadales	Sporomusaceae	Propionispora	CRISPR-associated protein, Cas5h family	cas5	cd09692	CAS-I-B			
51713..54062	+	SEP04878.1	FODY01000009.1	Propionispora vibrioides	Bacteria	Firmicutes	Negativicutes	Selenomonadales	Sporomusaceae	Propionispora	CRISPR-associated helicase, Cas3 family	cas3HD,cas3	cd09641,COG1203	CAS-I			
54257..54767	+	SEP04912.1	FODY01000009.1	Propionispora vibrioides	Bacteria	Firmicutes	Negativicutes	Selenomonadales	Sporomusaceae	Propionispora	CRISPR-associated exonuclease, Cas4 family	cas4	pfam01930	CAS-I			
54780..55776	+	SEP04942.1	FODY01000009.1	Propionispora vibrioides	Bacteria	Firmicutes	Negativicutes	Selenomonadales	Sporomusaceae	Propionispora	CRISPR-associated protein, Cas1 family	cas1	cd09722	CAS-I-B			
55832..56096	+	SEP04969.1	FODY01000009.1	Propionispora vibrioides	Bacteria	Firmicutes	Negativicutes	Selenomonadales	Sporomusaceae	Propionispora	CRISPR-associated protein, Cas2 family	cas2	cd09725	CAS-I,CAS-II,CAS-III,CAS-V			
59199..59692	array		FODY01000009.1	Propionispora vibrioides	Bacteria	Firmicutes	Negativicutes	Selenomonadales	Sporomusaceae	Propionispora		CRISPR array			8	35	31

81 CAS-I-B | ATW27429.1

5281557..5282253	+	ATW28865.1	CP017634.1	Peptococcaceae bacterium DCMF	Bacteria	Firmicutes	Clostridia	Clostridiales	Peptococcaceae		CRISPR-associated endoribonuclease Cas6	cas6	COG1583	CAS-I,CAS-III,CAS-IV			
5282306..5282524	array		CP017634.1	Peptococcaceae bacterium DCMF	Bacteria	Firmicutes	Clostridia	Clostridiales	Peptococcaceae			CRISPR array			4	47	19
5282477..5282693	+	ATW27423.1	CP017634.1	Peptococcaceae bacterium DCMF	Bacteria	Firmicutes	Clostridia	Clostridiales	Peptococcaceae		hypothetical protein						
5282785..5284522	+	ATW27424.1	CP017634.1	Peptococcaceae bacterium DCMF	Bacteria	Firmicutes	Clostridia	Clostridiales	Peptococcaceae		hypothetical protein	cas8b1	mkCas0195	CAS-I-B			
5284521..5285412	+	ATW27425.1	CP017634.1	Peptococcaceae bacterium DCMF	Bacteria	Firmicutes	Clostridia	Clostridiales	Peptococcaceae		type I-B CRISPR-associated protein Cas7/Cst2/DevR	cas7	cd09687	CAS-I-B			
5285421..5286129	+	ATW27426.1	CP017634.1	Peptococcaceae bacterium DCMF	Bacteria	Firmicutes	Clostridia	Clostridiales	Peptococcaceae		hypothetical protein	cas5	cd09658	CAS-I-B			
5286125..5288369	+	ATW27427.1	CP017634.1	Peptococcaceae bacterium DCMF	Bacteria	Firmicutes	Clostridia	Clostridiales	Peptococcaceae		hypothetical protein	cas3HD,cas3	cd09641,COG1203	CAS-I			
5288432..5288903	+	ATW28866.1	CP017634.1	Peptococcaceae bacterium DCMF	Bacteria	Firmicutes	Clostridia	Clostridiales	Peptococcaceae		CRISPR-associated protein Cas4	cas4	pfam01930	CAS-I			
5288915..5289908	+	ATW27428.1	CP017634.1	Peptococcaceae bacterium DCMF	Bacteria	Firmicutes	Clostridia	Clostridiales	Peptococcaceae		subtype I-B CRISPR-associated endonuclease Cas1	cas1	cd09722	CAS-I-B			
5289928..5290192	+	ATW27429.1	CP017634.1	Peptococcaceae bacterium DCMF	Bacteria	Firmicutes	Clostridia	Clostridiales	Peptococcaceae		CRISPR-associated endonuclease Cas2	cas2	cd09725	CAS-I,CAS-II,CAS-III,CAS-V			
5290394..5298227	array		CP017634.1	Peptococcaceae bacterium DCMF	Bacteria	Firmicutes	Clostridia	Clostridiales	Peptococcaceae			CRISPR array			119	36	30

82 CAS-I-B | ADG83325.1

2575925..2582292	array		CP002028.1	Thermincola potens JR	Bacteria	Firmicutes	Clostridia	Clostridiales	Peptococcaceae	Thermincola		CRISPR array			95	37	30
2582552..2582816	-	ADG83325.1	CP002028.1	Thermincola potens JR	Bacteria	Firmicutes	Clostridia	Clostridiales	Peptococcaceae	Thermincola	CRISPR-associated protein Cas2	cas2	cd09725	CAS-I,CAS-II,CAS-III,CAS-V			
2582838..2583831	-	ADG83326.1	CP002028.1	Thermincola potens JR	Bacteria	Firmicutes	Clostridia	Clostridiales	Peptococcaceae	Thermincola	CRISPR-associated protein Cas1	cas1	cd09722	CAS-I-B			
2583843..2584347	-	ADG83327.1	CP002028.1	Thermincola potens JR	Bacteria	Firmicutes	Clostridia	Clostridiales	Peptococcaceae	Thermincola	CRISPR-associated protein Cas4	cas4	pfam01930	CAS-I			
2584368..2586609	-	ADG83328.1	CP002028.1	Thermincola potens JR	Bacteria	Firmicutes	Clostridia	Clostridiales	Peptococcaceae	Thermincola	metal dependent phosphohydrolase	cas3HD,cas3	cd09641,cd09639	CAS-I			
2586559..2587276	-	ADG83329.1	CP002028.1	Thermincola potens JR	Bacteria	Firmicutes	Clostridia	Clostridiales	Peptococcaceae	Thermincola	CRISPR-associated protein Cas5, Hmari subtype	cas5,cas5	cls001430,mkCas0191	CAS-I-B			
2587281..2588190	-	ADG83330.1	CP002028.1	Thermincola potens JR	Bacteria	Firmicutes	Clostridia	Clostridiales	Peptococcaceae	Thermincola	CRISPR-associated protein, Csh2 family	cas7	cd09690	CAS-I-B			
2588193..2588616	-	ADG83331.1	CP002028.1	Thermincola potens JR	Bacteria	Firmicutes	Clostridia	Clostridiales	Peptococcaceae	Thermincola	conserved hypothetical protein	cas8b1	mkCas0119	CAS-I-B			
2588612..2590118	-	ADG83332.1	CP002028.1	Thermincola potens JR	Bacteria	Firmicutes	Clostridia	Clostridiales	Peptococcaceae	Thermincola	CRISPR-associated protein TM1802 family	cas8b1	cd09730	CAS-I-B			
2590512..2591082	+	ADG83333.1	CP002028.1	Thermincola potens JR	Bacteria	Firmicutes	Clostridia	Clostridiales	Peptococcaceae	Thermincola	protein of unknown function DUF16	CorA	icity0114				
2591113..2591851	-	ADG83334.1	CP002028.1	Thermincola potens JR	Bacteria	Firmicutes	Clostridia	Clostridiales	Peptococcaceae	Thermincola	CRISPR-associated protein Cas6	cas6	COG1583	CAS-I,CAS-III,CAS-IV			

83 CAS-I-B | KNZ69508.1

315..3148	array		LGTE01000011.1	Thermincola ferriacetica	Bacteria	Firmicutes	Clostridia	Clostridiales	Peptococcaceae	Thermincola		CRISPR array			43	36	30
3385..3649	-	KNZ69508.1	LGTE01000011.1	Thermincola ferriacetica	Bacteria	Firmicutes	Clostridia	Clostridiales	Peptococcaceae	Thermincola	CRISPR-associated protein Cas2	cas2	cd09725	CAS-I,CAS-II,CAS-III,CAS-V			
3671..4664	-	KNZ69509.1	LGTE01000011.1	Thermincola ferriacetica	Bacteria	Firmicutes	Clostridia	Clostridiales	Peptococcaceae	Thermincola	CRISPR-associated protein Cas1	cas1	cd09722	CAS-I-B			
4676..5108	-	KNZ69510.1	LGTE01000011.1	Thermincola ferriacetica	Bacteria	Firmicutes	Clostridia	Clostridiales	Peptococcaceae	Thermincola	CRISPR-associated protein Cas4	cas4	pfam01930	CAS-I			
5205..7740	-	KNZ69511.1	LGTE01000011.1	Thermincola ferriacetica	Bacteria	Firmicutes	Clostridia	Clostridiales	Peptococcaceae	Thermincola	metal dependent phosphohydrolase	cas3HD,cas3	cd09641,COG1203	CAS-I			
7740..8484	-	KNZ69512.1	LGTE01000011.1	Thermincola ferriacetica	Bacteria	Firmicutes	Clostridia	Clostridiales	Peptococcaceae	Thermincola	CRISPR-associated protein Cas5	cas5	cd09692	CAS-I-B			
8500..9448	-	KNZ69513.1	LGTE01000011.1	Thermincola ferriacetica	Bacteria	Firmicutes	Clostridia	Clostridiales	Peptococcaceae	Thermincola	Csh2 family CRISPR-associated protein	cas7b	pfam05107	CAS-I-B,CAS-I-C			
9422..11399	-	KNZ69514.1	LGTE01000011.1	Thermincola ferriacetica	Bacteria	Firmicutes	Clostridia	Clostridiales	Peptococcaceae	Thermincola	CRISPR-associated protein family	cas8b1	pfam09484	CAS-I-B			
11731..12238	+	KNZ69515.1	LGTE01000011.1	Thermincola ferriacetica	Bacteria	Firmicutes	Clostridia	Clostridiales	Peptococcaceae	Thermincola	hypothetical protein	CorA,CorA,CorA	icity0114,icity0114,icity0114				
12269..13007	-	KNZ69516.1	LGTE01000011.1	Thermincola ferriacetica	Bacteria	Firmicutes	Clostridia	Clostridiales	Peptococcaceae	Thermincola	CRISPR-associated protein Cas6	cas6	COG1583	CAS-I,CAS-III,CAS-IV			

84 CAS-I-B | KJS01564.1

38..2572	array		LADN01000033.1	Peptococcaceae bacterium BRH_c4a	Bacteria	Firmicutes	Clostridia	Clostridiales	Peptococcaceae			CRISPR array			39	35	30
2761..3025	-	KJS01564.1	LADN01000033.1	Peptococcaceae bacterium BRH_c4a	Bacteria	Firmicutes	Clostridia	Clostridiales	Peptococcaceae		CRISPR-associated protein Cas2	cas2	cd09725	CAS-I,CAS-II,CAS-III,CAS-V			
3046..4039	-	KJS01565.1	LADN01000033.1	Peptococcaceae bacterium BRH_c4a	Bacteria	Firmicutes	Clostridia	Clostridiales	Peptococcaceae		CRISPR-associated protein Cas1	cas1	cd09722	CAS-I-B			
4052..4577	-	KJS01612.1	LADN01000033.1	Peptococcaceae bacterium BRH_c4a	Bacteria	Firmicutes	Clostridia	Clostridiales	Peptococcaceae		CRISPR-associated protein Cas4	cas4	pfam01930	CAS-I			
4783..7312	-	KJS01613.1	LADN01000033.1	Peptococcaceae bacterium BRH_c4a	Bacteria	Firmicutes	Clostridia	Clostridiales	Peptococcaceae		hypothetical protein	cas3HD,cas3	cd09641,COG1203	CAS-I			
7335..8064	-	KJS01614.1	LADN01000033.1	Peptococcaceae bacterium BRH_c4a	Bacteria	Firmicutes	Clostridia	Clostridiales	Peptococcaceae		hypothetical protein	cas5	cls001430	CAS-I-B			
8099..9293	-	KJS01566.1	LADN01000033.1	Peptococcaceae bacterium BRH_c4a	Bacteria	Firmicutes	Clostridia	Clostridiales	Peptococcaceae		hypothetical protein	cas7b	pfam05107	CAS-I-B,CAS-I-C			
9319..11380	-	KJS01567.1	LADN01000033.1	Peptococcaceae bacterium BRH_c4a	Bacteria	Firmicutes	Clostridia	Clostridiales	Peptococcaceae		hypothetical protein	cas8b1	pfam09484	CAS-I-B			
11438..12176	-	KJS01568.1	LADN01000033.1	Peptococcaceae bacterium BRH_c4a	Bacteria	Firmicutes	Clostridia	Clostridiales	Peptococcaceae		CRISPR-associated protein Cas6	cas6	COG1583	CAS-I,CAS-III,CAS-IV			
12418..13456	-	KJS01569.1	LADN01000033.1	Peptococcaceae bacterium BRH_c4a	Bacteria	Firmicutes	Clostridia	Clostridiales	Peptococcaceae		transcriptional regulator	WYL	pfam13280	CAS-I,CAS-III			

85 CAS-I-B | PKM81768.1

117141..117858	+	PKM81767.1	PHAB01000008.1	Firmicutes bacterium HGW-Firmicutes-14	Bacteria	Firmicutes					CRISPR-associated endoribonuclease Cas6	cas6	COG1583	CAS-I,CAS-III,CAS-IV			
117936..118212	-	PKM81751.1	PHAB01000008.1	Firmicutes bacterium HGW-Firmicutes-14	Bacteria	Firmicutes					AbrB/MazE/SpoVT family DNA-binding domain-containing protein						
118412..120131	+	PKM81752.1	PHAB01000008.1	Firmicutes bacterium HGW-Firmicutes-14	Bacteria	Firmicutes					hypothetical protein	cas8b1	mkCas0195	CAS-I-B			
120202..121072	+	PKM81753.1	PHAB01000008.1	Firmicutes bacterium HGW-Firmicutes-14	Bacteria	Firmicutes					type I-B CRISPR-associated protein Cas7/Cst2/DevR	cas7	cd09687	CAS-I-B			
121092..121830	+	PKM81754.1	PHAB01000008.1	Firmicutes bacterium HGW-Firmicutes-14	Bacteria	Firmicutes					CRISPR-associated protein Cas5	cas5	cd09693	CAS-I			
121839..124161	+	PKM81755.1	PHAB01000008.1	Firmicutes bacterium HGW-Firmicutes-14	Bacteria	Firmicutes					CRISPR-associated helicase/endonuclease Cas3	cas3HD,cas3,cas3	cd09641,COG1203,cd09696	CAS-I			
124186..124696	+	PKM81756.1	PHAB01000008.1	Firmicutes bacterium HGW-Firmicutes-14	Bacteria	Firmicutes					CRISPR-associated protein Cas4	cas4	pfam01930	CAS-I			
124727..125720	+	PKM81757.1	PHAB01000008.1	Firmicutes bacterium HGW-Firmicutes-14	Bacteria	Firmicutes					subtype I-B CRISPR-associated endonuclease Cas1	cas1	cd09722	CAS-I-B			
125743..126007	+	PKM81768.1	PHAB01000008.1	Firmicutes bacterium HGW-Firmicutes-14	Bacteria	Firmicutes					CRISPR-associated endonuclease Cas2	cas2	cd09725	CAS-I,CAS-II,CAS-III,CAS-V			
126214..134752	array		PHAB01000008.1	Firmicutes bacterium HGW-Firmicutes-14	Bacteria	Firmicutes						CRISPR array			130	35	30

86 CAS-I-B | SNX53624.1

1257414..1257672	-	SNX53624.1	LT906662.1	Thermoanaerobacterium sp. RBIITD	Bacteria	Firmicutes	Clostridia	Thermoanaerobacterales	Thermoanaerobacterales Family III. Incertae Sedis	Thermoanaerobacterium	CRISPR-associated protein, Cas2 family	cas2	cd09725	CAS-I,CAS-II,CAS-III,CAS-V			
1257668..1257830	-	SNX53625.1	LT906662.1	Thermoanaerobacterium sp. RBIITD	Bacteria	Firmicutes	Clostridia	Thermoanaerobacterales	Thermoanaerobacterales Family III. Incertae Sedis	Thermoanaerobacterium	CRISPR associated protein Cas1	cas1	cd09722	CAS-I-B			
1257826..1258324	-	SNX53626.1	LT906662.1	Thermoanaerobacterium sp. RBIITD	Bacteria	Firmicutes	Clostridia	Thermoanaerobacterales	Thermoanaerobacterales Family III. Incertae Sedis	Thermoanaerobacterium	CRISPR-associated exonuclease Cas4	cas4	pfam01930	CAS-I			
1258409..1258572	array		LT906662.1	Thermoanaerobacterium sp. RBIITD	Bacteria	Firmicutes	Clostridia	Thermoanaerobacterales	Thermoanaerobacterales Family III. Incertae Sedis	Thermoanaerobacterium		CRISPR array			3	37	30
1258754..1261115	-	SNX53627.1	LT906662.1	Thermoanaerobacterium sp. RBIITD	Bacteria	Firmicutes	Clostridia	Thermoanaerobacterales	Thermoanaerobacterales Family III. Incertae Sedis	Thermoanaerobacterium	CRISPR-associated helicase, Cas3 family	cas3HD,cas3	cd09641,COG1203	CAS-I			
1261092..1261809	-	SNX53628.1	LT906662.1	Thermoanaerobacterium sp. RBIITD	Bacteria	Firmicutes	Clostridia	Thermoanaerobacterales	Thermoanaerobacterales Family III. Incertae Sedis	Thermoanaerobacterium	CRISPR-associated protein Cas5h	cas5	cls001430	CAS-I-B			
1261900..1262878	-	SNX53629.1	LT906662.1	Thermoanaerobacterium sp. RBIITD	Bacteria	Firmicutes	Clostridia	Thermoanaerobacterales	Thermoanaerobacterales Family III. Incertae Sedis	Thermoanaerobacterium	CRISPR-associated protein Csh2	cas7	cd09690	CAS-I-B			
1262878..1264636	-	SNX53630.1	LT906662.1	Thermoanaerobacterium sp. RBIITD	Bacteria	Firmicutes	Clostridia	Thermoanaerobacterales	Thermoanaerobacterales Family III. Incertae Sedis	Thermoanaerobacterium	CRISPR-associated protein, Csh1 family	cas8b1	cd09730	CAS-I-B			
1264651..1265392	-	SNX53631.1	LT906662.1	Thermoanaerobacterium sp. RBIITD	Bacteria	Firmicutes	Clostridia	Thermoanaerobacterales	Thermoanaerobacterales Family III. Incertae Sedis	Thermoanaerobacterium	CRISPR-associated endoribonuclease Cas6	cas6	COG1583	CAS-I,CAS-III,CAS-IV			

87 partial CAS-I | AGB20220.1

2635296..2635740	-	AGB20213.1	CP003066.1	Thermoanaerobacterium thermosaccharolyticum M0795	Bacteria	Firmicutes	Clostridia	Thermoanaerobacterales	Thermoanaerobacterales Family III. Incertae Sedis	Thermoanaerobacterium	transcriptional regulator	casR	cls001593	CAS-I			
2635739..2635877	-	AGB20214.1	CP003066.1	Thermoanaerobacterium thermosaccharolyticum M0795	Bacteria	Firmicutes	Clostridia	Thermoanaerobacterales	Thermoanaerobacterales Family III. Incertae Sedis	Thermoanaerobacterium	hypothetical protein						
2635873..2637439	-	AGB20215.1	CP003066.1	Thermoanaerobacterium thermosaccharolyticum M0795	Bacteria	Firmicutes	Clostridia	Thermoanaerobacterales	Thermoanaerobacterales Family III. Incertae Sedis	Thermoanaerobacterium	chloride channel protein EriC						
2637645..2638266	+	AGB20216.1	CP003066.1	Thermoanaerobacterium thermosaccharolyticum M0795	Bacteria	Firmicutes	Clostridia	Thermoanaerobacterales	Thermoanaerobacterales Family III. Incertae Sedis	Thermoanaerobacterium	putative regulatory ligand binding protein, C-terminal domain of K+ channels like protein	casR	cls001593	CAS-I			
2638386..2639496	+	AGB20217.1	CP003066.1	Thermoanaerobacterium thermosaccharolyticum M0795	Bacteria	Firmicutes	Clostridia	Thermoanaerobacterales	Thermoanaerobacterales Family III. Incertae Sedis	Thermoanaerobacterium	glycine betaine/L-proline transport ATP binding subunit	Cas_RecF	cluster17				
2639508..2640147	+	AGB20218.1	CP003066.1	Thermoanaerobacterium thermosaccharolyticum M0795	Bacteria	Firmicutes	Clostridia	Thermoanaerobacterales	Thermoanaerobacterales Family III. Incertae Sedis	Thermoanaerobacterium	ABC-type proline/glycine betaine transport system, permease component						
2640161..2641052	+	AGB20219.1	CP003066.1	Thermoanaerobacterium thermosaccharolyticum M0795	Bacteria	Firmicutes	Clostridia	Thermoanaerobacterales	Thermoanaerobacterales Family III. Incertae Sedis	Thermoanaerobacterium	periplasmic glycine betaine/choline-binding (lipo)protein of an ABC-type transport system (osmoprotectant binding protein)						
2641082..2641346	-	AGB20220.1	CP003066.1	Thermoanaerobacterium thermosaccharolyticum M0795	Bacteria	Firmicutes	Clostridia	Thermoanaerobacterales	Thermoanaerobacterales Family III. Incertae Sedis	Thermoanaerobacterium	CRISPR-associated endoribonuclease Cas2	cas2	cd09725	CAS-I,CAS-II,CAS-III,CAS-V			
2641358..2642351	-	AGB20221.1	CP003066.1	Thermoanaerobacterium thermosaccharolyticum M0795	Bacteria	Firmicutes	Clostridia	Thermoanaerobacterales	Thermoanaerobacterales Family III. Incertae Sedis	Thermoanaerobacterium	CRISPR-associated endonuclease Cas1	cas1	cd09722	CAS-I-B			
2642347..2642845	-	AGB20222.1	CP003066.1	Thermoanaerobacterium thermosaccharolyticum M0795	Bacteria	Firmicutes	Clostridia	Thermoanaerobacterales	Thermoanaerobacterales Family III. Incertae Sedis	Thermoanaerobacterium	CRISPR-associated protein Cas4	cas4	pfam01930	CAS-I			
2642829..2643153	-	AGB20223.1	CP003066.1	Thermoanaerobacterium thermosaccharolyticum M0795	Bacteria	Firmicutes	Clostridia	Thermoanaerobacterales	Thermoanaerobacterales Family III. Incertae Sedis	Thermoanaerobacterium	hypothetical protein						
2643118..2643337	-	AGB20224.1	CP003066.1	Thermoanaerobacterium thermosaccharolyticum M0795	Bacteria	Firmicutes	Clostridia	Thermoanaerobacterales	Thermoanaerobacterales Family III. Incertae Sedis	Thermoanaerobacterium	hypothetical protein						
2643367..2661243	array		CP003066.1	Thermoanaerobacterium thermosaccharolyticum M0795	Bacteria	Firmicutes	Clostridia	Thermoanaerobacterales	Thermoanaerobacterales Family III. Incertae Sedis	Thermoanaerobacterium		CRISPR array			267	37	30
2661344..2668991	array		CP003066.1	Thermoanaerobacterium thermosaccharolyticum M0795	Bacteria	Firmicutes	Clostridia	Thermoanaerobacterales	Thermoanaerobacterales Family III. Incertae Sedis	Thermoanaerobacterium		CRISPR array			115	36	30
2669159..2671523	-	AGB20225.1	CP003066.1	Thermoanaerobacterium thermosaccharolyticum M0795	Bacteria	Firmicutes	Clostridia	Thermoanaerobacterales	Thermoanaerobacterales Family III. Incertae Sedis	Thermoanaerobacterium	putative helicase	cas3HD,cas3	cd09641,COG1203	CAS-I			

88 CAS-I-B| ADL70063.1

2634520..2634964	-	ADL70059.1	CP002171.1	Thermoanaerobacterium thermosaccharolyticum DSM 571	Bacteria	Firmicutes	Clostridia	Thermoanaerobacterales	Thermoanaerobacterales Family III. Incertae Sedis	Thermoanaerobacterium	transcriptional regulator, MarR family	casR	cls001593	CAS-I			
2634963..2635101	-	ADL70060.1	CP002171.1	Thermoanaerobacterium thermosaccharolyticum DSM 571	Bacteria	Firmicutes	Clostridia	Thermoanaerobacterales	Thermoanaerobacterales Family III. Incertae Sedis	Thermoanaerobacterium	hypothetical protein						
2635097..2636663	-	ADL70061.1	CP002171.1	Thermoanaerobacterium thermosaccharolyticum DSM 571	Bacteria	Firmicutes	Clostridia	Thermoanaerobacterales	Thermoanaerobacterales Family III. Incertae Sedis	Thermoanaerobacterium	Cl- channel voltage-gated family protein						
2636802..2638005	-	ADL70062.1	CP002171.1	Thermoanaerobacterium thermosaccharolyticum DSM 571	Bacteria	Firmicutes	Clostridia	Thermoanaerobacterales	Thermoanaerobacterales Family III. Incertae Sedis	Thermoanaerobacterium	CRISPR-associated protein	csx1	cd09732	CAS-III			
2638027..2638291	-	ADL70063.1	CP002171.1	Thermoanaerobacterium thermosaccharolyticum DSM 571	Bacteria	Firmicutes	Clostridia	Thermoanaerobacterales	Thermoanaerobacterales Family III. Incertae Sedis	Thermoanaerobacterium	CRISPR-associated protein Cas2	cas2	cd09725	CAS-I,CAS-II,CAS-III,CAS-V			
2638303..2639296	-	ADL70064.1	CP002171.1	Thermoanaerobacterium thermosaccharolyticum DSM 571	Bacteria	Firmicutes	Clostridia	Thermoanaerobacterales	Thermoanaerobacterales Family III. Incertae Sedis	Thermoanaerobacterium	CRISPR-associated protein Cas1	cas1	cd09722	CAS-I-B			
2639292..2639790	-	ADL70065.1	CP002171.1	Thermoanaerobacterium thermosaccharolyticum DSM 571	Bacteria	Firmicutes	Clostridia	Thermoanaerobacterales	Thermoanaerobacterales Family III. Incertae Sedis	Thermoanaerobacterium	CRISPR-associated protein Cas4	cas4	pfam01930	CAS-I			
2639866..2639986	-	ADL70066.1	CP002171.1	Thermoanaerobacterium thermosaccharolyticum DSM 571	Bacteria	Firmicutes	Clostridia	Thermoanaerobacterales	Thermoanaerobacterales Family III. Incertae Sedis	Thermoanaerobacterium	hypothetical protein						
2639967..2640090	-	ADL70067.1	CP002171.1	Thermoanaerobacterium thermosaccharolyticum DSM 571	Bacteria	Firmicutes	Clostridia	Thermoanaerobacterales	Thermoanaerobacterales Family III. Incertae Sedis	Thermoanaerobacterium	hypothetical protein						
2640420..2641860	-	ADL70068.1	CP002171.1	Thermoanaerobacterium thermosaccharolyticum DSM 571	Bacteria	Firmicutes	Clostridia	Thermoanaerobacterales	Thermoanaerobacterales Family III. Incertae Sedis	Thermoanaerobacterium	S-layer domain protein						
2642658..2642820	-	ADL70069.1	CP002171.1	Thermoanaerobacterium thermosaccharolyticum DSM 571	Bacteria	Firmicutes	Clostridia	Thermoanaerobacterales	Thermoanaerobacterales Family III. Incertae Sedis	Thermoanaerobacterium	hypothetical protein						
2642908..2643124	-	ADL70070.1	CP002171.1	Thermoanaerobacterium thermosaccharolyticum DSM 571	Bacteria	Firmicutes	Clostridia	Thermoanaerobacterales	Thermoanaerobacterales Family III. Incertae Sedis	Thermoanaerobacterium	conserved hypothetical protein						
2643518..2647416	array		CP002171.1	Thermoanaerobacterium thermosaccharolyticum DSM 571	Bacteria	Firmicutes	Clostridia	Thermoanaerobacterales	Thermoanaerobacterales Family III. Incertae Sedis	Thermoanaerobacterium		CRISPR array			59	36	30
2647584..2649951	-	ADL70071.1	CP002171.1	Thermoanaerobacterium thermosaccharolyticum DSM 571	Bacteria	Firmicutes	Clostridia	Thermoanaerobacterales	Thermoanaerobacterales Family III. Incertae Sedis	Thermoanaerobacterium	metal dependent phosphohydrolase	cas3HD,cas3	cd09641,COG1203	CAS-I			
2649925..2650639	-	ADL70072.1	CP002171.1	Thermoanaerobacterium thermosaccharolyticum DSM 571	Bacteria	Firmicutes	Clostridia	Thermoanaerobacterales	Thermoanaerobacterales Family III. Incertae Sedis	Thermoanaerobacterium	CRISPR-associated protein Cas5, Hmari subtype	cas5	cls001430	CAS-I-B			
2650714..2651626	-	ADL70073.1	CP002171.1	Thermoanaerobacterium thermosaccharolyticum DSM 571	Bacteria	Firmicutes	Clostridia	Thermoanaerobacterales	Thermoanaerobacterales Family III. Incertae Sedis	Thermoanaerobacterium	CRISPR-associated protein, Csh2 family	cas7	cd09690	CAS-I-B			
2651622..2653383	-	ADL70074.1	CP002171.1	Thermoanaerobacterium thermosaccharolyticum DSM 571	Bacteria	Firmicutes	Clostridia	Thermoanaerobacterales	Thermoanaerobacterales Family III. Incertae Sedis	Thermoanaerobacterium	CRISPR-associated protein, TM1802 family	cas8b1	cd09730	CAS-I-B			
2653398..2654139	-	ADL70075.1	CP002171.1	Thermoanaerobacterium thermosaccharolyticum DSM 571	Bacteria	Firmicutes	Clostridia	Thermoanaerobacterales	Thermoanaerobacterales Family III. Incertae Sedis	Thermoanaerobacterium	CRISPR-associated protein Cas6	cas6	COG1583	CAS-I,CAS-III,CAS-IV			

89 partial CAS-I | OXT08473.1

55..2354	array		NKHD01000014.1	Thermoanaerobacterium thermosaccharolyticum	Bacteria	Firmicutes	Clostridia	Thermoanaerobacterales	Thermoanaerobacterales Family III. Incertae Sedis	Thermoanaerobacterium		CRISPR array			35	36	30
2871..3369	+	OXT08471.1	NKHD01000014.1	Thermoanaerobacterium thermosaccharolyticum	Bacteria	Firmicutes	Clostridia	Thermoanaerobacterales	Thermoanaerobacterales Family III. Incertae Sedis	Thermoanaerobacterium	CRISPR-associated protein Cas4	cas4	pfam01930	CAS-I			
3365..4358	+	OXT08472.1	NKHD01000014.1	Thermoanaerobacterium thermosaccharolyticum	Bacteria	Firmicutes	Clostridia	Thermoanaerobacterales	Thermoanaerobacterales Family III. Incertae Sedis	Thermoanaerobacterium	subtype I-B CRISPR-associated endonuclease Cas1	cas1	cd09722	CAS-I-B			
4370..4634	+	OXT08473.1	NKHD01000014.1	Thermoanaerobacterium thermosaccharolyticum	Bacteria	Firmicutes	Clostridia	Thermoanaerobacterales	Thermoanaerobacterales Family III. Incertae Sedis	Thermoanaerobacterium	CRISPR-associated endonuclease Cas2	cas2	cd09725	CAS-I,CAS-II,CAS-III,CAS-V			
4792..5683	-	OXT08474.1	NKHD01000014.1	Thermoanaerobacterium thermosaccharolyticum	Bacteria	Firmicutes	Clostridia	Thermoanaerobacterales	Thermoanaerobacterales Family III. Incertae Sedis	Thermoanaerobacterium	glycine/betaine ABC transporter substrate-binding protein						
5697..6336	-	OXT08475.1	NKHD01000014.1	Thermoanaerobacterium thermosaccharolyticum	Bacteria	Firmicutes	Clostridia	Thermoanaerobacterales	Thermoanaerobacterales Family III. Incertae Sedis	Thermoanaerobacterium	glycine/betaine ABC transporter						
6348..7458	-	OXT08476.1	NKHD01000014.1	Thermoanaerobacterium thermosaccharolyticum	Bacteria	Firmicutes	Clostridia	Thermoanaerobacterales	Thermoanaerobacterales Family III. Incertae Sedis	Thermoanaerobacterium	proline/glycine betaine ABC transporter ATP-binding protein	Cas_RecF	cluster17				
7578..8199	-	OXT08477.1	NKHD01000014.1	Thermoanaerobacterium thermosaccharolyticum	Bacteria	Firmicutes	Clostridia	Thermoanaerobacterales	Thermoanaerobacterales Family III. Incertae Sedis	Thermoanaerobacterium	GntR family transcriptional regulator	casR	cls001593	CAS-I			
8405..9971	+	OXT08478.1	NKHD01000014.1	Thermoanaerobacterium thermosaccharolyticum	Bacteria	Firmicutes	Clostridia	Thermoanaerobacterales	Thermoanaerobacterales Family III. Incertae Sedis	Thermoanaerobacterium	ClC family H(+)/Cl(-) exchange transporter						
10104..10548	+	OXT08479.1	NKHD01000014.1	Thermoanaerobacterium thermosaccharolyticum	Bacteria	Firmicutes	Clostridia	Thermoanaerobacterales	Thermoanaerobacterales Family III. Incertae Sedis	Thermoanaerobacterium	transcriptional regulator	casR	cls001593	CAS-I			

90 partial CAS-I | ORX22326.1

18359..18842	-	ORX22324.1	MSQD01000021.1	Thermoanaerobacterium sp. PSU-2	Bacteria	Firmicutes	Clostridia	Thermoanaerobacterales	Thermoanaerobacterales Family III. Incertae Sedis	Thermoanaerobacterium	transcriptional regulator	casR	cls001593	CAS-I			
18978..20547	-	ORX22325.1	MSQD01000021.1	Thermoanaerobacterium sp. PSU-2	Bacteria	Firmicutes	Clostridia	Thermoanaerobacterales	Thermoanaerobacterales Family III. Incertae Sedis	Thermoanaerobacterium	ClC family H(+)/Cl(-) exchange transporter						
20718..20982	-	ORX22326.1	MSQD01000021.1	Thermoanaerobacterium sp. PSU-2	Bacteria	Firmicutes	Clostridia	Thermoanaerobacterales	Thermoanaerobacterales Family III. Incertae Sedis	Thermoanaerobacterium	CRISPR-associated endonuclease Cas2	cas2	cd09725	CAS-I,CAS-II,CAS-III,CAS-V			
20994..21987	-	ORX22327.1	MSQD01000021.1	Thermoanaerobacterium sp. PSU-2	Bacteria	Firmicutes	Clostridia	Thermoanaerobacterales	Thermoanaerobacterales Family III. Incertae Sedis	Thermoanaerobacterium	subtype I-B CRISPR-associated endonuclease Cas1	cas1	cd09722	CAS-I-B			
21983..22481	-	ORX22328.1	MSQD01000021.1	Thermoanaerobacterium sp. PSU-2	Bacteria	Firmicutes	Clostridia	Thermoanaerobacterales	Thermoanaerobacterales Family III. Incertae Sedis	Thermoanaerobacterium	CRISPR-associated protein Cas4	cas4	pfam01930	CAS-I			
23542..24637	array		MSQD01000021.1	Thermoanaerobacterium sp. PSU-2	Bacteria	Firmicutes	Clostridia	Thermoanaerobacterales	Thermoanaerobacterales Family III. Incertae Sedis	Thermoanaerobacterium		CRISPR array			17	36	30

91 CAS-I-B | AEF18275.1																	
2384025..2384508	-	AEF18273.1	CP002739.1	Thermoanaerobacterium xylanolyticum LX-11	Bacteria	Firmicutes	Clostridia	Thermoanaerobacterales	Thermoanaerobacterales Family III. Incertae Sedis	Thermoanaerobacterium	transcriptional regulator, MarR family	casR	cls001593	CAS-I			
2384643..2386212	-	AEF18274.1	CP002739.1	Thermoanaerobacterium xylanolyticum LX-11	Bacteria	Firmicutes	Clostridia	Thermoanaerobacterales	Thermoanaerobacterales Family III. Incertae Sedis	Thermoanaerobacterium	Cl- channel voltage-gated family protein						
2386380..2386644	-	AEF18275.1	CP002739.1	Thermoanaerobacterium xylanolyticum LX-11	Bacteria	Firmicutes	Clostridia	Thermoanaerobacterales	Thermoanaerobacterales Family III. Incertae Sedis	Thermoanaerobacterium	CRISPR-associated protein Cas2	cas2	cd09725	CAS-I,CAS-II,CAS-III,CAS-V			
2386656..2387649	-	AEF18276.1	CP002739.1	Thermoanaerobacterium xylanolyticum LX-11	Bacteria	Firmicutes	Clostridia	Thermoanaerobacterales	Thermoanaerobacterales Family III. Incertae Sedis	Thermoanaerobacterium	CRISPR-associated protein Cas1	cas1	cd09722	CAS-I-B			
2387645..2388149	-	AEF18277.1	CP002739.1	Thermoanaerobacterium xylanolyticum LX-11	Bacteria	Firmicutes	Clostridia	Thermoanaerobacterales	Thermoanaerobacterales Family III. Incertae Sedis	Thermoanaerobacterium	CRISPR-associated protein Cas4	cas4	pfam01930	CAS-I			
2388185..2388491	-	AEF18278.1	CP002739.1	Thermoanaerobacterium xylanolyticum LX-11	Bacteria	Firmicutes	Clostridia	Thermoanaerobacterales	Thermoanaerobacterales Family III. Incertae Sedis	Thermoanaerobacterium	hypothetical protein						
2388534..2394161	array		CP002739.1	Thermoanaerobacterium xylanolyticum LX-11	Bacteria	Firmicutes	Clostridia	Thermoanaerobacterales	Thermoanaerobacterales Family III. Incertae Sedis	Thermoanaerobacterium		CRISPR array			85	36	30
2394324..2396688	-	AEF18279.1	CP002739.1	Thermoanaerobacterium xylanolyticum LX-11	Bacteria	Firmicutes	Clostridia	Thermoanaerobacterales	Thermoanaerobacterales Family III. Incertae Sedis	Thermoanaerobacterium	CRISPR-associated HD domain protein	cas3HD,cas3	cd09641,COG1203	CAS-I			
2396665..2397379	-	AEF18280.1	CP002739.1	Thermoanaerobacterium xylanolyticum LX-11	Bacteria	Firmicutes	Clostridia	Thermoanaerobacterales	Thermoanaerobacterales Family III. Incertae Sedis	Thermoanaerobacterium	CRISPR-associated protein Cas5, Hmari subtype	cas5	cls001430	CAS-I-B			
2397443..2398355	-	AEF18281.1	CP002739.1	Thermoanaerobacterium xylanolyticum LX-11	Bacteria	Firmicutes	Clostridia	Thermoanaerobacterales	Thermoanaerobacterales Family III. Incertae Sedis	Thermoanaerobacterium	CRISPR-associated protein, Csh2 family	cas7	cd09690	CAS-I-B			
2398354..2400112	-	AEF18282.1	CP002739.1	Thermoanaerobacterium xylanolyticum LX-11	Bacteria	Firmicutes	Clostridia	Thermoanaerobacterales	Thermoanaerobacterales Family III. Incertae Sedis	Thermoanaerobacterium	CRISPR-associated protein, TM1802 family	cas8b1	cd09730	CAS-I-B			
2400126..2400867	-	AEF18283.1	CP002739.1	Thermoanaerobacterium xylanolyticum LX-11	Bacteria	Firmicutes	Clostridia	Thermoanaerobacterales	Thermoanaerobacterales Family III. Incertae Sedis	Thermoanaerobacterium	CRISPR-associated protein Cas6	cas6	COG1583	CAS-I,CAS-III,CAS-IV			
92 CAS-I-B | ETO39374.1																	
10348..10963	-	ETO39372.1	AYSN01000014.1	Thermoanaerobacterium aotearoense SCUT27	Bacteria	Firmicutes	Clostridia	Thermoanaerobacterales	Thermoanaerobacterales Family III. Incertae Sedis	Thermoanaerobacterium	MarR family transcriptional regulator	casR	cls001593	CAS-I			
10965..11229	-	ETO39373.1	AYSN01000014.1	Thermoanaerobacterium aotearoense SCUT27	Bacteria	Firmicutes	Clostridia	Thermoanaerobacterales	Thermoanaerobacterales Family III. Incertae Sedis	Thermoanaerobacterium	TrkA-C domain-containing protein						
11453..11717	-	ETO39374.1	AYSN01000014.1	Thermoanaerobacterium aotearoense SCUT27	Bacteria	Firmicutes	Clostridia	Thermoanaerobacterales	Thermoanaerobacterales Family III. Incertae Sedis	Thermoanaerobacterium	CRISPR-associated protein Cas2	cas2	cd09725	CAS-I,CAS-II,CAS-III,CAS-V			
11726..12719	-	ETO39375.1	AYSN01000014.1	Thermoanaerobacterium aotearoense SCUT27	Bacteria	Firmicutes	Clostridia	Thermoanaerobacterales	Thermoanaerobacterales Family III. Incertae Sedis	Thermoanaerobacterium	CRISPR-associated protein Cas1	cas1	cd09722	CAS-I-B			
12715..13213	-	ETO39376.1	AYSN01000014.1	Thermoanaerobacterium aotearoense SCUT27	Bacteria	Firmicutes	Clostridia	Thermoanaerobacterales	Thermoanaerobacterales Family III. Incertae Sedis	Thermoanaerobacterium	CRISPR-associated protein Cas4	cas4	pfam01930	CAS-I			
13574..14321	-	ETO39377.1	AYSN01000014.1	Thermoanaerobacterium aotearoense SCUT27	Bacteria	Firmicutes	Clostridia	Thermoanaerobacterales	Thermoanaerobacterales Family III. Incertae Sedis	Thermoanaerobacterium	copper amine oxidase						
14334..18020	array		AYSN01000014.1	Thermoanaerobacterium aotearoense SCUT27	Bacteria	Firmicutes	Clostridia	Thermoanaerobacterales	Thermoanaerobacterales Family III. Incertae Sedis	Thermoanaerobacterium		CRISPR array			56	36	30
18183..20547	-	ETO39378.1	AYSN01000014.1	Thermoanaerobacterium aotearoense SCUT27	Bacteria	Firmicutes	Clostridia	Thermoanaerobacterales	Thermoanaerobacterales Family III. Incertae Sedis	Thermoanaerobacterium	CRISPR-associated HD domain-containing protein	cas3HD,cas3	cd09641,COG1203	CAS-I			
20524..21238	-	ETO39379.1	AYSN01000014.1	Thermoanaerobacterium aotearoense SCUT27	Bacteria	Firmicutes	Clostridia	Thermoanaerobacterales	Thermoanaerobacterales Family III. Incertae Sedis	Thermoanaerobacterium	CRISPR-associated protein Cas5	cas5	cls001430	CAS-I-B			
21301..22213	-	ETO39380.1	AYSN01000014.1	Thermoanaerobacterium aotearoense SCUT27	Bacteria	Firmicutes	Clostridia	Thermoanaerobacterales	Thermoanaerobacterales Family III. Incertae Sedis	Thermoanaerobacterium	Csh2 family CRISPR-associated protein	cas7	cd09690	CAS-I-B			
22212..23970	-	ETO39381.1	AYSN01000014.1	Thermoanaerobacterium aotearoense SCUT27	Bacteria	Firmicutes	Clostridia	Thermoanaerobacterales	Thermoanaerobacterales Family III. Incertae Sedis	Thermoanaerobacterium	CRISPR-associated protein	cas8b1	cd09730	CAS-I-B			
23984..24725	-	ETO39382.1	AYSN01000014.1	Thermoanaerobacterium aotearoense SCUT27	Bacteria	Firmicutes	Clostridia	Thermoanaerobacterales	Thermoanaerobacterales Family III. Incertae Sedis	Thermoanaerobacterium	CRISPR-associated protein Cas6	cas6	COG1583	CAS-I,CAS-III,CAS-IV			
93 partial CAS-I-B | KHO61714.1																	
562907..563663	+	KHO61704.1	JOOI01000002.1	Thermoanaerobacter sp. YS13	Bacteria	Firmicutes	Clostridia	Thermoanaerobacterales	Thermoanaerobacteraceae	Thermoanaerobacter	CRISPR-associated protein Cas6	cas6	cd09652	CAS-I,CAS-III			
563682..565794	+	KHO61705.1	JOOI01000002.1	Thermoanaerobacter sp. YS13	Bacteria	Firmicutes	Clostridia	Thermoanaerobacterales	Thermoanaerobacteraceae	Thermoanaerobacter	CRISPR-associated protein Cas8b/Csh1	cas8b1	pfam09484	CAS-I-B			
566897..567659	+	KHO61706.1	JOOI01000002.1	Thermoanaerobacter sp. YS13	Bacteria	Firmicutes	Clostridia	Thermoanaerobacterales	Thermoanaerobacteraceae	Thermoanaerobacter	CRISPR-associated protein Cas5	cas5	cd09692	CAS-I-B			
570400..571224	array		JOOI01000002.1	Thermoanaerobacter sp. YS13	Bacteria	Firmicutes	Clostridia	Thermoanaerobacterales	Thermoanaerobacteraceae	Thermoanaerobacter		CRISPR array			13	36	30
571355..572126	-	KHO61707.1	JOOI01000002.1	Thermoanaerobacter sp. YS13	Bacteria	Firmicutes	Clostridia	Thermoanaerobacterales	Thermoanaerobacteraceae	Thermoanaerobacter	ATPase AAA						
572118..573606	-	KHO61708.1	JOOI01000002.1	Thermoanaerobacter sp. YS13	Bacteria	Firmicutes	Clostridia	Thermoanaerobacterales	Thermoanaerobacteraceae	Thermoanaerobacter	integrase						
573986..574568	+	KHO61709.1	JOOI01000002.1	Thermoanaerobacter sp. YS13	Bacteria	Firmicutes	Clostridia	Thermoanaerobacterales	Thermoanaerobacteraceae	Thermoanaerobacter	hypothetical protein						
574704..576030	+	KHO61710.1	JOOI01000002.1	Thermoanaerobacter sp. YS13	Bacteria	Firmicutes	Clostridia	Thermoanaerobacterales	Thermoanaerobacteraceae	Thermoanaerobacter	integrase						
576047..576848	+	KHO61711.1	JOOI01000002.1	Thermoanaerobacter sp. YS13	Bacteria	Firmicutes	Clostridia	Thermoanaerobacterales	Thermoanaerobacteraceae	Thermoanaerobacter	ATPase AAA						
577077..577577	array		JOOI01000002.1	Thermoanaerobacter sp. YS13	Bacteria	Firmicutes	Clostridia	Thermoanaerobacterales	Thermoanaerobacteraceae	Thermoanaerobacter		CRISPR array			8	37	30
578469..578715	+	KHO61712.1	JOOI01000002.1	Thermoanaerobacter sp. YS13	Bacteria	Firmicutes	Clostridia	Thermoanaerobacterales	Thermoanaerobacteraceae	Thermoanaerobacter	hypothetical protein						
578820..579318	+	KHO61713.1	JOOI01000002.1	Thermoanaerobacter sp. YS13	Bacteria	Firmicutes	Clostridia	Thermoanaerobacterales	Thermoanaerobacteraceae	Thermoanaerobacter	CRISPR-associated protein Cas4	cas4	pfam01930	CAS-I			
580321..580585	+	KHO61714.1	JOOI01000002.1	Thermoanaerobacter sp. YS13	Bacteria	Firmicutes	Clostridia	Thermoanaerobacterales	Thermoanaerobacteraceae	Thermoanaerobacter	CRISPR-associated protein Cas2	cas2	cd09725	CAS-I,CAS-II,CAS-III,CAS-V			
94 partial CAS-I-B | SDG65044.1																	
3589..3853	-	SDG65044.1	FNBS01000106.1	Thermoanaerobacter thermohydrosulfuricus	Bacteria	Firmicutes	Clostridia	Thermoanaerobacterales	Thermoanaerobacteraceae	Thermoanaerobacter	CRISPR-associated protein, Cas2 family	cas2	cd09725	CAS-I,CAS-II,CAS-III,CAS-V			
3869..4862	-	SDG65079.1	FNBS01000106.1	Thermoanaerobacter thermohydrosulfuricus	Bacteria	Firmicutes	Clostridia	Thermoanaerobacterales	Thermoanaerobacteraceae	Thermoanaerobacter	CRISPR-associated protein, Cas1 family	cas1	cd09722	CAS-I-B			
4858..5356	-	SDG65117.1	FNBS01000106.1	Thermoanaerobacter thermohydrosulfuricus	Bacteria	Firmicutes	Clostridia	Thermoanaerobacterales	Thermoanaerobacteraceae	Thermoanaerobacter	CRISPR-associated exonuclease, Cas4 family	cas4	pfam01930	CAS-I			
95 partial CAS-I-B | SFE77437.1																	
1222..1720	+	SFE77389.1	FONI01000094.1	Thermoanaerobacter thermohydrosulfuricus	Bacteria	Firmicutes	Clostridia	Thermoanaerobacterales	Thermoanaerobacteraceae	Thermoanaerobacter	CRISPR-associated exonuclease, Cas4 family	cas4	pfam01930	CAS-I			
1716..2709	+	SFE77410.1	FONI01000094.1	Thermoanaerobacter thermohydrosulfuricus	Bacteria	Firmicutes	Clostridia	Thermoanaerobacterales	Thermoanaerobacteraceae	Thermoanaerobacter	CRISPR-associated protein, Cas1 family	cas1	cd09722	CAS-I-B			
2725..2989	+	SFE77437.1	FONI01000094.1	Thermoanaerobacter thermohydrosulfuricus	Bacteria	Firmicutes	Clostridia	Thermoanaerobacterales	Thermoanaerobacteraceae	Thermoanaerobacter	CRISPR-associated protein, Cas2 family	cas2	cd09725	CAS-I,CAS-II,CAS-III,CAS-V			
96 partial CAS-I-B | EEU62463.1																	
1299..1797	+	EEU62461.1	ACXY01000012.1	Thermoanaerobacter ethanolicus CCSD1	Bacteria	Firmicutes	Clostridia	Thermoanaerobacterales	Thermoanaerobacteraceae	Thermoanaerobacter	CRISPR-associated protein Cas4	cas4	pfam01930	CAS-I			
1793..2786	+	EEU62462.1	ACXY01000012.1	Thermoanaerobacter ethanolicus CCSD1	Bacteria	Firmicutes	Clostridia	Thermoanaerobacterales	Thermoanaerobacteraceae	Thermoanaerobacter	CRISPR-associated protein Cas1	cas1	cd09722	CAS-I-B			
2802..3066	+	EEU62463.1	ACXY01000012.1	Thermoanaerobacter ethanolicus CCSD1	Bacteria	Firmicutes	Clostridia	Thermoanaerobacterales	Thermoanaerobacteraceae	Thermoanaerobacter	CRISPR-associated protein Cas2	cas2	cd09725	CAS-I,CAS-II,CAS-III,CAS-V			
97 CAS-I-B | ADH61854.1																	
2204514..2205930	+	ADH61852.1	CP002032.1	Thermoanaerobacter mathranii subsp. mathranii str. A3	Bacteria	Firmicutes	Clostridia	Thermoanaerobacterales	Thermoanaerobacteraceae	Thermoanaerobacter	amino acid permease-associated region	C3a194	cluster194				
2205966..2206746	-	ADH61853.1	CP002032.1	Thermoanaerobacter mathranii subsp. mathranii str. A3	Bacteria	Firmicutes	Clostridia	Thermoanaerobacterales	Thermoanaerobacteraceae	Thermoanaerobacter	beta-lactamase domain protein						
2207068..2207332	-	ADH61854.1	CP002032.1	Thermoanaerobacter mathranii subsp. mathranii str. A3	Bacteria	Firmicutes	Clostridia	Thermoanaerobacterales	Thermoanaerobacteraceae	Thermoanaerobacter	CRISPR-associated protein Cas2	cas2	cd09725	CAS-I,CAS-II,CAS-III,CAS-V			
2207348..2208341	-	ADH61855.1	CP002032.1	Thermoanaerobacter mathranii subsp. mathranii str. A3	Bacteria	Firmicutes	Clostridia	Thermoanaerobacterales	Thermoanaerobacteraceae	Thermoanaerobacter	CRISPR-associated protein Cas1	cas1	cd09722	CAS-I-B			
2208337..2208835	-	ADH61856.1	CP002032.1	Thermoanaerobacter mathranii subsp. mathranii str. A3	Bacteria	Firmicutes	Clostridia	Thermoanaerobacterales	Thermoanaerobacteraceae	Thermoanaerobacter	CRISPR-associated protein Cas4	cas4	pfam01930	CAS-I			
2209618..2210530	+	ADH61857.1	CP002032.1	Thermoanaerobacter mathranii subsp. mathranii str. A3	Bacteria	Firmicutes	Clostridia	Thermoanaerobacterales	Thermoanaerobacteraceae	Thermoanaerobacter	copper amine oxidase domain protein						
2210634..2211180	+	ADH61858.1	CP002032.1	Thermoanaerobacter mathranii subsp. mathranii str. A3	Bacteria	Firmicutes	Clostridia	Thermoanaerobacterales	Thermoanaerobacteraceae	Thermoanaerobacter	hypothetical protein						
2211671..2212349	+	ADH61859.1	CP002032.1	Thermoanaerobacter mathranii subsp. mathranii str. A3	Bacteria	Firmicutes	Clostridia	Thermoanaerobacterales	Thermoanaerobacteraceae	Thermoanaerobacter	copper amine oxidase domain protein						
2212413..2212968	-	ADH61860.1	CP002032.1	Thermoanaerobacter mathranii subsp. mathranii str. A3	Bacteria	Firmicutes	Clostridia	Thermoanaerobacterales	Thermoanaerobacteraceae	Thermoanaerobacter	Rare lipoprotein A						
2213051..2213177	-	ADH61861.1	CP002032.1	Thermoanaerobacter mathranii subsp. mathranii str. A3	Bacteria	Firmicutes	Clostridia	Thermoanaerobacterales	Thermoanaerobacteraceae	Thermoanaerobacter	hypothetical protein						
2216141..2217710	array		CP002032.1	Thermoanaerobacter mathranii subsp. mathranii str. A3	Bacteria	Firmicutes	Clostridia	Thermoanaerobacterales	Thermoanaerobacteraceae	Thermoanaerobacter		CRISPR array			24	36	30
2217892..2220247	-	ADH61862.1	CP002032.1	Thermoanaerobacter mathranii subsp. mathranii str. A3	Bacteria	Firmicutes	Clostridia	Thermoanaerobacterales	Thermoanaerobacteraceae	Thermoanaerobacter	CRISPR-associated helicase Cas3	cas3	COG1203	CAS-I			
2220230..2220944	-	ADH61863.1	CP002032.1	Thermoanaerobacter mathranii subsp. mathranii str. A3	Bacteria	Firmicutes	Clostridia	Thermoanaerobacterales	Thermoanaerobacteraceae	Thermoanaerobacter	CRISPR-associated protein Cas5, Hmari subtype	cas5	cls001430	CAS-I-B			
2220956..2221871	-	ADH61864.1	CP002032.1	Thermoanaerobacter mathranii subsp. mathranii str. A3	Bacteria	Firmicutes	Clostridia	Thermoanaerobacterales	Thermoanaerobacteraceae	Thermoanaerobacter	CRISPR-associated protein, Csh2 family	cas7	cd09690	CAS-I-B			
2221854..2223633	-	ADH61865.1	CP002032.1	Thermoanaerobacter mathranii subsp. mathranii str. A3	Bacteria	Firmicutes	Clostridia	Thermoanaerobacterales	Thermoanaerobacteraceae	Thermoanaerobacter	CRISPR-associated protein, TM1802 family	cas8b1	cd09730	CAS-I-B			
2223651..2224395	-	ADH61866.1	CP002032.1	Thermoanaerobacter mathranii subsp. mathranii str. A3	Bacteria	Firmicutes	Clostridia	Thermoanaerobacterales	Thermoanaerobacteraceae	Thermoanaerobacter	CRISPR-associated protein Cas6	cas6	cd09652	CAS-I,CAS-III			
2224882..2225191	-	ADH61867.1	CP002032.1	Thermoanaerobacter mathranii subsp. mathranii str. A3	Bacteria	Firmicutes	Clostridia	Thermoanaerobacterales	Thermoanaerobacteraceae	Thermoanaerobacter	conserved hypothetical protein	cas2	mkCas0179	CAS-I,CAS-II,CAS-III			
98 partial CAS-I-B | ERM93083.1																	
151435..151699	-	ERM93083.1	AXDC01000003.1	Caldanaerobacter subterraneus subsp. yonseiensis KB-1	Bacteria	Firmicutes	Clostridia	Thermoanaerobacterales	Thermoanaerobacteraceae	Caldanaerobacter	CRISPR-associated protein Cas2	cas2	cd09725	CAS-I,CAS-II,CAS-III,CAS-V			
151712..152705	-	ERM93084.1	AXDC01000003.1	Caldanaerobacter subterraneus subsp. yonseiensis KB-1	Bacteria	Firmicutes	Clostridia	Thermoanaerobacterales	Thermoanaerobacteraceae	Caldanaerobacter	CRISPR-associated protein Cas1	cas1	cd09722	CAS-I-B			
152701..153199	-	ERM93085.1	AXDC01000003.1	Caldanaerobacter subterraneus subsp. yonseiensis KB-1	Bacteria	Firmicutes	Clostridia	Thermoanaerobacterales	Thermoanaerobacteraceae	Caldanaerobacter	CRISPR-associated protein Cas4	cas4	pfam01930	CAS-I			
99 CAS-I-B| SHF39031.1																	
29494..30232	+	SHF38647.1	FQVH01000020.1	Caldanaerobius fijiensis DSM 17918	Bacteria	Firmicutes	Clostridia	Thermoanaerobacterales	Thermoanaerobacteraceae	Caldanaerobius	CRISPR-associated endoribonuclease Cas6	cas6	COG1583	CAS-I,CAS-III,CAS-IV			
30407..32402	+	SHF38686.1	FQVH01000020.1	Caldanaerobius fijiensis DSM 17918	Bacteria	Firmicutes	Clostridia	Thermoanaerobacterales	Thermoanaerobacteraceae	Caldanaerobius	CRISPR-associated protein Csh1	cas8b1	pfam09484	CAS-I-B			
32388..33324	+	SHF38718.1	FQVH01000020.1	Caldanaerobius fijiensis DSM 17918	Bacteria	Firmicutes	Clostridia	Thermoanaerobacterales	Thermoanaerobacteraceae	Caldanaerobius	CRISPR-associated protein, Csh2 family	cas7b	pfam05107	CAS-I-B,CAS-I-C			
33344..34124	+	SHF38753.1	FQVH01000020.1	Caldanaerobius fijiensis DSM 17918	Bacteria	Firmicutes	Clostridia	Thermoanaerobacterales	Thermoanaerobacteraceae	Caldanaerobius	CRISPR-associated protein Cas5h	cas5	cd09692	CAS-I-B			
34123..36550	+	SHF38785.1	FQVH01000020.1	Caldanaerobius fijiensis DSM 17918	Bacteria	Firmicutes	Clostridia	Thermoanaerobacterales	Thermoanaerobacteraceae	Caldanaerobius	CRISPR-associated helicase, Cas3 family	cas3HD,cas3	cd09641,COG1203	CAS-I			
36583..37153	+	SHF38819.1	FQVH01000020.1	Caldanaerobius fijiensis DSM 17918	Bacteria	Firmicutes	Clostridia	Thermoanaerobacterales	Thermoanaerobacteraceae	Caldanaerobius	CRISPR-associated exonuclease, Cas4 family	cas4	pfam01930	CAS-I			
37170..38163	+	SHF38850.1	FQVH01000020.1	Caldanaerobius fijiensis DSM 17918	Bacteria	Firmicutes	Clostridia	Thermoanaerobacterales	Thermoanaerobacteraceae	Caldanaerobius	CRISP-associated protein Cas1	cas1	cd09722	CAS-I-B			
38397..38781	+	SHF38877.1	FQVH01000020.1	Caldanaerobius fijiensis DSM 17918	Bacteria	Firmicutes	Clostridia	Thermoanaerobacterales	Thermoanaerobacteraceae	Caldanaerobius	transcriptional regulator, GntR family	casR*	cd09655				
38777..39671	+	SHF38905.1	FQVH01000020.1	Caldanaerobius fijiensis DSM 17918	Bacteria	Firmicutes	Clostridia	Thermoanaerobacterales	Thermoanaerobacteraceae	Caldanaerobius	ABC-2 type transport system ATP-binding protein	Cas_RecF	cluster17				
39651..40320	+	SHF38937.1	FQVH01000020.1	Caldanaerobius fijiensis DSM 17918	Bacteria	Firmicutes	Clostridia	Thermoanaerobacterales	Thermoanaerobacteraceae	Caldanaerobius	ABC-2 family transporter protein						
40378..40549	+	SHF38966.1	FQVH01000020.1	Caldanaerobius fijiensis DSM 17918	Bacteria	Firmicutes	Clostridia	Thermoanaerobacterales	Thermoanaerobacteraceae	Caldanaerobius	hypothetical protein						
41197..41356	-	SHF38999.1	FQVH01000020.1	Caldanaerobius fijiensis DSM 17918	Bacteria	Firmicutes	Clostridia	Thermoanaerobacterales	Thermoanaerobacteraceae	Caldanaerobius	Helix-turn-helix domain						
41846..42110	+	SHF39031.1	FQVH01000020.1	Caldanaerobius fijiensis DSM 17918	Bacteria	Firmicutes	Clostridia	Thermoanaerobacterales	Thermoanaerobacteraceae	Caldanaerobius	CRISPR-associated protein Cas2	cas2	cd09725	CAS-I,CAS-II,CAS-III,CAS-V			
100 CAS-I-B | GAQ24762.1																	
149241..149505	-	GAQ24762.1	DF977000.1	Tepidanaerobacter syntrophicus	Bacteria	Firmicutes	Clostridia	Thermoanaerobacterales	Thermoanaerobacteraceae	Tepidanaerobacter	CRISPR-associated protein Cas2	cas2	cd09725	CAS-I,CAS-II,CAS-III,CAS-V			
149714..155606	array		DF977000.1	Tepidanaerobacter syntrophicus	Bacteria	Firmicutes	Clostridia	Thermoanaerobacterales	Thermoanaerobacteraceae	Tepidanaerobacter		CRISPR array			89	36	30
155801..156794	-	GAQ24763.1	DF977000.1	Tepidanaerobacter syntrophicus	Bacteria	Firmicutes	Clostridia	Thermoanaerobacterales	Thermoanaerobacteraceae	Tepidanaerobacter	CRISP-associated protein Cas1	cas1	cd09722	CAS-I-B			
156806..157313	-	GAQ24764.1	DF977000.1	Tepidanaerobacter syntrophicus	Bacteria	Firmicutes	Clostridia	Thermoanaerobacterales	Thermoanaerobacteraceae	Tepidanaerobacter	CRISPR-associated exonuclease Cas4	cas4	pfam01930	CAS-I			
157389..159630	-	GAQ24765.1	DF977000.1	Tepidanaerobacter syntrophicus	Bacteria	Firmicutes	Clostridia	Thermoanaerobacterales	Thermoanaerobacteraceae	Tepidanaerobacter	CRISPR-associated endonuclease/helicase Cas3	cas3HD,cas3	cd09641,cd09639	CAS-I			
159633..160377	-	GAQ24766.1	DF977000.1	Tepidanaerobacter syntrophicus	Bacteria	Firmicutes	Clostridia	Thermoanaerobacterales	Thermoanaerobacteraceae	Tepidanaerobacter	CRISPR-associated protein Cas5t	cas5	cd09658	CAS-I-B			
160411..161287	-	GAQ24767.1	DF977000.1	Tepidanaerobacter syntrophicus	Bacteria	Firmicutes	Clostridia	Thermoanaerobacterales	Thermoanaerobacteraceae	Tepidanaerobacter	CRISPR-associated protein Cst2	cas7	cd09687	CAS-I-B			
161273..162980	-	GAQ24768.1	DF977000.1	Tepidanaerobacter syntrophicus	Bacteria	Firmicutes	Clostridia	Thermoanaerobacterales	Thermoanaerobacteraceae	Tepidanaerobacter	hypothetical protein	cas8b1	mkCas0195	CAS-I-B			
162989..163739	-	GAQ24769.1	DF977000.1	Tepidanaerobacter syntrophicus	Bacteria	Firmicutes	Clostridia	Thermoanaerobacterales	Thermoanaerobacteraceae	Tepidanaerobacter	CRISPR-associated endoribonuclease Cas6	cas6	COG1583	CAS-I,CAS-III,CAS-IV			
101 CAS-I-B | CCP24806.1																	
113740..114481	+	CDI40287.1	HF563609.2	Tepidanaerobacter acetatoxydans Re1	Bacteria	Firmicutes	Clostridia	Thermoanaerobacterales	Thermoanaerobacteraceae	Tepidanaerobacter	CRISPR-associated protein Cas6	cas6	COG1583	CAS-I,CAS-III,CAS-IV			
114747..116847	+	CDI40288.1	HF563609.2	Tepidanaerobacter acetatoxydans Re1	Bacteria	Firmicutes	Clostridia	Thermoanaerobacterales	Thermoanaerobacteraceae	Tepidanaerobacter	conserved protein of unknown function	cas8b1	pfam09484	CAS-I-B			
116907..117960	+	CCP24802.1	HF563609.2	Tepidanaerobacter acetatoxydans Re1	Bacteria	Firmicutes	Clostridia	Thermoanaerobacterales	Thermoanaerobacteraceae	Tepidanaerobacter	conserved protein of unknown function	cas7b	pfam05107	CAS-I-B,CAS-I-C			
117962..118727	+	CCP24803.1	HF563609.2	Tepidanaerobacter acetatoxydans Re1	Bacteria	Firmicutes	Clostridia	Thermoanaerobacterales	Thermoanaerobacteraceae	Tepidanaerobacter	CRISPR-associated protein Cas5	cas5	cd09692	CAS-I-B			
118780..121270	+	CDI40289.1	HF563609.2	Tepidanaerobacter acetatoxydans Re1	Bacteria	Firmicutes	Clostridia	Thermoanaerobacterales	Thermoanaerobacteraceae	Tepidanaerobacter	CRISPR-associated HD domain protein	cas3HD,cas3	cd09641,COG1203	CAS-I			
121829..122093	+	CCP24806.1	HF563609.2	Tepidanaerobacter acetatoxydans Re1	Bacteria	Firmicutes	Clostridia	Thermoanaerobacterales	Thermoanaerobacteraceae	Tepidanaerobacter	CRISPR-associated endoribonuclease Cas2	cas2	cd09725	CAS-I,CAS-II,CAS-III,CAS-V			
102 CAS-I-B | SFP76577.1																	
128303..129059	+	SFP76335.1	FOXR01000003.1	Caldicoprobacter faecalis	Bacteria	Firmicutes	Clostridia	Clostridiales	Caldicoprobacteraceae	Caldicoprobacter	CRISPR-associated endoribonuclease Cas6	cas6	COG1583	CAS-I,CAS-III,CAS-IV			
129295..131077	+	SFP76364.1	FOXR01000003.1	Caldicoprobacter faecalis	Bacteria	Firmicutes	Clostridia	Clostridiales	Caldicoprobacteraceae	Caldicoprobacter	CRISPR-associated protein, Csh1 family	cas8b1	cd09730	CAS-I-B			
131116..132025	+	SFP76399.1	FOXR01000003.1	Caldicoprobacter faecalis	Bacteria	Firmicutes	Clostridia	Clostridiales	Caldicoprobacteraceae	Caldicoprobacter	CRISPR-associated protein Csh2	cas7	cd09690	CAS-I-B			
132037..132754	+	SFP76437.1	FOXR01000003.1	Caldicoprobacter faecalis	Bacteria	Firmicutes	Clostridia	Clostridiales	Caldicoprobacteraceae	Caldicoprobacter	CRISPR-associated protein Cas5h	cas5	cls001430	CAS-I-B			
132728..135101	+	SFP76472.1	FOXR01000003.1	Caldicoprobacter faecalis	Bacteria	Firmicutes	Clostridia	Clostridiales	Caldicoprobacteraceae	Caldicoprobacter	CRISPR-associated helicase, Cas3 family	cas3HD,cas3	cd09641,COG1203	CAS-I			
135094..135589	+	SFP76505.1	FOXR01000003.1	Caldicoprobacter faecalis	Bacteria	Firmicutes	Clostridia	Clostridiales	Caldicoprobacteraceae	Caldicoprobacter	CRISPR-associated exonuclease Cas4	cas4	pfam01930	CAS-I			
135585..136578	+	SFP76543.1	FOXR01000003.1	Caldicoprobacter faecalis	Bacteria	Firmicutes	Clostridia	Clostridiales	Caldicoprobacteraceae	Caldicoprobacter	CRISP-associated protein Cas1	cas1	cd09722	CAS-I-B			
136620..136884	+	SFP76577.1	FOXR01000003.1	Caldicoprobacter faecalis	Bacteria	Firmicutes	Clostridia	Clostridiales	Caldicoprobacteraceae	Caldicoprobacter	CRISPR-associated protein Cas2	cas2	cd09725	CAS-I,CAS-II,CAS-III,CAS-V			
137187..138686	array		FOXR01000003.1	Caldicoprobacter faecalis	Bacteria	Firmicutes	Clostridia	Clostridiales	Caldicoprobacteraceae	Caldicoprobacter		CRISPR array			23	36	30
105 CAS-I-B | SHM89755.1																	
16503..18291	+	SHM89600.1	FRCR01000021.1	Caldanaerovirga acetigignens	Bacteria	Firmicutes	Clostridia	Thermoanaerobacterales	Thermoanaerobacterales Family III. Incertae Sedis	Caldanaerovirga	CRISPR-associated protein, Csh1 family	cas8b1	cd09730	CAS-I-B			
18318..19227	+	SHM89628.1	FRCR01000021.1	Caldanaerovirga acetigignens	Bacteria	Firmicutes	Clostridia	Thermoanaerobacterales	Thermoanaerobacterales Family III. Incertae Sedis	Caldanaerovirga	CRISPR-associated protein Csh2	cas7	cd09690	CAS-I-B			
19241..19958	+	SHM89651.1	FRCR01000021.1	Caldanaerovirga acetigignens	Bacteria	Firmicutes	Clostridia	Thermoanaerobacterales	Thermoanaerobacterales Family III. Incertae Sedis	Caldanaerovirga	CRISPR-associated protein Cas5h	cas5	cls001430	CAS-I-B			
19932..22281	+	SHM89677.1	FRCR01000021.1	Caldanaerovirga acetigignens	Bacteria	Firmicutes	Clostridia	Thermoanaerobacterales	Thermoanaerobacterales Family III. Incertae Sedis	Caldanaerovirga	CRISPR-associated helicase, Cas3 family	cas3HD,cas3	cd09641,COG1203	CAS-I			
22274..22772	+	SHM89702.1	FRCR01000021.1	Caldanaerovirga acetigignens	Bacteria	Firmicutes	Clostridia	Thermoanaerobacterales	Thermoanaerobacterales Family III. Incertae Sedis	Caldanaerovirga	CRISPR-associated exonuclease, Cas4 family	cas4	pfam01930	CAS-I			
22768..23761	+	SHM89729.1	FRCR01000021.1	Caldanaerovirga acetigignens	Bacteria	Firmicutes	Clostridia	Thermoanaerobacterales	Thermoanaerobacterales Family III. Incertae Sedis	Caldanaerovirga	CRISP-associated protein Cas1	cas1	cd09722	CAS-I-B			
23797..24061	+	SHM89755.1	FRCR01000021.1	Caldanaerovirga acetigignens	Bacteria	Firmicutes	Clostridia	Thermoanaerobacterales	Thermoanaerobacterales Family III. Incertae Sedis	Caldanaerovirga	CRISPR-associated protein Cas2	cas2	cd09725	CAS-I,CAS-II,CAS-III,CAS-V			
26756..27649	array		FRCR01000021.1	Caldanaerovirga acetigignens	Bacteria	Firmicutes	Clostridia	Thermoanaerobacterales	Thermoanaerobacterales Family III. Incertae Sedis	Caldanaerovirga		CRISPR array			14	36	30
110 partial CAS-I-B | PSR32273.1																	
6995..7934	+	PSR32272.1	PXYW01000046.1	Sulfobacillus benefaciens	Bacteria	Firmicutes	Clostridia	Clostridiales	Clostridiales Family XVII. Incertae Sedis	Sulfobacillus	subtype I-B CRISPR-associated endonuclease Cas1	cas1	cd09722	CAS-I-B			
7943..8207	+	PSR32273.1	PXYW01000046.1	Sulfobacillus benefaciens	Bacteria	Firmicutes	Clostridia	Clostridiales	Clostridiales Family XVII. Incertae Sedis	Sulfobacillus	CRISPR-associated endonuclease Cas2	cas2	cd09725	CAS-I,CAS-II,CAS-III,CAS-V			
8353..10773	array		PXYW01000046.1	Sulfobacillus benefaciens	Bacteria	Firmicutes	Clostridia	Clostridiales	Clostridiales Family XVII. Incertae Sedis	Sulfobacillus		CRISPR array			37	36	30
111 CAS-I | SDH44542.1																	
38745..39103	array		FNDE01000025.1	Aneurinibacillus thermoaerophilus	Bacteria	Firmicutes	Bacilli	Bacillales	Paenibacillaceae	Aneurinibacillus		CRISPR array			6	35	30
39320..39584	-	SDH44542.1	FNDE01000025.1	Aneurinibacillus thermoaerophilus	Bacteria	Firmicutes	Bacilli	Bacillales	Paenibacillaceae	Aneurinibacillus	CRISPR-associated protein, Cas2 family	cas2	cd09725	CAS-I,CAS-II,CAS-III,CAS-V			
39596..40589	-	SDH44558.1	FNDE01000025.1	Aneurinibacillus thermoaerophilus	Bacteria	Firmicutes	Bacilli	Bacillales	Paenibacillaceae	Aneurinibacillus	CRISP-associated protein Cas1	cas1	cd09722	CAS-I-B			
40603..41116	-	SDH44585.1	FNDE01000025.1	Aneurinibacillus thermoaerophilus	Bacteria	Firmicutes	Bacilli	Bacillales	Paenibacillaceae	Aneurinibacillus	CRISPR-associated exonuclease, Cas4 family	cas4	pfam01930	CAS-I			
41130..43467	-	SDH44606.1	FNDE01000025.1	Aneurinibacillus thermoaerophilus	Bacteria	Firmicutes	Bacilli	Bacillales	Paenibacillaceae	Aneurinibacillus	CRISPR-associated helicase, Cas3 family	cas3HD,cas3	cd09641,cd09639	CAS-I			
43486..44251	-	SDH44630.1	FNDE01000025.1	Aneurinibacillus thermoaerophilus	Bacteria	Firmicutes	Bacilli	Bacillales	Paenibacillaceae	Aneurinibacillus	CRISPR-associated protein, Cas5 family	cas5	cd09693	CAS-I			

44275..45166	-	SDH44649.1	FNDE01000025.1	Aneurinibacillus thermoaerophilus	Bacteria	Firmicutes	Bacilli	Bacillales	Paenibacillaceae	Aneurinibacillus	CRISPR-associated autoregulator, Cst2 family	cas7	cd09687	CAS-I-B			
45158..46892	-	SDH44675.1	FNDE01000025.1	Aneurinibacillus thermoaerophilus	Bacteria	Firmicutes	Bacilli	Bacillales	Paenibacillaceae	Aneurinibacillus	hypothetical protein	cas8b1	mkCas0195	CAS-I-B			
47075..47834	-	SDH44696.1	FNDE01000025.1	Aneurinibacillus thermoaerophilus	Bacteria	Firmicutes	Bacilli	Bacillales	Paenibacillaceae	Aneurinibacillus	CRISPR-associated protein, Cas6 family	cas6	COG1583	CAS-I,CAS-III,CAS-IV			

112 CAS-I-B | ABP65721.1

60110..62054	array		CP000679.1	Caldicellulosiruptor saccharolyticus DSM 8903	Bacteria	Firmicutes	Clostridia	Thermoanaerobacterales	Thermoanaerobacterales Family III. Incertae Sedis	Caldicellulosiruptor		CRISPR array			30	36	30
62397..63519	-	ABP65710.1	CP000679.1	Caldicellulosiruptor saccharolyticus DSM 8903	Bacteria	Firmicutes	Clostridia	Thermoanaerobacterales	Thermoanaerobacterales Family III. Incertae Sedis	Caldicellulosiruptor	transposase						
63752..65825	+	ABP65711.1	CP000679.1	Caldicellulosiruptor saccharolyticus DSM 8903	Bacteria	Firmicutes	Clostridia	Thermoanaerobacterales	Thermoanaerobacterales Family III. Incertae Sedis	Caldicellulosiruptor	CRISPR-associated protein, Csh1 family	cas8b1	pfam09484	CAS-I-B			
65846..66731	+	ABP65712.1	CP000679.1	Caldicellulosiruptor saccharolyticus DSM 8903	Bacteria	Firmicutes	Clostridia	Thermoanaerobacterales	Thermoanaerobacterales Family III. Incertae Sedis	Caldicellulosiruptor	CRISPR-associated protein, Csh2 family	cas7b	pfam05107	CAS-I-B,CAS-I-C			
66751..67504	+	ABP65713.1	CP000679.1	Caldicellulosiruptor saccharolyticus DSM 8903	Bacteria	Firmicutes	Clostridia	Thermoanaerobacterales	Thermoanaerobacterales Family III. Incertae Sedis	Caldicellulosiruptor	CRISPR-associated protein Cas5	cas5	cd09692	CAS-I-B			
67596..68037	+	ABP65714.1	CP000679.1	Caldicellulosiruptor saccharolyticus DSM 8903	Bacteria	Firmicutes	Clostridia	Thermoanaerobacterales	Thermoanaerobacterales Family III. Incertae Sedis	Caldicellulosiruptor	hypothetical protein	cas3HD	cd09641	CAS-I			
68071..68863	-	ABP65715.1	CP000679.1	Caldicellulosiruptor saccharolyticus DSM 8903	Bacteria	Firmicutes	Clostridia	Thermoanaerobacterales	Thermoanaerobacterales Family III. Incertae Sedis	Caldicellulosiruptor	IstB domain protein ATP-binding protein						
69029..70259	+	ABP65716.1	CP000679.1	Caldicellulosiruptor saccharolyticus DSM 8903	Bacteria	Firmicutes	Clostridia	Thermoanaerobacterales	Thermoanaerobacterales Family III. Incertae Sedis	Caldicellulosiruptor	transposase, mutator type						
70188..71655	-	ABP65717.1	CP000679.1	Caldicellulosiruptor saccharolyticus DSM 8903	Bacteria	Firmicutes	Clostridia	Thermoanaerobacterales	Thermoanaerobacterales Family III. Incertae Sedis	Caldicellulosiruptor	Integrase, catalytic region	casR*	cls001593				
73847..74345	+	ABP65719.2	CP000679.1	Caldicellulosiruptor saccharolyticus DSM 8903	Bacteria	Firmicutes	Clostridia	Thermoanaerobacterales	Thermoanaerobacterales Family III. Incertae Sedis	Caldicellulosiruptor	CRISPR-associated protein Cas4	cas4	pfam01930	CAS-I			
74395..75388	+	ABP65720.1	CP000679.1	Caldicellulosiruptor saccharolyticus DSM 8903	Bacteria	Firmicutes	Clostridia	Thermoanaerobacterales	Thermoanaerobacterales Family III. Incertae Sedis	Caldicellulosiruptor	CRISPR-associated protein Cas1	cas1	cd09722	CAS-I-B			
75393..75657	+	ABP65721.1	CP000679.1	Caldicellulosiruptor saccharolyticus DSM 8903	Bacteria	Firmicutes	Clostridia	Thermoanaerobacterales	Thermoanaerobacterales Family III. Incertae Sedis	Caldicellulosiruptor	CRISPR-associated protein Cas2	**cas2**	cd09725	CAS-I,CAS-II,CAS-III,CAS-V			

114 CAS-I-B | ADQ47295.1

2725100..2725725	array		CP002330.1	Caldicellulosiruptor kronotskyensis 2002	Bacteria	Firmicutes	Clostridia	Thermoanaerobacterales	Thermoanaerobacterales Family III. Incertae Sedis	Caldicellulosiruptor		CRISPR array			10	36	30
2725202..2725802	-	ADQ47293.1	CP002330.1	Caldicellulosiruptor kronotskyensis 2002	Bacteria	Firmicutes	Clostridia	Thermoanaerobacterales	Thermoanaerobacterales Family III. Incertae Sedis	Caldicellulosiruptor	hypothetical protein						
2725925..2726222	+	ADQ47294.1	CP002330.1	Caldicellulosiruptor kronotskyensis 2002	Bacteria	Firmicutes	Clostridia	Thermoanaerobacterales	Thermoanaerobacterales Family III. Incertae Sedis	Caldicellulosiruptor	hypothetical protein						
2726569..2726833	-	ADQ47295.1	CP002330.1	Caldicellulosiruptor kronotskyensis 2002	Bacteria	Firmicutes	Clostridia	Thermoanaerobacterales	Thermoanaerobacterales Family III. Incertae Sedis	Caldicellulosiruptor	CRISPR-associated protein Cas2	**cas2**	cd09725	CAS-I,CAS-II,CAS-III,CAS-V			
2726838..2727831	-	ADQ47296.1	CP002330.1	Caldicellulosiruptor kronotskyensis 2002	Bacteria	Firmicutes	Clostridia	Thermoanaerobacterales	Thermoanaerobacterales Family III. Incertae Sedis	Caldicellulosiruptor	CRISPR-associated protein Cas1	cas1	cd09722	CAS-I-B			
2727877..2728375	-	ADQ47297.1	CP002330.1	Caldicellulosiruptor kronotskyensis 2002	Bacteria	Firmicutes	Clostridia	Thermoanaerobacterales	Thermoanaerobacterales Family III. Incertae Sedis	Caldicellulosiruptor	CRISPR-associated protein Cas4	cas4	pfam01930	CAS-I			
2728403..2730827	-	ADQ47298.1	CP002330.1	Caldicellulosiruptor kronotskyensis 2002	Bacteria	Firmicutes	Clostridia	Thermoanaerobacterales	Thermoanaerobacterales Family III. Incertae Sedis	Caldicellulosiruptor	metal dependent phosphohydrolase	cas3HD,cas3	cd09641,COG1203	CAS-I			
2730918..2731671	-	ADQ47299.1	CP002330.1	Caldicellulosiruptor kronotskyensis 2002	Bacteria	Firmicutes	Clostridia	Thermoanaerobacterales	Thermoanaerobacterales Family III. Incertae Sedis	Caldicellulosiruptor	CRISPR-associated protein Cas5, Hmari subtype	cas5	cd09692	CAS-I-B			
2731691..2732597	-	ADQ47300.1	CP002330.1	Caldicellulosiruptor kronotskyensis 2002	Bacteria	Firmicutes	Clostridia	Thermoanaerobacterales	Thermoanaerobacterales Family III. Incertae Sedis	Caldicellulosiruptor	CRISPR-associated protein, Csh2 family	cas7b	pfam05107	CAS-I-B,CAS-I-C			
2732617..2734681	-	ADQ47301.1	CP002330.1	Caldicellulosiruptor kronotskyensis 2002	Bacteria	Firmicutes	Clostridia	Thermoanaerobacterales	Thermoanaerobacterales Family III. Incertae Sedis	Caldicellulosiruptor	CRISPR-associated protein, Csh1 family	cas8b1	pfam09484	CAS-I-B			
2735113..2736658	array		CP002330.1	Caldicellulosiruptor kronotskyensis 2002	Bacteria	Firmicutes	Clostridia	Thermoanaerobacterales	Thermoanaerobacterales Family III. Incertae Sedis	Caldicellulosiruptor		CRISPR array			24	35	30

115 CAS-I-B | ADL43512.1

2464720..2466679	array		CP002164.1	Caldicellulosiruptor obsidiansis OB47	Bacteria	Firmicutes	Clostridia	Thermoanaerobacterales	Thermoanaerobacterales Family III. Incertae Sedis	Caldicellulosiruptor		CRISPR array			30	36	30
2467054..2467564	+	ADL43510.1	CP002164.1	Caldicellulosiruptor obsidiansis OB47	Bacteria	Firmicutes	Clostridia	Thermoanaerobacterales	Thermoanaerobacterales Family III. Incertae Sedis	Caldicellulosiruptor	CRISPR-associated protein Cas4	cas4	pfam01930	CAS-I			
2467560..2468556	+	ADL43511.1	CP002164.1	Caldicellulosiruptor obsidiansis OB47	Bacteria	Firmicutes	Clostridia	Thermoanaerobacterales	Thermoanaerobacterales Family III. Incertae Sedis	Caldicellulosiruptor	CRISPR-associated protein Cas1	cas1	cd09722	CAS-I-B			
2468572..2468836	+	ADL43512.1	CP002164.1	Caldicellulosiruptor obsidiansis OB47	Bacteria	Firmicutes	Clostridia	Thermoanaerobacterales	Thermoanaerobacterales Family III. Incertae Sedis	Caldicellulosiruptor	CRISPR-associated protein Cas2	**cas2**	cd09725	CAS-I,CAS-II,CAS-III,CAS-V			
2468916..2470848	+	ADL43513.1	CP002164.1	Caldicellulosiruptor obsidiansis OB47	Bacteria	Firmicutes	Clostridia	Thermoanaerobacterales	Thermoanaerobacterales Family III. Incertae Sedis	Caldicellulosiruptor	CRISPR-associated protein, Csh1 family	cas8b1	cd09730	CAS-I-B			
2470897..2471881	+	ADL43514.1	CP002164.1	Caldicellulosiruptor obsidiansis OB47	Bacteria	Firmicutes	Clostridia	Thermoanaerobacterales	Thermoanaerobacterales Family III. Incertae Sedis	Caldicellulosiruptor	CRISPR-associated protein, Csh2 family	cas7	cd09690	CAS-I-B			
2471903..2472635	+	ADL43515.1	CP002164.1	Caldicellulosiruptor obsidiansis OB47	Bacteria	Firmicutes	Clostridia	Thermoanaerobacterales	Thermoanaerobacterales Family III. Incertae Sedis	Caldicellulosiruptor	CRISPR-associated protein Cas5, Hmari subtype	cas5	cls001430	CAS-I-B			
2472609..2475081	+	ADL43516.1	CP002164.1	Caldicellulosiruptor obsidiansis OB47	Bacteria	Firmicutes	Clostridia	Thermoanaerobacterales	Thermoanaerobacterales Family III. Incertae Sedis	Caldicellulosiruptor	CRISPR-associated HD domain protein	cas3HD,cas3	cd09641,COG1203	CAS-I			
2475906..2476863	+	ADL43517.1	CP002164.1	Caldicellulosiruptor obsidiansis OB47	Bacteria	Firmicutes	Clostridia	Thermoanaerobacterales	Thermoanaerobacterales Family III. Incertae Sedis	Caldicellulosiruptor	hypothetical protein	C3a178	cluster178				
2477566..2478791	array		CP002164.1	Caldicellulosiruptor obsidiansis OB47	Bacteria	Firmicutes	Clostridia	Thermoanaerobacterales	Thermoanaerobacterales Family III. Incertae Sedis	Caldicellulosiruptor		CRISPR array			19	36	30

116 CAS-I-B | ADQ42003.1

2719682..2720090	-	ADQ41998.1	CP002326.1	Caldicellulosiruptor kristjanssonii I77R1B	Bacteria	Firmicutes	Clostridia	Thermoanaerobacterales	Thermoanaerobacterales Family III. Incertae Sedis	Caldicellulosiruptor	hypothetical protein	csx20	cls000023	CAS-I,CAS-III			
2720228..2720516	-	ADQ41999.1	CP002326.1	Caldicellulosiruptor kristjanssonii I77R1B	Bacteria	Firmicutes	Clostridia	Thermoanaerobacterales	Thermoanaerobacterales Family III. Incertae Sedis	Caldicellulosiruptor	hypothetical protein						
2720576..2722684	array		CP002326.1	Caldicellulosiruptor kristjanssonii I77R1B	Bacteria	Firmicutes	Clostridia	Thermoanaerobacterales	Thermoanaerobacterales Family III. Incertae Sedis	Caldicellulosiruptor		CRISPR array			32	37	30
2722834..2723215	+	ADQ42000.1	CP002326.1	Caldicellulosiruptor kristjanssonii I77R1B	Bacteria	Firmicutes	Clostridia	Thermoanaerobacterales	Thermoanaerobacterales Family III. Incertae Sedis	Caldicellulosiruptor	hypothetical protein						
2723362..2723866	+	ADQ42001.1	CP002326.1	Caldicellulosiruptor kristjanssonii I77R1B	Bacteria	Firmicutes	Clostridia	Thermoanaerobacterales	Thermoanaerobacterales Family III. Incertae Sedis	Caldicellulosiruptor	CRISPR-associated protein Cas4	cas4	pfam01930	CAS-I			
2723862..2724858	+	ADQ42002.1	CP002326.1	Caldicellulosiruptor kristjanssonii I77R1B	Bacteria	Firmicutes	Clostridia	Thermoanaerobacterales	Thermoanaerobacterales Family III. Incertae Sedis	Caldicellulosiruptor	CRISPR-associated protein Cas1	cas1	cd09722	CAS-I-B			
2724873..2725137	+	ADQ42003.1	CP002326.1	Caldicellulosiruptor kristjanssonii I77R1B	Bacteria	Firmicutes	Clostridia	Thermoanaerobacterales	Thermoanaerobacterales Family III. Incertae Sedis	Caldicellulosiruptor	CRISPR-associated protein Cas2	**cas2**	cd09725	CAS-I,CAS-II,CAS-III,CAS-V			
2725213..2727136	+	ADQ42004.1	CP002326.1	Caldicellulosiruptor kristjanssonii I77R1B	Bacteria	Firmicutes	Clostridia	Thermoanaerobacterales	Thermoanaerobacterales Family III. Incertae Sedis	Caldicellulosiruptor	CRISPR-associated protein, Csh1 family	cas8b1	cd09730	CAS-I-B			
2727200..2728181	+	ADQ42005.1	CP002326.1	Caldicellulosiruptor kristjanssonii I77R1B	Bacteria	Firmicutes	Clostridia	Thermoanaerobacterales	Thermoanaerobacterales Family III. Incertae Sedis	Caldicellulosiruptor	CRISPR-associated protein, Csh2 family	cas7	cd09690	CAS-I-B			
2728203..2728935	+	ADQ42006.1	CP002326.1	Caldicellulosiruptor kristjanssonii I77R1B	Bacteria	Firmicutes	Clostridia	Thermoanaerobacterales	Thermoanaerobacterales Family III. Incertae Sedis	Caldicellulosiruptor	CRISPR-associated protein Cas5, Hmari subtype	cas5	cls001430	CAS-I-B			
2728909..2731378	+	ADQ42007.1	CP002326.1	Caldicellulosiruptor kristjanssonii I77R1B	Bacteria	Firmicutes	Clostridia	Thermoanaerobacterales	Thermoanaerobacterales Family III. Incertae Sedis	Caldicellulosiruptor	CRISPR-associated HD domain protein	cas3HD,cas3	cd09641,COG1203	CAS-I			
2731763..2732192	array		CP002326.1	Caldicellulosiruptor kristjanssonii I77R1B	Bacteria	Firmicutes	Clostridia	Thermoanaerobacterales	Thermoanaerobacterales Family III. Incertae Sedis	Caldicellulosiruptor		CRISPR array			7	37	29
2732622..2733855	-	ADQ42008.1	CP002326.1	Caldicellulosiruptor kristjanssonii I77R1B	Bacteria	Firmicutes	Clostridia	Thermoanaerobacterales	Thermoanaerobacterales Family III. Incertae Sedis	Caldicellulosiruptor	CRISPR-associated protein DxTHG	csx1	cd09728	CAS-III			

117 CAS-I-B | AEM74968.1

2611321..2611729	-	AEM74964.1	CP003001.1	Caldicellulosiruptor lactoaceticus 6A	Bacteria	Firmicutes	Clostridia	Thermoanaerobacterales	Thermoanaerobacterales Family III. Incertae Sedis	Caldicellulosiruptor	hypothetical protein	csx20	cls000023	CAS-I,CAS-III			
2612217..2616136	array		CP003001.1	Caldicellulosiruptor lactoaceticus 6A	Bacteria	Firmicutes	Clostridia	Thermoanaerobacterales	Thermoanaerobacterales Family III. Incertae Sedis	Caldicellulosiruptor		CRISPR array			59	37	30
2616286..2616667	+	AEM74965.1	CP003001.1	Caldicellulosiruptor lactoaceticus 6A	Bacteria	Firmicutes	Clostridia	Thermoanaerobacterales	Thermoanaerobacterales Family III. Incertae Sedis	Caldicellulosiruptor	hypothetical protein						
2616814..2617318	+	AEM74966.1	CP003001.1	Caldicellulosiruptor lactoaceticus 6A	Bacteria	Firmicutes	Clostridia	Thermoanaerobacterales	Thermoanaerobacterales Family III. Incertae Sedis	Caldicellulosiruptor	CRISPR-associated protein Cas4	cas4	pfam01930	CAS-I			
2617314..2618310	+	AEM74967.1	CP003001.1	Caldicellulosiruptor lactoaceticus 6A	Bacteria	Firmicutes	Clostridia	Thermoanaerobacterales	Thermoanaerobacterales Family III. Incertae Sedis	Caldicellulosiruptor	CRISPR-associated protein Cas1	cas1	cd09722	CAS-I-B			
2618325..2618589	+	AEM74968.1	CP003001.1	Caldicellulosiruptor lactoaceticus 6A	Bacteria	Firmicutes	Clostridia	Thermoanaerobacterales	Thermoanaerobacterales Family III. Incertae Sedis	Caldicellulosiruptor	CRISPR-associated protein Cas2	**cas2**	cd09725	CAS-I,CAS-II,CAS-III,CAS-V			
2618665..2620588	+	AEM74969.1	CP003001.1	Caldicellulosiruptor lactoaceticus 6A	Bacteria	Firmicutes	Clostridia	Thermoanaerobacterales	Thermoanaerobacterales Family III. Incertae Sedis	Caldicellulosiruptor	CRISPR-associated protein Csh1 domain protein	cas8b1	cd09730	CAS-I-B			
2620652..2621633	+	AEM74970.1	CP003001.1	Caldicellulosiruptor lactoaceticus 6A	Bacteria	Firmicutes	Clostridia	Thermoanaerobacterales	Thermoanaerobacterales Family III. Incertae Sedis	Caldicellulosiruptor	CRISPR-associated protein, Csh2 family	cas7	cd09690	CAS-I-B			
2621655..2622387	+	AEM74971.1	CP003001.1	Caldicellulosiruptor lactoaceticus 6A	Bacteria	Firmicutes	Clostridia	Thermoanaerobacterales	Thermoanaerobacterales Family III. Incertae Sedis	Caldicellulosiruptor	CRISPR-associated protein Cas5, Hmari subtype	cas5	cls001430	CAS-I-B			
2622361..2624830	+	AEM74972.1	CP003001.1	Caldicellulosiruptor lactoaceticus 6A	Bacteria	Firmicutes	Clostridia	Thermoanaerobacterales	Thermoanaerobacterales Family III. Incertae Sedis	Caldicellulosiruptor	CRISPR-associated HD domain protein	cas3HD,cas3	cd09641,COG1203	CAS-I			
2625113..2625344	-	AEM74973.1	CP003001.1	Caldicellulosiruptor lactoaceticus 6A	Bacteria	Firmicutes	Clostridia	Thermoanaerobacterales	Thermoanaerobacterales Family III. Incertae Sedis	Caldicellulosiruptor	hypothetical protein						
2625215..2625640	array		CP003001.1	Caldicellulosiruptor lactoaceticus 6A	Bacteria	Firmicutes	Clostridia	Thermoanaerobacterales	Thermoanaerobacterales Family III. Incertae Sedis	Caldicellulosiruptor		CRISPR array			7	37	29

118 CAS-I-B | SHF05235.1

2171..3524	+	SHF05071.1	FQVG01000031.1	Caloramator proteoclasticus DSM 10124	Bacteria	Firmicutes	Clostridia	Clostridiales	Clostridiaceae	Caloramator	CRISPR-associated protein, TM1812 family	csx1	cd09732	CAS-III			
3520..3901	+	SHF05110.1	FQVG01000031.1	Caloramator proteoclasticus DSM 10124	Bacteria	Firmicutes	Clostridia	Clostridiales	Clostridiaceae	Caloramator	hypothetical protein	csx20	cls000023	CAS-I,CAS-III			
4084..5467	+	SHF05138.1	FQVG01000031.1	Caloramator proteoclasticus DSM 10124	Bacteria	Firmicutes	Clostridia	Clostridiales	Clostridiaceae	Caloramator	hypothetical protein						
6793..7285	+	SHF05166.1	FQVG01000031.1	Caloramator proteoclasticus DSM 10124	Bacteria	Firmicutes	Clostridia	Clostridiales	Clostridiaceae	Caloramator	CRISPR-associated exonuclease, Cas4 family	cas4	pfam01930	CAS-I			
7295..8288	+	SHF05200.1	FQVG01000031.1	Caloramator proteoclasticus DSM 10124	Bacteria	Firmicutes	Clostridia	Clostridiales	Clostridiaceae	Caloramator	CRISPR-associated protein, Cas1 family	cas1	cd09722	CAS-I-B			
8293..8557	+	SHF05235.1	FQVG01000031.1	Caloramator proteoclasticus DSM 10124	Bacteria	Firmicutes	Clostridia	Clostridiales	Clostridiaceae	Caloramator	CRISPR-associated protein, Cas2 family	**cas2**	cd09725	CAS-I,CAS-II,CAS-III,CAS-V			
8661..9387	+	SHF05270.1	FQVG01000031.1	Caloramator proteoclasticus DSM 10124	Bacteria	Firmicutes	Clostridia	Clostridiales	Clostridiaceae	Caloramator	CRISPR-associated protein, Cas6 family	cas6	COG1583	CAS-I,CAS-III,CAS-IV			
9398..11330	+	SHF05305.1	FQVG01000031.1	Caloramator proteoclasticus DSM 10124	Bacteria	Firmicutes	Clostridia	Clostridiales	Clostridiaceae	Caloramator	CRISPR-associated protein Csh1	cas8b1	pfam09484	CAS-I-B			
11319..12195	+	SHF05343.1	FQVG01000031.1	Caloramator proteoclasticus DSM 10124	Bacteria	Firmicutes	Clostridia	Clostridiales	Clostridiaceae	Caloramator	CRISPR-associated protein, Csh2 family	cas7	cd09690	CAS-I-B			
12204..12918	+	SHF05378.1	FQVG01000031.1	Caloramator proteoclasticus DSM 10124	Bacteria	Firmicutes	Clostridia	Clostridiales	Clostridiaceae	Caloramator	CRISPR-associated protein Cas5h	cas5	cd09692	CAS-I-B			
12955..15247	+	SHF05412.1	FQVG01000031.1	Caloramator proteoclasticus DSM 10124	Bacteria	Firmicutes	Clostridia	Clostridiales	Clostridiaceae	Caloramator	CRISPR-associated helicase, Cas3 family	cas3HD,cas3	cd09641,COG1203	CAS-I			

119 CAS-I-B | CVI72704.1

119750..121702	array		FCNS01000038.1	Clostridiales bacterium CHKCI001	Bacteria	Firmicutes	Clostridia	Clostridiales				CRISPR array			30	36	30
121856..122594	+	CVI72690.1	FCNS01000038.1	Clostridiales bacterium CHKCI001	Bacteria	Firmicutes	Clostridia	Clostridiales			CRISPR associated protein Cas6	cas6	COG1583	CAS-I,CAS-III,CAS-IV			
122614..124567	+	CVI72692.1	FCNS01000038.1	Clostridiales bacterium CHKCI001	Bacteria	Firmicutes	Clostridia	Clostridiales			CRISPR-associated protein (cas_TM1802)	cas8b1	pfam09484	CAS-I-B			
124553..125420	+	CVI72694.1	FCNS01000038.1	Clostridiales bacterium CHKCI001	Bacteria	Firmicutes	Clostridia	Clostridiales			hypothetical protein	cas7	cd09690	CAS-I-B			
125406..126156	+	CVI72696.1	FCNS01000038.1	Clostridiales bacterium CHKCI001	Bacteria	Firmicutes	Clostridia	Clostridiales			CRISPR-associated protein (Cas_Cas5)	cas5	cls001430	CAS-I-B			
126279..128601	+	CVI72698.1	FCNS01000038.1	Clostridiales bacterium CHKCI001	Bacteria	Firmicutes	Clostridia	Clostridiales			CRISPR-associated endonuclease/helicase Cas3	cas3HD,cas3	cd09641,COG1203	CAS-I			
128612..129110	+	CVI72700.1	FCNS01000038.1	Clostridiales bacterium CHKCI001	Bacteria	Firmicutes	Clostridia	Clostridiales			PD-(D/E)XK nuclease superfamily protein	cas4	pfam01930	CAS-I			
129290..130283	+	CVI72702.1	FCNS01000038.1	Clostridiales bacterium CHKCI001	Bacteria	Firmicutes	Clostridia	Clostridiales			CRISPR-associated endonuclease Cas1	cas1	cd09722	CAS-I-B			
130290..130554	+	CVI72704.1	FCNS01000038.1	Clostridiales bacterium CHKCI001	Bacteria	Firmicutes	Clostridia	Clostridiales			CRISPR-associated endoribonuclease Cas2	**cas2**	cd09725	CAS-I,CAS-II,CAS-III,CAS-V			
130660..130975	+	CVI72706.1	FCNS01000038.1	Clostridiales bacterium CHKCI001	Bacteria	Firmicutes	Clostridia	Clostridiales			hypothetical protein						
131110..131554	+	CVI72707.1	FCNS01000038.1	Clostridiales bacterium CHKCI001	Bacteria	Firmicutes	Clostridia	Clostridiales			hypothetical protein						
131896..132187	array		FCNS01000038.1	Clostridiales bacterium CHKCI001	Bacteria	Firmicutes	Clostridia	Clostridiales				CRISPR array			5	35	30

120 CAS-I-B | SCJ70961.1

30139..34452	array		FMGB01000031.1	uncultured Clostridium sp.	Bacteria	Firmicutes	Clostridia	Clostridiales	Clostridiaceae	Clostridium		CRISPR array			66	35	30
34597..34861	-	SCJ70961.1	FMGB01000031.1	uncultured Clostridium sp.	Bacteria	Firmicutes	Clostridia	Clostridiales	Clostridiaceae	Clostridium	CRISPR-associated endoribonuclease Cas2	**cas2**	cd09725	CAS-I,CAS-II,CAS-III,CAS-V			
34954..35947	-	SCJ70975.1	FMGB01000031.1	uncultured Clostridium sp.	Bacteria	Firmicutes	Clostridia	Clostridiales	Clostridiaceae	Clostridium	CRISPR-associated endonuclease Cas1%2C subtype I-B/HMARI/TNEAP	cas1	cd09722	CAS-I-B			
35968..36481	-	SCJ70985.1	FMGB01000031.1	uncultured Clostridium sp.	Bacteria	Firmicutes	Clostridia	Clostridiales	Clostridiaceae	Clostridium	CRISPR-associated protein Cas4	cas4	pfam01930	CAS-I			
36470..38795	-	SCJ71001.1	FMGB01000031.1	uncultured Clostridium sp.	Bacteria	Firmicutes	Clostridia	Clostridiales	Clostridiaceae	Clostridium	helicase Cas3	cas3HD,cas3	cd09641,COG1203	CAS-I			
38834..39566	-	SCJ71015.1	FMGB01000031.1	uncultured Clostridium sp.	Bacteria	Firmicutes	Clostridia	Clostridiales	Clostridiaceae	Clostridium	Uncharacterized protein predicted to be involved in DNA repair (RAMP superfamily)	cas5	cls001430	CAS-I-B			
39576..40452	-	SCJ71029.1	FMGB01000031.1	uncultured Clostridium sp.	Bacteria	Firmicutes	Clostridia	Clostridiales	Clostridiaceae	Clostridium	Uncharacterized protein predicted to be involved in DNA repair	cas7	cd09690	CAS-I-B			
40438..42427	-	SCJ71043.1	FMGB01000031.1	uncultured Clostridium sp.	Bacteria	Firmicutes	Clostridia	Clostridiales	Clostridiaceae	Clostridium	CRISPR-associated protein%2C TM1802 family	cas8b1	pfam09484	CAS-I-B			
42442..43183	-	SCJ71057.1	FMGB01000031.1	uncultured Clostridium sp.	Bacteria	Firmicutes	Clostridia	Clostridiales	Clostridiaceae	Clostridium	Uncharacterized protein predicted to be involved in DNA repair (RAMP superfamily)	cas6	COG1583	CAS-I,CAS-III,CAS-IV			

121 CAS-I-B | KIR01267.1

85458..87926	array		JPZU01000001.1	Lachnospiraceae bacterium TWA4	Bacteria	Firmicutes	Clostridia	Clostridiales	Lachnospiraceae			CRISPR array			38	35	30
90652..92221	-	KIR01260.1	JPZU01000001.1	Lachnospiraceae bacterium TWA4	Bacteria	Firmicutes	Clostridia	Clostridiales	Lachnospiraceae		GMP synthase	CysH	icity0075				
92302..92749	-	KIR01261.1	JPZU01000001.1	Lachnospiraceae bacterium TWA4	Bacteria	Firmicutes	Clostridia	Clostridiales	Lachnospiraceae		hypothetical protein						
92979..93435	-	KIR01262.1	JPZU01000001.1	Lachnospiraceae bacterium TWA4	Bacteria	Firmicutes	Clostridia	Clostridiales	Lachnospiraceae		Excinuclease ABC subunit A	Cas_RecF	cluster17				
99514..99703	-	KIR01263.1	JPZU01000001.1	Lachnospiraceae bacterium TWA4	Bacteria	Firmicutes	Clostridia	Clostridiales	Lachnospiraceae		hypothetical protein						
99699..101103	-	KIR01264.1	JPZU01000001.1	Lachnospiraceae bacterium TWA4	Bacteria	Firmicutes	Clostridia	Clostridiales	Lachnospiraceae		hypothetical protein						
101296..101863	-	KIR01265.1	JPZU01000001.1	Lachnospiraceae bacterium TWA4	Bacteria	Firmicutes	Clostridia	Clostridiales	Lachnospiraceae		hypothetical protein						
102639..103242	+	KIR01266.1	JPZU01000001.1	Lachnospiraceae bacterium TWA4	Bacteria	Firmicutes	Clostridia	Clostridiales	Lachnospiraceae		hypothetical protein						
110469..110733	-	KIR01267.1	JPZU01000001.1	Lachnospiraceae bacterium TWA4	Bacteria	Firmicutes	Clostridia	Clostridiales	Lachnospiraceae		CRISPR-associated protein Cas2	**cas2**	cd09725	CAS-I,CAS-II,CAS-III,CAS-V			
110751..111744	-	KIR01268.1	JPZU01000001.1	Lachnospiraceae bacterium TWA4	Bacteria	Firmicutes	Clostridia	Clostridiales	Lachnospiraceae		CRISPR-associated protein Cas1	cas1	cd09722	CAS-I-B			
111744..112257	-	KIR01269.1	JPZU01000001.1	Lachnospiraceae bacterium TWA4	Bacteria	Firmicutes	Clostridia	Clostridiales	Lachnospiraceae		CRISPR-associated RecB family exonuclease Cas4a	cas4	pfam01930	CAS-I			
112272..114027	-	KIR01270.1	JPZU01000001.1	Lachnospiraceae bacterium TWA4	Bacteria	Firmicutes	Clostridia	Clostridiales	Lachnospiraceae		CRISPR-associated helicase Cas3	cas3	COG1203	CAS-I			
114196..114640	-	KIR01271.1	JPZU01000001.1	Lachnospiraceae bacterium TWA4	Bacteria	Firmicutes	Clostridia	Clostridiales	Lachnospiraceae		hypothetical protein	cas3HD	cd09641	CAS-I			
114694..115444	-	KIR01272.1	JPZU01000001.1	Lachnospiraceae bacterium TWA4	Bacteria	Firmicutes	Clostridia	Clostridiales	Lachnospiraceae		CRISPR-associated protein, Cas5h family	cas5	cd09692	CAS-I-B			
115460..115772	-	KIR01273.1	JPZU01000001.1	Lachnospiraceae bacterium TWA4	Bacteria	Firmicutes	Clostridia	Clostridiales	Lachnospiraceae		CRISPR-associated protein, Csh2 family	cas7	cd09690	CAS-I-B			
115819..116362	-	KIR01274.1	JPZU01000001.1	Lachnospiraceae bacterium TWA4	Bacteria	Firmicutes	Clostridia	Clostridiales	Lachnospiraceae		CRISPR-associated protein, TM1801 family	cas7b	pfam05107	CAS-I-B,CAS-I-C			
116345..117470	-	KIR01275.1	JPZU01000001.1	Lachnospiraceae bacterium TWA4	Bacteria	Firmicutes	Clostridia	Clostridiales	Lachnospiraceae		CRISPR-associated protein, Csh1 family	cas8b1	pfam09484	CAS-I-B			

122 partial CAS-I | SCL81849.1

158321..159842	+	SCL81826.1	FMIF01000001.1	Sporanaerobacter sp. PP17-6a	Bacteria	Firmicutes	Tissierellia	Tissierellales		Sporanaerobacter	CRISPR-associated endonuclease/helicase Cas3	cas3	COG1203	CAS-I			
159901..160393	+	SCL81829.1	FMIF01000001.1	Sporanaerobacter sp. PP17-6a	Bacteria	Firmicutes	Tissierellia	Tissierellales		Sporanaerobacter	CRISPR-associated protein Cas4	cas4	pfam01930	CAS-I			
160337..161399	+	SCL81832.1	FMIF01000001.1	Sporanaerobacter sp. PP17-6a	Bacteria	Firmicutes	Tissierellia	Tissierellales		Sporanaerobacter	CRISPR-associated endonuclease Cas1	cas1	cd09722	CAS-I-B			
161412..161676	+	SCL81849.1	FMIF01000001.1	Sporanaerobacter sp. PP17-6a	Bacteria	Firmicutes	Tissierellia	Tissierellales		Sporanaerobacter	CRISPR-associated endoribonuclease Cas2	**cas2**	cd09725	CAS-I,CAS-II,CAS-III,CAS-V			
161675..161774	+	SCL81852.1	FMIF01000001.1	Sporanaerobacter sp. PP17-6a	Bacteria	Firmicutes	Tissierellia	Tissierellales		Sporanaerobacter	hypothetical protein						
161867..162348	array		FMIF01000001.1	Sporanaerobacter sp. PP17-6a	Bacteria	Firmicutes	Tissierellia	Tissierellales		Sporanaerobacter		CRISPR array			8	34	30

123 CAS-I-B | SHI82189.1

148199..149207	+	SHI81960.1	FQZS01000008.1	Lutispora thermophila DSM 19022	Bacteria	Firmicutes	Clostridia	Clostridiales	Clostridiaceae	Lutispora	proteasome accessory factor B	WYL	COG2378	CAS-I,CAS-III			
149510..150233	+	SHI81988.1	FQZS01000008.1	Lutispora thermophila DSM 19022	Bacteria	Firmicutes	Clostridia	Clostridiales	Clostridiaceae	Lutispora	CRISPR-associated endoribonuclease Cas6	cas6	COG1583	CAS-I,CAS-III,CAS-IV			
150466..151825	array		FQZS01000008.1	Lutispora thermophila DSM 19022	Bacteria	Firmicutes	Clostridia	Clostridiales	Clostridiaceae	Lutispora		CRISPR array			21	36	30
151863..151998	+	SHI82004.1	FQZS01000008.1	Lutispora thermophila DSM 19022	Bacteria	Firmicutes	Clostridia	Clostridiales	Clostridiaceae	Lutispora	hypothetical protein						
151981..152704	+	SHI82031.1	FQZS01000008.1	Lutispora thermophila DSM 19022	Bacteria	Firmicutes	Clostridia	Clostridiales	Clostridiaceae	Lutispora	CRISPR-associated endoribonuclease Cas6	cas6	COG1583	CAS-I,CAS-III,CAS-IV			
152719..154387	+	SHI82055.1	FQZS01000008.1	Lutispora thermophila DSM 19022	Bacteria	Firmicutes	Clostridia	Clostridiales	Clostridiaceae	Lutispora	hypothetical protein	cas8b1	mkCas0195	CAS-I-B			
154379..155264	+	SHI82076.1	FQZS01000008.1	Lutispora thermophila DSM 19022	Bacteria	Firmicutes	Clostridia	Clostridiales	Clostridiaceae	Lutispora	CRISPR-associated autoregulator, Cst2 family	cas7	cd09687	CAS-I-B			
155281..156007	+	SHI82099.1	FQZS01000008.1	Lutispora thermophila DSM 19022	Bacteria	Firmicutes	Clostridia	Clostridiales	Clostridiaceae	Lutispora	CRISPR-associated protein, Cas5 family	cas5	cd09693	CAS-I			
156003..158259	+	SHI82125.1	FQZS01000008.1	Lutispora thermophila DSM 19022	Bacteria	Firmicutes	Clostridia	Clostridiales	Clostridiaceae	Lutispora	CRISPR-associated endonuclease/helicase Cas3	cas3	COG1203	CAS-I			
158267..158759	+	SHI82143.1	FQZS01000008.1	Lutispora thermophila DSM 19022	Bacteria	Firmicutes	Clostridia	Clostridiales	Clostridiaceae	Lutispora	CRISPR-associated exonuclease, Cas4 family	cas4	pfam01930	CAS-I			
158771..159764	+	SHI82165.1	FQZS01000008.1	Lutispora thermophila DSM 19022	Bacteria	Firmicutes	Clostridia	Clostridiales	Clostridiaceae	Lutispora	CRISPR-associated protein, Cas1 family	cas1	cd09722	CAS-I-B			
159765..160029	+	SHI82189.1	FQZS01000008.1	Lutispora thermophila DSM 19022	Bacteria	Firmicutes	Clostridia	Clostridiales	Clostridiaceae	Lutispora	CRISPR-associated protein, Cas2 family	**cas2**	cd09725	CAS-I,CAS-II,CAS-III,CAS-V			

161323..161547	array		FQZS01000008.1	Lutispora thermophila DSM 19022	Bacteria	Firmicutes	Clostridia	Clostridiales	Clostridiaceae	Lutispora		CRISPR array			4	35	30
124 CAS-I-B | APM39055.1																	
2065983..2066882	array		CP018335.1	Clostridium kluyveri	Bacteria	Firmicutes	Clostridia	Clostridiales	Clostridiaceae	Clostridium		CRISPR array			14	36	30
2067050..2067809	-	APM39051.1	CP018335.1	Clostridium kluyveri	Bacteria	Firmicutes	Clostridia	Clostridiales	Clostridiaceae	Clostridium	hypothetical protein						
2067887..2068595	-	APM39052.1	CP018335.1	Clostridium kluyveri	Bacteria	Firmicutes	Clostridia	Clostridiales	Clostridiaceae	Clostridium	CRISPR-associated endoribonuclease Cas6	cas6	COG1583	CAS-I,CAS-III,CAS-IV			
2068743..2069910	-	APM39053.1	CP018335.1	Clostridium kluyveri	Bacteria	Firmicutes	Clostridia	Clostridiales	Clostridiaceae	Clostridium	transposase	c2c9	c2c9	CAS-V-U4			
2069902..2070511	-	APM39054.1	CP018335.1	Clostridium kluyveri	Bacteria	Firmicutes	Clostridia	Clostridiales	Clostridiaceae	Clostridium	IS607 family transposase						
2071104..2071368	-	APM39055.1	CP018335.1	Clostridium kluyveri	Bacteria	Firmicutes	Clostridia	Clostridiales	Clostridiaceae	Clostridium	CRISPR-associated endonuclease Cas2	cas2	cd09725	CAS-I,CAS-II,CAS-III,CAS-V			
2071370..2072363	-	APM39056.1	CP018335.1	Clostridium kluyveri	Bacteria	Firmicutes	Clostridia	Clostridiales	Clostridiaceae	Clostridium	subtype I-B CRISPR-associated endonuclease Cas1	cas1	cd09722	CAS-I-B			
2072383..2072896	-	APM39057.1	CP018335.1	Clostridium kluyveri	Bacteria	Firmicutes	Clostridia	Clostridiales	Clostridiaceae	Clostridium	CRISPR-associated protein Cas4	cas4	pfam01930	CAS-I			
2073041..2075074	array		CP018335.1	Clostridium kluyveri	Bacteria	Firmicutes	Clostridia	Clostridiales	Clostridiaceae	Clostridium		CRISPR array			31	36	30
127 CAS-I-B | KXG43099.1																	
607576..608311	+	KXG43091.1	LSKU01000001.1	Tepidibacillus decaturensis	Bacteria	Firmicutes	Bacilli	Bacillales	Bacillaceae	Tepidibacillus	CRISPR-associated protein	cas6	COG1583	CAS-I,CAS-III,CAS-IV			
608324..608624	+	KXG43092.1	LSKU01000001.1	Tepidibacillus decaturensis	Bacteria	Firmicutes	Bacilli	Bacillales	Bacillaceae	Tepidibacillus	hypothetical protein	cas8b1	cd09730	CAS-I-B			
608707..609205	+	KXG43093.1	LSKU01000001.1	Tepidibacillus decaturensis	Bacteria	Firmicutes	Bacilli	Bacillales	Bacillaceae	Tepidibacillus	hypothetical protein	cas8b1	cd09730	CAS-I-B			
609322..610252	+	KXG43094.1	LSKU01000001.1	Tepidibacillus decaturensis	Bacteria	Firmicutes	Bacilli	Bacillales	Bacillaceae	Tepidibacillus	hypothetical protein	cas8b1	pfam09484	CAS-I-B			
610244..611120	+	KXG43095.1	LSKU01000001.1	Tepidibacillus decaturensis	Bacteria	Firmicutes	Bacilli	Bacillales	Bacillaceae	Tepidibacillus	type I-B CRISPR-associated protein Cas7/Csh2	cas7	cd09690	CAS-I-B			
611130..611853	+	KXG43096.1	LSKU01000001.1	Tepidibacillus decaturensis	Bacteria	Firmicutes	Bacilli	Bacillales	Bacillaceae	Tepidibacillus	type I-B CRISPR-associated protein Cas5	cas5	cd09692	CAS-I-B			
611894..614228	+	KXG43097.1	LSKU01000001.1	Tepidibacillus decaturensis	Bacteria	Firmicutes	Bacilli	Bacillales	Bacillaceae	Tepidibacillus	CRISPR-associated protein Cas3	cas3HD,cas3	cd09641,COG1203	CAS-I			
614737..615730	+	KXG43098.1	LSKU01000001.1	Tepidibacillus decaturensis	Bacteria	Firmicutes	Bacilli	Bacillales	Bacillaceae	Tepidibacillus	type I-B CRISPR-associated endonuclease Cas1	cas1	cd09722	CAS-I-B			
615731..615995	+	KXG43099.1	LSKU01000001.1	Tepidibacillus decaturensis	Bacteria	Firmicutes	Bacilli	Bacillales	Bacillaceae	Tepidibacillus	CRISPR-associated protein Cas2	cas2	cd09725	CAS-I,CAS-II,CAS-III,CAS-V			
616155..618897	array		LSKU01000001.1	Tepidibacillus decaturensis	Bacteria	Firmicutes	Bacilli	Bacillales	Bacillaceae	Tepidibacillus		CRISPR array			42	36	30
129 Partial CAS-I-B | CCJ34755.1																	
4..563	array		CAKP01000154.1	Caloramator australicus RC3	Bacteria	Firmicutes	Clostridia	Clostridiales	Clostridiaceae	Caloramator		CRISPR array			9	36	30
79..199	-	CCJ34752.1	CAKP01000154.1	Caloramator australicus RC3	Bacteria	Firmicutes	Clostridia	Clostridiales	Clostridiaceae	Caloramator	hypothetical protein						
670..1162	+	CCJ34753.1	CAKP01000154.1	Caloramator australicus RC3	Bacteria	Firmicutes	Clostridia	Clostridiales	Clostridiaceae	Caloramator	CRISPR-associated RecB family exonuclease Cas4a	cas4	pfam01930	CAS-I			
1171..2164	+	CCJ34754.1	CAKP01000154.1	Caloramator australicus RC3	Bacteria	Firmicutes	Clostridia	Clostridiales	Clostridiaceae	Caloramator	CRISPR-associated protein Cas1	cas1	cd09722	CAS-I-B			
2171..2429	+	CCJ34755.1	CAKP01000154.1	Caloramator australicus RC3	Bacteria	Firmicutes	Clostridia	Clostridiales	Clostridiaceae	Caloramator	CRISPR-associated protein Cas2	cas2	cd09725	CAS-I,CAS-II,CAS-III,CAS-V			
2468..2861	+	CCJ34756.1	CAKP01000154.1	Caloramator australicus RC3	Bacteria	Firmicutes	Clostridia	Clostridiales	Clostridiaceae	Caloramator	Uncharacterized protein MJ1673	csx20	xls002255	CAS-I,CAS-III			
2866..3793	+	CCJ34757.1	CAKP01000154.1	Caloramator australicus RC3	Bacteria	Firmicutes	Clostridia	Clostridiales	Clostridiaceae	Caloramator	CRISPR repeat RNA endoribonuclease Cas6	cas6	mkCas0066	CAS-I,CAS-III			
130 CAS-I-B | ABS60793.1																	
1012611..1013334	+	ABS60786.1	CP000771.1	Fervidobacterium nodosum Rt17-B1	Bacteria	Thermotogae	Thermotogae	Thermotogales	Fervidobacteriaceae	Fervidobacterium	CRISPR-associated protein Cas6	cas6	COG1583	CAS-I,CAS-III,CAS-IV			
1013355..1015269	+	ABS60787.1	CP000771.1	Fervidobacterium nodosum Rt17-B1	Bacteria	Thermotogae	Thermotogae	Thermotogales	Fervidobacteriaceae	Fervidobacterium	CRISPR-associated protein, Csh1 family	cas8b1	pfam09484	CAS-I-B			
1015261..1016170	+	ABS60788.1	CP000771.1	Fervidobacterium nodosum Rt17-B1	Bacteria	Thermotogae	Thermotogae	Thermotogales	Fervidobacteriaceae	Fervidobacterium	CRISPR-associated protein, Csh2 family	cas7	cd09690	CAS-I-B			
1016174..1016885	+	ABS60789.1	CP000771.1	Fervidobacterium nodosum Rt17-B1	Bacteria	Thermotogae	Thermotogae	Thermotogales	Fervidobacteriaceae	Fervidobacterium	CRISPR-associated protein Cas5, Hmari subtype	cas5	cd09692	CAS-I-B			
1016894..1019297	+	ABS60790.1	CP000771.1	Fervidobacterium nodosum Rt17-B1	Bacteria	Thermotogae	Thermotogae	Thermotogales	Fervidobacteriaceae	Fervidobacterium	CRISPR-associated helicase Cas3	cas3HD,cas3	cd09641,COG1203	CAS-I			
1019473..1031294	array		CP000771.1	Fervidobacterium nodosum Rt17-B1	Bacteria	Thermotogae	Thermotogae	Thermotogales	Fervidobacteriaceae	Fervidobacterium		CRISPR array			179	36	30
1031475..1031967	+	ABS60791.1	CP000771.1	Fervidobacterium nodosum Rt17-B1	Bacteria	Thermotogae	Thermotogae	Thermotogales	Fervidobacteriaceae	Fervidobacterium	CRISPR-associated protein Cas4	cas4	pfam01930	CAS-I			
1031980..1032973	+	ABS60792.1	CP000771.1	Fervidobacterium nodosum Rt17-B1	Bacteria	Thermotogae	Thermotogae	Thermotogales	Fervidobacteriaceae	Fervidobacterium	CRISPR-associated protein Cas1	cas1	cd09722	CAS-I-B			
1032976..1033240	+	ABS60793.1	CP000771.1	Fervidobacterium nodosum Rt17-B1	Bacteria	Thermotogae	Thermotogae	Thermotogales	Fervidobacteriaceae	Fervidobacterium	CRISPR-associated protein Cas2	cas2	cd09725	CAS-I,CAS-II,CAS-III,CAS-V			
1033376..1034216	+	ABS60794.1	CP000771.1	Fervidobacterium nodosum Rt17-B1	Bacteria	Thermotogae	Thermotogae	Thermotogales	Fervidobacteriaceae	Fervidobacterium	protein of unknown function DUF6 transmembrane						
1034252..1035995	+	ABS60795.1	CP000771.1	Fervidobacterium nodosum Rt17-B1	Bacteria	Thermotogae	Thermotogae	Thermotogales	Fervidobacteriaceae	Fervidobacterium	ABC transporter related	Cas_RecF	cluster17				
131 CAS-I-B | ACJ75697.1																	
1258719..1258983	-	ACJ75697.1	CP001185.1	Thermosipho africanus TCF52B	Bacteria	Thermotogae	Thermotogae	Thermotogales	Fervidobacteriaceae	Thermosipho	crispr-associated protein Cas2	cas2	cd09725	CAS-I,CAS-II,CAS-III,CAS-V			
1258986..1259979	-	ACJ75698.1	CP001185.1	Thermosipho africanus TCF52B	Bacteria	Thermotogae	Thermotogae	Thermotogales	Fervidobacteriaceae	Thermosipho	cas crispr-associated protein Cas1	cas1	cd09722	CAS-I-B			
1259994..1260426	-	ACJ75699.1	CP001185.1	Thermosipho africanus TCF52B	Bacteria	Thermotogae	Thermotogae	Thermotogales	Fervidobacteriaceae	Thermosipho	cas crispr-associated protein Cas4	cas4	pfam01930	CAS-I			
1260669..1262686	array		CP001185.1	Thermosipho africanus TCF52B	Bacteria	Thermotogae	Thermotogae	Thermotogales	Fervidobacteriaceae	Thermosipho		CRISPR array			31	36	30
1261486..1261612	+	ACJ75700.1	CP001185.1	Thermosipho africanus TCF52B	Bacteria	Thermotogae	Thermotogae	Thermotogales	Fervidobacteriaceae	Thermosipho	hypothetical protein						
1261717..1261831	+	ACJ75701.1	CP001185.1	Thermosipho africanus TCF52B	Bacteria	Thermotogae	Thermotogae	Thermotogales	Fervidobacteriaceae	Thermosipho	hypothetical protein						
1262840..1265192	-	ACJ75702.1	CP001185.1	Thermosipho africanus TCF52B	Bacteria	Thermotogae	Thermotogae	Thermotogales	Fervidobacteriaceae	Thermosipho	crispr-associated helicase Cas3	cas3HD,cas3	cd09641,COG1203	CAS-I			
1265252..1265963	-	ACJ75703.1	CP001185.1	Thermosipho africanus TCF52B	Bacteria	Thermotogae	Thermotogae	Thermotogales	Fervidobacteriaceae	Thermosipho	cas5h crispr-associated protein Cas5, hmari subtype	cas5	cd09692	CAS-I-B			
1265967..1266876	-	ACJ75704.1	CP001185.1	Thermosipho africanus TCF52B	Bacteria	Thermotogae	Thermotogae	Thermotogales	Fervidobacteriaceae	Thermosipho	csh crispr-associated protein, Csh2 family	cas7	cd09690	CAS-I-B			
1266868..1268782	-	ACJ75705.1	CP001185.1	Thermosipho africanus TCF52B	Bacteria	Thermotogae	Thermotogae	Thermotogales	Fervidobacteriaceae	Thermosipho	crispr-associated protein, family	cas8b1	pfam09484	CAS-I-B			
1268803..1269526	-	ACJ75706.1	CP001185.1	Thermosipho africanus TCF52B	Bacteria	Thermotogae	Thermotogae	Thermotogales	Fervidobacteriaceae	Thermosipho	cas crispr-associated protein Cas6	cas6	COG1583	CAS-I,CAS-III,CAS-IV			
132 CAS-I-B | OFI05275.1																	
2519..8814	array		LZFO01000031.1	Clostridium acetireducens DSM 10703	Bacteria	Firmicutes	Clostridia	Clostridiales	Clostridiaceae	Clostridium		CRISPR array			95	36	30
8957..9221	-	OFI05275.1	LZFO01000031.1	Clostridium acetireducens DSM 10703	Bacteria	Firmicutes	Clostridia	Clostridiales	Clostridiaceae	Clostridium	CRISPR-associated endoribonuclease Cas2	cas2	cd09725	CAS-I,CAS-II,CAS-III,CAS-V			
9242..10235	-	OFI05276.1	LZFO01000031.1	Clostridium acetireducens DSM 10703	Bacteria	Firmicutes	Clostridia	Clostridiales	Clostridiaceae	Clostridium	CRISPR-associated endonuclease Cas1	cas1	cd09722	CAS-I-B			
10254..10752	-	OFI05277.1	LZFO01000031.1	Clostridium acetireducens DSM 10703	Bacteria	Firmicutes	Clostridia	Clostridiales	Clostridiaceae	Clostridium	hypothetical protein	cas4	pfam01930	CAS-I			
10776..13104	-	OFI05278.1	LZFO01000031.1	Clostridium acetireducens DSM 10703	Bacteria	Firmicutes	Clostridia	Clostridiales	Clostridiaceae	Clostridium	CRISPR-associated endonuclease/helicase Cas3	cas3HD,cas3	cd09641,COG1203	CAS-I			
13207..13948	-	OFI05279.1	LZFO01000031.1	Clostridium acetireducens DSM 10703	Bacteria	Firmicutes	Clostridia	Clostridiales	Clostridiaceae	Clostridium	CRISPR-associated protein Cas5	cas5	cd09692	CAS-I-B			
13958..14834	-	OFI05280.1	LZFO01000031.1	Clostridium acetireducens DSM 10703	Bacteria	Firmicutes	Clostridia	Clostridiales	Clostridiaceae	Clostridium	hypothetical protein	cas7	cd09690	CAS-I-B			
14823..16773	-	OFI05281.1	LZFO01000031.1	Clostridium acetireducens DSM 10703	Bacteria	Firmicutes	Clostridia	Clostridiales	Clostridiaceae	Clostridium	CRISPR-associated protein	cas8b1	pfam09484	CAS-I-B			
16895..17642	-	OFI05282.1	LZFO01000031.1	Clostridium acetireducens DSM 10703	Bacteria	Firmicutes	Clostridia	Clostridiales	Clostridiaceae	Clostridium	CRISPR associated protein Cas6	cas6	COG1583	CAS-I,CAS-III,CAS-IV			
18313..26519	array		LZFO01000031.1	Clostridium acetireducens DSM 10703	Bacteria	Firmicutes	Clostridia	Clostridiales	Clostridiaceae	Clostridium		CRISPR array			123	37	30
133 CAS-I-B | OPJ62159.1																	
71466..73337	array		MZGV01000017.1	Clostridium oryzae	Bacteria	Firmicutes	Clostridia	Clostridiales	Clostridiaceae	Clostridium		CRISPR array			29	35	30
73499..73763	-	OPJ62159.1	MZGV01000017.1	Clostridium oryzae	Bacteria	Firmicutes	Clostridia	Clostridiales	Clostridiaceae	Clostridium	CRISPR-associated endoribonuclease Cas2	cas2	cd09725	CAS-I,CAS-II,CAS-III,CAS-V			
73837..74830	-	OPJ62160.1	MZGV01000017.1	Clostridium oryzae	Bacteria	Firmicutes	Clostridia	Clostridiales	Clostridiaceae	Clostridium	CRISPR-associated endonuclease Cas1	cas1	cd09722	CAS-I-B			
74859..75351	-	OPJ62161.1	MZGV01000017.1	Clostridium oryzae	Bacteria	Firmicutes	Clostridia	Clostridiales	Clostridiaceae	Clostridium	hypothetical protein	cas4	pfam01930	CAS-I			
75374..77705	-	OPJ62162.1	MZGV01000017.1	Clostridium oryzae	Bacteria	Firmicutes	Clostridia	Clostridiales	Clostridiaceae	Clostridium	CRISPR-associated nuclease/helicase Cas3	cas3HD,cas3	cd09641,COG1203	CAS-I			
77721..78465	-	OPJ62163.1	MZGV01000017.1	Clostridium oryzae	Bacteria	Firmicutes	Clostridia	Clostridiales	Clostridiaceae	Clostridium	CRISPR-associated protein	cas5	cls001430	CAS-I-B			
78474..79353	-	OPJ62164.1	MZGV01000017.1	Clostridium oryzae	Bacteria	Firmicutes	Clostridia	Clostridiales	Clostridiaceae	Clostridium	hypothetical protein	cas7	cd09690	CAS-I-B			
79339..81289	-	OPJ62165.1	MZGV01000017.1	Clostridium oryzae	Bacteria	Firmicutes	Clostridia	Clostridiales	Clostridiaceae	Clostridium	CRISPR-associated protein	cas8b1	pfam09484	CAS-I-B			
81301..82039	-	OPJ62166.1	MZGV01000017.1	Clostridium oryzae	Bacteria	Firmicutes	Clostridia	Clostridiales	Clostridiaceae	Clostridium	CRISPR associated protein Cas6	cas6	COG1583	CAS-I,CAS-III,CAS-IV			
82897..83812	-	OPJ62167.1	MZGV01000017.1	Clostridium oryzae	Bacteria	Firmicutes	Clostridia	Clostridiales	Clostridiaceae	Clostridium	flagellar assembly protein H	C3a178	cluster178				
134 Partial CAS-I-B | SDL41597.1																	
372..864	+	SDL41549.1	FNGL01000031.1	Clostridium cochlearium	Bacteria	Firmicutes	Clostridia	Clostridiales	Clostridiaceae	Clostridium	CRISPR-associated exonuclease, Cas4 family	cas4	pfam01930	CAS-I			
879..1872	+	SDL41576.1	FNGL01000031.1	Clostridium cochlearium	Bacteria	Firmicutes	Clostridia	Clostridiales	Clostridiaceae	Clostridium	CRISPR-associated protein, Cas1 family	cas1	cd09722	CAS-I-B			
1921..2185	+	SDL41597.1	FNGL01000031.1	Clostridium cochlearium	Bacteria	Firmicutes	Clostridia	Clostridiales	Clostridiaceae	Clostridium	CRISPR-associated protein, Cas2 family	cas2	COG1343	CAS-I,CAS-II,CAS-III			
2416..3501	array		FNGL01000031.1	Clostridium cochlearium	Bacteria	Firmicutes	Clostridia	Clostridiales	Clostridiaceae	Clostridium		CRISPR array			17	37	29
135 CAS-I-B | SQB33504.1																	
473783..475343	array		UAWC01000001.1	Clostridium cochlearium	Bacteria	Firmicutes	Clostridia	Clostridiales	Clostridiaceae	Clostridium		CRISPR array			24	37	29
475573..475837	-	SQB33504.1	UAWC01000001.1	Clostridium cochlearium	Bacteria	Firmicutes	Clostridia	Clostridiales	Clostridiaceae	Clostridium	CRISPR-associated Cas2 family protein	cas2	cd09725	CAS-I,CAS-II,CAS-III,CAS-V			
475886..476879	-	SQB33505.1	UAWC01000001.1	Clostridium cochlearium	Bacteria	Firmicutes	Clostridia	Clostridiales	Clostridiaceae	Clostridium	CRISPR-associated Cas1 family protein	cas1	cd09722	CAS-I-B			
476894..477386	-	SQB33506.1	UAWC01000001.1	Clostridium cochlearium	Bacteria	Firmicutes	Clostridia	Clostridiales	Clostridiaceae	Clostridium	CRISPR-associated Cas4 family protein	cas4	pfam01930	CAS-I			
477455..479789	-	SQB33507.1	UAWC01000001.1	Clostridium cochlearium	Bacteria	Firmicutes	Clostridia	Clostridiales	Clostridiaceae	Clostridium	CRISPR-associated helicase, Cas3 family	cas3	COG1203	CAS-I			
479880..480615	-	SQB33508.1	UAWC01000001.1	Clostridium cochlearium	Bacteria	Firmicutes	Clostridia	Clostridiales	Clostridiaceae	Clostridium	CRISPR-associated protein, Cas5h family	cas5	cd09692	CAS-I-B			
480796..481528	-	SQB33509.1	UAWC01000001.1	Clostridium cochlearium	Bacteria	Firmicutes	Clostridia	Clostridiales	Clostridiaceae	Clostridium	CRISPR-associated Csh2 family protein	cas7b	pfam05107	CAS-I-B,CAS-I-C			
481517..483515	-	SQB33510.1	UAWC01000001.1	Clostridium cochlearium	Bacteria	Firmicutes	Clostridia	Clostridiales	Clostridiaceae	Clostridium	CRISPR-associated Csh1 family protein	cas8b1	pfam09484	CAS-I-B			
483538..484276	-	SQB33511.1	UAWC01000001.1	Clostridium cochlearium	Bacteria	Firmicutes	Clostridia	Clostridiales	Clostridiaceae	Clostridium	CRISPR-associated Cas family protein	cas6	COG1583	CAS-I,CAS-III,CAS-IV			
484486..484783	array		UAWC01000001.1	Clostridium cochlearium	Bacteria	Firmicutes	Clostridia	Clostridiales	Clostridiaceae	Clostridium		CRISPR array			5	35	31
136 CAS-I-B | STA92354.1																	
979012..981125	array		UFWH01000001.1	Clostridium cochlearium	Bacteria	Firmicutes	Clostridia	Clostridiales	Clostridiaceae	Clostridium		CRISPR array			32	37	30
981194..981527	+	STA92342.1	UFWH01000001.1	Clostridium cochlearium	Bacteria	Firmicutes	Clostridia	Clostridiales	Clostridiaceae	Clostridium	Uncharacterised protein						
981520..981871	+	STA92343.1	UFWH01000001.1	Clostridium cochlearium	Bacteria	Firmicutes	Clostridia	Clostridiales	Clostridiaceae	Clostridium	IS66 like transposase						
981960..983595	+	STA92344.1	UFWH01000001.1	Clostridium cochlearium	Bacteria	Firmicutes	Clostridia	Clostridiales	Clostridiaceae	Clostridium	transposase IS66						
983846..984584	+	STA92346.1	UFWH01000001.1	Clostridium cochlearium	Bacteria	Firmicutes	Clostridia	Clostridiales	Clostridiaceae	Clostridium	CRISPR-associated Cas family protein	cas6	COG1583	CAS-I,CAS-III,CAS-IV			
984607..986605	+	STA92347.1	UFWH01000001.1	Clostridium cochlearium	Bacteria	Firmicutes	Clostridia	Clostridiales	Clostridiaceae	Clostridium	CRISPR-associated Csh1 family protein	cas8b1	pfam09484	CAS-I-B			
986594..987467	+	STA92348.1	UFWH01000001.1	Clostridium cochlearium	Bacteria	Firmicutes	Clostridia	Clostridiales	Clostridiaceae	Clostridium	Csh2 family CRISPR-associated protein	cas7	cd09690	CAS-I-B			
987519..988254	+	STA92349.1	UFWH01000001.1	Clostridium cochlearium	Bacteria	Firmicutes	Clostridia	Clostridiales	Clostridiaceae	Clostridium	CRISPR-associated Cas5 family Hmari subtype protein	cas5	cd09692	CAS-I-B			
988345..990679	+	STA92350.1	UFWH01000001.1	Clostridium cochlearium	Bacteria	Firmicutes	Clostridia	Clostridiales	Clostridiaceae	Clostridium	CRISPR-associated helicase, Cas3 family	cas3	COG1203	CAS-I			
990762..991254	+	STA92351.1	UFWH01000001.1	Clostridium cochlearium	Bacteria	Firmicutes	Clostridia	Clostridiales	Clostridiaceae	Clostridium	CRISPR-associated Cas4 family protein	cas4	pfam01930	CAS-I			
991269..992262	+	STA92353.1	UFWH01000001.1	Clostridium cochlearium	Bacteria	Firmicutes	Clostridia	Clostridiales	Clostridiaceae	Clostridium	CRISPR-associated Cas1 family protein	cas1	cd09722	CAS-I-B			
992311..992575	+	STA92354.1	UFWH01000001.1	Clostridium cochlearium	Bacteria	Firmicutes	Clostridia	Clostridiales	Clostridiaceae	Clostridium	CRISPR-associated Cas2 family protein	cas2	cd09725	CAS-I,CAS-II,CAS-III,CAS-V			
992805..993298	array		UFWH01000001.1	Clostridium cochlearium	Bacteria	Firmicutes	Clostridia	Clostridiales	Clostridiaceae	Clostridium		CRISPR array			8	36	30
137 CAS-I-B | OOL91278.1																	
149041..149338	array		LZYT01000006.1	Clostridium felsineum DSM 794	Bacteria	Firmicutes	Clostridia	Clostridiales	Clostridiaceae	Clostridium		CRISPR array			5	35	31
149527..149957	array		LZYT01000006.1	Clostridium felsineum DSM 794	Bacteria	Firmicutes	Clostridia	Clostridiales	Clostridiaceae	Clostridium		CRISPR array			7	36	30
150770..151137	array		LZYT01000006.1	Clostridium felsineum DSM 794	Bacteria	Firmicutes	Clostridia	Clostridiales	Clostridiaceae	Clostridium		CRISPR array			6	36	31
151467..151819	array		LZYT01000006.1	Clostridium felsineum DSM 794	Bacteria	Firmicutes	Clostridia	Clostridiales	Clostridiaceae	Clostridium		CRISPR array			6	47	19
152142..152639	array		LZYT01000006.1	Clostridium felsineum DSM 794	Bacteria	Firmicutes	Clostridia	Clostridiales	Clostridiaceae	Clostridium		CRISPR array			8	36	30
153728..153957	array		LZYT01000006.1	Clostridium felsineum DSM 794	Bacteria	Firmicutes	Clostridia	Clostridiales	Clostridiaceae	Clostridium		CRISPR array			4	35	31
154276..154645	array		LZYT01000006.1	Clostridium felsineum DSM 794	Bacteria	Firmicutes	Clostridia	Clostridiales	Clostridiaceae	Clostridium		CRISPR array			6	38	30
154814..155669	-	OOL91277.1	LZYT01000006.1	Clostridium felsineum DSM 794	Bacteria	Firmicutes	Clostridia	Clostridiales	Clostridiaceae	Clostridium	PD-(D/E)XK nuclease family transposase						
156096..156326	array		LZYT01000006.1	Clostridium felsineum DSM 794	Bacteria	Firmicutes	Clostridia	Clostridiales	Clostridiaceae	Clostridium		CRISPR array			4	34	32
156752..157182	array		LZYT01000006.1	Clostridium felsineum DSM 794	Bacteria	Firmicutes	Clostridia	Clostridiales	Clostridiaceae	Clostridium		CRISPR array			7	36	30
157504..158269	array		LZYT01000006.1	Clostridium felsineum DSM 794	Bacteria	Firmicutes	Clostridia	Clostridiales	Clostridiaceae	Clostridium		CRISPR array			12	36	30
159318..159678	array		LZYT01000006.1	Clostridium felsineum DSM 794	Bacteria	Firmicutes	Clostridia	Clostridiales	Clostridiaceae	Clostridium		CRISPR array			6	36	30
160424..160688	-	OOL91278.1	LZYT01000006.1	Clostridium felsineum DSM 794	Bacteria	Firmicutes	Clostridia	Clostridiales	Clostridiaceae	Clostridium	CRISPR-associated endoribonuclease Cas2	cas2	cd09725	CAS-I,CAS-II,CAS-III,CAS-V			
160691..161684	-	OOL91279.1	LZYT01000006.1	Clostridium felsineum DSM 794	Bacteria	Firmicutes	Clostridia	Clostridiales	Clostridiaceae	Clostridium	CRISPR-associated endonuclease Cas1	cas1	cd09722	CAS-I-B			
161696..162188	-	OOL91280.1	LZYT01000006.1	Clostridium felsineum DSM 794	Bacteria	Firmicutes	Clostridia	Clostridiales	Clostridiaceae	Clostridium	hypothetical protein	cas4	pfam01930	CAS-I			
162198..164508	-	OOL91281.1	LZYT01000006.1	Clostridium felsineum DSM 794	Bacteria	Firmicutes	Clostridia	Clostridiales	Clostridiaceae	Clostridium	CRISPR-associated nuclease/helicase Cas3	cas3HD,cas3	cd09641,COG1203	CAS-I			
164519..165242	-	OOL91282.1	LZYT01000006.1	Clostridium felsineum DSM 794	Bacteria	Firmicutes	Clostridia	Clostridiales	Clostridiaceae	Clostridium	CRISPR-associated protein Cas5	cas5	cls001430	CAS-I-B			
165252..166128	-	OOL91283.1	LZYT01000006.1	Clostridium felsineum DSM 794	Bacteria	Firmicutes	Clostridia	Clostridiales	Clostridiaceae	Clostridium	hypothetical protein	cas7	cd09690	CAS-I-B			
166117..168067	-	OOL91284.1	LZYT01000006.1	Clostridium felsineum DSM 794	Bacteria	Firmicutes	Clostridia	Clostridiales	Clostridiaceae	Clostridium	CRISPR-associated protein	cas8b1	pfam09484	CAS-I-B			
138 CAS-I-B | PRR82599.1																	
12620..14113	array		PVXP01000053.1	Clostridium luticellarii	Bacteria	Firmicutes	Clostridia	Clostridiales	Clostridiaceae	Clostridium		CRISPR array			23	36	30
14317..14581	-	PRR82599.1	PVXP01000053.1	Clostridium luticellarii	Bacteria	Firmicutes	Clostridia	Clostridiales	Clostridiaceae	Clostridium	CRISPR-associated endoribonuclease Cas2	cas2	cd09725	CAS-I,CAS-II,CAS-III,CAS-V			
14584..15577	-	PRR82600.1	PVXP01000053.1	Clostridium luticellarii	Bacteria	Firmicutes	Clostridia	Clostridiales	Clostridiaceae	Clostridium	CRISPR-associated endonuclease Cas1	cas1	cd09722	CAS-I-B			
15586..16075	-	PRR82601.1	PVXP01000053.1	Clostridium luticellarii	Bacteria	Firmicutes	Clostridia	Clostridiales	Clostridiaceae	Clostridium	hypothetical protein	cas4	pfam01930	CAS-I			
16090..18607	-	PRR82602.1	PVXP01000053.1	Clostridium luticellarii	Bacteria	Firmicutes	Clostridia	Clostridiales	Clostridiaceae	Clostridium	CRISPR-associated nuclease/helicase Cas3	cas3HD,cas3	cd09641,COG1203	CAS-I			
18594..19320	-	PRR82603.1	PVXP01000053.1	Clostridium luticellarii	Bacteria	Firmicutes	Clostridia	Clostridiales	Clostridiaceae	Clostridium	hypothetical protein	cas5	cd09692	CAS-I-B			
19357..20419	-	PRR82604.1	PVXP01000053.1	Clostridium luticellarii	Bacteria	Firmicutes	Clostridia	Clostridiales	Clostridiaceae	Clostridium	hypothetical protein	cas7b	pfam05107	CAS-I-B,CAS-I-C			
20421..22449	-	PRR82605.1	PVXP01000053.1	Clostridium luticellarii	Bacteria	Firmicutes	Clostridia	Clostridiales	Clostridiaceae	Clostridium	CRISPR-associated protein	cas8b1	pfam09484	CAS-I-B			
140 CAS-I-B | OBR96939.1																	
38663..40486	array		LROS01000002.1	Clostridium ragsdalei P11	Bacteria	Firmicutes	Clostridia	Clostridiales	Clostridiaceae	Clostridium		CRISPR array			28	36	30

40790..40895	+	OBR96931.1	LROS01000002.1	Clostridium ragsdalei P11	Bacteria	Firmicutes	Clostridia	Clostridiales	Clostridiaceae	Clostridium	hypothetical protein						
41296..42103	+	OBR96932.1	LROS01000002.1	Clostridium ragsdalei P11	Bacteria	Firmicutes	Clostridia	Clostridiales	Clostridiaceae	Clostridium	CRISPR associated protein Cas6	cas6	COG1583	CAS-I,CAS-III,CAS-IV			
42095..44039	+	OBR96933.1	LROS01000002.1	Clostridium ragsdalei P11	Bacteria	Firmicutes	Clostridia	Clostridiales	Clostridiaceae	Clostridium	CRISPR-associated protein (cas_TM1802)	cas8b1	pfam09484	CAS-I-B			
44028..44904	+	OBR96934.1	LROS01000002.1	Clostridium ragsdalei P11	Bacteria	Firmicutes	Clostridia	Clostridiales	Clostridiaceae	Clostridium	hypothetical protein	cas7	cd09690	CAS-I-B			
44914..45637	+	OBR96935.1	LROS01000002.1	Clostridium ragsdalei P11	Bacteria	Firmicutes	Clostridia	Clostridiales	Clostridiaceae	Clostridium	CRISPR-associated protein (Cas_Cas5)	cas5	cls001430	CAS-I-B			
45648..47961	+	OBR96936.1	LROS01000002.1	Clostridium ragsdalei P11	Bacteria	Firmicutes	Clostridia	Clostridiales	Clostridiaceae	Clostridium	CRISPR-associated nuclease/helicase Cas3	cas3HD,cas3	cd09641,COG1203	CAS-I			
47971..48463	+	OBR96937.1	LROS01000002.1	Clostridium ragsdalei P11	Bacteria	Firmicutes	Clostridia	Clostridiales	Clostridiaceae	Clostridium	hypothetical protein	cas4	pfam01930	CAS-I			
48472..49465	+	OBR96938.1	LROS01000002.1	Clostridium ragsdalei P11	Bacteria	Firmicutes	Clostridia	Clostridiales	Clostridiaceae	Clostridium	CRISPR-associated endonuclease Cas1	cas1	cd09722	CAS-I-B			
49468..49732	+	OBR96939.1	LROS01000002.1	Clostridium ragsdalei P11	Bacteria	Firmicutes	Clostridia	Clostridiales	Clostridiaceae	Clostridium	CRISPR-associated endoribonuclease Cas2	cas2	cd09725	CAS-I,CAS-II,CAS-III,CAS-V			
141 CAS-I-B | OAA83838.1																	
32094..33736	array		LITT01000046.1	Clostridium ljungdahlii	Bacteria	Firmicutes	Clostridia	Clostridiales	Clostridiaceae	Clostridium		CRISPR array			25	37	30
33909..34173	-	OAA83838.1	LITT01000046.1	Clostridium ljungdahlii	Bacteria	Firmicutes	Clostridia	Clostridiales	Clostridiaceae	Clostridium	CRISPR-associated endoribonuclease Cas2	cas2	cd09725	CAS-I,CAS-II,CAS-III,CAS-V			
34176..35169	-	OAA83839.1	LITT01000046.1	Clostridium ljungdahlii	Bacteria	Firmicutes	Clostridia	Clostridiales	Clostridiaceae	Clostridium	CRISPR-associated endonuclease Cas1	cas1	cd09722	CAS-I-B			
35178..35670	-	OAA83840.1	LITT01000046.1	Clostridium ljungdahlii	Bacteria	Firmicutes	Clostridia	Clostridiales	Clostridiaceae	Clostridium	hypothetical protein	cas4	pfam01930	CAS-I			
35680..37993	-	OAA83841.1	LITT01000046.1	Clostridium ljungdahlii	Bacteria	Firmicutes	Clostridia	Clostridiales	Clostridiaceae	Clostridium	CRISPR-associated nuclease/helicase Cas3	cas3HD,cas3	cd09641,COG1203	CAS-I			
38004..38727	-	OAA83842.1	LITT01000046.1	Clostridium ljungdahlii	Bacteria	Firmicutes	Clostridia	Clostridiales	Clostridiaceae	Clostridium	CRISPR-associated protein	cas5	cls001430	CAS-I-B			
38738..39614	-	OAA83843.1	LITT01000046.1	Clostridium ljungdahlii	Bacteria	Firmicutes	Clostridia	Clostridiales	Clostridiaceae	Clostridium	hypothetical protein	cas7	cd09690	CAS-I-B			
39615..41535	-	OAA83844.1	LITT01000046.1	Clostridium ljungdahlii	Bacteria	Firmicutes	Clostridia	Clostridiales	Clostridiaceae	Clostridium	CRISPR-associated protein	cas8b1	pfam09484	CAS-I-B			
41930..42707	-	OAA83845.1	LITT01000046.1	Clostridium ljungdahlii	Bacteria	Firmicutes	Clostridia	Clostridiales	Clostridiaceae	Clostridium	CRISPR associated protein Cas6	cas6	COG1583	CAS-I,CAS-III,CAS-IV			
42961..43162	-	OAA83846.1	LITT01000046.1	Clostridium ljungdahlii	Bacteria	Firmicutes	Clostridia	Clostridiales	Clostridiaceae	Clostridium	hypothetical protein						
43230..43398	-	OAA83847.1	LITT01000046.1	Clostridium ljungdahlii	Bacteria	Firmicutes	Clostridia	Clostridiales	Clostridiaceae	Clostridium	hypothetical protein						
43854..47112	array		LITT01000046.1	Clostridium ljungdahlii	Bacteria	Firmicutes	Clostridia	Clostridiales	Clostridiaceae	Clostridium		CRISPR array			49	37	30
142 CAS-I-B | RMD02178.1																	
24813..25551	+	RMD02171.1	RFAQ01000015.1	Clostridium autoethanogenum	Bacteria	Firmicutes	Clostridia	Clostridiales	Clostridiaceae	Clostridium	CRISPR-associated endoribonuclease Cas6	cas6	COG1583	CAS-I,CAS-III,CAS-IV			
25716..27610	array		RFAQ01000015.1	Clostridium autoethanogenum	Bacteria	Firmicutes	Clostridia	Clostridiales	Clostridiaceae	Clostridium		CRISPR array			29	36	30
28130..30083	+	RMD02172.1	RFAQ01000015.1	Clostridium autoethanogenum	Bacteria	Firmicutes	Clostridia	Clostridiales	Clostridiaceae	Clostridium	TIGR02556 family CRISPR-associated protein	cas8b1	pfam09484	CAS-I-B			
30075..30951	+	RMD02173.1	RFAQ01000015.1	Clostridium autoethanogenum	Bacteria	Firmicutes	Clostridia	Clostridiales	Clostridiaceae	Clostridium	type I-B CRISPR-associated protein Cas7/Csh2	cas7	cd09690	CAS-I-B			
30961..31684	+	RMD02174.1	RFAQ01000015.1	Clostridium autoethanogenum	Bacteria	Firmicutes	Clostridia	Clostridiales	Clostridiaceae	Clostridium	type I-B CRISPR-associated protein Cas5	cas5	cls001430	CAS-I-B			
31695..34008	+	RMD02175.1	RFAQ01000015.1	Clostridium autoethanogenum	Bacteria	Firmicutes	Clostridia	Clostridiales	Clostridiaceae	Clostridium	CRISPR-associated helicase Cas3'	cas3HD,cas3	cd09641,COG1203	CAS-I			
34021..34513	+	RMD02176.1	RFAQ01000015.1	Clostridium autoethanogenum	Bacteria	Firmicutes	Clostridia	Clostridiales	Clostridiaceae	Clostridium	CRISPR-associated protein Cas4	cas4	pfam01930	CAS-I			
34522..35515	+	RMD02177.1	RFAQ01000015.1	Clostridium autoethanogenum	Bacteria	Firmicutes	Clostridia	Clostridiales	Clostridiaceae	Clostridium	type I-B CRISPR-associated endonuclease Cas1	cas1	cd09722	CAS-I-B			
35518..35782	+	RMD02178.1	RFAQ01000015.1	Clostridium autoethanogenum	Bacteria	Firmicutes	Clostridia	Clostridiales	Clostridiaceae	Clostridium	CRISPR-associated endonuclease Cas2	cas2	cd09725	CAS-I,CAS-II,CAS-III,CAS-V			
35956..37907	array		RFAQ01000015.1	Clostridium autoethanogenum	Bacteria	Firmicutes	Clostridia	Clostridiales	Clostridiaceae	Clostridium		CRISPR array			30	36	30
143 CAS-I-B | GAQ95513.1																	
401004..402633	array		BCNO01000002.1	Thermodesulfovibrio aggregans	Bacteria	Nitrospirae	Nitrospira	Nitrospirales	Nitrospiraceae	Thermodesulfovibrio		CRISPR array			25	36	30
402756..404524	array		BCNO01000002.1	Thermodesulfovibrio aggregans	Bacteria	Nitrospirae	Nitrospira	Nitrospirales	Nitrospiraceae	Thermodesulfovibrio		CRISPR array			27	36	30
404459..404543	-	GAQ95512.1	BCNO01000002.1	Thermodesulfovibrio aggregans	Bacteria	Nitrospirae	Nitrospira	Nitrospirales	Nitrospiraceae	Thermodesulfovibrio	hypothetical protein						
406706..408542	array		BCNO01000002.1	Thermodesulfovibrio aggregans	Bacteria	Nitrospirae	Nitrospira	Nitrospirales	Nitrospiraceae	Thermodesulfovibrio		CRISPR array			28	36	30
408931..409195	-	GAQ95513.1	BCNO01000002.1	Thermodesulfovibrio aggregans	Bacteria	Nitrospirae	Nitrospira	Nitrospirales	Nitrospiraceae	Thermodesulfovibrio	CRISPR-associated protein Cas2	cas2	cd09725	CAS-I,CAS-II,CAS-III,CAS-V			
409194..410187	-	GAQ95514.1	BCNO01000002.1	Thermodesulfovibrio aggregans	Bacteria	Nitrospirae	Nitrospira	Nitrospirales	Nitrospiraceae	Thermodesulfovibrio	CRISP-associated protein Cas1	cas1	cd09722	CAS-I-B			
410516..410966	-	GAQ95515.1	BCNO01000002.1	Thermodesulfovibrio aggregans	Bacteria	Nitrospirae	Nitrospira	Nitrospirales	Nitrospiraceae	Thermodesulfovibrio	CRISPR-associated exonuclease Cas4	cas4	pfam01930	CAS-I			
411031..413374	-	GAQ95516.1	BCNO01000002.1	Thermodesulfovibrio aggregans	Bacteria	Nitrospirae	Nitrospira	Nitrospirales	Nitrospiraceae	Thermodesulfovibrio	CRISPR-associated endonuclease/helicase Cas3	cas3HD,cas3	cd09641,COG1203	CAS-I			
413348..414068	-	GAQ95517.1	BCNO01000002.1	Thermodesulfovibrio aggregans	Bacteria	Nitrospirae	Nitrospira	Nitrospirales	Nitrospiraceae	Thermodesulfovibrio	CRISPR-associated protein Cas5h	cas5	cls001430	CAS-I-B			
414077..415001	-	GAQ95518.1	BCNO01000002.1	Thermodesulfovibrio aggregans	Bacteria	Nitrospirae	Nitrospira	Nitrospirales	Nitrospiraceae	Thermodesulfovibrio	CRISPR-associated protein Csh2	cas7	cd09690	CAS-I-B			
144 CAS-I | PMP93676.1																	
2333..3113	+	PMP93668.1	PNJE01000179.1	Candidatus Aminicenantes bacterium	Bacteria	Candidatus Aminicenantes					CRISPR-associated endoribonuclease Cas6	cas6	COG1583	CAS-I,CAS-III,CAS-IV			
3109..4336	+	PMP93669.1	PNJE01000179.1	Candidatus Aminicenantes bacterium	Bacteria	Candidatus Aminicenantes					type I-B CRISPR-associated protein Cas8b1/Cst1						
4307..4841	+	PMP93670.1	PNJE01000179.1	Candidatus Aminicenantes bacterium	Bacteria	Candidatus Aminicenantes					hypothetical protein	Cas11b	icity0129				
4855..5962	+	PMP93671.1	PNJE01000179.1	Candidatus Aminicenantes bacterium	Bacteria	Candidatus Aminicenantes					type I-B CRISPR-associated protein Cas7/Cst2/DevR	cas7	pfam01905	CAS-I			
6055..6880	+	PMP93672.1	PNJE01000179.1	Candidatus Aminicenantes bacterium	Bacteria	Candidatus Aminicenantes					type I-B CRISPR-associated protein Cas5	cas5	cd09658	CAS-I-B			
6876..9609	+	PMP93673.1	PNJE01000179.1	Candidatus Aminicenantes bacterium	Bacteria	Candidatus Aminicenantes					CRISPR-associated helicase/endonuclease Cas3	cas3HD,cas3	cd09641,COG1203	CAS-I			
9739..10231	+	PMP93674.1	PNJE01000179.1	Candidatus Aminicenantes bacterium	Bacteria	Candidatus Aminicenantes					CRISPR-associated protein Cas4	cas4	pfam01930	CAS-I			
10227..11220	+	PMP93675.1	PNJE01000179.1	Candidatus Aminicenantes bacterium	Bacteria	Candidatus Aminicenantes					subtype I-B CRISPR-associated endonuclease Cas1	cas1	cd09722	CAS-I-B			
11221..11485	+	PMP93676.1	PNJE01000179.1	Candidatus Aminicenantes bacterium	Bacteria	Candidatus Aminicenantes					CRISPR-associated endonuclease Cas2	cas2	cd09725	CAS-I,CAS-II,CAS-III,CAS-V			
145 Partial CAS-I-B | ROL57216.1																	
1..686	array		RCNP01000003.1	Bacteroidetes/Chlorobi group bacterium Naka2016	Bacteria							CRISPR array			11	46	20
839..1103	-	ROL57216.1	RCNP01000003.1	Bacteroidetes/Chlorobi group bacterium Naka2016	Bacteria						CRISPR-associated endonuclease Cas2	cas2	cd09725	CAS-I,CAS-II,CAS-III,CAS-V			
1102..2095	-	ROL57217.1	RCNP01000003.1	Bacteroidetes/Chlorobi group bacterium Naka2016	Bacteria						type I-B CRISPR-associated endonuclease Cas1	cas1	cd09722	CAS-I-B			
2091..2595	-	ROL57218.1	RCNP01000003.1	Bacteroidetes/Chlorobi group bacterium Naka2016	Bacteria						CRISPR-associated protein Cas4	cas4	pfam01930	CAS-I			
2628..3372	-	ROL57219.1	RCNP01000003.1	Bacteroidetes/Chlorobi group bacterium Naka2016	Bacteria						CRISPR-associated protein Cas5	cas5	cd09693	CAS-I			
3352..4324	-	ROL57220.1	RCNP01000003.1	Bacteroidetes/Chlorobi group bacterium Naka2016	Bacteria						DevR family CRISPR-associated autoregulator	cas7	pfam01905	CAS-I			
4347..5769	-	ROL57221.1	RCNP01000003.1	Bacteroidetes/Chlorobi group bacterium Naka2016	Bacteria						type I-A CRISPR-associated protein Csa5	csa5gr11	cd09653	CAS-I-A			
146 CAS-I | AIH04577.1																	
1452819..1453557	+	AIH04568.1	CP008796.1	Thermodesulfobacterium commune DSM 2178	Bacteria	Thermodesulfobacteria	Thermodesulfobacteria	Thermodesulfobacteriales	Thermodesulfobacteriaceae	Thermodesulfobacterium	hypothetical protein	cas6	COG1583	CAS-I,CAS-III,CAS-IV			
1453642..1455352	+	AIH04569.1	CP008796.1	Thermodesulfobacterium commune DSM 2178	Bacteria	Thermodesulfobacteria	Thermodesulfobacteria	Thermodesulfobacteriales	Thermodesulfobacteriaceae	Thermodesulfobacterium	hypothetical protein	cas3	COG1203	CAS-I			
1455332..1456091	+	AIH04570.1	CP008796.1	Thermodesulfobacterium commune DSM 2178	Bacteria	Thermodesulfobacteria	Thermodesulfobacteria	Thermodesulfobacteriales	Thermodesulfobacteriaceae	Thermodesulfobacterium	hypothetical protein	cas3HD	cd09641	CAS-I			
1456113..1457487	+	AIH04571.1	CP008796.1	Thermodesulfobacterium commune DSM 2178	Bacteria	Thermodesulfobacteria	Thermodesulfobacteria	Thermodesulfobacteriales	Thermodesulfobacteriaceae	Thermodesulfobacterium	hypothetical protein	csa5gr11	cd09653	CAS-I-A			
1457488..1458448	+	AIH04572.1	CP008796.1	Thermodesulfobacterium commune DSM 2178	Bacteria	Thermodesulfobacteria	Thermodesulfobacteria	Thermodesulfobacteriales	Thermodesulfobacteriaceae	Thermodesulfobacterium	hypothetical protein	cas7	pfam01905	CAS-I			
1458459..1459257	+	AIH04573.1	CP008796.1	Thermodesulfobacterium commune DSM 2178	Bacteria	Thermodesulfobacteria	Thermodesulfobacteria	Thermodesulfobacteriales	Thermodesulfobacteriaceae	Thermodesulfobacterium	hypothetical protein	cas5	cd09693	CAS-I			
1459580..1459874	+	AIH04574.1	CP008796.1	Thermodesulfobacterium commune DSM 2178	Bacteria	Thermodesulfobacteria	Thermodesulfobacteria	Thermodesulfobacteriales	Thermodesulfobacteriaceae	Thermodesulfobacterium	hypothetical protein						
1459876..1460371	+	AIH04575.1	CP008796.1	Thermodesulfobacterium commune DSM 2178	Bacteria	Thermodesulfobacteria	Thermodesulfobacteria	Thermodesulfobacteriales	Thermodesulfobacteriaceae	Thermodesulfobacterium	CRISPR-associated protein Cas4	cas4	pfam01930	CAS-I			
1460367..1461360	+	AIH04576.1	CP008796.1	Thermodesulfobacterium commune DSM 2178	Bacteria	Thermodesulfobacteria	Thermodesulfobacteria	Thermodesulfobacteriales	Thermodesulfobacteriaceae	Thermodesulfobacterium	CRISPR-associated protein Cas1	cas1	cd09722	CAS-I-B			
1461363..1461627	+	AIH04577.1	CP008796.1	Thermodesulfobacterium commune DSM 2178	Bacteria	Thermodesulfobacteria	Thermodesulfobacteria	Thermodesulfobacteriales	Thermodesulfobacteriaceae	Thermodesulfobacterium	CRISPR-associated protein Cas2	cas2	cd09725	CAS-I,CAS-II,CAS-III,CAS-V			
1461789..1463221	array		CP008796.1	Thermodesulfobacterium commune DSM 2178	Bacteria	Thermodesulfobacteria	Thermodesulfobacteria	Thermodesulfobacteriales	Thermodesulfobacteriaceae	Thermodesulfobacterium		CRISPR array			22	36	30
147 partial CAS-I-B | PMP82994.1																	
0..767	+	PMP82993.1	PNIX01000151.1	Caldisericum exile	Bacteria	Caldiserica	Caldisericia	Caldisericales	Caldisericaceae	Caldisericum	subtype I-B CRISPR-associated endonuclease Cas1	cas1	cd09722	CAS-I-B			
767..1031	+	PMP82994.1	PNIX01000151.1	Caldisericum exile	Bacteria	Caldiserica	Caldisericia	Caldisericales	Caldisericaceae	Caldisericum	CRISPR-associated endonuclease Cas2	cas2	cd09725	CAS-I,CAS-II,CAS-III,CAS-V			
148 CAS-I-B | BAT71822.1																	
1052392..1057198	array		AP013035.1	Thermosulfidibacter takaii ABI70S6	Bacteria	Aquificae	Aquificae	Aquificales		Thermosulfidibacter		CRISPR array			73	36	30
1057333..1057600	-	BAT71822.1	AP013035.1	Thermosulfidibacter takaii ABI70S6	Bacteria	Aquificae	Aquificae	Aquificales		Thermosulfidibacter	CRISPR-associated protein Cas2	cas2	cd09725	CAS-I,CAS-II,CAS-III,CAS-V			
1057603..1058596	-	BAT71823.1	AP013035.1	Thermosulfidibacter takaii ABI70S6	Bacteria	Aquificae	Aquificae	Aquificales		Thermosulfidibacter	CRISPR-associated protein Cas1	cas1	cd09722	CAS-I-B			
1058599..1059091	-	BAT71824.1	AP013035.1	Thermosulfidibacter takaii ABI70S6	Bacteria	Aquificae	Aquificae	Aquificales		Thermosulfidibacter	CRISPR-associated protein Cas4	cas4	pfam01930	CAS-I			
1059146..1061783	-	BAT71825.1	AP013035.1	Thermosulfidibacter takaii ABI70S6	Bacteria	Aquificae	Aquificae	Aquificales		Thermosulfidibacter	CRISPR-associated protein Cas3	cas3HD,cas3	cd09641,COG1203	CAS-I			
1061773..1062604	-	BAT71826.1	AP013035.1	Thermosulfidibacter takaii ABI70S6	Bacteria	Aquificae	Aquificae	Aquificales		Thermosulfidibacter	CRISPR-associated protein Cas5	cas5	cd09658	CAS-I-B			
1062615..1063635	-	BAT71827.1	AP013035.1	Thermosulfidibacter takaii ABI70S6	Bacteria	Aquificae	Aquificae	Aquificales		Thermosulfidibacter	CRISPR-associated regulatory protein, DevR family	cas7	cd09687	CAS-I-B			
1063636..1063945	-	BAT71828.1	AP013035.1	Thermosulfidibacter takaii ABI70S6	Bacteria	Aquificae	Aquificae	Aquificales		Thermosulfidibacter	conserved hypothetical protein	cas11b	mkCas0150	CAS-I-B			
1063937..1065767	-	BAT71829.1	AP013035.1	Thermosulfidibacter takaii ABI70S6	Bacteria	Aquificae	Aquificae	Aquificales		Thermosulfidibacter	conserved hypothetical protein	cas8b10	mkCas0151	CAS-I-B			
1065759..1066551	-	BAT71830.1	AP013035.1	Thermosulfidibacter takaii ABI70S6	Bacteria	Aquificae	Aquificae	Aquificales		Thermosulfidibacter	CRISPR-associated protein Cas6	cas6	COG1583	CAS-I,CAS-III,CAS-IV			
149 partial CAS-I-B | PIP11833.1																	
338..602	-	PIP11833.1	PCRC01000326.1	bacterium (Candidatus Stahlbacteria) CG23_combo_of_CG06-09_8_20_14_all_40_9	Bacteria						CRISPR-associated endonuclease Cas2	cas2	COG1343	CAS-I,CAS-II,CAS-III			
672..1029	-	PIP11834.1	PCRC01000326.1	bacterium (Candidatus Stahlbacteria) CG23_combo_of_CG06-09_8_20_14_all_40_9	Bacteria						hypothetical protein						
1094..2087	-	PIP11835.1	PCRC01000326.1	bacterium (Candidatus Stahlbacteria) CG23_combo_of_CG06-09_8_20_14_all_40_9	Bacteria						subtype I-B CRISPR-associated endonuclease Cas1	cas1	cd09722	CAS-I-B			
150 CAS-I-B | EFC90514.1																	
477409..479683	array		ABTR02000001.1	Dethiosulfovibrio peptidovorans DSM 11002	Bacteria	Synergistetes	Synergistia	Synergistales	Synergistaceae	Dethiosulfovibrio		CRISPR array			35	36	30
479402..479726	-	EFC90513.1	ABTR02000001.1	Dethiosulfovibrio peptidovorans DSM 11002	Bacteria	Synergistetes	Synergistia	Synergistales	Synergistaceae	Dethiosulfovibrio	hypothetical protein						
479896..480160	-	EFC90514.1	ABTR02000001.1	Dethiosulfovibrio peptidovorans DSM 11002	Bacteria	Synergistetes	Synergistia	Synergistales	Synergistaceae	Dethiosulfovibrio	CRISPR-associated protein Cas2	cas2	cd09725	CAS-I,CAS-II,CAS-III,CAS-V			
480166..481165	-	EFC90515.1	ABTR02000001.1	Dethiosulfovibrio peptidovorans DSM 11002	Bacteria	Synergistetes	Synergistia	Synergistales	Synergistaceae	Dethiosulfovibrio	CRISPR-associated protein Cas1	cas1	cd09722	CAS-I-B			
481169..481688	-	EFC90516.1	ABTR02000001.1	Dethiosulfovibrio peptidovorans DSM 11002	Bacteria	Synergistetes	Synergistia	Synergistales	Synergistaceae	Dethiosulfovibrio	CRISPR-associated protein Cas4	cas4	pfam01930	CAS-I			
481665..483804	-	EFC90517.1	ABTR02000001.1	Dethiosulfovibrio peptidovorans DSM 11002	Bacteria	Synergistetes	Synergistia	Synergistales	Synergistaceae	Dethiosulfovibrio	CRISPR-associated helicase Cas3	cas3HD,cas3	cd09641,COG1203	CAS-I			
483859..484570	-	EFC90518.1	ABTR02000001.1	Dethiosulfovibrio peptidovorans DSM 11002	Bacteria	Synergistetes	Synergistia	Synergistales	Synergistaceae	Dethiosulfovibrio	CRISPR-associated protein Cas5, Hmari subtype	cas5	cls001430	CAS-I-B			
484574..485483	-	EFC90519.1	ABTR02000001.1	Dethiosulfovibrio peptidovorans DSM 11002	Bacteria	Synergistetes	Synergistia	Synergistales	Synergistaceae	Dethiosulfovibrio	CRISPR-associated protein, Csh2 family	cas7	cd09690	CAS-I-B			
485484..485916	-	EFC90520.1	ABTR02000001.1	Dethiosulfovibrio peptidovorans DSM 11002	Bacteria	Synergistetes	Synergistia	Synergistales	Synergistaceae	Dethiosulfovibrio	conserved hypothetical protein	cas8b1	mkCas0119	CAS-I-B			
485921..487418	-	EFC90521.1	ABTR02000001.1	Dethiosulfovibrio peptidovorans DSM 11002	Bacteria	Synergistetes	Synergistia	Synergistales	Synergistaceae	Dethiosulfovibrio	conserved hypothetical protein	cas8b1	cd09730	CAS-I-B			
487449..488208	-	EFC90522.1	ABTR02000001.1	Dethiosulfovibrio peptidovorans DSM 11002	Bacteria	Synergistetes	Synergistia	Synergistales	Synergistaceae	Dethiosulfovibrio	CRISPR-associated protein Cas6	cas6	cd09652	CAS-I,CAS-III			
152 CAS-I-B | AUG56929.1																	
1088863..1099162	array		CP025197.1	Hungateiclostridium saccincola	Bacteria	Firmicutes	Clostridia	Clostridiales	Hungateiclostridiaceae	Hungateiclostridium		CRISPR array			155	36	30
1099427..1099691	-	AUG56929.1	CP025197.1	Hungateiclostridium saccincola	Bacteria	Firmicutes	Clostridia	Clostridiales	Hungateiclostridiaceae	Hungateiclostridium	CRISPR-associated endoribonuclease Cas2	cas2	cd09725	CAS-I,CAS-II,CAS-III,CAS-V			
1099668..1099899	-	AUG56930.1	CP025197.1	Hungateiclostridium saccincola	Bacteria	Firmicutes	Clostridia	Clostridiales	Hungateiclostridiaceae	Hungateiclostridium	hypothetical protein						
1099892..1100885	-	AUG56931.1	CP025197.1	Hungateiclostridium saccincola	Bacteria	Firmicutes	Clostridia	Clostridiales	Hungateiclostridiaceae	Hungateiclostridium	CRISPR-associated endonuclease Cas1	cas1	cd09722	CAS-I-B			
1100907..1101405	-	AUG56932.1	CP025197.1	Hungateiclostridium saccincola	Bacteria	Firmicutes	Clostridia	Clostridiales	Hungateiclostridiaceae	Hungateiclostridium	PD-(D/E)XK nuclease superfamily protein	cas4	pfam01930	CAS-I			
1101566..1103834	-	AUG56933.1	CP025197.1	Hungateiclostridium saccincola	Bacteria	Firmicutes	Clostridia	Clostridiales	Hungateiclostridiaceae	Hungateiclostridium	CRISPR-associated endonuclease/helicase Cas3	cas3	COG1203	CAS-I			
1103865..1104588	-	AUG56934.1	CP025197.1	Hungateiclostridium saccincola	Bacteria	Firmicutes	Clostridia	Clostridiales	Hungateiclostridiaceae	Hungateiclostridium	CRISPR-associated protein	cas5	cd09658	CAS-I-B			
1104605..1105487	-	AUG56935.1	CP025197.1	Hungateiclostridium saccincola	Bacteria	Firmicutes	Clostridia	Clostridiales	Hungateiclostridiaceae	Hungateiclostridium	CRISPR-associated protein Cas7/Cst2/DevR	cas7	cd09687	CAS-I-B			
1105490..1107155	-	AUG56936.1	CP025197.1	Hungateiclostridium saccincola	Bacteria	Firmicutes	Clostridia	Clostridiales	Hungateiclostridiaceae	Hungateiclostridium	hypothetical protein	cas8b1	mkCas0195	CAS-I-B			
1107170..1107890	-	AUG56937.1	CP025197.1	Hungateiclostridium saccincola	Bacteria	Firmicutes	Clostridia	Clostridiales	Hungateiclostridiaceae	Hungateiclostridium	CRISPR associated protein Cas6	cas6	COG1583	CAS-I,CAS-III,CAS-IV			
153 CAS-I-B | PQQ68312.1																	
2076927..2083770	array		NEMB01000003.1	Hungateiclostridium saccincola	Bacteria	Firmicutes	Clostridia	Clostridiales	Hungateiclostridiaceae	Hungateiclostridium		CRISPR array			103	36	30
2084035..2084299	-	PQQ68312.1	NEMB01000003.1	Hungateiclostridium saccincola	Bacteria	Firmicutes	Clostridia	Clostridiales	Hungateiclostridiaceae	Hungateiclostridium	CRISPR-associated endonuclease Cas2	cas2	cd09725	CAS-I,CAS-II,CAS-III,CAS-V			
2084499..2085492	-	PQQ66955.1	NEMB01000003.1	Hungateiclostridium saccincola	Bacteria	Firmicutes	Clostridia	Clostridiales	Hungateiclostridiaceae	Hungateiclostridium	subtype I-B CRISPR-associated endonuclease Cas1	cas1	cd09722	CAS-I-B			
2085514..2086012	-	PQQ66956.1	NEMB01000003.1	Hungateiclostridium saccincola	Bacteria	Firmicutes	Clostridia	Clostridiales	Hungateiclostridiaceae	Hungateiclostridium	CRISPR-associated protein Cas4	cas4	pfam01930	CAS-I			
2086173..2088441	-	PQQ66957.1	NEMB01000003.1	Hungateiclostridium saccincola	Bacteria	Firmicutes	Clostridia	Clostridiales	Hungateiclostridiaceae	Hungateiclostridium	CRISPR-associated helicase/endonuclease Cas3	cas3	COG1203	CAS-I			
2088472..2089195	-	PQQ66958.1	NEMB01000003.1	Hungateiclostridium saccincola	Bacteria	Firmicutes	Clostridia	Clostridiales	Hungateiclostridiaceae	Hungateiclostridium	CRISPR-associated protein Cas5	cas5	cd09658	CAS-I-B			
2089212..2090094	-	PQQ66959.1	NEMB01000003.1	Hungateiclostridium saccincola	Bacteria	Firmicutes	Clostridia	Clostridiales	Hungateiclostridiaceae	Hungateiclostridium	type I-B CRISPR-associated protein Cas7/Cst2/DevR	cas7	cd09687	CAS-I-B			
2090097..2091762	-	PQQ66960.1	NEMB01000003.1	Hungateiclostridium saccincola	Bacteria	Firmicutes	Clostridia	Clostridiales	Hungateiclostridiaceae	Hungateiclostridium	hypothetical protein	cas8b1	mkCas0195	CAS-I-B			
2091777..2092497	-	PQQ66961.1	NEMB01000003.1	Hungateiclostridium saccincola	Bacteria	Firmicutes	Clostridia	Clostridiales	Hungateiclostridiaceae	Hungateiclostridium	CRISPR-associated endoribonuclease Cas6	cas6	COG1583	CAS-I,CAS-III,CAS-IV			
2092958..2094407	+	PQQ66962.1	NEMB01000003.1	Hungateiclostridium saccincola	Bacteria	Firmicutes	Clostridia	Clostridiales	Hungateiclostridiaceae	Hungateiclostridium	hypothetical protein	cas10	mkCas0133	CAS-III			
154 CAS-I-B | ABN53498.1																	
2729745..2741081	array		CP000568.1	Hungateiclostridium thermocellum ATCC 27405	Bacteria	Firmicutes	Clostridia	Clostridiales	Hungateiclostridiaceae	Hungateiclostridium		CRISPR array			169	38	29
2741247..2741511	-	ABN53498.1	CP000568.1	Hungateiclostridium thermocellum ATCC 27405	Bacteria	Firmicutes	Clostridia	Clostridiales	Hungateiclostridiaceae	Hungateiclostridium	CRISPR-associated protein Cas2	cas2	cd09725	CAS-I,CAS-II,CAS-III,CAS-V			
2741524..2742517	-	ABN53499.1	CP000568.1	Hungateiclostridium thermocellum ATCC 27405	Bacteria	Firmicutes	Clostridia	Clostridiales	Hungateiclostridiaceae	Hungateiclostridium	CRISPR-associated protein Cas1	cas1	cd09722	CAS-I-B			
2742530..2743043	-	ABN53500.1	CP000568.1	Hungateiclostridium thermocellum ATCC 27405	Bacteria	Firmicutes	Clostridia	Clostridiales	Hungateiclostridiaceae	Hungateiclostridium	CRISPR-associated protein Cas4	cas4	pfam01930	CAS-I			
2743055..2745308	-	ABN53501.1	CP000568.1	Hungateiclostridium thermocellum ATCC 27405	Bacteria	Firmicutes	Clostridia	Clostridiales	Hungateiclostridiaceae	Hungateiclostridium	CRISPR-associated helicase Cas3	cas3HD,cas3	cd09641,COG1203	CAS-I			
2745329..2746055	-	ABN53502.1	CP000568.1	Hungateiclostridium thermocellum ATCC 27405	Bacteria	Firmicutes	Clostridia	Clostridiales	Hungateiclostridiaceae	Hungateiclostridium	CRISPR-associated protein Cas5	cas5	cd09693	CAS-I			
2746073..2746958	-	ABN53503.1	CP000568.1	Hungateiclostridium thermocellum ATCC 27405	Bacteria	Firmicutes	Clostridia	Clostridiales	Hungateiclostridiaceae	Hungateiclostridium	CRISPR-associated regulatory protein, DevR family	cas7	cd09687	CAS-I-B			
2746963..2748640	-	ABN53504.1	CP000568.1	Hungateiclostridium thermocellum ATCC 27405	Bacteria	Firmicutes	Clostridia	Clostridiales	Hungateiclostridiaceae	Hungateiclostridium	hypothetical protein	cas8b1	mkCas0195	CAS-I-B			
2748651..2749374	-	ABN53505.1	CP000568.1	Hungateiclostridium thermocellum ATCC 27405	Bacteria	Firmicutes	Clostridia	Clostridiales	Hungateiclostridiaceae	Hungateiclostridium	CRISPR-associated protein Cas6	cas6	COG1583	CAS-I,CAS-III,CAS-IV			
155 CAS-I-B | GAE87583.1																	

25382..29375	array		BAVR01000008.1	Hungateiclostridium straminisolvens JCM 21531	Bacteria	Firmicutes	Clostridia	Clostridiales	Hungateiclostridiaceae	Hungateiclostridium		CRISPR array			60	37	30
29539..29803	-	GAE87583.1	BAVR01000008.1	Hungateiclostridium straminisolvens JCM 21531	Bacteria	Firmicutes	Clostridia	Clostridiales	Hungateiclostridiaceae	Hungateiclostridium	CRISPR-associated protein Cas2	**cas2**	cd09725	CAS-I,CAS-II,CAS-III,CAS-V			
29816..30809	-	GAE87584.1	BAVR01000008.1	Hungateiclostridium straminisolvens JCM 21531	Bacteria	Firmicutes	Clostridia	Clostridiales	Hungateiclostridiaceae	Hungateiclostridium	CRISPR-associated protein Cas1	cas1	cd09722	CAS-I-B			
30824..31331	-	GAE87585.1	BAVR01000008.1	Hungateiclostridium straminisolvens JCM 21531	Bacteria	Firmicutes	Clostridia	Clostridiales	Hungateiclostridiaceae	Hungateiclostridium	CRISPR-associated RecB family exonuclease Cas4a	cas4	pfam01930	CAS-I			
31349..32354	-	GAE87586.1	BAVR01000008.1	Hungateiclostridium straminisolvens JCM 21531	Bacteria	Firmicutes	Clostridia	Clostridiales	Hungateiclostridiaceae	Hungateiclostridium	CRISPR-associated helicase Cas3	cas3	COG1203	CAS-I			
32395..33601	-	GAE87587.1	BAVR01000008.1	Hungateiclostridium straminisolvens JCM 21531	Bacteria	Firmicutes	Clostridia	Clostridiales	Hungateiclostridiaceae	Hungateiclostridium	CRISPR-associated helicase Cas3	cas3HD,cas3	cd09641,cd09639	CAS-I			
33615..33960	-	GAE87588.1	BAVR01000008.1	Hungateiclostridium straminisolvens JCM 21531	Bacteria	Firmicutes	Clostridia	Clostridiales	Hungateiclostridiaceae	Hungateiclostridium	hypothetical protein						
34004..34340	-	GAE87589.1	BAVR01000008.1	Hungateiclostridium straminisolvens JCM 21531	Bacteria	Firmicutes	Clostridia	Clostridiales	Hungateiclostridiaceae	Hungateiclostridium	hypothetical protein	cas5	cd09693	CAS-I			
34358..35243	-	GAE87590.1	BAVR01000008.1	Hungateiclostridium straminisolvens JCM 21531	Bacteria	Firmicutes	Clostridia	Clostridiales	Hungateiclostridiaceae	Hungateiclostridium	CRISPR-associated negative autoregulator	cas7	cd09687	CAS-I-B			
35248..36763	-	GAE87591.1	BAVR01000008.1	Hungateiclostridium straminisolvens JCM 21531	Bacteria	Firmicutes	Clostridia	Clostridiales	Hungateiclostridiaceae	Hungateiclostridium	hypothetical protein	cas8b1	mkCas0195	CAS-I-B			
36932..37376	-	GAE87592.1	BAVR01000008.1	Hungateiclostridium straminisolvens JCM 21531	Bacteria	Firmicutes	Clostridia	Clostridiales	Hungateiclostridiaceae	Hungateiclostridium	CRISPR repeat RNA endoribonuclease Cas6	cas6	pfam01881	CAS-I,CAS-III			
37357..37654	-	GAE87593.1	BAVR01000008.1	Hungateiclostridium straminisolvens JCM 21531	Bacteria	Firmicutes	Clostridia	Clostridiales	Hungateiclostridiaceae	Hungateiclostridium	CRISPR repeat RNA endoribonuclease Cas6	cas6	COG1583	CAS-I,CAS-III,CAS-IV			
156 CAS-I-B | PMP68059.1																	
4458..5241	+	PMP68052.1	PNIL01000029.1	Caldisericum exile	Bacteria	Caldiserica	Caldisericia	Caldisericales	Caldisericaceae	Caldisericum	CRISPR-associated endoribonuclease Cas6	cas6	COG1583	CAS-I,CAS-III,CAS-IV			
5261..7046	+	PMP68053.1	PNIL01000029.1	Caldisericum exile	Bacteria	Caldiserica	Caldisericia	Caldisericales	Caldisericaceae	Caldisericum	TIGR02556 family CRISPR-associated protein	cas8b1	cd09730	CAS-I-B			
7032..7998	+	PMP68054.1	PNIL01000029.1	Caldisericum exile	Bacteria	Caldiserica	Caldisericia	Caldisericales	Caldisericaceae	Caldisericum	type I-B CRISPR-associated protein Cas7/Csh2	cas7	cd09690	CAS-I-B			
8006..8723	+	PMP68055.1	PNIL01000029.1	Caldisericum exile	Bacteria	Caldiserica	Caldisericia	Caldisericales	Caldisericaceae	Caldisericum	type I-B CRISPR-associated protein Cas5	cas5	cls001430	CAS-I-B			
8676..11076	+	PMP68056.1	PNIL01000029.1	Caldisericum exile	Bacteria	Caldiserica	Caldisericia	Caldisericales	Caldisericaceae	Caldisericum	CRISPR-associated helicase/endonuclease Cas3	cas3HD,cas3	cd09641,COG1203	CAS-I			
11069..11573	+	PMP68057.1	PNIL01000029.1	Caldisericum exile	Bacteria	Caldiserica	Caldisericia	Caldisericales	Caldisericaceae	Caldisericum	CRISPR-associated protein Cas4	cas4	pfam01930	CAS-I			
11569..12562	+	PMP68058.1	PNIL01000029.1	Caldisericum exile	Bacteria	Caldiserica	Caldisericia	Caldisericales	Caldisericaceae	Caldisericum	subtype I-B CRISPR-associated endonuclease Cas1	cas1	cd09722	CAS-I-B			
12562..12826	+	PMP68059.1	PNIL01000029.1	Caldisericum exile	Bacteria	Caldiserica	Caldisericia	Caldisericales	Caldisericaceae	Caldisericum	CRISPR-associated endonuclease Cas2	**cas2**	COG1343	CAS-I,CAS-II,CAS-III			
157 partial CAS-I/CAS-II | ACI17783.1																	
1276962..1277226	-	ACI17783.1	CP001145.1	Coprothermobacter proteolyticus DSM 5265	Bacteria	Coprothermobacterota	Coprothermobacteria	Coprothermobacterales	Coprothermobacteraceae	Coprothermobacter	CRISPR-associated protein Cas2	**cas2**	COG1343	CAS-I,CAS-II,CAS-III			
159 CAS-I-B | OIP13913.1																	
13883..14669	+	OIP13906.1	MNXV01000011.1	Caldisericum sp. CG2_30_36_11	Bacteria	Caldiserica	Caldisericia	Caldisericales	Caldisericaceae	Caldisericum	CRISPR-associated endoribonuclease Cas6	cas6	cd09759	CAS-I-A			
14670..16488	+	OIP13907.1	MNXV01000011.1	Caldisericum sp. CG2_30_36_11	Bacteria	Caldiserica	Caldisericia	Caldisericales	Caldisericaceae	Caldisericum	type I-B CRISPR-associated protein Cas8b/Csh1	cas8b1	cd09730	CAS-I-B			
16484..17462	+	OIP13908.1	MNXV01000011.1	Caldisericum sp. CG2_30_36_11	Bacteria	Caldiserica	Caldisericia	Caldisericales	Caldisericaceae	Caldisericum	type I-B CRISPR-associated protein Cas7/Csh2	cas7	cd09690	CAS-I-B			
17463..18186	+	OIP13909.1	MNXV01000011.1	Caldisericum sp. CG2_30_36_11	Bacteria	Caldiserica	Caldisericia	Caldisericales	Caldisericaceae	Caldisericum	type I-B CRISPR-associated protein Cas5	cas5	cls001430	CAS-I-B			
18160..20530	+	OIP13910.1	MNXV01000011.1	Caldisericum sp. CG2_30_36_11	Bacteria	Caldiserica	Caldisericia	Caldisericales	Caldisericaceae	Caldisericum	CRISPR-associated helicase/endonuclease Cas3	cas3HD,cas3	cd09641,COG1203	CAS-I			
20523..21021	+	OIP13911.1	MNXV01000011.1	Caldisericum sp. CG2_30_36_11	Bacteria	Caldiserica	Caldisericia	Caldisericales	Caldisericaceae	Caldisericum	CRISPR-associated protein Cas4	cas4	pfam01930	CAS-I			
21017..22013	+	OIP13912.1	MNXV01000011.1	Caldisericum sp. CG2_30_36_11	Bacteria	Caldiserica	Caldisericia	Caldisericales	Caldisericaceae	Caldisericum	subtype I-B CRISPR-associated endonuclease Cas1	cas1	cd09722	CAS-I-B			
22205..22469	+	OIP13913.1	MNXV01000011.1	Caldisericum sp. CG2_30_36_11	Bacteria	Caldiserica	Caldisericia	Caldisericales	Caldisericaceae	Caldisericum	CRISPR-associated endonuclease Cas2	**cas2**	cd09725	CAS-I,CAS-II,CAS-III,CAS-V			
160 CAS-I-B | PMQ02055.1																	
164572..165358	+	PMQ02048.1	NIRF01000002.1	Dictyoglomus sp. NZ13-RE01	Bacteria	Dictyoglomi	Dictyoglomia	Dictyoglomales	Dictyoglomaceae	Dictyoglomus	CRISPR-associated endoribonuclease Cas6	cas6	COG1583	CAS-I,CAS-III,CAS-IV			
165342..167319	+	PMQ02049.1	NIRF01000002.1	Dictyoglomus sp. NZ13-RE01	Bacteria	Dictyoglomi	Dictyoglomia	Dictyoglomales	Dictyoglomaceae	Dictyoglomus	hypothetical protein	cas8b1	cd09730	CAS-I-B			
167319..168261	+	PMQ02050.1	NIRF01000002.1	Dictyoglomus sp. NZ13-RE01	Bacteria	Dictyoglomi	Dictyoglomia	Dictyoglomales	Dictyoglomaceae	Dictyoglomus	type I-B CRISPR-associated protein Cas7/Csh2	cas7	cd09690	CAS-I-B			
168275..169022	+	PMQ02051.1	NIRF01000002.1	Dictyoglomus sp. NZ13-RE01	Bacteria	Dictyoglomi	Dictyoglomia	Dictyoglomales	Dictyoglomaceae	Dictyoglomus	type I-B CRISPR-associated protein Cas5	cas5	cd09692	CAS-I-B			
169023..171390	+	PMQ02052.1	NIRF01000002.1	Dictyoglomus sp. NZ13-RE01	Bacteria	Dictyoglomi	Dictyoglomia	Dictyoglomales	Dictyoglomaceae	Dictyoglomus	CRISPR-associated helicase/endonuclease Cas3	cas3HD,cas3	cd09641,COG1203	CAS-I			
171386..171881	+	PMQ02053.1	NIRF01000002.1	Dictyoglomus sp. NZ13-RE01	Bacteria	Dictyoglomi	Dictyoglomia	Dictyoglomales	Dictyoglomaceae	Dictyoglomus	CRISPR-associated protein Cas4	cas4	pfam01930	CAS-I			
171877..172873	+	PMQ02054.1	NIRF01000002.1	Dictyoglomus sp. NZ13-RE01	Bacteria	Dictyoglomi	Dictyoglomia	Dictyoglomales	Dictyoglomaceae	Dictyoglomus	subtype I-B CRISPR-associated endonuclease Cas1	cas1	cd09722	CAS-I-B			
172876..173143	+	PMQ02055.1	NIRF01000002.1	Dictyoglomus sp. NZ13-RE01	Bacteria	Dictyoglomi	Dictyoglomia	Dictyoglomales	Dictyoglomaceae	Dictyoglomus	CRISPR-associated endonuclease Cas2	**cas2**	COG1343	CAS-I,CAS-II,CAS-III			
173263..175087	array		NIRF01000002.1	Dictyoglomus sp. NZ13-RE01	Bacteria	Dictyoglomi	Dictyoglomia	Dictyoglomales	Dictyoglomaceae	Dictyoglomus		CRISPR array			28	36	30
161 CAS-I-B | ACI19268.1																	
168677..169376	+	ACI20137.1	CP001146.1	Dictyoglomus thermophilum H-6-12	Bacteria	Dictyoglomi	Dictyoglomia	Dictyoglomales	Dictyoglomaceae	Dictyoglomus	GntR family transcriptional regulator	WYL	pfam13280	CAS-I,CAS-III			
169448..170231	+	ACI18721.1	CP001146.1	Dictyoglomus thermophilum H-6-12	Bacteria	Dictyoglomi	Dictyoglomia	Dictyoglomales	Dictyoglomaceae	Dictyoglomus	CRISPR-associated protein Cas6	cas6	COG1583	CAS-I,CAS-III,CAS-IV			
170220..172185	+	ACI19298.1	CP001146.1	Dictyoglomus thermophilum H-6-12	Bacteria	Dictyoglomi	Dictyoglomia	Dictyoglomales	Dictyoglomaceae	Dictyoglomus	CRISPR-associated protein, Csh1 family	cas8b1	cd09672	CAS-I-B			
172185..173139	+	ACI19906.1	CP001146.1	Dictyoglomus thermophilum H-6-12	Bacteria	Dictyoglomi	Dictyoglomia	Dictyoglomales	Dictyoglomaceae	Dictyoglomus	CRISPR-associated protein, Csh2 family	cas7b	pfam05107	CAS-I-B,CAS-I-C			
173155..173908	+	ACI18906.1	CP001146.1	Dictyoglomus thermophilum H-6-12	Bacteria	Dictyoglomi	Dictyoglomia	Dictyoglomales	Dictyoglomaceae	Dictyoglomus	CRISPR-associated protein Cas5, Hmari subtype	cas5	cd09692	CAS-I-B			
173909..176315	+	ACI18894.1	CP001146.1	Dictyoglomus thermophilum H-6-12	Bacteria	Dictyoglomi	Dictyoglomia	Dictyoglomales	Dictyoglomaceae	Dictyoglomus	CRISPR-associated helicase Cas3	cas3HD,cas3	cd09641,COG1203	CAS-I			
176331..176826	+	ACI19470.1	CP001146.1	Dictyoglomus thermophilum H-6-12	Bacteria	Dictyoglomi	Dictyoglomia	Dictyoglomales	Dictyoglomaceae	Dictyoglomus	CRISPR-associated protein Cas4	cas4	pfam01930	CAS-I			
176822..177818	+	ACI18336.1	CP001146.1	Dictyoglomus thermophilum H-6-12	Bacteria	Dictyoglomi	Dictyoglomia	Dictyoglomales	Dictyoglomaceae	Dictyoglomus	CRISPR-associated protein Cas1, HMARI/TNEAP subtype	cas1	cd09722	CAS-I-B			
177819..178083	+	ACI19268.1	CP001146.1	Dictyoglomus thermophilum H-6-12	Bacteria	Dictyoglomi	Dictyoglomia	Dictyoglomales	Dictyoglomaceae	Dictyoglomus	CRISPR-associated protein Cas2	**cas2**	cd09725	CAS-I,CAS-II,CAS-III,CAS-V			
178426..182925	array		CP001146.1	Dictyoglomus thermophilum H-6-12	Bacteria	Dictyoglomi	Dictyoglomia	Dictyoglomales	Dictyoglomaceae	Dictyoglomus		CRISPR array			68	36	30
162 CAS-I-B | ACK41906.1																	
615153..617311	array		CP001251.1	Dictyoglomus turgidum DSM 6724	Bacteria	Dictyoglomi	Dictyoglomia	Dictyoglomales	Dictyoglomaceae	Dictyoglomus		CRISPR array			33	36	30
617728..618106	+	ACK41902.1	CP001251.1	Dictyoglomus turgidum DSM 6724	Bacteria	Dictyoglomi	Dictyoglomia	Dictyoglomales	Dictyoglomaceae	Dictyoglomus	queuosine biosynthesis protein QueD						
618086..618860	+	ACK41903.1	CP001251.1	Dictyoglomus turgidum DSM 6724	Bacteria	Dictyoglomi	Dictyoglomia	Dictyoglomales	Dictyoglomaceae	Dictyoglomus	protein of unknown function DUF198						
618856..620059	+	ACK41904.1	CP001251.1	Dictyoglomus turgidum DSM 6724	Bacteria	Dictyoglomi	Dictyoglomia	Dictyoglomales	Dictyoglomaceae	Dictyoglomus	dihydropteroate synthase						
620034..620502	+	ACK41905.1	CP001251.1	Dictyoglomus turgidum DSM 6724	Bacteria	Dictyoglomi	Dictyoglomia	Dictyoglomales	Dictyoglomaceae	Dictyoglomus	2-amino-4-hydroxy-6- hydroxymethyldihydropteridine pyrophosphokinase						
620514..620778	-	ACK41906.1	CP001251.1	Dictyoglomus turgidum DSM 6724	Bacteria	Dictyoglomi	Dictyoglomia	Dictyoglomales	Dictyoglomaceae	Dictyoglomus	CRISPR-associated protein Cas2	**cas2**	cd09725	CAS-I,CAS-II,CAS-III,CAS-V			
620787..621783	-	ACK41907.1	CP001251.1	Dictyoglomus turgidum DSM 6724	Bacteria	Dictyoglomi	Dictyoglomia	Dictyoglomales	Dictyoglomaceae	Dictyoglomus	CRISPR-associated protein Cas1	cas1	cd09722	CAS-I-B			
621779..622286	-	ACK41908.1	CP001251.1	Dictyoglomus turgidum DSM 6724	Bacteria	Dictyoglomi	Dictyoglomia	Dictyoglomales	Dictyoglomaceae	Dictyoglomus	CRISPR-associated protein Cas4	cas4	pfam01930	CAS-I			
622279..624634	-	ACK41909.1	CP001251.1	Dictyoglomus turgidum DSM 6724	Bacteria	Dictyoglomi	Dictyoglomia	Dictyoglomales	Dictyoglomaceae	Dictyoglomus	CRISPR-associated helicase Cas3	cas3HD,cas3	cd09641,COG1203	CAS-I			
624617..625334	-	ACK41910.1	CP001251.1	Dictyoglomus turgidum DSM 6724	Bacteria	Dictyoglomi	Dictyoglomia	Dictyoglomales	Dictyoglomaceae	Dictyoglomus	CRISPR-associated protein Cas5, Hmari subtype	cas5	cls001430	CAS-I-B			
625347..626259	-	ACK41911.1	CP001251.1	Dictyoglomus turgidum DSM 6724	Bacteria	Dictyoglomi	Dictyoglomia	Dictyoglomales	Dictyoglomaceae	Dictyoglomus	CRISPR-associated protein, Csh2 family	cas7	cd09690	CAS-I-B			
626272..628030	-	ACK41912.1	CP001251.1	Dictyoglomus turgidum DSM 6724	Bacteria	Dictyoglomi	Dictyoglomia	Dictyoglomales	Dictyoglomaceae	Dictyoglomus	CRISPR-associated protein, TM1802 family	cas8b1	cd09730	CAS-I-B			
628022..628793	-	ACK41913.1	CP001251.1	Dictyoglomus turgidum DSM 6724	Bacteria	Dictyoglomi	Dictyoglomia	Dictyoglomales	Dictyoglomaceae	Dictyoglomus	CRISPR-associated protein Cas6	cas6	COG1583	CAS-I,CAS-III,CAS-IV			
163 CAS-I-B | SJK85391.1																	
1529346..1535487	array		LT719092.1	Cuniculiplasma divulgatum	Archaea	Euryarchaeota	Thermoplasmata	Thermoplasmatales	Cuniculiplasmataceae	Cuniculiplasma		CRISPR array			93	36	30
1535577..1535841	-	SJK85391.1	LT719092.1	Cuniculiplasma divulgatum	Archaea	Euryarchaeota	Thermoplasmata	Thermoplasmatales	Cuniculiplasmataceae	Cuniculiplasma	CRISPR-associated protein Cas2	**cas2**	COG1343	CAS-I,CAS-II,CAS-III			
1535847..1536840	-	SJK85392.1	LT719092.1	Cuniculiplasma divulgatum	Archaea	Euryarchaeota	Thermoplasmata	Thermoplasmatales	Cuniculiplasmataceae	Cuniculiplasma	CRISPR-associated endonuclease Cas1	cas1	cd09722	CAS-I-B			
1536841..1537345	-	SJK85393.1	LT719092.1	Cuniculiplasma divulgatum	Archaea	Euryarchaeota	Thermoplasmata	Thermoplasmatales	Cuniculiplasmataceae	Cuniculiplasma	CRISPR-associated endonuclease Cas4	cas4	pfam01930	CAS-I			
1537344..1539810	-	SJK85394.1	LT719092.1	Cuniculiplasma divulgatum	Archaea	Euryarchaeota	Thermoplasmata	Thermoplasmatales	Cuniculiplasmataceae	Cuniculiplasma	CRISPR-associated helicase Cas3	cas3HD,cas3	cd09641,COG1203	CAS-I			
1539797..1540577	-	SJK85395.1	LT719092.1	Cuniculiplasma divulgatum	Archaea	Euryarchaeota	Thermoplasmata	Thermoplasmatales	Cuniculiplasmataceae	Cuniculiplasma	CRISPR-associated crRNA-binding protein Cas5	cas5	cd09692	CAS-I-B			
1540569..1541502	-	SJK85396.1	LT719092.1	Cuniculiplasma divulgatum	Archaea	Euryarchaeota	Thermoplasmata	Thermoplasmatales	Cuniculiplasmataceae	Cuniculiplasma	CRISPR-associated pre-crRNA-binding protein Cas7	cas7b	pfam05107	CAS-I-B,CAS-I-C			
1541498..1543292	-	SJK85397.1	LT719092.1	Cuniculiplasma divulgatum	Archaea	Euryarchaeota	Thermoplasmata	Thermoplasmatales	Cuniculiplasmataceae	Cuniculiplasma	CRISPR-associated protein crRNA-binding Cas8b	cas8b1	pfam09484	CAS-I-B			
1543297..1544047	-	SJK85398.1	LT719092.1	Cuniculiplasma divulgatum	Archaea	Euryarchaeota	Thermoplasmata	Thermoplasmatales	Cuniculiplasmataceae	Cuniculiplasma	CRISPR-associated pre-crRNA processing enzyme Cas6	cas6	COG1583	CAS-I,CAS-III,CAS-IV			
164 CAS-I-B | AGO61204.1																	
1184605..1185349	+	AGO61196.1	CP004145.1	Ferroplasma acidarmanus fer1	Archaea	Euryarchaeota	Thermoplasmata	Thermoplasmatales	Ferroplasmaceae	Ferroplasma	hypothetical protein	cas6	pfam01881	CAS-I,CAS-III			
1185345..1187190	+	AGO61197.1	CP004145.1	Ferroplasma acidarmanus fer1	Archaea	Euryarchaeota	Thermoplasmata	Thermoplasmatales	Ferroplasmaceae	Ferroplasma	CRISPR-associated Csh1 family protein	cas8b1	pfam09484	CAS-I-B			
1187192..1188110	+	AGO61198.1	CP004145.1	Ferroplasma acidarmanus fer1	Archaea	Euryarchaeota	Thermoplasmata	Thermoplasmatales	Ferroplasmaceae	Ferroplasma	CRISPR-associated Csh2 family protein	cas7b	pfam05107	CAS-I-B,CAS-I-C			
1188112..1188883	+	AGO61199.1	CP004145.1	Ferroplasma acidarmanus fer1	Archaea	Euryarchaeota	Thermoplasmata	Thermoplasmatales	Ferroplasmaceae	Ferroplasma	CRISPR-associated Cas5 family Hmari subtype protein	cas5	cd09692	CAS-I-B			
1188863..1191275	+	AGO61200.1	CP004145.1	Ferroplasma acidarmanus fer1	Archaea	Euryarchaeota	Thermoplasmata	Thermoplasmatales	Ferroplasmaceae	Ferroplasma	hypothetical protein	cas3HD,cas3	cd09641,COG1203	CAS-I			
1191277..1191829	+	AGO61201.1	CP004145.1	Ferroplasma acidarmanus fer1	Archaea	Euryarchaeota	Thermoplasmata	Thermoplasmatales	Ferroplasmaceae	Ferroplasma	CRISPR-associated Cas4 family protein	cas4	pfam01930	CAS-I			
1191780..1192821	+	AGO61202.1	CP004145.1	Ferroplasma acidarmanus fer1	Archaea	Euryarchaeota	Thermoplasmata	Thermoplasmatales	Ferroplasmaceae	Ferroplasma	transposase						
1193053..1194046	+	AGO61203.1	CP004145.1	Ferroplasma acidarmanus fer1	Archaea	Euryarchaeota	Thermoplasmata	Thermoplasmatales	Ferroplasmaceae	Ferroplasma	hypothetical protein	cas1	cd09722	CAS-I-B			
1194051..1194315	+	AGO61204.1	CP004145.1	Ferroplasma acidarmanus fer1	Archaea	Euryarchaeota	Thermoplasmata	Thermoplasmatales	Ferroplasmaceae	Ferroplasma	CRISPR-associated protein Cas2	**cas2**	cd09725	CAS-I,CAS-II,CAS-III,CAS-V			
1194568..1195066	array		CP004145.1	Ferroplasma acidarmanus fer1	Archaea	Euryarchaeota	Thermoplasmata	Thermoplasmatales	Ferroplasmaceae	Ferroplasma		CRISPR array			8	37	30
165 CAS-I-B | SMD31391.1																	
289..565	-	SMD31391.1	FWYE01000004.1	Picrophilus oshimae DSM 9789	Archaea	Euryarchaeota	Thermoplasmata	Thermoplasmatales	Picrophilaceae	Picrophilus	CRISPR-associated protein Cas2	**cas2**	cd09725	CAS-I,CAS-II,CAS-III,CAS-V			
558..1551	-	SMD31392.1	FWYE01000004.1	Picrophilus oshimae DSM 9789	Archaea	Euryarchaeota	Thermoplasmata	Thermoplasmatales	Picrophilaceae	Picrophilus	CRISP-associated protein Cas1	cas1	cd09722	CAS-I-B			
1556..2054	-	SMD31393.1	FWYE01000004.1	Picrophilus oshimae DSM 9789	Archaea	Euryarchaeota	Thermoplasmata	Thermoplasmatales	Picrophilaceae	Picrophilus	CRISPR-associated exonuclease, Cas4 family	cas4	pfam01930	CAS-I			
2054..4484	-	SMD31394.1	FWYE01000004.1	Picrophilus oshimae DSM 9789	Archaea	Euryarchaeota	Thermoplasmata	Thermoplasmatales	Picrophilaceae	Picrophilus	CRISPR-associated helicase, Cas3 family	cas3HD,cas3	cd09641,COG1203	CAS-I			
4428..5193	-	SMD31395.1	FWYE01000004.1	Picrophilus oshimae DSM 9789	Archaea	Euryarchaeota	Thermoplasmata	Thermoplasmatales	Picrophilaceae	Picrophilus	CRISPR-associated protein Cas5h	cas5	cd09692	CAS-I-B			
5204..6116	-	SMD31396.1	FWYE01000004.1	Picrophilus oshimae DSM 9789	Archaea	Euryarchaeota	Thermoplasmata	Thermoplasmatales	Picrophilaceae	Picrophilus	CRISPR-associated protein, Csh2 family	cas7b	pfam05107	CAS-I-B,CAS-I-C			
6084..7947	-	SMD31397.1	FWYE01000004.1	Picrophilus oshimae DSM 9789	Archaea	Euryarchaeota	Thermoplasmata	Thermoplasmatales	Picrophilaceae	Picrophilus	CRISPR-associated protein Csh1	cas8b1	pfam09484	CAS-I-B			
7943..8690	-	SMD31398.1	FWYE01000004.1	Picrophilus oshimae DSM 9789	Archaea	Euryarchaeota	Thermoplasmata	Thermoplasmatales	Picrophilaceae	Picrophilus	CRISPR-associated endoribonuclease Cas6	cas6	COG1583	CAS-I,CAS-III,CAS-IV			
166 CAS-I-B | AEW06742.1																	
3246091..3248876	array		CP003179.1	Sulfobacillus acidophilus DSM 10332	Bacteria	Firmicutes	Clostridia	Clostridiales	Clostridiales Family XVII. Incertae Sedis	Sulfobacillus		CRISPR array			43	35	30
3249493..3249757	-	AEW06742.1	CP003179.1	Sulfobacillus acidophilus DSM 10332	Bacteria	Firmicutes	Clostridia	Clostridiales	Clostridiales Family XVII. Incertae Sedis	Sulfobacillus	CRISPR-associated protein, Cas2 family	**cas2**	cd09725	CAS-I,CAS-II,CAS-III,CAS-V			
3249770..3250763	-	AEW06743.1	CP003179.1	Sulfobacillus acidophilus DSM 10332	Bacteria	Firmicutes	Clostridia	Clostridiales	Clostridiales Family XVII. Incertae Sedis	Sulfobacillus	CRISPR-associated protein Cas1	cas1	cd09722	CAS-I-B			
3250767..3251280	-	AEW06744.1	CP003179.1	Sulfobacillus acidophilus DSM 10332	Bacteria	Firmicutes	Clostridia	Clostridiales	Clostridiales Family XVII. Incertae Sedis	Sulfobacillus	CRISPR-associated exonuclease, Cas4 family	cas4	pfam01930	CAS-I			
3251273..3253640	-	AEW06745.1	CP003179.1	Sulfobacillus acidophilus DSM 10332	Bacteria	Firmicutes	Clostridia	Clostridiales	Clostridiales Family XVII. Incertae Sedis	Sulfobacillus	CRISPR-associated HD domain protein	cas3HD,cas3	cd09641,COG1203	CAS-I			
3253639..3254338	-	AEW06746.1	CP003179.1	Sulfobacillus acidophilus DSM 10332	Bacteria	Firmicutes	Clostridia	Clostridiales	Clostridiales Family XVII. Incertae Sedis	Sulfobacillus	CRISPR-associated protein Cas5	cas5	cls001430	CAS-I-B			
3254348..3255308	-	AEW06747.1	CP003179.1	Sulfobacillus acidophilus DSM 10332	Bacteria	Firmicutes	Clostridia	Clostridiales	Clostridiales Family XVII. Incertae Sedis	Sulfobacillus	CRISPR-associated protein, Csh2 family	cas7b	pfam05107	CAS-I-B,CAS-I-C			
3255313..3257275	-	AEW06748.1	CP003179.1	Sulfobacillus acidophilus DSM 10332	Bacteria	Firmicutes	Clostridia	Clostridiales	Clostridiales Family XVII. Incertae Sedis	Sulfobacillus	hypothetical protein	cas8b1	mkCas0074	CAS-I-B			
3257275..3258034	-	AEW06749.1	CP003179.1	Sulfobacillus acidophilus DSM 10332	Bacteria	Firmicutes	Clostridia	Clostridiales	Clostridiales Family XVII. Incertae Sedis	Sulfobacillus	CRISPR-associated protein, Cas6 family	cas6	COG1583	CAS-I,CAS-III,CAS-IV			
3258232..3259962	array		CP003179.1	Sulfobacillus acidophilus DSM 10332	Bacteria	Firmicutes	Clostridia	Clostridiales	Clostridiales Family XVII. Incertae Sedis	Sulfobacillus		CRISPR array			27	35	30
3260381..3261569	-	AEW06750.1	CP003179.1	Sulfobacillus acidophilus DSM 10332	Bacteria	Firmicutes	Clostridia	Clostridiales	Clostridiales Family XVII. Incertae Sedis	Sulfobacillus	hypothetical protein						
3261565..3262504	-	AEW06751.1	CP003179.1	Sulfobacillus acidophilus DSM 10332	Bacteria	Firmicutes	Clostridia	Clostridiales	Clostridiales Family XVII. Incertae Sedis	Sulfobacillus	ABC transporter related protein	Cas_RecF	cluster17				
167 partial | POB09389.1																	
139860..140124	-	POB09389.1	NWUL01000047.1	Sulfobacillus sp. hq2	Bacteria	Firmicutes	Clostridia	Clostridiales	Clostridiales Family XVII. Incertae Sedis	Sulfobacillus	CRISPR-associated endonuclease Cas2	**cas2**	cd09725	CAS-I,CAS-II,CAS-III,CAS-V			
168 CAS-I-B | PSR26896.1																	
205..469	-	PSR26896.1	PXYT01000031.1	Sulfobacillus benefaciens	Bacteria	Firmicutes	Clostridia	Clostridiales	Clostridiales Family XVII. Incertae Sedis	Sulfobacillus	CRISPR-associated endonuclease Cas2	**cas2**	COG1343	CAS-I,CAS-II,CAS-III			
482..1475	-	PSR26897.1	PXYT01000031.1	Sulfobacillus benefaciens	Bacteria	Firmicutes	Clostridia	Clostridiales	Clostridiales Family XVII. Incertae Sedis	Sulfobacillus	subtype I-B CRISPR-associated endonuclease Cas1	cas1	cd09722	CAS-I-B			
1504..2014	-	PSR26924.1	PXYT01000031.1	Sulfobacillus benefaciens	Bacteria	Firmicutes	Clostridia	Clostridiales	Clostridiales Family XVII. Incertae Sedis	Sulfobacillus	CRISPR-associated protein Cas4	cas4	pfam01930	CAS-I			
2034..4398	-	PSR26898.1	PXYT01000031.1	Sulfobacillus benefaciens	Bacteria	Firmicutes	Clostridia	Clostridiales	Clostridiales Family XVII. Incertae Sedis	Sulfobacillus	CRISPR-associated helicase/endonuclease Cas3	cas3HD,cas3	cd09641,COG1203	CAS-I			
4419..5121	-	PSR26899.1	PXYT01000031.1	Sulfobacillus benefaciens	Bacteria	Firmicutes	Clostridia	Clostridiales	Clostridiales Family XVII. Incertae Sedis	Sulfobacillus	CRISPR-associated protein Cas5	cas5,cas5	cd09692,mkCas0191	CAS-I-B			
5146..6112	-	PSR26925.1	PXYT01000031.1	Sulfobacillus benefaciens	Bacteria	Firmicutes	Clostridia	Clostridiales	Clostridiales Family XVII. Incertae Sedis	Sulfobacillus	CRISPR-associated protein Csd2	cas7b	pfam05107	CAS-I-B,CAS-I-C			
6150..8133	-	PSR26900.1	PXYT01000031.1	Sulfobacillus benefaciens	Bacteria	Firmicutes	Clostridia	Clostridiales	Clostridiales Family XVII. Incertae Sedis	Sulfobacillus	hypothetical protein	cas8b1	mkCas0074	CAS-I-B			
8150..8924	-	PSR26901.1	PXYT01000031.1	Sulfobacillus benefaciens	Bacteria	Firmicutes	Clostridia	Clostridiales	Clostridiales Family XVII. Incertae Sedis	Sulfobacillus	CRISPR-associated endoribonuclease Cas6	cas6	COG1583	CAS-I,CAS-III,CAS-IV			
169 partial | GBD19029.1																	
87..351	+	GBD19029.1	BEID01000245.1	bacterium HR27	Bacteria						CRISPR-associated endoribonuclease Cas2	**cas2**	cd09725	CAS-I,CAS-II,CAS-III,CAS-V			
170 CAS-I-B | GBC92712.1																	
33839..34631	+	GBC92705.1	BEHR01000012.1	bacterium HR15	Bacteria						hypothetical protein	cas6	COG1583	CAS-I,CAS-III,CAS-IV			
34661..36119	+	GBC92706.1	BEHR01000012.1	bacterium HR15	Bacteria						hypothetical protein	cas8b1	mkCas0195	CAS-I-B			
36115..37042	+	GBC92707.1	BEHR01000012.1	bacterium HR15	Bacteria						hypothetical protein	cas7	cd09687	CAS-I-B			
37038..37755	+	GBC92708.1	BEHR01000012.1	bacterium HR15	Bacteria						hypothetical protein	cas5	cd09693	CAS-I			
37806..39999	+	GBC92709.1	BEHR01000012.1	bacterium HR15	Bacteria						CRISPR-associated endonuclease/helicase Cas3	cas3HD,cas3	cd09641,cd09639	CAS-I			
40002..40587	+	GBC92710.1	BEHR01000012.1	bacterium HR15	Bacteria						hypothetical protein	cas4	pfam01930	CAS-I			
40583..41576	+	GBC92711.1	BEHR01000012.1	bacterium HR15	Bacteria						CRISPR-associated endonuclease Cas1	cas1	cd09722	CAS-I-B			
41580..41844	+	GBC92712.1	BEHR01000012.1	bacterium HR15	Bacteria						CRISPR-associated endoribonuclease Cas2	**cas2**	COG1343	CAS-I,CAS-II,CAS-III			
42233..45388	array		BEHR01000012.1	bacterium HR15	Bacteria							CRISPR array			48	36	30
171 CAS-I-B | KPL89581.1																	
890274..892839	array		LGKN01000003.1	Ardenticatena maritima	Bacteria	Chloroflexi	Ardenticatenia	Ardenticatenales	Ardenticatenaceae	Ardenticatena		CRISPR array			39	36	30

893069..893333	+	KPL89581.1	LGKN01000003.1	Ardenticatena maritima	Bacteria	Chloroflexi	Ardenticatenia	Ardenticatenales	Ardenticatenaceae	Ardenticatena	CRISPR-associated protein Cas2	cas2	COG1343	CAS-I,CAS-II,CAS-III			
893344..894337	-	KPL89582.1	LGKN01000003.1	Ardenticatena maritima	Bacteria	Chloroflexi	Ardenticatenia	Ardenticatenales	Ardenticatenaceae	Ardenticatena	CRISPR-associated protein Cas1	cas1	cd09722	CAS-I-B			
894338..894851	-	KPL89583.1	LGKN01000003.1	Ardenticatena maritima	Bacteria	Chloroflexi	Ardenticatenia	Ardenticatenales	Ardenticatenaceae	Ardenticatena	CRISPR-associated protein Cas4	cas4	pfam01930	CAS-I			
895302..897804	-	KPL89584.1	LGKN01000003.1	Ardenticatena maritima	Bacteria	Chloroflexi	Ardenticatenia	Ardenticatenales	Ardenticatenaceae	Ardenticatena	hypothetical protein	cas3HD,cas3	cd09641,COG1203	CAS-I			
897764..898541	-	KPL89585.1	LGKN01000003.1	Ardenticatena maritima	Bacteria	Chloroflexi	Ardenticatenia	Ardenticatenales	Ardenticatenaceae	Ardenticatena	hypothetical protein	cas5	cls001430	CAS-I-B			
898537..899458	-	KPL89586.1	LGKN01000003.1	Ardenticatena maritima	Bacteria	Chloroflexi	Ardenticatenia	Ardenticatenales	Ardenticatenaceae	Ardenticatena	CRISPR-associated protein Csh2	cas7	cd09690	CAS-I-B			
899454..901200	-	KPL89587.1	LGKN01000003.1	Ardenticatena maritima	Bacteria	Chloroflexi	Ardenticatenia	Ardenticatenales	Ardenticatenaceae	Ardenticatena	hypothetical protein	cas8b1	cd09730	CAS-I-B			
901237..902008	-	KPL89588.1	LGKN01000003.1	Ardenticatena maritima	Bacteria	Chloroflexi	Ardenticatenia	Ardenticatenales	Ardenticatenaceae	Ardenticatena	hypothetical protein	cas6	COG1583	CAS-I,CAS-III,CAS-IV			
172 Partial CAS-I-B | ABG03233.1																	
262283..263239	array		CP000386.1	Rubrobacter xylanophilus DSM 9941	Bacteria	Actinobacteria	Rubrobacteria	Rubrobacterales	Rubrobacteraceae	Rubrobacter		CRISPR array			15	36	30
263397..263667	-	ABG03233.1	CP000386.1	Rubrobacter xylanophilus DSM 9941	Bacteria	Actinobacteria	Rubrobacteria	Rubrobacterales	Rubrobacteraceae	Rubrobacter	CRISPR-associated protein, Cas2 family	cas2	cd09725	CAS-I,CAS-II,CAS-III,CAS-V			
263670..264666	-	ABG03234.1	CP000386.1	Rubrobacter xylanophilus DSM 9941	Bacteria	Actinobacteria	Rubrobacteria	Rubrobacterales	Rubrobacteraceae	Rubrobacter	CRISPR-associated protein, Cas1 family	cas1	cd09722	CAS-I-B			
173 CAS-I-B | ACZ43326.1																	
570920..572600	+	ACZ43319.1	CP001826.1	Thermobaculum terrenum ATCC BAA-798	Bacteria					Thermobaculum	NERD domain protein	C3a187	cluster187				
572753..574739	+	ACZ43320.1	CP001826.1	Thermobaculum terrenum ATCC BAA-798	Bacteria					Thermobaculum	CRISPR-associated protein, Csh1 family	cas8b1	cd09730	CAS-I-B			
574745..575705	+	ACZ43321.1	CP001826.1	Thermobaculum terrenum ATCC BAA-798	Bacteria					Thermobaculum	CRISPR-associated protein, Csh2 family	cas7b	pfam05107	CAS-I-B,CAS-I-C			
575716..576511	+	ACZ43322.1	CP001826.1	Thermobaculum terrenum ATCC BAA-798	Bacteria					Thermobaculum	CRISPR-associated protein Cas5	cas5	mkCas0191	CAS-I-B			
576507..579084	+	ACZ43323.1	CP001826.1	Thermobaculum terrenum ATCC BAA-798	Bacteria					Thermobaculum	CRISPR-associated helicase Cas3	cas3HD,cas3,cas3	cd09641,COG1203,COG1203	CAS-I			
579080..579578	+	ACZ43324.1	CP001826.1	Thermobaculum terrenum ATCC BAA-798	Bacteria					Thermobaculum	CRISPR-associated protein Cas4	cas4	pfam01930	CAS-I			
579574..580567	+	ACZ43325.1	CP001826.1	Thermobaculum terrenum ATCC BAA-798	Bacteria					Thermobaculum	CRISPR-associated protein Cas1	cas1	cd09722	CAS-I-B			
580568..580832	+	ACZ43326.1	CP001826.1	Thermobaculum terrenum ATCC BAA-798	Bacteria					Thermobaculum	CRISPR-associated protein Cas2	cas2	COG1343	CAS-I,CAS-II,CAS-III			
174 CAS-I-B | AEH51236.1																	
1136555..1138406	array		CP002351.1	Pseudothermotoga thermarum DSM 5069	Bacteria	Thermotogae	Thermotogae	Thermotogales	Thermotogaceae	Pseudothermotoga		CRISPR array			28	37	30
1139400..1139622	+	AEH51225.1	CP002351.1	Pseudothermotoga thermarum DSM 5069	Bacteria	Thermotogae	Thermotogae	Thermotogales	Thermotogaceae	Pseudothermotoga	hypothetical protein						
1139680..1140508	+	AEH51226.1	CP002351.1	Pseudothermotoga thermarum DSM 5069	Bacteria	Thermotogae	Thermotogae	Thermotogales	Thermotogaceae	Pseudothermotoga	hypothetical protein						
1141127..1142006	+	AEH51227.1	CP002351.1	Pseudothermotoga thermarum DSM 5069	Bacteria	Thermotogae	Thermotogae	Thermotogales	Thermotogaceae	Pseudothermotoga	CRISPR-associated protein, Csx13 family	csx1	cd09741	CAS-III			
1142021..1143164	+	AEH51228.1	CP002351.1	Pseudothermotoga thermarum DSM 5069	Bacteria	Thermotogae	Thermotogae	Thermotogales	Thermotogaceae	Pseudothermotoga	CRISPR-associated protein, APE2256 family	csm6	cd09742	CAS-III-A,CAS-III-D			
1143177..1143927	+	AEH51229.1	CP002351.1	Pseudothermotoga thermarum DSM 5069	Bacteria	Thermotogae	Thermotogae	Thermotogales	Thermotogaceae	Pseudothermotoga	CRISPR-associated protein, Cas6 family	cas6	COG1583	CAS-I,CAS-III,CAS-IV			
1143968..1145471	+	AEH51230.1	CP002351.1	Pseudothermotoga thermarum DSM 5069	Bacteria	Thermotogae	Thermotogae	Thermotogales	Thermotogaceae	Pseudothermotoga	hypothetical protein	cas8b1	mkCas0195	CAS-I-B			
1145463..1146432	+	AEH51231.1	CP002351.1	Pseudothermotoga thermarum DSM 5069	Bacteria	Thermotogae	Thermotogae	Thermotogales	Thermotogaceae	Pseudothermotoga	CRISPR-associated autoregulator, Cst2 family	cas7	cd09687	CAS-I-B			
1146428..1147148	+	AEH51232.1	CP002351.1	Pseudothermotoga thermarum DSM 5069	Bacteria	Thermotogae	Thermotogae	Thermotogales	Thermotogaceae	Pseudothermotoga	CRISPR-associated protein Cas5	cas5	cd09693	CAS-I			
1147135..1149328	+	AEH51233.1	CP002351.1	Pseudothermotoga thermarum DSM 5069	Bacteria	Thermotogae	Thermotogae	Thermotogales	Thermotogaceae	Pseudothermotoga	CRISPR-associated helicase Cas3	cas3HD,cas3	cd09641,cd09639	CAS-I			
1149348..1149846	+	AEH51234.1	CP002351.1	Pseudothermotoga thermarum DSM 5069	Bacteria	Thermotogae	Thermotogae	Thermotogales	Thermotogaceae	Pseudothermotoga	CRISPR-associated exonuclease, Cas4 family	cas4	pfam01930	CAS-I			
1149859..1150855	+	AEH51235.1	CP002351.1	Pseudothermotoga thermarum DSM 5069	Bacteria	Thermotogae	Thermotogae	Thermotogales	Thermotogaceae	Pseudothermotoga	CRISPR-associated protein, Cas1 family	cas1	cd09722	CAS-I-B			
1150882..1151146	+	AEH51236.1	CP002351.1	Pseudothermotoga thermarum DSM 5069	Bacteria	Thermotogae	Thermotogae	Thermotogales	Thermotogaceae	Pseudothermotoga	CRISPR-associated protein, Cas2 family	cas2	cd09725	CAS-I,CAS-II,CAS-III,CAS-V			
1151411..1152854	array		CP002351.1	Pseudothermotoga thermarum DSM 5069	Bacteria	Thermotogae	Thermotogae	Thermotogales	Thermotogaceae	Pseudothermotoga		CRISPR array			22	37	30
175 partial CAS-I-B | CEP66644.1																	
384..1377	+	CEP66643.1	CELZ01000023.1	Moorella glycerini	Bacteria	Firmicutes	Clostridia	Thermoanaerobacterales	Thermoanaerobacteraceae	Moorella	CRISPR-associated protein Cas1, HMARI/TNEAP subtype	cas1	cd09722	CAS-I-B			
1397..1661	+	CEP66644.1	CELZ01000023.1	Moorella glycerini	Bacteria	Firmicutes	Clostridia	Thermoanaerobacterales	Thermoanaerobacteraceae	Moorella	CRISPR associated protein Cas2	cas2	cd09725	CAS-I,CAS-II,CAS-III,CAS-V			
1733..1871	+	CEP66645.1	CELZ01000023.1	Moorella glycerini	Bacteria	Firmicutes	Clostridia	Thermoanaerobacterales	Thermoanaerobacteraceae	Moorella	Uncharacterized						
2189..2573	+	CEP66646.1	CELZ01000023.1	Moorella glycerini	Bacteria	Firmicutes	Clostridia	Thermoanaerobacterales	Thermoanaerobacteraceae	Moorella	Uncharacterized	csx1	pfam09670	CAS-III			
2616..2895	+	CEP66647.1	CELZ01000023.1	Moorella glycerini	Bacteria	Firmicutes	Clostridia	Thermoanaerobacterales	Thermoanaerobacteraceae	Moorella	Uncharacterized	csx1	cd09747	CAS-III			
2863..3253	+	CEP66648.1	CELZ01000023.1	Moorella glycerini	Bacteria	Firmicutes	Clostridia	Thermoanaerobacterales	Thermoanaerobacteraceae	Moorella	Uncharacterized	csx1	pfam09670	CAS-III			
176 CAS-I-B | SMB98803.1																	
2499512..2501948	-	SMB98793.1	LT838272.1	Thermanaeromonas toyohensis ToBE	Bacteria	Firmicutes	Clostridia	Thermoanaerobacterales	Thermoanaerobacteraceae	Thermanaeromonas	CRISPR-associated helicase Cas3/CRISPR-associated endonuclease Cas3-HD	cas3HD,cas3	cd09641,COG1203	CAS-I			
2501941..2502679	-	SMB98798.1	LT838272.1	Thermanaeromonas toyohensis ToBE	Bacteria	Firmicutes	Clostridia	Thermoanaerobacterales	Thermoanaerobacteraceae	Thermanaeromonas	CRISPR-associated protein Cas5h	cas5,cas5	cls001430,mkCas0191	CAS-I-B			
2502817..2503759	-	SMB98800.1	LT838272.1	Thermanaeromonas toyohensis ToBE	Bacteria	Firmicutes	Clostridia	Thermoanaerobacterales	Thermoanaerobacteraceae	Thermanaeromonas	CRISPR-associated protein, Csh2 family	cas7b	pfam05107	CAS-I-B,CAS-I-C			
2503772..2505926	-	SMB98801.1	LT838272.1	Thermanaeromonas toyohensis ToBE	Bacteria	Firmicutes	Clostridia	Thermoanaerobacterales	Thermoanaerobacteraceae	Thermanaeromonas	CRISPR-associated protein TM1802 (cas_TM1802)	cas8b1	mkCas0074	CAS-I-B			
2506133..2507156	-	SMB98802.1	LT838272.1	Thermanaeromonas toyohensis ToBE	Bacteria	Firmicutes	Clostridia	Thermoanaerobacterales	Thermoanaerobacteraceae	Thermanaeromonas	Predicted DNA-binding transcriptional regulator YafY, contains an HTH and WYL domains	WYL	pfam13280	CAS-I,CAS-III			
2507281..2507545	-	SMB98803.1	LT838272.1	Thermanaeromonas toyohensis ToBE	Bacteria	Firmicutes	Clostridia	Thermoanaerobacterales	Thermoanaerobacteraceae	Thermanaeromonas	CRISPR-associated protein, Cas2 family	cas2	cd09725	CAS-I,CAS-II,CAS-III,CAS-V			
2507550..2508609	-	SMB98805.1	LT838272.1	Thermanaeromonas toyohensis ToBE	Bacteria	Firmicutes	Clostridia	Thermoanaerobacterales	Thermoanaerobacteraceae	Thermanaeromonas	CRISPR-associated protein, Cas1 family	cas1	cd09722	CAS-I-B			
2508618..2509137	-	SMB98807.1	LT838272.1	Thermanaeromonas toyohensis ToBE	Bacteria	Firmicutes	Clostridia	Thermoanaerobacterales	Thermoanaerobacteraceae	Thermanaeromonas	CRISPR-associated exonuclease, Cas4 family	cas4	pfam01930	CAS-I			
2510834..2515207	array		LT838272.1	Thermanaeromonas toyohensis ToBE	Bacteria	Firmicutes	Clostridia	Thermoanaerobacterales	Thermoanaerobacteraceae	Thermanaeromonas		CRISPR array			66	36	30
177 CAS-I-B | RPF46988.1																	
477535..478952	array		RKRE01000002.1	Thermodesulfitimonas autotrophica	Bacteria	Firmicutes	Clostridia	Thermoanaerobacterales	Thermoanaerobacteraceae	Thermodesulfitimonas		CRISPR array			22	36	30
477781..478138	-	RPF46985.1	RKRE01000002.1	Thermodesulfitimonas autotrophica	Bacteria	Firmicutes	Clostridia	Thermoanaerobacterales	Thermoanaerobacteraceae	Thermodesulfitimonas	hypothetical protein						
478309..478666	-	RPF46986.1	RKRE01000002.1	Thermodesulfitimonas autotrophica	Bacteria	Firmicutes	Clostridia	Thermoanaerobacterales	Thermoanaerobacteraceae	Thermodesulfitimonas	hypothetical protein						
478662..478917	-	RPF46987.1	RKRE01000002.1	Thermodesulfitimonas autotrophica	Bacteria	Firmicutes	Clostridia	Thermoanaerobacterales	Thermoanaerobacteraceae	Thermodesulfitimonas	hypothetical protein						
479128..479392	-	RPF46988.1	RKRE01000002.1	Thermodesulfitimonas autotrophica	Bacteria	Firmicutes	Clostridia	Thermoanaerobacterales	Thermoanaerobacteraceae	Thermodesulfitimonas	CRISPR-associated Cas2 family protein	cas2	cd09725	CAS-I,CAS-II,CAS-III,CAS-V			
479422..480418	-	RPF46989.1	RKRE01000002.1	Thermodesulfitimonas autotrophica	Bacteria	Firmicutes	Clostridia	Thermoanaerobacterales	Thermoanaerobacteraceae	Thermodesulfitimonas	CRISPR-associated Cas1 family protein	cas1	cd09722	CAS-I-B			
480442..480982	-	RPF46990.1	RKRE01000002.1	Thermodesulfitimonas autotrophica	Bacteria	Firmicutes	Clostridia	Thermoanaerobacterales	Thermoanaerobacteraceae	Thermodesulfitimonas	CRISPR-associated Cas4 family exonuclease	cas4	pfam01930	CAS-I			
481048..483526	-	RPF46991.1	RKRE01000002.1	Thermodesulfitimonas autotrophica	Bacteria	Firmicutes	Clostridia	Thermoanaerobacterales	Thermoanaerobacteraceae	Thermodesulfitimonas	CRISPR-associated Cas3 family helicase	cas3HD,cas3	cd09641,COG1203	CAS-I			
483528..484296	-	RPF46992.1	RKRE01000002.1	Thermodesulfitimonas autotrophica	Bacteria	Firmicutes	Clostridia	Thermoanaerobacterales	Thermoanaerobacteraceae	Thermodesulfitimonas	CRISPR-associated Cas5h family protein	cas5	mkCas0191	CAS-I-B			
484300..485269	-	RPF46993.1	RKRE01000002.1	Thermodesulfitimonas autotrophica	Bacteria	Firmicutes	Clostridia	Thermoanaerobacterales	Thermoanaerobacteraceae	Thermodesulfitimonas	CRISPR-associated Csh2 family protein	cas7	cd09690	CAS-I-B			
485268..487320	-	RPF46994.1	RKRE01000002.1	Thermodesulfitimonas autotrophica	Bacteria	Firmicutes	Clostridia	Thermoanaerobacterales	Thermoanaerobacteraceae	Thermodesulfitimonas	CRISPR-associated protein Csh1	cas8b1	pfam09484	CAS-I-B			
178 partial | ACX51816.1																	
693040..694036	+	ACX51815.1	CP001785.1	Ammonifex degensii KC4	Bacteria	Firmicutes	Clostridia	Thermoanaerobacterales	Thermoanaerobacteraceae	Ammonifex	CRISPR-associated protein Cas1	cas1	cd09722	CAS-I-B			
694062..694326	+	ACX51816.1	CP001785.1	Ammonifex degensii KC4	Bacteria	Firmicutes	Clostridia	Thermoanaerobacterales	Thermoanaerobacteraceae	Ammonifex	CRISPR-associated protein Cas2	cas2	cd09725	CAS-I,CAS-II,CAS-III,CAS-V			
694576..695935	array		CP001785.1	Ammonifex degensii KC4	Bacteria	Firmicutes	Clostridia	Thermoanaerobacterales	Thermoanaerobacteraceae	Ammonifex		CRISPR array			21	36	30
179 partial CAS-I-B | RDV81757.1																	
15820..16816	+	RDV81768.1	QSLN01000016.1	Ammonifex thiophilus	Bacteria	Firmicutes	Clostridia	Thermoanaerobacterales	Thermoanaerobacteraceae	Ammonifex	type I-B CRISPR-associated endonuclease Cas1	cas1	cd09722	CAS-I-B			
16842..17106	+	RDV81757.1	QSLN01000016.1	Ammonifex thiophilus	Bacteria	Firmicutes	Clostridia	Thermoanaerobacterales	Thermoanaerobacteraceae	Ammonifex	CRISPR-associated endonuclease Cas2	cas2	cd09725	CAS-I,CAS-II,CAS-III,CAS-V			
17354..21449	array		QSLN01000016.1	Ammonifex thiophilus	Bacteria	Firmicutes	Clostridia	Thermoanaerobacterales	Thermoanaerobacteraceae	Ammonifex		CRISPR array			62	36	30
180 CAS-I-B | SHF40633.1																	
32041..32785	+	SHF40425.1	FQUW01000027.1	Desulfofundulus australicus DSM 11792	Bacteria	Firmicutes	Clostridia	Clostridiales	Peptococcaceae	Desulfofundulus	CRISPR-associated protein, Cas6 family	cas6	pfam01881	CAS-I,CAS-III			
32883..34914	+	SHF40457.1	FQUW01000027.1	Desulfofundulus australicus DSM 11792	Bacteria	Firmicutes	Clostridia	Clostridiales	Peptococcaceae	Desulfofundulus	CRISPR-associated protein Csh1	cas8b1	pfam09484	CAS-I-B			
34929..35901	+	SHF40486.1	FQUW01000027.1	Desulfofundulus australicus DSM 11792	Bacteria	Firmicutes	Clostridia	Clostridiales	Peptococcaceae	Desulfofundulus	CRISPR-associated protein, Csh2 family	cas7	cd09690	CAS-I-B			
35905..36673	+	SHF40517.1	FQUW01000027.1	Desulfofundulus australicus DSM 11792	Bacteria	Firmicutes	Clostridia	Clostridiales	Peptococcaceae	Desulfofundulus	CRISPR-associated protein Cas5h	cas5	mkCas0191	CAS-I-B			
36674..39155	+	SHF40550.1	FQUW01000027.1	Desulfofundulus australicus DSM 11792	Bacteria	Firmicutes	Clostridia	Clostridiales	Peptococcaceae	Desulfofundulus	CRISPR-associated helicase, Cas3 family	cas3HD,cas3	cd09641,COG1203	CAS-I			
39223..39727	+	SHF40577.1	FQUW01000027.1	Desulfofundulus australicus DSM 11792	Bacteria	Firmicutes	Clostridia	Clostridiales	Peptococcaceae	Desulfofundulus	CRISPR-associated exonuclease, Cas4 family	cas4	pfam01930	CAS-I			
39793..40789	+	SHF40602.1	FQUW01000027.1	Desulfofundulus australicus DSM 11792	Bacteria	Firmicutes	Clostridia	Clostridiales	Peptococcaceae	Desulfofundulus	CRISPR-associated protein, Cas1 family	cas1	cd09722	CAS-I-B			
40903..41167	+	SHF40633.1	FQUW01000027.1	Desulfofundulus australicus DSM 11792	Bacteria	Firmicutes	Clostridia	Clostridiales	Peptococcaceae	Desulfofundulus	CRISPR-associated protein, Cas2 family	cas2	cd09725	CAS-I,CAS-II,CAS-III,CAS-V			
181 CAS-I-B | AEG14082.1																	
473071..473815	+	AEG14075.1	CP002770.1	Desulfofundulus kuznetsovii DSM 6115	Bacteria	Firmicutes	Clostridia	Clostridiales	Peptococcaceae	Desulfofundulus	CRISPR-associated protein Cas6	cas6	COG1583	CAS-I,CAS-III,CAS-IV			
473963..475958	+	AEG14076.1	CP002770.1	Desulfofundulus kuznetsovii DSM 6115	Bacteria	Firmicutes	Clostridia	Clostridiales	Peptococcaceae	Desulfofundulus	CRISPR-associated protein Csh1 domain protein	cas8b1	pfam09484	CAS-I-B			
475973..476945	+	AEG14077.1	CP002770.1	Desulfofundulus kuznetsovii DSM 6115	Bacteria	Firmicutes	Clostridia	Clostridiales	Peptococcaceae	Desulfofundulus	CRISPR-associated protein, Csh2 family	cas7b	pfam05107	CAS-I-B,CAS-I-C			
476949..477717	+	AEG14078.1	CP002770.1	Desulfofundulus kuznetsovii DSM 6115	Bacteria	Firmicutes	Clostridia	Clostridiales	Peptococcaceae	Desulfofundulus	CRISPR-associated protein Cas5	cas5	mkCas0191	CAS-I-B			
477719..480209	+	AEG14079.1	CP002770.1	Desulfofundulus kuznetsovii DSM 6115	Bacteria	Firmicutes	Clostridia	Clostridiales	Peptococcaceae	Desulfofundulus	CRISPR-associated HD domain protein	cas3HD,cas3	cd09641,COG1203	CAS-I			
480224..480782	+	AEG14080.1	CP002770.1	Desulfofundulus kuznetsovii DSM 6115	Bacteria	Firmicutes	Clostridia	Clostridiales	Peptococcaceae	Desulfofundulus	CRISPR-associated protein Cas4	cas4	pfam01930	CAS-I			
480879..481875	+	AEG14081.1	CP002770.1	Desulfofundulus kuznetsovii DSM 6115	Bacteria	Firmicutes	Clostridia	Clostridiales	Peptococcaceae	Desulfofundulus	CRISPR-associated protein Cas1	cas1	cd09722	CAS-I-B			
484184..484448	+	AEG14082.1	CP002770.1	Desulfofundulus kuznetsovii DSM 6115	Bacteria	Firmicutes	Clostridia	Clostridiales	Peptococcaceae	Desulfofundulus	CRISPR-associated protein Cas2	cas2	cd09725	CAS-I,CAS-II,CAS-III,CAS-V			
484645..486681	array		CP002770.1	Desulfofundulus kuznetsovii DSM 6115	Bacteria	Firmicutes	Clostridia	Clostridiales	Peptococcaceae	Desulfofundulus		CRISPR array			31	36	30
486908..488450	-	AEG14083.1	CP002770.1	Desulfofundulus kuznetsovii DSM 6115	Bacteria	Firmicutes	Clostridia	Clostridiales	Peptococcaceae	Desulfofundulus	hypothetical protein						
488724..489023	array		CP002770.1	Desulfofundulus kuznetsovii DSM 6115	Bacteria	Firmicutes	Clostridia	Clostridiales	Peptococcaceae	Desulfofundulus		CRISPR array			5	37	30
182 CAS-I-B | SHI96005.1																	
29..261	array		FQZM01000016.1	Desulfofundulus thermosubterraneus DSM 16057	Bacteria	Firmicutes	Clostridia	Clostridiales	Peptococcaceae	Desulfofundulus		CRISPR array			4	35	32
435..699	-	SHI96005.1	FQZM01000016.1	Desulfofundulus thermosubterraneus DSM 16057	Bacteria	Firmicutes	Clostridia	Clostridiales	Peptococcaceae	Desulfofundulus	CRISPR-associated protein, Cas2 family	cas2	cd09725	CAS-I,CAS-II,CAS-III,CAS-V			
830..1826	-	SHI96040.1	FQZM01000016.1	Desulfofundulus thermosubterraneus DSM 16057	Bacteria	Firmicutes	Clostridia	Clostridiales	Peptococcaceae	Desulfofundulus	CRISPR-associated protein, Cas1 family	cas1	cd09722	CAS-I-B			
1923..2427	-	SHI96071.1	FQZM01000016.1	Desulfofundulus thermosubterraneus DSM 16057	Bacteria	Firmicutes	Clostridia	Clostridiales	Peptococcaceae	Desulfofundulus	CRISPR-associated exonuclease, Cas4 family	cas4	pfam01930	CAS-I			
2494..4984	-	SHI96096.1	FQZM01000016.1	Desulfofundulus thermosubterraneus DSM 16057	Bacteria	Firmicutes	Clostridia	Clostridiales	Peptococcaceae	Desulfofundulus	CRISPR-associated helicase, Cas3 family	cas3HD,cas3	cd09641,COG1203	CAS-I			
4986..5754	-	SHI96129.1	FQZM01000016.1	Desulfofundulus thermosubterraneus DSM 16057	Bacteria	Firmicutes	Clostridia	Clostridiales	Peptococcaceae	Desulfofundulus	CRISPR-associated protein Cas5h	cas5	mkCas0191	CAS-I-B			
5758..6727	-	SHI96166.1	FQZM01000016.1	Desulfofundulus thermosubterraneus DSM 16057	Bacteria	Firmicutes	Clostridia	Clostridiales	Peptococcaceae	Desulfofundulus	CRISPR-associated protein, Csh2 family	cas7b	pfam05107	CAS-I-B,CAS-I-C			
6723..8715	-	SHI96202.1	FQZM01000016.1	Desulfofundulus thermosubterraneus DSM 16057	Bacteria	Firmicutes	Clostridia	Clostridiales	Peptococcaceae	Desulfofundulus	CRISPR-associated protein Csh1	cas8b1	pfam09484	CAS-I-B			
9020..9770	-	SHI96229.1	FQZM01000016.1	Desulfofundulus thermosubterraneus DSM 16057	Bacteria	Firmicutes	Clostridia	Clostridiales	Peptococcaceae	Desulfofundulus	CRISPR-associated protein, Cas6 family	cas6	COG1583	CAS-I,CAS-III,CAS-IV			
184 CAS-I-B | AAM25776.1																	
2555594..2555858	-	AAM25776.1	AE008691.1	Caldanaerobacter subterraneus subsp. tengcongensis MB4	Bacteria	Firmicutes	Clostridia	Thermoanaerobacterales	Thermoanaerobacteraceae	Caldanaerobacter	conserved hypothetical protein	cas2	cd09725	CAS-I,CAS-II,CAS-III,CAS-V			
2555922..2556915	-	AAM25777.1	AE008691.1	Caldanaerobacter subterraneus subsp. tengcongensis MB4	Bacteria	Firmicutes	Clostridia	Thermoanaerobacterales	Thermoanaerobacteraceae	Caldanaerobacter	conserved hypothetical protein	cas1	cd09722	CAS-I-B			
2556950..2557463	-	AAM25778.1	AE008691.1	Caldanaerobacter subterraneus subsp. tengcongensis MB4	Bacteria	Firmicutes	Clostridia	Thermoanaerobacterales	Thermoanaerobacteraceae	Caldanaerobacter	RecB family exonuclease	cas4	pfam01930	CAS-I			
2557547..2559746	-	AAM25779.1	AE008691.1	Caldanaerobacter subterraneus subsp. tengcongensis MB4	Bacteria	Firmicutes	Clostridia	Thermoanaerobacterales	Thermoanaerobacteraceae	Caldanaerobacter	predicted helicases	cas3HD,cas3	cd09641,COG1203	CAS-I			
2559724..2560447	-	AAM25780.1	AE008691.1	Caldanaerobacter subterraneus subsp. tengcongensis MB4	Bacteria	Firmicutes	Clostridia	Thermoanaerobacterales	Thermoanaerobacteraceae	Caldanaerobacter	hypothetical protein	cas5	cd09693	CAS-I			
2560443..2561415	-	AAM25781.1	AE008691.1	Caldanaerobacter subterraneus subsp. tengcongensis MB4	Bacteria	Firmicutes	Clostridia	Thermoanaerobacterales	Thermoanaerobacteraceae	Caldanaerobacter	conserved hypothetical protein	cas7	cd09687	CAS-I-B			
2561395..2562922	-	AAM25782.1	AE008691.1	Caldanaerobacter subterraneus subsp. tengcongensis MB4	Bacteria	Firmicutes	Clostridia	Thermoanaerobacterales	Thermoanaerobacteraceae	Caldanaerobacter	hypothetical protein	cas8b1	mkCas0195	CAS-I-B			
2562914..2563697	-	AAM25783.1	AE008691.1	Caldanaerobacter subterraneus subsp. tengcongensis MB4	Bacteria	Firmicutes	Clostridia	Thermoanaerobacterales	Thermoanaerobacteraceae	Caldanaerobacter	conserved hypothetical protein	cas6	cd09652	CAS-I,CAS-III			
2563734..2564115	-	AAM25784.1	AE008691.1	Caldanaerobacter subterraneus subsp. tengcongensis MB4	Bacteria	Firmicutes	Clostridia	Thermoanaerobacterales	Thermoanaerobacteraceae	Caldanaerobacter	hypothetical protein	csx15	cd09766	CAS-I,CAS-III			
2564143..2565034	-	AAM25785.1	AE008691.1	Caldanaerobacter subterraneus subsp. tengcongensis MB4	Bacteria	Firmicutes	Clostridia	Thermoanaerobacterales	Thermoanaerobacteraceae	Caldanaerobacter	hypothetical protein	csx1	cd09741	CAS-III			
2565127..2565883	-	AAM25786.1	AE008691.1	Caldanaerobacter subterraneus subsp. tengcongensis MB4	Bacteria	Firmicutes	Clostridia	Thermoanaerobacterales	Thermoanaerobacteraceae	Caldanaerobacter	conserved hypothetical protein	cas6	COG1583	CAS-I,CAS-III,CAS-IV			
185 CAS-I-B | ERM92128.1																	
17575..19221	array		AXDC01000014.1	Caldanaerobacter subterraneus subsp. yonseiensis KB-1	Bacteria	Firmicutes	Clostridia	Thermoanaerobacterales	Thermoanaerobacteraceae	Caldanaerobacter		CRISPR array			25	37	30
19719..19983	-	ERM92128.1	AXDC01000014.1	Caldanaerobacter subterraneus subsp. yonseiensis KB-1	Bacteria	Firmicutes	Clostridia	Thermoanaerobacterales	Thermoanaerobacteraceae	Caldanaerobacter	CRISPR-associated protein Cas2	cas2	cd09725	CAS-I,CAS-II,CAS-III,CAS-V			
20048..21041	-	ERM92129.1	AXDC01000014.1	Caldanaerobacter subterraneus subsp. yonseiensis KB-1	Bacteria	Firmicutes	Clostridia	Thermoanaerobacterales	Thermoanaerobacteraceae	Caldanaerobacter	CRISPR-associated protein Cas1	cas1	cd09722	CAS-I-B			
21076..21589	-	ERM92130.1	AXDC01000014.1	Caldanaerobacter subterraneus subsp. yonseiensis KB-1	Bacteria	Firmicutes	Clostridia	Thermoanaerobacterales	Thermoanaerobacteraceae	Caldanaerobacter	CRISPR-associated protein Cas4	cas4	pfam01930	CAS-I			
21673..22522	-	ERM92157.1	AXDC01000014.1	Caldanaerobacter subterraneus subsp. yonseiensis KB-1	Bacteria	Firmicutes	Clostridia	Thermoanaerobacterales	Thermoanaerobacteraceae	Caldanaerobacter	hypothetical protein	cas3	COG1203	CAS-I			
22458..23871	-	ERM92158.1	AXDC01000014.1	Caldanaerobacter subterraneus subsp. yonseiensis KB-1	Bacteria	Firmicutes	Clostridia	Thermoanaerobacterales	Thermoanaerobacteraceae	Caldanaerobacter	hypothetical protein	cas3HD,cas3	cd09641,cd09639	CAS-I			
23994..24786	-	ERM92159.1	AXDC01000014.1	Caldanaerobacter subterraneus subsp. yonseiensis KB-1	Bacteria	Firmicutes	Clostridia	Thermoanaerobacterales	Thermoanaerobacteraceae	Caldanaerobacter	hypothetical protein	cas5	cls000048	CAS-I-A			
24787..25846	-	ERM92160.1	AXDC01000014.1	Caldanaerobacter subterraneus subsp. yonseiensis KB-1	Bacteria	Firmicutes	Clostridia	Thermoanaerobacterales	Thermoanaerobacteraceae	Caldanaerobacter	hypothetical protein	cas7	pfam01905	CAS-I			
25863..27354	-	ERM92161.1	AXDC01000014.1	Caldanaerobacter subterraneus subsp. yonseiensis KB-1	Bacteria	Firmicutes	Clostridia	Thermoanaerobacterales	Thermoanaerobacteraceae	Caldanaerobacter	hypothetical protein	cas8a8	mkCas0180	CAS-I-A			
27360..28539	-	ERM92162.1	AXDC01000014.1	Caldanaerobacter subterraneus subsp. yonseiensis KB-1	Bacteria	Firmicutes	Clostridia	Thermoanaerobacterales	Thermoanaerobacteraceae	Caldanaerobacter	hypothetical protein	cas3HD	cd09641	CAS-I			
28541..29387	-	ERM92163.1	AXDC01000014.1	Caldanaerobacter subterraneus subsp. yonseiensis KB-1	Bacteria	Firmicutes	Clostridia	Thermoanaerobacterales	Thermoanaerobacteraceae	Caldanaerobacter	hypothetical protein	cas3	COG1203	CAS-I			
29437..29995	-	ERM92164.1	AXDC01000014.1	Caldanaerobacter subterraneus subsp. yonseiensis KB-1	Bacteria	Firmicutes	Clostridia	Thermoanaerobacterales	Thermoanaerobacteraceae	Caldanaerobacter	hypothetical protein	cas3	COG1203	CAS-I			
30177..30900	-	ERM92165.1	AXDC01000014.1	Caldanaerobacter subterraneus subsp. yonseiensis KB-1	Bacteria	Firmicutes	Clostridia	Thermoanaerobacterales	Thermoanaerobacteraceae	Caldanaerobacter	CRISPR-associated protein Cas5	cas5	cd09693	CAS-I			
30896..31868	-	ERM92166.1	AXDC01000014.1	Caldanaerobacter subterraneus subsp. yonseiensis KB-1	Bacteria	Firmicutes	Clostridia	Thermoanaerobacterales	Thermoanaerobacteraceae	Caldanaerobacter	CRISPR-associated protein DevR	cas7	cd09687	CAS-I-B			
31848..33375	-	ERM92167.1	AXDC01000014.1	Caldanaerobacter subterraneus subsp. yonseiensis KB-1	Bacteria	Firmicutes	Clostridia	Thermoanaerobacterales	Thermoanaerobacteraceae	Caldanaerobacter	hypothetical protein	cas8b1	mkCas0195	CAS-I-B			
33367..34150	-	ERM92131.1	AXDC01000014.1	Caldanaerobacter subterraneus subsp. yonseiensis KB-1	Bacteria	Firmicutes	Clostridia	Thermoanaerobacterales	Thermoanaerobacteraceae	Caldanaerobacter	CRISPR-associated protein Cas6	cas6	cd09652	CAS-I,CAS-III			
34187..34568	-	ERM92132.1	AXDC01000014.1	Caldanaerobacter subterraneus subsp. yonseiensis KB-1	Bacteria	Firmicutes	Clostridia	Thermoanaerobacterales	Thermoanaerobacteraceae	Caldanaerobacter	hypothetical protein	csx15	cd09766	CAS-I,CAS-III			
34590..35487	-	ERM92133.1	AXDC01000014.1	Caldanaerobacter subterraneus subsp. yonseiensis KB-1	Bacteria	Firmicutes	Clostridia	Thermoanaerobacterales	Thermoanaerobacteraceae	Caldanaerobacter	LuxR family transcriptional regulator	csx1	cd09741	CAS-III			
35580..36417	-	ERM92134.1	AXDC01000014.1	Caldanaerobacter subterraneus subsp. yonseiensis KB-1	Bacteria	Firmicutes	Clostridia	Thermoanaerobacterales	Thermoanaerobacteraceae	Caldanaerobacter	CRISPR-associated protein Cas6	cas6	COG1583	CAS-I,CAS-III,CAS-IV			

186 CAS-I-B | KUK08303.1

47..551	+	KUK08294.1	LGEY01000075.1	Caldanaerobacter subterraneus	Bacteria	Firmicutes	Clostridia	Thermoanaerobacterales	Thermoanaerobacteraceae	Caldanaerobacter	hypothetical protein	csx1	cd09741	CAS-III			
579..960	+	KUK08295.1	LGEY01000075.1	Caldanaerobacter subterraneus	Bacteria	Firmicutes	Clostridia	Thermoanaerobacterales	Thermoanaerobacteraceae	Caldanaerobacter	hypothetical protein	csx15	cd09766	CAS-I,CAS-III			
997..1780	+	KUK08296.1	LGEY01000075.1	Caldanaerobacter subterraneus	Bacteria	Firmicutes	Clostridia	Thermoanaerobacterales	Thermoanaerobacteraceae	Caldanaerobacter	hypothetical protein	cas6	cd09652	CAS-I,CAS-III			
1772..3299	+	KUK08297.1	LGEY01000075.1	Caldanaerobacter subterraneus	Bacteria	Firmicutes	Clostridia	Thermoanaerobacterales	Thermoanaerobacteraceae	Caldanaerobacter	hypothetical protein	cas8b1	mkCas0195	CAS-I-B			
3279..4251	+	KUK08298.1	LGEY01000075.1	Caldanaerobacter subterraneus	Bacteria	Firmicutes	Clostridia	Thermoanaerobacterales	Thermoanaerobacteraceae	Caldanaerobacter	hypothetical protein	cas7	cd09687	CAS-I-B			
4247..4970	+	KUK08299.1	LGEY01000075.1	Caldanaerobacter subterraneus	Bacteria	Firmicutes	Clostridia	Thermoanaerobacterales	Thermoanaerobacteraceae	Caldanaerobacter	hypothetical protein	cas5	cd09693	CAS-I			
4948..7147	+	KUK08300.1	LGEY01000075.1	Caldanaerobacter subterraneus	Bacteria	Firmicutes	Clostridia	Thermoanaerobacterales	Thermoanaerobacteraceae	Caldanaerobacter	putative helicase	cas3HD,cas3	cd09641,COG1203	CAS-I			
7231..7744	+	KUK08301.1	LGEY01000075.1	Caldanaerobacter subterraneus	Bacteria	Firmicutes	Clostridia	Thermoanaerobacterales	Thermoanaerobacteraceae	Caldanaerobacter	RecB family exonuclease	cas4	pfam01930	CAS-I			
7775..8768	+	KUK08302.1	LGEY01000075.1	Caldanaerobacter subterraneus	Bacteria	Firmicutes	Clostridia	Thermoanaerobacterales	Thermoanaerobacteraceae	Caldanaerobacter	CRISPR-associated endonuclease Cas1	cas1	cd09722	CAS-I-B			
8803..9067	+	KUK08303.1	LGEY01000075.1	Caldanaerobacter subterraneus	Bacteria	Firmicutes	Clostridia	Thermoanaerobacterales	Thermoanaerobacteraceae	Caldanaerobacter	CRISPR-associated endoribonuclease Cas2	cas2	cd09725	CAS-I,CAS-II,CAS-III,CAS-V			

187 CAS-I-B | AIS53483.1

2268104..2273799	array		CP009170.1	Thermoanaerobacter kivui	Bacteria	Firmicutes	Clostridia	Thermoanaerobacterales	Thermoanaerobacteraceae	Thermoanaerobacter		CRISPR array			86	37	29
2274301..2274565	-	AIS53483.1	CP009170.1	Thermoanaerobacter kivui	Bacteria	Firmicutes	Clostridia	Thermoanaerobacterales	Thermoanaerobacteraceae	Thermoanaerobacter	CRISPR-associated endoribonuclease Cas2	cas2	cd09725	CAS-I,CAS-II,CAS-III,CAS-V			
2274601..2275594	-	AIS53484.1	CP009170.1	Thermoanaerobacter kivui	Bacteria	Firmicutes	Clostridia	Thermoanaerobacterales	Thermoanaerobacteraceae	Thermoanaerobacter	CRISPR-associated metal-dependent DNA-specific endonuclease Cas1	cas1	cd09722	CAS-I-B			
2275636..2276161	-	AIS53485.1	CP009170.1	Thermoanaerobacter kivui	Bacteria	Firmicutes	Clostridia	Thermoanaerobacterales	Thermoanaerobacteraceae	Thermoanaerobacter	CRISPR-associated exonuclease Cas4	cas4	pfam01930	CAS-I			
2276157..2278626	-	AIS53486.1	CP009170.1	Thermoanaerobacter kivui	Bacteria	Firmicutes	Clostridia	Thermoanaerobacterales	Thermoanaerobacteraceae	Thermoanaerobacter	CRISPR-associated helicase Cas3'	cas3HD,cas3	cd09641,COG1203	CAS-I			
2278580..2279381	-	AIS53487.1	CP009170.1	Thermoanaerobacter kivui	Bacteria	Firmicutes	Clostridia	Thermoanaerobacterales	Thermoanaerobacteraceae	Thermoanaerobacter	CRISPR-associated protein Cas5h	cas5	mkCas0191	CAS-I-B			
2279397..2280381	-	AIS53488.1	CP009170.1	Thermoanaerobacter kivui	Bacteria	Firmicutes	Clostridia	Thermoanaerobacterales	Thermoanaerobacteraceae	Thermoanaerobacter	CRISPR-associated RAMP protein Cas7/Csh2	cas7	cd09690	CAS-I-B			
2280385..2282494	-	AIS53489.1	CP009170.1	Thermoanaerobacter kivui	Bacteria	Firmicutes	Clostridia	Thermoanaerobacterales	Thermoanaerobacteraceae	Thermoanaerobacter	CRISPR-associated protein Cas8b/Csh1	cas8b1	pfam09484	CAS-I-B			

188 CAS-I-B | KKC28636.1

51264..51528	-	KKC28636.1	ABXP02000122.1	Caldanaerobacter subterraneus subsp. pacificus DSM 12653	Bacteria	Firmicutes	Clostridia	Thermoanaerobacterales	Thermoanaerobacteraceae	Caldanaerobacter	hypothetical protein	cas2	cd09725	CAS-I,CAS-II,CAS-III,CAS-V			
51563..52556	-	KKC28637.1	ABXP02000122.1	Caldanaerobacter subterraneus subsp. pacificus DSM 12653	Bacteria	Firmicutes	Clostridia	Thermoanaerobacterales	Thermoanaerobacteraceae	Caldanaerobacter	hypothetical protein	cas1	cd09722	CAS-I-B			
52598..52937	-	KKC28638.1	ABXP02000122.1	Caldanaerobacter subterraneus subsp. pacificus DSM 12653	Bacteria	Firmicutes	Clostridia	Thermoanaerobacterales	Thermoanaerobacteraceae	Caldanaerobacter	endonuclease	cas4	pfam01930	CAS-I			
53119..55588	-	KKC28639.1	ABXP02000122.1	Caldanaerobacter subterraneus subsp. pacificus DSM 12653	Bacteria	Firmicutes	Clostridia	Thermoanaerobacterales	Thermoanaerobacteraceae	Caldanaerobacter	helicase	cas3HD,cas3	cd09641,COG1203	CAS-I			
55542..56343	-	KKC28640.1	ABXP02000122.1	Caldanaerobacter subterraneus subsp. pacificus DSM 12653	Bacteria	Firmicutes	Clostridia	Thermoanaerobacterales	Thermoanaerobacteraceae	Caldanaerobacter	hypothetical protein	cas5	mkCas0191	CAS-I-B			
56359..57352	-	KKC28641.1	ABXP02000122.1	Caldanaerobacter subterraneus subsp. pacificus DSM 12653	Bacteria	Firmicutes	Clostridia	Thermoanaerobacterales	Thermoanaerobacteraceae	Caldanaerobacter	hypothetical protein	cas7	cd09690	CAS-I-B			
57356..59471	-	KKC28642.1	ABXP02000122.1	Caldanaerobacter subterraneus subsp. pacificus DSM 12653	Bacteria	Firmicutes	Clostridia	Thermoanaerobacterales	Thermoanaerobacteraceae	Caldanaerobacter	hypothetical protein	cas8b1	pfam09484	CAS-I-B			

189 CAS-I-B | ELZ42758.1

82440..83127	array		AOJE01000010.1	Halorubrum saccharovorum DSM 1137	Archaea	Euryarchaeota	Halobacteria	Haloferacales	Halorubraceae	Halorubrum		CRISPR array			11	36	29
83238..83502	-	ELZ42758.1	AOJE01000010.1	Halorubrum saccharovorum DSM 1137	Archaea	Euryarchaeota	Halobacteria	Haloferacales	Halorubraceae	Halorubrum	CRISPR-associated protein Cas2	cas2	COG1343	CAS-I,CAS-II,CAS-III			
83498..84491	-	ELZ42759.1	AOJE01000010.1	Halorubrum saccharovorum DSM 1137	Archaea	Euryarchaeota	Halobacteria	Haloferacales	Halorubraceae	Halorubrum	CRISPR-associated protein Cas1	cas1	cd09722	CAS-I-B			
84492..85032	-	ELZ42760.1	AOJE01000010.1	Halorubrum saccharovorum DSM 1137	Archaea	Euryarchaeota	Halobacteria	Haloferacales	Halorubraceae	Halorubrum	CRISPR-associated protein Cas4	cas4	pfam01930	CAS-I			
85100..87476	-	ELZ42761.1	AOJE01000010.1	Halorubrum saccharovorum DSM 1137	Archaea	Euryarchaeota	Halobacteria	Haloferacales	Halorubraceae	Halorubrum	CRISPR-associated Cas3 family protein	cas3HD,cas3,cas3	cd09641,COG1203,COG1203	CAS-I			
87757..88552	-	ELZ42762.1	AOJE01000010.1	Halorubrum saccharovorum DSM 1137	Archaea	Euryarchaeota	Halobacteria	Haloferacales	Halorubraceae	Halorubrum	CRISPR-associated protein Cas5, Hmari subtype	cas5	cls001430	CAS-I-B			
88551..89682	-	ELZ42763.1	AOJE01000010.1	Halorubrum saccharovorum DSM 1137	Archaea	Euryarchaeota	Halobacteria	Haloferacales	Halorubraceae	Halorubrum	Csh2 family CRISPR-associated protein	cas7	cd09690	CAS-I-B			
89674..91828	-	ELZ42764.1	AOJE01000010.1	Halorubrum saccharovorum DSM 1137	Archaea	Euryarchaeota	Halobacteria	Haloferacales	Halorubraceae	Halorubrum	CRISPR-associated protein, Csh1 family	cas8b1	cd09691	CAS-I-B			
91824..92637	-	ELZ42765.1	AOJE01000010.1	Halorubrum saccharovorum DSM 1137	Archaea	Euryarchaeota	Halobacteria	Haloferacales	Halorubraceae	Halorubrum	CRISPR-associated protein Cas6	cas6,cas6	COG1583,cd09652	CAS-I,CAS-III,CAS-IV			
93050..93212	array		AOJE01000010.1	Halorubrum saccharovorum DSM 1137	Archaea	Euryarchaeota	Halobacteria	Haloferacales	Halorubraceae	Halorubrum		CRISPR array			3	36	30
93213..98267	array		AOJE01000010.1	Halorubrum saccharovorum DSM 1137	Archaea	Euryarchaeota	Halobacteria	Haloferacales	Halorubraceae	Halorubrum		CRISPR array			77	36	30

190 CAS-III-B | OUQ05161.1

142196..143597	+	OUQ05153.1	NFLB01000007.1	[Clostridium] spiroforme	Bacteria	Firmicutes	Erysipelotrichia	Erysipelotrichales	Erysipelotrichaceae	Erysipelatoclostridium	hypothetical protein	cmr1gr7	mkCas0086	CAS-III-B			
143600..145184	+	OUQ05154.1	NFLB01000007.1	[Clostridium] spiroforme	Bacteria	Firmicutes	Erysipelotrichia	Erysipelotrichales	Erysipelotrichaceae	Erysipelatoclostridium	hypothetical protein	cas10,cas10	pfam12469,cd09679	CAS-III-B			
145189..147136	+	OUQ05155.1	NFLB01000007.1	[Clostridium] spiroforme	Bacteria	Firmicutes	Erysipelotrichia	Erysipelotrichales	Erysipelotrichaceae	Erysipelatoclostridium	type III-B CRISPR module RAMP protein Cmr4	cmr3gr5,cmr4gr7	pfam09700,cd09682	CAS-III-B			
147149..147524	+	OUQ05156.1	NFLB01000007.1	[Clostridium] spiroforme	Bacteria	Firmicutes	Erysipelotrichia	Erysipelotrichales	Erysipelotrichaceae	Erysipelatoclostridium	hypothetical protein	cmr5gr11	mkCas0098	CAS-III-B			
147526..148396	+	OUQ05157.1	NFLB01000007.1	[Clostridium] spiroforme	Bacteria	Firmicutes	Erysipelotrichia	Erysipelotrichales	Erysipelotrichaceae	Erysipelatoclostridium	type III-B CRISPR module RAMP protein Cmr6	cmr6gr7	cd09661	CAS-III-B			
148388..150548	+	OUQ05158.1	NFLB01000007.1	[Clostridium] spiroforme	Bacteria	Firmicutes	Erysipelotrichia	Erysipelotrichales	Erysipelotrichaceae	Erysipelatoclostridium	hypothetical protein	CorA	icity0111				
150872..151931	+	OUQ05159.1	NFLB01000007.1	[Clostridium] spiroforme	Bacteria	Firmicutes	Erysipelotrichia	Erysipelotrichales	Erysipelotrichaceae	Erysipelatoclostridium	hypothetical protein						
152269..154159	+	OUQ05160.1	NFLB01000007.1	[Clostridium] spiroforme	Bacteria	Firmicutes	Erysipelotrichia	Erysipelotrichales	Erysipelotrichaceae	Erysipelatoclostridium	CRISPR-associated endonuclease Cas1	RT,cas1	pfam00078,cd09722	CAS-I,CAS-III			
154161..154422	+	OUQ05161.1	NFLB01000007.1	[Clostridium] spiroforme	Bacteria	Firmicutes	Erysipelotrichia	Erysipelotrichales	Erysipelotrichaceae	Erysipelatoclostridium	CRISPR-associated endonuclease Cas2	cas2	cd09725	CAS-I,CAS-II,CAS-III,CAS-V			
154693..155150	array		NFLB01000007.1	[Clostridium] spiroforme	Bacteria	Firmicutes	Erysipelotrichia	Erysipelotrichales	Erysipelotrichaceae	Erysipelatoclostridium		CRISPR array			7	42	29
155200..155935	+	OUQ05162.1	NFLB01000007.1	[Clostridium] spiroforme	Bacteria	Firmicutes	Erysipelotrichia	Erysipelotrichales	Erysipelotrichaceae	Erysipelatoclostridium	CRISPR-associated endoribonuclease Cas6	cas6	COG1583	CAS-I,CAS-III,CAS-IV			
156309..156552	array		NFLB01000007.1	[Clostridium] spiroforme	Bacteria	Firmicutes	Erysipelotrichia	Erysipelotrichales	Erysipelotrichaceae	Erysipelatoclostridium		CRISPR array			4	42	29

191 CAS-III-D | RGG82470.1

8633..9282	array		QTXB01000028.1	Coprobacillus sp. AF17-11AC	Bacteria	Firmicutes	Erysipelotrichia	Erysipelotrichales	Erysipelotrichaceae	Coprobacillus		CRISPR array			10	36	32
9404..10961	+	RGG82468.1	QTXB01000028.1	Coprobacillus sp. AF17-11AC	Bacteria	Firmicutes	Erysipelotrichia	Erysipelotrichales	Erysipelotrichaceae	Coprobacillus	hypothetical protein	csx1	pfam09455	CAS-III			
10963..12856	+	RGG82469.1	QTXB01000028.1	Coprobacillus sp. AF17-11AC	Bacteria	Firmicutes	Erysipelotrichia	Erysipelotrichales	Erysipelotrichaceae	Coprobacillus	CRISPR-associated endonuclease Cas1	RT,cas1	pfam00078,cd09722	CAS-I,CAS-III			
12855..13119	+	RGG82470.1	QTXB01000028.1	Coprobacillus sp. AF17-11AC	Bacteria	Firmicutes	Erysipelotrichia	Erysipelotrichales	Erysipelotrichaceae	Coprobacillus	CRISPR-associated endonuclease Cas2	cas2	cd09725	CAS-I,CAS-II,CAS-III,CAS-V			
13559..13775	array		QTXB01000028.1	Coprobacillus sp. AF17-11AC	Bacteria	Firmicutes	Erysipelotrichia	Erysipelotrichales	Erysipelotrichaceae	Coprobacillus		CRISPR array			4	41	23
14344..14779	+	RGG82471.1	QTXB01000028.1	Coprobacillus sp. AF17-11AC	Bacteria	Firmicutes	Erysipelotrichia	Erysipelotrichales	Erysipelotrichaceae	Coprobacillus	hypothetical protein						
14876..15560	+	RGG82472.1	QTXB01000028.1	Coprobacillus sp. AF17-11AC	Bacteria	Firmicutes	Erysipelotrichia	Erysipelotrichales	Erysipelotrichaceae	Coprobacillus	hypothetical protein						
15731..17222	+	RGG82473.1	QTXB01000028.1	Coprobacillus sp. AF17-11AC	Bacteria	Firmicutes	Erysipelotrichia	Erysipelotrichales	Erysipelotrichaceae	Coprobacillus	hypothetical protein	cas10	mkCas0133	CAS-III			
17214..19215	+	RGG82474.1	QTXB01000028.1	Coprobacillus sp. AF17-11AC	Bacteria	Firmicutes	Erysipelotrichia	Erysipelotrichales	Erysipelotrichaceae	Coprobacillus	hypothetical protein	csm3gr7	mkCas0198	CAS-III-A,CAS-III-D			
19211..20423	+	RGG82475.1	QTXB01000028.1	Coprobacillus sp. AF17-11AC	Bacteria	Firmicutes	Erysipelotrichia	Erysipelotrichales	Erysipelotrichaceae	Coprobacillus	hypothetical protein	csm3gr7,csm3gr7	cd09726,pfam03787	CAS-III-D			
20416..20875	+	RGG82476.1	QTXB01000028.1	Coprobacillus sp. AF17-11AC	Bacteria	Firmicutes	Erysipelotrichia	Erysipelotrichales	Erysipelotrichaceae	Coprobacillus	hypothetical protein	csx19	cls001376	CAS-III-D			
20867..22610	+	RGG82477.1	QTXB01000028.1	Coprobacillus sp. AF17-11AC	Bacteria	Firmicutes	Erysipelotrichia	Erysipelotrichales	Erysipelotrichaceae	Coprobacillus	hypothetical protein	csm3gr7,csm3gr7	cls000253,cls000253	CAS-III-A,CAS-III-D			
22575..23286	+	RGG82478.1	QTXB01000028.1	Coprobacillus sp. AF17-11AC	Bacteria	Firmicutes	Erysipelotrichia	Erysipelotrichales	Erysipelotrichaceae	Coprobacillus	CRISPR-associated endoribonuclease Cas6	cas6	COG1583	CAS-I,CAS-III,CAS-IV			
23300..23660	+	RGG82479.1	QTXB01000028.1	Coprobacillus sp. AF17-11AC	Bacteria	Firmicutes	Erysipelotrichia	Erysipelotrichales	Erysipelotrichaceae	Coprobacillus	CRISPR-associated protein	csx20	cls000118	CAS-I,CAS-III			
24023..26795	+	RGG82480.1	QTXB01000028.1	Coprobacillus sp. AF17-11AC	Bacteria	Firmicutes	Erysipelotrichia	Erysipelotrichales	Erysipelotrichaceae	Coprobacillus	WYL domain-containing protein	WYL,WYL	pfam13280,pfam13280	CAS-I,CAS-III			

192 CAS-III-D | RGI19744.1

8764..9283	array		QTYY01000006.1	Ruminococcus sp. TF08-4	Bacteria	Firmicutes	Clostridia	Clostridiales	Ruminococcaceae	Ruminococcus		CRISPR array			8	41	29
9580..11482	-	RGI19592.1	QTYY01000006.1	Ruminococcus sp. TF08-4	Bacteria	Firmicutes	Clostridia	Clostridiales	Ruminococcaceae	Ruminococcus	CRISPR-associated endonuclease Cas1	RT,cas1	pfam00078,cd09722	CAS-I,CAS-III			
11509..11770	-	RGI19744.1	QTYY01000006.1	Ruminococcus sp. TF08-4	Bacteria	Firmicutes	Clostridia	Clostridiales	Ruminococcaceae	Ruminococcus	CRISPR-associated endonuclease Cas2	cas2	cd09725	CAS-I,CAS-II,CAS-III,CAS-V			
11785..12349	array		QTYY01000006.1	Ruminococcus sp. TF08-4	Bacteria	Firmicutes	Clostridia	Clostridiales	Ruminococcaceae	Ruminococcus		CRISPR array			9	38	29
12606..13329	-	RGI19593.1	QTYY01000006.1	Ruminococcus sp. TF08-4	Bacteria	Firmicutes	Clostridia	Clostridiales	Ruminococcaceae	Ruminococcus	CRISPR-associated endoribonuclease Cas6	cas6	COG1583	CAS-I,CAS-III,CAS-IV			
13389..15555	-	RGI19594.1	QTYY01000006.1	Ruminococcus sp. TF08-4	Bacteria	Firmicutes	Clostridia	Clostridiales	Ruminococcaceae	Ruminococcus	TIGR03986 family CRISPR-associated RAMP protein	csm3gr7	cls000253	CAS-III-A,CAS-III-D			
15556..16093	-	RGI19595.1	QTYY01000006.1	Ruminococcus sp. TF08-4	Bacteria	Firmicutes	Clostridia	Clostridiales	Ruminococcaceae	Ruminococcus	hypothetical protein	Cas11/Csx19	cluster5				
16089..17364	-	RGI19596.1	QTYY01000006.1	Ruminococcus sp. TF08-4	Bacteria	Firmicutes	Clostridia	Clostridiales	Ruminococcaceae	Ruminococcus	hypothetical protein	csm3gr7,csm3gr7	cd09726,cd09726	CAS-III-D			
17360..19532	-	RGI19597.1	QTYY01000006.1	Ruminococcus sp. TF08-4	Bacteria	Firmicutes	Clostridia	Clostridiales	Ruminococcaceae	Ruminococcus	hypothetical protein	csm3gr7,csx10gr5	mkCas0198,cls000715	CAS-III-A,CAS-III-D			
19513..21022	-	RGI19598.1	QTYY01000006.1	Ruminococcus sp. TF08-4	Bacteria	Firmicutes	Clostridia	Clostridiales	Ruminococcaceae	Ruminococcus	hypothetical protein	cas10	mkCas0133	CAS-III			
21034..21397	-	RGI19599.1	QTYY01000006.1	Ruminococcus sp. TF08-4	Bacteria	Firmicutes	Clostridia	Clostridiales	Ruminococcaceae	Ruminococcus	CRISPR-associated protein	csx20	cls000118	CAS-I,CAS-III			
21426..22968	-	RGI19600.1	QTYY01000006.1	Ruminococcus sp. TF08-4	Bacteria	Firmicutes	Clostridia	Clostridiales	Ruminococcaceae	Ruminococcus	hypothetical protein	csx1	pfam09455	CAS-III			

193 CAS-III-D | CDC93229.1

4762..5050	array		FR891835.1	Firmicutes bacterium CAG:227	Bacteria	Firmicutes						CRISPR array			5	36	29
5180..7622	-	CDC93219.1	FR891835.1	Firmicutes bacterium CAG:227	Bacteria	Firmicutes					putative uncharacterized protein	CARF	icity0119				
7870..8230	+	CDC93221.1	FR891835.1	Firmicutes bacterium CAG:227	Bacteria	Firmicutes					putative uncharacterized protein	csx20	cls000118	CAS-I,CAS-III			
8280..9786	+	CDC93223.1	FR891835.1	Firmicutes bacterium CAG:227	Bacteria	Firmicutes					putative uncharacterized protein	cas10	mkCas0133	CAS-III			
9757..11911	+	CDC93224.1	FR891835.1	Firmicutes bacterium CAG:227	Bacteria	Firmicutes					putative uncharacterized protein	csm3gr7,csm4gr5,csx10gr5	mkCas0198,mkCas0089,cls000715	CAS-III-A,CAS-III-D			
11907..13179	+	CDC93225.1	FR891835.1	Firmicutes bacterium CAG:227	Bacteria	Firmicutes					putative uncharacterized protein	csm3gr7,csm3gr7	cd09726,cd09726	CAS-III-D			
13175..13556	+	CDC93226.1	FR891835.1	Firmicutes bacterium CAG:227	Bacteria	Firmicutes					putative uncharacterized protein	Csx19	icity0015				
13558..15685	+	CDC93227.1	FR891835.1	Firmicutes bacterium CAG:227	Bacteria	Firmicutes					putative uncharacterized protein	csm3gr7	cls000253	CAS-III-A,CAS-III-D			
15687..16413	+	CDC93228.1	FR891835.1	Firmicutes bacterium CAG:227	Bacteria	Firmicutes					putative uncharacterized protein	cas6	COG1583	CAS-I,CAS-III,CAS-IV			
16581..17085	array		FR891835.1	Firmicutes bacterium CAG:227	Bacteria	Firmicutes						CRISPR array			8	37	30
17139..17400	+	CDC93229.1	FR891835.1	Firmicutes bacterium CAG:227	Bacteria	Firmicutes					cRISPR-associated endoribonuclease Cas2	cas2	cd09725	CAS-I,CAS-II,CAS-III,CAS-V			
17409..18036	+	CDC93230.1	FR891835.1	Firmicutes bacterium CAG:227	Bacteria	Firmicutes					cRISPR-associated endonuclease Cas1 2	RT	pfam00078	CAS-I,CAS-III			
18036..19314	+	CDC93231.1	FR891835.1	Firmicutes bacterium CAG:227	Bacteria	Firmicutes					cRISPR-associated endonuclease Cas1 2	RT,cas1	pfam00078,cd09722	CAS-I,CAS-III			
19506..19874	array		FR891835.1	Firmicutes bacterium CAG:227	Bacteria	Firmicutes						CRISPR array			6	39	29

194 CAS-III-D | CUN56512.1

53426..55838	-	CUN55971.1	CYYV01000002.1	Fusicatenibacter saccharivorans	Bacteria	Firmicutes	Clostridia	Clostridiales	Lachnospiraceae	Fusicatenibacter	Uncharacterised protein	CARF	icity0119				
56025..56391	+	CUN56023.1	CYYV01000002.1	Fusicatenibacter saccharivorans	Bacteria	Firmicutes	Clostridia	Clostridiales	Lachnospiraceae	Fusicatenibacter	Uncharacterised protein	csx20	cls000118	CAS-I,CAS-III			
56410..57946	+	CUN56097.1	CYYV01000002.1	Fusicatenibacter saccharivorans	Bacteria	Firmicutes	Clostridia	Clostridiales	Lachnospiraceae	Fusicatenibacter	CRISPR-associated protein Cas10/Csm1%2C subtype III-A/MTUBE	cas10	mkCas0133	CAS-III			
57936..60066	+	CUN56168.1	CYYV01000002.1	Fusicatenibacter saccharivorans	Bacteria	Firmicutes	Clostridia	Clostridiales	Lachnospiraceae	Fusicatenibacter	CRISPR-associated RAMP protein%2C Csx10 family	csm3gr7,csx10gr5	mkCas0198,cls000715	CAS-III-A,CAS-III-D			
60062..61430	+	CUN56243.1	CYYV01000002.1	Fusicatenibacter saccharivorans	Bacteria	Firmicutes	Clostridia	Clostridiales	Lachnospiraceae	Fusicatenibacter	CRISPR-associated RAMP protein%2C SSO1426 family	csm3gr7,csm3gr7	cd09726,cd09726	CAS-III-D			
61435..61807	+	CUN56311.1	CYYV01000002.1	Fusicatenibacter saccharivorans	Bacteria	Firmicutes	Clostridia	Clostridiales	Lachnospiraceae	Fusicatenibacter	Uncharacterised protein	Csx19	icity0015				
61808..63626	+	CUN56377.1	CYYV01000002.1	Fusicatenibacter saccharivorans	Bacteria	Firmicutes	Clostridia	Clostridiales	Lachnospiraceae	Fusicatenibacter	CRISPR-associated protein	csm3gr7,csm3gr7	cls000253,cls000253	CAS-III-A,CAS-III-D			
63628..64351	+	CUN56445.1	CYYV01000002.1	Fusicatenibacter saccharivorans	Bacteria	Firmicutes	Clostridia	Clostridiales	Lachnospiraceae	Fusicatenibacter	Uncharacterized protein predicted to be involved in DNA repair (RAMP superfamily)	cas6	COG1583	CAS-I,CAS-III,CAS-IV			
64533..65386	array		CYYV01000002.1	Fusicatenibacter saccharivorans	Bacteria	Firmicutes	Clostridia	Clostridiales	Lachnospiraceae	Fusicatenibacter		CRISPR array			13	39	29
65408..65669	+	CUN56512.1	CYYV01000002.1	Fusicatenibacter saccharivorans	Bacteria	Firmicutes	Clostridia	Clostridiales	Lachnospiraceae	Fusicatenibacter	CRISPR-associated endoribonuclease Cas2	cas2	cd09725	CAS-I,CAS-II,CAS-III,CAS-V			
65676..67575	+	CUN56577.1	CYYV01000002.1	Fusicatenibacter saccharivorans	Bacteria	Firmicutes	Clostridia	Clostridiales	Lachnospiraceae	Fusicatenibacter	Group II intron-encoded protein ltrA	RT,cas1	pfam00078,cd09722	CAS-I,CAS-III			
67767..68125	array		CYYV01000002.1	Fusicatenibacter saccharivorans	Bacteria	Firmicutes	Clostridia	Clostridiales	Lachnospiraceae	Fusicatenibacter		CRISPR array			6	37	29

195 CAS-VI-A | WP_090127494.1

102556..102791	array		NZ_FCNR01000048.1	Eubacteriaceae bacterium CHKCI004	Bacteria	Firmicutes	Clostridia	Clostridiales	Eubacteriaceae			CRISPR array			4	38	30
102881..103142	+	WP_090127494.1	NZ_FCNR01000048.1	Eubacteriaceae bacterium CHKCI004	Bacteria	Firmicutes	Clostridia	Clostridiales	Eubacteriaceae		CRISPR-associated endonuclease Cas2	cas2	cd09725	CAS-I,CAS-II,CAS-III,CAS-V			
103162..105070	+	WP_090127495.1	NZ_FCNR01000048.1	Eubacteriaceae bacterium CHKCI004	Bacteria	Firmicutes	Clostridia	Clostridiales	Eubacteriaceae		type I-B CRISPR-associated endonuclease Cas1	RT,cas1	pfam00078,cd09722	CAS-I,CAS-III			
105345..105568	array		NZ_FCNR01000048.1	Eubacteriaceae bacterium CHKCI004	Bacteria	Firmicutes	Clostridia	Clostridiales	Eubacteriaceae			CRISPR array			4	37	28
105978..110100	+	WP_090127496.1	NZ_FCNR01000048.1	Eubacteriaceae bacterium CHKCI004	Bacteria	Firmicutes	Clostridia	Clostridiales	Eubacteriaceae		hypothetical protein	cas13a	cas13a	CAS-VI-A			

196 CAS-III-B | KXT61353.1

304043..304772	+	KXT61339.1	KQ969037.1	Streptococcus oralis	Bacteria	Firmicutes	Bacilli	Lactobacillales	Streptococcaceae	Streptococcus	CRISPR repeat RNA endoribonuclease Cas6	cas6	COG1583	CAS-I,CAS-III,CAS-IV			
304781..305978	+	KXT61340.1	KQ969037.1	Streptococcus oralis	Bacteria	Firmicutes	Bacilli	Lactobacillales	Streptococcaceae	Streptococcus	hypothetical protein	cmr1gr7	mkCas0086	CAS-III-B			
305978..307580	+	KXT61341.1	KQ969037.1	Streptococcus oralis	Bacteria	Firmicutes	Bacilli	Lactobacillales	Streptococcaceae	Streptococcus	CRISPR-associated RAMP Cmr2	cas10,cas10	pfam12469,cd09679	CAS-III-B			
307579..308869	+	KXT61342.1	KQ969037.1	Streptococcus oralis	Bacteria	Firmicutes	Bacilli	Lactobacillales	Streptococcaceae	Streptococcus	CRISPR-associated RAMP Cmr3	cmr3gr5,cmr3gr5	pfam09700,pfam09700	CAS-III-B			
308879..309593	+	KXT61343.1	KQ969037.1	Streptococcus oralis	Bacteria	Firmicutes	Bacilli	Lactobacillales	Streptococcaceae	Streptococcus	CRISPR-associated RAMP Cmr4	cmr4gr7,cmr4gr7	cd09682,cd09682	CAS-III-B			
309696..309987	+	KXT61344.1	KQ969037.1	Streptococcus oralis	Bacteria	Firmicutes	Bacilli	Lactobacillales	Streptococcaceae	Streptococcus	hypothetical protein	cmr5gr11	mkCas0098	CAS-III-B			
309989..310799	+	KXT61345.1	KQ969037.1	Streptococcus oralis	Bacteria	Firmicutes	Bacilli	Lactobacillales	Streptococcaceae	Streptococcus	CRISPR-associated RAMP Cmr6	cmr6gr7	cd09661	CAS-III-B			
310813..312013	+	KXT61346.1	KQ969037.1	Streptococcus oralis	Bacteria	Firmicutes	Bacilli	Lactobacillales	Streptococcaceae	Streptococcus	hypothetical protein						
311987..312779	+	KXT61347.1	KQ969037.1	Streptococcus oralis	Bacteria	Firmicutes	Bacilli	Lactobacillales	Streptococcaceae	Streptococcus	hypothetical protein						
312808..312958	+	KXT61348.1	KQ969037.1	Streptococcus oralis	Bacteria	Firmicutes	Bacilli	Lactobacillales	Streptococcaceae	Streptococcus	hypothetical protein						
312958..313471	+	KXT61349.1	KQ969037.1	Streptococcus oralis	Bacteria	Firmicutes	Bacilli	Lactobacillales	Streptococcaceae	Streptococcus	hypothetical protein						
313678..313828	+	KXT61350.1	KQ969037.1	Streptococcus oralis	Bacteria	Firmicutes	Bacilli	Lactobacillales	Streptococcaceae	Streptococcus	hypothetical protein	cas1	cd09722	CAS-I-B			
313981..314266	+	KXT61351.1	KQ969037.1	Streptococcus oralis	Bacteria	Firmicutes	Bacilli	Lactobacillales	Streptococcaceae	Streptococcus	hypothetical protein	cas1	cd09634	CAS-I,CAS-II,CAS-III,CAS-V			
314431..315216	array		KQ969037.1	Streptococcus oralis	Bacteria	Firmicutes	Bacilli	Lactobacillales	Streptococcaceae	Streptococcus		CRISPR array			11	47	28
315304..316780	+	KXT61352.1	KQ969037.1	Streptococcus oralis	Bacteria	Firmicutes	Bacilli	Lactobacillales	Streptococcaceae	Streptococcus	hypothetical protein	CorA	icity0111				
316780..317041	+	KXT61353.1	KQ969037.1	Streptococcus oralis	Bacteria	Firmicutes	Bacilli	Lactobacillales	Streptococcaceae	Streptococcus	CRISPR-associated protein Cas2	cas2	cd09725	CAS-I,CAS-II,CAS-III,CAS-V			
317286..318344	array		KQ969037.1	Streptococcus oralis	Bacteria	Firmicutes	Bacilli	Lactobacillales	Streptococcaceae	Streptococcus		CRISPR array			15	44	29

197 CAS-III-B | EGU65364.1

30452..31181	+	EGU65408.1	AFUB01000042.1	Streptococcus mitis bv. 2 str. SK95	Bacteria	Firmicutes	Bacilli	Lactobacillales	Streptococcaceae	Streptococcus	CRISPR-associated endoribonuclease Cas6	cas6	COG1583	CAS-I,CAS-III,CAS-IV			
31196..32252	+	EGU65363.1	AFUB01000042.1	Streptococcus mitis bv. 2 str. SK95	Bacteria	Firmicutes	Bacilli	Lactobacillales	Streptococcaceae	Streptococcus	hypothetical protein	cmr1gr7	mkCas0086	CAS-III-B			
32386..33991	+	EGU65414.1	AFUB01000042.1	Streptococcus mitis bv. 2 str. SK95	Bacteria	Firmicutes	Bacilli	Lactobacillales	Streptococcaceae	Streptococcus	CRISPR-associated protein, Cmr2 family	cas10,cas10	pfam12469,cd09679	CAS-III-B			
33990..35292	+	EGU65404.1	AFUB01000042.1	Streptococcus mitis bv. 2 str. SK95	Bacteria	Firmicutes	Bacilli	Lactobacillales	Streptococcaceae	Streptococcus	hypothetical protein	cmr3gr5	pfam09700	CAS-III-B			

35295..35997	+	EGU65455.1	AFUB01000042.1	Streptococcus mitis bv. 2 str. SK95	Bacteria	Firmicutes	Bacilli	Lactobacillales	Streptococcaceae	Streptococcus	CRISPR-associated RAMP protein, Cmr4 family	cmr4gr7,cmr4gr7	cd09682,cd09682	CAS-III-B			
35989..36403	+	EGU65430.1	AFUB01000042.1	Streptococcus mitis bv. 2 str. SK95	Bacteria	Firmicutes	Bacilli	Lactobacillales	Streptococcaceae	Streptococcus	hypothetical protein	cmr5gr11	mkCas0098	CAS-III-B			
36405..37242	+	EGU65456.1	AFUB01000042.1	Streptococcus mitis bv. 2 str. SK95	Bacteria	Firmicutes	Bacilli	Lactobacillales	Streptococcaceae	Streptococcus	CRISPR-associated RAMP protein, Cmr6 family	cmr6gr7	cd09661	CAS-III-B			
37256..39401	+	EGU65403.1	AFUB01000042.1	Streptococcus mitis bv. 2 str. SK95	Bacteria	Firmicutes	Bacilli	Lactobacillales	Streptococcaceae	Streptococcus	hypothetical protein						
39401..39914	+	EGU65386.1	AFUB01000042.1	Streptococcus mitis bv. 2 str. SK95	Bacteria	Firmicutes	Bacilli	Lactobacillales	Streptococcaceae	Streptococcus	hypothetical protein						
39938..40709	+	EGU65381.1	AFUB01000042.1	Streptococcus mitis bv. 2 str. SK95	Bacteria	Firmicutes	Bacilli	Lactobacillales	Streptococcaceae	Streptococcus	CRISPR-associated endonuclease Cas1	cas1	cd09634	CAS-I,CAS-II,CAS-III,CAS-V			
40874..42030	array		AFUB01000042.1	Streptococcus mitis bv. 2 str. SK95	Bacteria	Firmicutes	Bacilli	Lactobacillales	Streptococcaceae	Streptococcus		CRISPR array			16	46	29
42117..43593	+	EGU65365.1	AFUB01000042.1	Streptococcus mitis bv. 2 str. SK95	Bacteria	Firmicutes	Bacilli	Lactobacillales	Streptococcaceae	Streptococcus	conserved domain protein	CorA	icity0111				
43593..43854	+	EGU65364.1	AFUB01000042.1	Streptococcus mitis bv. 2 str. SK95	Bacteria	Firmicutes	Bacilli	Lactobacillales	Streptococcaceae	Streptococcus	CRISPR-associated protein Cas2	cas2	cd09725	CAS-I,CAS-II,CAS-III,CAS-V			
44713..45505	+	EGU65378.1	AFUB01000042.1	Streptococcus mitis bv. 2 str. SK95	Bacteria	Firmicutes	Bacilli	Lactobacillales	Streptococcaceae	Streptococcus	Cof-like hydrolase						
45504..46323	+	EGU65358.1	AFUB01000042.1	Streptococcus mitis bv. 2 str. SK95	Bacteria	Firmicutes	Bacilli	Lactobacillales	Streptococcaceae	Streptococcus	Cof-like hydrolase						
46326..47634	+	EGU65349.1	AFUB01000042.1	Streptococcus mitis bv. 2 str. SK95	Bacteria	Firmicutes	Bacilli	Lactobacillales	Streptococcaceae	Streptococcus	signal recognition particle-docking protein FtsY						
47678..49166	-	EGU65347.1	AFUB01000042.1	Streptococcus mitis bv. 2 str. SK95	Bacteria	Firmicutes	Bacilli	Lactobacillales	Streptococcaceae	Streptococcus	glucose-6-phosphate dehydrogenase						
49315..50056	-	EGU65427.1	AFUB01000042.1	Streptococcus mitis bv. 2 str. SK95	Bacteria	Firmicutes	Bacilli	Lactobacillales	Streptococcaceae	Streptococcus	arginine ABC transporter, ATP-binding protein ArtM	AbiEii,Cas_RecF	icity0065,cluster17				
198 partial| KXT86661.1																	
521158..521419	-	KXT86661.1	KQ969551.1	Streptococcus oralis	Bacteria	Firmicutes	Bacilli	Lactobacillales	Streptococcaceae	Streptococcus	CRISPR-associated protein Cas2	cas2	cd09725	CAS-I,CAS-II,CAS-III,CAS-V			
521419..522166	-	KXT86662.1	KQ969551.1	Streptococcus oralis	Bacteria	Firmicutes	Bacilli	Lactobacillales	Streptococcaceae	Streptococcus	hypothetical protein	CorA	icity0111				
199 CAS-VI-A | SFB66474.1																	
6257..10289	+	SFB66467.1	FOKR01000002.1	Butyrivibrio sp. YAB3001	Bacteria	Firmicutes	Clostridia	Clostridiales	Lachnospiraceae	Butyrivibrio	hypothetical protein	cas13a	cas13a	CAS-VI-A			
10440..11302	array		FOKR01000002.1	Butyrivibrio sp. YAB3001	Bacteria	Firmicutes	Clostridia	Clostridiales	Lachnospiraceae	Butyrivibrio		CRISPR array			13	32	36
11477..12524	+	SFB66471.1	FOKR01000002.1	Butyrivibrio sp. YAB3001	Bacteria	Firmicutes	Clostridia	Clostridiales	Lachnospiraceae	Butyrivibrio	CRISP-associated protein Cas1	cas1*	cd09722	CAS-I-B			
12537..12798	+	SFB66474.1	FOKR01000002.1	Butyrivibrio sp. YAB3001	Bacteria	Firmicutes	Clostridia	Clostridiales	Lachnospiraceae	Butyrivibrio	CRISPR-associated protein Cas2	cas2	cd09725	CAS-I,CAS-II,CAS-III,CAS-V			
200 CAS-VI-A | RGR54905.1																	
107860..108174	array		QRUJ01000006.1	[Eubacterium] rectale	Bacteria	Firmicutes	Clostridia	Clostridiales	Lachnospiraceae			CRISPR array			5	38	32
108543..112782	+	RGR54904.1	QRUJ01000006.1	[Eubacterium] rectale	Bacteria	Firmicutes	Clostridia	Clostridiales	Lachnospiraceae		hypothetical protein	cas13a	cas13a	CAS-VI-A			
112799..113060	+	RGR54905.1	QRUJ01000006.1	[Eubacterium] rectale	Bacteria	Firmicutes	Clostridia	Clostridiales	Lachnospiraceae		CRISPR-associated endonuclease Cas2	cas2	cd09725	CAS-I,CAS-II,CAS-III,CAS-V			
113078..114974	+	RGR54906.1	QRUJ01000006.1	[Eubacterium] rectale	Bacteria	Firmicutes	Clostridia	Clostridiales	Lachnospiraceae		CRISPR-associated endonuclease Cas1	RT,cas1	pfam00078,cd09722	CAS-I,CAS-III			
115140..115521	array		QRUJ01000006.1	[Eubacterium] rectale	Bacteria	Firmicutes	Clostridia	Clostridiales	Lachnospiraceae			CRISPR array			6	39	31
201 CAS-VI-A | WP_087253218.1																	
80478..84588	+	WP_087253216.1	NZ_NFHY01000010.1	Drancourtella sp. An57	Bacteria	Firmicutes	Clostridia	Clostridiales	Ruminococcaceae	Drancourtella	hypothetical protein	cas13a	cas13a	CAS-VI-A			
84591..84852	+	WP_087253218.1	NZ_NFHY01000010.1	Drancourtella sp. An57	Bacteria	Firmicutes	Clostridia	Clostridiales	Ruminococcaceae	Drancourtella	CRISPR-associated endonuclease Cas2	cas2	cd09725	CAS-I,CAS-II,CAS-III,CAS-V			
84864..85434	+	WP_087253220.1	NZ_NFHY01000010.1	Drancourtella sp. An57	Bacteria	Firmicutes	Clostridia	Clostridiales	Ruminococcaceae	Drancourtella	hypothetical protein	RT	pfam00078	CAS-I,CAS-III			
85406..86759	+	WP_087253222.1	NZ_NFHY01000010.1	Drancourtella sp. An57	Bacteria	Firmicutes	Clostridia	Clostridiales	Ruminococcaceae	Drancourtella	CRISPR-associated endonuclease Cas1	RT,cas1	pfam00078,cd09722	CAS-I,CAS-III			
86966..87387	array		NZ_NFHY01000010.1	Drancourtella sp. An57	Bacteria	Firmicutes	Clostridia	Clostridiales	Ruminococcaceae	Drancourtella		CRISPR array			6	47	31
202 CAS-III-D | SCW73718.1																	
39808..40543	-	SCW73595.1	FMTN01000026.1	Lachnospiraceae bacterium C10	Bacteria	Firmicutes	Clostridia	Clostridiales	Lachnospiraceae		CRISPR-associated endoribonuclease Cas6	cas6	COG1583	CAS-I,CAS-III,CAS-IV			
40724..41087	+	SCW73608.1	FMTN01000026.1	Lachnospiraceae bacterium C10	Bacteria	Firmicutes	Clostridia	Clostridiales	Lachnospiraceae		hypothetical protein	csx20	cls000118	CAS-I,CAS-III			
41279..41975	+	SCW73621.1	FMTN01000026.1	Lachnospiraceae bacterium C10	Bacteria	Firmicutes	Clostridia	Clostridiales	Lachnospiraceae		hypothetical protein	C3a43	cluster43				
41993..43490	+	SCW73633.1	FMTN01000026.1	Lachnospiraceae bacterium C10	Bacteria	Firmicutes	Clostridia	Clostridiales	Lachnospiraceae		CRISPR-associated (Cas) DxTHG family protein	CARF	icity0119				
43511..45170	+	SCW73646.1	FMTN01000026.1	Lachnospiraceae bacterium C10	Bacteria	Firmicutes	Clostridia	Clostridiales	Lachnospiraceae		hypothetical protein	cas10	mkCas0133	CAS-III			
45157..47359	+	SCW73658.1	FMTN01000026.1	Lachnospiraceae bacterium C10	Bacteria	Firmicutes	Clostridia	Clostridiales	Lachnospiraceae		CRISPR/Cas system CSM-associated protein Csm3, group 7 of RAMP superfamily	csm3gr7,csx10gr5	mkCas0198,cls000715	CAS-III-A,CAS-III-D			
47361..48687	+	SCW73674.1	FMTN01000026.1	Lachnospiraceae bacterium C10	Bacteria	Firmicutes	Clostridia	Clostridiales	Lachnospiraceae		CRISPR/Cas system CSM-associated protein Csm3, group 7 of RAMP superfamily	csm3gr7,csm3gr7	cd09726,cd09726	CAS-III-D			
48683..49160	+	SCW73687.1	FMTN01000026.1	Lachnospiraceae bacterium C10	Bacteria	Firmicutes	Clostridia	Clostridiales	Lachnospiraceae		hypothetical protein	Cas11/Csx19	cluster5				
49273..51037	+	SCW73700.1	FMTN01000026.1	Lachnospiraceae bacterium C10	Bacteria	Firmicutes	Clostridia	Clostridiales	Lachnospiraceae		CRISPR-associated protein	csm3gr7,csm3gr7	cls000253,cls000253	CAS-III-A,CAS-III-D			
51085..51346	+	SCW73718.1	FMTN01000026.1	Lachnospiraceae bacterium C10	Bacteria	Firmicutes	Clostridia	Clostridiales	Lachnospiraceae		CRISPR-associated protein Cas2	cas2	cd09725	CAS-I,CAS-II,CAS-III,CAS-V			
51326..53252	+	SCW73731.1	FMTN01000026.1	Lachnospiraceae bacterium C10	Bacteria	Firmicutes	Clostridia	Clostridiales	Lachnospiraceae		CRISPR-associated endonuclease Cas1	RT,cas1	pfam00078,cd09722	CAS-I,CAS-III			
53564..54087	array		FMTN01000026.1	Lachnospiraceae bacterium C10	Bacteria	Firmicutes	Clostridia	Clostridiales	Lachnospiraceae			CRISPR array			8	41	29
203 CAS-III-D | EFU76455.1																	
1666272..1666511	array		GL622296.1	Lachnoanaerobaculum saburreum DSM 3986	Bacteria	Firmicutes	Clostridia	Clostridiales	Lachnospiraceae	Lachnoanaerobaculum		CRISPR array			4	38	31
1667102..1667453	+	EFU76493.1	GL622296.1	Lachnoanaerobaculum saburreum DSM 3986	Bacteria	Firmicutes	Clostridia	Clostridiales	Lachnospiraceae	Lachnoanaerobaculum	CRISPR-associated DxTHG motif protein	csx1	cd09732	CAS-III			
1667430..1668009	+	EFU76494.1	GL622296.1	Lachnoanaerobaculum saburreum DSM 3986	Bacteria	Firmicutes	Clostridia	Clostridiales	Lachnospiraceae	Lachnoanaerobaculum	hypothetical protein	Csx1	cluster2				
1669598..1671155	+	EFU76445.1	GL622296.1	Lachnoanaerobaculum saburreum DSM 3986	Bacteria	Firmicutes	Clostridia	Clostridiales	Lachnospiraceae	Lachnoanaerobaculum	hypothetical protein	csx1	cd09732	CAS-III			
1671183..1672914	+	EFU76446.1	GL622296.1	Lachnoanaerobaculum saburreum DSM 3986	Bacteria	Firmicutes	Clostridia	Clostridiales	Lachnospiraceae	Lachnoanaerobaculum	hypothetical protein	cas10	mkCas0133	CAS-III			
1672861..1675195	+	EFU76447.1	GL622296.1	Lachnoanaerobaculum saburreum DSM 3986	Bacteria	Firmicutes	Clostridia	Clostridiales	Lachnospiraceae	Lachnoanaerobaculum	CRISPR-associated RAMP protein	csm3gr7,csx10gr5	mkCas0198,cls000715	CAS-III-A,CAS-III-D			
1675208..1676684	+	EFU76448.1	GL622296.1	Lachnoanaerobaculum saburreum DSM 3986	Bacteria	Firmicutes	Clostridia	Clostridiales	Lachnospiraceae	Lachnoanaerobaculum	CRISPR-associated RAMP protein	csm3gr7,csm3gr7	cd09726,cd09726	CAS-III-D			
1676658..1677195	+	EFU76449.1	GL622296.1	Lachnoanaerobaculum saburreum DSM 3986	Bacteria	Firmicutes	Clostridia	Clostridiales	Lachnospiraceae	Lachnoanaerobaculum	putative CRISPR-associated protein, TIGR03984 family	csx19	cls001376	CAS-III-D			
1677197..1679108	+	EFU76450.1	GL622296.1	Lachnoanaerobaculum saburreum DSM 3986	Bacteria	Firmicutes	Clostridia	Clostridiales	Lachnospiraceae	Lachnoanaerobaculum	CRISPR-associated protein	csm3gr7	cls000253	CAS-III-A,CAS-III-D			
1679200..1680706	+	EFU76451.1	GL622296.1	Lachnoanaerobaculum saburreum DSM 3986	Bacteria	Firmicutes	Clostridia	Clostridiales	Lachnospiraceae	Lachnoanaerobaculum	hypothetical protein	csx1	pfam09455	CAS-III			
1680719..1681025	+	EFU76452.1	GL622296.1	Lachnoanaerobaculum saburreum DSM 3986	Bacteria	Firmicutes	Clostridia	Clostridiales	Lachnospiraceae	Lachnoanaerobaculum	addiction module antitoxin, RelB/DinJ family						
1681122..1681997	array		GL622296.1	Lachnoanaerobaculum saburreum DSM 3986	Bacteria	Firmicutes	Clostridia	Clostridiales	Lachnospiraceae	Lachnoanaerobaculum		CRISPR array			13	40	30
1681899..1682019	+	EFU76453.1	GL622296.1	Lachnoanaerobaculum saburreum DSM 3986	Bacteria	Firmicutes	Clostridia	Clostridiales	Lachnospiraceae	Lachnoanaerobaculum	hypothetical protein						
1682448..1684368	-	EFU76454.1	GL622296.1	Lachnoanaerobaculum saburreum DSM 3986	Bacteria	Firmicutes	Clostridia	Clostridiales	Lachnospiraceae	Lachnoanaerobaculum	CRISPR-associated endonuclease Cas1	RT,cas1	pfam00078,cd09722	CAS-I,CAS-III			
1684376..1684637	-	EFU76455.1	GL622296.1	Lachnoanaerobaculum saburreum DSM 3986	Bacteria	Firmicutes	Clostridia	Clostridiales	Lachnospiraceae	Lachnoanaerobaculum	CRISPR-associated protein Cas2	cas2	cd09725	CAS-I,CAS-II,CAS-III,CAS-V			
1684689..1685706	array		GL622296.1	Lachnoanaerobaculum saburreum DSM 3986	Bacteria	Firmicutes	Clostridia	Clostridiales	Lachnospiraceae	Lachnoanaerobaculum		CRISPR array			15	40	30
204 CAS-III-D | AYA99694.1																	
1537755..1538541	array		CP032364.1	Lachnoanaerobaculum umeaense	Bacteria	Firmicutes	Clostridia	Clostridiales	Lachnospiraceae	Lachnoanaerobaculum		CRISPR array			12	38	30
1538605..1538866	+	AYA99694.1	CP032364.1	Lachnoanaerobaculum umeaense	Bacteria	Firmicutes	Clostridia	Clostridiales	Lachnospiraceae	Lachnoanaerobaculum	CRISPR-associated endonuclease Cas2	cas2	cd09725	CAS-I,CAS-II,CAS-III,CAS-V			
1538874..1540794	+	AYA99695.1	CP032364.1	Lachnoanaerobaculum umeaense	Bacteria	Firmicutes	Clostridia	Clostridiales	Lachnospiraceae	Lachnoanaerobaculum	type I-B CRISPR-associated endonuclease Cas1	RT,cas1	pfam00078,cd09722	CAS-I,CAS-III			
1540907..1542413	-	AYB00826.1	CP032364.1	Lachnoanaerobaculum umeaense	Bacteria	Firmicutes	Clostridia	Clostridiales	Lachnospiraceae	Lachnoanaerobaculum	hypothetical protein	csx1	pfam09455	CAS-III			
1544091..1545666	-	AYA99696.1	CP032364.1	Lachnoanaerobaculum umeaense	Bacteria	Firmicutes	Clostridia	Clostridiales	Lachnospiraceae	Lachnoanaerobaculum	IS1182 family transposase						
1545736..1547779	array		CP032364.1	Lachnoanaerobaculum umeaense	Bacteria	Firmicutes	Clostridia	Clostridiales	Lachnospiraceae	Lachnoanaerobaculum		CRISPR array			29	41	30
1547877..1548186	-	AYA99697.1	CP032364.1	Lachnoanaerobaculum umeaense	Bacteria	Firmicutes	Clostridia	Clostridiales	Lachnospiraceae	Lachnoanaerobaculum	type II toxin-antitoxin system RelB/DinJ family antitoxin						
1548739..1550749	-	AYA99698.1	CP032364.1	Lachnoanaerobaculum umeaense	Bacteria	Firmicutes	Clostridia	Clostridiales	Lachnospiraceae	Lachnoanaerobaculum	TIGR03986 family CRISPR-associated RAMP protein	csm3gr7	cls000253	CAS-III-A,CAS-III-D			
1550751..1551288	-	AYA99699.1	CP032364.1	Lachnoanaerobaculum umeaense	Bacteria	Firmicutes	Clostridia	Clostridiales	Lachnospiraceae	Lachnoanaerobaculum	hypothetical protein	csx19,csx19	mkCas0132,cls001376	CAS-III-D			
1551268..1552786	-	AYA99700.1	CP032364.1	Lachnoanaerobaculum umeaense	Bacteria	Firmicutes	Clostridia	Clostridiales	Lachnospiraceae	Lachnoanaerobaculum	CRISPR-associated protein	csm3gr7,csm3gr7	cd09726,cd09726	CAS-III-D			
1552799..1555268	-	AYA99701.1	CP032364.1	Lachnoanaerobaculum umeaense	Bacteria	Firmicutes	Clostridia	Clostridiales	Lachnospiraceae	Lachnoanaerobaculum	CRISPR-associated protein	csm3gr7,csx10gr5	mkCas0198,cls000715	CAS-III-A,CAS-III-D			
1555215..1556952	-	AYA99702.1	CP032364.1	Lachnoanaerobaculum umeaense	Bacteria	Firmicutes	Clostridia	Clostridiales	Lachnospiraceae	Lachnoanaerobaculum	hypothetical protein	cas10	mkCas0133	CAS-III			
1557006..1557363	-	AYA99703.1	CP032364.1	Lachnoanaerobaculum umeaense	Bacteria	Firmicutes	Clostridia	Clostridiales	Lachnospiraceae	Lachnoanaerobaculum	hypothetical protein	csx20	cls000118	CAS-I,CAS-III			
1558094..1559135	array		CP032364.1	Lachnoanaerobaculum umeaense	Bacteria	Firmicutes	Clostridia	Clostridiales	Lachnospiraceae	Lachnoanaerobaculum		CRISPR array			15	42	30
1559308..1561063	-	AYA99704.1	CP032364.1	Lachnoanaerobaculum umeaense	Bacteria	Firmicutes	Clostridia	Clostridiales	Lachnospiraceae	Lachnoanaerobaculum	IS1182 family transposase						
1561250..1562756	-	AYB00827.1	CP032364.1	Lachnoanaerobaculum umeaense	Bacteria	Firmicutes	Clostridia	Clostridiales	Lachnospiraceae	Lachnoanaerobaculum	hypothetical protein	csx1	pfam09455	CAS-III			
205 CAS-III-D | KXB53087.1																	
219899..220210	array		KQ959848.1	Lachnoanaerobaculum saburreum	Bacteria	Firmicutes	Clostridia	Clostridiales	Lachnospiraceae	Lachnoanaerobaculum		CRISPR array			5	40	30
220317..220701	array		KQ959848.1	Lachnoanaerobaculum saburreum	Bacteria	Firmicutes	Clostridia	Clostridiales	Lachnospiraceae	Lachnoanaerobaculum		CRISPR array			6	39	31
221536..221893	+	KXB53076.1	KQ959848.1	Lachnoanaerobaculum saburreum	Bacteria	Firmicutes	Clostridia	Clostridiales	Lachnospiraceae	Lachnoanaerobaculum	hypothetical protein	csx20	cls000118	CAS-I,CAS-III			
221919..222672	+	KXB53077.1	KQ959848.1	Lachnoanaerobaculum saburreum	Bacteria	Firmicutes	Clostridia	Clostridiales	Lachnospiraceae	Lachnoanaerobaculum	CRISPR-associated endoribonuclease Cas6	cas6	COG1583	CAS-I,CAS-III,CAS-IV			
222713..224258	+	KXB53078.1	KQ959848.1	Lachnoanaerobaculum saburreum	Bacteria	Firmicutes	Clostridia	Clostridiales	Lachnospiraceae	Lachnoanaerobaculum	hypothetical protein	csx1	cd09732	CAS-III			
224297..226022	+	KXB53079.1	KQ959848.1	Lachnoanaerobaculum saburreum	Bacteria	Firmicutes	Clostridia	Clostridiales	Lachnospiraceae	Lachnoanaerobaculum	hypothetical protein	cas10	mkCas0133	CAS-III			
225963..228459	+	KXB53080.1	KQ959848.1	Lachnoanaerobaculum saburreum	Bacteria	Firmicutes	Clostridia	Clostridiales	Lachnospiraceae	Lachnoanaerobaculum	CRISPR-associated RAMP protein	csm3gr7,csx10gr5	mkCas0198,cd09700	CAS-III-A,CAS-III-D			
228472..229990	+	KXB53081.1	KQ959848.1	Lachnoanaerobaculum saburreum	Bacteria	Firmicutes	Clostridia	Clostridiales	Lachnospiraceae	Lachnoanaerobaculum	CRISPR-associated RAMP protein	csm3gr7,csm3gr7	cd09726,cd09726	CAS-III-D			
229961..230495	+	KXB53082.1	KQ959848.1	Lachnoanaerobaculum saburreum	Bacteria	Firmicutes	Clostridia	Clostridiales	Lachnospiraceae	Lachnoanaerobaculum	CRISPR-associated protein family	csx19	cls001376	CAS-III-D			
230497..232495	+	KXB53083.1	KQ959848.1	Lachnoanaerobaculum saburreum	Bacteria	Firmicutes	Clostridia	Clostridiales	Lachnospiraceae	Lachnoanaerobaculum	CRISPR-associated protein	csm3gr7	cls000253	CAS-III-A,CAS-III-D			
232581..234087	+	KXB53084.1	KQ959848.1	Lachnoanaerobaculum saburreum	Bacteria	Firmicutes	Clostridia	Clostridiales	Lachnospiraceae	Lachnoanaerobaculum	hypothetical protein	csx1	pfam09455	CAS-III			
234100..234406	+	KXB53085.1	KQ959848.1	Lachnoanaerobaculum saburreum	Bacteria	Firmicutes	Clostridia	Clostridiales	Lachnospiraceae	Lachnoanaerobaculum	addiction module antitoxin, RelB/DinJ family						
234502..234998	array		KQ959848.1	Lachnoanaerobaculum saburreum	Bacteria	Firmicutes	Clostridia	Clostridiales	Lachnospiraceae	Lachnoanaerobaculum		CRISPR array			8	36	30
235460..237380	-	KXB53086.1	KQ959848.1	Lachnoanaerobaculum saburreum	Bacteria	Firmicutes	Clostridia	Clostridiales	Lachnospiraceae	Lachnoanaerobaculum	CRISPR-associated endonuclease Cas1	RT,cas1	pfam00078,cd09722	CAS-I,CAS-III			
237388..237649	-	KXB53087.1	KQ959848.1	Lachnoanaerobaculum saburreum	Bacteria	Firmicutes	Clostridia	Clostridiales	Lachnospiraceae	Lachnoanaerobaculum	CRISPR-associated protein Cas2	cas2	cd09725	CAS-I,CAS-II,CAS-III,CAS-V			
237698..238029	array		KQ959848.1	Lachnoanaerobaculum saburreum	Bacteria	Firmicutes	Clostridia	Clostridiales	Lachnospiraceae	Lachnoanaerobaculum		CRISPR array			5	45	30
206 CAS-III-D | EHO48117.1																	
27239..27747	array		JH591179.1	Lachnospiraceae bacterium oral taxon 082 str. F0431	Bacteria	Firmicutes	Clostridia	Clostridiales	Lachnospiraceae			CRISPR array			8	38	30
27476..27713	+	EHO48121.1	JH591179.1	Lachnospiraceae bacterium oral taxon 082 str. F0431	Bacteria	Firmicutes	Clostridia	Clostridiales	Lachnospiraceae		hypothetical protein						
27793..27919	+	EHO48116.1	JH591179.1	Lachnospiraceae bacterium oral taxon 082 str. F0431	Bacteria	Firmicutes	Clostridia	Clostridiales	Lachnospiraceae		hypothetical protein						
27896..28157	+	EHO48117.1	JH591179.1	Lachnospiraceae bacterium oral taxon 082 str. F0431	Bacteria	Firmicutes	Clostridia	Clostridiales	Lachnospiraceae		CRISPR-associated protein Cas2	cas2	cd09725	CAS-I,CAS-II,CAS-III,CAS-V			
28165..30085	+	EHO48118.1	JH591179.1	Lachnospiraceae bacterium oral taxon 082 str. F0431	Bacteria	Firmicutes	Clostridia	Clostridiales	Lachnospiraceae		CRISPR-associated endonuclease Cas1	RT,cas1	pfam00078,cd09722	CAS-I,CAS-III			
30809..31115	-	EHO48074.1	JH591179.1	Lachnospiraceae bacterium oral taxon 082 str. F0431	Bacteria	Firmicutes	Clostridia	Clostridiales	Lachnospiraceae		addiction module antitoxin, RelB/DinJ family						
31128..32673	-	EHO48075.1	JH591179.1	Lachnospiraceae bacterium oral taxon 082 str. F0431	Bacteria	Firmicutes	Clostridia	Clostridiales	Lachnospiraceae		hypothetical protein	csx1	pfam09455	CAS-III			
32726..34625	-	EHO48076.1	JH591179.1	Lachnospiraceae bacterium oral taxon 082 str. F0431	Bacteria	Firmicutes	Clostridia	Clostridiales	Lachnospiraceae		CRISPR-associated protein	csm3gr7	cls000253	CAS-III-A,CAS-III-D			
34614..35163	-	EHO48077.1	JH591179.1	Lachnospiraceae bacterium oral taxon 082 str. F0431	Bacteria	Firmicutes	Clostridia	Clostridiales	Lachnospiraceae		hypothetical protein	csx19	cls001376	CAS-III-D			
35137..36613	-	EHO48078.1	JH591179.1	Lachnospiraceae bacterium oral taxon 082 str. F0431	Bacteria	Firmicutes	Clostridia	Clostridiales	Lachnospiraceae		CRISPR-associated RAMP protein	csm3gr7,csm3gr7	cd09726,cd09726	CAS-III-D			
36626..38960	-	EHO48079.1	JH591179.1	Lachnospiraceae bacterium oral taxon 082 str. F0431	Bacteria	Firmicutes	Clostridia	Clostridiales	Lachnospiraceae		CRISPR-associated RAMP protein	csm3gr7,csx10gr5	mkCas0198,cls000715	CAS-III-A,CAS-III-D			
38907..40644	-	EHO48080.1	JH591179.1	Lachnospiraceae bacterium oral taxon 082 str. F0431	Bacteria	Firmicutes	Clostridia	Clostridiales	Lachnospiraceae		hypothetical protein	cas10	mkCas0133	CAS-III			
40672..42286	-	EHO48081.1	JH591179.1	Lachnospiraceae bacterium oral taxon 082 str. F0431	Bacteria	Firmicutes	Clostridia	Clostridiales	Lachnospiraceae		macrophage scavenger receptor	CARF	icity0119				
42336..43077	-	EHO48082.1	JH591179.1	Lachnospiraceae bacterium oral taxon 082 str. F0431	Bacteria	Firmicutes	Clostridia	Clostridiales	Lachnospiraceae		CRISPR-associated endoribonuclease Cas6	cas6	COG1583	CAS-I,CAS-III,CAS-IV			
43103..43460	-	EHO48083.1	JH591179.1	Lachnospiraceae bacterium oral taxon 082 str. F0431	Bacteria	Firmicutes	Clostridia	Clostridiales	Lachnospiraceae		hypothetical protein	csx20	cls000118	CAS-I,CAS-III			
207 CAS-III-D | OLA06490.1																	
40490..41231	-	OLA06483.1	MNSI01000033.1	Eubacterium sp. 38_16	Bacteria	Firmicutes	Clostridia	Clostridiales	Eubacteriaceae	Eubacterium	CRISPR-associated endoribonuclease Cas6	cas6	COG1583	CAS-I,CAS-III,CAS-IV			
41351..41621	+	OLA06484.1	MNSI01000033.1	Eubacterium sp. 38_16	Bacteria	Firmicutes	Clostridia	Clostridiales	Eubacteriaceae	Eubacterium	hypothetical protein	cas10	mkCas0133	CAS-III			
41655..43056	+	OLA06485.1	MNSI01000033.1	Eubacterium sp. 38_16	Bacteria	Firmicutes	Clostridia	Clostridiales	Eubacteriaceae	Eubacterium	hypothetical protein	cas10	mkCas0133	CAS-III			
43036..45400	+	OLA06486.1	MNSI01000033.1	Eubacterium sp. 38_16	Bacteria	Firmicutes	Clostridia	Clostridiales	Eubacteriaceae	Eubacterium	hypothetical protein	csm3gr7,csx10gr5	mkCas0198,cls000715	CAS-III-A,CAS-III-D			
45402..46752	+	OLA06487.1	MNSI01000033.1	Eubacterium sp. 38_16	Bacteria	Firmicutes	Clostridia	Clostridiales	Eubacteriaceae	Eubacterium	hypothetical protein	csm3gr7,csm3gr7,csm3gr7	cd09726,COG1337,pfam03787	CAS-III-D			
46751..47255	+	OLA06488.1	MNSI01000033.1	Eubacterium sp. 38_16	Bacteria	Firmicutes	Clostridia	Clostridiales	Eubacteriaceae	Eubacterium	hypothetical protein	csx19,csx19	mkCas0132,cls001376	CAS-III-D			
47267..49250	+	OLA06489.1	MNSI01000033.1	Eubacterium sp. 38_16	Bacteria	Firmicutes	Clostridia	Clostridiales	Eubacteriaceae	Eubacterium	CRISPR-associated RAMP family protein	csm3gr7	cls000253	CAS-III-A,CAS-III-D			
49640..50273	array		MNSI01000033.1	Eubacterium sp. 38_16	Bacteria	Firmicutes	Clostridia	Clostridiales	Eubacteriaceae	Eubacterium		CRISPR array			10	38	29
50353..51294	array		MNSI01000033.1	Eubacterium sp. 38_16	Bacteria	Firmicutes	Clostridia	Clostridiales	Eubacteriaceae	Eubacterium		CRISPR array			14	41	29
51362..53273	+	OLA06495.1	MNSI01000033.1	Eubacterium sp. 38_16	Bacteria	Firmicutes	Clostridia	Clostridiales	Eubacteriaceae	Eubacterium	CRISPR-associated endonuclease Cas1	RT,cas1	pfam00078,cd09722	CAS-I,CAS-III			
53299..53560	+	OLA06490.1	MNSI01000033.1	Eubacterium sp. 38_16	Bacteria	Firmicutes	Clostridia	Clostridiales	Eubacteriaceae	Eubacterium	CRISPR-associated endonuclease Cas2	cas2	cd09725	CAS-I,CAS-II,CAS-III,CAS-V			
53556..53919	+	OLA06491.1	MNSI01000033.1	Eubacterium sp. 38_16	Bacteria	Firmicutes	Clostridia	Clostridiales	Eubacteriaceae	Eubacterium	hypothetical protein	csx20	cls000118	CAS-I,CAS-III			
208 CAS-III-D | SOB72961.1																	
2457296..2458985	-	SOB72959.1	LT907978.1	Anaerobutyricum hallii	Bacteria	Firmicutes	Clostridia	Clostridiales	Lachnospiraceae	Anaerobutyricum	CRISPR-associated (Cas) DxTHG family	csx1	pfam09455	CAS-III			
2459007..2459370	-	SOB72960.1	LT907978.1	Anaerobutyricum hallii	Bacteria	Firmicutes	Clostridia	Clostridiales	Lachnospiraceae	Anaerobutyricum	Hypothetical protein	csx20	cls000118	CAS-I,CAS-III			
2459366..2459627	-	SOB72961.1	LT907978.1	Anaerobutyricum hallii	Bacteria	Firmicutes	Clostridia	Clostridiales	Lachnospiraceae	Anaerobutyricum	CRISPR-associated endoribonuclease Cas2 [cas2].	cas2	cd09725	CAS-I,CAS-II,CAS-III,CAS-V			
2459653..2461582	-	SOB72962.1	LT907978.1	Anaerobutyricum hallii	Bacteria	Firmicutes	Clostridia	Clostridiales	Lachnospiraceae	Anaerobutyricum	cas1: CRISPR-associated endonuclease Cas1	RT,cas1	pfam00078,cd09722	CAS-I,CAS-III			
2461821..2462679	-	SOB72963.1	LT907978.1	Anaerobutyricum hallii	Bacteria	Firmicutes	Clostridia	Clostridiales	Lachnospiraceae	Anaerobutyricum	Integrase, catalytic core						
2462629..2462977	-	SOB72964.1	LT907978.1	Anaerobutyricum hallii	Bacteria	Firmicutes	Clostridia	Clostridiales	Lachnospiraceae	Anaerobutyricum	Transposase						
2463065..2465456	array		LT907978.1	Anaerobutyricum hallii	Bacteria	Firmicutes	Clostridia	Clostridiales	Lachnospiraceae	Anaerobutyricum		CRISPR array			36	38	29
2465846..2467769	-	SOB72965.1	LT907978.1	Anaerobutyricum hallii	Bacteria	Firmicutes	Clostridia	Clostridiales	Lachnospiraceae	Anaerobutyricum	CRISPR type III-associated RAMP protein	csm3gr7	cls000253	CAS-III-A,CAS-III-D			
2467781..2468285	-	SOB72966.1	LT907978.1	Anaerobutyricum hallii	Bacteria	Firmicutes	Clostridia	Clostridiales	Lachnospiraceae	Anaerobutyricum	Hypothetical protein	csx19	cls001376	CAS-III-D			
2468284..2469634	-	SOB72967.1	LT907978.1	Anaerobutyricum hallii	Bacteria	Firmicutes	Clostridia	Clostridiales	Lachnospiraceae	Anaerobutyricum	CRISPR type III-associated RAMP protein	csm3gr7,csm3gr7,csm3gr7	cd09726,COG1337,pfam03787	CAS-III-D			
2469636..2472000	-	SOB72968.1	LT907978.1	Anaerobutyricum hallii	Bacteria	Firmicutes	Clostridia	Clostridiales	Lachnospiraceae	Anaerobutyricum	RAMP_I_III	csm3gr7,csx10gr5	mkCas0198,cls000715	CAS-III-A,CAS-III-D			

2471980..2473681	-	SOB72969.1	LT907978.1	Anaerobutyricum hallii	Bacteria	Firmicutes	Clostridia	Clostridiales	Lachnospiraceae	Anaerobutyricum	Hypothetical protein	cas10	mkCas0133	CAS-III			
2473801..2474542	+	SOB72970.1	LT907978.1	Anaerobutyricum hallii	Bacteria	Firmicutes	Clostridia	Clostridiales	Lachnospiraceae	Anaerobutyricum	CRISPR associated protein Cas6	cas6	COG1583	CAS-I,CAS-III,CAS-IV			
209 partial	CUP12250.1																
86633..86870	-	CUP12250.1	CZAY01000003.1	Dorea longicatena	Bacteria	Firmicutes	Clostridia	Clostridiales	Lachnospiraceae	Dorea	CRISPR-associated endoribonuclease Cas2	**cas2**	cd09725	CAS-I,CAS-II,CAS-III,CAS-V			
210 CAS-III-D	RGK37322.1																
9677..10469	+	RGK37312.1	QSQN01000041.1	Ruminococcus lactaris	Bacteria	Firmicutes	Clostridia	Clostridiales	Ruminococcaceae	Ruminococcus	HD domain-containing protein	cas3HD	cd09641	CAS-I			
10535..11996	+	RGK37313.1	QSQN01000041.1	Ruminococcus lactaris	Bacteria	Firmicutes	Clostridia	Clostridiales	Ruminococcaceae	Ruminococcus	hypothetical protein	csx1	pfam09455	CAS-III			
12020..12377	+	RGK37314.1	QSQN01000041.1	Ruminococcus lactaris	Bacteria	Firmicutes	Clostridia	Clostridiales	Ruminococcaceae	Ruminococcus	hypothetical protein	csx20	cls000118	CAS-I,CAS-III			
12400..13909	+	RGK37315.1	QSQN01000041.1	Ruminococcus lactaris	Bacteria	Firmicutes	Clostridia	Clostridiales	Ruminococcaceae	Ruminococcus	hypothetical protein	cas10	mkCas0133	CAS-III			
13892..15983	+	RGK37316.1	QSQN01000041.1	Ruminococcus lactaris	Bacteria	Firmicutes	Clostridia	Clostridiales	Ruminococcaceae	Ruminococcus	hypothetical protein	csm3gr7,csx10gr5	mkCas0198,cls000715	CAS-III-A,CAS-III-D			
15979..17305	+	RGK37317.1	QSQN01000041.1	Ruminococcus lactaris	Bacteria	Firmicutes	Clostridia	Clostridiales	Ruminococcaceae	Ruminococcus	hypothetical protein	csm3gr7,csm3gr7	cd09726,cd09726	CAS-III-D			
17304..17676	+	RGK37318.1	QSQN01000041.1	Ruminococcus lactaris	Bacteria	Firmicutes	Clostridia	Clostridiales	Ruminococcaceae	Ruminococcus	hypothetical protein	Csx19	icity0015				
17676..19677	+	RGK37319.1	QSQN01000041.1	Ruminococcus lactaris	Bacteria	Firmicutes	Clostridia	Clostridiales	Ruminococcaceae	Ruminococcus	TIGR03986 family CRISPR-associated RAMP protein	csm3gr7	cls000253	CAS-III-A,CAS-III-D			
19679..20402	+	RGK37320.1	QSQN01000041.1	Ruminococcus lactaris	Bacteria	Firmicutes	Clostridia	Clostridiales	Ruminococcaceae	Ruminococcus	CRISPR-associated endoribonuclease Cas6	cas6	COG1583	CAS-I,CAS-III,CAS-IV			
20420..20651	+	RGK37321.1	QSQN01000041.1	Ruminococcus lactaris	Bacteria	Firmicutes	Clostridia	Clostridiales	Ruminococcaceae	Ruminococcus	hypothetical protein						
20780..22591	array		QSQN01000041.1	Ruminococcus lactaris	Bacteria	Firmicutes	Clostridia	Clostridiales	Ruminococcaceae	Ruminococcus		CRISPR array			26	42	29
24618..24879	+	RGK37322.1	QSQN01000041.1	Ruminococcus lactaris	Bacteria	Firmicutes	Clostridia	Clostridiales	Ruminococcaceae	Ruminococcus	CRISPR-associated endonuclease Cas2	**cas2**	cd09725	CAS-I,CAS-II,CAS-III,CAS-V			
211 partial	HBZ63502.1																
304..564	+	HBZ63502.1	DOVI01000103	Lachnospiraceae bacterium isolate UBA11537	Bacteria	Lachnospiraceae bacterium (gut metagenome)					CRISPR-associated endoribonuclease Cas2	**cas2**	cd09725	CAS-I,CAS-II,CAS-III,CAS-V			
539..2494	+	HBZ63503.1	DOVI01000103	Lachnospiraceae bacterium isolate UBA11537	Bacteria	Lachnospiraceae bacterium (gut metagenome)					CRISPR-associated endoribonuclease Cas1	cas1	cd09722	CAS-I-B			
212 CAS-III-A/CAS-III-D	HAT88294.1																
59213..60169	array											CRISPR array			14	42	29
62392..62652	+	HAT88295.1	DMYD01000005	Roseburia sp. isolate UBA10666	Bacteria	Firmicutes	Clostridia	Clostridiales	Lachnospiraceae	Roseburia	CRISPR-associated endoribonuclease Cas1	cas1	cd09722	CAS-I-B			
60261..62175	+	HAT88294.1	DMYD01000005	Roseburia sp. isolate UBA10666	Bacteria	Firmicutes	Clostridia	Clostridiales	Lachnospiraceae	Roseburia	CRISPR-associated endoribonuclease Cas2	**cas2**	cd09725	CAS-I,CAS-II,CAS-III,CAS-V			
62832..63194	+	HAT88293.1	DMYD01000005	Roseburia sp. isolate UBA10666	Bacteria	Firmicutes	Clostridia	Clostridiales	Lachnospiraceae	Roseburia	hypothetical protein	csx20	cls000118	CAS-I,CAS-III			
63206..64616	+	HAT88292.1	DMYD01000005	Roseburia sp. isolate UBA10666	Bacteria	Firmicutes	Clostridia	Clostridiales	Lachnospiraceae	Roseburia	hypothetical protein						
64639..66171	+	HAT88291.1	DMYD01000005	Roseburia sp. isolate UBA10666	Bacteria	Firmicutes	Clostridia	Clostridiales	Lachnospiraceae	Roseburia	hypothetical protein	cas10	mkCas0133	CAS-III			
66095..68254	+	HAT88290.1	DMYD01000005	Roseburia sp. isolate UBA10666	Bacteria	Firmicutes	Clostridia	Clostridiales	Lachnospiraceae	Roseburia	hypothetical protein	csm3gr7,csx10gr5	mkCas0198,cd09700	CAS-III-A,CAS-III-D			
68251..69558	+	HAT88289.1	DMYD01000005	Roseburia sp. isolate UBA10666	Bacteria	Firmicutes	Clostridia	Clostridiales	Lachnospiraceae	Roseburia	hypothetical protein	csm3gr7,csm3gr7	cd09683,cd09683	CAS-III-A,CAS-III-D			
69555..69956	+	HAT88288.1	DMYD01000005	Roseburia sp. isolate UBA10666	Bacteria	Firmicutes	Clostridia	Clostridiales	Lachnospiraceae	Roseburia	hypothetical protein						
69959..71842	+	HAT88287.1	DMYD01000005	Roseburia sp. isolate UBA10666	Bacteria	Firmicutes	Clostridia	Clostridiales	Lachnospiraceae	Roseburia	TIGR03986 family CRISPR-associated RAMP protein	csm3gr7	cls000253	CAS-III-A,CAS-III-D			
71845..71567	+	HAT88286.1	DMYD01000005	Roseburia sp. isolate UBA10666	Bacteria	Firmicutes	Clostridia	Clostridiales	Lachnospiraceae	Roseburia	CRISPR-associated endoribonuclease Cas6	cas6	COG1583	CAS-I,CAS-III,CAS-IV			
72587..73666	-	HAT88285.1	DMYD01000005	Roseburia sp. isolate UBA10666	Bacteria	Firmicutes	Clostridia	Clostridiales	Lachnospiraceae	Roseburia	hypothetical protein	WYL	COG2378	CAS-I,CAS-III			
213 partial	EEG94368.1																
134..395	-	EEG94368.1	ACFY01000067.1	Roseburia inulinivorans DSM 16841	Bacteria	Firmicutes	Clostridia	Clostridiales	Lachnospiraceae	Roseburia	CRISPR-associated protein Cas2	**cas2**	cd09725	CAS-I,CAS-II,CAS-III,CAS-V			
403..1483	-	EEG94369.1	ACFY01000067.1	Roseburia inulinivorans DSM 16841	Bacteria	Firmicutes	Clostridia	Clostridiales	Lachnospiraceae	Roseburia	CRISPR-associated endonuclease Cas1	cas1	cd09722	CAS-I-B			
1483..2305	-	EEG94370.1	ACFY01000067.1	Roseburia inulinivorans DSM 16841	Bacteria	Firmicutes	Clostridia	Clostridiales	Lachnospiraceae	Roseburia	reverse transcriptase (RNA-dependent DNA polymerase)	RT	pfam00078	CAS-I,CAS-III			
2333..2633	array		ACFY01000067.1	Roseburia inulinivorans DSM 16841	Bacteria	Firmicutes	Clostridia	Clostridiales	Lachnospiraceae	Roseburia		CRISPR array			5	42	26
214 partial	CRL43258.1																
296..557	-	CRL43258.1	CVRS01000124.1	Roseburia inulinivorans	Bacteria	Firmicutes	Clostridia	Clostridiales	Lachnospiraceae	Roseburia	hypothetical protein	**cas2**	cd09725	CAS-I,CAS-II,CAS-III,CAS-V			
565..2467	-	CRL43259.1	CVRS01000124.1	Roseburia inulinivorans	Bacteria	Firmicutes	Clostridia	Clostridiales	Lachnospiraceae	Roseburia	hypothetical protein	RT,cas1	pfam00078,cd09722	CAS-I,CAS-III			
2495..3005	array		CVRS01000124.1	Roseburia inulinivorans	Bacteria	Firmicutes	Clostridia	Clostridiales	Lachnospiraceae	Roseburia		CRISPR array			8	39	29
215 partial	OPZ32671																
ND	ND	OPZ32671		Tenericutes bacterium ADurb.BinA124 - anaerobic digester metagenome	Bacteria	Tenericutes	unclassified Tenericutes				CRISPR-associated endoribonuclease Cas2	**cas2**	cd09725	CAS-I,CAS-II,CAS-III,CAS-V			
216 CAS-III-A	SET52351.1																
12567..13899	+	SET52155.1	FOIN01000016.1	[Clostridium] cocleatum	Bacteria	Firmicutes	Erysipelotrichia	Erysipelotrichales	Erysipelotrichaceae	Erysipelatoclostridium	CRISPR type III-A/MTUBE-associated protein Csm6	csm6	cd09746	CAS-III-A			
13909..16240	+	SET52184.1	FOIN01000016.1	[Clostridium] cocleatum	Bacteria	Firmicutes	Erysipelotrichia	Erysipelotrichales	Erysipelotrichaceae	Erysipelatoclostridium	CRISPR-associated protein Cas10/Csm1, subtype III-A/MTUBE	cas10	cd09680	CAS-III			
16253..16682	+	SET52213.1	FOIN01000016.1	[Clostridium] cocleatum	Bacteria	Firmicutes	Erysipelotrichia	Erysipelotrichales	Erysipelotrichaceae	Erysipelatoclostridium	CRISPR type III-A/MTUBE-associated protein Csm2	csm2gr11	COG1421	CAS-III-A			
16685..17321	+	SET52233.1	FOIN01000016.1	[Clostridium] cocleatum	Bacteria	Firmicutes	Erysipelotrichia	Erysipelotrichales	Erysipelotrichaceae	Erysipelatoclostridium	CRISPR-associated protein, Csm3 family	csm3gr7	cd09684	CAS-III-A,CAS-III-D			
17322..18258	+	SET52251.1	FOIN01000016.1	[Clostridium] cocleatum	Bacteria	Firmicutes	Erysipelotrichia	Erysipelotrichales	Erysipelotrichaceae	Erysipelatoclostridium	CRISPR type III-A/MTUBE-associated RAMP protein Csm4	csm4gr5	mkCas0089	CAS-III-A,CAS-III-D			
18235..19429	+	SET52271.1	FOIN01000016.1	[Clostridium] cocleatum	Bacteria	Firmicutes	Erysipelotrichia	Erysipelotrichales	Erysipelotrichaceae	Erysipelatoclostridium	CRISPR type III-A/MTUBE-associated RAMP protein Csm5	csm5gr7	cd09662	CAS-III-A			
19412..20147	+	SET52291.1	FOIN01000016.1	[Clostridium] cocleatum	Bacteria	Firmicutes	Erysipelotrichia	Erysipelotrichales	Erysipelotrichaceae	Erysipelatoclostridium	hypothetical protein	cas6*	mkCas0066				
20118..20856	+	SET52310.1	FOIN01000016.1	[Clostridium] cocleatum	Bacteria	Firmicutes	Erysipelotrichia	Erysipelotrichales	Erysipelotrichaceae	Erysipelatoclostridium	CRISPR-associated protein, Cas6 family	cas6	COG1583	CAS-I,CAS-III,CAS-IV			
20830..21820	+	SET52330.1	FOIN01000016.1	[Clostridium] cocleatum	Bacteria	Firmicutes	Erysipelotrichia	Erysipelotrichales	Erysipelotrichaceae	Erysipelatoclostridium	CRISP-associated protein Cas1	cas1	cd09722	CAS-I-B			
21822..22083	+	SET52351.1	FOIN01000016.1	[Clostridium] cocleatum	Bacteria	Firmicutes	Erysipelotrichia	Erysipelotrichales	Erysipelotrichaceae	Erysipelatoclostridium	CRISPR-associated protein Cas2	**cas2**	cd09725	CAS-I,CAS-II,CAS-III,CAS-V			
22236..23432	array		FOIN01000016.1	[Clostridium] cocleatum	Bacteria	Firmicutes	Erysipelotrichia	Erysipelotrichales	Erysipelotrichaceae	Erysipelatoclostridium		CRISPR array			18	37	31
217 CAS-III-A	OUQ05535.1																
122535..123888	+	OUQ05526.1	NFLB01000005.1	[Clostridium] spiroforme	Bacteria	Firmicutes	Erysipelotrichia	Erysipelotrichales	Erysipelotrichaceae	Erysipelatoclostridium	hypothetical protein	csm6	pfam09659	CAS-III-A			
123896..126248	+	OUQ05527.1	NFLB01000005.1	[Clostridium] spiroforme	Bacteria	Firmicutes	Erysipelotrichia	Erysipelotrichales	Erysipelotrichaceae	Erysipelatoclostridium	type III-A CRISPR-associated protein Cas10/Csm1	cas10	cd09680	CAS-III			
126260..126662	+	OUQ05528.1	NFLB01000005.1	[Clostridium] spiroforme	Bacteria	Firmicutes	Erysipelotrichia	Erysipelotrichales	Erysipelotrichaceae	Erysipelatoclostridium	type III-A CRISPR-associated protein Csm2	csm2gr11	pfam03750	CAS-III-A			
126663..127329	+	OUQ05529.1	NFLB01000005.1	[Clostridium] spiroforme	Bacteria	Firmicutes	Erysipelotrichia	Erysipelotrichales	Erysipelotrichaceae	Erysipelatoclostridium	type III-A CRISPR-associated RAMP protein Csm3	csm3gr7	cd09684	CAS-III-A,CAS-III-D			
127315..128251	+	OUQ05530.1	NFLB01000005.1	[Clostridium] spiroforme	Bacteria	Firmicutes	Erysipelotrichia	Erysipelotrichales	Erysipelotrichaceae	Erysipelatoclostridium	hypothetical protein	csm4gr5	mkCas0089	CAS-III-A,CAS-III-D			
128262..129432	+	OUQ05531.1	NFLB01000005.1	[Clostridium] spiroforme	Bacteria	Firmicutes	Erysipelotrichia	Erysipelotrichales	Erysipelotrichaceae	Erysipelatoclostridium	type III-A CRISPR-associated RAMP protein Csm5	csm5gr7	cd09662	CAS-III-A			
129409..130144	+	OUQ05532.1	NFLB01000005.1	[Clostridium] spiroforme	Bacteria	Firmicutes	Erysipelotrichia	Erysipelotrichales	Erysipelotrichaceae	Erysipelatoclostridium	hypothetical protein	cas6	mkCas0066	CAS-I,CAS-III			
130115..130853	+	OUQ05533.1	NFLB01000005.1	[Clostridium] spiroforme	Bacteria	Firmicutes	Erysipelotrichia	Erysipelotrichales	Erysipelotrichaceae	Erysipelatoclostridium	CRISPR-associated endoribonuclease Cas6	cas6	COG1583	CAS-I,CAS-III,CAS-IV			
130827..131817	+	OUQ05534.1	NFLB01000005.1	[Clostridium] spiroforme	Bacteria	Firmicutes	Erysipelotrichia	Erysipelotrichales	Erysipelotrichaceae	Erysipelatoclostridium	CRISPR-associated endonuclease Cas1	cas1	cd09722	CAS-I-B			
131818..132079	+	OUQ05535.1	NFLB01000005.1	[Clostridium] spiroforme	Bacteria	Firmicutes	Erysipelotrichia	Erysipelotrichales	Erysipelotrichaceae	Erysipelatoclostridium	CRISPR-associated endonuclease Cas2	**cas2**	cd09725	CAS-I,CAS-II,CAS-III,CAS-V			
132234..133370	array		NFLB01000005.1	[Clostridium] spiroforme	Bacteria	Firmicutes	Erysipelotrichia	Erysipelotrichales	Erysipelotrichaceae	Erysipelatoclostridium		CRISPR array			17	37	32
218 partial	OUQ07963.1																
60170..61831	array		NFLA01000012.1	Erysipelatoclostridium sp. An15	Bacteria	Firmicutes	Erysipelotrichia	Erysipelotrichales	Erysipelotrichaceae	Erysipelatoclostridium		CRISPR array			25	36	31
61985..62246	-	OUQ07963.1	NFLA01000012.1	Erysipelatoclostridium sp. An15	Bacteria	Firmicutes	Erysipelotrichia	Erysipelotrichales	Erysipelotrichaceae	Erysipelatoclostridium	CRISPR-associated endonuclease Cas2	**cas2**	cd09725	CAS-I,CAS-II,CAS-III,CAS-V			
62247..63237	-	OUQ07964.1	NFLA01000012.1	Erysipelatoclostridium sp. An15	Bacteria	Firmicutes	Erysipelotrichia	Erysipelotrichales	Erysipelotrichaceae	Erysipelatoclostridium	CRISPR-associated endonuclease Cas1	cas1	cd09722	CAS-I-B			
63211..63931	-	OUQ07965.1	NFLA01000012.1	Erysipelatoclostridium sp. An15	Bacteria	Firmicutes	Erysipelotrichia	Erysipelotrichales	Erysipelotrichaceae	Erysipelatoclostridium	CRISPR-associated endoribonuclease Cas6	cas6	COG1583	CAS-I,CAS-III,CAS-IV			
219 CAS-III-D	SCX12007.1																
29061..29783	array		FMUA01000006.1	Ruminococcaceae bacterium P7	Bacteria	Firmicutes	Clostridia	Clostridiales	Ruminococcaceae			CRISPR array			12	38	25
29961..30225	-	SCX12007.1	FMUA01000006.1	Ruminococcaceae bacterium P7	Bacteria	Firmicutes	Clostridia	Clostridiales	Ruminococcaceae		CRISPR-associated protein Cas2	**cas2**	cd09725	CAS-I,CAS-II,CAS-III,CAS-V			
30224..32138	-	SCX12018.1	FMUA01000006.1	Ruminococcaceae bacterium P7	Bacteria	Firmicutes	Clostridia	Clostridiales	Ruminococcaceae		CRISPR-associated endonuclease Cas1	RT,cas1	pfam00078,cd09722	CAS-I,CAS-III			
32368..32851	array		FMUA01000006.1	Ruminococcaceae bacterium P7	Bacteria	Firmicutes	Clostridia	Clostridiales	Ruminococcaceae			CRISPR array			8	37	28
32999..34973	-	SCX12029.1	FMUA01000006.1	Ruminococcaceae bacterium P7	Bacteria	Firmicutes	Clostridia	Clostridiales	Ruminococcaceae		CRISPR-associated protein	csm3gr7,csm3gr7	cls000253,cls000253	CAS-III-A,CAS-III-D			
34972..35419	-	SCX12041.1	FMUA01000006.1	Ruminococcaceae bacterium P7	Bacteria	Firmicutes	Clostridia	Clostridiales	Ruminococcaceae		hypothetical protein	Cas11/Csx19	cluster5				
35411..36632	-	SCX12054.1	FMUA01000006.1	Ruminococcaceae bacterium P7	Bacteria	Firmicutes	Clostridia	Clostridiales	Ruminococcaceae		CRISPR/Cas system CSM-associated protein Csm3, group 7 of RAMP superfamily	csm3gr7,csm3gr7	cd09726,cd09726	CAS-III-D			
36633..38643	-	SCX12065.1	FMUA01000006.1	Ruminococcaceae bacterium P7	Bacteria	Firmicutes	Clostridia	Clostridiales	Ruminococcaceae		CRISPR/Cas system CSM-associated protein Csm3, group 7 of RAMP superfamily	csm3gr7,csm3gr7,csx10gr5	mkCas0198,cd09726,cls000715	CAS-III-A,CAS-III-D			
38614..40042	-	SCX12077.1	FMUA01000006.1	Ruminococcaceae bacterium P7	Bacteria	Firmicutes	Clostridia	Clostridiales	Ruminococcaceae		hypothetical protein	cas10	mkCas0133	CAS-III			
40113..40821	+	SCX12088.1	FMUA01000006.1	Ruminococcaceae bacterium P7	Bacteria	Firmicutes	Clostridia	Clostridiales	Ruminococcaceae		CRISPR-associated endoribonuclease Cas6	cas6	COG1583	CAS-I,CAS-III,CAS-IV			
40908..41682	+	SCX12099.1	FMUA01000006.1	Ruminococcaceae bacterium P7	Bacteria	Firmicutes	Clostridia	Clostridiales	Ruminococcaceae		hypothetical protein	PD-DExK	mkCas0090	CAS-I,CAS-III			
220 CAS-III-D	SCX12118.1																
32999..34973	-	SCX12029.1	FMUA01000006.1	Ruminococcaceae bacterium P7	Bacteria	Firmicutes	Clostridia	Clostridiales	Ruminococcaceae		CRISPR-associated protein	csm3gr7,csm3gr7	cls000253,cls000253	CAS-III-A,CAS-III-D			
34972..35419	-	SCX12041.1	FMUA01000006.1	Ruminococcaceae bacterium P7	Bacteria	Firmicutes	Clostridia	Clostridiales	Ruminococcaceae		hypothetical protein	Cas11/Csx19	cluster5				
35411..36632	-	SCX12054.1	FMUA01000006.1	Ruminococcaceae bacterium P7	Bacteria	Firmicutes	Clostridia	Clostridiales	Ruminococcaceae		CRISPR/Cas system CSM-associated protein Csm3, group 7 of RAMP superfamily	csm3gr7,csm3gr7	cd09726,cd09726	CAS-III-D			
36633..38643	-	SCX12065.1	FMUA01000006.1	Ruminococcaceae bacterium P7	Bacteria	Firmicutes	Clostridia	Clostridiales	Ruminococcaceae		CRISPR/Cas system CSM-associated protein Csm3, group 7 of RAMP superfamily	csm3gr7,csm3gr7,csx10gr5	mkCas0198,cd09726,cls000715	CAS-III-A,CAS-III-D			
38614..40042	-	SCX12077.1	FMUA01000006.1	Ruminococcaceae bacterium P7	Bacteria	Firmicutes	Clostridia	Clostridiales	Ruminococcaceae		hypothetical protein	cas10	mkCas0133	CAS-III			
40113..40821	+	SCX12088.1	FMUA01000006.1	Ruminococcaceae bacterium P7	Bacteria	Firmicutes	Clostridia	Clostridiales	Ruminococcaceae		CRISPR-associated endoribonuclease Cas6	cas6	COG1583	CAS-I,CAS-III,CAS-IV			
40908..41682	+	SCX12099.1	FMUA01000006.1	Ruminococcaceae bacterium P7	Bacteria	Firmicutes	Clostridia	Clostridiales	Ruminococcaceae		hypothetical protein	PD-DExK	mkCas0090	CAS-I,CAS-III			
41727..42744	+	SCX12109.1	FMUA01000006.1	Ruminococcaceae bacterium P7	Bacteria	Firmicutes	Clostridia	Clostridiales	Ruminococcaceae		SIR2-like domain-containing protein						
42905..43320	array		FMUA01000006.1	Ruminococcaceae bacterium P7	Bacteria	Firmicutes	Clostridia	Clostridiales	Ruminococcaceae			CRISPR array			7	36	28
43499..43763	-	SCX12118.1	FMUA01000006.1	Ruminococcaceae bacterium P7	Bacteria	Firmicutes	Clostridia	Clostridiales	Ruminococcaceae		CRISPR-associated protein Cas2	**cas2**	cd09725	CAS-I,CAS-II,CAS-III,CAS-V			
43762..45676	-	SCX12129.1	FMUA01000006.1	Ruminococcaceae bacterium P7	Bacteria	Firmicutes	Clostridia	Clostridiales	Ruminococcaceae		CRISPR-associated endonuclease Cas1/group II intron reverse transcriptase/maturase,TIGR04416	RT,cas1	pfam00078,cd09722	CAS-I,CAS-III			
45905..46259	array		FMUA01000006.1	Ruminococcaceae bacterium P7	Bacteria	Firmicutes	Clostridia	Clostridiales	Ruminococcaceae			CRISPR array			6	37	28
221 CAS-III-A	SCG94033.1																
140058..140817	+	SCG93766.1	FMDZ01000001.1	uncultured Eubacterium sp.	Bacteria	Firmicutes	Clostridia	Clostridiales	Eubacteriaceae	Eubacterium	CRISPR-associated endoribonuclease Cas6	cas6	COG5551	CAS-III			
140820..143163	+	SCG93805.1	FMDZ01000001.1	uncultured Eubacterium sp.	Bacteria	Firmicutes	Clostridia	Clostridiales	Eubacteriaceae	Eubacterium	CRISPR-associated protein Cas10/Csm1%2C subtype III-A/MTUBE	cas10	cd09680	CAS-III			
143140..143527	+	SCG93843.1	FMDZ01000001.1	uncultured Eubacterium sp.	Bacteria	Firmicutes	Clostridia	Clostridiales	Eubacteriaceae	Eubacterium	CRISPR type III-A/MTUBE-associated protein Csm2	csm2gr11	cd09647	CAS-III-A			
143540..144209	+	SCG93882.1	FMDZ01000001.1	uncultured Eubacterium sp.	Bacteria	Firmicutes	Clostridia	Clostridiales	Eubacteriaceae	Eubacterium	CRISPR type III-A/MTUBE-associated RAMP protein Csm3	csm3gr7	cd09684	CAS-III-A,CAS-III-D			
144211..145144	+	SCG93924.1	FMDZ01000001.1	uncultured Eubacterium sp.	Bacteria	Firmicutes	Clostridia	Clostridiales	Eubacteriaceae	Eubacterium	CRISPR type III-A/MTUBE-associated RAMP protein Csm4	csm4gr5	COG1567	CAS-III-A			
145145..146273	+	SCG93960.1	FMDZ01000001.1	uncultured Eubacterium sp.	Bacteria	Firmicutes	Clostridia	Clostridiales	Eubacteriaceae	Eubacterium	CRISPR type III-A/MTUBE-associated RAMP protein Csm5	csm5gr7,csm5gr7	cd09662,cd09662	CAS-III-A			
146407..147021	array		FMDZ01000001.1	uncultured Eubacterium sp.	Bacteria	Firmicutes	Clostridia	Clostridiales	Eubacteriaceae	Eubacterium		CRISPR array			9	36	36
147123..148125	+	SCG93998.1	FMDZ01000001.1	uncultured Eubacterium sp.	Bacteria	Firmicutes	Clostridia	Clostridiales	Eubacteriaceae	Eubacterium	CRISPR-associated endonuclease Cas1	cas1	cd09634	CAS-I,CAS-II,CAS-III,CAS-V			
148132..148450	+	SCG94033.1	FMDZ01000001.1	uncultured Eubacterium sp.	Bacteria	Firmicutes	Clostridia	Clostridiales	Eubacteriaceae	Eubacterium	CRISPR-associated endoribonuclease Cas2	**cas2**	cd09725	CAS-I,CAS-II,CAS-III,CAS-V			
148469..149798	+	SCG94074.1	FMDZ01000001.1	uncultured Eubacterium sp.	Bacteria	Firmicutes	Clostridia	Clostridiales	Eubacteriaceae	Eubacterium	CRISPR type III-A/MTUBE-associated protein Csm6	csm6	pfam09659	CAS-III-A			
222 CAS-III-A	AMC51852.1																
3050860..3052195	array		CP010331.1	Mycobacterium tuberculosis variant bovis BCG	Bacteria	Actinobacteria	Actinobacteria	Corynebacteriales	Mycobacteriaceae	Mycobacterium		CRISPR array			19	45	27
3052292..3053177	-	AMC51848.1	CP010331.1	Mycobacterium tuberculosis variant bovis BCG	Bacteria	Actinobacteria	Actinobacteria	Corynebacteriales	Mycobacteriaceae	Mycobacterium	hypothetical protein						
3053227..3053554	-	AMC51849.1	CP010331.1	Mycobacterium tuberculosis variant bovis BCG	Bacteria	Actinobacteria	Actinobacteria	Corynebacteriales	Mycobacteriaceae	Mycobacterium	transposase						
3053592..3055729	array		CP010331.1	Mycobacterium tuberculosis variant bovis BCG	Bacteria	Actinobacteria	Actinobacteria	Corynebacteriales	Mycobacteriaceae	Mycobacterium		CRISPR array			30	47	25
3053946..3054105	-	AMC51850.1	CP010331.1	Mycobacterium tuberculosis variant bovis BCG	Bacteria	Actinobacteria	Actinobacteria	Corynebacteriales	Mycobacteriaceae	Mycobacterium	hypothetical protein						
3054899..3055175	-	AMC51851.1	CP010331.1	Mycobacterium tuberculosis variant bovis BCG	Bacteria	Actinobacteria	Actinobacteria	Corynebacteriales	Mycobacteriaceae	Mycobacterium	hypothetical protein						
3055787..3056084	-	AMC51852.1	CP010331.1	Mycobacterium tuberculosis variant bovis BCG	Bacteria	Actinobacteria	Actinobacteria	Corynebacteriales	Mycobacteriaceae	Mycobacterium	CRISPR-associated endoribonuclease Cas2	**cas2**	COG1343	CAS-I,CAS-II,CAS-III			
3056059..3057145	-	AMC51853.1	CP010331.1	Mycobacterium tuberculosis variant bovis BCG	Bacteria	Actinobacteria	Actinobacteria	Corynebacteriales	Mycobacteriaceae	Mycobacterium	CRISPR-associated endonuclease Cas1	cas1	COG1518	CAS-I,CAS-II,CAS-III			
3057394..3058405	-	AMC51854.1	CP010331.1	Mycobacterium tuberculosis variant bovis BCG	Bacteria	Actinobacteria	Actinobacteria	Corynebacteriales	Mycobacteriaceae	Mycobacterium	CRISPR-associated protein Csm6	csm6	cd09699	CAS-III-A			
3058401..3059529	-	AMC51855.1	CP010331.1	Mycobacterium tuberculosis variant bovis BCG	Bacteria	Actinobacteria	Actinobacteria	Corynebacteriales	Mycobacteriaceae	Mycobacterium	CRISPR type III-associated RAMP protein Csm5	csm5gr7	COG1332	CAS-III-A			
3059525..3060434	-	AMC51856.1	CP010331.1	Mycobacterium tuberculosis variant bovis BCG	Bacteria	Actinobacteria	Actinobacteria	Corynebacteriales	Mycobacteriaceae	Mycobacterium	CRISPR type III-associated RAMP protein Csm4	csm4gr5	COG1567	CAS-III-A			
3060414..3060759	-	AMC51857.1	CP010331.1	Mycobacterium tuberculosis variant bovis BCG	Bacteria	Actinobacteria	Actinobacteria	Corynebacteriales	Mycobacteriaceae	Mycobacterium	CRISPR type III-associated RAMP protein Csm3	csm3gr7	cd09684	CAS-III-A,CAS-III-D			
3060755..3061124	-	AMC51858.1	CP010331.1	Mycobacterium tuberculosis variant bovis BCG	Bacteria	Actinobacteria	Actinobacteria	Corynebacteriales	Mycobacteriaceae	Mycobacterium	CRISPR type III-associated RAMP protein Csm3	csm3gr7	cd09684	CAS-III-A,CAS-III-D			
3061133..3061508	-	AMC51859.1	CP010331.1	Mycobacterium tuberculosis variant bovis BCG	Bacteria	Actinobacteria	Actinobacteria	Corynebacteriales	Mycobacteriaceae	Mycobacterium	CRISPR type III-associated protein Csm2	csm2gr11	cd09647	CAS-III-A			
3061504..3063958	-	AMC51860.1	CP010331.1	Mycobacterium tuberculosis variant bovis BCG	Bacteria	Actinobacteria	Actinobacteria	Corynebacteriales	Mycobacteriaceae	Mycobacterium	CRISPR-associated protein Cas10/Csm1	cas10	COG1353	CAS-III			
3063939..3064728	-	AMC51861.1	CP010331.1	Mycobacterium tuberculosis variant bovis BCG	Bacteria	Actinobacteria	Actinobacteria	Corynebacteriales	Mycobacteriaceae	Mycobacterium	CRISPR-associated endoribonuclease Cas6	cas6	COG5551	CAS-III			
3065061..3065709	-	AMC51862.1	CP010331.1	Mycobacterium tuberculosis variant bovis BCG	Bacteria	Actinobacteria	Actinobacteria	Corynebacteriales	Mycobacteriaceae	Mycobacterium	hypothetical protein	csm3gr7*	cls000184				
223 partial	CKV02534.1																
126..393	+	CKV02534.1	CNGG01001637.1	Mycobacterium tuberculosis	Bacteria	Actinobacteria	Actinobacteria	Corynebacteriales	Mycobacteriaceae	Mycobacterium	Putative CRISPR-associated RNAse Cas2	**cas2**	COG1343	CAS-I,CAS-II,CAS-III			
442..916	array		CNGG01001637.1	Mycobacterium tuberculosis	Bacteria	Actinobacteria	Actinobacteria	Corynebacteriales	Mycobacteriaceae	Mycobacterium		CRISPR array			7	37	36
224 CAS-III-A	CCC45171.1																
3181323..3183563	array		HE572590.1	Mycobacterium canettii CIPT 140010059	Bacteria	Actinobacteria	Actinobacteria	Corynebacteriales	Mycobacteriaceae	Mycobacterium		CRISPR array			31	37	36
3183611..3183953	-	CCC45171.1	HE572590.1	Mycobacterium canettii CIPT 140010059	Bacteria	Actinobacteria	Actinobacteria	Corynebacteriales	Mycobacteriaceae	Mycobacterium	conserved hypothetical protein	**cas2**	COG1343	CAS-I,CAS-II,CAS-III			
3183953..3184970	-	CCC45172.1	HE572590.1	Mycobacterium canettii CIPT 140010059	Bacteria	Actinobacteria	Actinobacteria	Corynebacteriales	Mycobacteriaceae	Mycobacterium	conserved hypothetical protein	cas1	COG1518	CAS-I,CAS-II,CAS-III			

3184982..3186131	-	CCC45173.1	HE572590.1	Mycobacterium canettii CIPT 140010059	Bacteria	Actinobacteria	Actinobacteria	Corynebacteriales	Mycobacteriaceae	Mycobacterium	hypothetical protein	csm6	cd09699	CAS-III-A			
3186226..3187354	-	CCC45174.1	HE572590.1	Mycobacterium canettii CIPT 140010059	Bacteria	Actinobacteria	Actinobacteria	Corynebacteriales	Mycobacteriaceae	Mycobacterium	hypothetical protein	csm5gr7	COG1332	CAS-III-A			
3187350..3188259	-	CCC45175.1	HE572590.1	Mycobacterium canettii CIPT 140010059	Bacteria	Actinobacteria	Actinobacteria	Corynebacteriales	Mycobacteriaceae	Mycobacterium	hypothetical protein	csm4gr5	COG1567	CAS-III-A			
3188239..3188950	-	CCC45176.1	HE572590.1	Mycobacterium canettii CIPT 140010059	Bacteria	Actinobacteria	Actinobacteria	Corynebacteriales	Mycobacteriaceae	Mycobacterium	conserved hypothetical protein	csm3gr7	cd09684	CAS-III-A,CAS-III-D			
3188946..3189339	-	CCC45177.1	HE572590.1	Mycobacterium canettii CIPT 140010059	Bacteria	Actinobacteria	Actinobacteria	Corynebacteriales	Mycobacteriaceae	Mycobacterium	putative uncharacterized protein	csm2gr11	cd09647	CAS-III-A			
3189335..3191777	-	CCC45178.1	HE572590.1	Mycobacterium canettii CIPT 140010059	Bacteria	Actinobacteria	Actinobacteria	Corynebacteriales	Mycobacteriaceae	Mycobacterium	conserved hypothetical protein	cas10	cd09680	CAS-III			

225 CAS-III-A | AMC65164.1

3097177..3099316	array		CP010334.1	Mycobacterium tuberculosis variant africanum	Bacteria	Actinobacteria	Actinobacteria	Corynebacteriales	Mycobacteriaceae	Mycobacterium		CRISPR array			30	45	27
3097942..3098182	-	AMC65162.1	CP010334.1	Mycobacterium tuberculosis variant africanum	Bacteria	Actinobacteria	Actinobacteria	Corynebacteriales	Mycobacteriaceae	Mycobacterium	hypothetical protein						
3098462..3099272	-	AMC65163.1	CP010334.1	Mycobacterium tuberculosis variant africanum	Bacteria	Actinobacteria	Actinobacteria	Corynebacteriales	Mycobacteriaceae	Mycobacterium	hypothetical protein						
3099364..3099706	-	AMC65164.1	CP010334.1	Mycobacterium tuberculosis variant africanum	Bacteria	Actinobacteria	Actinobacteria	Corynebacteriales	Mycobacteriaceae	Mycobacterium	CRISPR-associated endoribonuclease Cas2	**cas2**	COG1343	CAS-I,CAS-II,CAS-III			
3099706..3100723	-	AMC65165.1	CP010334.1	Mycobacterium tuberculosis variant africanum	Bacteria	Actinobacteria	Actinobacteria	Corynebacteriales	Mycobacteriaceae	Mycobacterium	CRISPR-associated endonuclease Cas1	cas1	COG1518	CAS-I,CAS-II,CAS-III			
3100972..3101983	-	AMC65166.1	CP010334.1	Mycobacterium tuberculosis variant africanum	Bacteria	Actinobacteria	Actinobacteria	Corynebacteriales	Mycobacteriaceae	Mycobacterium	CRISPR-associated protein Csm6	csm6	cd09699	CAS-III-A			
3101979..3103056	-	AMC65167.1	CP010334.1	Mycobacterium tuberculosis variant africanum	Bacteria	Actinobacteria	Actinobacteria	Corynebacteriales	Mycobacteriaceae	Mycobacterium	CRISPR type III-associated RAMP protein Csm5	csm5gr7	COG1332	CAS-III-A			
3103102..3104011	-	AMC65168.1	CP010334.1	Mycobacterium tuberculosis variant africanum	Bacteria	Actinobacteria	Actinobacteria	Corynebacteriales	Mycobacteriaceae	Mycobacterium	CRISPR type III-associated RAMP protein Csm4	csm4gr5	COG1567	CAS-III-A			
3103991..3104702	-	AMC65169.1	CP010334.1	Mycobacterium tuberculosis variant africanum	Bacteria	Actinobacteria	Actinobacteria	Corynebacteriales	Mycobacteriaceae	Mycobacterium	CRISPR type III-associated RAMP protein Csm3	csm3gr7	cd09684	CAS-III-A,CAS-III-D			
3104711..3105086	-	AMC65170.1	CP010334.1	Mycobacterium tuberculosis variant africanum	Bacteria	Actinobacteria	Actinobacteria	Corynebacteriales	Mycobacteriaceae	Mycobacterium	CRISPR type III-associated protein Csm2	csm2gr11	cd09647	CAS-III-A			
3105082..3107260	-	AMC65171.1	CP010334.1	Mycobacterium tuberculosis variant africanum	Bacteria	Actinobacteria	Actinobacteria	Corynebacteriales	Mycobacteriaceae	Mycobacterium	CRISPR-associated protein Cas10/Csm1	cas10	COG1353	CAS-III			
3107518..3108307	-	AMC65172.1	CP010334.1	Mycobacterium tuberculosis variant africanum	Bacteria	Actinobacteria	Actinobacteria	Corynebacteriales	Mycobacteriaceae	Mycobacterium	CRISPR-associated endoribonuclease Cas6	cas6	COG5551	CAS-III			

226 CAS-III-A | CUO16880.1

154793..156119	+	CUO16576.1	CYZM01000004.1	Anaerostipes hadrus	Bacteria	Firmicutes	Clostridia	Clostridiales	Lachnospiraceae	Anaerostipes	CRISPR type III-A/MTUBE-associated protein Csm6	csm6	cd09746	CAS-III-A			
156115..156856	+	CUO16613.1	CYZM01000004.1	Anaerostipes hadrus	Bacteria	Firmicutes	Clostridia	Clostridiales	Lachnospiraceae	Anaerostipes	CRISPR-associated endoribonuclease Cas6	cas6	COG5551	CAS-III			
156860..159194	+	CUO16648.1	CYZM01000004.1	Anaerostipes hadrus	Bacteria	Firmicutes	Clostridia	Clostridiales	Lachnospiraceae	Anaerostipes	Predicted HD superfamily hydrolase	cas10	cd09680	CAS-III			
159194..159572	+	CUO16682.1	CYZM01000004.1	Anaerostipes hadrus	Bacteria	Firmicutes	Clostridia	Clostridiales	Lachnospiraceae	Anaerostipes	CRISPR type III-A/MTUBE-associated protein Csm2	csm2gr11	cd09647	CAS-III-A			
159589..160267	+	CUO16726.1	CYZM01000004.1	Anaerostipes hadrus	Bacteria	Firmicutes	Clostridia	Clostridiales	Lachnospiraceae	Anaerostipes	CRISPR type III-A/MTUBE-associated RAMP protein Csm3	csm3gr7	cd09684	CAS-III-A,CAS-III-D			
160266..161196	+	CUO16763.1	CYZM01000004.1	Anaerostipes hadrus	Bacteria	Firmicutes	Clostridia	Clostridiales	Lachnospiraceae	Anaerostipes	CRISPR type III-A/MTUBE-associated RAMP protein Csm4	csm4gr5	cd09663	CAS-III-A			
161195..162308	+	CUO16802.1	CYZM01000004.1	Anaerostipes hadrus	Bacteria	Firmicutes	Clostridia	Clostridiales	Lachnospiraceae	Anaerostipes	CRISPR type III-A/MTUBE-associated RAMP protein Csm5	csm5gr7,csm5gr7	cd09662,cd09662	CAS-III-A			
162444..163132	array		CYZM01000004.1	Anaerostipes hadrus	Bacteria	Firmicutes	Clostridia	Clostridiales	Lachnospiraceae	Anaerostipes		CRISPR array			10	37	35
163375..164230	+	CUO16841.1	CYZM01000004.1	Anaerostipes hadrus	Bacteria	Firmicutes	Clostridia	Clostridiales	Lachnospiraceae	Anaerostipes	CRISPR-associated endonuclease Cas1	cas1	cd09634	CAS-I,CAS-II,CAS-III,CAS-V			
164381..164699	+	CUO16880.1	CYZM01000004.1	Anaerostipes hadrus	Bacteria	Firmicutes	Clostridia	Clostridiales	Lachnospiraceae	Anaerostipes	CRISPR-associated endoribonuclease Cas2	**cas2**	cd09725	CAS-I,CAS-II,CAS-III,CAS-V			

227 CAS-III-A | EHI56779.1

250396..251758	-	EHI56778.1	JH378829.1	Johnsonella ignava ATCC 51276	Bacteria	Firmicutes	Clostridia	Clostridiales	Lachnospiraceae	Johnsonella	hypothetical protein	csm6	cd09746	CAS-III-A			
251785..252139	-	EHI56779.1	JH378829.1	Johnsonella ignava ATCC 51276	Bacteria	Firmicutes	Clostridia	Clostridiales	Lachnospiraceae	Johnsonella	hypothetical protein	**cas2**	cd09725	CAS-I,CAS-II,CAS-III,CAS-V			
252141..253152	-	EHI56780.1	JH378829.1	Johnsonella ignava ATCC 51276	Bacteria	Firmicutes	Clostridia	Clostridiales	Lachnospiraceae	Johnsonella	CRISPR-associated protein cas1	cas1	cd09634	CAS-I,CAS-II,CAS-III,CAS-V			
253797..254418	-	EHI56781.1	JH378829.1	Johnsonella ignava ATCC 51276	Bacteria	Firmicutes	Clostridia	Clostridiales	Lachnospiraceae	Johnsonella	hypothetical protein						
254799..255276	array		JH378829.1	Johnsonella ignava ATCC 51276	Bacteria	Firmicutes	Clostridia	Clostridiales	Lachnospiraceae	Johnsonella		CRISPR array			7	38	35
255358..255643	+	EHI56782.1	JH378829.1	Johnsonella ignava ATCC 51276	Bacteria	Firmicutes	Clostridia	Clostridiales	Lachnospiraceae	Johnsonella	hypothetical protein						
255987..256418	+	EHI56553.1	JH378829.1	Johnsonella ignava ATCC 51276	Bacteria	Firmicutes	Clostridia	Clostridiales	Lachnospiraceae	Johnsonella	hypothetical protein						
256499..257329	array		JH378829.1	Johnsonella ignava ATCC 51276	Bacteria	Firmicutes	Clostridia	Clostridiales	Lachnospiraceae	Johnsonella		CRISPR array			12	38	34
257498..258656	-	EHI56554.1	JH378829.1	Johnsonella ignava ATCC 51276	Bacteria	Firmicutes	Clostridia	Clostridiales	Lachnospiraceae	Johnsonella	csm5 family CRISPR-associated ramp protein	csm5gr7	cd09662	CAS-III-A			
258685..259753	-	EHI56555.1	JH378829.1	Johnsonella ignava ATCC 51276	Bacteria	Firmicutes	Clostridia	Clostridiales	Lachnospiraceae	Johnsonella	csm4 family CRISPR-associated ramp protein	csm4gr5,csm4gr5	cd09663,cd09663	CAS-III-A			
259742..260459	-	EHI56556.1	JH378829.1	Johnsonella ignava ATCC 51276	Bacteria	Firmicutes	Clostridia	Clostridiales	Lachnospiraceae	Johnsonella	csm3 family CRISPR-associated ramp protein	csm3gr7	cd09684	CAS-III-A,CAS-III-D			
260471..260900	-	EHI56557.1	JH378829.1	Johnsonella ignava ATCC 51276	Bacteria	Firmicutes	Clostridia	Clostridiales	Lachnospiraceae	Johnsonella	hypothetical protein	csm2gr11	cd09647	CAS-III-A			
260902..261955	-	EHI56558.1	JH378829.1	Johnsonella ignava ATCC 51276	Bacteria	Firmicutes	Clostridia	Clostridiales	Lachnospiraceae	Johnsonella	hypothetical protein	cas10	cd09680	CAS-III			
261969..263391	-	EHI56559.1	JH378829.1	Johnsonella ignava ATCC 51276	Bacteria	Firmicutes	Clostridia	Clostridiales	Lachnospiraceae	Johnsonella	hypothetical protein	cas10	cd09680	CAS-III			
263410..264163	-	EHI56560.1	JH378829.1	Johnsonella ignava ATCC 51276	Bacteria	Firmicutes	Clostridia	Clostridiales	Lachnospiraceae	Johnsonella	CRISPR-associated protein cas6	cas6	cd09652	CAS-I,CAS-III			
264317..265631	-	EHI56561.1	JH378829.1	Johnsonella ignava ATCC 51276	Bacteria	Firmicutes	Clostridia	Clostridiales	Lachnospiraceae	Johnsonella	hypothetical protein	C3a214	cluster214				

228 CAS-III-A | ESL02974.1

352442..352820	+	ESL02958.1	KI535368.1	Catonella morbi ATCC 51271	Bacteria	Firmicutes	Clostridia	Clostridiales	Lachnospiraceae	Catonella	hypothetical protein	WYL	pfam13280	CAS-I,CAS-III			
352843..353626	+	ESL02959.1	KI535368.1	Catonella morbi ATCC 51271	Bacteria	Firmicutes	Clostridia	Clostridiales	Lachnospiraceae	Catonella	CRISPR-associated endoribonuclease Cas6	cas6	COG5551	CAS-III			
353606..355874	+	ESL02960.1	KI535368.1	Catonella morbi ATCC 51271	Bacteria	Firmicutes	Clostridia	Clostridiales	Lachnospiraceae	Catonella	CRISPR-associated protein, Csm1 family	cas10	cd09680	CAS-III			
355870..356263	+	ESL02961.1	KI535368.1	Catonella morbi ATCC 51271	Bacteria	Firmicutes	Clostridia	Clostridiales	Lachnospiraceae	Catonella	CRISPR-associated protein, Csm2 family	csm2gr11	cd09647	CAS-III-A			
356252..356942	+	ESL02962.1	KI535368.1	Catonella morbi ATCC 51271	Bacteria	Firmicutes	Clostridia	Clostridiales	Lachnospiraceae	Catonella	CRISPR-associated RAMP protein, Csm3 family	csm3gr7	cd09684	CAS-III-A,CAS-III-D			
356941..357871	+	ESL02963.1	KI535368.1	Catonella morbi ATCC 51271	Bacteria	Firmicutes	Clostridia	Clostridiales	Lachnospiraceae	Catonella	CRISPR-associated RAMP protein, Csm4 family	csm4gr5	COG1567	CAS-III-A			
357870..359016	+	ESL02964.1	KI535368.1	Catonella morbi ATCC 51271	Bacteria	Firmicutes	Clostridia	Clostridiales	Lachnospiraceae	Catonella	CRISPR-associated RAMP protein, Csm5 family	csm5gr7	cd09662	CAS-III-A			
359096..359402	-	ESL02965.1	KI535368.1	Catonella morbi ATCC 51271	Bacteria	Firmicutes	Clostridia	Clostridiales	Lachnospiraceae	Catonella	hypothetical protein						
359151..362243	array		KI535368.1	Catonella morbi ATCC 51271	Bacteria	Firmicutes	Clostridia	Clostridiales	Lachnospiraceae	Catonella		CRISPR array			44	35	36
359423..359600	+	ESL02966.1	KI535368.1	Catonella morbi ATCC 51271	Bacteria	Firmicutes	Clostridia	Clostridiales	Lachnospiraceae	Catonella	hypothetical protein						
359767..359911	-	ESL02967.1	KI535368.1	Catonella morbi ATCC 51271	Bacteria	Firmicutes	Clostridia	Clostridiales	Lachnospiraceae	Catonella	hypothetical protein						
359959..360145	+	ESL02968.1	KI535368.1	Catonella morbi ATCC 51271	Bacteria	Firmicutes	Clostridia	Clostridiales	Lachnospiraceae	Catonella	hypothetical protein						
360396..360537	-	ESL02969.1	KI535368.1	Catonella morbi ATCC 51271	Bacteria	Firmicutes	Clostridia	Clostridiales	Lachnospiraceae	Catonella	hypothetical protein						
360883..361096	+	ESL02970.1	KI535368.1	Catonella morbi ATCC 51271	Bacteria	Firmicutes	Clostridia	Clostridiales	Lachnospiraceae	Catonella	hypothetical protein						
361681..361819	-	ESL02971.1	KI535368.1	Catonella morbi ATCC 51271	Bacteria	Firmicutes	Clostridia	Clostridiales	Lachnospiraceae	Catonella	hypothetical protein						
362040..362244	-	ESL02972.1	KI535368.1	Catonella morbi ATCC 51271	Bacteria	Firmicutes	Clostridia	Clostridiales	Lachnospiraceae	Catonella	hypothetical protein						
362258..363281	+	ESL02973.1	KI535368.1	Catonella morbi ATCC 51271	Bacteria	Firmicutes	Clostridia	Clostridiales	Lachnospiraceae	Catonella	CRISPR-associated endonuclease Cas1	cas1	cd09634	CAS-I,CAS-II,CAS-III,CAS-V			
363286..363574	+	ESL02974.1	KI535368.1	Catonella morbi ATCC 51271	Bacteria	Firmicutes	Clostridia	Clostridiales	Lachnospiraceae	Catonella	CRISPR-associated protein Cas2	**cas2**	cd09725	CAS-I,CAS-II,CAS-III,CAS-V			
363797..363995	-	ESL02975.1	KI535368.1	Catonella morbi ATCC 51271	Bacteria	Firmicutes	Clostridia	Clostridiales	Lachnospiraceae	Catonella	hypothetical protein						
364586..364724	+	ESL02976.1	KI535368.1	Catonella morbi ATCC 51271	Bacteria	Firmicutes	Clostridia	Clostridiales	Lachnospiraceae	Catonella	hypothetical protein						
364985..366338	+	ESL02977.1	KI535368.1	Catonella morbi ATCC 51271	Bacteria	Firmicutes	Clostridia	Clostridiales	Lachnospiraceae	Catonella	CRISPR-associated protein, Csm6 family	csm6	cd09746	CAS-III-A			

229 CAS-III-A | PTL29108.1

909119..912023	array		PYXF01000001.1	Lachnospiraceae bacterium oral taxon 096	Bacteria	Firmicutes	Clostridia	Clostridiales	Lachnospiraceae			CRISPR array			40	37	36
912685..913774	+	PTL29105.1	PYXF01000001.1	Lachnospiraceae bacterium oral taxon 096	Bacteria	Firmicutes	Clostridia	Clostridiales	Lachnospiraceae		transposase	c2c9	c2c9	CAS-V-U4			
914114..915239	-	PTL29106.1	PYXF01000001.1	Lachnospiraceae bacterium oral taxon 096	Bacteria	Firmicutes	Clostridia	Clostridiales	Lachnospiraceae		hypothetical protein	WYL	pfam13280	CAS-I,CAS-III			
915485..916490	+	PTL29107.1	PYXF01000001.1	Lachnospiraceae bacterium oral taxon 096	Bacteria	Firmicutes	Clostridia	Clostridiales	Lachnospiraceae		CRISPR-associated endonuclease Cas1	cas1	cd09634	CAS-I,CAS-II,CAS-III,CAS-V			
916500..916776	+	PTL29108.1	PYXF01000001.1	Lachnospiraceae bacterium oral taxon 096	Bacteria	Firmicutes	Clostridia	Clostridiales	Lachnospiraceae		CRISPR-associated endonuclease Cas2	**cas2**	cd09725	CAS-I,CAS-II,CAS-III,CAS-V			
917529..917808	+	PTL29109.1	PYXF01000001.1	Lachnospiraceae bacterium oral taxon 096	Bacteria	Firmicutes	Clostridia	Clostridiales	Lachnospiraceae		hypothetical protein						
917846..918758	+	PTL29110.1	PYXF01000001.1	Lachnospiraceae bacterium oral taxon 096	Bacteria	Firmicutes	Clostridia	Clostridiales	Lachnospiraceae		hypothetical protein						
918723..919482	+	PTL29111.1	PYXF01000001.1	Lachnospiraceae bacterium oral taxon 096	Bacteria	Firmicutes	Clostridia	Clostridiales	Lachnospiraceae		CRISPR-associated endoribonuclease Cas6	cas6	pfam10040	CAS-III,CAS-IV			
919478..921830	+	PTL29112.1	PYXF01000001.1	Lachnospiraceae bacterium oral taxon 096	Bacteria	Firmicutes	Clostridia	Clostridiales	Lachnospiraceae		type III-A CRISPR-associated protein Cas10/Csm1	cas10	cd09680	CAS-III			
921813..922206	+	PTL29113.1	PYXF01000001.1	Lachnospiraceae bacterium oral taxon 096	Bacteria	Firmicutes	Clostridia	Clostridiales	Lachnospiraceae		type III-A CRISPR-associated protein Csm2	csm2gr11	cd09647	CAS-III-A			
922207..922903	+	PTL29114.1	PYXF01000001.1	Lachnospiraceae bacterium oral taxon 096	Bacteria	Firmicutes	Clostridia	Clostridiales	Lachnospiraceae		type III-A CRISPR-associated RAMP protein Csm3	csm3gr7	cd09684	CAS-III-A,CAS-III-D			
922904..923840	+	PTL29115.1	PYXF01000001.1	Lachnospiraceae bacterium oral taxon 096	Bacteria	Firmicutes	Clostridia	Clostridiales	Lachnospiraceae		type III-A CRISPR-associated RAMP protein Csm4	csm4gr5	cd09663	CAS-III-A			
923841..924990	+	PTL29116.1	PYXF01000001.1	Lachnospiraceae bacterium oral taxon 096	Bacteria	Firmicutes	Clostridia	Clostridiales	Lachnospiraceae		type III-A CRISPR-associated RAMP protein Csm5	csm5gr7	COG1332	CAS-III-A			
925009..926341	+	PTL29117.1	PYXF01000001.1	Lachnospiraceae bacterium oral taxon 096	Bacteria	Firmicutes	Clostridia	Clostridiales	Lachnospiraceae		CRISPR-associated protein Csm6	csm6	pfam09659	CAS-III-A			

230 CAS-III-A | EGG91031.1

2021..2333	-	EGG91031.1	GL890597.1	Lachnospiraceae oral taxon 107 str. F0167	Bacteria	Firmicutes	Clostridia	Clostridiales	Lachnospiraceae		CRISPR-associated protein cas2	**cas2**	cd09725	CAS-I,CAS-II,CAS-III,CAS-V			
2340..3345	-	EGG91032.1	GL890597.1	Lachnospiraceae oral taxon 107 str. F0167	Bacteria	Firmicutes	Clostridia	Clostridiales	Lachnospiraceae		CRISPR-associated protein cas1	cas1	cd09634	CAS-I,CAS-II,CAS-III,CAS-V			
3354..4500	-	EGG91033.1	GL890597.1	Lachnospiraceae oral taxon 107 str. F0167	Bacteria	Firmicutes	Clostridia	Clostridiales	Lachnospiraceae		csm5 family CRISPR-associated ramp protein	csm5gr7	cd09662	CAS-III-A			
4499..5441	-	EGG91034.1	GL890597.1	Lachnospiraceae oral taxon 107 str. F0167	Bacteria	Firmicutes	Clostridia	Clostridiales	Lachnospiraceae		csm4 family CRISPR-associated ramp protein	csm4gr5	COG1567	CAS-III-A			
5442..6117	-	EGG91035.1	GL890597.1	Lachnospiraceae oral taxon 107 str. F0167	Bacteria	Firmicutes	Clostridia	Clostridiales	Lachnospiraceae		csm3 family CRISPR-associated ramp protein	csm3gr7	cd09684	CAS-III-A,CAS-III-D			
6129..6570	-	EGG91036.1	GL890597.1	Lachnospiraceae oral taxon 107 str. F0167	Bacteria	Firmicutes	Clostridia	Clostridiales	Lachnospiraceae		hypothetical protein	csm2gr11	cd09647	CAS-III-A			
6570..8778	-	EGG91037.1	GL890597.1	Lachnospiraceae oral taxon 107 str. F0167	Bacteria	Firmicutes	Clostridia	Clostridiales	Lachnospiraceae		csm1 family CRISPR-associated protein	cas10	cd09680	CAS-III			
8770..9502	-	EGG91038.1	GL890597.1	Lachnospiraceae oral taxon 107 str. F0167	Bacteria	Firmicutes	Clostridia	Clostridiales	Lachnospiraceae		CRISPR-associated protein cas6	cas6	pfam10040	CAS-III,CAS-IV			
9495..10884	-	EGG91039.1	GL890597.1	Lachnospiraceae oral taxon 107 str. F0167	Bacteria	Firmicutes	Clostridia	Clostridiales	Lachnospiraceae		hypothetical protein	csm6	cd09746	CAS-III-A			
11042..11675	-	EGG91040.1	GL890597.1	Lachnospiraceae oral taxon 107 str. F0167	Bacteria	Firmicutes	Clostridia	Clostridiales	Lachnospiraceae		hypothetical protein						
12336..12504	-	EGG91041.1	GL890597.1	Lachnospiraceae oral taxon 107 str. F0167	Bacteria	Firmicutes	Clostridia	Clostridiales	Lachnospiraceae		hypothetical protein						
13082..13342	array		GL890597.1	Lachnospiraceae oral taxon 107 str. F0167	Bacteria	Firmicutes	Clostridia	Clostridiales	Lachnospiraceae			CRISPR array			4	37	37

231 CAS-III-A | AYB00351.1

2270572..2271484	-	AYB00349.1	CP032364.1	Lachnoanaerobaculum umeaense	Bacteria	Firmicutes	Clostridia	Clostridiales	Lachnospiraceae	Lachnoanaerobaculum	transposase	C3a178	cluster178				
2271636..2272032	array		CP032364.1	Lachnoanaerobaculum umeaense	Bacteria	Firmicutes	Clostridia	Clostridiales	Lachnospiraceae	Lachnoanaerobaculum		CRISPR array			6	43	30
2272325..2274482	-	AYB00350.1	CP032364.1	Lachnoanaerobaculum umeaense	Bacteria	Firmicutes	Clostridia	Clostridiales	Lachnospiraceae	Lachnoanaerobaculum	anaerobic ribonucleoside-triphosphate reductase						
2274514..2274814	-	AYB00351.1	CP032364.1	Lachnoanaerobaculum umeaense	Bacteria	Firmicutes	Clostridia	Clostridiales	Lachnospiraceae	Lachnoanaerobaculum	CRISPR-associated endonuclease Cas2	**cas2**	cd09725	CAS-I,CAS-II,CAS-III,CAS-V			
2275670..2276810	-	AYB00352.1	CP032364.1	Lachnoanaerobaculum umeaense	Bacteria	Firmicutes	Clostridia	Clostridiales	Lachnospiraceae	Lachnoanaerobaculum	type III-A CRISPR-associated RAMP protein Csm5	csm5gr7	cd09662	CAS-III-A			
2276809..2277751	-	AYB00353.1	CP032364.1	Lachnoanaerobaculum umeaense	Bacteria	Firmicutes	Clostridia	Clostridiales	Lachnospiraceae	Lachnoanaerobaculum	type III-A CRISPR-associated RAMP protein Csm4	csm4gr5	COG1567	CAS-III-A			
2277752..2278430	-	AYB00354.1	CP032364.1	Lachnoanaerobaculum umeaense	Bacteria	Firmicutes	Clostridia	Clostridiales	Lachnospiraceae	Lachnoanaerobaculum	type III-A CRISPR-associated RAMP protein Csm3	csm3gr7	cd09684	CAS-III-A,CAS-III-D			
2278440..2278890	-	AYB00355.1	CP032364.1	Lachnoanaerobaculum umeaense	Bacteria	Firmicutes	Clostridia	Clostridiales	Lachnospiraceae	Lachnoanaerobaculum	type III-A CRISPR-associated protein Csm2	csm2gr11	cd09647	CAS-III-A			
2278890..2281098	-	AYB00356.1	CP032364.1	Lachnoanaerobaculum umeaense	Bacteria	Firmicutes	Clostridia	Clostridiales	Lachnospiraceae	Lachnoanaerobaculum	type III-A CRISPR-associated protein Cas10/Csm1	cas10	cd09680	CAS-III			
2281090..2281822	-	AYB00357.1	CP032364.1	Lachnoanaerobaculum umeaense	Bacteria	Firmicutes	Clostridia	Clostridiales	Lachnospiraceae	Lachnoanaerobaculum	CRISPR-associated endoribonuclease Cas6	cas6	COG5551	CAS-III			
2281815..2283204	-	AYB00358.1	CP032364.1	Lachnoanaerobaculum umeaense	Bacteria	Firmicutes	Clostridia	Clostridiales	Lachnospiraceae	Lachnoanaerobaculum	CRISPR-associated protein Csm6	csm6	cd09746	CAS-III-A			
2283406..2284039	-	AYB00359.1	CP032364.1	Lachnoanaerobaculum umeaense	Bacteria	Firmicutes	Clostridia	Clostridiales	Lachnospiraceae	Lachnoanaerobaculum	EcsC family protein						
2285156..2286218	+	AYB00360.1	CP032364.1	Lachnoanaerobaculum umeaense	Bacteria	Firmicutes	Clostridia	Clostridiales	Lachnospiraceae	Lachnoanaerobaculum	WYL domain-containing protein	WYL	pfam13280	CAS-I,CAS-III			

232 partial | EIC95117.1

414..1497	-	EIC95120.1	AJGH01000099.1	Lachnoanaerobaculum saburreum F0468	Bacteria	Firmicutes	Clostridia	Clostridiales	Lachnospiraceae	Lachnoanaerobaculum	anaerobic ribonucleoside-triphosphate reductase domain protein						
1531..1822	-	EIC95117.1	AJGH01000099.1	Lachnoanaerobaculum saburreum F0468	Bacteria	Firmicutes	Clostridia	Clostridiales	Lachnospiraceae	Lachnoanaerobaculum	CRISPR-associated endoribonuclease Cas2	**cas2**	cd09725	CAS-I,CAS-II,CAS-III,CAS-V			
1827..2256	-	EIC95118.1	AJGH01000099.1	Lachnoanaerobaculum saburreum F0468	Bacteria	Firmicutes	Clostridia	Clostridiales	Lachnospiraceae	Lachnoanaerobaculum	CRISPR-associated endonuclease Cas1-like protein	cas1	cd09634	CAS-I,CAS-II,CAS-III,CAS-V			
2308..2733	-	EIC95121.1	AJGH01000099.1	Lachnoanaerobaculum saburreum F0468	Bacteria	Firmicutes	Clostridia	Clostridiales	Lachnospiraceae	Lachnoanaerobaculum	transposase, IS605 OrfB family	c2c9	c2c9	CAS-V-U4			

233 CAS-III-D | EJZ68973.1

2798538..2799459	+	EJZ68961.1	JH815185.1	Lachnoanaerobaculum sp. OBRC5-5	Bacteria	Firmicutes	Clostridia	Clostridiales	Lachnospiraceae	Lachnoanaerobaculum	hypothetical protein	C3a178	cluster178				
2801325..2802060	+	EJZ68962.1	JH815185.1	Lachnoanaerobaculum sp. OBRC5-5	Bacteria	Firmicutes	Clostridia	Clostridiales	Lachnospiraceae	Lachnoanaerobaculum	CRISPR-associated endoribonuclease cas6	cas6	COG5551	CAS-III			
2802162..2802261	+	EJZ68963.1	JH815185.1	Lachnoanaerobaculum sp. OBRC5-5	Bacteria	Firmicutes	Clostridia	Clostridiales	Lachnospiraceae	Lachnoanaerobaculum	hypothetical protein						
2802483..2803128	+	EJZ68964.1	JH815185.1	Lachnoanaerobaculum sp. OBRC5-5	Bacteria	Firmicutes	Clostridia	Clostridiales	Lachnospiraceae	Lachnoanaerobaculum	hypothetical protein	csx1	cd09732	CAS-III			
2803145..2804444	+	EJZ68965.1	JH815185.1	Lachnoanaerobaculum sp. OBRC5-5	Bacteria	Firmicutes	Clostridia	Clostridiales	Lachnospiraceae	Lachnoanaerobaculum	hypothetical protein						
2804455..2806036	+	EJZ68966.1	JH815185.1	Lachnoanaerobaculum sp. OBRC5-5	Bacteria	Firmicutes	Clostridia	Clostridiales	Lachnospiraceae	Lachnoanaerobaculum	hypothetical protein	cas10	mkCas0133	CAS-III			
2806028..2808038	+	EJZ68967.1	JH815185.1	Lachnoanaerobaculum sp. OBRC5-5	Bacteria	Firmicutes	Clostridia	Clostridiales	Lachnospiraceae	Lachnoanaerobaculum	hypothetical protein	csm3gr7,csx10gr5	mkCas0198,cls000715	CAS-III-A,CAS-III-D			
2808034..2809258	+	EJZ68968.1	JH815185.1	Lachnoanaerobaculum sp. OBRC5-5	Bacteria	Firmicutes	Clostridia	Clostridiales	Lachnospiraceae	Lachnoanaerobaculum	hypothetical protein	csm3gr7,csm3gr7	cd09726,pfam03787	CAS-III-D			
2809260..2809776	+	EJZ68969.1	JH815185.1	Lachnoanaerobaculum sp. OBRC5-5	Bacteria	Firmicutes	Clostridia	Clostridiales	Lachnospiraceae	Lachnoanaerobaculum	tigr03984 family CRISPR-associated protein	csx19,csx19	mkCas0147,cls001376	CAS-III-D			
2809777..2811808	+	EJZ68970.1	JH815185.1	Lachnoanaerobaculum sp. OBRC5-5	Bacteria	Firmicutes	Clostridia	Clostridiales	Lachnospiraceae	Lachnoanaerobaculum	CRISPR-associated protein	csm3gr7	cls000253	CAS-III-A,CAS-III-D			
2811809..2813153	+	EJZ68971.1	JH815185.1	Lachnoanaerobaculum sp. OBRC5-5	Bacteria	Firmicutes	Clostridia	Clostridiales	Lachnospiraceae	Lachnoanaerobaculum	tm1812 family CRISPR-associated protein	csx1	cd09732	CAS-III			
2813170..2814175	+	EJZ68972.1	JH815185.1	Lachnoanaerobaculum sp. OBRC5-5	Bacteria	Firmicutes	Clostridia	Clostridiales	Lachnospiraceae	Lachnoanaerobaculum	CRISPR-associated endonuclease cas1	cas1	cd09634	CAS-I,CAS-II,CAS-III,CAS-V			
2814174..2814471	+	EJZ68973.1	JH815185.1	Lachnoanaerobaculum sp. OBRC5-5	Bacteria	Firmicutes	Clostridia	Clostridiales	Lachnospiraceae	Lachnoanaerobaculum	CRISPR-associated endoribonuclease cas2	**cas2**	cd09725	CAS-I,CAS-II,CAS-III,CAS-V			
2814506..2816663	+	EJZ68974.1	JH815185.1	Lachnoanaerobaculum sp. OBRC5-5	Bacteria	Firmicutes	Clostridia	Clostridiales	Lachnospiraceae	Lachnoanaerobaculum	anaerobic ribonucleoside-triphosphate reductase						
2816952..2818337	array		JH815185.1	Lachnoanaerobaculum sp. OBRC5-5	Bacteria	Firmicutes	Clostridia	Clostridiales	Lachnospiraceae	Lachnoanaerobaculum		CRISPR array			19	39	36
2818482..2819382	+	EJZ68975.1	JH815185.1	Lachnoanaerobaculum sp. OBRC5-5	Bacteria	Firmicutes	Clostridia	Clostridiales	Lachnospiraceae	Lachnoanaerobaculum	hypothetical protein	C3a178	cluster178				

235 CAS-III-A | EAX48914.1

202009..203311	-	EAX48906.1	AAWL01000001.1	Thermosinus carboxydivorans Nor1	Bacteria	Firmicutes	Negativicutes	Selenomonadales	Sporomusaceae	Thermosinus	hypothetical protein	csx1	cd09747	CAS-III			
203325..204516	-	EAX48907.1	AAWL01000001.1	Thermosinus carboxydivorans Nor1	Bacteria	Firmicutes	Negativicutes	Selenomonadales	Sporomusaceae	Thermosinus	hypothetical protein	csx1	pfam09002	CAS-III			
204522..205386	-	EAX48908.1	AAWL01000001.1	Thermosinus carboxydivorans Nor1	Bacteria	Firmicutes	Negativicutes	Selenomonadales	Sporomusaceae	Thermosinus	conserved hypothetical protein	cas6	pfam10040	CAS-III,CAS-IV			
205382..206567	-	EAX48909.1	AAWL01000001.1	Thermosinus carboxydivorans Nor1	Bacteria	Firmicutes	Negativicutes	Selenomonadales	Sporomusaceae	Thermosinus	CRISPR-associated RAMP protein, Csm5 family	csm5gr7	COG1332	CAS-III-A			
206563..207589	-	EAX48910.1	AAWL01000001.1	Thermosinus carboxydivorans Nor1	Bacteria	Firmicutes	Negativicutes	Selenomonadales	Sporomusaceae	Thermosinus	CRISPR-associated RAMP protein, Csm4 family	csm4gr5	COG1567	CAS-III-A			
207569..208286	-	EAX48911.1	AAWL01000001.1	Thermosinus carboxydivorans Nor1	Bacteria	Firmicutes	Negativicutes	Selenomonadales	Sporomusaceae	Thermosinus	CRISPR-associated RAMP protein, Csm3 family	csm3gr7	cd09684	CAS-III-A,CAS-III-D			

208288..208693	-	EAX48912.1	AAWL01000001.1	Thermosinus carboxydivorans Nor1	Bacteria	Firmicutes	Negativicutes	Selenomonadales	Sporomusaceae	Thermosinus	CRISPR-associated protein, Csm2 family	csm2gr11	pfam03750	CAS-III-A			
208692..211209	-	EAX48913.1	AAWL01000001.1	Thermosinus carboxydivorans Nor1	Bacteria	Firmicutes	Negativicutes	Selenomonadales	Sporomusaceae	Thermosinus	CRISPR-associated protein, Csm1 family	cas10	cd09680	CAS-III			
211443..214298	array		AAWL01000001.1	Thermosinus carboxydivorans Nor1	Bacteria	Firmicutes	Negativicutes	Selenomonadales	Sporomusaceae	Thermosinus		CRISPR array			37	43	35
214471..214807	-	EAX48914.1	AAWL01000001.1	Thermosinus carboxydivorans Nor1	Bacteria	Firmicutes	Negativicutes	Selenomonadales	Sporomusaceae	Thermosinus	CRISPR-associated protein Cas2	cas2	pfam09827	CAS-I,CAS-II,CAS-III			
214833..215832	-	EAX48915.1	AAWL01000001.1	Thermosinus carboxydivorans Nor1	Bacteria	Firmicutes	Negativicutes	Selenomonadales	Sporomusaceae	Thermosinus	CRISPR-associated protein Cas1	cas1	cd09634	CAS-I,CAS-II,CAS-III,CAS-V			
236 CAS-III-A | EAX49041.1																	
198420..200423	array		AAWL01000001.1	Thermosinus carboxydivorans Nor1	Bacteria	Firmicutes	Negativicutes	Selenomonadales	Sporomusaceae	Thermosinus		CRISPR array			27	40	35
200646..201645	-	EAX48905.1	AAWL01000001.1	Thermosinus carboxydivorans Nor1	Bacteria	Firmicutes	Negativicutes	Selenomonadales	Sporomusaceae	Thermosinus	CRISPR-associated protein Cas1	cas1	cd09634	CAS-I,CAS-II,CAS-III,CAS-V			
201664..202000	-	EAX49041.1	AAWL01000001.1	Thermosinus carboxydivorans Nor1	Bacteria	Firmicutes	Negativicutes	Selenomonadales	Sporomusaceae	Thermosinus	CRISPR-associated protein Cas2	cas2	cd09725	CAS-I,CAS-II,CAS-III,CAS-V			
202009..203311	-	EAX48906.1	AAWL01000001.1	Thermosinus carboxydivorans Nor1	Bacteria	Firmicutes	Negativicutes	Selenomonadales	Sporomusaceae	Thermosinus	hypothetical protein	csx1	cd09747	CAS-III			
203325..204516	-	EAX48907.1	AAWL01000001.1	Thermosinus carboxydivorans Nor1	Bacteria	Firmicutes	Negativicutes	Selenomonadales	Sporomusaceae	Thermosinus	hypothetical protein	csx1	pfam09002	CAS-III			
204522..205386	-	EAX48908.1	AAWL01000001.1	Thermosinus carboxydivorans Nor1	Bacteria	Firmicutes	Negativicutes	Selenomonadales	Sporomusaceae	Thermosinus	conserved hypothetical protein	cas6	pfam10040	CAS-III,CAS-IV			
205382..206567	-	EAX48909.1	AAWL01000001.1	Thermosinus carboxydivorans Nor1	Bacteria	Firmicutes	Negativicutes	Selenomonadales	Sporomusaceae	Thermosinus	CRISPR-associated RAMP protein, Csm5 family	csm5gr7	COG1332	CAS-III-A			
206563..207589	-	EAX48910.1	AAWL01000001.1	Thermosinus carboxydivorans Nor1	Bacteria	Firmicutes	Negativicutes	Selenomonadales	Sporomusaceae	Thermosinus	CRISPR-associated RAMP protein, Csm4 family	csm4gr5	COG1567	CAS-III-A			
207569..208286	-	EAX48911.1	AAWL01000001.1	Thermosinus carboxydivorans Nor1	Bacteria	Firmicutes	Negativicutes	Selenomonadales	Sporomusaceae	Thermosinus	CRISPR-associated RAMP protein, Csm3 family	csm3gr7	cd09684	CAS-III-A,CAS-III-D			
208288..208693	-	EAX48912.1	AAWL01000001.1	Thermosinus carboxydivorans Nor1	Bacteria	Firmicutes	Negativicutes	Selenomonadales	Sporomusaceae	Thermosinus	CRISPR-associated protein, Csm2 family	csm2gr11	pfam03750	CAS-III-A			
208692..211209	-	EAX48913.1	AAWL01000001.1	Thermosinus carboxydivorans Nor1	Bacteria	Firmicutes	Negativicutes	Selenomonadales	Sporomusaceae	Thermosinus	CRISPR-associated protein, Csm1 family	cas10	cd09680	CAS-III			
237 CAS-VI-A | SEQ67639.1																	
72908..77072	+	SEQ67578.1	FOEK01000016.1	Lachnospiraceae bacterium NE2001	Bacteria	Firmicutes	Clostridia	Clostridiales	Lachnospiraceae		hypothetical protein	cas13a	cas13a	CAS-VI-A			
77161..78199	+	SEQ67608.1	FOEK01000016.1	Lachnospiraceae bacterium NE2001	Bacteria	Firmicutes	Clostridia	Clostridiales	Lachnospiraceae		CRISP-associated protein Cas1	cas1	cd09634	CAS-I,CAS-II,CAS-III,CAS-V			
78207..78495	+	SEQ67639.1	FOEK01000016.1	Lachnospiraceae bacterium NE2001	Bacteria	Firmicutes	Clostridia	Clostridiales	Lachnospiraceae		CRISPR associated protein Cas2	cas2	cd09725	CAS-I,CAS-II,CAS-III,CAS-V			
78625..79288	array		FOEK01000016.1	Lachnospiraceae bacterium NE2001	Bacteria	Firmicutes	Clostridia	Clostridiales	Lachnospiraceae			CRISPR array			10	33	36
238 partial | SCJ11411.1																	
198188..198863	array		FMHC01000001.1	uncultured Eubacterium sp.	Bacteria	Firmicutes	Clostridia	Clostridiales	Eubacteriaceae	Eubacterium		CRISPR array			10	35	36
199058..199376	+	SCJ11411.1	FMHC01000001.1	uncultured Eubacterium sp.	Bacteria	Firmicutes	Clostridia	Clostridiales	Eubacteriaceae	Eubacterium	CRISPR-associated endoribonuclease Cas2	cas2	cd09725	CAS-I,CAS-II,CAS-III,CAS-V			
239 CAS-VI-A | SFR81225.1																	
1530..2330	array		FOZC01000010.1	[Clostridium] aminophilum	Bacteria	Firmicutes	Clostridia	Clostridiales	Lachnospiraceae	Lachnoclostridium		CRISPR array			12	34	35
2499..2781	+	SFR81207.1	FOZC01000010.1	[Clostridium] aminophilum	Bacteria	Firmicutes	Clostridia	Clostridiales	Lachnospiraceae	Lachnoclostridium	hypothetical protein						
2979..3984	+	SFR81217.1	FOZC01000010.1	[Clostridium] aminophilum	Bacteria	Firmicutes	Clostridia	Clostridiales	Lachnospiraceae	Lachnoclostridium	CRISP-associated protein Cas1	cas1	cd09634	CAS-I,CAS-II,CAS-III,CAS-V			
3991..4276	+	SFR81225.1	FOZC01000010.1	[Clostridium] aminophilum	Bacteria	Firmicutes	Clostridia	Clostridiales	Lachnospiraceae	Lachnoclostridium	CRISPR-associated protein Cas2	cas2	pfam09827	CAS-I,CAS-II,CAS-III			
4444..6202	+	SFR81231.1	FOZC01000010.1	[Clostridium] aminophilum	Bacteria	Firmicutes	Clostridia	Clostridiales	Lachnospiraceae	Lachnoclostridium	hypothetical protein						
6191..7937	+	SFR81241.1	FOZC01000010.1	[Clostridium] aminophilum	Bacteria	Firmicutes	Clostridia	Clostridiales	Lachnospiraceae	Lachnoclostridium	hypothetical protein						
7937..10130	+	SFR81248.1	FOZC01000010.1	[Clostridium] aminophilum	Bacteria	Firmicutes	Clostridia	Clostridiales	Lachnospiraceae	Lachnoclostridium	hypothetical protein						
10185..14343	+	SFR81258.1	FOZC01000010.1	[Clostridium] aminophilum	Bacteria	Firmicutes	Clostridia	Clostridiales	Lachnospiraceae	Lachnoclostridium	hypothetical protein	cas13a	cas13a	CAS-VI-A			
240 CAS-III-A | EHL19060.1																	
88..1149	array		JH414597.1	Peptoanaerobacter stomatis	Bacteria	Firmicutes	Clostridia	Clostridiales	Peptostreptococcaceae	Peptoanaerobacter		CRISPR array			14	42	36
1293..1608	-	EHL19060.1	JH414597.1	Peptoanaerobacter stomatis	Bacteria	Firmicutes	Clostridia	Clostridiales	Peptostreptococcaceae	Peptoanaerobacter	CRISPR-associated protein cas2	cas2	cd09725	CAS-I,CAS-II,CAS-III,CAS-V			
1612..2614	-	EHL19061.1	JH414597.1	Peptoanaerobacter stomatis	Bacteria	Firmicutes	Clostridia	Clostridiales	Peptostreptococcaceae	Peptoanaerobacter	CRISPR-associated protein cas1	cas1	cd09634	CAS-I,CAS-II,CAS-III,CAS-V			
2830..2953	+	EHL19062.1	JH414597.1	Peptoanaerobacter stomatis	Bacteria	Firmicutes	Clostridia	Clostridiales	Peptostreptococcaceae	Peptoanaerobacter	hypothetical protein						
2968..3058	-	EHL19063.1	JH414597.1	Peptoanaerobacter stomatis	Bacteria	Firmicutes	Clostridia	Clostridiales	Peptostreptococcaceae	Peptoanaerobacter	hypothetical protein						
3709..5017	+	EHL19064.1	JH414597.1	Peptoanaerobacter stomatis	Bacteria	Firmicutes	Clostridia	Clostridiales	Peptostreptococcaceae	Peptoanaerobacter	hypothetical protein	WYL	pfam13280	CAS-I,CAS-III			
5003..6092	+	EHL19065.1	JH414597.1	Peptoanaerobacter stomatis	Bacteria	Firmicutes	Clostridia	Clostridiales	Peptostreptococcaceae	Peptoanaerobacter	hypothetical protein	WYL	pfam13280	CAS-I,CAS-III			
6235..7807	-	EHL19066.1	JH414597.1	Peptoanaerobacter stomatis	Bacteria	Firmicutes	Clostridia	Clostridiales	Peptostreptococcaceae	Peptoanaerobacter	hypothetical protein	csm6	pfam09659	CAS-III-A			
7803..8514	-	EHL19067.1	JH414597.1	Peptoanaerobacter stomatis	Bacteria	Firmicutes	Clostridia	Clostridiales	Peptostreptococcaceae	Peptoanaerobacter	hypothetical protein	cas6	pfam10040	CAS-III,CAS-IV			
8497..9664	-	EHL19068.1	JH414597.1	Peptoanaerobacter stomatis	Bacteria	Firmicutes	Clostridia	Clostridiales	Peptostreptococcaceae	Peptoanaerobacter	hypothetical protein	csm5gr7	cd09662	CAS-III-A			
9651..10656	-	EHL19069.1	JH414597.1	Peptoanaerobacter stomatis	Bacteria	Firmicutes	Clostridia	Clostridiales	Peptostreptococcaceae	Peptoanaerobacter	csm4 family CRISPR-associated ramp protein	csm4gr5	cd09663	CAS-III-A			
10655..11342	-	EHL19070.1	JH414597.1	Peptoanaerobacter stomatis	Bacteria	Firmicutes	Clostridia	Clostridiales	Peptostreptococcaceae	Peptoanaerobacter	csm3 family CRISPR-associated ramp protein	csm3gr7	cd09684	CAS-III-A,CAS-III-D			
11356..11806	-	EHL19071.1	JH414597.1	Peptoanaerobacter stomatis	Bacteria	Firmicutes	Clostridia	Clostridiales	Peptostreptococcaceae	Peptoanaerobacter	hypothetical protein	csm2gr11	cd09647	CAS-III-A			
11792..14378	-	EHL19072.1	JH414597.1	Peptoanaerobacter stomatis	Bacteria	Firmicutes	Clostridia	Clostridiales	Peptostreptococcaceae	Peptoanaerobacter	csm1 family CRISPR-associated protein	cas10	cd09680	CAS-III			
241 CAS-III-A | ALQ42641.1																	
1567152..1567476	-	ALQ42641.1	CP013328.1	Fusobacterium nucleatum subsp. polymorphum	Bacteria	Fusobacteria	Fusobacteriia	Fusobacteriales	Fusobacteriaceae	Fusobacterium	CRISPR-associated protein Cas2	cas2	cd09725	CAS-I,CAS-II,CAS-III,CAS-V			
1567481..1568489	-	ALQ42642.1	CP013328.1	Fusobacterium nucleatum subsp. polymorphum	Bacteria	Fusobacteria	Fusobacteriia	Fusobacteriales	Fusobacteriaceae	Fusobacterium	CRISPR-associated protein Cas1	cas1	cd09634	CAS-I,CAS-II,CAS-III,CAS-V			
1568502..1569897	-	ALQ42643.1	CP013328.1	Fusobacterium nucleatum subsp. polymorphum	Bacteria	Fusobacteria	Fusobacteriia	Fusobacteriales	Fusobacteriaceae	Fusobacterium	CRISPR-associated protein	csx1,csm6	pfam09670,pfam09659	CAS-III			
1569889..1570612	-	ALQ42644.1	CP013328.1	Fusobacterium nucleatum subsp. polymorphum	Bacteria	Fusobacteria	Fusobacteriia	Fusobacteriales	Fusobacteriaceae	Fusobacterium	CRISPR-associated protein Cas6	cas6	pfam10040	CAS-III,CAS-IV			
1570595..1571765	-	ALQ42645.1	CP013328.1	Fusobacterium nucleatum subsp. polymorphum	Bacteria	Fusobacteria	Fusobacteriia	Fusobacteriales	Fusobacteriaceae	Fusobacterium	type III-A CRISPR-associated RAMP protein Csm5	csm5gr7	COG1332	CAS-III-A			
1571761..1572766	-	ALQ42646.1	CP013328.1	Fusobacterium nucleatum subsp. polymorphum	Bacteria	Fusobacteria	Fusobacteriia	Fusobacteriales	Fusobacteriaceae	Fusobacterium	type III-A CRISPR-associated RAMP protein Csm4	csm4gr5	COG1567	CAS-III-A			
1572765..1573473	-	ALQ42647.1	CP013328.1	Fusobacterium nucleatum subsp. polymorphum	Bacteria	Fusobacteria	Fusobacteriia	Fusobacteriales	Fusobacteriaceae	Fusobacterium	type III-A CRISPR-associated RAMP protein Csm3	csm3gr7	cd09684	CAS-III-A,CAS-III-D			
1573509..1573875	-	ALQ42648.1	CP013328.1	Fusobacterium nucleatum subsp. polymorphum	Bacteria	Fusobacteria	Fusobacteriia	Fusobacteriales	Fusobacteriaceae	Fusobacterium	type III-A CRISPR-associated protein Csm2	csm2gr11	cd09647	CAS-III-A			
1573878..1576404	-	ALQ42649.1	CP013328.1	Fusobacterium nucleatum subsp. polymorphum	Bacteria	Fusobacteria	Fusobacteriia	Fusobacteriales	Fusobacteriaceae	Fusobacterium	type III-A CRISPR-associated protein Cas10/Csm1	cas10	cd09680	CAS-III			
242 partial CAS-III-A | SFE73898.1																	
491..1349	+	SFE73823.1	FONP01000010.1	Peptostreptococcus sp. D1	Bacteria	Firmicutes	Clostridia	Clostridiales	Peptostreptococcaceae	Peptostreptococcus	CRISP-associated protein Cas1	cas1	cd09634	CAS-I,CAS-II,CAS-III,CAS-V			
1345..1486	+	SFE73888.1	FONP01000010.1	Peptostreptococcus sp. D1	Bacteria	Firmicutes	Clostridia	Clostridiales	Peptostreptococcaceae	Peptostreptococcus	hypothetical protein						
1492..1825	+	SFE73898.1	FONP01000010.1	Peptostreptococcus sp. D1	Bacteria	Firmicutes	Clostridia	Clostridiales	Peptostreptococcaceae	Peptostreptococcus	CRISPR-associated protein Cas2	cas2	cd09725	CAS-I,CAS-II,CAS-III,CAS-V			
1839..3183	+	SFE73908.1	FONP01000010.1	Peptostreptococcus sp. D1	Bacteria	Firmicutes	Clostridia	Clostridiales	Peptostreptococcaceae	Peptostreptococcus	CRISPR type III-A/MTUBE-associated protein Csm6	csm6	cd09746	CAS-III-A			
243 CAS-III-A | ASS38486.1																	
338281..339031	+	ASS37296.1	CP016199.1	Mogibacterium pumilum	Bacteria	Firmicutes	Clostridia	Clostridiales	Clostridiales Family XIII. Incertae Sedis	Mogibacterium	CRISPR-associated endoribonuclease Cas6	cas6	COG5551	CAS-III			
339034..341404	+	ASS37297.1	CP016199.1	Mogibacterium pumilum	Bacteria	Firmicutes	Clostridia	Clostridiales	Clostridiales Family XIII. Incertae Sedis	Mogibacterium	type III-A CRISPR-associated protein Cas10/Csm1	cas10	cd09680	CAS-III			
341406..341829	+	ASS37298.1	CP016199.1	Mogibacterium pumilum	Bacteria	Firmicutes	Clostridia	Clostridiales	Clostridiales Family XIII. Incertae Sedis	Mogibacterium	type III-A CRISPR-associated protein Csm2	csm2gr11	cd09647	CAS-III-A			
341843..342518	+	ASS37299.1	CP016199.1	Mogibacterium pumilum	Bacteria	Firmicutes	Clostridia	Clostridiales	Clostridiales Family XIII. Incertae Sedis	Mogibacterium	type III-A CRISPR-associated RAMP protein Csm3	csm3gr7	cd09684	CAS-III-A,CAS-III-D			
342520..343447	+	ASS37300.1	CP016199.1	Mogibacterium pumilum	Bacteria	Firmicutes	Clostridia	Clostridiales	Clostridiales Family XIII. Incertae Sedis	Mogibacterium	type III-A CRISPR-associated RAMP protein Csm4	csm4gr5	COG1567	CAS-III-A			
343436..344561	+	ASS37301.1	CP016199.1	Mogibacterium pumilum	Bacteria	Firmicutes	Clostridia	Clostridiales	Clostridiales Family XIII. Incertae Sedis	Mogibacterium	type III-A CRISPR-associated RAMP protein Csm5	csm5gr7,csm5gr7	cd09662,cd09662	CAS-III-A			
344690..345368	array		CP016199.1	Mogibacterium pumilum	Bacteria	Firmicutes	Clostridia	Clostridiales	Clostridiales Family XIII. Incertae Sedis	Mogibacterium		CRISPR array			10	36	35
345434..345785	+	ASS37302.1	CP016199.1	Mogibacterium pumilum	Bacteria	Firmicutes	Clostridia	Clostridiales	Clostridiales Family XIII. Incertae Sedis	Mogibacterium	hypothetical protein						
345835..346287	array		CP016199.1	Mogibacterium pumilum	Bacteria	Firmicutes	Clostridia	Clostridiales	Clostridiales Family XIII. Incertae Sedis	Mogibacterium		CRISPR array			7	51	21
346324..347329	+	ASS37303.1	CP016199.1	Mogibacterium pumilum	Bacteria	Firmicutes	Clostridia	Clostridiales	Clostridiales Family XIII. Incertae Sedis	Mogibacterium	CRISPR-associated endonuclease Cas1	cas1	cd09634	CAS-I,CAS-II,CAS-III,CAS-V			
347351..347663	+	ASS38486.1	CP016199.1	Mogibacterium pumilum	Bacteria	Firmicutes	Clostridia	Clostridiales	Clostridiales Family XIII. Incertae Sedis	Mogibacterium	CRISPR-associated endonuclease Cas2	cas2	cd09725	CAS-I,CAS-II,CAS-III,CAS-V			
347702..348713	+	ASS37304.1	CP016199.1	Mogibacterium pumilum	Bacteria	Firmicutes	Clostridia	Clostridiales	Clostridiales Family XIII. Incertae Sedis	Mogibacterium	hypothetical protein	csm6	pfam09659	CAS-III-A			
244 CAS-III-A | VEJ34624.1																	
236093..236852	+	VEJ34611.1	LR134523.1	Peptoniphilus ivorii	Bacteria	Firmicutes	Tissierellia	Tissierellales	Peptoniphilaceae	Peptoniphilus	CRISPR-associated endoribonuclease Cas6	cas6	pfam10040	CAS-III,CAS-IV			
236827..239158	+	VEJ34613.1	LR134523.1	Peptoniphilus ivorii	Bacteria	Firmicutes	Tissierellia	Tissierellales	Peptoniphilaceae	Peptoniphilus	CRISPR-associated protein Cas10/Csm1, subtype III-A/MTUBE	cas10	cd09680	CAS-III			
239147..239588	+	VEJ34615.1	LR134523.1	Peptoniphilus ivorii	Bacteria	Firmicutes	Tissierellia	Tissierellales	Peptoniphilaceae	Peptoniphilus	CRISPR type III-A/MTUBE-associated protein Csm2	csm2gr11	cd09647	CAS-III-A			
239587..240232	+	VEJ34617.1	LR134523.1	Peptoniphilus ivorii	Bacteria	Firmicutes	Tissierellia	Tissierellales	Peptoniphilaceae	Peptoniphilus	CRISPR type III-A/MTUBE-associated RAMP protein Csm3	csm3gr7	cd09684	CAS-III-A,CAS-III-D			
240233..241178	+	VEJ34619.1	LR134523.1	Peptoniphilus ivorii	Bacteria	Firmicutes	Tissierellia	Tissierellales	Peptoniphilaceae	Peptoniphilus	CRISPR type III-A/MTUBE-associated RAMP protein Csm4	csm4gr5	COG1567	CAS-III-A			
241174..242278	+	VEJ34621.1	LR134523.1	Peptoniphilus ivorii	Bacteria	Firmicutes	Tissierellia	Tissierellales	Peptoniphilaceae	Peptoniphilus	CRISPR type III-A/MTUBE-associated RAMP protein Csm5	csm5gr7,csm5gr7	cd09662,COG1332	CAS-III-A			
242274..243267	+	VEJ34623.1	LR134523.1	Peptoniphilus ivorii	Bacteria	Firmicutes	Tissierellia	Tissierellales	Peptoniphilaceae	Peptoniphilus	CRISPR-associated endonuclease Cas1	cas1	cd09634	CAS-I,CAS-II,CAS-III,CAS-V			
243254..243587	+	VEJ34624.1	LR134523.1	Peptoniphilus ivorii	Bacteria	Firmicutes	Tissierellia	Tissierellales	Peptoniphilaceae	Peptoniphilus	CRISPR-associated endoribonuclease Cas2	cas2	cd09725	CAS-I,CAS-II,CAS-III,CAS-V			
243595..244894	+	VEJ34626.1	LR134523.1	Peptoniphilus ivorii	Bacteria	Firmicutes	Tissierellia	Tissierellales	Peptoniphilaceae	Peptoniphilus	CRISPR type III-A/MTUBE-associated protein Csm6	csm6	cd09746	CAS-III-A			
244953..245091	-	VEJ34627.1	LR134523.1	Peptoniphilus ivorii	Bacteria	Firmicutes	Tissierellia	Tissierellales	Peptoniphilaceae	Peptoniphilus	Uncharacterised protein						
245038..245906	array		LR134523.1	Peptoniphilus ivorii	Bacteria	Firmicutes	Tissierellia	Tissierellales	Peptoniphilaceae	Peptoniphilus		CRISPR array			12	39	36
245 partial | RJW30883.1																	
190595..190850	+	RJW30883.1	QVHO01000001.1	Ruminococcus sp. OM02-16LB	Bacteria	Firmicutes	Clostridia	Clostridiales	Ruminococcaceae	Ruminococcus	CRISPR-associated endonuclease Cas2	cas2	cd09725	CAS-I,CAS-II,CAS-III,CAS-V			
246 CAS-VI-A | WP_062808329.1																	
1869431..1869746	-	WP_062808329.1	NZ_LT546010.1	Blautia sp. Marseille-P2398	Bacteria	Firmicutes	Clostridia	Clostridiales	Lachnospiraceae	Blautia	CRISPR-associated endonuclease Cas2	cas2	cd09725	CAS-I,CAS-II,CAS-III,CAS-V			
1869756..1870758	-	WP_062808097.1	NZ_LT546010.1	Blautia sp. Marseille-P2398	Bacteria	Firmicutes	Clostridia	Clostridiales	Lachnospiraceae	Blautia	CRISPR-associated endonuclease Cas1	cas1	cd09634	CAS-I,CAS-II,CAS-III,CAS-V			
1870784..1871888	array		NZ_LT546010.1	Blautia sp. Marseille-P2398	Bacteria	Firmicutes	Clostridia	Clostridiales	Lachnospiraceae	Blautia		CRISPR array			16	35	36
1872030..1875861	-	WP_062808098.1	NZ_LT546010.1	Blautia sp. Marseille-P2398	Bacteria	Firmicutes	Clostridia	Clostridiales	Lachnospiraceae	Blautia	hypothetical protein	cas13a	cas13a	CAS-VI-A			
247 CAS-III-A | OLR64066.1																	
520551..521017	array		MJIG01000003.1	Anaerostipes sp. 992a	Bacteria	Firmicutes	Clostridia	Clostridiales	Lachnospiraceae	Anaerostipes		CRISPR array			7	37	35
521184..522513	+	OLR64018.1	MJIG01000003.1	Anaerostipes sp. 992a	Bacteria	Firmicutes	Clostridia	Clostridiales	Lachnospiraceae	Anaerostipes	hypothetical protein	csm6	cd09746	CAS-III-A			
522514..523270	+	OLR64019.1	MJIG01000003.1	Anaerostipes sp. 992a	Bacteria	Firmicutes	Clostridia	Clostridiales	Lachnospiraceae	Anaerostipes	CRISPR-associated endoribonuclease Cas6	cas6	pfam10040	CAS-III,CAS-IV			
523259..525617	+	OLR64020.1	MJIG01000003.1	Anaerostipes sp. 992a	Bacteria	Firmicutes	Clostridia	Clostridiales	Lachnospiraceae	Anaerostipes	type III-A CRISPR-associated protein Cas10/Csm1	cas10,cas10	cd09680,cls000742	CAS-III			
525609..526041	+	OLR64021.1	MJIG01000003.1	Anaerostipes sp. 992a	Bacteria	Firmicutes	Clostridia	Clostridiales	Lachnospiraceae	Anaerostipes	type III-A CRISPR-associated protein Csm2	csm2gr11	cd09647	CAS-III-A			
526050..526713	+	OLR64022.1	MJIG01000003.1	Anaerostipes sp. 992a	Bacteria	Firmicutes	Clostridia	Clostridiales	Lachnospiraceae	Anaerostipes	type III-A CRISPR-associated RAMP protein Csm3	csm3gr7	cd09684	CAS-III-A,CAS-III-D			
526714..527647	+	OLR64023.1	MJIG01000003.1	Anaerostipes sp. 992a	Bacteria	Firmicutes	Clostridia	Clostridiales	Lachnospiraceae	Anaerostipes	type III-A CRISPR-associated RAMP protein Csm4	csm4gr5	COG1567	CAS-III-A			
527639..528752	+	OLR64024.1	MJIG01000003.1	Anaerostipes sp. 992a	Bacteria	Firmicutes	Clostridia	Clostridiales	Lachnospiraceae	Anaerostipes	type III-A CRISPR-associated RAMP protein Csm5	csm5gr7,csm5gr7	cd09662,cd09662	CAS-III-A			
528882..529641	array		MJIG01000003.1	Anaerostipes sp. 992a	Bacteria	Firmicutes	Clostridia	Clostridiales	Lachnospiraceae	Anaerostipes		CRISPR array			11	36	36
529673..530678	+	OLR64025.1	MJIG01000003.1	Anaerostipes sp. 992a	Bacteria	Firmicutes	Clostridia	Clostridiales	Lachnospiraceae	Anaerostipes	CRISPR-associated endonuclease Cas1	cas1	cd09634	CAS-I,CAS-II,CAS-III,CAS-V			
530694..531009	+	OLR64066.1	MJIG01000003.1	Anaerostipes sp. 992a	Bacteria	Firmicutes	Clostridia	Clostridiales	Lachnospiraceae	Anaerostipes	CRISPR-associated endonuclease Cas2	cas2	cd09725	CAS-I,CAS-II,CAS-III,CAS-V			
248 CAS-III-A | ADG82859.1																	
2078271..2080300	array		CP002028.1	Thermincola potens JR	Bacteria	Firmicutes	Clostridia	Clostridiales	Peptococcaceae	Thermincola		CRISPR array			28	37	36
2080473..2080809	-	ADG82859.1	CP002028.1	Thermincola potens JR	Bacteria	Firmicutes	Clostridia	Clostridiales	Peptococcaceae	Thermincola	CRISPR-associated protein Cas2	cas2	cd09725	CAS-I,CAS-II,CAS-III,CAS-V			
2080819..2081827	-	ADG82860.1	CP002028.1	Thermincola potens JR	Bacteria	Firmicutes	Clostridia	Clostridiales	Peptococcaceae	Thermincola	CRISPR-associated protein Cas1	cas1	cd09634	CAS-I,CAS-II,CAS-III,CAS-V			
2081823..2083206	-	ADG82861.1	CP002028.1	Thermincola potens JR	Bacteria	Firmicutes	Clostridia	Clostridiales	Peptococcaceae	Thermincola	CRISPR-associated protein Csm6	csm6	cd09746	CAS-III-A			
2083198..2083948	-	ADG82862.1	CP002028.1	Thermincola potens JR	Bacteria	Firmicutes	Clostridia	Clostridiales	Peptococcaceae	Thermincola	Protein of unknown function DUF2276	cas6	COG5551	CAS-III			
2083941..2085231	-	ADG82863.1	CP002028.1	Thermincola potens JR	Bacteria	Firmicutes	Clostridia	Clostridiales	Peptococcaceae	Thermincola	CRISPR-associated RAMP protein, Csm5 family	csm5gr7	COG1332	CAS-III-A			
2085227..2086208	-	ADG82864.1	CP002028.1	Thermincola potens JR	Bacteria	Firmicutes	Clostridia	Clostridiales	Peptococcaceae	Thermincola	CRISPR-associated RAMP protein, Csm4 family	csm4gr5	COG1567	CAS-III-A			
2086204..2086948	-	ADG82865.1	CP002028.1	Thermincola potens JR	Bacteria	Firmicutes	Clostridia	Clostridiales	Peptococcaceae	Thermincola	CRISPR-associated RAMP protein, Csm3 family	csm3gr7	cd09684	CAS-III-A,CAS-III-D			
2086952..2087330	-	ADG82866.1	CP002028.1	Thermincola potens JR	Bacteria	Firmicutes	Clostridia	Clostridiales	Peptococcaceae	Thermincola	CRISPR-associated protein, Csm2 family	csm2gr11	cd09647	CAS-III-A			
2087322..2090037	-	ADG82867.1	CP002028.1	Thermincola potens JR	Bacteria	Firmicutes	Clostridia	Clostridiales	Peptococcaceae	Thermincola	CRISPR-associated protein, Csm1 family	cas10	cd09680	CAS-III			
249 CAS-III-A | ABI69046.1																	
1994951..1996193	-	ABI69044.1	CP000448.1	Syntrophomonas wolfei subsp. wolfei str. Goettingen G311	Bacteria	Firmicutes	Clostridia	Clostridiales	Syntrophomonadaceae	Syntrophomonas	hypothetical protein	csm6	pfam09659	CAS-III-A			
1996473..1997694	-	ABI69045.1	CP000448.1	Syntrophomonas wolfei subsp. wolfei str. Goettingen G311	Bacteria	Firmicutes	Clostridia	Clostridiales	Syntrophomonadaceae	Syntrophomonas	hypothetical protein						
1997890..1998220	-	ABI69046.1	CP000448.1	Syntrophomonas wolfei subsp. wolfei str. Goettingen G311	Bacteria	Firmicutes	Clostridia	Clostridiales	Syntrophomonadaceae	Syntrophomonas	CRISPR-associated protein, Cas2 family	cas2	cd09725	CAS-I,CAS-II,CAS-III,CAS-V			
1998216..1999227	-	ABI69047.1	CP000448.1	Syntrophomonas wolfei subsp. wolfei str. Goettingen G311	Bacteria	Firmicutes	Clostridia	Clostridiales	Syntrophomonadaceae	Syntrophomonas	CRISPR-associated protein, Cas1 family	cas1	cd09634	CAS-I,CAS-II,CAS-III,CAS-V			
1999255..2000383	array		CP000448.1	Syntrophomonas wolfei subsp. wolfei str. Goettingen G311	Bacteria	Firmicutes	Clostridia	Clostridiales	Syntrophomonadaceae	Syntrophomonas		CRISPR array			16	36	36
2000509..2001787	+	ABI69048.1	CP000448.1	Syntrophomonas wolfei subsp. wolfei str. Goettingen G311	Bacteria	Firmicutes	Clostridia	Clostridiales	Syntrophomonadaceae	Syntrophomonas	transposase	c2c4*	c2c4				
2002263..2002514	array		CP000448.1	Syntrophomonas wolfei subsp. wolfei str. Goettingen G311	Bacteria	Firmicutes	Clostridia	Clostridiales	Syntrophomonadaceae	Syntrophomonas		CRISPR array			4	36	36
2002711..2003461	-	ABI69049.1	CP000448.1	Syntrophomonas wolfei subsp. wolfei str. Goettingen G311	Bacteria	Firmicutes	Clostridia	Clostridiales	Syntrophomonadaceae	Syntrophomonas	CRISPR-associated protein, Cas6 family	cas6	COG5551	CAS-III			
2003611..2004754	-	ABI69050.1	CP000448.1	Syntrophomonas wolfei subsp. wolfei str. Goettingen G311	Bacteria	Firmicutes	Clostridia	Clostridiales	Syntrophomonadaceae	Syntrophomonas	CRISPR-associated protein, Csm5 family	csm5gr7	COG1332	CAS-III-A			
2004750..2005698	-	ABI69051.1	CP000448.1	Syntrophomonas wolfei subsp. wolfei str. Goettingen G311	Bacteria	Firmicutes	Clostridia	Clostridiales	Syntrophomonadaceae	Syntrophomonas	CRISPR-associated protein, Csm4 family	csm4gr5	COG1567	CAS-III-A			
2005672..2006374	-	ABI69052.1	CP000448.1	Syntrophomonas wolfei subsp. wolfei str. Goettingen G311	Bacteria	Firmicutes	Clostridia	Clostridiales	Syntrophomonadaceae	Syntrophomonas	CRISPR-associated protein, Csm3 family	csm3gr7	cd09684	CAS-III-A,CAS-III-D			
2006387..2006864	-	ABI69053.1	CP000448.1	Syntrophomonas wolfei subsp. wolfei str. Goettingen G311	Bacteria	Firmicutes	Clostridia	Clostridiales	Syntrophomonadaceae	Syntrophomonas	hypothetical protein	csm2gr11	pfam03750	CAS-III-A			
2006856..2009283	-	ABI69054.1	CP000448.1	Syntrophomonas wolfei subsp. wolfei str. Goettingen G311	Bacteria	Firmicutes	Clostridia	Clostridiales	Syntrophomonadaceae	Syntrophomonas	CRISPR-associated protein, Csm1 family	cas10	cd09680	CAS-III			
251 CAS-III-A | EEF92712.1																	
43627..45062	array		ACCK01000438.1	Catenibacterium mitsuokai DSM 15897	Bacteria	Firmicutes	Erysipelotrichia	Erysipelotrichales	Erysipelotrichaceae	Catenibacterium		CRISPR array			20	38	35

43655..43808	+	EEF92709.1	ACCK01000438.1	Catenibacterium mitsuokai DSM 15897	Bacteria	Firmicutes	Erysipelotrichia	Erysipelotrichales	Erysipelotrichaceae	Catenibacterium	hypothetical protein						
44774..44993	+	EEF92710.1	ACCK01000438.1	Catenibacterium mitsuokai DSM 15897	Bacteria	Firmicutes	Erysipelotrichia	Erysipelotrichales	Erysipelotrichaceae	Catenibacterium	hypothetical protein						
45249..46587	-	EEF92711.1	ACCK01000438.1	Catenibacterium mitsuokai DSM 15897	Bacteria	Firmicutes	Erysipelotrichia	Erysipelotrichales	Erysipelotrichaceae	Catenibacterium	putative CRISPR-associated protein, Csm6 family	csm6	cd09746	CAS-III-A			
46588..46909	-	EEF92712.1	ACCK01000438.1	Catenibacterium mitsuokai DSM 15897	Bacteria	Firmicutes	Erysipelotrichia	Erysipelotrichales	Erysipelotrichaceae	Catenibacterium	CRISPR-associated protein Cas2	cas2	cd09725	CAS-I,CAS-II,CAS-III,CAS-V			
46914..47913	-	EEF92713.1	ACCK01000438.1	Catenibacterium mitsuokai DSM 15897	Bacteria	Firmicutes	Erysipelotrichia	Erysipelotrichales	Erysipelotrichaceae	Catenibacterium	CRISPR-associated endonuclease Cas1	cas1	cd09634	CAS-I,CAS-II,CAS-III,CAS-V			
47978..49121	-	EEF92714.1	ACCK01000438.1	Catenibacterium mitsuokai DSM 15897	Bacteria	Firmicutes	Erysipelotrichia	Erysipelotrichales	Erysipelotrichaceae	Catenibacterium	CRISPR-associated RAMP protein, Csm5 family	csm5gr7	cd09662	CAS-III-A			
49117..50035	-	EEF92715.1	ACCK01000438.1	Catenibacterium mitsuokai DSM 15897	Bacteria	Firmicutes	Erysipelotrichia	Erysipelotrichales	Erysipelotrichaceae	Catenibacterium	CRISPR-associated RAMP protein, Csm4 family	csm4gr5	COG1567	CAS-III-A			
50053..50719	-	EEF92716.1	ACCK01000438.1	Catenibacterium mitsuokai DSM 15897	Bacteria	Firmicutes	Erysipelotrichia	Erysipelotrichales	Erysipelotrichaceae	Catenibacterium	CRISPR-associated RAMP protein, Csm3 family	csm3gr7	cd09684	CAS-III-A,CAS-III-D			
50723..51101	-	EEF92717.1	ACCK01000438.1	Catenibacterium mitsuokai DSM 15897	Bacteria	Firmicutes	Erysipelotrichia	Erysipelotrichales	Erysipelotrichaceae	Catenibacterium	CRISPR-associated protein, Csm2 family	csm2gr11	cd09647	CAS-III-A			
51104..53453	-	EEF92718.1	ACCK01000438.1	Catenibacterium mitsuokai DSM 15897	Bacteria	Firmicutes	Erysipelotrichia	Erysipelotrichales	Erysipelotrichaceae	Catenibacterium	CRISPR-associated protein, Csm1 family	cas10	cd09680	CAS-III			
53449..54193	-	EEF92719.1	ACCK01000438.1	Catenibacterium mitsuokai DSM 15897	Bacteria	Firmicutes	Erysipelotrichia	Erysipelotrichales	Erysipelotrichaceae	Catenibacterium	CRISPR-associated endoribonuclease Cas6	cas6	pfam10040	CAS-III,CAS-IV			
54581..55286	array		ACCK01000438.1	Catenibacterium mitsuokai DSM 15897	Bacteria	Firmicutes	Erysipelotrichia	Erysipelotrichales	Erysipelotrichaceae	Catenibacterium		CRISPR array			10	39	35
252 CAS-III-A | EFV01913.1																	
1259337..1260747	-	EFV01912.1	GL622359.1	Pseudoramibacter alactolyticus ATCC 23263	Bacteria	Firmicutes	Clostridia	Clostridiales	Eubacteriaceae	Pseudoramibacter	putative CRISPR-associated protein, Csm6 family	csm6	cd09746	CAS-III-A			
1260767..1261091	-	EFV01913.1	GL622359.1	Pseudoramibacter alactolyticus ATCC 23263	Bacteria	Firmicutes	Clostridia	Clostridiales	Eubacteriaceae	Pseudoramibacter	CRISPR-associated protein Cas2	cas2	cd09725	CAS-I,CAS-II,CAS-III,CAS-V			
1261095..1262094	-	EFV01914.1	GL622359.1	Pseudoramibacter alactolyticus ATCC 23263	Bacteria	Firmicutes	Clostridia	Clostridiales	Eubacteriaceae	Pseudoramibacter	CRISPR-associated endonuclease Cas1	cas1	cd09634	CAS-I,CAS-II,CAS-III,CAS-V			
1262135..1262454	array		GL622359.1	Pseudoramibacter alactolyticus ATCC 23263	Bacteria	Firmicutes	Clostridia	Clostridiales	Eubacteriaceae	Pseudoramibacter		CRISPR array			5	35	36
1262300..1262468	+	EFV01915.1	GL622359.1	Pseudoramibacter alactolyticus ATCC 23263	Bacteria	Firmicutes	Clostridia	Clostridiales	Eubacteriaceae	Pseudoramibacter	hypothetical protein						
1262448..1262628	-	EFV01916.1	GL622359.1	Pseudoramibacter alactolyticus ATCC 23263	Bacteria	Firmicutes	Clostridia	Clostridiales	Eubacteriaceae	Pseudoramibacter	hypothetical protein						
1262650..1262830	-	EFV01917.1	GL622359.1	Pseudoramibacter alactolyticus ATCC 23263	Bacteria	Firmicutes	Clostridia	Clostridiales	Eubacteriaceae	Pseudoramibacter	hypothetical protein						
1262826..1262946	-	EFV01918.1	GL622359.1	Pseudoramibacter alactolyticus ATCC 23263	Bacteria	Firmicutes	Clostridia	Clostridiales	Eubacteriaceae	Pseudoramibacter	hypothetical protein						
1263015..1263775	array		GL622359.1	Pseudoramibacter alactolyticus ATCC 23263	Bacteria	Firmicutes	Clostridia	Clostridiales	Eubacteriaceae	Pseudoramibacter		CRISPR array			11	36	36
1263769..1263943	-	EFV01919.1	GL622359.1	Pseudoramibacter alactolyticus ATCC 23263	Bacteria	Firmicutes	Clostridia	Clostridiales	Eubacteriaceae	Pseudoramibacter	hypothetical protein						
1263908..1265000	-	EFV01920.1	GL622359.1	Pseudoramibacter alactolyticus ATCC 23263	Bacteria	Firmicutes	Clostridia	Clostridiales	Eubacteriaceae	Pseudoramibacter	CRISPR-associated RAMP protein, Csm5 family	csm5gr7	cd09662	CAS-III-A			
1264996..1265941	-	EFV01921.1	GL622359.1	Pseudoramibacter alactolyticus ATCC 23263	Bacteria	Firmicutes	Clostridia	Clostridiales	Eubacteriaceae	Pseudoramibacter	CRISPR-associated RAMP protein, Csm4 family	csm4gr5	COG1567	CAS-III-A			
1265937..1266639	-	EFV01922.1	GL622359.1	Pseudoramibacter alactolyticus ATCC 23263	Bacteria	Firmicutes	Clostridia	Clostridiales	Eubacteriaceae	Pseudoramibacter	CRISPR-associated RAMP protein, Csm3 family	csm3gr7	cd09684	CAS-III-A,CAS-III-D			
1266644..1267043	-	EFV01923.1	GL622359.1	Pseudoramibacter alactolyticus ATCC 23263	Bacteria	Firmicutes	Clostridia	Clostridiales	Eubacteriaceae	Pseudoramibacter	CRISPR-associated protein, Csm2 family	csm2gr11	cd09647	CAS-III-A			
1267029..1269441	-	EFV01924.1	GL622359.1	Pseudoramibacter alactolyticus ATCC 23263	Bacteria	Firmicutes	Clostridia	Clostridiales	Eubacteriaceae	Pseudoramibacter	CRISPR-associated protein, Csm1 family	cas10	cd09680	CAS-III			
1269430..1270195	-	EFV01925.1	GL622359.1	Pseudoramibacter alactolyticus ATCC 23263	Bacteria	Firmicutes	Clostridia	Clostridiales	Eubacteriaceae	Pseudoramibacter	putative CRISPR-associated endoribonuclease Cas6	cas6	COG5551	CAS-III			
253 CAS-III-A | SER78324.1																	
25105..25840	+	SER78179.1	FOGW01000010.1	Lachnobacterium bovis	Bacteria	Firmicutes	Clostridia	Clostridiales	Lachnospiraceae	Lachnobacterium	CRISPR-associated endoribonuclease Cas6	cas6	COG5551	CAS-III			
25843..28270	+	SER78199.1	FOGW01000010.1	Lachnobacterium bovis	Bacteria	Firmicutes	Clostridia	Clostridiales	Lachnospiraceae	Lachnobacterium	CRISPR-associated protein Csm1	cas10	cd09680	CAS-III			
28277..28742	+	SER78218.1	FOGW01000010.1	Lachnobacterium bovis	Bacteria	Firmicutes	Clostridia	Clostridiales	Lachnospiraceae	Lachnobacterium	CRISPR-associated protein Csm2	csm2gr11	pfam03750	CAS-III-A			
28761..29442	+	SER78242.1	FOGW01000010.1	Lachnobacterium bovis	Bacteria	Firmicutes	Clostridia	Clostridiales	Lachnospiraceae	Lachnobacterium	CRISPR-associated protein Csm3	csm3gr7	cd09684	CAS-III-A,CAS-III-D			
29444..30386	+	SER78262.1	FOGW01000010.1	Lachnobacterium bovis	Bacteria	Firmicutes	Clostridia	Clostridiales	Lachnospiraceae	Lachnobacterium	CRISPR-associated protein Csm4	csm4gr5	COG1567	CAS-III-A			
30385..31525	+	SER78280.1	FOGW01000010.1	Lachnobacterium bovis	Bacteria	Firmicutes	Clostridia	Clostridiales	Lachnospiraceae	Lachnobacterium	CRISPR-associated protein Csm5	csm5gr7	cd09662	CAS-III-A			
31550..32552	+	SER78299.1	FOGW01000010.1	Lachnobacterium bovis	Bacteria	Firmicutes	Clostridia	Clostridiales	Lachnospiraceae	Lachnobacterium	CRISP-associated protein Cas1	cas1	cd09634	CAS-I,CAS-II,CAS-III,CAS-V			
32563..32881	+	SER78324.1	FOGW01000010.1	Lachnobacterium bovis	Bacteria	Firmicutes	Clostridia	Clostridiales	Lachnospiraceae	Lachnobacterium	CRISPR-associated protein Cas2	cas2	cd09725	CAS-I,CAS-II,CAS-III,CAS-V			
32903..34253	+	SER78348.1	FOGW01000010.1	Lachnobacterium bovis	Bacteria	Firmicutes	Clostridia	Clostridiales	Lachnospiraceae	Lachnobacterium	CRISPR type III-A/MTUBE-associated protein Csm6	csm6	pfam09659	CAS-III-A			
36500..37361	+	SER78366.1	FOGW01000010.1	Lachnobacterium bovis	Bacteria	Firmicutes	Clostridia	Clostridiales	Lachnospiraceae	Lachnobacterium	membrane protein						
37487..38807	+	SER78388.1	FOGW01000010.1	Lachnobacterium bovis	Bacteria	Firmicutes	Clostridia	Clostridiales	Lachnospiraceae	Lachnobacterium	hypothetical protein						
39030..39495	+	SER78412.1	FOGW01000010.1	Lachnobacterium bovis	Bacteria	Firmicutes	Clostridia	Clostridiales	Lachnospiraceae	Lachnobacterium	hypothetical protein						
39565..40570	+	SER78435.1	FOGW01000010.1	Lachnobacterium bovis	Bacteria	Firmicutes	Clostridia	Clostridiales	Lachnospiraceae	Lachnobacterium	Predicted DNA-binding transcriptional regulator YafY, contains an HTH and WYL domains	WYL	pfam13280	CAS-I,CAS-III			
254 CAS-III-A | CUO70252.1																	
15999..17328	+	CUO69714.1	CYZL01000021.1	Anaerobutyricum hallii	Bacteria	Firmicutes	Clostridia	Clostridiales	Lachnospiraceae	Anaerobutyricum	CRISPR type III-A/MTUBE-associated protein Csm6	csm6	cd09746	CAS-III-A			
17329..18085	+	CUO69760.1	CYZL01000021.1	Anaerobutyricum hallii	Bacteria	Firmicutes	Clostridia	Clostridiales	Lachnospiraceae	Anaerobutyricum	Uncharacterized protein predicted to be involved in DNA repair (RAMP superfamily)	cas6	pfam10040	CAS-III,CAS-IV			
18074..20447	+	CUO69866.1	CYZL01000021.1	Anaerobutyricum hallii	Bacteria	Firmicutes	Clostridia	Clostridiales	Lachnospiraceae	Anaerobutyricum	CRISPR-associated protein Cas10/Csm1%2C subtype III-A/MTUBE	cas10	cd09680	CAS-III			
20430..20820	+	CUO69962.1	CYZL01000021.1	Anaerobutyricum hallii	Bacteria	Firmicutes	Clostridia	Clostridiales	Lachnospiraceae	Anaerobutyricum	CRISPR type III-A/MTUBE-associated protein Csm2	csm2gr11	cd09647	CAS-III-A			
20829..21492	+	CUO70065.1	CYZL01000021.1	Anaerobutyricum hallii	Bacteria	Firmicutes	Clostridia	Clostridiales	Lachnospiraceae	Anaerobutyricum	CRISPR type III-A/MTUBE-associated RAMP protein Csm3	csm3gr7	cd09684	CAS-III-A,CAS-III-D			
21495..23529	+	CUO70173.1	CYZL01000021.1	Anaerobutyricum hallii	Bacteria	Firmicutes	Clostridia	Clostridiales	Lachnospiraceae	Anaerobutyricum	CRISPR type III-A/MTUBE-associated RAMP protein Csm4	csm4gr5,csm5gr7	cd09663,cd09662	CAS-III-A			
23691..24312	array		CYZL01000021.1	Anaerobutyricum hallii	Bacteria	Firmicutes	Clostridia	Clostridiales	Lachnospiraceae	Anaerobutyricum		CRISPR array			9	37	36
24346..25348	+	CUO70210.1	CYZL01000021.1	Anaerobutyricum hallii	Bacteria	Firmicutes	Clostridia	Clostridiales	Lachnospiraceae	Anaerobutyricum	CRISPR-associated endonuclease Cas1	cas1	cd09634	CAS-I,CAS-II,CAS-III,CAS-V			
25325..25682	+	CUO70252.1	CYZL01000021.1	Anaerobutyricum hallii	Bacteria	Firmicutes	Clostridia	Clostridiales	Lachnospiraceae	Anaerobutyricum	CRISPR-associated endoribonuclease Cas2	cas2	cd09725	CAS-I,CAS-II,CAS-III,CAS-V			
256 partial | CCX82922.1																	
334..1336	+	CCX82921.1	HF987621.1	Eubacterium sp. CAG:86	Bacteria	Firmicutes	Clostridia	Clostridiales	Eubacteriaceae	Eubacterium	cRISPR-associated endonuclease Cas1	cas1	cd09634	CAS-I,CAS-II,CAS-III,CAS-V			
1344..1689	+	CCX82922.1	HF987621.1	Eubacterium sp. CAG:86	Bacteria	Firmicutes	Clostridia	Clostridiales	Eubacteriaceae	Eubacterium	cRISPR-associated endoribonuclease Cas2 2	cas2	pfam09827	CAS-I,CAS-II,CAS-III			
257 CAS-III-A | OKZ95403.1																	
1471..2209	+	OKZ95396.1	MNSH01000002.1	Eubacterium sp. 36_13	Bacteria	Firmicutes	Clostridia	Clostridiales	Eubacteriaceae	Eubacterium	CRISPR-associated endoribonuclease Cas6	cas6	COG5551	CAS-III			
2215..4684	+	OKZ95397.1	MNSH01000002.1	Eubacterium sp. 36_13	Bacteria	Firmicutes	Clostridia	Clostridiales	Eubacteriaceae	Eubacterium	type III-A CRISPR-associated protein Cas10/Csm1	cas10	cd09680	CAS-III			
4667..5105	+	OKZ95398.1	MNSH01000002.1	Eubacterium sp. 36_13	Bacteria	Firmicutes	Clostridia	Clostridiales	Eubacteriaceae	Eubacterium	type III-A CRISPR-associated protein Csm2	csm2gr11	cd09647	CAS-III-A			
5117..5792	+	OKZ95399.1	MNSH01000002.1	Eubacterium sp. 36_13	Bacteria	Firmicutes	Clostridia	Clostridiales	Eubacteriaceae	Eubacterium	type III-A CRISPR-associated RAMP protein Csm3	csm3gr7	cd09684	CAS-III-A,CAS-III-D			
5802..6726	+	OKZ95400.1	MNSH01000002.1	Eubacterium sp. 36_13	Bacteria	Firmicutes	Clostridia	Clostridiales	Eubacteriaceae	Eubacterium	type III-A CRISPR-associated RAMP protein Csm4	csm4gr5	COG1567	CAS-III-A			
6722..7850	+	OKZ95401.1	MNSH01000002.1	Eubacterium sp. 36_13	Bacteria	Firmicutes	Clostridia	Clostridiales	Eubacteriaceae	Eubacterium	type III-A CRISPR-associated RAMP protein Csm5	csm5gr7,csm5gr7	cd09662,cd09662	CAS-III-A			
8065..9310	array		MNSH01000002.1	Eubacterium sp. 36_13	Bacteria	Firmicutes	Clostridia	Clostridiales	Eubacteriaceae	Eubacterium		CRISPR array			18	36	35
9403..10405	+	OKZ95402.1	MNSH01000002.1	Eubacterium sp. 36_13	Bacteria	Firmicutes	Clostridia	Clostridiales	Eubacteriaceae	Eubacterium	CRISPR-associated endonuclease Cas1	cas1	cd09634	CAS-I,CAS-II,CAS-III,CAS-V			
10413..10707	+	OKZ95403.1	MNSH01000002.1	Eubacterium sp. 36_13	Bacteria	Firmicutes	Clostridia	Clostridiales	Eubacteriaceae	Eubacterium	CRISPR-associated endonuclease Cas2	cas2	cd09725	CAS-I,CAS-II,CAS-III,CAS-V			
258 CAS-III-A | CCX73453.1																	
54366..55686	-	CCX73451.1	HF987137.1	Dorea sp. CAG:105	Bacteria	Firmicutes	Clostridia	Clostridiales	Lachnospiraceae	Dorea	putative uncharacterized protein	csm6	cd09746	CAS-III-A			
55700..56021	-	CCX73453.1	HF987137.1	Dorea sp. CAG:105	Bacteria	Firmicutes	Clostridia	Clostridiales	Lachnospiraceae	Dorea	cRISPR-associated endoribonuclease Cas2 2	cas2	cd09725	CAS-I,CAS-II,CAS-III,CAS-V			
56032..57034	-	CCX73455.1	HF987137.1	Dorea sp. CAG:105	Bacteria	Firmicutes	Clostridia	Clostridiales	Lachnospiraceae	Dorea	cRISPR-associated endonuclease Cas1	cas1	cd09634	CAS-I,CAS-II,CAS-III,CAS-V			
57361..57571	-	CCX73456.1	HF987137.1	Dorea sp. CAG:105	Bacteria	Firmicutes	Clostridia	Clostridiales	Lachnospiraceae	Dorea	putative uncharacterized protein						
58069..59339	array		HF987137.1	Dorea sp. CAG:105	Bacteria	Firmicutes	Clostridia	Clostridiales	Lachnospiraceae	Dorea		CRISPR array			18	37	35
58074..58599	array		HF987137.1	Dorea sp. CAG:105	Bacteria	Firmicutes	Clostridia	Clostridiales	Lachnospiraceae	Dorea		CRISPR array			8	42	29
59474..60596	-	CCX73457.1	HF987137.1	Dorea sp. CAG:105	Bacteria	Firmicutes	Clostridia	Clostridiales	Lachnospiraceae	Dorea	cRISPR-associated protein	csm5gr7,csm5gr7	cd09662,cd09662	CAS-III-A			
60592..61513	-	CCX73458.1	HF987137.1	Dorea sp. CAG:105	Bacteria	Firmicutes	Clostridia	Clostridiales	Lachnospiraceae	Dorea	cRISPR-associated protein	csm4gr5	COG1567	CAS-III-A			
61534..62200	-	CCX73459.1	HF987137.1	Dorea sp. CAG:105	Bacteria	Firmicutes	Clostridia	Clostridiales	Lachnospiraceae	Dorea	putative uncharacterized protein	csm3gr7	cd09684	CAS-III-A,CAS-III-D			
62221..62623	-	CCX73460.1	HF987137.1	Dorea sp. CAG:105	Bacteria	Firmicutes	Clostridia	Clostridiales	Lachnospiraceae	Dorea	cRISPR-associated protein Csm2 family	csm2gr11	cd09647	CAS-III-A			
62626..64975	-	CCX73461.1	HF987137.1	Dorea sp. CAG:105	Bacteria	Firmicutes	Clostridia	Clostridiales	Lachnospiraceae	Dorea	putative uncharacterized protein	cas10	cd09680	CAS-III			
64985..65711	-	CCX73462.1	HF987137.1	Dorea sp. CAG:105	Bacteria	Firmicutes	Clostridia	Clostridiales	Lachnospiraceae	Dorea	putative uncharacterized protein	cas6	COG5551	CAS-III			
259 CAS-III-A | SCI59180.1																	
132400..133138	+	SCI58937.1	FMFY01000003.1	uncultured Ruminococcus sp.	Bacteria	Firmicutes	Clostridia	Clostridiales	Ruminococcaceae	Ruminococcus	CRISPR-associated endoribonuclease Cas6	cas6	COG5551	CAS-III			
133130..135467	+	SCI58967.1	FMFY01000003.1	uncultured Ruminococcus sp.	Bacteria	Firmicutes	Clostridia	Clostridiales	Ruminococcaceae	Ruminococcus	CRISPR-associated protein Cas10/Csm1%2C subtype III-A/MTUBE	cas10	cd09680	CAS-III			
135468..135870	+	SCI58998.1	FMFY01000003.1	uncultured Ruminococcus sp.	Bacteria	Firmicutes	Clostridia	Clostridiales	Ruminococcaceae	Ruminococcus	CRISPR type III-A/MTUBE-associated protein Csm2	csm2gr11	cd09647	CAS-III-A			
135882..136548	+	SCI59029.1	FMFY01000003.1	uncultured Ruminococcus sp.	Bacteria	Firmicutes	Clostridia	Clostridiales	Ruminococcaceae	Ruminococcus	CRISPR type III-A/MTUBE-associated RAMP protein Csm3	csm3gr7	cd09684	CAS-III-A,CAS-III-D			
136567..137494	+	SCI59059.1	FMFY01000003.1	uncultured Ruminococcus sp.	Bacteria	Firmicutes	Clostridia	Clostridiales	Ruminococcaceae	Ruminococcus	CRISPR type III-A/MTUBE-associated RAMP protein Csm4	csm4gr5	COG1567	CAS-III-A			
137490..138588	+	SCI59089.1	FMFY01000003.1	uncultured Ruminococcus sp.	Bacteria	Firmicutes	Clostridia	Clostridiales	Ruminococcaceae	Ruminococcus	CRISPR type III-A/MTUBE-associated RAMP protein Csm5	csm5gr7	cd09662	CAS-III-A			
138723..140119	array		FMFY01000003.1	uncultured Ruminococcus sp.	Bacteria	Firmicutes	Clostridia	Clostridiales	Ruminococcaceae	Ruminococcus		CRISPR array			20	35	36
138763..139150	+	SCI59118.1	FMFY01000003.1	uncultured Ruminococcus sp.	Bacteria	Firmicutes	Clostridia	Clostridiales	Ruminococcaceae	Ruminococcus	Uncharacterised protein						
140136..141138	+	SCI59147.1	FMFY01000003.1	uncultured Ruminococcus sp.	Bacteria	Firmicutes	Clostridia	Clostridiales	Ruminococcaceae	Ruminococcus	CRISPR-associated endonuclease Cas1	cas1	cd09634	CAS-I,CAS-II,CAS-III,CAS-V			
141115..141454	+	SCI59180.1	FMFY01000003.1	uncultured Ruminococcus sp.	Bacteria	Firmicutes	Clostridia	Clostridiales	Ruminococcaceae	Ruminococcus	CRISPR-associated endoribonuclease Cas2	cas2	cd09725	CAS-I,CAS-II,CAS-III,CAS-V			
141466..142789	+	SCI59210.1	FMFY01000003.1	uncultured Ruminococcus sp.	Bacteria	Firmicutes	Clostridia	Clostridiales	Ruminococcaceae	Ruminococcus	CRISPR type III-A/MTUBE-associated protein Csm6	csm6	pfam09659	CAS-III-A			
260 partial CAS-III-A | RGB74595.1																	
14899..16282	-	RGB74593.1	QVEP01000053.1	Coprococcus catus	Bacteria	Firmicutes	Clostridia	Clostridiales	Lachnospiraceae	Coprococcus	CRISPR-associated protein Csm6	csm6	pfam09659	CAS-III-A			
16343..16685	-	RGB74595.1	QVEP01000053.1	Coprococcus catus	Bacteria	Firmicutes	Clostridia	Clostridiales	Lachnospiraceae	Coprococcus	CRISPR-associated endonuclease Cas2	cas2	cd09725	CAS-I,CAS-II,CAS-III,CAS-V			
16731..17733	-	RGB74594.1	QVEP01000053.1	Coprococcus catus	Bacteria	Firmicutes	Clostridia	Clostridiales	Lachnospiraceae	Coprococcus	CRISPR-associated endonuclease Cas1	cas1	cd09634	CAS-I,CAS-II,CAS-III,CAS-V			
17771..18023	array		QVEP01000053.1	Coprococcus catus	Bacteria	Firmicutes	Clostridia	Clostridiales	Lachnospiraceae	Coprococcus		CRISPR array			4	39	34
261 CAS-III-A | RGC44690.1																	
12824..13562	+	RGC44622.1	QVFD01000014.1	Coprococcus catus	Bacteria	Firmicutes	Clostridia	Clostridiales	Lachnospiraceae	Coprococcus	CRISPR-associated endoribonuclease Cas6	cas6	COG5551	CAS-III			
13558..15919	+	RGC44623.1	QVFD01000014.1	Coprococcus catus	Bacteria	Firmicutes	Clostridia	Clostridiales	Lachnospiraceae	Coprococcus	type III-A CRISPR-associated protein Cas10/Csm1	cas10	cd09680	CAS-III			
15935..16346	+	RGC44624.1	QVFD01000014.1	Coprococcus catus	Bacteria	Firmicutes	Clostridia	Clostridiales	Lachnospiraceae	Coprococcus	type III-A CRISPR-associated protein Csm2	csm2gr11	cd09647	CAS-III-A			
16363..17029	+	RGC44625.1	QVFD01000014.1	Coprococcus catus	Bacteria	Firmicutes	Clostridia	Clostridiales	Lachnospiraceae	Coprococcus	type III-A CRISPR-associated RAMP protein Csm3	csm3gr7	cd09684	CAS-III-A,CAS-III-D			
17046..18012	+	RGC44626.1	QVFD01000014.1	Coprococcus catus	Bacteria	Firmicutes	Clostridia	Clostridiales	Lachnospiraceae	Coprococcus	type III-A CRISPR-associated RAMP protein Csm4	csm4gr5	COG1567	CAS-III-A			
18008..19130	+	RGC44627.1	QVFD01000014.1	Coprococcus catus	Bacteria	Firmicutes	Clostridia	Clostridiales	Lachnospiraceae	Coprococcus	type III-A CRISPR-associated RAMP protein Csm5	csm5gr7	COG1332	CAS-III-A			
19425..20978	array		QVFD01000014.1	Coprococcus catus	Bacteria	Firmicutes	Clostridia	Clostridiales	Lachnospiraceae	Coprococcus		CRISPR array			22	36	36
21013..22015	+	RGC44628.1	QVFD01000014.1	Coprococcus catus	Bacteria	Firmicutes	Clostridia	Clostridiales	Lachnospiraceae	Coprococcus	CRISPR-associated endonuclease Cas1	cas1	cd09634	CAS-I,CAS-II,CAS-III,CAS-V			
22061..22403	+	RGC44690.1	QVFD01000014.1	Coprococcus catus	Bacteria	Firmicutes	Clostridia	Clostridiales	Lachnospiraceae	Coprococcus	CRISPR-associated endonuclease Cas2	cas2	cd09725	CAS-I,CAS-II,CAS-III,CAS-V			
22464..23838	+	RGC44629.1	QVFD01000014.1	Coprococcus catus	Bacteria	Firmicutes	Clostridia	Clostridiales	Lachnospiraceae	Coprococcus	CRISPR-associated protein Csm6	csm6	pfam09659	CAS-III-A			
263 CAS-III-A | SCG89712.1																	
45644..46964	-	SCG89711.1	FMDP01000019.1	uncultured Clostridium sp.	Bacteria	Firmicutes	Clostridia	Clostridiales	Clostridiaceae	Clostridium	CRISPR type III-A/MTUBE-associated protein Csm6	csm6	cd09746	CAS-III-A			
46975..47317	-	SCG89712.1	FMDP01000019.1	uncultured Clostridium sp.	Bacteria	Firmicutes	Clostridia	Clostridiales	Clostridiaceae	Clostridium	CRISPR-associated endoribonuclease Cas2	cas2	pfam09827	CAS-I,CAS-II,CAS-III			
47294..48296	-	SCG89713.1	FMDP01000019.1	uncultured Clostridium sp.	Bacteria	Firmicutes	Clostridia	Clostridiales	Clostridiaceae	Clostridium	CRISPR-associated endonuclease Cas1	cas1	cd09634	CAS-I,CAS-II,CAS-III,CAS-V			
48463..49814	array		FMDP01000019.1	uncultured Clostridium sp.	Bacteria	Firmicutes	Clostridia	Clostridiales	Clostridiaceae	Clostridium		CRISPR array			19	37	36
49948..51037	-	SCG89714.1	FMDP01000019.1	uncultured Clostridium sp.	Bacteria	Firmicutes	Clostridia	Clostridiales	Clostridiaceae	Clostridium	CRISPR type III-A/MTUBE-associated RAMP protein Csm5	csm5gr7	cd09662	CAS-III-A			
51033..51960	-	SCG89715.1	FMDP01000019.1	uncultured Clostridium sp.	Bacteria	Firmicutes	Clostridia	Clostridiales	Clostridiaceae	Clostridium	CRISPR type III-A/MTUBE-associated RAMP protein Csm4	csm4gr5	COG1567	CAS-III-A			
51971..52643	-	SCG89716.1	FMDP01000019.1	uncultured Clostridium sp.	Bacteria	Firmicutes	Clostridia	Clostridiales	Clostridiaceae	Clostridium	CRISPR type III-A/MTUBE-associated RAMP protein Csm3	csm3gr7	cd09684	CAS-III-A,CAS-III-D			
52657..53053	-	SCG89717.1	FMDP01000019.1	uncultured Clostridium sp.	Bacteria	Firmicutes	Clostridia	Clostridiales	Clostridiaceae	Clostridium	CRISPR type III-A/MTUBE-associated protein Csm2	csm2gr11	cd09647	CAS-III-A			
53054..55397	-	SCG89718.1	FMDP01000019.1	uncultured Clostridium sp.	Bacteria	Firmicutes	Clostridia	Clostridiales	Clostridiaceae	Clostridium	Predicted HD superfamily hydrolase	cas10	cd09680	CAS-III			
55371..56130	-	SCG89719.1	FMDP01000019.1	uncultured Clostridium sp.	Bacteria	Firmicutes	Clostridia	Clostridiales	Clostridiaceae	Clostridium	CRISPR-associated endoribonuclease Cas6	cas6	COG5551	CAS-III			
264 CAS-III-A | EQM98871.1																	
2298275..2299592	-	EES78308.1	ACII02000003.1	Ruminococcus sp. 5_1_39BFAA	Bacteria	Firmicutes	Clostridia	Clostridiales	Ruminococcaceae	Ruminococcus	CRISPR type III-a/mtube-associated protein csm6	csm6	cd09746	CAS-III-A			
2299625..2299961	-	EQM98871.1	ACII02000003.1	Ruminococcus sp. 5_1_39BFAA	Bacteria	Firmicutes	Clostridia	Clostridiales	Ruminococcaceae	Ruminococcus	CRISPR-associated endoribonuclease cas2	cas2	cd09725	CAS-I,CAS-II,CAS-III,CAS-V			
2299938..2300940	-	EES78311.1	ACII02000003.1	Ruminococcus sp. 5_1_39BFAA	Bacteria	Firmicutes	Clostridia	Clostridiales	Ruminococcaceae	Ruminococcus	CRISPR-associated endonuclease cas1	cas1	cd09634	CAS-I,CAS-II,CAS-III,CAS-V			
2301013..2302414	array		ACII02000003.1	Ruminococcus sp. 5_1_39BFAA	Bacteria	Firmicutes	Clostridia	Clostridiales	Ruminococcaceae	Ruminococcus		CRISPR array			20	35	36
2302581..2303676	-	EES78312.2	ACII02000003.1	Ruminococcus sp. 5_1_39BFAA	Bacteria	Firmicutes	Clostridia	Clostridiales	Ruminococcaceae	Ruminococcus	CRISPR type III-a/mtube-associated ramp protein csm5	csm5gr7	COG1332	CAS-III-A			
2303677..2304598	-	EES78313.1	ACII02000003.1	Ruminococcus sp. 5_1_39BFAA	Bacteria	Firmicutes	Clostridia	Clostridiales	Ruminococcaceae	Ruminococcus	CRISPR type III-a/mtube-associated ramp protein csm4	csm4gr5	COG1567	CAS-III-A			
2304618..2305284	-	EES78314.1	ACII02000003.1	Ruminococcus sp. 5_1_39BFAA	Bacteria	Firmicutes	Clostridia	Clostridiales	Ruminococcaceae	Ruminococcus	CRISPR type III-a/mtube-associated ramp protein csm3	csm3gr7	cd09684	CAS-III-A,CAS-III-D			
2305296..2305692	-	EES78315.1	ACII02000003.1	Ruminococcus sp. 5_1_39BFAA	Bacteria	Firmicutes	Clostridia	Clostridiales	Ruminococcaceae	Ruminococcus	CRISPR type III-a/mtube-associated protein csm2	csm2gr11	cd09647	CAS-III-A			
2305681..2308066	-	EES78316.2	ACII02000003.1	Ruminococcus sp. 5_1_39BFAA	Bacteria	Firmicutes	Clostridia	Clostridiales	Ruminococcaceae	Ruminococcus	CRISPR-associated protein cas10/csm1, subtype III-a/mtube	cas10	cd09680	CAS-III			
2308058..2308799	-	EES78317.1	ACII02000003.1	Ruminococcus sp. 5_1_39BFAA	Bacteria	Firmicutes	Clostridia	Clostridiales	Ruminococcaceae	Ruminococcus	CRISPR-associated endoribonuclease cas6	cas6	pfam10040	CAS-III,CAS-IV			
265 partial CAS-III-A/CAS-I | ERI97393.1																	
1..598	array		KE993343.1	Blautia sp. KLE 1732	Bacteria	Firmicutes	Clostridia	Clostridiales	Lachnospiraceae	Blautia		CRISPR array			9	42	29
463..640	+	ERI97391.1	KE993343.1	Blautia sp. KLE 1732	Bacteria	Firmicutes	Clostridia	Clostridiales	Lachnospiraceae	Blautia	hypothetical protein						
651..1668	+	ERI97392.1	KE993343.1	Blautia sp. KLE 1732	Bacteria	Firmicutes	Clostridia	Clostridiales	Lachnospiraceae	Blautia	CRISPR-associated endonuclease Cas1	cas1	cd09634	CAS-I,CAS-II,CAS-III,CAS-V			
1645..1981	+	ERI97393.1	KE993343.1	Blautia sp. KLE 1732	Bacteria	Firmicutes	Clostridia	Clostridiales	Lachnospiraceae	Blautia	CRISPR-associated protein Cas2	cas2	pfam09827	CAS-I,CAS-II,CAS-III			
2017..3331	+	ERI97394.1	KE993343.1	Blautia sp. KLE 1732	Bacteria	Firmicutes	Clostridia	Clostridiales	Lachnospiraceae	Blautia	CRISPR-associated protein, Csm6 family	csm6	cd09746	CAS-III-A			
266 CAS-III-A | KQC84609.1																	
193480..194821	-	KQC84608.1	LLKB01000005.1	Lachnospiraceae bacterium TF01-11	Bacteria	Firmicutes	Clostridia	Clostridiales	Lachnospiraceae		hypothetical protein	csm6	pfam09659	CAS-III-A			

194838..195168	-	KQC84609.1	LLKB01000005.1	Lachnospiraceae bacterium TF01-11	Bacteria	Firmicutes	Clostridia	Clostridiales	Lachnospiraceae		hypothetical protein	cas2	cd09725	CAS-I,CAS-II,CAS-III,CAS-V			
195235..196246	-	KQC84610.1	LLKB01000005.1	Lachnospiraceae bacterium TF01-11	Bacteria	Firmicutes	Clostridia	Clostridiales	Lachnospiraceae		CRISPR-associated protein Cas1	cas1	cd09634	CAS-I,CAS-II,CAS-III,CAS-V			
197362..198972	array		LLKB01000005.1	Lachnospiraceae bacterium TF01-11	Bacteria	Firmicutes	Clostridia	Clostridiales	Lachnospiraceae			CRISPR array			23	35	36
199107..200217	-	KQC84611.1	LLKB01000005.1	Lachnospiraceae bacterium TF01-11	Bacteria	Firmicutes	Clostridia	Clostridiales	Lachnospiraceae		hypothetical protein	csm5gr7,csm5gr7	cd09662,cd09662	CAS-III-A			
200220..201144	-	KQC84612.1	LLKB01000005.1	Lachnospiraceae bacterium TF01-11	Bacteria	Firmicutes	Clostridia	Clostridiales	Lachnospiraceae		hypothetical protein	csm4gr5	COG1567	CAS-III-A			
201158..201815	-	KQC84613.1	LLKB01000005.1	Lachnospiraceae bacterium TF01-11	Bacteria	Firmicutes	Clostridia	Clostridiales	Lachnospiraceae		type III-A CRISPR-associated RAMP protein Csm3	csm3gr7	cd09684	CAS-III-A,CAS-III-D			
201851..202199	-	KQC85580.1	LLKB01000005.1	Lachnospiraceae bacterium TF01-11	Bacteria	Firmicutes	Clostridia	Clostridiales	Lachnospiraceae		hypothetical protein	csm2gr11	pfam03750	CAS-III-A			
202307..204698	-	KQC84614.1	LLKB01000005.1	Lachnospiraceae bacterium TF01-11	Bacteria	Firmicutes	Clostridia	Clostridiales	Lachnospiraceae		type III-A CRISPR-associated protein Cas10/Csm1	cas10	cd09680	CAS-III			
204699..205455	-	KQC84615.1	LLKB01000005.1	Lachnospiraceae bacterium TF01-11	Bacteria	Firmicutes	Clostridia	Clostridiales	Lachnospiraceae		hypothetical protein	cas6	COG5551	CAS-III			
267 CAS-III-D | SES98352.1																	
44548..44779	-	SES97982.1	FOHN01000006.1	[Clostridium] polysaccharolyticum	Bacteria	Firmicutes	Clostridia	Clostridiales	Lachnospiraceae	Lachnoclostridium	hypothetical protein	csx1	cd09732	CAS-III			
45069..47058	-	SES98000.1	FOHN01000006.1	[Clostridium] polysaccharolyticum	Bacteria	Firmicutes	Clostridia	Clostridiales	Lachnospiraceae	Lachnoclostridium	CRISPR-associated protein	csm3gr7	cls000253	CAS-III-A,CAS-III-D			
47062..47434	-	SES98017.1	FOHN01000006.1	[Clostridium] polysaccharolyticum	Bacteria	Firmicutes	Clostridia	Clostridiales	Lachnospiraceae	Lachnoclostridium	hypothetical protein	csx19	mkCas0132	CAS-III-D			
47423..48752	-	SES98041.1	FOHN01000006.1	[Clostridium] polysaccharolyticum	Bacteria	Firmicutes	Clostridia	Clostridiales	Lachnospiraceae	Lachnoclostridium	CRISPR/Cas system CSM-associated protein Csm3, group 7 of RAMP superfamily	csm3gr7,cmr6gr7,csm3gr7	cd09726,COG1604,cd09726	CAS-III-D			
48755..50963	-	SES98068.1	FOHN01000006.1	[Clostridium] polysaccharolyticum	Bacteria	Firmicutes	Clostridia	Clostridiales	Lachnospiraceae	Lachnoclostridium	CRISPR/Cas system CSM-associated protein Csm3, group 7 of RAMP superfamily	csm3gr7,csx10gr5	mkCas0198,cls000715	CAS-III-A,CAS-III-D			
50964..52614	-	SES98079.1	FOHN01000006.1	[Clostridium] polysaccharolyticum	Bacteria	Firmicutes	Clostridia	Clostridiales	Lachnospiraceae	Lachnoclostridium	hypothetical protein	cas10	mkCas0133	CAS-III			
52606..54295	-	SES98230.1	FOHN01000006.1	[Clostridium] polysaccharolyticum	Bacteria	Firmicutes	Clostridia	Clostridiales	Lachnospiraceae	Lachnoclostridium	hypothetical protein	Csx1	cluster2				
54291..55050	-	SES98245.1	FOHN01000006.1	[Clostridium] polysaccharolyticum	Bacteria	Firmicutes	Clostridia	Clostridiales	Lachnospiraceae	Lachnoclostridium	CRISPR-associated endoribonuclease Cas6	cas6	pfam10040	CAS-III,CAS-IV			
55186..55441	-	SES98267.1	FOHN01000006.1	[Clostridium] polysaccharolyticum	Bacteria	Firmicutes	Clostridia	Clostridiales	Lachnospiraceae	Lachnoclostridium	hypothetical protein						
55433..55889	-	SES98290.1	FOHN01000006.1	[Clostridium] polysaccharolyticum	Bacteria	Firmicutes	Clostridia	Clostridiales	Lachnospiraceae	Lachnoclostridium	4Fe-4S single cluster domain-containing protein						
55878..56586	-	SES98303.1	FOHN01000006.1	[Clostridium] polysaccharolyticum	Bacteria	Firmicutes	Clostridia	Clostridiales	Lachnospiraceae	Lachnoclostridium	putative peptide transport system permease protein						
56592..56919	-	SES98326.1	FOHN01000006.1	[Clostridium] polysaccharolyticum	Bacteria	Firmicutes	Clostridia	Clostridiales	Lachnospiraceae	Lachnoclostridium	ABC transporter	Cas_RecF	cluster17				
57029..57221	-	SES98334.1	FOHN01000006.1	[Clostridium] polysaccharolyticum	Bacteria	Firmicutes	Clostridia	Clostridiales	Lachnospiraceae	Lachnoclostridium	ABC transporter						
59730..60057	-	SES98352.1	FOHN01000006.1	[Clostridium] polysaccharolyticum	Bacteria	Firmicutes	Clostridia	Clostridiales	Lachnospiraceae	Lachnoclostridium	CRISPR-associated protein, Cas2 family	cas2	pfam09827	CAS-I,CAS-II,CAS-III			
60068..61070	-	SES98376.1	FOHN01000006.1	[Clostridium] polysaccharolyticum	Bacteria	Firmicutes	Clostridia	Clostridiales	Lachnospiraceae	Lachnoclostridium	CRISP-associated protein Cas1	cas1	cd09634	CAS-I,CAS-II,CAS-III,CAS-V			
61177..61648	-	SES98400.1	FOHN01000006.1	[Clostridium] polysaccharolyticum	Bacteria	Firmicutes	Clostridia	Clostridiales	Lachnospiraceae	Lachnoclostridium	Probable zinc-ribbon domain-containing protein						
268 CAS-III-A | RRD93721.1																	
24032..25412	-	RRD93719.1	RQYD01000028.1	Clostridiales bacterium COT073_COT-073	Bacteria	Firmicutes	Clostridia	Clostridiales			CRISPR-associated protein Csm6	csm6	cd09746	CAS-III-A			
25426..25615	-	RRD93720.1	RQYD01000028.1	Clostridiales bacterium COT073_COT-073	Bacteria	Firmicutes	Clostridia	Clostridiales			hypothetical protein						
25625..27390	array		RQYD01000028.1	Clostridiales bacterium COT073_COT-073	Bacteria	Firmicutes	Clostridia	Clostridiales				CRISPR array			25	36	36
27633..27948	-	RRD93721.1	RQYD01000028.1	Clostridiales bacterium COT073_COT-073	Bacteria	Firmicutes	Clostridia	Clostridiales			CRISPR-associated endonuclease Cas2	cas2	cd09725	CAS-I,CAS-II,CAS-III,CAS-V			
27959..28958	-	RRD93722.1	RQYD01000028.1	Clostridiales bacterium COT073_COT-073	Bacteria	Firmicutes	Clostridia	Clostridiales			CRISPR-associated endonuclease Cas1	cas1	cd09634	CAS-I,CAS-II,CAS-III,CAS-V			
29211..30423	-	RRD93723.1	RQYD01000028.1	Clostridiales bacterium COT073_COT-073	Bacteria	Firmicutes	Clostridia	Clostridiales			type III-A CRISPR-associated RAMP protein Csm5	csm5gr7	COG1332	CAS-III-A			
30349..31279	-	RRD93724.1	RQYD01000028.1	Clostridiales bacterium COT073_COT-073	Bacteria	Firmicutes	Clostridia	Clostridiales			type III-A CRISPR-associated RAMP protein Csm4	csm4gr5	COG1567	CAS-III-A			
31288..31948	-	RRD93737.1	RQYD01000028.1	Clostridiales bacterium COT073_COT-073	Bacteria	Firmicutes	Clostridia	Clostridiales			type III-A CRISPR-associated RAMP protein Csm3	csm3gr7	cd09684	CAS-III-A,CAS-III-D			
31998..32403	-	RRD93725.1	RQYD01000028.1	Clostridiales bacterium COT073_COT-073	Bacteria	Firmicutes	Clostridia	Clostridiales			type III-A CRISPR-associated protein Csm2	csm2gr11	cd09647	CAS-III-A			
32421..34716	-	RRD93726.1	RQYD01000028.1	Clostridiales bacterium COT073_COT-073	Bacteria	Firmicutes	Clostridia	Clostridiales			type III-A CRISPR-associated protein Cas10/Csm1	cas10	cd09680	CAS-III			
34681..35461	-	RRD93727.1	RQYD01000028.1	Clostridiales bacterium COT073_COT-073	Bacteria	Firmicutes	Clostridia	Clostridiales			CRISPR-associated endoribonuclease Cas6	cas6	pfam10040	CAS-III,CAS-IV			
270 CAS-III-A | RGK40246.1																	
58604..59354	+	RGK40239.1	QSQN01000015.1	Ruminococcus lactaris	Bacteria	Firmicutes	Clostridia	Clostridiales	Ruminococcaceae	Ruminococcus	CRISPR-associated endoribonuclease Cas6	cas6	pfam10040	CAS-III,CAS-IV			
59350..61663	+	RGK40240.1	QSQN01000015.1	Ruminococcus lactaris	Bacteria	Firmicutes	Clostridia	Clostridiales	Ruminococcaceae	Ruminococcus	type III-A CRISPR-associated protein Cas10/Csm1	cas10	cd09680	CAS-III			
61662..62061	+	RGK40241.1	QSQN01000015.1	Ruminococcus lactaris	Bacteria	Firmicutes	Clostridia	Clostridiales	Ruminococcaceae	Ruminococcus	type III-A CRISPR-associated protein Csm2	csm2gr11	cd09647	CAS-III-A			
62097..62754	+	RGK40242.1	QSQN01000015.1	Ruminococcus lactaris	Bacteria	Firmicutes	Clostridia	Clostridiales	Ruminococcaceae	Ruminococcus	type III-A CRISPR-associated RAMP protein Csm3	csm3gr7	cd09684	CAS-III-A,CAS-III-D			
62766..63684	+	RGK40243.1	QSQN01000015.1	Ruminococcus lactaris	Bacteria	Firmicutes	Clostridia	Clostridiales	Ruminococcaceae	Ruminococcus	type III-A CRISPR-associated RAMP protein Csm4	csm4gr5	COG1567	CAS-III-A			
63680..64814	+	RGK40244.1	QSQN01000015.1	Ruminococcus lactaris	Bacteria	Firmicutes	Clostridia	Clostridiales	Ruminococcaceae	Ruminococcus	type III-A CRISPR-associated RAMP protein Csm5	csm5gr7,csm5gr7	cd09662,COG1332	CAS-III-A			
64997..66900	array		QSQN01000015.1	Ruminococcus lactaris	Bacteria	Firmicutes	Clostridia	Clostridiales	Ruminococcaceae	Ruminococcus		CRISPR array			27	35	36
66956..67964	+	RGK40245.1	QSQN01000015.1	Ruminococcus lactaris	Bacteria	Firmicutes	Clostridia	Clostridiales	Ruminococcaceae	Ruminococcus	CRISPR-associated endonuclease Cas1	cas1	cd09634	CAS-I,CAS-II,CAS-III,CAS-V			
67968..68283	+	RGK40246.1	QSQN01000015.1	Ruminococcus lactaris	Bacteria	Firmicutes	Clostridia	Clostridiales	Ruminococcaceae	Ruminococcus	CRISPR-associated endonuclease Cas2	cas2	cd09725	CAS-I,CAS-II,CAS-III,CAS-V			
68311..69694	+	RGK40247.1	QSQN01000015.1	Ruminococcus lactaris	Bacteria	Firmicutes	Clostridia	Clostridiales	Ruminococcaceae	Ruminococcus	hypothetical protein	csm6	pfam09659	CAS-III-A			
271 CAS-III-A | EOS81373.1																	
234272..235004	+	EOS81499.1	KE159726.1	Dorea sp. 5-2	Bacteria	Firmicutes	Clostridia	Clostridiales	Lachnospiraceae	Dorea	CRISPR-associated endoribonuclease cas6	cas6	COG5551	CAS-III			
235051..237364	+	EOS81500.1	KE159726.1	Dorea sp. 5-2	Bacteria	Firmicutes	Clostridia	Clostridiales	Lachnospiraceae	Dorea	CRISPR-associated protein cas10/csm1, subtype III-a/mtube	cas10	cd09680	CAS-III			
237360..237753	+	EOS81501.1	KE159726.1	Dorea sp. 5-2	Bacteria	Firmicutes	Clostridia	Clostridiales	Lachnospiraceae	Dorea	CRISPR type III-a/mtube-associated protein csm2	csm2gr11	cd09647	CAS-III-A			
237765..238422	+	EOS81502.1	KE159726.1	Dorea sp. 5-2	Bacteria	Firmicutes	Clostridia	Clostridiales	Lachnospiraceae	Dorea	CRISPR type III-a/mtube-associated ramp protein csm3	csm3gr7	cd09684	CAS-III-A,CAS-III-D			
238425..239343	+	EOS81503.1	KE159726.1	Dorea sp. 5-2	Bacteria	Firmicutes	Clostridia	Clostridiales	Lachnospiraceae	Dorea	CRISPR type III-a/mtube-associated ramp protein csm4	csm4gr5	COG1567	CAS-III-A			
239346..240471	+	EOS81504.1	KE159726.1	Dorea sp. 5-2	Bacteria	Firmicutes	Clostridia	Clostridiales	Lachnospiraceae	Dorea	CRISPR type III-a/mtube-associated ramp protein csm5	csm5gr7,csm5gr7	cd09662,cd09662	CAS-III-A			
240638..241035	array		KE159726.1	Dorea sp. 5-2	Bacteria	Firmicutes	Clostridia	Clostridiales	Lachnospiraceae	Dorea		CRISPR array			6	36	36
241411..242419	+	EOS81372.1	KE159726.1	Dorea sp. 5-2	Bacteria	Firmicutes	Clostridia	Clostridiales	Lachnospiraceae	Dorea	CRISPR-associated endonuclease cas1	cas1	cd09634	CAS-I,CAS-II,CAS-III,CAS-V			
242423..242738	+	EOS81373.1	KE159726.1	Dorea sp. 5-2	Bacteria	Firmicutes	Clostridia	Clostridiales	Lachnospiraceae	Dorea	CRISPR-associated endoribonuclease cas2	cas2	pfam09827	CAS-I,CAS-II,CAS-III			
242749..244120	+	EOS81374.1	KE159726.1	Dorea sp. 5-2	Bacteria	Firmicutes	Clostridia	Clostridiales	Lachnospiraceae	Dorea	CRISPR type III-a/mtube-associated protein csm6	csm6	cd09746	CAS-III-A			
272 CAS-III-A | CDD66307.1																	
38460..39894	-	CDD66305.1	FR895390.1	Firmicutes bacterium CAG:882	Bacteria	Firmicutes					predicted transcriptional regulator containing an HTH domain and an uncharacterized domain shared with the mammalian protein Schlafen	casR	cls001593	CAS-I			
39983..41327	-	CDD66306.1	FR895390.1	Firmicutes bacterium CAG:882	Bacteria	Firmicutes					cRISPR-associated protein Csm6 family	csm6	cd09746	CAS-III-A			
41390..41705	-	CDD66307.1	FR895390.1	Firmicutes bacterium CAG:882	Bacteria	Firmicutes					cRISPR-associated endoribonuclease Cas2 2	cas2	pfam09827	CAS-I,CAS-II,CAS-III			
41764..42769	-	CDD66308.1	FR895390.1	Firmicutes bacterium CAG:882	Bacteria	Firmicutes					cRISPR-associated endonuclease Cas1 2	cas1	cd09634	CAS-I,CAS-II,CAS-III,CAS-V			
43002..44145	-	CDD66309.1	FR895390.1	Firmicutes bacterium CAG:882	Bacteria	Firmicutes					cRISPR-associated RAMP protein Csm5 family	csm5gr7	COG1332	CAS-III-A			
44182..45091	-	CDD66310.1	FR895390.1	Firmicutes bacterium CAG:882	Bacteria	Firmicutes					cRISPR-associated RAMP protein Csm4 family	csm4gr5	COG1567	CAS-III-A			
45163..45838	-	CDD66311.1	FR895390.1	Firmicutes bacterium CAG:882	Bacteria	Firmicutes					cRISPR-associated protein Csm3 family	csm3gr7	cd09684	CAS-III-A,CAS-III-D			
45849..46248	-	CDD66312.1	FR895390.1	Firmicutes bacterium CAG:882	Bacteria	Firmicutes					cRISPR-associated protein Csm2 family	csm2gr11	cd09647	CAS-III-A			
46244..48581	-	CDD66313.1	FR895390.1	Firmicutes bacterium CAG:882	Bacteria	Firmicutes					cRISPR-associated protein Csm1 family	cas10	cd09680	CAS-III			
48577..49327	-	CDD66314.1	FR895390.1	Firmicutes bacterium CAG:882	Bacteria	Firmicutes					cRISPR-associated protein Cas6	cas6	pfam10040	CAS-III,CAS-IV			
273 CAS-III-A | EFE91104.1																	
364979..366131	-	EFE91097.1	GG729935.1	Oribacterium sp. oral taxon 078 str. F0262	Bacteria	Firmicutes	Clostridia	Clostridiales	Lachnospiraceae	Oribacterium	CRISPR-associated RAMP protein, Csm5 family	csm5gr7	cd09662	CAS-III-A			
366127..367045	-	EFE91098.1	GG729935.1	Oribacterium sp. oral taxon 078 str. F0262	Bacteria	Firmicutes	Clostridia	Clostridiales	Lachnospiraceae	Oribacterium	CRISPR-associated RAMP protein, Csm4 family	csm4gr5	cd09663	CAS-III-A			
367041..367779	-	EFE91099.1	GG729935.1	Oribacterium sp. oral taxon 078 str. F0262	Bacteria	Firmicutes	Clostridia	Clostridiales	Lachnospiraceae	Oribacterium	CRISPR-associated RAMP protein, Csm3 family	csm3gr7	cd09684	CAS-III-A,CAS-III-D			
367794..368268	-	EFE91100.1	GG729935.1	Oribacterium sp. oral taxon 078 str. F0262	Bacteria	Firmicutes	Clostridia	Clostridiales	Lachnospiraceae	Oribacterium	CRISPR-associated protein, Csm2 family	csm2gr11	cd09647	CAS-III-A			
368245..370636	-	EFE91101.1	GG729935.1	Oribacterium sp. oral taxon 078 str. F0262	Bacteria	Firmicutes	Clostridia	Clostridiales	Lachnospiraceae	Oribacterium	CRISPR-associated protein, Csm1 family	cas10	cd09680	CAS-III			
370589..371378	-	EFE91102.1	GG729935.1	Oribacterium sp. oral taxon 078 str. F0262	Bacteria	Firmicutes	Clostridia	Clostridiales	Lachnospiraceae	Oribacterium	CRISPR-associated endoribonuclease Cas6	cas6	pfam10040	CAS-III,CAS-IV			
371536..372841	-	EFE91103.1	GG729935.1	Oribacterium sp. oral taxon 078 str. F0262	Bacteria	Firmicutes	Clostridia	Clostridiales	Lachnospiraceae	Oribacterium	putative CRISPR-associated protein, Csm6 family	csm6	pfam09659	CAS-III-A			
372912..373230	-	EFE91104.1	GG729935.1	Oribacterium sp. oral taxon 078 str. F0262	Bacteria	Firmicutes	Clostridia	Clostridiales	Lachnospiraceae	Oribacterium	CRISPR-associated protein Cas2	cas2	pfam09827	CAS-I,CAS-II,CAS-III			
373235..374234	-	EFE91105.1	GG729935.1	Oribacterium sp. oral taxon 078 str. F0262	Bacteria	Firmicutes	Clostridia	Clostridiales	Lachnospiraceae	Oribacterium	CRISPR-associated endonuclease Cas1	cas1	cd09634	CAS-I,CAS-II,CAS-III,CAS-V			
374505..377305	array		GG729935.1	Oribacterium sp. oral taxon 078 str. F0262	Bacteria	Firmicutes	Clostridia	Clostridiales	Lachnospiraceae	Oribacterium		CRISPR array			40	34	36
274 CAS-III-A | ERL20508.1																	
58653..59793	-	ERL20500.1	KI273116.1	Oribacterium sp. oral taxon 078 str. F0263	Bacteria	Firmicutes	Clostridia	Clostridiales	Lachnospiraceae	Oribacterium	CRISPR-associated RAMP protein, Csm5 family	csm5gr7	cd09662	CAS-III-A			
59789..60707	-	ERL20501.1	KI273116.1	Oribacterium sp. oral taxon 078 str. F0263	Bacteria	Firmicutes	Clostridia	Clostridiales	Lachnospiraceae	Oribacterium	CRISPR-associated RAMP protein, Csm4 family	csm4gr5	cd09663	CAS-III-A			
60675..60966	-	ERL20502.1	KI273116.1	Oribacterium sp. oral taxon 078 str. F0263	Bacteria	Firmicutes	Clostridia	Clostridiales	Lachnospiraceae	Oribacterium	hypothetical protein	csm3gr7	cd09684	CAS-III-A,CAS-III-D			
60910..61423	-	ERL20503.1	KI273116.1	Oribacterium sp. oral taxon 078 str. F0263	Bacteria	Firmicutes	Clostridia	Clostridiales	Lachnospiraceae	Oribacterium	CRISPR-associated RAMP protein, Csm3 family	csm3gr7	cd09684	CAS-III-A,CAS-III-D			
61432..61957	-	ERL20504.1	KI273116.1	Oribacterium sp. oral taxon 078 str. F0263	Bacteria	Firmicutes	Clostridia	Clostridiales	Lachnospiraceae	Oribacterium	CRISPR-associated protein, Csm2 family	csm2gr11	cd09647	CAS-III-A			
61934..64328	-	ERL20505.1	KI273116.1	Oribacterium sp. oral taxon 078 str. F0263	Bacteria	Firmicutes	Clostridia	Clostridiales	Lachnospiraceae	Oribacterium	CRISPR-associated protein, Csm1 family	cas10	cd09680	CAS-III			
64281..65070	-	ERL20506.1	KI273116.1	Oribacterium sp. oral taxon 078 str. F0263	Bacteria	Firmicutes	Clostridia	Clostridiales	Lachnospiraceae	Oribacterium	CRISPR-associated endoribonuclease Cas6	cas6	pfam10040	CAS-III,CAS-IV			
65228..66521	-	ERL20507.1	KI273116.1	Oribacterium sp. oral taxon 078 str. F0263	Bacteria	Firmicutes	Clostridia	Clostridiales	Lachnospiraceae	Oribacterium	CRISPR-associated protein, Csm6 family	csm6	cd09746	CAS-III-A			
66592..66919	-	ERL20508.1	KI273116.1	Oribacterium sp. oral taxon 078 str. F0263	Bacteria	Firmicutes	Clostridia	Clostridiales	Lachnospiraceae	Oribacterium	CRISPR-associated protein Cas2	cas2	pfam09827	CAS-I,CAS-II,CAS-III			
66915..67758	-	ERL20509.1	KI273116.1	Oribacterium sp. oral taxon 078 str. F0263	Bacteria	Firmicutes	Clostridia	Clostridiales	Lachnospiraceae	Oribacterium	CRISPR-associated endonuclease Cas1	cas1	cd09634	CAS-I,CAS-II,CAS-III,CAS-V			
67773..67914	-	ERL20510.1	KI273116.1	Oribacterium sp. oral taxon 078 str. F0263	Bacteria	Firmicutes	Clostridia	Clostridiales	Lachnospiraceae	Oribacterium	hypothetical protein	cas1*	pfam01867				
68184..68501	array		KI273116.1	Oribacterium sp. oral taxon 078 str. F0263	Bacteria	Firmicutes	Clostridia	Clostridiales	Lachnospiraceae	Oribacterium		CRISPR array			5	34	36
68593..68837	array		KI273116.1	Oribacterium sp. oral taxon 078 str. F0263	Bacteria	Firmicutes	Clostridia	Clostridiales	Lachnospiraceae	Oribacterium		CRISPR array			4	32	37
68927..69241	array		KI273116.1	Oribacterium sp. oral taxon 078 str. F0263	Bacteria	Firmicutes	Clostridia	Clostridiales	Lachnospiraceae	Oribacterium		CRISPR array			5	33	36
68989..69193	+	ERL20424.1	KI273116.1	Oribacterium sp. oral taxon 078 str. F0263	Bacteria	Firmicutes	Clostridia	Clostridiales	Lachnospiraceae	Oribacterium	hypothetical protein						
69332..69657	array		KI273116.1	Oribacterium sp. oral taxon 078 str. F0263	Bacteria	Firmicutes	Clostridia	Clostridiales	Lachnospiraceae	Oribacterium		CRISPR array			5	36	36
69753..69996	array		KI273116.1	Oribacterium sp. oral taxon 078 str. F0263	Bacteria	Firmicutes	Clostridia	Clostridiales	Lachnospiraceae	Oribacterium		CRISPR array			4	38	32
69855..69990	+	ERL20285.1	KI273116.1	Oribacterium sp. oral taxon 078 str. F0263	Bacteria	Firmicutes	Clostridia	Clostridiales	Lachnospiraceae	Oribacterium	hypothetical protein						
70085..70403	array		KI273116.1	Oribacterium sp. oral taxon 078 str. F0263	Bacteria	Firmicutes	Clostridia	Clostridiales	Lachnospiraceae	Oribacterium		CRISPR array			5	34	36
70090..70398	+	ERL20222.1	KI273116.1	Oribacterium sp. oral taxon 078 str. F0263	Bacteria	Firmicutes	Clostridia	Clostridiales	Lachnospiraceae	Oribacterium	hypothetical protein						
70493..70742	array		KI273116.1	Oribacterium sp. oral taxon 078 str. F0263	Bacteria	Firmicutes	Clostridia	Clostridiales	Lachnospiraceae	Oribacterium		CRISPR array			4	35	36
70603..70738	-	ERL20156.1	KI273116.1	Oribacterium sp. oral taxon 078 str. F0263	Bacteria	Firmicutes	Clostridia	Clostridiales	Lachnospiraceae	Oribacterium	hypothetical protein						
70831..71218	array		KI273116.1	Oribacterium sp. oral taxon 078 str. F0263	Bacteria	Firmicutes	Clostridia	Clostridiales	Lachnospiraceae	Oribacterium		CRISPR array			6	34	36
71074..71214	-	ERL20017.1	KI273116.1	Oribacterium sp. oral taxon 078 str. F0263	Bacteria	Firmicutes	Clostridia	Clostridiales	Lachnospiraceae	Oribacterium	hypothetical protein						
71310..71559	array		KI273116.1	Oribacterium sp. oral taxon 078 str. F0263	Bacteria	Firmicutes	Clostridia	Clostridiales	Lachnospiraceae	Oribacterium		CRISPR array			4	35	36
71651..71899	array		KI273116.1	Oribacterium sp. oral taxon 078 str. F0263	Bacteria	Firmicutes	Clostridia	Clostridiales	Lachnospiraceae	Oribacterium		CRISPR array			4	35	36
275 CAS-III-A | RGG19528.1																	
114279..115023	+	RGG19521.1	QTWD01000001.1	Blautia sp. AF26-2	Bacteria	Firmicutes	Clostridia	Clostridiales	Lachnospiraceae	Blautia	CRISPR-associated endoribonuclease Cas6	cas6	cd09652	CAS-I,CAS-III			
115019..117329	+	RGG19522.1	QTWD01000001.1	Blautia sp. AF26-2	Bacteria	Firmicutes	Clostridia	Clostridiales	Lachnospiraceae	Blautia	type III-A CRISPR-associated protein Cas10/Csm1	cas10	cd09680	CAS-III			
117325..117718	+	RGG19523.1	QTWD01000001.1	Blautia sp. AF26-2	Bacteria	Firmicutes	Clostridia	Clostridiales	Lachnospiraceae	Blautia	type III-A CRISPR-associated protein Csm2	csm2gr11	cd09647	CAS-III-A			
117730..118384	+	RGG19524.1	QTWD01000001.1	Blautia sp. AF26-2	Bacteria	Firmicutes	Clostridia	Clostridiales	Lachnospiraceae	Blautia	type III-A CRISPR-associated RAMP protein Csm3	csm3gr7	cd09684	CAS-III-A,CAS-III-D			
118394..119306	+	RGG19525.1	QTWD01000001.1	Blautia sp. AF26-2	Bacteria	Firmicutes	Clostridia	Clostridiales	Lachnospiraceae	Blautia	type III-A CRISPR-associated RAMP protein Csm4	csm4gr5	COG1567	CAS-III-A			
119308..120445	+	RGG19526.1	QTWD01000001.1	Blautia sp. AF26-2	Bacteria	Firmicutes	Clostridia	Clostridiales	Lachnospiraceae	Blautia	type III-A CRISPR-associated RAMP protein Csm5	csm5gr7	cd09662	CAS-III-A			
120586..121699	array		QTWD01000001.1	Blautia sp. AF26-2	Bacteria	Firmicutes	Clostridia	Clostridiales	Lachnospiraceae	Blautia		CRISPR array			16	35	36
121828..122833	+	RGG19527.1	QTWD01000001.1	Blautia sp. AF26-2	Bacteria	Firmicutes	Clostridia	Clostridiales	Lachnospiraceae	Blautia	CRISPR-associated endonuclease Cas1	cas1	cd09634	CAS-I,CAS-II,CAS-III,CAS-V			
122852..123170	+	RGG19528.1	QTWD01000001.1	Blautia sp. AF26-2	Bacteria	Firmicutes	Clostridia	Clostridiales	Lachnospiraceae	Blautia	CRISPR-associated endonuclease Cas2	cas2	pfam09827	CAS-I,CAS-II,CAS-III			
123182..124529	+	RGG19529.1	QTWD01000001.1	Blautia sp. AF26-2	Bacteria	Firmicutes	Clostridia	Clostridiales	Lachnospiraceae	Blautia	CRISPR-associated protein Csm6	csm6	pfam09659	CAS-III-A			
276 CAS-III-A | RHP10705.1																	
329478..330225	+	RHP11205.1	QUDV01000001.1	Dorea sp. AF36-15AT	Bacteria	Firmicutes	Clostridia	Clostridiales	Lachnospiraceae	Dorea	CRISPR-associated endoribonuclease Cas6	cas6	cd09652	CAS-I,CAS-III			
330221..332534	+	RHP10699.1	QUDV01000001.1	Dorea sp. AF36-15AT	Bacteria	Firmicutes	Clostridia	Clostridiales	Lachnospiraceae	Dorea	type III-A CRISPR-associated protein Cas10/Csm1	cas10	cd09680	CAS-III			
332533..332938	+	RHP10700.1	QUDV01000001.1	Dorea sp. AF36-15AT	Bacteria	Firmicutes	Clostridia	Clostridiales	Lachnospiraceae	Dorea	type III-A CRISPR-associated protein Csm2	csm2gr11	cd09647	CAS-III-A			
332953..333619	+	RHP10701.1	QUDV01000001.1	Dorea sp. AF36-15AT	Bacteria	Firmicutes	Clostridia	Clostridiales	Lachnospiraceae	Dorea	type III-A CRISPR-associated RAMP protein Csm3	csm3gr7	cd09684	CAS-III-A,CAS-III-D			
333630..334539	+	RHP10702.1	QUDV01000001.1	Dorea sp. AF36-15AT	Bacteria	Firmicutes	Clostridia	Clostridiales	Lachnospiraceae	Dorea	type III-A CRISPR-associated RAMP protein Csm4	csm4gr5	COG1567	CAS-III-A			
334541..335663	+	RHP10703.1	QUDV01000001.1	Dorea sp. AF36-15AT	Bacteria	Firmicutes	Clostridia	Clostridiales	Lachnospiraceae	Dorea	type III-A CRISPR-associated RAMP protein Csm5	csm5gr7	cd09662	CAS-III-A			
335800..337241	array		QUDV01000001.1	Dorea sp. AF36-15AT	Bacteria	Firmicutes	Clostridia	Clostridiales	Lachnospiraceae	Dorea		CRISPR array			21	35	35
337370..338375	+	RHP10704.1	QUDV01000001.1	Dorea sp. AF36-15AT	Bacteria	Firmicutes	Clostridia	Clostridiales	Lachnospiraceae	Dorea	CRISPR-associated endonuclease Cas1	cas1	cd09634	CAS-I,CAS-II,CAS-III,CAS-V			
338398..338716	+	RHP10705.1	QUDV01000001.1	Dorea sp. AF36-15AT	Bacteria	Firmicutes	Clostridia	Clostridiales	Lachnospiraceae	Dorea	CRISPR-associated endonuclease Cas2	cas2	cd09725	CAS-I,CAS-II,CAS-III,CAS-V			
338740..340078	+	RHP10706.1	QUDV01000001.1	Dorea sp. AF36-15AT	Bacteria	Firmicutes	Clostridia	Clostridiales	Lachnospiraceae	Dorea	CRISPR-associated protein Csm6	csm6	cd09746	CAS-III-A			
278 CAS-III-A | CDE69449.1																	
49976..51305	-	CDE69448.1	FR900940.1	Clostridium sp. CAG:277	Bacteria	Firmicutes	Clostridia	Clostridiales	Clostridiaceae	Clostridium	cRISPR-associated protein Csm6 family	csm6	cd09746	CAS-III-A			
51320..51638	-	CDE69449.1	FR900940.1	Clostridium sp. CAG:277	Bacteria	Firmicutes	Clostridia	Clostridiales	Clostridiaceae	Clostridium	cRISPR-associated endoribonuclease Cas2 2	cas2	cd09725	CAS-I,CAS-II,CAS-III,CAS-V			
51654..52659	-	CDE69450.1	FR900940.1	Clostridium sp. CAG:277	Bacteria	Firmicutes	Clostridia	Clostridiales	Clostridiaceae	Clostridium	cRISPR-associated endonuclease Cas1 2	cas1	cd09634	CAS-I,CAS-II,CAS-III,CAS-V			
52709..53027	array		FR900940.1	Clostridium sp. CAG:277	Bacteria	Firmicutes	Clostridia	Clostridiales	Clostridiaceae	Clostridium		CRISPR array			5	36	35
53230..54349	-	CDE69451.1	FR900940.1	Clostridium sp. CAG:277	Bacteria	Firmicutes	Clostridia	Clostridiales	Clostridiaceae	Clostridium	cRISPR-associated RAMP protein Csm5 family	csm5gr7,csm5gr7	cd09662,COG1332	CAS-III-A			
54351..55260	-	CDE69452.1	FR900940.1	Clostridium sp. CAG:277	Bacteria	Firmicutes	Clostridia	Clostridiales	Clostridiaceae	Clostridium	cRISPR-associated RAMP protein Csm4 family	csm4gr5	COG1567	CAS-III-A			
55271..55928	-	CDE69453.1	FR900940.1	Clostridium sp. CAG:277	Bacteria	Firmicutes	Clostridia	Clostridiales	Clostridiaceae	Clostridium	cRISPR-associated RAMP protein Csm3 family	csm3gr7	cd09684	CAS-III-A,CAS-III-D			
55942..56335	-	CDE69454.1	FR900940.1	Clostridium sp. CAG:277	Bacteria	Firmicutes	Clostridia	Clostridiales	Clostridiaceae	Clostridium	cRISPR-associated protein Csm2 family	csm2gr11	pfam03750	CAS-III-A			

56335..58642	-	CDE69455.1	FR900940.1	Clostridium sp. CAG:277	Bacteria	Firmicutes	Clostridia	Clostridiales	Clostridiaceae	Clostridium	cRISPR-associated protein Csm1 family	cas10	cd09680	CAS-III			
58638..59385	-	CDE69456.1	FR900940.1	Clostridium sp. CAG:277	Bacteria	Firmicutes	Clostridia	Clostridiales	Clostridiaceae	Clostridium	cRISPR-associated protein Cas6	cas6	pfam10040	CAS-III,CAS-IV			

279 partial CAS-III-A/CAS-I | RHV04444.1

229..1234	+	RHV04443.1	QULC01000016.1	Clostridium sp. OM07-10AC	Bacteria	Firmicutes	Clostridia	Clostridiales	Clostridiaceae	Clostridium	CRISPR-associated endonuclease Cas1	cas1	cd09634	CAS-I,CAS-II,CAS-III,CAS-V			
1250..1568	+	RHV04444.1	QULC01000016.1	Clostridium sp. OM07-10AC	Bacteria	Firmicutes	Clostridia	Clostridiales	Clostridiaceae	Clostridium	CRISPR-associated endonuclease Cas2	cas2	pfam09827	CAS-I,CAS-II,CAS-III			
1583..2912	+	RHV04445.1	QULC01000016.1	Clostridium sp. OM07-10AC	Bacteria	Firmicutes	Clostridia	Clostridiales	Clostridiaceae	Clostridium	CRISPR-associated protein Csm6	csm6	cd09746	CAS-III-A			

280 CAS-III-A | CUO30698.1

2502..3828	-	CUO30670.1	CYYY01000022.1	Dorea longicatena	Bacteria	Firmicutes	Clostridia	Clostridiales	Lachnospiraceae	Dorea	CRISPR type III-A/MTUBE-associated protein Csm6	csm6	cd09746	CAS-III-A			
3846..4164	-	CUO30698.1	CYYY01000022.1	Dorea longicatena	Bacteria	Firmicutes	Clostridia	Clostridiales	Lachnospiraceae	Dorea	CRISPR-associated endoribonuclease Cas2	cas2	pfam09827	CAS-I,CAS-II,CAS-III			
4200..5205	-	CUO30722.1	CYYY01000022.1	Dorea longicatena	Bacteria	Firmicutes	Clostridia	Clostridiales	Lachnospiraceae	Dorea	CRISPR-associated endonuclease Cas1	cas1	cd09634	CAS-I,CAS-II,CAS-III,CAS-V			
5307..7261	array		CYYY01000022.1	Dorea longicatena	Bacteria	Firmicutes	Clostridia	Clostridiales	Lachnospiraceae	Dorea		CRISPR array			28	35	36
7398..8520	-	CUO30749.1	CYYY01000022.1	Dorea longicatena	Bacteria	Firmicutes	Clostridia	Clostridiales	Lachnospiraceae	Dorea	CRISPR type III-A/MTUBE-associated RAMP protein Csm5	csm5gr7	cd09662	CAS-III-A			
8522..9431	-	CUO30768.1	CYYY01000022.1	Dorea longicatena	Bacteria	Firmicutes	Clostridia	Clostridiales	Lachnospiraceae	Dorea	CRISPR type III-A/MTUBE-associated RAMP protein Csm4	csm4gr5	COG1567	CAS-III-A			
9442..10099	-	CUO30796.1	CYYY01000022.1	Dorea longicatena	Bacteria	Firmicutes	Clostridia	Clostridiales	Lachnospiraceae	Dorea	CRISPR type III-A/MTUBE-associated RAMP protein Csm3	csm3gr7	cd09684	CAS-III-A,CAS-III-D			
10113..10506	-	CUO30826.1	CYYY01000022.1	Dorea longicatena	Bacteria	Firmicutes	Clostridia	Clostridiales	Lachnospiraceae	Dorea	CRISPR type III-A/MTUBE-associated protein Csm2	csm2gr11	cd09647	CAS-III-A			
10486..12790	-	CUO30856.1	CYYY01000022.1	Dorea longicatena	Bacteria	Firmicutes	Clostridia	Clostridiales	Lachnospiraceae	Dorea	CRISPR-associated protein Cas10/Csm1%2C subtype III-A/MTUBE	cas10	cd09680	CAS-III			
12783..13533	-	CUO30879.1	CYYY01000022.1	Dorea longicatena	Bacteria	Firmicutes	Clostridia	Clostridiales	Lachnospiraceae	Dorea	CRISPR-associated endoribonuclease Cas6	cas6	pfam10040	CAS-III,CAS-IV			

281 CAS-III-A | CUN22631.1

28298..29042	+	CUN22556.1	CYYA01000022.1	Eubacterium ramulus	Bacteria	Firmicutes	Clostridia	Clostridiales	Eubacteriaceae	Eubacterium	CRISPR-associated endoribonuclease Cas6	cas6	COG5551	CAS-III			
29038..31393	+	CUN22566.1	CYYA01000022.1	Eubacterium ramulus	Bacteria	Firmicutes	Clostridia	Clostridiales	Eubacteriaceae	Eubacterium	Predicted HD superfamily hydrolase	cas10	cd09680	CAS-III			
31389..31782	+	CUN22575.1	CYYA01000022.1	Eubacterium ramulus	Bacteria	Firmicutes	Clostridia	Clostridiales	Eubacteriaceae	Eubacterium	CRISPR type III-A/MTUBE-associated protein Csm2	csm2gr11	cd09647	CAS-III-A			
31797..32466	+	CUN22584.1	CYYA01000022.1	Eubacterium ramulus	Bacteria	Firmicutes	Clostridia	Clostridiales	Eubacteriaceae	Eubacterium	CRISPR type III-A/MTUBE-associated RAMP protein Csm3	csm3gr7	cd09684	CAS-III-A,CAS-III-D			
32484..33393	+	CUN22593.1	CYYA01000022.1	Eubacterium ramulus	Bacteria	Firmicutes	Clostridia	Clostridiales	Eubacteriaceae	Eubacterium	CRISPR type III-A/MTUBE-associated RAMP protein Csm4	csm4gr5	COG1567	CAS-III-A			
33396..34521	+	CUN22602.1	CYYA01000022.1	Eubacterium ramulus	Bacteria	Firmicutes	Clostridia	Clostridiales	Eubacteriaceae	Eubacterium	CRISPR type III-A/MTUBE-associated RAMP protein Csm5	csm5gr7,csm5gr7	cd09662,cd09662	CAS-III-A			
34657..36490	array		CYYA01000022.1	Eubacterium ramulus	Bacteria	Firmicutes	Clostridia	Clostridiales	Eubacteriaceae	Eubacterium		CRISPR array			26	35	36
35075..35555	-	CUN22611.1	CYYA01000022.1	Eubacterium ramulus	Bacteria	Firmicutes	Clostridia	Clostridiales	Eubacteriaceae	Eubacterium	Uncharacterised protein						
36522..37527	+	CUN22620.1	CYYA01000022.1	Eubacterium ramulus	Bacteria	Firmicutes	Clostridia	Clostridiales	Eubacteriaceae	Eubacterium	CRISPR-associated endonuclease Cas1	cas1	cd09634	CAS-I,CAS-II,CAS-III,CAS-V			
37553..37871	+	CUN22631.1	CYYA01000022.1	Eubacterium ramulus	Bacteria	Firmicutes	Clostridia	Clostridiales	Eubacteriaceae	Eubacterium	CRISPR-associated endoribonuclease Cas2	cas2	cd09725	CAS-I,CAS-II,CAS-III,CAS-V			
37889..39215	+	CUN22640.1	CYYA01000022.1	Eubacterium ramulus	Bacteria	Firmicutes	Clostridia	Clostridiales	Eubacteriaceae	Eubacterium	CRISPR type III-A/MTUBE-associated protein Csm6	csm6	pfam09659	CAS-III-A			

282 CAS-III-A | RHG29043.1

138356..139595	-	RHG29041.1	QRID01000006.1	Roseburia intestinalis	Bacteria	Firmicutes	Clostridia	Clostridiales	Lachnospiraceae	Roseburia	ATP-binding protein	Cas_RecF	cluster17				
139700..141056	-	RHG29042.1	QRID01000006.1	Roseburia intestinalis	Bacteria	Firmicutes	Clostridia	Clostridiales	Lachnospiraceae	Roseburia	CRISPR-associated protein Csm6	csm6	pfam09659	CAS-III-A			
141114..142006	array		QRID01000006.1	Roseburia intestinalis	Bacteria	Firmicutes	Clostridia	Clostridiales	Lachnospiraceae	Roseburia		CRISPR array			13	37	34
142309..142630	-	RHG29043.1	QRID01000006.1	Roseburia intestinalis	Bacteria	Firmicutes	Clostridia	Clostridiales	Lachnospiraceae	Roseburia	CRISPR-associated endonuclease Cas2	cas2	cd09725	CAS-I,CAS-II,CAS-III,CAS-V			
142660..143665	-	RHG29044.1	QRID01000006.1	Roseburia intestinalis	Bacteria	Firmicutes	Clostridia	Clostridiales	Lachnospiraceae	Roseburia	CRISPR-associated endonuclease Cas1	cas1	cd09634	CAS-I,CAS-II,CAS-III,CAS-V			
143753..144294	array		QRID01000006.1	Roseburia intestinalis	Bacteria	Firmicutes	Clostridia	Clostridiales	Lachnospiraceae	Roseburia		CRISPR array			8	36	36
144432..145554	-	RHG29045.1	QRID01000006.1	Roseburia intestinalis	Bacteria	Firmicutes	Clostridia	Clostridiales	Lachnospiraceae	Roseburia	type III-A CRISPR-associated RAMP protein Csm5	csm5gr7	cd09662	CAS-III-A			
145550..146477	-	RHG29126.1	QRID01000006.1	Roseburia intestinalis	Bacteria	Firmicutes	Clostridia	Clostridiales	Lachnospiraceae	Roseburia	type III-A CRISPR-associated RAMP protein Csm4	csm4gr5	COG1567	CAS-III-A			
146520..147183	-	RHG29046.1	QRID01000006.1	Roseburia intestinalis	Bacteria	Firmicutes	Clostridia	Clostridiales	Lachnospiraceae	Roseburia	type III-A CRISPR-associated RAMP protein Csm3	csm3gr7	cd09684	CAS-III-A,CAS-III-D			
147198..147591	-	RHG29047.1	QRID01000006.1	Roseburia intestinalis	Bacteria	Firmicutes	Clostridia	Clostridiales	Lachnospiraceae	Roseburia	type III-A CRISPR-associated protein Csm2	csm2gr11	cd09647	CAS-III-A			
147591..149901	-	RHG29048.1	QRID01000006.1	Roseburia intestinalis	Bacteria	Firmicutes	Clostridia	Clostridiales	Lachnospiraceae	Roseburia	type III-A CRISPR-associated protein Cas10/Csm1	cas10	cd09680	CAS-III			
149897..150644	-	RHG29049.1	QRID01000006.1	Roseburia intestinalis	Bacteria	Firmicutes	Clostridia	Clostridiales	Lachnospiraceae	Roseburia	CRISPR-associated endoribonuclease Cas6	cas6	pfam10040	CAS-III,CAS-IV			

283 CAS-III-A | RHA66005.1

26194..27433	-	RHA66003.1	QSFP01000014.1	Roseburia intestinalis	Bacteria	Firmicutes	Clostridia	Clostridiales	Lachnospiraceae	Roseburia	ATP-binding protein	Cas_RecF	cluster17				
27538..28894	-	RHA66004.1	QSFP01000014.1	Roseburia intestinalis	Bacteria	Firmicutes	Clostridia	Clostridiales	Lachnospiraceae	Roseburia	CRISPR-associated protein Csm6	csm6	cd09746	CAS-III-A			
28950..29268	array		QSFP01000014.1	Roseburia intestinalis	Bacteria	Firmicutes	Clostridia	Clostridiales	Lachnospiraceae	Roseburia		CRISPR array			5	34	36
29571..29910	-	RHA66005.1	QSFP01000014.1	Roseburia intestinalis	Bacteria	Firmicutes	Clostridia	Clostridiales	Lachnospiraceae	Roseburia	CRISPR-associated endonuclease Cas2	cas2	cd09725	CAS-I,CAS-II,CAS-III,CAS-V			
29922..30927	-	RHA66006.1	QSFP01000014.1	Roseburia intestinalis	Bacteria	Firmicutes	Clostridia	Clostridiales	Lachnospiraceae	Roseburia	CRISPR-associated endonuclease Cas1	cas1	cd09634	CAS-I,CAS-II,CAS-III,CAS-V			
31015..31766	array		QSFP01000014.1	Roseburia intestinalis	Bacteria	Firmicutes	Clostridia	Clostridiales	Lachnospiraceae	Roseburia		CRISPR array			11	35	36
31904..33026	-	RHA66007.1	QSFP01000014.1	Roseburia intestinalis	Bacteria	Firmicutes	Clostridia	Clostridiales	Lachnospiraceae	Roseburia	type III-A CRISPR-associated RAMP protein Csm5	csm5gr7,csm5gr7	cd09662,COG1332	CAS-III-A			
33022..33949	-	RHA66078.1	QSFP01000014.1	Roseburia intestinalis	Bacteria	Firmicutes	Clostridia	Clostridiales	Lachnospiraceae	Roseburia	type III-A CRISPR-associated RAMP protein Csm4	csm4gr5	COG1567	CAS-III-A			
33992..34655	-	RHA66008.1	QSFP01000014.1	Roseburia intestinalis	Bacteria	Firmicutes	Clostridia	Clostridiales	Lachnospiraceae	Roseburia	type III-A CRISPR-associated RAMP protein Csm3	csm3gr7	cd09684	CAS-III-A,CAS-III-D			
34670..35063	-	RHA66009.1	QSFP01000014.1	Roseburia intestinalis	Bacteria	Firmicutes	Clostridia	Clostridiales	Lachnospiraceae	Roseburia	type III-A CRISPR-associated protein Csm2	csm2gr11	cd09647	CAS-III-A			
35063..37373	-	RHA66010.1	QSFP01000014.1	Roseburia intestinalis	Bacteria	Firmicutes	Clostridia	Clostridiales	Lachnospiraceae	Roseburia	type III-A CRISPR-associated protein Cas10/Csm1	cas10	cd09680	CAS-III			
37369..38116	-	RHA66011.1	QSFP01000014.1	Roseburia intestinalis	Bacteria	Firmicutes	Clostridia	Clostridiales	Lachnospiraceae	Roseburia	CRISPR-associated endoribonuclease Cas6	cas6	pfam10040	CAS-III,CAS-IV			

284 CAS-III-A | RHM06244.1

111094..112333	-	RHM06242.1	QRPI01000004.1	Roseburia intestinalis	Bacteria	Firmicutes	Clostridia	Clostridiales	Lachnospiraceae	Roseburia	ATP-binding protein	Cas_RecF	cluster17				
112438..113794	-	RHM06243.1	QRPI01000004.1	Roseburia intestinalis	Bacteria	Firmicutes	Clostridia	Clostridiales	Lachnospiraceae	Roseburia	CRISPR-associated protein Csm6	csm6	cd09746	CAS-III-A			
114400..114739	-	RHM06244.1	QRPI01000004.1	Roseburia intestinalis	Bacteria	Firmicutes	Clostridia	Clostridiales	Lachnospiraceae	Roseburia	CRISPR-associated endonuclease Cas2	cas2	cd09725	CAS-I,CAS-II,CAS-III,CAS-V			
114751..115756	-	RHM06245.1	QRPI01000004.1	Roseburia intestinalis	Bacteria	Firmicutes	Clostridia	Clostridiales	Lachnospiraceae	Roseburia	CRISPR-associated endonuclease Cas1	cas1	cd09634	CAS-I,CAS-II,CAS-III,CAS-V			
115843..116960	array		QRPI01000004.1	Roseburia intestinalis	Bacteria	Firmicutes	Clostridia	Clostridiales	Lachnospiraceae	Roseburia		CRISPR array			16	36	36
117098..118220	-	RHM06246.1	QRPI01000004.1	Roseburia intestinalis	Bacteria	Firmicutes	Clostridia	Clostridiales	Lachnospiraceae	Roseburia	type III-A CRISPR-associated RAMP protein Csm5	csm5gr7,csm5gr7	cd09662,COG1332	CAS-III-A			
118216..119143	-	RHM06430.1	QRPI01000004.1	Roseburia intestinalis	Bacteria	Firmicutes	Clostridia	Clostridiales	Lachnospiraceae	Roseburia	type III-A CRISPR-associated RAMP protein Csm4	csm4gr5	COG1567	CAS-III-A			
119186..119849	-	RHM06247.1	QRPI01000004.1	Roseburia intestinalis	Bacteria	Firmicutes	Clostridia	Clostridiales	Lachnospiraceae	Roseburia	type III-A CRISPR-associated RAMP protein Csm3	csm3gr7	cd09684	CAS-III-A,CAS-III-D			
119864..120257	-	RHM06248.1	QRPI01000004.1	Roseburia intestinalis	Bacteria	Firmicutes	Clostridia	Clostridiales	Lachnospiraceae	Roseburia	type III-A CRISPR-associated protein Csm2	csm2gr11	cd09647	CAS-III-A			
120257..122567	-	RHM06249.1	QRPI01000004.1	Roseburia intestinalis	Bacteria	Firmicutes	Clostridia	Clostridiales	Lachnospiraceae	Roseburia	type III-A CRISPR-associated protein Cas10/Csm1	cas10	cd09680	CAS-III			
122563..123310	-	RHM06250.1	QRPI01000004.1	Roseburia intestinalis	Bacteria	Firmicutes	Clostridia	Clostridiales	Lachnospiraceae	Roseburia	CRISPR-associated endoribonuclease Cas6	cas6	pfam10040	CAS-III,CAS-IV			

285 CAS-III-A | EEV02629.1

112599..112938	-	EEV02629.1	GG692714.1	Roseburia intestinalis L1-82	Bacteria	Firmicutes	Clostridia	Clostridiales	Lachnospiraceae	Roseburia	CRISPR-associated protein Cas2	cas2	cd09725	CAS-I,CAS-II,CAS-III,CAS-V			
112950..113955	-	EEV02630.1	GG692714.1	Roseburia intestinalis L1-82	Bacteria	Firmicutes	Clostridia	Clostridiales	Lachnospiraceae	Roseburia	CRISPR-associated endonuclease Cas1	cas1	cd09634	CAS-I,CAS-II,CAS-III,CAS-V			
114042..114708	array		GG692714.1	Roseburia intestinalis L1-82	Bacteria	Firmicutes	Clostridia	Clostridiales	Lachnospiraceae	Roseburia		CRISPR array			10	46	25
114727..114871	-	EEV02631.1	GG692714.1	Roseburia intestinalis L1-82	Bacteria	Firmicutes	Clostridia	Clostridiales	Lachnospiraceae	Roseburia	hypothetical protein						
114857..115979	-	EEV02632.1	GG692714.1	Roseburia intestinalis L1-82	Bacteria	Firmicutes	Clostridia	Clostridiales	Lachnospiraceae	Roseburia	CRISPR-associated RAMP protein, Csm5 family	csm5gr7,csm5gr7	cd09662,COG1332	CAS-III-A			
115975..116902	-	EEV02633.1	GG692714.1	Roseburia intestinalis L1-82	Bacteria	Firmicutes	Clostridia	Clostridiales	Lachnospiraceae	Roseburia	CRISPR-associated RAMP protein, Csm4 family	csm4gr5	COG1567	CAS-III-A			
116945..117608	-	EEV02634.1	GG692714.1	Roseburia intestinalis L1-82	Bacteria	Firmicutes	Clostridia	Clostridiales	Lachnospiraceae	Roseburia	CRISPR-associated RAMP protein, Csm3 family	csm3gr7	cd09684	CAS-III-A,CAS-III-D			
117623..118016	-	EEV02635.1	GG692714.1	Roseburia intestinalis L1-82	Bacteria	Firmicutes	Clostridia	Clostridiales	Lachnospiraceae	Roseburia	CRISPR-associated protein, Csm2 family	csm2gr11	cd09647	CAS-III-A			
118016..120326	-	EEV02636.1	GG692714.1	Roseburia intestinalis L1-82	Bacteria	Firmicutes	Clostridia	Clostridiales	Lachnospiraceae	Roseburia	CRISPR-associated protein, Csm1 family	cas10	cd09680	CAS-III			
120322..121069	-	EEV02637.1	GG692714.1	Roseburia intestinalis L1-82	Bacteria	Firmicutes	Clostridia	Clostridiales	Lachnospiraceae	Roseburia	CRISPR-associated endoribonuclease Cas6	cas6	pfam10040	CAS-III,CAS-IV			

286 CAS-III-A | RHC19824.1

198531..199770	-	RHC19822.1	QSHO01000002.1	Roseburia intestinalis	Bacteria	Firmicutes	Clostridia	Clostridiales	Lachnospiraceae	Roseburia	ATP-binding protein	Cas_RecF	cluster17				
199875..201231	-	RHC19823.1	QSHO01000002.1	Roseburia intestinalis	Bacteria	Firmicutes	Clostridia	Clostridiales	Lachnospiraceae	Roseburia	CRISPR-associated protein Csm6	csm6	pfam09659	CAS-III-A			
201288..201602	array		QSHO01000002.1	Roseburia intestinalis	Bacteria	Firmicutes	Clostridia	Clostridiales	Lachnospiraceae	Roseburia		CRISPR array			5	33	36
201906..202227	-	RHC19824.1	QSHO01000002.1	Roseburia intestinalis	Bacteria	Firmicutes	Clostridia	Clostridiales	Lachnospiraceae	Roseburia	CRISPR-associated endonuclease Cas2	cas2	cd09725	CAS-I,CAS-II,CAS-III,CAS-V			
202257..203262	-	RHC19825.1	QSHO01000002.1	Roseburia intestinalis	Bacteria	Firmicutes	Clostridia	Clostridiales	Lachnospiraceae	Roseburia	CRISPR-associated endonuclease Cas1	cas1	cd09634	CAS-I,CAS-II,CAS-III,CAS-V			
203349..203735	array		QSHO01000002.1	Roseburia intestinalis	Bacteria	Firmicutes	Clostridia	Clostridiales	Lachnospiraceae	Roseburia		CRISPR array			6	47	25
203884..205006	-	RHC19826.1	QSHO01000002.1	Roseburia intestinalis	Bacteria	Firmicutes	Clostridia	Clostridiales	Lachnospiraceae	Roseburia	type III-A CRISPR-associated RAMP protein Csm5	csm5gr7,csm5gr7	cd09662,COG1332	CAS-III-A			
205002..205929	-	RHC19827.1	QSHO01000002.1	Roseburia intestinalis	Bacteria	Firmicutes	Clostridia	Clostridiales	Lachnospiraceae	Roseburia	type III-A CRISPR-associated RAMP protein Csm4	csm4gr5	COG1567	CAS-III-A			
205972..206635	-	RHC19828.1	QSHO01000002.1	Roseburia intestinalis	Bacteria	Firmicutes	Clostridia	Clostridiales	Lachnospiraceae	Roseburia	type III-A CRISPR-associated RAMP protein Csm3	csm3gr7	cd09684	CAS-III-A,CAS-III-D			
206650..207043	-	RHC19829.1	QSHO01000002.1	Roseburia intestinalis	Bacteria	Firmicutes	Clostridia	Clostridiales	Lachnospiraceae	Roseburia	type III-A CRISPR-associated protein Csm2	csm2gr11	cd09647	CAS-III-A			
207043..209353	-	RHC19830.1	QSHO01000002.1	Roseburia intestinalis	Bacteria	Firmicutes	Clostridia	Clostridiales	Lachnospiraceae	Roseburia	type III-A CRISPR-associated protein Cas10/Csm1	cas10	cd09680	CAS-III			
209349..210096	-	RHC19831.1	QSHO01000002.1	Roseburia intestinalis	Bacteria	Firmicutes	Clostridia	Clostridiales	Lachnospiraceae	Roseburia	CRISPR-associated endoribonuclease Cas6	cas6	pfam10040	CAS-III,CAS-IV			

287 CAS-III-A | RHN04100.1

32406..33153	+	RHN04094.1	QRQN01000028.1	Roseburia intestinalis	Bacteria	Firmicutes	Clostridia	Clostridiales	Lachnospiraceae	Roseburia	CRISPR-associated endoribonuclease Cas6	cas6	pfam10040	CAS-III,CAS-IV			
33149..35459	+	RHN04095.1	QRQN01000028.1	Roseburia intestinalis	Bacteria	Firmicutes	Clostridia	Clostridiales	Lachnospiraceae	Roseburia	type III-A CRISPR-associated protein Cas10/Csm1	cas10	cd09680	CAS-III			
35459..35852	+	RHN04096.1	QRQN01000028.1	Roseburia intestinalis	Bacteria	Firmicutes	Clostridia	Clostridiales	Lachnospiraceae	Roseburia	type III-A CRISPR-associated protein Csm2	csm2gr11	cd09647	CAS-III-A			
35867..36530	+	RHN04097.1	QRQN01000028.1	Roseburia intestinalis	Bacteria	Firmicutes	Clostridia	Clostridiales	Lachnospiraceae	Roseburia	type III-A CRISPR-associated RAMP protein Csm3	csm3gr7	cd09684	CAS-III-A,CAS-III-D			
36573..37500	+	RHN04106.1	QRQN01000028.1	Roseburia intestinalis	Bacteria	Firmicutes	Clostridia	Clostridiales	Lachnospiraceae	Roseburia	type III-A CRISPR-associated RAMP protein Csm4	csm4gr5	COG1567	CAS-III-A			
37496..38612	+	RHN04098.1	QRQN01000028.1	Roseburia intestinalis	Bacteria	Firmicutes	Clostridia	Clostridiales	Lachnospiraceae	Roseburia	type III-A CRISPR-associated RAMP protein Csm5	csm5gr7,csm5gr7	cd09662,COG1332	CAS-III-A			
39167..40172	+	RHN04099.1	QRQN01000028.1	Roseburia intestinalis	Bacteria	Firmicutes	Clostridia	Clostridiales	Lachnospiraceae	Roseburia	CRISPR-associated endonuclease Cas1	cas1	cd09634	CAS-I,CAS-II,CAS-III,CAS-V			
40202..40523	+	RHN04100.1	QRQN01000028.1	Roseburia intestinalis	Bacteria	Firmicutes	Clostridia	Clostridiales	Lachnospiraceae	Roseburia	CRISPR-associated endonuclease Cas2	cas2	cd09725	CAS-I,CAS-II,CAS-III,CAS-V			
40880..41415	array		QRQN01000028.1	Roseburia intestinalis	Bacteria	Firmicutes	Clostridia	Clostridiales	Lachnospiraceae	Roseburia		CRISPR array			8	35	36
41470..42826	+	RHN04101.1	QRQN01000028.1	Roseburia intestinalis	Bacteria	Firmicutes	Clostridia	Clostridiales	Lachnospiraceae	Roseburia	CRISPR-associated protein Csm6	csm6	cd09746	CAS-III-A			
42931..44170	+	RHN04102.1	QRQN01000028.1	Roseburia intestinalis	Bacteria	Firmicutes	Clostridia	Clostridiales	Lachnospiraceae	Roseburia	ATP-binding protein	Cas_RecF	cluster17				

288 CAS-III-A | OLA55794.1

92975..94214	-	OLA55793.1	MNTJ01000057.1	Roseburia intestinalis	Bacteria	Firmicutes	Clostridia	Clostridiales	Lachnospiraceae	Roseburia	hypothetical protein	Cas_RecF	cluster17				
96339..96660	-	OLA55794.1	MNTJ01000057.1	Roseburia intestinalis	Bacteria	Firmicutes	Clostridia	Clostridiales	Lachnospiraceae	Roseburia	CRISPR-associated endonuclease Cas2	cas2	cd09725	CAS-I,CAS-II,CAS-III,CAS-V			
96690..97695	-	OLA55795.1	MNTJ01000057.1	Roseburia intestinalis	Bacteria	Firmicutes	Clostridia	Clostridiales	Lachnospiraceae	Roseburia	CRISPR-associated endonuclease Cas1	cas1	cd09634	CAS-I,CAS-II,CAS-III,CAS-V			
98098..99220	-	OLA55796.1	MNTJ01000057.1	Roseburia intestinalis	Bacteria	Firmicutes	Clostridia	Clostridiales	Lachnospiraceae	Roseburia	type III-A CRISPR-associated RAMP protein Csm5	csm5gr7,csm5gr7	cd09662,COG1332	CAS-III-A			
99216..100131	-	OLA55811.1	MNTJ01000057.1	Roseburia intestinalis	Bacteria	Firmicutes	Clostridia	Clostridiales	Lachnospiraceae	Roseburia	type III-A CRISPR-associated RAMP protein Csm4	csm4gr5	COG1567	CAS-III-A			
100186..100849	-	OLA55797.1	MNTJ01000057.1	Roseburia intestinalis	Bacteria	Firmicutes	Clostridia	Clostridiales	Lachnospiraceae	Roseburia	type III-A CRISPR-associated RAMP protein Csm3	csm3gr7	cd09684	CAS-III-A,CAS-III-D			
100864..101257	-	OLA55798.1	MNTJ01000057.1	Roseburia intestinalis	Bacteria	Firmicutes	Clostridia	Clostridiales	Lachnospiraceae	Roseburia	type III-A CRISPR-associated protein Csm2	csm2gr11	cd09647	CAS-III-A			
101257..103567	-	OLA55799.1	MNTJ01000057.1	Roseburia intestinalis	Bacteria	Firmicutes	Clostridia	Clostridiales	Lachnospiraceae	Roseburia	type III-A CRISPR-associated protein Cas10/Csm1	cas10	cd09680	CAS-III			
103563..104310	-	OLA55800.1	MNTJ01000057.1	Roseburia intestinalis	Bacteria	Firmicutes	Clostridia	Clostridiales	Lachnospiraceae	Roseburia	CRISPR-associated endoribonuclease Cas6	cas6	pfam10040	CAS-III,CAS-IV			

289 CAS-I-B | ABR30217.1

360035..360533	array		CP000716.1	Thermosipho melanesiensis BI429	Bacteria	Thermotogae	Thermotogae	Thermotogales	Fervidobacteriaceae	Thermosipho		CRISPR array			8	37	30
360706..360967	-	ABR30217.1	CP000716.1	Thermosipho melanesiensis BI429	Bacteria	Thermotogae	Thermotogae	Thermotogales	Fervidobacteriaceae	Thermosipho	CRISPR-associated protein Cas2	cas2	cd09725	CAS-I,CAS-II,CAS-III,CAS-V			
360969..361965	-	ABR30218.1	CP000716.1	Thermosipho melanesiensis BI429	Bacteria	Thermotogae	Thermotogae	Thermotogales	Fervidobacteriaceae	Thermosipho	CRISPR-associated protein Cas1	cas1	cd09722	CAS-I-B			
361970..362447	-	ABR30219.1	CP000716.1	Thermosipho melanesiensis BI429	Bacteria	Thermotogae	Thermotogae	Thermotogales	Fervidobacteriaceae	Thermosipho	CRISPR-associated protein Cas4	cas4	pfam01930	CAS-I			
362443..362836	-	ABR30220.1	CP000716.1	Thermosipho melanesiensis BI429	Bacteria	Thermotogae	Thermotogae	Thermotogales	Fervidobacteriaceae	Thermosipho	hypothetical protein						
362897..364853	-	ABR30221.1	CP000716.1	Thermosipho melanesiensis BI429	Bacteria	Thermotogae	Thermotogae	Thermotogales	Fervidobacteriaceae	Thermosipho	CRISPR-associated helicase Cas3	cas3HD,cas3	cd09641,COG1203	CAS-I			
364830..365592	-	ABR30222.1	CP000716.1	Thermosipho melanesiensis BI429	Bacteria	Thermotogae	Thermotogae	Thermotogales	Fervidobacteriaceae	Thermosipho	CRISPR-associated protein Cas5	cas5	cls001430	CAS-I-B			
365603..366527	-	ABR30223.1	CP000716.1	Thermosipho melanesiensis BI429	Bacteria	Thermotogae	Thermotogae	Thermotogales	Fervidobacteriaceae	Thermosipho	hypothetical protein	cas7b	pfam05107	CAS-I-B,CAS-I-C			
366541..368242	-	ABR30224.1	CP000716.1	Thermosipho melanesiensis BI429	Bacteria	Thermotogae	Thermotogae	Thermotogales	Fervidobacteriaceae	Thermosipho	hypothetical protein	cas8b1	mkCas0106	CAS-I-B			
368253..368982	-	ABR30225.1	CP000716.1	Thermosipho melanesiensis BI429	Bacteria	Thermotogae	Thermotogae	Thermotogales	Fervidobacteriaceae	Thermosipho	CRISPR-associated protein Cas6	cas6	COG1583	CAS-I,CAS-III,CAS-IV			

290 CAS-I-B | ACJ76376.1

1959890..1963518	array		CP001185.1	Thermosipho africanus TCF52B	Bacteria	Thermotogae	Thermotogae	Thermotogales	Fervidobacteriaceae	Thermosipho		CRISPR array			55	36	30
1959938..1960223	+	ACJ76371.1	CP001185.1	Thermosipho africanus TCF52B	Bacteria	Thermotogae	Thermotogae	Thermotogales	Fervidobacteriaceae	Thermosipho	conserved hypothetical protein						
1961288..1961426	+	ACJ76372.1	CP001185.1	Thermosipho africanus TCF52B	Bacteria	Thermotogae	Thermotogae	Thermotogales	Fervidobacteriaceae	Thermosipho	hypothetical protein						
1962521..1962671	+	ACJ76373.1	CP001185.1	Thermosipho africanus TCF52B	Bacteria	Thermotogae	Thermotogae	Thermotogales	Fervidobacteriaceae	Thermosipho	hypothetical protein						
1963017..1963152	-	ACJ76374.1	CP001185.1	Thermosipho africanus TCF52B	Bacteria	Thermotogae	Thermotogae	Thermotogales	Fervidobacteriaceae	Thermosipho	hypothetical protein						
1963524..1963680	-	ACJ76375.1	CP001185.1	Thermosipho africanus TCF52B	Bacteria	Thermotogae	Thermotogae	Thermotogales	Fervidobacteriaceae	Thermosipho	hypothetical protein						
1963693..1963954	-	ACJ76376.1	CP001185.1	Thermosipho africanus TCF52B	Bacteria	Thermotogae	Thermotogae	Thermotogales	Fervidobacteriaceae	Thermosipho	crispr-associated protein Cas2	cas2	cd09725	CAS-I,CAS-II,CAS-III,CAS-V			
1963956..1964952	-	ACJ76377.1	CP001185.1	Thermosipho africanus TCF52B	Bacteria	Thermotogae	Thermotogae	Thermotogales	Fervidobacteriaceae	Thermosipho	cas crispr-associated protein Cas1	cas1	cd09722	CAS-I-B			
1964957..1965434	-	ACJ76378.1	CP001185.1	Thermosipho africanus TCF52B	Bacteria	Thermotogae	Thermotogae	Thermotogales	Fervidobacteriaceae	Thermosipho	cas crispr-associated protein Cas4	cas4	pfam01930	CAS-I			
1965430..1967836	-	ACJ76379.1	CP001185.1	Thermosipho africanus TCF52B	Bacteria	Thermotogae	Thermotogae	Thermotogales	Fervidobacteriaceae	Thermosipho	crispr-associated helicase Cas3 domain protein, putative	cas3HD,cas3	cd09641,COG1203	CAS-I			
1967813..1968587	-	ACJ76380.1	CP001185.1	Thermosipho africanus TCF52B	Bacteria	Thermotogae	Thermotogae	Thermotogales	Fervidobacteriaceae	Thermosipho	cas crispr-associated protein Cas5	cas5	cls001430	CAS-I-B			
1968599..1969505	-	ACJ76381.1	CP001185.1	Thermosipho africanus TCF52B	Bacteria	Thermotogae	Thermotogae	Thermotogales	Fervidobacteriaceae	Thermosipho	conserved hypothetical protein	cas7b	pfam05107	CAS-I-B,CAS-I-C			
1969518..1971213	-	ACJ76382.1	CP001185.1	Thermosipho africanus TCF52B	Bacteria	Thermotogae	Thermotogae	Thermotogales	Fervidobacteriaceae	Thermosipho	conserved hypothetical protein	cas8b1	mkCas0106	CAS-I-B			

1971225..1971960	-	ACJ76383.1	CP001185.1	Thermosipho africanus TCF52B	Bacteria	Thermotogae	Thermotogae	Thermotogales	Fervidobacteriaceae	Thermosipho	cas crispr-associated protein Cas6	cas6	COG1583	CAS-I,CAS-III,CAS-IV			
291 CAS-I-B | AEX84731.1																	
281543..282308	+	AEX84724.1	CP003257.1	Marinitoga piezophila KA3	Bacteria	Thermotogae	Thermotogae	Petrotogales	Petrotogaceae	Marinitoga	CRISPR-associated endoribonuclease Cas6	cas6	COG1583	CAS-I,CAS-III,CAS-IV			
282403..283783	+	AEX84725.1	CP003257.1	Marinitoga piezophila KA3	Bacteria	Thermotogae	Thermotogae	Petrotogales	Petrotogaceae	Marinitoga	CRISPR-associated protein (Cas_CXXC_CXXC)	cas8b2	cls000029	CAS-I-B			
283795..284848	+	AEX84726.1	CP003257.1	Marinitoga piezophila KA3	Bacteria	Thermotogae	Thermotogae	Petrotogales	Petrotogaceae	Marinitoga	CRISPR-associated protein Cas7/Cst2/DevR, subtype I-B/TNEAP	cas7	cd09687	CAS-I-B			
284850..285507	+	AEX84727.1	CP003257.1	Marinitoga piezophila KA3	Bacteria	Thermotogae	Thermotogae	Petrotogales	Petrotogaceae	Marinitoga	CRISPR-associated protein Cas5, subtype I-B/TNEAP	cas5	COG1688	CAS-I			
285557..287909	+	AEX84728.1	CP003257.1	Marinitoga piezophila KA3	Bacteria	Thermotogae	Thermotogae	Petrotogales	Petrotogaceae	Marinitoga	CRISPR-associated helicase Cas3/CRISPR-associated endonuclease Cas3-HD	cas3	cd09639	CAS-I			
287991..288495	+	AEX84729.1	CP003257.1	Marinitoga piezophila KA3	Bacteria	Thermotogae	Thermotogae	Petrotogales	Petrotogaceae	Marinitoga	CRISPR-associated protein Cas4	cas4	pfam01930	CAS-I			
288499..289495	+	AEX84730.1	CP003257.1	Marinitoga piezophila KA3	Bacteria	Thermotogae	Thermotogae	Petrotogales	Petrotogaceae	Marinitoga	CRISPR-associated endonuclease Cas1	cas1	cd09722	CAS-I-B			
289497..289758	+	AEX84731.1	CP003257.1	Marinitoga piezophila KA3	Bacteria	Thermotogae	Thermotogae	Petrotogales	Petrotogaceae	Marinitoga	CRISPR-associated endoribonuclease Cas2	cas2	cd09725	CAS-I,CAS-II,CAS-III,CAS-V			
289919..290816	array		CP003257.1	Marinitoga piezophila KA3	Bacteria	Thermotogae	Thermotogae	Petrotogales	Petrotogaceae	Marinitoga		CRISPR array			14	36	30
292566..293253	-	AEX84732.1	CP003257.1	Marinitoga piezophila KA3	Bacteria	Thermotogae	Thermotogae	Petrotogales	Petrotogaceae	Marinitoga	transposase						
293444..293720	-	AEX84733.1	CP003257.1	Marinitoga piezophila KA3	Bacteria	Thermotogae	Thermotogae	Petrotogales	Petrotogaceae	Marinitoga	transposase						
293786..294416	array		CP003257.1	Marinitoga piezophila KA3	Bacteria	Thermotogae	Thermotogae	Petrotogales	Petrotogaceae	Marinitoga		CRISPR array			10	39	28
292 CAS-I-B | OQY08079.1																	
190..451	-	OQY08079.1	NBLP01000107.1	Fusobacteriia bacterium 4572_132	Bacteria	Fusobacteria	Fusobacteriia				CRISPR-associated endonuclease Cas2	cas2	cd09725	CAS-I,CAS-II,CAS-III,CAS-V			
457..1453	-	OQY08080.1	NBLP01000107.1	Fusobacteriia bacterium 4572_132	Bacteria	Fusobacteria	Fusobacteriia				subtype I-B CRISPR-associated endonuclease Cas1	cas1	cd09722	CAS-I-B			
1458..1959	-	OQY08081.1	NBLP01000107.1	Fusobacteriia bacterium 4572_132	Bacteria	Fusobacteria	Fusobacteriia				CRISPR-associated protein Cas4	cas4	pfam01930	CAS-I			
1982..4310	-	OQY08082.1	NBLP01000107.1	Fusobacteriia bacterium 4572_132	Bacteria	Fusobacteria	Fusobacteriia				hypothetical protein	cas3HD,cas3	cd09641,COG1203	CAS-I			
4324..5053	-	OQY08083.1	NBLP01000107.1	Fusobacteriia bacterium 4572_132	Bacteria	Fusobacteria	Fusobacteriia				type I-B CRISPR-associated protein Cas5	cas5	cls001430	CAS-I-B			
5063..5813	-	OQY08084.1	NBLP01000107.1	Fusobacteriia bacterium 4572_132	Bacteria	Fusobacteria	Fusobacteriia				type I-B CRISPR-associated protein Cas7/Csh2	cas7b	pfam05107	CAS-I-B,CAS-I-C			
5815..7660	-	OQY08085.1	NBLP01000107.1	Fusobacteriia bacterium 4572_132	Bacteria	Fusobacteria	Fusobacteriia				hypothetical protein	cas8b1	cd09730	CAS-I-B			
7664..8468	-	OQY08086.1	NBLP01000107.1	Fusobacteriia bacterium 4572_132	Bacteria	Fusobacteria	Fusobacteriia				CRISPR-associated endoribonuclease Cas6	cas6	COG1583	CAS-I,CAS-III,CAS-IV			
293 CAS-I-B | OWP56425.1																	
14569..15325	+	OWP56428.1	MWKR01000018.1	Thermoplasmatales archaeon B_DKE	Archaea	Euryarchaeota	Thermoplasmata	Thermoplasmatales			CRISPR-associated endoribonuclease Cas6	cas6	COG1583	CAS-I,CAS-III,CAS-IV			
15327..17313	+	OWP56419.1	MWKR01000018.1	Thermoplasmatales archaeon B_DKE	Archaea	Euryarchaeota	Thermoplasmata	Thermoplasmatales			hypothetical protein	cas8b1	cd09730	CAS-I-B			
17302..18226	+	OWP56420.1	MWKR01000018.1	Thermoplasmatales archaeon B_DKE	Archaea	Euryarchaeota	Thermoplasmata	Thermoplasmatales			type I-B CRISPR-associated protein Cas7/Csh2	cas7	cd09690	CAS-I-B			
18225..19035	+	OWP56421.1	MWKR01000018.1	Thermoplasmatales archaeon B_DKE	Archaea	Euryarchaeota	Thermoplasmata	Thermoplasmatales			hypothetical protein	cas5	cd09692	CAS-I-B			
19034..21527	+	OWP56422.1	MWKR01000018.1	Thermoplasmatales archaeon B_DKE	Archaea	Euryarchaeota	Thermoplasmata	Thermoplasmatales			hypothetical protein	cas3HD,cas3	cd09641,COG1203	CAS-I			
21568..22066	+	OWP56423.1	MWKR01000018.1	Thermoplasmatales archaeon B_DKE	Archaea	Euryarchaeota	Thermoplasmata	Thermoplasmatales			CRISPR-associated protein Cas4	cas4	pfam01930	CAS-I			
22075..23068	+	OWP56424.1	MWKR01000018.1	Thermoplasmatales archaeon B_DKE	Archaea	Euryarchaeota	Thermoplasmata	Thermoplasmatales			subtype I-B CRISPR-associated endonuclease Cas1	cas1	cd09722	CAS-I-B			
23073..23337	+	OWP56425.1	MWKR01000018.1	Thermoplasmatales archaeon B_DKE	Archaea	Euryarchaeota	Thermoplasmata	Thermoplasmatales			CRISPR-associated endonuclease Cas2	cas2	cd09725	CAS-I,CAS-II,CAS-III,CAS-V			
23566..24266	array		MWKR01000018.1	Thermoplasmatales archaeon B_DKE	Archaea	Euryarchaeota	Thermoplasmata	Thermoplasmatales				CRISPR array			11	37	30
294 CAS-I-B | SDY05883.1																	
1..496	array		FNOU01000015.1	Eubacterium barkeri	Bacteria	Firmicutes	Clostridia	Clostridiales	Eubacteriaceae	Eubacterium		CRISPR array			8	36	30
687..951	-	SDY05883.1	FNOU01000015.1	Eubacterium barkeri	Bacteria	Firmicutes	Clostridia	Clostridiales	Eubacteriaceae	Eubacterium	CRISPR-associated protein Cas2	cas2	cd09725	CAS-I,CAS-II,CAS-III,CAS-V			
953..1946	-	SDY05910.1	FNOU01000015.1	Eubacterium barkeri	Bacteria	Firmicutes	Clostridia	Clostridiales	Eubacteriaceae	Eubacterium	CRISPR-associated protein, Cas1 family	cas1	cd09722	CAS-I-B			
1948..2452	-	SDY05938.1	FNOU01000015.1	Eubacterium barkeri	Bacteria	Firmicutes	Clostridia	Clostridiales	Eubacteriaceae	Eubacterium	CRISPR-associated exonuclease, Cas4 family	cas4	pfam01930	CAS-I			
2468..4853	-	SDY06024.1	FNOU01000015.1	Eubacterium barkeri	Bacteria	Firmicutes	Clostridia	Clostridiales	Eubacteriaceae	Eubacterium	CRISPR-associated helicase, Cas3 family	cas3HD,cas3	cd09641,COG1203	CAS-I			
4860..5574	-	SDY06053.1	FNOU01000015.1	Eubacterium barkeri	Bacteria	Firmicutes	Clostridia	Clostridiales	Eubacteriaceae	Eubacterium	CRISPR-associated protein, Cas5h family	cas5	cls001430	CAS-I-B			
5582..6470	-	SDY06084.1	FNOU01000015.1	Eubacterium barkeri	Bacteria	Firmicutes	Clostridia	Clostridiales	Eubacteriaceae	Eubacterium	CRISPR-associated protein, Csh2 family	cas7	cd09690	CAS-I-B			
6466..8392	-	SDY06112.1	FNOU01000015.1	Eubacterium barkeri	Bacteria	Firmicutes	Clostridia	Clostridiales	Eubacteriaceae	Eubacterium	CRISPR-associated protein, Csh1 family	cas8b1	pfam09484	CAS-I-B			
8388..9141	-	SDY06145.1	FNOU01000015.1	Eubacterium barkeri	Bacteria	Firmicutes	Clostridia	Clostridiales	Eubacteriaceae	Eubacterium	CRISPR-associated protein, Cas6 family	cas6	COG1583	CAS-I,CAS-III,CAS-IV			
295 CAS-I-B | EDP75377.1																	
35503..35731	+	EDP75365.1	ABHJ01000005.1	Hydrogenivirga sp. 128-5-R1-1	Bacteria	Aquificae	Aquificae	Aquificales	Aquificaceae	Hydrogenivirga	hypothetical protein	cas2*	COG1343				
35773..36502	+	EDP75366.1	ABHJ01000005.1	Hydrogenivirga sp. 128-5-R1-1	Bacteria	Aquificae	Aquificae	Aquificales	Aquificaceae	Hydrogenivirga	hypothetical protein	cas6	COG1583	CAS-I,CAS-III,CAS-IV			
36501..38211	+	EDP75367.1	ABHJ01000005.1	Hydrogenivirga sp. 128-5-R1-1	Bacteria	Aquificae	Aquificae	Aquificales	Aquificaceae	Hydrogenivirga	hypothetical protein	cas8b1	cd09730	CAS-I-B			
38194..39085	+	EDP75368.1	ABHJ01000005.1	Hydrogenivirga sp. 128-5-R1-1	Bacteria	Aquificae	Aquificae	Aquificales	Aquificaceae	Hydrogenivirga	hypothetical protein	cas7	cd09690	CAS-I-B			
39118..39325	+	EDP75369.1	ABHJ01000005.1	Hydrogenivirga sp. 128-5-R1-1	Bacteria	Aquificae	Aquificae	Aquificales	Aquificaceae	Hydrogenivirga	hypothetical protein						
39321..39558	+	EDP75370.1	ABHJ01000005.1	Hydrogenivirga sp. 128-5-R1-1	Bacteria	Aquificae	Aquificae	Aquificales	Aquificaceae	Hydrogenivirga	hypothetical protein						
39554..40316	+	EDP75371.1	ABHJ01000005.1	Hydrogenivirga sp. 128-5-R1-1	Bacteria	Aquificae	Aquificae	Aquificales	Aquificaceae	Hydrogenivirga	hypothetical protein	cas5	cls001430	CAS-I-B			
40270..42592	+	EDP75372.1	ABHJ01000005.1	Hydrogenivirga sp. 128-5-R1-1	Bacteria	Aquificae	Aquificae	Aquificales	Aquificaceae	Hydrogenivirga	predicted helicase	cas3HD,cas3	cd09641,COG1203	CAS-I			
40468..40612	-	EDP75373.1	ABHJ01000005.1	Hydrogenivirga sp. 128-5-R1-1	Bacteria	Aquificae	Aquificae	Aquificales	Aquificaceae	Hydrogenivirga	hypothetical protein						
42581..43088	+	EDP75374.1	ABHJ01000005.1	Hydrogenivirga sp. 128-5-R1-1	Bacteria	Aquificae	Aquificae	Aquificales	Aquificaceae	Hydrogenivirga	hypothetical protein	cas4	pfam01930	CAS-I			
43084..44074	+	EDP75375.1	ABHJ01000005.1	Hydrogenivirga sp. 128-5-R1-1	Bacteria	Aquificae	Aquificae	Aquificales	Aquificaceae	Hydrogenivirga	hypothetical protein	cas1	cd09722	CAS-I-B			
43588..43750	-	EDP75376.1	ABHJ01000005.1	Hydrogenivirga sp. 128-5-R1-1	Bacteria	Aquificae	Aquificae	Aquificales	Aquificaceae	Hydrogenivirga	hypothetical protein						
44073..44340	+	EDP75377.1	ABHJ01000005.1	Hydrogenivirga sp. 128-5-R1-1	Bacteria	Aquificae	Aquificae	Aquificales	Aquificaceae	Hydrogenivirga	hypothetical protein	cas2	COG1343	CAS-I,CAS-II,CAS-III			
44503..45067	array		ABHJ01000005.1	Hydrogenivirga sp. 128-5-R1-1	Bacteria	Aquificae	Aquificae	Aquificales	Aquificaceae	Hydrogenivirga		CRISPR array			9	36	30
296 CAS-I-B | BAI69698.1																	
1122544..1124919	array		AP011112.1	Hydrogenobacter thermophilus TK-6	Bacteria	Aquificae	Aquificae	Aquificales	Aquificaceae	Hydrogenobacter		CRISPR array			36	37	30
1125080..1125347	-	BAI69698.1	AP011112.1	Hydrogenobacter thermophilus TK-6	Bacteria	Aquificae	Aquificae	Aquificales	Aquificaceae	Hydrogenobacter	CRISPR-associated protein	cas2	COG1343	CAS-I,CAS-II,CAS-III			
1125347..1126337	-	BAI69699.1	AP011112.1	Hydrogenobacter thermophilus TK-6	Bacteria	Aquificae	Aquificae	Aquificales	Aquificaceae	Hydrogenobacter	CRISPR-associated protein	cas1	cd09722	CAS-I-B			
1126333..1126840	-	BAI69700.1	AP011112.1	Hydrogenobacter thermophilus TK-6	Bacteria	Aquificae	Aquificae	Aquificales	Aquificaceae	Hydrogenobacter	CRISPR-associated exonuclease	cas4	pfam01930	CAS-I			
1126829..1129136	-	BAI69701.1	AP011112.1	Hydrogenobacter thermophilus TK-6	Bacteria	Aquificae	Aquificae	Aquificales	Aquificaceae	Hydrogenobacter	CRISPR-associated helicase	cas3	COG1203	CAS-I			
1129090..1129852	-	BAI69702.1	AP011112.1	Hydrogenobacter thermophilus TK-6	Bacteria	Aquificae	Aquificae	Aquificales	Aquificaceae	Hydrogenobacter	CRISPR-associated protein	cas5	cls001430	CAS-I-B			
1129858..1130761	-	BAI69703.1	AP011112.1	Hydrogenobacter thermophilus TK-6	Bacteria	Aquificae	Aquificae	Aquificales	Aquificaceae	Hydrogenobacter	CRISPR-associated protein	cas7	cd09690	CAS-I-B			
1130744..1132487	-	BAI69704.1	AP011112.1	Hydrogenobacter thermophilus TK-6	Bacteria	Aquificae	Aquificae	Aquificales	Aquificaceae	Hydrogenobacter	CRISPR-associated protein	cas8b1	pfam09484	CAS-I-B			
1132470..1133208	-	BAI69705.1	AP011112.1	Hydrogenobacter thermophilus TK-6	Bacteria	Aquificae	Aquificae	Aquificales	Aquificaceae	Hydrogenobacter	CRISPR-associated protein	cas6	COG1583	CAS-I,CAS-III,CAS-IV			
297 CAS-I-B | BAI80039.1																	
519842..520535	-	BAI80034.1	AP011529.1	Deferribacter desulfuricans SSM1	Bacteria	Deferribacteres	Deferribacteres	Deferribacterales	Deferribacteraceae	Deferribacter	CRISPR-associated protein Cas5	cas5	cls001430	CAS-I-B			
520515..520872	-	BAI80035.1	AP011529.1	Deferribacter desulfuricans SSM1	Bacteria	Deferribacteres	Deferribacteres	Deferribacterales	Deferribacteraceae	Deferribacter	conserved hypothetical protein						
520886..521792	-	BAI80036.1	AP011529.1	Deferribacter desulfuricans SSM1	Bacteria	Deferribacteres	Deferribacteres	Deferribacterales	Deferribacteraceae	Deferribacter	CRISPR-associated protein, Csh2 family	cas7	cd09690	CAS-I-B			
521834..523511	-	BAI80037.1	AP011529.1	Deferribacter desulfuricans SSM1	Bacteria	Deferribacteres	Deferribacteres	Deferribacterales	Deferribacteraceae	Deferribacter	CRISPR-associated protein, Csh1 family	cas8b1	cd09672	CAS-I-B			
523494..524271	-	BAI80038.1	AP011529.1	Deferribacter desulfuricans SSM1	Bacteria	Deferribacteres	Deferribacteres	Deferribacterales	Deferribacteraceae	Deferribacter	CRISPR-associated protein Cas6	cas6	COG1583	CAS-I,CAS-III,CAS-IV			
524315..524582	-	BAI80039.1	AP011529.1	Deferribacter desulfuricans SSM1	Bacteria	Deferribacteres	Deferribacteres	Deferribacterales	Deferribacteraceae	Deferribacter	CRISPR-associated protein Cas2	cas2	cd09725	CAS-I,CAS-II,CAS-III,CAS-V			
524585..525578	-	BAI80040.1	AP011529.1	Deferribacter desulfuricans SSM1	Bacteria	Deferribacteres	Deferribacteres	Deferribacterales	Deferribacteraceae	Deferribacter	CRISPR-associated protein Cas1	cas1	cd09722	CAS-I-B			
525584..526079	-	BAI80041.1	AP011529.1	Deferribacter desulfuricans SSM1	Bacteria	Deferribacteres	Deferribacteres	Deferribacterales	Deferribacteraceae	Deferribacter	CRISPR-associated protein Cas4	cas4	pfam01930	CAS-I			
526129..527564	array		AP011529.1	Deferribacter desulfuricans SSM1	Bacteria	Deferribacteres	Deferribacteres	Deferribacterales	Deferribacteraceae	Deferribacter		CRISPR array			22	36	30
298 partial | SNZ07756.1																	
26822..27089	+	SNZ07756.1	OBEI01000003.1	Persephonella hydrogeniphila	Bacteria	Aquificae	Aquificae	Aquificales	Hydrogenothermaceae	Persephonella	CRISPR-associated protein Cas2	cas2	cd09725	CAS-I,CAS-II,CAS-III,CAS-V			
27425..28466	array		OBEI01000003.1	Persephonella hydrogeniphila	Bacteria	Aquificae	Aquificae	Aquificales	Hydrogenothermaceae	Persephonella		CRISPR array			16	37	30
299 CAS-I-B | ACN99300.1																	
787449..788196	-	ACN98535.1	CP001229.1	Sulfurihydrogenibium azorense Az-Fu1	Bacteria	Aquificae	Aquificae	Aquificales	Hydrogenothermaceae	Sulfurihydrogenibium	crispr-associated protein Cas6	cas6	COG1583	CAS-I,CAS-III,CAS-IV			
788247..788740	array		CP001229.1	Sulfurihydrogenibium azorense Az-Fu1	Bacteria	Aquificae	Aquificae	Aquificales	Hydrogenothermaceae	Sulfurihydrogenibium		CRISPR array			8	36	30
788987..789254	-	ACN99300.1	CP001229.1	Sulfurihydrogenibium azorense Az-Fu1	Bacteria	Aquificae	Aquificae	Aquificales	Hydrogenothermaceae	Sulfurihydrogenibium	crispr-associated protein Cas2	cas2	cd09725	CAS-I,CAS-II,CAS-III,CAS-V			
789256..789661	-	ACN99643.1	CP001229.1	Sulfurihydrogenibium azorense Az-Fu1	Bacteria	Aquificae	Aquificae	Aquificales	Hydrogenothermaceae	Sulfurihydrogenibium	crispr-associated protein Cas1	cas1	cd09722	CAS-I-B			
789614..789965	-	ACN98661.1	CP001229.1	Sulfurihydrogenibium azorense Az-Fu1	Bacteria	Aquificae	Aquificae	Aquificales	Hydrogenothermaceae	Sulfurihydrogenibium	crispr-associated protein Cas1	cas1	cd09722	CAS-I-B			
790167..790350	-	ACN98974.1	CP001229.1	Sulfurihydrogenibium azorense Az-Fu1	Bacteria	Aquificae	Aquificae	Aquificales	Hydrogenothermaceae	Sulfurihydrogenibium	hypothetical protein	cas4	pfam01930	CAS-I			
790620..792909	-	ACN98263.1	CP001229.1	Sulfurihydrogenibium azorense Az-Fu1	Bacteria	Aquificae	Aquificae	Aquificales	Hydrogenothermaceae	Sulfurihydrogenibium	crispr-associated helicase Cas3	cas3HD,cas3	cd09641,COG1203	CAS-I			
792917..793607	-	ACN98723.1	CP001229.1	Sulfurihydrogenibium azorense Az-Fu1	Bacteria	Aquificae	Aquificae	Aquificales	Hydrogenothermaceae	Sulfurihydrogenibium	crispr-associated protein Cas5, hmari subtype	cas5	cls001430	CAS-I-B			
793608..794556	-	ACN99478.1	CP001229.1	Sulfurihydrogenibium azorense Az-Fu1	Bacteria	Aquificae	Aquificae	Aquificales	Hydrogenothermaceae	Sulfurihydrogenibium	crispr-associated protein, Csh2 family	cas7	cd09690	CAS-I-B			
794542..796246	-	ACN98938.1	CP001229.1	Sulfurihydrogenibium azorense Az-Fu1	Bacteria	Aquificae	Aquificae	Aquificales	Hydrogenothermaceae	Sulfurihydrogenibium	crispr-associated protein, family	cas8b1	cd09672	CAS-I-B			
300 CAS-I-B | SNR89705.1																	
35870..37586	+	SNR89603.1	FZOB01000014.1	Desulfurobacterium atlanticum	Bacteria	Aquificae	Aquificae	Desulfurobacteriales	Desulfurobacteriaceae	Desulfurobacterium	CRISPR-associated protein, Csh1 family	cas8b1	pfam09484	CAS-I-B			
37578..38496	+	SNR89620.1	FZOB01000014.1	Desulfurobacterium atlanticum	Bacteria	Aquificae	Aquificae	Desulfurobacteriales	Desulfurobacteriaceae	Desulfurobacterium	CRISPR-associated protein Csh2	cas7	cd09690	CAS-I-B			
38480..39227	+	SNR89634.1	FZOB01000014.1	Desulfurobacterium atlanticum	Bacteria	Aquificae	Aquificae	Desulfurobacteriales	Desulfurobacteriaceae	Desulfurobacterium	CRISPR-associated protein Cas5h	cas5	cls001430	CAS-I-B			
39165..41451	+	SNR89663.1	FZOB01000014.1	Desulfurobacterium atlanticum	Bacteria	Aquificae	Aquificae	Desulfurobacteriales	Desulfurobacteriaceae	Desulfurobacterium	CRISPR-associated helicase, Cas3 family	cas3HD,cas3	cd09641,COG1203	CAS-I			
41452..41956	+	SNR89674.1	FZOB01000014.1	Desulfurobacterium atlanticum	Bacteria	Aquificae	Aquificae	Desulfurobacteriales	Desulfurobacteriaceae	Desulfurobacterium	CRISPR-associated exonuclease, Cas4 family	cas4	pfam01930	CAS-I			
41957..42932	+	SNR89693.1	FZOB01000014.1	Desulfurobacterium atlanticum	Bacteria	Aquificae	Aquificae	Desulfurobacteriales	Desulfurobacteriaceae	Desulfurobacterium	CRISP-associated protein Cas1	cas1	cd09722	CAS-I-B			
42931..43198	+	SNR89705.1	FZOB01000014.1	Desulfurobacterium atlanticum	Bacteria	Aquificae	Aquificae	Desulfurobacteriales	Desulfurobacteriaceae	Desulfurobacterium	CRISPR-associated protein Cas2	cas2	cd09725	CAS-I,CAS-II,CAS-III,CAS-V			
43390..45091	array		FZOB01000014.1	Desulfurobacterium atlanticum	Bacteria	Aquificae	Aquificae	Desulfurobacteriales	Desulfurobacteriaceae	Desulfurobacterium		CRISPR array			26	36	30
301 CAS-I-B | AWR95398.1																	
2379221..2381409	array		CP029289.1	Acidianus brierleyi	Archaea	Crenarchaeota	Thermoprotei	Sulfolobales	Sulfolobaceae	Acidianus		CRISPR array			34	40	25
2382422..2382977	+	AWR95395.1	CP029289.1	Acidianus brierleyi	Archaea	Crenarchaeota	Thermoprotei	Sulfolobales	Sulfolobaceae	Acidianus	CRISPR-associated protein	csx1	cd09723	CAS-III			
2383714..2384047	+	AWR95396.1	CP029289.1	Acidianus brierleyi	Archaea	Crenarchaeota	Thermoprotei	Sulfolobales	Sulfolobaceae	Acidianus	nucleotidyltransferase domain-containing protein						
2384362..2386618	+	AWR95397.1	CP029289.1	Acidianus brierleyi	Archaea	Crenarchaeota	Thermoprotei	Sulfolobales	Sulfolobaceae	Acidianus	hypothetical protein	cas3HD,cas3,cas3	cd09641,cd09639,COG1203	CAS-I			
2386695..2388538	array		CP029289.1	Acidianus brierleyi	Archaea	Crenarchaeota	Thermoprotei	Sulfolobales	Sulfolobaceae	Acidianus		CRISPR array			28	37	30
2389487..2389745	+	AWR95398.1	CP029289.1	Acidianus brierleyi	Archaea	Crenarchaeota	Thermoprotei	Sulfolobales	Sulfolobaceae	Acidianus	CRISPR-associated endonuclease Cas2	cas2	cd09725	CAS-I,CAS-II,CAS-III,CAS-V			
2390451..2393892	array		CP029289.1	Acidianus brierleyi	Archaea	Crenarchaeota	Thermoprotei	Sulfolobales	Sulfolobaceae	Acidianus		CRISPR array			52	36	30
2394060..2394585	+	AWR95399.1	CP029289.1	Acidianus brierleyi	Archaea	Crenarchaeota	Thermoprotei	Sulfolobales	Sulfolobaceae	Acidianus	CRISPR-associated protein Cas4	cas4	pfam01930	CAS-I			
2394600..2395536	+	AWR95400.1	CP029289.1	Acidianus brierleyi	Archaea	Crenarchaeota	Thermoprotei	Sulfolobales	Sulfolobaceae	Acidianus	subtype I-B CRISPR-associated endonuclease Cas1	cas1	cd09722	CAS-I-B			
2395699..2397466	-	AWR95401.1	CP029289.1	Acidianus brierleyi	Archaea	Crenarchaeota	Thermoprotei	Sulfolobales	Sulfolobaceae	Acidianus	hypothetical protein	cas8b1	pfam09484	CAS-I-B			
2397763..2398669	+	AWR95402.1	CP029289.1	Acidianus brierleyi	Archaea	Crenarchaeota	Thermoprotei	Sulfolobales	Sulfolobaceae	Acidianus	type I-B CRISPR-associated protein Cas7/Csh2	cas7	cd09690	CAS-I-B			
2398675..2399374	+	AWR95403.1	CP029289.1	Acidianus brierleyi	Archaea	Crenarchaeota	Thermoprotei	Sulfolobales	Sulfolobaceae	Acidianus	hypothetical protein	cas5	cls001430	CAS-I-B			
2399437..2400172	+	AWR95404.1	CP029289.1	Acidianus brierleyi	Archaea	Crenarchaeota	Thermoprotei	Sulfolobales	Sulfolobaceae	Acidianus	CRISPR-associated endoribonuclease Cas6	cas6	COG1583	CAS-I,CAS-III,CAS-IV			
2401616..2403605	+	AWR95405.1	CP029289.1	Acidianus brierleyi	Archaea	Crenarchaeota	Thermoprotei	Sulfolobales	Sulfolobaceae	Acidianus	hypothetical protein	cas10	mkCas0133	CAS-III			
2403591..2404122	+	AWR95406.1	CP029289.1	Acidianus brierleyi	Archaea	Crenarchaeota	Thermoprotei	Sulfolobales	Sulfolobaceae	Acidianus	hypothetical protein	csm3gr7	pfam03787	CAS-III-A,CAS-III-D			
2404102..2404528	+	AWR95407.1	CP029289.1	Acidianus brierleyi	Archaea	Crenarchaeota	Thermoprotei	Sulfolobales	Sulfolobaceae	Acidianus	hypothetical protein	csm3gr7	pfam03787	CAS-III-A,CAS-III-D			
302 CAS-I-B | ODN29671.1																	
465..1377	+	ODN29667.1	LWAF01000027.1	Fervidobacterium thailandense	Bacteria	Thermotogae	Thermotogae	Thermotogales	Fervidobacteriaceae	Fervidobacterium	hypothetical protein	cas7	cd09690	CAS-I-B			
1390..2122	+	ODN29668.1	LWAF01000027.1	Fervidobacterium thailandense	Bacteria	Thermotogae	Thermotogae	Thermotogales	Fervidobacteriaceae	Fervidobacterium	hypothetical protein	cas5	cd09692	CAS-I-B			
2194..4645	+	ODN29669.1	LWAF01000027.1	Fervidobacterium thailandense	Bacteria	Thermotogae	Thermotogae	Thermotogales	Fervidobacteriaceae	Fervidobacterium	hypothetical protein	cas3HD,cas3	cd09641,COG1203	CAS-I			
4638..5148	+	ODN29670.1	LWAF01000027.1	Fervidobacterium thailandense	Bacteria	Thermotogae	Thermotogae	Thermotogales	Fervidobacteriaceae	Fervidobacterium	hypothetical protein	cas4	pfam01930	CAS-I			
5147..6140	+	ODN29673.1	LWAF01000027.1	Fervidobacterium thailandense	Bacteria	Thermotogae	Thermotogae	Thermotogales	Fervidobacteriaceae	Fervidobacterium	subtype I-B CRISPR-associated endonuclease Cas1	cas1	cd09722	CAS-I-B			
6149..6410	+	ODN29671.1	LWAF01000027.1	Fervidobacterium thailandense	Bacteria	Thermotogae	Thermotogae	Thermotogales	Fervidobacteriaceae	Fervidobacterium	CRISPR-associated endonuclease Cas2	cas2	cd09725	CAS-I,CAS-II,CAS-III,CAS-V			
6601..6841	-	ODN29672.1	LWAF01000027.1	Fervidobacterium thailandense	Bacteria	Thermotogae	Thermotogae	Thermotogales	Fervidobacteriaceae	Fervidobacterium	hypothetical protein						
6669..7221	array		LWAF01000027.1	Fervidobacterium thailandense	Bacteria	Thermotogae	Thermotogae	Thermotogales	Fervidobacteriaceae	Fervidobacterium		CRISPR array			9	43	23
303 CAS-I-B | AEH51739.1																	
1706221..1706968	-	AEH51736.1	CP002351.1	Pseudothermotoga thermarum DSM 5069	Bacteria	Thermotogae	Thermotogae	Thermotogales	Thermotogaceae	Pseudothermotoga	CRISPR-associated protein Cas6	cas6	COG1583	CAS-I,CAS-III,CAS-IV			
1707168..1707660	+	AEH51737.1	CP002351.1	Pseudothermotoga thermarum DSM 5069	Bacteria	Thermotogae	Thermotogae	Thermotogales	Thermotogaceae	Pseudothermotoga	CRISPR-associated exonuclease, Cas4 family	cas4	pfam01930	CAS-I			
1707671..1708652	+	AEH51738.1	CP002351.1	Pseudothermotoga thermarum DSM 5069	Bacteria	Thermotogae	Thermotogae	Thermotogales	Thermotogaceae	Pseudothermotoga	CRISPR-associated protein, Cas1 family	cas1	cd09722	CAS-I-B			
1708654..1708915	+	AEH51739.1	CP002351.1	Pseudothermotoga thermarum DSM 5069	Bacteria	Thermotogae	Thermotogae	Thermotogales	Thermotogaceae	Pseudothermotoga	CRISPR-associated protein, Cas2 family	cas2	cd09725	CAS-I,CAS-II,CAS-III,CAS-V			
1708934..1710662	+	AEH51740.1	CP002351.1	Pseudothermotoga thermarum DSM 5069	Bacteria	Thermotogae	Thermotogae	Thermotogales	Thermotogaceae	Pseudothermotoga	CRISPR-associated protein, TM1802 family	cas8b1	pfam09484	CAS-I-B			
1710654..1711593	+	AEH51741.1	CP002351.1	Pseudothermotoga thermarum DSM 5069	Bacteria	Thermotogae	Thermotogae	Thermotogales	Thermotogaceae	Pseudothermotoga	CRISPR-associated protein, Csh2 family	cas7	cd09690	CAS-I-B			
1711589..1712297	+	AEH51742.1	CP002351.1	Pseudothermotoga thermarum DSM 5069	Bacteria	Thermotogae	Thermotogae	Thermotogales	Thermotogaceae	Pseudothermotoga	CRISPR-associated protein Cas5, Hmari subtype	cas5	cls001430	CAS-I-B			
1712281..1714510	+	AEH51743.1	CP002351.1	Pseudothermotoga thermarum DSM 5069	Bacteria	Thermotogae	Thermotogae	Thermotogales	Thermotogaceae	Pseudothermotoga	CRISPR-associated HD domain protein	cas3HD,cas3	cd09641,COG1203	CAS-I			
1714941..1717563	array		CP002351.1	Pseudothermotoga thermarum DSM 5069	Bacteria	Thermotogae	Thermotogae	Thermotogales	Thermotogaceae	Pseudothermotoga		CRISPR array			40	36	30

304 CAS-I-B | ABV32777.1

205528..208415	array		CP000812.1	Pseudothermotoga lettingae TMO	Bacteria	Thermotogae	Thermotogae	Thermotogales	Thermotogaceae	Pseudothermotoga		CRISPR array			44	36	30
208842..211077	-	ABV32773.1	CP000812.1	Pseudothermotoga lettingae TMO	Bacteria	Thermotogae	Thermotogae	Thermotogales	Thermotogaceae	Pseudothermotoga	CRISPR-associated helicase Cas3	cas3HD,cas3	cd09641,COG1203	CAS-I			
211061..211754	-	ABV32774.1	CP000812.1	Pseudothermotoga lettingae TMO	Bacteria	Thermotogae	Thermotogae	Thermotogales	Thermotogaceae	Pseudothermotoga	CRISPR-associated protein Cas5, Hmari subtype	cas5	cls001430	CAS-I-B			
211750..212635	-	ABV32775.1	CP000812.1	Pseudothermotoga lettingae TMO	Bacteria	Thermotogae	Thermotogae	Thermotogales	Thermotogaceae	Pseudothermotoga	CRISPR-associated protein, Csh2 family	cas7	cd09690	CAS-I-B			
212618..214346	-	ABV32776.1	CP000812.1	Pseudothermotoga lettingae TMO	Bacteria	Thermotogae	Thermotogae	Thermotogales	Thermotogaceae	Pseudothermotoga	CRISPR-associated protein, TM1802 family	cas8b1	cd09730	CAS-I-B			
214357..214621	-	ABV32777.1	CP000812.1	Pseudothermotoga lettingae TMO	Bacteria	Thermotogae	Thermotogae	Thermotogales	Thermotogaceae	Pseudothermotoga	CRISPR-associated protein Cas2	**cas2**	cd09725	CAS-I,CAS-II,CAS-III,CAS-V			
214627..215611	-	ABV32778.1	CP000812.1	Pseudothermotoga lettingae TMO	Bacteria	Thermotogae	Thermotogae	Thermotogales	Thermotogaceae	Pseudothermotoga	CRISPR-associated protein Cas1	cas1	cd09722	CAS-I-B			
215621..216113	-	ABV32779.1	CP000812.1	Pseudothermotoga lettingae TMO	Bacteria	Thermotogae	Thermotogae	Thermotogales	Thermotogaceae	Pseudothermotoga	CRISPR-associated protein Cas4	cas4	pfam01930	CAS-I			
216129..216891	-	ABV32780.1	CP000812.1	Pseudothermotoga lettingae TMO	Bacteria	Thermotogae	Thermotogae	Thermotogales	Thermotogaceae	Pseudothermotoga	CRISPR-associated protein Cas6	cas6	COG1583	CAS-I,CAS-III,CAS-IV			

305 partial CAS-I-B | KUK23932.1

0..252	+	KUK23929.1	LGFH01000097.1	Thermotoga sp. 50_1627	Bacteria	Thermotogae	Thermotogae	Thermotogales	Thermotogaceae	Thermotoga	CRISPR-associated protein Cas6	cas6	COG1583	CAS-I,CAS-III,CAS-IV			
248..749	+	KUK23930.1	LGFH01000097.1	Thermotoga sp. 50_1627	Bacteria	Thermotogae	Thermotogae	Thermotogales	Thermotogaceae	Thermotoga	CRISPR-associated protein Cas4	cas4	pfam01930	CAS-I			
758..1742	+	KUK23931.1	LGFH01000097.1	Thermotoga sp. 50_1627	Bacteria	Thermotogae	Thermotogae	Thermotogales	Thermotogaceae	Thermotoga	CRISPR-associated endonuclease Cas1	cas1	cd09722	CAS-I-B			
1815..2079	+	KUK23932.1	LGFH01000097.1	Thermotoga sp. 50_1627	Bacteria	Thermotogae	Thermotogae	Thermotogales	Thermotogaceae	Thermotoga	CRISPR-associated endoribonuclease Cas2	**cas2**	cd09725	CAS-I,CAS-II,CAS-III,CAS-V			
2094..2565	+	KUK23933.1	LGFH01000097.1	Thermotoga sp. 50_1627	Bacteria	Thermotogae	Thermotogae	Thermotogales	Thermotogaceae	Thermotoga	hypothetical protein	cas8b1	pfam09484	CAS-I-B			

307 CAS-I-B | OUP77394.1

61874..62567	+	OUP77387.1	NFKR01000010.1	Erysipelatoclostridium sp. An173	Bacteria	Firmicutes	Erysipelotrichia	Erysipelotrichales	Erysipelotrichaceae	Erysipelatoclostridium	CRISPR-associated endoribonuclease Cas6	cas6	cd09652	CAS-I,CAS-III			
62584..64303	+	OUP77388.1	NFKR01000010.1	Erysipelatoclostridium sp. An173	Bacteria	Firmicutes	Erysipelotrichia	Erysipelotrichales	Erysipelotrichaceae	Erysipelatoclostridium	hypothetical protein	cas8b1	mkCas0106	CAS-I-B			
64295..65234	+	OUP77389.1	NFKR01000010.1	Erysipelatoclostridium sp. An173	Bacteria	Firmicutes	Erysipelotrichia	Erysipelotrichales	Erysipelotrichaceae	Erysipelatoclostridium	CRISPR-associated protein	cas7b	pfam05107	CAS-I-B,CAS-I-C			
65238..65943	+	OUP77390.1	NFKR01000010.1	Erysipelatoclostridium sp. An173	Bacteria	Firmicutes	Erysipelotrichia	Erysipelotrichales	Erysipelotrichaceae	Erysipelatoclostridium	type I-B CRISPR-associated protein Cas5	cas5	cls001430	CAS-I-B			
65980..68485	+	OUP77391.1	NFKR01000010.1	Erysipelatoclostridium sp. An173	Bacteria	Firmicutes	Erysipelotrichia	Erysipelotrichales	Erysipelotrichaceae	Erysipelatoclostridium	CRISPR-associated helicase/endonuclease Cas3	cas3HD,cas3	cd09641,cd09639	CAS-I			
68495..68990	+	OUP77392.1	NFKR01000010.1	Erysipelatoclostridium sp. An173	Bacteria	Firmicutes	Erysipelotrichia	Erysipelotrichales	Erysipelotrichaceae	Erysipelatoclostridium	CRISPR-associated protein Cas4	cas4	pfam01930	CAS-I			
69001..70000	+	OUP77393.1	NFKR01000010.1	Erysipelatoclostridium sp. An173	Bacteria	Firmicutes	Erysipelotrichia	Erysipelotrichales	Erysipelotrichaceae	Erysipelatoclostridium	subtype I-B CRISPR-associated endonuclease Cas1	cas1	cd09722	CAS-I-B			
69999..70290	+	OUP77394.1	NFKR01000010.1	Erysipelatoclostridium sp. An173	Bacteria	Firmicutes	Erysipelotrichia	Erysipelotrichales	Erysipelotrichaceae	Erysipelatoclostridium	CRISPR-associated endonuclease Cas2	**cas2**	cd09725	CAS-I,CAS-II,CAS-III,CAS-V			
70466..73850	array		NFKR01000010.1	Erysipelatoclostridium sp. An173	Bacteria	Firmicutes	Erysipelotrichia	Erysipelotrichales	Erysipelotrichaceae	Erysipelatoclostridium		CRISPR array			52	35	30

308 CAS-I-B | EEA85704.1

7082..7778	+	EEA85697.1	DS995684.1	[Clostridium] hiranonis DSM 13275	Bacteria	Firmicutes	Clostridia	Clostridiales	Peptostreptococcaceae		CRISPR-associated endoribonuclease Cas6	cas6	cd09652	CAS-I,CAS-III			
7843..9610	+	EEA85698.1	DS995684.1	[Clostridium] hiranonis DSM 13275	Bacteria	Firmicutes	Clostridia	Clostridiales	Peptostreptococcaceae		hypothetical protein	cas8b1	mkCas0106	CAS-I-B			
9625..10588	+	EEA85699.1	DS995684.1	[Clostridium] hiranonis DSM 13275	Bacteria	Firmicutes	Clostridia	Clostridiales	Peptostreptococcaceae		CRISPR-associated protein, CT1132 family	cas7b	pfam05107	CAS-I-B,CAS-I-C			
10591..11341	+	EEA85700.1	DS995684.1	[Clostridium] hiranonis DSM 13275	Bacteria	Firmicutes	Clostridia	Clostridiales	Peptostreptococcaceae		CRISPR-associated protein Cas5, Hmari subtype	cas5	cls001430	CAS-I-B			
11393..13979	+	EEA85701.1	DS995684.1	[Clostridium] hiranonis DSM 13275	Bacteria	Firmicutes	Clostridia	Clostridiales	Peptostreptococcaceae		CRISPR-associated helicase Cas3	cas3HD,cas3	cd09641,cd09639	CAS-I			
14012..14501	+	EEA85702.1	DS995684.1	[Clostridium] hiranonis DSM 13275	Bacteria	Firmicutes	Clostridia	Clostridiales	Peptostreptococcaceae		CRISPR-associated protein Cas4	cas4	pfam01930	CAS-I			
14513..15512	+	EEA85703.1	DS995684.1	[Clostridium] hiranonis DSM 13275	Bacteria	Firmicutes	Clostridia	Clostridiales	Peptostreptococcaceae		CRISPR-associated endonuclease Cas1, HMARI/TNEAP subtype	cas1	cd09722	CAS-I-B			
15514..15796	+	EEA85704.1	DS995684.1	[Clostridium] hiranonis DSM 13275	Bacteria	Firmicutes	Clostridia	Clostridiales	Peptostreptococcaceae		CRISPR-associated protein Cas2	**cas2**	cd09725	CAS-I,CAS-II,CAS-III,CAS-V			
15934..18010	array		DS995684.1	[Clostridium] hiranonis DSM 13275	Bacteria	Firmicutes	Clostridia	Clostridiales	Peptostreptococcaceae			CRISPR array			32	36	30

309 CAS-I-B | SNU99538.1

1166382..1167862	array		LT906446.1	Megamonas hypermegale	Bacteria	Firmicutes	Negativicutes	Selenomonadales	Selenomonadaceae	Megamonas		CRISPR array			23	35	30
1168043..1168334	-	SNU99538.1	LT906446.1	Megamonas hypermegale	Bacteria	Firmicutes	Negativicutes	Selenomonadales	Selenomonadaceae	Megamonas	CRISPR-associated endoribonuclease Cas2	**cas2**	cd09725	CAS-I,CAS-II,CAS-III,CAS-V			
1168337..1169336	-	SNU99541.1	LT906446.1	Megamonas hypermegale	Bacteria	Firmicutes	Negativicutes	Selenomonadales	Selenomonadaceae	Megamonas	CRISPR-associated endonuclease Cas1, subtype I-B/HMARI/TNEAP	cas1	cd09722	CAS-I-B			
1169362..1169854	-	SNU99547.1	LT906446.1	Megamonas hypermegale	Bacteria	Firmicutes	Negativicutes	Selenomonadales	Selenomonadaceae	Megamonas	CRISPR-associated protein Cas4	cas4	pfam01930	CAS-I			
1169873..1172543	-	SNU99552.1	LT906446.1	Megamonas hypermegale	Bacteria	Firmicutes	Negativicutes	Selenomonadales	Selenomonadaceae	Megamonas	helicase Cas3	cas3HD,cas3	cd09641,cd09639	CAS-I			
1172609..1173428	-	SNU99558.1	LT906446.1	Megamonas hypermegale	Bacteria	Firmicutes	Negativicutes	Selenomonadales	Selenomonadaceae	Megamonas	Uncharacterized protein predicted to be involved in DNA repair (RAMP superfamily)	cas5	cls001430	CAS-I-B			
1173431..1174448	-	SNU99563.1	LT906446.1	Megamonas hypermegale	Bacteria	Firmicutes	Negativicutes	Selenomonadales	Selenomonadaceae	Megamonas	Uncharacterized protein predicted to be involved in DNA repair	cas7b	pfam05107	CAS-I-B,CAS-I-C			
1174471..1176307	-	SNU99570.1	LT906446.1	Megamonas hypermegale	Bacteria	Firmicutes	Negativicutes	Selenomonadales	Selenomonadaceae	Megamonas	Uncharacterised protein	cas8b1	mkCas0106	CAS-I-B			
1176334..1177027	-	SNU99576.1	LT906446.1	Megamonas hypermegale	Bacteria	Firmicutes	Negativicutes	Selenomonadales	Selenomonadaceae	Megamonas	Uncharacterized protein predicted to be involved in DNA repair (RAMP superfamily)	cas6	cd09652	CAS-I,CAS-III			

310 CAS-I-B | KNZ42391.1

51005..51698	+	KNZ42384.1	LGYO01000015.1	Acetobacterium bakii	Bacteria	Firmicutes	Clostridia	Clostridiales	Eubacteriaceae	Acetobacterium	hypothetical protein	cas6	cd09652	CAS-I,CAS-III			
51709..53470	+	KNZ42385.1	LGYO01000015.1	Acetobacterium bakii	Bacteria	Firmicutes	Clostridia	Clostridiales	Eubacteriaceae	Acetobacterium	hypothetical protein	cas8b1	mkCas0106	CAS-I-B			
53459..54446	+	KNZ42386.1	LGYO01000015.1	Acetobacterium bakii	Bacteria	Firmicutes	Clostridia	Clostridiales	Eubacteriaceae	Acetobacterium	CRISPR-associated protein	cas7b	pfam05107	CAS-I-B,CAS-I-C			
54448..55204	+	KNZ42387.1	LGYO01000015.1	Acetobacterium bakii	Bacteria	Firmicutes	Clostridia	Clostridiales	Eubacteriaceae	Acetobacterium	hypothetical protein	cas5	cls001430	CAS-I-B			
55213..57841	+	KNZ42388.1	LGYO01000015.1	Acetobacterium bakii	Bacteria	Firmicutes	Clostridia	Clostridiales	Eubacteriaceae	Acetobacterium	hypothetical protein	cas3HD,cas3	cd09641,cd09639	CAS-I			
57852..58347	+	KNZ42389.1	LGYO01000015.1	Acetobacterium bakii	Bacteria	Firmicutes	Clostridia	Clostridiales	Eubacteriaceae	Acetobacterium	CRISPR-associated protein Cas4	cas4	pfam01930	CAS-I			
58359..59358	+	KNZ42390.1	LGYO01000015.1	Acetobacterium bakii	Bacteria	Firmicutes	Clostridia	Clostridiales	Eubacteriaceae	Acetobacterium	CRISPR-associated protein Cas1	cas1	cd09722	CAS-I-B			
59361..59646	+	KNZ42391.1	LGYO01000015.1	Acetobacterium bakii	Bacteria	Firmicutes	Clostridia	Clostridiales	Eubacteriaceae	Acetobacterium	CRISPR-associated protein Cas2	**cas2**	cd09725	CAS-I,CAS-II,CAS-III,CAS-V			
59806..60360	array		LGYO01000015.1	Acetobacterium bakii	Bacteria	Firmicutes	Clostridia	Clostridiales	Eubacteriaceae	Acetobacterium		CRISPR array			9	35	30

311 CAS-I-B | PWW54092.1

344565..345258	+	PWW54085.1	QGUD01000005.1	Eubacterium limosum	Bacteria	Firmicutes	Clostridia	Clostridiales	Eubacteriaceae	Eubacterium	CRISPR-associated endoribonuclease Cas6	cas6	cd09652	CAS-I,CAS-III			
345272..347012	+	PWW54086.1	QGUD01000005.1	Eubacterium limosum	Bacteria	Firmicutes	Clostridia	Clostridiales	Eubacteriaceae	Eubacterium	CRISPR-associated protein Csh1	cas8b1	mkCas0106	CAS-I-B			
347004..347940	+	PWW54087.1	QGUD01000005.1	Eubacterium limosum	Bacteria	Firmicutes	Clostridia	Clostridiales	Eubacteriaceae	Eubacterium	CRISPR-associated protein Csh2	cas7b	pfam05107	CAS-I-B,CAS-I-C			
347941..348682	+	PWW54088.1	QGUD01000005.1	Eubacterium limosum	Bacteria	Firmicutes	Clostridia	Clostridiales	Eubacteriaceae	Eubacterium	CRISPR-associated protein Cas5h	cas5	cls001430	CAS-I-B			
348668..351254	+	PWW54089.1	QGUD01000005.1	Eubacterium limosum	Bacteria	Firmicutes	Clostridia	Clostridiales	Eubacteriaceae	Eubacterium	CRISPR-associated endonuclease/helicase Cas3	cas3HD,cas3	cd09641,cd09639	CAS-I			
351264..351759	+	PWW54090.1	QGUD01000005.1	Eubacterium limosum	Bacteria	Firmicutes	Clostridia	Clostridiales	Eubacteriaceae	Eubacterium	CRISPR-associated Cas4 family exonuclease	cas4	pfam01930	CAS-I			
351770..352769	+	PWW54091.1	QGUD01000005.1	Eubacterium limosum	Bacteria	Firmicutes	Clostridia	Clostridiales	Eubacteriaceae	Eubacterium	CRISPR-associated Cas1 family protein	cas1	cd09722	CAS-I-B			
352765..353056	+	PWW54092.1	QGUD01000005.1	Eubacterium limosum	Bacteria	Firmicutes	Clostridia	Clostridiales	Eubacteriaceae	Eubacterium	CRISPR-associated Cas2 family protein	**cas2**	cd09725	CAS-I,CAS-II,CAS-III,CAS-V			
353237..355230	array		QGUD01000005.1	Eubacterium limosum	Bacteria	Firmicutes	Clostridia	Clostridiales	Eubacteriaceae	Eubacterium		CRISPR array			31	35	30

312 CAS-III-C/CAS-I-B | ACR79377.1

702542..702812	-	ACR79377.1	CP001634.1	Kosmotoga olearia TBF 19.5.1	Bacteria	Thermotogae	Thermotogae	Kosmotogales	Kosmotogaceae	Kosmotoga	CRISPR-associated protein Cas2	**cas2**	cd09725	CAS-I,CAS-II,CAS-III,CAS-V			
703620..704451	-	ACR79378.1	CP001634.1	Kosmotoga olearia TBF 19.5.1	Bacteria	Thermotogae	Thermotogae	Kosmotogales	Kosmotogaceae	Kosmotoga	protein of unknown function DUF324	cmr3gr5	mkCas0067	CAS-III-C			
704450..704849	-	ACR79379.1	CP001634.1	Kosmotoga olearia TBF 19.5.1	Bacteria	Thermotogae	Thermotogae	Kosmotogales	Kosmotogaceae	Kosmotoga	hypothetical protein	cmr5gr11	mkCas0060	CAS-III-C			
704848..705793	-	ACR79380.1	CP001634.1	Kosmotoga olearia TBF 19.5.1	Bacteria	Thermotogae	Thermotogae	Kosmotogales	Kosmotogaceae	Kosmotoga	CRISPR-associated RAMP protein, Cmr4 family	cmr4gr7	COG1336	CAS-III-C			
705789..708771	-	ACR79381.1	CP001634.1	Kosmotoga olearia TBF 19.5.1	Bacteria	Thermotogae	Thermotogae	Kosmotogales	Kosmotogaceae	Kosmotoga	CRISPR-associated protein, Csx11 family	cas10	cd09701	CAS-III-C			
708804..710007	-	ACR79382.1	CP001634.1	Kosmotoga olearia TBF 19.5.1	Bacteria	Thermotogae	Thermotogae	Kosmotogales	Kosmotogaceae	Kosmotoga	protein of unknown function DUF324	cmr6gr7	mkCas0142	CAS-III-C			
709996..711076	-	ACR79383.1	CP001634.1	Kosmotoga olearia TBF 19.5.1	Bacteria	Thermotogae	Thermotogae	Kosmotogales	Kosmotogaceae	Kosmotoga	CRISPR-associated RAMP protein, Cmr1 family	cmr1gr7	cd09657	CAS-III-C			
711096..711372	-	ACR79384.1	CP001634.1	Kosmotoga olearia TBF 19.5.1	Bacteria	Thermotogae	Thermotogae	Kosmotogales	Kosmotogaceae	Kosmotoga	CRISPR-associated protein Cas2	cas2	COG1343	CAS-I,CAS-II,CAS-III			
711368..712358	-	ACR79385.1	CP001634.1	Kosmotoga olearia TBF 19.5.1	Bacteria	Thermotogae	Thermotogae	Kosmotogales	Kosmotogaceae	Kosmotoga	CRISPR-associated protein Cas1	cas1	cd09722	CAS-I-B			
712365..712863	-	ACR79386.1	CP001634.1	Kosmotoga olearia TBF 19.5.1	Bacteria	Thermotogae	Thermotogae	Kosmotogales	Kosmotogaceae	Kosmotoga	CRISPR-associated protein Cas4	cas4	pfam01930	CAS-I			
712859..715190	-	ACR79387.1	CP001634.1	Kosmotoga olearia TBF 19.5.1	Bacteria	Thermotogae	Thermotogae	Kosmotogales	Kosmotogaceae	Kosmotoga	CRISPR-associated helicase Cas3	cas3HD,cas3	cd09641,COG1203	CAS-I			
715182..715893	-	ACR79388.1	CP001634.1	Kosmotoga olearia TBF 19.5.1	Bacteria	Thermotogae	Thermotogae	Kosmotogales	Kosmotogaceae	Kosmotoga	CRISPR-associated protein Cas5, Hmari subtype	cas5	cls001430	CAS-I-B			
715904..716843	-	ACR79389.1	CP001634.1	Kosmotoga olearia TBF 19.5.1	Bacteria	Thermotogae	Thermotogae	Kosmotogales	Kosmotogaceae	Kosmotoga	CRISPR-associated protein, Csh2 family	cas7	cd09690	CAS-I-B			
716845..718645	-	ACR79390.1	CP001634.1	Kosmotoga olearia TBF 19.5.1	Bacteria	Thermotogae	Thermotogae	Kosmotogales	Kosmotogaceae	Kosmotoga	hypothetical protein	cas8b1	pfam09484	CAS-I-B			
718651..719380	-	ACR79391.1	CP001634.1	Kosmotoga olearia TBF 19.5.1	Bacteria	Thermotogae	Thermotogae	Kosmotogales	Kosmotogaceae	Kosmotoga	CRISPR-associated protein Cas6	cas6	COG1583	CAS-I,CAS-III,CAS-IV			
719736..725328	array		CP001634.1	Kosmotoga olearia TBF 19.5.1	Bacteria	Thermotogae	Thermotogae	Kosmotogales	Kosmotogaceae	Kosmotoga		CRISPR array			86	35	30

313 CAS-I-B | PNS37164.1

12..4025	array		JWIM01000059.1	Mesotoga sp. B105.6.4	Bacteria	Thermotogae	Thermotogae	Kosmotogales	Kosmotogaceae	Mesotoga		CRISPR array			61	36	30
4192..4462	-	PNS37164.1	JWIM01000059.1	Mesotoga sp. B105.6.4	Bacteria	Thermotogae	Thermotogae	Kosmotogales	Kosmotogaceae	Mesotoga	CRISPR-associated protein Cas2	**cas2**	cd09725	CAS-I,CAS-II,CAS-III,CAS-V			
4465..5455	-	PNS37165.1	JWIM01000059.1	Mesotoga sp. B105.6.4	Bacteria	Thermotogae	Thermotogae	Kosmotogales	Kosmotogaceae	Mesotoga	CRISPR-associated protein Cas1	cas1	cd09722	CAS-I-B			
5459..5954	-	PNS37194.1	JWIM01000059.1	Mesotoga sp. B105.6.4	Bacteria	Thermotogae	Thermotogae	Kosmotogales	Kosmotogaceae	Mesotoga	CRISPR-associated protein Cas4	cas4	pfam01930	CAS-I			
5953..8290	-	PNS37166.1	JWIM01000059.1	Mesotoga sp. B105.6.4	Bacteria	Thermotogae	Thermotogae	Kosmotogales	Kosmotogaceae	Mesotoga	hypothetical protein	cas3HD,cas3	cd09641,COG1203	CAS-I			
8282..8987	-	PNS37167.1	JWIM01000059.1	Mesotoga sp. B105.6.4	Bacteria	Thermotogae	Thermotogae	Kosmotogales	Kosmotogaceae	Mesotoga	hypothetical protein	cas5	cls001430	CAS-I-B			
8998..9988	-	PNS37168.1	JWIM01000059.1	Mesotoga sp. B105.6.4	Bacteria	Thermotogae	Thermotogae	Kosmotogales	Kosmotogaceae	Mesotoga	CRISPR-associated protein Csh2	cas7	cd09690	CAS-I-B			
9990..11781	-	PNS37169.1	JWIM01000059.1	Mesotoga sp. B105.6.4	Bacteria	Thermotogae	Thermotogae	Kosmotogales	Kosmotogaceae	Mesotoga	hypothetical protein	cas8b1	pfam09484	CAS-I-B			
11787..12522	-	PNS37170.1	JWIM01000059.1	Mesotoga sp. B105.6.4	Bacteria	Thermotogae	Thermotogae	Kosmotogales	Kosmotogaceae	Mesotoga	hypothetical protein	cas6	COG1583	CAS-I,CAS-III,CAS-IV			

314 CAS-I-B | PVD17378.1

68940..77722	array		AYTW01000028.1	Mesotoga sp. Brook.08.105.5.1	Bacteria	Thermotogae	Thermotogae	Kosmotogales	Kosmotogaceae	Mesotoga		CRISPR array			134	35	30
78654..78924	-	PVD17378.1	AYTW01000028.1	Mesotoga sp. Brook.08.105.5.1	Bacteria	Thermotogae	Thermotogae	Kosmotogales	Kosmotogaceae	Mesotoga	CRISPR-associated protein Cas2	**cas2**	COG1343	CAS-I,CAS-II,CAS-III			
78927..79917	-	PVD17379.1	AYTW01000028.1	Mesotoga sp. Brook.08.105.5.1	Bacteria	Thermotogae	Thermotogae	Kosmotogales	Kosmotogaceae	Mesotoga	CRISPR-associated protein Cas1	cas1	cd09722	CAS-I-B			
79921..80419	-	PVD17380.1	AYTW01000028.1	Mesotoga sp. Brook.08.105.5.1	Bacteria	Thermotogae	Thermotogae	Kosmotogales	Kosmotogaceae	Mesotoga	CRISPR-associated protein Cas4	cas4	pfam01930	CAS-I			
80415..82752	-	PVD17381.1	AYTW01000028.1	Mesotoga sp. Brook.08.105.5.1	Bacteria	Thermotogae	Thermotogae	Kosmotogales	Kosmotogaceae	Mesotoga	hypothetical protein	cas3HD,cas3	cd09641,COG1203	CAS-I			
82744..83449	-	PVD17382.1	AYTW01000028.1	Mesotoga sp. Brook.08.105.5.1	Bacteria	Thermotogae	Thermotogae	Kosmotogales	Kosmotogaceae	Mesotoga	hypothetical protein	cas5	cls001430	CAS-I-B			
83460..84411	-	PVD17383.1	AYTW01000028.1	Mesotoga sp. Brook.08.105.5.1	Bacteria	Thermotogae	Thermotogae	Kosmotogales	Kosmotogaceae	Mesotoga	CRISPR-associated protein Csh2	cas7	cd09690	CAS-I-B			
84413..86204	-	PVD17384.1	AYTW01000028.1	Mesotoga sp. Brook.08.105.5.1	Bacteria	Thermotogae	Thermotogae	Kosmotogales	Kosmotogaceae	Mesotoga	hypothetical protein	cas8b1	pfam09484	CAS-I-B			
86210..86945	-	PVD17385.1	AYTW01000028.1	Mesotoga sp. Brook.08.105.5.1	Bacteria	Thermotogae	Thermotogae	Kosmotogales	Kosmotogaceae	Mesotoga	hypothetical protein	cas6	COG1583	CAS-I,CAS-III,CAS-IV			

315 CAS-I-B | AFK07931.1

2484839..2491949	array		CP003532.1	Mesotoga prima MesG1.Ag.4.2	Bacteria	Thermotogae	Thermotogae	Kosmotogales	Kosmotogaceae	Mesotoga		CRISPR array			108	36	30
2492116..2492386	-	AFK07931.1	CP003532.1	Mesotoga prima MesG1.Ag.4.2	Bacteria	Thermotogae	Thermotogae	Kosmotogales	Kosmotogaceae	Mesotoga	CRISPR-associated endoribonuclease Cas2	**cas2**	cd09725	CAS-I,CAS-II,CAS-III,CAS-V			
2492389..2493379	-	AFK07932.1	CP003532.1	Mesotoga prima MesG1.Ag.4.2	Bacteria	Thermotogae	Thermotogae	Kosmotogales	Kosmotogaceae	Mesotoga	CRISPR-associated endonuclease Cas1	cas1	cd09722	CAS-I-B			
2493383..2493881	-	AFK07933.1	CP003532.1	Mesotoga prima MesG1.Ag.4.2	Bacteria	Thermotogae	Thermotogae	Kosmotogales	Kosmotogaceae	Mesotoga	CRISPR-associated protein Cas4	cas4	pfam01930	CAS-I			
2493877..2496223	-	AFK07934.1	CP003532.1	Mesotoga prima MesG1.Ag.4.2	Bacteria	Thermotogae	Thermotogae	Kosmotogales	Kosmotogaceae	Mesotoga	CRISPR-associated helicase Cas3/CRISPR-associated endonuclease Cas3-HD	cas3HD,cas3,cas3	cd09641,COG1203,cd09696	CAS-I			
2496219..2496945	-	AFK07935.1	CP003532.1	Mesotoga prima MesG1.Ag.4.2	Bacteria	Thermotogae	Thermotogae	Kosmotogales	Kosmotogaceae	Mesotoga	CRISPR-associated protein Cas5, subtype I-B/HMARI	cas5	cls001430	CAS-I-B			
2496956..2497907	-	AFK07936.1	CP003532.1	Mesotoga prima MesG1.Ag.4.2	Bacteria	Thermotogae	Thermotogae	Kosmotogales	Kosmotogaceae	Mesotoga	CRISPR-associated protein Cas7/Csh2, subtype I-B/HMARI	cas7	cd09690	CAS-I-B			
2497927..2499682	-	AFK07937.1	CP003532.1	Mesotoga prima MesG1.Ag.4.2	Bacteria	Thermotogae	Thermotogae	Kosmotogales	Kosmotogaceae	Mesotoga	CRISPR-associated protein, TM1802 family	cas8b1	pfam09484	CAS-I-B			
2499686..2500421	-	AFK07938.1	CP003532.1	Mesotoga prima MesG1.Ag.4.2	Bacteria	Thermotogae	Thermotogae	Kosmotogales	Kosmotogaceae	Mesotoga	CRISPR-associated endoribonuclease Cas6	cas6	COG1583	CAS-I,CAS-III,CAS-IV			

316 CAS-I-B | PZC51854.1

57..820	array		AYSI01000129.1	Mesotoga sp. TolDC	Bacteria	Thermotogae	Thermotogae	Kosmotogales	Kosmotogaceae	Mesotoga		CRISPR array			12	36	30
849..3191	array		AYSI01000129.1	Mesotoga sp. TolDC	Bacteria	Thermotogae	Thermotogae	Kosmotogales	Kosmotogaceae	Mesotoga		CRISPR array			36	36	30
3358..3628	-	PZC51854.1	AYSI01000129.1	Mesotoga sp. TolDC	Bacteria	Thermotogae	Thermotogae	Kosmotogales	Kosmotogaceae	Mesotoga	CRISPR-associated protein Cas2	**cas2**	cd09725	CAS-I,CAS-II,CAS-III,CAS-V			
3631..4621	-	PZC51855.1	AYSI01000129.1	Mesotoga sp. TolDC	Bacteria	Thermotogae	Thermotogae	Kosmotogales	Kosmotogaceae	Mesotoga	CRISPR-associated protein Cas1	cas1	cd09722	CAS-I-B			
4625..5120	-	PZC51856.1	AYSI01000129.1	Mesotoga sp. TolDC	Bacteria	Thermotogae	Thermotogae	Kosmotogales	Kosmotogaceae	Mesotoga	CRISPR-associated protein Cas4	cas4	pfam01930	CAS-I			
5119..7456	-	PZC51857.1	AYSI01000129.1	Mesotoga sp. TolDC	Bacteria	Thermotogae	Thermotogae	Kosmotogales	Kosmotogaceae	Mesotoga	hypothetical protein	cas3HD,cas3	cd09641,COG1203	CAS-I			
8164..9115	-	PZC51858.1	AYSI01000129.1	Mesotoga sp. TolDC	Bacteria	Thermotogae	Thermotogae	Kosmotogales	Kosmotogaceae	Mesotoga	CRISPR-associated protein Csh2	cas7	cd09690	CAS-I-B			
9117..10650	-	PZC51859.1	AYSI01000129.1	Mesotoga sp. TolDC	Bacteria	Thermotogae	Thermotogae	Kosmotogales	Kosmotogaceae	Mesotoga	hypothetical protein	cas8b1	cd09730	CAS-I-B			

318 CAS-I-B | OKY78761.1

1217182..1219687	-	OKY78756.1	MSDW01000001.1	Candidatus Methanohalarchaeum thermophilum	Archaea	Euryarchaeota	Methanonatronarchaeia	Methanonatronarchaeales	Methanonatronarchaeaceae	Candidatus Methanohalarchaeum	CRISPR-associated helicase Cas3	cas3HD,cas3	cd09641,cd09639	CAS-I			
1219673..1220405	-	OKY78757.1	MSDW01000001.1	Candidatus Methanohalarchaeum thermophilum	Archaea	Euryarchaeota	Methanonatronarchaeia	Methanonatronarchaeales	Methanonatronarchaeaceae	Candidatus Methanohalarchaeum	CRISPR-Cas system related protein Cas5, RAMP superfamily	cas5	cd09692	CAS-I-B			
1220406..1221369	-	OKY78758.1	MSDW01000001.1	Candidatus Methanohalarchaeum thermophilum	Archaea	Euryarchaeota	Methanonatronarchaeia	Methanonatronarchaeales	Methanonatronarchaeaceae	Candidatus Methanohalarchaeum	CRISPR-Cas system related protein Cas7, RAMP superfamily	cas7	cd09690	CAS-I-B			
1221361..1223251	-	OKY78759.1	MSDW01000001.1	Candidatus Methanohalarchaeum thermophilum	Archaea	Euryarchaeota	Methanonatronarchaeia	Methanonatronarchaeales	Methanonatronarchaeaceae	Candidatus Methanohalarchaeum	CRISPR-associated protein, large subunit of CASCADE complex, Cas8b1	cas8b1	pfam09484	CAS-I-B			
1223265..1224006	-	OKY78760.1	MSDW01000001.1	Candidatus Methanohalarchaeum thermophilum	Archaea	Euryarchaeota	Methanonatronarchaeia	Methanonatronarchaeales	Methanonatronarchaeaceae	Candidatus Methanohalarchaeum	CRISPR-Cas system related protein Cas6, RAMP superfamily	cas6	cd09759	CAS-I-A			
1224089..1225472	array		MSDW01000001.1	Candidatus Methanohalarchaeum thermophilum	Archaea	Euryarchaeota	Methanonatronarchaeia	Methanonatronarchaeales	Methanonatronarchaeaceae	Candidatus Methanohalarchaeum		CRISPR array			21	37	30
1226296..1226560	-	OKY78761.1	MSDW01000001.1	Candidatus Methanohalarchaeum thermophilum	Archaea	Euryarchaeota	Methanonatronarchaeia	Methanonatronarchaeales	Methanonatronarchaeaceae	Candidatus Methanohalarchaeum	CRISPR-associated protein Cas2	**cas2**	COG1343	CAS-I,CAS-II,CAS-III			
1226621..1227542	-	OKY78762.1	MSDW01000001.1	Candidatus Methanohalarchaeum thermophilum	Archaea	Euryarchaeota	Methanonatronarchaeia	Methanonatronarchaeales	Methanonatronarchaeaceae	Candidatus Methanohalarchaeum	CRISPR-associated protein Cas1	cas1	cd09722	CAS-I-B			
1227610..1228198	-	OKY78763.1	MSDW01000001.1	Candidatus Methanohalarchaeum thermophilum	Archaea	Euryarchaeota	Methanonatronarchaeia	Methanonatronarchaeales	Methanonatronarchaeaceae	Candidatus Methanohalarchaeum	CRISPR-associated protein Cas4, RecB family nuclease	cas4	pfam01930	CAS-I			

319 CAS-I | EYE89395.1

73377..74130	+	EYE89391.1	AZQP01000005.1	Fervidicella metallireducens AeB	Bacteria	Firmicutes	Clostridia	Clostridiales	Clostridiaceae	Fervidicella	CRISPR-associated protein Cas6	cas6	COG1583	CAS-I,CAS-III,CAS-IV			
74174..76613	+	EYE89416.1	AZQP01000005.1	Fervidicella metallireducens AeB	Bacteria	Firmicutes	Clostridia	Clostridiales	Clostridiaceae	Fervidicella	hypothetical protein	cas3,cas3HD	COG1203,cd09641	CAS-I			
76622..78464	+	EYE89417.1	AZQP01000005.1	Fervidicella metallireducens AeB	Bacteria	Firmicutes	Clostridia	Clostridiales	Clostridiaceae	Fervidicella	hypothetical protein	cas8a4	cls000706	CAS-I-A			

78486..79443	+	EYE89392.1	AZQP01000005.1	Fervidicella metallireducens AeB	Bacteria	Firmicutes	Clostridia	Clostridiales	Clostridiaceae	Fervidicella	CRISPR-associated protein DevR	cas7	pfam01905	CAS-I			
79479..80106	+	EYE89418.1	AZQP01000005.1	Fervidicella metallireducens AeB	Bacteria	Firmicutes	Clostridia	Clostridiales	Clostridiaceae	Fervidicella	hypothetical protein	cas5	cls000048	CAS-I-A			
80288..80798	+	EYE89393.1	AZQP01000005.1	Fervidicella metallireducens AeB	Bacteria	Firmicutes	Clostridia	Clostridiales	Clostridiaceae	Fervidicella	hypothetical protein	cas4	pfam01930	CAS-I			
80809..81799	+	EYE89394.1	AZQP01000005.1	Fervidicella metallireducens AeB	Bacteria	Firmicutes	Clostridia	Clostridiales	Clostridiaceae	Fervidicella	CRISPR-associated protein Cas1	cas1	cd09722	CAS-I-B			
81804..82068	+	EYE89395.1	AZQP01000005.1	Fervidicella metallireducens AeB	Bacteria	Firmicutes	Clostridia	Clostridiales	Clostridiaceae	Fervidicella	CRISPR-associated protein Cas2	cas2	COG1343	CAS-I,CAS-II,CAS-III			
82274..84958	array		AZQP01000005.1	Fervidicella metallireducens AeB	Bacteria	Firmicutes	Clostridia	Clostridiales	Clostridiaceae	Fervidicella		CRISPR array			41	36	30
320 CAS-I-B | SHE32866.1																	
56958..57517	array		FQVH01000001.1	Caldanaerobius fijiensis DSM 17918	Bacteria	Firmicutes	Clostridia	Thermoanaerobacterales	Thermoanaerobacteraceae	Caldanaerobius		CRISPR array			9	36	30
58613..58775	array		FQVH01000001.1	Caldanaerobius fijiensis DSM 17918	Bacteria	Firmicutes	Clostridia	Thermoanaerobacterales	Thermoanaerobacteraceae	Caldanaerobius		CRISPR array			3	36	30
58930..59917	-	SHE32555.1	FQVH01000001.1	Caldanaerobius fijiensis DSM 17918	Bacteria	Firmicutes	Clostridia	Thermoanaerobacterales	Thermoanaerobacteraceae	Caldanaerobius	CRISPR-associated protein, Cas1 family	cas1	cd09722	CAS-I-B			
59926..60436	-	SHE32610.1	FQVH01000001.1	Caldanaerobius fijiensis DSM 17918	Bacteria	Firmicutes	Clostridia	Thermoanaerobacterales	Thermoanaerobacteraceae	Caldanaerobius	CRISPR-associated exonuclease Cas4	cas4	pfam01930	CAS-I			
60419..62867	-	SHE32663.1	FQVH01000001.1	Caldanaerobius fijiensis DSM 17918	Bacteria	Firmicutes	Clostridia	Thermoanaerobacterales	Thermoanaerobacteraceae	Caldanaerobius	CRISPR-associated endonuclease/helicase Cas3	cas3HD,cas3	cd09641,cd09639	CAS-I			
62877..63528	-	SHE32717.1	FQVH01000001.1	Caldanaerobius fijiensis DSM 17918	Bacteria	Firmicutes	Clostridia	Thermoanaerobacterales	Thermoanaerobacteraceae	Caldanaerobius	CRISPR-associated protein Cas5h	cas5	cd09658	CAS-I-B			
63532..64468	-	SHE32761.1	FQVH01000001.1	Caldanaerobius fijiensis DSM 17918	Bacteria	Firmicutes	Clostridia	Thermoanaerobacterales	Thermoanaerobacteraceae	Caldanaerobius	CRISPR-associated protein Cst2	cas7	cd09687	CAS-I-B			
64481..66053	-	SHE32816.1	FQVH01000001.1	Caldanaerobius fijiensis DSM 17918	Bacteria	Firmicutes	Clostridia	Thermoanaerobacterales	Thermoanaerobacteraceae	Caldanaerobius	hypothetical protein	cas8b1	mkCas0209	CAS-I-B			
66121..66379	-	SHE32866.1	FQVH01000001.1	Caldanaerobius fijiensis DSM 17918	Bacteria	Firmicutes	Clostridia	Thermoanaerobacterales	Thermoanaerobacteraceae	Caldanaerobius	CRISPR-associated protein Cas2	cas2	cd09725	CAS-I,CAS-II,CAS-III,CAS-V			
66362..67157	-	SHE32912.1	FQVH01000001.1	Caldanaerobius fijiensis DSM 17918	Bacteria	Firmicutes	Clostridia	Thermoanaerobacterales	Thermoanaerobacteraceae	Caldanaerobius	CRISPR-associated endoribonuclease Cas6	cas6	COG1583	CAS-I,CAS-III,CAS-IV			
67206..68436	-	SHE32962.1	FQVH01000001.1	Caldanaerobius fijiensis DSM 17918	Bacteria	Firmicutes	Clostridia	Thermoanaerobacterales	Thermoanaerobacteraceae	Caldanaerobius	cell division protein DivIC	csx1	cd09732	CAS-III			
68722..70342	array		FQVH01000001.1	Caldanaerobius fijiensis DSM 17918	Bacteria	Firmicutes	Clostridia	Thermoanaerobacterales	Thermoanaerobacteraceae	Caldanaerobius		CRISPR array			25	36	30
321 partial CAS-I | OGF54888.1																	
2..413	+	OGF54886.1	MFGX01000070.1	Candidatus Fraserbacteria bacterium RBG_16_55_9	Bacteria	Candidatus Fraserbacteria					CRISPR-associated protein Cas4	cas4	pfam01930	CAS-I			
1483..1690	+	OGF54887.1	MFGX01000070.1	Candidatus Fraserbacteria bacterium RBG_16_55_9	Bacteria	Candidatus Fraserbacteria					hypothetical protein						
1927..2191	+	OGF54888.1	MFGX01000070.1	Candidatus Fraserbacteria bacterium RBG_16_55_9	Bacteria	Candidatus Fraserbacteria					CRISPR-associated endonuclease Cas2	cas2	cd09725	CAS-I,CAS-II,CAS-III,CAS-V			
322 CAS-I-B | KXG77807.1																	
14241..15278	array		LOED01000007.1	Fervidicola ferrireducens	Bacteria	Firmicutes	Clostridia	Thermoanaerobacterales	Thermoanaerobacteraceae	Fervidicola		CRISPR array			16	37	30
15377..17570	-	KXG77802.1	LOED01000007.1	Fervidicola ferrireducens	Bacteria	Firmicutes	Clostridia	Thermoanaerobacterales	Thermoanaerobacteraceae	Fervidicola	CRISPR-associated nuclease/helicase Cas3	cas3HD,cas3	cd09641,cd09639	CAS-I			
17554..18271	-	KXG77803.1	LOED01000007.1	Fervidicola ferrireducens	Bacteria	Firmicutes	Clostridia	Thermoanaerobacterales	Thermoanaerobacteraceae	Fervidicola	hypothetical protein	cas5	cd09693	CAS-I			
18249..19179	-	KXG77804.1	LOED01000007.1	Fervidicola ferrireducens	Bacteria	Firmicutes	Clostridia	Thermoanaerobacterales	Thermoanaerobacteraceae	Fervidicola	hypothetical protein	cas7	cd09687	CAS-I-B			
19171..20683	-	KXG77805.1	LOED01000007.1	Fervidicola ferrireducens	Bacteria	Firmicutes	Clostridia	Thermoanaerobacterales	Thermoanaerobacteraceae	Fervidicola	hypothetical protein	cas8b1	mkCas0195	CAS-I-B			
20697..21441	-	KXG77806.1	LOED01000007.1	Fervidicola ferrireducens	Bacteria	Firmicutes	Clostridia	Thermoanaerobacterales	Thermoanaerobacteraceae	Fervidicola	hypothetical protein	cas6	COG1583	CAS-I,CAS-III,CAS-IV			
21451..21718	-	KXG77807.1	LOED01000007.1	Fervidicola ferrireducens	Bacteria	Firmicutes	Clostridia	Thermoanaerobacterales	Thermoanaerobacteraceae	Fervidicola	CRISPR-associated endoribonuclease Cas2	cas2	cd09725	CAS-I,CAS-II,CAS-III,CAS-V			
21723..22593	-	KXG77808.1	LOED01000007.1	Fervidicola ferrireducens	Bacteria	Firmicutes	Clostridia	Thermoanaerobacterales	Thermoanaerobacteraceae	Fervidicola	CRISPR-associated endonuclease Cas1	cas1	cd09722	CAS-I-B			
323 CAS-I-B | ADI02106.1																	
1385517..1395709	array		CP002048.1	Syntrophothermus lipocalidus DSM 12680	Bacteria	Firmicutes	Clostridia	Clostridiales	Syntrophomonadaceae	Syntrophothermus		CRISPR array			154	36	30
1395922..1396699	-	ADI02104.1	CP002048.1	Syntrophothermus lipocalidus DSM 12680	Bacteria	Firmicutes	Clostridia	Clostridiales	Syntrophomonadaceae	Syntrophothermus	CRISPR-associated protein Cas6	cas6	COG1583	CAS-I,CAS-III,CAS-IV			
1396695..1396893	-	ADI02105.1	CP002048.1	Syntrophothermus lipocalidus DSM 12680	Bacteria	Firmicutes	Clostridia	Clostridiales	Syntrophomonadaceae	Syntrophothermus	hypothetical protein						
1396917..1397184	-	ADI02106.1	CP002048.1	Syntrophothermus lipocalidus DSM 12680	Bacteria	Firmicutes	Clostridia	Clostridiales	Syntrophomonadaceae	Syntrophothermus	CRISPR-associated protein Cas2	cas2	COG1343	CAS-I,CAS-II,CAS-III			
1397189..1398200	-	ADI02107.1	CP002048.1	Syntrophothermus lipocalidus DSM 12680	Bacteria	Firmicutes	Clostridia	Clostridiales	Syntrophomonadaceae	Syntrophothermus	CRISPR-associated protein Cas1	cas1	cd09722	CAS-I-B			
1398228..1398768	-	ADI02108.1	CP002048.1	Syntrophothermus lipocalidus DSM 12680	Bacteria	Firmicutes	Clostridia	Clostridiales	Syntrophomonadaceae	Syntrophothermus	CRISPR-associated protein Cas4	cas4	pfam01930	CAS-I			
1398748..1401031	-	ADI02109.1	CP002048.1	Syntrophothermus lipocalidus DSM 12680	Bacteria	Firmicutes	Clostridia	Clostridiales	Syntrophomonadaceae	Syntrophothermus	CRISPR-associated helicase Cas3	cas3HD,cas3	cd09641,COG1203	CAS-I			
1401064..1401793	-	ADI02110.1	CP002048.1	Syntrophothermus lipocalidus DSM 12680	Bacteria	Firmicutes	Clostridia	Clostridiales	Syntrophomonadaceae	Syntrophothermus	CRISPR-associated protein Cas5 family	cas5	cd09693	CAS-I			
1401789..1402881	-	ADI02111.1	CP002048.1	Syntrophothermus lipocalidus DSM 12680	Bacteria	Firmicutes	Clostridia	Clostridiales	Syntrophomonadaceae	Syntrophothermus	CRISPR-associated regulatory protein, DevR family	cas7	cd09687	CAS-I-B			
1402896..1404660	-	ADI02112.1	CP002048.1	Syntrophothermus lipocalidus DSM 12680	Bacteria	Firmicutes	Clostridia	Clostridiales	Syntrophomonadaceae	Syntrophothermus	CRISPR-associated CXXC_CXXC protein Cst1	cas8b2	cls000029	CAS-I-B			
324 CAS-I-A | GBC98668.1																	
74908..75175	-	GBC98668.1	BEHT01000013.1	bacterium HR17	Bacteria						CRISPR-associated endoribonuclease Cas2	cas2	cd09725	CAS-I,CAS-II,CAS-III,CAS-V			
75193..76066	-	GBC98669.1	BEHT01000013.1	bacterium HR17	Bacteria						hypothetical protein	cas5	cls000048	CAS-I-A			
76100..77156	-	GBC98670.1	BEHT01000013.1	bacterium HR17	Bacteria						hypothetical protein	cas7	pfam01905	CAS-I			
77155..77560	-	GBC98671.1	BEHT01000013.1	bacterium HR17	Bacteria						hypothetical protein	csa5gr11	mkCas0080	CAS-I-A			
77531..78419	-	GBC98672.1	BEHT01000013.1	bacterium HR17	Bacteria						hypothetical protein	cas8a2	mkCas0113	CAS-I-A			
325 CAS-I | ODS31541.1																	
3948..4272	+	ODS31529.1	MAYW01000111.1	Candidatus Scalindua rubra	Bacteria	Planctomycetes	Candidatus Brocadiae	Candidatus Brocadiales	Candidatus Brocadiaceae	Candidatus Scalindua	CRISPR-associated protein	cas1	cd09636	CAS-I,CAS-II,CAS-III			
4361..5210	+	ODS31530.1	MAYW01000111.1	Candidatus Scalindua rubra	Bacteria	Planctomycetes	Candidatus Brocadiae	Candidatus Brocadiales	Candidatus Brocadiaceae	Candidatus Scalindua	CRISPR-associated protein	cas6	COG1583	CAS-I,CAS-III,CAS-IV			
5291..5471	+	ODS31531.1	MAYW01000111.1	Candidatus Scalindua rubra	Bacteria	Planctomycetes	Candidatus Brocadiae	Candidatus Brocadiales	Candidatus Brocadiaceae	Candidatus Scalindua	hypothetical protein						
5632..8131	+	ODS31532.1	MAYW01000111.1	Candidatus Scalindua rubra	Bacteria	Planctomycetes	Candidatus Brocadiae	Candidatus Brocadiales	Candidatus Brocadiaceae	Candidatus Scalindua	hypothetical protein	cas3,cas3HD	COG1203,cd09641	CAS-I			
8140..9922	+	ODS31533.1	MAYW01000111.1	Candidatus Scalindua rubra	Bacteria	Planctomycetes	Candidatus Brocadiae	Candidatus Brocadiales	Candidatus Brocadiaceae	Candidatus Scalindua	hypothetical protein	cas8a4	cls000706	CAS-I-A			
9914..11141	+	ODS31534.1	MAYW01000111.1	Candidatus Scalindua rubra	Bacteria	Planctomycetes	Candidatus Brocadiae	Candidatus Brocadiales	Candidatus Brocadiaceae	Candidatus Scalindua	hypothetical protein	cas7,cas7	pfam01905,pfam01905	CAS-I			
11152..11851	+	ODS31535.1	MAYW01000111.1	Candidatus Scalindua rubra	Bacteria	Planctomycetes	Candidatus Brocadiae	Candidatus Brocadiales	Candidatus Brocadiaceae	Candidatus Scalindua	hypothetical protein	cas5	cls000048	CAS-I-A			
11831..11960	+	ODS31536.1	MAYW01000111.1	Candidatus Scalindua rubra	Bacteria	Planctomycetes	Candidatus Brocadiae	Candidatus Brocadiales	Candidatus Brocadiaceae	Candidatus Scalindua	hypothetical protein						
12127..12517	-	ODS31537.1	MAYW01000111.1	Candidatus Scalindua rubra	Bacteria	Planctomycetes	Candidatus Brocadiae	Candidatus Brocadiales	Candidatus Brocadiaceae	Candidatus Scalindua	prophage maintenance system killer protein						
12516..12762	-	ODS31538.1	MAYW01000111.1	Candidatus Scalindua rubra	Bacteria	Planctomycetes	Candidatus Brocadiae	Candidatus Brocadiales	Candidatus Brocadiaceae	Candidatus Scalindua	SpoVT / AbrB like domain protein						
13069..13579	+	ODS31539.1	MAYW01000111.1	Candidatus Scalindua rubra	Bacteria	Planctomycetes	Candidatus Brocadiae	Candidatus Brocadiales	Candidatus Brocadiaceae	Candidatus Scalindua	hypothetical protein	cas4	pfam01930	CAS-I			
13717..14710	+	ODS31540.1	MAYW01000111.1	Candidatus Scalindua rubra	Bacteria	Planctomycetes	Candidatus Brocadiae	Candidatus Brocadiales	Candidatus Brocadiaceae	Candidatus Scalindua	CRISPR-associated protein	cas1	cd09722	CAS-I-B			
14763..15027	+	ODS31541.1	MAYW01000111.1	Candidatus Scalindua rubra	Bacteria	Planctomycetes	Candidatus Brocadiae	Candidatus Brocadiales	Candidatus Brocadiaceae	Candidatus Scalindua	CRISPR-associated protein Cas2	cas2	cd09725	CAS-I,CAS-II,CAS-III,CAS-V			
15174..15464	array		MAYW01000111.1	Candidatus Scalindua rubra	Bacteria	Planctomycetes	Candidatus Brocadiae	Candidatus Brocadiales	Candidatus Brocadiaceae	Candidatus Scalindua		CRISPR array			5	35	30
326 CAS-I-B | RPI19238.1																	
62054..62906	+	RPI19231.1	RPQH01000007.1	Ignavibacteriae bacterium	Bacteria	Ignavibacteriae					CRISPR-associated endoribonuclease Cas6	cas6	COG1583	CAS-I,CAS-III,CAS-IV			
63005..64838	+	RPI19232.1	RPQH01000007.1	Ignavibacteriae bacterium	Bacteria	Ignavibacteriae					hypothetical protein	cas8b1	mkCas0106	CAS-I-B			
64827..65856	+	RPI19233.1	RPQH01000007.1	Ignavibacteriae bacterium	Bacteria	Ignavibacteriae					CRISPR-associated protein	cas7b	pfam05107	CAS-I-B,CAS-I-C			
65870..66671	+	RPI19234.1	RPQH01000007.1	Ignavibacteriae bacterium	Bacteria	Ignavibacteriae					type I-B CRISPR-associated protein Cas5	cas5	cls001430	CAS-I-B			
66695..69368	+	RPI19235.1	RPQH01000007.1	Ignavibacteriae bacterium	Bacteria	Ignavibacteriae					CRISPR-associated helicase Cas3'	cas3HD,cas3	cd09641,cd09639	CAS-I			
69374..69893	+	RPI19236.1	RPQH01000007.1	Ignavibacteriae bacterium	Bacteria	Ignavibacteriae					CRISPR-associated protein Cas4	cas4	pfam01930	CAS-I			
69894..71028	+	RPI19237.1	RPQH01000007.1	Ignavibacteriae bacterium	Bacteria	Ignavibacteriae					type I-B CRISPR-associated endonuclease Cas1	cas1	cd09722	CAS-I-B			
71034..71301	+	RPI19238.1	RPQH01000007.1	Ignavibacteriae bacterium	Bacteria	Ignavibacteriae					CRISPR-associated endonuclease Cas2	cas2	cd09725	CAS-I,CAS-II,CAS-III,CAS-V			
71407..78525	array		RPQH01000007.1	Ignavibacteriae bacterium	Bacteria	Ignavibacteriae						CRISPR array			110	36	29
327 CAS-I-B | CUU01221.1																	
233893..236173	array		FAOP01000001.1	Candidatus Kryptonium thompsoni	Bacteria	Candidatus Kryptonia				Candidatus Kryptonium		CRISPR array			35	36	30
236352..236613	-	CUU01221.1	FAOP01000001.1	Candidatus Kryptonium thompsoni	Bacteria	Candidatus Kryptonia				Candidatus Kryptonium	CRISPR-associated protein Cas2	cas2	cd09725	CAS-I,CAS-II,CAS-III,CAS-V			
236624..237659	-	CUU01226.1	FAOP01000001.1	Candidatus Kryptonium thompsoni	Bacteria	Candidatus Kryptonia				Candidatus Kryptonium	CRISPR-associated protein, Cas1 family	cas1	cd09722	CAS-I-B			
237661..238171	-	CUU01230.1	FAOP01000001.1	Candidatus Kryptonium thompsoni	Bacteria	Candidatus Kryptonia				Candidatus Kryptonium	CRISPR-associated exonuclease Cas4	cas4	pfam01930	CAS-I			
238200..240420	-	CUU01236.1	FAOP01000001.1	Candidatus Kryptonium thompsoni	Bacteria	Candidatus Kryptonia				Candidatus Kryptonium	CRISPR-associated endonuclease/helicase Cas3	cas3HD,cas3	cd09641,COG1203	CAS-I			
240404..241139	-	CUU01239.1	FAOP01000001.1	Candidatus Kryptonium thompsoni	Bacteria	Candidatus Kryptonia				Candidatus Kryptonium	CRISPR-associated protein Cas5t	cas5	cd09658	CAS-I-B			
241149..242058	-	CUU01243.1	FAOP01000001.1	Candidatus Kryptonium thompsoni	Bacteria	Candidatus Kryptonia				Candidatus Kryptonium	CRISPR-associated protein Cst2	cas7	cd09687	CAS-I-B			
242054..243821	-	CUU01246.1	FAOP01000001.1	Candidatus Kryptonium thompsoni	Bacteria	Candidatus Kryptonia				Candidatus Kryptonium	hypothetical protein	Cas11b	icity0129				
243843..244587	-	CUU01249.1	FAOP01000001.1	Candidatus Kryptonium thompsoni	Bacteria	Candidatus Kryptonia				Candidatus Kryptonium	CRISPR-associated endoribonuclease Cas6	cas6	COG1583	CAS-I,CAS-III,CAS-IV			
328 Partial CAS-I | CUT04207.1																	
12691..13777	array		CZVW01000021.1	Candidatus Chrysopegis kryptomonas	Bacteria	Candidatus Kryptonia				Candidatus Chrysopegis		CRISPR array			17	36	30
13923..15111	+	CUT04172.1	CZVW01000021.1	Candidatus Chrysopegis kryptomonas	Bacteria	Candidatus Kryptonia				Candidatus Chrysopegis	CRISPR-associated protein Cst1	cas8b2	pfam09706	CAS-I-B			
15101..15623	+	CUT04175.1	CZVW01000021.1	Candidatus Chrysopegis kryptomonas	Bacteria	Candidatus Kryptonia				Candidatus Chrysopegis	hypothetical protein	Cas11b	icity0129				
15615..16764	+	CUT04180.1	CZVW01000021.1	Candidatus Chrysopegis kryptomonas	Bacteria	Candidatus Kryptonia				Candidatus Chrysopegis	CRISPR-associated autoregulator, Cst2 family	cas7	pfam01905	CAS-I			
16788..17070	+	CUT04185.1	CZVW01000021.1	Candidatus Chrysopegis kryptomonas	Bacteria	Candidatus Kryptonia				Candidatus Chrysopegis	hypothetical protein						
17042..17597	+	CUT04187.1	CZVW01000021.1	Candidatus Chrysopegis kryptomonas	Bacteria	Candidatus Kryptonia				Candidatus Chrysopegis	hypothetical protein	C3a174	cluster174				
17581..17878	+	CUT04192.1	CZVW01000021.1	Candidatus Chrysopegis kryptomonas	Bacteria	Candidatus Kryptonia				Candidatus Chrysopegis	hypothetical protein						
17846..18407	+	CUT04194.1	CZVW01000021.1	Candidatus Chrysopegis kryptomonas	Bacteria	Candidatus Kryptonia				Candidatus Chrysopegis	Nucleotidyl transferase AbiEii toxin, Type IV TA system	C3a174	cluster174				
18408..19212	+	CUT04199.1	CZVW01000021.1	Candidatus Chrysopegis kryptomonas	Bacteria	Candidatus Kryptonia				Candidatus Chrysopegis	CRISPR-associated protein, Cas5t family	cas5	cd09658	CAS-I-B			
19215..21897	+	CUT04204.1	CZVW01000021.1	Candidatus Chrysopegis kryptomonas	Bacteria	Candidatus Kryptonia				Candidatus Chrysopegis	CRISPR-associated endonuclease/helicase Cas3	cas3HD,cas3	cd09641,COG1203	CAS-I			
22018..22279	+	CUT04207.1	CZVW01000021.1	Candidatus Chrysopegis kryptomonas	Bacteria	Candidatus Kryptonia				Candidatus Chrysopegis	CRISPR-associated protein, Cas2 family	cas2	cd09725	CAS-I,CAS-II,CAS-III,CAS-V			
22457..22615	array		CZVW01000021.1	Candidatus Chrysopegis kryptomonas	Bacteria	Candidatus Kryptonia				Candidatus Chrysopegis		CRISPR array			3	39	27
329 CAS-I-B | CUU07896.1																	
114488..115793	+	CUU07871.1	FAOP01000007.1	Candidatus Kryptonium thompsoni	Bacteria	Candidatus Kryptonia				Candidatus Kryptonium	CRISPR-associated protein, Cst1 family	cas8b2	cd09665	CAS-I-B			
115828..116860	+	CUU07872.1	FAOP01000007.1	Candidatus Kryptonium thompsoni	Bacteria	Candidatus Kryptonia				Candidatus Kryptonium	CRISPR-associated autoregulator, Cst2 family	cas7	cd09687	CAS-I-B			
116916..117555	+	CUU07876.1	FAOP01000007.1	Candidatus Kryptonium thompsoni	Bacteria	Candidatus Kryptonia				Candidatus Kryptonium	CRISPR-associated protein, Cas5t family	cas5	COG1688	CAS-I			
117544..118009	+	CUU07880.1	FAOP01000007.1	Candidatus Kryptonium thompsoni	Bacteria	Candidatus Kryptonia				Candidatus Kryptonium	hypothetical protein						
118010..120245	+	CUU07884.1	FAOP01000007.1	Candidatus Kryptonium thompsoni	Bacteria	Candidatus Kryptonia				Candidatus Kryptonium	CRISPR-associated endonuclease/helicase Cas3	cas3	COG1203	CAS-I			
120245..120749	+	CUU07886.1	FAOP01000007.1	Candidatus Kryptonium thompsoni	Bacteria	Candidatus Kryptonia				Candidatus Kryptonium	CRISPR-associated exonuclease, Cas4 family	cas4	pfam01930	CAS-I			
120771..121842	+	CUU07892.1	FAOP01000007.1	Candidatus Kryptonium thompsoni	Bacteria	Candidatus Kryptonia				Candidatus Kryptonium	CRISPR-associated protein, Cas1 family	cas1	cd09722	CAS-I-B			
121863..122124	+	CUU07896.1	FAOP01000007.1	Candidatus Kryptonium thompsoni	Bacteria	Candidatus Kryptonia				Candidatus Kryptonium	CRISPR-associated protein Cas2	cas2	cd09725	CAS-I,CAS-II,CAS-III,CAS-V			
122410..128747	array		FAOP01000007.1	Candidatus Kryptonium thompsoni	Bacteria	Candidatus Kryptonia				Candidatus Kryptonium		CRISPR array			96	36	30
330 CAS-III-C | PKP54661.1																	
5..300	array		PHDW01000002.1	Candidatus Altiarchaeales archaeon HGW-Altiarchaeales-3	Archaea	Euryarchaeota		Candidatus Altiarchaeales				CRISPR array			5	36	30
442..706	-	PKP54661.1	PHDW01000002.1	Candidatus Altiarchaeales archaeon HGW-Altiarchaeales-3	Archaea	Euryarchaeota		Candidatus Altiarchaeales			CRISPR-associated endonuclease Cas2	cas2	cd09725	CAS-I,CAS-II,CAS-III,CAS-V			
683..1622	-	PKP54662.1	PHDW01000002.1	Candidatus Altiarchaeales archaeon HGW-Altiarchaeales-3	Archaea	Euryarchaeota		Candidatus Altiarchaeales			hypothetical protein	cmr3gr5	mkCas0067	CAS-III-C			
1602..1992	-	PKP54663.1	PHDW01000002.1	Candidatus Altiarchaeales archaeon HGW-Altiarchaeales-3	Archaea	Euryarchaeota		Candidatus Altiarchaeales			HEPN domain-containing protein						
1984..2293	-	PKP54664.1	PHDW01000002.1	Candidatus Altiarchaeales archaeon HGW-Altiarchaeales-3	Archaea	Euryarchaeota		Candidatus Altiarchaeales			nucleotidyltransferase domain-containing protein						
2289..2670	-	PKP54665.1	PHDW01000002.1	Candidatus Altiarchaeales archaeon HGW-Altiarchaeales-3	Archaea	Euryarchaeota		Candidatus Altiarchaeales			hypothetical protein	cmr5gr11	mkCas0060	CAS-III-C			
2659..3592	-	PKP54666.1	PHDW01000002.1	Candidatus Altiarchaeales archaeon HGW-Altiarchaeales-3	Archaea	Euryarchaeota		Candidatus Altiarchaeales			type III-B CRISPR module RAMP protein Cmr4	cmr4gr7	COG1336	CAS-III-C			
3584..4337	-	PKP54667.1	PHDW01000002.1	Candidatus Altiarchaeales archaeon HGW-Altiarchaeales-3	Archaea	Euryarchaeota		Candidatus Altiarchaeales			hypothetical protein	cas10	cd09701	CAS-III-C			
4489..7948	-	PKP54668.1	PHDW01000002.1	Candidatus Altiarchaeales archaeon HGW-Altiarchaeales-3	Archaea	Euryarchaeota		Candidatus Altiarchaeales			hypothetical protein	cas10,cas10	cd09701,cd09701	CAS-III-C			
7949..8774	-	PKP54669.1	PHDW01000002.1	Candidatus Altiarchaeales archaeon HGW-Altiarchaeales-3	Archaea	Euryarchaeota		Candidatus Altiarchaeales			hypothetical protein						
8790..9696	-	PKP54670.1	PHDW01000002.1	Candidatus Altiarchaeales archaeon HGW-Altiarchaeales-3	Archaea	Euryarchaeota		Candidatus Altiarchaeales			hypothetical protein	cmr6gr7	mkCas0142	CAS-III-C			
9685..10603	-	PKP54671.1	PHDW01000002.1	Candidatus Altiarchaeales archaeon HGW-Altiarchaeales-3	Archaea	Euryarchaeota		Candidatus Altiarchaeales			type III-B CRISPR module RAMP protein Cmr1	cmr1gr7	cd09657	CAS-III-C			
331 partial CAS-I-B | RMF70322.1																	
137..1229	+	RMF70321.1	RFIG01000023.1	Calditrichaeota bacterium	Bacteria	Calditrichaeota					type I-B CRISPR-associated endonuclease Cas1	cas1	cd09722	CAS-I-B			
1252..1516	+	RMF70322.1	RFIG01000023.1	Calditrichaeota bacterium	Bacteria	Calditrichaeota					CRISPR-associated endonuclease Cas2	cas2	cd09725	CAS-I,CAS-II,CAS-III,CAS-V			
332 partial CAS-I-B | ACF14797.1																	
2813147..2819595	array		CP001100.1	Chloroherpeton thalassium ATCC 35110	Bacteria	Chlorobi	Chlorobia	Chlorobiales	Chlorobiaceae	Chloroherpeton		CRISPR array			98	36	30
2819812..2820340	-	ACF14796.1	CP001100.1	Chloroherpeton thalassium ATCC 35110	Bacteria	Chlorobi	Chlorobia	Chlorobiales	Chlorobiaceae	Chloroherpeton	CRISPR-associated protein Cas4	cas4	pfam01930	CAS-I			
2820343..2820631	-	ACF14797.1	CP001100.1	Chloroherpeton thalassium ATCC 35110	Bacteria	Chlorobi	Chlorobia	Chlorobiales	Chlorobiaceae	Chloroherpeton	CRISPR-associated protein Cas2	cas2	cd09725	CAS-I,CAS-II,CAS-III,CAS-V			
2820637..2821792	-	ACF14798.1	CP001100.1	Chloroherpeton thalassium ATCC 35110	Bacteria	Chlorobi	Chlorobia	Chlorobiales	Chlorobiaceae	Chloroherpeton	CRISPR-associated protein Cas1	cas1	cd09722	CAS-I-B			
2821834..2822638	-	ACF14799.1	CP001100.1	Chloroherpeton thalassium ATCC 35110	Bacteria	Chlorobi	Chlorobia	Chlorobiales	Chlorobiaceae	Chloroherpeton	CRISPR-associated protein Cas6	cas6	COG1583	CAS-I,CAS-III,CAS-IV			
333 CAS-I-B | PKM49939.1																	
50814..53078	array		PGZO01000024.1	Firmicutes bacterium HGW-Firmicutes-7	Bacteria	Firmicutes						CRISPR array			35	35	30
53179..53464	-	PKM49939.1	PGZO01000024.1	Firmicutes bacterium HGW-Firmicutes-7	Bacteria	Firmicutes					CRISPR-associated endonuclease Cas2	cas2	COG1343	CAS-I,CAS-II,CAS-III			
53470..54463	-	PKM49940.1	PGZO01000024.1	Firmicutes bacterium HGW-Firmicutes-7	Bacteria	Firmicutes					subtype I-B CRISPR-associated endonuclease Cas1	cas1	cd09722	CAS-I-B			
54506..55010	-	PKM49941.1	PGZO01000024.1	Firmicutes bacterium HGW-Firmicutes-7	Bacteria	Firmicutes					CRISPR-associated protein Cas4	cas4	pfam01930	CAS-I			
55110..57309	-	PKM49942.1	PGZO01000024.1	Firmicutes bacterium HGW-Firmicutes-7	Bacteria	Firmicutes					CRISPR-associated helicase/endonuclease Cas3	cas3	COG1203	CAS-I			
57317..58070	-	PKM49943.1	PGZO01000024.1	Firmicutes bacterium HGW-Firmicutes-7	Bacteria	Firmicutes					type I-B CRISPR-associated protein Cas5	cas5	cd09658	CAS-I-B			

58056..58926	-	PKM49944.1	PGZO01000024.1	Firmicutes bacterium HGW-Firmicutes-7	Bacteria	Firmicutes					type I-B CRISPR-associated protein Cas7/Cst2/DevR	cas7	cd09687	CAS-I-B			
58928..60539	-	PKM49945.1	PGZO01000024.1	Firmicutes bacterium HGW-Firmicutes-7	Bacteria	Firmicutes					type I-B CRISPR-associated protein Cas8b1/Cst1	cas8b2,cas8b2	cd09754,cd09665	CAS-I-B			
60519..61350	-	PKM49946.1	PGZO01000024.1	Firmicutes bacterium HGW-Firmicutes-7	Bacteria	Firmicutes					CRISPR-associated endoribonuclease Cas6	cas6	cd09652	CAS-I,CAS-III			
334 CAS-I-B | ANQ52936.1																	
67016..67670	+	ANQ52929.1	CP003562.1	Flammeovirga sp. MY04	Bacteria	Bacteroidetes	Cytophagia	Cytophagales	Flammeovirgaceae	Flammeovirga	CRISPR-associated protein Cas6	cas6	cd09652	CAS-I,CAS-III			
67672..68197	+	ANQ52930.1	CP003562.1	Flammeovirga sp. MY04	Bacteria	Bacteroidetes	Cytophagia	Cytophagales	Flammeovirgaceae	Flammeovirga	Hypothetical protein	cas5	mkCas0069	CAS-I-B			
68180..70319	+	ANQ52931.1	CP003562.1	Flammeovirga sp. MY04	Bacteria	Bacteroidetes	Cytophagia	Cytophagales	Flammeovirgaceae	Flammeovirga	CRISPR-associated helicase Cas3 domain protein	cas3HD,cas3	cd09641,COG1203	CAS-I			
70284..71547	+	ANQ52932.1	CP003562.1	Flammeovirga sp. MY04	Bacteria	Bacteroidetes	Cytophagia	Cytophagales	Flammeovirgaceae	Flammeovirga	Hypothetical protein	cas8b6	mkCas0111	CAS-I-B			
71521..72601	+	ANQ52933.1	CP003562.1	Flammeovirga sp. MY04	Bacteria	Bacteroidetes	Cytophagia	Cytophagales	Flammeovirgaceae	Flammeovirga	Hypothetical protein	cas7	mkCas0088	CAS-I-B			
72737..73163	+	ANQ52934.1	CP003562.1	Flammeovirga sp. MY04	Bacteria	Bacteroidetes	Cytophagia	Cytophagales	Flammeovirgaceae	Flammeovirga	Crispr-associated protein Cas4	cas4	pfam01930	CAS-I			
73170..74175	+	ANQ52935.1	CP003562.1	Flammeovirga sp. MY04	Bacteria	Bacteroidetes	Cytophagia	Cytophagales	Flammeovirgaceae	Flammeovirga	Crispr-associated protein Cas1	cas1	cd09722	CAS-I-B			
74204..74465	+	ANQ52936.1	CP003562.1	Flammeovirga sp. MY04	Bacteria	Bacteroidetes	Cytophagia	Cytophagales	Flammeovirgaceae	Flammeovirga	CRISPR-associated protein Cas2	cas2	cd09725	CAS-I,CAS-II,CAS-III,CAS-V			
74674..77069	array		CP003562.1	Flammeovirga sp. MY04	Bacteria	Bacteroidetes	Cytophagia	Cytophagales	Flammeovirgaceae	Flammeovirga		CRISPR array			37	35	30
335 CAS-I-B | GBU08795.1																	
160..1189	+	GBU08790.1	BHEP01000088.1	Bacteroidales bacterium	Bacteria	Bacteroidetes	Bacteroidia	Bacteroidales			type I-B CRISPR-associated protein Cas7/Cst2/DevR	cas7	cd09687	CAS-I-B			
1188..1854	+	GBU08791.1	BHEP01000088.1	Bacteroidales bacterium	Bacteria	Bacteroidetes	Bacteroidia	Bacteroidales			hypothetical protein	cas5	cd09658	CAS-I-B			
1850..4067	+	GBU08792.1	BHEP01000088.1	Bacteroidales bacterium	Bacteria	Bacteroidetes	Bacteroidia	Bacteroidales			CRISPR-associated helicase/endonuclease Cas3	cas3HD,cas3	cd09641,COG1203	CAS-I			
4085..4598	+	GBU08793.1	BHEP01000088.1	Bacteroidales bacterium	Bacteria	Bacteroidetes	Bacteroidia	Bacteroidales			CRISPR-associated protein Cas4	cas4	pfam01930	CAS-I			
4607..5627	+	GBU08794.1	BHEP01000088.1	Bacteroidales bacterium	Bacteria	Bacteroidetes	Bacteroidia	Bacteroidales			CRISPR-associated endonuclease Cas1	cas1	cd09722	CAS-I-B			
5626..5887	+	GBU08795.1	BHEP01000088.1	Bacteroidales bacterium	Bacteria	Bacteroidetes	Bacteroidia	Bacteroidales			CRISPR-associated endoribonuclease Cas2	cas2	cd09725	CAS-I,CAS-II,CAS-III,CAS-V			
6107..7181	array		BHEP01000088.1	Bacteroidales bacterium	Bacteria	Bacteroidetes	Bacteroidia	Bacteroidales				CRISPR array			17	35	30
336 CAS-I-B | PSR02143.1																	
8765..9593	+	PSR02129.1	PXTV01000202.1	Bacteroidetes bacterium SW_11_45_7	Bacteria	Bacteroidetes					CRISPR-associated endoribonuclease Cas6	cas6	COG1583	CAS-I,CAS-III,CAS-IV			
9727..10111	+	PSR02130.1	PXTV01000202.1	Bacteroidetes bacterium SW_11_45_7	Bacteria	Bacteroidetes					hypothetical protein						
10337..10982	+	PSR02131.1	PXTV01000202.1	Bacteroidetes bacterium SW_11_45_7	Bacteria	Bacteroidetes					hypothetical protein	WYL	COG2378	CAS-I,CAS-III			
10978..11215	+	PSR02132.1	PXTV01000202.1	Bacteroidetes bacterium SW_11_45_7	Bacteria	Bacteroidetes					hypothetical protein	WYL*	pfam13280				
11323..11524	+	PSR02133.1	PXTV01000202.1	Bacteroidetes bacterium SW_11_45_7	Bacteria	Bacteroidetes					hypothetical protein						
11585..11816	+	PSR02134.1	PXTV01000202.1	Bacteroidetes bacterium SW_11_45_7	Bacteria	Bacteroidetes					hypothetical protein						
11861..12191	+	PSR02135.1	PXTV01000202.1	Bacteroidetes bacterium SW_11_45_7	Bacteria	Bacteroidetes					hypothetical protein						
12465..13197	+	PSR02136.1	PXTV01000202.1	Bacteroidetes bacterium SW_11_45_7	Bacteria	Bacteroidetes					type I-PGING CRISPR-associated protein Cas5p	cas5	cd09716	CAS-I-B			
13183..14788	+	PSR02137.1	PXTV01000202.1	Bacteroidetes bacterium SW_11_45_7	Bacteria	Bacteroidetes					type I-PGING CRISPR-associated protein Cas8c/Csp2	cas8b4	cd09715	CAS-I-B			
14813..15806	+	PSR02138.1	PXTV01000202.1	Bacteroidetes bacterium SW_11_45_7	Bacteria	Bacteroidetes					type I-PGING CRISPR-associated protein Cas7/Csp1	cas7b,cas7,cas7b	cd09717,cd09685,cd09717	CAS-I-B			
15879..18060	+	PSR02144.1	PXTV01000202.1	Bacteroidetes bacterium SW_11_45_7	Bacteria	Bacteroidetes					CRISPR-associated helicase Cas3'	cas3HD,cas3	cd09641,cd09639	CAS-I			
18299..19380	+	PSR02145.1	PXTV01000202.1	Bacteroidetes bacterium SW_11_45_7	Bacteria	Bacteroidetes					hypothetical protein	Cas_RecF,Cas_RecF	cluster17,cluster17				
19529..20087	+	PSR02139.1	PXTV01000202.1	Bacteroidetes bacterium SW_11_45_7	Bacteria	Bacteroidetes					hypothetical protein						
20227..20749	+	PSR02140.1	PXTV01000202.1	Bacteroidetes bacterium SW_11_45_7	Bacteria	Bacteroidetes					CRISPR-associated protein Cas4	cas4	pfam01930	CAS-I			
20961..21351	+	PSR02146.1	PXTV01000202.1	Bacteroidetes bacterium SW_11_45_7	Bacteria	Bacteroidetes					GxxExxY protein						
21556..22561	+	PSR02141.1	PXTV01000202.1	Bacteroidetes bacterium SW_11_45_7	Bacteria	Bacteroidetes					subtype I-B CRISPR-associated endonuclease Cas1	cas1	cd09722	CAS-I-B			
22571..22838	+	PSR02142.1	PXTV01000202.1	Bacteroidetes bacterium SW_11_45_7	Bacteria	Bacteroidetes					hypothetical protein						
22844..23108	+	PSR02143.1	PXTV01000202.1	Bacteroidetes bacterium SW_11_45_7	Bacteria	Bacteroidetes					CRISPR-associated endonuclease Cas2	cas2	COG1343	CAS-I,CAS-II,CAS-III			
338 CAS-I-B | RCW32758.1																	
124306..126238	array		QPIZ01000014.1	Marinilabilia salmonicolor	Bacteria	Bacteroidetes	Bacteroidia	Marinilabiliales	Marinilabiliaceae	Marinilabilia		CRISPR array			30	35	30
126462..126726	-	RCW32758.1	QPIZ01000014.1	Marinilabilia salmonicolor	Bacteria	Bacteroidetes	Bacteroidia	Marinilabiliales	Marinilabiliaceae	Marinilabilia	CRISPR-associated Cas2 family protein	cas2	cd09725	CAS-I,CAS-II,CAS-III,CAS-V			
126815..127820	-	RCW32759.1	QPIZ01000014.1	Marinilabilia salmonicolor	Bacteria	Bacteroidetes	Bacteroidia	Marinilabiliales	Marinilabiliaceae	Marinilabilia	CRISPR-associated Cas1 family protein	cas1	cd09722	CAS-I-B			
127931..128438	-	RCW32760.1	QPIZ01000014.1	Marinilabilia salmonicolor	Bacteria	Bacteroidetes	Bacteroidia	Marinilabiliales	Marinilabiliaceae	Marinilabilia	CRISPR-associated Cas4 family exonuclease	cas4	pfam01930	CAS-I			
128502..128616	-	RCW32761.1	QPIZ01000014.1	Marinilabilia salmonicolor	Bacteria	Bacteroidetes	Bacteroidia	Marinilabiliales	Marinilabiliaceae	Marinilabilia	hypothetical protein						
128628..129141	-	RCW32762.1	QPIZ01000014.1	Marinilabilia salmonicolor	Bacteria	Bacteroidetes	Bacteroidia	Marinilabiliales	Marinilabiliaceae	Marinilabilia	hypothetical protein						
129160..129958	-	RCW32763.1	QPIZ01000014.1	Marinilabilia salmonicolor	Bacteria	Bacteroidetes	Bacteroidia	Marinilabiliales	Marinilabiliaceae	Marinilabilia	hypothetical protein						
130162..132442	-	RCW32764.1	QPIZ01000014.1	Marinilabilia salmonicolor	Bacteria	Bacteroidetes	Bacteroidia	Marinilabiliales	Marinilabiliaceae	Marinilabilia	CRISPR-associated endonuclease/helicase Cas3	cas3HD,cas3	cd09641,cd09639	CAS-I			
132410..133334	-	RCW32765.1	QPIZ01000014.1	Marinilabilia salmonicolor	Bacteria	Bacteroidetes	Bacteroidia	Marinilabiliales	Marinilabiliaceae	Marinilabilia	CRISPR-associated protein Cst2	cas7b	cd09717	CAS-I-B			
133345..134926	-	RCW32766.1	QPIZ01000014.1	Marinilabilia salmonicolor	Bacteria	Bacteroidetes	Bacteroidia	Marinilabiliales	Marinilabiliaceae	Marinilabilia	CRISPR-associated protein Cas8c/Csp2	cas8b4	cd09715	CAS-I-B			
134922..135645	-	RCW32767.1	QPIZ01000014.1	Marinilabilia salmonicolor	Bacteria	Bacteroidetes	Bacteroidia	Marinilabiliales	Marinilabiliaceae	Marinilabilia	CRISPR-associated protein Cas5	cas5	cd09716	CAS-I-B			
135671..136328	-	RCW32768.1	QPIZ01000014.1	Marinilabilia salmonicolor	Bacteria	Bacteroidetes	Bacteroidia	Marinilabiliales	Marinilabiliaceae	Marinilabilia	CRISPR-associated Cas6 family protein	cas6	cd09652	CAS-I,CAS-III			
136380..137247	-	RCW32769.1	QPIZ01000014.1	Marinilabilia salmonicolor	Bacteria	Bacteroidetes	Bacteroidia	Marinilabiliales	Marinilabiliaceae	Marinilabilia	putative DNA-binding transcriptional regulator YafY	WYL	COG2378	CAS-I,CAS-III			
339 CAS-I-B | SKC24160.1																	
258..522	-	SKC24160.1	FUYV01000035.1	Alkalitalea saponilacus	Bacteria	Bacteroidetes	Bacteroidia	Marinilabiliales	Marinilabiliaceae	Alkalitalea	CRISPR-associated protein Cas2	cas2	cd09725	CAS-I,CAS-II,CAS-III,CAS-V			
583..1588	-	SKC24161.1	FUYV01000035.1	Alkalitalea saponilacus	Bacteria	Bacteroidetes	Bacteroidia	Marinilabiliales	Marinilabiliaceae	Alkalitalea	CRISP-associated protein Cas1	cas1	cd09722	CAS-I-B			
1679..2192	-	SKC24162.1	FUYV01000035.1	Alkalitalea saponilacus	Bacteria	Bacteroidetes	Bacteroidia	Marinilabiliales	Marinilabiliaceae	Alkalitalea	CRISPR-associated exonuclease Cas4	cas4	pfam01930	CAS-I			
2323..2827	-	SKC24163.1	FUYV01000035.1	Alkalitalea saponilacus	Bacteria	Bacteroidetes	Bacteroidia	Marinilabiliales	Marinilabiliaceae	Alkalitalea	hypothetical protein						
3025..4087	-	SKC24164.1	FUYV01000035.1	Alkalitalea saponilacus	Bacteria	Bacteroidetes	Bacteroidia	Marinilabiliales	Marinilabiliaceae	Alkalitalea	hypothetical protein	cas7	mkCas0088	CAS-I-B			
4165..5539	-	SKC24165.1	FUYV01000035.1	Alkalitalea saponilacus	Bacteria	Bacteroidetes	Bacteroidia	Marinilabiliales	Marinilabiliaceae	Alkalitalea	hypothetical protein	cas8b6	mkCas0111	CAS-I-B			
5535..7716	-	SKC24166.1	FUYV01000035.1	Alkalitalea saponilacus	Bacteria	Bacteroidetes	Bacteroidia	Marinilabiliales	Marinilabiliaceae	Alkalitalea	CRISPR-associated endonuclease/helicase Cas3	cas3HD,cas3	cd09641,COG1203	CAS-I			
7911..8367	-	SKC24167.1	FUYV01000035.1	Alkalitalea saponilacus	Bacteria	Bacteroidetes	Bacteroidia	Marinilabiliales	Marinilabiliaceae	Alkalitalea	REP element-mobilizing transposase RayT						
8754..9351	-	SKC24168.1	FUYV01000035.1	Alkalitalea saponilacus	Bacteria	Bacteroidetes	Bacteroidia	Marinilabiliales	Marinilabiliaceae	Alkalitalea	hypothetical protein	cas5	mkCas0069	CAS-I-B			
9356..9785	-	SKC24169.1	FUYV01000035.1	Alkalitalea saponilacus	Bacteria	Bacteroidetes	Bacteroidia	Marinilabiliales	Marinilabiliaceae	Alkalitalea	hypothetical protein						
9807..10464	-	SKC24170.1	FUYV01000035.1	Alkalitalea saponilacus	Bacteria	Bacteroidetes	Bacteroidia	Marinilabiliales	Marinilabiliaceae	Alkalitalea	CRISPR-associated endoribonuclease Cas6	cas6	cd09759	CAS-I-A			
10525..11350	-	SKC24171.1	FUYV01000035.1	Alkalitalea saponilacus	Bacteria	Bacteroidetes	Bacteroidia	Marinilabiliales	Marinilabiliaceae	Alkalitalea	Predicted DNA-binding transcriptional regulator YafY, contains an HTH and WYL domains	WYL	COG2378	CAS-I,CAS-III			
340 CAS-I | GAO28630.1																	
195449..196310	+	GAO28621.1	BAZW01000003.1	Geofilum rubicundum JCM 15548	Bacteria	Bacteroidetes	Bacteroidia	Marinilabiliales	Marinilabiliaceae	Geofilum	hypothetical protein	WYL	pfam13280	CAS-I,CAS-III			
196409..197177	+	GAO28622.1	BAZW01000003.1	Geofilum rubicundum JCM 15548	Bacteria	Bacteroidetes	Bacteroidia	Marinilabiliales	Marinilabiliaceae	Geofilum	CRISPR repeat RNA endoribonuclease Cas6	cas6	COG1583	CAS-I,CAS-III,CAS-IV			
197242..198667	+	GAO28623.1	BAZW01000003.1	Geofilum rubicundum JCM 15548	Bacteria	Bacteroidetes	Bacteroidia	Marinilabiliales	Marinilabiliaceae	Geofilum	hypothetical protein	cas8b2	cls000029	CAS-I-B			
198679..199777	+	GAO28624.1	BAZW01000003.1	Geofilum rubicundum JCM 15548	Bacteria	Bacteroidetes	Bacteroidia	Marinilabiliales	Marinilabiliaceae	Geofilum	CRISPR-associated negative autoregulator	cas7	cd09687	CAS-I-B			
199776..200436	+	GAO28625.1	BAZW01000003.1	Geofilum rubicundum JCM 15548	Bacteria	Bacteroidetes	Bacteroidia	Marinilabiliales	Marinilabiliaceae	Geofilum	CRISPR-associated protein, Cas5t family	cas5	COG1688	CAS-I			
200432..202715	+	GAO28626.1	BAZW01000003.1	Geofilum rubicundum JCM 15548	Bacteria	Bacteroidetes	Bacteroidia	Marinilabiliales	Marinilabiliaceae	Geofilum	CRISPR-associated helicase Cas3	cas3HD,cas3	cd09641,COG1203	CAS-I			
202957..203479	+	GAO28627.1	BAZW01000003.1	Geofilum rubicundum JCM 15548	Bacteria	Bacteroidetes	Bacteroidia	Marinilabiliales	Marinilabiliaceae	Geofilum	CRISPR-associated RecB family exonuclease Cas4a	cas4	pfam01930	CAS-I			
203494..205186	+	GAO28628.1	BAZW01000003.1	Geofilum rubicundum JCM 15548	Bacteria	Bacteroidetes	Bacteroidia	Marinilabiliales	Marinilabiliaceae	Geofilum	hypothetical protein						
205220..206228	+	GAO28629.1	BAZW01000003.1	Geofilum rubicundum JCM 15548	Bacteria	Bacteroidetes	Bacteroidia	Marinilabiliales	Marinilabiliaceae	Geofilum	CRISPR-associated protein Cas1	cas1	cd09722	CAS-I-B			
206280..206544	+	GAO28630.1	BAZW01000003.1	Geofilum rubicundum JCM 15548	Bacteria	Bacteroidetes	Bacteroidia	Marinilabiliales	Marinilabiliaceae	Geofilum	CRISPR-associated protein Cas2	cas2	cd09725	CAS-I,CAS-II,CAS-III,CAS-V			
206995..207295	+	GAO28631.1	BAZW01000003.1	Geofilum rubicundum JCM 15548	Bacteria	Bacteroidetes	Bacteroidia	Marinilabiliales	Marinilabiliaceae	Geofilum	hypothetical protein						
207764..208513	array		BAZW01000003.1	Geofilum rubicundum JCM 15548	Bacteria	Bacteroidetes	Bacteroidia	Marinilabiliales	Marinilabiliaceae	Geofilum		CRISPR array			12	35	30
341 CAS-I | SFE65352.1																	
96428..96692	-	SFE65352.1	FONA01000015.1	Thermophagus xiamenensis	Bacteria	Bacteroidetes	Bacteroidia	Marinilabiliales	Marinilabiliaceae	Thermophagus	CRISPR-associated protein, Cas2 family	cas2	cd09725	CAS-I,CAS-II,CAS-III,CAS-V			
96820..97825	-	SFE65379.1	FONA01000015.1	Thermophagus xiamenensis	Bacteria	Bacteroidetes	Bacteroidia	Marinilabiliales	Marinilabiliaceae	Thermophagus	CRISPR-associated protein, Cas1 family	cas1	cd09722	CAS-I-B			
97940..98297	-	SFE65405.1	FONA01000015.1	Thermophagus xiamenensis	Bacteria	Bacteroidetes	Bacteroidia	Marinilabiliales	Marinilabiliaceae	Thermophagus	four helix bundle protein						
98431..98938	-	SFE65432.1	FONA01000015.1	Thermophagus xiamenensis	Bacteria	Bacteroidetes	Bacteroidia	Marinilabiliales	Marinilabiliaceae	Thermophagus	CRISPR-associated exonuclease, Cas4 family	cas4	pfam01930	CAS-I			
99068..101345	-	SFE65456.1	FONA01000015.1	Thermophagus xiamenensis	Bacteria	Bacteroidetes	Bacteroidia	Marinilabiliales	Marinilabiliaceae	Thermophagus	CRISPR-associated helicase, Cas3 family	cas3HD,cas3	cd09641,COG1203	CAS-I			
101368..102052	-	SFE65483.1	FONA01000015.1	Thermophagus xiamenensis	Bacteria	Bacteroidetes	Bacteroidia	Marinilabiliales	Marinilabiliaceae	Thermophagus	CRISPR-associated protein, Cas5t family	cas5	COG1688	CAS-I			
102055..103135	-	SFE65507.1	FONA01000015.1	Thermophagus xiamenensis	Bacteria	Bacteroidetes	Bacteroidia	Marinilabiliales	Marinilabiliaceae	Thermophagus	CRISPR-associated autoregulator, Cst2 family	cas7	cd09687	CAS-I-B			
103146..104544	-	SFE65529.1	FONA01000015.1	Thermophagus xiamenensis	Bacteria	Bacteroidetes	Bacteroidia	Marinilabiliales	Marinilabiliaceae	Thermophagus	CRISPR-associated protein, Cst1 family	cas8b2	cls000029	CAS-I-B			
104778..105423	-	SFE65559.1	FONA01000015.1	Thermophagus xiamenensis	Bacteria	Bacteroidetes	Bacteroidia	Marinilabiliales	Marinilabiliaceae	Thermophagus	hypothetical protein						
105897..106689	-	SFE65587.1	FONA01000015.1	Thermophagus xiamenensis	Bacteria	Bacteroidetes	Bacteroidia	Marinilabiliales	Marinilabiliaceae	Thermophagus	CRISPR-associated protein, Cas6 family	cas6	COG1583	CAS-I,CAS-III,CAS-IV			
343 partial CAS-I-B | OGW02464.1																	
0..436	+	OGW02463.1	MHDL01000150.1	Nitrospinae bacterium RIFCSPLOWO2_02_39_17	Bacteria	Nitrospinae					CRISPR-associated endonuclease Cas1	cas1	cd09722	CAS-I-B			
585..852	+	OGW02464.1	MHDL01000150.1	Nitrospinae bacterium RIFCSPLOWO2_02_39_17	Bacteria	Nitrospinae					CRISPR-associated endonuclease Cas2	cas2	cd09725	CAS-I,CAS-II,CAS-III,CAS-V			
932..1160	+	OGW02465.1	MHDL01000150.1	Nitrospinae bacterium RIFCSPLOWO2_02_39_17	Bacteria	Nitrospinae					hypothetical protein						
1662..4813	array		MHDL01000150.1	Nitrospinae bacterium RIFCSPLOWO2_02_39_17	Bacteria	Nitrospinae						CRISPR array			49	36	29
344 CAS-I-B | GAB63525.1																	
224502..225414	+	GAB63509.1	BAFH01000004.1	Candidatus Jettenia caeni	Bacteria	Planctomycetes	Candidatus Brocadiae	Candidatus Brocadiales	Candidatus Brocadiaceae	Candidatus Jettenia	CRISPR-associated protein	cas6,cas6	COG1583,pfam01881	CAS-I,CAS-III,CAS-IV			
225469..225934	+	GAB63510.1	BAFH01000004.1	Candidatus Jettenia caeni	Bacteria	Planctomycetes	Candidatus Brocadiae	Candidatus Brocadiales	Candidatus Brocadiaceae	Candidatus Jettenia	ribosomal protein						
226063..227254	-	GAB63511.1	BAFH01000004.1	Candidatus Jettenia caeni	Bacteria	Planctomycetes	Candidatus Brocadiae	Candidatus Brocadiales	Candidatus Brocadiaceae	Candidatus Jettenia	transposase						
227419..227587	+	GAB63512.1	BAFH01000004.1	Candidatus Jettenia caeni	Bacteria	Planctomycetes	Candidatus Brocadiae	Candidatus Brocadiales	Candidatus Brocadiaceae	Candidatus Jettenia	hypothetical protein						
227664..227949	+	GAB63513.1	BAFH01000004.1	Candidatus Jettenia caeni	Bacteria	Planctomycetes	Candidatus Brocadiae	Candidatus Brocadiales	Candidatus Brocadiaceae	Candidatus Jettenia	hypothetical protein						
228196..229333	+	GAB63514.1	BAFH01000004.1	Candidatus Jettenia caeni	Bacteria	Planctomycetes	Candidatus Brocadiae	Candidatus Brocadiales	Candidatus Brocadiaceae	Candidatus Jettenia	putative transposase						
229519..230287	+	GAB63515.1	BAFH01000004.1	Candidatus Jettenia caeni	Bacteria	Planctomycetes	Candidatus Brocadiae	Candidatus Brocadiales	Candidatus Brocadiaceae	Candidatus Jettenia	hypothetical protein						
230298..231921	+	GAB63516.1	BAFH01000004.1	Candidatus Jettenia caeni	Bacteria	Planctomycetes	Candidatus Brocadiae	Candidatus Brocadiales	Candidatus Brocadiaceae	Candidatus Jettenia	conserved hypothetical protein	cas8b1	mkCas0209	CAS-I-B			
231921..232875	+	GAB63517.1	BAFH01000004.1	Candidatus Jettenia caeni	Bacteria	Planctomycetes	Candidatus Brocadiae	Candidatus Brocadiales	Candidatus Brocadiaceae	Candidatus Jettenia	CRISPR-associated protein	cas7	cd09687	CAS-I-B			
232876..233539	+	GAB63518.1	BAFH01000004.1	Candidatus Jettenia caeni	Bacteria	Planctomycetes	Candidatus Brocadiae	Candidatus Brocadiales	Candidatus Brocadiaceae	Candidatus Jettenia	CRISPR-associated protein	cas5	cd09658	CAS-I-B			
233535..235929	+	GAB63519.1	BAFH01000004.1	Candidatus Jettenia caeni	Bacteria	Planctomycetes	Candidatus Brocadiae	Candidatus Brocadiales	Candidatus Brocadiaceae	Candidatus Jettenia	CRISPR-associated protein	cas3HD,cas3	cd09641,cd09639	CAS-I			
236030..237035	+	GAB63520.1	BAFH01000004.1	Candidatus Jettenia caeni	Bacteria	Planctomycetes	Candidatus Brocadiae	Candidatus Brocadiales	Candidatus Brocadiaceae	Candidatus Jettenia	transposase						
238170..238881	+	GAB63521.1	BAFH01000004.1	Candidatus Jettenia caeni	Bacteria	Planctomycetes	Candidatus Brocadiae	Candidatus Brocadiales	Candidatus Brocadiaceae	Candidatus Jettenia	hypothetical protein						
239038..239548	+	GAB63522.1	BAFH01000004.1	Candidatus Jettenia caeni	Bacteria	Planctomycetes	Candidatus Brocadiae	Candidatus Brocadiales	Candidatus Brocadiaceae	Candidatus Jettenia	CRISPR-associated protein	cas4	pfam01930	CAS-I			
239525..239990	+	GAB63523.1	BAFH01000004.1	Candidatus Jettenia caeni	Bacteria	Planctomycetes	Candidatus Brocadiae	Candidatus Brocadiales	Candidatus Brocadiaceae	Candidatus Jettenia	ribosomal protein						
240079..241072	+	GAB63524.1	BAFH01000004.1	Candidatus Jettenia caeni	Bacteria	Planctomycetes	Candidatus Brocadiae	Candidatus Brocadiales	Candidatus Brocadiaceae	Candidatus Jettenia	CRISPR-associated protein	cas1	cd09722	CAS-I-B			
241304..241571	+	GAB63525.1	BAFH01000004.1	Candidatus Jettenia caeni	Bacteria	Planctomycetes	Candidatus Brocadiae	Candidatus Brocadiales	Candidatus Brocadiaceae	Candidatus Jettenia	CRISPR-associated protein	cas2	cd09725	CAS-I,CAS-II,CAS-III,CAS-V			
241897..242503	array		BAFH01000004.1	Candidatus Jettenia caeni	Bacteria	Planctomycetes	Candidatus Brocadiae	Candidatus Brocadiales	Candidatus Brocadiaceae	Candidatus Jettenia		CRISPR array			10	35	29
345 CAS-I | RIJ91415.1																	
5810..14745	array		QEUJ01000017.1	Candidatus Brocadia sp.	Bacteria	Planctomycetes	Candidatus Brocadiae	Candidatus Brocadiales	Candidatus Brocadiaceae	Candidatus Brocadia		CRISPR array			139	35	29
15062..15329	-	RIJ91415.1	QEUJ01000017.1	Candidatus Brocadia sp.	Bacteria	Planctomycetes	Candidatus Brocadiae	Candidatus Brocadiales	Candidatus Brocadiaceae	Candidatus Brocadia	CRISPR-associated endonuclease Cas2	cas2	cd09725	CAS-I,CAS-II,CAS-III,CAS-V			
15522..16515	-	RIJ91416.1	QEUJ01000017.1	Candidatus Brocadia sp.	Bacteria	Planctomycetes	Candidatus Brocadiae	Candidatus Brocadiales	Candidatus Brocadiaceae	Candidatus Brocadia	type I-B CRISPR-associated endonuclease Cas1	cas1	cd09722	CAS-I-B			
16626..16989	-	RIJ91471.1	QEUJ01000017.1	Candidatus Brocadia sp.	Bacteria	Planctomycetes	Candidatus Brocadiae	Candidatus Brocadiales	Candidatus Brocadiaceae	Candidatus Brocadia	GxxExxY protein						
17074..17584	-	RIJ91417.1	QEUJ01000017.1	Candidatus Brocadia sp.	Bacteria	Planctomycetes	Candidatus Brocadiae	Candidatus Brocadiales	Candidatus Brocadiaceae	Candidatus Brocadia	CRISPR-associated protein Cas4	cas4	pfam01930	CAS-I			
17876..18263	-	RIJ91472.1	QEUJ01000017.1	Candidatus Brocadia sp.	Bacteria	Planctomycetes	Candidatus Brocadiae	Candidatus Brocadiales	Candidatus Brocadiaceae	Candidatus Brocadia	type II toxin-antitoxin system death-on-curing family toxin						
18262..18490	-	RIJ91418.1	QEUJ01000017.1	Candidatus Brocadia sp.	Bacteria	Planctomycetes	Candidatus Brocadiae	Candidatus Brocadiales	Candidatus Brocadiaceae	Candidatus Brocadia	AbrB family transcriptional regulator						
18783..19158	-	RIJ91419.1	QEUJ01000017.1	Candidatus Brocadia sp.	Bacteria	Planctomycetes	Candidatus Brocadiae	Candidatus Brocadiales	Candidatus Brocadiaceae	Candidatus Brocadia	hypothetical protein						
19157..19433	-	RIJ91420.1	QEUJ01000017.1	Candidatus Brocadia sp.	Bacteria	Planctomycetes	Candidatus Brocadiae	Candidatus Brocadiales	Candidatus Brocadiaceae	Candidatus Brocadia	hypothetical protein						
19819..20578	-	RIJ91421.1	QEUJ01000017.1	Candidatus Brocadia sp.	Bacteria	Planctomycetes	Candidatus Brocadiae	Candidatus Brocadiales	Candidatus Brocadiaceae	Candidatus Brocadia	hypothetical protein	cas5*	cls000048				
20546..21641	-	RIJ91422.1	QEUJ01000017.1	Candidatus Brocadia sp.	Bacteria	Planctomycetes	Candidatus Brocadiae	Candidatus Brocadiales	Candidatus Brocadiaceae	Candidatus Brocadia	hypothetical protein	cas7	pfam01905	CAS-I			
21637..23083	-	RIJ91423.1	QEUJ01000017.1	Candidatus Brocadia sp.	Bacteria	Planctomycetes	Candidatus Brocadiae	Candidatus Brocadiales	Candidatus Brocadiaceae	Candidatus Brocadia	hypothetical protein	cas8a8	mkCas0180	CAS-I-A			
23079..25485	-	RIJ91424.1	QEUJ01000017.1	Candidatus Brocadia sp.	Bacteria	Planctomycetes	Candidatus Brocadiae	Candidatus Brocadiales	Candidatus Brocadiaceae	Candidatus Brocadia	CRISPR-associated helicase Cas3'	cas3,cas3HD	COG1203,cd09641	CAS-I			
346 partial CAS-I-B | OGW14360.1																	
1594..2107	+	OGW14364.1	MHDS01000099.1	Nitrospirae bacterium GWA2_42_11	Bacteria	Nitrospirae					CRISPR-associated protein Cas4	cas4	pfam01930	CAS-I			
2103..3096	+	OGW14359.1	MHDS01000099.1	Nitrospirae bacterium GWA2_42_11	Bacteria	Nitrospirae					subtype I-B CRISPR-associated endonuclease Cas1	cas1	cd09722	CAS-I-B			
3201..3468	+	OGW14360.1	MHDS01000099.1	Nitrospirae bacterium GWA2_42_11	Bacteria	Nitrospirae					CRISPR-associated endonuclease Cas2	cas2	cd09725	CAS-I,CAS-II,CAS-III,CAS-V			
3700..5281	array		MHDS01000099.1	Nitrospirae bacterium GWA2_42_11	Bacteria	Nitrospirae						CRISPR array			25	35	29
5694..5895	+	OGW14365.1	MHDS01000099.1	Nitrospirae bacterium GWA2_42_11	Bacteria	Nitrospirae					cold-shock protein						
5966..6635	-	OGW14361.1	MHDS01000099.1	Nitrospirae bacterium GWA2_42_11	Bacteria	Nitrospirae					hypothetical protein	C3a174	cluster174				
347 CAS-I-A | CAJ74868.1																	
1684059..1685238	+	CAJ74855.1	CT573071.1	Candidatus Kuenenia stuttgartiensis	Bacteria	Planctomycetes	Candidatus Brocadiae	Candidatus Brocadiales	Candidatus Brocadiaceae	Candidatus Kuenenia	hypothetical protein	cas6,cas6,cas6	COG1583,cd09759,pfam01881	CAS-I,CAS-III,CAS-IV			

1685341..1685590	+	CAJ74856.1	CT573071.1	Candidatus Kuenenia stuttgartiensis	Bacteria	Planctomycetes	Candidatus Brocadiae	Candidatus Brocadiales	Candidatus Brocadiaceae	Candidatus Kuenenia	conserved hypothetical protein						
1685992..1686790	+	CAJ74857.1	CT573071.1	Candidatus Kuenenia stuttgartiensis	Bacteria	Planctomycetes	Candidatus Brocadiae	Candidatus Brocadiales	Candidatus Brocadiaceae	Candidatus Kuenenia	conserved hypothetical protein						
1686789..1687629	+	CAJ74858.1	CT573071.1	Candidatus Kuenenia stuttgartiensis	Bacteria	Planctomycetes	Candidatus Brocadiae	Candidatus Brocadiales	Candidatus Brocadiaceae	Candidatus Kuenenia	hypothetical protein	C3a174	cluster174				
1687625..1690052	+	CAJ74859.1	CT573071.1	Candidatus Kuenenia stuttgartiensis	Bacteria	Planctomycetes	Candidatus Brocadiae	Candidatus Brocadiales	Candidatus Brocadiaceae	Candidatus Kuenenia	conserved hypothetical protein	cas3,cas3HD	COG1203,cd09641	CAS-I			
1690048..1691755	+	CAJ74860.1	CT573071.1	Candidatus Kuenenia stuttgartiensis	Bacteria	Planctomycetes	Candidatus Brocadiae	Candidatus Brocadiales	Candidatus Brocadiaceae	Candidatus Kuenenia	unknown protein	cas8a4	cls000706	CAS-I-A			
1691757..1692741	+	CAJ74861.1	CT573071.1	Candidatus Kuenenia stuttgartiensis	Bacteria	Planctomycetes	Candidatus Brocadiae	Candidatus Brocadiales	Candidatus Brocadiaceae	Candidatus Kuenenia	conserved hypothetical protein	cas7	pfam01905	CAS-I			
1692730..1693504	+	CAJ74862.1	CT573071.1	Candidatus Kuenenia stuttgartiensis	Bacteria	Planctomycetes	Candidatus Brocadiae	Candidatus Brocadiales	Candidatus Brocadiaceae	Candidatus Kuenenia	hypothetical protein	cas5	cls000048	CAS-I-A			
1693895..1694123	+	CAJ74863.1	CT573071.1	Candidatus Kuenenia stuttgartiensis	Bacteria	Planctomycetes	Candidatus Brocadiae	Candidatus Brocadiales	Candidatus Brocadiaceae	Candidatus Kuenenia	conserved hypothetical protein						
1694119..1694509	+	CAJ74864.1	CT573071.1	Candidatus Kuenenia stuttgartiensis	Bacteria	Planctomycetes	Candidatus Brocadiae	Candidatus Brocadiales	Candidatus Brocadiaceae	Candidatus Kuenenia	conserved hypothetical protein						
1694522..1695035	+	CAJ74865.1	CT573071.1	Candidatus Kuenenia stuttgartiensis	Bacteria	Planctomycetes	Candidatus Brocadiae	Candidatus Brocadiales	Candidatus Brocadiaceae	Candidatus Kuenenia	conserved hypothetical protein	cas4	pfam01930	CAS-I			
1695142..1696135	+	CAJ74866.1	CT573071.1	Candidatus Kuenenia stuttgartiensis	Bacteria	Planctomycetes	Candidatus Brocadiae	Candidatus Brocadiales	Candidatus Brocadiaceae	Candidatus Kuenenia	conserved hypothetical protein	cas1	cd09722	CAS-I-B			
1696131..1696437	+	CAJ74867.1	CT573071.1	Candidatus Kuenenia stuttgartiensis	Bacteria	Planctomycetes	Candidatus Brocadiae	Candidatus Brocadiales	Candidatus Brocadiaceae	Candidatus Kuenenia	hypothetical protein						
1696439..1696706	+	CAJ74868.1	CT573071.1	Candidatus Kuenenia stuttgartiensis	Bacteria	Planctomycetes	Candidatus Brocadiae	Candidatus Brocadiales	Candidatus Brocadiaceae	Candidatus Kuenenia	conserved hypothetical protein	cas2	cd09725	CAS-I,CAS-II,CAS-III,CAS-V			
1697022..1698279	array		CT573071.1	Candidatus Kuenenia stuttgartiensis	Bacteria	Planctomycetes	Candidatus Brocadiae	Candidatus Brocadiales	Candidatus Brocadiaceae	Candidatus Kuenenia		CRISPR array			20	35	29

348 CAS-I-A | SOH02987.1

489910..490524	array		LT934425.1	Candidatus Kuenenia stuttgartiensis	Bacteria	Planctomycetes	Candidatus Brocadiae	Candidatus Brocadiales	Candidatus Brocadiaceae	Candidatus Kuenenia		CRISPR array			10	36	29
490642..491746	+	SOH02984.1	LT934425.1	Candidatus Kuenenia stuttgartiensis	Bacteria	Planctomycetes	Candidatus Brocadiae	Candidatus Brocadiales	Candidatus Brocadiaceae	Candidatus Kuenenia	hypothetical protein						
492003..493063	array		LT934425.1	Candidatus Kuenenia stuttgartiensis	Bacteria	Planctomycetes	Candidatus Brocadiae	Candidatus Brocadiales	Candidatus Brocadiaceae	Candidatus Kuenenia		CRISPR array			17	35	29
493140..494877	-	SOH02985.1	LT934425.1	Candidatus Kuenenia stuttgartiensis	Bacteria	Planctomycetes	Candidatus Brocadiae	Candidatus Brocadiales	Candidatus Brocadiaceae	Candidatus Kuenenia	Hypothetical Protein						
495108..496174	array		LT934425.1	Candidatus Kuenenia stuttgartiensis	Bacteria	Planctomycetes	Candidatus Brocadiae	Candidatus Brocadiales	Candidatus Brocadiaceae	Candidatus Kuenenia		CRISPR array			17	35	29
496298..497627	+	SOH02986.1	LT934425.1	Candidatus Kuenenia stuttgartiensis	Bacteria	Planctomycetes	Candidatus Brocadiae	Candidatus Brocadiales	Candidatus Brocadiaceae	Candidatus Kuenenia	putative transposase						
498283..498766	array		LT934425.1	Candidatus Kuenenia stuttgartiensis	Bacteria	Planctomycetes	Candidatus Brocadiae	Candidatus Brocadiales	Candidatus Brocadiaceae	Candidatus Kuenenia		CRISPR array			8	36	29
499080..499347	-	SOH02987.1	LT934425.1	Candidatus Kuenenia stuttgartiensis	Bacteria	Planctomycetes	Candidatus Brocadiae	Candidatus Brocadiales	Candidatus Brocadiaceae	Candidatus Kuenenia	hypothetical protein	cas2	cd09725	CAS-I,CAS-II,CAS-III,CAS-V			
499525..500518	-	SOH02988.1	LT934425.1	Candidatus Kuenenia stuttgartiensis	Bacteria	Planctomycetes	Candidatus Brocadiae	Candidatus Brocadiales	Candidatus Brocadiaceae	Candidatus Kuenenia	hypothetical protein	cas1	cd09722	CAS-I-B			
500625..501138	-	SOH02989.1	LT934425.1	Candidatus Kuenenia stuttgartiensis	Bacteria	Planctomycetes	Candidatus Brocadiae	Candidatus Brocadiales	Candidatus Brocadiaceae	Candidatus Kuenenia	hypothetical protein	cas4	pfam01930	CAS-I			
501151..501538	-	SOH02990.1	LT934425.1	Candidatus Kuenenia stuttgartiensis	Bacteria	Planctomycetes	Candidatus Brocadiae	Candidatus Brocadiales	Candidatus Brocadiaceae	Candidatus Kuenenia	hypothetical protein						
501537..501765	-	SOH02991.1	LT934425.1	Candidatus Kuenenia stuttgartiensis	Bacteria	Planctomycetes	Candidatus Brocadiae	Candidatus Brocadiales	Candidatus Brocadiaceae	Candidatus Kuenenia	hypothetical protein						
502156..502918	-	SOH02992.1	LT934425.1	Candidatus Kuenenia stuttgartiensis	Bacteria	Planctomycetes	Candidatus Brocadiae	Candidatus Brocadiales	Candidatus Brocadiaceae	Candidatus Kuenenia	hypothetical protein	cas5	cls000048	CAS-I-A			
502919..503903	-	SOH02993.1	LT934425.1	Candidatus Kuenenia stuttgartiensis	Bacteria	Planctomycetes	Candidatus Brocadiae	Candidatus Brocadiales	Candidatus Brocadiaceae	Candidatus Kuenenia	hypothetical protein	cas7	pfam01905	CAS-I			
503905..505612	-	SOH02994.1	LT934425.1	Candidatus Kuenenia stuttgartiensis	Bacteria	Planctomycetes	Candidatus Brocadiae	Candidatus Brocadiales	Candidatus Brocadiaceae	Candidatus Kuenenia	hypothetical protein	cas8a4	cls000706	CAS-I-A			
505608..508011	-	SOH02995.1	LT934425.1	Candidatus Kuenenia stuttgartiensis	Bacteria	Planctomycetes	Candidatus Brocadiae	Candidatus Brocadiales	Candidatus Brocadiaceae	Candidatus Kuenenia	hypothetical protein	cas3,cas3HD	COG1203,cd09641	CAS-I			
508031..508871	-	SOH02996.1	LT934425.1	Candidatus Kuenenia stuttgartiensis	Bacteria	Planctomycetes	Candidatus Brocadiae	Candidatus Brocadiales	Candidatus Brocadiaceae	Candidatus Kuenenia	hypothetical protein	C3a174	cluster174				
508870..509668	-	SOH02997.1	LT934425.1	Candidatus Kuenenia stuttgartiensis	Bacteria	Planctomycetes	Candidatus Brocadiae	Candidatus Brocadiales	Candidatus Brocadiaceae	Candidatus Kuenenia	hypothetical protein						
510011..510467	-	SOH02998.1	LT934425.1	Candidatus Kuenenia stuttgartiensis	Bacteria	Planctomycetes	Candidatus Brocadiae	Candidatus Brocadiales	Candidatus Brocadiaceae	Candidatus Kuenenia	hypothetical protein						
510570..511614	-	SOH02999.1	LT934425.1	Candidatus Kuenenia stuttgartiensis	Bacteria	Planctomycetes	Candidatus Brocadiae	Candidatus Brocadiales	Candidatus Brocadiaceae	Candidatus Kuenenia	CRISPR associated protein Cas6	cas6,cas6	cd09759,pfam01881	CAS-I-A			

349 CAS-I-B | ALO17063.1

4154252..4155059	+	ALO17049.1	CP013118.1	Salinivirga cyanobacteriivorans	Bacteria	Bacteroidetes	Bacteroidia	Marinilabiliales	Salinivirgaceae	Salinivirga	CRISPR-associated endoribonuclease Cas6	cas6	cd09759	CAS-I-A			
4155055..4155691	+	ALO17050.1	CP013118.1	Salinivirga cyanobacteriivorans	Bacteria	Bacteroidetes	Bacteroidia	Marinilabiliales	Salinivirgaceae	Salinivirga	hypothetical protein	PD-DExK,PD-DExK	COG4804,COG4804	CAS-I,CAS-III			
4155735..4157139	+	ALO17051.1	CP013118.1	Salinivirga cyanobacteriivorans	Bacteria	Bacteroidetes	Bacteroidia	Marinilabiliales	Salinivirgaceae	Salinivirga	hypothetical protein						
4157278..4159138	+	ALO17052.1	CP013118.1	Salinivirga cyanobacteriivorans	Bacteria	Bacteroidetes	Bacteroidia	Marinilabiliales	Salinivirgaceae	Salinivirga	hypothetical protein	cas8b1	mkCas0106	CAS-I-B			
4159150..4160185	+	ALO17053.1	CP013118.1	Salinivirga cyanobacteriivorans	Bacteria	Bacteroidetes	Bacteroidia	Marinilabiliales	Salinivirgaceae	Salinivirga	CRISPR-associated protein Cas7/Csh2, subtype I-B/HMARI	cas7b	pfam05107	CAS-I-B,CAS-I-C			
4160168..4160975	+	ALO17054.1	CP013118.1	Salinivirga cyanobacteriivorans	Bacteria	Bacteroidetes	Bacteroidia	Marinilabiliales	Salinivirgaceae	Salinivirga	CRISPR-associated protein Cas5, subtype I-B/HMARI	cas5	cls001430	CAS-I-B			
4160949..4163652	+	ALO17055.1	CP013118.1	Salinivirga cyanobacteriivorans	Bacteria	Bacteroidetes	Bacteroidia	Marinilabiliales	Salinivirgaceae	Salinivirga	CRISPR-associated nuclease/helicase Cas3	cas3HD,cas3	cd09641,cd09639	CAS-I			
4163859..4165278	+	ALO17056.1	CP013118.1	Salinivirga cyanobacteriivorans	Bacteria	Bacteroidetes	Bacteroidia	Marinilabiliales	Salinivirgaceae	Salinivirga	hypothetical protein						
4165497..4165746	+	ALO17057.1	CP013118.1	Salinivirga cyanobacteriivorans	Bacteria	Bacteroidetes	Bacteroidia	Marinilabiliales	Salinivirgaceae	Salinivirga	CRISPR-associated protein Cas4	cas4	pfam01930	CAS-I			
4165838..4166603	+	ALO17058.1	CP013118.1	Salinivirga cyanobacteriivorans	Bacteria	Bacteroidetes	Bacteroidia	Marinilabiliales	Salinivirgaceae	Salinivirga	hypothetical protein						
4166607..4167459	+	ALO17059.1	CP013118.1	Salinivirga cyanobacteriivorans	Bacteria	Bacteroidetes	Bacteroidia	Marinilabiliales	Salinivirgaceae	Salinivirga	hypothetical protein	C3a174	cluster174				
4167539..4167977	+	ALO17060.1	CP013118.1	Salinivirga cyanobacteriivorans	Bacteria	Bacteroidetes	Bacteroidia	Marinilabiliales	Salinivirgaceae	Salinivirga	CRISPR-associated protein Cas4	cas4	pfam01930	CAS-I			
4168027..4169032	+	ALO17061.1	CP013118.1	Salinivirga cyanobacteriivorans	Bacteria	Bacteroidetes	Bacteroidia	Marinilabiliales	Salinivirgaceae	Salinivirga	CRISPR-associated endonuclease Cas1	cas1	cd09722	CAS-I-B			
4169144..4169522	+	ALO17062.1	CP013118.1	Salinivirga cyanobacteriivorans	Bacteria	Bacteroidetes	Bacteroidia	Marinilabiliales	Salinivirgaceae	Salinivirga	four helix bundle protein						
4169765..4170029	+	ALO17063.1	CP013118.1	Salinivirga cyanobacteriivorans	Bacteria	Bacteroidetes	Bacteroidia	Marinilabiliales	Salinivirgaceae	Salinivirga	CRISPR-associated endoribonuclease Cas2	cas2	cd09725	CAS-I,CAS-II,CAS-III,CAS-V			
4170253..4176363	array		CP013118.1	Salinivirga cyanobacteriivorans	Bacteria	Bacteroidetes	Bacteroidia	Marinilabiliales	Salinivirgaceae	Salinivirga		CRISPR array			93	36	30
4176451..4177618	+	ALO17064.1	CP013118.1	Salinivirga cyanobacteriivorans	Bacteria	Bacteroidetes	Bacteroidia	Marinilabiliales	Salinivirgaceae	Salinivirga	Transposase						
4177839..4193842	array		CP013118.1	Salinivirga cyanobacteriivorans	Bacteria	Bacteroidetes	Bacteroidia	Marinilabiliales	Salinivirgaceae	Salinivirga		CRISPR array			243	36	30
4197068..4197353	-	ALO17065.1	CP013118.1	Salinivirga cyanobacteriivorans	Bacteria	Bacteroidetes	Bacteroidia	Marinilabiliales	Salinivirgaceae	Salinivirga	co-chaperonin GroES						
4197377..4197944	-	ALO17066.1	CP013118.1	Salinivirga cyanobacteriivorans	Bacteria	Bacteroidetes	Bacteroidia	Marinilabiliales	Salinivirgaceae	Salinivirga	Glutaredoxin-3						
4198084..4200043	+	ALO17067.1	CP013118.1	Salinivirga cyanobacteriivorans	Bacteria	Bacteroidetes	Bacteroidia	Marinilabiliales	Salinivirgaceae	Salinivirga	ATP-dependent DNA helicase RecQ	cas3	cd09639	CAS-I			

350 CAS-I-B | SHE83445.1

669842..674722	array		FQUM01000002.1	Mariniphaga anaerophila	Bacteria	Bacteroidetes	Bacteroidia	Marinilabiliales	Prolixibacteraceae	Mariniphaga		CRISPR array			75	35	30
670964..671387	+	SHE83319.1	FQUM01000002.1	Mariniphaga anaerophila	Bacteria	Bacteroidetes	Bacteroidia	Marinilabiliales	Prolixibacteraceae	Mariniphaga	hypothetical protein						
672572..672797	+	SHE83346.1	FQUM01000002.1	Mariniphaga anaerophila	Bacteria	Bacteroidetes	Bacteroidia	Marinilabiliales	Prolixibacteraceae	Mariniphaga	hypothetical protein						
672968..673229	+	SHE83380.1	FQUM01000002.1	Mariniphaga anaerophila	Bacteria	Bacteroidetes	Bacteroidia	Marinilabiliales	Prolixibacteraceae	Mariniphaga	hypothetical protein						
674666..674849	-	SHE83411.1	FQUM01000002.1	Mariniphaga anaerophila	Bacteria	Bacteroidetes	Bacteroidia	Marinilabiliales	Prolixibacteraceae	Mariniphaga	hypothetical protein						
674947..675211	-	SHE83445.1	FQUM01000002.1	Mariniphaga anaerophila	Bacteria	Bacteroidetes	Bacteroidia	Marinilabiliales	Prolixibacteraceae	Mariniphaga	CRISPR-associated protein, Cas2 family	cas2	cd09725	CAS-I,CAS-II,CAS-III,CAS-V			
675210..676215	-	SHE83477.1	FQUM01000002.1	Mariniphaga anaerophila	Bacteria	Bacteroidetes	Bacteroidia	Marinilabiliales	Prolixibacteraceae	Mariniphaga	CRISPR-associated protein, Cas1 family	cas1	cd09722	CAS-I-B			
676283..676787	-	SHE83510.1	FQUM01000002.1	Mariniphaga anaerophila	Bacteria	Bacteroidetes	Bacteroidia	Marinilabiliales	Prolixibacteraceae	Mariniphaga	CRISPR-associated exonuclease, Cas4 family	cas4	pfam01930	CAS-I			
676826..677411	-	SHE83541.1	FQUM01000002.1	Mariniphaga anaerophila	Bacteria	Bacteroidetes	Bacteroidia	Marinilabiliales	Prolixibacteraceae	Mariniphaga	pRiA4b ORF-3-like protein						
677555..679811	-	SHE83575.1	FQUM01000002.1	Mariniphaga anaerophila	Bacteria	Bacteroidetes	Bacteroidia	Marinilabiliales	Prolixibacteraceae	Mariniphaga	CRISPR-associated endonuclease/helicase Cas3	cas3	cd09639	CAS-I			
679803..680787	-	SHE83606.1	FQUM01000002.1	Mariniphaga anaerophila	Bacteria	Bacteroidetes	Bacteroidia	Marinilabiliales	Prolixibacteraceae	Mariniphaga	CRISPR-associated protein Cst2	cas7b,cas7,cas7b	cd09717,cd09685,cd09717	CAS-I-B			
680814..682332	-	SHE83638.1	FQUM01000002.1	Mariniphaga anaerophila	Bacteria	Bacteroidetes	Bacteroidia	Marinilabiliales	Prolixibacteraceae	Mariniphaga	CRISPR-associated protein Cas8c/Csp2, subtype PGING	cas8b4	cd09715	CAS-I-B			
682328..683087	-	SHE83674.1	FQUM01000002.1	Mariniphaga anaerophila	Bacteria	Bacteroidetes	Bacteroidia	Marinilabiliales	Prolixibacteraceae	Mariniphaga	CRISPR-associated protein Cas5, subtype PGING	cas5	cd09716	CAS-I-B			
683094..683214	-	SHE83712.1	FQUM01000002.1	Mariniphaga anaerophila	Bacteria	Bacteroidetes	Bacteroidia	Marinilabiliales	Prolixibacteraceae	Mariniphaga	hypothetical protein						
683301..683949	-	SHE83747.1	FQUM01000002.1	Mariniphaga anaerophila	Bacteria	Bacteroidetes	Bacteroidia	Marinilabiliales	Prolixibacteraceae	Mariniphaga	hypothetical protein						
683972..684491	+	SHE83778.1	FQUM01000002.1	Mariniphaga anaerophila	Bacteria	Bacteroidetes	Bacteroidia	Marinilabiliales	Prolixibacteraceae	Mariniphaga	hypothetical protein						
684332..685130	-	SHE83815.1	FQUM01000002.1	Mariniphaga anaerophila	Bacteria	Bacteroidetes	Bacteroidia	Marinilabiliales	Prolixibacteraceae	Mariniphaga	CRISPR-associated protein, Cas6 family	cas6	COG1583	CAS-I,CAS-III,CAS-IV			

352 CAS-I-B | GAP44181.1

3040335..3046749	array		DF968182.1	Lentimicrobium saccharophilum	Bacteria	Bacteroidetes	Bacteroidia	Bacteroidales	Lentimicrobiaceae	Lentimicrobium		CRISPR array			96	36	31
3046970..3047234	-	GAP44181.1	DF968182.1	Lentimicrobium saccharophilum	Bacteria	Bacteroidetes	Bacteroidia	Bacteroidales	Lentimicrobiaceae	Lentimicrobium	CRISPR-associated protein, Cas2 family	cas2	cd09725	CAS-I,CAS-II,CAS-III,CAS-V			
3047248..3048253	-	GAP44182.1	DF968182.1	Lentimicrobium saccharophilum	Bacteria	Bacteroidetes	Bacteroidia	Bacteroidales	Lentimicrobiaceae	Lentimicrobium	CRISPR-associated endonuclease Cas1	cas1	cd09722	CAS-I-B			
3048356..3048863	-	GAP44183.1	DF968182.1	Lentimicrobium saccharophilum	Bacteria	Bacteroidetes	Bacteroidia	Bacteroidales	Lentimicrobiaceae	Lentimicrobium	CRISPR-associated protein Cas4	cas4	pfam01930	CAS-I			
3049001..3049676	-	GAP44184.1	DF968182.1	Lentimicrobium saccharophilum	Bacteria	Bacteroidetes	Bacteroidia	Bacteroidales	Lentimicrobiaceae	Lentimicrobium	hypothetical protein						
3049752..3050805	-	GAP44185.1	DF968182.1	Lentimicrobium saccharophilum	Bacteria	Bacteroidetes	Bacteroidia	Bacteroidales	Lentimicrobiaceae	Lentimicrobium	hypothetical protein	cas7	mkCas0088	CAS-I-B			
3050826..3052221	-	GAP44186.1	DF968182.1	Lentimicrobium saccharophilum	Bacteria	Bacteroidetes	Bacteroidia	Bacteroidales	Lentimicrobiaceae	Lentimicrobium	hypothetical protein	cas8b6	mkCas0111	CAS-I-B			
3052224..3054393	-	GAP44187.1	DF968182.1	Lentimicrobium saccharophilum	Bacteria	Bacteroidetes	Bacteroidia	Bacteroidales	Lentimicrobiaceae	Lentimicrobium	CRISPR-associated helicase Cas3	cas3HD,cas3	cd09641,COG1203	CAS-I			
3054389..3054986	-	GAP44188.1	DF968182.1	Lentimicrobium saccharophilum	Bacteria	Bacteroidetes	Bacteroidia	Bacteroidales	Lentimicrobiaceae	Lentimicrobium	hypothetical protein	cas5	mkCas0069	CAS-I-B			
3055006..3055663	-	GAP44189.1	DF968182.1	Lentimicrobium saccharophilum	Bacteria	Bacteroidetes	Bacteroidia	Bacteroidales	Lentimicrobiaceae	Lentimicrobium	CRISPR-associated endoribonuclease Cas6	cas6	cd09652	CAS-I,CAS-III			
3055692..3056088	-	GAP44190.1	DF968182.1	Lentimicrobium saccharophilum	Bacteria	Bacteroidetes	Bacteroidia	Bacteroidales	Lentimicrobiaceae	Lentimicrobium	protein containing HIRAN domain						
3056142..3057072	-	GAP44191.1	DF968182.1	Lentimicrobium saccharophilum	Bacteria	Bacteroidetes	Bacteroidia	Bacteroidales	Lentimicrobiaceae	Lentimicrobium	predicted DNA-binding transcriptional regulator YafY	WYL	COG2378	CAS-I,CAS-III			
3057110..3057914	-	GAP44192.1	DF968182.1	Lentimicrobium saccharophilum	Bacteria	Bacteroidetes	Bacteroidia	Bacteroidales	Lentimicrobiaceae	Lentimicrobium	CRISPR-associated endoribonuclease Cas6	cas6	cd09759	CAS-I-A			

353 CAS-I-B | PIQ29316.1

23557..37948	array		PCUL01000325.1	Bacteroidetes bacterium CG18_big_fil_WC_8_21_14_2_50_41_14	Bacteria	Bacteroidetes						CRISPR array			219	35	30
38165..38429	-	PIQ29316.1	PCUL01000325.1	Bacteroidetes bacterium CG18_big_fil_WC_8_21_14_2_50_41_14	Bacteria	Bacteroidetes					CRISPR-associated endonuclease Cas2	cas2	cd09725	CAS-I,CAS-II,CAS-III,CAS-V			
38623..39628	-	PIQ29317.1	PCUL01000325.1	Bacteroidetes bacterium CG18_big_fil_WC_8_21_14_2_50_41_14	Bacteria	Bacteroidetes					subtype I-B CRISPR-associated endonuclease Cas1	cas1	cd09722	CAS-I-B			
39624..40140	-	PIQ29318.1	PCUL01000325.1	Bacteroidetes bacterium CG18_big_fil_WC_8_21_14_2_50_41_14	Bacteria	Bacteroidetes					CRISPR-associated protein Cas4	cas4	pfam01930	CAS-I			
40146..42843	-	PIQ29319.1	PCUL01000325.1	Bacteroidetes bacterium CG18_big_fil_WC_8_21_14_2_50_41_14	Bacteria	Bacteroidetes					CRISPR-associated protein	cas3HD,cas3	cd09641,COG1203	CAS-I			
42893..43664	-	PIQ29320.1	PCUL01000325.1	Bacteroidetes bacterium CG18_big_fil_WC_8_21_14_2_50_41_14	Bacteria	Bacteroidetes					type I-B CRISPR-associated protein Cas5	cas5	cls001430	CAS-I-B			
43660..44614	-	PIQ29321.1	PCUL01000325.1	Bacteroidetes bacterium CG18_big_fil_WC_8_21_14_2_50_41_14	Bacteria	Bacteroidetes					CRISPR-associated protein	cas7b	pfam05107	CAS-I-B,CAS-I-C			
44625..46572	-	PIQ29322.1	PCUL01000325.1	Bacteroidetes bacterium CG18_big_fil_WC_8_21_14_2_50_41_14	Bacteria	Bacteroidetes					hypothetical protein	cas8b1	mkCas0106	CAS-I-B			

354 partial CAS-I-B | KUK56786.1

0..503	+	KUK56783.1	LGGC01000332.1	Bacteroidetes bacterium 38_7	Bacteria	Bacteroidetes					Uncharacterized protein	cas5	cls000048	CAS-I-A			
640..1075	+	KUK56784.1	LGGC01000332.1	Bacteroidetes bacterium 38_7	Bacteria	Bacteroidetes					hypothetical protein	cas4	pfam01930	CAS-I			
1071..2076	+	KUK56785.1	LGGC01000332.1	Bacteroidetes bacterium 38_7	Bacteria	Bacteroidetes					CRISPR-associated protein Cas1	cas1	cd09722	CAS-I-B			
2075..2339	+	KUK56786.1	LGGC01000332.1	Bacteroidetes bacterium 38_7	Bacteria	Bacteroidetes					CRISPR-associated protein Cas2	cas2	cd09725	CAS-I,CAS-II,CAS-III,CAS-V			

355 CAS-I | RIJ47245.1

170555..177553	array		QWGR01000009.1	Prolixibacteraceae bacterium XSD2	Bacteria	Bacteroidetes	Bacteroidia	Marinilabiliales	Prolixibacteraceae			CRISPR array			108	39	26
177782..178046	-	RIJ47245.1	QWGR01000009.1	Prolixibacteraceae bacterium XSD2	Bacteria	Bacteroidetes	Bacteroidia	Marinilabiliales	Prolixibacteraceae		CRISPR-associated endonuclease Cas2	cas2	cd09725	CAS-I,CAS-II,CAS-III,CAS-V			
178045..179050	-	RIJ47246.1	QWGR01000009.1	Prolixibacteraceae bacterium XSD2	Bacteria	Bacteroidetes	Bacteroidia	Marinilabiliales	Prolixibacteraceae		type I-B CRISPR-associated endonuclease Cas1	cas1	cd09722	CAS-I-B			
179062..179479	-	RIJ47247.1	QWGR01000009.1	Prolixibacteraceae bacterium XSD2	Bacteria	Bacteroidetes	Bacteroidia	Marinilabiliales	Prolixibacteraceae		GxxExxY protein						
179508..180015	-	RIJ47248.1	QWGR01000009.1	Prolixibacteraceae bacterium XSD2	Bacteria	Bacteroidetes	Bacteroidia	Marinilabiliales	Prolixibacteraceae		CRISPR-associated protein Cas4	cas4	pfam01930	CAS-I			
180077..180734	-	RIJ47249.1	QWGR01000009.1	Prolixibacteraceae bacterium XSD2	Bacteria	Bacteroidetes	Bacteroidia	Marinilabiliales	Prolixibacteraceae		hypothetical protein	cas5	cls000048	CAS-I-A			
180736..181714	-	RIJ47250.1	QWGR01000009.1	Prolixibacteraceae bacterium XSD2	Bacteria	Bacteroidetes	Bacteroidia	Marinilabiliales	Prolixibacteraceae		DevR family CRISPR-associated autoregulator	cas7	pfam01905	CAS-I			
181733..183470	-	RIJ47251.1	QWGR01000009.1	Prolixibacteraceae bacterium XSD2	Bacteria	Bacteroidetes	Bacteroidia	Marinilabiliales	Prolixibacteraceae		hypothetical protein	cas8a4	cls000706	CAS-I-A			
183474..185976	-	RIJ47252.1	QWGR01000009.1	Prolixibacteraceae bacterium XSD2	Bacteria	Bacteroidetes	Bacteroidia	Marinilabiliales	Prolixibacteraceae		CRISPR-associated helicase Cas3'	cas3,cas3HD	COG1203,cd09641	CAS-I			
186048..186837	-	RIJ47272.1	QWGR01000009.1	Prolixibacteraceae bacterium XSD2	Bacteria	Bacteroidetes	Bacteroidia	Marinilabiliales	Prolixibacteraceae		CRISPR-associated endoribonuclease Cas6	cas6	COG1583	CAS-I,CAS-III,CAS-IV			
186934..187846	-	RIJ47253.1	QWGR01000009.1	Prolixibacteraceae bacterium XSD2	Bacteria	Bacteroidetes	Bacteroidia	Marinilabiliales	Prolixibacteraceae		WYL domain-containing protein	WYL	COG2378	CAS-I,CAS-III			

356 CAS-I-B | GAP70877.1

365352..366135	+	GAP70870.1	DF970690.1	Bacteroidales bacterium 6E	Bacteria	Bacteroidetes	Bacteroidia	Bacteroidales			CRISPR-associated protein, Cas6 family	cas6	COG1583	CAS-I,CAS-III,CAS-IV			
366211..367837	+	GAP70871.1	DF970690.1	Bacteroidales bacterium 6E	Bacteria	Bacteroidetes	Bacteroidia	Bacteroidales			hypothetical protein	Cas8b1,Cas8b1,Cas8b1	icity0005,icity0005,icity0005				
367860..368805	+	GAP70872.1	DF970690.1	Bacteroidales bacterium 6E	Bacteria	Bacteroidetes	Bacteroidia	Bacteroidales			CRISPR-associated protein, CT1132 family	cas7	cd09690	CAS-I-B			
368804..369518	+	GAP70873.1	DF970690.1	Bacteroidales bacterium 6E	Bacteria	Bacteroidetes	Bacteroidia	Bacteroidales			CRISPR-associated protein, Cas5h family	cas5	cls001430	CAS-I-B			
369502..371935	+	GAP70874.1	DF970690.1	Bacteroidales bacterium 6E	Bacteria	Bacteroidetes	Bacteroidia	Bacteroidales			CRISPR-associated helicase, Cas3 family	cas3HD,cas3	cd09641,COG1203	CAS-I			
372026..372551	+	GAP70875.1	DF970690.1	Bacteroidales bacterium 6E	Bacteria	Bacteroidetes	Bacteroidia	Bacteroidales			CRISPR-associated exonuclease, Cas4 family	cas4	pfam01930	CAS-I			
372560..373565	+	GAP70876.1	DF970690.1	Bacteroidales bacterium 6E	Bacteria	Bacteroidetes	Bacteroidia	Bacteroidales			CRISPR-associated protein, Cas1 family	cas1	cd09722	CAS-I-B			
373564..373828	+	GAP70877.1	DF970690.1	Bacteroidales bacterium 6E	Bacteria	Bacteroidetes	Bacteroidia	Bacteroidales			CRISPR-associated protein, Cas2 family	cas2	cd09725	CAS-I,CAS-II,CAS-III,CAS-V			
374057..377361	array		DF970690.1	Bacteroidales bacterium 6E	Bacteria	Bacteroidetes	Bacteroidia	Bacteroidales				CRISPR array			51	35	30
377744..379295	+	GAP70878.1	DF970690.1	Bacteroidales bacterium 6E	Bacteria	Bacteroidetes	Bacteroidia	Bacteroidales			transposase						
379307..380027	+	GAP70879.1	DF970690.1	Bacteroidales bacterium 6E	Bacteria	Bacteroidetes	Bacteroidia	Bacteroidales			DNA replication protein DnaC						
380132..380743	array		DF970690.1	Bacteroidales bacterium 6E	Bacteria	Bacteroidetes	Bacteroidia	Bacteroidales				CRISPR array			10	34	30

358 CAS-I-B | OFX53969.1

24457..25114	+	OFX53965.1	MENL01000170.1	Bacteroidetes bacterium GWB2_41_8	Bacteria	Bacteroidetes					CRISPR-associated endoribonuclease Cas6	cas6	cd09652	CAS-I,CAS-III			
25157..25703	+	OFX53984.1	MENL01000170.1	Bacteroidetes bacterium GWB2_41_8	Bacteria	Bacteroidetes					hypothetical protein	cas5	mkCas0069	CAS-I-B			
25702..27844	+	OFX53985.1	MENL01000170.1	Bacteroidetes bacterium GWB2_41_8	Bacteria	Bacteroidetes					CRISPR-associated helicase/endonuclease Cas3	cas3HD,cas3	cd09641,COG1203	CAS-I			
27855..28751	+	OFX53966.1	MENL01000170.1	Bacteroidetes bacterium GWB2_41_8	Bacteria	Bacteroidetes					hypothetical protein	cas8b6	mkCas0111	CAS-I-B			
29126..30188	+	OFX53967.1	MENL01000170.1	Bacteroidetes bacterium GWB2_41_8	Bacteria	Bacteroidetes					CRISPR-associated protein Cas7	cas7	mkCas0088	CAS-I-B			
30265..30778	+	OFX53968.1	MENL01000170.1	Bacteroidetes bacterium GWB2_41_8	Bacteria	Bacteroidetes					CRISPR-associated protein Cas4	cas4	pfam01930	CAS-I			
31820..32084	+	OFX53969.1	MENL01000170.1	Bacteroidetes bacterium GWB2_41_8	Bacteria	Bacteroidetes					CRISPR-associated endonuclease Cas2	cas2	cd09725	CAS-I,CAS-II,CAS-III,CAS-V			
32310..39600	array		MENL01000170.1	Bacteroidetes bacterium GWB2_41_8	Bacteria	Bacteroidetes						CRISPR array			112	35	30

359 CAS-I | OFX76356.1

7955..8735	+	OFX76349.1	MENW01000016.1	Bacteroidetes bacterium GWE2_39_28	Bacteria	Bacteroidetes					CRISPR-associated endoribonuclease Cas6	cas6	COG1583	CAS-I,CAS-III,CAS-IV			
8746..11206	+	OFX76350.1	MENW01000016.1	Bacteroidetes bacterium GWE2_39_28	Bacteria	Bacteroidetes					hypothetical protein	cas3,cas3HD	COG1203,cd09641	CAS-I			

11210..12944	+	OFX76351.1	MENW01000016.1	Bacteroidetes bacterium GWE2_39_28	Bacteria	Bacteroidetes					hypothetical protein	cas8a4	cls000706	CAS-I-A			
12969..13947	+	OFX76352.1	MENW01000016.1	Bacteroidetes bacterium GWE2_39_28	Bacteria	Bacteroidetes					CRISPR-associated protein	cas7	pfam01905	CAS-I			
13949..14606	+	OFX76353.1	MENW01000016.1	Bacteroidetes bacterium GWE2_39_28	Bacteria	Bacteroidetes					hypothetical protein	cas5	cls000048	CAS-I-A			
14614..15133	+	OFX76354.1	MENW01000016.1	Bacteroidetes bacterium GWE2_39_28	Bacteria	Bacteroidetes					CRISPR-associated protein Cas4	cas4	pfam01930	CAS-I			
15144..16149	+	OFX76355.1	MENW01000016.1	Bacteroidetes bacterium GWE2_39_28	Bacteria	Bacteroidetes					subtype I-B CRISPR-associated endonuclease Cas1	cas1	cd09722	CAS-I-B			
16150..16414	+	OFX76356.1	MENW01000016.1	Bacteroidetes bacterium GWE2_39_28	Bacteria	Bacteroidetes					CRISPR-associated endonuclease Cas2	cas2	cd09725	CAS-I,CAS-II,CAS-III,CAS-V			
16643..19872	array		MENW01000016.1	Bacteroidetes bacterium GWE2_39_28	Bacteria	Bacteroidetes						CRISPR array			50	35	30
360 CAS-I-B | OJV19806.1																	
13774..14434	+	OJV19799.1	MKTA01000023.1	Bacteroidetes bacterium 41-46	Bacteria	Bacteroidetes					CRISPR-associated endoribonuclease Cas6	cas6	cd09652	CAS-I,CAS-III			
14492..15038	+	OJV19800.1	MKTA01000023.1	Bacteroidetes bacterium 41-46	Bacteria	Bacteroidetes					hypothetical protein	cas5	mkCas0069	CAS-I-B			
15037..17179	+	OJV19801.1	MKTA01000023.1	Bacteroidetes bacterium 41-46	Bacteria	Bacteroidetes					CRISPR-associated helicase/endonuclease Cas3	cas3HD,cas3	cd09641,COG1203	CAS-I			
17191..18514	+	OJV19802.1	MKTA01000023.1	Bacteroidetes bacterium 41-46	Bacteria	Bacteroidetes					hypothetical protein	cas8b6	mkCas0111	CAS-I-B			
18525..19623	+	OJV19803.1	MKTA01000023.1	Bacteroidetes bacterium 41-46	Bacteria	Bacteroidetes					CRISPR-associated protein Cas7	cas7	mkCas0088	CAS-I-B			
19679..20198	+	OJV19804.1	MKTA01000023.1	Bacteroidetes bacterium 41-46	Bacteria	Bacteroidetes					CRISPR-associated protein Cas4	cas4	pfam01930	CAS-I			
20209..21214	+	OJV19805.1	MKTA01000023.1	Bacteroidetes bacterium 41-46	Bacteria	Bacteroidetes					subtype I-B CRISPR-associated endonuclease Cas1	cas1	cd09722	CAS-I-B			
21215..21479	+	OJV19806.1	MKTA01000023.1	Bacteroidetes bacterium 41-46	Bacteria	Bacteroidetes					CRISPR-associated endonuclease Cas2	cas2	cd09725	CAS-I,CAS-II,CAS-III,CAS-V			
21707..26989	array		MKTA01000023.1	Bacteroidetes bacterium 41-46	Bacteria	Bacteroidetes						CRISPR array			82	34	30
361 CAS-I | PTN09617.1																	
224693..225608	+	PTN09605.1	QAAD01000004.1	Mangrovibacterium marinum	Bacteria	Bacteroidetes	Bacteroidia	Marinilabiliales	Prolixibacteraceae	Mangrovibacterium	putative DNA-binding transcriptional regulator YafY	WYL	COG2378	CAS-I,CAS-III			
225652..225985	+	PTN09606.1	QAAD01000004.1	Mangrovibacterium marinum	Bacteria	Bacteroidetes	Bacteroidia	Marinilabiliales	Prolixibacteraceae	Mangrovibacterium	HIRAN domain-containing protein						
226019..226898	+	PTN09607.1	QAAD01000004.1	Mangrovibacterium marinum	Bacteria	Bacteroidetes	Bacteroidia	Marinilabiliales	Prolixibacteraceae	Mangrovibacterium	CRISPR-associated endoribonuclease Cas6	cas6	COG1583	CAS-I,CAS-III,CAS-IV			
227128..228553	+	PTN09608.1	QAAD01000004.1	Mangrovibacterium marinum	Bacteria	Bacteroidetes	Bacteroidia	Marinilabiliales	Prolixibacteraceae	Mangrovibacterium	CRISPR-associated Cst1 family protein	cas8b2	cls000029	CAS-I-B			
228565..229663	+	PTN09609.1	QAAD01000004.1	Mangrovibacterium marinum	Bacteria	Bacteroidetes	Bacteroidia	Marinilabiliales	Prolixibacteraceae	Mangrovibacterium	CRISPR-associated Cst2 family autoregulator	cas7	cd09687	CAS-I-B			
229662..230322	+	PTN09610.1	QAAD01000004.1	Mangrovibacterium marinum	Bacteria	Bacteroidetes	Bacteroidia	Marinilabiliales	Prolixibacteraceae	Mangrovibacterium	CRISPR-associated Cas5t family protein	cas5	COG1688	CAS-I			
230318..232655	+	PTN09611.1	QAAD01000004.1	Mangrovibacterium marinum	Bacteria	Bacteroidetes	Bacteroidia	Marinilabiliales	Prolixibacteraceae	Mangrovibacterium	CRISPR-associated Cas3 family helicase	cas3HD,cas3	cd09641,COG1203	CAS-I			
232715..233297	+	PTN09612.1	QAAD01000004.1	Mangrovibacterium marinum	Bacteria	Bacteroidetes	Bacteroidia	Marinilabiliales	Prolixibacteraceae	Mangrovibacterium	hypothetical protein						
233318..234158	+	PTN09613.1	QAAD01000004.1	Mangrovibacterium marinum	Bacteria	Bacteroidetes	Bacteroidia	Marinilabiliales	Prolixibacteraceae	Mangrovibacterium	hypothetical protein						
234195..234705	+	PTN09614.1	QAAD01000004.1	Mangrovibacterium marinum	Bacteria	Bacteroidetes	Bacteroidia	Marinilabiliales	Prolixibacteraceae	Mangrovibacterium	CRISPR-associated Cas4 family exonuclease	cas4	pfam01930	CAS-I			
234799..235168	+	PTN09615.1	QAAD01000004.1	Mangrovibacterium marinum	Bacteria	Bacteroidetes	Bacteroidia	Marinilabiliales	Prolixibacteraceae	Mangrovibacterium	four helix bundle protein						
235207..236212	+	PTN09616.1	QAAD01000004.1	Mangrovibacterium marinum	Bacteria	Bacteroidetes	Bacteroidia	Marinilabiliales	Prolixibacteraceae	Mangrovibacterium	CRISPR-associated Cas1 family protein	cas1	cd09722	CAS-I-B			
236211..236475	+	PTN09617.1	QAAD01000004.1	Mangrovibacterium marinum	Bacteria	Bacteroidetes	Bacteroidia	Marinilabiliales	Prolixibacteraceae	Mangrovibacterium	CRISPR-associated Cas2 family protein	cas2	cd09725	CAS-I,CAS-II,CAS-III,CAS-V			
236702..241964	array		QAAD01000004.1	Mangrovibacterium marinum	Bacteria	Bacteroidetes	Bacteroidia	Marinilabiliales	Prolixibacteraceae	Mangrovibacterium		CRISPR array			81	35	30
362 CAS-I-B | SHJ71167.1																	
307..6804	array		FQZE01000027.1	Tangfeifania diversioriginum	Bacteria	Bacteroidetes	Bacteroidia	Marinilabiliales	Prolixibacteraceae	Tangfeifania		CRISPR array			100	41	24
499..601	-	SHJ70969.1	FQZE01000027.1	Tangfeifania diversioriginum	Bacteria	Bacteroidetes	Bacteroidia	Marinilabiliales	Prolixibacteraceae	Tangfeifania	hypothetical protein						
724..871	-	SHJ71005.1	FQZE01000027.1	Tangfeifania diversioriginum	Bacteria	Bacteroidetes	Bacteroidia	Marinilabiliales	Prolixibacteraceae	Tangfeifania	hypothetical protein						
1402..1504	-	SHJ71041.1	FQZE01000027.1	Tangfeifania diversioriginum	Bacteria	Bacteroidetes	Bacteroidia	Marinilabiliales	Prolixibacteraceae	Tangfeifania	hypothetical protein						
3370..3625	-	SHJ71070.1	FQZE01000027.1	Tangfeifania diversioriginum	Bacteria	Bacteroidetes	Bacteroidia	Marinilabiliales	Prolixibacteraceae	Tangfeifania	hypothetical protein						
5004..5121	-	SHJ71106.1	FQZE01000027.1	Tangfeifania diversioriginum	Bacteria	Bacteroidetes	Bacteroidia	Marinilabiliales	Prolixibacteraceae	Tangfeifania	hypothetical protein						
6816..6969	-	SHJ71139.1	FQZE01000027.1	Tangfeifania diversioriginum	Bacteria	Bacteroidetes	Bacteroidia	Marinilabiliales	Prolixibacteraceae	Tangfeifania	hypothetical protein						
7029..7293	-	SHJ71167.1	FQZE01000027.1	Tangfeifania diversioriginum	Bacteria	Bacteroidetes	Bacteroidia	Marinilabiliales	Prolixibacteraceae	Tangfeifania	CRISPR-associated protein, Cas2 family	cas2	cd09725	CAS-I,CAS-II,CAS-III,CAS-V			
7292..8297	-	SHJ71201.1	FQZE01000027.1	Tangfeifania diversioriginum	Bacteria	Bacteroidetes	Bacteroidia	Marinilabiliales	Prolixibacteraceae	Tangfeifania	CRISPR-associated protein, Cas1 family	cas1	cd09722	CAS-I-B			
8293..8806	-	SHJ71234.1	FQZE01000027.1	Tangfeifania diversioriginum	Bacteria	Bacteroidetes	Bacteroidia	Marinilabiliales	Prolixibacteraceae	Tangfeifania	CRISPR-associated exonuclease, Cas4 family	cas4	pfam01930	CAS-I			
8815..11233	-	SHJ71264.1	FQZE01000027.1	Tangfeifania diversioriginum	Bacteria	Bacteroidetes	Bacteroidia	Marinilabiliales	Prolixibacteraceae	Tangfeifania	CRISPR-associated helicase, Cas3 family	cas3HD,cas3	cd09641,COG1203	CAS-I			
11217..11928	-	SHJ71297.1	FQZE01000027.1	Tangfeifania diversioriginum	Bacteria	Bacteroidetes	Bacteroidia	Marinilabiliales	Prolixibacteraceae	Tangfeifania	CRISPR-associated protein Cas5, subtype I-B/HMARI	cas5	cls001430	CAS-I-B			
11930..12878	-	SHJ71329.1	FQZE01000027.1	Tangfeifania diversioriginum	Bacteria	Bacteroidetes	Bacteroidia	Marinilabiliales	Prolixibacteraceae	Tangfeifania	CRISPR-associated protein, Csh2 family	cas7	cd09690	CAS-I-B			
12899..14528	-	SHJ71362.1	FQZE01000027.1	Tangfeifania diversioriginum	Bacteria	Bacteroidetes	Bacteroidia	Marinilabiliales	Prolixibacteraceae	Tangfeifania	hypothetical protein	Cas8b1,Cas8b1,Cas8b1	icity0005,icity0005,icity0005				
14586..15363	-	SHJ71392.1	FQZE01000027.1	Tangfeifania diversioriginum	Bacteria	Bacteroidetes	Bacteroidia	Marinilabiliales	Prolixibacteraceae	Tangfeifania	CRISPR-associated endoribonuclease Cas6	cas6	COG1583	CAS-I,CAS-III,CAS-IV			
15453..16365	-	SHJ71420.1	FQZE01000027.1	Tangfeifania diversioriginum	Bacteria	Bacteroidetes	Bacteroidia	Marinilabiliales	Prolixibacteraceae	Tangfeifania	Predicted DNA-binding transcriptional regulator YafY, contains an HTH and WYL domains	WYL	COG2378	CAS-I,CAS-III			
363 partial CAS-I-B | KGN99047.1																	
196089..196353	-	KGN99047.1	JQZW01000002.1	Porphyromonas gingivicanis	Bacteria	Bacteroidetes	Bacteroidia	Bacteroidales	Porphyromonadaceae	Porphyromonas	CRISPR-associated protein Cas2	cas2	cd09725	CAS-I,CAS-II,CAS-III,CAS-V			
196352..197369	-	KGN99048.1	JQZW01000002.1	Porphyromonas gingivicanis	Bacteria	Bacteroidetes	Bacteroidia	Bacteroidales	Porphyromonadaceae	Porphyromonas	CRISPR-associated protein Cas1	cas1	cd09722	CAS-I-B			
197365..197878	-	KGN99049.1	JQZW01000002.1	Porphyromonas gingivicanis	Bacteria	Bacteroidetes	Bacteroidia	Bacteroidales	Porphyromonadaceae	Porphyromonas	CRISPR-associated protein Cas4	cas4	pfam01930	CAS-I			
197950..198730	-	KGN99050.1	JQZW01000002.1	Porphyromonas gingivicanis	Bacteria	Bacteroidetes	Bacteroidia	Bacteroidales	Porphyromonadaceae	Porphyromonas	CRISPR-associated protein Cas6	cas6	COG1583	CAS-I,CAS-III,CAS-IV			
364 partial CAS-I-B | SJZ67919.1																	
34692..34956	-	SJZ67919.1	FUXE01000007.1	Porphyromonas circumdentaria	Bacteria	Bacteroidetes	Bacteroidia	Bacteroidales	Porphyromonadaceae	Porphyromonas	CRISPR-associated protein Cas2	cas2	cd09725	CAS-I,CAS-II,CAS-III,CAS-V			
34955..35972	-	SJZ67935.1	FUXE01000007.1	Porphyromonas circumdentaria	Bacteria	Bacteroidetes	Bacteroidia	Bacteroidales	Porphyromonadaceae	Porphyromonas	CRISPR-associated protein, Cas1 family	cas1	cd09722	CAS-I-B			
35968..36481	-	SJZ67955.1	FUXE01000007.1	Porphyromonas circumdentaria	Bacteria	Bacteroidetes	Bacteroidia	Bacteroidales	Porphyromonadaceae	Porphyromonas	CRISPR-associated exonuclease Cas4	cas4	pfam01930	CAS-I			
36598..37378	-	SJZ67972.1	FUXE01000007.1	Porphyromonas circumdentaria	Bacteria	Bacteroidetes	Bacteroidia	Bacteroidales	Porphyromonadaceae	Porphyromonas	CRISPR-associated endoribonuclease Cas6	cas6	COG1583	CAS-I,CAS-III,CAS-IV			
365 CAS-I-B | KGN69281.1																	
70567..70831	-	KGN69281.1	JRAH01000020.1	Porphyromonas sp. COT-108 OH1349	Bacteria	Bacteroidetes	Bacteroidia	Bacteroidales	Porphyromonadaceae	Porphyromonas	CRISPR-associated protein Cas2	cas2	cd09725	CAS-I,CAS-II,CAS-III,CAS-V			
70830..71847	-	KGN69282.1	JRAH01000020.1	Porphyromonas sp. COT-108 OH1349	Bacteria	Bacteroidetes	Bacteroidia	Bacteroidales	Porphyromonadaceae	Porphyromonas	CRISPR-associated protein Cas1	cas1	cd09722	CAS-I-B			
71843..72362	-	KGN69283.1	JRAH01000020.1	Porphyromonas sp. COT-108 OH1349	Bacteria	Bacteroidetes	Bacteroidia	Bacteroidales	Porphyromonadaceae	Porphyromonas	CRISPR-associated protein Cas4	cas4	pfam01930	CAS-I			
72376..74650	-	KGN69284.1	JRAH01000020.1	Porphyromonas sp. COT-108 OH1349	Bacteria	Bacteroidetes	Bacteroidia	Bacteroidales	Porphyromonadaceae	Porphyromonas	CRISPR-associated protein Cas3	cas3HD,cas3	cd09641,cd09639	CAS-I			
74657..75578	-	KGN69285.1	JRAH01000020.1	Porphyromonas sp. COT-108 OH1349	Bacteria	Bacteroidetes	Bacteroidia	Bacteroidales	Porphyromonadaceae	Porphyromonas	CRISPR-associated protein Cas7	cas7b	cd09717	CAS-I-B			
75590..77084	-	KGN69286.1	JRAH01000020.1	Porphyromonas sp. COT-108 OH1349	Bacteria	Bacteroidetes	Bacteroidia	Bacteroidales	Porphyromonadaceae	Porphyromonas	hypothetical protein	cas8b4	cd09715	CAS-I-B			
77070..77778	-	KGN69287.1	JRAH01000020.1	Porphyromonas sp. COT-108 OH1349	Bacteria	Bacteroidetes	Bacteroidia	Bacteroidales	Porphyromonadaceae	Porphyromonas	hypothetical protein	cas5	cd09716	CAS-I-B			
77787..78459	-	KGN69288.1	JRAH01000020.1	Porphyromonas sp. COT-108 OH1349	Bacteria	Bacteroidetes	Bacteroidia	Bacteroidales	Porphyromonadaceae	Porphyromonas	CRISPR-associated protein Cas5	cas6	cd09652	CAS-I,CAS-III			
366 CAS-I-B | KGN74969.1																	
180246..180912	+	KGN74962.1	JRFA01000009.1	Porphyromonas macacae	Bacteria	Bacteroidetes	Bacteroidia	Bacteroidales	Porphyromonadaceae	Porphyromonas	CRISPR-associated protein Cas5	cas6	cd09652	CAS-I,CAS-III			
180922..181501	+	KGN74963.1	JRFA01000009.1	Porphyromonas macacae	Bacteria	Bacteroidetes	Bacteroidia	Bacteroidales	Porphyromonadaceae	Porphyromonas	hypothetical protein	cas5	mkCas0069	CAS-I-B			
181524..183633	+	KGN74964.1	JRFA01000009.1	Porphyromonas macacae	Bacteria	Bacteroidetes	Bacteroidia	Bacteroidales	Porphyromonadaceae	Porphyromonas	CRISPR-associated protein Cas3	cas3HD,cas3	cd09641,COG1203	CAS-I			
183645..184941	+	KGN74965.1	JRFA01000009.1	Porphyromonas macacae	Bacteria	Bacteroidetes	Bacteroidia	Bacteroidales	Porphyromonadaceae	Porphyromonas	hypothetical protein	cas8b6	mkCas0111	CAS-I-B			
184963..186022	+	KGN74966.1	JRFA01000009.1	Porphyromonas macacae	Bacteria	Bacteroidetes	Bacteroidia	Bacteroidales	Porphyromonadaceae	Porphyromonas	CRISPR-associated protein Cas7/Cst2/DevR, subtype I-B/TNEAP	cas7	mkCas0088	CAS-I-B			
186226..186736	+	KGN74967.1	JRFA01000009.1	Porphyromonas macacae	Bacteria	Bacteroidetes	Bacteroidia	Bacteroidales	Porphyromonadaceae	Porphyromonas	CRISPR-associated protein Cas4	cas4	pfam01930	CAS-I			
186732..187743	+	KGN74968.1	JRFA01000009.1	Porphyromonas macacae	Bacteria	Bacteroidetes	Bacteroidia	Bacteroidales	Porphyromonadaceae	Porphyromonas	CRISPR-associated protein Cas1	cas1	cd09722	CAS-I-B			
187742..188006	+	KGN74969.1	JRFA01000009.1	Porphyromonas macacae	Bacteria	Bacteroidetes	Bacteroidia	Bacteroidales	Porphyromonadaceae	Porphyromonas	CRISPR-associated protein Cas2	cas2	COG1343	CAS-I,CAS-II,CAS-III			
188233..190125	array		JRFA01000009.1	Porphyromonas macacae	Bacteria	Bacteroidetes	Bacteroidia	Bacteroidales	Porphyromonadaceae	Porphyromonas		CRISPR array			29	36	30
367 CAS-I-B | SUB89152.1																	
1409648..1410314	+	SUB89144.1	UGTF01000002.1	Porphyromonas macacae	Bacteria	Bacteroidetes	Bacteroidia	Bacteroidales	Porphyromonadaceae	Porphyromonas	Uncharacterized protein predicted to be involved in DNA repair (RAMP superfamily)	cas6	cd09652	CAS-I,CAS-III			
1410324..1410903	+	SUB89145.1	UGTF01000002.1	Porphyromonas macacae	Bacteria	Bacteroidetes	Bacteroidia	Bacteroidales	Porphyromonadaceae	Porphyromonas	Uncharacterised protein	cas5	mkCas0069	CAS-I-B			
1410926..1413035	+	SUB89146.1	UGTF01000002.1	Porphyromonas macacae	Bacteria	Bacteroidetes	Bacteroidia	Bacteroidales	Porphyromonadaceae	Porphyromonas	helicase Cas3	cas3HD,cas3	cd09641,COG1203	CAS-I			
1413047..1414343	+	SUB89147.1	UGTF01000002.1	Porphyromonas macacae	Bacteria	Bacteroidetes	Bacteroidia	Bacteroidales	Porphyromonadaceae	Porphyromonas	Uncharacterised protein	cas8b6	mkCas0111	CAS-I-B			
1414366..1415425	+	SUB89148.1	UGTF01000002.1	Porphyromonas macacae	Bacteria	Bacteroidetes	Bacteroidia	Bacteroidales	Porphyromonadaceae	Porphyromonas	Uncharacterised protein	cas7	mkCas0088	CAS-I-B			
1415503..1415674	-	SUB89149.1	UGTF01000002.1	Porphyromonas macacae	Bacteria	Bacteroidetes	Bacteroidia	Bacteroidales	Porphyromonadaceae	Porphyromonas	Uncharacterised protein						
1415707..1416139	+	SUB89150.1	UGTF01000002.1	Porphyromonas macacae	Bacteria	Bacteroidetes	Bacteroidia	Bacteroidales	Porphyromonadaceae	Porphyromonas	CRISPR-associated protein Cas4	cas4	pfam01930	CAS-I			
1416135..1417146	+	SUB89151.1	UGTF01000002.1	Porphyromonas macacae	Bacteria	Bacteroidetes	Bacteroidia	Bacteroidales	Porphyromonadaceae	Porphyromonas	CRISPR-associated endonuclease Cas1, subtype I-B/HMARI/TNEAP	cas1	cd09722	CAS-I-B			
1417145..1417409	+	SUB89152.1	UGTF01000002.1	Porphyromonas macacae	Bacteria	Bacteroidetes	Bacteroidia	Bacteroidales	Porphyromonadaceae	Porphyromonas	CRISPR-associated endoribonuclease Cas2	cas2	COG1343	CAS-I,CAS-II,CAS-III			
1417636..1421624	array		UGTF01000002.1	Porphyromonas macacae	Bacteria	Bacteroidetes	Bacteroidia	Bacteroidales	Porphyromonadaceae	Porphyromonas		CRISPR array			61	35	30
368 CAS-I-B | KGN83847.1																	
88337..89003	+	KGN83840.1	JRAI01000082.1	Porphyromonas gulae	Bacteria	Bacteroidetes	Bacteroidia	Bacteroidales	Porphyromonadaceae	Porphyromonas	CRISPR-associated protein Cas5	cas6	cd09652	CAS-I,CAS-III			
89009..89612	+	KGN83841.1	JRAI01000082.1	Porphyromonas gulae	Bacteria	Bacteroidetes	Bacteroidia	Bacteroidales	Porphyromonadaceae	Porphyromonas	hypothetical protein	cas5	mkCas0069	CAS-I-B			
89608..91741	+	KGN83842.1	JRAI01000082.1	Porphyromonas gulae	Bacteria	Bacteroidetes	Bacteroidia	Bacteroidales	Porphyromonadaceae	Porphyromonas	CRISPR-associated protein Cas3	cas3HD,cas3	cd09641,COG1203	CAS-I			
91752..93051	+	KGN83843.1	JRAI01000082.1	Porphyromonas gulae	Bacteria	Bacteroidetes	Bacteroidia	Bacteroidales	Porphyromonadaceae	Porphyromonas	hypothetical protein	cas8b6	mkCas0111	CAS-I-B			
93059..94169	+	KGN83844.1	JRAI01000082.1	Porphyromonas gulae	Bacteria	Bacteroidetes	Bacteroidia	Bacteroidales	Porphyromonadaceae	Porphyromonas	CRISPR-associated protein Cas7/Cst2/DevR, subtype I-B/TNEAP	cas7	mkCas0088	CAS-I-B			
94226..94739	+	KGN83845.1	JRAI01000082.1	Porphyromonas gulae	Bacteria	Bacteroidetes	Bacteroidia	Bacteroidales	Porphyromonadaceae	Porphyromonas	CRISPR-associated protein Cas4	cas4	pfam01930	CAS-I			
94735..95752	+	KGN83846.1	JRAI01000082.1	Porphyromonas gulae	Bacteria	Bacteroidetes	Bacteroidia	Bacteroidales	Porphyromonadaceae	Porphyromonas	CRISPR-associated protein Cas1	cas1	cd09722	CAS-I-B			
95751..96015	+	KGN83847.1	JRAI01000082.1	Porphyromonas gulae	Bacteria	Bacteroidetes	Bacteroidia	Bacteroidales	Porphyromonadaceae	Porphyromonas	CRISPR-associated protein Cas2	cas2	cd09725	CAS-I,CAS-II,CAS-III,CAS-V			
96261..96488	array		JRAI01000082.1	Porphyromonas gulae	Bacteria	Bacteroidetes	Bacteroidia	Bacteroidales	Porphyromonadaceae	Porphyromonas		CRISPR array			4	36	30
369 CAS-I-B | KGN88495.1																	
1542..5647	array		JRAK01000070.1	Porphyromonas gulae	Bacteria	Bacteroidetes	Bacteroidia	Bacteroidales	Porphyromonadaceae	Porphyromonas		CRISPR array			63	35	30
5892..6156	-	KGN88495.1	JRAK01000070.1	Porphyromonas gulae	Bacteria	Bacteroidetes	Bacteroidia	Bacteroidales	Porphyromonadaceae	Porphyromonas	CRISPR-associated protein Cas2	cas2	cd09725	CAS-I,CAS-II,CAS-III,CAS-V			
6155..7172	-	KGN88496.1	JRAK01000070.1	Porphyromonas gulae	Bacteria	Bacteroidetes	Bacteroidia	Bacteroidales	Porphyromonadaceae	Porphyromonas	CRISPR-associated protein Cas1	cas1	cd09722	CAS-I-B			
7168..7678	-	KGN88497.1	JRAK01000070.1	Porphyromonas gulae	Bacteria	Bacteroidetes	Bacteroidia	Bacteroidales	Porphyromonadaceae	Porphyromonas	CRISPR-associated protein Cas4	cas4	pfam01930	CAS-I			
7796..8855	-	KGN88498.1	JRAK01000070.1	Porphyromonas gulae	Bacteria	Bacteroidetes	Bacteroidia	Bacteroidales	Porphyromonadaceae	Porphyromonas	CRISPR-associated protein Cas7/Cst2/DevR, subtype I-B/TNEAP	cas7	mkCas0088	CAS-I-B			
8875..10171	-	KGN88499.1	JRAK01000070.1	Porphyromonas gulae	Bacteria	Bacteroidetes	Bacteroidia	Bacteroidales	Porphyromonadaceae	Porphyromonas	hypothetical protein	cas8b6	mkCas0111	CAS-I-B			
10181..12311	-	KGN88500.1	JRAK01000070.1	Porphyromonas gulae	Bacteria	Bacteroidetes	Bacteroidia	Bacteroidales	Porphyromonadaceae	Porphyromonas	CRISPR-associated protein Cas3	cas3HD,cas3	cd09641,COG1203	CAS-I			
12303..12909	-	KGN88501.1	JRAK01000070.1	Porphyromonas gulae	Bacteria	Bacteroidetes	Bacteroidia	Bacteroidales	Porphyromonadaceae	Porphyromonas	hypothetical protein	cas5	mkCas0069	CAS-I-B			
12915..13581	-	KGN88502.1	JRAK01000070.1	Porphyromonas gulae	Bacteria	Bacteroidetes	Bacteroidia	Bacteroidales	Porphyromonadaceae	Porphyromonas	CRISPR-associated protein Cas5	cas6	cd09652	CAS-I,CAS-III			
370 CAS-I-B | AAQ66980.1																	
2102526..2104069	array		AE015924.1	Porphyromonas gingivalis W83	Bacteria	Bacteroidetes	Bacteroidia	Bacteroidales	Porphyromonadaceae	Porphyromonas		CRISPR array			24	35	30
2104315..2104579	-	AAQ66980.1	AE015924.1	Porphyromonas gingivalis W83	Bacteria	Bacteroidetes	Bacteroidia	Bacteroidales	Porphyromonadaceae	Porphyromonas	CRISPR-associated protein Cas2	cas2	cd09725	CAS-I,CAS-II,CAS-III,CAS-V			
2104578..2105595	-	AAQ66981.1	AE015924.1	Porphyromonas gingivalis W83	Bacteria	Bacteroidetes	Bacteroidia	Bacteroidales	Porphyromonadaceae	Porphyromonas	CRISPR-associated protein Cas1	cas1	cd09722	CAS-I-B			
2105591..2106104	-	AAQ66982.1	AE015924.1	Porphyromonas gingivalis W83	Bacteria	Bacteroidetes	Bacteroidia	Bacteroidales	Porphyromonadaceae	Porphyromonas	CRISPR-associated protein Cas4	cas4	pfam01930	CAS-I			
2106123..2108460	-	AAQ66983.1	AE015924.1	Porphyromonas gingivalis W83	Bacteria	Bacteroidetes	Bacteroidia	Bacteroidales	Porphyromonadaceae	Porphyromonas	CRISPR-associated helicase Cas3	cas3HD,cas3	cd09641,cd09639	CAS-I			
2108434..2109451	-	AAQ66984.1	AE015924.1	Porphyromonas gingivalis W83	Bacteria	Bacteroidetes	Bacteroidia	Bacteroidales	Porphyromonadaceae	Porphyromonas	hypothetical protein	cas7b	cd09717	CAS-I-B			
2109461..2110961	-	AAQ66985.1	AE015924.1	Porphyromonas gingivalis W83	Bacteria	Bacteroidetes	Bacteroidia	Bacteroidales	Porphyromonadaceae	Porphyromonas	hypothetical protein	cas8b4	cd09715	CAS-I-B			
2110957..2111752	-	AAQ66986.1	AE015924.1	Porphyromonas gingivalis W83	Bacteria	Bacteroidetes	Bacteroidia	Bacteroidales	Porphyromonadaceae	Porphyromonas	hypothetical protein	cas5	cd09716	CAS-I-B			
2111697..2112363	-	AAQ66987.1	AE015924.1	Porphyromonas gingivalis W83	Bacteria	Bacteroidetes	Bacteroidia	Bacteroidales	Porphyromonadaceae	Porphyromonas	CRISPR-associated protein, TM1814 family	cas6	cd09652	CAS-I,CAS-III			
371 CAS-I-B | BAG34477.1																	
2181484..2189363	array		AP009380.1	Porphyromonas gingivalis ATCC 33277	Bacteria	Bacteroidetes	Bacteroidia	Bacteroidales	Porphyromonadaceae	Porphyromonas		CRISPR array			120	35	30
2189609..2189873	-	BAG34477.1	AP009380.1	Porphyromonas gingivalis ATCC 33277	Bacteria	Bacteroidetes	Bacteroidia	Bacteroidales	Porphyromonadaceae	Porphyromonas	CRISPR-associated protein Cas2	cas2	COG1343	CAS-I,CAS-II,CAS-III			
2189872..2190889	-	BAG34478.1	AP009380.1	Porphyromonas gingivalis ATCC 33277	Bacteria	Bacteroidetes	Bacteroidia	Bacteroidales	Porphyromonadaceae	Porphyromonas	CRISPR-associated protein Cas1	cas1	cd09722	CAS-I-B			
2190885..2191395	-	BAG34479.1	AP009380.1	Porphyromonas gingivalis ATCC 33277	Bacteria	Bacteroidetes	Bacteroidia	Bacteroidales	Porphyromonadaceae	Porphyromonas	CRISPR-associated protein Cas4	cas4	pfam01930	CAS-I			
2191512..2192571	-	BAG34480.1	AP009380.1	Porphyromonas gingivalis ATCC 33277	Bacteria	Bacteroidetes	Bacteroidia	Bacteroidales	Porphyromonadaceae	Porphyromonas	conserved hypothetical protein	cas7	mkCas0088	CAS-I-B			
2192591..2193887	-	BAG34481.1	AP009380.1	Porphyromonas gingivalis ATCC 33277	Bacteria	Bacteroidetes	Bacteroidia	Bacteroidales	Porphyromonadaceae	Porphyromonas	conserved hypothetical protein	cas8b6	mkCas0111	CAS-I-B			
2193897..2196018	-	BAG34482.1	AP009380.1	Porphyromonas gingivalis ATCC 33277	Bacteria	Bacteroidetes	Bacteroidia	Bacteroidales	Porphyromonadaceae	Porphyromonas	probable CRISPR-associated helicase Cas3 core	cas3HD,cas3	cd09641,COG1203	CAS-I			
2196034..2196622	-	BAG34483.1	AP009380.1	Porphyromonas gingivalis ATCC 33277	Bacteria	Bacteroidetes	Bacteroidia	Bacteroidales	Porphyromonadaceae	Porphyromonas	hypothetical protein	cas5	mkCas0069	CAS-I-B			
2196628..2197294	-	BAG34484.1	AP009380.1	Porphyromonas gingivalis ATCC 33277	Bacteria	Bacteroidetes	Bacteroidia	Bacteroidales	Porphyromonadaceae	Porphyromonas	conserved hypothetical protein	cas6	cd09652	CAS-I,CAS-III			
372 CAS-I-B | PVZ08790.1																	
46130..48161	+	PVZ08789.1	QEKY01000011.1	Porphyromonas loveana	Bacteria	Bacteroidetes	Bacteroidia	Bacteroidales	Porphyromonadaceae	Porphyromonas	tetratricopeptide repeat protein	ATPase_01	icity0067				
48196..51116	array		QEKY01000011.1	Porphyromonas loveana	Bacteria	Bacteroidetes	Bacteroidia	Bacteroidales	Porphyromonadaceae	Porphyromonas		CRISPR array			45	35	30
51360..51624	-	PVZ08790.1	QEKY01000011.1	Porphyromonas loveana	Bacteria	Bacteroidetes	Bacteroidia	Bacteroidales	Porphyromonadaceae	Porphyromonas	CRISPR-associated Cas2 family protein	cas2	cd09725	CAS-I,CAS-II,CAS-III,CAS-V			
51623..52640	-	PVZ08791.1	QEKY01000011.1	Porphyromonas loveana	Bacteria	Bacteroidetes	Bacteroidia	Bacteroidales	Porphyromonadaceae	Porphyromonas	CRISPR-associated Cas1 family protein	cas1	cd09722	CAS-I-B			
52636..53185	-	PVZ08792.1	QEKY01000011.1	Porphyromonas loveana	Bacteria	Bacteroidetes	Bacteroidia	Bacteroidales	Porphyromonadaceae	Porphyromonas	CRISPR-associated Cas4 family exonuclease	cas4	pfam01930	CAS-I			
53250..54360	-	PVZ08793.1	QEKY01000011.1	Porphyromonas loveana	Bacteria	Bacteroidetes	Bacteroidia	Bacteroidales	Porphyromonadaceae	Porphyromonas	hypothetical protein	cas7	mkCas0088	CAS-I-B			
54371..55661	-	PVZ08794.1	QEKY01000011.1	Porphyromonas loveana	Bacteria	Bacteroidetes	Bacteroidia	Bacteroidales	Porphyromonadaceae	Porphyromonas	hypothetical protein	cas8b6	mkCas0111	CAS-I-B			

55673..57797	-	PVZ08795.1	QEKY01000011.1	Porphyromonas loveana	Bacteria	Bacteroidetes	Bacteroidia	Bacteroidales	Porphyromonadaceae	Porphyromonas	CRISPR-associated endonuclease/helicase Cas3	cas3HD,cas3	cd09641,cd09639	CAS-I			
57793..58378	-	PVZ08796.1	QEKY01000011.1	Porphyromonas loveana	Bacteria	Bacteroidetes	Bacteroidia	Bacteroidales	Porphyromonadaceae	Porphyromonas	hypothetical protein	cas5	mkCas0069	CAS-I-B			
58396..59062	-	PVZ08797.1	QEKY01000011.1	Porphyromonas loveana	Bacteria	Bacteroidetes	Bacteroidia	Bacteroidales	Porphyromonadaceae	Porphyromonas	CRISPR-associated Cas6 family protein	cas6	cd09652	CAS-I,CAS-III			

373 partial CAS-I-B | GAD05596.1

31..3842	array		BAOU01000035.1	Porphyromonas crevioricanis JCM 15906	Bacteria	Bacteroidetes	Bacteroidia	Bacteroidales	Porphyromonadaceae	Porphyromonas		CRISPR array			58	36	30
44..257	+	GAD05592.1	BAOU01000035.1	Porphyromonas crevioricanis JCM 15906	Bacteria	Bacteroidetes	Bacteroidia	Bacteroidales	Porphyromonadaceae	Porphyromonas	hypothetical protein						
268..403	-	GAD05593.1	BAOU01000035.1	Porphyromonas crevioricanis JCM 15906	Bacteria	Bacteroidetes	Bacteroidia	Bacteroidales	Porphyromonadaceae	Porphyromonas	hypothetical protein						
1815..1971	-	GAD05594.1	BAOU01000035.1	Porphyromonas crevioricanis JCM 15906	Bacteria	Bacteroidetes	Bacteroidia	Bacteroidales	Porphyromonadaceae	Porphyromonas	hypothetical protein						
3729..3849	-	GAD05595.1	BAOU01000035.1	Porphyromonas crevioricanis JCM 15906	Bacteria	Bacteroidetes	Bacteroidia	Bacteroidales	Porphyromonadaceae	Porphyromonas	hypothetical protein						
4064..4328	-	GAD05596.1	BAOU01000035.1	Porphyromonas crevioricanis JCM 15906	Bacteria	Bacteroidetes	Bacteroidia	Bacteroidales	Porphyromonadaceae	Porphyromonas	CRISPR-associated protein Cas2	cas2	cd09725	CAS-I,CAS-II,CAS-III,CAS-V			
4327..5344	-	GAD05597.1	BAOU01000035.1	Porphyromonas crevioricanis JCM 15906	Bacteria	Bacteroidetes	Bacteroidia	Bacteroidales	Porphyromonadaceae	Porphyromonas	CRISPR-associated protein Cas1	cas1	cd09722	CAS-I-B			
5340..5772	-	GAD05598.1	BAOU01000035.1	Porphyromonas crevioricanis JCM 15906	Bacteria	Bacteroidetes	Bacteroidia	Bacteroidales	Porphyromonadaceae	Porphyromonas	CRISPR-associated RecB family exonuclease Cas4a	cas4	pfam01930	CAS-I			

374 partial CAS-I-B | KGN88693.1

54360..54578	array		JQJB01000013.1	Porphyromonas crevioricanis	Bacteria	Bacteroidetes	Bacteroidia	Bacteroidales	Porphyromonadaceae	Porphyromonas		CRISPR array			4	45	21
54727..54988	+	KGN88690.1	JQJB01000013.1	Porphyromonas crevioricanis	Bacteria	Bacteroidetes	Bacteroidia	Bacteroidales	Porphyromonadaceae	Porphyromonas	hypothetical protein						
55433..55856	-	KGN88691.1	JQJB01000013.1	Porphyromonas crevioricanis	Bacteria	Bacteroidetes	Bacteroidia	Bacteroidales	Porphyromonadaceae	Porphyromonas	hypothetical protein						
55873..56398	-	KGN88692.1	JQJB01000013.1	Porphyromonas crevioricanis	Bacteria	Bacteroidetes	Bacteroidia	Bacteroidales	Porphyromonadaceae	Porphyromonas	hypothetical protein						
57513..59128	array		JQJB01000013.1	Porphyromonas crevioricanis	Bacteria	Bacteroidetes	Bacteroidia	Bacteroidales	Porphyromonadaceae	Porphyromonas		CRISPR array			25	36	30
59350..59614	-	KGN88693.1	JQJB01000013.1	Porphyromonas crevioricanis	Bacteria	Bacteroidetes	Bacteroidia	Bacteroidales	Porphyromonadaceae	Porphyromonas	CRISPR-associated protein Cas2	cas2	cd09725	CAS-I,CAS-II,CAS-III,CAS-V			
59613..60630	-	KGN88694.1	JQJB01000013.1	Porphyromonas crevioricanis	Bacteria	Bacteroidetes	Bacteroidia	Bacteroidales	Porphyromonadaceae	Porphyromonas	CRISPR-associated protein Cas1	cas1	cd09722	CAS-I-B			
60626..61136	-	KGN88695.1	JQJB01000013.1	Porphyromonas crevioricanis	Bacteria	Bacteroidetes	Bacteroidia	Bacteroidales	Porphyromonadaceae	Porphyromonas	CRISPR-associated protein Cas4	cas4	pfam01930	CAS-I			

375 partial CAS-I-B | SQH73697.1

1795617..1796127	+	SQH73695.1	LS483447.1	Porphyromonas crevioricanis	Bacteria	Bacteroidetes	Bacteroidia	Bacteroidales	Porphyromonadaceae	Porphyromonas	CRISPR-associated protein Cas4	cas4	pfam01930	CAS-I			
1796123..1797140	+	SQH73696.1	LS483447.1	Porphyromonas crevioricanis	Bacteria	Bacteroidetes	Bacteroidia	Bacteroidales	Porphyromonadaceae	Porphyromonas	CRISPR-associated endonuclease Cas1, subtype I-B/HMARI/TNEAP	cas1	cd09722	CAS-I-B			
1797139..1797403	+	SQH73697.1	LS483447.1	Porphyromonas crevioricanis	Bacteria	Bacteroidetes	Bacteroidia	Bacteroidales	Porphyromonadaceae	Porphyromonas	CRISPR-associated endoribonuclease Cas2	cas2	cd09725	CAS-I,CAS-II,CAS-III,CAS-V			
1797627..1800033	array		LS483447.1	Porphyromonas crevioricanis	Bacteria	Bacteroidetes	Bacteroidia	Bacteroidales	Porphyromonadaceae	Porphyromonas		CRISPR array			37	36	30

376 CAS-I-B | SDF11929.1

66878..67676	+	SDF11653.1	FNBQ01000003.1	Bacteroidales bacterium KHT7	Bacteria	Bacteroidetes	Bacteroidia	Bacteroidales			CRISPR-associated endoribonuclease Cas6	cas6	COG1583	CAS-I,CAS-III,CAS-IV			
67750..69610	+	SDF11703.1	FNBQ01000003.1	Bacteroidales bacterium KHT7	Bacteria	Bacteroidetes	Bacteroidia	Bacteroidales			CRISPR-associated protein Csh1	cas8b1	mkCas0106	CAS-I-B			
69621..70641	+	SDF11739.1	FNBQ01000003.1	Bacteroidales bacterium KHT7	Bacteria	Bacteroidetes	Bacteroidia	Bacteroidales			CRISPR-associated protein Csh2	cas7b	pfam05107	CAS-I-B,CAS-I-C			
70643..71423	+	SDF11770.1	FNBQ01000003.1	Bacteroidales bacterium KHT7	Bacteria	Bacteroidetes	Bacteroidia	Bacteroidales			CRISPR-associated protein Cas5h	cas5	cls001430	CAS-I-B			
71430..74142	+	SDF11815.1	FNBQ01000003.1	Bacteroidales bacterium KHT7	Bacteria	Bacteroidetes	Bacteroidia	Bacteroidales			CRISPR-associated endonuclease/helicase Cas3	cas3HD,cas3	cd09641,COG1203	CAS-I			
74144..74657	+	SDF11853.1	FNBQ01000003.1	Bacteroidales bacterium KHT7	Bacteria	Bacteroidetes	Bacteroidia	Bacteroidales			CRISPR-associated exonuclease, Cas4 family	cas4	pfam01930	CAS-I			
74659..75676	+	SDF11891.1	FNBQ01000003.1	Bacteroidales bacterium KHT7	Bacteria	Bacteroidetes	Bacteroidia	Bacteroidales			CRISPR-associated protein, Cas1 family	cas1	cd09722	CAS-I-B			
75737..76001	+	SDF11929.1	FNBQ01000003.1	Bacteroidales bacterium KHT7	Bacteria	Bacteroidetes	Bacteroidia	Bacteroidales			CRISPR-associated protein, Cas2 family	cas2	COG1343	CAS-I,CAS-II,CAS-III			
76209..76764	array		FNBQ01000003.1	Bacteroidales bacterium KHT7	Bacteria	Bacteroidetes	Bacteroidia	Bacteroidales				CRISPR array			9	35	30

377 CAS-I-B | EGN57514.1

2367504..2368180	array		GL945017.1	Prevotella multisaccharivorax DSM 17128	Bacteria	Bacteroidetes	Bacteroidia	Bacteroidales	Prevotellaceae	Prevotella		CRISPR array			11	34	30
2368272..2369737	+	EGN57513.1	GL945017.1	Prevotella multisaccharivorax DSM 17128	Bacteria	Bacteroidetes	Bacteroidia	Bacteroidales	Prevotellaceae	Prevotella	transposase						
2369819..2378638	array		GL945017.1	Prevotella multisaccharivorax DSM 17128	Bacteria	Bacteroidetes	Bacteroidia	Bacteroidales	Prevotellaceae	Prevotella		CRISPR array			136	35	30
2378852..2379116	-	EGN57514.1	GL945017.1	Prevotella multisaccharivorax DSM 17128	Bacteria	Bacteroidetes	Bacteroidia	Bacteroidales	Prevotellaceae	Prevotella	CRISPR-associated protein, Cas2 family	cas2	cd09725	CAS-I,CAS-II,CAS-III,CAS-V			
2379119..2380136	-	EGN57515.1	GL945017.1	Prevotella multisaccharivorax DSM 17128	Bacteria	Bacteroidetes	Bacteroidia	Bacteroidales	Prevotellaceae	Prevotella	CRISPR-associated protein, Cas1 family	cas1	cd09722	CAS-I-B			
2380132..2380672	-	EGN57516.1	GL945017.1	Prevotella multisaccharivorax DSM 17128	Bacteria	Bacteroidetes	Bacteroidia	Bacteroidales	Prevotellaceae	Prevotella	CRISPR-associated exonuclease, Cas4 family	cas4	pfam01930	CAS-I			
2380727..2381792	-	EGN57517.1	GL945017.1	Prevotella multisaccharivorax DSM 17128	Bacteria	Bacteroidetes	Bacteroidia	Bacteroidales	Prevotellaceae	Prevotella	hypothetical protein	cas7	mkCas0088	CAS-I-B			
2381799..2383116	-	EGN57518.1	GL945017.1	Prevotella multisaccharivorax DSM 17128	Bacteria	Bacteroidetes	Bacteroidia	Bacteroidales	Prevotellaceae	Prevotella	hypothetical protein	cas8b6	mkCas0111	CAS-I-B			
2383132..2385274	-	EGN57519.1	GL945017.1	Prevotella multisaccharivorax DSM 17128	Bacteria	Bacteroidetes	Bacteroidia	Bacteroidales	Prevotellaceae	Prevotella	CRISPR-associated helicase, Cas3 family	cas3HD,cas3	cd09641,COG1203	CAS-I			
2385275..2385833	-	EGN57520.1	GL945017.1	Prevotella multisaccharivorax DSM 17128	Bacteria	Bacteroidetes	Bacteroidia	Bacteroidales	Prevotellaceae	Prevotella	hypothetical protein	cas5	mkCas0069	CAS-I-B			
2385842..2386508	-	EGN57521.1	GL945017.1	Prevotella multisaccharivorax DSM 17128	Bacteria	Bacteroidetes	Bacteroidia	Bacteroidales	Prevotellaceae	Prevotella	CRISPR-associated protein, Cas6 family	cas6	cd09652	CAS-I,CAS-III			

379 CAS-I-B | BBD45981.1

2367997..2368786	+	BBD45973.1	AP018040.2	Petrimonas sp. IBARAKI	Bacteria	Bacteroidetes	Bacteroidia	Bacteroidales	Porphyromonadaceae	Petrimonas	CRISPR-associated endoribonuclease	cas6	cd09759	CAS-I-A			
2368921..2369581	+	BBD45974.1	AP018040.2	Petrimonas sp. IBARAKI	Bacteria	Bacteroidetes	Bacteroidia	Bacteroidales	Porphyromonadaceae	Petrimonas	DNA-binding protein	cas6	cd09652	CAS-I,CAS-III			
2369587..2370175	+	BBD45975.1	AP018040.2	Petrimonas sp. IBARAKI	Bacteria	Bacteroidetes	Bacteroidia	Bacteroidales	Porphyromonadaceae	Petrimonas	hypothetical protein	cas5	mkCas0069	CAS-I-B			
2370174..2372316	+	BBD45976.1	AP018040.2	Petrimonas sp. IBARAKI	Bacteria	Bacteroidetes	Bacteroidia	Bacteroidales	Porphyromonadaceae	Petrimonas	metal dependent phosphohydrolase	cas3HD,cas3	cd09641,COG1203	CAS-I			
2372312..2373632	+	BBD45977.1	AP018040.2	Petrimonas sp. IBARAKI	Bacteria	Bacteroidetes	Bacteroidia	Bacteroidales	Porphyromonadaceae	Petrimonas	Hypothetical protein	cas8b6	mkCas0111	CAS-I-B			
2373656..2374709	+	BBD45978.1	AP018040.2	Petrimonas sp. IBARAKI	Bacteria	Bacteroidetes	Bacteroidia	Bacteroidales	Porphyromonadaceae	Petrimonas	hypothetical protein	cas7	mkCas0088	CAS-I-B			
2374746..2375277	+	BBD45979.1	AP018040.2	Petrimonas sp. IBARAKI	Bacteria	Bacteroidetes	Bacteroidia	Bacteroidales	Porphyromonadaceae	Petrimonas	CRISPR-associated protein Cas4	cas4	pfam01930	CAS-I			
2375343..2376360	+	BBD45980.1	AP018040.2	Petrimonas sp. IBARAKI	Bacteria	Bacteroidetes	Bacteroidia	Bacteroidales	Porphyromonadaceae	Petrimonas	CRISPR-associated endonuclease Cas1, HMARI/TNEAPsubtype	cas1	cd09722	CAS-I-B			
2376361..2376625	+	BBD45981.1	AP018040.2	Petrimonas sp. IBARAKI	Bacteria	Bacteroidetes	Bacteroidia	Bacteroidales	Porphyromonadaceae	Petrimonas	CRISPR-associated protein Cas2	cas2	cd09725	CAS-I,CAS-II,CAS-III,CAS-V			
2376836..2381247	array		AP018040.2	Petrimonas sp. IBARAKI	Bacteria	Bacteroidetes	Bacteroidia	Bacteroidales	Porphyromonadaceae	Petrimonas		CRISPR array			68	35	30

380 CAS-III-A/CAS-I-B | AEW20121.1

2491035..2491836	+	AEW21051.1	CP003191.1	Tannerella forsythia 92A2	Bacteria	Bacteroidetes	Bacteroidia	Bacteroidales	Tannerellaceae	Tannerella	CRISPR-associated endoribonuclease Cas6	cas6	COG1583	CAS-I,CAS-III,CAS-IV			
2491861..2492485	+	AEW20687.1	CP003191.1	Tannerella forsythia 92A2	Bacteria	Bacteroidetes	Bacteroidia	Bacteroidales	Tannerellaceae	Tannerella	hypothetical protein						
2492552..2493278	+	AEW21544.1	CP003191.1	Tannerella forsythia 92A2	Bacteria	Bacteroidetes	Bacteroidia	Bacteroidales	Tannerellaceae	Tannerella	CRISPR-associated protein, Cas5p family	cas5	cd09716	CAS-I-B			
2493270..2494812	+	AEW21654.1	CP003191.1	Tannerella forsythia 92A2	Bacteria	Bacteroidetes	Bacteroidia	Bacteroidales	Tannerellaceae	Tannerella	CRISPR-associated protein, Csp2 family	cas8b4	cd09715	CAS-I-B			
2494831..2495749	+	AEW22456.1	CP003191.1	Tannerella forsythia 92A2	Bacteria	Bacteroidetes	Bacteroidia	Bacteroidales	Tannerellaceae	Tannerella	CRISPR-associated protein, Csp1 family	cas7b	cd09717	CAS-I-B			
2495936..2498021	+	AEW20272.1	CP003191.1	Tannerella forsythia 92A2	Bacteria	Bacteroidetes	Bacteroidia	Bacteroidales	Tannerellaceae	Tannerella	CRISPR-associated helicase Cas3	cas3	cd09639	CAS-I			
2498053..2498608	+	AEW20522.1	CP003191.1	Tannerella forsythia 92A2	Bacteria	Bacteroidetes	Bacteroidia	Bacteroidales	Tannerellaceae	Tannerella	hypothetical protein						
2498630..2499629	+	AEW21219.1	CP003191.1	Tannerella forsythia 92A2	Bacteria	Bacteroidetes	Bacteroidia	Bacteroidales	Tannerellaceae	Tannerella	Fic family protein	casR	cls001593	CAS-I			
2499903..2500434	+	AEW22266.1	CP003191.1	Tannerella forsythia 92A2	Bacteria	Bacteroidetes	Bacteroidia	Bacteroidales	Tannerellaceae	Tannerella	CRISPR-associated protein Cas4	cas4	pfam01930	CAS-I			
2500480..2501923	+	AEW19973.1	CP003191.1	Tannerella forsythia 92A2	Bacteria	Bacteroidetes	Bacteroidia	Bacteroidales	Tannerellaceae	Tannerella	hypothetical protein	cas10	cd09680	CAS-III			
2501928..2502399	+	AEW20762.1	CP003191.1	Tannerella forsythia 92A2	Bacteria	Bacteroidetes	Bacteroidia	Bacteroidales	Tannerellaceae	Tannerella	CRISPR-associated protein, Csm2 family	csm2gr11	pfam03750	CAS-III-A			
2502408..2503047	+	AEW20734.1	CP003191.1	Tannerella forsythia 92A2	Bacteria	Bacteroidetes	Bacteroidia	Bacteroidales	Tannerellaceae	Tannerella	CRISPR-associated RAMP protein, Csm3 family	csm3gr7	cd09684	CAS-III-A,CAS-III-D			
2503046..2504042	+	AEW19746.1	CP003191.1	Tannerella forsythia 92A2	Bacteria	Bacteroidetes	Bacteroidia	Bacteroidales	Tannerellaceae	Tannerella	CRISPR-associated RAMP protein, Csm4 family	csm4gr5	cd09663	CAS-III-A			
2504054..2505209	+	AEW22620.1	CP003191.1	Tannerella forsythia 92A2	Bacteria	Bacteroidetes	Bacteroidia	Bacteroidales	Tannerellaceae	Tannerella	CRISPR-associated RAMP protein, Csm5 family	csm5gr7	COG1332	CAS-III-A			
2505210..2506455	+	AEW21781.1	CP003191.1	Tannerella forsythia 92A2	Bacteria	Bacteroidetes	Bacteroidia	Bacteroidales	Tannerellaceae	Tannerella	CRISPR-associated protein, TM1812 family	csx1	cd09732	CAS-III			
2506847..2507864	+	AEW20944.1	CP003191.1	Tannerella forsythia 92A2	Bacteria	Bacteroidetes	Bacteroidia	Bacteroidales	Tannerellaceae	Tannerella	CRISPR-associated endonuclease Cas1, HMARI/TNEAP subtype	cas1	cd09722	CAS-I-B			
2507863..2508127	+	AEW20121.1	CP003191.1	Tannerella forsythia 92A2	Bacteria	Bacteroidetes	Bacteroidia	Bacteroidales	Tannerellaceae	Tannerella	CRISPR-associated protein Cas2	cas2	cd09725	CAS-I,CAS-II,CAS-III,CAS-V			
2508338..2513170	array		CP003191.1	Tannerella forsythia 92A2	Bacteria	Bacteroidetes	Bacteroidia	Bacteroidales	Tannerellaceae	Tannerella		CRISPR array			73	36	30

381 CAS-I-B/CAS-III-A | EPT33673.1

38660..40886	array		AUTU01000017.1	Bacteroidetes bacterium oral taxon 272 str. F0290	Bacteria	Bacteroidetes						CRISPR array			35	35	29
41095..41359	-	EPT33673.1	AUTU01000017.1	Bacteroidetes bacterium oral taxon 272 str. F0290	Bacteria	Bacteroidetes					putative CRISPR-associated endonuclease Cas2	cas2	cd09725	CAS-I,CAS-II,CAS-III,CAS-V			
41358..42375	-	EPT33620.1	AUTU01000017.1	Bacteroidetes bacterium oral taxon 272 str. F0290	Bacteria	Bacteroidetes					CRISPR-associated endonuclease Cas1, subtype I-B/HMARI/TNEAP	cas1	cd09722	CAS-I-B			
42371..43319	-	EPT33621.1	AUTU01000017.1	Bacteroidetes bacterium oral taxon 272 str. F0290	Bacteria	Bacteroidetes					hypothetical protein	PD-DExK	mkCas0090	CAS-I,CAS-III			
43315..44791	-	EPT33628.1	AUTU01000017.1	Bacteroidetes bacterium oral taxon 272 str. F0290	Bacteria	Bacteroidetes					CRISPR type III-A/MTUBE-associated RAMP protein Csm5	csm5gr7	cd09662	CAS-III-A			
44933..45947	-	EPT33665.1	AUTU01000017.1	Bacteroidetes bacterium oral taxon 272 str. F0290	Bacteria	Bacteroidetes					CRISPR type III-A/MTUBE-associated RAMP protein Csm4	csm4gr5	COG1567	CAS-III-A			
45943..46636	-	EPT33661.1	AUTU01000017.1	Bacteroidetes bacterium oral taxon 272 str. F0290	Bacteria	Bacteroidetes					CRISPR type III-A/MTUBE-associated RAMP protein Csm3	csm3gr7	cd09684	CAS-III-A,CAS-III-D			
47047..48643	-	EPT33654.1	AUTU01000017.1	Bacteroidetes bacterium oral taxon 272 str. F0290	Bacteria	Bacteroidetes					hypothetical protein	cas10	cd09680	CAS-III			
48689..49220	-	EPT33619.1	AUTU01000017.1	Bacteroidetes bacterium oral taxon 272 str. F0290	Bacteria	Bacteroidetes					CRISPR-associated protein Cas4	cas4	pfam01930	CAS-I			
49230..49767	-	EPT33674.1	AUTU01000017.1	Bacteroidetes bacterium oral taxon 272 str. F0290	Bacteria	Bacteroidetes					hypothetical protein						
49773..52257	-	EPT33668.1	AUTU01000017.1	Bacteroidetes bacterium oral taxon 272 str. F0290	Bacteria	Bacteroidetes					CRISPR-associated helicase Cas3	cas3	COG1203	CAS-I			
52241..52952	-	EPT33611.1	AUTU01000017.1	Bacteroidetes bacterium oral taxon 272 str. F0290	Bacteria	Bacteroidetes					CRISPR-associated protein Cas5	cas5	cd09692	CAS-I-B			
52948..53902	-	EPT33657.1	AUTU01000017.1	Bacteroidetes bacterium oral taxon 272 str. F0290	Bacteria	Bacteroidetes					PF05107 family	cas7b	pfam05107	CAS-I-B,CAS-I-C			
53914..55894	-	EPT33603.1	AUTU01000017.1	Bacteroidetes bacterium oral taxon 272 str. F0290	Bacteria	Bacteroidetes					hypothetical protein	Cas8b1	icity0005				
56255..56879	-	EPT33658.1	AUTU01000017.1	Bacteroidetes bacterium oral taxon 272 str. F0290	Bacteria	Bacteroidetes					hypothetical protein						
56895..57690	-	EPT33622.1	AUTU01000017.1	Bacteroidetes bacterium oral taxon 272 str. F0290	Bacteria	Bacteroidetes					CRISPR-associated endoribonuclease Cas6	cas6	COG1583	CAS-I,CAS-III,CAS-IV			

382 CAS-I-B | SEA34640.1

26632..28336	array		FNQW01000036.1	Porphyromonadaceae bacterium KH3R12	Bacteria	Bacteroidetes	Bacteroidia	Bacteroidales	Porphyromonadaceae			CRISPR array			27	35	29
29498..30621	array		FNQW01000036.1	Porphyromonadaceae bacterium KH3R12	Bacteria	Bacteroidetes	Bacteroidia	Bacteroidales	Porphyromonadaceae			CRISPR array			18	35	29
30833..31100	-	SEA34640.1	FNQW01000036.1	Porphyromonadaceae bacterium KH3R12	Bacteria	Bacteroidetes	Bacteroidia	Bacteroidales	Porphyromonadaceae		CRISPR-associated protein, Cas2 family	cas2	cd09725	CAS-I,CAS-II,CAS-III,CAS-V			
31100..32120	-	SEA34657.1	FNQW01000036.1	Porphyromonadaceae bacterium KH3R12	Bacteria	Bacteroidetes	Bacteroidia	Bacteroidales	Porphyromonadaceae		CRISPR-associated protein, Cas1 family	cas1	cd09722	CAS-I-B			
32116..32629	-	SEA34675.1	FNQW01000036.1	Porphyromonadaceae bacterium KH3R12	Bacteria	Bacteroidetes	Bacteroidia	Bacteroidales	Porphyromonadaceae		CRISPR-associated exonuclease, Cas4 family	cas4	pfam01930	CAS-I			
32721..35184	-	SEA34693.1	FNQW01000036.1	Porphyromonadaceae bacterium KH3R12	Bacteria	Bacteroidetes	Bacteroidia	Bacteroidales	Porphyromonadaceae		CRISPR-associated helicase, Cas3 family	cas3HD,cas3	cd09641,COG1203	CAS-I			
35168..35879	-	SEA34707.1	FNQW01000036.1	Porphyromonadaceae bacterium KH3R12	Bacteria	Bacteroidetes	Bacteroidia	Bacteroidales	Porphyromonadaceae		CRISPR-associated protein Cas5, subtype I-B/HMARI	cas5	cls001430	CAS-I-B			
35875..36826	-	SEA34730.1	FNQW01000036.1	Porphyromonadaceae bacterium KH3R12	Bacteria	Bacteroidetes	Bacteroidia	Bacteroidales	Porphyromonadaceae		CRISPR-associated protein, Csh2 family	cas7b	pfam05107	CAS-I-B,CAS-I-C			
36838..38989	-	SEA34754.1	FNQW01000036.1	Porphyromonadaceae bacterium KH3R12	Bacteria	Bacteroidetes	Bacteroidia	Bacteroidales	Porphyromonadaceae		hypothetical protein	Cas8b1	icity0005				
39042..39813	-	SEA34769.1	FNQW01000036.1	Porphyromonadaceae bacterium KH3R12	Bacteria	Bacteroidetes	Bacteroidia	Bacteroidales	Porphyromonadaceae		CRISPR-associated protein, Cas6 family	cas6	COG1583	CAS-I,CAS-III,CAS-IV			

383 CAS-III-A/CAS-I-B | PLB85966.1

28412..29198	+	PLB85970.1	PKMK01000022.1	Dysgonamonadaceae bacterium	Bacteria	Bacteroidetes	Bacteroidia	Bacteroidales	Dysgonamonadaceae		CRISPR-associated endoribonuclease Cas6	cas6	COG1583	CAS-I,CAS-III,CAS-IV			
29214..30654	+	PLB85952.1	PKMK01000022.1	Dysgonamonadaceae bacterium	Bacteria	Bacteroidetes	Bacteroidia	Bacteroidales	Dysgonamonadaceae		type I-B CRISPR-associated protein Cas8b1/Cst1	cas8b2	cls000029	CAS-I-B			
30663..31764	+	PLB85953.1	PKMK01000022.1	Dysgonamonadaceae bacterium	Bacteria	Bacteroidetes	Bacteroidia	Bacteroidales	Dysgonamonadaceae		type I-B CRISPR-associated protein Cas7/Cst2/DevR	cas7	cd09687	CAS-I-B			
31763..32432	+	PLB85954.1	PKMK01000022.1	Dysgonamonadaceae bacterium	Bacteria	Bacteroidetes	Bacteroidia	Bacteroidales	Dysgonamonadaceae		type I-B CRISPR-associated protein Cas5	cas5	COG1688	CAS-I			
32428..34714	+	PLB85955.1	PKMK01000022.1	Dysgonamonadaceae bacterium	Bacteria	Bacteroidetes	Bacteroidia	Bacteroidales	Dysgonamonadaceae		CRISPR-associated helicase/endonuclease Cas3	cas3	COG1203	CAS-I			
34837..35455	+	PLB85956.1	PKMK01000022.1	Dysgonamonadaceae bacterium	Bacteria	Bacteroidetes	Bacteroidia	Bacteroidales	Dysgonamonadaceae		plasmid pRiA4b ORF-3 family protein						
35485..35992	+	PLB85957.1	PKMK01000022.1	Dysgonamonadaceae bacterium	Bacteria	Bacteroidetes	Bacteroidia	Bacteroidales	Dysgonamonadaceae		CRISPR-associated protein Cas4	cas4	pfam01930	CAS-I			
36013..37492	+	PLB85958.1	PKMK01000022.1	Dysgonamonadaceae bacterium	Bacteria	Bacteroidetes	Bacteroidia	Bacteroidales	Dysgonamonadaceae		CRISPR-associated protein Cas10	cas10,cas10,cas10	cd09680,cd09680,cls000742	CAS-III			
37520..37943	+	PLB85959.1	PKMK01000022.1	Dysgonamonadaceae bacterium	Bacteria	Bacteroidetes	Bacteroidia	Bacteroidales	Dysgonamonadaceae		type III-A CRISPR-associated protein Csm2	csm2gr11	pfam03750	CAS-III-A			
37969..38629	+	PLB85960.1	PKMK01000022.1	Dysgonamonadaceae bacterium	Bacteria	Bacteroidetes	Bacteroidia	Bacteroidales	Dysgonamonadaceae		type III-A CRISPR-associated RAMP protein Csm3	csm3gr7	cd09684	CAS-III-A,CAS-III-D			
38625..39675	+	PLB85961.1	PKMK01000022.1	Dysgonamonadaceae bacterium	Bacteria	Bacteroidetes	Bacteroidia	Bacteroidales	Dysgonamonadaceae		type III-A CRISPR-associated RAMP protein Csm4	csm4gr5	COG1567	CAS-III-A			
39675..40860	+	PLB85962.1	PKMK01000022.1	Dysgonamonadaceae bacterium	Bacteria	Bacteroidetes	Bacteroidia	Bacteroidales	Dysgonamonadaceae		type III-A CRISPR-associated RAMP protein Csm5	csm5gr7	COG1332	CAS-III-A			
40856..41963	+	PLB85963.1	PKMK01000022.1	Dysgonamonadaceae bacterium	Bacteria	Bacteroidetes	Bacteroidia	Bacteroidales	Dysgonamonadaceae		CRISPR-associated protein	csm6	cd09742	CAS-III-A,CAS-III-D			
41975..42353	+	PLB85964.1	PKMK01000022.1	Dysgonamonadaceae bacterium	Bacteria	Bacteroidetes	Bacteroidia	Bacteroidales	Dysgonamonadaceae		CRISPR-associated protein	csx20	cls000118	CAS-I,CAS-III			
42356..43373	+	PLB85965.1	PKMK01000022.1	Dysgonamonadaceae bacterium	Bacteria	Bacteroidetes	Bacteroidia	Bacteroidales	Dysgonamonadaceae		subtype I-B CRISPR-associated endonuclease Cas1	cas1	cd09722	CAS-I-B			
43377..43641	+	PLB85966.1	PKMK01000022.1	Dysgonamonadaceae bacterium	Bacteria	Bacteroidetes	Bacteroidia	Bacteroidales	Dysgonamonadaceae		CRISPR-associated endonuclease Cas2	cas2	cd09725	CAS-I,CAS-II,CAS-III,CAS-V			
43855..45375	array		PKMK01000022.1	Dysgonamonadaceae bacterium	Bacteria	Bacteroidetes	Bacteroidia	Bacteroidales	Dysgonamonadaceae			CRISPR array			24	35	29

384 CAS-I-B | PLB87385.1

76824..77613	+	PLB87500.1	PKMK01000001.1	Dysgonamonadaceae bacterium	Bacteria	Bacteroidetes	Bacteroidia	Bacteroidales	Dysgonamonadaceae		CRISPR-associated endoribonuclease Cas6	cas6	COG1583	CAS-I,CAS-III,CAS-IV			
77624..78359	+	PLB87380.1	PKMK01000001.1	Dysgonamonadaceae bacterium	Bacteria	Bacteroidetes	Bacteroidia	Bacteroidales	Dysgonamonadaceae		type I-PGING CRISPR-associated protein Cas5p	cas5	cd09716	CAS-I-B			
78355..79918	+	PLB87381.1	PKMK01000001.1	Dysgonamonadaceae bacterium	Bacteria	Bacteroidetes	Bacteroidia	Bacteroidales	Dysgonamonadaceae		type I-PGING CRISPR-associated protein Cas8c/Csp2	cas8b4	cd09715	CAS-I-B			
79945..80935	+	PLB87382.1	PKMK01000001.1	Dysgonamonadaceae bacterium	Bacteria	Bacteroidetes	Bacteroidia	Bacteroidales	Dysgonamonadaceae		type I-PGING CRISPR-associated protein Cas7/Csp1	cas7b,cas7b	cd09717,cd09717	CAS-I-B			
81125..83174	+	PLB87501.1	PKMK01000001.1	Dysgonamonadaceae bacterium	Bacteria	Bacteroidetes	Bacteroidia	Bacteroidales	Dysgonamonadaceae		CRISPR-associated helicase Cas3'	cas3	cd09639	CAS-I			
83267..83885	+	PLB87502.1	PKMK01000001.1	Dysgonamonadaceae bacterium	Bacteria	Bacteroidetes	Bacteroidia	Bacteroidales	Dysgonamonadaceae		plasmid pRiA4b ORF-3 family protein						
83915..84434	+	PLB87383.1	PKMK01000001.1	Dysgonamonadaceae bacterium	Bacteria	Bacteroidetes	Bacteroidia	Bacteroidales	Dysgonamonadaceae		CRISPR-associated protein Cas4	cas4	pfam01930	CAS-I			
84457..85474	+	PLB87384.1	PKMK01000001.1	Dysgonamonadaceae bacterium	Bacteria	Bacteroidetes	Bacteroidia	Bacteroidales	Dysgonamonadaceae		subtype I-B CRISPR-associated endonuclease Cas1	cas1	cd09722	CAS-I-B			
85527..85791	+	PLB87385.1	PKMK01000001.1	Dysgonamonadaceae bacterium	Bacteria	Bacteroidetes	Bacteroidia	Bacteroidales	Dysgonamonadaceae		CRISPR-associated endonuclease Cas2	cas2	cd09725	CAS-I,CAS-II,CAS-III,CAS-V			
86005..93463	array		PKMK01000001.1	Dysgonamonadaceae bacterium	Bacteria	Bacteroidetes	Bacteroidia	Bacteroidales	Dysgonamonadaceae			CRISPR array			115	36	29

387 CAS-I-B | OIN58179.1

263012..269163	array		MORL01000008.1	Arsenicibacter rosenii	Bacteria	Bacteroidetes	Cytophagia	Cytophagales	Cytophagaceae	Arsenicibacter		CRISPR array			94	35	30
269378..269642	-	OIN58179.1	MORL01000008.1	Arsenicibacter rosenii	Bacteria	Bacteroidetes	Cytophagia	Cytophagales	Cytophagaceae	Arsenicibacter	CRISPR-associated endonuclease Cas2	cas2	COG1343	CAS-I,CAS-II,CAS-III			

269739..270747	-	OIN58180.1	MORL01000008.1	Arsenicibacter rosenii	Bacteria	Bacteroidetes	Cytophagia	Cytophagales	Cytophagaceae	Arsenicibacter	subtype I-B CRISPR-associated endonuclease Cas1	cas1	cd09722	CAS-I-B			
270833..271337	-	OIN58181.1	MORL01000008.1	Arsenicibacter rosenii	Bacteria	Bacteroidetes	Cytophagia	Cytophagales	Cytophagaceae	Arsenicibacter	CRISPR-associated protein Cas4	cas4	pfam01930	CAS-I			
271340..271859	-	OIN58182.1	MORL01000008.1	Arsenicibacter rosenii	Bacteria	Bacteroidetes	Cytophagia	Cytophagales	Cytophagaceae	Arsenicibacter	hypothetical protein						
271871..274031	-	OIN58183.1	MORL01000008.1	Arsenicibacter rosenii	Bacteria	Bacteroidetes	Cytophagia	Cytophagales	Cytophagaceae	Arsenicibacter	CRISPR-associated helicase Cas3'	cas3	cd09639	CAS-I			
274623..275457	-	OIN58218.1	MORL01000008.1	Arsenicibacter rosenii	Bacteria	Bacteroidetes	Cytophagia	Cytophagales	Cytophagaceae	Arsenicibacter	type I-B CRISPR-associated protein Cas5	cas5	cls001430	CAS-I-B			
275465..276389	-	OIN58184.1	MORL01000008.1	Arsenicibacter rosenii	Bacteria	Bacteroidetes	Cytophagia	Cytophagales	Cytophagaceae	Arsenicibacter	CRISPR-associated protein	cas7b	pfam05107	CAS-I-B,CAS-I-C			
276401..278315	-	OIN58185.1	MORL01000008.1	Arsenicibacter rosenii	Bacteria	Bacteroidetes	Cytophagia	Cytophagales	Cytophagaceae	Arsenicibacter	hypothetical protein	cas8b1	mkCas0106	CAS-I-B			
278529..278820	+	OIN58186.1	MORL01000008.1	Arsenicibacter rosenii	Bacteria	Bacteroidetes	Cytophagia	Cytophagales	Cytophagaceae	Arsenicibacter	hypothetical protein						
278986..279205	-	OIN58187.1	MORL01000008.1	Arsenicibacter rosenii	Bacteria	Bacteroidetes	Cytophagia	Cytophagales	Cytophagaceae	Arsenicibacter	hypothetical protein						
279344..280103	-	OIN58188.1	MORL01000008.1	Arsenicibacter rosenii	Bacteria	Bacteroidetes	Cytophagia	Cytophagales	Cytophagaceae	Arsenicibacter	CRISPR-associated endoribonuclease Cas6	cas6	COG1583	CAS-I,CAS-III,CAS-IV			
280297..281296	-	OIN58189.1	MORL01000008.1	Arsenicibacter rosenii	Bacteria	Bacteroidetes	Cytophagia	Cytophagales	Cytophagaceae	Arsenicibacter	WYL domain-containing protein	WYL	pfam13280	CAS-I,CAS-III			
388 CAS-I-B | AUD05852.1																	
7325678..7329266	array		CP025096.1	Spirosoma pollinicola	Bacteria	Bacteroidetes	Cytophagia	Cytophagales	Cytophagaceae	Spirosoma		CRISPR array			55	39	26
7329486..7329750	-	AUD05852.1	CP025096.1	Spirosoma pollinicola	Bacteria	Bacteroidetes	Cytophagia	Cytophagales	Cytophagaceae	Spirosoma	CRISPR-associated endonuclease Cas2	cas2	cd09725	CAS-I,CAS-II,CAS-III,CAS-V			
7329762..7330776	-	AUD05853.1	CP025096.1	Spirosoma pollinicola	Bacteria	Bacteroidetes	Cytophagia	Cytophagales	Cytophagaceae	Spirosoma	subtype I-B CRISPR-associated endonuclease Cas1	cas1	cd09722	CAS-I-B			
7330821..7331334	-	AUD05854.1	CP025096.1	Spirosoma pollinicola	Bacteria	Bacteroidetes	Cytophagia	Cytophagales	Cytophagaceae	Spirosoma	CRISPR-associated protein Cas4	cas4	pfam01930	CAS-I			
7331338..7332349	-	AUD05855.1	CP025096.1	Spirosoma pollinicola	Bacteria	Bacteroidetes	Cytophagia	Cytophagales	Cytophagaceae	Spirosoma	DUF1016 domain-containing protein	PD-DExK,PD-DExK	COG4804,COG4804	CAS-I,CAS-III			
7332352..7334629	-	AUD05856.1	CP025096.1	Spirosoma pollinicola	Bacteria	Bacteroidetes	Cytophagia	Cytophagales	Cytophagaceae	Spirosoma	hypothetical protein	cas3HD,cas3	cd09641,COG1203	CAS-I			
7334635..7335340	-	AUD05857.1	CP025096.1	Spirosoma pollinicola	Bacteria	Bacteroidetes	Cytophagia	Cytophagales	Cytophagaceae	Spirosoma	hypothetical protein						
7335332..7336430	-	AUD05858.1	CP025096.1	Spirosoma pollinicola	Bacteria	Bacteroidetes	Cytophagia	Cytophagales	Cytophagaceae	Spirosoma	hypothetical protein	cas7	cd09687	CAS-I-B			
7336416..7338429	-	AUD05859.1	CP025096.1	Spirosoma pollinicola	Bacteria	Bacteroidetes	Cytophagia	Cytophagales	Cytophagaceae	Spirosoma	hypothetical protein	cas8b2	cls000029	CAS-I-B			
7338568..7339387	-	AUD05860.1	CP025096.1	Spirosoma pollinicola	Bacteria	Bacteroidetes	Cytophagia	Cytophagales	Cytophagaceae	Spirosoma	CRISPR-associated endoribonuclease Cas6	cas6	COG1583	CAS-I,CAS-III,CAS-IV			
7339530..7340511	+	AUD05861.1	CP025096.1	Spirosoma pollinicola	Bacteria	Bacteroidetes	Cytophagia	Cytophagales	Cytophagaceae	Spirosoma	hypothetical protein	WYL	COG2378	CAS-I,CAS-III			
389 CAS-I-B | OZI05906.1																	
215503..216538	-	OZI05897.1	NMPS01000220.1	Siphonobacter sp. BAB-5385	Bacteria	Bacteroidetes	Cytophagia	Cytophagales	Cytophagaceae	Siphonobacter	hypothetical protein	WYL	COG2378	CAS-I,CAS-III			
216633..217422	+	OZI05898.1	NMPS01000220.1	Siphonobacter sp. BAB-5385	Bacteria	Bacteroidetes	Cytophagia	Cytophagales	Cytophagaceae	Siphonobacter	CRISPR-associated endoribonuclease Cas6	cas6	COG1583	CAS-I,CAS-III,CAS-IV			
217743..219747	+	OZI05899.1	NMPS01000220.1	Siphonobacter sp. BAB-5385	Bacteria	Bacteroidetes	Cytophagia	Cytophagales	Cytophagaceae	Siphonobacter	hypothetical protein	cas8b2	cls000029	CAS-I-B			
219748..220849	+	OZI05900.1	NMPS01000220.1	Siphonobacter sp. BAB-5385	Bacteria	Bacteroidetes	Cytophagia	Cytophagales	Cytophagaceae	Siphonobacter	hypothetical protein	cas7	cd09687	CAS-I-B			
220841..221546	+	OZI05901.1	NMPS01000220.1	Siphonobacter sp. BAB-5385	Bacteria	Bacteroidetes	Cytophagia	Cytophagales	Cytophagaceae	Siphonobacter	hypothetical protein	cas5*	cd09645				
221536..221791	+	OZI05902.1	NMPS01000220.1	Siphonobacter sp. BAB-5385	Bacteria	Bacteroidetes	Cytophagia	Cytophagales	Cytophagaceae	Siphonobacter	hypothetical protein	cas3HD	cd09641	CAS-I			
222138..223827	+	OZI05903.1	NMPS01000220.1	Siphonobacter sp. BAB-5385	Bacteria	Bacteroidetes	Cytophagia	Cytophagales	Cytophagaceae	Siphonobacter	CRISPR-associated helicase Cas3'	cas3	COG1203	CAS-I			
223955..224468	+	OZI05904.1	NMPS01000220.1	Siphonobacter sp. BAB-5385	Bacteria	Bacteroidetes	Cytophagia	Cytophagales	Cytophagaceae	Siphonobacter	CRISPR-associated protein Cas4	cas4	pfam01930	CAS-I			
224517..225528	+	OZI05905.1	NMPS01000220.1	Siphonobacter sp. BAB-5385	Bacteria	Bacteroidetes	Cytophagia	Cytophagales	Cytophagaceae	Siphonobacter	subtype I-B CRISPR-associated endonuclease Cas1	cas1	cd09722	CAS-I-B			
225601..225865	+	OZI05906.1	NMPS01000220.1	Siphonobacter sp. BAB-5385	Bacteria	Bacteroidetes	Cytophagia	Cytophagales	Cytophagaceae	Siphonobacter	CRISPR-associated endonuclease Cas2	cas2	cd09725	CAS-I,CAS-II,CAS-III,CAS-V			
226080..226241	array		NMPS01000220.1	Siphonobacter sp. BAB-5385	Bacteria	Bacteroidetes	Cytophagia	Cytophagales	Cytophagaceae	Siphonobacter		CRISPR array			3	36	30
390 CAS-I-B | OJW70712.1																	
730..1444	+	OJW70704.1	MKUD01000084.1	Spirosoma sp. 48-14	Bacteria	Bacteroidetes	Cytophagia	Cytophagales	Cytophagaceae	Spirosoma	type I-PGING CRISPR-associated protein Cas5p	cas5	cd09716	CAS-I-B			
1440..2076	+	OJW70705.1	MKUD01000084.1	Spirosoma sp. 48-14	Bacteria	Bacteroidetes	Cytophagia	Cytophagales	Cytophagaceae	Spirosoma	hypothetical protein	cas8b4	cd09715	CAS-I-B			
2082..3033	+	OJW70706.1	MKUD01000084.1	Spirosoma sp. 48-14	Bacteria	Bacteroidetes	Cytophagia	Cytophagales	Cytophagaceae	Spirosoma	hypothetical protein	cas8b4	cd09715	CAS-I-B			
3061..3982	+	OJW70707.1	MKUD01000084.1	Spirosoma sp. 48-14	Bacteria	Bacteroidetes	Cytophagia	Cytophagales	Cytophagaceae	Spirosoma	type I-PGING CRISPR-associated protein Cas7/Csp1	cas7b	cd09717	CAS-I-B			
4060..6334	+	OJW70708.1	MKUD01000084.1	Spirosoma sp. 48-14	Bacteria	Bacteroidetes	Cytophagia	Cytophagales	Cytophagaceae	Spirosoma	CRISPR-associated helicase Cas3'	cas3HD,cas3	cd09641,cd09639	CAS-I			
6345..6858	+	OJW70709.1	MKUD01000084.1	Spirosoma sp. 48-14	Bacteria	Bacteroidetes	Cytophagia	Cytophagales	Cytophagaceae	Spirosoma	CRISPR-associated protein Cas4	cas4	pfam01930	CAS-I			
6972..7344	+	OJW70710.1	MKUD01000084.1	Spirosoma sp. 48-14	Bacteria	Bacteroidetes	Cytophagia	Cytophagales	Cytophagaceae	Spirosoma	GxxExxY protein						
7428..8439	+	OJW70711.1	MKUD01000084.1	Spirosoma sp. 48-14	Bacteria	Bacteroidetes	Cytophagia	Cytophagales	Cytophagaceae	Spirosoma	subtype I-B CRISPR-associated endonuclease Cas1	cas1	cd09722	CAS-I-B			
8525..8789	+	OJW70712.1	MKUD01000084.1	Spirosoma sp. 48-14	Bacteria	Bacteroidetes	Cytophagia	Cytophagales	Cytophagaceae	Spirosoma	CRISPR-associated endonuclease Cas2	cas2	cd09725	CAS-I,CAS-II,CAS-III,CAS-V			
9005..9227	array		MKUD01000084.1	Spirosoma sp. 48-14	Bacteria	Bacteroidetes	Cytophagia	Cytophagales	Cytophagaceae	Spirosoma		CRISPR array			4	34	30
391 CAS-I | PRY39964.1																	
55070..55637	+	PRY39953.1	PVTE01000007.1	Spirosoma oryzae	Bacteria	Bacteroidetes	Cytophagia	Cytophagales	Cytophagaceae	Spirosoma	precorrin-2 dehydrogenase/sirohydrochlorin ferrochelatase	csn2*	cd12218				
55645..56647	-	PRY39954.1	PVTE01000007.1	Spirosoma oryzae	Bacteria	Bacteroidetes	Cytophagia	Cytophagales	Cytophagaceae	Spirosoma	putative DNA-binding transcriptional regulator YafY	WYL	pfam13280	CAS-I,CAS-III			
56796..57612	+	PRY39955.1	PVTE01000007.1	Spirosoma oryzae	Bacteria	Bacteroidetes	Cytophagia	Cytophagales	Cytophagaceae	Spirosoma	CRISPR-associated Cas6 family protein	cas6	COG1583	CAS-I,CAS-III,CAS-IV			
57961..60559	+	PRY39956.1	PVTE01000007.1	Spirosoma oryzae	Bacteria	Bacteroidetes	Cytophagia	Cytophagales	Cytophagaceae	Spirosoma	CRISPR-associated endonuclease/helicase Cas3	cas3,cas3HD	COG1203,cd09641	CAS-I			
60561..62409	+	PRY39957.1	PVTE01000007.1	Spirosoma oryzae	Bacteria	Bacteroidetes	Cytophagia	Cytophagales	Cytophagaceae	Spirosoma	hypothetical protein	cas8a4	cls000706	CAS-I-A			
62420..63398	+	PRY39958.1	PVTE01000007.1	Spirosoma oryzae	Bacteria	Bacteroidetes	Cytophagia	Cytophagales	Cytophagaceae	Spirosoma	CRISPR-associated Csa2 family autoregulator	cas7	pfam01905	CAS-I			
63401..64079	+	PRY39959.1	PVTE01000007.1	Spirosoma oryzae	Bacteria	Bacteroidetes	Cytophagia	Cytophagales	Cytophagaceae	Spirosoma	hypothetical protein	cas5	cls000048	CAS-I-A			
64082..64808	+	PRY39960.1	PVTE01000007.1	Spirosoma oryzae	Bacteria	Bacteroidetes	Cytophagia	Cytophagales	Cytophagaceae	Spirosoma	putative nuclease of restriction endonuclease-like (RecB) superfamily	PD-DExK,PD-DExK	COG4804,COG4804	CAS-I,CAS-III			
64809..65058	-	PRY39961.1	PVTE01000007.1	Spirosoma oryzae	Bacteria	Bacteroidetes	Cytophagia	Cytophagales	Cytophagaceae	Spirosoma	hypothetical protein						
65116..65626	+	PRY39962.1	PVTE01000007.1	Spirosoma oryzae	Bacteria	Bacteroidetes	Cytophagia	Cytophagales	Cytophagaceae	Spirosoma	CRISPR-associated Cas4 family exonuclease	cas4	pfam01930	CAS-I			
65917..66940	+	PRY39963.1	PVTE01000007.1	Spirosoma oryzae	Bacteria	Bacteroidetes	Cytophagia	Cytophagales	Cytophagaceae	Spirosoma	CRISPR-associated Cas1 family protein	cas1	cd09722	CAS-I-B			
67035..67299	+	PRY39964.1	PVTE01000007.1	Spirosoma oryzae	Bacteria	Bacteroidetes	Cytophagia	Cytophagales	Cytophagaceae	Spirosoma	CRISPR-associated Cas2 family protein	cas2	COG1343	CAS-I,CAS-II,CAS-III			
67514..72119	array		PVTE01000007.1	Spirosoma oryzae	Bacteria	Bacteroidetes	Cytophagia	Cytophagales	Cytophagaceae	Spirosoma		CRISPR array			70	36	30
392 CAS-I-B | AEE48390.1																	
602093..603095	-	AEE48382.1	CP002691.1	Haliscomenobacter hydrossis DSM 1100	Bacteria	Bacteroidetes	Saprospiria	Saprospirales	Haliscomenobacteraceae	Haliscomenobacter	hypothetical protein	WYL	pfam13280	CAS-I,CAS-III			
603242..604043	+	AEE48383.1	CP002691.1	Haliscomenobacter hydrossis DSM 1100	Bacteria	Bacteroidetes	Saprospiria	Saprospirales	Haliscomenobacteraceae	Haliscomenobacter	CRISPR-associated protein Cas6	cas6	COG1583	CAS-I,CAS-III,CAS-IV			
604082..605510	+	AEE48384.1	CP002691.1	Haliscomenobacter hydrossis DSM 1100	Bacteria	Bacteroidetes	Saprospiria	Saprospirales	Haliscomenobacteraceae	Haliscomenobacter	hypothetical protein	cas8b2	cls000029	CAS-I-B			
605530..606607	+	AEE48385.1	CP002691.1	Haliscomenobacter hydrossis DSM 1100	Bacteria	Bacteroidetes	Saprospiria	Saprospirales	Haliscomenobacteraceae	Haliscomenobacter	CRISPR-associated autoregulator, DevR family	cas7	cd09687	CAS-I-B			
606603..607272	+	AEE48386.1	CP002691.1	Haliscomenobacter hydrossis DSM 1100	Bacteria	Bacteroidetes	Saprospiria	Saprospirales	Haliscomenobacteraceae	Haliscomenobacter	CRISPR-associated protein Cas5 family	cas5	COG1688	CAS-I			
607276..609544	+	AEE48387.1	CP002691.1	Haliscomenobacter hydrossis DSM 1100	Bacteria	Bacteroidetes	Saprospiria	Saprospirales	Haliscomenobacteraceae	Haliscomenobacter	CRISPR-associated helicase Cas3	cas3HD,cas3	cd09641,COG1203	CAS-I			
609553..610072	+	AEE48388.1	CP002691.1	Haliscomenobacter hydrossis DSM 1100	Bacteria	Bacteroidetes	Saprospiria	Saprospirales	Haliscomenobacteraceae	Haliscomenobacter	CRISPR-associated protein Cas4	cas4	pfam01930	CAS-I			
610123..611128	+	AEE48389.1	CP002691.1	Haliscomenobacter hydrossis DSM 1100	Bacteria	Bacteroidetes	Saprospiria	Saprospirales	Haliscomenobacteraceae	Haliscomenobacter	CRISPR-associated protein Cas1	cas1	cd09722	CAS-I-B			
611204..611468	+	AEE48390.1	CP002691.1	Haliscomenobacter hydrossis DSM 1100	Bacteria	Bacteroidetes	Saprospiria	Saprospirales	Haliscomenobacteraceae	Haliscomenobacter	CRISPR-associated protein Cas2	cas2	COG1343	CAS-I,CAS-II,CAS-III			
611640..617749	array		CP002691.1	Haliscomenobacter hydrossis DSM 1100	Bacteria	Bacteroidetes	Saprospiria	Saprospirales	Haliscomenobacteraceae	Haliscomenobacter		CRISPR array			93	36	30
617837..619289	+	AEE48391.1	CP002691.1	Haliscomenobacter hydrossis DSM 1100	Bacteria	Bacteroidetes	Saprospiria	Saprospirales	Haliscomenobacteraceae	Haliscomenobacter	Integrase catalytic region						
619337..620084	+	AEE48392.1	CP002691.1	Haliscomenobacter hydrossis DSM 1100	Bacteria	Bacteroidetes	Saprospiria	Saprospirales	Haliscomenobacteraceae	Haliscomenobacter	IstB domain protein ATP-binding protein						
620125..625304	array		CP002691.1	Haliscomenobacter hydrossis DSM 1100	Bacteria	Bacteroidetes	Saprospiria	Saprospirales	Haliscomenobacteraceae	Haliscomenobacter		CRISPR array			79	36	30
393 partial CAS-I-B | RMF22101.1																	
0..270	+	RMF22099.1	RFJC01001181.1	Bacteroidetes bacterium	Bacteria	Bacteroidetes					Dna2/Cas4 domain-containing protein	cas4	pfam01930	CAS-I			
450..1455	+	RMF22100.1	RFJC01001181.1	Bacteroidetes bacterium	Bacteria	Bacteroidetes					type I-B CRISPR-associated endonuclease Cas1	cas1	cd09722	CAS-I-B			
1454..1672	+	RMF22101.1	RFJC01001181.1	Bacteroidetes bacterium	Bacteria	Bacteroidetes					CRISPR-associated endonuclease Cas2	cas2	cd09725	CAS-I,CAS-II,CAS-III,CAS-V			
394 partial CAS-I-B | RMH46293.1																	
0..349	+	RMH46292.1	RFGJ01000114.1	Gammaproteobacteria bacterium	Bacteria	Proteobacteria	Gammaproteobacteria				CRISPR-associated endonuclease Cas1	cas1	cd09722	CAS-I-B			
348..612	+	RMH46293.1	RFGJ01000114.1	Gammaproteobacteria bacterium	Bacteria	Proteobacteria	Gammaproteobacteria				CRISPR-associated endonuclease Cas2	cas2	COG1343	CAS-I,CAS-II,CAS-III			
819..1043	array		RFGJ01000114.1	Gammaproteobacteria bacterium	Bacteria	Proteobacteria	Gammaproteobacteria					CRISPR array			4	35	30
395 CAS-I-B | PAC31305.1																	
322546..323287	+	PAC31157.1	NJFZ01000009.1	Flectobacillus sp. BAB-3569	Bacteria	Bacteroidetes	Cytophagia	Cytophagales	Cytophagaceae	Flectobacillus	CRISPR-associated endoribonuclease Cas6	cas6	pfam01881	CAS-I,CAS-III			
323318..324245	+	PAC31158.1	NJFZ01000009.1	Flectobacillus sp. BAB-3569	Bacteria	Bacteroidetes	Cytophagia	Cytophagales	Cytophagaceae	Flectobacillus	hypothetical protein	WYL	pfam13280	CAS-I,CAS-III			
324327..324993	+	PAC31159.1	NJFZ01000009.1	Flectobacillus sp. BAB-3569	Bacteria	Bacteroidetes	Cytophagia	Cytophagales	Cytophagaceae	Flectobacillus	CRISPR-associated endoribonuclease Cas6	cas6	cd09652	CAS-I,CAS-III			
324999..325587	+	PAC31160.1	NJFZ01000009.1	Flectobacillus sp. BAB-3569	Bacteria	Bacteroidetes	Cytophagia	Cytophagales	Cytophagaceae	Flectobacillus	hypothetical protein	cas5	mkCas0069	CAS-I-B			
327733..329083	+	PAC31161.1	NJFZ01000009.1	Flectobacillus sp. BAB-3569	Bacteria	Bacteroidetes	Cytophagia	Cytophagales	Cytophagaceae	Flectobacillus	hypothetical protein	cas8b6	mkCas0111	CAS-I-B			
329063..330113	+	PAC31162.1	NJFZ01000009.1	Flectobacillus sp. BAB-3569	Bacteria	Bacteroidetes	Cytophagia	Cytophagales	Cytophagaceae	Flectobacillus	CRISPR-associated protein Cas7	cas7	mkCas0088	CAS-I-B			
331476..332025	+	PAC31163.1	NJFZ01000009.1	Flectobacillus sp. BAB-3569	Bacteria	Bacteroidetes	Cytophagia	Cytophagales	Cytophagaceae	Flectobacillus	transposase						
332021..332561	+	PAC31164.1	NJFZ01000009.1	Flectobacillus sp. BAB-3569	Bacteria	Bacteroidetes	Cytophagia	Cytophagales	Cytophagaceae	Flectobacillus	CRISPR-associated protein Cas4	cas4	pfam01930	CAS-I			
333670..333934	+	PAC31305.1	NJFZ01000009.1	Flectobacillus sp. BAB-3569	Bacteria	Bacteroidetes	Cytophagia	Cytophagales	Cytophagaceae	Flectobacillus	CRISPR-associated endonuclease Cas2	cas2	cd09725	CAS-I,CAS-II,CAS-III,CAS-V			
334150..334505	array		NJFZ01000009.1	Flectobacillus sp. BAB-3569	Bacteria	Bacteroidetes	Cytophagia	Cytophagales	Cytophagaceae	Flectobacillus		CRISPR array			6	35	30
396 CAS-I | RFS17569.1																	
594707..598451	array		QVMT01000002.1	Emticicia sp. C21	Bacteria	Bacteroidetes	Cytophagia	Cytophagales	Cytophagaceae	Emticicia		CRISPR array			58	36	29
598552..601577	array		QVMT01000002.1	Emticicia sp. C21	Bacteria	Bacteroidetes	Cytophagia	Cytophagales	Cytophagaceae	Emticicia		CRISPR array			47	36	29
601779..602043	-	RFS17569.1	QVMT01000002.1	Emticicia sp. C21	Bacteria	Bacteroidetes	Cytophagia	Cytophagales	Cytophagaceae	Emticicia	CRISPR-associated endonuclease Cas2	cas2	cd09725	CAS-I,CAS-II,CAS-III,CAS-V			
603096..603642	-	RFS17570.1	QVMT01000002.1	Emticicia sp. C21	Bacteria	Bacteroidetes	Cytophagia	Cytophagales	Cytophagaceae	Emticicia	CRISPR-associated protein Cas4	cas4	pfam01930	CAS-I			
603644..605975	-	RFS17571.1	QVMT01000002.1	Emticicia sp. C21	Bacteria	Bacteroidetes	Cytophagia	Cytophagales	Cytophagaceae	Emticicia	CRISPR-associated helicase Cas3'	cas3HD,cas3	cd09641,cd09639	CAS-I			
605961..606627	-	RFS17572.1	QVMT01000002.1	Emticicia sp. C21	Bacteria	Bacteroidetes	Cytophagia	Cytophagales	Cytophagaceae	Emticicia	type I-B CRISPR-associated protein Cas5	cas5	COG1688	CAS-I			
606628..607714	-	RFS17573.1	QVMT01000002.1	Emticicia sp. C21	Bacteria	Bacteroidetes	Cytophagia	Cytophagales	Cytophagaceae	Emticicia	type I-B CRISPR-associated protein Cas7/Cst2/DevR	cas7	cd09687	CAS-I-B			
607727..609125	-	RFS17574.1	QVMT01000002.1	Emticicia sp. C21	Bacteria	Bacteroidetes	Cytophagia	Cytophagales	Cytophagaceae	Emticicia	type I-B CRISPR-associated protein Cas8b1/Cst1	cas8b2	cls000029	CAS-I-B			
609288..609486	+	RFS17575.1	QVMT01000002.1	Emticicia sp. C21	Bacteria	Bacteroidetes	Cytophagia	Cytophagales	Cytophagaceae	Emticicia	hypothetical protein						
609546..610470	-	RFS17576.1	QVMT01000002.1	Emticicia sp. C21	Bacteria	Bacteroidetes	Cytophagia	Cytophagales	Cytophagaceae	Emticicia	WYL domain-containing protein	WYL	COG2378	CAS-I,CAS-III			
610482..611292	-	RFS17577.1	QVMT01000002.1	Emticicia sp. C21	Bacteria	Bacteroidetes	Cytophagia	Cytophagales	Cytophagaceae	Emticicia	CRISPR-associated endoribonuclease Cas6	cas6	cd09759	CAS-I-A			
397 CAS-I-B | PLK45909.1																	
153292..154102	+	PLK45899.1	PKRS01000001.1	Emticicia sp. TH156	Bacteria	Bacteroidetes	Cytophagia	Cytophagales	Cytophagaceae	Emticicia	CRISPR-associated endoribonuclease Cas6	cas6	cd09759	CAS-I-A			
154114..155038	+	PLK45900.1	PKRS01000001.1	Emticicia sp. TH156	Bacteria	Bacteroidetes	Cytophagia	Cytophagales	Cytophagaceae	Emticicia	hypothetical protein	WYL	COG2378	CAS-I,CAS-III			
155273..155792	-	PLK45901.1	PKRS01000001.1	Emticicia sp. TH156	Bacteria	Bacteroidetes	Cytophagia	Cytophagales	Cytophagaceae	Emticicia	hypothetical protein						
155971..156637	+	PLK45902.1	PKRS01000001.1	Emticicia sp. TH156	Bacteria	Bacteroidetes	Cytophagia	Cytophagales	Cytophagaceae	Emticicia	CRISPR-associated endoribonuclease Cas6	cas6	cd09652	CAS-I,CAS-III			
156649..157237	+	PLK45903.1	PKRS01000001.1	Emticicia sp. TH156	Bacteria	Bacteroidetes	Cytophagia	Cytophagales	Cytophagaceae	Emticicia	hypothetical protein	cas5	mkCas0069	CAS-I-B			
157236..159390	+	PLK45904.1	PKRS01000001.1	Emticicia sp. TH156	Bacteria	Bacteroidetes	Cytophagia	Cytophagales	Cytophagaceae	Emticicia	CRISPR-associated helicase/endonuclease Cas3	cas3HD,cas3	cd09641,cd09639	CAS-I			
159386..160709	+	PLK45905.1	PKRS01000001.1	Emticicia sp. TH156	Bacteria	Bacteroidetes	Cytophagia	Cytophagales	Cytophagaceae	Emticicia	hypothetical protein	cas8b6	mkCas0111	CAS-I-B			
160717..161791	+	PLK45906.1	PKRS01000001.1	Emticicia sp. TH156	Bacteria	Bacteroidetes	Cytophagia	Cytophagales	Cytophagaceae	Emticicia	CRISPR-associated protein Cas7	cas7	mkCas0088	CAS-I-B			
161794..162340	+	PLK45907.1	PKRS01000001.1	Emticicia sp. TH156	Bacteria	Bacteroidetes	Cytophagia	Cytophagales	Cytophagaceae	Emticicia	CRISPR-associated protein Cas4	cas4	pfam01930	CAS-I			
162349..163354	+	PLK45908.1	PKRS01000001.1	Emticicia sp. TH156	Bacteria	Bacteroidetes	Cytophagia	Cytophagales	Cytophagaceae	Emticicia	subtype I-B CRISPR-associated endonuclease Cas1	cas1	cd09722	CAS-I-B			
163382..163646	+	PLK45909.1	PKRS01000001.1	Emticicia sp. TH156	Bacteria	Bacteroidetes	Cytophagia	Cytophagales	Cytophagaceae	Emticicia	CRISPR-associated endonuclease Cas2	cas2	cd09725	CAS-I,CAS-II,CAS-III,CAS-V			
163841..172095	array		PKRS01000001.1	Emticicia sp. TH156	Bacteria	Bacteroidetes	Cytophagia	Cytophagales	Cytophagaceae	Emticicia		CRISPR array			125	36	30
398 CAS-I-B | AYQ36249.1																	
2274776..2275069	array		CP031030.1	Runella sp. SP2	Bacteria	Bacteroidetes	Cytophagia	Cytophagales	Cytophagaceae	Runella		CRISPR array			5	34	31
2275272..2275536	-	AYQ36249.1	CP031030.1	Runella sp. SP2	Bacteria	Bacteroidetes	Cytophagia	Cytophagales	Cytophagaceae	Runella	CRISPR-associated endonuclease Cas2	cas2	cd09725	CAS-I,CAS-II,CAS-III,CAS-V			
2275577..2276582	-	AYQ32410.1	CP031030.1	Runella sp. SP2	Bacteria	Bacteroidetes	Cytophagia	Cytophagales	Cytophagaceae	Runella	type I-B CRISPR-associated endonuclease Cas1	cas1	cd09722	CAS-I-B			
2276602..2277139	-	AYQ32411.1	CP031030.1	Runella sp. SP2	Bacteria	Bacteroidetes	Cytophagia	Cytophagales	Cytophagaceae	Runella	CRISPR-associated protein Cas4	cas4	pfam01930	CAS-I			
2277135..2277648	-	AYQ32412.1	CP031030.1	Runella sp. SP2	Bacteria	Bacteroidetes	Cytophagia	Cytophagales	Cytophagaceae	Runella	transposase						
2277708..2278764	-	AYQ32413.1	CP031030.1	Runella sp. SP2	Bacteria	Bacteroidetes	Cytophagia	Cytophagales	Cytophagaceae	Runella	CRISPR-associated protein Cas7	cas7	mkCas0088	CAS-I-B			
2278784..2280101	-	AYQ32414.1	CP031030.1	Runella sp. SP2	Bacteria	Bacteroidetes	Cytophagia	Cytophagales	Cytophagaceae	Runella	hypothetical protein	cas8b6	mkCas0111	CAS-I-B			
2280097..2282242	-	AYQ32415.1	CP031030.1	Runella sp. SP2	Bacteria	Bacteroidetes	Cytophagia	Cytophagales	Cytophagaceae	Runella	CRISPR-associated helicase Cas3'	cas3HD,cas3	cd09641,COG1203	CAS-I			
2282241..2282829	-	AYQ32416.1	CP031030.1	Runella sp. SP2	Bacteria	Bacteroidetes	Cytophagia	Cytophagales	Cytophagaceae	Runella	hypothetical protein	cas5	mkCas0069	CAS-I-B			
2282835..2283501	-	AYQ32417.1	CP031030.1	Runella sp. SP2	Bacteria	Bacteroidetes	Cytophagia	Cytophagales	Cytophagaceae	Runella	CRISPR-associated endoribonuclease Cas6	cas6	cd09652	CAS-I,CAS-III			
2283588..2284515	-	AYQ32418.1	CP031030.1	Runella sp. SP2	Bacteria	Bacteroidetes	Cytophagia	Cytophagales	Cytophagaceae	Runella	WYL domain-containing protein	WYL	pfam13280	CAS-I,CAS-III			
2284788..2285238	+	AYQ32419.1	CP031030.1	Runella sp. SP2	Bacteria	Bacteroidetes	Cytophagia	Cytophagales	Cytophagaceae	Runella	hypothetical protein						
2285383..2286199	-	AYQ32420.1	CP031030.1	Runella sp. SP2	Bacteria	Bacteroidetes	Cytophagia	Cytophagales	Cytophagaceae	Runella	CRISPR-associated endoribonuclease Cas6	cas6	cd09759	CAS-I-A			
399 CAS-I-B | PKO99099.1																	
301469..302126	+	PKO99092.1	PHCZ01000006.1	Bacteroidetes bacterium HGW-Bacteroidetes-8	Bacteria	Bacteroidetes					CRISPR-associated endoribonuclease Cas6	cas6	COG1583	CAS-I,CAS-III,CAS-IV			
302132..302678	+	PKO99093.1	PHCZ01000006.1	Bacteroidetes bacterium HGW-Bacteroidetes-8	Bacteria	Bacteroidetes					hypothetical protein	cas5	mkCas0069	CAS-I-B			
302677..304819	+	PKO99094.1	PHCZ01000006.1	Bacteroidetes bacterium HGW-Bacteroidetes-8	Bacteria	Bacteroidetes					CRISPR-associated helicase/endonuclease Cas3	cas3HD,cas3	cd09641,COG1203	CAS-I			
304831..306145	+	PKO99095.1	PHCZ01000006.1	Bacteroidetes bacterium HGW-Bacteroidetes-8	Bacteria	Bacteroidetes					hypothetical protein	cas8b6	mkCas0111	CAS-I-B			
306156..307206	+	PKO99096.1	PHCZ01000006.1	Bacteroidetes bacterium HGW-Bacteroidetes-8	Bacteria	Bacteroidetes					CRISPR-associated protein Cas7	cas7	mkCas0088	CAS-I-B			

307266..307776	+	PKO99097.1	PHCZ01000006.1	Bacteroidetes bacterium HGW-Bacteroidetes-8	Bacteria	Bacteroidetes					CRISPR-associated protein Cas4	cas4	pfam01930	CAS-I			
307801..308821	+	PKO99098.1	PHCZ01000006.1	Bacteroidetes bacterium HGW-Bacteroidetes-8	Bacteria	Bacteroidetes					subtype I-B CRISPR-associated endonuclease Cas1	cas1	cd09722	CAS-I-B			
308820..309084	+	PKO99099.1	PHCZ01000006.1	Bacteroidetes bacterium HGW-Bacteroidetes-8	Bacteria	Bacteroidetes					CRISPR-associated endonuclease Cas2	cas2	COG1343	CAS-I,CAS-II,CAS-III			
309309..315201	array		PHCZ01000006.1	Bacteroidetes bacterium HGW-Bacteroidetes-8	Bacteria	Bacteroidetes						CRISPR array			91	35	30
400 CAS-I-B | AEW01073.1																	
5986432..5989880	array		CP003178.1	Niastella koreensis GR20-10	Bacteria	Bacteroidetes	Chitinophagia	Chitinophagales	Chitinophagaceae	Niastella		CRISPR array			53	35	30
5990087..5990351	-	AEW01073.1	CP003178.1	Niastella koreensis GR20-10	Bacteria	Bacteroidetes	Chitinophagia	Chitinophagales	Chitinophagaceae	Niastella	CRISPR-associated protein, Cas2 family	cas2	cd09725	CAS-I,CAS-II,CAS-III,CAS-V			
5991815..5992322	-	AEW01074.1	CP003178.1	Niastella koreensis GR20-10	Bacteria	Bacteroidetes	Chitinophagia	Chitinophagales	Chitinophagaceae	Niastella	CRISPR-associated exonuclease, Cas4 family	cas4	pfam01930	CAS-I			
5992324..5995096	-	AEW01075.1	CP003178.1	Niastella koreensis GR20-10	Bacteria	Bacteroidetes	Chitinophagia	Chitinophagales	Chitinophagaceae	Niastella	CRISPR-associated HD domain protein	cas3HD,cas3	cd09641,COG1203	CAS-I			
5995095..5995872	-	AEW01076.1	CP003178.1	Niastella koreensis GR20-10	Bacteria	Bacteroidetes	Chitinophagia	Chitinophagales	Chitinophagaceae	Niastella	CRISPR-associated protein Cas5	cas5	cls001430	CAS-I-B			
5995878..5996817	-	AEW01077.1	CP003178.1	Niastella koreensis GR20-10	Bacteria	Bacteroidetes	Chitinophagia	Chitinophagales	Chitinophagaceae	Niastella	CRISPR-associated protein TM1801	cas7b	pfam05107	CAS-I-B,CAS-I-C			
5996840..5998748	-	AEW01078.1	CP003178.1	Niastella koreensis GR20-10	Bacteria	Bacteroidetes	Chitinophagia	Chitinophagales	Chitinophagaceae	Niastella	hypothetical protein	cas8b1	mkCas0106	CAS-I-B			
5998771..5999581	-	AEW01079.1	CP003178.1	Niastella koreensis GR20-10	Bacteria	Bacteroidetes	Chitinophagia	Chitinophagales	Chitinophagaceae	Niastella	CRISPR-associated protein Cas6	cas6	COG1583	CAS-I,CAS-III,CAS-IV			
401 CAS-I-B | OQP59767.1																	
1..553	array		LWBP01000167.1	Niastella populi	Bacteria	Bacteroidetes	Chitinophagia	Chitinophagales	Chitinophagaceae	Niastella		CRISPR array			9	36	29
759..1023	-	OQP59767.1	LWBP01000167.1	Niastella populi	Bacteria	Bacteroidetes	Chitinophagia	Chitinophagales	Chitinophagaceae	Niastella	CRISPR-associated endonuclease Cas2	cas2	cd09725	CAS-I,CAS-II,CAS-III,CAS-V			
1024..2029	-	OQP59768.1	LWBP01000167.1	Niastella populi	Bacteria	Bacteroidetes	Chitinophagia	Chitinophagales	Chitinophagaceae	Niastella	subtype I-B CRISPR-associated endonuclease Cas1	cas1	cd09722	CAS-I-B			
2206..2710	-	OQP59769.1	LWBP01000167.1	Niastella populi	Bacteria	Bacteroidetes	Chitinophagia	Chitinophagales	Chitinophagaceae	Niastella	CRISPR-associated protein Cas4	cas4	pfam01930	CAS-I			
2725..5056	-	OQP59770.1	LWBP01000167.1	Niastella populi	Bacteria	Bacteroidetes	Chitinophagia	Chitinophagales	Chitinophagaceae	Niastella	hypothetical protein	cas3HD,cas3	cd09641,COG1203	CAS-I			
5079..5754	-	OQP59771.1	LWBP01000167.1	Niastella populi	Bacteria	Bacteroidetes	Chitinophagia	Chitinophagales	Chitinophagaceae	Niastella	type I-B CRISPR-associated protein Cas5	cas5	COG1688	CAS-I			
5753..6851	-	OQP59772.1	LWBP01000167.1	Niastella populi	Bacteria	Bacteroidetes	Chitinophagia	Chitinophagales	Chitinophagaceae	Niastella	type I-B CRISPR-associated protein Cas7/Cst2/DevR	cas7	cd09687	CAS-I-B			
6853..8281	-	OQP59773.1	LWBP01000167.1	Niastella populi	Bacteria	Bacteroidetes	Chitinophagia	Chitinophagales	Chitinophagaceae	Niastella	type I-B CRISPR-associated protein Cas8b1/Cst1	cas8b2	cls000029	CAS-I-B			
8665..9466	-	OQP59774.1	LWBP01000167.1	Niastella populi	Bacteria	Bacteroidetes	Chitinophagia	Chitinophagales	Chitinophagaceae	Niastella	CRISPR-associated endoribonuclease Cas6	cas6	COG1583	CAS-I,CAS-III,CAS-IV			
402 CAS-I-B | SEA90597.1																	
110030..110822	+	SEA90523.1	FNRL01000021.1	Chitinophaga terrae Kim and Jung 2007	Bacteria	Bacteroidetes	Chitinophagia	Chitinophagales	Chitinophagaceae	Chitinophaga	CRISPR-associated endoribonuclease Cas6	cas6	COG1583	CAS-I,CAS-III,CAS-IV			
111563..112982	+	SEA90530.1	FNRL01000021.1	Chitinophaga terrae Kim and Jung 2007	Bacteria	Bacteroidetes	Chitinophagia	Chitinophagales	Chitinophagaceae	Chitinophaga	CRISPR-associated protein Cst1	cas8b2	cd09665	CAS-I-B			
112995..114084	+	SEA90538.1	FNRL01000021.1	Chitinophaga terrae Kim and Jung 2007	Bacteria	Bacteroidetes	Chitinophagia	Chitinophagales	Chitinophagaceae	Chitinophaga	CRISPR-associated autoregulator, Cst2 family	cas7	cd09687	CAS-I-B			
114083..114752	+	SEA90551.1	FNRL01000021.1	Chitinophaga terrae Kim and Jung 2007	Bacteria	Bacteroidetes	Chitinophagia	Chitinophagales	Chitinophagaceae	Chitinophaga	CRISPR-associated protein, Cas5t family	cas5	COG1688	CAS-I			
114754..116971	+	SEA90564.1	FNRL01000021.1	Chitinophaga terrae Kim and Jung 2007	Bacteria	Bacteroidetes	Chitinophagia	Chitinophagales	Chitinophagaceae	Chitinophaga	CRISPR-associated endonuclease/helicase Cas3	cas3HD,cas3	cd09641,COG1203	CAS-I			
117035..117503	+	SEA90568.1	FNRL01000021.1	Chitinophaga terrae Kim and Jung 2007	Bacteria	Bacteroidetes	Chitinophagia	Chitinophagales	Chitinophagaceae	Chitinophaga	CRISPR-associated exonuclease Cas4	cas4	pfam01930	CAS-I			
117499..118504	+	SEA90583.1	FNRL01000021.1	Chitinophaga terrae Kim and Jung 2007	Bacteria	Bacteroidetes	Chitinophagia	Chitinophagales	Chitinophagaceae	Chitinophaga	CRISP-associated protein Cas1	cas1	cd09722	CAS-I-B			
118505..118769	+	SEA90597.1	FNRL01000021.1	Chitinophaga terrae Kim and Jung 2007	Bacteria	Bacteroidetes	Chitinophagia	Chitinophagales	Chitinophagaceae	Chitinophaga	CRISPR-associated protein Cas2	cas2	COG1343	CAS-I,CAS-II,CAS-III			
118967..119129	array		FNRL01000021.1	Chitinophaga terrae Kim and Jung 2007	Bacteria	Bacteroidetes	Chitinophagia	Chitinophagales	Chitinophagaceae	Chitinophaga		CRISPR array			3	36	30
403 CAS-I-B | AYD48412.1																	
3047517..3048333	+	AYD48405.1	CP032489.1	Arachidicoccus sp. KIS59-12	Bacteria	Bacteroidetes	Chitinophagia	Chitinophagales	Chitinophagaceae	Arachidicoccus	CRISPR-associated endoribonuclease Cas6	cas6	COG1583	CAS-I,CAS-III,CAS-IV			
3048448..3050107	+	AYD48406.1	CP032489.1	Arachidicoccus sp. KIS59-12	Bacteria	Bacteroidetes	Chitinophagia	Chitinophagales	Chitinophagaceae	Arachidicoccus	hypothetical protein	cas8b2,cas8b2	cls000029,cls000029	CAS-I-B			
3050137..3051229	+	AYD48407.1	CP032489.1	Arachidicoccus sp. KIS59-12	Bacteria	Bacteroidetes	Chitinophagia	Chitinophagales	Chitinophagaceae	Arachidicoccus	type I-B CRISPR-associated protein Cas7/Cst2/DevR	cas7	cd09687	CAS-I-B			
3051230..3051899	+	AYD48408.1	CP032489.1	Arachidicoccus sp. KIS59-12	Bacteria	Bacteroidetes	Chitinophagia	Chitinophagales	Chitinophagaceae	Arachidicoccus	type I-B CRISPR-associated protein Cas5	cas5	COG1688	CAS-I			
3051901..3054184	+	AYD48409.1	CP032489.1	Arachidicoccus sp. KIS59-12	Bacteria	Bacteroidetes	Chitinophagia	Chitinophagales	Chitinophagaceae	Arachidicoccus	CRISPR-associated helicase Cas3'	cas3HD,cas3	cd09641,COG1203	CAS-I			
3054187..3054718	+	AYD48410.1	CP032489.1	Arachidicoccus sp. KIS59-12	Bacteria	Bacteroidetes	Chitinophagia	Chitinophagales	Chitinophagaceae	Arachidicoccus	CRISPR-associated protein Cas4	cas4	pfam01930	CAS-I			
3054717..3055722	+	AYD48411.1	CP032489.1	Arachidicoccus sp. KIS59-12	Bacteria	Bacteroidetes	Chitinophagia	Chitinophagales	Chitinophagaceae	Arachidicoccus	type I-B CRISPR-associated endonuclease Cas1	cas1	cd09722	CAS-I-B			
3055723..3055987	+	AYD48412.1	CP032489.1	Arachidicoccus sp. KIS59-12	Bacteria	Bacteroidetes	Chitinophagia	Chitinophagales	Chitinophagaceae	Arachidicoccus	CRISPR-associated endonuclease Cas2	cas2	cd09725	CAS-I,CAS-II,CAS-III,CAS-V			
3056196..3057403	array		CP032489.1	Arachidicoccus sp. KIS59-12	Bacteria	Bacteroidetes	Chitinophagia	Chitinophagales	Chitinophagaceae	Arachidicoccus		CRISPR array			19	35	30
404 CAS-I-B | ASZ11931.1																	
3340230..3342232	array		CP023254.1	Chitinophaga sp. MD30	Bacteria	Bacteroidetes	Chitinophagia	Chitinophagales	Chitinophagaceae	Chitinophaga		CRISPR array			31	35	30
3342440..3342704	-	ASZ11931.1	CP023254.1	Chitinophaga sp. MD30	Bacteria	Bacteroidetes	Chitinophagia	Chitinophagales	Chitinophagaceae	Chitinophaga	CRISPR-associated endonuclease Cas2	cas2	cd09725	CAS-I,CAS-II,CAS-III,CAS-V			
3342704..3343709	-	ASZ11932.1	CP023254.1	Chitinophaga sp. MD30	Bacteria	Bacteroidetes	Chitinophagia	Chitinophagales	Chitinophagaceae	Chitinophaga	subtype I-B CRISPR-associated endonuclease Cas1	cas1	cd09722	CAS-I-B			
3343718..3344264	-	ASZ11933.1	CP023254.1	Chitinophaga sp. MD30	Bacteria	Bacteroidetes	Chitinophagia	Chitinophagales	Chitinophagaceae	Chitinophaga	CRISPR-associated protein Cas4	cas4	pfam01930	CAS-I			
3344266..3347053	-	ASZ11934.1	CP023254.1	Chitinophaga sp. MD30	Bacteria	Bacteroidetes	Chitinophagia	Chitinophagales	Chitinophagaceae	Chitinophaga	CRISPR-associated protein	cas3HD,cas3	cd09641,COG1203	CAS-I			
3347036..3347804	-	ASZ11935.1	CP023254.1	Chitinophaga sp. MD30	Bacteria	Bacteroidetes	Chitinophagia	Chitinophagales	Chitinophagaceae	Chitinophaga	type I-B CRISPR-associated protein Cas5	cas5	cls001430	CAS-I-B			
3347814..3348729	-	ASZ11936.1	CP023254.1	Chitinophaga sp. MD30	Bacteria	Bacteroidetes	Chitinophagia	Chitinophagales	Chitinophagaceae	Chitinophaga	CRISPR-associated protein	cas7b	pfam05107	CAS-I-B,CAS-I-C			
3348761..3350678	-	ASZ11937.1	CP023254.1	Chitinophaga sp. MD30	Bacteria	Bacteroidetes	Chitinophagia	Chitinophagales	Chitinophagaceae	Chitinophaga	hypothetical protein	cas8b1	mkCas0106	CAS-I-B			
3350674..3351490	-	ASZ11938.1	CP023254.1	Chitinophaga sp. MD30	Bacteria	Bacteroidetes	Chitinophagia	Chitinophagales	Chitinophagaceae	Chitinophaga	CRISPR-associated endoribonuclease Cas6	cas6	COG1583	CAS-I,CAS-III,CAS-IV			
405 CAS-I-B | RBL91812.1																	
1065928..1066729	+	RBL91805.1	QFFJ01000001.1	Chitinophaga sp. GDMCC 1.1325	Bacteria	Bacteroidetes	Chitinophagia	Chitinophagales	Chitinophagaceae	Chitinophaga	CRISPR-associated endoribonuclease Cas6	cas6	COG1583	CAS-I,CAS-III,CAS-IV			
1067058..1068927	+	RBL91806.1	QFFJ01000001.1	Chitinophaga sp. GDMCC 1.1325	Bacteria	Bacteroidetes	Chitinophagia	Chitinophagales	Chitinophagaceae	Chitinophaga	hypothetical protein	cas8b1	mkCas0106	CAS-I-B			
1068987..1069920	+	RBL91807.1	QFFJ01000001.1	Chitinophaga sp. GDMCC 1.1325	Bacteria	Bacteroidetes	Chitinophagia	Chitinophagales	Chitinophagaceae	Chitinophaga	CRISPR-associated protein	cas7b	pfam05107	CAS-I-B,CAS-I-C			
1069925..1070675	+	RBL91808.1	QFFJ01000001.1	Chitinophaga sp. GDMCC 1.1325	Bacteria	Bacteroidetes	Chitinophagia	Chitinophagales	Chitinophagaceae	Chitinophaga	type I-B CRISPR-associated protein Cas5	cas5	cls001430	CAS-I-B			
1070674..1073428	+	RBL91809.1	QFFJ01000001.1	Chitinophaga sp. GDMCC 1.1325	Bacteria	Bacteroidetes	Chitinophagia	Chitinophagales	Chitinophagaceae	Chitinophaga	hypothetical protein	cas3HD,cas3	cd09641,cd09639	CAS-I			
1073430..1073958	+	RBL91810.1	QFFJ01000001.1	Chitinophaga sp. GDMCC 1.1325	Bacteria	Bacteroidetes	Chitinophagia	Chitinophagales	Chitinophagaceae	Chitinophaga	CRISPR-associated protein Cas4	cas4	pfam01930	CAS-I			
1073969..1074974	+	RBL91811.1	QFFJ01000001.1	Chitinophaga sp. GDMCC 1.1325	Bacteria	Bacteroidetes	Chitinophagia	Chitinophagales	Chitinophagaceae	Chitinophaga	subtype I-B CRISPR-associated endonuclease Cas1	cas1	cd09722	CAS-I-B			
1074997..1075261	+	RBL91812.1	QFFJ01000001.1	Chitinophaga sp. GDMCC 1.1325	Bacteria	Bacteroidetes	Chitinophagia	Chitinophagales	Chitinophagaceae	Chitinophaga	CRISPR-associated endonuclease Cas2	cas2	cd09725	CAS-I,CAS-II,CAS-III,CAS-V			
1075469..1078741	array		QFFJ01000001.1	Chitinophaga sp. GDMCC 1.1325	Bacteria	Bacteroidetes	Chitinophagia	Chitinophagales	Chitinophagaceae	Chitinophaga		CRISPR array			50	36	30
406 partial CAS-I-B | KKX47645.1																	
19..2894	array		ASZT02000133.1	Sphingobacterium sp. IITKGP-BTPF85	Bacteria	Bacteroidetes	Sphingobacteriia	Sphingobacteriales	Sphingobacteriaceae	Sphingobacterium		CRISPR array			44	36	30
3112..3376	-	KKX47645.1	ASZT02000133.1	Sphingobacterium sp. IITKGP-BTPF85	Bacteria	Bacteroidetes	Sphingobacteriia	Sphingobacteriales	Sphingobacteriaceae	Sphingobacterium	CRISPR-associated protein Cas2	cas2	cd09725	CAS-I,CAS-II,CAS-III,CAS-V			
4404..4623	-	KKX47651.1	ASZT02000133.1	Sphingobacterium sp. IITKGP-BTPF85	Bacteria	Bacteroidetes	Sphingobacteriia	Sphingobacteriales	Sphingobacteriaceae	Sphingobacterium	hypothetical protein						
4619..4985	-	KKX47652.1	ASZT02000133.1	Sphingobacterium sp. IITKGP-BTPF85	Bacteria	Bacteroidetes	Sphingobacteriia	Sphingobacteriales	Sphingobacteriaceae	Sphingobacterium	hypothetical protein						
4971..5481	-	KKX47646.1	ASZT02000133.1	Sphingobacterium sp. IITKGP-BTPF85	Bacteria	Bacteroidetes	Sphingobacteriia	Sphingobacteriales	Sphingobacteriaceae	Sphingobacterium	CRISPR-associated protein Cas4	cas4	pfam01930	CAS-I			
10096..10681	-	KKX47647.1	ASZT02000133.1	Sphingobacterium sp. IITKGP-BTPF85	Bacteria	Bacteroidetes	Sphingobacteriia	Sphingobacteriales	Sphingobacteriaceae	Sphingobacterium	hypothetical protein	cas5	mkCas0069	CAS-I-B			
10687..11347	-	KKX47648.1	ASZT02000133.1	Sphingobacterium sp. IITKGP-BTPF85	Bacteria	Bacteroidetes	Sphingobacteriia	Sphingobacteriales	Sphingobacteriaceae	Sphingobacterium	CRISPR-associated protein Cas6	cas6	COG1583	CAS-I,CAS-III,CAS-IV			
407 CAS-I-B | PZX51588.1																	
75659..76451	+	PZX51580.1	QKZU01000018.1	Algoriphagus ratkowskyi	Bacteria	Bacteroidetes	Cytophagia	Cytophagales	Cyclobacteriaceae	Algoriphagus	CRISPR-associated endoribonuclease Cas6	cas6	cd09759	CAS-I-A			
76556..77240	+	PZX51581.1	QKZU01000018.1	Algoriphagus ratkowskyi	Bacteria	Bacteroidetes	Cytophagia	Cytophagales	Cyclobacteriaceae	Algoriphagus	CRISPR-associated endoribonuclease Cas6	cas6	cd09652	CAS-I,CAS-III			
77247..77844	+	PZX51582.1	QKZU01000018.1	Algoriphagus ratkowskyi	Bacteria	Bacteroidetes	Cytophagia	Cytophagales	Cyclobacteriaceae	Algoriphagus	hypothetical protein	cas5	mkCas0069	CAS-I-B			
77845..80017	+	PZX51583.1	QKZU01000018.1	Algoriphagus ratkowskyi	Bacteria	Bacteroidetes	Cytophagia	Cytophagales	Cyclobacteriaceae	Algoriphagus	CRISPR-associated endonuclease/helicase Cas3	cas3HD,cas3	cd09641,COG1203	CAS-I			
80019..81351	+	PZX51584.1	QKZU01000018.1	Algoriphagus ratkowskyi	Bacteria	Bacteroidetes	Cytophagia	Cytophagales	Cyclobacteriaceae	Algoriphagus	hypothetical protein	cas8b6	mkCas0111	CAS-I-B			
81377..82448	+	PZX51585.1	QKZU01000018.1	Algoriphagus ratkowskyi	Bacteria	Bacteroidetes	Cytophagia	Cytophagales	Cyclobacteriaceae	Algoriphagus	hypothetical protein	cas7	mkCas0088	CAS-I-B			
82476..83010	+	PZX51586.1	QKZU01000018.1	Algoriphagus ratkowskyi	Bacteria	Bacteroidetes	Cytophagia	Cytophagales	Cyclobacteriaceae	Algoriphagus	CRISPR-associated exonuclease Cas4	cas4	pfam01930	CAS-I			
83006..84011	+	PZX51587.1	QKZU01000018.1	Algoriphagus ratkowskyi	Bacteria	Bacteroidetes	Cytophagia	Cytophagales	Cyclobacteriaceae	Algoriphagus	CRISPR-associated protein Cas1	cas1	cd09722	CAS-I-B			
84012..84276	+	PZX51588.1	QKZU01000018.1	Algoriphagus ratkowskyi	Bacteria	Bacteroidetes	Cytophagia	Cytophagales	Cyclobacteriaceae	Algoriphagus	CRISPR-associated protein Cas2	cas2	COG1343	CAS-I,CAS-II,CAS-III			
84489..85109	array		QKZU01000018.1	Algoriphagus ratkowskyi	Bacteria	Bacteroidetes	Cytophagia	Cytophagales	Cyclobacteriaceae	Algoriphagus		CRISPR array			10	35	30
408 CAS-I-B | SEA59703.1																	
15443..16863	array		FNQF01000008.1	Psychroflexus halocasei	Bacteria	Bacteroidetes	Flavobacteriia	Flavobacteriales	Flavobacteriaceae	Psychroflexus		CRISPR array			22	36	30
16194..16371	-	SEA59607.1	FNQF01000008.1	Psychroflexus halocasei	Bacteria	Bacteroidetes	Flavobacteriia	Flavobacteriales	Flavobacteriaceae	Psychroflexus	hypothetical protein						
17069..17333	-	SEA59703.1	FNQF01000008.1	Psychroflexus halocasei	Bacteria	Bacteroidetes	Flavobacteriia	Flavobacteriales	Flavobacteriaceae	Psychroflexus	CRISPR-associated protein Cas2	cas2	cd09725	CAS-I,CAS-II,CAS-III,CAS-V			
17364..18369	-	SEA59739.1	FNQF01000008.1	Psychroflexus halocasei	Bacteria	Bacteroidetes	Flavobacteriia	Flavobacteriales	Flavobacteriaceae	Psychroflexus	CRISP-associated protein Cas1	cas1	cd09722	CAS-I-B			
18372..18897	-	SEA59769.1	FNQF01000008.1	Psychroflexus halocasei	Bacteria	Bacteroidetes	Flavobacteriia	Flavobacteriales	Flavobacteriaceae	Psychroflexus	CRISPR-associated exonuclease Cas4	cas4	pfam01930	CAS-I			
18896..21689	-	SEA59802.1	FNQF01000008.1	Psychroflexus halocasei	Bacteria	Bacteroidetes	Flavobacteriia	Flavobacteriales	Flavobacteriaceae	Psychroflexus	CRISPR-associated endonuclease/helicase Cas3	cas3HD,cas3	cd09641,COG1203	CAS-I			
21672..22443	-	SEA59834.1	FNQF01000008.1	Psychroflexus halocasei	Bacteria	Bacteroidetes	Flavobacteriia	Flavobacteriales	Flavobacteriaceae	Psychroflexus	CRISPR-associated protein Cas5h	cas5	cls001430	CAS-I-B			
22460..23375	-	SEA59876.1	FNQF01000008.1	Psychroflexus halocasei	Bacteria	Bacteroidetes	Flavobacteriia	Flavobacteriales	Flavobacteriaceae	Psychroflexus	CRISPR-associated protein Csh2	cas7b	pfam05107	CAS-I-B,CAS-I-C			
23371..25336	-	SEA59908.1	FNQF01000008.1	Psychroflexus halocasei	Bacteria	Bacteroidetes	Flavobacteriia	Flavobacteriales	Flavobacteriaceae	Psychroflexus	CRISPR-associated protein Csh1	cas8b1	mkCas0106	CAS-I-B			
25328..26129	-	SEA59943.1	FNQF01000008.1	Psychroflexus halocasei	Bacteria	Bacteroidetes	Flavobacteriia	Flavobacteriales	Flavobacteriaceae	Psychroflexus	CRISPR-associated endoribonuclease Cas6	cas6	COG1583	CAS-I,CAS-III,CAS-IV			
409 partial CAS-I | OJU50685.1																	
30..2265	array		MKRS01000417.1	Bacteroidales bacterium 45-6	Bacteria	Bacteroidetes	Bacteroidia	Bacteroidales				CRISPR array			35	35	29
2485..2749	-	OJU50685.1	MKRS01000417.1	Bacteroidales bacterium 45-6	Bacteria	Bacteroidetes	Bacteroidia	Bacteroidales			CRISPR-associated endonuclease Cas2	cas2	cd09725	CAS-I,CAS-II,CAS-III,CAS-V			
2797..3133	-	OJU50689.1	MKRS01000417.1	Bacteroidales bacterium 45-6	Bacteria	Bacteroidetes	Bacteroidia	Bacteroidales			four helix bundle protein						
3239..4247	-	OJU50686.1	MKRS01000417.1	Bacteroidales bacterium 45-6	Bacteria	Bacteroidetes	Bacteroidia	Bacteroidales			subtype I-B CRISPR-associated endonuclease Cas1	cas1	cd09722	CAS-I-B			
4356..4722	-	OJU50690.1	MKRS01000417.1	Bacteroidales bacterium 45-6	Bacteria	Bacteroidetes	Bacteroidia	Bacteroidales			four helix bundle protein						
4762..5275	-	OJU50687.1	MKRS01000417.1	Bacteroidales bacterium 45-6	Bacteria	Bacteroidetes	Bacteroidia	Bacteroidales			CRISPR-associated protein Cas4	cas4	pfam01930	CAS-I			
5279..5945	-	OJU50691.1	MKRS01000417.1	Bacteroidales bacterium 45-6	Bacteria	Bacteroidetes	Bacteroidia	Bacteroidales			hypothetical protein						
6101..8384	-	OJU50688.1	MKRS01000417.1	Bacteroidales bacterium 45-6	Bacteria	Bacteroidetes	Bacteroidia	Bacteroidales			hypothetical protein	cas3HD,cas3	cd09641,COG1203	CAS-I			
410 CAS-I-B | OJV54070.1																	
114449..116583	array		MKTB01000010.1	Bacteroidetes bacterium 43-16	Bacteria	Bacteroidetes						CRISPR array			33	35	30
116791..117055	-	OJV54070.1	MKTB01000010.1	Bacteroidetes bacterium 43-16	Bacteria	Bacteroidetes					CRISPR-associated endonuclease Cas2	cas2	cd09725	CAS-I,CAS-II,CAS-III,CAS-V			
117056..118061	-	OJV54071.1	MKTB01000010.1	Bacteroidetes bacterium 43-16	Bacteria	Bacteroidetes					subtype I-B CRISPR-associated endonuclease Cas1	cas1	cd09722	CAS-I-B			
118070..119030	-	OJV54072.1	MKTB01000010.1	Bacteroidetes bacterium 43-16	Bacteria	Bacteroidetes					death-on-curing protein						
119026..119533	-	OJV54073.1	MKTB01000010.1	Bacteroidetes bacterium 43-16	Bacteria	Bacteroidetes					CRISPR-associated protein Cas4	cas4	pfam01930	CAS-I			
119576..120632	-	OJV54074.1	MKTB01000010.1	Bacteroidetes bacterium 43-16	Bacteria	Bacteroidetes					CRISPR-associated protein Cas7	cas7	mkCas0088	CAS-I-B			
120653..121970	-	OJV54075.1	MKTB01000010.1	Bacteroidetes bacterium 43-16	Bacteria	Bacteroidetes					hypothetical protein	cas8b6	mkCas0111	CAS-I-B			
121966..124117	-	OJV54076.1	MKTB01000010.1	Bacteroidetes bacterium 43-16	Bacteria	Bacteroidetes					CRISPR-associated helicase/endonuclease Cas3	cas3HD,cas3	cd09641,COG1203	CAS-I			
124117..124702	-	OJV54077.1	MKTB01000010.1	Bacteroidetes bacterium 43-16	Bacteria	Bacteroidetes					hypothetical protein	cas5	mkCas0069	CAS-I-B			
124708..125368	-	OJV54078.1	MKTB01000010.1	Bacteroidetes bacterium 43-16	Bacteria	Bacteroidetes					CRISPR-associated endoribonuclease Cas6	cas6	COG1583	CAS-I,CAS-III,CAS-IV			
411 partial CAS-I-B | OJU29194.1																	
6705..9159	array		MKRO01000011.1	Sphingobacteriales bacterium 41-5	Bacteria	Bacteroidetes	Sphingobacteriia	Sphingobacteriales				CRISPR array			38	35	30
9236..9528	array		MKRO01000011.1	Sphingobacteriales bacterium 41-5	Bacteria	Bacteroidetes	Sphingobacteriia	Sphingobacteriales				CRISPR array			5	35	30
9815..11560	array		MKRO01000011.1	Sphingobacteriales bacterium 41-5	Bacteria	Bacteroidetes	Sphingobacteriia	Sphingobacteriales				CRISPR array			27	36	30
11639..12441	array		MKRO01000011.1	Sphingobacteriales bacterium 41-5	Bacteria	Bacteroidetes	Sphingobacteriia	Sphingobacteriales				CRISPR array			13	45	20
12648..12912	-	OJU29194.1	MKRO01000011.1	Sphingobacteriales bacterium 41-5	Bacteria	Bacteroidetes	Sphingobacteriia	Sphingobacteriales			CRISPR-associated endonuclease Cas2	cas2	cd09725	CAS-I,CAS-II,CAS-III,CAS-V			
12913..13918	-	OJU29195.1	MKRO01000011.1	Sphingobacteriales bacterium 41-5	Bacteria	Bacteroidetes	Sphingobacteriia	Sphingobacteriales			subtype I-B CRISPR-associated endonuclease Cas1	cas1	cd09722	CAS-I-B			
13914..14421	-	OJU29196.1	MKRO01000011.1	Sphingobacteriales bacterium 41-5	Bacteria	Bacteroidetes	Sphingobacteriia	Sphingobacteriales			CRISPR-associated protein Cas4	cas4	pfam01930	CAS-I			
412 CAS-I-B | AYO58291.1																	
1990244..1997068	array		CP023540.1	Chryseobacterium sp. 6424	Bacteria	Bacteroidetes	Flavobacteriia	Flavobacteriales	Flavobacteriaceae	Chryseobacterium		CRISPR array			104	35	30
1997282..1997546	-	AYO58291.1	CP023540.1	Chryseobacterium sp. 6424	Bacteria	Bacteroidetes	Flavobacteriia	Flavobacteriales	Flavobacteriaceae	Chryseobacterium	CRISPR-associated endonuclease Cas2	cas2	cd09725	CAS-I,CAS-II,CAS-III,CAS-V			
1997547..1998552	-	AYO58292.1	CP023540.1	Chryseobacterium sp. 6424	Bacteria	Bacteroidetes	Flavobacteriia	Flavobacteriales	Flavobacteriaceae	Chryseobacterium	subtype I-B CRISPR-associated endonuclease Cas1	cas1	cd09722	CAS-I-B			
1998560..1999316	-	AYO58293.1	CP023540.1	Chryseobacterium sp. 6424	Bacteria	Bacteroidetes	Flavobacteriia	Flavobacteriales	Flavobacteriaceae	Chryseobacterium	hypothetical protein						
1999318..1999822	-	AYO58294.1	CP023540.1	Chryseobacterium sp. 6424	Bacteria	Bacteroidetes	Flavobacteriia	Flavobacteriales	Flavobacteriaceae	Chryseobacterium	CRISPR-associated protein Cas4	cas4	pfam01930	CAS-I			
1999824..2002128	-	AYO58295.1	CP023540.1	Chryseobacterium sp. 6424	Bacteria	Bacteroidetes	Flavobacteriia	Flavobacteriales	Flavobacteriaceae	Chryseobacterium	hypothetical protein	cas3HD,cas3	cd09641,COG1203	CAS-I			
2002117..2002798	-	AYO58296.1	CP023540.1	Chryseobacterium sp. 6424	Bacteria	Bacteroidetes	Flavobacteriia	Flavobacteriales	Flavobacteriaceae	Chryseobacterium	hypothetical protein	cas5	cd09658	CAS-I-B			
2002790..2003819	-	AYO58297.1	CP023540.1	Chryseobacterium sp. 6424	Bacteria	Bacteroidetes	Flavobacteriia	Flavobacteriales	Flavobacteriaceae	Chryseobacterium	type I-B CRISPR-associated protein Cas7/Cst2/DevR	cas7	cd09687	CAS-I-B			
2003830..2005855	-	AYO58298.1	CP023540.1	Chryseobacterium sp. 6424	Bacteria	Bacteroidetes	Flavobacteriia	Flavobacteriales	Flavobacteriaceae	Chryseobacterium	hypothetical protein	Cas8b12	icity0007				
2005901..2006684	-	AYO58299.1	CP023540.1	Chryseobacterium sp. 6424	Bacteria	Bacteroidetes	Flavobacteriia	Flavobacteriales	Flavobacteriaceae	Chryseobacterium	CRISPR-associated endoribonuclease Cas6	cas6	COG1583	CAS-I,CAS-III,CAS-IV			
413 CAS-I-B | SIT97704.1																	
15323..15973	array		FTPU01000029.1	Chryseobacterium bovis DSM 19482	Bacteria	Bacteroidetes	Flavobacteriia	Flavobacteriales	Flavobacteriaceae	Chryseobacterium		CRISPR array			11	44	19
16191..16455	-	SIT97704.1	FTPU01000029.1	Chryseobacterium bovis DSM 19482	Bacteria	Bacteroidetes	Flavobacteriia	Flavobacteriales	Flavobacteriaceae	Chryseobacterium	CRISPR-associated protein Cas2	cas2	cd09725	CAS-I,CAS-II,CAS-III,CAS-V			
16455..17460	-	SIT97705.1	FTPU01000029.1	Chryseobacterium bovis DSM 19482	Bacteria	Bacteroidetes	Flavobacteriia	Flavobacteriales	Flavobacteriaceae	Chryseobacterium	CRISP-associated protein Cas1	cas1	cd09722	CAS-I-B			

17686..18235	-	SIT97706.1	FTPU01000029.1	Chryseobacterium bovis DSM 19482	Bacteria	Bacteroidetes	Flavobacteriia	Flavobacteriales	Flavobacteriaceae	Chryseobacterium	REP element-mobilizing transposase RayT						
18240..18747	-	SIT97707.1	FTPU01000029.1	Chryseobacterium bovis DSM 19482	Bacteria	Bacteroidetes	Flavobacteriia	Flavobacteriales	Flavobacteriaceae	Chryseobacterium	CRISPR-associated exonuclease Cas4	cas4	pfam01930	CAS-I			
18988..20053	-	SIT97708.1	FTPU01000029.1	Chryseobacterium bovis DSM 19482	Bacteria	Bacteroidetes	Flavobacteriia	Flavobacteriales	Flavobacteriaceae	Chryseobacterium	hypothetical protein	cas7	mkCas0088	CAS-I-B			
20073..21396	-	SIT97709.1	FTPU01000029.1	Chryseobacterium bovis DSM 19482	Bacteria	Bacteroidetes	Flavobacteriia	Flavobacteriales	Flavobacteriaceae	Chryseobacterium	hypothetical protein	cas8b6	mkCas0111	CAS-I-B			
21392..23540	-	SIT97710.1	FTPU01000029.1	Chryseobacterium bovis DSM 19482	Bacteria	Bacteroidetes	Flavobacteriia	Flavobacteriales	Flavobacteriaceae	Chryseobacterium	CRISPR-associated endonuclease/helicase Cas3	cas3HD,cas3	cd09641,COG1203	CAS-I			
23539..24130	-	SIT97711.1	FTPU01000029.1	Chryseobacterium bovis DSM 19482	Bacteria	Bacteroidetes	Flavobacteriia	Flavobacteriales	Flavobacteriaceae	Chryseobacterium	hypothetical protein	cas5	mkCas0069	CAS-I-B			
24138..24798	-	SIT97712.1	FTPU01000029.1	Chryseobacterium bovis DSM 19482	Bacteria	Bacteroidetes	Flavobacteriia	Flavobacteriales	Flavobacteriaceae	Chryseobacterium	CRISPR-associated endoribonuclease Cas6	cas6	COG1583	CAS-I,CAS-III,CAS-IV			
25026..25377	-	SIT97713.1	FTPU01000029.1	Chryseobacterium bovis DSM 19482	Bacteria	Bacteroidetes	Flavobacteriia	Flavobacteriales	Flavobacteriaceae	Chryseobacterium	CRISPR-associated endoribonuclease Cas6	cas6	COG1583	CAS-I,CAS-III,CAS-IV			
414 CAS-I | OJY86468.1																	
159269..160067	+	OJY86459.1	MKWG01000027.1	Sphingobacteriales bacterium 44-15	Bacteria	Bacteroidetes	Sphingobacteriia	Sphingobacteriales			CRISPR-associated endoribonuclease Cas6	cas6	COG1583	CAS-I,CAS-III,CAS-IV			
160203..160608	+	OJY86460.1	MKWG01000027.1	Sphingobacteriales bacterium 44-15	Bacteria	Bacteroidetes	Sphingobacteriia	Sphingobacteriales			transcriptional regulator						
161179..162595	+	OJY86461.1	MKWG01000027.1	Sphingobacteriales bacterium 44-15	Bacteria	Bacteroidetes	Sphingobacteriia	Sphingobacteriales			hypothetical protein	cas8b2	cls000029	CAS-I-B			
162612..163701	+	OJY86462.1	MKWG01000027.1	Sphingobacteriales bacterium 44-15	Bacteria	Bacteroidetes	Sphingobacteriia	Sphingobacteriales			type I-B CRISPR-associated protein Cas7/Cst2/DevR	cas7	cd09687	CAS-I-B			
163704..164373	+	OJY86463.1	MKWG01000027.1	Sphingobacteriales bacterium 44-15	Bacteria	Bacteroidetes	Sphingobacteriia	Sphingobacteriales			type I-B CRISPR-associated protein Cas5	cas5	COG1688	CAS-I			
164373..166674	+	OJY86464.1	MKWG01000027.1	Sphingobacteriales bacterium 44-15	Bacteria	Bacteroidetes	Sphingobacteriia	Sphingobacteriales			hypothetical protein	cas3HD,cas3	cd09641,COG1203	CAS-I			
166749..167280	+	OJY86465.1	MKWG01000027.1	Sphingobacteriales bacterium 44-15	Bacteria	Bacteroidetes	Sphingobacteriia	Sphingobacteriales			CRISPR-associated protein Cas4	cas4	pfam01930	CAS-I			
167440..167869	+	OJY86466.1	MKWG01000027.1	Sphingobacteriales bacterium 44-15	Bacteria	Bacteroidetes	Sphingobacteriia	Sphingobacteriales			GxxExxY protein						
167865..168870	+	OJY86467.1	MKWG01000027.1	Sphingobacteriales bacterium 44-15	Bacteria	Bacteroidetes	Sphingobacteriia	Sphingobacteriales			subtype I-B CRISPR-associated endonuclease Cas1	cas1	cd09722	CAS-I-B			
168871..169135	+	OJY86468.1	MKWG01000027.1	Sphingobacteriales bacterium 44-15	Bacteria	Bacteroidetes	Sphingobacteriia	Sphingobacteriales			CRISPR-associated endonuclease Cas2	cas2	cd09725	CAS-I,CAS-II,CAS-III,CAS-V			
169344..173136	array		MKWG01000027.1	Sphingobacteriales bacterium 44-15	Bacteria	Bacteroidetes	Sphingobacteriia	Sphingobacteriales				CRISPR array			59	35	29
415 CAS-I | PVD52179.1																	
43577..44375	+	PVD52169.1	QCZJ01000008.1	Terrimonas sp. NS-102	Bacteria	Bacteroidetes	Chitinophagia	Chitinophagales	Chitinophagaceae	Terrimonas	CRISPR-associated endoribonuclease Cas6	cas6	COG1583	CAS-I,CAS-III,CAS-IV			
44553..44916	+	PVD52170.1	QCZJ01000008.1	Terrimonas sp. NS-102	Bacteria	Bacteroidetes	Chitinophagia	Chitinophagales	Chitinophagaceae	Terrimonas	transcriptional regulator						
45134..45413	+	PVD52171.1	QCZJ01000008.1	Terrimonas sp. NS-102	Bacteria	Bacteroidetes	Chitinophagia	Chitinophagales	Chitinophagaceae	Terrimonas	hypothetical protein						
45462..47997	+	PVD52172.1	QCZJ01000008.1	Terrimonas sp. NS-102	Bacteria	Bacteroidetes	Chitinophagia	Chitinophagales	Chitinophagaceae	Terrimonas	CRISPR-associated helicase Cas3'	cas3,cas3HD	COG1203,cd09641	CAS-I			
47986..49756	+	PVD52173.1	QCZJ01000008.1	Terrimonas sp. NS-102	Bacteria	Bacteroidetes	Chitinophagia	Chitinophagales	Chitinophagaceae	Terrimonas	hypothetical protein	cas8a4	cls000706	CAS-I-A			
49781..50768	+	PVD52174.1	QCZJ01000008.1	Terrimonas sp. NS-102	Bacteria	Bacteroidetes	Chitinophagia	Chitinophagales	Chitinophagaceae	Terrimonas	DevR family CRISPR-associated autoregulator	cas7	pfam01905	CAS-I			
50773..51442	+	PVD52175.1	QCZJ01000008.1	Terrimonas sp. NS-102	Bacteria	Bacteroidetes	Chitinophagia	Chitinophagales	Chitinophagaceae	Terrimonas	hypothetical protein	cas5	cls000048	CAS-I-A			
51641..52793	+	PVD52305.1	QCZJ01000008.1	Terrimonas sp. NS-102	Bacteria	Bacteroidetes	Chitinophagia	Chitinophagales	Chitinophagaceae	Terrimonas	ATPase						
52792..53299	+	PVD52176.1	QCZJ01000008.1	Terrimonas sp. NS-102	Bacteria	Bacteroidetes	Chitinophagia	Chitinophagales	Chitinophagaceae	Terrimonas	CRISPR-associated protein Cas4	cas4	pfam01930	CAS-I			
53486..53888	+	PVD52177.1	QCZJ01000008.1	Terrimonas sp. NS-102	Bacteria	Bacteroidetes	Chitinophagia	Chitinophagales	Chitinophagaceae	Terrimonas	GxxExxY protein						
53884..54889	+	PVD52178.1	QCZJ01000008.1	Terrimonas sp. NS-102	Bacteria	Bacteroidetes	Chitinophagia	Chitinophagales	Chitinophagaceae	Terrimonas	subtype I-B CRISPR-associated endonuclease Cas1	cas1	cd09722	CAS-I-B			
54946..55210	+	PVD52179.1	QCZJ01000008.1	Terrimonas sp. NS-102	Bacteria	Bacteroidetes	Chitinophagia	Chitinophagales	Chitinophagaceae	Terrimonas	CRISPR-associated endonuclease Cas2	cas2	cd09725	CAS-I,CAS-II,CAS-III,CAS-V			
55419..57714	array		QCZJ01000008.1	Terrimonas sp. NS-102	Bacteria	Bacteroidetes	Chitinophagia	Chitinophagales	Chitinophagaceae	Terrimonas		CRISPR array			36	35	29
416 CAS-I-B | EHQ30583.1																	
7590628..7592521	+	EHQ30574.1	CM001403.1	Mucilaginibacter paludis DSM 18603	Bacteria	Bacteroidetes	Sphingobacteriia	Sphingobacteriales	Sphingobacteriaceae	Mucilaginibacter	ATP-dependent DNA helicase, RecQ family	cas3	cd09639	CAS-I			
7592886..7594011	-	EHQ30575.1	CM001403.1	Mucilaginibacter paludis DSM 18603	Bacteria	Bacteroidetes	Sphingobacteriia	Sphingobacteriales	Sphingobacteriaceae	Mucilaginibacter	FO synthase, subunit 2						
7594206..7594989	+	EHQ30576.1	CM001403.1	Mucilaginibacter paludis DSM 18603	Bacteria	Bacteroidetes	Sphingobacteriia	Sphingobacteriales	Sphingobacteriaceae	Mucilaginibacter	CRISPR-associated protein Cas6	cas6	COG1583	CAS-I,CAS-III,CAS-IV			
7595314..7597177	+	EHQ30577.1	CM001403.1	Mucilaginibacter paludis DSM 18603	Bacteria	Bacteroidetes	Sphingobacteriia	Sphingobacteriales	Sphingobacteriaceae	Mucilaginibacter	hypothetical protein	cas8b1	mkCas0106	CAS-I-B			
7597197..7598130	+	EHQ30578.1	CM001403.1	Mucilaginibacter paludis DSM 18603	Bacteria	Bacteroidetes	Sphingobacteriia	Sphingobacteriales	Sphingobacteriaceae	Mucilaginibacter	CRISPR-associated protein	cas7b	pfam05107	CAS-I-B,CAS-I-C			
7598136..7598910	+	EHQ30579.1	CM001403.1	Mucilaginibacter paludis DSM 18603	Bacteria	Bacteroidetes	Sphingobacteriia	Sphingobacteriales	Sphingobacteriaceae	Mucilaginibacter	CRISPR-associated protein Cas5	cas5	cls001430	CAS-I-B			
7598884..7601641	+	EHQ30580.1	CM001403.1	Mucilaginibacter paludis DSM 18603	Bacteria	Bacteroidetes	Sphingobacteriia	Sphingobacteriales	Sphingobacteriaceae	Mucilaginibacter	CRISPR-associated helicase Cas3	cas3HD,cas3	cd09641,COG1203	CAS-I			
7601647..7602160	+	EHQ30581.1	CM001403.1	Mucilaginibacter paludis DSM 18603	Bacteria	Bacteroidetes	Sphingobacteriia	Sphingobacteriales	Sphingobacteriaceae	Mucilaginibacter	CRISPR-associated protein Cas4	cas4	pfam01930	CAS-I			
7602172..7603177	+	EHQ30582.1	CM001403.1	Mucilaginibacter paludis DSM 18603	Bacteria	Bacteroidetes	Sphingobacteriia	Sphingobacteriales	Sphingobacteriaceae	Mucilaginibacter	CRISPR-associated protein Cas1	cas1	cd09722	CAS-I-B			
7603201..7603465	+	EHQ30583.1	CM001403.1	Mucilaginibacter paludis DSM 18603	Bacteria	Bacteroidetes	Sphingobacteriia	Sphingobacteriales	Sphingobacteriaceae	Mucilaginibacter	CRISPR-associated protein Cas2	cas2	cd09725	CAS-I,CAS-II,CAS-III,CAS-V			
7603673..7619567	array		CM001403.1	Mucilaginibacter paludis DSM 18603	Bacteria	Bacteroidetes	Sphingobacteriia	Sphingobacteriales	Sphingobacteriaceae	Mucilaginibacter		CRISPR array			243	35	30
417 CAS-I-B | EHO40941.1																	
1700677..1701475	+	EHO40934.1	CM001402.1	Caldithrix abyssi DSM 13497	Bacteria	Calditrichaeota	Calditrichae	Calditrichales	Calditrichaceae	Caldithrix	CRISPR-associated protein Cas6	cas6	COG1583	CAS-I,CAS-III,CAS-IV			
1701498..1703322	+	EHO40935.1	CM001402.1	Caldithrix abyssi DSM 13497	Bacteria	Calditrichaeota	Calditrichae	Calditrichales	Calditrichaceae	Caldithrix	hypothetical protein	cas8b1	mkCas0106	CAS-I-B			
1703356..1704304	+	EHO40936.1	CM001402.1	Caldithrix abyssi DSM 13497	Bacteria	Calditrichaeota	Calditrichae	Calditrichales	Calditrichaceae	Caldithrix	CRISPR-associated protein TM1801	cas7b	pfam05107	CAS-I-B,CAS-I-C			
1704306..1705098	+	EHO40937.1	CM001402.1	Caldithrix abyssi DSM 13497	Bacteria	Calditrichaeota	Calditrichae	Calditrichales	Calditrichaceae	Caldithrix	CRISPR-associated protein Cas5, Hmari subtype	cas5	cls001430	CAS-I-B			
1705078..1707796	+	EHO40938.1	CM001402.1	Caldithrix abyssi DSM 13497	Bacteria	Calditrichaeota	Calditrichae	Calditrichales	Calditrichaceae	Caldithrix	CRISPR-associated HD domain protein	cas3HD,cas3	cd09641,COG1203	CAS-I			
1707811..1708321	+	EHO40939.1	CM001402.1	Caldithrix abyssi DSM 13497	Bacteria	Calditrichaeota	Calditrichae	Calditrichales	Calditrichaceae	Caldithrix	CRISPR-associated protein Cas4	cas4	pfam01930	CAS-I			
1708363..1709368	+	EHO40940.1	CM001402.1	Caldithrix abyssi DSM 13497	Bacteria	Calditrichaeota	Calditrichae	Calditrichales	Calditrichaceae	Caldithrix	CRISPR-associated protein Cas1	cas1	cd09722	CAS-I-B			
1709388..1709652	+	EHO40941.1	CM001402.1	Caldithrix abyssi DSM 13497	Bacteria	Calditrichaeota	Calditrichae	Calditrichales	Calditrichaceae	Caldithrix	CRISPR-associated protein Cas2	cas2	COG1343	CAS-I,CAS-II,CAS-III			
1709776..1711789	array		CM001402.1	Caldithrix abyssi DSM 13497	Bacteria	Calditrichaeota	Calditrichae	Calditrichales	Calditrichaceae	Caldithrix		CRISPR array			31	36	30
418 CAS-I-B | APF20548.1																	
4498966..4500979	array		CP018099.1	Caldithrix abyssi DSM 13497	Bacteria	Calditrichaeota	Calditrichae	Calditrichales	Calditrichaceae	Caldithrix		CRISPR array			31	36	30
4501102..4501366	-	APF20548.1	CP018099.1	Caldithrix abyssi DSM 13497	Bacteria	Calditrichaeota	Calditrichae	Calditrichales	Calditrichaceae	Caldithrix	cas2 CRISPR-associated protein, Cas2 family	cas2	COG1343	CAS-I,CAS-II,CAS-III			
4501386..4502391	-	APF20549.1	CP018099.1	Caldithrix abyssi DSM 13497	Bacteria	Calditrichaeota	Calditrichae	Calditrichales	Calditrichaceae	Caldithrix	cas1 CRISPR-associated protein, Cas1 family	cas1	cd09722	CAS-I-B			
4502433..4502943	-	APF20550.1	CP018099.1	Caldithrix abyssi DSM 13497	Bacteria	Calditrichaeota	Calditrichae	Calditrichales	Calditrichaceae	Caldithrix	CRISPR-associated exonuclease, Cas4 family	cas4	pfam01930	CAS-I			
4502958..4505676	-	APF20551.1	CP018099.1	Caldithrix abyssi DSM 13497	Bacteria	Calditrichaeota	Calditrichae	Calditrichales	Calditrichaceae	Caldithrix	CRISPR-associated endonuclease/helicase Cas3	cas3HD,cas3	cd09641,COG1203	CAS-I			
4505656..4506448	-	APF20552.1	CP018099.1	Caldithrix abyssi DSM 13497	Bacteria	Calditrichaeota	Calditrichae	Calditrichales	Calditrichaceae	Caldithrix	CRISPR-associated protein Cas5h	cas5	cls001430	CAS-I-B			
4506450..4507398	-	APF20553.1	CP018099.1	Caldithrix abyssi DSM 13497	Bacteria	Calditrichaeota	Calditrichae	Calditrichales	Calditrichaceae	Caldithrix	CRISPR-associated protein Csh2	cas7b	pfam05107	CAS-I-B,CAS-I-C			
4507432..4509256	-	APF20554.1	CP018099.1	Caldithrix abyssi DSM 13497	Bacteria	Calditrichaeota	Calditrichae	Calditrichales	Calditrichaceae	Caldithrix	CRISPR-associated protein Csh1	cas8b1	mkCas0106	CAS-I-B			
4509279..4510077	-	APF20555.1	CP018099.1	Caldithrix abyssi DSM 13497	Bacteria	Calditrichaeota	Calditrichae	Calditrichales	Calditrichaceae	Caldithrix	CRISPR-associated endoribonuclease Cas6	cas6	COG1583	CAS-I,CAS-III,CAS-IV			
419 CAS-I-B | SFO06540.1																	
345888..346686	+	SFO06373.1	FOVW01000003.1	Algoriphagus ornithinivorans	Bacteria	Bacteroidetes	Cytophagia	Cytophagales	Cyclobacteriaceae	Algoriphagus	CRISPR-associated protein, Cas6 family	cas6	cd09759	CAS-I-A			
346682..348551	+	SFO06397.1	FOVW01000003.1	Algoriphagus ornithinivorans	Bacteria	Bacteroidetes	Cytophagia	Cytophagales	Cyclobacteriaceae	Algoriphagus	CRISPR-associated protein Csh1	cas8b1	mkCas0106	CAS-I-B			
348547..349480	+	SFO06416.1	FOVW01000003.1	Algoriphagus ornithinivorans	Bacteria	Bacteroidetes	Cytophagia	Cytophagales	Cyclobacteriaceae	Algoriphagus	CRISPR-associated protein Csh2	cas7b	pfam05107	CAS-I-B,CAS-I-C			
349482..350265	+	SFO06432.1	FOVW01000003.1	Algoriphagus ornithinivorans	Bacteria	Bacteroidetes	Cytophagia	Cytophagales	Cyclobacteriaceae	Algoriphagus	CRISPR-associated protein Cas5h	cas5	cls001430	CAS-I-B			
350267..352946	+	SFO06457.1	FOVW01000003.1	Algoriphagus ornithinivorans	Bacteria	Bacteroidetes	Cytophagia	Cytophagales	Cyclobacteriaceae	Algoriphagus	CRISPR-associated endonuclease/helicase Cas3	cas3HD,cas3	cd09641,COG1203	CAS-I			
352947..353451	+	SFO06477.1	FOVW01000003.1	Algoriphagus ornithinivorans	Bacteria	Bacteroidetes	Cytophagia	Cytophagales	Cyclobacteriaceae	Algoriphagus	CRISPR-associated exonuclease, Cas4 family	cas4	pfam01930	CAS-I			
353447..354452	+	SFO06512.1	FOVW01000003.1	Algoriphagus ornithinivorans	Bacteria	Bacteroidetes	Cytophagia	Cytophagales	Cyclobacteriaceae	Algoriphagus	CRISPR-associated protein, Cas1 family	cas1	cd09722	CAS-I-B			
354453..354717	+	SFO06540.1	FOVW01000003.1	Algoriphagus ornithinivorans	Bacteria	Bacteroidetes	Cytophagia	Cytophagales	Cyclobacteriaceae	Algoriphagus	CRISPR-associated protein, Cas2 family	cas2	COG1343	CAS-I,CAS-II,CAS-III			
356708..356935	array		FOVW01000003.1	Algoriphagus ornithinivorans	Bacteria	Bacteroidetes	Cytophagia	Cytophagales	Cyclobacteriaceae	Algoriphagus		CRISPR array			4	36	30
420 CAS-I-B | SEG40668.1																	
7..168	array		FNVR01000033.1	Algoriphagus boritolerans DSM 17298 = JCM 18970	Bacteria	Bacteroidetes	Cytophagia	Cytophagales	Cyclobacteriaceae	Algoriphagus		CRISPR array			3	36	30
381..645	-	SEG40668.1	FNVR01000033.1	Algoriphagus boritolerans DSM 17298 = JCM 18970	Bacteria	Bacteroidetes	Cytophagia	Cytophagales	Cyclobacteriaceae	Algoriphagus	CRISPR-associated protein Cas2	cas2	COG1343	CAS-I,CAS-II,CAS-III			
646..1651	-	SEG40685.1	FNVR01000033.1	Algoriphagus boritolerans DSM 17298 = JCM 18970	Bacteria	Bacteroidetes	Cytophagia	Cytophagales	Cyclobacteriaceae	Algoriphagus	CRISP-associated protein Cas1	cas1	cd09722	CAS-I-B			
1710..2274	-	SEG40697.1	FNVR01000033.1	Algoriphagus boritolerans DSM 17298 = JCM 18970	Bacteria	Bacteroidetes	Cytophagia	Cytophagales	Cyclobacteriaceae	Algoriphagus	pRiA4b ORF-3-like protein						
2311..2833	-	SEG40711.1	FNVR01000033.1	Algoriphagus boritolerans DSM 17298 = JCM 18970	Bacteria	Bacteroidetes	Cytophagia	Cytophagales	Cyclobacteriaceae	Algoriphagus	CRISPR-associated exonuclease Cas4	cas4	pfam01930	CAS-I			
2829..3900	-	SEG40729.1	FNVR01000033.1	Algoriphagus boritolerans DSM 17298 = JCM 18970	Bacteria	Bacteroidetes	Cytophagia	Cytophagales	Cyclobacteriaceae	Algoriphagus	Predicted nuclease of restriction endonuclease-like (RecB) superfamily, DUF1016 family	PD-DExK	COG4804	CAS-I,CAS-III			
3896..6689	-	SEG40741.1	FNVR01000033.1	Algoriphagus boritolerans DSM 17298 = JCM 18970	Bacteria	Bacteroidetes	Cytophagia	Cytophagales	Cyclobacteriaceae	Algoriphagus	CRISPR-associated endonuclease/helicase Cas3	cas3HD,cas3	cd09641,COG1203	CAS-I			
6672..7446	-	SEG40756.1	FNVR01000033.1	Algoriphagus boritolerans DSM 17298 = JCM 18970	Bacteria	Bacteroidetes	Cytophagia	Cytophagales	Cyclobacteriaceae	Algoriphagus	CRISPR-associated protein Cas5h	cas5	cls001430	CAS-I-B			
7460..8372	-	SEG40775.1	FNVR01000033.1	Algoriphagus boritolerans DSM 17298 = JCM 18970	Bacteria	Bacteroidetes	Cytophagia	Cytophagales	Cyclobacteriaceae	Algoriphagus	CRISPR-associated protein Csh2	cas7b	pfam05107	CAS-I-B,CAS-I-C			
8377..10318	-	SEG40796.1	FNVR01000033.1	Algoriphagus boritolerans DSM 17298 = JCM 18970	Bacteria	Bacteroidetes	Cytophagia	Cytophagales	Cyclobacteriaceae	Algoriphagus	CRISPR-associated protein Csh1	cas8b1	mkCas0106	CAS-I-B			
10314..11112	-	SEG40814.1	FNVR01000033.1	Algoriphagus boritolerans DSM 17298 = JCM 18970	Bacteria	Bacteroidetes	Cytophagia	Cytophagales	Cyclobacteriaceae	Algoriphagus	CRISPR-associated endoribonuclease Cas6	cas6	COG1583	CAS-I,CAS-III,CAS-IV			
421 CAS-I-B | PRY48837.1																	
19266..20536	array		PVTH01000013.1	Arcticibacter pallidicorallinus	Bacteria	Bacteroidetes	Sphingobacteriia	Sphingobacteriales	Sphingobacteriaceae	Arcticibacter		CRISPR array			20	35	30
20742..21006	-	PRY48837.1	PVTH01000013.1	Arcticibacter pallidicorallinus	Bacteria	Bacteroidetes	Sphingobacteriia	Sphingobacteriales	Sphingobacteriaceae	Arcticibacter	CRISPR-associated Cas2 family protein	cas2	COG1343	CAS-I,CAS-II,CAS-III			
21028..22033	-	PRY48838.1	PVTH01000013.1	Arcticibacter pallidicorallinus	Bacteria	Bacteroidetes	Sphingobacteriia	Sphingobacteriales	Sphingobacteriaceae	Arcticibacter	CRISPR-associated Cas1 family protein	cas1	cd09722	CAS-I-B			
22092..22602	-	PRY48839.1	PVTH01000013.1	Arcticibacter pallidicorallinus	Bacteria	Bacteroidetes	Sphingobacteriia	Sphingobacteriales	Sphingobacteriaceae	Arcticibacter	CRISPR-associated Cas4 family exonuclease	cas4	pfam01930	CAS-I			
22608..25314	-	PRY48840.1	PVTH01000013.1	Arcticibacter pallidicorallinus	Bacteria	Bacteroidetes	Sphingobacteriia	Sphingobacteriales	Sphingobacteriaceae	Arcticibacter	CRISPR-associated endonuclease/helicase Cas3	cas3HD,cas3	cd09641,COG1203	CAS-I			
25330..26128	-	PRY48841.1	PVTH01000013.1	Arcticibacter pallidicorallinus	Bacteria	Bacteroidetes	Sphingobacteriia	Sphingobacteriales	Sphingobacteriaceae	Arcticibacter	CRISPR-associated protein Cas5h	cas5	cls001430	CAS-I-B			
26129..27062	-	PRY48842.1	PVTH01000013.1	Arcticibacter pallidicorallinus	Bacteria	Bacteroidetes	Sphingobacteriia	Sphingobacteriales	Sphingobacteriaceae	Arcticibacter	CRISPR-associated protein Csh2	cas7b	pfam05107	CAS-I-B,CAS-I-C			
27080..28952	-	PRY48843.1	PVTH01000013.1	Arcticibacter pallidicorallinus	Bacteria	Bacteroidetes	Sphingobacteriia	Sphingobacteriales	Sphingobacteriaceae	Arcticibacter	CRISPR-associated protein Csh1	cas8b1	mkCas0106	CAS-I-B			
28979..29768	-	PRY48844.1	PVTH01000013.1	Arcticibacter pallidicorallinus	Bacteria	Bacteroidetes	Sphingobacteriia	Sphingobacteriales	Sphingobacteriaceae	Arcticibacter	CRISPR-associated Cas6 family protein	cas6	COG1583	CAS-I,CAS-III,CAS-IV			
422 CAS-I-B | PTB97787.1																	
0..320	+	PTB97780.1	PYVU01000005.1	Marivirga lumbricoides	Bacteria	Bacteroidetes	Cytophagia	Cytophagales	Flammeovirgaceae	Marivirga	CRISPR-associated endoribonuclease Cas6	cas6	pfam01881	CAS-I,CAS-III			
375..978	+	PTB97781.1	PYVU01000005.1	Marivirga lumbricoides	Bacteria	Bacteroidetes	Cytophagia	Cytophagales	Flammeovirgaceae	Marivirga	hypothetical protein	cas5	mkCas0069	CAS-I-B			
977..3125	+	PTB97782.1	PYVU01000005.1	Marivirga lumbricoides	Bacteria	Bacteroidetes	Cytophagia	Cytophagales	Flammeovirgaceae	Marivirga	CRISPR-associated helicase/endonuclease Cas3	cas3HD,cas3	cd09641,COG1203	CAS-I			
3121..4462	+	PTB97783.1	PYVU01000005.1	Marivirga lumbricoides	Bacteria	Bacteroidetes	Cytophagia	Cytophagales	Flammeovirgaceae	Marivirga	hypothetical protein	cas8b6	mkCas0111	CAS-I-B			
4523..5585	+	PTB97784.1	PYVU01000005.1	Marivirga lumbricoides	Bacteria	Bacteroidetes	Cytophagia	Cytophagales	Flammeovirgaceae	Marivirga	CRISPR-associated protein Cas7	cas7	mkCas0088	CAS-I-B			
5645..6158	+	PTB97785.1	PYVU01000005.1	Marivirga lumbricoides	Bacteria	Bacteroidetes	Cytophagia	Cytophagales	Flammeovirgaceae	Marivirga	CRISPR-associated protein Cas4	cas4	pfam01930	CAS-I			
6175..7180	+	PTB97786.1	PYVU01000005.1	Marivirga lumbricoides	Bacteria	Bacteroidetes	Cytophagia	Cytophagales	Flammeovirgaceae	Marivirga	subtype I-B CRISPR-associated endonuclease Cas1	cas1	cd09722	CAS-I-B			
7701..7965	+	PTB97787.1	PYVU01000005.1	Marivirga lumbricoides	Bacteria	Bacteroidetes	Cytophagia	Cytophagales	Flammeovirgaceae	Marivirga	CRISPR-associated endonuclease Cas2	cas2	cd09725	CAS-I,CAS-II,CAS-III,CAS-V			
8180..13995	array		PYVU01000005.1	Marivirga lumbricoides	Bacteria	Bacteroidetes	Cytophagia	Cytophagales	Flammeovirgaceae	Marivirga		CRISPR array			89	35	30
423 CAS-I-B | SHN04424.1																	
473817..474474	+	SHN04270.1	FRCY01000005.1	Cyclobacterium lianum	Bacteria	Bacteroidetes	Cytophagia	Cytophagales	Cyclobacteriaceae	Cyclobacterium	CRISPR-associated endoribonuclease Cas6	cas6	cd09652	CAS-I,CAS-III			
474481..475066	+	SHN04289.1	FRCY01000005.1	Cyclobacterium lianum	Bacteria	Bacteroidetes	Cytophagia	Cytophagales	Cyclobacteriaceae	Cyclobacterium	hypothetical protein	cas5	mkCas0069	CAS-I-B			
475065..477213	+	SHN04304.1	FRCY01000005.1	Cyclobacterium lianum	Bacteria	Bacteroidetes	Cytophagia	Cytophagales	Cyclobacteriaceae	Cyclobacterium	CRISPR-associated endonuclease/helicase Cas3	cas3HD,cas3	cd09641,COG1203	CAS-I			
477209..478526	+	SHN04322.1	FRCY01000005.1	Cyclobacterium lianum	Bacteria	Bacteroidetes	Cytophagia	Cytophagales	Cyclobacteriaceae	Cyclobacterium	hypothetical protein	cas8b6	mkCas0111	CAS-I-B			
478544..479618	+	SHN04340.1	FRCY01000005.1	Cyclobacterium lianum	Bacteria	Bacteroidetes	Cytophagia	Cytophagales	Cyclobacteriaceae	Cyclobacterium	hypothetical protein	cas7	mkCas0088	CAS-I-B			
479689..480055	+	SHN04359.1	FRCY01000005.1	Cyclobacterium lianum	Bacteria	Bacteroidetes	Cytophagia	Cytophagales	Cyclobacteriaceae	Cyclobacterium	hypothetical protein						
480717..481242	+	SHN04375.1	FRCY01000005.1	Cyclobacterium lianum	Bacteria	Bacteroidetes	Cytophagia	Cytophagales	Cyclobacteriaceae	Cyclobacterium	CRISPR-associated exonuclease Cas4	cas4	pfam01930	CAS-I			
481378..482383	+	SHN04389.1	FRCY01000005.1	Cyclobacterium lianum	Bacteria	Bacteroidetes	Cytophagia	Cytophagales	Cyclobacteriaceae	Cyclobacterium	CRISP-associated protein Cas1	cas1	cd09722	CAS-I-B			
482447..482807	+	SHN04409.1	FRCY01000005.1	Cyclobacterium lianum	Bacteria	Bacteroidetes	Cytophagia	Cytophagales	Cyclobacteriaceae	Cyclobacterium	four helix bundle protein						
482848..483112	+	SHN04424.1	FRCY01000005.1	Cyclobacterium lianum	Bacteria	Bacteroidetes	Cytophagia	Cytophagales	Cyclobacteriaceae	Cyclobacterium	CRISPR-associated protein Cas2	cas2	cd09725	CAS-I,CAS-II,CAS-III,CAS-V			
424 CAS-I-B | SMD44428.1																	
3531028..3531584	array		LT838813.1	Aquiflexum balticum DSM 16537	Bacteria	Bacteroidetes	Cytophagia	Cytophagales	Cyclobacteriaceae	Aquiflexum		CRISPR array			9	35	30
3531669..3532356	array		LT838813.1	Aquiflexum balticum DSM 16537	Bacteria	Bacteroidetes	Cytophagia	Cytophagales	Cyclobacteriaceae	Aquiflexum		CRISPR array			11	35	30
3532365..3533886	-	SMD44427.1	LT838813.1	Aquiflexum balticum DSM 16537	Bacteria	Bacteroidetes	Cytophagia	Cytophagales	Cyclobacteriaceae	Aquiflexum	Transposase						
3533945..3540879	array		LT838813.1	Aquiflexum balticum DSM 16537	Bacteria	Bacteroidetes	Cytophagia	Cytophagales	Cyclobacteriaceae	Aquiflexum		CRISPR array			106	35	30
3541092..3541356	-	SMD44428.1	LT838813.1	Aquiflexum balticum DSM 16537	Bacteria	Bacteroidetes	Cytophagia	Cytophagales	Cyclobacteriaceae	Aquiflexum	CRISPR-associated protein Cas2	cas2	COG1343	CAS-I,CAS-II,CAS-III			
3541410..3542433	-	SMD44429.1	LT838813.1	Aquiflexum balticum DSM 16537	Bacteria	Bacteroidetes	Cytophagia	Cytophagales	Cyclobacteriaceae	Aquiflexum	CRISP-associated protein Cas1	cas1	cd09722	CAS-I-B			
3542389..3542911	-	SMD44430.1	LT838813.1	Aquiflexum balticum DSM 16537	Bacteria	Bacteroidetes	Cytophagia	Cytophagales	Cyclobacteriaceae	Aquiflexum	CRISPR-associated exonuclease Cas4	cas4	pfam01930	CAS-I			
3542963..3544049	-	SMD44431.1	LT838813.1	Aquiflexum balticum DSM 16537	Bacteria	Bacteroidetes	Cytophagia	Cytophagales	Cyclobacteriaceae	Aquiflexum	hypothetical protein	cas7	mkCas0088	CAS-I-B			
3544051..3545395	-	SMD44432.1	LT838813.1	Aquiflexum balticum DSM 16537	Bacteria	Bacteroidetes	Cytophagia	Cytophagales	Cyclobacteriaceae	Aquiflexum	hypothetical protein	cas8b6	mkCas0111	CAS-I-B			
3545391..3547542	-	SMD44433.1	LT838813.1	Aquiflexum balticum DSM 16537	Bacteria	Bacteroidetes	Cytophagia	Cytophagales	Cyclobacteriaceae	Aquiflexum	CRISPR-associated endonuclease/helicase Cas3	cas3HD,cas3	cd09641,COG1203	CAS-I			
3547545..3548127	-	SMD44434.1	LT838813.1	Aquiflexum balticum DSM 16537	Bacteria	Bacteroidetes	Cytophagia	Cytophagales	Cyclobacteriaceae	Aquiflexum	hypothetical protein	cas5	mkCas0069	CAS-I-B			
3548135..3548792	-	SMD44435.1	LT838813.1	Aquiflexum balticum DSM 16537	Bacteria	Bacteroidetes	Cytophagia	Cytophagales	Cyclobacteriaceae	Aquiflexum	CRISPR-associated endoribonuclease Cas6	cas6	cd09652	CAS-I,CAS-III			
3548879..3549665	-	SMD44436.1	LT838813.1	Aquiflexum balticum DSM 16537	Bacteria	Bacteroidetes	Cytophagia	Cytophagales	Cyclobacteriaceae	Aquiflexum	CRISPR-associated endoribonuclease Cas6	cas6	cd09759	CAS-I-A			
425 CAS-I-B | PSL07189.1																	
132247..133042	+	PSL07181.1	PYGF01000001.1	Cecembia rubra	Bacteria	Bacteroidetes	Cytophagia	Cytophagales	Cyclobacteriaceae	Cecembia	CRISPR-associated Cas6 family protein	cas6	cd09759	CAS-I-A			
133038..133239	+	PSL07182.1	PYGF01000001.1	Cecembia rubra	Bacteria	Bacteroidetes	Cytophagia	Cytophagales	Cyclobacteriaceae	Cecembia	hypothetical protein						
133250..135080	+	PSL07183.1	PYGF01000001.1	Cecembia rubra	Bacteria	Bacteroidetes	Cytophagia	Cytophagales	Cyclobacteriaceae	Cecembia	CRISPR-associated protein Csh1	cas8b1	mkCas0106	CAS-I-B			
135092..136034	+	PSL07184.1	PYGF01000001.1	Cecembia rubra	Bacteria	Bacteroidetes	Cytophagia	Cytophagales	Cyclobacteriaceae	Cecembia	CRISPR-associated protein Csh2	cas7b	pfam05107	CAS-I-B,CAS-I-C			

136036..136819	+	PSL07185.1	PYGF01000001.1	Cecembia rubra	Bacteria	Bacteroidetes	Cytophagia	Cytophagales	Cyclobacteriaceae	Cecembia	CRISPR-associated protein Cas5h	cas5	cls001430	CAS-I-B			
136820..139484	+	PSL07186.1	PYGF01000001.1	Cecembia rubra	Bacteria	Bacteroidetes	Cytophagia	Cytophagales	Cyclobacteriaceae	Cecembia	CRISPR-associated Cas3 family helicase	cas3HD,cas3	cd09641,cd09639	CAS-I			
139532..140036	+	PSL07187.1	PYGF01000001.1	Cecembia rubra	Bacteria	Bacteroidetes	Cytophagia	Cytophagales	Cyclobacteriaceae	Cecembia	CRISPR-associated Cas4 family exonuclease	cas4	pfam01930	CAS-I			
140118..141123	+	PSL07188.1	PYGF01000001.1	Cecembia rubra	Bacteria	Bacteroidetes	Cytophagia	Cytophagales	Cyclobacteriaceae	Cecembia	CRISPR-associated Cas1 family protein	cas1	cd09722	CAS-I-B			
141176..141440	+	PSL07189.1	PYGF01000001.1	Cecembia rubra	Bacteria	Bacteroidetes	Cytophagia	Cytophagales	Cyclobacteriaceae	Cecembia	CRISPR-associated Cas2 family protein	cas2	COG1343	CAS-I,CAS-II,CAS-III			
141653..148190	array		PYGF01000001.1	Cecembia rubra	Bacteria	Bacteroidetes	Cytophagia	Cytophagales	Cyclobacteriaceae	Cecembia		CRISPR array			100	35	30
426 CAS-I-B | AJC73847.1																	
1224427..1225246	+	AJC73840.1	CP007141.1	Pseudothermotoga hypogea DSM 11164 = NBRC 106472	Bacteria	Thermotogae	Thermotogae	Thermotogales	Thermotogaceae	Pseudothermotoga	CRISPR-associated protein Cas6	cas6	cd09652	CAS-I,CAS-III			
1225247..1226399	+	AJC73841.1	CP007141.1	Pseudothermotoga hypogea DSM 11164 = NBRC 106472	Bacteria	Thermotogae	Thermotogae	Thermotogales	Thermotogaceae	Pseudothermotoga	hypothetical protein	cas8b2	cd09665	CAS-I-B			
1226395..1226950	+	AJC73842.1	CP007141.1	Pseudothermotoga hypogea DSM 11164 = NBRC 106472	Bacteria	Thermotogae	Thermotogae	Thermotogales	Thermotogaceae	Pseudothermotoga	hypothetical protein	Cas11b	icity0129				
1226933..1228088	+	AJC73843.1	CP007141.1	Pseudothermotoga hypogea DSM 11164 = NBRC 106472	Bacteria	Thermotogae	Thermotogae	Thermotogales	Thermotogaceae	Pseudothermotoga	CRISPR-associated protein DevR	cas7,cas7	pfam01905,pfam01905	CAS-I			
1228910..1231415	+	AJC73844.1	CP007141.1	Pseudothermotoga hypogea DSM 11164 = NBRC 106472	Bacteria	Thermotogae	Thermotogae	Thermotogales	Thermotogaceae	Pseudothermotoga	CRISPR-associated protein Cas3	cas3HD,cas3	cd09641,COG1203	CAS-I			
1231434..1231947	+	AJC73845.1	CP007141.1	Pseudothermotoga hypogea DSM 11164 = NBRC 106472	Bacteria	Thermotogae	Thermotogae	Thermotogales	Thermotogaceae	Pseudothermotoga	CRISPR-associated protein Cas4	cas4	pfam01930	CAS-I			
1231959..1232937	+	AJC73846.1	CP007141.1	Pseudothermotoga hypogea DSM 11164 = NBRC 106472	Bacteria	Thermotogae	Thermotogae	Thermotogales	Thermotogaceae	Pseudothermotoga	CRISPR-associated protein Cas1	cas1	cd09722	CAS-I-B			
1232936..1233203	+	AJC73847.1	CP007141.1	Pseudothermotoga hypogea DSM 11164 = NBRC 106472	Bacteria	Thermotogae	Thermotogae	Thermotogales	Thermotogaceae	Pseudothermotoga	CRISPR-associated protein Cas2	cas2	cd09725	CAS-I,CAS-II,CAS-III,CAS-V			
1233417..1235361	array		CP007141.1	Pseudothermotoga hypogea DSM 11164 = NBRC 106472	Bacteria	Thermotogae	Thermotogae	Thermotogales	Thermotogaceae	Pseudothermotoga		CRISPR array			30	37	29
427 CAS-I-B | ADR19823.1																	
1980414..1981209	+	ADR19816.1	CP002347.1	Calditerrivibrio nitroreducens DSM 19672	Bacteria	Deferribacteres	Deferribacteres	Deferribacterales	Deferribacteraceae	Calditerrivibrio	CRISPR-associated protein Cas6	cas6	cd09652	CAS-I,CAS-III			
1981201..1982665	+	ADR19817.1	CP002347.1	Calditerrivibrio nitroreducens DSM 19672	Bacteria	Deferribacteres	Deferribacteres	Deferribacterales	Deferribacteraceae	Calditerrivibrio	hypothetical protein						
1982678..1984094	+	ADR19818.1	CP002347.1	Calditerrivibrio nitroreducens DSM 19672	Bacteria	Deferribacteres	Deferribacteres	Deferribacterales	Deferribacteraceae	Calditerrivibrio	CRISPR-associated autoregulator DevR family	cas7	pfam01905	CAS-I			
1984115..1984790	+	ADR19819.1	CP002347.1	Calditerrivibrio nitroreducens DSM 19672	Bacteria	Deferribacteres	Deferribacteres	Deferribacterales	Deferribacteraceae	Calditerrivibrio	CRISPR-associated protein Cas5	cas5	cd09658	CAS-I-B			
1984773..1987080	+	ADR19820.1	CP002347.1	Calditerrivibrio nitroreducens DSM 19672	Bacteria	Deferribacteres	Deferribacteres	Deferribacterales	Deferribacteraceae	Calditerrivibrio	CRISPR-associated helicase Cas3	cas3HD,cas3	cd09641,cd09639	CAS-I			
1987495..1988602	+	ADR19821.1	CP002347.1	Calditerrivibrio nitroreducens DSM 19672	Bacteria	Deferribacteres	Deferribacteres	Deferribacterales	Deferribacteraceae	Calditerrivibrio	transposase mutator type						
1988928..1989909	+	ADR19822.1	CP002347.1	Calditerrivibrio nitroreducens DSM 19672	Bacteria	Deferribacteres	Deferribacteres	Deferribacterales	Deferribacteraceae	Calditerrivibrio	CRISPR-associated protein, Cas1 family	cas1	cd09722	CAS-I-B			
1989924..1990188	+	ADR19823.1	CP002347.1	Calditerrivibrio nitroreducens DSM 19672	Bacteria	Deferribacteres	Deferribacteres	Deferribacterales	Deferribacteraceae	Calditerrivibrio	CRISPR-associated protein, Cas2 family	cas2	cd09725	CAS-I,CAS-II,CAS-III,CAS-V			
1990498..1990853	array		CP002347.1	Calditerrivibrio nitroreducens DSM 19672	Bacteria	Deferribacteres	Deferribacteres	Deferribacterales	Deferribacteraceae	Calditerrivibrio		CRISPR array			6	36	29
428 CAS-I-B | ACI21703.1																	
1937714..1937981	-	ACI21703.1	CP001147.1	Thermodesulfovibrio yellowstonii DSM 11347	Bacteria	Nitrospirae	Nitrospira	Nitrospirales	Nitrospiraceae	Thermodesulfovibrio	CRISPR-associated protein Cas2	cas2	cd09725	CAS-I,CAS-II,CAS-III,CAS-V			
1937998..1938139	-	ACI20706.1	CP001147.1	Thermodesulfovibrio yellowstonii DSM 11347	Bacteria	Nitrospirae	Nitrospira	Nitrospirales	Nitrospiraceae	Thermodesulfovibrio	hypothetical protein						
1938113..1939091	-	ACI20555.1	CP001147.1	Thermodesulfovibrio yellowstonii DSM 11347	Bacteria	Nitrospirae	Nitrospira	Nitrospirales	Nitrospiraceae	Thermodesulfovibrio	CRISPR-associated protein Cas1, HMARI/TNEAP subtype	cas1	cd09722	CAS-I-B			
1939094..1939616	-	ACI21023.1	CP001147.1	Thermodesulfovibrio yellowstonii DSM 11347	Bacteria	Nitrospirae	Nitrospira	Nitrospirales	Nitrospiraceae	Thermodesulfovibrio	CRISPR-associated protein Cas4	cas4	pfam01930	CAS-I			
1939622..1940834	-	ACI21876.1	CP001147.1	Thermodesulfovibrio yellowstonii DSM 11347	Bacteria	Nitrospirae	Nitrospira	Nitrospirales	Nitrospiraceae	Thermodesulfovibrio	ATPase						
1940908..1943095	-	ACI20256.1	CP001147.1	Thermodesulfovibrio yellowstonii DSM 11347	Bacteria	Nitrospirae	Nitrospira	Nitrospirales	Nitrospiraceae	Thermodesulfovibrio	CRISPR-associated helicase Cas3	cas3HD,cas3	cd09641,cd09639	CAS-I			
1943097..1943817	-	ACI22198.1	CP001147.1	Thermodesulfovibrio yellowstonii DSM 11347	Bacteria	Nitrospirae	Nitrospira	Nitrospirales	Nitrospiraceae	Thermodesulfovibrio	CRISPR-associated protein Cas5	cas5	cd09658	CAS-I-B			
1943813..1944713	-	ACI20183.1	CP001147.1	Thermodesulfovibrio yellowstonii DSM 11347	Bacteria	Nitrospirae	Nitrospira	Nitrospirales	Nitrospiraceae	Thermodesulfovibrio	CRISPR-associated regulatory protein, DevR family	cas7	cd09687	CAS-I-B			
1944715..1946380	-	ACI21434.1	CP001147.1	Thermodesulfovibrio yellowstonii DSM 11347	Bacteria	Nitrospirae	Nitrospira	Nitrospirales	Nitrospiraceae	Thermodesulfovibrio	CRISPR-associated CXXC_CXXC protein Cst1	cas8b2	cd09665	CAS-I-B			
1946536..1947325	-	ACI21552.1	CP001147.1	Thermodesulfovibrio yellowstonii DSM 11347	Bacteria	Nitrospirae	Nitrospira	Nitrospirales	Nitrospiraceae	Thermodesulfovibrio	CRISPR-associated protein Cas6	cas6	cd09652	CAS-I,CAS-III			
430 CAS-I-B | ACI19344.1																	
71018..73143	array		CP001146.1	Dictyoglomus thermophilum H-6-12	Bacteria	Dictyoglomi	Dictyoglomia	Dictyoglomales	Dictyoglomaceae	Dictyoglomus		CRISPR array			33	36	29
73359..73623	-	ACI19344.1	CP001146.1	Dictyoglomus thermophilum H-6-12	Bacteria	Dictyoglomi	Dictyoglomia	Dictyoglomales	Dictyoglomaceae	Dictyoglomus	CRISPR-associated protein Cas2	cas2	cd09725	CAS-I,CAS-II,CAS-III,CAS-V			
73625..74606	-	ACI18526.1	CP001146.1	Dictyoglomus thermophilum H-6-12	Bacteria	Dictyoglomi	Dictyoglomia	Dictyoglomales	Dictyoglomaceae	Dictyoglomus	CRISPR-associated protein Cas1, HMARI/TNEAP subtype	cas1	cd09722	CAS-I-B			
74610..75123	-	ACI20136.1	CP001146.1	Dictyoglomus thermophilum H-6-12	Bacteria	Dictyoglomi	Dictyoglomia	Dictyoglomales	Dictyoglomaceae	Dictyoglomus	CRISPR-associated protein Cas4	cas4	pfam01930	CAS-I			
75143..77411	-	ACI19282.1	CP001146.1	Dictyoglomus thermophilum H-6-12	Bacteria	Dictyoglomi	Dictyoglomia	Dictyoglomales	Dictyoglomaceae	Dictyoglomus	CRISPR-associated helicase Cas3	cas3HD,cas3	cd09641,COG1203	CAS-I			
77388..78090	-	ACI19854.1	CP001146.1	Dictyoglomus thermophilum H-6-12	Bacteria	Dictyoglomi	Dictyoglomia	Dictyoglomales	Dictyoglomaceae	Dictyoglomus	CRISPR-associated protein Cas5, Tneap subtype	cas5	cd09658	CAS-I-B			
78076..78976	-	ACI19005.1	CP001146.1	Dictyoglomus thermophilum H-6-12	Bacteria	Dictyoglomi	Dictyoglomia	Dictyoglomales	Dictyoglomaceae	Dictyoglomus	CRISPR-associated regulatory protein, DevR family	cas7	cd09687	CAS-I-B			
78991..80668	-	ACI19672.1	CP001146.1	Dictyoglomus thermophilum H-6-12	Bacteria	Dictyoglomi	Dictyoglomia	Dictyoglomales	Dictyoglomaceae	Dictyoglomus	CRISPR-associated CXXC_CXXC protein Cst1	cas8b2	cd09665	CAS-I-B			
80669..81473	-	ACI19864.1	CP001146.1	Dictyoglomus thermophilum H-6-12	Bacteria	Dictyoglomi	Dictyoglomia	Dictyoglomales	Dictyoglomaceae	Dictyoglomus	CRISPR-associated protein Cas6	cas6	cd09652	CAS-I,CAS-III			
431 CAS-I-B | PMQ01379.1																	
15451..16180	+	PMQ01372.1	NIRF01000007.1	Dictyoglomus sp. NZ13-RE01	Bacteria	Dictyoglomi	Dictyoglomia	Dictyoglomales	Dictyoglomaceae	Dictyoglomus	CRISPR-associated endoribonuclease Cas6	cas6	cd09652	CAS-I,CAS-III			
16182..17826	+	PMQ01373.1	NIRF01000007.1	Dictyoglomus sp. NZ13-RE01	Bacteria	Dictyoglomi	Dictyoglomia	Dictyoglomales	Dictyoglomaceae	Dictyoglomus	type I-B CRISPR-associated protein Cas8b1/Cst1	cas8b2	cd09665	CAS-I-B			
17846..18740	+	PMQ01374.1	NIRF01000007.1	Dictyoglomus sp. NZ13-RE01	Bacteria	Dictyoglomi	Dictyoglomia	Dictyoglomales	Dictyoglomaceae	Dictyoglomus	type I-B CRISPR-associated protein Cas7/Cst2/DevR	cas7	cd09687	CAS-I-B			
18726..19419	+	PMQ01375.1	NIRF01000007.1	Dictyoglomus sp. NZ13-RE01	Bacteria	Dictyoglomi	Dictyoglomia	Dictyoglomales	Dictyoglomaceae	Dictyoglomus	type I-B CRISPR-associated protein Cas5	cas5	cd09658	CAS-I-B			
19411..21679	+	PMQ01376.1	NIRF01000007.1	Dictyoglomus sp. NZ13-RE01	Bacteria	Dictyoglomi	Dictyoglomia	Dictyoglomales	Dictyoglomaceae	Dictyoglomus	CRISPR-associated helicase/endonuclease Cas3	cas3HD,cas3	cd09641,COG1203	CAS-I			
21703..22213	+	PMQ01377.1	NIRF01000007.1	Dictyoglomus sp. NZ13-RE01	Bacteria	Dictyoglomi	Dictyoglomia	Dictyoglomales	Dictyoglomaceae	Dictyoglomus	CRISPR-associated protein Cas4	cas4	pfam01930	CAS-I			
22219..23200	+	PMQ01378.1	NIRF01000007.1	Dictyoglomus sp. NZ13-RE01	Bacteria	Dictyoglomi	Dictyoglomia	Dictyoglomales	Dictyoglomaceae	Dictyoglomus	subtype I-B CRISPR-associated endonuclease Cas1	cas1	cd09722	CAS-I-B			
23202..23466	+	PMQ01379.1	NIRF01000007.1	Dictyoglomus sp. NZ13-RE01	Bacteria	Dictyoglomi	Dictyoglomia	Dictyoglomales	Dictyoglomaceae	Dictyoglomus	CRISPR-associated endonuclease Cas2	cas2	cd09725	CAS-I,CAS-II,CAS-III,CAS-V			
23680..26928	array		NIRF01000007.1	Dictyoglomus sp. NZ13-RE01	Bacteria	Dictyoglomi	Dictyoglomia	Dictyoglomales	Dictyoglomaceae	Dictyoglomus		CRISPR array			50	36	29
27084..28432	array		NIRF01000007.1	Dictyoglomus sp. NZ13-RE01	Bacteria	Dictyoglomi	Dictyoglomia	Dictyoglomales	Dictyoglomaceae	Dictyoglomus		CRISPR array			21	37	29
432 CAS-I-B | ADL41492.1																	
136831..137617	+	ADL41485.1	CP002164.1	Caldicellulosiruptor obsidiansis OB47	Bacteria	Firmicutes	Clostridia	Thermoanaerobacterales	Thermoanaerobacterales Family III. Incertae Sedis	Caldicellulosiruptor	CRISPR-associated protein Cas6	cas6	pfam01881	CAS-I,CAS-III			
137648..139304	+	ADL41486.1	CP002164.1	Caldicellulosiruptor obsidiansis OB47	Bacteria	Firmicutes	Clostridia	Thermoanaerobacterales	Thermoanaerobacterales Family III. Incertae Sedis	Caldicellulosiruptor	CRISPR-associated CXXC_CXXC protein Cst1	cas8b2	cd09665	CAS-I-B			
139296..140226	+	ADL41487.1	CP002164.1	Caldicellulosiruptor obsidiansis OB47	Bacteria	Firmicutes	Clostridia	Thermoanaerobacterales	Thermoanaerobacterales Family III. Incertae Sedis	Caldicellulosiruptor	CRISPR-associated regulatory protein, DevR family	cas7	cd09687	CAS-I-B			
140228..140975	+	ADL41488.1	CP002164.1	Caldicellulosiruptor obsidiansis OB47	Bacteria	Firmicutes	Clostridia	Thermoanaerobacterales	Thermoanaerobacterales Family III. Incertae Sedis	Caldicellulosiruptor	CRISPR-associated protein Cas5 family	cas5	cd09658	CAS-I-B			
140964..143298	+	ADL41489.1	CP002164.1	Caldicellulosiruptor obsidiansis OB47	Bacteria	Firmicutes	Clostridia	Thermoanaerobacterales	Thermoanaerobacterales Family III. Incertae Sedis	Caldicellulosiruptor	CRISPR-associated helicase Cas3	cas3HD,cas3	cd09641,cd09639	CAS-I			
143319..143832	+	ADL41490.1	CP002164.1	Caldicellulosiruptor obsidiansis OB47	Bacteria	Firmicutes	Clostridia	Thermoanaerobacterales	Thermoanaerobacterales Family III. Incertae Sedis	Caldicellulosiruptor	CRISPR-associated protein Cas4	cas4	pfam01930	CAS-I			
143836..144817	+	ADL41491.1	CP002164.1	Caldicellulosiruptor obsidiansis OB47	Bacteria	Firmicutes	Clostridia	Thermoanaerobacterales	Thermoanaerobacterales Family III. Incertae Sedis	Caldicellulosiruptor	CRISPR-associated protein Cas1	cas1	cd09722	CAS-I-B			
144841..145105	+	ADL41492.1	CP002164.1	Caldicellulosiruptor obsidiansis OB47	Bacteria	Firmicutes	Clostridia	Thermoanaerobacterales	Thermoanaerobacterales Family III. Incertae Sedis	Caldicellulosiruptor	CRISPR-associated protein Cas2	cas2	cd09725	CAS-I,CAS-II,CAS-III,CAS-V			
145343..157027	array		CP002164.1	Caldicellulosiruptor obsidiansis OB47	Bacteria	Firmicutes	Clostridia	Thermoanaerobacterales	Thermoanaerobacterales Family III. Incertae Sedis	Caldicellulosiruptor		CRISPR array			178	36	29
433 CAS-I-B | ADQ03685.1																	
81873..82659	+	ADQ03677.1	CP002216.1	Caldicellulosiruptor owensensis OL	Bacteria	Firmicutes	Clostridia	Thermoanaerobacterales	Thermoanaerobacterales Family III. Incertae Sedis	Caldicellulosiruptor	CRISPR-associated protein Cas6	cas6	pfam01881	CAS-I,CAS-III			
82663..83779	+	ADQ03678.1	CP002216.1	Caldicellulosiruptor owensensis OL	Bacteria	Firmicutes	Clostridia	Thermoanaerobacterales	Thermoanaerobacterales Family III. Incertae Sedis	Caldicellulosiruptor	hypothetical protein						
83775..84282	+	ADQ03679.1	CP002216.1	Caldicellulosiruptor owensensis OL	Bacteria	Firmicutes	Clostridia	Thermoanaerobacterales	Thermoanaerobacterales Family III. Incertae Sedis	Caldicellulosiruptor	hypothetical protein	Cas11b	icity0129				
84316..85420	+	ADQ03680.1	CP002216.1	Caldicellulosiruptor owensensis OL	Bacteria	Firmicutes	Clostridia	Thermoanaerobacterales	Thermoanaerobacterales Family III. Incertae Sedis	Caldicellulosiruptor	CRISPR-associated regulatory protein, DevR family	cas7	pfam01905	CAS-I			
85468..86290	+	ADQ03681.1	CP002216.1	Caldicellulosiruptor owensensis OL	Bacteria	Firmicutes	Clostridia	Thermoanaerobacterales	Thermoanaerobacterales Family III. Incertae Sedis	Caldicellulosiruptor	CRISPR-associated protein Cas5 family	cas5	cd09658	CAS-I-B			
86282..89027	+	ADQ03682.1	CP002216.1	Caldicellulosiruptor owensensis OL	Bacteria	Firmicutes	Clostridia	Thermoanaerobacterales	Thermoanaerobacterales Family III. Incertae Sedis	Caldicellulosiruptor	CRISPR-associated helicase Cas3	cas3HD,cas3	cd09641,COG1203	CAS-I			
89045..89558	+	ADQ03683.1	CP002216.1	Caldicellulosiruptor owensensis OL	Bacteria	Firmicutes	Clostridia	Thermoanaerobacterales	Thermoanaerobacterales Family III. Incertae Sedis	Caldicellulosiruptor	CRISPR-associated protein Cas4	cas4	pfam01930	CAS-I			
89562..90543	+	ADQ03684.1	CP002216.1	Caldicellulosiruptor owensensis OL	Bacteria	Firmicutes	Clostridia	Thermoanaerobacterales	Thermoanaerobacterales Family III. Incertae Sedis	Caldicellulosiruptor	CRISPR-associated protein Cas1	cas1	cd09722	CAS-I-B			
90567..90831	+	ADQ03685.1	CP002216.1	Caldicellulosiruptor owensensis OL	Bacteria	Firmicutes	Clostridia	Thermoanaerobacterales	Thermoanaerobacterales Family III. Incertae Sedis	Caldicellulosiruptor	CRISPR-associated protein Cas2	cas2	cd09725	CAS-I,CAS-II,CAS-III,CAS-V			
91068..95458	array		CP002216.1	Caldicellulosiruptor owensensis OL	Bacteria	Firmicutes	Clostridia	Thermoanaerobacterales	Thermoanaerobacterales Family III. Incertae Sedis	Caldicellulosiruptor		CRISPR array			67	37	29
434 CAS-I-B | ADQ41852.1																	
2496983..2506688	array		CP002326.1	Caldicellulosiruptor kristjanssonii I77R1B	Bacteria	Firmicutes	Clostridia	Thermoanaerobacterales	Thermoanaerobacterales Family III. Incertae Sedis	Caldicellulosiruptor		CRISPR array			148	37	28
2506925..2507189	-	ADQ41852.1	CP002326.1	Caldicellulosiruptor kristjanssonii I77R1B	Bacteria	Firmicutes	Clostridia	Thermoanaerobacterales	Thermoanaerobacterales Family III. Incertae Sedis	Caldicellulosiruptor	CRISPR-associated protein Cas2	cas2	cd09725	CAS-I,CAS-II,CAS-III,CAS-V			
2507281..2508262	-	ADQ41853.1	CP002326.1	Caldicellulosiruptor kristjanssonii I77R1B	Bacteria	Firmicutes	Clostridia	Thermoanaerobacterales	Thermoanaerobacterales Family III. Incertae Sedis	Caldicellulosiruptor	CRISPR-associated protein Cas1	cas1	cd09722	CAS-I-B			
2508268..2508781	-	ADQ41854.1	CP002326.1	Caldicellulosiruptor kristjanssonii I77R1B	Bacteria	Firmicutes	Clostridia	Thermoanaerobacterales	Thermoanaerobacterales Family III. Incertae Sedis	Caldicellulosiruptor	CRISPR-associated protein Cas4	cas4	pfam01930	CAS-I			
2508800..2511134	-	ADQ41855.1	CP002326.1	Caldicellulosiruptor kristjanssonii I77R1B	Bacteria	Firmicutes	Clostridia	Thermoanaerobacterales	Thermoanaerobacterales Family III. Incertae Sedis	Caldicellulosiruptor	CRISPR-associated helicase Cas3	cas3HD,cas3	cd09641,cd09639	CAS-I			
2511123..2511870	-	ADQ41856.1	CP002326.1	Caldicellulosiruptor kristjanssonii I77R1B	Bacteria	Firmicutes	Clostridia	Thermoanaerobacterales	Thermoanaerobacterales Family III. Incertae Sedis	Caldicellulosiruptor	CRISPR-associated protein Cas5 family	cas5	cd09658	CAS-I-B			
2511872..2512802	-	ADQ41857.1	CP002326.1	Caldicellulosiruptor kristjanssonii I77R1B	Bacteria	Firmicutes	Clostridia	Thermoanaerobacterales	Thermoanaerobacterales Family III. Incertae Sedis	Caldicellulosiruptor	CRISPR-associated autoregulator, DevR family	cas7	cd09687	CAS-I-B			
2512794..2514207	-	ADQ41858.1	CP002326.1	Caldicellulosiruptor kristjanssonii I77R1B	Bacteria	Firmicutes	Clostridia	Thermoanaerobacterales	Thermoanaerobacterales Family III. Incertae Sedis	Caldicellulosiruptor	CRISPR-associated CXXC_CXXC protein Cst1	cas8b2	cd09665	CAS-I-B			
2514380..2515598	-	ADQ41859.1	CP002326.1	Caldicellulosiruptor kristjanssonii I77R1B	Bacteria	Firmicutes	Clostridia	Thermoanaerobacterales	Thermoanaerobacterales Family III. Incertae Sedis	Caldicellulosiruptor	transposase IS116/IS110/IS902 family protein						
2515662..2516037	-	ADQ41860.1	CP002326.1	Caldicellulosiruptor kristjanssonii I77R1B	Bacteria	Firmicutes	Clostridia	Thermoanaerobacterales	Thermoanaerobacterales Family III. Incertae Sedis	Caldicellulosiruptor	hypothetical protein						
2516065..2516851	-	ADQ41861.1	CP002326.1	Caldicellulosiruptor kristjanssonii I77R1B	Bacteria	Firmicutes	Clostridia	Thermoanaerobacterales	Thermoanaerobacterales Family III. Incertae Sedis	Caldicellulosiruptor	CRISPR-associated protein Cas6	cas6	pfam01881	CAS-I,CAS-III			
435 CAS-I-B | ACM59286.1																	
142060..142768	+	ACM59278.1	CP001393.1	Caldicellulosiruptor bescii DSM 6725	Bacteria	Firmicutes	Clostridia	Thermoanaerobacterales	Thermoanaerobacterales Family III. Incertae Sedis	Caldicellulosiruptor	CRISPR-associated Cas family protein	cas6	pfam01881	CAS-I,CAS-III			
142982..143768	+	ACM59279.1	CP001393.1	Caldicellulosiruptor bescii DSM 6725	Bacteria	Firmicutes	Clostridia	Thermoanaerobacterales	Thermoanaerobacterales Family III. Incertae Sedis	Caldicellulosiruptor	CRISPR-associated protein Cas6	cas6	pfam01881	CAS-I,CAS-III			
143800..145441	+	ACM59280.1	CP001393.1	Caldicellulosiruptor bescii DSM 6725	Bacteria	Firmicutes	Clostridia	Thermoanaerobacterales	Thermoanaerobacterales Family III. Incertae Sedis	Caldicellulosiruptor	CRISPR-associated CXXC_CXXC protein Cst1	cas8b2	cd09665	CAS-I-B			
145437..146367	+	ACM59281.1	CP001393.1	Caldicellulosiruptor bescii DSM 6725	Bacteria	Firmicutes	Clostridia	Thermoanaerobacterales	Thermoanaerobacterales Family III. Incertae Sedis	Caldicellulosiruptor	CRISPR-associated autoregulator, DevR family	cas7	cd09687	CAS-I-B			
146369..147119	+	ACM59282.1	CP001393.1	Caldicellulosiruptor bescii DSM 6725	Bacteria	Firmicutes	Clostridia	Thermoanaerobacterales	Thermoanaerobacterales Family III. Incertae Sedis	Caldicellulosiruptor	CRISPR-associated protein Cas5 family	cas5	cd09658	CAS-I-B			
147105..149433	+	ACM59283.1	CP001393.1	Caldicellulosiruptor bescii DSM 6725	Bacteria	Firmicutes	Clostridia	Thermoanaerobacterales	Thermoanaerobacterales Family III. Incertae Sedis	Caldicellulosiruptor	CRISPR-associated helicase Cas3	cas3HD,cas3	cd09641,COG1203	CAS-I			
149462..149978	+	ACM59284.1	CP001393.1	Caldicellulosiruptor bescii DSM 6725	Bacteria	Firmicutes	Clostridia	Thermoanaerobacterales	Thermoanaerobacterales Family III. Incertae Sedis	Caldicellulosiruptor	CRISPR-associated protein Cas4	cas4	pfam01930	CAS-I			
149983..150964	+	ACM59285.1	CP001393.1	Caldicellulosiruptor bescii DSM 6725	Bacteria	Firmicutes	Clostridia	Thermoanaerobacterales	Thermoanaerobacterales Family III. Incertae Sedis	Caldicellulosiruptor	CRISPR-associated protein Cas1	cas1	cd09722	CAS-I-B			
151152..151416	+	ACM59286.1	CP001393.1	Caldicellulosiruptor bescii DSM 6725	Bacteria	Firmicutes	Clostridia	Thermoanaerobacterales	Thermoanaerobacterales Family III. Incertae Sedis	Caldicellulosiruptor	CRISPR-associated protein Cas2	cas2	cd09725	CAS-I,CAS-II,CAS-III,CAS-V			
151654..160400	array		CP001393.1	Caldicellulosiruptor bescii DSM 6725	Bacteria	Firmicutes	Clostridia	Thermoanaerobacterales	Thermoanaerobacterales Family III. Incertae Sedis	Caldicellulosiruptor		CRISPR array			134	36	29
162207..163410	+	ACM59287.1	CP001393.1	Caldicellulosiruptor bescii DSM 6725	Bacteria	Firmicutes	Clostridia	Thermoanaerobacterales	Thermoanaerobacterales Family III. Incertae Sedis	Caldicellulosiruptor	Integrase catalytic region						
163430..164195	+	ACM59288.1	CP001393.1	Caldicellulosiruptor bescii DSM 6725	Bacteria	Firmicutes	Clostridia	Thermoanaerobacterales	Thermoanaerobacterales Family III. Incertae Sedis	Caldicellulosiruptor	IstB domain protein ATP-binding protein						
164244..165518	array		CP001393.1	Caldicellulosiruptor bescii DSM 6725	Bacteria	Firmicutes	Clostridia	Thermoanaerobacterales	Thermoanaerobacterales Family III. Incertae Sedis	Caldicellulosiruptor		CRISPR array			20	36	29
436 CAS-I-B | ADQ08182.1																	
2613496..2618702	array		CP002219.1	Caldicellulosiruptor hydrothermalis 108	Bacteria	Firmicutes	Clostridia	Thermoanaerobacterales	Thermoanaerobacterales Family III. Incertae Sedis	Caldicellulosiruptor		CRISPR array			80	36	29
2618906..2619170	-	ADQ08182.1	CP002219.1	Caldicellulosiruptor hydrothermalis 108	Bacteria	Firmicutes	Clostridia	Thermoanaerobacterales	Thermoanaerobacterales Family III. Incertae Sedis	Caldicellulosiruptor	CRISPR-associated protein Cas2	cas2	cd09725	CAS-I,CAS-II,CAS-III,CAS-V			
2619340..2620321	-	ADQ08183.1	CP002219.1	Caldicellulosiruptor hydrothermalis 108	Bacteria	Firmicutes	Clostridia	Thermoanaerobacterales	Thermoanaerobacterales Family III. Incertae Sedis	Caldicellulosiruptor	CRISPR-associated protein Cas1	cas1	cd09722	CAS-I-B			
2620328..2620841	-	ADQ08184.1	CP002219.1	Caldicellulosiruptor hydrothermalis 108	Bacteria	Firmicutes	Clostridia	Thermoanaerobacterales	Thermoanaerobacterales Family III. Incertae Sedis	Caldicellulosiruptor	CRISPR-associated protein Cas4	cas4	pfam01930	CAS-I			
2620860..2623194	-	ADQ08185.1	CP002219.1	Caldicellulosiruptor hydrothermalis 108	Bacteria	Firmicutes	Clostridia	Thermoanaerobacterales	Thermoanaerobacterales Family III. Incertae Sedis	Caldicellulosiruptor	CRISPR-associated helicase Cas3	cas3HD,cas3	cd09641,cd09639	CAS-I			
2623183..2623930	-	ADQ08186.1	CP002219.1	Caldicellulosiruptor hydrothermalis 108	Bacteria	Firmicutes	Clostridia	Thermoanaerobacterales	Thermoanaerobacterales Family III. Incertae Sedis	Caldicellulosiruptor	CRISPR-associated protein Cas5 family	cas5	cd09658	CAS-I-B			
2623932..2624862	-	ADQ08187.1	CP002219.1	Caldicellulosiruptor hydrothermalis 108	Bacteria	Firmicutes	Clostridia	Thermoanaerobacterales	Thermoanaerobacterales Family III. Incertae Sedis	Caldicellulosiruptor	CRISPR-associated regulatory protein, DevR family	cas7	cd09687	CAS-I-B			
2624854..2626765	-	ADQ08188.1	CP002219.1	Caldicellulosiruptor hydrothermalis 108	Bacteria	Firmicutes	Clostridia	Thermoanaerobacterales	Thermoanaerobacterales Family III. Incertae Sedis	Caldicellulosiruptor	CRISPR-associated CXXC_CXXC protein Cst1	cas8b2	cd09665	CAS-I-B			
2626793..2627579	-	ADQ08189.1	CP002219.1	Caldicellulosiruptor hydrothermalis 108	Bacteria	Firmicutes	Clostridia	Thermoanaerobacterales	Thermoanaerobacterales Family III. Incertae Sedis	Caldicellulosiruptor	CRISPR-associated protein Cas6	cas6	pfam01881	CAS-I,CAS-III			
437 CAS-I-B | KHO63178.1																	
1272206..1273814	array		JOOI01000001.1	Thermoanaerobacter sp. YS13	Bacteria	Firmicutes	Clostridia	Thermoanaerobacterales	Thermoanaerobacteraceae	Thermoanaerobacter		CRISPR array			25	36	29
1274021..1274285	-	KHO63178.1	JOOI01000001.1	Thermoanaerobacter sp. YS13	Bacteria	Firmicutes	Clostridia	Thermoanaerobacterales	Thermoanaerobacteraceae	Thermoanaerobacter	CRISPR-associated protein, Cas2 family	cas2	cd09725	CAS-I,CAS-II,CAS-III,CAS-V			
1274475..1275456	-	KHO63179.1	JOOI01000001.1	Thermoanaerobacter sp. YS13	Bacteria	Firmicutes	Clostridia	Thermoanaerobacterales	Thermoanaerobacteraceae	Thermoanaerobacter	CRISPR-associated protein Cas1	cas1	cd09722	CAS-I-B			
1275461..1275974	-	KHO63180.1	JOOI01000001.1	Thermoanaerobacter sp. YS13	Bacteria	Firmicutes	Clostridia	Thermoanaerobacterales	Thermoanaerobacteraceae	Thermoanaerobacter	CRISPR-associated protein Cas4	cas4	pfam01930	CAS-I			
1275994..1278328	-	KHO63181.1	JOOI01000001.1	Thermoanaerobacter sp. YS13	Bacteria	Firmicutes	Clostridia	Thermoanaerobacterales	Thermoanaerobacteraceae	Thermoanaerobacter	CRISPR-associated protein Cas3	cas3	cd09639	CAS-I			
1278317..1279064	-	KHO63182.1	JOOI01000001.1	Thermoanaerobacter sp. YS13	Bacteria	Firmicutes	Clostridia	Thermoanaerobacterales	Thermoanaerobacteraceae	Thermoanaerobacter	CRISPR-associated protein Cas5	cas5	cd09658	CAS-I-B			
1279066..1279996	-	KHO63183.1	JOOI01000001.1	Thermoanaerobacter sp. YS13	Bacteria	Firmicutes	Clostridia	Thermoanaerobacterales	Thermoanaerobacteraceae	Thermoanaerobacter	CRISPR-associated protein	cas7	cd09687	CAS-I-B			
1279988..1280312	-	KHO63184.1	JOOI01000001.1	Thermoanaerobacter sp. YS13	Bacteria	Firmicutes	Clostridia	Thermoanaerobacterales	Thermoanaerobacteraceae	Thermoanaerobacter	CRISPR-associated protein Cas8a1/Cst1, subtype I-B/TNEAP	cas8b2	cd09665	CAS-I-B			
1280505..1281783	+	KHO63185.1	JOOI01000001.1	Thermoanaerobacter sp. YS13	Bacteria	Firmicutes	Clostridia	Thermoanaerobacterales	Thermoanaerobacteraceae	Thermoanaerobacter	transposase						
1281984..1283286	-	KHO63186.1	JOOI01000001.1	Thermoanaerobacter sp. YS13	Bacteria	Firmicutes	Clostridia	Thermoanaerobacterales	Thermoanaerobacteraceae	Thermoanaerobacter	CRISPR-associated protein Cst1	cas8b2	cd09665	CAS-I-B			
1283314..1284100	-	KHO63187.1	JOOI01000001.1	Thermoanaerobacter sp. YS13	Bacteria	Firmicutes	Clostridia	Thermoanaerobacterales	Thermoanaerobacteraceae	Thermoanaerobacter	CRISPR-associated protein Cas6	cas6	pfam01881	CAS-I,CAS-III			
438 CAS-I-B | ADH59808.1																	
21980..25625	array		CP002032.1	Thermoanaerobacter mathranii subsp. mathranii str. A3	Bacteria	Firmicutes	Clostridia	Thermoanaerobacterales	Thermoanaerobacteraceae	Thermoanaerobacter		CRISPR array			56	36	29
25832..26102	-	ADH59808.1	CP002032.1	Thermoanaerobacter mathranii subsp. mathranii str. A3	Bacteria	Firmicutes	Clostridia	Thermoanaerobacterales	Thermoanaerobacteraceae	Thermoanaerobacter	CRISPR-associated protein Cas2	cas2	cd09725	CAS-I,CAS-II,CAS-III,CAS-V			
26286..27267	-	ADH59809.1	CP002032.1	Thermoanaerobacter mathranii subsp. mathranii str. A3	Bacteria	Firmicutes	Clostridia	Thermoanaerobacterales	Thermoanaerobacteraceae	Thermoanaerobacter	CRISPR-associated protein Cas1	cas1	cd09722	CAS-I-B			
27272..27785	-	ADH59810.1	CP002032.1	Thermoanaerobacter mathranii subsp. mathranii str. A3	Bacteria	Firmicutes	Clostridia	Thermoanaerobacterales	Thermoanaerobacteraceae	Thermoanaerobacter	CRISPR-associated protein Cas4	cas4	pfam01930	CAS-I			
27804..30138	-	ADH59811.1	CP002032.1	Thermoanaerobacter mathranii subsp. mathranii str. A3	Bacteria	Firmicutes	Clostridia	Thermoanaerobacterales	Thermoanaerobacteraceae	Thermoanaerobacter	metal dependent phosphohydrolase	cas3HD,cas3	cd09641,cd09639	CAS-I			
30127..30874	-	ADH59812.1	CP002032.1	Thermoanaerobacter mathranii subsp. mathranii str. A3	Bacteria	Firmicutes	Clostridia	Thermoanaerobacterales	Thermoanaerobacteraceae	Thermoanaerobacter	CRISPR-associated protein Cas5 family	cas5	cd09658	CAS-I-B			
30876..31806	-	ADH59813.1	CP002032.1	Thermoanaerobacter mathranii subsp. mathranii str. A3	Bacteria	Firmicutes	Clostridia	Thermoanaerobacterales	Thermoanaerobacteraceae	Thermoanaerobacter	CRISPR-associated autoregulator, DevR family	cas7	cd09687	CAS-I-B			
31798..33445	-	ADH59814.1	CP002032.1	Thermoanaerobacter mathranii subsp. mathranii str. A3	Bacteria	Firmicutes	Clostridia	Thermoanaerobacterales	Thermoanaerobacteraceae	Thermoanaerobacter	CRISPR-associated CXXC_CXXC protein Cst1	cas8b2	cd09665	CAS-I-B			
33473..34259	-	ADH59815.1	CP002032.1	Thermoanaerobacter mathranii subsp. mathranii str. A3	Bacteria	Firmicutes	Clostridia	Thermoanaerobacterales	Thermoanaerobacteraceae	Thermoanaerobacter	CRISPR-associated protein Cas6	cas6	pfam01881	CAS-I,CAS-III			

439 CAS-I-B | ERM91434.1																	
691..3604	array		AXDC01000030.1	Caldanaerobacter subterraneus subsp. yonseiensis KB-1	Bacteria	Firmicutes	Clostridia	Thermoanaerobacterales	Thermoanaerobacteraceae	Caldanaerobacter		CRISPR array			45	36	29
3811..4081	-	ERM91434.1	AXDC01000030.1	Caldanaerobacter subterraneus subsp. yonseiensis KB-1	Bacteria	Firmicutes	Clostridia	Thermoanaerobacterales	Thermoanaerobacteraceae	Caldanaerobacter	CRISPR-associated protein Cas2	cas2	cd09725	CAS-I,CAS-II,CAS-III,CAS-V			
4438..4768	-	ERM91444.1	AXDC01000030.1	Caldanaerobacter subterraneus subsp. yonseiensis KB-1	Bacteria	Firmicutes	Clostridia	Thermoanaerobacterales	Thermoanaerobacteraceae	Caldanaerobacter	transposase						
4870..4978	-	ERM91445.1	AXDC01000030.1	Caldanaerobacter subterraneus subsp. yonseiensis KB-1	Bacteria	Firmicutes	Clostridia	Thermoanaerobacterales	Thermoanaerobacteraceae	Caldanaerobacter	hypothetical protein						
5006..5114	-	ERM91446.1	AXDC01000030.1	Caldanaerobacter subterraneus subsp. yonseiensis KB-1	Bacteria	Firmicutes	Clostridia	Thermoanaerobacterales	Thermoanaerobacteraceae	Caldanaerobacter	hypothetical protein						
5265..6246	-	ERM91435.1	AXDC01000030.1	Caldanaerobacter subterraneus subsp. yonseiensis KB-1	Bacteria	Firmicutes	Clostridia	Thermoanaerobacterales	Thermoanaerobacteraceae	Caldanaerobacter	CRISPR-associated protein Cas1	cas1	cd09722	CAS-I-B			
6251..6764	-	ERM91447.1	AXDC01000030.1	Caldanaerobacter subterraneus subsp. yonseiensis KB-1	Bacteria	Firmicutes	Clostridia	Thermoanaerobacterales	Thermoanaerobacteraceae	Caldanaerobacter	CRISPR-associated protein Cas4	cas4	pfam01930	CAS-I			
6783..9117	-	ERM91448.1	AXDC01000030.1	Caldanaerobacter subterraneus subsp. yonseiensis KB-1	Bacteria	Firmicutes	Clostridia	Thermoanaerobacterales	Thermoanaerobacteraceae	Caldanaerobacter	CRISPR-associated protein Cas3	cas3	cd09639	CAS-I			
9106..9853	-	ERM91449.1	AXDC01000030.1	Caldanaerobacter subterraneus subsp. yonseiensis KB-1	Bacteria	Firmicutes	Clostridia	Thermoanaerobacterales	Thermoanaerobacteraceae	Caldanaerobacter	CRISPR-associated protein Cas5	cas5	cd09658	CAS-I-B			
9855..10785	-	ERM91450.1	AXDC01000030.1	Caldanaerobacter subterraneus subsp. yonseiensis KB-1	Bacteria	Firmicutes	Clostridia	Thermoanaerobacterales	Thermoanaerobacteraceae	Caldanaerobacter	CRISPR-associated protein	cas7	cd09687	CAS-I-B			
10777..12424	-	ERM91451.1	AXDC01000030.1	Caldanaerobacter subterraneus subsp. yonseiensis KB-1	Bacteria	Firmicutes	Clostridia	Thermoanaerobacterales	Thermoanaerobacteraceae	Caldanaerobacter	CRISPR-associated protein Cst1	cas8b2	cd09665	CAS-I-B			
12452..13241	-	ERM91452.1	AXDC01000030.1	Caldanaerobacter subterraneus subsp. yonseiensis KB-1	Bacteria	Firmicutes	Clostridia	Thermoanaerobacterales	Thermoanaerobacteraceae	Caldanaerobacter	CRISPR-associated protein Cas6	cas6	pfam01881	CAS-I,CAS-III			
440 CAS-I-B | AMW33132.1																	
1606807..1609456	array		CP014334.1	Fervidobacterium islandicum	Bacteria	Thermotogae	Thermotogae	Thermotogales	Fervidobacteriaceae	Fervidobacterium		CRISPR array			41	40	25
1609682..1609946	-	AMW33132.1	CP014334.1	Fervidobacterium islandicum	Bacteria	Thermotogae	Thermotogae	Thermotogales	Fervidobacteriaceae	Fervidobacterium	CRISPR-associated protein Cas2	cas2	COG1343	CAS-I,CAS-II,CAS-III			
1609947..1610925	-	AMW33133.1	CP014334.1	Fervidobacterium islandicum	Bacteria	Thermotogae	Thermotogae	Thermotogales	Fervidobacteriaceae	Fervidobacterium	type I-B CRISPR-associated endonuclease Cas1	cas1	cd09722	CAS-I-B			
1610921..1611458	-	AMW33134.1	CP014334.1	Fervidobacterium islandicum	Bacteria	Thermotogae	Thermotogae	Thermotogales	Fervidobacteriaceae	Fervidobacterium	hypothetical protein	cas4	pfam01930	CAS-I			
1611494..1613678	-	AMW33135.1	CP014334.1	Fervidobacterium islandicum	Bacteria	Thermotogae	Thermotogae	Thermotogales	Fervidobacteriaceae	Fervidobacterium	hypothetical protein	cas3HD,cas3	cd09641,cd09639	CAS-I			
1613721..1614408	-	AMW33136.1	CP014334.1	Fervidobacterium islandicum	Bacteria	Thermotogae	Thermotogae	Thermotogales	Fervidobacteriaceae	Fervidobacterium	hypothetical protein	cas5	cd09658	CAS-I-B			
1614414..1615335	-	AMW33137.1	CP014334.1	Fervidobacterium islandicum	Bacteria	Thermotogae	Thermotogae	Thermotogales	Fervidobacteriaceae	Fervidobacterium	type I-B CRISPR-associated protein Cas7/Cst2/DevR	cas7	cd09687	CAS-I-B			
1616511..1617441	+	AMW33138.1	CP014334.1	Fervidobacterium islandicum	Bacteria	Thermotogae	Thermotogae	Thermotogales	Fervidobacteriaceae	Fervidobacterium	transposase						
1617462..1619193	-	AMW33139.1	CP014334.1	Fervidobacterium islandicum	Bacteria	Thermotogae	Thermotogae	Thermotogales	Fervidobacteriaceae	Fervidobacterium	hypothetical protein	cas8b2	cd09665	CAS-I-B			
1619194..1619920	-	AMW33140.1	CP014334.1	Fervidobacterium islandicum	Bacteria	Thermotogae	Thermotogae	Thermotogales	Fervidobacteriaceae	Fervidobacterium	hypothetical protein	cas6	COG1583	CAS-I,CAS-III,CAS-IV			
1621195..1621756	-	AMW33769.1	CP014334.1	Fervidobacterium islandicum	Bacteria	Thermotogae	Thermotogae	Thermotogales	Fervidobacteriaceae	Fervidobacterium	cell wall biosynthesis protein						
1622584..1624899	array		CP014334.1	Fervidobacterium islandicum	Bacteria	Thermotogae	Thermotogae	Thermotogales	Fervidobacteriaceae	Fervidobacterium		CRISPR array			31	41	35
1625507..1626947	-	AMW33141.1	CP014334.1	Fervidobacterium islandicum	Bacteria	Thermotogae	Thermotogae	Thermotogales	Fervidobacteriaceae	Fervidobacterium	hypothetical protein	csx1	cd09747	CAS-III			
441 CAS-I-B | ACN99600.1																	
747321..748119	+	ACN99082.1	CP001229.1	Sulfurihydrogenibium azorense Az-Fu1	Bacteria	Aquificae	Aquificae	Aquificales	Hydrogenothermaceae	Sulfurihydrogenibium	crispr-associated protein Cas6	cas6	cd09652	CAS-I,CAS-III			
748118..749840	+	ACN98336.1	CP001229.1	Sulfurihydrogenibium azorense Az-Fu1	Bacteria	Aquificae	Aquificae	Aquificales	Hydrogenothermaceae	Sulfurihydrogenibium	crispr-associated cxxc_cxxc protein Cst1	cas8b2	cd09665	CAS-I-B			
749832..750759	+	ACN98791.1	CP001229.1	Sulfurihydrogenibium azorense Az-Fu1	Bacteria	Aquificae	Aquificae	Aquificales	Hydrogenothermaceae	Sulfurihydrogenibium	crispr-associated regulatory protein, devr family	cas7	cd09687	CAS-I-B			
750761..751472	+	ACN99737.1	CP001229.1	Sulfurihydrogenibium azorense Az-Fu1	Bacteria	Aquificae	Aquificae	Aquificales	Hydrogenothermaceae	Sulfurihydrogenibium	crispr-associated protein Cas5, tneap subtype	cas5	cd09658	CAS-I-B			
751473..753741	+	ACN98177.1	CP001229.1	Sulfurihydrogenibium azorense Az-Fu1	Bacteria	Aquificae	Aquificae	Aquificales	Hydrogenothermaceae	Sulfurihydrogenibium	crispr-associated helicase Cas3	cas3HD,cas3	cd09641,COG1203	CAS-I			
753737..754250	+	ACN99121.1	CP001229.1	Sulfurihydrogenibium azorense Az-Fu1	Bacteria	Aquificae	Aquificae	Aquificales	Hydrogenothermaceae	Sulfurihydrogenibium	crispr-associated protein Cas4	cas4	pfam01930	CAS-I			
754266..755262	+	ACN98621.1	CP001229.1	Sulfurihydrogenibium azorense Az-Fu1	Bacteria	Aquificae	Aquificae	Aquificales	Hydrogenothermaceae	Sulfurihydrogenibium	crispr-associated protein Cas1	cas1	cd09722	CAS-I-B			
755264..755531	+	ACN99600.1	CP001229.1	Sulfurihydrogenibium azorense Az-Fu1	Bacteria	Aquificae	Aquificae	Aquificales	Hydrogenothermaceae	Sulfurihydrogenibium	crispr-associated protein Cas2	cas2	cd09725	CAS-I,CAS-II,CAS-III,CAS-V			
755556..757154	array		CP001229.1	Sulfurihydrogenibium azorense Az-Fu1	Bacteria	Aquificae	Aquificae	Aquificales	Hydrogenothermaceae	Sulfurihydrogenibium		CRISPR array			25	36	29
442 CAS-I-B | AUS09238.1																	
2191817..2194523	array		CP025943.1	Laceyella sacchari	Bacteria	Firmicutes	Bacilli	Bacillales	Thermoactinomycetaceae	Laceyella		CRISPR array			42	36	29
2194967..2195231	-	AUS09238.1	CP025943.1	Laceyella sacchari	Bacteria	Firmicutes	Bacilli	Bacillales	Thermoactinomycetaceae	Laceyella	CRISPR-associated endonuclease Cas2	cas2	cd09725	CAS-I,CAS-II,CAS-III,CAS-V			
2195233..2196217	-	AUS09239.1	CP025943.1	Laceyella sacchari	Bacteria	Firmicutes	Bacilli	Bacillales	Thermoactinomycetaceae	Laceyella	subtype I-B CRISPR-associated endonuclease Cas1	cas1	cd09722	CAS-I-B			
2196213..2196726	-	AUS09240.1	CP025943.1	Laceyella sacchari	Bacteria	Firmicutes	Bacilli	Bacillales	Thermoactinomycetaceae	Laceyella	CRISPR-associated protein Cas4	cas4	pfam01930	CAS-I			
2196737..2199023	-	AUS09241.1	CP025943.1	Laceyella sacchari	Bacteria	Firmicutes	Bacilli	Bacillales	Thermoactinomycetaceae	Laceyella	CRISPR-associated helicase/endonuclease Cas3	cas3HD,cas3	cd09641,COG1203	CAS-I			
2199052..2199706	-	AUS09242.1	CP025943.1	Laceyella sacchari	Bacteria	Firmicutes	Bacilli	Bacillales	Thermoactinomycetaceae	Laceyella	type I-B CRISPR-associated protein Cas5	cas5	cd09658	CAS-I-B			
2199686..2200607	-	AUS09243.1	CP025943.1	Laceyella sacchari	Bacteria	Firmicutes	Bacilli	Bacillales	Thermoactinomycetaceae	Laceyella	type I-B CRISPR-associated protein Cas7/Cst2/DevR	cas7	cd09687	CAS-I-B			
2200599..2202336	-	AUS09244.1	CP025943.1	Laceyella sacchari	Bacteria	Firmicutes	Bacilli	Bacillales	Thermoactinomycetaceae	Laceyella	hypothetical protein	cas8b2,cas8b2	cd09665,cd09665	CAS-I-B			
2202332..2203067	-	AUS09245.1	CP025943.1	Laceyella sacchari	Bacteria	Firmicutes	Bacilli	Bacillales	Thermoactinomycetaceae	Laceyella	CRISPR-associated endoribonuclease Cas6	cas6	COG1583	CAS-I,CAS-III,CAS-IV			
2203338..2205321	array		CP025943.1	Laceyella sacchari	Bacteria	Firmicutes	Bacilli	Bacillales	Thermoactinomycetaceae	Laceyella		CRISPR array			31	36	29
443 CAS-I-B | RAL26195.1																	
161255..161741	array		KZ845664.1	Thermoflavimicrobium sp. FBKL4.011	Bacteria	Firmicutes	Bacilli	Bacillales	Thermoactinomycetaceae	Thermoflavimicrobium		CRISPR array			8	36	29
162010..162751	+	RAL26188.1	KZ845664.1	Thermoflavimicrobium sp. FBKL4.011	Bacteria	Firmicutes	Bacilli	Bacillales	Thermoactinomycetaceae	Thermoflavimicrobium	CRISPR-associated endoribonuclease Cas6	cas6	COG1583	CAS-I,CAS-III,CAS-IV			
162765..164487	+	RAL26189.1	KZ845664.1	Thermoflavimicrobium sp. FBKL4.011	Bacteria	Firmicutes	Bacilli	Bacillales	Thermoactinomycetaceae	Thermoflavimicrobium	hypothetical protein	cas8b2,cas8b2	cd09665,cd09665	CAS-I-B			
164479..165382	+	RAL26190.1	KZ845664.1	Thermoflavimicrobium sp. FBKL4.011	Bacteria	Firmicutes	Bacilli	Bacillales	Thermoactinomycetaceae	Thermoflavimicrobium	type I-B CRISPR-associated protein Cas7/Cst2/DevR	cas7	cd09687	CAS-I-B			
165381..166038	+	RAL26191.1	KZ845664.1	Thermoflavimicrobium sp. FBKL4.011	Bacteria	Firmicutes	Bacilli	Bacillales	Thermoactinomycetaceae	Thermoflavimicrobium	type I-B CRISPR-associated protein Cas5	cas5	cd09658	CAS-I-B			
166080..168402	+	RAL26192.1	KZ845664.1	Thermoflavimicrobium sp. FBKL4.011	Bacteria	Firmicutes	Bacilli	Bacillales	Thermoactinomycetaceae	Thermoflavimicrobium	CRISPR-associated helicase/endonuclease Cas3	cas3HD,cas3	cd09641,cd09639	CAS-I			
168414..168933	+	RAL26193.1	KZ845664.1	Thermoflavimicrobium sp. FBKL4.011	Bacteria	Firmicutes	Bacilli	Bacillales	Thermoactinomycetaceae	Thermoflavimicrobium	CRISPR-associated protein Cas4	cas4	pfam01930	CAS-I			
168929..169913	+	RAL26194.1	KZ845664.1	Thermoflavimicrobium sp. FBKL4.011	Bacteria	Firmicutes	Bacilli	Bacillales	Thermoactinomycetaceae	Thermoflavimicrobium	subtype I-B CRISPR-associated endonuclease Cas1	cas1	cd09722	CAS-I-B			
169914..170178	+	RAL26195.1	KZ845664.1	Thermoflavimicrobium sp. FBKL4.011	Bacteria	Firmicutes	Bacilli	Bacillales	Thermoactinomycetaceae	Thermoflavimicrobium	CRISPR-associated endonuclease Cas2	cas2	cd09725	CAS-I,CAS-II,CAS-III,CAS-V			
170385..171594	array		KZ845664.1	Thermoflavimicrobium sp. FBKL4.011	Bacteria	Firmicutes	Bacilli	Bacillales	Thermoactinomycetaceae	Thermoflavimicrobium		CRISPR array			19	36	29
444 CAS-I-B | PZX05811.1																	
257542..258277	+	PZX05805.1	QKZI01000002.1	Psychrobacillus insolitus	Bacteria	Firmicutes	Bacilli	Bacillales	Bacillaceae	Psychrobacillus	CRISPR-associated Cas6 family protein	cas6	pfam01881	CAS-I,CAS-III			
258292..259972	+	PZX05806.1	QKZI01000002.1	Psychrobacillus insolitus	Bacteria	Firmicutes	Bacilli	Bacillales	Bacillaceae	Psychrobacillus	CRISPR-associated protein Cst1	cas8b2,cas8b2	cd09754,cd09665	CAS-I-B			
259964..260849	+	PZX05807.1	QKZI01000002.1	Psychrobacillus insolitus	Bacteria	Firmicutes	Bacilli	Bacillales	Bacillaceae	Psychrobacillus	CRISPR-associated Cst2 family autoregulator	cas7	cd09687	CAS-I-B			
260861..261596	+	PZX05808.1	QKZI01000002.1	Psychrobacillus insolitus	Bacteria	Firmicutes	Bacilli	Bacillales	Bacillaceae	Psychrobacillus	CRISPR-associated protein Cas5t	cas5	cd09658	CAS-I-B			
261605..263819	+	PZX05809.1	QKZI01000002.1	Psychrobacillus insolitus	Bacteria	Firmicutes	Bacilli	Bacillales	Bacillaceae	Psychrobacillus	CRISPR-associated Cas3 family helicase	cas3HD,cas3	cd09641,cd09639	CAS-I			
263884..264361	+	PZX05810.1	QKZI01000002.1	Psychrobacillus insolitus	Bacteria	Firmicutes	Bacilli	Bacillales	Bacillaceae	Psychrobacillus	CRISPR-associated endonuclease Cas1	cas1	cd09722	CAS-I-B			
264545..264809	+	PZX05811.1	QKZI01000002.1	Psychrobacillus insolitus	Bacteria	Firmicutes	Bacilli	Bacillales	Bacillaceae	Psychrobacillus	CRISPR-associated protein Cas2	cas2	cd09725	CAS-I,CAS-II,CAS-III,CAS-V			
264992..265213	array		QKZI01000002.1	Psychrobacillus insolitus	Bacteria	Firmicutes	Bacilli	Bacillales	Bacillaceae	Psychrobacillus		CRISPR array			4	35	29
445 partial CAS-I-B | PGA47418.1																	
9..279	+	PGA47419.1	NVHB01000420.1	Bacillus pseudomycoides	Bacteria	Firmicutes	Bacilli	Bacillales	Bacillaceae	Bacillus	CRISPR-associated endonuclease Cas1	cas1	cd09722	CAS-I-B			
280..544	+	PGA47418.1	NVHB01000420.1	Bacillus pseudomycoides	Bacteria	Firmicutes	Bacilli	Bacillales	Bacillaceae	Bacillus	CRISPR-associated endonuclease Cas2	cas2	cd09725	CAS-I,CAS-II,CAS-III,CAS-V			
446 CAS-I-B | PFW64696.1																	
9198..9969	+	PFW64689.1	NVET01000208.1	Bacillus pseudomycoides	Bacteria	Firmicutes	Bacilli	Bacillales	Bacillaceae	Bacillus	CRISPR-associated endoribonuclease Cas6	cas6	COG1583	CAS-I,CAS-III,CAS-IV			
9980..11678	+	PFW64690.1	NVET01000208.1	Bacillus pseudomycoides	Bacteria	Firmicutes	Bacilli	Bacillales	Bacillaceae	Bacillus	type I-B CRISPR-associated protein Cas8b1/Cst1	cas8b2	cd09665	CAS-I-B			
11652..12531	+	PFW64691.1	NVET01000208.1	Bacillus pseudomycoides	Bacteria	Firmicutes	Bacilli	Bacillales	Bacillaceae	Bacillus	type I-B CRISPR-associated protein Cas7/Cst2/DevR	cas7	cd09687	CAS-I-B			
12540..13272	+	PFW64692.1	NVET01000208.1	Bacillus pseudomycoides	Bacteria	Firmicutes	Bacilli	Bacillales	Bacillaceae	Bacillus	type I-B CRISPR-associated protein Cas5	cas5	cd09658	CAS-I-B			
13322..15569	+	PFW64693.1	NVET01000208.1	Bacillus pseudomycoides	Bacteria	Firmicutes	Bacilli	Bacillales	Bacillaceae	Bacillus	CRISPR-associated helicase/endonuclease Cas3	cas3HD,cas3	cd09641,COG1203	CAS-I			
15581..16088	+	PFW64694.1	NVET01000208.1	Bacillus pseudomycoides	Bacteria	Firmicutes	Bacilli	Bacillales	Bacillaceae	Bacillus	CRISPR-associated protein Cas4	cas4	pfam01930	CAS-I			
16084..17074	+	PFW64695.1	NVET01000208.1	Bacillus pseudomycoides	Bacteria	Firmicutes	Bacilli	Bacillales	Bacillaceae	Bacillus	subtype I-B CRISPR-associated endonuclease Cas1	cas1	cd09722	CAS-I-B			
17075..17339	+	PFW64696.1	NVET01000208.1	Bacillus pseudomycoides	Bacteria	Firmicutes	Bacilli	Bacillales	Bacillaceae	Bacillus	CRISPR-associated endonuclease Cas2	cas2	cd09725	CAS-I,CAS-II,CAS-III,CAS-V			
17530..18207	array		NVET01000208.1	Bacillus pseudomycoides	Bacteria	Firmicutes	Bacilli	Bacillales	Bacillaceae	Bacillus		CRISPR array			11	35	29
447 CAS-I-B | PFW73874.1																	
29017..29694	array		NVEU01000079.1	Bacillus pseudomycoides	Bacteria	Firmicutes	Bacilli	Bacillales	Bacillaceae	Bacillus		CRISPR array			11	35	29
29884..30148	-	PFW73874.1	NVEU01000079.1	Bacillus pseudomycoides	Bacteria	Firmicutes	Bacilli	Bacillales	Bacillaceae	Bacillus	CRISPR-associated endonuclease Cas2	cas2	cd09725	CAS-I,CAS-II,CAS-III,CAS-V			
30149..31139	-	PFW73875.1	NVEU01000079.1	Bacillus pseudomycoides	Bacteria	Firmicutes	Bacilli	Bacillales	Bacillaceae	Bacillus	subtype I-B CRISPR-associated endonuclease Cas1	cas1	cd09722	CAS-I-B			
31135..31642	-	PFW73876.1	NVEU01000079.1	Bacillus pseudomycoides	Bacteria	Firmicutes	Bacilli	Bacillales	Bacillaceae	Bacillus	CRISPR-associated protein Cas4	cas4	pfam01930	CAS-I			
31654..33901	-	PFW73877.1	NVEU01000079.1	Bacillus pseudomycoides	Bacteria	Firmicutes	Bacilli	Bacillales	Bacillaceae	Bacillus	CRISPR-associated helicase/endonuclease Cas3	cas3HD,cas3	cd09641,COG1203	CAS-I			
33951..34683	-	PFW73878.1	NVEU01000079.1	Bacillus pseudomycoides	Bacteria	Firmicutes	Bacilli	Bacillales	Bacillaceae	Bacillus	type I-B CRISPR-associated protein Cas5	cas5	cd09658	CAS-I-B			
34692..35571	-	PFW73879.1	NVEU01000079.1	Bacillus pseudomycoides	Bacteria	Firmicutes	Bacilli	Bacillales	Bacillaceae	Bacillus	type I-B CRISPR-associated protein Cas7/Cst2/DevR	cas7	cd09687	CAS-I-B			
35545..37243	-	PFW73880.1	NVEU01000079.1	Bacillus pseudomycoides	Bacteria	Firmicutes	Bacilli	Bacillales	Bacillaceae	Bacillus	type I-B CRISPR-associated protein Cas8b1/Cst1	cas8b2	cd09665	CAS-I-B			
37254..38025	-	PFW73881.1	NVEU01000079.1	Bacillus pseudomycoides	Bacteria	Firmicutes	Bacilli	Bacillales	Bacillaceae	Bacillus	CRISPR-associated endoribonuclease Cas6	cas6	COG1583	CAS-I,CAS-III,CAS-IV			
448 CAS-I-B | PFZ53138.1																	
9198..9969	+	PFZ53131.1	NVGI01000070.1	Bacillus pseudomycoides	Bacteria	Firmicutes	Bacilli	Bacillales	Bacillaceae	Bacillus	CRISPR-associated endoribonuclease Cas6	cas6	COG1583	CAS-I,CAS-III,CAS-IV			
9980..11678	+	PFZ53132.1	NVGI01000070.1	Bacillus pseudomycoides	Bacteria	Firmicutes	Bacilli	Bacillales	Bacillaceae	Bacillus	type I-B CRISPR-associated protein Cas8b1/Cst1	cas8b2	cd09665	CAS-I-B			
11652..12531	+	PFZ53133.1	NVGI01000070.1	Bacillus pseudomycoides	Bacteria	Firmicutes	Bacilli	Bacillales	Bacillaceae	Bacillus	type I-B CRISPR-associated protein Cas7/Cst2/DevR	cas7	cd09687	CAS-I-B			
12540..13272	+	PFZ53134.1	NVGI01000070.1	Bacillus pseudomycoides	Bacteria	Firmicutes	Bacilli	Bacillales	Bacillaceae	Bacillus	type I-B CRISPR-associated protein Cas5	cas5	cd09658	CAS-I-B			
13322..15569	+	PFZ53135.1	NVGI01000070.1	Bacillus pseudomycoides	Bacteria	Firmicutes	Bacilli	Bacillales	Bacillaceae	Bacillus	CRISPR-associated helicase/endonuclease Cas3	cas3HD,cas3	cd09641,COG1203	CAS-I			
15581..16088	+	PFZ53136.1	NVGI01000070.1	Bacillus pseudomycoides	Bacteria	Firmicutes	Bacilli	Bacillales	Bacillaceae	Bacillus	CRISPR-associated protein Cas4	cas4	pfam01930	CAS-I			
16084..17074	+	PFZ53137.1	NVGI01000070.1	Bacillus pseudomycoides	Bacteria	Firmicutes	Bacilli	Bacillales	Bacillaceae	Bacillus	subtype I-B CRISPR-associated endonuclease Cas1	cas1	cd09722	CAS-I-B			
17075..17339	+	PFZ53138.1	NVGI01000070.1	Bacillus pseudomycoides	Bacteria	Firmicutes	Bacilli	Bacillales	Bacillaceae	Bacillus	CRISPR-associated endonuclease Cas2	cas2	cd09725	CAS-I,CAS-II,CAS-III,CAS-V			
17530..18207	array		NVGI01000070.1	Bacillus pseudomycoides	Bacteria	Firmicutes	Bacilli	Bacillales	Bacillaceae	Bacillus		CRISPR array			11	35	29
449 CAS-I-B | PHA49748.1																	
9531..10302	+	PHA49741.1	NUQM01000027.1	Bacillus pseudomycoides	Bacteria	Firmicutes	Bacilli	Bacillales	Bacillaceae	Bacillus	CRISPR-associated endoribonuclease Cas6	cas6	COG1583	CAS-I,CAS-III,CAS-IV			
10313..12011	+	PHA49742.1	NUQM01000027.1	Bacillus pseudomycoides	Bacteria	Firmicutes	Bacilli	Bacillales	Bacillaceae	Bacillus	type I-B CRISPR-associated protein Cas8b1/Cst1	cas8b2	cd09665	CAS-I-B			
11985..12864	+	PHA49743.1	NUQM01000027.1	Bacillus pseudomycoides	Bacteria	Firmicutes	Bacilli	Bacillales	Bacillaceae	Bacillus	type I-B CRISPR-associated protein Cas7/Cst2/DevR	cas7	cd09687	CAS-I-B			
12873..13605	+	PHA49744.1	NUQM01000027.1	Bacillus pseudomycoides	Bacteria	Firmicutes	Bacilli	Bacillales	Bacillaceae	Bacillus	type I-B CRISPR-associated protein Cas5	cas5	cd09658	CAS-I-B			
13655..15902	+	PHA49745.1	NUQM01000027.1	Bacillus pseudomycoides	Bacteria	Firmicutes	Bacilli	Bacillales	Bacillaceae	Bacillus	CRISPR-associated helicase/endonuclease Cas3	cas3HD,cas3	cd09641,COG1203	CAS-I			
15914..16421	+	PHA49746.1	NUQM01000027.1	Bacillus pseudomycoides	Bacteria	Firmicutes	Bacilli	Bacillales	Bacillaceae	Bacillus	CRISPR-associated protein Cas4	cas4	pfam01930	CAS-I			
16417..17407	+	PHA49747.1	NUQM01000027.1	Bacillus pseudomycoides	Bacteria	Firmicutes	Bacilli	Bacillales	Bacillaceae	Bacillus	subtype I-B CRISPR-associated endonuclease Cas1	cas1	cd09722	CAS-I-B			
17408..17672	+	PHA49748.1	NUQM01000027.1	Bacillus pseudomycoides	Bacteria	Firmicutes	Bacilli	Bacillales	Bacillaceae	Bacillus	CRISPR-associated endonuclease Cas2	cas2	cd09725	CAS-I,CAS-II,CAS-III,CAS-V			
17863..18540	array		NUQM01000027.1	Bacillus pseudomycoides	Bacteria	Firmicutes	Bacilli	Bacillales	Bacillaceae	Bacillus		CRISPR array			11	35	29
450 CAS-I-B | PHA54520.1																	
233..910	array		NUQN01000091.1	Bacillus pseudomycoides	Bacteria	Firmicutes	Bacilli	Bacillales	Bacillaceae	Bacillus		CRISPR array			11	35	29
1100..1364	-	PHA54520.1	NUQN01000091.1	Bacillus pseudomycoides	Bacteria	Firmicutes	Bacilli	Bacillales	Bacillaceae	Bacillus	CRISPR-associated endonuclease Cas2	cas2	cd09725	CAS-I,CAS-II,CAS-III,CAS-V			
1365..2355	-	PHA54521.1	NUQN01000091.1	Bacillus pseudomycoides	Bacteria	Firmicutes	Bacilli	Bacillales	Bacillaceae	Bacillus	subtype I-B CRISPR-associated endonuclease Cas1	cas1	cd09722	CAS-I-B			
2351..2858	-	PHA54522.1	NUQN01000091.1	Bacillus pseudomycoides	Bacteria	Firmicutes	Bacilli	Bacillales	Bacillaceae	Bacillus	CRISPR-associated protein Cas4	cas4	pfam01930	CAS-I			
2870..5117	-	PHA54523.1	NUQN01000091.1	Bacillus pseudomycoides	Bacteria	Firmicutes	Bacilli	Bacillales	Bacillaceae	Bacillus	CRISPR-associated helicase/endonuclease Cas3	cas3HD,cas3	cd09641,COG1203	CAS-I			
5167..5899	-	PHA54524.1	NUQN01000091.1	Bacillus pseudomycoides	Bacteria	Firmicutes	Bacilli	Bacillales	Bacillaceae	Bacillus	type I-B CRISPR-associated protein Cas5	cas5	cd09658	CAS-I-B			
5908..6787	-	PHA54525.1	NUQN01000091.1	Bacillus pseudomycoides	Bacteria	Firmicutes	Bacilli	Bacillales	Bacillaceae	Bacillus	type I-B CRISPR-associated protein Cas7/Cst2/DevR	cas7	cd09687	CAS-I-B			
6761..8459	-	PHA54526.1	NUQN01000091.1	Bacillus pseudomycoides	Bacteria	Firmicutes	Bacilli	Bacillales	Bacillaceae	Bacillus	type I-B CRISPR-associated protein Cas8b1/Cst1	cas8b2	cd09665	CAS-I-B			
8470..9241	-	PHA54527.1	NUQN01000091.1	Bacillus pseudomycoides	Bacteria	Firmicutes	Bacilli	Bacillales	Bacillaceae	Bacillus	CRISPR-associated endoribonuclease Cas6	cas6	COG1583	CAS-I,CAS-III,CAS-IV			
451 CAS-I-B | PHB78532.1																	
6770..7541	+	PHB78525.1	NURH01000016.1	Bacillus pseudomycoides	Bacteria	Firmicutes	Bacilli	Bacillales	Bacillaceae	Bacillus	CRISPR-associated endoribonuclease Cas6	cas6	COG1583	CAS-I,CAS-III,CAS-IV			
7552..9250	+	PHB78526.1	NURH01000016.1	Bacillus pseudomycoides	Bacteria	Firmicutes	Bacilli	Bacillales	Bacillaceae	Bacillus	type I-B CRISPR-associated protein Cas8b1/Cst1	cas8b2	cd09665	CAS-I-B			
9224..10103	+	PHB78527.1	NURH01000016.1	Bacillus pseudomycoides	Bacteria	Firmicutes	Bacilli	Bacillales	Bacillaceae	Bacillus	type I-B CRISPR-associated protein Cas7/Cst2/DevR	cas7	cd09687	CAS-I-B			
10112..10844	+	PHB78528.1	NURH01000016.1	Bacillus pseudomycoides	Bacteria	Firmicutes	Bacilli	Bacillales	Bacillaceae	Bacillus	type I-B CRISPR-associated protein Cas5	cas5	cd09658	CAS-I-B			
10894..13141	+	PHB78529.1	NURH01000016.1	Bacillus pseudomycoides	Bacteria	Firmicutes	Bacilli	Bacillales	Bacillaceae	Bacillus	CRISPR-associated helicase/endonuclease Cas3	cas3HD,cas3	cd09641,COG1203	CAS-I			
13153..13660	+	PHB78530.1	NURH01000016.1	Bacillus pseudomycoides	Bacteria	Firmicutes	Bacilli	Bacillales	Bacillaceae	Bacillus	CRISPR-associated protein Cas4	cas4	pfam01930	CAS-I			
13656..14646	+	PHB78531.1	NURH01000016.1	Bacillus pseudomycoides	Bacteria	Firmicutes	Bacilli	Bacillales	Bacillaceae	Bacillus	subtype I-B CRISPR-associated endonuclease Cas1	cas1	cd09722	CAS-I-B			
14647..14911	+	PHB78532.1	NURH01000016.1	Bacillus pseudomycoides	Bacteria	Firmicutes	Bacilli	Bacillales	Bacillaceae	Bacillus	CRISPR-associated endonuclease Cas2	cas2	cd09725	CAS-I,CAS-II,CAS-III,CAS-V			
452 CAS-I-B | PHC60331.1																	
9353..10124	+	PHC60324.1	NURV01000002.1	Bacillus pseudomycoides	Bacteria	Firmicutes	Bacilli	Bacillales	Bacillaceae	Bacillus	CRISPR-associated endoribonuclease Cas6	cas6	COG1583	CAS-I,CAS-III,CAS-IV			
10135..11833	+	PHC60325.1	NURV01000002.1	Bacillus pseudomycoides	Bacteria	Firmicutes	Bacilli	Bacillales	Bacillaceae	Bacillus	type I-B CRISPR-associated protein Cas8b1/Cst1	cas8b2	cd09665	CAS-I-B			
11807..12686	+	PHC60326.1	NURV01000002.1	Bacillus pseudomycoides	Bacteria	Firmicutes	Bacilli	Bacillales	Bacillaceae	Bacillus	type I-B CRISPR-associated protein Cas7/Cst2/DevR	cas7	cd09687	CAS-I-B			
12695..13427	+	PHC60327.1	NURV01000002.1	Bacillus pseudomycoides	Bacteria	Firmicutes	Bacilli	Bacillales	Bacillaceae	Bacillus	type I-B CRISPR-associated protein Cas5	cas5	cd09658	CAS-I-B			
13477..15724	+	PHC60328.1	NURV01000002.1	Bacillus pseudomycoides	Bacteria	Firmicutes	Bacilli	Bacillales	Bacillaceae	Bacillus	CRISPR-associated helicase/endonuclease Cas3	cas3HD,cas3	cd09641,COG1203	CAS-I			
15736..16243	+	PHC60329.1	NURV01000002.1	Bacillus pseudomycoides	Bacteria	Firmicutes	Bacilli	Bacillales	Bacillaceae	Bacillus	CRISPR-associated protein Cas4	cas4	pfam01930	CAS-I			

16239..17229	+	PHC60330.1	NURV01000002.1	Bacillus pseudomycoides	Bacteria	Firmicutes	Bacilli	Bacillales	Bacillaceae	Bacillus	subtype I-B CRISPR-associated endonuclease Cas1	cas1	cd09722	CAS-I-B			
17230..17494	+	PHC60331.1	NURV01000002.1	Bacillus pseudomycoides	Bacteria	Firmicutes	Bacilli	Bacillales	Bacillaceae	Bacillus	CRISPR-associated endonuclease Cas2	cas2	cd09725	CAS-I,CAS-II,CAS-III,CAS-V			
17685..18362	array		NURV01000002.1	Bacillus pseudomycoides	Bacteria	Firmicutes	Bacilli	Bacillales	Bacillaceae	Bacillus		CRISPR array			11	35	29

453 CAS-I-B | PHE42109.1

304..981	array		NUTD01000128.1	Bacillus pseudomycoides	Bacteria	Firmicutes	Bacilli	Bacillales	Bacillaceae	Bacillus		CRISPR array			11	35	29
1171..1435	-	PHE42109.1	NUTD01000128.1	Bacillus pseudomycoides	Bacteria	Firmicutes	Bacilli	Bacillales	Bacillaceae	Bacillus	CRISPR-associated endonuclease Cas2	cas2	cd09725	CAS-I,CAS-II,CAS-III,CAS-V			
1436..2426	-	PHE42110.1	NUTD01000128.1	Bacillus pseudomycoides	Bacteria	Firmicutes	Bacilli	Bacillales	Bacillaceae	Bacillus	subtype I-B CRISPR-associated endonuclease Cas1	cas1	cd09722	CAS-I-B			
2422..2929	-	PHE42111.1	NUTD01000128.1	Bacillus pseudomycoides	Bacteria	Firmicutes	Bacilli	Bacillales	Bacillaceae	Bacillus	CRISPR-associated protein Cas4	cas4	pfam01930	CAS-I			
2941..5188	-	PHE42112.1	NUTD01000128.1	Bacillus pseudomycoides	Bacteria	Firmicutes	Bacilli	Bacillales	Bacillaceae	Bacillus	CRISPR-associated helicase/endonuclease Cas3	cas3HD,cas3	cd09641,COG1203	CAS-I			
5238..5970	-	PHE42113.1	NUTD01000128.1	Bacillus pseudomycoides	Bacteria	Firmicutes	Bacilli	Bacillales	Bacillaceae	Bacillus	type I-B CRISPR-associated protein Cas5	cas5	cd09658	CAS-I-B			
5979..6858	-	PHE42114.1	NUTD01000128.1	Bacillus pseudomycoides	Bacteria	Firmicutes	Bacilli	Bacillales	Bacillaceae	Bacillus	type I-B CRISPR-associated protein Cas7/Cst2/DevR	cas7	cd09687	CAS-I-B			
6832..8530	-	PHE42115.1	NUTD01000128.1	Bacillus pseudomycoides	Bacteria	Firmicutes	Bacilli	Bacillales	Bacillaceae	Bacillus	type I-B CRISPR-associated protein Cas8b1/Cst1	cas8b2	cd09665	CAS-I-B			
8541..9312	-	PHE42116.1	NUTD01000128.1	Bacillus pseudomycoides	Bacteria	Firmicutes	Bacilli	Bacillales	Bacillaceae	Bacillus	CRISPR-associated endoribonuclease Cas6	cas6	COG1583	CAS-I,CAS-III,CAS-IV			

454 CAS-I-B | EOD01826.1

1743..2649	+	EOD01818.1	ARZA01000015.1	Caldisalinibacter kiritimatiensis	Bacteria	Firmicutes	Clostridia	Clostridiales	Clostridiaceae	Caldisalinibacter	hypothetical protein	WYL	COG2378	CAS-I,CAS-III			
2727..3456	+	EOD01819.1	ARZA01000015.1	Caldisalinibacter kiritimatiensis	Bacteria	Firmicutes	Clostridia	Clostridiales	Clostridiaceae	Caldisalinibacter	hypothetical protein	cas6	COG1583	CAS-I,CAS-III,CAS-IV			
3496..5179	+	EOD01820.1	ARZA01000015.1	Caldisalinibacter kiritimatiensis	Bacteria	Firmicutes	Clostridia	Clostridiales	Clostridiaceae	Caldisalinibacter	hypothetical protein	cas8b2	cd09665	CAS-I-B			
5180..6089	+	EOD01821.1	ARZA01000015.1	Caldisalinibacter kiritimatiensis	Bacteria	Firmicutes	Clostridia	Clostridiales	Clostridiaceae	Caldisalinibacter	CRISPR-associated negative autoregulator	cas7	cd09687	CAS-I-B			
6091..6751	+	EOD01822.1	ARZA01000015.1	Caldisalinibacter kiritimatiensis	Bacteria	Firmicutes	Clostridia	Clostridiales	Clostridiaceae	Caldisalinibacter	CRISPR-associated protein	cas5	cd09658	CAS-I-B			
6781..8950	+	EOD01823.1	ARZA01000015.1	Caldisalinibacter kiritimatiensis	Bacteria	Firmicutes	Clostridia	Clostridiales	Clostridiaceae	Caldisalinibacter	CRISPR-associated helicase Cas3	cas3	cd09639	CAS-I			
8962..9481	+	EOD01824.1	ARZA01000015.1	Caldisalinibacter kiritimatiensis	Bacteria	Firmicutes	Clostridia	Clostridiales	Clostridiaceae	Caldisalinibacter	CRISPR-associated RecB family exonuclease Cas4a	cas4	pfam01930	CAS-I			
9483..10467	+	EOD01825.1	ARZA01000015.1	Caldisalinibacter kiritimatiensis	Bacteria	Firmicutes	Clostridia	Clostridiales	Clostridiaceae	Caldisalinibacter	CRISPR-associated protein Cas1	cas1	cd09722	CAS-I-B			
10468..10732	+	EOD01826.1	ARZA01000015.1	Caldisalinibacter kiritimatiensis	Bacteria	Firmicutes	Clostridia	Clostridiales	Clostridiaceae	Caldisalinibacter	CRISPR-associated protein Cas2	cas2	cd09725	CAS-I,CAS-II,CAS-III,CAS-V			
10920..12664	array		ARZA01000015.1	Caldisalinibacter kiritimatiensis	Bacteria	Firmicutes	Clostridia	Clostridiales	Clostridiaceae	Caldisalinibacter		CRISPR array			27	37	29

455 CAS-I-B | AOT70862.1

3332598..3333555	+	AOT70854.1	CP017269.1	Geosporobacter ferrireducens	Bacteria	Firmicutes	Clostridia	Clostridiales	Clostridiaceae	Geosporobacter	transcriptional regulator	WYL	COG2378	CAS-I,CAS-III			
3333620..3334349	+	AOT70855.1	CP017269.1	Geosporobacter ferrireducens	Bacteria	Firmicutes	Clostridia	Clostridiales	Clostridiaceae	Geosporobacter	CRISPR-associated endoribonuclease Cas6	cas6	pfam01881	CAS-I,CAS-III			
3334367..3336155	+	AOT70856.1	CP017269.1	Geosporobacter ferrireducens	Bacteria	Firmicutes	Clostridia	Clostridiales	Clostridiaceae	Geosporobacter	type I-B CRISPR-associated protein Cas8b1/Cst1	cas8b2	cd09665	CAS-I-B			
3336156..3337068	+	AOT70857.1	CP017269.1	Geosporobacter ferrireducens	Bacteria	Firmicutes	Clostridia	Clostridiales	Clostridiaceae	Geosporobacter	type I-B CRISPR-associated protein Cas7/Cst2/DevR	cas7	cd09687	CAS-I-B			
3337071..3337749	+	AOT70858.1	CP017269.1	Geosporobacter ferrireducens	Bacteria	Firmicutes	Clostridia	Clostridiales	Clostridiaceae	Geosporobacter	type I-B CRISPR-associated protein Cas5	cas5	cd09658	CAS-I-B			
3337778..3340025	+	AOT70859.1	CP017269.1	Geosporobacter ferrireducens	Bacteria	Firmicutes	Clostridia	Clostridiales	Clostridiaceae	Geosporobacter	hypothetical protein	cas3HD,cas3	cd09641,cd09639	CAS-I			
3340055..3340580	+	AOT70860.1	CP017269.1	Geosporobacter ferrireducens	Bacteria	Firmicutes	Clostridia	Clostridiales	Clostridiaceae	Geosporobacter	CRISPR-associated protein Cas4	cas4	pfam01930	CAS-I			
3340576..3341557	+	AOT70861.1	CP017269.1	Geosporobacter ferrireducens	Bacteria	Firmicutes	Clostridia	Clostridiales	Clostridiaceae	Geosporobacter	subtype I-B CRISPR-associated endonuclease Cas1	cas1	cd09722	CAS-I-B			
3341558..3341822	+	AOT70862.1	CP017269.1	Geosporobacter ferrireducens	Bacteria	Firmicutes	Clostridia	Clostridiales	Clostridiaceae	Geosporobacter	CRISPR-associated endonuclease Cas2	cas2	cd09725	CAS-I,CAS-II,CAS-III,CAS-V			
3341917..3345719	array		CP017269.1	Geosporobacter ferrireducens	Bacteria	Firmicutes	Clostridia	Clostridiales	Clostridiaceae	Geosporobacter		CRISPR array			58	37	29

456 CAS-I-B | SHK58781.1

2178..3189	+	SHK58531.1	FRAJ01000030.1	Caminicella sporogenes DSM 14501	Bacteria	Firmicutes	Clostridia	Clostridiales	Clostridiaceae	Caminicella	Predicted DNA-binding transcriptional regulator YafY, contains an HTH and WYL domains	WYL	pfam13280	CAS-I,CAS-III			
3286..4018	+	SHK58563.1	FRAJ01000030.1	Caminicella sporogenes DSM 14501	Bacteria	Firmicutes	Clostridia	Clostridiales	Clostridiaceae	Caminicella	CRISPR-associated protein, Cas6 family	cas6	COG1583	CAS-I,CAS-III,CAS-IV			
4021..5728	+	SHK58591.1	FRAJ01000030.1	Caminicella sporogenes DSM 14501	Bacteria	Firmicutes	Clostridia	Clostridiales	Clostridiaceae	Caminicella	CRISPR-associated protein Cst1	cas8b2,cas8b2	cd09754,cd09665	CAS-I-B			
5720..6623	+	SHK58623.1	FRAJ01000030.1	Caminicella sporogenes DSM 14501	Bacteria	Firmicutes	Clostridia	Clostridiales	Clostridiaceae	Caminicella	CRISPR-associated autoregulator, Cst2 family	cas7	cd09687	CAS-I-B			
6631..7390	+	SHK58652.1	FRAJ01000030.1	Caminicella sporogenes DSM 14501	Bacteria	Firmicutes	Clostridia	Clostridiales	Clostridiaceae	Caminicella	CRISPR-associated protein, Cas5t family	cas5	cd09658	CAS-I-B			
7431..9708	+	SHK58687.1	FRAJ01000030.1	Caminicella sporogenes DSM 14501	Bacteria	Firmicutes	Clostridia	Clostridiales	Clostridiaceae	Caminicella	CRISPR-associated helicase, Cas3 family	cas3HD,cas3	cd09641,COG1203	CAS-I			
9727..10246	+	SHK58716.1	FRAJ01000030.1	Caminicella sporogenes DSM 14501	Bacteria	Firmicutes	Clostridia	Clostridiales	Clostridiaceae	Caminicella	CRISPR-associated exonuclease Cas4	cas4	pfam01930	CAS-I			
10247..11228	+	SHK58751.1	FRAJ01000030.1	Caminicella sporogenes DSM 14501	Bacteria	Firmicutes	Clostridia	Clostridiales	Clostridiaceae	Caminicella	CRISPR-associated protein, Cas1 family	cas1	cd09722	CAS-I-B			
11253..11517	+	SHK58781.1	FRAJ01000030.1	Caminicella sporogenes DSM 14501	Bacteria	Firmicutes	Clostridia	Clostridiales	Clostridiaceae	Caminicella	CRISPR-associated protein Cas2	cas2	cd09725	CAS-I,CAS-II,CAS-III,CAS-V			

457 CAS-I-B | KGG79718.1

3559..4820	array		AZTB01000064.1	Caloranaerobacter azorensis H53214	Bacteria	Firmicutes	Clostridia	Clostridiales	Clostridiaceae	Caloranaerobacter		CRISPR array			20	35	29
5271..5535	-	KGG79718.1	AZTB01000064.1	Caloranaerobacter azorensis H53214	Bacteria	Firmicutes	Clostridia	Clostridiales	Clostridiaceae	Caloranaerobacter	CRISPR-associated protein Cas2	cas2	cd09725	CAS-I,CAS-II,CAS-III,CAS-V			
5536..6520	-	KGG79719.1	AZTB01000064.1	Caloranaerobacter azorensis H53214	Bacteria	Firmicutes	Clostridia	Clostridiales	Clostridiaceae	Caloranaerobacter	CRISPR-associated protein Cas1	cas1	cd09722	CAS-I-B			
6522..7038	-	KGG79720.1	AZTB01000064.1	Caloranaerobacter azorensis H53214	Bacteria	Firmicutes	Clostridia	Clostridiales	Clostridiaceae	Caloranaerobacter	CRISPR-associated protein Cas4	cas4	pfam01930	CAS-I			
7052..9293	-	KGG79721.1	AZTB01000064.1	Caloranaerobacter azorensis H53214	Bacteria	Firmicutes	Clostridia	Clostridiales	Clostridiaceae	Caloranaerobacter	CRISPR-associated protein Cas3	cas3	COG1203	CAS-I			
9401..10163	-	KGG79722.1	AZTB01000064.1	Caloranaerobacter azorensis H53214	Bacteria	Firmicutes	Clostridia	Clostridiales	Clostridiaceae	Caloranaerobacter	CRISPR-associated protein Cas5	cas5	cd09658	CAS-I-B			
10171..11056	-	KGG79723.1	AZTB01000064.1	Caloranaerobacter azorensis H53214	Bacteria	Firmicutes	Clostridia	Clostridiales	Clostridiaceae	Caloranaerobacter	CRISPR-associated protein	cas7	cd09687	CAS-I-B			
11048..12743	-	KGG79724.1	AZTB01000064.1	Caloranaerobacter azorensis H53214	Bacteria	Firmicutes	Clostridia	Clostridiales	Clostridiaceae	Caloranaerobacter	CRISPR-associated protein Cst1	cas8b2	cd09665	CAS-I-B			

458 CAS-I-B | KPU26819.1

38543..39494	+	KPU26812.1	JXLL01000012.1	Caloranaerobacter sp. TR13	Bacteria	Firmicutes	Clostridia	Clostridiales	Clostridiaceae	Caloranaerobacter	hypothetical protein	WYL	COG2378	CAS-I,CAS-III			
39564..40296	+	KPU26813.1	JXLL01000012.1	Caloranaerobacter sp. TR13	Bacteria	Firmicutes	Clostridia	Clostridiales	Clostridiaceae	Caloranaerobacter	CRISPR-associated protein Cas6	cas6	pfam01881	CAS-I,CAS-III			
40308..42000	+	KPU26824.1	JXLL01000012.1	Caloranaerobacter sp. TR13	Bacteria	Firmicutes	Clostridia	Clostridiales	Clostridiaceae	Caloranaerobacter	CRISPR-associated protein Cst1	cas8b2	cd09665	CAS-I-B			
41992..42877	+	KPU26814.1	JXLL01000012.1	Caloranaerobacter sp. TR13	Bacteria	Firmicutes	Clostridia	Clostridiales	Clostridiaceae	Caloranaerobacter	CRISPR-associated protein	cas7	cd09687	CAS-I-B			
42885..43647	+	KPU26815.1	JXLL01000012.1	Caloranaerobacter sp. TR13	Bacteria	Firmicutes	Clostridia	Clostridiales	Clostridiaceae	Caloranaerobacter	CRISPR-associated protein Cas5	cas5	cd09658	CAS-I-B			
43698..45933	+	KPU26816.1	JXLL01000012.1	Caloranaerobacter sp. TR13	Bacteria	Firmicutes	Clostridia	Clostridiales	Clostridiaceae	Caloranaerobacter	CRISPR-associated protein Cas3	cas3	COG1203	CAS-I			
45947..46466	+	KPU26817.1	JXLL01000012.1	Caloranaerobacter sp. TR13	Bacteria	Firmicutes	Clostridia	Clostridiales	Clostridiaceae	Caloranaerobacter	CRISPR-associated protein Cas4	cas4	pfam01930	CAS-I			
46468..47452	+	KPU26818.1	JXLL01000012.1	Caloranaerobacter sp. TR13	Bacteria	Firmicutes	Clostridia	Clostridiales	Clostridiaceae	Caloranaerobacter	CRISPR-associated protein Cas1	cas1	cd09722	CAS-I-B			
47453..47717	+	KPU26819.1	JXLL01000012.1	Caloranaerobacter sp. TR13	Bacteria	Firmicutes	Clostridia	Clostridiales	Clostridiaceae	Caloranaerobacter	CRISPR-associated protein Cas2	cas2	cd09725	CAS-I,CAS-II,CAS-III,CAS-V			
48121..52444	array		JXLL01000012.1	Caloranaerobacter sp. TR13	Bacteria	Firmicutes	Clostridia	Clostridiales	Clostridiaceae	Caloranaerobacter		CRISPR array			66	43	23

459 CAS-I-B | CCJ34801.1

11732..13549	array		CAKP01000159.1	Caloramator australicus RC3	Bacteria	Firmicutes	Clostridia	Clostridiales	Clostridiaceae	Caloramator		CRISPR array			28	37	29
12108..12231	+	CCJ34798.1	CAKP01000159.1	Caloramator australicus RC3	Bacteria	Firmicutes	Clostridia	Clostridiales	Clostridiaceae	Caloramator	hypothetical protein						
12760..12877	-	CCJ34799.1	CAKP01000159.1	Caloramator australicus RC3	Bacteria	Firmicutes	Clostridia	Clostridiales	Clostridiaceae	Caloramator	hypothetical protein						
13064..13214	-	CCJ34800.1	CAKP01000159.1	Caloramator australicus RC3	Bacteria	Firmicutes	Clostridia	Clostridiales	Clostridiaceae	Caloramator	hypothetical protein						
13733..13997	-	CCJ34801.1	CAKP01000159.1	Caloramator australicus RC3	Bacteria	Firmicutes	Clostridia	Clostridiales	Clostridiaceae	Caloramator	CRISPR-associated protein Cas2	cas2	cd09725	CAS-I,CAS-II,CAS-III,CAS-V			
13998..14922	-	CCJ34802.1	CAKP01000159.1	Caloramator australicus RC3	Bacteria	Firmicutes	Clostridia	Clostridiales	Clostridiaceae	Caloramator	CRISPR-associated protein Cas1	cas1	cd09722	CAS-I-B			
14981..15500	-	CCJ34803.1	CAKP01000159.1	Caloramator australicus RC3	Bacteria	Firmicutes	Clostridia	Clostridiales	Clostridiaceae	Caloramator	CRISPR-associated RecB family exonuclease Cas4a	cas4	pfam01930	CAS-I			
15510..17664	-	CCJ34804.1	CAKP01000159.1	Caloramator australicus RC3	Bacteria	Firmicutes	Clostridia	Clostridiales	Clostridiaceae	Caloramator	CRISPR-associated helicase Cas3	cas3HD,cas3	cd09641,cd09639	CAS-I			
17660..18335	-	CCJ34805.1	CAKP01000159.1	Caloramator australicus RC3	Bacteria	Firmicutes	Clostridia	Clostridiales	Clostridiaceae	Caloramator	hypothetical protein	cas5	cd09658	CAS-I-B			
18339..19233	-	CCJ34806.1	CAKP01000159.1	Caloramator australicus RC3	Bacteria	Firmicutes	Clostridia	Clostridiales	Clostridiaceae	Caloramator	CRISPR-associated negative autoregulator	cas7	cd09687	CAS-I-B			
19225..20410	-	CCJ34807.1	CAKP01000159.1	Caloramator australicus RC3	Bacteria	Firmicutes	Clostridia	Clostridiales	Clostridiaceae	Caloramator	hypothetical protein	cas8b2	cd09665	CAS-I-B			
20438..20972	-	CCJ34808.1	CAKP01000159.1	Caloramator australicus RC3	Bacteria	Firmicutes	Clostridia	Clostridiales	Clostridiaceae	Caloramator	hypothetical protein						
20972..21644	-	CCJ34809.1	CAKP01000159.1	Caloramator australicus RC3	Bacteria	Firmicutes	Clostridia	Clostridiales	Clostridiaceae	Caloramator	hypothetical protein	cas6	pfam01881	CAS-I,CAS-III			

460 CAS-I-B | KRQ85886.1

173..1580	array		LKHP01000021.1	Caloramator mitchellensis	Bacteria	Firmicutes	Clostridia	Clostridiales	Clostridiaceae	Caloramator		CRISPR array			22	36	29
1766..2030	-	KRQ85886.1	LKHP01000021.1	Caloramator mitchellensis	Bacteria	Firmicutes	Clostridia	Clostridiales	Clostridiaceae	Caloramator	CRISPR-associated endoribonuclease Cas2	cas2	cd09725	CAS-I,CAS-II,CAS-III,CAS-V			
2031..3012	-	KRQ85887.1	LKHP01000021.1	Caloramator mitchellensis	Bacteria	Firmicutes	Clostridia	Clostridiales	Clostridiaceae	Caloramator	CRISPR-associated endonuclease Cas1	cas1	cd09722	CAS-I-B			
3004..3532	-	KRQ85888.1	LKHP01000021.1	Caloramator mitchellensis	Bacteria	Firmicutes	Clostridia	Clostridiales	Clostridiaceae	Caloramator	PD-(D/E)XK nuclease superfamily protein	cas4	pfam01930	CAS-I			
3543..5685	-	KRQ85889.1	LKHP01000021.1	Caloramator mitchellensis	Bacteria	Firmicutes	Clostridia	Clostridiales	Clostridiaceae	Caloramator	CRISPR-associated nuclease/helicase Cas3	cas3	COG1203	CAS-I			
5681..6374	-	KRQ85890.1	LKHP01000021.1	Caloramator mitchellensis	Bacteria	Firmicutes	Clostridia	Clostridiales	Clostridiaceae	Caloramator	CRISPR-associated protein (Cas_Cas5)	cas5	cd09658	CAS-I-B			
6363..7257	-	KRQ85891.1	LKHP01000021.1	Caloramator mitchellensis	Bacteria	Firmicutes	Clostridia	Clostridiales	Clostridiaceae	Caloramator	hypothetical protein	cas7	cd09687	CAS-I-B			
7249..9019	-	KRQ85892.1	LKHP01000021.1	Caloramator mitchellensis	Bacteria	Firmicutes	Clostridia	Clostridiales	Clostridiaceae	Caloramator	CRISPR-associated protein (Cas_CXXC_CXXC)	cas8b2	cd09665	CAS-I-B			
9019..9745	-	KRQ85893.1	LKHP01000021.1	Caloramator mitchellensis	Bacteria	Firmicutes	Clostridia	Clostridiales	Clostridiaceae	Caloramator	CRISPR associated protein Cas6	cas6	pfam01881	CAS-I,CAS-III			
9979..10906	-	KRQ85894.1	LKHP01000021.1	Caloramator mitchellensis	Bacteria	Firmicutes	Clostridia	Clostridiales	Clostridiaceae	Caloramator	3'-5' exoribonuclease YhaM	cas3HD	cd09641	CAS-I			

461 CAS-I-B | SDH76309.1

3..226	array		FNDE01000054.1	Aneurinibacillus thermoaerophilus	Bacteria	Firmicutes	Bacilli	Bacillales	Paenibacillaceae	Aneurinibacillus		CRISPR array			4	37	28
1376..2870	-	SDH76173.1	FNDE01000054.1	Aneurinibacillus thermoaerophilus	Bacteria	Firmicutes	Bacilli	Bacillales	Paenibacillaceae	Aneurinibacillus	proton-dependent oligopeptide transporter, POT family						
3859..4585	+	SDH76189.1	FNDE01000054.1	Aneurinibacillus thermoaerophilus	Bacteria	Firmicutes	Bacilli	Bacillales	Paenibacillaceae	Aneurinibacillus	CRISPR-associated endoribonuclease Cas6	cas6	COG1583	CAS-I,CAS-III,CAS-IV			
4592..6419	+	SDH76205.1	FNDE01000054.1	Aneurinibacillus thermoaerophilus	Bacteria	Firmicutes	Bacilli	Bacillales	Paenibacillaceae	Aneurinibacillus	CRISPR-associated protein Cst1	cas8b2,cas8b2	cd09754,cd09665	CAS-I-B			
6415..7306	+	SDH76226.1	FNDE01000054.1	Aneurinibacillus thermoaerophilus	Bacteria	Firmicutes	Bacilli	Bacillales	Paenibacillaceae	Aneurinibacillus	CRISPR-associated protein Cst2	cas7	cd09687	CAS-I-B			
7309..8026	+	SDH76241.1	FNDE01000054.1	Aneurinibacillus thermoaerophilus	Bacteria	Firmicutes	Bacilli	Bacillales	Paenibacillaceae	Aneurinibacillus	CRISPR-associated protein Cas5t	cas5	cd09658	CAS-I-B			
8042..10241	+	SDH76258.1	FNDE01000054.1	Aneurinibacillus thermoaerophilus	Bacteria	Firmicutes	Bacilli	Bacillales	Paenibacillaceae	Aneurinibacillus	CRISPR-associated endonuclease/helicase Cas3	cas3HD,cas3	cd09641,cd09639	CAS-I			
10254..10770	+	SDH76275.1	FNDE01000054.1	Aneurinibacillus thermoaerophilus	Bacteria	Firmicutes	Bacilli	Bacillales	Paenibacillaceae	Aneurinibacillus	CRISPR-associated exonuclease Cas4	cas4	pfam01930	CAS-I			
10763..11747	+	SDH76295.1	FNDE01000054.1	Aneurinibacillus thermoaerophilus	Bacteria	Firmicutes	Bacilli	Bacillales	Paenibacillaceae	Aneurinibacillus	CRISP-associated protein Cas1	cas1	cd09722	CAS-I-B			
11750..12014	+	SDH76309.1	FNDE01000054.1	Aneurinibacillus thermoaerophilus	Bacteria	Firmicutes	Bacilli	Bacillales	Paenibacillaceae	Aneurinibacillus	CRISPR-associated protein Cas2	cas2	cd09725	CAS-I,CAS-II,CAS-III,CAS-V			
12223..12450	array		FNDE01000054.1	Aneurinibacillus thermoaerophilus	Bacteria	Firmicutes	Bacilli	Bacillales	Paenibacillaceae	Aneurinibacillus		CRISPR array			4	37	29
13030..13520	array		FNDE01000054.1	Aneurinibacillus thermoaerophilus	Bacteria	Firmicutes	Bacilli	Bacillales	Paenibacillaceae	Aneurinibacillus		CRISPR array			8	37	29

462 CAS-I-B | SFA87797.1

21417..22134	+	SFA87683.1	FOJW01000003.1	Lentibacillus halodurans	Bacteria	Firmicutes	Bacilli	Bacillales	Bacillaceae	Lentibacillus	CRISPR-associated endoribonuclease Cas6	cas6	COG1583	CAS-I,CAS-III,CAS-IV			
22134..23832	+	SFA87700.1	FOJW01000003.1	Lentibacillus halodurans	Bacteria	Firmicutes	Bacilli	Bacillales	Bacillaceae	Lentibacillus	CRISPR-associated protein Cst1	cas8b2	cd09665	CAS-I-B			
23824..24715	+	SFA87715.1	FOJW01000003.1	Lentibacillus halodurans	Bacteria	Firmicutes	Bacilli	Bacillales	Bacillaceae	Lentibacillus	CRISPR-associated protein Cst2	cas7	cd09687	CAS-I-B			
24711..25422	+	SFA87732.1	FOJW01000003.1	Lentibacillus halodurans	Bacteria	Firmicutes	Bacilli	Bacillales	Bacillaceae	Lentibacillus	CRISPR-associated protein Cas5t	cas5	cd09658	CAS-I-B			
25478..27704	+	SFA87748.1	FOJW01000003.1	Lentibacillus halodurans	Bacteria	Firmicutes	Bacilli	Bacillales	Bacillaceae	Lentibacillus	CRISPR-associated endonuclease/helicase Cas3	cas3HD,cas3	cd09641,cd09639	CAS-I			
27718..28213	+	SFA87766.1	FOJW01000003.1	Lentibacillus halodurans	Bacteria	Firmicutes	Bacilli	Bacillales	Bacillaceae	Lentibacillus	CRISPR-associated exonuclease, Cas4 family	cas4	pfam01930	CAS-I			
28213..29203	+	SFA87783.1	FOJW01000003.1	Lentibacillus halodurans	Bacteria	Firmicutes	Bacilli	Bacillales	Bacillaceae	Lentibacillus	CRISP-associated protein Cas1	cas1	cd09722	CAS-I-B			
29205..29469	+	SFA87797.1	FOJW01000003.1	Lentibacillus halodurans	Bacteria	Firmicutes	Bacilli	Bacillales	Bacillaceae	Lentibacillus	CRISPR-associated protein Cas2	cas2	cd09725	CAS-I,CAS-II,CAS-III,CAS-V			
29649..29938	array		FOJW01000003.1	Lentibacillus halodurans	Bacteria	Firmicutes	Bacilli	Bacillales	Bacillaceae	Lentibacillus		CRISPR array			5	36	29
30303..32153	array		FOJW01000003.1	Lentibacillus halodurans	Bacteria	Firmicutes	Bacilli	Bacillales	Bacillaceae	Lentibacillus		CRISPR array			29	36	29
32950..33876	array		FOJW01000003.1	Lentibacillus halodurans	Bacteria	Firmicutes	Bacilli	Bacillales	Bacillaceae	Lentibacillus		CRISPR array			15	35	29

463 CAS-I-B | OUM91842.1

2475..3207	+	OUM91836.1	LZRS01000080.1	Parageobacillus thermoglucosidasius	Bacteria	Firmicutes	Bacilli	Bacillales	Bacillaceae	Parageobacillus	CRISPR-associated endoribonuclease Cas6	cas6	COG1583	CAS-I,CAS-III,CAS-IV			
3194..4892	+	OUM91837.1	LZRS01000080.1	Parageobacillus thermoglucosidasius	Bacteria	Firmicutes	Bacilli	Bacillales	Bacillaceae	Parageobacillus	hypothetical protein	cas8b2,cas8b2	cd09754,cd09665	CAS-I-B			
4884..5781	+	OUM91838.1	LZRS01000080.1	Parageobacillus thermoglucosidasius	Bacteria	Firmicutes	Bacilli	Bacillales	Bacillaceae	Parageobacillus	type I-B CRISPR-associated protein Cas7/Cst2/DevR	cas7	cd09687	CAS-I-B			
5784..6504	+	OUM91839.1	LZRS01000080.1	Parageobacillus thermoglucosidasius	Bacteria	Firmicutes	Bacilli	Bacillales	Bacillaceae	Parageobacillus	type I-B CRISPR-associated protein Cas5	cas5	cd09658	CAS-I-B			
6799..9025	+	OUM91851.1	LZRS01000080.1	Parageobacillus thermoglucosidasius	Bacteria	Firmicutes	Bacilli	Bacillales	Bacillaceae	Parageobacillus	hypothetical protein	cas3HD,cas3	cd09641,COG1203	CAS-I			
9024..9525	+	OUM91840.1	LZRS01000080.1	Parageobacillus thermoglucosidasius	Bacteria	Firmicutes	Bacilli	Bacillales	Bacillaceae	Parageobacillus	CRISPR-associated protein Cas4	cas4	pfam01930	CAS-I			
9526..10519	+	OUM91841.1	LZRS01000080.1	Parageobacillus thermoglucosidasius	Bacteria	Firmicutes	Bacilli	Bacillales	Bacillaceae	Parageobacillus	subtype I-B CRISPR-associated endonuclease Cas1	cas1	cd09722	CAS-I-B			
10649..10913	+	OUM91842.1	LZRS01000080.1	Parageobacillus thermoglucosidasius	Bacteria	Firmicutes	Bacilli	Bacillales	Bacillaceae	Parageobacillus	CRISPR-associated endonuclease Cas2	cas2	cd09725	CAS-I,CAS-II,CAS-III,CAS-V			
11088..15860	array		LZRS01000080.1	Parageobacillus thermoglucosidasius	Bacteria	Firmicutes	Bacilli	Bacillales	Bacillaceae	Parageobacillus		CRISPR array			74	35	29

466 CAS-I-B | KFN04669.1

56756..57632	array		JMQC01000007.1	Bacillus mycoides	Bacteria	Firmicutes	Bacilli	Bacillales	Bacillaceae	Bacillus		CRISPR array			14	36	29
57817..58744	+	KFN04683.1	JMQC01000007.1	Bacillus mycoides	Bacteria	Firmicutes	Bacilli	Bacillales	Bacillaceae	Bacillus	transcriptional regulator family protein	WYL	pfam13280	CAS-I,CAS-III			
58915..59617	+	KFN04660.1	JMQC01000007.1	Bacillus mycoides	Bacteria	Firmicutes	Bacilli	Bacillales	Bacillaceae	Bacillus	CRISPR-associated endoribonuclease Cas6	cas6	pfam01881	CAS-I,CAS-III			
59616..61368	+	KFN04732.1	JMQC01000007.1	Bacillus mycoides	Bacteria	Firmicutes	Bacilli	Bacillales	Bacillaceae	Bacillus	putative cRISPR-associated CXXC CXXC protein Cst1	cas8b2	cd09665	CAS-I-B			
61357..62251	+	KFN04735.1	JMQC01000007.1	Bacillus mycoides	Bacteria	Firmicutes	Bacilli	Bacillales	Bacillaceae	Bacillus	CRISPR-associated autoregulator DevR family protein	cas7	cd09687	CAS-I-B			
62247..62946	+	KFN04680.1	JMQC01000007.1	Bacillus mycoides	Bacteria	Firmicutes	Bacilli	Bacillales	Bacillaceae	Bacillus	CRISPR-associated Cas5 domain protein	cas5	cd09658	CAS-I-B			
62989..65221	+	KFN04684.1	JMQC01000007.1	Bacillus mycoides	Bacteria	Firmicutes	Bacilli	Bacillales	Bacillaceae	Bacillus	CRISPR-associated endonuclease Cas3-HD	cas3HD,cas3	cd09641,COG1203	CAS-I			
65283..65781	+	KFN04693.1	JMQC01000007.1	Bacillus mycoides	Bacteria	Firmicutes	Bacilli	Bacillales	Bacillaceae	Bacillus	CRISPR-associated protein Cas4	cas4	pfam01930	CAS-I			
65774..66758	+	KFN04691.1	JMQC01000007.1	Bacillus mycoides	Bacteria	Firmicutes	Bacilli	Bacillales	Bacillaceae	Bacillus	CRISPR-associated endonuclease Cas1	cas1	cd09722	CAS-I-B			
66762..67026	+	KFN04669.1	JMQC01000007.1	Bacillus mycoides	Bacteria	Firmicutes	Bacilli	Bacillales	Bacillaceae	Bacillus	CRISPR-associated endoribonuclease Cas2	cas2	cd09725	CAS-I,CAS-II,CAS-III,CAS-V			
67255..68392	array		JMQC01000007.1	Bacillus mycoides	Bacteria	Firmicutes	Bacilli	Bacillales	Bacillaceae	Bacillus		CRISPR array			18	36	29

467 CAS-I-B | OEH93026.1																	
4639..6429	array		MJEH01000018.1	Bacillus solimangrovi	Bacteria	Firmicutes	Bacilli	Bacillales	Bacillaceae	Bacillus		CRISPR array			28	36	29
6731..7472	+	OEH93019.1	MJEH01000018.1	Bacillus solimangrovi	Bacteria	Firmicutes	Bacilli	Bacillales	Bacillaceae	Bacillus	CRISPR-associated endoribonuclease Cas6	cas6	COG1583	CAS-I,CAS-III,CAS-IV			
7464..9276	+	OEH93020.1	MJEH01000018.1	Bacillus solimangrovi	Bacteria	Firmicutes	Bacilli	Bacillales	Bacillaceae	Bacillus	hypothetical protein	cas8b2	cd09665	CAS-I-B			
9275..10172	+	OEH93021.1	MJEH01000018.1	Bacillus solimangrovi	Bacteria	Firmicutes	Bacilli	Bacillales	Bacillaceae	Bacillus	type I-B CRISPR-associated protein Cas7/Cst2/DevR	cas7	cd09687	CAS-I-B			
10168..10870	+	OEH93022.1	MJEH01000018.1	Bacillus solimangrovi	Bacteria	Firmicutes	Bacilli	Bacillales	Bacillaceae	Bacillus	type I-B CRISPR-associated protein Cas5	cas5	cd09658	CAS-I-B			
10927..13147	+	OEH93023.1	MJEH01000018.1	Bacillus solimangrovi	Bacteria	Firmicutes	Bacilli	Bacillales	Bacillaceae	Bacillus	hypothetical protein	cas3HD,cas3	cd09641,COG1203	CAS-I			
13162..13681	+	OEH93024.1	MJEH01000018.1	Bacillus solimangrovi	Bacteria	Firmicutes	Bacilli	Bacillales	Bacillaceae	Bacillus	CRISPR-associated protein Cas4	cas4	pfam01930	CAS-I			
13670..14657	+	OEH93025.1	MJEH01000018.1	Bacillus solimangrovi	Bacteria	Firmicutes	Bacilli	Bacillales	Bacillaceae	Bacillus	subtype I-B CRISPR-associated endonuclease Cas1	cas1	cd09722	CAS-I-B			
14725..14989	+	OEH93026.1	MJEH01000018.1	Bacillus solimangrovi	Bacteria	Firmicutes	Bacilli	Bacillales	Bacillaceae	Bacillus	CRISPR-associated endonuclease Cas2	cas2	cd09725	CAS-I,CAS-II,CAS-III,CAS-V			
15220..17473	array		MJEH01000018.1	Bacillus solimangrovi	Bacteria	Firmicutes	Bacilli	Bacillales	Bacillaceae	Bacillus		CRISPR array			35	36	29
468 CAS-I/CAS-III-B | PAE89157.1																	
131562..132513	-	PAE89147.1	NPCC01000011.1	Bacillus clausii	Bacteria	Firmicutes	Bacilli	Bacillales	Bacillaceae	Bacillus	hypothetical protein	PD-DExK	mkCas0090	CAS-I,CAS-III			
132548..133382	-	PAE89148.1	NPCC01000011.1	Bacillus clausii	Bacteria	Firmicutes	Bacilli	Bacillales	Bacillaceae	Bacillus	type III-B CRISPR module RAMP protein Cmr6	cmr6gr7	COG1604	CAS-III-B			
133400..133790	-	PAE89149.1	NPCC01000011.1	Bacillus clausii	Bacteria	Firmicutes	Bacilli	Bacillales	Bacillaceae	Bacillus	type III-B CRISPR module-associated protein Cmr5	cmr5gr11	COG3337	CAS-III-B			
133786..134743	-	PAE89150.1	NPCC01000011.1	Bacillus clausii	Bacteria	Firmicutes	Bacilli	Bacillales	Bacillaceae	Bacillus	type III-B CRISPR module RAMP protein Cmr4	cmr4gr7	COG1336	CAS-III-C			
134765..136103	-	PAE89151.1	NPCC01000011.1	Bacillus clausii	Bacteria	Firmicutes	Bacilli	Bacillales	Bacillaceae	Bacillus	type III-B CRISPR module-associated protein Cmr3	cmr3gr5	pfam09700	CAS-III-B			
136092..137958	-	PAE89152.1	NPCC01000011.1	Bacillus clausii	Bacteria	Firmicutes	Bacilli	Bacillales	Bacillaceae	Bacillus	type III-B CRISPR-associated protein Cas10/Cmr2	cas10	cls000742	CAS-III			
137903..139292	-	PAE89153.1	NPCC01000011.1	Bacillus clausii	Bacteria	Firmicutes	Bacilli	Bacillales	Bacillaceae	Bacillus	type III-B CRISPR module RAMP protein Cmr1	cmr1gr7	COG1367	CAS-III-B			
140271..140569	array		NPCC01000011.1	Bacillus clausii	Bacteria	Firmicutes	Bacilli	Bacillales	Bacillaceae	Bacillus		CRISPR array			5	34	32
140964..142524	-	PAE89154.1	NPCC01000011.1	Bacillus clausii	Bacteria	Firmicutes	Bacilli	Bacillales	Bacillaceae	Bacillus	choline transporter						
142904..143261	-	PAE89155.1	NPCC01000011.1	Bacillus clausii	Bacteria	Firmicutes	Bacilli	Bacillales	Bacillaceae	Bacillus	hypothetical protein						
143751..144222	+	PAE89156.1	NPCC01000011.1	Bacillus clausii	Bacteria	Firmicutes	Bacilli	Bacillales	Bacillaceae	Bacillus	hypothetical protein						
144453..145791	array		NPCC01000011.1	Bacillus clausii	Bacteria	Firmicutes	Bacilli	Bacillales	Bacillaceae	Bacillus		CRISPR array			21	37	28
146001..146265	-	PAE89157.1	NPCC01000011.1	Bacillus clausii	Bacteria	Firmicutes	Bacilli	Bacillales	Bacillaceae	Bacillus	CRISPR-associated endonuclease Cas2	cas2	cd09725	CAS-I,CAS-II,CAS-III,CAS-V			
147237..147756	-	PAE89158.1	NPCC01000011.1	Bacillus clausii	Bacteria	Firmicutes	Bacilli	Bacillales	Bacillaceae	Bacillus	CRISPR-associated protein Cas4	cas4	pfam01930	CAS-I			
147775..150091	-	PAE89159.1	NPCC01000011.1	Bacillus clausii	Bacteria	Firmicutes	Bacilli	Bacillales	Bacillaceae	Bacillus	hypothetical protein	cas3HD,cas3,cas3	cd09641,cd09639,COG1203	CAS-I			
150149..150839	-	PAE89160.1	NPCC01000011.1	Bacillus clausii	Bacteria	Firmicutes	Bacilli	Bacillales	Bacillaceae	Bacillus	type I-B CRISPR-associated protein Cas5	cas5	cd09658	CAS-I-B			
150838..151732	-	PAE89161.1	NPCC01000011.1	Bacillus clausii	Bacteria	Firmicutes	Bacilli	Bacillales	Bacillaceae	Bacillus	type I-B CRISPR-associated protein Cas7/Cst2/DevR	cas7	cd09687	CAS-I-B			
151724..153524	-	PAE89162.1	NPCC01000011.1	Bacillus clausii	Bacteria	Firmicutes	Bacilli	Bacillales	Bacillaceae	Bacillus	hypothetical protein	cas8b2	cd09665	CAS-I-B			
153520..154255	-	PAE89163.1	NPCC01000011.1	Bacillus clausii	Bacteria	Firmicutes	Bacilli	Bacillales	Bacillaceae	Bacillus	CRISPR-associated endoribonuclease Cas6	cas6	COG1583	CAS-I,CAS-III,CAS-IV			
154704..155890	array		NPCC01000011.1	Bacillus clausii	Bacteria	Firmicutes	Bacilli	Bacillales	Bacillaceae	Bacillus		CRISPR array			19	35	29
156027..157053	+	PAE89164.1	NPCC01000011.1	Bacillus clausii	Bacteria	Firmicutes	Bacilli	Bacillales	Bacillaceae	Bacillus	hypothetical protein	WYL	COG2378	CAS-I,CAS-III			
469 CAS-I-B | OEH84878.1																	
40602..40866	+	OEH84878.1	MJAT01000035.1	Desulfuribacillus stibiiarsenatis	Bacteria	Firmicutes	Bacilli	Bacillales		Desulfuribacillus	CRISPR-associated endonuclease Cas2	cas2	cd09725	CAS-I,CAS-II,CAS-III,CAS-V			
41198..41921	+	OEH84879.1	MJAT01000035.1	Desulfuribacillus stibiiarsenatis	Bacteria	Firmicutes	Bacilli	Bacillales		Desulfuribacillus	CRISPR-associated endoribonuclease Cas6	cas6	pfam01881	CAS-I,CAS-III			
41923..43720	+	OEH84880.1	MJAT01000035.1	Desulfuribacillus stibiiarsenatis	Bacteria	Firmicutes	Bacilli	Bacillales		Desulfuribacillus	hypothetical protein	cas8b2	cd09665	CAS-I-B			
43712..44606	+	OEH84881.1	MJAT01000035.1	Desulfuribacillus stibiiarsenatis	Bacteria	Firmicutes	Bacilli	Bacillales		Desulfuribacillus	type I-B CRISPR-associated protein Cas7/Cst2/DevR	cas7	cd09687	CAS-I-B			
44605..45304	+	OEH84882.1	MJAT01000035.1	Desulfuribacillus stibiiarsenatis	Bacteria	Firmicutes	Bacilli	Bacillales		Desulfuribacillus	type I-B CRISPR-associated protein Cas5	cas5	cd09658	CAS-I-B			
45347..47579	+	OEH84883.1	MJAT01000035.1	Desulfuribacillus stibiiarsenatis	Bacteria	Firmicutes	Bacilli	Bacillales		Desulfuribacillus	hypothetical protein	cas3HD,cas3	cd09641,COG1203	CAS-I			
47592..48099	+	OEH84884.1	MJAT01000035.1	Desulfuribacillus stibiiarsenatis	Bacteria	Firmicutes	Bacilli	Bacillales		Desulfuribacillus	CRISPR-associated protein Cas4	cas4	pfam01930	CAS-I			
48101..49088	+	OEH84885.1	MJAT01000035.1	Desulfuribacillus stibiiarsenatis	Bacteria	Firmicutes	Bacilli	Bacillales		Desulfuribacillus	subtype I-B CRISPR-associated endonuclease Cas1	cas1	cd09722	CAS-I-B			
49335..53060	array		MJAT01000035.1	Desulfuribacillus stibiiarsenatis	Bacteria	Firmicutes	Bacilli	Bacillales		Desulfuribacillus		CRISPR array			58	35	29
470 CAS-I-B | SIT17613.1																	
133..772	array		FTOD01000018.1	Kroppenstedtia eburnea	Bacteria	Firmicutes	Bacilli	Bacillales	Thermoactinomycetaceae	Kroppenstedtia		CRISPR array			10	38	29
962..1226	-	SIT17613.1	FTOD01000018.1	Kroppenstedtia eburnea	Bacteria	Firmicutes	Bacilli	Bacillales	Thermoactinomycetaceae	Kroppenstedtia	CRISPR-associated protein Cas2	cas2	cd09725	CAS-I,CAS-II,CAS-III,CAS-V			
1306..2287	-	SIT17625.1	FTOD01000018.1	Kroppenstedtia eburnea	Bacteria	Firmicutes	Bacilli	Bacillales	Thermoactinomycetaceae	Kroppenstedtia	CRISP-associated protein Cas1	cas1	cd09722	CAS-I-B			
2283..2793	-	SIT17637.1	FTOD01000018.1	Kroppenstedtia eburnea	Bacteria	Firmicutes	Bacilli	Bacillales	Thermoactinomycetaceae	Kroppenstedtia	CRISPR-associated exonuclease Cas4	cas4	pfam01930	CAS-I			
2840..5072	-	SIT17650.1	FTOD01000018.1	Kroppenstedtia eburnea	Bacteria	Firmicutes	Bacilli	Bacillales	Thermoactinomycetaceae	Kroppenstedtia	CRISPR-associated endonuclease/helicase Cas3	cas3HD,cas3	cd09641,COG1203	CAS-I			
5159..5858	-	SIT17665.1	FTOD01000018.1	Kroppenstedtia eburnea	Bacteria	Firmicutes	Bacilli	Bacillales	Thermoactinomycetaceae	Kroppenstedtia	CRISPR-associated protein Cas5t	cas5	cd09658	CAS-I-B			
5854..6754	-	SIT17680.1	FTOD01000018.1	Kroppenstedtia eburnea	Bacteria	Firmicutes	Bacilli	Bacillales	Thermoactinomycetaceae	Kroppenstedtia	CRISPR-associated protein Cst2	cas7	cd09687	CAS-I-B			
6746..8621	-	SIT17694.1	FTOD01000018.1	Kroppenstedtia eburnea	Bacteria	Firmicutes	Bacilli	Bacillales	Thermoactinomycetaceae	Kroppenstedtia	CRISPR-associated protein Cst1	cas8b2	cd09665	CAS-I-B			
8649..9402	-	SIT17708.1	FTOD01000018.1	Kroppenstedtia eburnea	Bacteria	Firmicutes	Bacilli	Bacillales	Thermoactinomycetaceae	Kroppenstedtia	CRISPR-associated endoribonuclease Cas6	cas6	cd09652	CAS-I,CAS-III			
471 CAS-I-B | OYD07557.1																	
139434..140466	array		NOWF01000006.1	Paludifilum halophilum	Bacteria	Firmicutes	Bacilli	Bacillales	Thermoactinomycetaceae	Paludifilum		CRISPR array			16	37	29
140667..140931	-	OYD07557.1	NOWF01000006.1	Paludifilum halophilum	Bacteria	Firmicutes	Bacilli	Bacillales	Thermoactinomycetaceae	Paludifilum	CRISPR-associated endonuclease Cas2	cas2	cd09725	CAS-I,CAS-II,CAS-III,CAS-V			
140972..141953	-	OYD07425.1	NOWF01000006.1	Paludifilum halophilum	Bacteria	Firmicutes	Bacilli	Bacillales	Thermoactinomycetaceae	Paludifilum	subtype I-B CRISPR-associated endonuclease Cas1	cas1	cd09722	CAS-I-B			
141949..142459	-	OYD07558.1	NOWF01000006.1	Paludifilum halophilum	Bacteria	Firmicutes	Bacilli	Bacillales	Thermoactinomycetaceae	Paludifilum	CRISPR-associated protein Cas4	cas4	pfam01930	CAS-I			
142485..144720	-	OYD07559.1	NOWF01000006.1	Paludifilum halophilum	Bacteria	Firmicutes	Bacilli	Bacillales	Thermoactinomycetaceae	Paludifilum	CRISPR-associated helicase/endonuclease Cas3	cas3HD,cas3	cd09641,COG1203	CAS-I			
144806..145514	-	OYD07426.1	NOWF01000006.1	Paludifilum halophilum	Bacteria	Firmicutes	Bacilli	Bacillales	Thermoactinomycetaceae	Paludifilum	type I-B CRISPR-associated protein Cas5	cas5	cd09658	CAS-I-B			
145510..146410	-	OYD07427.1	NOWF01000006.1	Paludifilum halophilum	Bacteria	Firmicutes	Bacilli	Bacillales	Thermoactinomycetaceae	Paludifilum	type I-B CRISPR-associated protein Cas7/Cst2/DevR	cas7	cd09687	CAS-I-B			
146402..148277	-	OYD07428.1	NOWF01000006.1	Paludifilum halophilum	Bacteria	Firmicutes	Bacilli	Bacillales	Thermoactinomycetaceae	Paludifilum	hypothetical protein	cas8b2	cd09665	CAS-I-B			
148302..149058	-	OYD07429.1	NOWF01000006.1	Paludifilum halophilum	Bacteria	Firmicutes	Bacilli	Bacillales	Thermoactinomycetaceae	Paludifilum	CRISPR-associated endoribonuclease Cas6	cas6	COG1583	CAS-I,CAS-III,CAS-IV			
473 CAS-I-B | OCS84763.1																	
186497..187214	+	OCS84756.1	MASJ01000023.1	Caryophanon tenue	Bacteria	Firmicutes	Bacilli	Bacillales	Planococcaceae	Caryophanon	CRISPR-associated endoribonuclease Cas6	cas6	COG1583	CAS-I,CAS-III,CAS-IV			
187206..188976	+	OCS84757.1	MASJ01000023.1	Caryophanon tenue	Bacteria	Firmicutes	Bacilli	Bacillales	Planococcaceae	Caryophanon	hypothetical protein	cas8b2	cd09665	CAS-I-B			
188972..189872	+	OCS84758.1	MASJ01000023.1	Caryophanon tenue	Bacteria	Firmicutes	Bacilli	Bacillales	Planococcaceae	Caryophanon	type I-B CRISPR-associated protein Cas7/Cst2/DevR	cas7	cd09687	CAS-I-B			
189852..190560	+	OCS84759.1	MASJ01000023.1	Caryophanon tenue	Bacteria	Firmicutes	Bacilli	Bacillales	Planococcaceae	Caryophanon	type I-B CRISPR-associated protein Cas5	cas5	cd09658	CAS-I-B			
190567..192883	+	OCS84760.1	MASJ01000023.1	Caryophanon tenue	Bacteria	Firmicutes	Bacilli	Bacillales	Planococcaceae	Caryophanon	hypothetical protein	cas3HD,cas3	cd09641,cd09639	CAS-I			
192895..193402	+	OCS84761.1	MASJ01000023.1	Caryophanon tenue	Bacteria	Firmicutes	Bacilli	Bacillales	Planococcaceae	Caryophanon	CRISPR-associated protein Cas4	cas4	pfam01930	CAS-I			
193391..194378	+	OCS84762.1	MASJ01000023.1	Caryophanon tenue	Bacteria	Firmicutes	Bacilli	Bacillales	Planococcaceae	Caryophanon	subtype I-B CRISPR-associated endonuclease Cas1	cas1	cd09722	CAS-I-B			
194383..194647	+	OCS84763.1	MASJ01000023.1	Caryophanon tenue	Bacteria	Firmicutes	Bacilli	Bacillales	Planococcaceae	Caryophanon	CRISPR-associated endonuclease Cas2	cas2	cd09725	CAS-I,CAS-II,CAS-III,CAS-V			
194798..195738	array		MASJ01000023.1	Caryophanon tenue	Bacteria	Firmicutes	Bacilli	Bacillales	Planococcaceae	Caryophanon		CRISPR array			15	36	29
474 CAS-I-B | KGR92399.1																	
159967..160699	+	KGR92393.1	JPVQ01000001.1	Lysinibacillus massiliensis 4400831 = CIP 108448 = CCUG 49529	Bacteria	Firmicutes	Bacilli	Bacillales	Bacillaceae	Lysinibacillus	hypothetical protein	cas6	COG1583	CAS-I,CAS-III,CAS-IV			
160691..162464	+	KGR92394.1	JPVQ01000001.1	Lysinibacillus massiliensis 4400831 = CIP 108448 = CCUG 49529	Bacteria	Firmicutes	Bacilli	Bacillales	Bacillaceae	Lysinibacillus	hypothetical protein	Cas11b	icity0129				
162480..163386	+	KGR92395.1	JPVQ01000001.1	Lysinibacillus massiliensis 4400831 = CIP 108448 = CCUG 49529	Bacteria	Firmicutes	Bacilli	Bacillales	Bacillaceae	Lysinibacillus	CRISPR-associated protein DevR	cas7	cd09687	CAS-I-B			
163366..164074	+	KGR92396.1	JPVQ01000001.1	Lysinibacillus massiliensis 4400831 = CIP 108448 = CCUG 49529	Bacteria	Firmicutes	Bacilli	Bacillales	Bacillaceae	Lysinibacillus	hypothetical protein	cas5	cd09658	CAS-I-B			
164083..166393	+	KGR92397.1	JPVQ01000001.1	Lysinibacillus massiliensis 4400831 = CIP 108448 = CCUG 49529	Bacteria	Firmicutes	Bacilli	Bacillales	Bacillaceae	Lysinibacillus	hypothetical protein	cas3HD,cas3	cd09641,COG1203	CAS-I			
166911..167895	+	KGR92398.1	JPVQ01000001.1	Lysinibacillus massiliensis 4400831 = CIP 108448 = CCUG 49529	Bacteria	Firmicutes	Bacilli	Bacillales	Bacillaceae	Lysinibacillus	CRISPR-associated protein Cas1	cas1	cd09722	CAS-I-B			
167896..168160	+	KGR92399.1	JPVQ01000001.1	Lysinibacillus massiliensis 4400831 = CIP 108448 = CCUG 49529	Bacteria	Firmicutes	Bacilli	Bacillales	Bacillaceae	Lysinibacillus	CRISPR-associated protein Cas2	cas2	cd09725	CAS-I,CAS-II,CAS-III,CAS-V			
168313..170226	array		JPVQ01000001.1	Lysinibacillus massiliensis 4400831 = CIP 108448 = CCUG 49529	Bacteria	Firmicutes	Bacilli	Bacillales	Bacillaceae	Lysinibacillus		CRISPR array			30	36	29
476 CAS-I-B | KYD09963.1																	
93..253	array		LQYN01000019.1	Bacillus sporothermodurans	Bacteria	Firmicutes	Bacilli	Bacillales	Bacillaceae	Bacillus		CRISPR array			3	37	29
262..1988	array		LQYN01000019.1	Bacillus sporothermodurans	Bacteria	Firmicutes	Bacilli	Bacillales	Bacillaceae	Bacillus		CRISPR array			27	36	29
2206..2470	-	KYD09963.1	LQYN01000019.1	Bacillus sporothermodurans	Bacteria	Firmicutes	Bacilli	Bacillales	Bacillaceae	Bacillus	hypothetical protein	cas2	cd09725	CAS-I,CAS-II,CAS-III,CAS-V			
2471..3455	-	KYD09964.1	LQYN01000019.1	Bacillus sporothermodurans	Bacteria	Firmicutes	Bacilli	Bacillales	Bacillaceae	Bacillus	hypothetical protein	cas1	cd09722	CAS-I-B			
3454..3952	-	KYD09965.1	LQYN01000019.1	Bacillus sporothermodurans	Bacteria	Firmicutes	Bacilli	Bacillales	Bacillaceae	Bacillus	hypothetical protein	cas4	pfam01930	CAS-I			
4140..6333	-	KYD09966.1	LQYN01000019.1	Bacillus sporothermodurans	Bacteria	Firmicutes	Bacilli	Bacillales	Bacillaceae	Bacillus	hypothetical protein	cas3HD,cas3	cd09641,COG1203	CAS-I			
6373..7093	-	KYD09967.1	LQYN01000019.1	Bacillus sporothermodurans	Bacteria	Firmicutes	Bacilli	Bacillales	Bacillaceae	Bacillus	hypothetical protein	cas5	cd09658	CAS-I-B			
7092..7983	-	KYD09968.1	LQYN01000019.1	Bacillus sporothermodurans	Bacteria	Firmicutes	Bacilli	Bacillales	Bacillaceae	Bacillus	hypothetical protein	cas7	cd09687	CAS-I-B			
7987..9781	-	KYD09969.1	LQYN01000019.1	Bacillus sporothermodurans	Bacteria	Firmicutes	Bacilli	Bacillales	Bacillaceae	Bacillus	hypothetical protein	cas8b2	cd09665	CAS-I-B			
9782..10514	-	KYD09970.1	LQYN01000019.1	Bacillus sporothermodurans	Bacteria	Firmicutes	Bacilli	Bacillales	Bacillaceae	Bacillus	hypothetical protein	cas6	COG1583	CAS-I,CAS-III,CAS-IV			
477 CAS-I-B | PTY77974.1																	
37014..37746	+	PTY77967.1	NAZB01000026.1	Bacillus sporothermodurans	Bacteria	Firmicutes	Bacilli	Bacillales	Bacillaceae	Bacillus	CRISPR-associated endoribonuclease Cas6	cas6	COG1583	CAS-I,CAS-III,CAS-IV			
37747..39541	+	PTY77968.1	NAZB01000026.1	Bacillus sporothermodurans	Bacteria	Firmicutes	Bacilli	Bacillales	Bacillaceae	Bacillus	type I-B CRISPR-associated protein Cas8b1/Cst1	cas8b2	cd09665	CAS-I-B			
39545..40436	+	PTY77969.1	NAZB01000026.1	Bacillus sporothermodurans	Bacteria	Firmicutes	Bacilli	Bacillales	Bacillaceae	Bacillus	type I-B CRISPR-associated protein Cas7/Cst2/DevR	cas7	cd09687	CAS-I-B			
40435..41155	+	PTY77970.1	NAZB01000026.1	Bacillus sporothermodurans	Bacteria	Firmicutes	Bacilli	Bacillales	Bacillaceae	Bacillus	type I-B CRISPR-associated protein Cas5	cas5	cd09658	CAS-I-B			
41195..43388	+	PTY77971.1	NAZB01000026.1	Bacillus sporothermodurans	Bacteria	Firmicutes	Bacilli	Bacillales	Bacillaceae	Bacillus	CRISPR-associated helicase/endonuclease Cas3	cas3HD,cas3	cd09641,COG1203	CAS-I			
43576..44074	+	PTY77972.1	NAZB01000026.1	Bacillus sporothermodurans	Bacteria	Firmicutes	Bacilli	Bacillales	Bacillaceae	Bacillus	CRISPR-associated protein Cas4	cas4	pfam01930	CAS-I			
44073..45057	+	PTY77973.1	NAZB01000026.1	Bacillus sporothermodurans	Bacteria	Firmicutes	Bacilli	Bacillales	Bacillaceae	Bacillus	subtype I-B CRISPR-associated endonuclease Cas1	cas1	cd09722	CAS-I-B			
45058..45322	+	PTY77974.1	NAZB01000026.1	Bacillus sporothermodurans	Bacteria	Firmicutes	Bacilli	Bacillales	Bacillaceae	Bacillus	CRISPR-associated endonuclease Cas2	cas2	cd09725	CAS-I,CAS-II,CAS-III,CAS-V			
45541..46740	array		NAZB01000026.1	Bacillus sporothermodurans	Bacteria	Firmicutes	Bacilli	Bacillales	Bacillaceae	Bacillus		CRISPR array			19	36	29
478 CAS-I-B | SMG36678.1																	
362741..364946	array		FXAZ01000002.1	Paenibacillus aquistagni	Bacteria	Firmicutes	Bacilli	Bacillales	Paenibacillaceae	Paenibacillus		CRISPR array			34	36	29
364852..365029	-	SMG36671.1	FXAZ01000002.1	Paenibacillus aquistagni	Bacteria	Firmicutes	Bacilli	Bacillales	Paenibacillaceae	Paenibacillus	hypothetical protein						
365131..365392	-	SMG36678.1	FXAZ01000002.1	Paenibacillus aquistagni	Bacteria	Firmicutes	Bacilli	Bacillales	Paenibacillaceae	Paenibacillus	CRISPR-associated protein Cas2	cas2	cd09725	CAS-I,CAS-II,CAS-III,CAS-V			
365393..366380	-	SMG36685.1	FXAZ01000002.1	Paenibacillus aquistagni	Bacteria	Firmicutes	Bacilli	Bacillales	Paenibacillaceae	Paenibacillus	CRISP-associated protein Cas1	cas1	cd09722	CAS-I-B			
366376..366865	-	SMG36690.1	FXAZ01000002.1	Paenibacillus aquistagni	Bacteria	Firmicutes	Bacilli	Bacillales	Paenibacillaceae	Paenibacillus	CRISPR-associated exonuclease Cas4	cas4	pfam01930	CAS-I			
366902..369155	-	SMG36702.1	FXAZ01000002.1	Paenibacillus aquistagni	Bacteria	Firmicutes	Bacilli	Bacillales	Paenibacillaceae	Paenibacillus	CRISPR-associated endonuclease/helicase Cas3	cas3HD,cas3	cd09641,COG1203	CAS-I			
369229..369925	-	SMG36710.1	FXAZ01000002.1	Paenibacillus aquistagni	Bacteria	Firmicutes	Bacilli	Bacillales	Paenibacillaceae	Paenibacillus	CRISPR-associated protein Cas5t	cas5	cd09658	CAS-I-B			
369924..370821	-	SMG36769.1	FXAZ01000002.1	Paenibacillus aquistagni	Bacteria	Firmicutes	Bacilli	Bacillales	Paenibacillaceae	Paenibacillus	CRISPR-associated protein Cst2	cas7	cd09687	CAS-I-B			
370810..372631	-	SMG36792.1	FXAZ01000002.1	Paenibacillus aquistagni	Bacteria	Firmicutes	Bacilli	Bacillales	Paenibacillaceae	Paenibacillus	CRISPR-associated protein Cst1	cas8b2	cd09665	CAS-I-B			
372633..373350	-	SMG36807.1	FXAZ01000002.1	Paenibacillus aquistagni	Bacteria	Firmicutes	Bacilli	Bacillales	Paenibacillaceae	Paenibacillus	CRISPR-associated endoribonuclease Cas6	cas6	COG1583	CAS-I,CAS-III,CAS-IV			
373781..374144	-	SMG36823.1	FXAZ01000002.1	Paenibacillus aquistagni	Bacteria	Firmicutes	Bacilli	Bacillales	Paenibacillaceae	Paenibacillus	Protein of unknown function						
374288..375656	-	SMG36829.1	FXAZ01000002.1	Paenibacillus aquistagni	Bacteria	Firmicutes	Bacilli	Bacillales	Paenibacillaceae	Paenibacillus	hypothetical protein						
376038..376146	+	SMG36835.1	FXAZ01000002.1	Paenibacillus aquistagni	Bacteria	Firmicutes	Bacilli	Bacillales	Paenibacillaceae	Paenibacillus	hypothetical protein						
376440..377915	array		FXAZ01000002.1	Paenibacillus aquistagni	Bacteria	Firmicutes	Bacilli	Bacillales	Paenibacillaceae	Paenibacillus		CRISPR array			23	36	29
480 CAS-I-B | OUM88544.1																	
82323..83040	+	OUM88537.1	LZRT01000060.1	Bacillus thermozeamaize	Bacteria	Firmicutes	Bacilli	Bacillales	Bacillaceae	Bacillus	CRISPR-associated endoribonuclease Cas6	cas6	COG1583	CAS-I,CAS-III,CAS-IV			
83065..84916	+	OUM88538.1	LZRT01000060.1	Bacillus thermozeamaize	Bacteria	Firmicutes	Bacilli	Bacillales	Bacillaceae	Bacillus	type I-B CRISPR-associated protein Cas8b1/Cst1	cas8b2	cd09665	CAS-I-B			
84908..85805	+	OUM88539.1	LZRT01000060.1	Bacillus thermozeamaize	Bacteria	Firmicutes	Bacilli	Bacillales	Bacillaceae	Bacillus	type I-B CRISPR-associated protein Cas7/Cst2/DevR	cas7	cd09687	CAS-I-B			
85801..86515	+	OUM88540.1	LZRT01000060.1	Bacillus thermozeamaize	Bacteria	Firmicutes	Bacilli	Bacillales	Bacillaceae	Bacillus	type I-B CRISPR-associated protein Cas5	cas5	cd09658	CAS-I-B			
86526..88740	+	OUM88541.1	LZRT01000060.1	Bacillus thermozeamaize	Bacteria	Firmicutes	Bacilli	Bacillales	Bacillaceae	Bacillus	CRISPR-associated helicase/endonuclease Cas3	cas3HD,cas3	cd09641,COG1203	CAS-I			
88776..89301	+	OUM88542.1	LZRT01000060.1	Bacillus thermozeamaize	Bacteria	Firmicutes	Bacilli	Bacillales	Bacillaceae	Bacillus	CRISPR-associated protein Cas4	cas4	pfam01930	CAS-I			
89297..90278	+	OUM88543.1	LZRT01000060.1	Bacillus thermozeamaize	Bacteria	Firmicutes	Bacilli	Bacillales	Bacillaceae	Bacillus	subtype I-B CRISPR-associated endonuclease Cas1	cas1	cd09722	CAS-I-B			
90305..90569	+	OUM88544.1	LZRT01000060.1	Bacillus thermozeamaize	Bacteria	Firmicutes	Bacilli	Bacillales	Bacillaceae	Bacillus	CRISPR-associated endonuclease Cas2	cas2	cd09725	CAS-I,CAS-II,CAS-III,CAS-V			
90725..91952	+	OUM88545.1	LZRT01000060.1	Bacillus thermozeamaize	Bacteria	Firmicutes	Bacilli	Bacillales	Bacillaceae	Bacillus	transposase						
92416..97150	array		LZRT01000060.1	Bacillus thermozeamaize	Bacteria	Firmicutes	Bacilli	Bacillales	Bacillaceae	Bacillus		CRISPR array			73	36	29
481 CAS-I-B | AXM89704.1																	
2145513..2146137	-	AXM89697.1	CP025535.1	Anoxybacillus kamchatkensis G10	Bacteria	Firmicutes	Bacilli	Bacillales	Bacillaceae	Anoxybacillus	YHYH domain-containing protein	C3a212	cluster212				
2146385..2147111	+	AXM89698.1	CP025535.1	Anoxybacillus kamchatkensis G10	Bacteria	Firmicutes	Bacilli	Bacillales	Bacillaceae	Anoxybacillus	CRISPR-associated endoribonuclease Cas6	cas6	COG1583	CAS-I,CAS-III,CAS-IV			
2147107..2148883	+	AXM89699.1	CP025535.1	Anoxybacillus kamchatkensis G10	Bacteria	Firmicutes	Bacilli	Bacillales	Bacillaceae	Anoxybacillus	type I-B CRISPR-associated protein Cas8b1/Cst1	cas8b2	cd09665	CAS-I-B			
2148879..2149767	+	AXM89700.1	CP025535.1	Anoxybacillus kamchatkensis G10	Bacteria	Firmicutes	Bacilli	Bacillales	Bacillaceae	Anoxybacillus	type I-B CRISPR-associated protein Cas7/Cst2/DevR	cas7	cd09687	CAS-I-B			
2149769..2150477	+	AXM89701.1	CP025535.1	Anoxybacillus kamchatkensis G10	Bacteria	Firmicutes	Bacilli	Bacillales	Bacillaceae	Anoxybacillus	type I-B CRISPR-associated protein Cas5	cas5	cd09658	CAS-I-B			
2152681..2153179	+	AXM89702.1	CP025535.1	Anoxybacillus kamchatkensis G10	Bacteria	Firmicutes	Bacilli	Bacillales	Bacillaceae	Anoxybacillus	CRISPR-associated protein Cas4	cas4	pfam01930	CAS-I			
2153180..2154164	+	AXM89703.1	CP025535.1	Anoxybacillus kamchatkensis G10	Bacteria	Firmicutes	Bacilli	Bacillales	Bacillaceae	Anoxybacillus	type I-B CRISPR-associated endonuclease Cas1	cas1	cd09722	CAS-I-B			
2154166..2154430	+	AXM89704.1	CP025535.1	Anoxybacillus kamchatkensis G10	Bacteria	Firmicutes	Bacilli	Bacillales	Bacillaceae	Anoxybacillus	CRISPR-associated endonuclease Cas2	cas2	cd09725	CAS-I,CAS-II,CAS-III,CAS-V			
2154618..2155440	array		CP025535.1	Anoxybacillus kamchatkensis G10	Bacteria	Firmicutes	Bacilli	Bacillales	Bacillaceae	Anoxybacillus		CRISPR array			13	37	29

482 CAS-I-B | SFA58200.1

512..1241	+	SFA58173.1	FOJQ01000071.1	Anoxybacillus pushchinoensis	Bacteria	Firmicutes	Bacilli	Bacillales	Bacillaceae	Anoxybacillus	CRISPR-associated endoribonuclease Cas6	cas6	COG1583	CAS-I,CAS-III,CAS-IV			
1237..3025	+	SFA58177.1	FOJQ01000071.1	Anoxybacillus pushchinoensis	Bacteria	Firmicutes	Bacilli	Bacillales	Bacillaceae	Anoxybacillus	CRISPR-associated protein Cst1	cas8b2	cd09665	CAS-I-B			
3021..3927	+	SFA58182.1	FOJQ01000071.1	Anoxybacillus pushchinoensis	Bacteria	Firmicutes	Bacilli	Bacillales	Bacillaceae	Anoxybacillus	CRISPR-associated protein Cst2	cas7	cd09687	CAS-I-B			
3929..4631	+	SFA58186.1	FOJQ01000071.1	Anoxybacillus pushchinoensis	Bacteria	Firmicutes	Bacilli	Bacillales	Bacillaceae	Anoxybacillus	CRISPR-associated protein Cas5t	cas5	cd09658	CAS-I-B			
4642..6820	+	SFA58190.1	FOJQ01000071.1	Anoxybacillus pushchinoensis	Bacteria	Firmicutes	Bacilli	Bacillales	Bacillaceae	Anoxybacillus	CRISPR-associated endonuclease/helicase Cas3	cas3HD,cas3	cd09641,cd09639	CAS-I			
6834..7332	+	SFA58194.1	FOJQ01000071.1	Anoxybacillus pushchinoensis	Bacteria	Firmicutes	Bacilli	Bacillales	Bacillaceae	Anoxybacillus	CRISPR-associated exonuclease Cas4	cas4	pfam01930	CAS-I			
7331..8315	+	SFA58197.1	FOJQ01000071.1	Anoxybacillus pushchinoensis	Bacteria	Firmicutes	Bacilli	Bacillales	Bacillaceae	Anoxybacillus	CRISP-associated protein Cas1	cas1	cd09722	CAS-I-B			
8316..8580	+	SFA58200.1	FOJQ01000071.1	Anoxybacillus pushchinoensis	Bacteria	Firmicutes	Bacilli	Bacillales	Bacillaceae	Anoxybacillus	CRISPR-associated protein Cas2	cas2	cd09725	CAS-I,CAS-II,CAS-III,CAS-V			
8770..8931	array		FOJQ01000071.1	Anoxybacillus pushchinoensis	Bacteria	Firmicutes	Bacilli	Bacillales	Bacillaceae	Anoxybacillus		CRISPR array			3	37	29

483 CAS-I-B | EMT47063.1

881..1610	+	EMT47056.1	APCD01000001.1	Anoxybacillus flavithermus AK1	Bacteria	Firmicutes	Bacilli	Bacillales	Bacillaceae	Anoxybacillus	CRISPR system-like protein, RAMP superfamily	cas6	COG1583	CAS-I,CAS-III,CAS-IV			
1606..3382	+	EMT47057.1	APCD01000001.1	Anoxybacillus flavithermus AK1	Bacteria	Firmicutes	Bacilli	Bacillales	Bacillaceae	Anoxybacillus	CRISPR system-like protein	cas8b2	cd09665	CAS-I-B			
3378..4266	+	EMT47058.1	APCD01000001.1	Anoxybacillus flavithermus AK1	Bacteria	Firmicutes	Bacilli	Bacillales	Bacillaceae	Anoxybacillus	CRISPR system-like protein	cas7	cd09687	CAS-I-B			
4268..4976	+	EMT47059.1	APCD01000001.1	Anoxybacillus flavithermus AK1	Bacteria	Firmicutes	Bacilli	Bacillales	Bacillaceae	Anoxybacillus	CRISPR system-like protein, RAMP superfamily	cas5	cd09658	CAS-I-B			
4987..7168	+	EMT47060.1	APCD01000001.1	Anoxybacillus flavithermus AK1	Bacteria	Firmicutes	Bacilli	Bacillales	Bacillaceae	Anoxybacillus	CRISPR-associated protein Cas3	cas3	COG1203	CAS-I			
7183..7681	+	EMT47061.1	APCD01000001.1	Anoxybacillus flavithermus AK1	Bacteria	Firmicutes	Bacilli	Bacillales	Bacillaceae	Anoxybacillus	CRISPR-associated protein Cas4	cas4	pfam01930	CAS-I			
7681..8665	+	EMT47062.1	APCD01000001.1	Anoxybacillus flavithermus AK1	Bacteria	Firmicutes	Bacilli	Bacillales	Bacillaceae	Anoxybacillus	CRISPR-associated protein Cas1	cas1	cd09722	CAS-I-B			
8666..8930	+	EMT47063.1	APCD01000001.1	Anoxybacillus flavithermus AK1	Bacteria	Firmicutes	Bacilli	Bacillales	Bacillaceae	Anoxybacillus	CRISPR-associated protein Cas2	cas2	cd09725	CAS-I,CAS-II,CAS-III,CAS-V			
9118..10936	array		APCD01000001.1	Anoxybacillus flavithermus AK1	Bacteria	Firmicutes	Bacilli	Bacillales	Bacillaceae	Anoxybacillus		CRISPR array			28	37	29

484 CAS-I-B | ADP74268.1

1440046..1440289	+	ADP74258.1	CP002293.1	Geobacillus sp. Y4.1MC1	Bacteria	Firmicutes	Bacilli	Bacillales	Bacillaceae	Geobacillus	hypothetical protein	cas6	mkCas0091	CAS-I,CAS-III			
1440304..1441246	+	ADP74259.1	CP002293.1	Geobacillus sp. Y4.1MC1	Bacteria	Firmicutes	Bacilli	Bacillales	Bacillaceae	Geobacillus	CRISPR-associated protein, APE2256 family	cas6,csm6	mkCas0066,cd09742	CAS-I,CAS-III			
1441263..1441689	+	ADP74260.1	CP002293.1	Geobacillus sp. Y4.1MC1	Bacteria	Firmicutes	Bacilli	Bacillales	Bacillaceae	Geobacillus	hypothetical protein	Csx1	cluster2				
1441941..1442670	+	ADP74261.1	CP002293.1	Geobacillus sp. Y4.1MC1	Bacteria	Firmicutes	Bacilli	Bacillales	Bacillaceae	Geobacillus	CRISPR-associated protein Cas6	cas6	COG1583	CAS-I,CAS-III,CAS-IV			
1442666..1444472	+	ADP74262.1	CP002293.1	Geobacillus sp. Y4.1MC1	Bacteria	Firmicutes	Bacilli	Bacillales	Bacillaceae	Geobacillus	CRISPR-associated CXXC_CXXC protein Cst1	cas8b2	cd09665	CAS-I-B			
1444468..1445356	+	ADP74263.1	CP002293.1	Geobacillus sp. Y4.1MC1	Bacteria	Firmicutes	Bacilli	Bacillales	Bacillaceae	Geobacillus	CRISPR-associated autoregulator, DevR family	cas7	cd09687	CAS-I-B			
1445358..1446060	+	ADP74264.1	CP002293.1	Geobacillus sp. Y4.1MC1	Bacteria	Firmicutes	Bacilli	Bacillales	Bacillaceae	Geobacillus	CRISPR-associated protein Cas5 family	cas5	cd09658	CAS-I-B			
1446072..1448253	+	ADP74265.1	CP002293.1	Geobacillus sp. Y4.1MC1	Bacteria	Firmicutes	Bacilli	Bacillales	Bacillaceae	Geobacillus	metal dependent phosphohydrolase	cas3HD,cas3	cd09641,COG1203	CAS-I			
1448266..1448764	+	ADP74266.1	CP002293.1	Geobacillus sp. Y4.1MC1	Bacteria	Firmicutes	Bacilli	Bacillales	Bacillaceae	Geobacillus	CRISPR-associated protein Cas4	cas4	pfam01930	CAS-I			
1448764..1449748	+	ADP74267.1	CP002293.1	Geobacillus sp. Y4.1MC1	Bacteria	Firmicutes	Bacilli	Bacillales	Bacillaceae	Geobacillus	CRISPR-associated protein Cas1	cas1	cd09722	CAS-I-B			
1449749..1450013	+	ADP74268.1	CP002293.1	Geobacillus sp. Y4.1MC1	Bacteria	Firmicutes	Bacilli	Bacillales	Bacillaceae	Geobacillus	CRISPR-associated protein Cas2	cas2	cd09725	CAS-I,CAS-II,CAS-III,CAS-V			
1450222..1453456	array		CP002293.1	Geobacillus sp. Y4.1MC1	Bacteria	Firmicutes	Bacilli	Bacillales	Bacillaceae	Geobacillus		CRISPR array			50	36	29

485 CAS-I-B | OXB92095.1

649896..650167	array		NDYL01000002.1	Geobacillus galactosidasius	Bacteria	Firmicutes	Bacilli	Bacillales	Bacillaceae	Geobacillus		CRISPR array			5	41	21
650567..651891	array		NDYL01000002.1	Geobacillus galactosidasius	Bacteria	Firmicutes	Bacilli	Bacillales	Bacillaceae	Geobacillus		CRISPR array			21	36	28
652110..652374	-	OXB92095.1	NDYL01000002.1	Geobacillus galactosidasius	Bacteria	Firmicutes	Bacilli	Bacillales	Bacillaceae	Geobacillus	CRISPR-associated endonuclease Cas2	cas2	cd09725	CAS-I,CAS-II,CAS-III,CAS-V			
652376..653360	-	OXB92096.1	NDYL01000002.1	Geobacillus galactosidasius	Bacteria	Firmicutes	Bacilli	Bacillales	Bacillaceae	Geobacillus	subtype I-B CRISPR-associated endonuclease Cas1	cas1	cd09722	CAS-I-B			
653359..653857	-	OXB92097.1	NDYL01000002.1	Geobacillus galactosidasius	Bacteria	Firmicutes	Bacilli	Bacillales	Bacillaceae	Geobacillus	CRISPR-associated protein Cas4	cas4	pfam01930	CAS-I			
653870..656051	-	OXB92098.1	NDYL01000002.1	Geobacillus galactosidasius	Bacteria	Firmicutes	Bacilli	Bacillales	Bacillaceae	Geobacillus	CRISPR-associated helicase/endonuclease Cas3	cas3HD,cas3	cd09641,COG1203	CAS-I			
656063..656765	-	OXB92099.1	NDYL01000002.1	Geobacillus galactosidasius	Bacteria	Firmicutes	Bacilli	Bacillales	Bacillaceae	Geobacillus	type I-B CRISPR-associated protein Cas5	cas5	cd09658	CAS-I-B			
656767..657655	-	OXB92100.1	NDYL01000002.1	Geobacillus galactosidasius	Bacteria	Firmicutes	Bacilli	Bacillales	Bacillaceae	Geobacillus	type I-B CRISPR-associated protein Cas7/Cst2/DevR	cas7	cd09687	CAS-I-B			
657651..659457	-	OXB92101.1	NDYL01000002.1	Geobacillus galactosidasius	Bacteria	Firmicutes	Bacilli	Bacillales	Bacillaceae	Geobacillus	type I-B CRISPR-associated protein Cas8b1/Cst1	cas8b2	cd09665	CAS-I-B			
659453..660182	-	OXB92102.1	NDYL01000002.1	Geobacillus galactosidasius	Bacteria	Firmicutes	Bacilli	Bacillales	Bacillaceae	Geobacillus	CRISPR-associated endoribonuclease Cas6	cas6	COG1583	CAS-I,CAS-III,CAS-IV			

486 CAS-I-B | ASS99179.1

1815635..1816361	+	ASS99171.1	CP018058.1	Geobacillus thermocatenulatus	Bacteria	Firmicutes	Bacilli	Bacillales	Bacillaceae	Geobacillus	CRISPR-associated endoribonuclease Cas6	cas6	COG1583	CAS-I,CAS-III,CAS-IV			
1816357..1818154	+	ASS99172.1	CP018058.1	Geobacillus thermocatenulatus	Bacteria	Firmicutes	Bacilli	Bacillales	Bacillaceae	Geobacillus	type I-B CRISPR-associated protein Cas8b1/Cst1	cas8b2	cd09665	CAS-I-B			
1818246..1819527	+	ASS99173.1	CP018058.1	Geobacillus thermocatenulatus	Bacteria	Firmicutes	Bacilli	Bacillales	Bacillaceae	Geobacillus	IS110 family transposase						
1819923..1820811	+	ASS99174.1	CP018058.1	Geobacillus thermocatenulatus	Bacteria	Firmicutes	Bacilli	Bacillales	Bacillaceae	Geobacillus	type I-B CRISPR-associated protein Cas7/Cst2/DevR	cas7	cd09687	CAS-I-B			
1820813..1821515	+	ASS99175.1	CP018058.1	Geobacillus thermocatenulatus	Bacteria	Firmicutes	Bacilli	Bacillales	Bacillaceae	Geobacillus	type I-B CRISPR-associated protein Cas5	cas5	cd09658	CAS-I-B			
1821538..1823710	+	ASS99176.1	CP018058.1	Geobacillus thermocatenulatus	Bacteria	Firmicutes	Bacilli	Bacillales	Bacillaceae	Geobacillus	CRISPR-associated helicase/endonuclease Cas3	cas3HD,cas3	cd09641,COG1203	CAS-I			
1823724..1824222	+	ASS99177.1	CP018058.1	Geobacillus thermocatenulatus	Bacteria	Firmicutes	Bacilli	Bacillales	Bacillaceae	Geobacillus	CRISPR-associated protein Cas4	cas4	pfam01930	CAS-I			
1824221..1825205	+	ASS99178.1	CP018058.1	Geobacillus thermocatenulatus	Bacteria	Firmicutes	Bacilli	Bacillales	Bacillaceae	Geobacillus	subtype I-B CRISPR-associated endonuclease Cas1	cas1	cd09722	CAS-I-B			
1825208..1825472	+	ASS99179.1	CP018058.1	Geobacillus thermocatenulatus	Bacteria	Firmicutes	Bacilli	Bacillales	Bacillaceae	Geobacillus	CRISPR-associated endonuclease Cas2	cas2	cd09725	CAS-I,CAS-II,CAS-III,CAS-V			
1825669..1827069	array		CP018058.1	Geobacillus thermocatenulatus	Bacteria	Firmicutes	Bacilli	Bacillales	Bacillaceae	Geobacillus		CRISPR array			22	36	29

487 CAS-I-B | KJE26697.1

863454..864180	+	KJE29070.1	JYBP01000003.1	Geobacillus kaustophilus	Bacteria	Firmicutes	Bacilli	Bacillales	Bacillaceae	Geobacillus	CRISPR-associated endoribonuclease Cas6	cas6	COG1583	CAS-I,CAS-III,CAS-IV			
864176..865976	+	KJE26073.1	JYBP01000003.1	Geobacillus kaustophilus	Bacteria	Firmicutes	Bacilli	Bacillales	Bacillaceae	Geobacillus	putative cRISPR-associated CXXC-CXXC protein Cst1	cas8b2	cd09665	CAS-I-B			
865972..866860	+	KJE28974.1	JYBP01000003.1	Geobacillus kaustophilus	Bacteria	Firmicutes	Bacilli	Bacillales	Bacillaceae	Geobacillus	CRISPR-associated autoregulator DevR family protein	cas7	cd09687	CAS-I-B			
866862..867564	+	KJE28586.1	JYBP01000003.1	Geobacillus kaustophilus	Bacteria	Firmicutes	Bacilli	Bacillales	Bacillaceae	Geobacillus	CRISPR-associated Cas5 domain protein	cas5	cd09658	CAS-I-B			
867586..869764	+	KJE27248.1	JYBP01000003.1	Geobacillus kaustophilus	Bacteria	Firmicutes	Bacilli	Bacillales	Bacillaceae	Geobacillus	CRISPR-associated endonuclease Cas3-HD	cas3HD,cas3	cd09641,COG1203	CAS-I			
869778..869970	+	KJE26141.1	JYBP01000003.1	Geobacillus kaustophilus	Bacteria	Firmicutes	Bacilli	Bacillales	Bacillaceae	Geobacillus	hypothetical protein	cas4	pfam01930	CAS-I			
870064..871237	+	KJE27405.1	JYBP01000003.1	Geobacillus kaustophilus	Bacteria	Firmicutes	Bacilli	Bacillales	Bacillaceae	Geobacillus	transposase family protein						
871373..871619	+	KJE27109.1	JYBP01000003.1	Geobacillus kaustophilus	Bacteria	Firmicutes	Bacilli	Bacillales	Bacillaceae	Geobacillus	hypothetical protein	cas4	pfam01930	CAS-I			
871618..872602	+	KJE26459.1	JYBP01000003.1	Geobacillus kaustophilus	Bacteria	Firmicutes	Bacilli	Bacillales	Bacillaceae	Geobacillus	CRISPR-associated endonuclease Cas1	cas1	cd09722	CAS-I-B			
872605..872869	+	KJE26697.1	JYBP01000003.1	Geobacillus kaustophilus	Bacteria	Firmicutes	Bacilli	Bacillales	Bacillaceae	Geobacillus	CRISPR-associated endonuclease Cas2	cas2	cd09725	CAS-I,CAS-II,CAS-III,CAS-V			
873067..874849	array		JYBP01000003.1	Geobacillus kaustophilus	Bacteria	Firmicutes	Bacilli	Bacillales	Bacillaceae	Geobacillus		CRISPR array			28	35	29

488 CAS-I-B | KYD32180.1

16880..18319	array		LQYY01000122.1	Geobacillus stearothermophilus	Bacteria	Firmicutes	Bacilli	Bacillales	Bacillaceae	Geobacillus		CRISPR array			23	35	29
18516..18780	-	KYD32180.1	LQYY01000122.1	Geobacillus stearothermophilus	Bacteria	Firmicutes	Bacilli	Bacillales	Bacillaceae	Geobacillus	hypothetical protein	cas2	cd09725	CAS-I,CAS-II,CAS-III,CAS-V			
18783..19770	-	KYD32181.1	LQYY01000122.1	Geobacillus stearothermophilus	Bacteria	Firmicutes	Bacilli	Bacillales	Bacillaceae	Geobacillus	hypothetical protein	cas1	cd09722	CAS-I-B			
19766..20264	-	KYD32182.1	LQYY01000122.1	Geobacillus stearothermophilus	Bacteria	Firmicutes	Bacilli	Bacillales	Bacillaceae	Geobacillus	hypothetical protein	cas4	pfam01930	CAS-I			
20278..22456	-	KYD32183.1	LQYY01000122.1	Geobacillus stearothermophilus	Bacteria	Firmicutes	Bacilli	Bacillales	Bacillaceae	Geobacillus	hypothetical protein	cas3HD,cas3	cd09641,cd09639	CAS-I			
22479..23181	-	KYD32184.1	LQYY01000122.1	Geobacillus stearothermophilus	Bacteria	Firmicutes	Bacilli	Bacillales	Bacillaceae	Geobacillus	hypothetical protein	cas5	cd09658	CAS-I-B			
23183..24071	-	KYD32185.1	LQYY01000122.1	Geobacillus stearothermophilus	Bacteria	Firmicutes	Bacilli	Bacillales	Bacillaceae	Geobacillus	hypothetical protein	cas7	cd09687	CAS-I-B			
24067..25846	-	KYD32186.1	LQYY01000122.1	Geobacillus stearothermophilus	Bacteria	Firmicutes	Bacilli	Bacillales	Bacillaceae	Geobacillus	hypothetical protein	cas8b2	cd09665	CAS-I-B			
25860..26586	-	KYD32187.1	LQYY01000122.1	Geobacillus stearothermophilus	Bacteria	Firmicutes	Bacilli	Bacillales	Bacillaceae	Geobacillus	hypothetical protein	cas6	COG1583	CAS-I,CAS-III,CAS-IV			

489 CAS-I-B | ATA60318.1

1957205..1957469	-	ATA60318.1	CP016552.1	Geobacillus stearothermophilus	Bacteria	Firmicutes	Bacilli	Bacillales	Bacillaceae	Geobacillus	CRISPR-associated protein Cas2	cas2	cd09725	CAS-I,CAS-II,CAS-III,CAS-V			
1957472..1958459	-	ATA60319.1	CP016552.1	Geobacillus stearothermophilus	Bacteria	Firmicutes	Bacilli	Bacillales	Bacillaceae	Geobacillus	CRISPR-associated protein Cas1	cas1	cd09722	CAS-I-B			
1958455..1958953	-	ATA60320.1	CP016552.1	Geobacillus stearothermophilus	Bacteria	Firmicutes	Bacilli	Bacillales	Bacillaceae	Geobacillus	CRISPR-associated protein Cas4	cas4	pfam01930	CAS-I			
1958967..1961145	-	ATA60321.1	CP016552.1	Geobacillus stearothermophilus	Bacteria	Firmicutes	Bacilli	Bacillales	Bacillaceae	Geobacillus	CRISPR-associated helicase cas3 domain protein	cas3HD,cas3	cd09641,cd09639	CAS-I			
1961168..1961876	-	ATA60322.1	CP016552.1	Geobacillus stearothermophilus	Bacteria	Firmicutes	Bacilli	Bacillales	Bacillaceae	Geobacillus	CRISPR-associated protein Cas5	cas5	cd09658	CAS-I-B			
1961872..1962760	-	ATA60323.1	CP016552.1	Geobacillus stearothermophilus	Bacteria	Firmicutes	Bacilli	Bacillales	Bacillaceae	Geobacillus	crispr-associated regulatory protein devr family	cas7	cd09687	CAS-I-B			
1962756..1964553	-	ATA60324.1	CP016552.1	Geobacillus stearothermophilus	Bacteria	Firmicutes	Bacilli	Bacillales	Bacillaceae	Geobacillus	CRISPR-associated protein Cst1	cas8b2	cd09665	CAS-I-B			
1964549..1965275	-	ATA60325.1	CP016552.1	Geobacillus stearothermophilus	Bacteria	Firmicutes	Bacilli	Bacillales	Bacillaceae	Geobacillus	crispr-associated protein cas6	cas6	COG1583	CAS-I,CAS-III,CAS-IV			

490 CAS-I-B | OON98294.1

4709..5651	+	OON98286.1	LNZQ01000224.1	Epulopiscium sp. Nele67-Bin004	Bacteria	Firmicutes	Clostridia	Clostridiales		Epulopiscium	hypothetical protein	WYL	COG2378	CAS-I,CAS-III			
5715..6447	+	OON98287.1	LNZQ01000224.1	Epulopiscium sp. Nele67-Bin004	Bacteria	Firmicutes	Clostridia	Clostridiales		Epulopiscium	CRISPR-associated endoribonuclease Cas6	cas6	pfam01881	CAS-I,CAS-III			
6460..8137	+	OON98288.1	LNZQ01000224.1	Epulopiscium sp. Nele67-Bin004	Bacteria	Firmicutes	Clostridia	Clostridiales		Epulopiscium	type I-B CRISPR-associated protein Cas8b1/Cst1	cas8b2,cas8b2	cd09754,cd09665	CAS-I-B			
8129..9014	+	OON98289.1	LNZQ01000224.1	Epulopiscium sp. Nele67-Bin004	Bacteria	Firmicutes	Clostridia	Clostridiales		Epulopiscium	type I-B CRISPR-associated protein Cas7/Cst2/DevR	cas7	cd09687	CAS-I-B			
9032..9779	+	OON98290.1	LNZQ01000224.1	Epulopiscium sp. Nele67-Bin004	Bacteria	Firmicutes	Clostridia	Clostridiales		Epulopiscium	type I-B CRISPR-associated protein Cas5	cas5	cd09658	CAS-I-B			
9822..12069	+	OON98291.1	LNZQ01000224.1	Epulopiscium sp. Nele67-Bin004	Bacteria	Firmicutes	Clostridia	Clostridiales		Epulopiscium	hypothetical protein	cas3	cd09639	CAS-I			
12088..12592	+	OON98292.1	LNZQ01000224.1	Epulopiscium sp. Nele67-Bin004	Bacteria	Firmicutes	Clostridia	Clostridiales		Epulopiscium	CRISPR-associated protein Cas4	cas4	pfam01930	CAS-I			
12597..13578	+	OON98293.1	LNZQ01000224.1	Epulopiscium sp. Nele67-Bin004	Bacteria	Firmicutes	Clostridia	Clostridiales		Epulopiscium	subtype I-B CRISPR-associated endonuclease Cas1	cas1	cd09722	CAS-I-B			
13594..13858	+	OON98294.1	LNZQ01000224.1	Epulopiscium sp. Nele67-Bin004	Bacteria	Firmicutes	Clostridia	Clostridiales		Epulopiscium	CRISPR-associated endonuclease Cas2	cas2	cd09725	CAS-I,CAS-II,CAS-III,CAS-V			
14043..14266	array		LNZQ01000224.1	Epulopiscium sp. Nele67-Bin004	Bacteria	Firmicutes	Clostridia	Clostridiales		Epulopiscium		CRISPR array			4	36	29

491 CAS-I-B | SHK10179.1

6..164	array		FRAD01000014.1	Hathewaya proteolytica DSM 3090	Bacteria	Firmicutes	Clostridia	Clostridiales	Clostridiaceae	Hathewaya		CRISPR array			3	36	29
347..611	-	SHK10179.1	FRAD01000014.1	Hathewaya proteolytica DSM 3090	Bacteria	Firmicutes	Clostridia	Clostridiales	Clostridiaceae	Hathewaya	CRISPR-associated protein, Cas2 family	cas2	cd09725	CAS-I,CAS-II,CAS-III,CAS-V			
612..1593	-	SHK10210.1	FRAD01000014.1	Hathewaya proteolytica DSM 3090	Bacteria	Firmicutes	Clostridia	Clostridiales	Clostridiaceae	Hathewaya	CRISP-associated protein Cas1	cas1	cd09722	CAS-I-B			
1592..2111	-	SHK10240.1	FRAD01000014.1	Hathewaya proteolytica DSM 3090	Bacteria	Firmicutes	Clostridia	Clostridiales	Clostridiaceae	Hathewaya	CRISPR-associated exonuclease Cas4	cas4	pfam01930	CAS-I			
2127..4512	-	SHK10258.1	FRAD01000014.1	Hathewaya proteolytica DSM 3090	Bacteria	Firmicutes	Clostridia	Clostridiales	Clostridiaceae	Hathewaya	CRISPR-associated endonuclease/helicase Cas3	cas3HD,cas3	cd09641,COG1203	CAS-I			
4489..5161	-	SHK10290.1	FRAD01000014.1	Hathewaya proteolytica DSM 3090	Bacteria	Firmicutes	Clostridia	Clostridiales	Clostridiaceae	Hathewaya	CRISPR-associated protein Cas5t	cas5	cd09658	CAS-I-B			
5164..6073	-	SHK10325.1	FRAD01000014.1	Hathewaya proteolytica DSM 3090	Bacteria	Firmicutes	Clostridia	Clostridiales	Clostridiaceae	Hathewaya	CRISPR-associated protein Cst2	cas7	cd09687	CAS-I-B			
6074..6383	-	SHK10350.1	FRAD01000014.1	Hathewaya proteolytica DSM 3090	Bacteria	Firmicutes	Clostridia	Clostridiales	Clostridiaceae	Hathewaya	CRISPR-associated protein Cas8b1/Cst1, subtype I-B/TNEAP	cas8b2	cd09665	CAS-I-B			
6364..7900	-	SHK10383.1	FRAD01000014.1	Hathewaya proteolytica DSM 3090	Bacteria	Firmicutes	Clostridia	Clostridiales	Clostridiaceae	Hathewaya	CRISPR-associated protein Cst1	cas8b2	cd09665	CAS-I-B			
7914..8649	-	SHK10416.1	FRAD01000014.1	Hathewaya proteolytica DSM 3090	Bacteria	Firmicutes	Clostridia	Clostridiales	Clostridiaceae	Hathewaya	CRISPR-associated endoribonuclease Cas6	cas6	COG1583	CAS-I,CAS-III,CAS-IV			
8808..9756	-	SHK10445.1	FRAD01000014.1	Hathewaya proteolytica DSM 3090	Bacteria	Firmicutes	Clostridia	Clostridiales	Clostridiaceae	Hathewaya	Predicted DNA-binding transcriptional regulator YafY, contains an HTH and WYL domains	WYL,WYL	COG2378,pfam13280	CAS-I,CAS-III			

492 partial CAS-I-B | ETI96722.1

202..469	-	ETI96722.1	AZLW01000033.1	Intestinibacter bartlettii DORA_8_9	Bacteria	Firmicutes	Clostridia	Clostridiales	Peptostreptococcaceae	Intestinibacter	CRISPR-associated protein Cas2	cas2	cd09725	CAS-I,CAS-II,CAS-III,CAS-V			
470..1451	-	ETI96723.1	AZLW01000033.1	Intestinibacter bartlettii DORA_8_9	Bacteria	Firmicutes	Clostridia	Clostridiales	Peptostreptococcaceae	Intestinibacter	CRISPR-associated protein cas1	cas1	cd09722	CAS-I-B			
1450..1969	-	ETI96724.1	AZLW01000033.1	Intestinibacter bartlettii DORA_8_9	Bacteria	Firmicutes	Clostridia	Clostridiales	Peptostreptococcaceae	Intestinibacter	Crispr-associated protein Cas4	cas4	pfam01930	CAS-I			

493 CAS-I-B | CED93821.1

1128114..1129065	+	CED93813.1	LN555523.1	Romboutsia ilealis	Bacteria	Firmicutes	Clostridia	Clostridiales	Peptostreptococcaceae	Romboutsia	HTH domain protein	WYL	pfam13280	CAS-I,CAS-III			
1129134..1129866	+	CED93814.1	LN555523.1	Romboutsia ilealis	Bacteria	Firmicutes	Clostridia	Clostridiales	Peptostreptococcaceae	Romboutsia	CRISPR-associated protein cas6	cas6	COG1583	CAS-I,CAS-III,CAS-IV			
1129878..1131708	+	CED93815.1	LN555523.1	Romboutsia ilealis	Bacteria	Firmicutes	Clostridia	Clostridiales	Peptostreptococcaceae	Romboutsia	CRISPR-associated cxxc cxxc protein Cst1	cas8b2	cd09665	CAS-I-B			
1131721..1132633	+	CED93816.1	LN555523.1	Romboutsia ilealis	Bacteria	Firmicutes	Clostridia	Clostridiales	Peptostreptococcaceae	Romboutsia	Devr CRISPR-associated regulatory protein	cas7	cd09687	CAS-I-B			
1132634..1133339	+	CED93817.1	LN555523.1	Romboutsia ilealis	Bacteria	Firmicutes	Clostridia	Clostridiales	Peptostreptococcaceae	Romboutsia	CRISPR-associated protein cas5	cas5	cd09658	CAS-I-B			
1133360..1135673	+	CED93818.1	LN555523.1	Romboutsia ilealis	Bacteria	Firmicutes	Clostridia	Clostridiales	Peptostreptococcaceae	Romboutsia	CRISPR-associated nuclease/helicase Cas3	cas3	cd09639	CAS-I			
1135705..1136263	+	CED93819.1	LN555523.1	Romboutsia ilealis	Bacteria	Firmicutes	Clostridia	Clostridiales	Peptostreptococcaceae	Romboutsia	CRISPR-associated protein cas4	cas4	pfam01930	CAS-I			
1136262..1137240	+	CED93820.1	LN555523.1	Romboutsia ilealis	Bacteria	Firmicutes	Clostridia	Clostridiales	Peptostreptococcaceae	Romboutsia	CRISPR-associated endonuclease Cas1	cas1	cd09722	CAS-I-B			
1137241..1137508	+	CED93821.1	LN555523.1	Romboutsia ilealis	Bacteria	Firmicutes	Clostridia	Clostridiales	Peptostreptococcaceae	Romboutsia	CRISPR-associated endoribonuclease Cas2	cas2	cd09725	CAS-I,CAS-II,CAS-III,CAS-V			
1137691..1142348	array		LN555523.1	Romboutsia ilealis	Bacteria	Firmicutes	Clostridia	Clostridiales	Peptostreptococcaceae	Romboutsia		CRISPR array			71	37	29

494 CAS-I-B | SHH12206.1

24626..25511	+	SHH11997.1	FQWX01000020.1	Asaccharospora irregularis DSM 2635	Bacteria	Firmicutes	Clostridia	Clostridiales	Peptostreptococcaceae	Asaccharospora	Predicted DNA-binding transcriptional regulator YafY, contains an HTH and WYL domains	WYL	pfam13280	CAS-I,CAS-III			
25663..26401	+	SHH12021.1	FQWX01000020.1	Asaccharospora irregularis DSM 2635	Bacteria	Firmicutes	Clostridia	Clostridiales	Peptostreptococcaceae	Asaccharospora	CRISPR-associated protein, Cas6 family	cas6	pfam01881	CAS-I,CAS-III			
26406..28236	+	SHH12045.1	FQWX01000020.1	Asaccharospora irregularis DSM 2635	Bacteria	Firmicutes	Clostridia	Clostridiales	Peptostreptococcaceae	Asaccharospora	CRISPR-associated protein Cst1	cas8b2	cd09665	CAS-I-B			
28237..29149	+	SHH12069.1	FQWX01000020.1	Asaccharospora irregularis DSM 2635	Bacteria	Firmicutes	Clostridia	Clostridiales	Peptostreptococcaceae	Asaccharospora	CRISPR-associated autoregulator, Cst2 family	cas7	cd09687	CAS-I-B			
29150..29849	+	SHH12100.1	FQWX01000020.1	Asaccharospora irregularis DSM 2635	Bacteria	Firmicutes	Clostridia	Clostridiales	Peptostreptococcaceae	Asaccharospora	CRISPR-associated protein, Cas5t family	cas5	cd09658	CAS-I-B			
29975..32294	+	SHH12128.1	FQWX01000020.1	Asaccharospora irregularis DSM 2635	Bacteria	Firmicutes	Clostridia	Clostridiales	Peptostreptococcaceae	Asaccharospora	CRISPR-associated helicase, Cas3 family	cas3HD,cas3	cd09641,COG1203	CAS-I			
32325..32847	+	SHH12152.1	FQWX01000020.1	Asaccharospora irregularis DSM 2635	Bacteria	Firmicutes	Clostridia	Clostridiales	Peptostreptococcaceae	Asaccharospora	CRISPR-associated exonuclease, Cas4 family	cas4	pfam01930	CAS-I			
32846..33827	+	SHH12182.1	FQWX01000020.1	Asaccharospora irregularis DSM 2635	Bacteria	Firmicutes	Clostridia	Clostridiales	Peptostreptococcaceae	Asaccharospora	CRISPR-associated protein, Cas1 family	cas1	cd09722	CAS-I-B			
33828..34095	+	SHH12206.1	FQWX01000020.1	Asaccharospora irregularis DSM 2635	Bacteria	Firmicutes	Clostridia	Clostridiales	Peptostreptococcaceae	Asaccharospora	CRISPR-associated protein, Cas2 family	cas2	cd09725	CAS-I,CAS-II,CAS-III,CAS-V			
34210..34838	array		FQWX01000020.1	Asaccharospora irregularis DSM 2635	Bacteria	Firmicutes	Clostridia	Clostridiales	Peptostreptococcaceae	Asaccharospora		CRISPR array			10	37	29
36096..37258	array		FQWX01000020.1	Asaccharospora irregularis DSM 2635	Bacteria	Firmicutes	Clostridia	Clostridiales	Peptostreptococcaceae	Asaccharospora		CRISPR array			18	37	29

495 CAS-I-B | EFH05805.1

490636..491554	+	EFH05797.1	GG770711.1	Clostridioides difficile NAP08	Bacteria	Firmicutes	Clostridia	Clostridiales	Peptostreptococcaceae	Clostridioides	HTH domain protein	WYL	pfam13280	CAS-I,CAS-III			
491631..492369	+	EFH05798.1	GG770711.1	Clostridioides difficile NAP08	Bacteria	Firmicutes	Clostridia	Clostridiales	Peptostreptococcaceae	Clostridioides	CRISPR-associated endoribonuclease Cas6	cas6	pfam01881	CAS-I,CAS-III			
492374..494225	+	EFH05799.1	GG770711.1	Clostridioides difficile NAP08	Bacteria	Firmicutes	Clostridia	Clostridiales	Peptostreptococcaceae	Clostridioides	CRISPR-associated cxxc_cxxc protein Cst1	cas8b2	cd09665	CAS-I-B			
494181..495141	+	EFH05800.1	GG770711.1	Clostridioides difficile NAP08	Bacteria	Firmicutes	Clostridia	Clostridiales	Peptostreptococcaceae	Clostridioides	CRISPR-associated regulatory protein, DevR family	cas7	cd09687	CAS-I-B			
495142..495844	+	EFH05801.1	GG770711.1	Clostridioides difficile NAP08	Bacteria	Firmicutes	Clostridia	Clostridiales	Peptostreptococcaceae	Clostridioides	CRISPR-associated protein Cas5, Tneap subtype	cas5	cd09658	CAS-I-B			
495854..498176	+	EFH05802.1	GG770711.1	Clostridioides difficile NAP08	Bacteria	Firmicutes	Clostridia	Clostridiales	Peptostreptococcaceae	Clostridioides	CRISPR-associated helicase Cas3	cas3HD,cas3	cd09641,COG1203	CAS-I			
498190..498742	+	EFH05803.1	GG770711.1	Clostridioides difficile NAP08	Bacteria	Firmicutes	Clostridia	Clostridiales	Peptostreptococcaceae	Clostridioides	CRISPR-associated protein Cas4	cas4	pfam01930	CAS-I			
498744..499725	+	EFH05804.1	GG770711.1	Clostridioides difficile NAP08	Bacteria	Firmicutes	Clostridia	Clostridiales	Peptostreptococcaceae	Clostridioides	CRISPR-associated endonuclease Cas1, HMARI/TNEAP subtype	cas1	cd09722	CAS-I-B			
499727..499994	+	EFH05805.1	GG770711.1	Clostridioides difficile NAP08	Bacteria	Firmicutes	Clostridia	Clostridiales	Peptostreptococcaceae	Clostridioides	CRISPR-associated protein Cas2	cas2	cd09725	CAS-I,CAS-II,CAS-III,CAS-V			
500137..500257	+	EFH05806.1	GG770711.1	Clostridioides difficile NAP08	Bacteria	Firmicutes	Clostridia	Clostridiales	Peptostreptococcaceae	Clostridioides	hypothetical protein						
500178..501122	array		GG770711.1	Clostridioides difficile NAP08	Bacteria	Firmicutes	Clostridia	Clostridiales	Peptostreptococcaceae	Clostridioides		CRISPR array			15	36	29
500390..500645	+	EFH05807.1	GG770711.1	Clostridioides difficile NAP08	Bacteria	Firmicutes	Clostridia	Clostridiales	Peptostreptococcaceae	Clostridioides	hypothetical protein						
501157..501404	+	EFH05756.1	GG770711.1	Clostridioides difficile NAP08	Bacteria	Firmicutes	Clostridia	Clostridiales	Peptostreptococcaceae	Clostridioides	hypothetical protein						
501196..502333	array		GG770711.1	Clostridioides difficile NAP08	Bacteria	Firmicutes	Clostridia	Clostridiales	Peptostreptococcaceae	Clostridioides		CRISPR array			18	36	29

496 CAS-I-B | EQF23392.1

101329..103914	array		AVHW01000095.1	Clostridioides difficile CD160	Bacteria	Firmicutes	Clostridia	Clostridiales	Peptostreptococcaceae	Clostridioides		CRISPR array			40	36	29
102592..102751	-	EQF23441.1	AVHW01000095.1	Clostridioides difficile CD160	Bacteria	Firmicutes	Clostridia	Clostridiales	Peptostreptococcaceae	Clostridioides	hypothetical protein						
104097..104364	-	EQF23392.1	AVHW01000095.1	Clostridioides difficile CD160	Bacteria	Firmicutes	Clostridia	Clostridiales	Peptostreptococcaceae	Clostridioides	CRISPR-associated endoribonuclease Cas2	cas2	cd09725	CAS-I,CAS-II,CAS-III,CAS-V			
104367..105348	-	EQF23357.1	AVHW01000095.1	Clostridioides difficile CD160	Bacteria	Firmicutes	Clostridia	Clostridiales	Peptostreptococcaceae	Clostridioides	CRISPR-associated endonuclease Cas1	cas1	cd09722	CAS-I-B			
105350..105902	-	EQF23448.1	AVHW01000095.1	Clostridioides difficile CD160	Bacteria	Firmicutes	Clostridia	Clostridiales	Peptostreptococcaceae	Clostridioides	CRISPR-associated protein Cas4	cas4	pfam01930	CAS-I			
105916..108238	-	EQF23416.1	AVHW01000095.1	Clostridioides difficile CD160	Bacteria	Firmicutes	Clostridia	Clostridiales	Peptostreptococcaceae	Clostridioides	CRISPR-associated endonuclease Cas3-HD	cas3HD,cas3	cd09641,COG1203	CAS-I			
108218..108953	-	EQF23356.1	AVHW01000095.1	Clostridioides difficile CD160	Bacteria	Firmicutes	Clostridia	Clostridiales	Peptostreptococcaceae	Clostridioides	CRISPR-associated Cas5 domain protein	cas5,csm5gr7	cd09658,COG1332	CAS-I-B			
108954..109869	-	EQF23378.1	AVHW01000095.1	Clostridioides difficile CD160	Bacteria	Firmicutes	Clostridia	Clostridiales	Peptostreptococcaceae	Clostridioides	CRISPR-associated autoregulator DevR family protein	cas7	cd09687	CAS-I-B			
109870..111727	-	EQF23389.1	AVHW01000095.1	Clostridioides difficile CD160	Bacteria	Firmicutes	Clostridia	Clostridiales	Peptostreptococcaceae	Clostridioides	CRISPR-associated family protein	cas8b2	cd09665	CAS-I-B			
111732..112470	-	EQF23420.1	AVHW01000095.1	Clostridioides difficile CD160	Bacteria	Firmicutes	Clostridia	Clostridiales	Peptostreptococcaceae	Clostridioides	CRISPR-associated endoribonuclease Cas6	cas6	pfam01881	CAS-I,CAS-III			
112547..113465	-	EQF23394.1	AVHW01000095.1	Clostridioides difficile CD160	Bacteria	Firmicutes	Clostridia	Clostridiales	Peptostreptococcaceae	Clostridioides	HTH domain protein	WYL	pfam13280	CAS-I,CAS-III			

497 CAS-I-B | KPI49094.1

158462..160397	array		JPPA01000086.1	Clostridioides difficile	Bacteria	Firmicutes	Clostridia	Clostridiales	Peptostreptococcaceae	Clostridioides		CRISPR array			30	36	29
160580..160847	-	KPI49094.1	JPPA01000086.1	Clostridioides difficile	Bacteria	Firmicutes	Clostridia	Clostridiales	Peptostreptococcaceae	Clostridioides	CRISPR-associated protein Cas2	cas2	cd09725	CAS-I,CAS-II,CAS-III,CAS-V			
160850..161831	-	KPI49095.1	JPPA01000086.1	Clostridioides difficile	Bacteria	Firmicutes	Clostridia	Clostridiales	Peptostreptococcaceae	Clostridioides	CRISPR-associated protein Cas1	cas1	cd09722	CAS-I-B			
161833..162352	-	KPI49096.1	JPPA01000086.1	Clostridioides difficile	Bacteria	Firmicutes	Clostridia	Clostridiales	Peptostreptococcaceae	Clostridioides	CRISPR-associated protein Cas4	cas4	pfam01930	CAS-I			
162399..164721	-	KPI49097.1	JPPA01000086.1	Clostridioides difficile	Bacteria	Firmicutes	Clostridia	Clostridiales	Peptostreptococcaceae	Clostridioides	CRISPR-associated protein Cas3	cas3HD,cas3	cd09641,cd09639	CAS-I			
164731..165433	-	KPI49098.1	JPPA01000086.1	Clostridioides difficile	Bacteria	Firmicutes	Clostridia	Clostridiales	Peptostreptococcaceae	Clostridioides	CRISPR-associated protein Cas5	cas5	cd09658	CAS-I-B			
165434..166349	-	KPI49099.1	JPPA01000086.1	Clostridioides difficile	Bacteria	Firmicutes	Clostridia	Clostridiales	Peptostreptococcaceae	Clostridioides	CRISPR-associated protein DevR	cas7	cd09687	CAS-I-B			
166350..168219	-	KPI49100.1	JPPA01000086.1	Clostridioides difficile	Bacteria	Firmicutes	Clostridia	Clostridiales	Peptostreptococcaceae	Clostridioides	CRISPR-associated protein	cas8b2	cd09665	CAS-I-B			
168224..168962	-	KPI49101.1	JPPA01000086.1	Clostridioides difficile	Bacteria	Firmicutes	Clostridia	Clostridiales	Peptostreptococcaceae	Clostridioides	CRISPR-associated protein Cas6	cas6	pfam01881	CAS-I,CAS-III			
169039..169927	-	KPI49102.1	JPPA01000086.1	Clostridioides difficile	Bacteria	Firmicutes	Clostridia	Clostridiales	Peptostreptococcaceae	Clostridioides	DNA-binding protein	WYL	pfam13280	CAS-I,CAS-III			

498 CAS-I-B | SJP84228.1

259660..261273	array		FUNQ01000002.1	Clostridioides difficile	Bacteria	Firmicutes	Clostridia	Clostridiales	Peptostreptococcaceae	Clostridioides		CRISPR array			25	37	29
261456..261723	-	SJP84228.1	FUNQ01000002.1	Clostridioides difficile	Bacteria	Firmicutes	Clostridia	Clostridiales	Peptostreptococcaceae	Clostridioides	CRISPR-associated endoribonuclease Cas2	cas2	cd09725	CAS-I,CAS-II,CAS-III,CAS-V			
261725..262706	-	SJP84235.1	FUNQ01000002.1	Clostridioides difficile	Bacteria	Firmicutes	Clostridia	Clostridiales	Peptostreptococcaceae	Clostridioides	CRISPR-associated endonuclease Cas1, subtype I-B/HMARI/TNEAP	cas1	cd09722	CAS-I-B			
262708..263278	-	SJP84245.1	FUNQ01000002.1	Clostridioides difficile	Bacteria	Firmicutes	Clostridia	Clostridiales	Peptostreptococcaceae	Clostridioides	CRISPR-associated protein Cas4	cas4	pfam01930	CAS-I			
263274..265596	-	SJP84254.1	FUNQ01000002.1	Clostridioides difficile	Bacteria	Firmicutes	Clostridia	Clostridiales	Peptostreptococcaceae	Clostridioides	helicase Cas3	cas3HD,cas3	cd09641,cd09639	CAS-I			
265606..266308	-	SJP84265.1	FUNQ01000002.1	Clostridioides difficile	Bacteria	Firmicutes	Clostridia	Clostridiales	Peptostreptococcaceae	Clostridioides	Uncharacterized protein predicted to be involved in DNA repair (RAMP superfamily)	cas5	cd09658	CAS-I-B			
266309..267224	-	SJP84272.1	FUNQ01000002.1	Clostridioides difficile	Bacteria	Firmicutes	Clostridia	Clostridiales	Peptostreptococcaceae	Clostridioides	CRISPR-associated protein Cas7/Cst2/DevR, subtype I-B/TNEAP	cas7	cd09687	CAS-I-B			
267225..269076	-	SJP84283.1	FUNQ01000002.1	Clostridioides difficile	Bacteria	Firmicutes	Clostridia	Clostridiales	Peptostreptococcaceae	Clostridioides	CRISPR-associated protein Cas8a1/Cst1, subtype I-B/TNEAP	cas8b2	cd09665	CAS-I-B			
269081..269819	-	SJP84291.1	FUNQ01000002.1	Clostridioides difficile	Bacteria	Firmicutes	Clostridia	Clostridiales	Peptostreptococcaceae	Clostridioides	Uncharacterized protein predicted to be involved in DNA repair (RAMP superfamily)	cas6	pfam01881	CAS-I,CAS-III			
269896..270784	-	SJP84303.1	FUNQ01000002.1	Clostridioides difficile	Bacteria	Firmicutes	Clostridia	Clostridiales	Peptostreptococcaceae	Clostridioides	Uncharacterised protein	WYL	pfam13280	CAS-I,CAS-III			

500 CAS-I-B | CBE06405.1

3325155..3326772	array		FN545816.1	Clostridioides difficile R20291	Bacteria	Firmicutes	Clostridia	Clostridiales	Peptostreptococcaceae	Clostridioides		CRISPR array			25	37	29
3326955..3327234	-	CBE06405.1	FN545816.1	Clostridioides difficile R20291	Bacteria	Firmicutes	Clostridia	Clostridiales	Peptostreptococcaceae	Clostridioides	conserved hypothetical protein	cas2	cd09725	CAS-I,CAS-II,CAS-III,CAS-V			
3327224..3328205	-	CBE06407.1	FN545816.1	Clostridioides difficile R20291	Bacteria	Firmicutes	Clostridia	Clostridiales	Peptostreptococcaceae	Clostridioides	conserved hypothetical protein	cas1	cd09722	CAS-I-B			
3328207..3328759	-	CBE06408.1	FN545816.1	Clostridioides difficile R20291	Bacteria	Firmicutes	Clostridia	Clostridiales	Peptostreptococcaceae	Clostridioides	conserved hypothetical protein	cas4	pfam01930	CAS-I			
3328773..3331095	-	CBE06410.1	FN545816.1	Clostridioides difficile R20291	Bacteria	Firmicutes	Clostridia	Clostridiales	Peptostreptococcaceae	Clostridioides	conserved hypothetical protein	cas3	COG1203	CAS-I			
3331105..3331807	-	CBE06412.1	FN545816.1	Clostridioides difficile R20291	Bacteria	Firmicutes	Clostridia	Clostridiales	Peptostreptococcaceae	Clostridioides	conserved hypothetical protein	cas5	cd09658	CAS-I-B			
3331808..3332723	-	CBE06414.1	FN545816.1	Clostridioides difficile R20291	Bacteria	Firmicutes	Clostridia	Clostridiales	Peptostreptococcaceae	Clostridioides	conserved hypothetical protein	cas7	cd09687	CAS-I-B			
3332724..3334602	-	CBE06415.1	FN545816.1	Clostridioides difficile R20291	Bacteria	Firmicutes	Clostridia	Clostridiales	Peptostreptococcaceae	Clostridioides	hypothetical protein	cas8b2	cd09665	CAS-I-B			
3334607..3335345	-	CBE06417.1	FN545816.1	Clostridioides difficile R20291	Bacteria	Firmicutes	Clostridia	Clostridiales	Peptostreptococcaceae	Clostridioides	conserved hypothetical protein	cas6	pfam01881	CAS-I,CAS-III			
3335422..3336340	-	CBE06419.1	FN545816.1	Clostridioides difficile R20291	Bacteria	Firmicutes	Clostridia	Clostridiales	Peptostreptococcaceae	Clostridioides	conserved hypothetical protein	WYL	pfam13280	CAS-I,CAS-III			

501 CAS-I-B | OFU27205.1

16388..17276	+	OFU27206.1	KV785056.1	Clostridium sp. HMSC19B10	Bacteria	Firmicutes	Clostridia	Clostridiales	Clostridiaceae	Clostridium	DNA-binding protein	WYL	pfam13280	CAS-I,CAS-III			
17353..18091	+	OFU27198.1	KV785056.1	Clostridium sp. HMSC19B10	Bacteria	Firmicutes	Clostridia	Clostridiales	Clostridiaceae	Clostridium	CRISPR-associated endoribonuclease Cas6	cas6	pfam01881	CAS-I,CAS-III			
18096..19974	+	OFU27199.1	KV785056.1	Clostridium sp. HMSC19B10	Bacteria	Firmicutes	Clostridia	Clostridiales	Clostridiaceae	Clostridium	type I-B CRISPR-associated protein Cas8b1/Cst1	cas8b2	cd09665	CAS-I-B			
19975..20890	+	OFU27200.1	KV785056.1	Clostridium sp. HMSC19B10	Bacteria	Firmicutes	Clostridia	Clostridiales	Clostridiaceae	Clostridium	type I-B CRISPR-associated protein Cas7/Cst2/DevR	cas7	cd09687	CAS-I-B			
20891..21593	+	OFU27201.1	KV785056.1	Clostridium sp. HMSC19B10	Bacteria	Firmicutes	Clostridia	Clostridiales	Clostridiaceae	Clostridium	type I-B CRISPR-associated protein Cas5	cas5	cd09658	CAS-I-B			
21603..23925	+	OFU27202.1	KV785056.1	Clostridium sp. HMSC19B10	Bacteria	Firmicutes	Clostridia	Clostridiales	Clostridiaceae	Clostridium	CRISPR-associated helicase/endonuclease Cas3	cas3	COG1203	CAS-I			
23972..24491	+	OFU27203.1	KV785056.1	Clostridium sp. HMSC19B10	Bacteria	Firmicutes	Clostridia	Clostridiales	Clostridiaceae	Clostridium	CRISPR-associated protein Cas4	cas4	pfam01930	CAS-I			
24493..25474	+	OFU27204.1	KV785056.1	Clostridium sp. HMSC19B10	Bacteria	Firmicutes	Clostridia	Clostridiales	Clostridiaceae	Clostridium	subtype I-B CRISPR-associated endonuclease Cas1	cas1	cd09722	CAS-I-B			
25476..25743	+	OFU27205.1	KV785056.1	Clostridium sp. HMSC19B10	Bacteria	Firmicutes	Clostridia	Clostridiales	Clostridiaceae	Clostridium	CRISPR-associated endonuclease Cas2	cas2	cd09725	CAS-I,CAS-II,CAS-III,CAS-V			
25927..27511	array		KV785056.1	Clostridium sp. HMSC19B10	Bacteria	Firmicutes	Clostridia	Clostridiales	Clostridiaceae	Clostridium		CRISPR array			25	35	29

502 CAS-I-B | KYO67328.1

101329..102058	+	KYO67321.1	LOHZ01000022.1	Thermovenabulum gondwanense	Bacteria	Firmicutes	Clostridia	Thermoanaerobacterales	Thermoanaerobacterales Family III. Incertae Sedis	Thermovenabulum	hypothetical protein	cas6	pfam01881	CAS-I,CAS-III			
102058..103687	+	KYO67322.1	LOHZ01000022.1	Thermovenabulum gondwanense	Bacteria	Firmicutes	Clostridia	Thermoanaerobacterales	Thermoanaerobacterales Family III. Incertae Sedis	Thermovenabulum	hypothetical protein	cas8b2	cd09665	CAS-I-B			
103688..104633	+	KYO67323.1	LOHZ01000022.1	Thermovenabulum gondwanense	Bacteria	Firmicutes	Clostridia	Thermoanaerobacterales	Thermoanaerobacterales Family III. Incertae Sedis	Thermovenabulum	CRISPR-associated protein Cas7/Cst2/DevR	cas7	cd09687	CAS-I-B			
104635..105382	+	KYO67324.1	LOHZ01000022.1	Thermovenabulum gondwanense	Bacteria	Firmicutes	Clostridia	Thermoanaerobacterales	Thermoanaerobacterales Family III. Incertae Sedis	Thermovenabulum	hypothetical protein	cas5	cd09658	CAS-I-B			
105371..107720	+	KYO67325.1	LOHZ01000022.1	Thermovenabulum gondwanense	Bacteria	Firmicutes	Clostridia	Thermoanaerobacterales	Thermoanaerobacterales Family III. Incertae Sedis	Thermovenabulum	CRISPR-associated endonuclease/helicase Cas3	cas3HD,cas3	cd09641,cd09639	CAS-I			
107767..108286	+	KYO67326.1	LOHZ01000022.1	Thermovenabulum gondwanense	Bacteria	Firmicutes	Clostridia	Thermoanaerobacterales	Thermoanaerobacterales Family III. Incertae Sedis	Thermovenabulum	hypothetical protein	cas4	pfam01930	CAS-I			
108287..109271	+	KYO67327.1	LOHZ01000022.1	Thermovenabulum gondwanense	Bacteria	Firmicutes	Clostridia	Thermoanaerobacterales	Thermoanaerobacterales Family III. Incertae Sedis	Thermovenabulum	CRISPR-associated endonuclease Cas1	cas1	cd09722	CAS-I-B			
109272..109536	+	KYO67328.1	LOHZ01000022.1	Thermovenabulum gondwanense	Bacteria	Firmicutes	Clostridia	Thermoanaerobacterales	Thermoanaerobacterales Family III. Incertae Sedis	Thermovenabulum	CRISPR-associated endoribonuclease Cas2	cas2	cd09725	CAS-I,CAS-II,CAS-III,CAS-V			
109711..122243	array		LOHZ01000022.1	Thermovenabulum gondwanense	Bacteria	Firmicutes	Clostridia	Thermoanaerobacterales	Thermoanaerobacterales Family III. Incertae Sedis	Thermovenabulum		CRISPR array			187	38	29
122403..124353	array		LOHZ01000022.1	Thermovenabulum gondwanense	Bacteria	Firmicutes	Clostridia	Thermoanaerobacterales	Thermoanaerobacterales Family III. Incertae Sedis	Thermovenabulum		CRISPR array			30	37	29
124504..124780	-	KYO67329.1	LOHZ01000022.1	Thermovenabulum gondwanense	Bacteria	Firmicutes	Clostridia	Thermoanaerobacterales	Thermoanaerobacterales Family III. Incertae Sedis	Thermovenabulum	hypothetical protein						
124831..129101	array		LOHZ01000022.1	Thermovenabulum gondwanense	Bacteria	Firmicutes	Clostridia	Thermoanaerobacterales	Thermoanaerobacterales Family III. Incertae Sedis	Thermovenabulum		CRISPR array			65	37	29
129191..129440	-	KYO67330.1	LOHZ01000022.1	Thermovenabulum gondwanense	Bacteria	Firmicutes	Clostridia	Thermoanaerobacterales	Thermoanaerobacterales Family III. Incertae Sedis	Thermovenabulum	hypothetical protein						
129436..130000	-	KYO67331.1	LOHZ01000022.1	Thermovenabulum gondwanense	Bacteria	Firmicutes	Clostridia	Thermoanaerobacterales	Thermoanaerobacterales Family III. Incertae Sedis	Thermovenabulum	hypothetical protein						
130004..133954	array		LOHZ01000022.1	Thermovenabulum gondwanense	Bacteria	Firmicutes	Clostridia	Thermoanaerobacterales	Thermoanaerobacterales Family III. Incertae Sedis	Thermovenabulum		CRISPR array			60	38	28
134138..135674	+	KYO67332.1	LOHZ01000022.1	Thermovenabulum gondwanense	Bacteria	Firmicutes	Clostridia	Thermoanaerobacterales	Thermoanaerobacterales Family III. Incertae Sedis	Thermovenabulum	hypothetical protein						
135660..136392	+	KYO67333.1	LOHZ01000022.1	Thermovenabulum gondwanense	Bacteria	Firmicutes	Clostridia	Thermoanaerobacterales	Thermoanaerobacterales Family III. Incertae Sedis	Thermovenabulum	Chromosomal replication initiator protein DnaA						
136479..138029	array		LOHZ01000022.1	Thermovenabulum gondwanense	Bacteria	Firmicutes	Clostridia	Thermoanaerobacterales	Thermoanaerobacterales Family III. Incertae Sedis	Thermovenabulum		CRISPR array			24	37	29
138156..138986	array		LOHZ01000022.1	Thermovenabulum gondwanense	Bacteria	Firmicutes	Clostridia	Thermoanaerobacterales	Thermoanaerobacterales Family III. Incertae Sedis	Thermovenabulum		CRISPR array			13	37	29
139072..141694	array		LOHZ01000022.1	Thermovenabulum gondwanense	Bacteria	Firmicutes	Clostridia	Thermoanaerobacterales	Thermoanaerobacterales Family III. Incertae Sedis	Thermovenabulum		CRISPR array			40	38	28

503 CAS-I-B | KIE48477.1

4258..5176	+	KIE48458.1	AYSO01000007.1	Clostridium argentinense CDC 2741	Bacteria	Firmicutes	Clostridia	Clostridiales	Clostridiaceae	Clostridium	transcriptional regulator family protein	WYL	COG2378	CAS-I,CAS-III			
5404..5665	+	KIE48492.1	AYSO01000007.1	Clostridium argentinense CDC 2741	Bacteria	Firmicutes	Clostridia	Clostridiales	Clostridiaceae	Clostridium	transposase family protein						
5800..6529	+	KIE48470.1	AYSO01000007.1	Clostridium argentinense CDC 2741	Bacteria	Firmicutes	Clostridia	Clostridiales	Clostridiaceae	Clostridium	CRISPR-associated endoribonuclease Cas6	cas6	COG1583	CAS-I,CAS-III,CAS-IV			
6541..8350	+	KIE48482.1	AYSO01000007.1	Clostridium argentinense CDC 2741	Bacteria	Firmicutes	Clostridia	Clostridiales	Clostridiaceae	Clostridium	CRISPR-associated family protein	cas8b2	cd09665	CAS-I-B			
8342..9251	+	KIE48461.1	AYSO01000007.1	Clostridium argentinense CDC 2741	Bacteria	Firmicutes	Clostridia	Clostridiales	Clostridiaceae	Clostridium	CRISPR-associated autoregulator DevR family protein	cas7	cd09687	CAS-I-B			
9256..9904	+	KIE48494.1	AYSO01000007.1	Clostridium argentinense CDC 2741	Bacteria	Firmicutes	Clostridia	Clostridiales	Clostridiaceae	Clostridium	CRISPR-associated Cas5 domain protein	cas5	cd09658	CAS-I-B			
10060..12232	+	KIE48457.1	AYSO01000007.1	Clostridium argentinense CDC 2741	Bacteria	Firmicutes	Clostridia	Clostridiales	Clostridiaceae	Clostridium	CRISPR-associated endonuclease Cas3-HD	cas3	cd09639	CAS-I			
12243..12771	+	KIE48463.1	AYSO01000007.1	Clostridium argentinense CDC 2741	Bacteria	Firmicutes	Clostridia	Clostridiales	Clostridiaceae	Clostridium	CRISPR-associated protein Cas4	cas4	pfam01930	CAS-I			
12763..13747	+	KIE48478.1	AYSO01000007.1	Clostridium argentinense CDC 2741	Bacteria	Firmicutes	Clostridia	Clostridiales	Clostridiaceae	Clostridium	CRISPR-associated endonuclease Cas1	cas1	cd09722	CAS-I-B			
13750..14014	+	KIE48477.1	AYSO01000007.1	Clostridium argentinense CDC 2741	Bacteria	Firmicutes	Clostridia	Clostridiales	Clostridiaceae	Clostridium	CRISPR-associated endoribonuclease Cas2	cas2	cd09725	CAS-I,CAS-II,CAS-III,CAS-V			
14200..16310	array		AYSO01000007.1	Clostridium argentinense CDC 2741	Bacteria	Firmicutes	Clostridia	Clostridiales	Clostridiaceae	Clostridium		CRISPR array			33	36	29

504 CAS-I-B | ABG85728.1

1044986..1045940	+	ABG85314.1	CP000312.1	Clostridium perfringens SM101	Bacteria	Firmicutes	Clostridia	Clostridiales	Clostridiaceae	Clostridium	conserved hypothetical protein	WYL	COG2378	CAS-I,CAS-III			
1046046..1046568	+	ABG86009.1	CP000312.1	Clostridium perfringens SM101	Bacteria	Firmicutes	Clostridia	Clostridiales	Clostridiaceae	Clostridium	CRISPR-associated protein Cas4	cas4	pfam01930	CAS-I			
1046569..1047130	+	ABG86928.1	CP000312.1	Clostridium perfringens SM101	Bacteria	Firmicutes	Clostridia	Clostridiales	Clostridiaceae	Clostridium	CRISPR-associated protein Cas1	cas1	cd09722	CAS-I-B			
1047598..1047862	+	ABG85728.1	CP000312.1	Clostridium perfringens SM101	Bacteria	Firmicutes	Clostridia	Clostridiales	Clostridiaceae	Clostridium	CRISPR-associated protein Cas2	cas2	cd09725	CAS-I,CAS-II,CAS-III,CAS-V			
1048253..1048976	+	ABG86309.1	CP000312.1	Clostridium perfringens SM101	Bacteria	Firmicutes	Clostridia	Clostridiales	Clostridiaceae	Clostridium	CRISPR-associated protein Cas6	cas6	cd09652	CAS-I,CAS-III			
1048999..1050343	+	ABG85850.1	CP000312.1	Clostridium perfringens SM101	Bacteria	Firmicutes	Clostridia	Clostridiales	Clostridiaceae	Clostridium	putative CRISPR-associated protein Csx8	cas8b2	cd09744	CAS-I-B			
1050342..1051230	+	ABG86299.1	CP000312.1	Clostridium perfringens SM101	Bacteria	Firmicutes	Clostridia	Clostridiales	Clostridiaceae	Clostridium	CRISPR-associated regulatory protein, DevR family	cas7	cd09687	CAS-I-B			
1051383..1052328	+	ABG87443.1	CP000312.1	Clostridium perfringens SM101	Bacteria	Firmicutes	Clostridia	Clostridiales	Clostridiaceae	Clostridium	hypothetical protein	cas5	cd09658	CAS-I-B			
1052340..1054551	+	ABG86645.1	CP000312.1	Clostridium perfringens SM101	Bacteria	Firmicutes	Clostridia	Clostridiales	Clostridiaceae	Clostridium	CRISPR-associated helicase Cas3 domain protein	cas3	COG1203	CAS-I			
1054741..1058679	array		CP000312.1	Clostridium perfringens SM101	Bacteria	Firmicutes	Clostridia	Clostridiales	Clostridiaceae	Clostridium		CRISPR array			61	36	29

505 CAS-I-B | PRR80308.1

506..5180	array		PVXP01000087.1	Clostridium luticellarii	Bacteria	Firmicutes	Clostridia	Clostridiales	Clostridiaceae	Clostridium		CRISPR array			72	36	29
5365..5629	-	PRR80308.1	PVXP01000087.1	Clostridium luticellarii	Bacteria	Firmicutes	Clostridia	Clostridiales	Clostridiaceae	Clostridium	CRISPR-associated endoribonuclease Cas2	cas2	cd09725	CAS-I,CAS-II,CAS-III,CAS-V			
5630..6614	-	PRR80309.1	PVXP01000087.1	Clostridium luticellarii	Bacteria	Firmicutes	Clostridia	Clostridiales	Clostridiaceae	Clostridium	CRISPR-associated endonuclease Cas1	cas1	cd09722	CAS-I-B			
6616..7135	-	PRR80310.1	PVXP01000087.1	Clostridium luticellarii	Bacteria	Firmicutes	Clostridia	Clostridiales	Clostridiaceae	Clostridium	hypothetical protein	cas4	pfam01930	CAS-I			
7139..9347	-	PRR80311.1	PVXP01000087.1	Clostridium luticellarii	Bacteria	Firmicutes	Clostridia	Clostridiales	Clostridiaceae	Clostridium	CRISPR-associated nuclease/helicase Cas3	cas3HD,cas3	cd09641,COG1203	CAS-I			
9374..10058	-	PRR80312.1	PVXP01000087.1	Clostridium luticellarii	Bacteria	Firmicutes	Clostridia	Clostridiales	Clostridiaceae	Clostridium	CRISPR-associated protein Cas5	cas5	cd09658	CAS-I-B			
10054..10963	-	PRR80313.1	PVXP01000087.1	Clostridium luticellarii	Bacteria	Firmicutes	Clostridia	Clostridiales	Clostridiaceae	Clostridium	hypothetical protein	cas7	cd09687	CAS-I-B			
10955..11273	-	PRR80314.1	PVXP01000087.1	Clostridium luticellarii	Bacteria	Firmicutes	Clostridia	Clostridiales	Clostridiaceae	Clostridium	hypothetical protein	cas8b2	cd09665	CAS-I-B			

506 CAS-I-B | AYD40188.1

1351562..1352498	+	AYD40181.1	CP032416.1	Clostridium sp. JN500901	Bacteria	Firmicutes	Clostridia	Clostridiales	Clostridiaceae	Clostridium	transcriptional regulator	WYL	pfam13280	CAS-I,CAS-III			
1352618..1353350	+	AYD40182.1	CP032416.1	Clostridium sp. JN500901	Bacteria	Firmicutes	Clostridia	Clostridiales	Clostridiaceae	Clostridium	CRISPR-associated endoribonuclease Cas6	cas6	COG1583	CAS-I,CAS-III,CAS-IV			
1353365..1355027	+	AYD40183.1	CP032416.1	Clostridium sp. JN500901	Bacteria	Firmicutes	Clostridia	Clostridiales	Clostridiaceae	Clostridium	type I-B CRISPR-associated protein Cas8b1/Cst1	cas8b2	cd09665	CAS-I-B			
1355028..1355904	+	AYD40184.1	CP032416.1	Clostridium sp. JN500901	Bacteria	Firmicutes	Clostridia	Clostridiales	Clostridiaceae	Clostridium	type I-B CRISPR-associated protein Cas7/Cst2/DevR	cas7	cd09687	CAS-I-B			
1355912..1356656	+	AYD40185.1	CP032416.1	Clostridium sp. JN500901	Bacteria	Firmicutes	Clostridia	Clostridiales	Clostridiaceae	Clostridium	CRISPR-associated protein Cas5	cas5	cd09658	CAS-I-B			
1356776..1359044	+	AYD40186.1	CP032416.1	Clostridium sp. JN500901	Bacteria	Firmicutes	Clostridia	Clostridiales	Clostridiaceae	Clostridium	CRISPR-associated helicase Cas3'	cas3HD,cas3	cd09641,COG1203	CAS-I			
1359054..1359570	+	AYD40187.1	CP032416.1	Clostridium sp. JN500901	Bacteria	Firmicutes	Clostridia	Clostridiales	Clostridiaceae	Clostridium	CRISPR-associated protein Cas4	cas4	pfam01930	CAS-I			
1359575..1360559	+	AYD41437.1	CP032416.1	Clostridium sp. JN500901	Bacteria	Firmicutes	Clostridia	Clostridiales	Clostridiaceae	Clostridium	type I-B CRISPR-associated endonuclease Cas1	cas1	cd09722	CAS-I-B			
1360560..1360824	+	AYD40188.1	CP032416.1	Clostridium sp. JN500901	Bacteria	Firmicutes	Clostridia	Clostridiales	Clostridiaceae	Clostridium	CRISPR-associated endonuclease Cas2	cas2	cd09725	CAS-I,CAS-II,CAS-III,CAS-V			
1361009..1363123	array		CP032416.1	Clostridium sp. JN500901	Bacteria	Firmicutes	Clostridia	Clostridiales	Clostridiaceae	Clostridium		CRISPR array			33	36	29

508 CAS-I-B | OOM45176.1

14352..17079	array		LZZF01000058.1	Clostridium beijerinckii	Bacteria	Firmicutes	Clostridia	Clostridiales	Clostridiaceae	Clostridium		CRISPR array			43	36	28
17260..17524	-	OOM45176.1	LZZF01000058.1	Clostridium beijerinckii	Bacteria	Firmicutes	Clostridia	Clostridiales	Clostridiaceae	Clostridium	CRISPR-associated endoribonuclease Cas2	cas2	cd09725	CAS-I,CAS-II,CAS-III,CAS-V			
17525..18509	-	OOM45177.1	LZZF01000058.1	Clostridium beijerinckii	Bacteria	Firmicutes	Clostridia	Clostridiales	Clostridiaceae	Clostridium	CRISPR-associated endonuclease Cas1	cas1	cd09722	CAS-I-B			

18514..19030	-	OOM45178.1	LZZF01000058.1	Clostridium beijerinckii	Bacteria	Firmicutes	Clostridia	Clostridiales	Clostridiaceae	Clostridium	hypothetical protein	cas4	pfam01930	CAS-I			
19042..21304	-	OOM45179.1	LZZF01000058.1	Clostridium beijerinckii	Bacteria	Firmicutes	Clostridia	Clostridiales	Clostridiaceae	Clostridium	CRISPR-associated nuclease/helicase Cas3	cas3HD,cas3	cd09641,COG1203	CAS-I			
21342..22077	-	OOM45180.1	LZZF01000058.1	Clostridium beijerinckii	Bacteria	Firmicutes	Clostridia	Clostridiales	Clostridiaceae	Clostridium	CRISPR-associated protein Cas5	cas5	cd09658	CAS-I-B			
22085..22961	-	OOM45181.1	LZZF01000058.1	Clostridium beijerinckii	Bacteria	Firmicutes	Clostridia	Clostridiales	Clostridiaceae	Clostridium	CRISPR-associated negative autoregulator DevR/Csa2	cas7	cd09687	CAS-I-B			
22981..24670	-	OOM45182.1	LZZF01000058.1	Clostridium beijerinckii	Bacteria	Firmicutes	Clostridia	Clostridiales	Clostridiaceae	Clostridium	CRISPR-associated protein	cas8b2	cd09665	CAS-I-B			
24741..25482	-	OOM45183.1	LZZF01000058.1	Clostridium beijerinckii	Bacteria	Firmicutes	Clostridia	Clostridiales	Clostridiaceae	Clostridium	CRISPR associated protein Cas6	cas6	pfam01881	CAS-I,CAS-III			
25649..26591	-	OOM45184.1	LZZF01000058.1	Clostridium beijerinckii	Bacteria	Firmicutes	Clostridia	Clostridiales	Clostridiaceae	Clostridium	HTH domain protein	WYL	COG2378	CAS-I,CAS-III			

509 CAS-I-B | AYE33254.1

356529..358370	array		CP023671.1	Clostridium septicum	Bacteria	Firmicutes	Clostridia	Clostridiales	Clostridiaceae	Clostridium		CRISPR array			29	36	28
358508..359729	-	AYE33252.1	CP023671.1	Clostridium septicum	Bacteria	Firmicutes	Clostridia	Clostridiales	Clostridiaceae	Clostridium	IS256 family transposase						
359797..365277	array		CP023671.1	Clostridium septicum	Bacteria	Firmicutes	Clostridia	Clostridiales	Clostridiaceae	Clostridium		CRISPR array			85	36	28
365456..366677	-	AYE33253.1	CP023671.1	Clostridium septicum	Bacteria	Firmicutes	Clostridia	Clostridiales	Clostridiaceae	Clostridium	IS256 family transposase						
366769..368289	array		CP023671.1	Clostridium septicum	Bacteria	Firmicutes	Clostridia	Clostridiales	Clostridiaceae	Clostridium		CRISPR array			24	36	28
368470..368734	-	AYE33254.1	CP023671.1	Clostridium septicum	Bacteria	Firmicutes	Clostridia	Clostridiales	Clostridiaceae	Clostridium	CRISPR-associated endonuclease Cas2	cas2	cd09725	CAS-I,CAS-II,CAS-III,CAS-V			
368735..369719	-	AYE33255.1	CP023671.1	Clostridium septicum	Bacteria	Firmicutes	Clostridia	Clostridiales	Clostridiaceae	Clostridium	subtype I-B CRISPR-associated endonuclease Cas1	cas1	cd09722	CAS-I-B			
372549..373284	-	AYE33256.1	CP023671.1	Clostridium septicum	Bacteria	Firmicutes	Clostridia	Clostridiales	Clostridiaceae	Clostridium	type I-B CRISPR-associated protein Cas5	cas5	cd09658	CAS-I-B			
373293..374169	-	AYE33257.1	CP023671.1	Clostridium septicum	Bacteria	Firmicutes	Clostridia	Clostridiales	Clostridiaceae	Clostridium	type I-B CRISPR-associated protein Cas7/Cst2/DevR	cas7	cd09687	CAS-I-B			
374168..375860	-	AYE33258.1	CP023671.1	Clostridium septicum	Bacteria	Firmicutes	Clostridia	Clostridiales	Clostridiaceae	Clostridium	type I-B CRISPR-associated protein Cas8b1/Cst1	cas8b2	cd09665	CAS-I-B			
375871..376606	-	AYE33259.1	CP023671.1	Clostridium septicum	Bacteria	Firmicutes	Clostridia	Clostridiales	Clostridiaceae	Clostridium	CRISPR-associated endoribonuclease Cas6	cas6	pfam01881	CAS-I,CAS-III			
376730..377669	-	AYE33260.1	CP023671.1	Clostridium septicum	Bacteria	Firmicutes	Clostridia	Clostridiales	Clostridiaceae	Clostridium	WYL domain-containing protein	WYL	COG2378	CAS-I,CAS-III			

510 CAS-I-B | QAS61425.1

2545844..2551817	array		CP034358.1	Clostridium septicum	Bacteria	Firmicutes	Clostridia	Clostridiales	Clostridiaceae	Clostridium		CRISPR array			93	36	28
2551998..2552262	-	QAS61425.1	CP034358.1	Clostridium septicum	Bacteria	Firmicutes	Clostridia	Clostridiales	Clostridiaceae	Clostridium	CRISPR-associated endonuclease Cas2	cas2	cd09725	CAS-I,CAS-II,CAS-III,CAS-V			
2552263..2553247	-	QAS61426.1	CP034358.1	Clostridium septicum	Bacteria	Firmicutes	Clostridia	Clostridiales	Clostridiaceae	Clostridium	type I-B CRISPR-associated endonuclease Cas1	cas1	cd09722	CAS-I-B			
2556077..2556812	-	QAS61427.1	CP034358.1	Clostridium septicum	Bacteria	Firmicutes	Clostridia	Clostridiales	Clostridiaceae	Clostridium	type I-B CRISPR-associated protein Cas5	cas5	cd09658	CAS-I-B			
2556821..2557697	-	QAS61428.1	CP034358.1	Clostridium septicum	Bacteria	Firmicutes	Clostridia	Clostridiales	Clostridiaceae	Clostridium	type I-B CRISPR-associated protein Cas7/Cst2/DevR	cas7	cd09687	CAS-I-B			
2557696..2559388	-	QAS61429.1	CP034358.1	Clostridium septicum	Bacteria	Firmicutes	Clostridia	Clostridiales	Clostridiaceae	Clostridium	type I-B CRISPR-associated protein Cas8b1/Cst1	cas8b2	cd09665	CAS-I-B			
2559399..2560134	-	QAS61430.1	CP034358.1	Clostridium septicum	Bacteria	Firmicutes	Clostridia	Clostridiales	Clostridiaceae	Clostridium	CRISPR-associated endoribonuclease Cas6	cas6	pfam01881	CAS-I,CAS-III			
2560258..2561197	-	QAS61431.1	CP034358.1	Clostridium septicum	Bacteria	Firmicutes	Clostridia	Clostridiales	Clostridiaceae	Clostridium	WYL domain-containing protein	WYL	COG2378	CAS-I,CAS-III			

511 CAS-I-B | ENY99756.1

89579..91373	array		KB850958.1	Clostridium colicanis 209318	Bacteria	Firmicutes	Clostridia	Clostridiales	Clostridiaceae	Clostridium		CRISPR array			28	36	29
91562..91823	-	ENY99756.1	KB850958.1	Clostridium colicanis 209318	Bacteria	Firmicutes	Clostridia	Clostridiales	Clostridiaceae	Clostridium	CRISPR-associated endoribonuclease cas2	cas2	cd09725	CAS-I,CAS-II,CAS-III,CAS-V			
91823..92807	-	ENY99757.1	KB850958.1	Clostridium colicanis 209318	Bacteria	Firmicutes	Clostridia	Clostridiales	Clostridiaceae	Clostridium	CRISPR-associated endonuclease cas1, subtype I-b/hmari/tneap	cas1	cd09722	CAS-I-B			
92811..93330	-	ENY99758.1	KB850958.1	Clostridium colicanis 209318	Bacteria	Firmicutes	Clostridia	Clostridiales	Clostridiaceae	Clostridium	CRISPR-associated protein cas4	cas4	pfam01930	CAS-I			
93396..95682	-	ENY99759.1	KB850958.1	Clostridium colicanis 209318	Bacteria	Firmicutes	Clostridia	Clostridiales	Clostridiaceae	Clostridium	CRISPR-associated helicase cas3	cas3	cd09639	CAS-I			
95808..96546	-	ENY99760.1	KB850958.1	Clostridium colicanis 209318	Bacteria	Firmicutes	Clostridia	Clostridiales	Clostridiaceae	Clostridium	CRISPR-associated protein cas5, subtype I-b/tneap	cas5	cd09658	CAS-I-B			
96555..97434	-	ENY99761.1	KB850958.1	Clostridium colicanis 209318	Bacteria	Firmicutes	Clostridia	Clostridiales	Clostridiaceae	Clostridium	CRISPR-associated protein cas7/cst2/devr, subtype I-b/tneap	cas7	cd09687	CAS-I-B			
97423..99109	-	ENY99762.1	KB850958.1	Clostridium colicanis 209318	Bacteria	Firmicutes	Clostridia	Clostridiales	Clostridiaceae	Clostridium	CRISPR-associated protein cas8a1/cst1, subtype I-b/tneap	cas8b2,cas8b2	cd09754,cd09665	CAS-I-B			
99123..99852	-	ENY99763.1	KB850958.1	Clostridium colicanis 209318	Bacteria	Firmicutes	Clostridia	Clostridiales	Clostridiaceae	Clostridium	CRISPR-associated endoribonuclease cas6	cas6	cd09652	CAS-I,CAS-III			
99978..100917	-	ENY99764.1	KB850958.1	Clostridium colicanis 209318	Bacteria	Firmicutes	Clostridia	Clostridiales	Clostridiaceae	Clostridium	hypothetical protein	WYL	pfam13280	CAS-I,CAS-III			

512 CAS-I-B | EES47852.1

1677054..1677990	+	EES47909.1	ACSC01000002.1	Clostridium botulinum E1 str. 'BoNT E Beluga'	Bacteria	Firmicutes	Clostridia	Clostridiales	Clostridiaceae	Clostridium	HTH domain family protein	WYL	pfam13280	CAS-I,CAS-III			
1678077..1678815	+	EES48500.1	ACSC01000002.1	Clostridium botulinum E1 str. 'BoNT E Beluga'	Bacteria	Firmicutes	Clostridia	Clostridiales	Clostridiaceae	Clostridium	CRISPR-associated protein Cas6	cas6	pfam01881	CAS-I,CAS-III			
1678826..1680512	+	EES51331.1	ACSC01000002.1	Clostridium botulinum E1 str. 'BoNT E Beluga'	Bacteria	Firmicutes	Clostridia	Clostridiales	Clostridiaceae	Clostridium	CRISPR-associated cxxc_cxxc protein Cst1	cas8b2,cas8b2	cd09754,cd09665	CAS-I-B			
1680514..1681396	+	EES49840.1	ACSC01000002.1	Clostridium botulinum E1 str. 'BoNT E Beluga'	Bacteria	Firmicutes	Clostridia	Clostridiales	Clostridiaceae	Clostridium	CRISPR-associated regulatory protein, DevR family	cas7	cd09687	CAS-I-B			
1681405..1682149	+	EES49281.1	ACSC01000002.1	Clostridium botulinum E1 str. 'BoNT E Beluga'	Bacteria	Firmicutes	Clostridia	Clostridiales	Clostridiaceae	Clostridium	crispr-associated protein Cas5 family	cas5	cd09658	CAS-I-B			
1682213..1684541	+	EES50580.1	ACSC01000002.1	Clostridium botulinum E1 str. 'BoNT E Beluga'	Bacteria	Firmicutes	Clostridia	Clostridiales	Clostridiaceae	Clostridium	metal dependent phosphohydrolase	cas3HD,cas3	cd09641,COG1203	CAS-I			
1684619..1685141	+	EES49876.1	ACSC01000002.1	Clostridium botulinum E1 str. 'BoNT E Beluga'	Bacteria	Firmicutes	Clostridia	Clostridiales	Clostridiaceae	Clostridium	CRISPR-associated protein Cas4	cas4	pfam01930	CAS-I			
1685140..1686124	+	EES50339.1	ACSC01000002.1	Clostridium botulinum E1 str. 'BoNT E Beluga'	Bacteria	Firmicutes	Clostridia	Clostridiales	Clostridiaceae	Clostridium	crispr-associated protein Cas1	cas1	cd09722	CAS-I-B			
1686127..1686391	+	EES47852.1	ACSC01000002.1	Clostridium botulinum E1 str. 'BoNT E Beluga'	Bacteria	Firmicutes	Clostridia	Clostridiales	Clostridiaceae	Clostridium	CRISPR-associated protein Cas2	cas2	cd09725	CAS-I,CAS-II,CAS-III,CAS-V			
1686579..1687522	array		ACSC01000002.1	Clostridium botulinum E1 str. 'BoNT E Beluga'	Bacteria	Firmicutes	Clostridia	Clostridiales	Clostridiaceae	Clostridium		CRISPR array			15	36	29

513 CAS-I-B | KZL91874.1

91443..92382	+	KZL91866.1	LWAE01000002.1	Clostridium magnum DSM 2767	Bacteria	Firmicutes	Clostridia	Clostridiales	Clostridiaceae	Clostridium	hypothetical protein	WYL	pfam13280	CAS-I,CAS-III			
92474..93209	+	KZL91867.1	LWAE01000002.1	Clostridium magnum DSM 2767	Bacteria	Firmicutes	Clostridia	Clostridiales	Clostridiaceae	Clostridium	CRISPR associated protein Cas6	cas6	pfam01881	CAS-I,CAS-III			
93220..94897	+	KZL91868.1	LWAE01000002.1	Clostridium magnum DSM 2767	Bacteria	Firmicutes	Clostridia	Clostridiales	Clostridiaceae	Clostridium	CRISPR-associated protein	cas8b2,cas8b2,cas8b2	pfam09657,cd09754,cd09665	CAS-I-B			
94899..95781	+	KZL91869.1	LWAE01000002.1	Clostridium magnum DSM 2767	Bacteria	Firmicutes	Clostridia	Clostridiales	Clostridiaceae	Clostridium	CRISPR-associated negative autoregulator DevR/Csa2	cas7	cd09687	CAS-I-B			
95791..96532	+	KZL91870.1	LWAE01000002.1	Clostridium magnum DSM 2767	Bacteria	Firmicutes	Clostridia	Clostridiales	Clostridiaceae	Clostridium	CRISPR-associated protein Cas5	cas5	cd09658	CAS-I-B			
96583..98905	+	KZL91871.1	LWAE01000002.1	Clostridium magnum DSM 2767	Bacteria	Firmicutes	Clostridia	Clostridiales	Clostridiaceae	Clostridium	CRISPR-associated endonuclease/helicase Cas3	cas3HD,cas3	cd09641,COG1203	CAS-I			
98992..99514	+	KZL91872.1	LWAE01000002.1	Clostridium magnum DSM 2767	Bacteria	Firmicutes	Clostridia	Clostridiales	Clostridiaceae	Clostridium	hypothetical protein	cas4	pfam01930	CAS-I			
99514..100498	+	KZL91873.1	LWAE01000002.1	Clostridium magnum DSM 2767	Bacteria	Firmicutes	Clostridia	Clostridiales	Clostridiaceae	Clostridium	CRISPR-associated endonuclease Cas1	cas1	cd09722	CAS-I-B			
100502..100766	+	KZL91874.1	LWAE01000002.1	Clostridium magnum DSM 2767	Bacteria	Firmicutes	Clostridia	Clostridiales	Clostridiaceae	Clostridium	CRISPR-associated endoribonuclease Cas2	cas2	cd09725	CAS-I,CAS-II,CAS-III,CAS-V			
100978..103154	array		LWAE01000002.1	Clostridium magnum DSM 2767	Bacteria	Firmicutes	Clostridia	Clostridiales	Clostridiaceae	Clostridium		CRISPR array			34	36	29

514 CAS-I-B | ERI93848.1

6..1222	array		KE993473.1	Clostridiales bacterium oral taxon 876 str. F0540	Bacteria	Firmicutes	Clostridia	Clostridiales				CRISPR array			19	37	29
882..1050	-	ERI93847.1	KE993473.1	Clostridiales bacterium oral taxon 876 str. F0540	Bacteria	Firmicutes	Clostridia	Clostridiales			hypothetical protein						
1414..1678	-	ERI93848.1	KE993473.1	Clostridiales bacterium oral taxon 876 str. F0540	Bacteria	Firmicutes	Clostridia	Clostridiales			CRISPR-associated protein Cas2	cas2	cd09725	CAS-I,CAS-II,CAS-III,CAS-V			
1679..2663	-	ERI93849.1	KE993473.1	Clostridiales bacterium oral taxon 876 str. F0540	Bacteria	Firmicutes	Clostridia	Clostridiales			CRISPR-associated endonuclease Cas1, HMARI/TNEAP subtype	cas1	cd09722	CAS-I-B			
2666..3185	-	ERI93850.1	KE993473.1	Clostridiales bacterium oral taxon 876 str. F0540	Bacteria	Firmicutes	Clostridia	Clostridiales			CRISPR-associated protein Cas4	cas4	pfam01930	CAS-I			
3354..5511	-	ERI93851.1	KE993473.1	Clostridiales bacterium oral taxon 876 str. F0540	Bacteria	Firmicutes	Clostridia	Clostridiales			CRISPR-associated helicase Cas3	cas3HD,cas3	cd09641,cd09639	CAS-I			
5556..6219	-	ERI93852.1	KE993473.1	Clostridiales bacterium oral taxon 876 str. F0540	Bacteria	Firmicutes	Clostridia	Clostridiales			CRISPR-associated protein Cas5, Tneap subtype	cas5	cd09658	CAS-I-B			
6215..7124	-	ERI93853.1	KE993473.1	Clostridiales bacterium oral taxon 876 str. F0540	Bacteria	Firmicutes	Clostridia	Clostridiales			CRISPR-associated regulatory protein, DevR family	cas7	cd09687	CAS-I-B			
7166..8984	-	ERI93854.1	KE993473.1	Clostridiales bacterium oral taxon 876 str. F0540	Bacteria	Firmicutes	Clostridia	Clostridiales			CRISPR-associated cxxc_cxxc protein Cst1	cas8b2	cd09665	CAS-I-B			
8998..9742	-	ERI93855.1	KE993473.1	Clostridiales bacterium oral taxon 876 str. F0540	Bacteria	Firmicutes	Clostridia	Clostridiales			CRISPR-associated endoribonuclease Cas6	cas6	pfam01881	CAS-I,CAS-III			
9791..10733	-	ERI93856.1	KE993473.1	Clostridiales bacterium oral taxon 876 str. F0540	Bacteria	Firmicutes	Clostridia	Clostridiales			HTH domain protein	WYL	COG2378	CAS-I,CAS-III			

515 CAS-I-B | CDM67383.1

208475..209417	+	CDM67375.1	HG917868.1	Clostridium bornimense	Bacteria	Firmicutes	Clostridia	Clostridiales	Clostridiaceae	Clostridium	putative transcriptional regulator	WYL	COG2378	CAS-I,CAS-III			
209512..210250	+	CDM67376.1	HG917868.1	Clostridium bornimense	Bacteria	Firmicutes	Clostridia	Clostridiales	Clostridiaceae	Clostridium	crispr-associated protein Cas6	cas6	cd09652	CAS-I,CAS-III			
210262..212062	+	CDM67377.1	HG917868.1	Clostridium bornimense	Bacteria	Firmicutes	Clostridia	Clostridiales	Clostridiaceae	Clostridium	CRISPR-associated protein CXXC_CXXC region	cas8b2	cd09665	CAS-I-B			
212062..212968	+	CDM67378.1	HG917868.1	Clostridium bornimense	Bacteria	Firmicutes	Clostridia	Clostridiales	Clostridiaceae	Clostridium	CRISPR-associated regulatory protein Cst2	cas7	cd09687	CAS-I-B			
212972..213635	+	CDM67379.1	HG917868.1	Clostridium bornimense	Bacteria	Firmicutes	Clostridia	Clostridiales	Clostridiaceae	Clostridium	CRISPR-associated protein Cas5 family	cas5	cd09658	CAS-I-B			
213684..215877	+	CDM67380.1	HG917868.1	Clostridium bornimense	Bacteria	Firmicutes	Clostridia	Clostridiales	Clostridiaceae	Clostridium	CRISPR-associated helicase Cas3	cas3HD,cas3	cd09641,cd09639	CAS-I			
215889..216408	+	CDM67381.1	HG917868.1	Clostridium bornimense	Bacteria	Firmicutes	Clostridia	Clostridiales	Clostridiaceae	Clostridium	CRISPR-associated Cas4 family protein	cas4	pfam01930	CAS-I			
216407..217391	+	CDM67382.1	HG917868.1	Clostridium bornimense	Bacteria	Firmicutes	Clostridia	Clostridiales	Clostridiaceae	Clostridium	CRISPR-associated endonuclease Cas1	cas1	cd09722	CAS-I-B			
217393..217657	+	CDM67383.1	HG917868.1	Clostridium bornimense	Bacteria	Firmicutes	Clostridia	Clostridiales	Clostridiaceae	Clostridium	Hypothetical protein	cas2	cd09725	CAS-I,CAS-II,CAS-III,CAS-V			
217843..223120	array		HG917868.1	Clostridium bornimense	Bacteria	Firmicutes	Clostridia	Clostridiales	Clostridiaceae	Clostridium		CRISPR array			81	36	29

516 CAS-I-B | AGF58562.1

5276899..5280855	array		CP004121.1	Clostridium saccharoperbutylacetonicum N1-4(HMT)	Bacteria	Firmicutes	Clostridia	Clostridiales	Clostridiaceae	Clostridium		CRISPR array			61	36	29
5281036..5281300	-	AGF58562.1	CP004121.1	Clostridium saccharoperbutylacetonicum N1-4(HMT)	Bacteria	Firmicutes	Clostridia	Clostridiales	Clostridiaceae	Clostridium	CRISPR-associated endoribonuclease Cas2	cas2	cd09725	CAS-I,CAS-II,CAS-III,CAS-V			
5281301..5282285	-	AGF58563.1	CP004121.1	Clostridium saccharoperbutylacetonicum N1-4(HMT)	Bacteria	Firmicutes	Clostridia	Clostridiales	Clostridiaceae	Clostridium	CRISPR-associated endonuclease Cas1	cas1	cd09722	CAS-I-B			
5282289..5282808	-	AGF58564.1	CP004121.1	Clostridium saccharoperbutylacetonicum N1-4(HMT)	Bacteria	Firmicutes	Clostridia	Clostridiales	Clostridiaceae	Clostridium	CRISPR-associated RecB-family exonuclease Cas4	cas4	pfam01930	CAS-I			
5282851..5285068	-	AGF58565.1	CP004121.1	Clostridium saccharoperbutylacetonicum N1-4(HMT)	Bacteria	Firmicutes	Clostridia	Clostridiales	Clostridiaceae	Clostridium	CRISPR-associated nuclease/helicase Cas3	cas3HD,cas3	cd09641,COG1203	CAS-I			
5285207..5285864	-	AGF58566.1	CP004121.1	Clostridium saccharoperbutylacetonicum N1-4(HMT)	Bacteria	Firmicutes	Clostridia	Clostridiales	Clostridiaceae	Clostridium	CRISPR-associated protein Cas5	cas5	cd09658	CAS-I-B			
5285885..5286794	-	AGF58567.1	CP004121.1	Clostridium saccharoperbutylacetonicum N1-4(HMT)	Bacteria	Firmicutes	Clostridia	Clostridiales	Clostridiaceae	Clostridium	CRISPR-associated regulatory protein Cst2	cas7	cd09687	CAS-I-B			
5286795..5288619	-	AGF58568.1	CP004121.1	Clostridium saccharoperbutylacetonicum N1-4(HMT)	Bacteria	Firmicutes	Clostridia	Clostridiales	Clostridiaceae	Clostridium	CRISPR-associated CXXC_CXXC protein Cst1	cas8b2	cd09665	CAS-I-B			
5288633..5289371	-	AGF58569.1	CP004121.1	Clostridium saccharoperbutylacetonicum N1-4(HMT)	Bacteria	Firmicutes	Clostridia	Clostridiales	Clostridiaceae	Clostridium	CRISPR-associated endoribonuclease Cas6	cas6	COG1583	CAS-I,CAS-III,CAS-IV			
5289462..5290413	-	AGF58570.1	CP004121.1	Clostridium saccharoperbutylacetonicum N1-4(HMT)	Bacteria	Firmicutes	Clostridia	Clostridiales	Clostridiaceae	Clostridium	putative transcriptional regulator	WYL	COG2378	CAS-I,CAS-III			

517 CAS-I-B | EHJ01144.1

4885612..4887342	array		CM001240.1	Clostridium sp. DL-VIII	Bacteria	Firmicutes	Clostridia	Clostridiales	Clostridiaceae	Clostridium		CRISPR array			27	36	29
4887523..4887787	-	EHJ01144.1	CM001240.1	Clostridium sp. DL-VIII	Bacteria	Firmicutes	Clostridia	Clostridiales	Clostridiaceae	Clostridium	CRISPR-associated protein Cas2	cas2	cd09725	CAS-I,CAS-II,CAS-III,CAS-V			
4887788..4888772	-	EHJ01145.1	CM001240.1	Clostridium sp. DL-VIII	Bacteria	Firmicutes	Clostridia	Clostridiales	Clostridiaceae	Clostridium	CRISPR-associated protein Cas1	cas1	cd09722	CAS-I-B			
4888775..4889294	-	EHJ01146.1	CM001240.1	Clostridium sp. DL-VIII	Bacteria	Firmicutes	Clostridia	Clostridiales	Clostridiaceae	Clostridium	CRISPR-associated protein Cas4	cas4	pfam01930	CAS-I			
4889420..4891640	-	EHJ01147.1	CM001240.1	Clostridium sp. DL-VIII	Bacteria	Firmicutes	Clostridia	Clostridiales	Clostridiaceae	Clostridium	CRISPR-associated helicase Cas3	cas3HD,cas3	cd09641,COG1203	CAS-I			
4891777..4892449	-	EHJ01148.1	CM001240.1	Clostridium sp. DL-VIII	Bacteria	Firmicutes	Clostridia	Clostridiales	Clostridiaceae	Clostridium	CRISPR-associated protein Cas5 family	cas5	cd09658	CAS-I-B			
4892455..4893364	-	EHJ01149.1	CM001240.1	Clostridium sp. DL-VIII	Bacteria	Firmicutes	Clostridia	Clostridiales	Clostridiaceae	Clostridium	CRISPR-associated autoregulator, DevR family	cas7	cd09687	CAS-I-B			
4893365..4895171	-	EHJ01150.1	CM001240.1	Clostridium sp. DL-VIII	Bacteria	Firmicutes	Clostridia	Clostridiales	Clostridiaceae	Clostridium	CRISPR-associated protein CXXC_CXXC region	cas8b2	cd09665	CAS-I-B			
4895185..4895923	-	EHJ01151.1	CM001240.1	Clostridium sp. DL-VIII	Bacteria	Firmicutes	Clostridia	Clostridiales	Clostridiaceae	Clostridium	CRISPR-associated protein Cas6	cas6	COG1583	CAS-I,CAS-III,CAS-IV			
4896016..4896967	-	EHJ01152.1	CM001240.1	Clostridium sp. DL-VIII	Bacteria	Firmicutes	Clostridia	Clostridiales	Clostridiaceae	Clostridium	hypothetical protein	WYL	COG2378	CAS-I,CAS-III			

518 CAS-I-B | SFF89272.1

36552..36974	array		FOOE01000014.1	Clostridium cadaveris	Bacteria	Firmicutes	Clostridia	Clostridiales	Clostridiaceae	Clostridium		CRISPR array			7	35	30
38972..39720	array		FOOE01000014.1	Clostridium cadaveris	Bacteria	Firmicutes	Clostridia	Clostridiales	Clostridiaceae	Clostridium		CRISPR array			12	36	29
39902..40166	-	SFF89272.1	FOOE01000014.1	Clostridium cadaveris	Bacteria	Firmicutes	Clostridia	Clostridiales	Clostridiaceae	Clostridium	CRISPR-associated protein Cas2	cas2	cd09725	CAS-I,CAS-II,CAS-III,CAS-V			
40167..41151	-	SFF89285.1	FOOE01000014.1	Clostridium cadaveris	Bacteria	Firmicutes	Clostridia	Clostridiales	Clostridiaceae	Clostridium	CRISP-associated protein Cas1	cas1	cd09722	CAS-I-B			
41150..41648	-	SFF89304.1	FOOE01000014.1	Clostridium cadaveris	Bacteria	Firmicutes	Clostridia	Clostridiales	Clostridiaceae	Clostridium	CRISPR-associated exonuclease, Cas4 family	cas4	pfam01930	CAS-I			
41675..43901	-	SFF89321.1	FOOE01000014.1	Clostridium cadaveris	Bacteria	Firmicutes	Clostridia	Clostridiales	Clostridiaceae	Clostridium	CRISPR-associated helicase, Cas3 family	cas3HD,cas3	cd09641,COG1203	CAS-I			
43952..44612	-	SFF89340.1	FOOE01000014.1	Clostridium cadaveris	Bacteria	Firmicutes	Clostridia	Clostridiales	Clostridiaceae	Clostridium	CRISPR-associated protein, Cas5t family	cas5	cd09658	CAS-I-B			
44617..45517	-	SFF89353.1	FOOE01000014.1	Clostridium cadaveris	Bacteria	Firmicutes	Clostridia	Clostridiales	Clostridiaceae	Clostridium	CRISPR-associated autoregulator, Cst2 family	cas7	cd09687	CAS-I-B			
45500..47306	-	SFF89372.1	FOOE01000014.1	Clostridium cadaveris	Bacteria	Firmicutes	Clostridia	Clostridiales	Clostridiaceae	Clostridium	CRISPR-associated protein Cst1	cas8b2,cas8b2	cd09754,cd09665	CAS-I-B			
47317..48052	-	SFF89388.1	FOOE01000014.1	Clostridium cadaveris	Bacteria	Firmicutes	Clostridia	Clostridiales	Clostridiaceae	Clostridium	CRISPR-associated endoribonuclease Cas6	cas6	pfam01881	CAS-I,CAS-III			

519 CAS-I-B | PWL53907.1

45213..45948	+	PWL53900.1	QAMZ01000029.1	Clostridium cadaveris	Bacteria	Firmicutes	Clostridia	Clostridiales	Clostridiaceae	Clostridium	CRISPR-associated endoribonuclease Cas6	cas6	pfam01881	CAS-I,CAS-III			
45959..47765	+	PWL53901.1	QAMZ01000029.1	Clostridium cadaveris	Bacteria	Firmicutes	Clostridia	Clostridiales	Clostridiaceae	Clostridium	type I-B CRISPR-associated protein Cas8b1/Cst1	cas8b2,cas8b2	cd09754,cd09665	CAS-I-B			
47757..48648	+	PWL53902.1	QAMZ01000029.1	Clostridium cadaveris	Bacteria	Firmicutes	Clostridia	Clostridiales	Clostridiaceae	Clostridium	type I-B CRISPR-associated protein Cas7/Cst2/DevR	cas7	cd09687	CAS-I-B			
48653..49313	+	PWL53903.1	QAMZ01000029.1	Clostridium cadaveris	Bacteria	Firmicutes	Clostridia	Clostridiales	Clostridiaceae	Clostridium	type I-B CRISPR-associated protein Cas5	cas5	cd09658	CAS-I-B			
49364..51590	+	PWL53904.1	QAMZ01000029.1	Clostridium cadaveris	Bacteria	Firmicutes	Clostridia	Clostridiales	Clostridiaceae	Clostridium	CRISPR-associated helicase/endonuclease Cas3	cas3HD,cas3	cd09641,COG1203	CAS-I			
51593..52115	+	PWL53905.1	QAMZ01000029.1	Clostridium cadaveris	Bacteria	Firmicutes	Clostridia	Clostridiales	Clostridiaceae	Clostridium	CRISPR-associated protein Cas4	cas4	pfam01930	CAS-I			
52114..53098	+	PWL53906.1	QAMZ01000029.1	Clostridium cadaveris	Bacteria	Firmicutes	Clostridia	Clostridiales	Clostridiaceae	Clostridium	type I-B CRISPR-associated endonuclease Cas1	cas1	cd09722	CAS-I-B			
53099..53363	+	PWL53907.1	QAMZ01000029.1	Clostridium cadaveris	Bacteria	Firmicutes	Clostridia	Clostridiales	Clostridiaceae	Clostridium	CRISPR-associated endonuclease Cas2	cas2	cd09725	CAS-I,CAS-II,CAS-III,CAS-V			
53546..56527	array		QAMZ01000029.1	Clostridium cadaveris	Bacteria	Firmicutes	Clostridia	Clostridiales	Clostridiaceae	Clostridium		CRISPR array			46	36	29

520 CAS-I-B | SHH05386.1

292..556	-	SHH05386.1	FQXH01000007.1	Tepidibacter thalassicus DSM 15285	Bacteria	Firmicutes	Clostridia	Clostridiales	Peptostreptococcaceae	Tepidibacter	CRISPR-associated protein Cas2	cas2	cd09725	CAS-I,CAS-II,CAS-III,CAS-V			
557..1544	-	SHH05410.1	FQXH01000007.1	Tepidibacter thalassicus DSM 15285	Bacteria	Firmicutes	Clostridia	Clostridiales	Peptostreptococcaceae	Tepidibacter	CRISP-associated protein Cas1	cas1	cd09722	CAS-I-B			
1546..2062	-	SHH05420.1	FQXH01000007.1	Tepidibacter thalassicus DSM 15285	Bacteria	Firmicutes	Clostridia	Clostridiales	Peptostreptococcaceae	Tepidibacter	CRISPR-associated exonuclease Cas4	cas4	pfam01930	CAS-I			
2178..4551	-	SHH05444.1	FQXH01000007.1	Tepidibacter thalassicus DSM 15285	Bacteria	Firmicutes	Clostridia	Clostridiales	Peptostreptococcaceae	Tepidibacter	CRISPR-associated helicase Cas3/CRISPR-associated endonuclease Cas3-HD	cas3	cd09639	CAS-I			
4565..5402	-	SHH05465.1	FQXH01000007.1	Tepidibacter thalassicus DSM 15285	Bacteria	Firmicutes	Clostridia	Clostridiales	Peptostreptococcaceae	Tepidibacter	CRISPR-associated protein Cas5, N-terminal domain-containing protein	cas5	cd09658	CAS-I-B			
5394..6426	-	SHH05485.1	FQXH01000007.1	Tepidibacter thalassicus DSM 15285	Bacteria	Firmicutes	Clostridia	Clostridiales	Peptostreptococcaceae	Tepidibacter	CRISPR-associated protein Cas7/Cst2/DevR, subtype I-B/TNEAP	cas7	cd09687	CAS-I-B			
6427..7768	-	SHH05503.1	FQXH01000007.1	Tepidibacter thalassicus DSM 15285	Bacteria	Firmicutes	Clostridia	Clostridiales	Peptostreptococcaceae	Tepidibacter	hypothetical protein	Cas8b11,Cas8b11	icity0056,icity0056				
7771..8506	-	SHH05527.1	FQXH01000007.1	Tepidibacter thalassicus DSM 15285	Bacteria	Firmicutes	Clostridia	Clostridiales	Peptostreptococcaceae	Tepidibacter	CRISPR-associated protein, Cas6 family	cas6	cd09652	CAS-I,CAS-III			
8574..9525	-	SHH05545.1	FQXH01000007.1	Tepidibacter thalassicus DSM 15285	Bacteria	Firmicutes	Clostridia	Clostridiales	Peptostreptococcaceae	Tepidibacter	Predicted DNA-binding transcriptional regulator YafY, contains an HTH and WYL domains	WYL	pfam13280	CAS-I,CAS-III			

521 CAS-I-B | KXZ39967.1

1020595..1022226	array		LSFY01000001.1	[Clostridium] paradoxum JW-YL-7 = DSM 7308	Bacteria	Firmicutes	Clostridia	Clostridiales	Peptostreptococcaceae			CRISPR array			25	37	29
1022421..1022685	-	KXZ39967.1	LSFY01000001.1	[Clostridium] paradoxum JW-YL-7 = DSM 7308	Bacteria	Firmicutes	Clostridia	Clostridiales	Peptostreptococcaceae		CRISPR associated protein Cas2	cas2	cd09725	CAS-I,CAS-II,CAS-III,CAS-V			
1022686..1023673	-	KXZ39968.1	LSFY01000001.1	[Clostridium] paradoxum JW-YL-7 = DSM 7308	Bacteria	Firmicutes	Clostridia	Clostridiales	Peptostreptococcaceae		CRISPR-associated protein Cas1	cas1	cd09722	CAS-I-B			
1023675..1024191	-	KXZ39969.1	LSFY01000001.1	[Clostridium] paradoxum JW-YL-7 = DSM 7308	Bacteria	Firmicutes	Clostridia	Clostridiales	Peptostreptococcaceae		CRISPR-associated protein Cas4	cas4	pfam01930	CAS-I			
1024216..1026508	-	KXZ39970.1	LSFY01000001.1	[Clostridium] paradoxum JW-YL-7 = DSM 7308	Bacteria	Firmicutes	Clostridia	Clostridiales	Peptostreptococcaceae		CRISPR-associated helicase Cas3	cas3HD,cas3	cd09641,cd09639	CAS-I			
1026611..1027286	-	KXZ39971.1	LSFY01000001.1	[Clostridium] paradoxum JW-YL-7 = DSM 7308	Bacteria	Firmicutes	Clostridia	Clostridiales	Peptostreptococcaceae		CRISPR-associated protein Cas5 family	cas5	cd09658	CAS-I-B			
1027288..1028209	-	KXZ39972.1	LSFY01000001.1	[Clostridium] paradoxum JW-YL-7 = DSM 7308	Bacteria	Firmicutes	Clostridia	Clostridiales	Peptostreptococcaceae		CRISPR-associated autoregulator, DevR family	cas7	cd09687	CAS-I-B			
1028211..1030005	-	KXZ39973.1	LSFY01000001.1	[Clostridium] paradoxum JW-YL-7 = DSM 7308	Bacteria	Firmicutes	Clostridia	Clostridiales	Peptostreptococcaceae		CRISPR-associated protein CXXC-CXXC region	cas8b2	cd09665	CAS-I-B			
1030016..1030751	-	KXZ39974.1	LSFY01000001.1	[Clostridium] paradoxum JW-YL-7 = DSM 7308	Bacteria	Firmicutes	Clostridia	Clostridiales	Peptostreptococcaceae		CRISPR-associated protein Cas6	cas6	cd09652	CAS-I,CAS-III			
1030818..1031775	-	KXZ39975.1	LSFY01000001.1	[Clostridium] paradoxum JW-YL-7 = DSM 7308	Bacteria	Firmicutes	Clostridia	Clostridiales	Peptostreptococcaceae		WYL domain containing protein	WYL	pfam13280	CAS-I,CAS-III			

522 CAS-I-B | WP_066070038.1

1020595..1022226	array		NZ_LSFY01000001.1	[Clostridium] paradoxum	Bacteria	Firmicutes	Clostridia	Clostridiales	Peptostreptococcaceae			CRISPR array			25	37	29
1022421..1022685	-	WP_066070038.1	NZ_LSFY01000001.1	[Clostridium] paradoxum	Bacteria	Firmicutes	Clostridia	Clostridiales	Peptostreptococcaceae		CRISPR-associated endonuclease Cas2	cas2	cd09725	CAS-I,CAS-II,CAS-III,CAS-V			
1022686..1023673	-	WP_066070042.1	NZ_LSFY01000001.1	[Clostridium] paradoxum	Bacteria	Firmicutes	Clostridia	Clostridiales	Peptostreptococcaceae		type I-B CRISPR-associated endonuclease Cas1	cas1	cd09722	CAS-I-B			
1023675..1024191	-	WP_066070046.1	NZ_LSFY01000001.1	[Clostridium] paradoxum	Bacteria	Firmicutes	Clostridia	Clostridiales	Peptostreptococcaceae		CRISPR-associated protein Cas4	cas4	pfam01930	CAS-I			
1024216..1026508	-	WP_066070049.1	NZ_LSFY01000001.1	[Clostridium] paradoxum	Bacteria	Firmicutes	Clostridia	Clostridiales	Peptostreptococcaceae		CRISPR-associated helicase/endonuclease Cas3	cas3HD,cas3	cd09641,cd09639	CAS-I			
1026611..1027286	-	WP_066070052.1	NZ_LSFY01000001.1	[Clostridium] paradoxum	Bacteria	Firmicutes	Clostridia	Clostridiales	Peptostreptococcaceae		type I-B CRISPR-associated protein Cas5	cas5	cd09658	CAS-I-B			
1027288..1028209	-	WP_066070055.1	NZ_LSFY01000001.1	[Clostridium] paradoxum	Bacteria	Firmicutes	Clostridia	Clostridiales	Peptostreptococcaceae		type I-B CRISPR-associated protein Cas7/Cst2/DevR	cas7	cd09687	CAS-I-B			
1028211..1030005	-	WP_066070058.1	NZ_LSFY01000001.1	[Clostridium] paradoxum	Bacteria	Firmicutes	Clostridia	Clostridiales	Peptostreptococcaceae		type I-B CRISPR-associated protein Cas8b1/Cst1	cas8b2	cd09665	CAS-I-B			
1030016..1030751	-	WP_066070061.1	NZ_LSFY01000001.1	[Clostridium] paradoxum	Bacteria	Firmicutes	Clostridia	Clostridiales	Peptostreptococcaceae		CRISPR-associated endoribonuclease Cas6	cas6	cd09652	CAS-I,CAS-III			
1030818..1031739	-	WP_072280177.1	NZ_LSFY01000001.1	[Clostridium] paradoxum	Bacteria	Firmicutes	Clostridia	Clostridiales	Peptostreptococcaceae		transcriptional regulator	WYL	pfam13280	CAS-I,CAS-III			

523 CAS-I-B | ODM28179.1

3339806..3340487	array		LXSB01000001.1	Clostridium sp. Bc-iso-3	Bacteria	Firmicutes	Clostridia	Clostridiales	Clostridiaceae	Clostridium		CRISPR array			11	36	29
3340792..3342010	-	ODM27283.1	LXSB01000001.1	Clostridium sp. Bc-iso-3	Bacteria	Firmicutes	Clostridia	Clostridiales	Clostridiaceae	Clostridium	transposase						
3342111..3344231	array		LXSB01000001.1	Clostridium sp. Bc-iso-3	Bacteria	Firmicutes	Clostridia	Clostridiales	Clostridiaceae	Clostridium		CRISPR array			33	36	29
3344243..3347717	array		LXSB01000001.1	Clostridium sp. Bc-iso-3	Bacteria	Firmicutes	Clostridia	Clostridiales	Clostridiaceae	Clostridium		CRISPR array			54	36	29
3347899..3348163	-	ODM28179.1	LXSB01000001.1	Clostridium sp. Bc-iso-3	Bacteria	Firmicutes	Clostridia	Clostridiales	Clostridiaceae	Clostridium	CRISPR-associated endonuclease Cas2	cas2	cd09725	CAS-I,CAS-II,CAS-III,CAS-V			
3348165..3349143	-	ODM27284.1	LXSB01000001.1	Clostridium sp. Bc-iso-3	Bacteria	Firmicutes	Clostridia	Clostridiales	Clostridiaceae	Clostridium	subtype I-B CRISPR-associated endonuclease Cas1	cas1	cd09722	CAS-I-B			
3349135..3349669	-	ODM27285.1	LXSB01000001.1	Clostridium sp. Bc-iso-3	Bacteria	Firmicutes	Clostridia	Clostridiales	Clostridiaceae	Clostridium	CRISPR-associated protein Cas4	cas4	pfam01930	CAS-I			
3349704..3352110	-	ODM27286.1	LXSB01000001.1	Clostridium sp. Bc-iso-3	Bacteria	Firmicutes	Clostridia	Clostridiales	Clostridiaceae	Clostridium	hypothetical protein	cas3HD,cas3	cd09641,COG1203	CAS-I			
3352100..3352916	-	ODM27287.1	LXSB01000001.1	Clostridium sp. Bc-iso-3	Bacteria	Firmicutes	Clostridia	Clostridiales	Clostridiaceae	Clostridium	hypothetical protein	cas5	cd09658	CAS-I-B			
3352917..3353919	-	ODM27288.1	LXSB01000001.1	Clostridium sp. Bc-iso-3	Bacteria	Firmicutes	Clostridia	Clostridiales	Clostridiaceae	Clostridium	type I-B CRISPR-associated protein Cas7/Cst2/DevR	cas7	cd09687	CAS-I-B			
3353915..3355409	-	ODM27289.1	LXSB01000001.1	Clostridium sp. Bc-iso-3	Bacteria	Firmicutes	Clostridia	Clostridiales	Clostridiaceae	Clostridium	hypothetical protein	Cas8b11	icity0056				
3355418..3356150	-	ODM27290.1	LXSB01000001.1	Clostridium sp. Bc-iso-3	Bacteria	Firmicutes	Clostridia	Clostridiales	Clostridiaceae	Clostridium	CRISPR-associated endoribonuclease Cas6	cas6	COG1583	CAS-I,CAS-III,CAS-IV			
3356201..3357167	-	ODM27291.1	LXSB01000001.1	Clostridium sp. Bc-iso-3	Bacteria	Firmicutes	Clostridia	Clostridiales	Clostridiaceae	Clostridium	transcriptional regulator	WYL	pfam13280	CAS-I,CAS-III			

524 CAS-I-B | AIY84916.1

2319740..2320685	+	AIY83954.1	CP006905.1	Clostridium baratii str. Sullivan	Bacteria	Firmicutes	Clostridia	Clostridiales	Clostridiaceae	Clostridium	winged helix-turn-helix transcription repressor, HrcA DNA-binding family protein	WYL	pfam13280	CAS-I,CAS-III			
2320754..2321276	+	AIY84736.1	CP006905.1	Clostridium baratii str. Sullivan	Bacteria	Firmicutes	Clostridia	Clostridiales	Clostridiaceae	Clostridium	CRISPR-associated protein Cas4	cas4	pfam01930	CAS-I			
2321275..2322256	+	AIY84443.1	CP006905.1	Clostridium baratii str. Sullivan	Bacteria	Firmicutes	Clostridia	Clostridiales	Clostridiaceae	Clostridium	CRISPR-associated endonuclease Cas1	cas1	cd09722	CAS-I-B			
2322257..2322521	+	AIY84916.1	CP006905.1	Clostridium baratii str. Sullivan	Bacteria	Firmicutes	Clostridia	Clostridiales	Clostridiaceae	Clostridium	CRISPR-associated endoribonuclease Cas2	cas2	cd09725	CAS-I,CAS-II,CAS-III,CAS-V			
2322725..2324465	array		CP006905.1	Clostridium baratii str. Sullivan	Bacteria	Firmicutes	Clostridia	Clostridiales	Clostridiaceae	Clostridium		CRISPR array			27	36	29
2324720..2325458	+	AIY82967.1	CP006905.1	Clostridium baratii str. Sullivan	Bacteria	Firmicutes	Clostridia	Clostridiales	Clostridiaceae	Clostridium	CRISPR-associated endoribonuclease Cas6	cas6	COG1583	CAS-I,CAS-III,CAS-IV			
2325471..2327127	+	AIY84148.1	CP006905.1	Clostridium baratii str. Sullivan	Bacteria	Firmicutes	Clostridia	Clostridiales	Clostridiaceae	Clostridium	CRISPR-associated family protein	cas8b2	cd09665	CAS-I-B			
2327128..2328010	+	AIY83552.1	CP006905.1	Clostridium baratii str. Sullivan	Bacteria	Firmicutes	Clostridia	Clostridiales	Clostridiaceae	Clostridium	CRISPR-associated autoregulator DevR family protein	cas7	cd09687	CAS-I-B			
2328010..2328763	+	AIY83031.1	CP006905.1	Clostridium baratii str. Sullivan	Bacteria	Firmicutes	Clostridia	Clostridiales	Clostridiaceae	Clostridium	CRISPR-associated Cas5 domain protein	cas5	cd09658	CAS-I-B			
2328821..2331161	+	AIY83368.1	CP006905.1	Clostridium baratii str. Sullivan	Bacteria	Firmicutes	Clostridia	Clostridiales	Clostridiaceae	Clostridium	CRISPR-associated endonuclease Cas3-HD	cas3	COG1203	CAS-I			
2331404..2332022	array		CP006905.1	Clostridium baratii str. Sullivan	Bacteria	Firmicutes	Clostridia	Clostridiales	Clostridiaceae	Clostridium		CRISPR array			10	36	29

525 CAS-I-B | KJU72924.1

408186..409131	+	KJU72921.1	JZTY01000002.1	Clostridium baratii	Bacteria	Firmicutes	Clostridia	Clostridiales	Clostridiaceae	Clostridium	hypothetical protein	WYL	pfam13280	CAS-I,CAS-III			
409202..409724	+	KJU72922.1	JZTY01000002.1	Clostridium baratii	Bacteria	Firmicutes	Clostridia	Clostridiales	Clostridiaceae	Clostridium	CRISPR-associated protein Cas4	cas4	pfam01930	CAS-I			
409723..410704	+	KJU72923.1	JZTY01000002.1	Clostridium baratii	Bacteria	Firmicutes	Clostridia	Clostridiales	Clostridiaceae	Clostridium	CRISPR-associated protein Cas1	cas1	cd09722	CAS-I-B			
410705..410969	+	KJU72924.1	JZTY01000002.1	Clostridium baratii	Bacteria	Firmicutes	Clostridia	Clostridiales	Clostridiaceae	Clostridium	CRISPR-associated protein Cas2	cas2	cd09725	CAS-I,CAS-II,CAS-III,CAS-V			
411170..411783	array		JZTY01000002.1	Clostridium baratii	Bacteria	Firmicutes	Clostridia	Clostridiales	Clostridiaceae	Clostridium		CRISPR array			10	36	29
412036..412774	+	KJU72925.1	JZTY01000002.1	Clostridium baratii	Bacteria	Firmicutes	Clostridia	Clostridiales	Clostridiaceae	Clostridium	hypothetical protein	cas6	COG1583	CAS-I,CAS-III,CAS-IV			
412787..414443	+	KJU72926.1	JZTY01000002.1	Clostridium baratii	Bacteria	Firmicutes	Clostridia	Clostridiales	Clostridiaceae	Clostridium	CRISPR-associated protein Cas8	cas8b2,cas8b2	cd09754,cd09665	CAS-I-B			
414444..415326	+	KJU72927.1	JZTY01000002.1	Clostridium baratii	Bacteria	Firmicutes	Clostridia	Clostridiales	Clostridiaceae	Clostridium	CRISPR-associated protein	cas7	cd09687	CAS-I-B			
415338..416079	+	KJU73075.1	JZTY01000002.1	Clostridium baratii	Bacteria	Firmicutes	Clostridia	Clostridiales	Clostridiaceae	Clostridium	CRISPR-associated protein Cas5	cas5	cd09658	CAS-I-B			
416138..418478	+	KJU72928.1	JZTY01000002.1	Clostridium baratii	Bacteria	Firmicutes	Clostridia	Clostridiales	Clostridiaceae	Clostridium	CRISPR-associated protein Cas3	cas3	COG1203	CAS-I			
418721..420581	array		JZTY01000002.1	Clostridium baratii	Bacteria	Firmicutes	Clostridia	Clostridiales	Clostridiaceae	Clostridium		CRISPR array			29	36	29

526 CAS-I-B | KYH34075.1

425..11097	array		LTBA01000026.1	Clostridium tepidiprofundi DSM 19306	Bacteria	Firmicutes	Clostridia	Clostridiales	Clostridiaceae	Clostridium		CRISPR array			163	36	29
11286..11550	-	KYH34075.1	LTBA01000026.1	Clostridium tepidiprofundi DSM 19306	Bacteria	Firmicutes	Clostridia	Clostridiales	Clostridiaceae	Clostridium	CRISPR-associated endoribonuclease Cas2	cas2	cd09725	CAS-I,CAS-II,CAS-III,CAS-V			
11619..12600	-	KYH34076.1	LTBA01000026.1	Clostridium tepidiprofundi DSM 19306	Bacteria	Firmicutes	Clostridia	Clostridiales	Clostridiaceae	Clostridium	CRISPR-associated endonuclease Cas1	cas1	cd09722	CAS-I-B			
12599..13121	-	KYH34077.1	LTBA01000026.1	Clostridium tepidiprofundi DSM 19306	Bacteria	Firmicutes	Clostridia	Clostridiales	Clostridiaceae	Clostridium	hypothetical protein	cas4	pfam01930	CAS-I			
13262..15641	-	KYH34078.1	LTBA01000026.1	Clostridium tepidiprofundi DSM 19306	Bacteria	Firmicutes	Clostridia	Clostridiales	Clostridiaceae	Clostridium	putative CRISPR-associated nuclease/helicase Cas3	cas3HD,cas3	cd09641,COG1203	CAS-I			
15656..16502	-	KYH34079.1	LTBA01000026.1	Clostridium tepidiprofundi DSM 19306	Bacteria	Firmicutes	Clostridia	Clostridiales	Clostridiaceae	Clostridium	CRISPR-associated protein	cas5	cd09658	CAS-I-B			
16485..17547	-	KYH34080.1	LTBA01000026.1	Clostridium tepidiprofundi DSM 19306	Bacteria	Firmicutes	Clostridia	Clostridiales	Clostridiaceae	Clostridium	hypothetical protein	cas7	cd09687	CAS-I-B			
17549..19022	-	KYH34081.1	LTBA01000026.1	Clostridium tepidiprofundi DSM 19306	Bacteria	Firmicutes	Clostridia	Clostridiales	Clostridiaceae	Clostridium	hypothetical protein	Cas8b11	icity0056				
19028..19763	-	KYH34082.1	LTBA01000026.1	Clostridium tepidiprofundi DSM 19306	Bacteria	Firmicutes	Clostridia	Clostridiales	Clostridiaceae	Clostridium	CRISPR associated protein Cas6	cas6	pfam01881	CAS-I,CAS-III			
19898..20099	-	KYH34083.1	LTBA01000026.1	Clostridium tepidiprofundi DSM 19306	Bacteria	Firmicutes	Clostridia	Clostridiales	Clostridiaceae	Clostridium	HTH domain protein	WYL	COG2378	CAS-I,CAS-III			
20506..22840	-	KYH34084.1	LTBA01000026.1	Clostridium tepidiprofundi DSM 19306	Bacteria	Firmicutes	Clostridia	Clostridiales	Clostridiaceae	Clostridium	CRISPR-associated nuclease/helicase Cas3	cas3HD,cas3	cd09641,COG1203	CAS-I			
22995..23757	-	KYH34085.1	LTBA01000026.1	Clostridium tepidiprofundi DSM 19306	Bacteria	Firmicutes	Clostridia	Clostridiales	Clostridiaceae	Clostridium	CRISPR-associated protein	cas5	cd09658	CAS-I-B			
23765..24653	-	KYH34086.1	LTBA01000026.1	Clostridium tepidiprofundi DSM 19306	Bacteria	Firmicutes	Clostridia	Clostridiales	Clostridiaceae	Clostridium	CRISPR-associated negative autoregulator DevR/Csa2	cas7	cd09687	CAS-I-B			
24645..26337	-	KYH34087.1	LTBA01000026.1	Clostridium tepidiprofundi DSM 19306	Bacteria	Firmicutes	Clostridia	Clostridiales	Clostridiaceae	Clostridium	CRISPR-associated protein	cas8b2,cas8b2	cd09754,cd09665	CAS-I-B			
26340..27078	-	KYH34088.1	LTBA01000026.1	Clostridium tepidiprofundi DSM 19306	Bacteria	Firmicutes	Clostridia	Clostridiales	Clostridiaceae	Clostridium	CRISPR associated protein Cas6	cas6	cd09652	CAS-I,CAS-III			
27140..28091	-	KYH34089.1	LTBA01000026.1	Clostridium tepidiprofundi DSM 19306	Bacteria	Firmicutes	Clostridia	Clostridiales	Clostridiaceae	Clostridium	HTH domain protein	WYL	pfam13280	CAS-I,CAS-III			

527 CAS-I-B | OOO69967.1

195372..196356	+	OOO69959.1	MRAE01000001.1	Clostridium tepidum	Bacteria	Firmicutes	Clostridia	Clostridiales	Clostridiaceae	Clostridium	transcriptional regulator	WYL	COG2378	CAS-I,CAS-III			
196589..197324	+	OOO69960.1	MRAE01000001.1	Clostridium tepidum	Bacteria	Firmicutes	Clostridia	Clostridiales	Clostridiaceae	Clostridium	CRISPR-associated endoribonuclease Cas6	cas6	pfam01881	CAS-I,CAS-III			
197330..199013	+	OOO69961.1	MRAE01000001.1	Clostridium tepidum	Bacteria	Firmicutes	Clostridia	Clostridiales	Clostridiaceae	Clostridium	type I-B CRISPR-associated protein Cas8b1/Cst1	cas8b2	cd09665	CAS-I-B			
199005..199911	+	OOO69962.1	MRAE01000001.1	Clostridium tepidum	Bacteria	Firmicutes	Clostridia	Clostridiales	Clostridiaceae	Clostridium	type I-B CRISPR-associated protein Cas7/Cst2/DevR	cas7	cd09687	CAS-I-B			
199919..200825	+	OOO69963.1	MRAE01000001.1	Clostridium tepidum	Bacteria	Firmicutes	Clostridia	Clostridiales	Clostridiaceae	Clostridium	type I-B CRISPR-associated protein Cas5	cas5	cd09658	CAS-I-B			
200892..203160	+	OOO69964.1	MRAE01000001.1	Clostridium tepidum	Bacteria	Firmicutes	Clostridia	Clostridiales	Clostridiaceae	Clostridium	CRISPR-associated helicase/endonuclease Cas3	cas3HD,cas3	cd09641,COG1203	CAS-I			
203175..203691	+	OOO69965.1	MRAE01000001.1	Clostridium tepidum	Bacteria	Firmicutes	Clostridia	Clostridiales	Clostridiaceae	Clostridium	CRISPR-associated protein Cas4	cas4	pfam01930	CAS-I			
203693..204674	+	OOO69966.1	MRAE01000001.1	Clostridium tepidum	Bacteria	Firmicutes	Clostridia	Clostridiales	Clostridiaceae	Clostridium	subtype I-B CRISPR-associated endonuclease Cas1	cas1	cd09722	CAS-I-B			
204673..204893	array		MRAE01000001.1	Clostridium tepidum	Bacteria	Firmicutes	Clostridia	Clostridiales	Clostridiaceae	Clostridium		CRISPR array			4	40	25
204876..205140	+	OOO69967.1	MRAE01000001.1	Clostridium tepidum	Bacteria	Firmicutes	Clostridia	Clostridiales	Clostridiaceae	Clostridium	CRISPR-associated endonuclease Cas2	cas2	cd09725	CAS-I,CAS-II,CAS-III,CAS-V			
205331..216549	array		MRAE01000001.1	Clostridium tepidum	Bacteria	Firmicutes	Clostridia	Clostridiales	Clostridiaceae	Clostridium		CRISPR array			170	37	29

528 CAS-I-B | EDU38645.1

1049893..1050952	+	EDU38637.1	DS981518.1	Clostridium sporogenes ATCC 15579	Bacteria	Firmicutes	Clostridia	Clostridiales	Clostridiaceae	Clostridium	HTH domain protein	WYL	pfam13280	CAS-I,CAS-III			
1050975..1051719	+	EDU38638.1	DS981518.1	Clostridium sporogenes ATCC 15579	Bacteria	Firmicutes	Clostridia	Clostridiales	Clostridiaceae	Clostridium	CRISPR-associated endoribonuclease Cas6	cas6	COG1583	CAS-I,CAS-III,CAS-IV			
1051722..1053420	+	EDU38639.1	DS981518.1	Clostridium sporogenes ATCC 15579	Bacteria	Firmicutes	Clostridia	Clostridiales	Clostridiaceae	Clostridium	CRISPR-associated cxxc_cxxc protein Cst1	cas8b2,cas8b2	cd09754,cd09665	CAS-I-B			
1053412..1054318	+	EDU38640.1	DS981518.1	Clostridium sporogenes ATCC 15579	Bacteria	Firmicutes	Clostridia	Clostridiales	Clostridiaceae	Clostridium	CRISPR-associated regulatory protein, DevR family	cas7	cd09687	CAS-I-B			
1054326..1055088	+	EDU38641.1	DS981518.1	Clostridium sporogenes ATCC 15579	Bacteria	Firmicutes	Clostridia	Clostridiales	Clostridiaceae	Clostridium	CRISPR-associated protein Cas5, Tneap subtype	cas5	cd09658	CAS-I-B			
1055158..1057411	+	EDU38642.1	DS981518.1	Clostridium sporogenes ATCC 15579	Bacteria	Firmicutes	Clostridia	Clostridiales	Clostridiaceae	Clostridium	CRISPR-associated helicase Cas3	cas3HD,cas3	cd09641,COG1203	CAS-I			
1057426..1057945	+	EDU38643.1	DS981518.1	Clostridium sporogenes ATCC 15579	Bacteria	Firmicutes	Clostridia	Clostridiales	Clostridiaceae	Clostridium	CRISPR-associated protein Cas4	cas4	pfam01930	CAS-I			
1057944..1058925	+	EDU38644.1	DS981518.1	Clostridium sporogenes ATCC 15579	Bacteria	Firmicutes	Clostridia	Clostridiales	Clostridiaceae	Clostridium	CRISPR-associated endonuclease Cas1, HMARI/TNEAP subtype	cas1	cd09722	CAS-I-B			
1058931..1059195	+	EDU38645.1	DS981518.1	Clostridium sporogenes ATCC 15579	Bacteria	Firmicutes	Clostridia	Clostridiales	Clostridiaceae	Clostridium	CRISPR-associated protein Cas2	cas2	cd09725	CAS-I,CAS-II,CAS-III,CAS-V			
1059386..1062014	array		DS981518.1	Clostridium sporogenes ATCC 15579	Bacteria	Firmicutes	Clostridia	Clostridiales	Clostridiaceae	Clostridium		CRISPR array			40	37	29

529 partial CAS-I | OQP93145.1

45934..50438	array		LKKY02000014.1	Clostridium sporogenes	Bacteria	Firmicutes	Clostridia	Clostridiales	Clostridiaceae	Clostridium		CRISPR array			69	36	29
50628..50892	-	OQP93145.1	LKKY02000014.1	Clostridium sporogenes	Bacteria	Firmicutes	Clostridia	Clostridiales	Clostridiaceae	Clostridium	CRISPR-associated endoribonuclease Cas2	cas2	cd09725	CAS-I,CAS-II,CAS-III,CAS-V			
50898..51879	-	OQP93146.1	LKKY02000014.1	Clostridium sporogenes	Bacteria	Firmicutes	Clostridia	Clostridiales	Clostridiaceae	Clostridium	CRISPR-associated endonuclease Cas1	cas1	cd09722	CAS-I-B			
51878..52397	-	OQP93147.1	LKKY02000014.1	Clostridium sporogenes	Bacteria	Firmicutes	Clostridia	Clostridiales	Clostridiaceae	Clostridium	CRISPR-associated protein Cas4	cas4	pfam01930	CAS-I			
52411..54679	-	OQP93148.1	LKKY02000014.1	Clostridium sporogenes	Bacteria	Firmicutes	Clostridia	Clostridiales	Clostridiaceae	Clostridium	CRISPR-associated endonuclease/helicase Cas3	cas3HD,cas3	cd09641,COG1203	CAS-I			

530 CAS-I-B | APH14746.1

1358885..1361081	array		CP013243.1	Clostridium botulinum	Bacteria	Firmicutes	Clostridia	Clostridiales	Clostridiaceae	Clostridium		CRISPR array			34	36	29
1360807..1361023	-	APH16383.1	CP013243.1	Clostridium botulinum	Bacteria	Firmicutes	Clostridia	Clostridiales	Clostridiaceae	Clostridium	putative membrane protein						
1361271..1361535	-	APH14746.1	CP013243.1	Clostridium botulinum	Bacteria	Firmicutes	Clostridia	Clostridiales	Clostridiaceae	Clostridium	CRISPR-associated endonuclease Cas2	cas2	cd09725	CAS-I,CAS-II,CAS-III,CAS-V			
1361541..1362522	-	APH17039.1	CP013243.1	Clostridium botulinum	Bacteria	Firmicutes	Clostridia	Clostridiales	Clostridiaceae	Clostridium	CRISPR-associated endonuclease Cas1	cas1	cd09722	CAS-I-B			
1362521..1363040	-	APH16897.1	CP013243.1	Clostridium botulinum	Bacteria	Firmicutes	Clostridia	Clostridiales	Clostridiaceae	Clostridium	CRISPR-associated protein Cas4	cas4	pfam01930	CAS-I			
1363055..1365323	-	APH14926.1	CP013243.1	Clostridium botulinum	Bacteria	Firmicutes	Clostridia	Clostridiales	Clostridiaceae	Clostridium	CRISPR-associated endonuclease Cas3-HD	cas3HD,cas3	cd09641,COG1203	CAS-I			
1365378..1366140	-	APH13882.1	CP013243.1	Clostridium botulinum	Bacteria	Firmicutes	Clostridia	Clostridiales	Clostridiaceae	Clostridium	CRISPR-associated Cas5 domain protein	cas5	cd09658	CAS-I-B			
1366148..1367054	-	APH14113.1	CP013243.1	Clostridium botulinum	Bacteria	Firmicutes	Clostridia	Clostridiales	Clostridiaceae	Clostridium	CRISPR-associated autoregulator DevR family protein	cas7	cd09687	CAS-I-B			
1367046..1368744	-	APH14747.1	CP013243.1	Clostridium botulinum	Bacteria	Firmicutes	Clostridia	Clostridiales	Clostridiaceae	Clostridium	CRISPR-associated family protein	cas8b2,cas8b2	cd09754,cd09665	CAS-I-B			
1368747..1369482	-	APH17102.1	CP013243.1	Clostridium botulinum	Bacteria	Firmicutes	Clostridia	Clostridiales	Clostridiaceae	Clostridium	CRISPR-associated endoribonuclease Cas6	cas6	pfam01881	CAS-I,CAS-III			
1369514..1370573	-	APH16253.1	CP013243.1	Clostridium botulinum	Bacteria	Firmicutes	Clostridia	Clostridiales	Clostridiaceae	Clostridium	transcriptional regulator family protein	WYL	pfam13280	CAS-I,CAS-III			

531 CAS-I-B | APQ78505.1

1100306..1100732	+	APQ75740.1	CP013683.1	Clostridium botulinum	Bacteria	Firmicutes	Clostridia	Clostridiales	Clostridiaceae	Clostridium	deoR-like helix-turn-helix domain protein	WYL	COG2378	CAS-I,CAS-III			
1100872..1101289	+	APQ76192.1	CP013683.1	Clostridium botulinum	Bacteria	Firmicutes	Clostridia	Clostridiales	Clostridiaceae	Clostridium	WYL domain protein	WYL	pfam13280	CAS-I,CAS-III			
1101321..1102056	+	APQ75867.1	CP013683.1	Clostridium botulinum	Bacteria	Firmicutes	Clostridia	Clostridiales	Clostridiaceae	Clostridium	CRISPR-associated endoribonuclease Cas6	cas6	pfam01881	CAS-I,CAS-III			
1102059..1103766	+	APQ76792.1	CP013683.1	Clostridium botulinum	Bacteria	Firmicutes	Clostridia	Clostridiales	Clostridiaceae	Clostridium	CRISPR-associated family protein	cas8b2	cd09665	CAS-I-B			
1103758..1104664	+	APQ75617.1	CP013683.1	Clostridium botulinum	Bacteria	Firmicutes	Clostridia	Clostridiales	Clostridiaceae	Clostridium	CRISPR-associated autoregulator DevR family protein	cas7	cd09687	CAS-I-B			
1104672..1105578	+	APQ76776.1	CP013683.1	Clostridium botulinum	Bacteria	Firmicutes	Clostridia	Clostridiales	Clostridiaceae	Clostridium	CRISPR-associated Cas5 domain protein	cas5	cd09658	CAS-I-B			
1105632..1107900	+	APQ76676.1	CP013683.1	Clostridium botulinum	Bacteria	Firmicutes	Clostridia	Clostridiales	Clostridiaceae	Clostridium	CRISPR-associated endonuclease Cas3-HD	cas3HD,cas3	cd09641,COG1203	CAS-I			
1107914..1108433	+	APQ77519.1	CP013683.1	Clostridium botulinum	Bacteria	Firmicutes	Clostridia	Clostridiales	Clostridiaceae	Clostridium	CRISPR-associated protein Cas4	cas4	pfam01930	CAS-I			
1108432..1109413	+	APQ75332.1	CP013683.1	Clostridium botulinum	Bacteria	Firmicutes	Clostridia	Clostridiales	Clostridiaceae	Clostridium	CRISPR-associated endonuclease Cas1	cas1	cd09722	CAS-I-B			
1109419..1109683	+	APQ78505.1	CP013683.1	Clostridium botulinum	Bacteria	Firmicutes	Clostridia	Clostridiales	Clostridiaceae	Clostridium	CRISPR-associated endonuclease Cas2	cas2	cd09725	CAS-I,CAS-II,CAS-III,CAS-V			
1109874..1110754	array		CP013683.1	Clostridium botulinum	Bacteria	Firmicutes	Clostridia	Clostridiales	Clostridiaceae	Clostridium		CRISPR array			14	36	29

532 CAS-I-B | SQB33600.1

569963..571171	array		UAWC01000001.1	Clostridium cochlearium	Bacteria	Firmicutes	Clostridia	Clostridiales	Clostridiaceae	Clostridium		CRISPR array			19	36	29
571376..571640	-	SQB33600.1	UAWC01000001.1	Clostridium cochlearium	Bacteria	Firmicutes	Clostridia	Clostridiales	Clostridiaceae	Clostridium	CRISPR-associated endoribonuclease Cas2	cas2	cd09725	CAS-I,CAS-II,CAS-III,CAS-V			
571644..572037	-	SQB33601.1	UAWC01000001.1	Clostridium cochlearium	Bacteria	Firmicutes	Clostridia	Clostridiales	Clostridiaceae	Clostridium	CRISPR-associated protein Cas1	cas1	cd09722	CAS-I-B			
572050..572698	-	SQB33603.1	UAWC01000001.1	Clostridium cochlearium	Bacteria	Firmicutes	Clostridia	Clostridiales	Clostridiaceae	Clostridium	CRISPR-associated endonuclease Cas1	cas1	cd09722	CAS-I-B			
572697..573219	-	SQB33605.1	UAWC01000001.1	Clostridium cochlearium	Bacteria	Firmicutes	Clostridia	Clostridiales	Clostridiaceae	Clostridium	CRISPR-associated Cas4 family protein	cas4	pfam01930	CAS-I			
573234..575544	-	SQB33606.1	UAWC01000001.1	Clostridium cochlearium	Bacteria	Firmicutes	Clostridia	Clostridiales	Clostridiaceae	Clostridium	CRISPR-associated helicase, Cas3 family	cas3HD,cas3	cd09641,COG1203	CAS-I			
575605..576337	-	SQB33608.1	UAWC01000001.1	Clostridium cochlearium	Bacteria	Firmicutes	Clostridia	Clostridiales	Clostridiaceae	Clostridium	CRISPR-associated protein Cas5	cas5	cd09658	CAS-I-B			

576345..577248	-	SQB33610.1	UAWC01000001.1	Clostridium cochlearium	Bacteria	Firmicutes	Clostridia	Clostridiales	Clostridiaceae	Clostridium	CRISPR-associated autoregulator, Cst2 family	cas7	cd09687	CAS-I-B			
577237..578938	-	SQB33612.1	UAWC01000001.1	Clostridium cochlearium	Bacteria	Firmicutes	Clostridia	Clostridiales	Clostridiaceae	Clostridium	CRISPR-associated CXXC_CXXC protein Cst1	cas8b2	cd09665	CAS-I-B			
578942..579680	-	SQB33614.1	UAWC01000001.1	Clostridium cochlearium	Bacteria	Firmicutes	Clostridia	Clostridiales	Clostridiaceae	Clostridium	CRISPR-associated endoribonuclease Cas6	cas6	cd09652	CAS-I,CAS-III			
579742..579955	-	SQB33616.1	UAWC01000001.1	Clostridium cochlearium	Bacteria	Firmicutes	Clostridia	Clostridiales	Clostridiaceae	Clostridium	putative transcriptional regulator	WYL	pfam13280	CAS-I,CAS-III			
579970..580693	-	SQB33617.1	UAWC01000001.1	Clostridium cochlearium	Bacteria	Firmicutes	Clostridia	Clostridiales	Clostridiaceae	Clostridium	putative transcriptional regulator	WYL	COG2378	CAS-I,CAS-III			
533 CAS-I-B | STA92279.1																	
906674..907625	+	STA92270.1	UFWH01000001.1	Clostridium cochlearium	Bacteria	Firmicutes	Clostridia	Clostridiales	Clostridiaceae	Clostridium	putative transcriptional regulator	WYL	COG2378	CAS-I,CAS-III			
907687..908425	+	STA92271.1	UFWH01000001.1	Clostridium cochlearium	Bacteria	Firmicutes	Clostridia	Clostridiales	Clostridiaceae	Clostridium	CRISPR-associated endoribonuclease Cas6	cas6	cd09652	CAS-I,CAS-III			
908429..910130	+	STA92272.1	UFWH01000001.1	Clostridium cochlearium	Bacteria	Firmicutes	Clostridia	Clostridiales	Clostridiaceae	Clostridium	CRISPR-associated CXXC_CXXC protein Cst1	cas8b2	cd09665	CAS-I-B			
910119..911022	+	STA92274.1	UFWH01000001.1	Clostridium cochlearium	Bacteria	Firmicutes	Clostridia	Clostridiales	Clostridiaceae	Clostridium	CRISPR-associated autoregulator, Cst2 family	cas7	cd09687	CAS-I-B			
911030..911762	+	STA92275.1	UFWH01000001.1	Clostridium cochlearium	Bacteria	Firmicutes	Clostridia	Clostridiales	Clostridiaceae	Clostridium	CRISPR-associated protein Cas5	cas5	cd09658	CAS-I-B			
911823..914133	+	STA92276.1	UFWH01000001.1	Clostridium cochlearium	Bacteria	Firmicutes	Clostridia	Clostridiales	Clostridiaceae	Clostridium	CRISPR-associated helicase, Cas3 family	cas3HD,cas3	cd09641,COG1203	CAS-I			
914148..914670	+	STA92277.1	UFWH01000001.1	Clostridium cochlearium	Bacteria	Firmicutes	Clostridia	Clostridiales	Clostridiaceae	Clostridium	CRISPR-associated Cas4 family protein	cas4	pfam01930	CAS-I			
914669..915656	+	STA92278.1	UFWH01000001.1	Clostridium cochlearium	Bacteria	Firmicutes	Clostridia	Clostridiales	Clostridiaceae	Clostridium	CRISPR-associated endonuclease Cas1	cas1	cd09722	CAS-I-B			
915660..915924	+	STA92279.1	UFWH01000001.1	Clostridium cochlearium	Bacteria	Firmicutes	Clostridia	Clostridiales	Clostridiaceae	Clostridium	CRISPR-associated endoribonuclease Cas2	**cas2**	cd09725	CAS-I,CAS-II,CAS-III,CAS-V			
916133..919060	array		UFWH01000001.1	Clostridium cochlearium	Bacteria	Firmicutes	Clostridia	Clostridiales	Clostridiaceae	Clostridium		CRISPR array			45	36	29
534 CAS-I-B | KGN04936.1																	
647..2259	array		JDSA01000007.1	Clostridium haemolyticum NCTC 8350	Bacteria	Firmicutes	Clostridia	Clostridiales	Clostridiaceae	Clostridium		CRISPR array			25	37	29
2454..2718	-	KGN04936.1	JDSA01000007.1	Clostridium haemolyticum NCTC 8350	Bacteria	Firmicutes	Clostridia	Clostridiales	Clostridiaceae	Clostridium	CRISPR-associated protein Cas2	**cas2**	cd09725	CAS-I,CAS-II,CAS-III,CAS-V			
2721..3705	-	KGN04937.1	JDSA01000007.1	Clostridium haemolyticum NCTC 8350	Bacteria	Firmicutes	Clostridia	Clostridiales	Clostridiaceae	Clostridium	CRISPR-associated protein Cas1	cas1	cd09722	CAS-I-B			
3704..4226	-	KGN04938.1	JDSA01000007.1	Clostridium haemolyticum NCTC 8350	Bacteria	Firmicutes	Clostridia	Clostridiales	Clostridiaceae	Clostridium	CRISPR-associated protein Cas4	cas4	pfam01930	CAS-I			
4240..6535	-	KGN04939.1	JDSA01000007.1	Clostridium haemolyticum NCTC 8350	Bacteria	Firmicutes	Clostridia	Clostridiales	Clostridiaceae	Clostridium	CRISPR-associated protein Cas3	cas3HD,cas3	cd09641,COG1203	CAS-I			
6602..7331	-	KGN04940.1	JDSA01000007.1	Clostridium haemolyticum NCTC 8350	Bacteria	Firmicutes	Clostridia	Clostridiales	Clostridiaceae	Clostridium	CRISPR-associated protein Cas5	cas5	cd09658	CAS-I-B			
7339..8224	-	KGN04941.1	JDSA01000007.1	Clostridium haemolyticum NCTC 8350	Bacteria	Firmicutes	Clostridia	Clostridiales	Clostridiaceae	Clostridium	CRISPR-associated protein	cas7	cd09687	CAS-I-B			
8216..9920	-	KGN04942.1	JDSA01000007.1	Clostridium haemolyticum NCTC 8350	Bacteria	Firmicutes	Clostridia	Clostridiales	Clostridiaceae	Clostridium	CRISPR-associated protein Cst1	cas8b2,cas8b2	cd09754,cd09665	CAS-I-B			
9936..10671	-	KGN04943.1	JDSA01000007.1	Clostridium haemolyticum NCTC 8350	Bacteria	Firmicutes	Clostridia	Clostridiales	Clostridiaceae	Clostridium	CRISPR-associated protein Cas6	cas6	pfam01881	CAS-I,CAS-III			
10734..11685	-	KGN04944.1	JDSA01000007.1	Clostridium haemolyticum NCTC 8350	Bacteria	Firmicutes	Clostridia	Clostridiales	Clostridiaceae	Clostridium	hypothetical protein	WYL	COG2378	CAS-I,CAS-III			
535 CAS-I-B | KGM99193.1																	
52601..53614	array		JDRY01000038.1	Clostridium botulinum C/D str. DC5	Bacteria	Firmicutes	Clostridia	Clostridiales	Clostridiaceae	Clostridium		CRISPR array			16	36	29
53805..54069	-	KGM99193.1	JDRY01000038.1	Clostridium botulinum C/D str. DC5	Bacteria	Firmicutes	Clostridia	Clostridiales	Clostridiaceae	Clostridium	CRISPR-associated protein Cas2	**cas2**	cd09725	CAS-I,CAS-II,CAS-III,CAS-V			
54072..55056	-	KGM99194.1	JDRY01000038.1	Clostridium botulinum C/D str. DC5	Bacteria	Firmicutes	Clostridia	Clostridiales	Clostridiaceae	Clostridium	CRISPR-associated protein Cas1	cas1	cd09722	CAS-I-B			
55055..55577	-	KGM99195.1	JDRY01000038.1	Clostridium botulinum C/D str. DC5	Bacteria	Firmicutes	Clostridia	Clostridiales	Clostridiaceae	Clostridium	CRISPR-associated protein Cas4	cas4	pfam01930	CAS-I			
57953..58688	-	KGM99196.1	JDRY01000038.1	Clostridium botulinum C/D str. DC5	Bacteria	Firmicutes	Clostridia	Clostridiales	Clostridiaceae	Clostridium	CRISPR-associated protein Cas5	cas5	cd09658	CAS-I-B			
58696..59581	-	KGM99197.1	JDRY01000038.1	Clostridium botulinum C/D str. DC5	Bacteria	Firmicutes	Clostridia	Clostridiales	Clostridiaceae	Clostridium	CRISPR-associated protein	cas7	cd09687	CAS-I-B			
59582..61277	-	KGM99198.1	JDRY01000038.1	Clostridium botulinum C/D str. DC5	Bacteria	Firmicutes	Clostridia	Clostridiales	Clostridiaceae	Clostridium	CRISPR-associated protein Cst1	cas8b2,cas8b2	cd09754,cd09665	CAS-I-B			
61293..62028	-	KGM99199.1	JDRY01000038.1	Clostridium botulinum C/D str. DC5	Bacteria	Firmicutes	Clostridia	Clostridiales	Clostridiaceae	Clostridium	CRISPR-associated protein Cas6	cas6	COG1583	CAS-I,CAS-III,CAS-IV			
62091..63042	-	KGM99200.1	JDRY01000038.1	Clostridium botulinum C/D str. DC5	Bacteria	Firmicutes	Clostridia	Clostridiales	Clostridiaceae	Clostridium	hypothetical protein	WYL	COG2378	CAS-I,CAS-III			
536 CAS-I-B | EDS77981.1																	
142396..142870	+	EDS77933.1	ABDQ01000003.1	Clostridium botulinum C str. Eklund	Bacteria	Firmicutes	Clostridia	Clostridiales	Clostridiaceae	Clostridium	HTH domain family	WYL	COG2378	CAS-I,CAS-III			
143229..143358	+	EDS77897.1	ABDQ01000003.1	Clostridium botulinum C str. Eklund	Bacteria	Firmicutes	Clostridia	Clostridiales	Clostridiaceae	Clostridium	conserved hypothetical protein	WYL	pfam13280	CAS-I,CAS-III			
143408..144143	+	EDS77834.1	ABDQ01000003.1	Clostridium botulinum C str. Eklund	Bacteria	Firmicutes	Clostridia	Clostridiales	Clostridiaceae	Clostridium	crispr-associated protein Cas6	cas6	COG1583	CAS-I,CAS-III,CAS-IV			
144156..145857	+	EDS77895.1	ABDQ01000003.1	Clostridium botulinum C str. Eklund	Bacteria	Firmicutes	Clostridia	Clostridiales	Clostridiaceae	Clostridium	crispr-associated cxxc_cxxc protein Cst1	cas8b2,cas8b2	cd09754,cd09665	CAS-I-B			
145849..146743	+	EDS77882.1	ABDQ01000003.1	Clostridium botulinum C str. Eklund	Bacteria	Firmicutes	Clostridia	Clostridiales	Clostridiaceae	Clostridium	crispr-associated regulatory protein, devr family	cas7	cd09687	CAS-I-B			
146751..147486	+	EDS77817.1	ABDQ01000003.1	Clostridium botulinum C str. Eklund	Bacteria	Firmicutes	Clostridia	Clostridiales	Clostridiaceae	Clostridium	crispr-associated protein Cas5, tneap subtype	cas5	cd09658	CAS-I-B			
147556..149842	+	EDS77863.1	ABDQ01000003.1	Clostridium botulinum C str. Eklund	Bacteria	Firmicutes	Clostridia	Clostridiales	Clostridiaceae	Clostridium	crispr-associated helicase Cas3 domain protein	cas3HD,cas3	cd09641,COG1203	CAS-I			
149857..150379	+	EDS77797.1	ABDQ01000003.1	Clostridium botulinum C str. Eklund	Bacteria	Firmicutes	Clostridia	Clostridiales	Clostridiaceae	Clostridium	crispr-associated protein Cas4	cas4	pfam01930	CAS-I			
150378..151362	+	EDS77879.1	ABDQ01000003.1	Clostridium botulinum C str. Eklund	Bacteria	Firmicutes	Clostridia	Clostridiales	Clostridiaceae	Clostridium	crispr-associated protein Cas1	cas1	cd09722	CAS-I-B			
151364..151628	+	EDS77981.1	ABDQ01000003.1	Clostridium botulinum C str. Eklund	Bacteria	Firmicutes	Clostridia	Clostridiales	Clostridiaceae	Clostridium	crispr-associated protein Cas2	**cas2**	cd09725	CAS-I,CAS-II,CAS-III,CAS-V			
151814..152303	array		ABDQ01000003.1	Clostridium botulinum C str. Eklund	Bacteria	Firmicutes	Clostridia	Clostridiales	Clostridiaceae	Clostridium		CRISPR array			8	35	30
537 CAS-I-B | KEH93512.1																	
3156..3713	array		JENP01000053.1	Clostridium botulinum C/D str. BKT12695	Bacteria	Firmicutes	Clostridia	Clostridiales	Clostridiaceae	Clostridium		CRISPR array			9	37	29
3897..4161	-	KEH93512.1	JENP01000053.1	Clostridium botulinum C/D str. BKT12695	Bacteria	Firmicutes	Clostridia	Clostridiales	Clostridiaceae	Clostridium	CRISPR-associated protein Cas2	**cas2**	cd09725	CAS-I,CAS-II,CAS-III,CAS-V			
4163..5147	-	KEH93513.1	JENP01000053.1	Clostridium botulinum C/D str. BKT12695	Bacteria	Firmicutes	Clostridia	Clostridiales	Clostridiaceae	Clostridium	CRISPR-associated protein Cas1	cas1	cd09722	CAS-I-B			
5146..5668	-	KEH93514.1	JENP01000053.1	Clostridium botulinum C/D str. BKT12695	Bacteria	Firmicutes	Clostridia	Clostridiales	Clostridiaceae	Clostridium	CRISPR-associated protein Cas4	cas4	pfam01930	CAS-I			
8037..8766	-	KEH93515.1	JENP01000053.1	Clostridium botulinum C/D str. BKT12695	Bacteria	Firmicutes	Clostridia	Clostridiales	Clostridiaceae	Clostridium	CRISPR-associated protein Cas5	cas5	cd09658	CAS-I-B			
8774..9659	-	KEH93516.1	JENP01000053.1	Clostridium botulinum C/D str. BKT12695	Bacteria	Firmicutes	Clostridia	Clostridiales	Clostridiaceae	Clostridium	CRISPR-associated protein	cas7	cd09687	CAS-I-B			
9651..11358	-	KEH93517.1	JENP01000053.1	Clostridium botulinum C/D str. BKT12695	Bacteria	Firmicutes	Clostridia	Clostridiales	Clostridiaceae	Clostridium	CRISPR-associated protein Cst1	cas8b2,cas8b2,cas8b2	cd09697,cd09754,cd09665	CAS-I-B			

Table S5: List of metagenomic Cas13a used in this study

Node	Mgnify Protein ID	Mgnify ID	ENA accession	Analysis acce	Type of association to RT	Biome/Environment
1	MGYP000319188410	MGYS00001705	PRJEB1220	MGYA00113607		Fecal (Human Gut)
2	MGYP000109810180	MGYS00001705	PRJEB1220	MGYA00113496		Fecal (Human Gut)
3	MGYP000010847157	MGYS00003511	PRJEB26924	MGYA00248290		Fecal (Human Gut)
4	MGYP000684851047	MGYS00001705	PRJEB1220	MGYA00114193		Fecal (Human Gut)
5	MGYP000619297365	MGYS00002338	PRJEB24529	MGYA00156229		Fecal (Human Gut)
7	MGYP000192585983	MGYS00002417	PRJEB24766	MGYA00170107		Fecal (Human Gut)
8	MGYP000252681734	MGYS00003511	PRJEB26924	MGYA002482	Frameshifted RT-Cas1	Fecal (Human Gut)
10	MGYP000167051486	MGYS00003511	PRJEB26924	MGYA00248317		Fecal (Human Gut)
11	MGYP000165542897	MGYS00001799	PRJEB6456	MGYA00128896		Fecal (Human Gut)
12	MGYP000162821430	MGYS00003469	PRJEB26280	MGYA00242000		Fecal (Human Gut)
13	MGYP000058379904	MGYS00002331	PRJEB24528	MGYA00153894		Fecal (Human Gut)
14	MGYP000029912630	MGYS00002019	PRJEB22360	MGYA00377259		Fecal (Human Gut)
15	MGYP000109203063	MGYS00002338	PRJEB24529	MGYA00156217		Fecal (Human Gut)
16	MGYP000120911204	MGYS00002338	PRJEB24529	MGYA00156303		Fecal (Human Gut)
17	MGYP000668338691	not available	not available	not available		Fecal (Human Gut)
18	MGYP000515010317	not available	not available	not available		Fecal (Human Gut)
19	MGYP000728120183	MGYS00002415	PRJEB24748	MGYA00170074		Fecal (Human Gut)
20	MGYP000705176138	MGYS00001799	PRJEB6456	MGYA00128808		Fecal (Human Gut)
22	MGYP000160729454	MGYS00001985	PRJEB22368	MGYA00137949		Fecal (Human Gut)
31	MGYP000178941783	MGYS00003511	PRJEB26924	MGYA002482	RT-Cas1	Fecal (Human Gut)
33	MGYP000505721386	MGYS00001985	PRJEB22368	MGYA00376144		Fecal (Human Gut)
35	MGYP000742642845	not available	not available	not available		Fecal (Human Gut)
36	MGYP000661043385	MGYS00003469	PRJEB26280	MGYA002420	RT-Cas1	Fecal (Human Gut)
37	MGYP000217302039	MGYS00003511	PRJEB26924	MGYA00248302		Fecal (Human Gut)
38	MGYP000615622314	not available	not available	not available		Fecal (Human Gut)
39	MGYP000644147385	MGYS00003479	PRJEB24818	MGYA00243182		Fecal (Human Gut)
41	MGYP000516651569	MGYS00003469	PRJEB26280	MGYA00241986		Fecal (Human Gut)
43	MGYP000038574051	MGYS00003468	PRJEB25962	MGYA00241137		Fecal (Human Gut)
45	MGYP000546064296	MGYS00003469	PRJEB26280	MGYA00242065		Fecal (Human Gut)
46	MGYP000077493486	MGYS00001705	PRJEB1220	MGYA00113606		Fecal (Human Gut)
47	MGYP000027519385	MGYS00002417	PRJEB24766	MGYA00170173		Fecal (Human Gut)
48	MGYP000518759138	MGYS00002415	PRJEB24748	MGYA00169965		Fecal (Human Gut)
49	MGYP000309732945	MGYS00002685	PRJEB25193	MGYA00198716		Fecal (Human Gut)
59	MGYP000123498965	MGYS00003372	PRJEB26909	MGYA00238139		Wet fermentation
61	MGYP000698521930	MGYS00003372	PRJEB26909	MGYA00238139		Wet fermentation
62	MGYP000751596458	MGYS00003373	PRJEB26910	MGYA00238140		Wet fermentation
63	MGYP000128950304	MGYS00003322	PRJEB26297	MGYA002378	Cas6-RT-Cas1	Sediment
78	MGYP000324658027	MGYS00001980	PRJEB22283	MGYA00137045		Oral
79	MGYP000365881605	MGYS00001980	PRJEB22283	MGYA00136941		Oral
80	MGYP000442596835	MGYS00001980	PRJEB22283	MGYA00136917		Oral
81	MGYP000041399292	MGYS00001980	PRJEB22283	MGYA00137408		Oral
83	MGYP000053356571	MGYS00001980	PRJEB22283	MGYA00137385		Oral
84	MGYP000578087195	MGYS00001980	PRJEB22283	MGYA00137035		Oral
85	MGYP000209636426	MGYS00001980	PRJEB22283	MGYA00136823		Oral
86	MGYP000191921353	MGYS00001980	PRJEB22283	MGYA00136787		Oral
87	MGYP000728506240	MGYS00001980	PRJEB22283	MGYA00136765		Oral
88	MGYP000710930945	MGYS00001980	PRJEB22283	MGYA00137422		Oral
91	MGYP000295084724	MGYS00001980	PRJEB22283	MGYA00137292		Oral
93	MGYP000309810837	MGYS00001980	PRJEB22283	MGYA00137190		Oral
94	MGYP000486752824	MGYS00001980	PRJEB22283	MGYA00137137		Oral
95	MGYP000424752507	MGYS00001980	PRJEB22283	MGYA00137466		Oral
96	MGYP000070896523	MGYS00001980	PRJEB22283	MGYA00137213		Oral
97	MGYP000356942902	MGYS00001980	PRJEB22283	MGYA00137027		Oral
98	MGYP000430768964	MGYS00001980	PRJEB22283	MGYA00136787		Oral
99	MGYP000684248307	MGYS00001980	PRJEB22283	MGYA00136829		Oral
100	MGYP000130031563	MGYS00001980	PRJEB22283	MGYA00137246		Oral
101	MGYP000666568610	MGYS00001980	PRJEB22283	MGYA00137052		Oral
102	MGYP000250759845	MGYS00001980	PRJEB22283	MGYA00136998		Oral
103	MGYP000353980233	MGYS00001980	PRJEB22283	MGYA00137035		Oral
104	MGYP000168334215	MGYS00001980	PRJEB22283	MGYA00136787		Oral

105	MGYP000280275645	MGYS00001980	PRJEB22283	MGYA00137409	Oral
106	MGYP000082840345	MGYS00001980	PRJEB22283	MGYA00136890	Oral
107	MGYP000578150587	MGYS00001980	PRJEB22283	MGYA00137012	Oral
108	MGYP000701927337	MGYS00001980	PRJEB22283	MGYA00136999	Oral
109	MGYP000194886005	MGYS00001980	PRJEB22283	MGYA00137422	Oral
110	MGYP000643044933	MGYS00001980	PRJEB22283	MGYA00137468	Oral
112	MGYP000421818960	MGYS00001980	PRJEB22283	MGYA00136806	Oral
113	MGYP000501496954	MGYS00001980	PRJEB22283	MGYA00137306	Oral
114	MGYP000439636926	MGYS00001980	PRJEB22283	MGYA00137422	Oral
115	MGYP000271550373	MGYS00001980	PRJEB22283	MGYA00137073	Oral
116	MGYP000513213728	MGYS00001980	PRJEB22283	MGYA00137038	Oral
119	MGYP000177060630	MGYS00001980	PRJEB22283	MGYA00137332	Oral
121	MGYP000318745293	MGYS00001980	PRJEB22283	MGYA00137025	Oral
122	MGYP000722609020	MGYS00001980	PRJEB22283	MGYA00136765	Oral
123	MGYP000103429604	MGYS00001980	PRJEB22283	MGYA00136820	Oral
124	MGYP000740329489	MGYS00001980	PRJEB22283	MGYA00136865	Oral
125	MGYP000374686211	MGYS00001980	PRJEB22283	MGYA00136947	Oral
126	MGYP000256833066	MGYS00001980	PRJEB22283	MGYA00137246	Oral

Table S6: Analysis of metagenomic Type VI-A/RT systems

Node	Metagenome - Contig/Coordinates	strand	Mgnify ID	cas_gene	profiles
8	ERZ651911 (ENA-OWSA01001969-OWSA01001969.1-human-gut-metagenome-genome-assembly--contig:-NODE-1969-length-21349-cov-3.830563)				
	1893..2118			array (4 spacers)	
	4820..5056			array (4 spacers)	
	5357..5674	+		csx21	mkCas0125
	5730..5936	+			
	6055..6315	+		cas2	cd09725
	6329..7645	+		**RT,cas1***	pfam00078,cd09722
	7731..8294	+		cas1	cd09722
	8518..12591	+	MGYP000252681734	**cas13a**	cas13a
31	ERZ651842 (ENA-OWPD01000104-OWPD01000104.1-human-gut-metagenome-genome-assembly--contig:-NODE-104-length-107636-cov-7.514431)				
	16200..16511			array (5 spacers)	
	16717..18612	-		**RT,cas1**	pfam00078,cd09722
	18625..18885	-		cas2	cd09725
	18889..23103	-	MGYP000178941783	**cas13a**	cas13a
36	ERZ509143 (ENA-ORRV01000023-ORRV01000023.1-human-gut-metagenome-genome-assembly--contig:-NODE-23-length-119425-cov-4.85302)				
	39046..43146	+	MGYP000661043385	**cas13a**	cas13a
	43146..43406	+		cas2	cd09725
	43424..45316	+		**RT,cas1**	pfam00078,cd09722
	45482..46319			array (5 spacers)	
40	ERZ511465 (ENA-ORXH01001121-ORXH01001121.1-sediment-metagenome-genome-assembly--contig:-NODE-1121-length-27569-cov-548.055034)				
	14978..16216	-		csx1	pfam09002
	16250..16522	-		cas2	pfam09827
	16526..19495	-		**cas6,RT,cas1**	pfam10040,pfam00078,cd09634
	19850..20227			array	
	20451..24398	-	MGYP000128950304	**cas13a**	cas13a
	24534..26091	+		corA	cluster29

File 1

((MGYP000103429604:0.03669000000000011,MGYP000740329489:0.01778000000000013):0.012199999999999989,(MGYP000374686211:0.04863000000000017,('WP_021746774.1 [Leptotrichia wadei F0279]':0.01202000000000586,MGYP000256833066:0.00769000000000197):0.012299999999999756):0.07542999999999989,(MGYP000722609020:0.0066600000000011,(MGYP000318745293:0.03798000000000125,(('WP_071125398.1 [Leptotrichia massiliensis]':0.003219999999997786,(MGYP000177060630:0.02134000000000036,'WP_021768357.1 [Leptotrichia sp. oral taxon 225 str. F0581]':0.01268000000000469):0.01487000000000016):0.06524000000000019,((MGYP000439636926:0.06400000000000006,(MGYP000271550373:0.01681000000000436,(MGYP000513213728:0.12740999999999936,'WP_015770004.1 CRISPR-associated endoribonuclease Cas13a [Leptotrichia buccalis C-1013-b]':0.003109999999999502):0.03129000000000026):0.011560000000000237):0.034569999999999546,(((MGYP000353980233:0.01004999999999967,(MGYP000168334215:0.020299999999999763,(MGYP000250759845:0.02885000000000264,MGYP000666568610:0.04389999999999983):0.014999999999999968):0.012190000000000367):0.10447000000000006,(MGYP000280275645:0.11043000000000003,((MGYP000082840345:0.04557000000000555,(MGYP000578150587:0.05919000000000076,(MGYP000701927337:0.029959999999999987,MGYP000194886005:0.05093000000000014):0.02613999999999983):0.02974000000000032):0.07861999999999991,(MGYP000643044933:0.0828199999999999,('A_ERK47820.1 hypothetical protein HMPREF9015_02301 [Leptotrichia wadei F0279] Copy':0.09128000000000025,(MGYP000421818960:0.02617000000000047,MGYP000501496954:0.03186):0.019289999999999807):0.040919999999999845):0.012430000000000163):0.028599999999999996):0.05259000000000036):0.4427899999999996,((MGYP000295084724:0.04516999999999971,(('WP_071124126.1 [Leptotrichia massiliensis]':0.04970000000000052,(MGYP000309810837:0.01667000000000407,(MGYP000486752824:0.023439999999999905,MGYP000424752507:0.0078699999999996):0.02291000000000043):0.04096999999999973):0.015789999999999997,(MGYP000070896523:0.01784000000000522,(MGYP000356942902:0.03600000000000476,(MGYP000430768964:0.02130000000000097,(MGYP000130031563:0.000190,MGYP000684248307:0.028539999999999566):0.01742000000000435):0.011099999999999888):0.02102000000000004):0.04429000000000016):0.02285000000000037):0.3803599999999996,(('WP_018451595.1 type VI-A CRISPR-associated effector C2c2 [Leptotrichia shahii DSM 19757]':0.11447999999999992,'WP_021744063.1 type VI-A CRISPR-associated effector C2c2 [Leptotrichia sp. oral taxon 879]':0.20070999999999994):2.4209999999999994,(('WP_079495749.1 [Maledivibacter halophilus]':1.6884899999999998,('WP_118572797.1 [Ruminococcus sp. AM40-10AC]':1.2144399999999997,(('WP_117998314.1 [Eubacterium rectale AF19-4]':0.8900800000000002,('WP_118614261.1 [Ruminococcus sp. TF11-2AC]':0.9056999999999995,'WP_062808098.1 [Blautia sp. Marseille-P2398]':0.7869299999999999):0.1234099999999998):0.20211000000000023,(('WP_022785443.1 CRISPR-associated endoribonuclease Cas13a [Lachnospiraceae bacterium NK4A179]':0.7418900000000002,('WP_089928016.1 [Lachnospiraceae bacterium NE2001]':0.6804000000000001,('WP_092321585.1 [Butyrivibrio sp. YAB3001]':0.5730899999999997,('WP_044921188.1 [Lachnospiraceae bacterium MA2020]':0.5878399999999999,'WP_090551759.1 [Pseudobutyrivibrio sp. OR37]':0.46628999999999987):0.11029):0.12456999999999985):0.15542999999999996):0.10067000000000004,(('WP_027114339.1 [Lachnospiraceae bacterium NK4A144]':0.5036,'WP_031473346.1 [Clostridium] aminophilum strain F]':0.53152):0.16588000000000003,(('WP_087253216.1 [Drancourtella sp. An57]':0.1110199999999999,'WP_090127496.1[Eubacteriaceae bacterium CHKCI004]':0.06496999999999975):0.3734899999999999,('WP_055061018.1

[Eubacterium rectale T1-815]':0.0011999999999998678,('WP_117482613.1
[Eubacterium rectale TM10-3]':0.009970000000000034,'WP_118003838.1
[Eubacterium rectale AF25-25/AF18-
16LB]':0.000760):0.005090000000000039):0.46533999999999986):0.42507999999
99999):0.12597000000000014):0.41186999999999996):0.16868999999999978):1.1
358800000000002):0.20164999999999988,(('WP_103203632.1 CRISPR-associated
endoribonuclease Cas13a [Herbinix
hemicellulosilytica]':1.6666299999999996,'WP_021746003.1 CRISPR-
associated endoribonuclease Cas13a [Leptotrichia wadei
F0279]':1.1635):0.2255900000000004,((('WP_071146234.1 [Bacteroides
ihuae]':0.8769800000000001,'WP_013443710.1 CRISPR-associated
endoribonuclease Cas13a [Paludibacter
propionicigenes]':0.6133500000000001):0.3482799999999999,(('WP_034560163.
1 [Carnobacterium gallinarum]':0.6043600000000002,'WP_034563842.1
[Carnobacterium
gallinarum]':0.5808599999999999):0.47219999999999995,(('WP_036091002.1
[Listeria newyorkensis]':0.024630000000000152,'WP_118907415.1 [Listeria
weihenstephanensis]':0.006060000000000176):0.68824,('WP_099225408.1
[Listeria costaricensis]':0.7599399999999998,'WP_012985477.1 CRISPR-
associated endoribonuclease Cas13a [Listeria
seeligeri]':0.6244000000000001):0.18751000000000007):0.23184000000000005)
:0.08113999999999999):0.46060999999999996,(MGYP000128950304:1.12963999999
99998,(('WP_133318297.1 [Rhizobium sp. SPY-1]':1.19238,('WP_137134457.1
[Rhizobium sp. FKY42]':1.1632100000000003,('WP_080615427.1 hypothetical
protein [Rhodovulum sp. MB263]':0.0210600000000003,'WP_108028905.1
hypothetical protein [Rhodovulum
kholense]':0.05811999999999973):0.9795299999999996):0.22865000000000002):
0.8541400000000001,('WP_023911507.1 [Rhodobacter
capsulatus]':1.24486,('OQX30025.1 hypothetical protein B0D92_00635,
partial [Spirochaeta sp. LUC14_002_19_P3]':1.0509,('WP_133357912.1
[Ruegeria sp. 318-1]':1.2563500000000003,(('WP_076398593.1
[Insolitispirillum peregrinum]':1.09091,'WP_112317339.1 [Rhodovulum
viride]':0.8513799999999998):0.16633999999999993,(('WP_073955355.1
[Thalassospira sp. TSL5-1]':0.06113999999999997,'WP_114086813.1
[Thalassospira
profundimaris]':0.07535999999999987):0.7751100000000002,('WP_132694182.1
[Rhodovulum steppense]':0.6561200000000005,('PJI41863.1 hypothetical
protein CTR53_05225 [Ferrovibrio sp.]':0.7410199999999998,'WP_100176879.1
[Bradyrhizobium sp.
TSA1]':0.6814799999999996):0.10904999999999987):0.2228800000000004):0.156
26000000000007):0.12727000000000022):0.07217999999999991):0.1305999999999
9983):0.14292000000000016):0.27281999999999984):1.14132):0.13243000000000
027):0.11902999999999953):0.21072999999999986):0.7106500000000002):0.1469
3999999999985):0.35541):0.032900000000000595):0.016439999999999344):0.017
82000000000039):0.022009999999999863);

File 2

((((((((((((((((((('EHO48118.1':0.009609999999999896,'KXB53086.1':0.0192
1000000000017):0.012570000000000192,'ETO97675.1':0.05331000000000019):0.0
2932999999999856,'AYA99695.1':0.04356999999999989):0.03218999999999994,'
EFU76454.1':0.04029000000000016):0.3896799999999998,'SCW73731.1':0.480989
9999999998):0.20441999999999982,'WP_090127495.1':0.5854500000000002):0.09
467999999999988,('WP_087253222.1':0.25580000000000025,'RGR54906.1':0.3365
2000000000015):0.32098000000000004):0.05561999999999978,((((('EEG94369.1'
:0.002909999999999968,'CRL43259.1':0.000160):0.24241000000000001,('RGX906
66.1':0.09629999999999983,'HAT88295.1':0.05188000000000015):0.17923):0.05
663000000000018,'HCR68574.1':0.2791100000000002):0.10853000000000002,(('O
LA06495.1':0.002489999999999881,'SOB72962.1':0.009329999999999838):0.2852
7000000000013,'HBZ63503.1':0.5019900000000002):0.08228999999999997):0.189
28000000000011,'SFB66471.1':0.9236599999999999):0.08298000000000005):0.09
018000000000015,(('CDC93231.1':0.22463999999999995,'CUN56577.1':0.2484099
999999998):0.3230900000000001,'RGI19592.1':0.4778500000000001):0.23367000
000000004):0.10171000000000019,'RGG82469.1':0.6176599999999999):0.1155599
9999999989,'OUQ05160.1':0.7907800000000003):0.09297999999999984,('SCX1201
8.1':0.04805999999999999,'SCX12129.1':0.029900000000000038):1.15525999999
99997):0.2715099999999999,(((('ADQ41960.1':0.0033599999999998076,'AEM7491
9.1':0.003369999999999873):0.006769999999999943,'ACM61721.1':0.0066799999
99999797):0.012290000000000134,'ADQ47296.1':0.015080000000000204):0.36955
99999999999,'AEM74967.1':0.5193500000000002):0.23468000000000022):1.40174
,'SFR81217.1':0.8797199999999998):0.16796999999999995,'SFE73823.1':0.4237
700000000002):0.09194000000000013,(((((((((((('EEV02630.1':0.0,'VCV21263
.1':0.0):0.000180,('RHG29044.1':0.0043199999999998795,'CBL13036.1':0.0001
70):0.008730000000000349):0.0032600000000000406,'RHF95484.1':0.0032600000
000000406):0.003229999999999844,'OLA55795.1':0.006590000000000096):0.0032
399999999999096,('RHA66006.1':0.0,'RHM06245.1':0.0):0.000170):0.103949999
99999976,'HAD66488.1':0.18574999999999964):0.03941000000000017,('CUO30722
.1':0.11990999999999996,'EOS81372.1':0.19278999999999957):0.0491200000000
00275):0.01322000000000001,(('CDE69450.1':0.0853299999999999,'RHP82145.1'
:0.1226799999999999):0.12227999999999994,'CUN22620.1':0.13144):0.03912000
0000000044):0.02768000000000015,('RHP10704.1':0.15134999999999987,'CDD663
08.1':0.2591700000000001):0.02588999999999997):0.03637999999999986,'RGG19
527.1':0.14653000000000027):0.07335000000000003,('EFE91105.1':0.000170,'E
RL20509.1':0.01637999999999984):0.3865700000000003):0.07103999999999999,'
RGK40245.1':0.2616399999999999):0.10307999999999984,'RRD93722.1':0.507979
9999999999):0.10777000000000037):0.0623800000000001,(((((((((('EES78311.
1':0.003369999999999873,'RJW30536.1':0.000160):0.08349000000000029,'ERI97
392.1':0.08144000000000018):0.03915999999999986,'SCG89713.1':0.1462200000
0000002):0.03892999999999969,(('HAJ41005.1':0.008919999999999817,'HCS8389
7.1':0.014800000000000146):0.12186999999999992,('RGB74594.1':0.0060000000
00000227,'RGC44628.1':0.00387000000000004):0.13976999999999995):0.0480399
9999999986):0.04190000000000005,'CUQ01118.1':0.15151999999999965):0.09566
000000000008,'CUO70210.1':0.2516099999999999):0.03559000000000001,'CCX734
55.1':0.17552999999999974):0.05361000000000038,(('CCX82921.1':0.007690000
000000197,'OKZ95402.1':0.008929999999999882):0.09681999999999968,'HCW3873
0.1':0.12901000000000007):0.20804000000000045):0.05512999999999968,'EMZ39
669.1':0.3133300000000001):0.10096000000000016,(((('SET01848.1':0.0212900
0000000003,'SFR79451.1':0.0017399999999998528):0.33972000000000024,'EFV01
914.1':0.40961000000000025):0.07742000000000004,'SER78299.1':0.3985500000
000002):0.04213999999999844,'EEF92713.1':0.36213000000000006):0.11091000
000000006):0.0898399999999997,('WP_062808097.1':0.4680399999999998,'SEQ67
608.1':0.7954099999999995):0.18255):0.10500000000000043):0.10580999999999

996,((((('EFG29102.1':0.0323500000000001,'EKA93813.1':0.02374000000000009
4):0.73081,('ABI69047.1':0.10720000000000018,'OQB73705.1':0.1438799999999
9979):0.4852000000000003):0.13352999999999993,'ADG82860.1':0.555140000000
0002):0.07168999999999981,(('EFQ03341.1':0.5949999999999998,'EFR42041.1':
0.44395000000000007):0.20797,'EAX48915.1':0.5992199999999999):0.230329999
99999992):0.16735000000000033,'RGF90396.1':0.5432800000000002):0.08906999
999999998):0.06476999999999977,(((('EGG91032.1':0.11122999999999994,'EJZ6
8972.1':0.13180000000000014):0.23763999999999985,'EHI56780.1':0.399229999
99999975):0.09970999999999997,('ESL02973.1':0.40144,'PTL29107.1':0.513739
9999999999):0.09106999999999976):0.09548000000000023,'CUO16841.1':0.31604
00000000001):0.1964999999999999):0.03268000000000004,(((('CCZ41463.1':0.0
00170,'KQC84610.1':0.000160):0.0031400000000001427,'RHP28154.1':0.000160)
:0.4097200000000001,'SCG93998.1':0.4741700000000004):0.11573999999999973,
(('EMZ15703.1':0.3232499999999998,'RHR83755.1':0.45387999999999984):0.098
99999999999975,'ASS37303.1':0.8021199999999999):0.09471000000000007):0.05
729000000000006,('SES98376.1':0.4644599999999999,'OLR64025.1':0.424490000
00000003):0.09406999999999988);

File 3

(('SHJ82104.1':0.2099599999999997,('SFM78002.1':0.3569400000000007,('ACV6
4952.1':0.37351000000000045,'SDZ02948.1':0.16039000000000048):0.108530000
00000002):0.011869999999999159):0.021010000000000417,((('SHI17228.1':0.11
294000000000004,'SHD77697.1':0.10476999999999936):0.08694999999999986,('S
DZ39330.1':0.10588000000000086,('SDW90812.1':0.2435199999999993,(('SES905
32.1':0.049430000000000085,'SHK38018.1':0.09793000000000074):0.1300199999
9999914,('KXG74356.1':0.09936999999999951,('KGG79513.1':0.015559999999999
796,'SHK18635.1':0.029619999999999536):0.04903999999999975):0.05611999999
999995):0.021160000000000068):0.05265000000000075):0.09003999999999923):0
.09813000000000027,((('AEE97372.1':0.20785999999999927,('SHJ60937.1':0.23
30300000000003,('ABB14072.1':0.014810000000000656,'GAV23064.1':0.000200):
0.14571999999999985):0.1290299999999993):0.08215000000000039,(('OQX85653.
1':0.42048999999999914,('AMM41387.1':0.23155000000000037,'PWI57015.1':0.4
192):0.03608999999999973):0.12130000000000063,('SHG49963.1':0.23704000000
000036,('KUK35957.1':0.3385100000000003,('GAF26200.1':0.13015000000000043
,'PRR75058.1':0.06899000000000033):0.01881999999999837):0.09339000000000
03):0.02059999999999995):0.05229999999999979):0.02656999999999954,((('EGL
83992.1':0.09309000000000012,'KYC61680.1':0.19993999999999978):0.04827000
000000048,('AXF57070.1':0.0774499999999998,('ONK21387.1':0.07446000000000
019,'PWA13090.1':0.12706000000000017):0.1272599999999997):0.1690500000000
0037):0.04610999999999965,(('PDO09916.1':0.17708999999999975,('KIO69860.1
':0.000180,'PAC34137.1':0.04577000000000009):0.2836399999999992):0.055150
000000000254,('OBY76623.1':0.18473000000000006,(('GAC90887.1':0.139380000
00000006,('ADP76105.1':0.000200,('ANB64529.1':0.060580000000000744,('KYD3
2438.1':0.015139999999998821,'SFA45838.1':0.014999999999998792):0.000200)
:0.030079999999999885):0.05293999999999954):0.0889300000000004,((('ACS232
38.1':0.000190,'OAT71453.1':0.02958000000000016):0.02750999999999948,(('G
AD15322.1':0.015269999999999229,'ALA70693.1':0.000180):0.0300900000000003
94,('KYD25558.1':0.015060000000000073,'AMX84678.1':0.000180):0.000190):0.
07094999999999985):0.03884000000000043,(('RAK19108.1':0.01046000000000013
6,('KZM55205.1':0.0,'KZN97410.1':0.0):0.022540000000000227):0.07137999999
999955,('OOE05107.1':0.01515999999999984,('CUA79980.1':0.0147000000000003
8,('OAO81655.1':0.014980000000000437,'ASA97889.1':0.000150):0.03219999999
999956):0.014750000000000263):0.024309999999999832):0.05257000000000023):
0.02118999999999982):0.000210):0.056549999999999656):0.020970000000000155
):0.20418999999999965):0.05442000000000036):0.03200000000000003,(((('OYD0
6232.1':0.36972999999999967,('AQS56075.1':0.11869999999999958,('OUM88221.
1':0.0475500000000002,('SFU92278.1':0.34050000000000047,('ADG07371.1':0.0
4242000000000079,'ATY85843.1':0.003169999999999895):0.08382999999999985):
0.13316000000000017):0.027259999999999174):0.1342800000000004):0.11756999
999999973,('ABO49086.1':0.2120999999999995,(('AEG61207.1':0.1690699999999
996,('CUH97861.1':0.10562999999999967,'BBB93527.1':0.17173000000000016):0
.24765000000000015):0.039189999999999614,(('KFD40924.1':0.047229999999999
88,'HAG08308.1':0.026189999999999714):0.12274999999999991,(('OPX90215.1':
0.1301300000000003,'HBC94872.1':0.05677000000000021):0.05356000000000005,
('BAF60094.1':0.14072999999999958,(('AGL00364.1':0.06111999999999984,'OAT
86764.1':0.04389000000000021):0.026720000000000077,(('CCO07749.1':0.05339
999999999989,'AQS58408.1':0.14010999999999996):0.04537000000000013,('SHF2
9859.1':0.11897000000000002,'PHJ39528.1':0.017400000000000304):0.02992999
9999999346):0.029640000000000555):0.019089999999999385):0.077620000000000
47):0.04317999999999955):0.028200000000000003):0.02043000000000017):0.062
6199999999999):0.0745900000000006,((('KEQ26917.1':0.15137,('AGA59185.1':0
.07509000000000032,'OUM86956.1':0.062289999999999957):0.25577000000000005
):0.0942400000000001,(('KGP75685.1':0.000210,'HBW37344.1':0.0439300000000

0047):0.16936999999999998,('OZC22041.1':0.08165000000000067,('EHL03891.1':
0.06490000000000062,'SEP04969.1':0.09109999999999996):0.016210000000000005
8):0.08563999999999972):0.060900000000000176):0.058390000000000164,(('ATW
27429.1':0.23245000000000005,(('ADG83325.1':0.01407999999999987,'KNZ69508
.1':0.000180):0.052810000000000024,('KJS01564.1':0.019089999999999385,'PK
M81768.1':0.17225000000000001):0.0935700000000006):0.21617999999999996):0.
09752000000000027,(('SNX53624.1':0.12385000000000002,('AGB20220.1':0.0001
90,(('ADL70063.1':0.01470000000000038,'OXT08473.1':0.0142199999999999):0.
028889999999999638,('ORX22326.1':0.043260000000000076,('AEF18275.1':0.000
200,'ETO39374.1':0.014259999999999273):0.000200):0.028539999999999566):0.
000210):0.1018699999999999):0.04476000000000013,(('KHO61714.1':0.01472000
000000051,(('SDG65044.1':0.0,'SFE77437.1':0.0):0.000140,('EEU62463.1':0.0
00190,'ADH61854.1':0.04647999999999897):0.014649999999999608):0.000200):0
.07946000000000009,('ERM93083.1':0.14832999999999963,('SHF39031.1':0.0707
1000000000005,('GAQ24762.1':0.17349999999999977,('CCP24806.1':0.072659999
99999995,(('SFP76577.1':0.10728999999999989,('RKL62013.1':0.0862100000000
0034,'AYO29338.1':0.08924999999999983):0.03878999999999966):0.04320000000
000057,('SHM89755.1':0.13246000000000002,('ADL06868.1':0.1959900000000001
,('KXG75851.1':0.04043000000000063,'SHM76385.1':0.07719000000000076):0.02
346000000000036):0.0814199999999996):0.07549000000000028):0.046979999999
99958):0.06369999999999987):0.08227000000000029):0.04502000000000006):0.0
4051000000000027):0.06135999999999964):0.023550000000000182):0.0406000000
00000414):0.022009999999999863):0.08721999999999941,((('OUN00039.1':0.539
32,'PSR32273.1':0.6657599999999997):0.08396000000000026,(('SDH44542.1':0.
2802900000000008,(('ABP65721.1':0.000190,('ACM61720.1':0.0440399999999998
6,'ADQ47295.1':0.000190):0.029139999999999944):0.3654599999999997,('ADL43
512.1':0.000200,('ADQ42003.1':0.01420999999999939,'AEM74968.1':0.000200):
0.014660000000000117):0.2816299999999998):0.11747000000000085):0.03493999
999999975,(('SHF05235.1':0.24627,('CVI72704.1':0.15303000000000022,'SCJ70
961.1':0.19453999999999994):0.09405000000000019):0.06442000000000014,('KI
R01267.1':0.3132999999999999,(('SCL81849.1':0.33394999999999975,'SHI82189
.1':0.16548000000000052):0.13555000000000028,(('APM39055.1':0.29199000000
00002,(('HBF78048.1':0.0,'HBG37782.1':0.0):0.19257999999999953,(('KXG4309
9.1':0.014760000000000773,'GAU64090.1':0.000180):0.1607099999999999,('CCJ
34755.1':0.17049999999999965,('ABS60793.1':0.000190,'ACJ75697.1':0.059249
99999999958):0.18097999999999992):0.10277000000000047):0.1320499999999995
6):0.1083400000000001):0.000190,('OFI05275.1':0.1763199999999996,(('OPJ62
159.1':0.12405000000000044,('SDL41597.1':0.014520000000000088,('SQB33504.
1':0.0,'STA92354.1':0.0):0.000190):0.14544000000000068):0.025369999999999
67,('OOL91278.1':0.11724999999999941,(('PRR82599.1':0.02878000000000025,'
HBC95768.1':0.000140):0.051889999999999326,('OBR96939.1':0.01480999999999
9768,('OAA83838.1':0.015229999999999855,'RMD02178.1':0.000190):0.000190):
0.06034999999999968):0.03852000000000011):0.14965000000000028):0.08530999
999999977):0.0501100000000001):0.03715000000000046):0.05433999999999983):
0.05708000000000002):0.03612999999999995):0.07939000000000007):0.0499000
00000000055,(('GAQ95513.1':0.17368000000000006,('PMP93676.1':0.2223399999
9999998,('ROL57216.1':0.3062100000000001,('AIH04577.1':0.3458800000000002
,'PMP82994.1':0.21828000000000003):0.1364200000000002):0.000190):0.037759
99999999957):0.07901000000000025,(('BAT71822.1':0.5232000000000001,'PIP11
833.1':0.2131400000000001):0.1309699999999996,((((('EFC90514.1':0.1888399
999999999,'HAG23229.1':0.2896200000000002):0.22656999999999972,(('AUG5692
9.1':0.0,'PQQ68312.1':0.0):0.2605699999999995,('ABN53498.1':0.000200,'GAE
87583.1':0.07555000000000067):0.20825999999999922):0.30511):0.20846000000
000053,(('PMP68059.1':0.35951999999999984,('ACI17783.1':0.027090000000000

28,'HAR40633.1':0.06052999999999997):0.23353999999999964):0.1668000000000
0028,('OIP13913.1':0.44693999999999967,(('PMQ02055.1':0.21518000000000015
,('ACI19268.1':0.08082999999999974,'ACK41906.1':0.0867300000000002):0.257
48000000000015):0.06852999999999998,('SJK85391.1':0.43290000000000006,('A
GO61204.1':0.2023999999999999,'SMD31391.1':0.14885000000000037):0.4000399
9999999973):0.08105999999999991):0.04244000000000003):0.04581000000000035
):0.038899999999999935):0.08671999999999969,((('AEW06742.1':0.38253000000
000004,('POB09389.1':0.18551000000000029,'PSR26896.1':0.2128300000000003)
:0.14423999999999992):0.5365700000000002,(('GBD19029.1':0.59266,('GBC9271
2.1':0.30189999999999984,('KPL89581.1':0.4691900000000002,('ABG03233.1':0
.3384900000000002,'ACZ43326.1':0.48204):0.2890800000000002):0.07255999999
999929):0.08485000000000031):0.1498999999999997,('AEH51236.1':0.374329999
9999996,('CEP66644.1':0.2532399999999999,(('SMB98803.1':0.148800000000000
04,('RPF46988.1':0.19084000000000012,('ACX51816.1':0.000190,'RDV81757.1':
0.031489999999999796):0.09191000000000038):0.09175999999999984):0.0675499
9999999978,(('SHF40633.1':0.07127000000000017,('AEG14082.1':0.01540000000
000008,'SHI96005.1':0.000190):0.027000000000000135):0.000190,('HBT47360.1
':0.12675999999999998,(('AAM25776.1':0.000180,'ERM92128.1':0.015159999999
99984):0.016820000000000057,('KUK08303.1':0.030990000000000073,('AIS53483
.1':0.015160000000000284,'KKC28636.1':0.015190000000000037):0.000200):0.0
13869999999999827):0.13604000000000038):0.18958999999999993):0.1024399999
9999964):0.12335000000000029):0.20094999999999974):0.16849999999999987):0
.0861300000000007):0.06851999999999947,('ELZ42758.1':1.4132499999999997,(
(('OUQ05161.1':0.4385300000000001,'RGG82470.1':0.5059100000000001):0.1530
3000000000022,(('RGI19744.1':0.5004300000000002,('CDC93229.1':0.149610000
00000002,'CUN56512.1':0.14956999999999976):0.07699999999999996):0.1906600
0000000027,('WP_090127494.1':0.7082000000000006,('KXT61353.1':0.000190,('
EGU65364.1':0.07536999999999994,'KXT86661.1':0.10758000000000001):0.01215
0000000000105):0.2516900000000004):0.1661299999999999):0.1032999999999999
5):0.11878999999999973,('SFB66474.1':0.6528899999999997,((('RGR54905.1':0
.31196999999999964,'WP_087253218.1':0.1268199999999995):0.159279999999999
87,(('SCW73718.1':0.16197000000000017,('EFU76455.1':0.07035999999999998,(
'AYA99694.1':0.000200,('KXB53087.1':0.029990000000000183,'EHO48117.1':0.0
14819999999999833):0.000210):0.004930000000000323):0.5876099999999997):0.
14444000000000035,(('OLA06490.1':0.029389999999999805,'SOB72961.1':0.0002
00):0.22510999999999992,('CUP12250.1':0.2702,(('RGK37322.1':0.21037000000
000017,'HBZ63502.1':0.5060799999999999):0.06810999999999989,('HAT88294.1'
:0.16233999999999993,('EEG94368.1':0.01456000000000035,'CRL43258.1':0.000
210):0.23370999999999986):0.1689099999999999):0.047410000000000174):0.117
52999999999947):0.22971000000000075):0.1632599999999993):0.07826000000000
022,(('OPZ32671.1':0.5266000000000002,('SET52351.1':0.07801999999999998,(
'OUQ05535.1':0.0759000000000003,'OUQ07963.1':0.030939999999999745):0.1577
6000000000012):0.68546):0.20613999999999955,(('SCX12007.1':0.011410000000
000142,'SCX12118.1':0.0547200000000001):0.57619,('SCG94033.1':0.406820000
0000002,(('AMC51852.1':0.000300,('CKV02534.1':0.000180,('CCC45171.1':0.01
0720000000000063,'AMC65164.1':0.000210):0.012809999999999988):0.000550):1
.1765500000000002,(('CUO16880.1':0.6949399999999999,('EHI56779.1':0.68274
99999999995,('ESL02974.1':0.5097900000000002,('PTL29108.1':0.636480000000
0002,('EGG91031.1':0.1235400000000002,('AYB00351.1':0.06876999999999978,(
'EIC95117.1':0.05680999999999958,'EJZ68973.1':0.0830099999999998):0.04943
0000000000085):0.10123999999999977):0.41017000000000037):0.23587999999999
987):0.14272000000000018):0.14179999999999993):0.24592999999999998,((('OP
Z94290.1':0.8427800000000003,('EAX48914.1':0.2374900000000002,'EAX49041.1
':0.14766999999999975):0.49758999999999975):0.32287,('SEQ67639.1':1.44251

99999999996,('SCJ11411.1':0.6195900000000001,'SFR81225.1':1.2792999999999
997):0.1559400000000002):0.2770199999999998):0.18846000000000007,((('EHL1
9060.1':0.6146599999999998,'ALQ42641.1':0.28176999999999985):0.8986900000
000002,(('SFE73898.1':0.4807999999999999,'ASS38486.1':0.6911900000000002)
:0.14154,('VEJ34624.1':1.0545900000000001,('RJW30883.1':0.031139999999999
723,'WP_062808329.1':0.000210):0.4732000000000003):0.21571000000000007):0
.04817999999999989):0.09756000000000009,(('OLR64066.1':0.4239600000000001
,('ADG82859.1':0.5755400000000002,('ABI69046.1':0.13403999999999971,'OQB7
3704.1':0.059800000000000075):0.4245099999999997):0.31354000000000015):0.
12769999999999992,((('EEF92712.1':0.5105600000000003,('EFV01913.1':0.8313
399999999995,'SER78324.1':0.4146399999999999):0.25459999999999994):0.1249
4000000000005,('CUO70252.1':0.7286800000000002,(('HCW38729.1':0.214879999
99999996,('CCX82922.1':0.013639999999999652,'OKZ95403.1':0.000180):0.2409
699999999999):0.33607999999999993,('CCX73453.1':0.3155699999999997,(('SCI
59180.1':0.19153999999999982,('RGB74595.1':0.028400000000000425,'RGC44690
.1':0.026110000000000078):0.3429599999999997):0.06440999999999963,('HAJ41
004.1':0.2219199999999999,('SCG89712.1':0.19008000000000003,('EQM98871.1'
:0.07699999999999996,'ERI97393.1':0.13964):0.09748000000000001):0.0817300
000000003):0.05689000000000011):0.2874500000000002):0.07091000000000003):
0.14103999999999983):0.11018000000000017):0.09019999999999984,(('KQC84609
.1':0.7810999999999999,'SES98352.1':0.8031299999999999):0.145389999999999
9,('RRD93721.1':0.2547799999999998,('HAJ73900.1':0.3177100000000004,(('RG
K40246.1':0.2639899999999997,'EOS81373.1':0.16917000000000026):0.05677999
999999983,(('CDD66307.1':0.3655699999999995,('EFE91104.1':0.006070000000
000242,'ERL20508.1':0.01841999999999988):0.53857):0.11324999999999985,('R
GG19528.1':0.13619999999999965,('RHP10705.1':0.21750000000000025,('HAD664
87.1':0.20716999999999963,(('CDE69449.1':0.010919999999999597,'RHV04444.1
':0.000200):0.2904600000000004,(('CUO30698.1':0.18039999999999967,'CUN226
31.1':0.13942999999999994):0.12957000000000019,('RHG29043.1':0.000190,(('
RHA66005.1':0.0,'RHM06244.1':0.0):0.000180,(('EEV02629.1':0.000180,'RHC19
824.1':0.000210):0.010440000000000005,('RHN04100.1':0.011250000000000426,
'OLA55794.1':0.011429999999999983):0.000210):0.010399999999999743):0.00020
0):0.16357999999999961):0.019989999999999952):0.033190000000000275):0.089
85999999999983):0.05567000000000011):0.054839999999999556):0.028520000000
000323):0.1390100000000003):0.13684999999999992):0.20807999999999982):0.0
8999999999999986):0.060550000000000104):0.08933999999999997):0.0931000000
0000018):0.08932999999999991):0.12380000000000013):0.2946100000000004):1
.4505999999999997):0.4472699999999996):0.14202000000000048):0.02632999999
9999742):0.09020000000000028):0.0659799999999997):0.13912000000000013):0.
08108000000000004):0.023399999999999643,(((('ABR30217.1':0.08406000000000
002,'ACJ76376.1':0.14474000000000053):0.2895599999999998,('AEX84731.1':0.
13946000000000058,'OQY08079.1':0.10195000000000043):0.049399999999999444)
:0.2261899999999999,('OWP56425.1':0.5250899999999996,(('SDY05883.1':0.567
1399999999993,('EDP75377.1':0.11615999999999982,'BAI69698.1':0.0910799999
9999983):0.07435999999999954):0.05672999999999995,(('BAI80039.1':0.076740
00000000003,'SNZ07756.1':0.1539299999999999):0.09921000000000024,('ACN993
00.1':0.17849999999999966,'SNR89705.1':0.2001999999999997):0.077680000000
00086):0.12506999999999913):0.18261000000000038):0.02259999999999973):0.0
9264000000000028,(('AWR95398.1':0.6744400000000002,('ODN29671.1':0.450630
0000000003,('AEH51739.1':0.5214700000000003,('ABV32777.1':0.3070200000000
005,('KUK23932.1':0.2440300000000004,'HCO98530.1':0.15212000000000003):0.
19887999999999995):0.22928000000000015):0.2250899999999998):0.12443999999
999988):0.13914000000000026,((('OUP77394.1':0.16096000000000021,('EEA8570
4.1':0.4223600000000003,('SNU99538.1':0.23467000000000038,('KNZ42391.1':0

.20882000000000023,'PWW54092.1':0.21715000000000018):0.11812000000000022)
:0.06226999999999825):0.30252000000000034):0.4235299999999995,('ACR79377
.1':0.2295500000000006,('PNS37164.1':0.000200,('PVD17378.1':0.13409000000
00005,('AFK07931.1':0.014050000000000118,'PZC51854.1':0.01481000000000021
2):0.013889999999999958):0.04391999999999996):0.1651100000000003):0.29298
999999999964):0.20796999999999954,(('HCZ05830.1':0.5777900000000002,(('OK
Y78761.1':0.43754000000000026,(('EYE89395.1':0.3494200000000003,'SHE32866
.1':0.3144800000000001):0.10046,('OGF54888.1':0.20094999999999974,('KXG77
807.1':0.31571000000000016,('ADI02106.1':0.28485000000000005,'GBC98668.1'
:0.30452999999999975):0.11228000000000016):0.20087999999999973):0.3103199
9999999993):0.08169000000000004):0.11617999999999995,(('ODS31541.1':0.426
64999999999953,('RPI19238.1':0.47663999999999973,(('CUU01221.1':0.1103699
9999999964,'CUT04207.1':0.1908599999999998):0.2743000000000002,('CUU07896
.1':0.4504699999999997,'PKP54661.1':0.56114):0.10625000000000018):0.19188
99999999999):0.09572999999999965):0.09909999999999997,('RMF70322.1':0.504
4000000000004,(('ACF14797.1':0.5944699999999998,'PKM49939.1':0.62568):0.1
3425000000000065,('ANQ52936.1':0.3813000000000004,('GBU08795.1':0.1859099
999999998,(((('PSR02143.1':0.29380000000000006,'HCY00221.1':0.18162999999
999974):0.08748000000000022,('RCW32758.1':0.18134000000000006,('SKC24160.
1':0.03868000000000027,('GAO28630.1':0.1366900000000002,'SFE65352.1':0.08
70700000000002):0.17264999999999997):0.09478999999999971):0.0591900000000
00076):0.04095000000000004,(('MAT55591.1':0.3493900000000001,(('OGW02464.
1':0.14805000000000046,'GAB63525.1':0.05266000000000037):0.03046999999999
9775,('RIJ91415.1':0.03636000000000017,('OGW14360.1':0.11495000000000033,
('CAJ74868.1':0.0,'SOH02987.1':0.0):0.10415000000000019):0.04007999999999
967):0.032340000000000035):0.19489):0.11760999999999999,('ALO17063.1':0.1
6403999999999996,('SHE83445.1':0.09970000000000034,(('GAH49936.1':0.17097
00000000005,('GAP44181.1':0.2633199999999998,'PIQ29316.1':0.1911999999999
9981):0.04799000000000042):0.028089999999999726,(('KUK56786.1':0.15104000
000000006,'RIJ47245.1':0.11063):0.058149999999999924,(('GAP70877.1':0.096
24000000000033,'OQC39844.1':0.22128000000000014):0.0855699999999997,('OFX
53969.1':0.05828000000000033,(('OFX76356.1':0.014720000000000066,'OJV1980
6.1':0.015060000000000073):0.15464999999999973,('PTN09617.1':0.1548600000
0000022,'SHJ71167.1':0.0532099999999998):0.03925999999999963):0.05252000
0000000344):0.041859999999999786):0.010569999999999968):0.046440000000000
04):0.09818000000000016):0.04628999999999994):0.014209999999999834):0.050
040000000000084):0.04492999999999947,((('KGN99047.1':0.0638399999999999,'
SJZ67919.1':0.06148000000000042):0.08618999999999977,('KGN69281.1':0.1628
1000000000034,(('KGN74969.1':0.0,'SUB89152.1':0.0):0.18557000000000023,((
('KGN83847.1':0.0,'KGN88495.1':0.0):0.014359999999999928,('AAQ66980.1':0.
014599999999999724,'BAG34477.1':0.000190):0.014699999999999935):0.0717300
0000000007,('PVZ08790.1':0.12117000000000022,('GAD05596.1':0.015210000000
000168,('KGN88693.1':0.000210,'SQH73697.1':0.015109999999999957):0.000180
):0.0928899999999997):0.028270000000000017):0.0319799999999999):0.0362200
0000000014):0.028289999999999704):0.08581999999999956,(('SDF11929.1':0.33
29599999999999,('EGN57514.1':0.13253999999999966,'HCN52431.1':0.193469999
9999996):0.3659600000000003):0.13069000000000042,((('BBD45981.1':0.063620
00000000023,('AEW20121.1':0.04716000000000031,'EPT33673.1':0.111029999999
99996):0.008550000000000058):0.06566000000000027,('SEA34640.1':0.09016000
000000002,('PLB85966.1':0.014659999999999673,('PLB87385.1':0.058889999999
99989,('HAL65591.1':0.0,'HAW59420.1':0.0):0.030060000000000198):0.000210)
:0.04658999999999969):0.07802000000000042):0.015600000000000058,((('OIN58
179.1':0.17859999999999987,(('AUD05852.1':0.04178999999999977,'OZI05906.1
':0.04800000000000004):0.04321000000000019,('OJW70712.1':0.02353999999999

9672,'PRY39964.1':0.06869000000000014):0.000540):0.12199999999999989):0.1
0223000000000049,(('AEE48390.1':0.1800299999999999,('RMF22101.1':0.081199
99999999994,'RMH46293.1':0.07160000000000011):0.1764199999999998):0.09010
999999999969,('PAC31305.1':0.09950999999999954,('RFS17569.1':0.1721200000
000005,('PLK45909.1':0.09586000000000006,'AYQ36249.1':0.13974999999999982
):0.01800000000000238):0.04831999999999474):0.05736000000000008):0.0413
5000000000044):0.0771799999999998,('PKO99099.1':0.2290399999999999,((('AE
W01073.1':0.01990000000000025,'OQP59767.1':0.09068000000000032):0.1957299
9999999974,('SEA90597.1':0.25207999999999986,('AYD48412.1':0.080950000000
00008,('ASZ11931.1':0.17551000000000005,'RBL91812.1':0.19772999999999996)
:0.12272000000000016):0.08914):0.03784000000000096):0.04233999999999982,
(('KKX47645.1':0.16793999999999976,'PZX51588.1':0.10522999999999971):0.07
312999999999992,(('SEA59703.1':0.20954999999999968,(('OJU50685.1':0.19236
999999999949,'OJV54070.1':0.09051999999999971):0.05398000000000014,('OJU2
9194.1':0.1797200000000001,('AYO58291.1':0.1509100000000001,('SIT97704.1'
:0.14724000000000004,('OJY86468.1':0.02201999999999993,'PVD52179.1':0.069
44000000000017):0.05473999999999979):0.05712000000000028):0.000210):0.024
72999999999992):0.03205999999999998):0.05711999999999984,(('EHQ30583.1':0
.27556999999999965,('EHO40941.1':0.0,'APF20548.1':0.0):0.2375799999999999
):0.000390,('SFO06540.1':0.000190,('SEG40668.1':0.000180,('PRY48837.1':0.
2938399999999999,('PTB97787.1':0.15399999999999991,('SHN04424.1':0.117030
00000000019,('SMD44428.1':0.03085000000000044,'PSL07189.1':0.01235999999
9999705):0.01926000000000055):0.02791999999999945):0.04081000000000001)
:0.10142999999999969):0.04395000000000156):0.05941000000000185):0.03144
9999999999534):0.02974000000000032):0.02984999999999971):0.04659000000000
013):0.03069999999999995):0.03306000000000031):0.03873000000000015):0.021
419999999999106):0.0018299999999999983):0.04603000000000015):0.070410000
00000075):0.13614000000000015):0.07681000000000004):0.07100999999999935):
0.06831000000000031):0.04037000000000024):0.07209999999999983,(('AJC73847
.1':0.4891000000000001,(('ADR19823.1':0.13023000000000007,('ACI21703.1':0
.06498999999999988,'HCC69471.1':0.17251999999999956):0.10322000000000031)
:0.2239899999999997,(('ACI19344.1':0.17630999999999997,'PMQ01379.1':0.098
20999999999991):0.21425000000000027,(('ADL41492.1':0.000200,'ADQ03685.1':
0.01437000000000438):0.0142199999999999,('ADQ41852.1':0.000190,('ACM5928
6.1':0.01454999999999984,('ADQ08182.1':0.04414000000000007,('KHO63178.1':
0.04522000000000004,('ADH59808.1':0.014000000000000234,'ERM91434.1':0.000
180):0.028979999999999784):0.04574999999999996):0.03038000000000074):0.0
1435000000000307):0.000210):0.2957000000000001):0.14112999999999953):0.1
7262000000000022):0.07563000000000031,('AMW33132.1':0.46395000000000053,(
'ACN99600.1':0.5590599999999997,(((('AUS09238.1':0.23573999999999984,'RAL
26195.1':0.18344000000000005):0.03964000000000034,('PZX05811.1':0.1827899
9999999979,('PGA47418.1':0.014880000000000226,('PFW64696.1':0.0,'PFW73874
.1':0.0,'PFZ53138.1':0.0,'PHA49748.1':0.0,'PHA54520.1':0.0,'PHB78532.1':0
.0,'PHC60331.1':0.0,'PHE42109.1':0.0):0.000190):0.3192999999999997):0.207
08000000000037):0.1713300000000002,((('EOD01826.1':0.19377999999999984,('
AOT70862.1':0.23004999999999987,'SHK58781.1':0.13241999999999976):0.08545
000000000025):0.0858399999999997,(('KGG79718.1':0.01472000000000051,'KPU2
6819.1':0.014080000000000314):0.11107999999999985,('CCJ34801.1':0.06128,'
KRQ85886.1':0.10863000000000023):0.2906299999999997):0.14598999999999984)
:0.05603000000000246,(('SDH76309.1':0.26381999999999994,(('SFA87797.1':0
.2619500000000001,'OUM91842.1':0.39830999999999994):0.08651999999999971,(
'ERI07854.1':0.0648500000000003,('ABS22294.1':0.0900599999999998,'KFN0466
9.1':0.13554999999999984):0.1120000000000001):0.2571699999999999):0.07954
000000000017):0.02891000000000213,((('OEH93026.1':0.10625000000000018,'P

AE89157.1':0.31309999999999993):0.2097800000000003,('OEH84878.1':0.204060
00000000013,('SIT17613.1':0.2068500000000002,'OYD07557.1':0.0603199999999
9993):0.20645999999999987):0.06139000000000028):0.000210,(('EGL83975.1':0
.1564399999999999,('OCS84763.1':0.15653000000000006,('KGR92399.1':0.11104
000000000003,'RPJ96057.1':0.15061000000000035):0.11518999999999968):0.281
9799999999999):0.07187999999999972,((('KYD09963.1':0.0,'PTY77974.1':0.0):
0.21686999999999967,('SMG36678.1':0.1566799999999997,'MNW41669.1':0.25166
999999999984):0.2019099999999998):0.05812000000000017,('OUM88544.1':0.088
42000000000017,(('AXM89704.1':0.014029999999999987,('SFA58200.1':0.000200
,'EMT47063.1':0.042669999999999764):0.000210):0.11721000000000004,(('ADP7
4268.1':0.10402000000000022,'OXB92095.1':0.000210):0.09570999999999996,('
ASS99179.1':0.014130000000000198,('KJE26697.1':0.01446000000000014,('KYD3
2180.1':0.0,'ATA60318.1':0.0):0.000190):0.014939999999999731):0.040869999
99999996):0.07311999999999985):0.06754000000000016):0.03876999999999997):
0.017049999999999788):0.02413000000000043):0.06763999999999992):0.1059699
9999999968):0.048730000000000384):0.08179999999999943,(('OON98294.1':0.52
00300000000002,('SHK10179.1':0.29628999999999994,('ETI96722.1':0.25485000
000000024,(('CED93821.1':0.17202000000000028,'SHH12206.1':0.2457499999999
997):0.02300000000000013,('EFH05805.1':0.000180,(('EQF23392.1':0.01372000
0000000176,'KPI49094.1':0.013440000000000119):0.04110999999999976,('SJP84
228.1':0.02674999999999983,('KLR55548.1':0.013309999999999711,('CBE06405.
1':0.000180,'OFU27205.1':0.000170):0.000200):0.013499999999999623):0.0001
80):0.04149000000000047):0.22405000000000008):0.04764999999999997):0.1196
5999999999966):0.11558000000000046):0.026849999999999596,(('KYO67328.1':0
.3452499999999996,('KIE48477.1':0.2810299999999999,(('ABG85728.1':0.16672
00000000002,(('PRR80308.1':0.16351000000000004,'AYD40188.1':0.000190):0.1
7063000000000006,('HAK42158.1':0.37444999999999995,('OOM45176.1':0.194729
99999999985,('AYE33254.1':0.0,'QAS61425.1':0.0):0.0848399999999998):0.149
3199999999999):0.11128999999999989):0.02829000000000015):0.02417999999999
987,(('ENY99756.1':0.58847,('EES47852.1':0.2481100000000005,'KZL91874.1':
0.14998000000000022):0.11353999999999953):0.0938699999999999,('ERI93848.1
':0.1640299999999999,('CDM67383.1':0.14083000000000023,('AGF58562.1':0.10
898999999999992,('EHJ01144.1':0.09487000000000023,('SFF89272.1':0.0,'PWL5
3907.1':0.0):0.1788700000000003):0.013729999999999798):0.0470399999999999
7):0.09421999999999997):0.01949999999999985):0.07799000000000023):0.11278
999999999995):0.06447999999999965):0.0035700000000007392,(('SHH05386.1':0
.09640000000000004,('KXZ39967.1':0.0,'WP_066070038.1':0.0):0.148639999999
99988):0.2301700000000002,('ODM28179.1':0.38380999999999954,(('AIY84916.1
':0.000210,'KJU72924.1':0.05629000000000017):0.31766000000000005,(('KYH34
075.1':0.04540000000000033,('OOO69967.1':0.000190,('EDU38645.1':0.000210,
('OQP93145.1':0.028890000000000082,('APH14746.1':0.0,'APQ78505.1':0.0):0.
000210):0.014390000000000125):0.014339999999999797):0.09250000000000025):
0.012699999999999712,(('SQB33600.1':0.014270000000000227,'STA92279.1':0.0
00180):0.2214999999999998,('KGN04936.1':0.026289999999999925,('KGM99193.1
':0.017859999999999765,('EDS77981.1':0.013879999999999448,'KEH93512.1':0.
000180):0.02754000000000012):0.000210):0.03092000000000006):0.01693999999
9999955):0.04228999999999994):0.06476999999999977):0.05838000000000054):0
.05245999999999995):0.0226999999999995):0.011730000000000018):0.111670000
00000016):0.09829000000000043):0.06228999999999957):0.2575599999999998):
0.028659999999999464):0.07792000000000066):0.05851999999999968):0.1025799
9999999967):0.09550000000000036):0.015390000000000015):0.1121100000000003
8):0.04179999999999939):0.020990000000000286);

File 4

((MGYP000319188410:1.1602100000000002,(MGYP000109810180:0.972470000000000
4,((MGYP000010847157:0.00782000000000604,MGYP000684851047:0.005880000000
000329):0.4920200000000001,(MGYP000619297365:0.26516999999999946,('WP_117
998314.1 [Eubacterium rectale AF19-
4]':0.000200,MGYP000192585983:0.000170):0.26992999999999956):0.2295700000
0000072):0.37246999999999986):0.16929000000000016):0.08399999999999963,(M
GYP000252681734:0.9150300000000007,(('WP_062808098.1__[Blautia_sp._Marsei
lle-
P2398]':0.040160000000000196,MGYP000167051486:0.023100000000000342):0.731
5800000000001,((MGYP000165542897:0.21136,(MGYP000162821430:0.012439999999
999785,MGYP000058379904:0.015600000000000058):0.10740000000000016):0.0711
1,(MGYP000029912630:0.04147999999999996,((MGYP000109203063:0.348129999999
9994,MGYP000120911204:0.14416999999999902):0.19248000000000065,((MGYP0006
68338691:0.006680000000000241,MGYP000515010317:0.011820000000000164):0.00
6149999999999878,(MGYP000728120183:0.000170,(MGYP000705176138:0.000790,'W
P_118614261.1__[Ruminococcus_sp._TF11-
2AC]':0.000170):0.002060000000000173):0.010449999999999626):0.00799000000
0000386):0.04472999999999949):0.1886600000000005):0.75387):0.111790000000
00006):0.10719999999999974,((MGYP000160729454:0.000150,'WP_118572797.1__[
Ruminococcus_sp._AM40-10AC]':0.000690):1.39942,((('WP_022785443.1_CRISPR-
associated_endoribonuclease_Cas13a_[Lachnospiraceae_bacterium_NK4A179]':0
.7762700000000002,('WP_089928016.1__[Lachnospiraceae_bacterium_NE2001]':0
.6731499999999997,('WP_092321585.1__[Butyrivibrio_sp._YAB3001]':0.5302000
000000007,('WP_044921188.1_[Lachnospiraceae_bacterium_MA2020]':0.61943999
99999991,'WP_090551759.1_[Pseudobutyrivibrio_sp._OR37]':0.454909999999999
9):0.09912000000000099):0.15836999999999968):0.1860900000000001):0.111259
99999999969,(('WP_027114339.1_[Lachnospiraceae_bacterium_NK4A144]':0.4596
200000000014,'WP_031473346.1_[Clostridium]_aminophilum_strain_F]':0.5287
500000000005):0.20941999999999972,((MGYP000178941783:0.0861200000000002,(
'WP_087253216.1_[Drancourtella_sp._An57]':0.07891000000000048,(MGYP000505
721386:0.008989999999999831,'WP_090127496.1[Eubacteriaceae_bacterium_CHKC
I004]':0.009079999999999977):0.09156999999999993):0.0577399999999999):0.3
8673,((MGYP000742642845:0.07830000000000048,MGYP000661043385:0.0793400000
0000019):0.18489999999999984,((MGYP000217302039:0.019499999999999985,MGYP0
00615622314:0.016059999999999952):0.2259000000000002,(MGYP000644147385:0.0
846000000000001,((('WP_117482613.1__[Eubacterium_rectale_TM10-
3]':0.0038799999999996615,MGYP000516651569:0.021529999999999383):0.003659
9999999999966,('WP_118003838.1__[Eubacterium_rectale_AF25-25/AF18-
16LB]':0.000920,(MGYP000038574051:0.000190,'WP_055061018.1_[Eubacterium_r
ectale_T1-
815]':0.000790):0.005860000000000198):0.0026599999999996626):0.0533900000
0000027,(MGYP000546064296:0.028939999999999952,(MGYP000077493486:0.0191699
9999999991,(MGYP000027519385:0.010190000000000587,(MGYP000518759138:0.000
170,MGYP000309732945:0.0011999999999998678):0.004000000000000448):0.00458
9999999999428):0.02651000000000034):0.02625000000000107):0.032650000000
00029):0.12054000000000009):0.08847999999999967):0.22146000000000043):0.3
8211999999999957):0.13926000000000016):0.31467000000000045,('WP_079495749
.1__[Maledivibacter_halophilus]':1.7591200000000002,(((('WP_071146234.1__
[Bacteroides_ihuae]':0.8113800000000002,'WP_013443710.1_CRISPR-
associated_endoribonuclease_Cas13a_[Paludibacter_propionicigenes]':0.5953
399999999998):0.44272,(('WP_034560163.1_[Carnobacterium_gallinarum]':0.63
9470000000002,'WP_034563842.1_[Carnobacterium_gallinarum]':0.60619999999
99999):0.53213,(('WP_036091002.1__[Listeria_newyorkensis]':0.024420000000
00011,'WP_118907415.1_[Listeria_weihenstephanensis]':0.007159999999999833

):0.7242999999999999,('WP_099225408.1_[Listeria_costaricensis]':0.7927499
999999998,'WP_012985477.1_CRISPR-
associated_endoribonuclease_Cas13a_[Listeria_seeligeri]':0.68798):0.17830
000000000013):0.19863000000000008):0.1469999999999998):0.4897299999999998
,(((MGYP000123498965:0.3363499999999999,'WP_103203632.1_CRISPR-
associated_endoribonuclease_Cas13a_[Herbinix_hemicellulosilytica]':0.2982
800000000001):1.0113000000000003,(MGYP000698521930:0.43474999999999975,MG
YP000751596458:0.4828199999999998):0.9887299999999999):0.5702600000000002
,(MGYP000128950304:1.1677599999999995,('OQX30025.1_hypothetical_protein_B
0D92_00635,_partial_[Spirochaeta_sp._LUC14_002_19_P3]':1.0011200000000002
,(('WP_023911507.1_[Rhodobacter_capsulatus]':1.21265,('WP_133318297.1__[R
hizobium_sp._SPY-
1]':1.11775,('WP_137134457.1_[Rhizobium_sp._FKY42]':0.9315600000000002,('
WP_080615427.1_hypothetical_protein_[Rhodovulum_sp._MB263]':0.03158999999
999956,'WP_108028905.1_hypothetical_protein_[Rhodovulum_kholense]':0.0593
0000000000035):0.9745400000000002):0.3388200000000001):0.9100700000000002
):0.2512399999999997,(('WP_112317339.1__[Rhodovulum_viride]':0.9365100000
000002,'WP_133357912.1_[Ruegeria_sp._318-
1]':1.22098):0.09384000000000015,('WP_076398593.1_[Insolitispirillum_pere
grinum]':1.2186199999999996,(('WP_073955355.1_[Thalassospira_sp._TSL5-
1]':0.06594999999999995,'WP_114086813.1_[Thalassospira_profundimaris]':0.
06998999999999977):0.74336,('WP_132694182.1_[Rhodovulum_steppense]':0.653
5000000000002,('WP_100176879.1_[Bradyrhizobium_sp._TSA1]':0.6728300000000
003,'PJI41863.1_hypothetical_protein_CTR53_05225_[Ferrovibrio_sp.]':0.731
7999999999998):0.10329999999999995):0.23432999999999993):0.10576999999999
97):0.1519199999999996):0.08782000000000023):0.13287000000000004):0.32714
):1.4785500000000003):0.09602999999999895):0.1759800000000009,(((MGYP0003
24658027:0.01839000000000013,(MGYP000365881605:0.05642000000000014,MGYP00
0442596835:0.020700000000000163):0.018879999999999786):0.1639599999999998
8,((MGYP000041399292:0.11587000000000014,('WP_021746003.1_CRISPR-
associated_endoribonuclease_Cas13a_[Leptotrichia_wadei_F0279]':0.03078000
000000003,MGYP000053356571:0.04389999999999983):0.09988999999999981):0.01
3879999999999892,(MGYP000578087195:0.006050000000000111,(MGYP000209636426
:0.007810000000000095,(MGYP000191921353:0.0015299999999998093,(MGYP000728
506240:0.05119000000000007,MGYP000710930945:0.009110000000000174):0.05527
000000000015):0.09563999999999995):0.21075):0.010749999999999815):0.11182
999999999987):1.1139399999999995,(('WP_018451595.1_type_VI-A_CRISPR-
associated_effector_C2c2_[Leptotrichia_shahii_DSM_19757]':0.1874200000000
0036,'WP_021744063.1_type_VI-A_CRISPR-
associated_effector_C2c2_[Leptotrichia_sp._oral_taxon_879]':0.14398999999
999962):2.2818499999999995,((MGYP000295084724:0.04212000000000016,(('WP_0
71124126.1__[Leptotrichia_massiliensis]':0.04997000000000007,(MGYP0003098
10837:0.01670999999999978,(MGYP000486752824:0.02361000000000013,MGYP00042
4752507:0.00796000000000019):0.023070000000000146):0.04131000000000018):0
.015569999999999862,(MGYP000070896523:0.018240000000000034,(MGYP000356942
902:0.03655000000000008,(MGYP000430768964:0.021700000000000053,(MGYP00068
4248307:0.029240000000000155,MGYP000130031563:0.000180):0.016900000000000
137):0.011130000000000084):0.02129000000000003):0.04483000000000015):0.02
5949999999999918):0.3860800000000002,(((MGYP000666568610:0.02448000000000
0057,(MGYP000250759845:0.027530000000000054,(MGYP000353980233:0.021199999
999999886,MGYP000168334215:0.02137000000000011):0.014250000000000096):0.0
16020000000000145):0.09855999999999998,(MGYP000280275645:0.09999000000000
002,((MGYP000082840345:0.04685000000000006,(MGYP000578150587:0.0614300000
00000096,(MGYP000701927337:0.030670000000000197,MGYP000194886005:0.050829

99999999993):0.02829000000000015):0.03055999999999992):0.0792399999999999
8,(MGYP000643044933:0.08191000000000015,('A_ERK47820.1_hypothetical_prote
in_HMPREF9015_02301_[Leptotrichia_wadei_F0279]_Copy':0.08374000000000015,
(MGYP000421818960:0.02754000000000012,MGYP000501496954:0.0312199999999998
03):0.02459999999999955):0.03996999999999984):0.01319999999999878):0.03
5960000000000214):0.05202000000000018):0.45352999999999977,((MGYP00043963
6926:0.06467,(MGYP000271550373:0.016999999999999904,(MGYP000513213728:0.1
2836999999999987,'WP_015770004.1_CRISPR-
associated_endoribonuclease_Cas13a_[Leptotrichia_buccalis_C-1013-
b]':0.003179999999999606):0.03155000000000019):0.01185999999999982):0.0
3512000000000004,(('WP_071125398.1__[Leptotrichia_massiliensis]':0.003039
9999999999316,(MGYP000177060630:0.02156000000000024,'WP_021768357.1__[Le
ptotrichia_sp._oral_taxon_225_str._F0581]':0.01288000000000003):0.015190
000000000037):0.06652000000000013,(MGYP000318745293:0.03851000000000044,
(MGYP000722609020:0.006720000000000059,((MGYP000103429604:0.03708,MGYP000
740329489:0.018019999999999925):0.011660000000000004,(MGYP000374686211:0.
049329999999999874,(MGYP000256833066:0.007789999999999964,'WP_021746774.1
__[Leptotrichia_wadei__F0279]':0.012199999999999989):0.01203000000000207
):0.07421999999999995):0.02281999999999984):0.01778000000000013):0.016150
00000000011):0.03277999999999981):0.35973999999999995):0.1298499999999998
):0.7119899999999997):0.19465000000000002):0.18915000000000006):0.126559999
99999956):1.3260800000000001):0.14114999999999966):0.17842000000000002);
